# Patient derived tumoroids of high grade neuroendocrine neoplasms for more personalized therapies

**DOI:** 10.1038/s41698-024-00549-2

**Published:** 2024-03-01

**Authors:** Simon L. April-Monn, Philipp Kirchner, Katharina Detjen, Konstantin Bräutigam, Mafalda A. Trippel, Tobias Grob, Cyril Statzer, Renaud S. Maire, Attila Kollàr, Aziz Chouchane, Catarina A. Kunze, David Horst, Martin C. Sadowski, Jörg Schrader, Ilaria Marinoni, Bertram Wiedenmann, Aurel Perren

**Affiliations:** 1 Institute of Tissue Medicine and Pathology, University of Bern, 3008 Bern, Switzerland; 2 Graduate School for Cellular and Biomedical Sciences, University of Bern, 3008 Bern, Switzerland; 3 Charité—Universitaetsmedizin Berlin, Corporate Member of Freie Universitaet Berlin and Humboldt-Universitaet zu Berlin, Hepatology and Gastroenterology, Berlin, Germany; 4 Department of Health Sciences and Technology, Eidgenoessische Technische Hochschule Zuerich, Schwerzenbach-Zuerich, 8603 Switzerland; 5 Department of Medical Oncology, Inselspital, Bern University Hospital, University of Bern, Freiburgstrasse, CH-3010 Bern, Switzerland; 6 Institute of Pathology, Charité Universitaetsmedizin Berlin, Rudolf-Virchow-Haus, Berlin, Germany; 7 Department of Medicine, University Medical Center Hamburg-Eppendorf, 20251 Hamburg, Germany; 8 Bern Center for Precision Medicine, University & University Hospital of Bern, 3008 Bern, Switzerland

**Keywords:** Oncology, Cancer, Endocrine cancer, Neuroendocrine cancer

## Abstract

There are no therapeutic predictive biomarkers or representative preclinical models for high-grade gastroenteropancreatic neuroendocrine neoplasms (GEP-NEN), a highly aggressive, fatal, and heterogeneous malignancy. We established patient-derived (PD) tumoroids from biobanked tissue samples of advanced high-grade GEP-NEN patients and applied this model for targeted rapid ex vivo pharmacotyping, next-generation sequencing, and perturbational profiling. We used tissue-matched PD tumoroids to profile individual patients, compared ex vivo drug response to patients’ clinical response to chemotherapy, and investigated treatment-induced adaptive stress responses.

PD tumoroids recapitulated biological key features of high-grade GEP-NEN and mimicked clinical response to cisplatin and temozolomide ex vivo. When we investigated treatment-induced adaptive stress responses in PD tumoroids in silico, we discovered and functionally validated Lysine demethylase 5 A and interferon-beta, which act synergistically in combination with cisplatin. Since ex vivo drug response in PD tumoroids matched clinical patient responses to standard-of-care chemotherapeutics for GEP-NEN, our rapid and functional precision oncology approach could expand personalized therapeutic options for patients with advanced high-grade GEP-NEN.

## Introduction

High-grade gastroenteropancreatic neuroendocrine neoplasm (GEP-NEN), which comprise poorly differentiated neuroendocrine carcinomas (GEP-NEC) and high-grade well-differentiated neuroendocrine tumors (GEP-NET), are highly aggressive and heterogeneous cancers and there is strong need of therapies to treat them^1–4^. Median overall survival for metastatic GEP-NECs patients is less than 1 year^1–4^. Slightly better outcomes are reported in high-grade GEP-NET patients but with high and unpredictable variations in overall survival^3^. Existing therapeutic strategies for GEP-NENs have been adopted from small-cell lung cancers (SCLC) due to their apparent clinical- and histomorphological similarities^5–7^. Platinum-based chemotherapy is frequently used in GEP-NECs treatment. Temozolomide-based chemotherapy is currently in clinical use for high-grade GEP-NET^8^ as response rates of platinum-based therapies seem lower^9^.

Due to the rarity and heterogeneity of the disease, extensive multi-arm clinical trials, and even exploratory and confirmatory studies, are challenging to perform. No predictive therapeutic biomarkers for high-grade GEP-NEN are in clinical use. Thus, the precise, clinical therapeutic regimes are mainly empirical, relatively uniform, and based only on small case series^5,6^. This modus operandi has increasingly been scrutinized because uniform therapy does not account for the heterogeneity of GEP-NEN patients^1,10,11^.

Preclinical GEP-NEN models were developed during the search for predictive therapeutic biomarkers and more efficient therapy options. But these GEP-NEN preclinical models were not successfully used to develop novel or combined treatments based on mechanistic insights—this is a pressing unmet need in the field. Patient-derived (PD) xenografts of GEP-NENs have low success rates, and the few available NEN cell lines fail to accurately recapitulate the biology of high-grade GEP-NENs^12,13^. Current GEP-NEN models provide insufficient functional and mechanistic insight into drug responses and it remains difficult to develop novel- and co-treatments for GEP-NEN patients.

We recently described a patient-derived 3-D tumoroid model that facilitates multi-center collections, efficient processing, characterization, and short-term drug screening of low abundant tumor tissues from human low-grade NET with high success rates^14^. Sato et al. described a tumor organoid biobank that included stable organoid lines from a few patients with neuroendocrine neoplasms that the authors used for their longer-term cultures^15^.

Without sufficient clinical data, it is difficult to define the translational relevance of these 3-D ex vivo models that are derived from high-grade GEP-NEN patients. We thus set out to determine how well a patient-derived rapid ex vivo model recapitulates an individual patient’s response to therapy, and to test whether the rapid ex vivo model can provide functional insight on drug- and stress responses of individual patients.

We used targeted ex vivo pharmacotyping and next-generation sequencing in tumor tissues and matching patient-derived (PD) tumoroids to determine if PD tumoroids enable rapid ex vivo pharmacotyping and if the subsidiary biological information and the adaptive stress response patterns they provided could be used to personalize therapy strategies in individual advanced high-grade GEP-NEN patients.

We show high success rates in culturing PD tumoroids of high-grade GEP-NENs within a 2-week time window. These patient-derived tumoroids recapitulated key biological features of high-grade GEP-NEN and mimicked clinical response to cisplatin and temozolomide ex vivo. We also investigated molecular stress responses in PD tumoroids in silico, discovering and functionally validating Lysine demethylase 5 A (KDM5A) and interferon-beta (IFNB1)—two vulnerabilities that are synergistic in combination with cisplatin. Either KDM5A or IFNB1 can be combined with cisplatin to boost the effectiveness of the treatments, opening new therapeutic options for high-grade GEP-NENs. Together, our findings suggest that we can translate patient-centered subsidiary information from PD GEP-NEN tumoroids into potentially more effective personalized treatment strategies.

## Results

### In-depth characterization of the high-grade GEP-NEN patient cohort

**Fig. 1:**
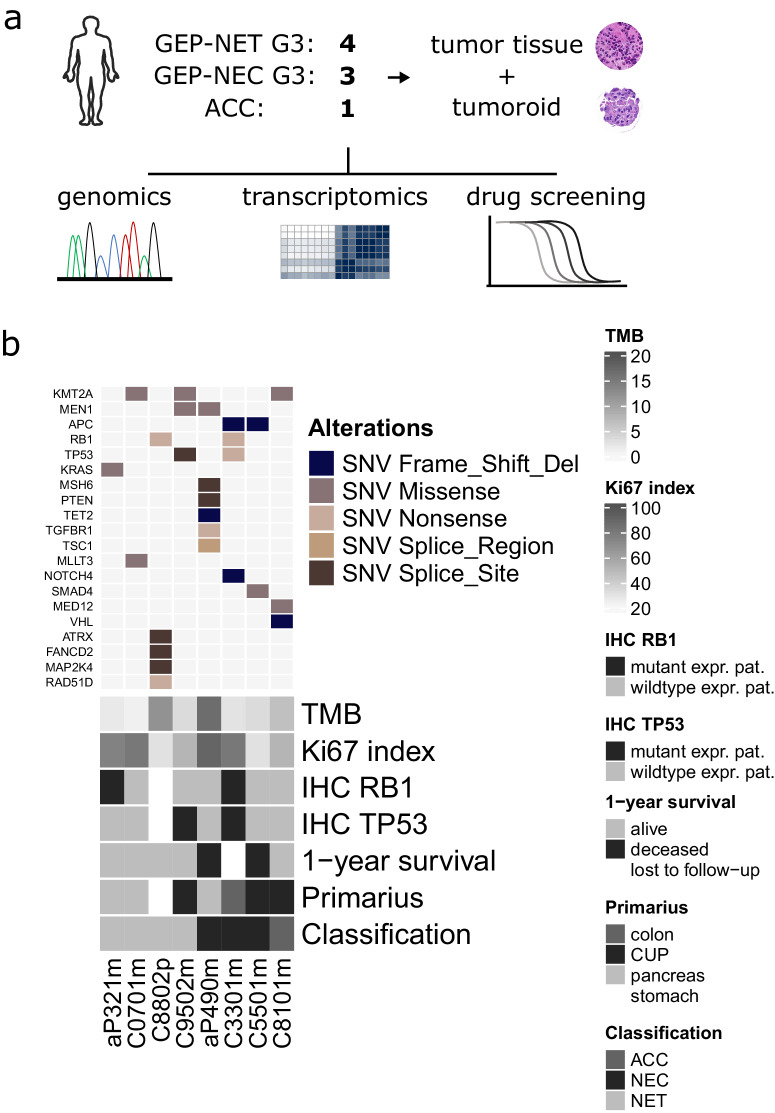
Study overview and clinical presentation of high-grade GEP-NEN patient cohort. **a** Schematic diagram of study outline, material processing, and analysis performed in the present study. **b** Oncoplot showing common genetic alterations of GEP-NENs found in the study cohort together with a selection of clinical parameters. The upper panel indicates specific types of single nucleotide variations (SNV) found in fresh frozen original tumor tissue from high-grade GEP-NEN patients. The lower panel displays the patient’s clinical parameters, including tumor mutation burden (TMB; mutation/Mb),1-year survival, IHC-based proliferation status (Ki-67; percent positive cells per tissue), RB1 protein expression, TP53 protein expression, location of primaries, and the diagnostic classification. NET neuroendocrine tumor, NEC neuroendocrine carcinoma, ACC acinar cell carcinoma, CUP cancer of unknown primary, Mutant expr. pat. Mutant expression pattern (For TP53 loss of protein (0% positive tumor cells) or overexpression (≥90% positive tumor cell); For RB1 complete loss of protein), wildtype expr. pat. wildtype expression pattern.

To investigate whether patient-derived tumoroids can successfully model advanced malignant GEP-NENs and to elucidate the biology of the disease, we conducted a retrospective cohort study of human high-grade GEP-NEN patients who were operated at the University Hospital of Bern (CH) or Charité University Hospital Berlin (DE). During a systematic retrospective review of hospital biobank records, we identified and retrieved those cases in which fresh-frozen tissue-matched cryopreserved G3 NEN tumor tissues were available. Between 1987 to 2022, we identified eight patient cases from the cryopreserved GEP-NEN patient samples (Fig. 1a). This small sample reflects the rarity of this type of tissue resource, as most patients are diagnosed at an advanced metastatic stage and undergo diagnostic biopsies rather than surgery.

The cohort comprises high-grade metastatic neuroendocrine tumors (NET G3, *n* = 4), neuroendocrine carcinomas (NEC, *n* = 3) of gastric- (Ga), pancreatic- (Pan), or unknown primary (CUP) site, and one additional case that had been diagnosed as NEN (*n* = 1) at the time of initial diagnosis, but the liver metastasis that we obtained at a later disease stage was reclassified as acinar cell carcinoma during our case review (Table 1). Patient demographics, clinicopathological classification, and comprehensive clinical course records are presented in Tables 1 and 2 and Supplementary Table S1.

**Table 1:** Patient demographics and clinicopathological classification

Patient ID	sex	Age at surgery[y]	tissue source	primary tumor localization	1_year survival	Classification	Morphology IFP Subtype	histological differentiation	CgA	Ki67	MCT4	PDX1	RB1	SOX9	SSTR2A	SYN	TP53	DAXX	ATRX	ARX
C9502m	f	57	liver metastasis	CUP	alive	NET	NA	WD	moderate positive (++)	50	heterogenous (2)	negative (−)	wildtype expr. pat.	negative (−)	moderate positive (++)	strong positive (+++)	mutant expr. pat.	NA	NA	NA
C8802p	m	66	primarius	stomach	alive	NET	NA	WD	NA	30	NA	NA	NA	NA	NA	NA	NA	NA	NA	NA
C8101m*	f	44	liver metastasis	CUP	alive	ACC	Large-cell	NA	weak positive (+)	50	heterogenous (2)	negative (−)	wildtype expr. pat.	positiv	negative (−)	negative (−)	wildtype expr. pat.	NA	NA	NA
C5501m	f	39	metastasis ovary	CUP	deceased	NEC	Large-cell	PD	strong positive (+++)	30	positive (1)	negative (−)	wildtype expr. pat.	negative (−)	weak positive (+)	strong positive (+++)	wildtype expr. pat.	NA	NA	NA
C3301m	m	70	liver metastasis	colon	lost to follow-up	NEC	Small-cell	PD	negative (−)	80	heterogenous (2)	negative (−)	mutant expr. pat.	positive	negative (−)	strong positive (+++)	mutant expr. pat.	NA	NA	NA
C0701m	m	69	liver metastasis	pancreas	alive	NET	NA	WD	moderate positive (++)	80	heterogenous (2)	negative (−)	wildtype expr. pat.	negative (−)	negative (−)	strong positive (+++)	wildtype expr. pat.	negative (−)	X	negative (−)
aP490m	m	53	liver metastasis	pancreas	deceased	NEC	Large-cell	PD	strong positive (+++)	90	heterogenous (2)	negative (−)	wildtype expr. pat.	strong positive (+++)	negative (−)	strong positive (+++)	wildtype expr. pat.	positive	positive	negative (−)
aP321m	m	66	liver metastasis	pancreas	alive	NET	NA	WD	weak positive (+)	75	negative (0)	positive	mutant expr. pat.	positiv	moderate positive (++)	strong positive (+++)	wildtype expr. pat.	negative (−)	positive	positive

*At initial diagnosis, clinical differentiation between NET G3 and NEC was ambiguous. Due to an unexpected clinical course deviation, at that time a second expert opinion was obtained, which suggested a NET G3 differential diagnosis. Evaluation of the collected cryo-specimen after therapy revealed signs of acinar cell differentiation.

**Table 2:** Clinical course records of GEP-NEN patients

	Treatments *PRIOR* to tissue sampling							Tissue collection	Treatments *AFTER* tissue sampling		
Sample ID	Clinical course 1	Clinical course 2	Clinical course 3	Clinical course 4	Clinical course 5	Clinical course 6	Clinical course 7		Clinical course A	Clinical course B	Clinical course C
C8802	Cisplatin & Etoposide^a^ (PR)	*FOLFOX (Mixed response)*	*FOLFIRI (Mixed response)*	*Irinotecan & Carboplatin (Discont. due to AE)*	CAPTEM & Bevacizumab (PD)			Surgery primary tumor			
C3301	*Surgery primary tumor*	Cisplatin & Etoposide (PR)	*Doxorubicin & Cyclophosphamide (PR)*					Resection liver metastasis	*Brachytherapy*		
C5501	Cisplatin & Etoposide (SD)	*FOLFIRINOX*	*CAPTEM (PD)*					Debulking	*CAPTEM & Bevacizumab (PD)*	*Debulking*	*CAPTEM & Bevacizumab (PD)*
C0701	*Streptozotocin/5-FU (SD)*	CAPTEM (PD)	*FOLFOX (PR)*	*FOLFIRI (PR)*				Resection liver metastasis	*Carboplatin & Etoposid (PD)*		
C9502	*Surgery primary tumor*	*PRRT (PR)*	*Resection liver metastasis*	PRRT & Temozolomide^b^ (PR)	*SSA*			Resection liver metastasis			
C8101	Cisplatin & Etoposide^a^ (PR)	*Carboplatin & Etoposide (PR)*	*FOLFIRI (PD)*	*Topotecan (Discont. due to AE)*	*Best supportive care*			Resection liver metastasis & SIRT	*FOLFOX & RTX (PD)*		
aP321	*Streptozotocin & Doxorubicin (SD; Delayed PR)*	*Surgery primary tumor*	*Streptozotocine & Doxorubicin (PR)*	*PRRT (PR)*	*SSA*	*PRRT (PR)*	*SSA*	Resection liver metastasis	CAPTEM^c^ (PR)	*TAE*	*FOLFOX (PR)*
aP490								Surgery primary tumor & liver metastases	Cisplatin & Etoposide (PD)	CAPTEM (PD)	*Best supportive care*

*PR* partial response, *SD* stable disease, *PD* progressive disease.

^a^Stable and long-lasting response.

^b^Temozolomide discontinued due to bone marrow toxicity.

^c^Complete response in peritoneal-, pleural-, and cutaneous metastasis; stable hepatic lesions.

The patients’ fresh frozen material was subjected to a transcriptomic molecular analysis and next-generation sequencing to profile the tumor’s cancer-related gene mutation burden (Supplementary Fig. S1a). The tumor mutation burden (TMB) in all patients was low (median 3.1 mt/Mb; IQR 2.13–7.68 mt/Mb) except in two patients whose TMB was elevated (aP490m 11.8 mt/Mb; C8802p 16.4 mt/Mb) (Supplementary Table S1). Microsatellite instability (MSI) was low (2.4 ± 1.9%; mean ± SD), and we detected no alterations in copy number (Supplementary Table S1). The most frequent single nucleotide variants (SNV) were missense mutations. Among the SNVs in our samples, we found well-known prototypic genetic drivers of GEP-NET (MEN1, ATRX) in two NET samples and drivers of GEP-NECs (TP53, RB1, APC, SMAD4) in 2 GEP-NECsamples (Fig. 1b, Supplementary Table S2).

### Phenotypic characteristics of high-grade GEP-NEN patient-derived tumoroids resemble original tumor tissue

**Fig. 2:**
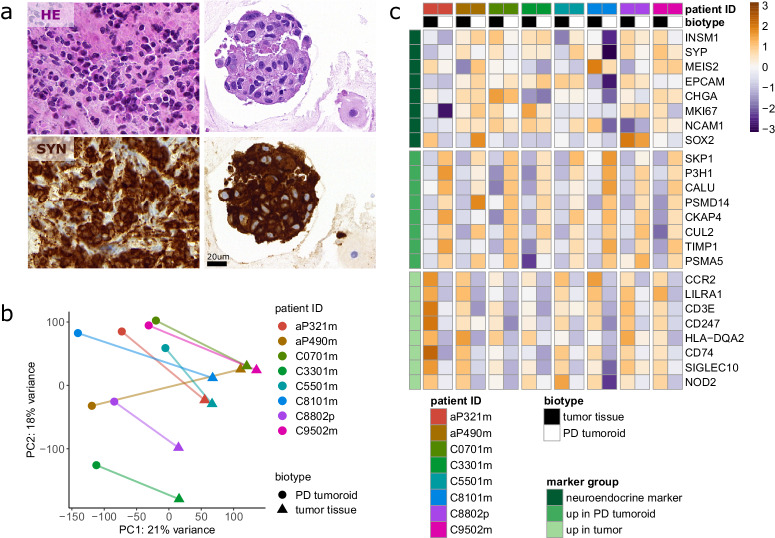
Patient-derived tumoroids recapitulate biological key features of original tumors. **a** Representative Hematoxylin and eosin (HE) staining and neuroendocrine diagnostic marker synaptophysin (SYN) immunolabeling in original tumor tissue and tissue-matched patient-derived (PD) tumoroids. Scale bar, 20 um. **b** Principal component analysis (PCA) plot of normalized gene expression in original tumor tissue and PD tumoroids. The color indicates patient identity with lines connecting matched original tumor and PD tumoroid samples. The variance explained by each principal component is indicated on the respective axis label. **c** Heatmap of gene expression for 8 neuroendocrine marker genes and the 8 top genes from the most strongly enriched gene ontology pathways. The pathways are “adaptive immune response” for the original tumor tissue and “post-translational protein modification” for the PD tumoroids. Gene expression values are centered and scaled row-wise (dark orange = highest expression, dark blue = lowest expression across all samples). Rows and columns are ordered by sample and gene identity.

We successfully generated PD tumoroids from all cryopreserved tissue-matched specimens based on the criteria specified in our methods section and found support for translational application of GEP-NEN PD tumoroids (see Methods,). We first determined if PD tumoroids preserve relevant histomorphological features of original high-grade GEP-NENs in culture. Two board-certified pathologists (A.P., M.T.) confirmed PD tumoroids were alike the original tumor tissue in high tumor content, in tumor cell morphology, and in the expression of diagnostic neuroendocrine biomarker synaptophysin, based on the cytology of micro-cell-blocks from cultured cells (Fig. 2a, Supplementary Fig. S2a, Supplementary Table S3). Thus, patient-derived tumoroids did preserve the neuroendocrine phenotype of GEP-NEN tumor cells. Moreover, we detected a focal presence of extracellular matrix (C9502m, C8802p, C5501m) and a focal presence of calcifications (C9502m) (Supplementary Fig. S2b, Supplementary Table S3). Since we intentionally depleted stromal cells in the 3-D culture workflow, non-neoplastic cells, including fibroblasts and macrophages, were less abundant in PD tumoroids than in original tumor tissue (Supplementary Fig. S2b, Supplementary Table S3). Patient-derived tumoroids also exhibited increased metabolic activity ex vivo over time (Supplementary Fig S2c), and this increase significantly correlated with the proliferation indices in the donor tissues.

We used next-generation RNA sequencing to assess the extent to which transcriptional expression patterns of original tumors had been retained in matching PD tumoroids. In the PCA plot of the expression data the PD tumoroids are separated from the original tumor tissue along the first principal component explaining 21% of variance (Fig. 2b). Compared to established NEN cell lines QGP1 and NT3, the PD tumoroids remained closer to the original tumor tissues (Supplementary Fig. S3a).

Transcriptional changes between PD tumoroids and original tumors were modeled using the patient as a random effect (Supplementary Fig. 3b, Supplementary Table S4). Gene set enrichment analysis (GSEA) on the expression differences revealed gene ontology (GO) biological processes more expressed in the original tumors or the PD tumoroids (Supplementary Fig. S3c, Supplementary Table S5). For the original tumor samples (positive normalized enrichment score, NES) the majority of pathways is related to immune function. This confirms the cytology findings that there were very few immune- and/or stromal cells in our patient-derived GEP-NEN tumoroids (Supplementary Fig. S2b, Supplementary Table S3). In contrast, for the PD tumoroids (negative NES) pathways linked to protein expression and stress response were found frequently. We looked at the expression of 8 neuroendocrine marker genes in the PDT and in the donor tissues as well as the top 8 genes from each of the most significantly up- or down-regulated GO terms (Fig. 2c). In most cases, the expression of neuroendocrine markers is comparable between original tumor and matched PD tumoroid. In contrast, genes from the GO terms “adaptive immune response” and “post-translational protein modification” are clearly overexpressed in original tumor or PD tumoroids respectively.

Altogether, transcriptomic profiles, histomorphology, and functional readouts underlined that GEP-NEN PD tumoroids are biologically complex and retain key traits of original GEP-NEN donor tumors, and that PD tumoroids harbor a degree of histological, cellular, and molecular diversity closer to original tumors than conventional permanent NEN cell lines.

### High-grade GEP-NEN patient-derived tumoroids mimic clinical response to platin and temozolomide treatment ex vivo

**Fig. 3:**
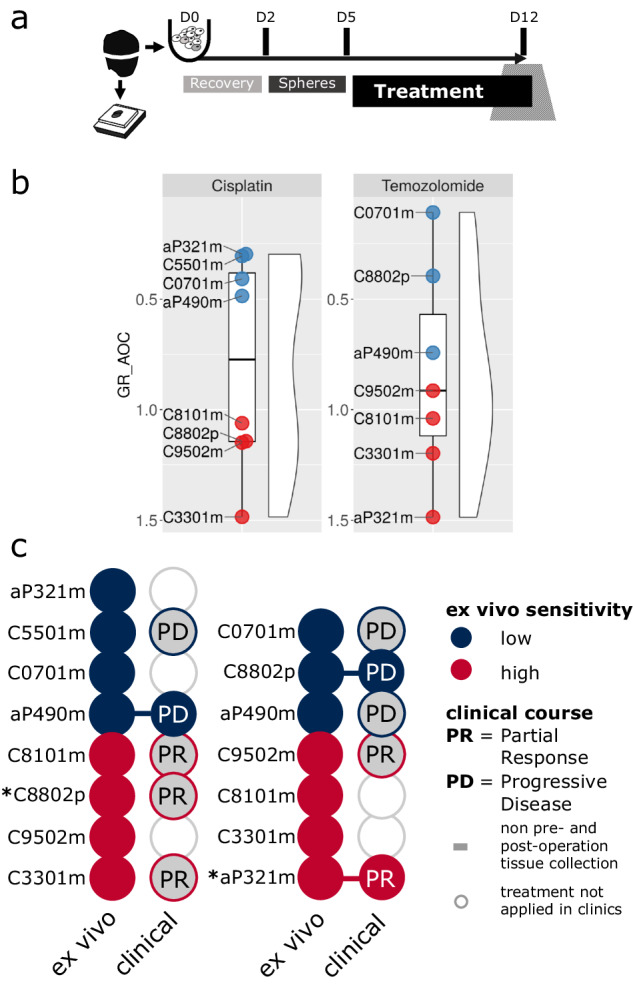
Patient-derived GEP-NEN tumoroids mimic the clinical patient response. **a** Schematic diagram of ex vivo drug screening workflow in patient-derived GEP-NEN tumoroids. Cryopreserved tissue of GEP-NEN were dissociated using gentle MACS. Isolated primary tumoroids were maintained in AdvDMEM + Grow Factors (EGF, bFGF, PlGF, IGF-1) in ultra-low attachment (ULA) plates. Tumor cells were allowed to form spheres for 2 days following a 3-day recovery period. All drug sensitivity measurements were obtained after 168 h (7 d) of treatment. For drug screening, 3000–4000 cells per well were plated in ULA plates and viability monitored in a continuous manner using RealTimeGlo (Promega). Micro cell blocks were made to assess tumor cell content. **b** Drug sensitivity in PD tumoroids measured as area over the growth rate (GR) corrected drug sensitivity curve (GR AOC). PD tumoroids were treated with DMSO (ctrl) or different concentrations of cisplatin or temozolomide for 168 h. Cell counts were normalized relative to control treated samples, converted to GR values and summarized as the GR AOC using the GRmetrics R package. Based on the median of the GR AOC values, PD tumoroids were equally split into high (bottom) and low (top) drug sensitivity. **c** Comparison between ex vivo sensitivity of PD tumoroids and clinical patient response for cisplatin (left) and temozolomide (right). Colors show drug sensitivity in vivo or in vitro. Solid circles connected with lines highlight patients with the indicated therapy applied directly before or after specimen collection. Circles with a gray fill represent cases where the treatment was applied at some other point in treatment history. Uncolored circles are samples not treated for the drugs investigated. Asterisks indicate patients (C8802p and aP321m) with accentuated and long-lasting clinical responses to either cisplatin or temozolomide systemic therapy.

To test if drug sensitivities in PD tumoroids mimicked clinical patient responses, we performed ex vivo drug pharmacotyping in all samples (Fig. 3a). Based on established first-line therapy recommendations for GEP-NEC and high-grade GEP-NET patients^5^, we screened all PD tumoroids for their ex vivo sensitivity to cisplatin (CPT) or temozolomide (TEM) chemotherapy (Supplementary Fig. 4a, b, Supplementary Table S6). For both treatments, PD tumoroid drug sensitivities varied between patients. The ex vivo responses from naïve-passage PD tumoroids were converted into parametrized drug sensitivities using growth rate (GR) adjusted metrics^16^ to account for differences in proliferation rates among samples, based on state-of-the-art protocols for tumor organoid and other 3D-culture screens^17^.

Growth rate adjusted drug sensitivities for increasing drug concentrations after 168 h of treatment were summarized as the area over the GR curve (GR AOC). Based on median GR AOC per drug, patient samples were classified into high or low sensitivity groups (Fig. 3b). The ex vivo sensitivity we observed in PD tumoroids was consistent with the patient’s response to clinical therapy (Fig. 3c and Table 2). For those cases in which we could directly compare the patient’s nearest clinical responses - (±2 months) post- and/or pre-operative to the cryo-specimen collected from the patients—we found sensitivity in the PD tumoroids mimicked clinical patient responses for both temozolomide (*n* = 2) and cisplatin (*n* = 1) therapy (Fig. 3b, c). The functional readout derived from the screen also complemented the pathological and clinical features (Ki-67 index, differentiation, TP53/RB1/KRAS mutational status, MGMT promoter methylation status) (Supplementary Fig. S4c) that recommendations suggest be consolidated *before* selecting a therapy in individual cases^5^.

Patients whose response to systemic therapy was accentuated and long-lasting (C8802p and aP321m) also exhibited high ex vivo drug sensitivity (Fig. 3b, c, Table 2). PD tumoroids from these patients were exclusively sensitive to either cisplatin- or temozolomide-based treatment but not both (Fig. 3b, c), which aligned with their clinical records. These findings suggest that patient-specific drug sensitivities and inter-patient susceptibilities are retained in PD GEP-NEN tumoroids and that cultured PD GEP-NEN tumoroids provide sensitive and direct functional information on ex vivo drug responses in individual patients.

### Transcriptional perturbational profiling in high-grade GEP-NEN PD tumoroids defines adaptive stress response to chemotherapy

We then sought to determine if molecular perturbation profiles from PD tumoroids could provide mechanistic insights into adaptive stress responses and reveal treatment vulnerabilities. To accomplish this, we generated transcriptional perturbation profiles from matched PD tumoroids after DMSO control, cisplatin, or temozolomide treatment. Earlier gene expression studies found that focusing on sublethal drug concentrations prevented artificially exaggerating non-specific cellular stress or death processes caused by high drug dosages^18,19^. Hence we analyzed sublethal concentrations of cisplatin (0.53 uM) and temozolomide (11.52 uM) to determine the drug-related mode of action. PCA of gene expression profiles of treated PD tumoroids revealed that patient-specific expression differences were greater than cisplatin- or temozolomide-induced expressional effects (Supplementary Fig. S5a). Grouping the cohort based on changes in their global gene expression did not clearly separate PD tumoroids with high- and low cisplatin- or temozolomide ex vivo sensitivities. (Supplementary Fig. S5b). Neither did the magnitude of gene expression changes correlate with ex vivo sensitivity (Supplementary Fig. S5c). We considered that treatment-independent sources of variation precluded the detection of correlations and hence sought to factor in such sources using surrogate variable analysis (SVA)^18^. As we expected, these surrogate variables correlated with known biological variables, including patient age, gender, tumor type, Ki-67 index, and sequencing depth (Supplementary Fig. S5d, e).

**Fig. 4:**
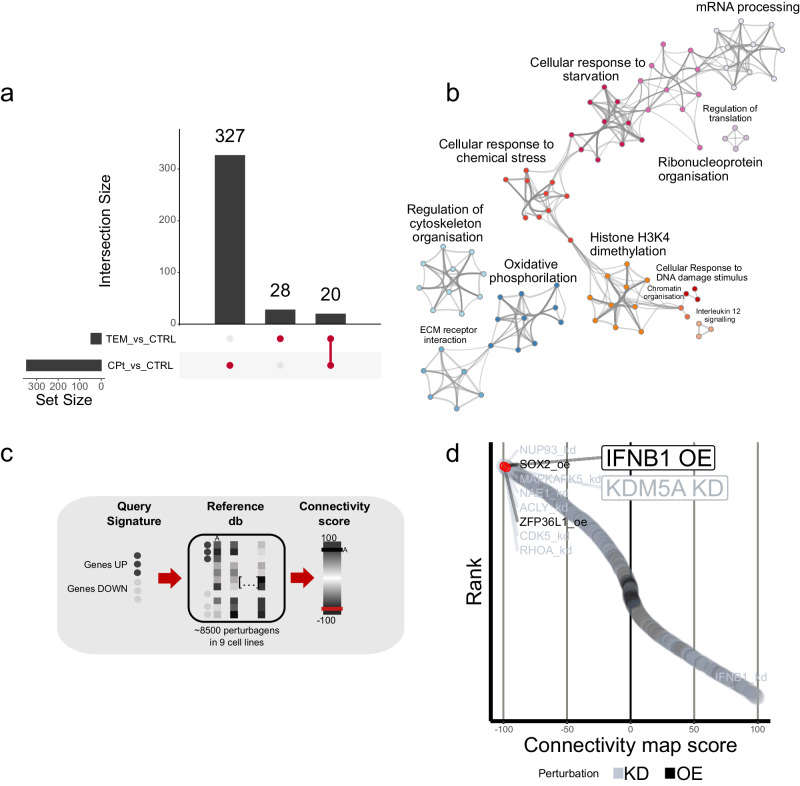
Molecular stress response in patient-derived tumoroids reveals IFNB1 and KDM5A as targets for combination therapy with cisplatin. **a** Intersection of genes differentially expressed between cisplatin (CPT) and control (DMSO) treated or temozolomide (TEM) and control (DMSO) treated PD tumoroids respectively. Bar height and numbers in the main plot shows the size of intersection sets indicated by the connected dots on the *x* axis. Bar height on the *y* axis indicates the size of the individual gene sets. **b** Network representation of pathways significantly enriched (*p* < 0.01, enrichment factor > 1.5) among genes differentially expressed with cisplatin treatment (*n* = 327). Enrichment analysis was performed in using a hypergeometric test. Pathways were clustered based on similarity using metascape. Each node represents an enriched term and is colored by its functional cluster. Edges indicate the similarity between terms. **c** Schematic diagram of connectivity map (cMap) workflow to detect connectivity between stress response signatures from PD tumoroids and perturbational signatures in the database. The top 150 significantly up- or down-regulated genes were matched to the cMap reference data. The similarity to a signature is calculated as the connectivity score τ (range: −100 to 100). **d** Waterfall plot of cMap signatures compared to the cisplatin-induced stress response in PD tumoroids. Signatures were ranked by connectivity score (τ) assigning the highest rank to the lowest τ. A more negative τ indicates a signature with a stronger inverse relation to the query signature. The 10 signatures with the highest rank (most negative connectivity score) are labeled. Overexpression (oe) of IFNB1 and knockdown (kd) of KDM5A are highlighted further.

Notably, we found ex vivo sensitivity was associated with the surrogate variables (Supplementary Fig. S5d); testing differential gene expression and factoring in all surrogate variables, yielded a clear cisplatin-induced perturbation signature (327 DEGs, FDR = 0.1, *p*-adj < 0.05) (Fig. 4a, Supplementary Table S7), and greatly enriched significant *p* values in the *p* value distribution (Supplementary Fig. S4f). Since differential expression results for temozolomide were smaller (28 DEGs, FDR = 0.1, *p*-adj < 0.05, Supplementary Fig. S5f, Supplementary Table S7), we focused only on cisplatin in our subsequent analyses.

Pathway (REACTOME; KEGG; WIKI) and GO over-representation analysis on cisplatin-induced perturbation signatures revealed well-known underlying biological themes such as response to chemical stress or DNA damage (Fig. 4b), DNA repair, and apoptosis (Supplementary Fig. S5g)^20^. Histone H3K4 methylation also prominently contributed to the perturbation gene signature (Fig. 4b), suggesting the possible relevance of epigenetic targets. To explore the underlying biological themes further, we compared the cisplatin-induced perturbational signature to the Connectivity Map (cMap)^19^, a large perturbation signature database (Fig. 4c). When we focused on pathways annotated in cMap as “DNA directed compounds,” we found Amonafide (a DNA intercalating agent) was among the top-ranked compounds and had very high connectivity score (τ = 96.05) while temozolomide (a DNA alkylating agent) had a nearly neutral connectivity score (τ = −6.38). These findings corroborate the specificity of the cisplatin-induced perturbation signature (Supplementary Fig. S5h, Supplementary Table S7).

### IFNB1 and KDM5A genetic perturbation induces inverse expression signatures to cisplatin chemotherapy of high-grade GEP-NEN PD tumoroids

Cancer escape mechanisms and the inevitable emergence of resistance to monotherapies make it imperative to formulate effective combinational chemotherapies, which are now fundamental to modern cancer therapy^20–24^. We used transcriptional perturbation profiles from treated patient-derived GEP-NEN tumoroids to characterize the cisplatin-induced perturbation signature and identify possible combinational treatment options. To prioritize and evaluate complementary combinations, we examined perturbation candidates that created gene expression signatures inversely related to cisplatin-treated PD tumoroids. Overexpression of Interferon Beta 1 (*IFNB1*) and knock-down of Lysine Demethylase 5 A (*KDM5A*) in cMap’s core cell panel (3147 genetic perturbations) were among the top-ranked perturbational candidates, with highly inverse connectivity map scores (*IFNB1*, rank 15, τ = −99.54; *KDM5A*, rank 52, τ = −97.68) (Fig. 4d; Supplementary Table S8) and this pattern was robust and specific (Supplementary Fig. S5i; Supplementary Table S8). Both, KDM5 isoforms and IFNB1 receptors (IFNAR1 and 2) mRNA, were expressed in PD tumoroids (Supplementary Fig. S5j). Together, these findings indicate that molecular stress responses in PD tumoroids are specific and can be exploited to, in silico, predict treatment vulnerabilities.

### In silico-predicted combinational therapies induce effective and synergistic treatment responses in patient-derived GEP-NEN tumoroids

**Fig. 5:**
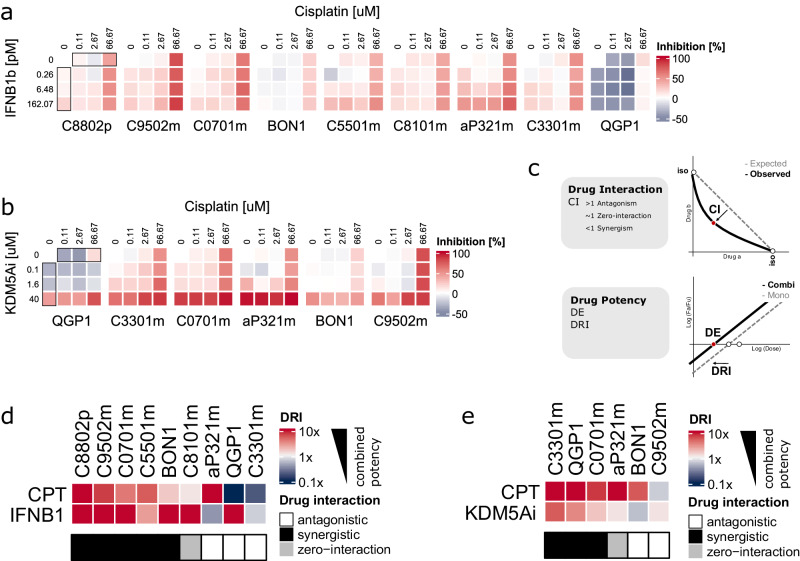
Combinational treatment of cisplatin and KDM5A or IFNB1 induces synergistic and potent treatment response ex vivo. **a** Drug response heatmap of mono- and combinational treatment of cisplatin and recombinant IFNB1b. The short-term treatment response was assessed after 24 h RTG measurements were normalized to DMSO and converted to inhibition values by subtracting the normalized percentage from 100. Red and blue indicate inhibition (reduced growth) and lack of inhibition (increased growth) respectively. **b** Same as in A for the combination of cisplatin and KDM5A inhibitor (CPI-455). **c** Schematic representation of parameters to assess combination therapy. Drug interaction was assessed by determining the combination index (CI) as the deviation of the observed drug combination activity from a purely additive null model (dashed line between the drug concentrations required in isoactive monotherapy (iso)) at a defined effect level. CI values above 1 indicate antagonism and values below 1 synergism. The drug specific effect level (DE) is the drug dose necessary to achieve 50% inhibition. Drug potency (drug reduction index, DRI) was calculated as the fold-change in drug dose between combination- and monotherapy at 50% inhibition. **d** Heatmap displaying drug potency parameters for the combination therapy of cisplatin and IFNB1b. The color scale shows the drug reduction index (DRI). Red (high DRI) or blue (low DRI) indicate an increase or decrease in the drug dose required to achieve 50% inhibition compared to monotherapy. The combination index (CI) at 30% inhibition (Supplemental Fig. S6b) was used to classify drug interaction into synergistic (black), antagonistic (white), or zero-interaction (gray). **e** Same as in D for the combination of cisplatin and KDM5A inhibitor (CPI-455).

To evaluate the functional activity of in silico-predicted candidates in combinational drug therapy, we applied either human recombinant IFNB1b or KDM5A-inhibitor CPI-455 with cisplatin in high-grade PD tumoroids and NEN cell line spheroids. We found that high-grade GEP-NEN tumoroids were susceptible to mono- and combination treatment respectively (Fig. 5a, b). Activity of the KDM5A inhibitor in vitro was confirmed in NEN cell lines by western blotting for methylated Histone 3 (Supplementary Fig. S6a). We then used the inhibitory effect of cisplatin monotherapy at a physiologically relevant concentration (Cmax 14.4 uM; inhibition 0.29 ± 0.24, mean ± SD) as a reference level for comparing drug interaction and drug potency among tumoroids. To analyze synergistic drug interaction and combined drug potency we used the combination index theorem^25,26^. The degree of drug interaction was determined relative to a purely additive null model and summarized as the drug combination index (CI) (Fig. 5c). The dose reduction achieved in the combination treatment is quantified in the drug reduction index (DRI) (Fig. 5c).

In line with our in silico findings, exposure to CPT + IFNB1b combination treatment indicated synergistic drug interaction in five of the screened tumoroids ex vivo (5/9) (CI = 0.43 ± 0.32, mean ± SD) (Supplementary Fig. S6b). Similarly, exposure to the CPT + KDM5A inhibitor combination yielded synergistic drug interaction in three tumoroids ex vivo (3/6) (CI = 0.43 ± 0.23, mean ± SD) (Supplementary Fig. S6c). In PD tumoroids where synergy was detected, combinational dosages needed to obtain equipotent inhibitory effects were considerably lower than in monotherapies (Supplementary Fig. S6d, e), showing highly favorable dose-reduction indices (DRI ≫ 1) for each individual drug (Fig. 5d, e) and emphasizing the increased potency of combination therapy ex vivo.

Altogether, our findings show that NEN PD tumoroid ex vivo drug screening and perturbational profiling can be successfully applied for the timely assessment of standard-of-care therapies and the likely effects of experimental drugs. Our analysis of therapy-induced adaptive stress responses revealed two clinically attractive co-vulnerabilities, which proved that our findings have direct functional significance for patient-derived and cell line GEP-NEN tumoroids.

## Discussion

Therapeutic target discovery, validation, and translational applications face severe obstacles in rare cancers such as high-grade GEP-NEN. The selection of therapies for high-grade GEP-NENs is largely based on clinical experience in the absence of large clinical trials^1,11^ and the absence of predictive biomarkers for therapy^5^. Our data demonstrate that high-grade GEP-NEN PD tumoroids are well suited for rapid ex vivo pharmacotyping and provide biological information on this lethal malignancy. Pharmacotyping may also provide useful therapeutic information, helping oncologists select the best therapies for high-grade GEP-NENs.

The lack of existing preclinical disease models is a major hurdle in the study of rare cancers^12,13^. We combined a patient-derived model system of high-grade GEP-NEN, extensive characterization of matched tumor tissues, and comprehensive patient clinical follow-up for the study of these rare cancers. PD tumoroids provided a faithful representation of defining features of high-grade NENs. Inter-patient molecular transcriptional patterns were retained in tissue-matched PD tumoroids, further demonstrating that key biological features are recapitulated in the model (Fig. 2). The relevance of patient-derived models is underscored by the clear difference between transcriptomes of classical NEN cell line compared to the patient material and PD tumoroids.

PD tumoroids of high-grade GEP-NEN patients mimic patient response to established first-line chemotherapies (Fig. 3, Supplementary Fig. S4). Nevertheless, we are aware of the limited size of our sample collective, further studies with a larger number of samples and pretreatment biopsies are needed.

While our cohort was small, our results align with those of similarly sized studies of similar sizes of various cancer entities that demonstrated clinical applicability using patient-derived ex vivo models, e.g., in colorectal cancer^27,28^, pancreatic cancer^29^, and lung cancer^30^.

We efficiently and successfully processed low abundant GEP-NEN tissues with minimal cell requirements, included critical quality control steps, and ensured turnaround time was only 2 weeks (Figs. 2, 3, Supplementary Figs. S2–4)—far less than the 2 to 6 months reported in other precision medicine studies^30,31^. Because patients with high-grade NEN are not expected to live long without effective treatment, a rapid turnaround using PD tumoroids better matches the clinical course of these patients. As a trade-off for rapid information, our workflow and model are focused on “one round of experimentation” per preparation. The rapid ex vivo culture and analysis of individual tumor specimens can however be expanded with additional screens if sufficient (i.e., biopsy-sized) additional donor tissue is available. The amount of tissue needed for a targeted rapid ex vivo screen is similar to the amount needed for an additional tumor biopsy, which facilitates translational applications. Other groups, e.g., Sato et al., have successfully generated NEN organoid lines as excellent models for comprehensive mechanistic studies^15^. However, the organoid expansion process took from several months to years^15^.

Our research lays the groundwork for prospective validation of patient-derived tumoroids as faithful ex vivo models for personalized screening of treatment efficacies. Larger prospective studies could evaluate the predictive relevance in more detail.

Molecular drivers of the divergent clinical course of G3 NET and NEC are poorly understood, making individual treatment decisions challenging. Because advanced tumors often resist monotherapies, antineoplastic agents are combined to be more efficacious at lower doses^20–23,28,30–32^. Patient-to-patient heterogeneity, intra-tumoral heterogeneity, and intracellular pathway dysregulation open new avenues for combining therapies to induce potent responses that monotherapy cannot achieve. We offer a strategy for using gene expression profiles to suggest treatments that can be combined with cisplatin chemotherapy; cisplatin-induced molecular stress response in high-grade GEP-NEN PD tumoroids is specific and mirrors perturbational effects (Fig. 4, Supplementary Fig. S5). Using perturbational profiles we pinpoint two novel candidates for combinational therapy: Lysine Demethylase 5 A (KDM5A) and interferon beta 1 (IFN1B) (Fig. 4, Supplementary Fig. S5).

KDM5A is a histone demethylase that often represses target genes at transcriptional start sites^33^ and its role in neuroendocrine differentiation and tumorigenesis was recently described^34,35^. Kaelin et al. demonstrated that Kdm5a promotes SCLC tumorigenesis in vivo and tumor proliferation and proposing inhibiting KDM5A as a therapeutic strategy^35^. Genomic analysis of GEP-NENs has shown that in 45% - 52% of the tumors there is a KDM5A copy number gain^10^. The findings of these two independent studies closely align with our finding that KDM5A plays a prominent role in neuroendocrine neoplasms. Upon combinational treatment of KDM5A inhibitor with cisplatin, three GEP-NENs we tested showed strong synergism and clinically attractive efficacies (Fig. 5, Supplementary Fig. S6).

Interestingly, KMD5A and cisplatin susceptibility have a functional relationship in lung adenocarcinoma, pointing towards altered chromatin regulation as a potential molecular mechanism for drug tolerance^36^. Note that the sample in which the Cisplatin+KDM5A combination was ineffective had a mutational disfunction upstream of the H3K4 methylation axis. Mutations in lysine methyltransferase 2 A (KMT2A) and menin (MEN1) regulate H3K4 methylation, so this dysfunction may have rendered the combination ineffective.

Type I interferons (IFN-α and IFN-β) are pro-inflammatory cytokines that can rapidly cause myriad downstream effects in tumor cells and promote antitumor immunity in immune cells^37,38^. Type I interferons activate transcription factors of the signal transducer and activator of transcription (STAT) family, initiating protein synthesis from interferon-stimulated genes^38^. Type 1 interferons are FDA-approved for mono- or combinational therapy because they cause tumor regression and may prolong survival in many other highly proliferative hematological and disseminated solid malignancies^37^. IFN-α was used to treat advanced low-grade GEP-NETs^39–41^ but was superseded by other regimens (e.g., somatostatin analogs)^42^. Recently, two independent studies proposed that IFN-β be used to treat GEP-NETs because at low doses it effectively inhibits cell proliferation and stimulates apoptosis in cell lines in vitro^43,44^. In the clinically more relevant scenario of patient-derived high-grade GEP-NET tumoroids, we found IFNB1 was associated with the GEP-NEN perturbational signature. Exposure to Cisplatin+IFNB1 revealed they were synergistic and highly efficacious in treating a subset of high-grade GEP-NEN tumoroids (Fig. 5, Supplementary Fig. S6). This combinational approach may be an attractive option for patients with high-grade GEP-NETs, who now have few treatment options^3,5^.

Further studies in larger cohorts are needed to determine to what extent KDM5A- or IFNB1 combinations are NEC- or NET G3 specific and further efforts are needed to delineate the exact mechanisms behind treatment susceptibilities. Exact treatment schedules and/or therapeutic priming should also be evaluated in vitro. A recent extensive and comprehensive high-throughput combinational drug screen in breast, colon, and pancreatic cancer indicated that chemotherapeutics combined with apoptotic inducers or cell cycle inhibitors show promise for translational applications^21^. Both KDM5A and IFNB1 fall into this category, and our study underlines their functional potency. KDM5A and IFNB1 may prove to be the Achilles Heels for high-grade GEP-NEN if combined with cisplatin.

In summary, we successfully cultured PD tumoroids of high-grade GEP-NENs for a rapid ex vivo drug screen. These tumoroids recapitulated key biological features of high-grade GEP-NEN and mimicked clinical response to cisplatin and temozolomide ex vivo. We also investigated molecular stress responses in PD tumoroids in silico, discovering and functionally validating Lysine demethylase 5 A (KDM5A) and interferon-beta (IFNB1)—two vulnerabilities that interact when combined with cisplatin. Either KDM5A or IFNB1 can be combined with cisplatin, opening new therapeutic options in high-grade GEP-NENs.

Our findings, that GEP-NEN PD tumoroids are promising candidates for rapid and biologically meaningful ex vivo pharmacotyping and that they can provide subsidiary therapy information, brings us closer to developing more personalized clinical protocols for later-line therapies in patients with aggressive high-grade GEP-NEN.

## Methods

### Patient studies

We assembled a cohort of eight high-grade GEP-NEN patients from two ENETS Centers of Excellence; The University Hospital Charité Berlin (Germany) and The University Cancer Institute of the Inselspital and the University of Bern (Switzerland). Inclusion criteria were histopathologic diagnosis of G3 gastroenteropancreatic neuroendocrine neoplasm, availability of both tumor tissue- and matching cryomaterial for ex vivo culture, and tumor purity of >70%. A board-certified pathologist (A.P.) reviewed all cases and reclassified them according to WHO 2019 criteria (ISBN 978-92-832-4499-8) (Table 1 and Supplementary Table S1). TNM staging was based on the 8^th^ edition UICC/AJCC (ISBN: 978-1-119-26356-2). We obtained treatment and outcome information from interdisciplinary NEN tumor board records of both centers. Assessment of clinical therapy response accorded with investigator based RECIST criteria. The final classification was based on all information about immunohistochemistry, clinical course records, and mutational status from targeted sequencing. The cohort included 3 female and 5 male patients; their ages varied from 39 to 70 years (mean = 58.0; SD = 11.8). For comprehensive cohort features, patient demographics, and patient characteristics, see Tables 1, 2 and Supplementary Table S1. Chemotherapy indication was based on clinical judgement and patient preferences. The specimens were processed as described in April-Monn, et al.^14^. In brief, upon surgical resection a pathologist processed the left-over of the donor tumor tissue to 8-mm^3 cubes under sterile conditions, avoiding necrotic regions when possible. These cubes were suspended in recovery cell culture freezing medium (Thermo Fisher Scientific, USA), cryopreserved in an isopropyl alcohol freezing container (Nalgene, USA), and stored in liquid nitrogen. A consecutive block was snap-frozen (freshfrozen) in liquid nitrogen. A mirror block was fixed in formalin and embedded in paraffin. The study was approved by cantonal authorities (Kantonale Ethikkomission Bern, Ref.-Nr. KEK-BE 105/2015) in accord with the Swiss Federal Human Research Act and by the ethics committee at Charité Universitätsmedizin Berlin (Ref.-Nr. EA1/229/17) which both ensure adherence to the Declaration of Helsinki. All patients included in this study signed an institutional informed consent for the use of their residual samples for future research. However, informed consent was not possible for this specific study since it was developed up to 10 years after the patient’s death.

### Cancer mutation panel

The TruSight Oncology 500 Kit (TSO500, Illumina) was used for DNA library preparation and enrichment following the manufacturer’s protocol. DNA (80 ng) were sheared on a Covaris E220 ultrasonicator. DNA fragments were end-repaired, and adapters containing unique molecular identifiers (UMIs) were ligated to each fragment end. Fragments enriched by capture hybridization were analyzed by high-throughput sequencing on a NovaSeq 6000 instrument (Illumina). TSO500 alignment and variant calling were performed using the TSO500 bioinformatics pipeline v2.1.0. UMI-filtered total read counts were 103 M ± 19 M, median exon coverage was 1131 ± 253, median DNA-insert size was 136 ± 14, and % aligned reads were 98.9 ± 1.0. Sources of population frequencies that were used for auto-classification of benign variation include gnomAD (RRID:SCR_014964) and ExAC (RRID:SCR_004068). We retrieved annotations of oncogenic effects of identified variants from the OncoKB precision oncology knowledge database (RRID:SCR_014782) and assessed known activating mutations in oncogenes and inactivating mutations in tumor suppressors (Tier 1 and Tier 2). For the few cases in which IHC was inconclusive, we investigated both known mutations and mutations with unknown and unclear effects (Tier 3). OncoPrint function from ComplexHeatmap v2.6.2^45^ (RRID:SCR_017270) was used for visualization.

### Primary and cell line culture

For the study, we focused on naïve passage PD tumoroids to minimize clonal drift^46^ and used NEN cell line spheroids for comparison. All therapeutic studies were completed in 12 days. All screening plates contained vehicle control wells (DMSO-treated, *n* = 10) and blank wells (medium-only, *n* = 6) and for each plate, the raw luminescent intensity values were normalized to a relative scale using the blank (B) value. Luminescence was measured relative to the baseline of each well (BC) (Relative scale = (Luminescence of treated cells − B)/(BC − B)).

### Primary cell isolation and culture

Cryopreserved tumor tissues were used for ex vivo drug screening. For primary cell isolation, micro-cell block manufacture, and quantification, we followed the workflow described previously^14^. In brief, cryopreserved tissues were dissociated with a gentle MACS® dissociator (Myltenyi Biotec, Switzerland) in AdvDMEM medium, 0.25% Trypsin (Sigma-Aldrich, Switzerland), 10 mg/ml collagenase IV, (Worthington, USA), 10 mg/ml DNAse (Roche, Switzerland). Isolated primary tumoroids were maintained in AdvDMEM plus 5% FBS, Hepes 10 mM, 1% L-glutamine, 1% penicillin-streptomycin-amphotericin B and growth factors (20 ng/mL EGF, 10 ng/mL bFGF (Thermo Fisher Scientific, USA), 100 ng/mL PlGF, 769 ng/mL IGF-1 (Selleckchem, USA)) in ultra-low attachment (ULA) plates. After dissociation red blood cells were lysed for 3 min with ACK lysis buffer (Thermo Fisher Scientific, USA) at room temperature. Fibroblast were segregated by attachment, incubating the cells at 37 for 1 h in coated plates. Supernatant was collected and plated on ULA plates to recover for 48 h. After 2 days of recovery phase, cellular aggregates were collected and centrifuged at 120 g for 5 min to separate cells and aggregates from debris/apoptotic cells. Cells were counted and resuspended in fresh AdvDMEM + GF medium supplemented with 123 μg/mL growth-factor-reduced Matrigel and plated in 96-well ULA plates (50 μL/well, 3000–4000 cells/well). In this study of high-grade GEP-NENs and in our earlier studies of lower-grade PanNENs^14,47^, our definition of “culture success” for patient-derived tumoroids was based on six factors that support translational application of patient-derived GEP-NEN tumoroids: (1) Successfully isolating and culturing viable tumor cells; (2) retaining ± 70% of the isolated cells before drug screening; (3) passing quality controls, including cytological, morphological, and histopathological examinations of clinically applied neuroendocrine marker expression in micro-cell-blocks; (4) attaining sufficient technical replicates (*n* ≥ 4) in drug screenings; (5) attaining stable RealTime- Glo™ (RTG) baseline and cell growth; (6) and extending culture life spans of up to 12 days ex vivo.

### NEN cell line culture

The QGP1 cell line (RRID:CVCL_3143) was purchased from the Japanese Health Sciences Foundation in 2011. QGP1 cells were kept in RPMI 1640 medium (10% FBS, 100 IU/mL penicillin, 0.1 mg/mL streptomycin). The BON1 cell line (RRID:CVCL_3985) was provided by E.J.M. Speel in 2011. BON1 cells were cultured in DMEM-F12 (10% FBS, 100 IU/mL penicillin, 0.1 mg/mL streptomycin). NT3 cells were provided by J. Schrader and kept in RPMI 1640 + growth factor medium (10% FBS, 100 IU/mL penicillin, 0.1 mg/mL streptomycin, 20 ng/mL EGF, 10 ng/mL bFGF) and cultured in collagen IV coated culture flasks^48^. All cells were kept in a humidified incubator at 5% CO2 and 37 °C and cultured for no longer than 2 months. For all cell lines, short tandem repeat (STR) analysis by PCR was performed (QGP1 in 2011/2016/2020; BON1 in 2014/2016/2020; NT3 in 2018/2020). QGP1 cells were authenticated by their specific cancer cell profile. A BON1- or NT3-specific cancer cell profile does not exist yet, but contamination with other common cell lines can be excluded due to non-match to any known cancer cell line profile. Expression of the specific neuroendocrine markers chromogranin A and synaptophysin were routinely tested by IHC on cell blocks.

### Compounds

Temozolomide (#S1237, Selleckchem), cisplatin (#4333164, Teva Pharma), CPI-455 (#S6389, Selleckchem), IFNB1b (#I7662-14S, Biomol) were obtained from commercial vendors and stored as stock aliquots, as indicated by the manufacturers. We selected drug concentrations for chemotherapeutics (cisplatin; temozolomide) based on physiologically relevant concentrations at each drug’s Cmax (the maximum tolerated serum concentration of each drug, drawn from published human studies)^49^. We based concentrations for combinational exploratory compounds (CPI-455, IFNB1b) on primary literature and in-house in vitro testing of a 625-fold concentration range, optimized to induce a range of responses across classical NEN cell line spheroids (BON1, QGP1). Compounds were screened at equidistant 5-point, 625-fold concentration ranges using four technical replicates for long-term (168 h) chemotherapeutics screens or in equidistant 3-point, 625-fold concentration ranges with three technical replicates for short-term (24 h) combinational screens^25^.

### Ex vivo drug screening

3000–5000 cells were plated per well. Cell viability was quantified with RealTime-Glo™ MT Cell Viability (RTG) Assay (Promega, #G9712). Assay plates were incubated for 72 h at 37 °C in a humidified atmosphere at 5% CO2 to allow sphere formation.

### Evaluating drug sensitivity to mono chemotherapeutics

After a baseline measurement (Day 0), we tested spheroids with titrations of cisplatin, temozolomide, or DMSO (0.16% v/v) as vehicle control. Assay plates were incubated, and RTG luminescence measurements were recorded at 96 h and 168 h with an Infinite 200 PRO plate reader (Tecan). A blinded experimenter scored ex vivo experiments and sensitivities to treatments. GR metrics: Raw luminescence values were normalized to each individual baseline control value at Day 0 for the same well. Ex vivo responses were converted into parametrized drug sensitivity metrics, as did state-of-the-art protocols^17^, In brief, drug effect estimates were obtained by adjusting normalized luminescence measurements to the growth rate (GR) of the control treated sample using GRmetrics v1.16.0 as described in ref. ^16^. Samples were treated with increasing drug concentrations to obtain a curve of GR values after 168 h of treatment. Drug sensitivity was then calculated as the area over this GR value curve (GR AOC) with larger GR AOC values indicating a stronger inhibition of cell growth. Samples with an GR AOC above the median of the distribution were considered responders and samples with an GR OAC below the median distribution were considered non-responders (2016)^16^.

### Evaluating drug sensitivity to combination therapy

After baseline measurement (Day 0), PD tumoroids or cell line spheroids were dosed with titrations of cisplatin (0.11, 2.67, 66.67 uM), recombinant IFNB1b (0.26, 6.48, 162.07 pM), or CPI-455 (0.1, 1.6, 40 uM) alone or all combinations. We incubated assay plates and recorded luminescence measurements at 24 h with an Infinite 200 PRO plate reader. Raw luminescence values in the presence of the drug were normalized to baseline control values and DMSO-treated controls at 24 h. Technical replicates were averaged to yield a mean relative cell count per condition. The analysis of drug-drug interaction was based on the combination index theorem, a mechanism-independent model for assessing drug interaction and drug potency^25,26^. The combination Cisplatin plus IFNB1b was evaluated in a ratio of 411365:1 and Cisplatin plus CPI-455 in a ratio of 1.67:1 accounting for differences in effective doses. The combination index (CI) can be derived by comparing the concentrations in the combination treatment required to achieve a fixed fractional inhibition effect (fraction affected / fraction unaffected) to a purely additive null model. Values above 1 indicate antagonism and values below 1 synergism. The drug reduction index (DRI) indicates the relative change in concentration per drug required to obtain the same fractional effect in monotherapy. CompuSyn v1.0 was used to calculate drug interaction and drug potency metrics^26^. We summarized the degree of drug interaction by drug combination indices (CI), and then used an isobologram to describe how the drug combination activity we observed deviated from isoactive monotherapies^25^. The median-effect equation was used to derive drug potency parameters (Dose Reduction Index [DRI]; Effect at Dose X [DE])^25^.

### Nucleic acid extraction

A DNA Purification Micro Kit (Norgen Biotek, #50300) was used to extract genomic DNA from fresh frozen tumor tissue. Total RNA was extracted from fresh frozen tumor tissue or cultured cells with a Single Cell RNA Purification Kit (Norgen Biotek, #51800). Nucleic acid quantification was performed with the Qubit DNA/RNA HS detection kit (Thermo Fisher Scientific, #Q32852). We used a Femto Pulse system with an Ultra Sensitivity RNA kit (Agilent, #FP-1201-0275) to analyze quality control metrics.

### Immunohistochemistry

All the IHC markers were repeated on freshly cut tissue blocks and re-evaluated by a NEN expert pathologist (A.P.). For immunohistochemistry, we cut the paraffin-embedded material into 2.5-µm-thick serial sections and then deparaffinized, rehydrated, and retrieved antigens with an automated immunostainer (Bond RX, Leica Biosystems). Antigen retrieval was performed in a Tris-EDTA buffer for 30 min at 95 °C for Ki-67 (1:200, Dako, M7240), ATRX (1:400, Sigma-Aldrich, HPA001906), MCT4 (1:50, Santa Cruz, sc376140 D1), SOX9 (1:100, Cell Signaling, 82630 T D8G8H), ARX (1:1500, R&D Systems, AF7068), PDX1 (1:100, R&D Systems, MAB2419); in a Tris-EDTA buffer for 30 min at 100 °C for synaptophysin (1:100, Novocastra, 27G12), CgA (1:400, CellMarque, 238M-94 LK2H10), SSTR2A (1;50, BioTrend, SS-8000-RM UMB-1); in a proteinase K solution for Trypsin 1 (1:20000, Chemicon, MAB1482); in a citric buffer for 30 min at 100° for DAXX (1:40, Sigma-Aldrich, HPA008736), RB1 (1:200, BP Pharmingen, 554136 G3-245); and, in a citric buffer for 20 min at 95 °C for TP53 (1:800, Dako, M7001 DO-7), BCL-10 (1:1000, Santa Cruz, sc-5273 331.3). Primary antibody incubation lasted 30 min at the specified dilutions. Visualization used a Bond Polymer Refine Detection Kit (Leica, #DS9800) (RRID:AB_2891238) for visualization; DAB (3,3′-Diaminobenzidine) was the chromogen. Slides were counterstained with hematoxylin. We used an automated slide scanner Panoramic 250 (3DHistech) at 20x magnification to capture scans and acquired images with QuPath software^50^.

### Bulk RNA sequencing

#### Library preparation and sequencing

Sequencing libraries were prepared from RNA using the SMARTer Stranded Total RNA-Seq Kit v3 for picogram input material (Takara, #634488). Libraries were sequenced as paired-end 101 bp (tumoroid samples) or paired-end 81 bp (original tumor tissues) reads on a NovaSeq 6000 (Illumina) platform at ~30 M reads/sample. Reads were demultiplexed and converted to FASTQ format with bcl2fastq v2.20.0.422 (RRID:SCR_015058). Cutadapt v2.5^51^ (RRID:SCR_011841) was used to trim Illumina adapter sequences and mask 3’ homopolymers longer than 10 bp. We removed reads containing more than 20 masked bases or shorter than 65 bp (tumoroid samples) or 50 bp (original tumor tissue). Trimmed reads were mapped against a custom list of ribosomal RNAs and repetitive RNA elements with bwa v0.7.17^52^ (RRID:SCR_010910); mapping reads to this custom list were discarded. At each step, we used FastQC v0.11.7 (RRID:SCR_014583) to track read quality. Processed reads were mapped to the human genome (GRCh37, GENCODE annotation v37) with STAR v2.7.3a^53^ (RRID:SCR_004463). Mapped reads were deduplicated based on the 8 bp UMI in the R2; we used UMI-tools v0.5^54^ (RRID:SCR_017048) and the default directional method. Deduplicated reads were assigned to GENCODE v37 genes in subread v2.0.1^55^ (RRID:SCR_009803). We excluded one drug-treated sample from the tumoroid culture of patient C5501m because input and library quality was low.

#### Differential gene expression

For the comparison of original tumor tissue and PD tumoroids expression data was normalized using the trimmed mean of M values (TMM). Differentially expressed genes were then determined using limma v3.48.1 with voom precision weights^56^ (RRID:SCR_010943). The repeated measurements of tumor tissue and tumoroids from the same patient were modeled using the duplicateCorrelation function in limma, treating the patient as a random effect. For drug-treated tumoroids, we determined differential expression with DESeq2 v1.32.0^57^ (RRID:SCR_000154). Treatment-independent expression variability was modeled using surrogate variable analysis (SVA) from sva v3.40.0^58^ (RRID:SCR_002155). All available surrogate variables were added to the DESeq2 model. Log2 expression fold changes of highly variable genes were shrunk with the apeglm v1.14.0 algorithm^59^.

#### Functional enrichment analysis

UpSetR v1.4.0^60^ was used to visualize intersections between gene sets. Gene set enrichment analysis (GSEA) on differential expression results from original tumors vs PD tumoroids or cisplatin vs DMSO treated PD tumoroids was performed in clusterProfiler v.3.18.1^61^ (RRID:SCR_016884). GO terms were obtained from the human annotation package org.Hs.en.db v3.13.0, Hallmark gene sets were obtained from MSigDB v7.5.1. Genes were ranked on the test statistic from the respective differential expression test. Terms smaller than 100 or larger than 400 elements were excluded. Additional functional analysis on the 327 differentially expressed genes from cisplatin and DMSO treated PD tumoroids was performed in metascape, data base update 2022-01-01^62^. All genes in the genome were used as the enrichment background. Significant pathways (*p* < 0.01, enrichment factor >1.5) were clustered by similarity into thematic groups.

#### Perturbational profiling in cMap

We compared the top and bottom 150 genes from drug versus control-treated tumoroids (adjusted *p* < 0.05, sorted by the Wald statistic) to the compendium of perturbational reference signatures from Connectivity Map (L1000, Touchstone v1.0)^63^ (RRID:SCR_015674) and extracted connectivity map scores (τ) for all available knock-down (kd), overexpression (oe), and compound perturbagens. To estimate the robustness of matching signatures, we rarified or permutated the input lists of differentially expressed genes.

### Western blotting

BON1 and QGP1 cells were seeded to 6 well plates (800 K cells / well). The next day cells were treated with DMSO or CPI-455 (0.1 uM, 1.6 uM, 40 uM) for 24 h. Histones were extracted using an acid extraction protocol as described previously^47^ and the same Bio-Rad system was used for protein quantification, Western blotting and imaging. The primary antibodies histone H3 (1:5000, Abcam ab12079) was diluted in 5% BSA-TBST. The primary antibody H3K4me3 (1:2000, Abcam ab8580), the secondary antibodies DyLight 650 conjugate goat anti-rabbit (ImmunoReagent, GtxRb-003-D650NHSX) and HRP-conjugated rabbit Anti-Goat (Abcam, ab6741) were diluted in 5% Milk-TBST. Band intensity was measured using ImageJ after background subtraction using a sliding paraboloid with size 100 pixel. Uncropped and original membranes are shown in Fig. S7.

### Reporting summary

Further information on research design is available in the Nature Research Reporting Summary linked to this article.

### Supplementary information

Reporting Summary

Supplementary Material

## Data Availability

Sequence data that support the findings of this study have been deposited in Gene Expression Omnibus (GEO); primary accession code is GSE213504.

## Code availability

All code is available from the corresponding author upon request. Data collection: RNA-Seq data was processed using bcl2fastq v2.20.0.422. Illumina adapter and homopolymer sequences were trimmed and UMIs extracted with cutadapt v2.5, discarding reads shorter than 65 bp. Reads were mapped to GRCh38, GENCODE v37 with STAR v2.7.3a, UMI-tools v0.5 and subread v2.0.1. Data analysis: All data analysis was performed under R v4.1.0. Differential expression was analyzed using limma voom v3.48.1 with the duplicate Correlation function or DESeq2 v1.32.0 plus sva v3.40.0 surrogate variables. Functional analysis was performed with clusterProfiler v3.18.1, org.Hs.en.db v3.13.0 and MSigDB v7.5.1 or with metascape (update 2022-01-01). Drug response was quantified using GRmetrics v1.16.0. Combination therapies were evaluated with CompuSyn v1.0. Drug perturbation profiles were scored using ConnectivityMap (https://clue.io/) v1.0 on the top 150 up- and down-regulated genes.
