# Supplementary material for: Patient derived tumoroids of high grade neuroendocrine neoplasms for more personalized therapies
Source: NPJ Precis Oncol. 2024 Mar 1;8:59. doi: 10.1038/s41698-024-00549-2 (PMC10907580; doi:10.1038/s41698-024-00549-2)
Supplement: Supplementary file 2 — Supplementary Material [file 41698_2024_549_MOESM2_ESM.pdf]

SUPPLEMENTARY MATERIAL

Supplementary Table 1: Demographics, clinical description, and MSI

Supplementary Table 2: Mutation profiling

Supplementary Table 3: Cytology of patient-derived tumoroids

Supplementary Table 4: Differential expression original tumor tissue vs. PD tumroids

Supplementary Table 5: GSEA of original tumor tissue vs PD tumoroids

Supplementary Table 6: GRmetrics

Supplementary Table 7: Differential expression of drug treatment vs DMSO in PD tumoroids

Supplementary Table 8: cMAP signatures

Supplementary Figures S1 to S7

Supplementary Table 1: Demographics, clinical description, and MSI

Patient_ID	sex	age_at_surgery[y]	tissue_source	primary_tumor_localisation	1_year_survival
C9502m	f	57	liver metastasis	CUP	alive
C8802p[1]	m	66	primarius	stomach	alive
C8101m[2]	f	44	liver metastasis	CUP	alive
C5501m[3]	f	39	metastasis in ovary	CUP	deceased
C3301m	m	70	liver metastasis	colon	lost to follow-up
C0701m	m	69	liver metastasis	pancreas	alive
aP490m[4]	m	53	lymph node metastasis	pancreas	deceased
aP321m[5]	m	66	liver metastasis	pancreas	alive

[1] C8802p: Initially diagnosed as WD NET G3 (year 2006) with no identifiable mitosis and no aggressive morphology; transformation into "secondary NEC" with NEC-like morphology including infiltrative pattern and desı

[2] C8101m was at time of diagnosis was classified as NEN. In a first opinion as NEC and in a second opinion as NET G3.

[3] C5501m: Initially higher Ki-67 index (~60%) combined with an APC frameshift mutation (26 bases) and early stop codon (TGA); SMAD4 statistically significant hot spot oncogenic mutation; initial therapy response to c

[4] aP490m: Likely transformation into "secondary NEC" with NEC-like morphology; MSH6 splice site mutation but MSI-low MSH6 mutation has less significance for this patient; PTEN splice site inactivating mutation (nor

[5] aP321m NET G3 with NEC-like phenotype and likely secondary KRAS activating oncogenic mutation during clinical treatment course; reconfirmation of non-mutated RB1 and non-mutated ATRX (excellent coverage in

Classification	Morphology_IFP_Subtype	histological_differentiation	IHC_IFP_CgA	IHC_IFP_Ki67
NET	NA	WD	moderate positive (++)	50
NET	NA	WD	NA	30
ACC	NA	NA	weak positive (+)	50
NEC	Large-cell	PD	strong positive (+++)	30
NEC	Small-cell	PD	negative (-)	80
NET	NA	WD	moderate positive (++)	80
NEC	Large-cell	PD	strong positive (+++)	90
NET	NA	WD	weak positive (+)	75

moplastic stroma; RB1 mutation (stop codon exon 6) with allele frequency 90%)

isplatin followed by recurrence

n-coding); TSC1 splice site variant synonym mutation (C>T); TGFBR1 nonsense mutation (stop codon exon 4)

TSO500)

IHC_IFP_MCT4	IHC_IFP_PDX1	IHC_IFP_RB1	IHC_IFP_SOX9	IHC_IFP_SSTR2A	IHC_IFP_SYN	IHC_IFP_TP53	IHC_IFP_DAXX
heterogenous (2)	negative (-)	wildtype expr.	negative (-)	moderate positive	strong positive	mutant expr.	NA
NA	NA	NA	NA	NA	NA	NA	NA
heterogenous (2)	negative (-)	wildtype expr.	positiv	negative (-)	negative (-)	wildtype expr.	NA
positive (1)	negative (-)	wildtype expr.	negative (-)	weak positive (+)	strong positive	wildtype expr.	NA
heterogenous (2)	negative (-)	mutant expr.	positive	negative (-)	strong positive	mutant expr.	NA
heterogenous (2)	negative (-)	wildtype expr.	negative (-)	negative (-)	strong positive	wildtype expr.	negative (-)
heterogenous (2)	negative (-)	wildtype expr. pat.	strong positive (+++)	negative (-)	strong positive (+++)	wildtype expr. pat.	positive
negative (0)	positive	mutant expr.	positiv	moderate positive	strong positive	wildtype expr.	negative (-)

IHC_IFP_ATRX	IHC_IFP_ARX	IHC_IFP_TRY1	IHC_IFP_BCL10	IHC_IFP_FF_SYN	IHC_IFP_FF_TRY1
NA	NA	negative (-)	negative (-)	NA	NA
NA	NA	NA	negative (-)	positive (++)	negative(-)
NA	NA	positive	positive	NA	NA
NA	NA	negative (-)	negative (-)	NA	NA
NA	NA	negative (-)	negative (-)	NA	NA
X	negative (-)	negative (-)	negative (-)	NA	NA
positive	negative (-)	negative (-)	negative (-)	NA	NA
positive	positive	negative (-)	negative (-)	NA	NA

Supplementary Table 2: Mutation profiling

**TSO500_TMBtotal_[mt/Mb]**	**MSI_usable_sites**	**MSI_unstable_sites**	**MSI_status**	**TSO500_MEN1**	**TSO500_KMT2A**
3,1	123	1	low	MEN1	*KMT2A*
11,8	122	5	low	NA	NA
6,3	123	2	low	NA	NA
3,1	110	6	low	NA	NA
2,3	123	2	low	NA	NA
0,8	119	5	low	NA	NA
16,4	122	1	low	MEN1	NA
1,6	125	1	low	NA	NA

TSO500_TP53	TSO500_ATRX	TSO500_RB1	TSO500_MAP2K4	TSO500_RAD51D	TSO500_FANCD2	TSO500_VHL	TSO500_MED12
TP53	ATRX	NA	NA	NA	NA	NA	NA
NA	ATRX	RB1	MAP2K4	RAD51D	FANCD2	NA	NA
NA	NA	NA	NA	NA	NA	VHL	MED12 (2x)
NA	NA	NA	NA	NA	NA	NA	NA
TP53	NA	RB1 (2x)	NA	NA	NA	NA	NA
NA	NA	NA	NA	NA	NA	NA	NA
NA	NA	NA	NA	NA	NA	NA	NA
NA	NA	NA	NA	NA	NA	NA	NA

TSO500_SMAD4	TSO500_APC	TSO500_NOTCH4	TSO500_PTEN	TSO500_MSH6	TSO500_TET2	TSO500_TGFBR1	TSO500_TSC1
NA	NA	NA	NA	NA	NA	NA	NA
NA	NA	NA	NA	NA	NA	NA	NA
NA	NA	NA	NA	NA	NA	NA	NA
SMAD4	APC	NA	NA	NA	NA	NA	NA
NA	APC (2x)	NOTCH4	NA	NA	NA	NA	NA
NA	NA	NA	NA	NA	NA	NA	NA
NA	NA	NA	PTEN	MSH6	TET2	TGFBR1	TSC1
NA	NA	NA	NA	NA	NA	NA	NA

TSO500_KRAS	TSO500_MLLT3
NA	NA
NA	NA
NA	NA
NA	NA
NA	NA
NA	MLLT3
NA	NA
KRAS	NA

GeneSymbol	PositionCoding	PositionProtein	Exon	Chr	Ref	Alt	GeneEnsemb	ExistingVariat	Classification	Impact	OKB_MutationEffect	OKB_Oncogenic	TranscriptEns	**AlleleFrequency**	**ReadDepth**	CNVfoldChange	Tier	
PDGFRA									CNV							1,436	T5	C9502.mFF202103
EGFR									CNV							1,449	T5	C9502.mFF202103
**MEN1**	c.125G>A	p.Gly42Asp	exon 2/10	chr11	C	T	ENSG000001	CM981250,C	SNV Missense	MODERATE	Unknown	NA	ENST000003	0,6829	1050		T2	C9502.mFF202103
KMT2A	c.5630C>T	p.Ala1877Val	exon 20/36	chr11	C	T	ENSG000001	rs782094920	SNV Missense	MODERATE	Unknown	NA	ENST000005	0,141	858		T3	C9502.mFF202103
FGF14	c.758A>G	p.Ter253TrpextTer1	exon 5/5	chr13	T	C	ENSG000001	NA	SNV Nonstop	HIGH	Unknown	NA	ENST000003	0,0426	1503		T5	C9502.mFF202103
IGF1R	c.1168A>G	p.Ile390Val	exon 5/21	chr15	A	G	ENSG000001	NA	SNV Missense	MODERATE	Unknown	NA	ENST000002	0,4779	1586		T3	C9502.mFF202103
**TSC2**	c.2296G>A	p.Val766Met	exon 21/42	chr16	G	A	ENSG000001	rs150672640,	SNV Missense	MODERATE	Unknown	NA	ENST000002	0,6966	1048		T3	C9502.mFF202103
**TSC2**	c.2566_2571del	p.Leu856_Tyr857del	exon 23/42	chr16	ACCTCT	-	ENSG000001	NA	SNV In_Frame_Del	MODERATE	Unknown	NA	ENST000002	0,6677	1014		T3	C9502.mFF202103
**TRAF7**	c.138A>G	p.Lys46=	exon 3/21	chr16	A	G	ENSG000001	NA	SNV Splice_Region	LOW	Unknown	NA	ENST000003	0,6615	780		T3	C9502.mFF202103
**TP53**	c.673-1G>C	NA	exon NA	chr17	C	G	ENSG000001	CS011061,C	SNV Splice_Site	HIGH	Likely Loss-of-function	Likely Oncogenic	ENST000002	0,8114	1071		T2	C9502.mFF202103
MALT1	c.1174C>T	p.Arg392Cys	exon 10/17	chr18	C	T	ENSG000001	rs757096789,	SNV Missense	MODERATE	Unknown	NA	ENST000003	0,4532	801		T3	C9502.mFF202103
ALK	c.1670G>A	p.Arg557His	exon 9/29	chr2	C	T	ENSG000001	rs200468507	SNV Missense	MODERATE	Unknown	NA	ENST000003	0,1508	1141		T4	C9502.mFF202103
ATRX	c.6874C>T	p.Pro2292Ser	exon 32/35	chrX	G	A	ENSG00000	NA	SNV Missense	MODERATE	Unknown	NA	ENST000003	0,1494	656		T3	C9502.mFF202103
MYCL									CNV							2,355	T5	C8802.mFF202103
FGF10									CNV							2,031	T5	C8802.mFF202103
EGFR									CNV							2,589	T5	C8802.mFF202103
CDK6									CNV							2,051	T5	C8802.mFF202103
MET									CNV							1,729	T5	C8802.mFF202103
BRAF									CNV							1,537	T5	C8802.mFF202103
ATM									CNV							1,653	T5	C8802.mFF202103
CHEK1									CNV							1,62	T5	C8802.mFF202103
ERBB3									CNV							2,006	T5	C8802.mFF202103
FGF14									CNV							1,587	T5	C8802.mFF202103
LAMP1									CNV							1,584	T5	C8802.mFF202103
CCNE1									CNV							1,926	T5	C8802.mFF202103
AKT2									CNV							3,217	T5	C8802.mFF202103
ERCC2									CNV							1,799	T5	C8802.mFF202103
ERCC1									CNV							1,619	T5	C8802.mFF202103
RET	c.1408A>T	p.Thr470Ser	exon 7/20	chr10	A	T	ENSG000001	NA	SNV Missense	MODERATE	Unknown	NA	ENST000003	0,4634	1353		T4	C8802.mFF202103
KDM5A	c.943G>C	p.Asp315His	exon 8/28	chr12	C	G	ENSG00000	NA	SNV Missense	MODERATE	Unknown	NA	ENST000003	0,1852	1026		T3	C8802.mFF202103
CHD4	c.3512C>T	p.Ala1171Val	exon 24/40	chr12	G	A	ENSG000001	rs143588475	SNV Missense	MODERATE	Unknown	NA	ENST000005	0,2008	1265		T3	C8802.mFF202103
POLE	c.1745T>C	p.Leu582Pro	exon 16/49	chr12	A	G	ENSG000001	NA	SNV Missense	MODERATE	Unknown	NA	ENST000003	0,0434	1266		T5	C8802.mFF202103
LATS2	c.3057_3058deli	p.Lys1020Glu	exon 8/8	chr13	TG	CT	ENSG000001	NA	SNV Missense	MODERATE	Unknown	NA	ENST000003	0,3074	1106		T3	C8802.mFF202103
FOXO1	c.123C>A	p.Ser41Arg	exon 1/3	chr13	G	T	ENSG000001	rs144827287	SNV Missense	MODERATE	Unknown	NA	ENST000003	0,9541	109		T3	C8802.mFF202103
**RB1**	c.585G>A	p.Trp195Ter	exon 6/27	chr13	G	A	ENSG000001	COSV572977	SNV Nonsense	HIGH	Likely Loss-of-function	Likely Oncogenic	ENST000002	0,9183	747		T2	C8802.mFF202103
NTRK3	c.125A>C	p.Glu42Ala	exon 2/19	chr15	T	G	ENSG000001	NA	SNV Missense	MODERATE	Unknown	NA	ENST000003	0,0332	814		T5	C8802.mFF202103
SLX4	c.2854_2855deli	p.Ala952Met	exon 12/15	chr16	GC	AT	ENSG000001	rs863224277	SNV Missense	MODERATE	Unknown	NA	ENST000002	0,0406	1132		T5	C8802.mFF202103
CREBBP	c.6796G>A	p.Ala2266Thr	exon 31/31	chr16	C	T	ENSG00000	rs776123525	SNV Missense	MODERATE	Unknown	NA	ENST000002	0,4346	1422		T3	C8802.mFF202103
PALB2	c.284A>C	p.Lys95Thr	exon 4/13	chr16	T	G	ENSG00000	NA	SNV Missense	MODERATE	Unknown	NA	ENST000002	0,0258	893		T5	C8802.mFF202103
ANKRD11	c.2513G>A	p.Arg838Gln	exon 9/13	chr16	C	T	ENSG000001	rs145893630	SNV Missense	MODERATE	Unknown	NA	ENST000003	0,9184	1128		T4	C8802.mFF202103
FANCA	c.2741G>C	p.Arg914Thr	exon 28/43	chr16	C	G	ENSG000001	rs748454613	SNV Missense	MODERATE	Unknown	NA	ENST000003	0,9507	1116		T4	C8802.mFF202103
**MAP2K4**	c.514-2A>T	NA	exon NA	chr17	A	T	ENSG00000	COSV622618	SNV Splice_Site	HIGH	Likely Loss-of-function	Likely Oncogenic	ENST000003	0,794	364		T2	C8802.mFF202103
**RAD51D**	c.43G>T	p.Glu15Ter	exon 1/10	chr17	C	A	ENSG000001	NA	SNV Nonsense	HIGH	Likely Loss-of-function	Likely Oncogenic	ENST000003	0,5565	1689		T2	C8802.mFF202103
STK11	c.986A>C	p.Lys329Thr	exon 8/10	chr19	A	C	ENSG000001	NA	SNV Missense	MODERATE	Unknown	NA	ENST000003	0,01	1397		T5	C8802.mFF202103
ZBTB7A	c.1498A>G	p.Lys500Glu	exon 3/3	chr19	T	C	ENSG000001	NA	SNV Missense	MODERATE	Unknown	NA	ENST000003	0,1698	265		T3	C8802.mFF202103
PTPRS	c.1067A>C	p.Asn356Thr	exon 11/38	chr19	T	G	ENSG000001	rs148759350	SNV Missense	MODERATE	Unknown	NA	ENST000003	0,0435	1611		T5	C8802.mFF202103
AXL	c.711A>C	p.Gln237His	exon 6/20	chr19	A	C	ENSG000001	NA	SNV Missense	MODERATE	Unknown	NA	ENST000003	0,0215	1674		T5	C8802.mFF202103
INPP4A	c.14A>G	p.Glu5Gly	exon 3/26	chr2	A	G	ENSG00000	rs104180883	SNV Missense	MODERATE	Unknown	NA	ENST000000	0,035	1144		T5	C8802.mFF202103
RANBP2	c.4921G>T	p.Ala1641Ser	exon 20/29	chr2	G	T	ENSG000001	NA	SNV Missense	MODERATE	Unknown	NA	ENST000002	0,4141	256		T4	C8802.mFF202103
LRP1B	c.11008G>A	p.Gly3670Arg	exon 71/91	chr2	C	T	ENSG000001	COSV672524	SNV Missense	MODERATE	Unknown	NA	ENST000003	0,2384	730		T3	C8802.mFF202103
BARD1	c.1518_1519inv	p.Val507Met	exon 6/11	chr2	CA	TG	ENSG000001	rs386654966	SNV Missense	MODERATE	Unknown	NA	ENST000002	1	701		T4	C8802.mFF202103
BARD1	c.829G>A	p.Glu277Lys	exon 4/11	chr2	C	T	ENSG000001	rs142604522	SNV Missense	MODERATE	Unknown	NA	ENST000002	0,6271	909		T4	C8802.mFF202103
IRS1	c.1729G>A	p.Val577Met	exon 1/2	chr2	C	T	ENSG000001	rs760281604	SNV Missense	MODERATE	Unknown	NA	ENST000003	0,1296	2439		T3	C8802.mFF202103
SRC	c.739G>C	p.Val247Leu	exon 9/14	chr20	G	C	ENSG000001	COSV624402	SNV Missense	MODERATE	Unknown	NA	ENST000003	0,0976	3013		T3	C8802.mFF202103
GNAS	c.1819T>G	p.Leu607Val	exon 1/13	chr20	T	G	ENSG00000	rs134352626	SNV Missense	MODERATE	Unknown	NA	ENST000003	0,0117	2387		T5	C8802.mFF202103
NF2	c.299T>C	p.Phe100Ser	exon 3/16	chr22	T	C	ENSG000001	NA	SNV Missense	MODERATE	Unknown	NA	ENST000003	0,013	848		T5	C8802.mFF202103
EP300	c.5991A>C	p.Gln1997His	exon 31/31	chr22	A	C	ENSG000001	NA	SNV Missense	MODERATE	Unknown	NA	ENST000002	0,0215	1069		T5	C8802.mFF202103
FANCD2	c.3889-2A>C	NA	exon NA	chr3	A	C	ENSG000001	NA	SNV Splice_Site	HIGH	Likely Loss-of-function	Likely Oncogenic	ENST000002	0,0786	611		T2	C8802.mFF202103
MST1R	c.3197T>C	p.Leu1066Pro	exon 14/20	chr3	A	G	ENSG000001	rs123600284	SNV Missense	MODERATE	Unknown	NA	ENST000002	0,0137	1097		T5	C8802.mFF202103
ATR	c.5690A>G	p.Lys1897Arg	exon 33/47	chr3	T	C	ENSG000001	NA	SNV Missense	MODERATE	Unknown	NA	ENST000003	0,2215	605		T3	C8802.mFF202103
IL7R	c.1184A>C	p.Lys395Thr	exon 8/8	chr5	A	C	ENSG000001	COSV574061	SNV Missense	MODERATE	Unknown	Predicted Oncogenic	ENST000003	0,0073	2054		T5	C8802.mFF202103
IL7R	c.1217T>G	p.Leu406Arg	exon 8/8	chr5	T	G	ENSG000001	COSV574058	SNV Missense	MODERATE	Unknown	NA	ENST000003	0,1557	2029		T4	C8802.mFF202103
NSD1	c.7465A>C	p.Ser2489Arg	exon 23/23	chr5	A	C	ENSG000001	NA	SNV Missense	MODERATE	Unknown	NA	ENST000004	0,0085	1406		T5	C8802.mFF202103
NOTCH4	c.2026T>A	p.Ser676Thr	exon 13/30	chr6	A	T	ENSG000002	NA	SNV Missense	MODERATE	Unknown	NA	ENST000003	0,0961	1363		T4	C8802.mFF202103
EGFR	c.3187G>T	p.Asp1063Tyr	exon 27/28	chr7	G	T	ENSG000001	NA	SNV Missense	MODERATE	Unknown	NA	ENST000002	0,0223	2195		T5	C8802.mFF202103
EGFR	c.3283C>T	p.Gln1095Ter	exon 28/28	chr7	C	T	ENSG000001	NA	SNV Nonsense	HIGH	Unknown	NA	ENST000002	0,0297	1953		T5	C8802.mFF202103
RECQL4	c.1030C>T	p.Arg344Trp	exon 5/22	chr8	G	A	ENSG000001	rs768850000	SNV Missense	MODERATE	Unknown	NA	ENST000004	0,0511	1017		T3	C8802.mFF202103
**ATRX**	c.6849+1G>T	NA	exon NA	chrX	C	A	ENSG00000	COSV648710	SNV Splice_Site	HIGH	Likely Loss-of-function	Likely Oncogenic	ENST000003	0,933	537		T2	C8802.mFF202103
ERRFI1	c.842C>T	p.Pro281Leu	exon 4/4	chr1	G	A	ENSG000001	NA	SNV Missense	MODERATE	Unknown	NA	ENST000003	0,0182	1318		T5	C8101.mFF202103
PIK3CD	c.1406C>T	p.Ala469Val	exon 11/24	chr1	C	T	ENSG000001	NA	SNV Missense	MODERATE	Unknown	NA	ENST000003	0,0191	1048		T5	C8101.mFF202103
MTOR	c.82G>A	p.Gly28Ser	exon 2/58	chr1	C	T	ENSG000001	NA	SNV Missense	MODERATE	Unknown	NA	ENST000003	0,0125	1044		T5	C8101.mFF202103
SPEN	c.700G>A	p.Glu234Lys	exon 3/15	chr1	G	A	ENSG00000	NA	SNV Missense	MODERATE	Unknown	NA	ENST000003	0,012	1505		T5	C8101.mFF202103
SPEN	c.5108C>T	p.Pro1703Leu	exon 11/15	chr1	C	T	ENSG00000	rs750957886	SNV Missense	MODERATE	Unknown	NA	ENST000003	0,0149	1476		T5	C8101.mFF202103
SPEN	c.5959C>T	p.Gln1987Ter	exon 11/15	chr1	C	T	ENSG00000	NA	SNV Nonsense	HIGH	Likely Loss-of-function	Likely Oncogenic	ENST000003	0,0148	1486		T5	C8101.mFF202103
SPEN	c.6683G>A	p.Gly2228Glu	exon 11/15	chr1	G	A	ENSG00000	NA	SNV Missense	MODERATE	Unknown	NA	ENST000003	0,0254	1420		T5	C8101.mFF202103
SPEN	c.7262C>T	p.Thr2421Ile	exon 11/15	chr1	C	T	ENSG00000	rs133376830	SNV Missense	MODERATE	Unknown	NA	ENST000003	0,0118	1359		T5	C8101.mFF202103
SPEN	c.8530G>A	p.Asp2844Asn	exon 11/15	chr1	G	A	ENSG00000	NA	SNV Missense	MODERATE	Unknown	NA	ENST000003	0,0168	1672		T5	C8101.mFF202103
SPEN	c.8723C>T	p.Ser2908Phe	exon 11/15	chr1	C	T	ENSG00000	NA	SNV Missense	MODERATE	Unknown	NA	ENST000003	0,0203	1672		T5	C8101.mFF202103
PAX7	c.482G>A	p.Arg161Lys	exon 4/8	chr1	G	A	ENSG00000	NA	SNV Missense	MODERATE	Unknown	NA	ENST000003	0,0111	1258		T5	C8101.mFF202103
ARID1A	c.2988+1G>A	NA	exon NA	chr1	G	A	ENSG000001	rs886042036,	SNV Splice_Site	HIGH	Likely Loss-of-function	Likely Oncogenic	ENST000003	0,0135	888		T5	C8101.mFF202103

ARID1A	c.5582G>A	p.Ser1861Asn	exon 20/20	chr1	G	A	ENSG000001	NA	SNV Missense	MODERATE	Unknown	NA	ENST000003	0,0172	1687	T5	C8101.mFF202103
CSF3R	c.2396C>T	p.Thr799Ile	exon 17/17	chr1	G	A	ENSG000001	NA	SNV Missense	MODERATE	Unknown	NA	ENST000003	0,0137	1241	T5	C8101.mFF202103
CSF3R	c.1853C>T	p.Thr618Ile	exon 14/17	chr1	G	A	ENSG000001	rs796065343,	SNV Missense	MODERATE	Gain-of-function	Oncogenic	ENST000003	0,017	1120	T5	C8101.mFF202103
CSF3R	c.1068G>A	p.Trp356Ter	exon 9/17	chr1	C	T	ENSG000001	NA	SNV Nonsense	HIGH	Unknown	NA	ENST000003	0,0393	1119	T5	C8101.mFF202103
MUTYH	c.1400C>T	p.Pro467Leu	exon 14/16	chr1	G	A	ENSG000001	rs786202930	SNV Missense	MODERATE	Unknown	NA	ENST000003	0,016	1312	T5	C8101.mFF202103
RAD54L	c.421G>A	p.Val141Ile	exon 7/19	chr1	G	A	ENSG000000	COSV643369	SNV Missense	MODERATE	Unknown	NA	ENST000004	0,0142	1128	T5	C8101.mFF202103
JAK1	c.2296G>A	p.Asp766Asn	exon 17/25	chr1	C	T	ENSG000001	rs124566165	SNV Missense	MODERATE	Unknown	NA	ENST000003	0,0206	1215	T5	C8101.mFF202103
JAK1	c.736G>A	p.Asp246Asn	exon 7/25	chr1	C	T	ENSG000001	COSV610934	SNV Missense	MODERATE	Unknown	NA	ENST000003	0,0158	1268	T5	C8101.mFF202103
JAK1	c.356C>T	p.Thr119Ile	exon 5/25	chr1	G	A	ENSG000001	rs778588460	SNV Missense	MODERATE	Unknown	NA	ENST000003	0,0147	1159	T5	C8101.mFF202103
JAK1	c.206G>A	p.Arg69His	exon 4/25	chr1	C	T	ENSG000001	COSV610870	SNV Missense	MODERATE	Unknown	NA	ENST000003	0,0217	923	T5	C8101.mFF202103
NOTCH2	c.2507C>T	p.Pro836Leu	exon 16/34	chr1	G	A	ENSG000001	COSV566823	SNV Missense	MODERATE	Unknown	NA	ENST000002	0,016	1191	T5	C8101.mFF202103
NTRK1	c.677G>A	p.Gly226Asp	exon 6/17	chr1	G	A	ENSG000001	rs137378150	SNV Missense	MODERATE	Unknown	NA	ENST000005	0,0189	1375	T5	C8101.mFF202103
SPTA1	c.5008G>A	p.Ala1670Thr	exon 36/52	chr1	C	T	ENSG000001	NA	SNV Missense	MODERATE	Unknown	NA	ENST000003	0,0122	1311	T5	C8101.mFF202103
SPTA1	c.4879G>A	p.Glu1627Lys	exon 35/52	chr1	C	T	ENSG000001	rs748582690,	SNV Missense	MODERATE	Unknown	NA	ENST000003	0,0128	939	T5	C8101.mFF202103
SPTA1	c.3640G>A	p.Asp1214Asn	exon 26/52	chr1	C	T	ENSG000001	COSV637624	SNV Missense	MODERATE	Unknown	NA	ENST000003	0,0156	1411	T5	C8101.mFF202103
SDHC	c.179+1G>A	NA	exon NA	chr1	G	A	ENSG000001	NA	SNV Splice_Site	HIGH	Likely Loss-of-function	Likely Oncogenic	ENST000003	0,0216	789	T5	C8101.mFF202103
ABL2	c.3034G>A	p.Gly1012Arg	exon 12/12	chr1	C	T	ENSG000001	NA	SNV Missense	MODERATE	Unknown	NA	ENST000005	0,0133	1584	T5	C8101.mFF202103
ABL2	c.1572G>A	p.Trp524Ter	exon 10/12	chr1	C	T	ENSG000001	NA	SNV Nonsense	HIGH	Unknown	NA	ENST000005	0,0116	1036	T5	C8101.mFF202103
PIK3C2B	c.3973G>A	p.Gly1325Ser	exon 28/34	chr1	C	T	ENSG000001	NA	SNV Missense	MODERATE	Unknown	NA	ENST000003	0,0199	1358	T5	C8101.mFF202103
PIK3C2B	c.2437G>A	p.Glu813Lys	exon 16/34	chr1	C	T	ENSG000001	rs140652808	SNV Missense	MODERATE	Unknown	NA	ENST000003	0,0124	1369	T5	C8101.mFF202103
PIK3C2B	c.1990G>A	p.Glu664Lys	exon 13/34	chr1	C	T	ENSG000001	NA	SNV Missense	MODERATE	Unknown	NA	ENST000003	0,0151	1191	T5	C8101.mFF202103
PIK3C2B	c.361G>A	p.Gly121Ser	exon 3/34	chr1	C	T	ENSG000001	NA	SNV Missense	MODERATE	Unknown	NA	ENST000003	0,0172	1683	T5	C8101.mFF202103
MDM4	c.596C>T	p.Pro199Leu	exon 8/11	chr1	C	T	ENSG000001	COSV657950	SNV Missense	MODERATE	Unknown	NA	ENST000003	0,0219	1003	T5	C8101.mFF202103
IKBKE	c.862G>A	p.Glu288Lys	exon 9/22	chr1	G	A	ENSG000001	NA	SNV Missense	MODERATE	Unknown	NA	ENST000003	0,0116	1382	T5	C8101.mFF202103
IKBKE	c.1139C>T	p.Thr380Ile	exon 10/22	chr1	C	T	ENSG000001	NA	SNV Missense	MODERATE	Unknown	NA	ENST000003	0,0343	1253	T5	C8101.mFF202103
IKBKE	c.1348C>T	p.Leu450Phe	exon 13/22	chr1	C	T	ENSG000001	rs133630965	SNV Missense	MODERATE	Unknown	NA	ENST000003	0,0141	1066	T5	C8101.mFF202103
IKBKE	c.1589G>A	p.Ser530Asn	exon 15/22	chr1	G	A	ENSG000001	NA	SNV Missense	MODERATE	Unknown	NA	ENST000003	0,0147	1225	T5	C8101.mFF202103
IL10	c.337G>A	p.Gly113Arg	exon 3/5	chr1	C	T	ENSG000001	rs155860281	SNV Missense	MODERATE	Unknown	NA	ENST000004	0,016	1250	T5	C8101.mFF202103
PARP1	c.1423G>A	p.Ala475Thr	exon 10/23	chr1	C	T	ENSG000001	COSV646892	SNV Missense	MODERATE	Unknown	NA	ENST000003	0,0167	1500	T5	C8101.mFF202103
PARP1	c.1369G>A	p.Asp457Asn	exon 10/23	chr1	C	T	ENSG000001	NA	SNV Missense	MODERATE	Unknown	NA	ENST000003	0,0185	1517	T5	C8101.mFF202103
FH	c.1481C>T	p.Ala494Val	exon 10/10	chr1	G	A	ENSG000000	rs752369363	SNV Missense	MODERATE	Unknown	NA	ENST000003	0,0206	1070	T5	C8101.mFF202103
AKT3	c.823G>A	p.Glu275Lys	exon 9/13	chr1	C	T	ENSG000001	NA	SNV Missense	MODERATE	Unknown	NA	ENST000002	0,0158	761	T5	C8101.mFF202103
AKT3	c.68G>A	p.Arg23Lys	exon 2/13	chr1	C	T	ENSG000001	NA	SNV Missense	MODERATE	Unknown	NA	ENST000002	0,0163	799	T5	C8101.mFF202103
AKT3	c.3G>A	p.Met1?	exon 1/13	chr1	C	T	ENSG000001	NA	SNV Translation_Sta	HIGH	Unknown	NA	ENST000002	0,0215	884	T5	C8101.mFF202103
KIF5B	c.1870G>A	p.Glu624Lys	exon 16/26	chr10	C	T	ENSG000001	rs143519342	SNV Missense	MODERATE	Unknown	NA	ENST000003	0,0191	784	T5	C8101.mFF202103
ARID5B	c.142C>T	p.Pro48Ser	exon 2/10	chr10	C	T	ENSG000001	NA	SNV Missense	MODERATE	Unknown	NA	ENST000002	0,0163	1231	T5	C8101.mFF202103
ARID5B	c.2855C>T	p.Pro952Leu	exon 10/10	chr10	C	T	ENSG000001	NA	SNV Missense	MODERATE	Unknown	NA	ENST000002	0,016	1563	T5	C8101.mFF202103
TET1	c.374C>T	p.Ser125Phe	exon 2/12	chr10	C	T	ENSG000001	rs866518744	SNV Missense	MODERATE	Unknown	NA	ENST000003	0,0198	1311	T5	C8101.mFF202103
TET1	c.1051G>A	p.Ala351Thr	exon 2/12	chr10	G	A	ENSG000001	COSV653886	SNV Missense	MODERATE	Unknown	NA	ENST000003	0,0331	1391	T5	C8101.mFF202103
TET1	c.5128C>T	p.Leu1710Phe	exon 11/12	chr10	C	T	ENSG000001	rs147777668	SNV Missense	MODERATE	Unknown	NA	ENST000003	0,0446	1166	T5	C8101.mFF202103
FGF8	c.724G>A	p.Glu242Lys	exon 6/6	chr10	C	T	ENSG000001	rs137456496	SNV Missense	MODERATE	Unknown	NA	ENST000003	0,0209	1003	T5	C8101.mFF202103
FGF8	c.175C>T	p.Pro59Ser	exon 4/6	chr10	G	A	ENSG000001	NA	SNV Missense	MODERATE	Unknown	NA	ENST000003	0,0161	1369	T5	C8101.mFF202103
SUFU	c.299G>A	p.Gly100Asp	exon 2/12	chr10	G	A	ENSG000001	NA	SNV Missense	MODERATE	Unknown	NA	ENST000003	0,0218	1100	T5	C8101.mFF202103
SMC3	c.1255G>A	p.Asp419Asn	exon 13/29	chr10	G	A	ENSG000001	NA	SNV Missense	MODERATE	Unknown	NA	ENST000003	0,0229	829	T5	C8101.mFF202103
TCF7L2	c.1150C>T	p.Leu384Phe	exon 10/14	chr10	C	T	ENSG000001	COSV533460	SNV Missense	MODERATE	Unknown	NA	ENST000005	0,0121	907	T5	C8101.mFF202103
FGFR2	c.416G>A	p.Gly139Asp	exon 4/18	chr10	C	T	ENSG000000	rs132470324	SNV Missense	MODERATE	Unknown	NA	ENST000003	0,0236	1061	T5	C8101.mFF202103
HRAS	c.436G>A	p.Ala146Thr	exon 4/5	chr11	C	T	ENSG000001	rs104894231,	SNV Missense	MODERATE	Gain-of-function	Oncogenic	ENST000004	0,0148	1013	T5	C8101.mFF202103
MYOD1	c.14C>T	p.Ser5Leu	exon 1/3	chr11	C	T	ENSG000001	NA	SNV Missense	MODERATE	Unknown	NA	ENST000002	0,014	1001	T5	C8101.mFF202103
WT1	c.64C>T	p.Pro22Ser	exon 1/10	chr11	G	A	ENSG000001	NA	SNV Missense	MODERATE	Unknown	NA	ENST000003	0,0214	513	T5	C8101.mFF202103
RPS6KA4	c.581C>T	p.Thr194Ile	exon 6/17	chr11	C	T	ENSG000001	NA	SNV Missense	MODERATE	Unknown	NA	ENST000003	0,0109	1376	T5	C8101.mFF202103
RPS6KA4	c.1033C>T	p.Pro345Ser	exon 9/17	chr11	C	T	ENSG000001	NA	SNV Missense	MODERATE	Unknown	NA	ENST000003	0,0125	1043	T5	C8101.mFF202103
MEN1	c.973G>A	p.Ala325Thr	exon 7/10	chr11	C	T	ENSG000001	CM031240	SNV Missense	MODERATE	Unknown	NA	ENST000003	0,0184	1470	T5	C8101.mFF202103
AP001888.1	c.50C>T	p.Ala17Val	exon 1/1	chr11	G	A	ENSG000002	NA	SNV Missense	MODERATE	Unknown	NA	ENST000006	0,0199	1404	T5	C8101.mFF202103
EED	c.689G>A	p.Gly230Glu	exon 7/13	chr11	G	A	ENSG000000	COSV545580	SNV Missense	MODERATE	Unknown	NA	ENST000003	0,0126	1108	T5	C8101.mFF202103
EED	c.1055C>T	p.Ala352Val	exon 11/13	chr11	C	T	ENSG000000	NA	SNV Missense	MODERATE	Unknown	NA	ENST000003	0,011	998	T5	C8101.mFF202103
ATM	c.413G>A	p.Gly138Glu	exon 5/63	chr11	G	A	ENSG000001	NA	SNV Missense	MODERATE	Unknown	NA	ENST000002	0,0184	761	T5	C8101.mFF202103
ATM	c.2383C>T	p.Pro795Ser	exon 16/63	chr11	C	T	ENSG000001	rs106050165	SNV Missense	MODERATE	Unknown	NA	ENST000002	0,0144	763	T5	C8101.mFF202103
ATM	c.3952G>A	p.Val1318Ile	exon 26/63	chr11	G	A	ENSG000001	NA	SNV Missense	MODERATE	Unknown	NA	ENST000002	0,0137	1096	T5	C8101.mFF202103
ATM	c.4144C>T	p.Pro1382Ser	exon 28/63	chr11	C	T	ENSG000001	rs55859590	SNV Missense	MODERATE	Unknown	NA	ENST000002	0,0154	777	T5	C8101.mFF202103
ATM	c.5453G>A	p.Gly1818Asp	exon 36/63	chr11	G	A	ENSG000001	NA	SNV Missense	MODERATE	Unknown	NA	ENST000002	0,0191	944	T5	C8101.mFF202103
ATM	c.6910G>A	p.Glu2304Lys	exon 47/63	chr11	G	A	ENSG000001	CX068644,C(	SNV Missense	MODERATE	Unknown	NA	ENST000002	0,0163	1103	T5	C8101.mFF202103
SDHD	c.238C>T	p.Leu80Phe	exon 3/4	chr11	C	T	ENSG000002	NA	SNV Missense	MODERATE	Unknown	NA	ENST000003	0,0164	1101	T5	C8101.mFF202103
KMT2A	c.307C>T	p.Pro103Ser	exon 1/36	chr11	C	T	ENSG000001	NA	SNV Missense	MODERATE	Unknown	NA	ENST000005	0,0205	487	T5	C8101.mFF202103
KMT2A	c.1135G>A	p.Ala379Thr	exon 3/36	chr11	G	A	ENSG000001	NA	SNV Missense	MODERATE	Unknown	NA	ENST000005	0,0154	1300	T5	C8101.mFF202103
KMT2A	c.1175C>T	p.Ala392Val	exon 3/36	chr11	C	T	ENSG000001	NA	SNV Missense	MODERATE	Unknown	NA	ENST000005	0,0133	1433	T5	C8101.mFF202103
KMT2A	c.2371G>A	p.Ala791Thr	exon 3/36	chr11	G	A	ENSG000001	COSV632899	SNV Missense	MODERATE	Unknown	NA	ENST000005	0,0137	1464	T5	C8101.mFF202103
KMT2A	c.2422G>A	p.Glu808Lys	exon 3/36	chr11	G	A	ENSG000001	NA	SNV Missense	MODERATE	Unknown	NA	ENST000005	0,0143	1402	T5	C8101.mFF202103
KMT2A	c.4219G>A	p.Glu1407Lys	exon 10/36	chr11	G	A	ENSG000001	COSV632852	SNV Missense	MODERATE	Unknown	NA	ENST000005	0,0279	896	T5	C8101.mFF202103
KMT2A	c.5236G>A	p.Gly1746Arg	exon 17/36	chr11	G	A	ENSG000001	NA	SNV Missense	MODERATE	Unknown	NA	ENST000005	0,0138	1089	T5	C8101.mFF202103
KMT2A	c.7528G>A	p.Gly2510Arg	exon 27/36	chr11	G	A	ENSG000001	rs130550737	SNV Missense	MODERATE	Unknown	NA	ENST000005	0,0197	1371	T5	C8101.mFF202103
KMT2A	c.8281C>T	p.Pro2761Ser	exon 27/36	chr11	C	T	ENSG000001	rs782608868	SNV Missense	MODERATE	Unknown	NA	ENST000005	0,0181	1435	T5	C8101.mFF202103
CBL	c.1721C>T	p.Ser574Phe	exon 11/16	chr11	C	T	ENSG000001	NA	SNV Missense	MODERATE	Unknown	NA	ENST000002	0,0161	1557	T5	C8101.mFF202103
ETS1	c.926C>T	p.Thr309Ile	exon 8/10	chr11	G	A	ENSG000001	NA	SNV Missense	MODERATE	Unknown	NA	ENST000003	0,015	1399	T5	C8101.mFF202103
FLI1	c.1235C>T	p.Ser412Phe	exon 9/9	chr11	C	T	ENSG000001	NA	SNV Missense	MODERATE	Unknown	NA	ENST000005	0,0138	1742	T5	C8101.mFF202103
KDM5A	c.2002C>T	p.Leu668Phe	exon 15/28	chr12	G	A	ENSG000000	COSV669924	SNV Missense	MODERATE	Unknown	NA	ENST000003	0,0217	1062	T5	C8101.mFF202103
KDM5A	c.577G>A	p.Glu193Lys	exon 5/28	chr12	C	T	ENSG000000	COSV669923	SNV Missense	MODERATE	Unknown	NA	ENST000003	0,0193	986	T5	C8101.mFF202103
CCND2	c.850G>A	p.Val284Met	exon 5/5	chr12	G	A	ENSG000001	rs746964971,	SNV Missense	MODERATE	Unknown	NA	ENST000002	0,0152	1249	T5	C8101.mFF202103
FGF23	c.77C>T	p.Pro26Leu	exon 1/3	chr12	G	A	ENSG000001	rs118002520	SNV Missense	MODERATE	Unknown	NA	ENST000002	0,0384	1147	T5	C8101.mFF202103
CHD4	c.2173G>A	p.Glu725Lys	exon 15/40	chr12	C	T	ENSG000001	NA	SNV Missense	MODERATE	Unknown	NA	ENST000005	0,0123	1463	T5	C8101.mFF202103
ETV6	c.785G>A	p.Ser262Asn	exon 5/8	chr12	G	A	ENSG000001	NA	SNV Missense	MODERATE	Unknown	NA	ENST000003	0,0161	1859	T5	C8101.mFF202103
KRAS	c.38G>A	p.Gly13Asp	exon 2/6	chr12	C	T	ENSG000001	rs112445441,	SNV Missense	MODERATE	Gain-of-function	Oncogenic	ENST000002	0,0214	842	T5	C8101.mFF202103

KRAS	c.35G>A	p.Gly12Asp	exon 2/6	chr12	C	T	ENSG000001	rs121913529,	SNV Missense	MODERATE	Gain-of-function	Oncogenic	ENST000002	0,0141	849	T5	C8101.mFF202103
ARID2	c.4552C>T	p.Pro1518Ser	exon 15/21	chr12	C	T	ENSG000001	COSV576177	SNV Missense	MODERATE	Unknown	NA	ENST000003	0,0217	1477	T5	C8101.mFF202103
SMARCD1	c.307C>T	p.Pro103Ser	exon 2/13	chr12	C	T	ENSG000000	NA	SNV Missense	MODERATE	Unknown	NA	ENST000003	0,0129	1320	T5	C8101.mFF202103
ACVR1B	c.1364C>T	p.Ala455Val	exon 8/10	chr12	C	T	ENSG000001	NA	SNV Missense	MODERATE	Unknown	NA	ENST000005	0,0177	1300	T5	C8101.mFF202103
GLI1	c.2119C>T	p.Pro707Ser	exon 12/12	chr12	C	T	ENSG000001	NA	SNV Missense	MODERATE	Unknown	NA	ENST000002	0,0171	1757	T5	C8101.mFF202103
GLI1	c.2521C>T	p.Pro841Ser	exon 12/12	chr12	C	T	ENSG000001	rs1398037874	SNV Missense	MODERATE	Unknown	NA	ENST000002	0,0131	1224	T5	C8101.mFF202103
TSPAN31	c.95C>T	p.Ala32Val	exon 2/6	chr12	C	T	ENSG000001	rs746291400	SNV Missense	MODERATE	Unknown	NA	ENST000002	0,0145	1378	T5	C8101.mFF202103
MDM2	c.14G>A	p.Arg5Lys	exon 1/11	chr12	G	A	ENSG000001	rs1231258383	SNV Missense	MODERATE	Unknown	NA	ENST000004	0,014	1003	T5	C8101.mFF202103
MDM2	c.685G>A	p.Asp229Asn	exon 9/11	chr12	G	A	ENSG000001	NA	SNV Missense	MODERATE	Unknown	NA	ENST000004	0,043	744	T5	C8101.mFF202103
FRS2	c.1366C>T	p.Pro456Ser	exon 7/7	chr12	C	T	ENSG000001	rs772087017	SNV Missense	MODERATE	Unknown	NA	ENST000005	0,014	1430	T5	C8101.mFF202103
PTPN11	c.232G>A	p.Val78Ile	exon 3/16	chr12	G	A	ENSG000001	COSV610087	SNV Missense	MODERATE	Unknown	NA	ENST000003	0,0187	1232	T5	C8101.mFF202103
PTPN11	c.890C>T	p.Pro297Leu	exon 8/16	chr12	C	T	ENSG000001	NA	SNV Missense	MODERATE	Unknown	NA	ENST000003	0,0173	984	T5	C8101.mFF202103
PTPN11	c.1676C>T	p.Pro559Leu	exon 14/16	chr12	C	T	ENSG000001	NA	SNV Missense	MODERATE	Unknown	NA	ENST000003	0,0131	1147	T5	C8101.mFF202103
TBX3	c.1298C>T	p.Pro433Leu	exon 7/8	chr12	G	A	ENSG000001	COSV574708	SNV Missense	MODERATE	Unknown	NA	ENST000002	0,0138	1013	T5	C8101.mFF202103
TBX3	c.46G>A	p.Ala16Thr	exon 1/8	chr12	C	T	ENSG000001	NA	SNV Missense	MODERATE	Unknown	NA	ENST000002	0,0106	1043	T5	C8101.mFF202103
HNF1A	c.1555C>T	p.Pro519Ser	exon 8/10	chr12	C	T	ENSG000001	rs200639058	SNV Missense	MODERATE	Unknown	NA	ENST000002	0,0162	1424	T5	C8101.mFF202103
POLE	c.6466G>A	p.Val2156Ile	exon 46/49	chr12	C	T	ENSG000001	rs542978638	SNV Missense	MODERATE	Unknown	NA	ENST000003	0,0115	1647	T5	C8101.mFF202103
POLE	c.6071C>T	p.Pro2024Leu	exon 44/49	chr12	G	A	ENSG000001	COSV576846	SNV Missense	MODERATE	Unknown	NA	ENST000003	0,0302	1127	T5	C8101.mFF202103
POLE	c.278G>A	p.Arg93Lys	exon 3/49	chr12	C	T	ENSG000001	COSV576781	SNV Missense	MODERATE	Unknown	NA	ENST000003	0,0137	1022	T5	C8101.mFF202103
LATS2	c.3010C>T	p.Pro1004Ser	exon 8/8	chr13	G	A	ENSG000001	rs369166799	SNV Missense	MODERATE	Unknown	NA	ENST000003	0,0142	1477	T5	C8101.mFF202103
LATS2	c.883G>A	p.Gly295Arg	exon 4/8	chr13	C	T	ENSG000001	NA	SNV Missense	MODERATE	Unknown	NA	ENST000003	0,0303	627	T5	C8101.mFF202103
LATS2	c.766C>T	p.Pro256Ser	exon 4/8	chr13	G	A	ENSG000001	COSV668807	SNV Missense	MODERATE	Unknown	NA	ENST000003	0,0198	859	T5	C8101.mFF202103
CDK8	c.266C>T	p.Ala89Val	exon 3/13	chr13	C	T	ENSG000001	NA	SNV Missense	MODERATE	Unknown	NA	ENST000003	0,022	862	T5	C8101.mFF202103
CDK8	c.515-1G>A	NA	exon NA	chr13	G	A	ENSG000001	NA	SNV Splice_Site	HIGH	Unknown	NA	ENST000003	0,0207	531	T5	C8101.mFF202103
CDK8	c.1222C>T	p.Pro408Ser	exon 12/13	chr13	C	T	ENSG000001	COSV674229	SNV Missense	MODERATE	Unknown	NA	ENST000003	0,012	1163	T5	C8101.mFF202103
FLT1	c.2719G>A	p.Val907Met	exon 20/30	chr13	C	T	ENSG000001	NA	SNV Missense	MODERATE	Unknown	NA	ENST000002	0,0126	1113	T5	C8101.mFF202103
BRCA2	c.590C>T	p.Ser197Phe	exon 7/28	chr13	C	T	ENSG000001	rs876659940,	SNV Missense	MODERATE	Unknown	Unknown	ENST000005	0,0138	869	T5	C8101.mFF202103
ERCC5	c.275G>A	p.Arg92Lys	exon 3/15	chr13	G	A	ENSG000001	NA	SNV Missense	MODERATE	Unknown	NA	ENST000003	0,0367	709	T5	C8101.mFF202103
ERCC5	c.973C>T	p.Pro325Ser	exon 8/15	chr13	C	T	ENSG000001	rs1406728466	SNV Missense	MODERATE	Unknown	NA	ENST000003	0,0278	1189	T5	C8101.mFF202103
ERCC5	c.1366G>A	p.Glu456Lys	exon 8/15	chr13	G	A	ENSG000001	NA	SNV Missense	MODERATE	Unknown	NA	ENST000003	0,0193	1454	T5	C8101.mFF202103
ERCC5	c.2852G>A	p.Gly951Glu	exon 13/15	chr13	G	A	ENSG000001	NA	SNV Missense	MODERATE	Unknown	NA	ENST000003	0,0133	1050	T5	C8101.mFF202103
NKX2-1	c.673G>A	p.Ala225Thr	exon 3/3	chr14	C	T	ENSG000001	COSV613888	SNV Missense	MODERATE	Unknown	NA	ENST000003	0,0111	994	T5	C8101.mFF202103
DICER1	c.5527G>A	p.Glu1843Lys	exon 25/27	chr14	C	T	ENSG000001	COSV586299	SNV Missense	MODERATE	Unknown	NA	ENST000003	0,0236	764	T5	C8101.mFF202103
DICER1	c.4207-1G>A	NA	exon NA	chr14	C	T	ENSG000001	NA	SNV Splice_Site	HIGH	Likely Loss-of-function	Likely Oncogenic	ENST000003	0,0215	836	T5	C8101.mFF202103
DICER1	c.2449C>T	p.Pro817Ser	exon 16/27	chr14	G	A	ENSG000001	NA	SNV Missense	MODERATE	Unknown	NA	ENST000003	0,0353	906	T5	C8101.mFF202103
DICER1	c.2275G>A	p.Asp759Asn	exon 15/27	chr14	C	T	ENSG000001	NA	SNV Missense	MODERATE	Unknown	NA	ENST000003	0,0115	954	T5	C8101.mFF202103
DICER1	c.10C>T	p.Pro4Ser	exon 2/27	chr14	G	A	ENSG000001	rs772433602	SNV Missense	MODERATE	Unknown	NA	ENST000003	0,0157	830	T5	C8101.mFF202103
AKT1	c.1029G>A	p.Met343Ile	exon 11/14	chr14	C	T	ENSG000001	NA	SNV Missense	MODERATE	Unknown	NA	ENST000004	0,0187	1175	T5	C8101.mFF202103
AKT1	c.200G>A	p.Arg67Gln	exon 4/14	chr14	C	T	ENSG000001	rs1396308032	SNV Missense	MODERATE	Unknown	NA	ENST000004	0,016	939	T5	C8101.mFF202103
NUTM1	c.623C>T	p.Pro208Leu	exon 3/8	chr15	C	T	ENSG000001	NA	SNV Missense	MODERATE	Unknown	NA	ENST000005	0,0214	1166	T5	C8101.mFF202103
NUTM1	c.1462G>A	p.Gly488Arg	exon 7/8	chr15	G	A	ENSG000001	NA	SNV Missense	MODERATE	Unknown	NA	ENST000005	0,0119	1095	T5	C8101.mFF202103
NUTM1	c.2127G>A	p.Trp709Ter	exon 8/8	chr15	G	A	ENSG000001	NA	SNV Nonsense	HIGH	Unknown	NA	ENST000005	0,0126	1275	T5	C8101.mFF202103
NUTM1	c.2935G>A	p.Glu979Lys	exon 8/8	chr15	G	A	ENSG000001	COSV592169	SNV Missense	MODERATE	Unknown	NA	ENST000005	0,0121	1327	T5	C8101.mFF202103
MGA	c.302G>A	p.Gly101Glu	exon 2/24	chr15	G	A	ENSG000001	NA	SNV Missense	MODERATE	Unknown	NA	ENST000002	0,0135	1411	T5	C8101.mFF202103
MGA	c.1544C>T	p.Ser515Phe	exon 3/24	chr15	C	T	ENSG000001	COSV549578	SNV Missense	MODERATE	Unknown	NA	ENST000002	0,0105	1143	T5	C8101.mFF202103
MGA	c.4406C>T	p.Pro1469Leu	exon 13/24	chr15	C	T	ENSG000001	COSV549487	SNV Missense	MODERATE	Unknown	NA	ENST000002	0,0186	1022	T5	C8101.mFF202103
MGA	c.5992G>A	p.Gly1998Arg	exon 17/24	chr15	G	A	ENSG000001	NA	SNV Missense	MODERATE	Unknown	NA	ENST000002	0,0187	1069	T5	C8101.mFF202103
MGA	c.6502G>A	p.Glu2168Lys	exon 17/24	chr15	G	A	ENSG000001	NA	SNV Missense	MODERATE	Unknown	NA	ENST000002	0,0133	1127	T5	C8101.mFF202103
MGA	c.8471C>T	p.Thr2824Ile	exon 24/24	chr15	C	T	ENSG000001	COSV549630	SNV Missense	MODERATE	Unknown	NA	ENST000002	0,0137	1391	T5	C8101.mFF202103
NTRK3	c.1765G>A	p.Ala589Thr	exon 15/19	chr15	C	T	ENSG000001	rs1195860832	SNV Missense	MODERATE	Unknown	NA	ENST000003	0,0154	1036	T5	C8101.mFF202103
FANCI	c.1060G>A	p.Val354Ile	exon 12/38	chr15	G	A	ENSG000001	NA	SNV Missense	MODERATE	Unknown	NA	ENST000003	0,0164	733	T5	C8101.mFF202103
BLM	c.256G>A	p.Val86Ile	exon 3/22	chr15	G	A	ENSG000001	NA	SNV Missense	MODERATE	Unknown	NA	ENST000003	0,0171	1169	T5	C8101.mFF202103
BLM	c.4136C>T	p.Ser1379Phe	exon 22/22	chr15	C	T	ENSG000001	NA	SNV Missense	MODERATE	Unknown	NA	ENST000003	0,0114	1055	T5	C8101.mFF202103
CHD2	c.232G>A	p.Val78Ile	exon 3/39	chr15	G	A	ENSG000001	NA	SNV Missense	MODERATE	Unknown	NA	ENST000003	0,0175	1314	T5	C8101.mFF202103
IGF1R	c.61G>A	p.Ala21Thr	exon 1/21	chr15	G	A	ENSG000001	NA	SNV Missense	MODERATE	Unknown	NA	ENST000002	0,0152	659	T5	C8101.mFF202103
AXIN1	c.386C>T	p.Pro129Leu	exon 2/11	chr16	G	A	ENSG000001	NA	SNV Missense	MODERATE	Unknown	NA	ENST000002	0,0102	1370	T5	C8101.mFF202103
TSC2	c.3736G>A	p.Asp1246Asn	exon 31/42	chr16	G	A	ENSG000001	NA	SNV Missense	MODERATE	Unknown	NA	ENST000002	0,0165	1333	T5	C8101.mFF202103
SLX4	c.5357G>A	p.Arg1786Lys	exon 15/15	chr16	C	T	ENSG000001	NA	SNV Missense	MODERATE	Unknown	NA	ENST000002	0,0135	1630	T5	C8101.mFF202103
SLX4	c.5011C>T	p.His1671Tyr	exon 14/15	chr16	G	A	ENSG000001	NA	SNV Missense	MODERATE	Unknown	NA	ENST000002	0,014	1288	T5	C8101.mFF202103
CREBBP	c.6707G>A	p.Gly2236Glu	exon 31/31	chr16	C	T	ENSG000000	rs774272248,	SNV Missense	MODERATE	Unknown	NA	ENST000002	0,0232	1295	T5	C8101.mFF202103
CREBBP	c.2209G>A	p.Ala737Thr	exon 12/31	chr16	C	T	ENSG000000	rs1436634025	SNV Missense	MODERATE	Unknown	NA	ENST000002	0,0116	1119	T5	C8101.mFF202103
CREBBP	c.458C>T	p.Pro153Leu	exon 2/31	chr16	G	A	ENSG000000	rs146538907	SNV Missense	MODERATE	Unknown	NA	ENST000002	0,4381	1349	T4	C8101.mFF202103
CREBBP	c.236G>A	p.Gly79Asp	exon 2/31	chr16	C	T	ENSG000000	rs1200676852	SNV Missense	MODERATE	Unknown	NA	ENST000002	0,0204	1179	T5	C8101.mFF202103
ERCC4	c.1997C>T	p.Ser666Phe	exon 10/11	chr16	C	T	ENSG000001	NA	SNV Missense	MODERATE	Unknown	NA	ENST000003	0,0163	979	T5	C8101.mFF202103
ERCC4	c.2177G>A	p.Arg726His	exon 11/11	chr16	G	A	ENSG000001	rs368096448	SNV Missense	MODERATE	Unknown	NA	ENST000003	0,0205	1513	T5	C8101.mFF202103
CYLD	c.1642G>A	p.Val548Ile	exon 9/18	chr16	G	A	ENSG000000	rs1279948701	SNV Missense	MODERATE	Unknown	NA	ENST000005	0,0149	870	T5	C8101.mFF202103
NUP93	c.476C>T	p.Thr159Ile	exon 5/22	chr16	C	T	ENSG000001	rs369482440	SNV Missense	MODERATE	Unknown	NA	ENST000003	0,0228	788	T5	C8101.mFF202103
NUP93	c.1234G>A	p.Asp412Asn	exon 11/22	chr16	G	A	ENSG000001	NA	SNV Missense	MODERATE	Unknown	NA	ENST000003	0,014	1216	T5	C8101.mFF202103
NUP93	c.1553G>A	p.Gly518Asp	exon 14/22	chr16	G	A	ENSG000001	NA	SNV Missense	MODERATE	Unknown	NA	ENST000003	0,0179	1173	T5	C8101.mFF202103
CBFB	c.176C>T	p.Ala59Val	exon 3/6	chr16	C	T	ENSG000000	NA	SNV Missense	MODERATE	Unknown	NA	ENST000002	0,0196	818	T5	C8101.mFF202103
CTCF	c.1876G>A	p.Glu626Lys	exon 11/12	chr16	G	A	ENSG000001	NA	SNV Missense	MODERATE	Unknown	NA	ENST000002	0,0214	1030	T5	C8101.mFF202103
ZFHX3	c.9830C>T	p.Pro3277Leu	exon 10/10	chr16	G	A	ENSG000001	rs990041797	SNV Missense	MODERATE	Unknown	NA	ENST000002	0,0124	1610	T5	C8101.mFF202103
ZFHX3	c.9260C>T	p.Ala3087Val	exon 9/10	chr16	G	A	ENSG000001	NA	SNV Missense	MODERATE	Unknown	NA	ENST000002	0,0136	1618	T5	C8101.mFF202103
ZFHX3	c.6446G>A	p.Arg2149His	exon 9/10	chr16	C	T	ENSG000001	COSV517177	SNV Missense	MODERATE	Unknown	NA	ENST000002	0,0122	1481	T5	C8101.mFF202103
PLCG2	c.1405G>A	p.Glu469Lys	exon 15/33	chr16	G	A	ENSG000001	COSV638776	SNV Missense	MODERATE	Unknown	NA	ENST000003	0,0133	1277	T5	C8101.mFF202103
ANKRD11	c.4835G>A	p.Arg1612Lys	exon 9/13	chr16	C	T	ENSG000001	rs1431469157	SNV Missense	MODERATE	Unknown	NA	ENST000003	0,0134	1194	T5	C8101.mFF202103
ANKRD11	c.3322G>A	p.Gly1108Ser	exon 9/13	chr16	C	T	ENSG000001	NA	SNV Missense	MODERATE	Unknown	NA	ENST000003	0,0176	1081	T5	C8101.mFF202103
ANKRD11	c.3275C>T	p.Pro1092Leu	exon 9/13	chr16	G	A	ENSG000001	rs769125026	SNV Missense	MODERATE	Unknown	NA	ENST000003	0,0116	1034	T5	C8101.mFF202103
ANKRD11	c.1748G>A	p.Gly583Asp	exon 9/13	chr16	C	T	ENSG000001	NA	SNV Missense	MODERATE	Unknown	NA	ENST000003	0,0233	1415	T5	C8101.mFF202103
FANCA	c.3824C>T	p.Ser1275Leu	exon 38/43	chr16	G	A	ENSG000001	NA	SNV Missense	MODERATE	Unknown	NA	ENST000003	0,0221	904	T5	C8101.mFF202103
TP53	c.731G>A	p.Gly244Asp	exon 7/11	chr17	C	T	ENSG000001	rs985033810,	SNV Missense	MODERATE	Likely Loss-of-function	Likely Oncogenic	ENST000002	0,0124	1294	T5	C8101.mFF202103

TP53	c.580C>T	p.Leu194Phe	exon 6/11	chr17	G	A	ENSG000001	rs587780071,	SNV	Missense	MODERATE	Likely Loss-of-function	Likely Oncogenic	ENST000002	0,0145	1519	T5	C8101.mFF202103
TP53	c.293C>T	p.Pro98Leu	exon 4/11	chr17	G	A	ENSG000001	rs124572311!	SNV	Missense	MODERATE	Unknown	NA	ENST000002	0,0115	1300	T5	C8101.mFF202103
NCOR1	c.5044C>T	p.Pro1682Ser	exon 34/46	chr17	G	A	ENSG000001	NA	SNV	Missense	MODERATE	Unknown	NA	ENST000002	0,0158	1016	T5	C8101.mFF202103
NCOR1	c.4634G>A	p.Ser1545Asn	exon 32/46	chr17	C	T	ENSG000001	NA	SNV	Missense	MODERATE	Unknown	NA	ENST000002	0,0148	1349	T5	C8101.mFF202103
NF1	c.1392+1G>A	NA	exon NA	chr17	G	A	ENSG000001	rs267604791,	SNV	Splice_Site	HIGH	Likely Loss-of-function	Likely Oncogenic	ENST000003	0,0188	906	T5	C8101.mFF202103
NF1	c.4783C>T	p.Gln1595Ter	exon 36/58	chr17	C	T	ENSG000001	COSV622112	SNV	Nonsense	HIGH	Likely Loss-of-function	Likely Oncogenic	ENST000003	0,016	686	T5	C8101.mFF202103
CDK12	c.742C>T	p.Leu248Phe	exon 1/14	chr17	C	T	ENSG000001	NA	SNV	Missense	MODERATE	Unknown	NA	ENST000004	0,0168	1430	T5	C8101.mFF202103
ERBB2	c.1388G>A	p.Ser463Asn	exon 12/27	chr17	G	A	ENSG000001	NA	SNV	Missense	MODERATE	Unknown	NA	ENST000002	0,0099	1513	T5	C8101.mFF202103
ERBB2	c.1801C>T	p.Pro601Ser	exon 15/27	chr17	C	T	ENSG000001	COSV540696	SNV	Missense	MODERATE	Unknown	NA	ENST000002	0,0169	1067	T5	C8101.mFF202103
ERBB2	c.1949C>T	p.Pro650Leu	exon 17/27	chr17	C	T	ENSG000001	rs778798172	SNV	Missense	MODERATE	Likely Gain-of-function	Likely Oncogenic	ENST000002	0,015	934	T5	C8101.mFF202103
RARA	c.925G>A	p.Val309Ile	exon 7/9	chr17	G	A	ENSG000001	NA	SNV	Missense	MODERATE	Unknown	NA	ENST000002	0,0131	1223	T5	C8101.mFF202103
STAT5B	c.2186C>T	p.Ala729Val	exon 18/19	chr17	G	A	ENSG000001	NA	SNV	Missense	MODERATE	Unknown	NA	ENST000002	0,0143	910	T5	C8101.mFF202103
STAT5B	c.2053C>T	p.Pro685Ser	exon 16/19	chr17	G	A	ENSG000001	NA	SNV	Missense	MODERATE	Unknown	NA	ENST000002	0,0146	890	T5	C8101.mFF202103
BRCA1	c.2813C>T	p.Pro938Leu	exon 10/23	chr17	G	A	ENSG00000(	rs106479399!	SNV	Missense	MODERATE	Unknown	Unknown	ENST000003	0,0148	1288	T5	C8101.mFF202103
HOXB13	c.362C>T	p.Pro121Leu	exon 1/2	chr17	G	A	ENSG000001	NA	SNV	Missense	MODERATE	Unknown	NA	ENST000002	0,0167	1557	T5	C8101.mFF202103
SPOP	c.481G>A	p.Val161Met	exon 6/10	chr17	C	T	ENSG000001	COSV616562	SNV	Missense	MODERATE	Unknown	NA	ENST000005	0,0139	721	T5	C8101.mFF202103
RNF43	c.1003C>T	p.Pro335Ser	exon 9/10	chr17	G	A	ENSG000001	NA	SNV	Missense	MODERATE	Unknown	NA	ENST000005	0,0444	1260	T5	C8101.mFF202103
RPS6KB1	c.141+1G>A	NA	exon NA	chr17	G	A	ENSG000001	COSV566749	SNV	Splice_Site	HIGH	Unknown	NA	ENST000002	0,0223	940	T5	C8101.mFF202103
SRSF2	c.605C>T	p.Ser202Phe	exon 2/2	chr17	G	A	ENSG000001	NA	SNV	Missense	MODERATE	Unknown	NA	ENST000003	0,0101	1284	T5	C8101.mFF202103
RPTOR	c.466C>T	p.Pro156Ser	exon 4/34	chr17	C	T	ENSG000001	rs132158887!	SNV	Missense	MODERATE	Unknown	NA	ENST000003	0,0143	908	T5	C8101.mFF202103
PIK3C3	c.196G>A	p.Glu66Lys	exon 2/25	chr18	G	A	ENSG00000(	NA	SNV	Missense	MODERATE	Unknown	NA	ENST000002	0,0148	1149	T5	C8101.mFF202103
PIK3C3	c.1526G>A	p.Arg509Lys	exon 14/25	chr18	G	A	ENSG00000(	COSV562792	SNV	Missense	MODERATE	Unknown	NA	ENST000002	0,0136	1027	T5	C8101.mFF202103
PIK3C3	c.1913G>A	p.Gly638Glu	exon 17/25	chr18	G	A	ENSG00000(	NA	SNV	Missense	MODERATE	Unknown	NA	ENST000002	0,0132	984	T5	C8101.mFF202103
SETBP1	c.241G>A	p.Val81Met	exon 2/6	chr18	G	A	ENSG000001	NA	SNV	Missense	MODERATE	Unknown	NA	ENST000002	0,011	1730	T5	C8101.mFF202103
SETBP1	c.812G>A	p.Gly271Glu	exon 4/6	chr18	G	A	ENSG000001	COSV563128	SNV	Missense	MODERATE	Unknown	NA	ENST000002	0,0157	1910	T5	C8101.mFF202103
SETBP1	c.1063G>A	p.Val355Ile	exon 4/6	chr18	G	A	ENSG000001	rs752918225,	SNV	Missense	MODERATE	Unknown	NA	ENST000002	0,0117	1706	T5	C8101.mFF202103
SETBP1	c.1112G>A	p.Gly371Asp	exon 4/6	chr18	G	A	ENSG000001	COSV563136	SNV	Missense	MODERATE	Unknown	NA	ENST000002	0,0117	1802	T5	C8101.mFF202103
SETBP1	c.1166C>T	p.Ser389Phe	exon 4/6	chr18	C	T	ENSG000001	NA	SNV	Missense	MODERATE	Unknown	NA	ENST000002	0,0178	1686	T5	C8101.mFF202103
SETBP1	c.3418G>A	p.Ala1140Thr	exon 4/6	chr18	G	A	ENSG000001	NA	SNV	Missense	MODERATE	Unknown	NA	ENST000002	0,012	2009	T5	C8101.mFF202103
SETBP1	c.3936G>C	p.Lys1312Asn	exon 4/6	chr18	G	C	ENSG000001	NA	SNV	Missense	MODERATE	Unknown	NA	ENST000002	0,1118	1744	T3	C8101.mFF202103
SETBP1	c.4373G>A	p.Arg1458Lys	exon 6/6	chr18	G	A	ENSG000001	COSV563143	SNV	Missense	MODERATE	Unknown	NA	ENST000002	0,0196	1586	T5	C8101.mFF202103
SMAD4	c.299G>A	p.Arg100Lys	exon 3/12	chr18	G	A	ENSG000001	COSV616891	SNV	Missense	MODERATE	Likely Loss-of-function	Likely Oncogenic	ENST000003	0,0129	1088	T5	C8101.mFF202103
SMAD4	c.454G>A	p.Ala152Thr	exon 4/12	chr18	G	A	ENSG000001	NA	SNV	Missense	MODERATE	Unknown	NA	ENST000003	0,0189	687	T5	C8101.mFF202103
SMAD4	c.1522G>A	p.Gly508Ser	exon 12/12	chr18	G	A	ENSG000001	CM056701,C(	SNV	Missense	MODERATE	Likely Loss-of-function	Likely Oncogenic	ENST000003	0,0204	1371	T5	C8101.mFF202103
BCL2	c.585G>A	p.Trp195Ter	exon 1/2	chr18	C	T	ENSG000001	NA	SNV	Nonsense	HIGH	Unknown	NA	ENST000003	0,0161	1742	T5	C8101.mFF202103
STK11	c.808G>A	p.Gly270Arg	exon 6/10	chr19	G	A	ENSG000001	NA	SNV	Missense	MODERATE	Unknown	NA	ENST000003	0,0186	1289	T5	C8101.mFF202103
TCF3	c.499+1G>A	NA	exon NA	chr19	C	T	ENSG00000(	NA	SNV	Splice_Site	HIGH	Likely Loss-of-function	Likely Oncogenic	ENST000002	0,0146	754	T5	C8101.mFF202103
MAP2K2	c.294G>A	p.Met98Ile	exon 2/11	chr19	C	T	ENSG000001	NA	SNV	Missense	MODERATE	Unknown	NA	ENST000002	0,0478	899	T5	C8101.mFF202103
PTPRS	c.1288C>T	p.Pro430Ser	exon 11/38	chr19	G	A	ENSG000001	NA	SNV	Missense	MODERATE	Unknown	NA	ENST000003	0,0414	1087	T5	C8101.mFF202103
INSR	c.3890G>A	p.Ser1297Asn	exon 22/22	chr19	C	T	ENSG000001	NA	SNV	Missense	MODERATE	Unknown	NA	ENST000003	0,0207	1495	T5	C8101.mFF202103
INSR	c.574G>A	p.Gly192Ser	exon 2/22	chr19	C	T	ENSG000001	NA	SNV	Missense	MODERATE	Unknown	NA	ENST000003	0,0234	1284	T5	C8101.mFF202103
DNMT1	c.3592C>T	p.Pro1198Ser	exon 33/41	chr19	G	A	ENSG000001	NA	SNV	Missense	MODERATE	Unknown	NA	ENST000003	0,0139	1435	T5	C8101.mFF202103
DNMT1	c.3275C>T	p.Ser1092Phe	exon 30/41	chr19	G	A	ENSG000001	NA	SNV	Missense	MODERATE	Unknown	NA	ENST000003	0,0212	1274	T5	C8101.mFF202103
DNMT1	c.2746G>A	p.Ala916Thr	exon 28/41	chr19	C	T	ENSG000001	NA	SNV	Missense	MODERATE	Unknown	NA	ENST000003	0,0186	1024	T5	C8101.mFF202103
SMARCA4	c.248G>A	p.Gly83Asp	exon 3/35	chr19	G	A	ENSG000001	COSV608104	SNV	Missense	MODERATE	Unknown	NA	ENST000003	0,0255	1217	T5	C8101.mFF202103
SMARCA4	c.1241G>A	p.Arg414Lys	exon 7/35	chr19	G	A	ENSG000001	NA	SNV	Missense	MODERATE	Unknown	NA	ENST000003	0,0142	1338	T5	C8101.mFF202103
SMARCA4	c.1761G>A	p.Lys587=	exon 10/35	chr19	G	A	ENSG000001	NA	SNV	Splice_Region	LOW	Likely Loss-of-function	Likely Oncogenic	ENST000003	0,0123	896	T5	C8101.mFF202103
SMARCA4	c.2641G>A	p.Asp881Asn	exon 19/35	chr19	G	A	ENSG000001	COSV607897	SNV	Missense	MODERATE	Unknown	NA	ENST000003	0,0151	1659	T5	C8101.mFF202103
CALR	c.1054-1G>A	NA	exon NA	chr19	G	A	ENSG000001	NA	SNV	Splice_Site	HIGH	Likely Gain-of-function	Likely Oncogenic	ENST000003	0,017	1236	T5	C8101.mFF202103
NOTCH3	c.6658G>A	p.Glu2220Lys	exon 33/33	chr19	C	T	ENSG00000(	NA	SNV	Missense	MODERATE	Unknown	NA	ENST000002	0,021	1237	T5	C8101.mFF202103
NOTCH3	c.2742_2744deli	p.Gly915Asp	exon 17/33	chr19	CCT	TCC	ENSG00000(	NA	SNV	Missense	MODERATE	Unknown	NA	ENST000002	0,0208	1152	T5	C8101.mFF202103
NOTCH3	c.2144G>A	p.Gly715Glu	exon 13/33	chr19	C	T	ENSG00000(	NA	SNV	Missense	MODERATE	Unknown	NA	ENST000002	0,0167	1439	T5	C8101.mFF202103
BRD4	c.1147G>A	p.Glu383Lys	exon 6/20	chr19	C	T	ENSG000001	NA	SNV	Missense	MODERATE	Unknown	NA	ENST000002	0,0131	1531	T5	C8101.mFF202103
JAK3	c.2929G>A	p.Asp977Asn	exon 21/24	chr19	C	T	ENSG000001	NA	SNV	Missense	MODERATE	Unknown	NA	ENST000004	0,0211	1138	T5	C8101.mFF202103
AXL	c.433C>T	p.Pro145Ser	exon 4/20	chr19	C	T	ENSG000001	NA	SNV	Missense	MODERATE	Unknown	NA	ENST000003	0,0207	821	T5	C8101.mFF202103
AXL	c.1186G>A	p.Asp396Asn	exon 9/20	chr19	G	A	ENSG000001	rs117792610!	SNV	Missense	MODERATE	Unknown	NA	ENST000003	0,0158	1200	T5	C8101.mFF202103
CD79A	c.28G>A	p.Ala10Thr	exon 1/5	chr19	G	A	ENSG000001	rs371184689,	SNV	Missense	MODERATE	Unknown	NA	ENST000002	0,0155	1422	T5	C8101.mFF202103
CIC	c.983C>T	p.Thr328Ile	exon 7/20	chr19	C	T	ENSG00000(	rs134210730:	SNV	Missense	MODERATE	Unknown	NA	ENST000005	0,0178	958	T5	C8101.mFF202103
ERCC2	c.1973G>A	p.Arg658His	exon 21/23	chr19	C	T	ENSG000001	rs762141272,	SNV	Missense	MODERATE	Unknown	NA	ENST000003	0,0249	1484	T5	C8101.mFF202103
ERCC2	c.951G>A	p.Glu317=	exon 11/23	chr19	C	T	ENSG000001	NA	SNV	Splice_Region	LOW	Likely Loss-of-function	Likely Oncogenic	ENST000003	0,0139	793	T5	C8101.mFF202103
CD3EAP	c.278C>T	p.Thr93Ile	exon 3/3	chr19	C	T	ENSG000001	NA	SNV	Missense	MODERATE	Unknown	NA	ENST000003	0,0233	1158	T5	C8101.mFF202103
POLD1	c.3304C>T	p.Pro1102Ser	exon 27/27	chr19	C	T	ENSG00000(	rs757442072,	SNV	Missense	MODERATE	Unknown	NA	ENST000004	0,0167	899	T5	C8101.mFF202103
PPP2R1A	c.1659C>T	p.Asn553=	exon 13/15	chr19	C	T	ENSG000001	NA	SNV	Splice_Region	LOW	Likely Loss-of-function	Likely Oncogenic	ENST000003	0,0207	1110	T5	C8101.mFF202103
DNMT3A	c.338G>A	p.Gly113Glu	exon 4/23	chr2	C	T	ENSG000001	NA	SNV	Missense	MODERATE	Unknown	NA	ENST000003	0,0173	1215	T5	C8101.mFF202103
ASXL2	c.1982C>T	p.Thr661Ile	exon 12/12	chr2	G	A	ENSG000001	NA	SNV	Missense	MODERATE	Unknown	NA	ENST000003	0,0314	700	T5	C8101.mFF202103
ALK	c.1853G>A	p.Gly618Glu	exon 10/29	chr2	C	T	ENSG000001	rs766821017,	SNV	Missense	MODERATE	Unknown	NA	ENST000003	0,0184	1466	T5	C8101.mFF202103
ALK	c.1718C>T	p.Thr573Ile	exon 9/29	chr2	G	A	ENSG000001	NA	SNV	Missense	MODERATE	Unknown	NA	ENST000003	0,0193	1400	T5	C8101.mFF202103
MSH6	c.994G>A	p.Glu332Lys	exon 4/10	chr2	G	A	ENSG000001	COSV522878	SNV	Missense	MODERATE	Unknown	NA	ENST000002	0,012	1337	T5	C8101.mFF202103
MSH6	c.2902G>A	p.Val968Ile	exon 4/10	chr2	G	A	ENSG000001	NA	SNV	Missense	MODERATE	Unknown	NA	ENST000002	0,019	1422	T5	C8101.mFF202103
XPO1	c.2926G>A	p.Glu976Lys	exon 23/25	chr2	C	T	ENSG00000(	COSV689475	SNV	Missense	MODERATE	Unknown	NA	ENST000004	0,0223	987	T5	C8101.mFF202103
RANBP2	c.2102C>T	p.Pro701Leu	exon 15/29	chr2	C	T	ENSG000001	COSV517074	SNV	Missense	MODERATE	Unknown	NA	ENST000002	0,014	715	T5	C8101.mFF202103
RANBP2	c.2554C>T	p.Pro852Ser	exon 18/29	chr2	C	T	ENSG000001	NA	SNV	Missense	MODERATE	Unknown	NA	ENST000002	0,02	801	T5	C8101.mFF202103
PAX8	c.1147C>G	p.Pro383Ala	exon 10/10	chr2	G	C	ENSG000001	rs369313070,	SNV	Missense	MODERATE	Unknown	NA	ENST000002	0,4911	1071	T3	C8101.mFF202103
LRP1B	c.7967C>T	p.Thr2656Ile	exon 49/91	chr2	G	A	ENSG000001	rs201766309	SNV	Missense	MODERATE	Unknown	NA	ENST000003	0,0216	972	T5	C8101.mFF202103
LRP1B	c.6824C>T	p.Ala2275Val	exon 42/91	chr2	G	A	ENSG000001	NA	SNV	Missense	MODERATE	Unknown	NA	ENST000003	0,0164	978	T5	C8101.mFF202103
LRP1B	c.5599C>T	p.Leu1867Phe	exon 34/91	chr2	G	A	ENSG000001	NA	SNV	Missense	MODERATE	Unknown	NA	ENST000003	0,0188	743	T5	C8101.mFF202103
LRP1B	c.2489G>A	p.Gly830Glu	exon 15/91	chr2	C	T	ENSG000001	rs866996598,	SNV	Missense	MODERATE	Unknown	NA	ENST000003	0,0174	922	T5	C8101.mFF202103
PMS1	c.1354G>A	p.Glu452Lys	exon 9/13	chr2	G	A	ENSG00000(	COSV597183	SNV	Missense	MODERATE	Unknown	NA	ENST000004	0,017	941	T5	C8101.mFF202103
SF3B1	c.686C>T	p.Ser229Phe	exon 7/25	chr2	G	A	ENSG000001	COSV592119	SNV	Missense	MODERATE	Unknown	NA	ENST000003	0,0206	730	T5	C8101.mFF202103
CTLA4	c.260C>T	p.Ala87Val	exon 2/4	chr2	C	T	ENSG000001	NA	SNV	Missense	MODERATE	Unknown	NA	ENST000003	0,012	1586	T5	C8101.mFF202103
ERBB4	c.3458C>T	p.Pro1153Leu	exon 27/28	chr2	G	A	ENSG000001	rs905120734,	SNV	Missense	MODERATE	Unknown	NA	ENST000003	0,0178	900	T5	C8101.mFF202103

ERBB4	c.46G>A	p.Ala16Thr	exon 1/28	chr2	C	T	ENSG000001	COSV535785	SNV Missense	MODERATE	Unknown	NA	ENST000003	0,0231	1167	T5	C8101.mFF202103
BARD1	c.1961C>T	p.Pro654Leu	exon 10/11	chr2	G	A	ENSG000001	rs587780025	SNV Missense	MODERATE	Unknown	NA	ENST000002	0,0186	916	T5	C8101.mFF202103
PAX3	c.994C>T	p.Gln332Ter	exon 7/10	chr2	G	A	ENSG000001	NA	SNV Nonsense	HIGH	Unknown	NA	ENST000003	0,0154	1554	T5	C8101.mFF202103
PAX3	c.925G>T	p.Glu309Ter	exon 6/10	chr2	C	A	ENSG000001	NA	SNV Nonsense	HIGH	Unknown	NA	ENST000003	0,0154	1560	T5	C8101.mFF202103
PAX3	c.535G>A	p.Glu179Lys	exon 4/10	chr2	C	T	ENSG000001	NA	SNV Missense	MODERATE	Unknown	NA	ENST000003	0,0145	1515	T5	C8101.mFF202103
PAK5	c.1607C>T	p.Thr536Ile	exon 6/10	chr20	G	A	ENSG000001	rs760772824,	SNV Missense	MODERATE	Unknown	NA	ENST000003	0,0182	1155	T5	C8101.mFF202103
PAK5	c.325G>A	p.Asp109Asn	exon 4/10	chr20	C	T	ENSG000001	COSV620263	SNV Missense	MODERATE	Unknown	NA	ENST000003	0,0103	1359	T5	C8101.mFF202103
PAK5	c.117G>A	p.Trp39Ter	exon 3/10	chr20	C	T	ENSG000001	COSV620245	SNV Nonsense	HIGH	Unknown	NA	ENST000003	0,0141	1210	T5	C8101.mFF202103
ASXL1	c.1126G>A	p.Gly376Ser	exon 12/13	chr20	G	A	ENSG000001	COSV601237	SNV Missense	MODERATE	Unknown	NA	ENST000003	0,0165	1331	T5	C8101.mFF202103
ASXL1	c.1838C>T	p.Thr613Ile	exon 13/13	chr20	C	T	ENSG000001	rs128775333	SNV Missense	MODERATE	Unknown	NA	ENST000003	0,0142	1484	T5	C8101.mFF202103
ASXL1	c.2813C>T	p.Pro938Leu	exon 13/13	chr20	C	T	ENSG000001	COSV601104	SNV Missense	MODERATE	Unknown	NA	ENST000003	0,0152	1517	T5	C8101.mFF202103
ASXL1	c.3104C>T	p.Pro1035Leu	exon 13/13	chr20	C	T	ENSG000001	NA	SNV Missense	MODERATE	Unknown	NA	ENST000003	0,0146	1712	T5	C8101.mFF202103
DNMT3B	c.872C>T	p.Thr291Ile	exon 8/23	chr20	C	T	ENSG00000C	NA	SNV Missense	MODERATE	Unknown	NA	ENST000003	0,0205	1123	T5	C8101.mFF202103
PTPRT	c.3497G>A	p.Gly1166Asp	exon 26/32	chr20	C	T	ENSG000001	rs759776463,	SNV Missense	MODERATE	Unknown	NA	ENST000003	0,012	1080	T5	C8101.mFF202103
PTPRT	c.152G>A	p.Gly51Glu	exon 2/32	chr20	C	T	ENSG000001	NA	SNV Missense	MODERATE	Unknown	NA	ENST000003	0,022	1044	T5	C8101.mFF202103
NCOA3	c.115G>A	p.Glu39Lys	exon 4/23	chr20	G	A	ENSG000001	NA	SNV Missense	MODERATE	Unknown	NA	ENST000003	0,0306	851	T5	C8101.mFF202103
NCOA3	c.1738G>A	p.Val580Met	exon 12/23	chr20	G	A	ENSG000001	NA	SNV Missense	MODERATE	Unknown	NA	ENST000003	0,012	1335	T5	C8101.mFF202103
NCOA3	c.3322G>A	p.Ala1108Thr	exon 18/23	chr20	G	A	ENSG000001	NA	SNV Missense	MODERATE	Unknown	NA	ENST000003	0,013	1309	T5	C8101.mFF202103
ZNF217	c.2408C>T	p.Pro803Leu	exon 4/6	chr20	G	A	ENSG000001	rs563497204	SNV Missense	MODERATE	Unknown	NA	ENST000003	0,016	1442	T5	C8101.mFF202103
ZNF217	c.913G>A	p.Gly305Arg	exon 2/6	chr20	C	T	ENSG000001	NA	SNV Missense	MODERATE	Unknown	NA	ENST000003	0,0176	1478	T5	C8101.mFF202103
GNAS	c.86C>T	p.Pro29Leu	exon 1/13	chr20	C	T	ENSG00000C	NA	SNV Missense	MODERATE	Unknown	NA	ENST000003	0,0228	1184	T5	C8101.mFF202103
GNAS	c.1012A>G	p.Lys338Glu	exon 1/13	chr20	A	G	ENSG00000C	NA	SNV Missense	MODERATE	Unknown	NA	ENST000003	0,0135	1558	T5	C8101.mFF202103
GNAS	c.1148C>T	p.Ala383Val	exon 1/13	chr20	C	T	ENSG00000C	NA	SNV Missense	MODERATE	Unknown	NA	ENST000003	0,0179	1283	T5	C8101.mFF202103
GNAS	c.1307C>A	p.Ala436Asp	exon 1/13	chr20	C	A	ENSG00000C	rs61749698	SNV Missense	MODERATE	Unknown	NA	ENST000003	0,5063	877	T4	C8101.mFF202103
GNAS	c.1465C>T	p.Pro489Ser	exon 1/13	chr20	C	T	ENSG00000C	NA	SNV Missense	MODERATE	Unknown	NA	ENST000003	0,0242	372	T5	C8101.mFF202103
ARFRP1	c.320G>A	p.Arg107Lys	exon 4/7	chr20	C	T	ENSG000001	NA	SNV Missense	MODERATE	Unknown	NA	ENST000003	0,0138	1305	T5	C8101.mFF202103
RUNX1	c.527C>T	p.Thr176Ile	exon 6/9	chr21	G	A	ENSG000001	NA	SNV Missense	MODERATE	Unknown	NA	ENST000004	0,0182	1430	T5	C8101.mFF202103
ERG	c.541C>T	p.Pro181Ser	exon 4/10	chr21	G	A	ENSG000001	COSV557269	SNV Missense	MODERATE	Unknown	NA	ENST000002	0,0163	1652	T5	C8101.mFF202103
ERG	c.436G>A	p.Ala146Thr	exon 4/10	chr21	C	T	ENSG000001	rs143233807	SNV Missense	MODERATE	Unknown	NA	ENST000002	0,0127	1575	T5	C8101.mFF202103
ERG	c.65C>T	p.Ala22Val	exon 2/10	chr21	G	A	ENSG000001	rs143730253	SNV Missense	MODERATE	Unknown	NA	ENST000002	0,0142	1334	T5	C8101.mFF202103
U2AF1	c.281G>A	p.Gly94Glu	exon 5/8	chr21	C	T	ENSG000001	NA	SNV Missense	MODERATE	Unknown	NA	ENST000002	0,0118	1187	T5	C8101.mFF202103
ICOSLG	c.1125G>A	p.Trp375Ter	exon 6/6	chr21	C	T	ENSG000001	NA	SNV Nonsense	HIGH	Unknown	NA	ENST000004	0,0131	1143	T5	C8101.mFF202103
CRKL	c.277C>G	p.Leu93Val	exon 1/3	chr22	C	G	ENSG00000C	NA	SNV Missense	MODERATE	Unknown	NA	ENST000003	0,0507	1361	T3	C8101.mFF202103
CRKL	c.331G>A	p.Gly111Arg	exon 2/3	chr22	G	A	ENSG00000C	NA	SNV Missense	MODERATE	Unknown	NA	ENST000003	0,0176	1022	T5	C8101.mFF202103
LZTR1	c.201-1G>A	NA	exon NA	chr22	G	A	ENSG00000C	NA	SNV Splice_Site	HIGH	Likely Loss-of-function	Likely Oncogenic	ENST000002	0,0174	1325	T5	C8101.mFF202103
LZTR1	c.1241G>A	p.Gly414Glu	exon 11/21	chr22	G	A	ENSG00000C	NA	SNV Missense	MODERATE	Unknown	NA	ENST000002	0,0157	1337	T5	C8101.mFF202103
LZTR1	c.1478C>T	p.Pro493Leu	exon 14/21	chr22	C	T	ENSG00000C	NA	SNV Missense	MODERATE	Unknown	NA	ENST000002	0,0204	441	T5	C8101.mFF202103
LZTR1	c.1909C>T	p.Pro637Ser	exon 16/21	chr22	C	T	ENSG00000C	NA	SNV Missense	MODERATE	Unknown	NA	ENST000002	0,017	1060	T5	C8101.mFF202103
MAPK1	c.203C>T	p.Thr68Ile	exon 2/9	chr22	G	A	ENSG000001	NA	SNV Missense	MODERATE	Inconclusive	Inconclusive	ENST000002	0,0198	1159	T5	C8101.mFF202103
BCR	c.46C>T	p.Pro16Ser	exon 1/23	chr22	C	T	ENSG000001	NA	SNV Missense	MODERATE	Unknown	NA	ENST000003	0,0182	826	T5	C8101.mFF202103
BCR	c.109C>T	p.Arg37Cys	exon 1/23	chr22	C	T	ENSG000001	rs774029303	SNV Missense	MODERATE	Unknown	NA	ENST000003	0,0115	1046	T5	C8101.mFF202103
BCR	c.427G>A	p.Asp143Asn	exon 1/23	chr22	G	A	ENSG000001	NA	SNV Missense	MODERATE	Unknown	NA	ENST000003	0,0274	365	T5	C8101.mFF202103
CHEK2	c.573G>A	p.Arg191=	exon 4/16	chr22	C	T	ENSG000001	rs129810994	SNV Splice_Region	LOW	Likely Loss-of-function	Likely Oncogenic	ENST000003	0,0493	892	T5	C8101.mFF202103
CHEK2	c.196G>A	p.Val66Met	exon 2/16	chr22	C	T	ENSG000001	rs730881696	SNV Missense	MODERATE	Unknown	NA	ENST000003	0,017	1356	T5	C8101.mFF202103
EWSR1	c.299G>A	p.Gly100Asp	exon 6/18	chr22	G	A	ENSG000001	NA	SNV Missense	MODERATE	Unknown	NA	ENST000004	0,0154	1232	T5	C8101.mFF202103
EWSR1	c.1753G>A	p.Asp585Asn	exon 17/18	chr22	G	A	ENSG000001	NA	SNV Missense	MODERATE	Unknown	NA	ENST000004	0,0118	1607	T5	C8101.mFF202103
EWSR1	c.1853G>A	p.Gly618Glu	exon 17/18	chr22	G	A	ENSG000001	NA	SNV Missense	MODERATE	Unknown	NA	ENST000004	0,0152	1509	T5	C8101.mFF202103
SOX10	c.505C>T	p.Pro169Ser	exon 3/4	chr22	G	A	ENSG000001	NA	SNV Missense	MODERATE	Unknown	NA	ENST000003	0,0306	1375	T5	C8101.mFF202103
SOX10	c.265C>T	p.Pro89Ser	exon 2/4	chr22	G	A	ENSG000001	COSV610055	SNV Missense	MODERATE	Unknown	NA	ENST000003	0,0162	1419	T5	C8101.mFF202103
EP300	c.941G>A	p.Gly314Asp	exon 4/31	chr22	G	A	ENSG000001	rs102224355	SNV Missense	MODERATE	Unknown	NA	ENST000002	0,0119	1346	T5	C8101.mFF202103
EP300	c.3853G>A	p.Glu1285Lys	exon 23/31	chr22	G	A	ENSG000001	COSV543284	SNV Missense	MODERATE	Unknown	NA	ENST000002	0,0156	707	T5	C8101.mFF202103
FANCD2	c.4123C>T	p.Leu1375Phe	exon 42/43	chr3	C	T	ENSG000001	NA	SNV Missense	MODERATE	Unknown	NA	ENST000002	0,0197	1064	T5	C8101.mFF202103
VHL	c.213del	p.Ser72ProfsTer87	exon 1/3	chr3	C	-	ENSG000001	COSV565467	SNV Frame_Shift_D	HIGH	Likely Loss-of-function	Likely Oncogenic	ENST000002	0,1304	851	T2	C8101.mFF202103
MLH1	c.545+1G>A	NA	exon NA	chr3	G	A	ENSG00000C	rs267607765,	SNV Splice_Site	HIGH	Likely Loss-of-function	Likely Oncogenic	ENST000002	0,0435	827	T5	C8101.mFF202103
MLH1	c.1731+1G>A	NA	exon NA	chr3	G	A	ENSG00000C	rs267607853,	SNV Splice_Site	HIGH	Likely Loss-of-function	Likely Oncogenic	ENST000002	0,0231	779	T5	C8101.mFF202103
CTNNB1	c.157G>A	p.Glu53Lys	exon 3/15	chr3	G	A	ENSG000001	COSV626945	SNV Missense	MODERATE	Unknown	NA	ENST000003	0,0111	1078	T5	C8101.mFF202103
CTNNB1	c.1441G>A	p.Ala481Thr	exon 9/15	chr3	G	A	ENSG000001	NA	SNV Missense	MODERATE	Unknown	NA	ENST000003	0,0224	1386	T5	C8101.mFF202103
MST1	c.494G>A	p.Gly165Asp	exon 5/18	chr3	C	T	ENSG000001	NA	SNV Missense	MODERATE	Unknown	NA	ENST000004	0,0137	1312	T5	C8101.mFF202103
MST1	c.106C>T	p.Pro36Ser	exon 2/18	chr3	G	A	ENSG000001	NA	SNV Missense	MODERATE	Unknown	NA	ENST000004	0,0131	1218	T5	C8101.mFF202103
BAP1	c.1891G>A	p.Glu631Lys	exon 15/17	chr3	C	T	ENSG000001	COSV562353	SNV Missense	MODERATE	Unknown	NA	ENST000004	0,0146	1300	T5	C8101.mFF202103
BAP1	c.1451C>T	p.Pro484Leu	exon 13/17	chr3	G	A	ENSG000001	NA	SNV Missense	MODERATE	Unknown	NA	ENST000004	0,01	1397	T5	C8101.mFF202103
PBRM1	c.953G>A	p.Gly318Asp	exon 10/30	chr3	C	T	ENSG000001	rs959810153	SNV Missense	MODERATE	Unknown	NA	ENST000003	0,0185	1080	T5	C8101.mFF202103
MITF	c.167G>A	p.Arg56His	exon 2/10	chr3	G	A	ENSG000001	COSV588864	SNV Missense	MODERATE	Unknown	NA	ENST000003	0,0163	1226	T5	C8101.mFF202103
RYBP	c.702G>A	p.Arg234=	exon 4/4	chr3	C	T	ENSG000001	NA	SNV Splice_Region	LOW	Likely Loss-of-function	Likely Oncogenic	ENST000004	0,0156	1029	T5	C8101.mFF202103
SHQ1	c.1532G>A	p.Arg511Lys	exon 11/11	chr3	C	T	ENSG000001	NA	SNV Missense	MODERATE	Unknown	NA	ENST000003	0,0172	1334	T5	C8101.mFF202103
EPHA3	c.2610G>C	p.Lys870Asn	exon 15/17	chr3	G	C	ENSG00000C	NA	SNV Missense	MODERATE	Unknown	NA	ENST000003	0,0175	1261	T5	C8101.mFF202103
EPHB1	c.88G>A	p.Ala30Thr	exon 2/16	chr3	G	A	ENSG000001	rs746365113,	SNV Missense	MODERATE	Unknown	NA	ENST000003	0,0331	1118	T5	C8101.mFF202103
EPHB1	c.943C>T	p.Pro315Ser	exon 4/16	chr3	C	T	ENSG000001	NA	SNV Missense	MODERATE	Unknown	NA	ENST000003	0,0142	1125	T5	C8101.mFF202103
EPHB1	c.1735C>T	p.Leu579Phe	exon 9/16	chr3	C	T	ENSG000001	NA	SNV Missense	MODERATE	Unknown	NA	ENST000003	0,0115	1303	T5	C8101.mFF202103
EPHB1	c.1990G>A	p.Asp664Asn	exon 11/16	chr3	G	A	ENSG000001	NA	SNV Missense	MODERATE	Unknown	NA	ENST000003	0,0118	1615	T5	C8101.mFF202103
STAG1	c.2222C>T	p.Ser741Phe	exon 22/34	chr3	G	A	ENSG000001	NA	SNV Missense	MODERATE	Unknown	NA	ENST000003	0,0188	798	T5	C8101.mFF202103
ATR	c.6870G>A	p.Trp2290Ter	exon 40/47	chr3	C	T	ENSG000001	NA	SNV Nonsense	HIGH	Likely Loss-of-function	Likely Oncogenic	ENST000003	0,0132	909	T5	C8101.mFF202103
ATR	c.3197G>A	p.Ser1066Asn	exon 16/47	chr3	C	T	ENSG000001	NA	SNV Missense	MODERATE	Unknown	NA	ENST000003	0,0178	844	T5	C8101.mFF202103
ATR	c.413G>A	p.Ser138Asn	exon 4/47	chr3	C	T	ENSG000001	NA	SNV Missense	MODERATE	Unknown	NA	ENST000003	0,0331	938	T5	C8101.mFF202103
PIK3CA	c.2240C>T	p.Ala747Val	exon 15/21	chr3	C	T	ENSG000001	COSV559362	SNV Missense	MODERATE	Unknown	NA	ENST000002	0,0145	899	T5	C8101.mFF202103
MAP3K13	c.2488C>T	p.Pro830Ser	exon 12/14	chr3	C	T	ENSG00000C	NA	SNV Missense	MODERATE	Unknown	NA	ENST000002	0,023	1044	T5	C8101.mFF202103
ETV5	c.136C>T	p.Leu46=	exon 4/13	chr3	G	A	ENSG000002	rs136795048	SNV Splice_Region	LOW	Unknown	NA	ENST000003	0,0166	662	T5	C8101.mFF202103
TFRC	c.1677G>A	p.Glu559=	exon 16/19	chr3	C	T	ENSG00000C	NA	SNV Splice_Region	LOW	Unknown	NA	ENST000003	0,0175	1083	T5	C8101.mFF202103
FGFR3	c.895G>A	p.Gly299Ser	exon 7/18	chr4	G	A	ENSG00000C	COSV990381	SNV Missense	MODERATE	Unknown	NA	ENST000004	0,0186	1075	T5	C8101.mFF202103
FGFR3	c.1405C>T	p.Arg469Trp	exon 10/18	chr4	C	T	ENSG00000C	NA	SNV Missense	MODERATE	Unknown	NA	ENST000004	0,0143	1046	T5	C8101.mFF202103
FGFR3	c.1849G>A	p.Asp617Asn	exon 14/18	chr4	G	A	ENSG00000C	NA	SNV Missense	MODERATE	Likely Loss-of-function	Unknown	ENST000004	0,0135	1189	T5	C8101.mFF202103

DHX15	c.677G>A	p.Cys226Tyr	exon 3/14	chr4	C	T	ENSG000001	NA	SNV Missense	MODERATE	Unknown	NA	ENST000003	0,0128	1169	T5	C8101.mFF202103
PDGFRA	c.937G>A	p.Gly313Ser	exon 7/23	chr4	G	A	ENSG000001	COSV572730	SNV Missense	MODERATE	Unknown	NA	ENST000002	0,0109	1288	T5	C8101.mFF202103
PDGFRA	c.3161C>T	p.Ser1054Phe	exon 23/23	chr4	C	T	ENSG000001	COSV572748	SNV Missense	MODERATE	Unknown	NA	ENST000002	0,0115	1216	T5	C8101.mFF202103
KDR	c.3572G>A	p.Gly1191Glu	exon 27/30	chr4	C	T	ENSG000001	COSV557694	SNV Missense	MODERATE	Unknown	NA	ENST000002	0,046	1282	T5	C8101.mFF202103
KDR	c.3169C>T	p.Pro1057Ser	exon 23/30	chr4	G	A	ENSG000001	rs867547352,	SNV Missense	MODERATE	Unknown	NA	ENST000002	0,0213	1125	T5	C8101.mFF202103
EPHA5	c.98C>T	p.Ser33Phe	exon 1/18	chr4	G	A	ENSG000001	NA	SNV Missense	MODERATE	Unknown	NA	ENST000002	0,0214	701	T5	C8101.mFF202103
EPHA5	c.76G>A	p.Ala26Thr	exon 1/18	chr4	C	T	ENSG000001	NA	SNV Missense	MODERATE	Unknown	NA	ENST000002	0,0225	489	T5	C8101.mFF202103
TET2	c.3902C>T	p.Ala1301Val	exon 7/11	chr4	C	T	ENSG000001	NA	SNV Missense	MODERATE	Likely Loss-of-function	Likely Oncogenic	ENST000003	0,0184	980	T5	C8101.mFF202103
INPP4B	c.1301C>T	p.Ser434Phe	exon 14/24	chr4	G	A	ENSG000001	COSV537391	SNV Missense	MODERATE	Unknown	NA	ENST000002	0,0127	1025	T5	C8101.mFF202103
FBXW7	c.1478G>A	p.Cys493Tyr	exon 10/12	chr4	C	T	ENSG000001	NA	SNV Missense	MODERATE	Unknown	NA	ENST000002	0,0204	1129	T5	C8101.mFF202103
FBXW7	c.1102G>A	p.Gly368Arg	exon 7/12	chr4	C	T	ENSG000001	COSV559346	SNV Missense	MODERATE	Unknown	NA	ENST000002	0,0188	1011	T5	C8101.mFF202103
FBXW7	c.200G>A	p.Gly67Glu	exon 2/12	chr4	C	T	ENSG000001	NA	SNV Missense	MODERATE	Unknown	NA	ENST000002	0,0204	1419	T5	C8101.mFF202103
FBXW7	c.50G>A	p.Gly17Asp	exon 2/12	chr4	C	T	ENSG000001	NA	SNV Missense	MODERATE	Unknown	NA	ENST000002	0,0133	1205	T5	C8101.mFF202103
FAT1	c.13232C>T	p.Pro4411Leu	exon 27/27	chr4	G	A	ENSG000000	NA	SNV Missense	MODERATE	Unknown	NA	ENST000004	0,0158	1392	T5	C8101.mFF202103
FAT1	c.6554C>T	p.Pro2185Leu	exon 10/27	chr4	G	A	ENSG000000	NA	SNV Missense	MODERATE	Unknown	NA	ENST000004	0,0188	1436	T5	C8101.mFF202103
FAT1	c.6507C>G	p.Ile2169Met	exon 10/27	chr4	G	C	ENSG000000	NA	SNV Missense	MODERATE	Unknown	NA	ENST000004	0,5066	1516	T4	C8101.mFF202103
FAT1	c.5968G>A	p.Glu1990Lys	exon 10/27	chr4	C	T	ENSG000000	NA	SNV Missense	MODERATE	Unknown	NA	ENST000004	0,0228	1493	T5	C8101.mFF202103
FAT1	c.1990G>A	p.Ala664Thr	exon 2/27	chr4	C	T	ENSG000000	NA	SNV Missense	MODERATE	Unknown	NA	ENST000004	0,0198	1363	T5	C8101.mFF202103
IL7R	c.778G>A	p.Ala260Thr	exon 6/8	chr5	G	A	ENSG000001	rs147153824	SNV Missense	MODERATE	Unknown	NA	ENST000003	0,0168	1014	T5	C8101.mFF202103
RICTOR	c.2689G>A	p.Glu897Lys	exon 27/38	chr5	C	T	ENSG000001	NA	SNV Missense	MODERATE	Unknown	NA	ENST000003	0,0197	711	T5	C8101.mFF202103
RICTOR	c.764C>T	p.Ala255Val	exon 9/38	chr5	G	A	ENSG000001	NA	SNV Missense	MODERATE	Unknown	NA	ENST000003	0,0181	554	T5	C8101.mFF202103
FGF10	c.412G>A	p.Gly138Arg	exon 2/3	chr5	C	T	ENSG000000	rs866583243	SNV Missense	MODERATE	Unknown	NA	ENST000002	0,015	802	T5	C8101.mFF202103
FGF10	c.145G>A	p.Ala49Thr	exon 1/3	chr5	C	T	ENSG000000	NA	SNV Missense	MODERATE	Unknown	NA	ENST000002	0,0177	1582	T5	C8101.mFF202103
MAP3K1	c.3910C>T	p.Pro1304Ser	exon 16/20	chr5	C	T	ENSG000000	NA	SNV Missense	MODERATE	Unknown	NA	ENST000003	0,0187	750	T5	C8101.mFF202103
PIK3R1	c.871G>A	p.Glu291Lys	exon 7/16	chr5	G	A	ENSG000001	COSV571408	SNV Missense	MODERATE	Unknown	NA	ENST000005	0,0127	867	T5	C8101.mFF202103
APC	c.1610G>A	p.Ser537Asn	exon 13/16	chr5	G	A	ENSG000001	rs100152663(	SNV Missense	MODERATE	Unknown	NA	ENST000002	0,0179	1005	T5	C8101.mFF202103
APC	c.3488G>A	p.Ser1163Asn	exon 16/16	chr5	G	A	ENSG000001	COSV573551	SNV Missense	MODERATE	Unknown	NA	ENST000002	0,0189	1164	T5	C8101.mFF202103
APC	c.5552G>A	p.Gly1851Glu	exon 16/16	chr5	G	A	ENSG000001	COSV573691	SNV Missense	MODERATE	Unknown	NA	ENST000002	0,0143	1260	T5	C8101.mFF202103
APC	c.7473G>A	p.Met2491Ile	exon 16/16	chr5	G	A	ENSG000001	rs155408838(	SNV Missense	MODERATE	Unknown	NA	ENST000002	0,0175	1547	T5	C8101.mFF202103
CTNNA1	c.365G>A	p.Gly122Asp	exon 4/18	chr5	G	A	ENSG000000	NA	SNV Missense	MODERATE	Unknown	NA	ENST000003	0,0151	1060	T5	C8101.mFF202103
CSF1R	c.2036C>T	p.Ala679Val	exon 15/22	chr5	G	A	ENSG000001	NA	SNV Missense	MODERATE	Unknown	NA	ENST000002	0,0134	1498	T5	C8101.mFF202103
CSF1R	c.1558G>A	p.Val520Ile	exon 11/22	chr5	C	T	ENSG000001	NA	SNV Missense	MODERATE	Unknown	NA	ENST000002	0,0301	598	T5	C8101.mFF202103
CSF1R	c.107C>T	p.Ala36Val	exon 3/22	chr5	G	A	ENSG000001	NA	SNV Missense	MODERATE	Unknown	NA	ENST000002	0,0373	1636	T5	C8101.mFF202103
FGFR4	c.563G>A	p.Gly188Glu	exon 5/18	chr5	G	A	ENSG000001	NA	SNV Missense	MODERATE	Unknown	NA	ENST000002	0,01	1395	T5	C8101.mFF202103
FGFR4	c.1862C>T	p.Thr621Ile	exon 14/18	chr5	C	T	ENSG000001	NA	SNV Missense	MODERATE	Unknown	NA	ENST000002	0,0138	1449	T5	C8101.mFF202103
FGFR4	c.2204C>T	p.Thr735Ile	exon 17/18	chr5	C	T	ENSG000001	NA	SNV Missense	MODERATE	Unknown	NA	ENST000002	0,014	1073	T5	C8101.mFF202103
NSD1	c.1508C>T	p.Ser503Phe	exon 5/23	chr5	C	T	ENSG000001	NA	SNV Missense	MODERATE	Unknown	NA	ENST000004	0,0115	1221	T5	C8101.mFF202103
NSD1	c.4874C>T	p.Pro1625Leu	exon 13/23	chr5	C	T	ENSG000001	NA	SNV Missense	MODERATE	Unknown	NA	ENST000004	0,0125	1276	T5	C8101.mFF202103
NSD1	c.7991C>T	p.Ser2664Phe	exon 23/23	chr5	C	T	ENSG000001	NA	SNV Missense	MODERATE	Unknown	NA	ENST000004	0,0097	1440	T5	C8101.mFF202103
FLT4	c.2647+8G>A	NA	exon NA	chr5	C	T	ENSG000000	NA	SNV Splice_Region	LOW	Unknown	NA	ENST000002	0,0114	1493	T5	C8101.mFF202103
IRF4	c.1082C>T	p.Thr361Ile	exon 7/9	chr6	C	T	ENSG000001	NA	SNV Missense	MODERATE	Unknown	NA	ENST000003	0,0145	1514	T5	C8101.mFF202103
H3C4	c.101G>A	p.Gly34Asp	exon 1/1	chr6	C	T	ENSG000001	COSV619125	SNV Missense	MODERATE	Unknown	NA	ENST000003	0,0195	921	T5	C8101.mFF202103
NOTCH4	c.5839G>A	p.Asp1947Asn	exon 30/30	chr6	C	T	ENSG000002	NA	SNV Missense	MODERATE	Unknown	NA	ENST000003	0,0158	1454	T5	C8101.mFF202103
NOTCH4	c.3656G>A	p.Arg1219Gln	exon 21/30	chr6	C	T	ENSG000002	rs151325272,	SNV Missense	MODERATE	Unknown	NA	ENST000003	0,4483	1401	T4	C8101.mFF202103
NOTCH4	c.3451G>A	p.Gly1151Ser	exon 21/30	chr6	C	T	ENSG000002	rs121675888(	SNV Missense	MODERATE	Unknown	NA	ENST000003	0,0366	1119	T5	C8101.mFF202103
NOTCH4	c.107C>T	p.Ala36Val	exon 2/30	chr6	G	A	ENSG000002	NA	SNV Missense	MODERATE	Unknown	NA	ENST000003	0,022	1044	T5	C8101.mFF202103
DAXX	c.1717G>A	p.Ala573Thr	exon 6/8	chr6	C	T	ENSG000002	NA	SNV Missense	MODERATE	Unknown	NA	ENST000003	0,0169	1476	T5	C8101.mFF202103
FANCE	c.901G>A	p.Gly301Arg	exon 4/10	chr6	G	A	ENSG000001	NA	SNV Missense	MODERATE	Unknown	NA	ENST000002	0,0125	1041	T5	C8101.mFF202103
PIM1	c.164G>A	p.Gly55Asp	exon 2/6	chr6	G	A	ENSG000001	COSV651664	SNV Missense	MODERATE	Unknown	NA	ENST000003	0,0309	745	T5	C8101.mFF202103
PIM1	c.823C>T	p.Pro275Ser	exon 6/6	chr6	C	T	ENSG000001	NA	SNV Missense	MODERATE	Unknown	NA	ENST000003	0,0117	1030	T5	C8101.mFF202103
EPHA7	c.236C>T	p.Pro79Leu	exon 3/17	chr6	G	A	ENSG000001	COSV651696	SNV Missense	MODERATE	Unknown	NA	ENST000003	0,0147	1021	T5	C8101.mFF202103
ROS1	c.3164G>A	p.Gly1055Glu	exon 21/43	chr6	C	T	ENSG000000	COSV638563	SNV Missense	MODERATE	Unknown	NA	ENST000003	0,0163	920	T5	C8101.mFF202103
ROS1	c.2814G>A	p.Gly938=	exon 19/43	chr6	C	T	ENSG000000	COSV638514	SNV Splice_Region	LOW	Unknown	NA	ENST000003	0,021	429	T5	C8101.mFF202103
LATS1	c.3181C>T	p.Leu1061Phe	exon 8/8	chr6	G	A	ENSG000001	NA	SNV Missense	MODERATE	Unknown	NA	ENST000005	0,0163	1044	T5	C8101.mFF202103
ZBTB2	c.874C>T	p.Pro292Ser	exon 3/3	chr6	G	A	ENSG000001	rs771958176	SNV Missense	MODERATE	Unknown	NA	ENST000003	0,0181	1385	T5	C8101.mFF202103
ZBTB2	c.349G>A	p.Gly117Arg	exon 3/3	chr6	C	T	ENSG000001	NA	SNV Missense	MODERATE	Unknown	NA	ENST000003	0,0205	1266	T5	C8101.mFF202103
ARID1B	c.1846C>T	p.Gln616Ter	exon 4/20	chr6	C	T	ENSG000000	NA	SNV Nonsense	HIGH	Likely Loss-of-function	Likely Oncogenic	ENST000003	0,0201	1044	T5	C8101.mFF202103
MAP3K4	c.2816G>A	p.Ser939Asn	exon 10/27	chr6	G	A	ENSG000000	NA	SNV Missense	MODERATE	Unknown	NA	ENST000003	0,0147	748	T5	C8101.mFF202103
MAP3K4	c.3011C>T	p.Ala1004Val	exon 12/27	chr6	C	T	ENSG000000	NA	SNV Missense	MODERATE	Unknown	NA	ENST000003	0,0167	957	T5	C8101.mFF202103
CARD11	c.2759G>A	p.Arg920His	exon 21/25	chr7	C	T	ENSG000001	NA	SNV Missense	MODERATE	Unknown	NA	ENST000003	0,0171	1109	T5	C8101.mFF202103
ETV1	c.1148C>T	p.Ala383Val	exon 13/14	chr7	G	A	ENSG000000	COSV541466	SNV Missense	MODERATE	Unknown	NA	ENST000004	0,0114	874	T5	C8101.mFF202103
EGFR	c.1376G>A	p.Gly459Glu	exon 12/28	chr7	G	A	ENSG000001	COSV518173	SNV Missense	MODERATE	Unknown	NA	ENST000002	0,014	1069	T5	C8101.mFF202103
MAGI2	c.2094G>A	p.Trp698Ter	exon 12/22	chr7	C	T	ENSG000001	NA	SNV Nonsense	HIGH	Unknown	NA	ENST000003	0,0169	1065	T5	C8101.mFF202103
GRM3	c.2528G>C	p.Arg843Thr	exon 5/6	chr7	G	C	ENSG000001	rs120374142(	SNV Missense	MODERATE	Unknown	NA	ENST000003	0,4792	1104	T3	C8101.mFF202103
CDK6	c.871G>A	p.Ala291Thr	exon 8/8	chr7	C	T	ENSG000001	NA	SNV Missense	MODERATE	Unknown	NA	ENST000004	0,0156	1088	T5	C8101.mFF202103
CUX1	c.2354G>A	p.Gly785Asp	exon 18/24	chr7	G	A	ENSG000002	NA	SNV Missense	MODERATE	Unknown	NA	ENST000003	0,0166	1388	T5	C8101.mFF202103
CUX1	c.2505G>A	p.Trp835Ter	exon 18/24	chr7	G	A	ENSG000002	NA	SNV Nonsense	HIGH	Likely Loss-of-function	Likely Oncogenic	ENST000003	0,0155	1480	T5	C8101.mFF202103
CUX1	c.1610G>A	p.Ser537Asn	exon 18/23	chr7	G	A	ENSG000002	NA	SNV Missense	MODERATE	Unknown	NA	ENST000004	0,0158	1015	T5	C8101.mFF202103
PIK3CG	c.1313C>T	p.Ala438Val	exon 2/11	chr7	C	T	ENSG000001	rs127144373(	SNV Missense	MODERATE	Unknown	NA	ENST000003	0,017	1533	T5	C8101.mFF202103
SMO	c.446C>T	p.Ala149Val	exon 2/12	chr7	C	T	ENSG000001	NA	SNV Missense	MODERATE	Unknown	NA	ENST000002	0,0215	1259	T5	C8101.mFF202103
SMO	c.1805G>A	p.Gly602Asp	exon 11/12	chr7	G	A	ENSG000001	NA	SNV Missense	MODERATE	Unknown	NA	ENST000002	0,0185	1027	T5	C8101.mFF202103
BRAF	c.388G>A	p.Val130Met	exon 3/18	chr7	C	T	ENSG000001	NA	SNV Missense	MODERATE	Unknown	NA	ENST000002	0,0161	1116	T5	C8101.mFF202103
KEL	c.1816G>A	p.Glu606Lys	exon 17/19	chr7	C	T	ENSG000001	NA	SNV Missense	MODERATE	Unknown	NA	ENST000003	0,0148	1288	T5	C8101.mFF202103
KEL	c.1772-1G>A	NA	exon NA	chr7	C	T	ENSG000001	NA	SNV Splice_Site	HIGH	Unknown	NA	ENST000003	0,017	825	T5	C8101.mFF202103
KEL	c.1541C>T	p.Ser514Phe	exon 14/19	chr7	G	A	ENSG000001	NA	SNV Missense	MODERATE	Unknown	NA	ENST000003	0,0164	1339	T5	C8101.mFF202103
KEL	c.443C>T	p.Ala148Val	exon 5/19	chr7	G	A	ENSG000001	NA	SNV Missense	MODERATE	Unknown	NA	ENST000003	0,0222	1172	T5	C8101.mFF202103
NRG1	c.1831C>T	p.Pro611Ser	exon 12/12	chr8	C	T	ENSG000001	rs777461776,	SNV Missense	MODERATE	Unknown	NA	ENST000002	0,0097	1555	T5	C8101.mFF202103
ZNF703	c.1045G>A	p.Gly349Ser	exon 2/2	chr8	G	A	ENSG000001	NA	SNV Missense	MODERATE	Unknown	NA	ENST000003	0,0211	997	T5	C8101.mFF202103
ADGRA2	c.227C>T	p.Pro76Leu	exon 1/19	chr8	C	T	ENSG000000	rs747200083	SNV Missense	MODERATE	Unknown	NA	ENST000004	0,0124	809	T5	C8101.mFF202103
ADGRA2	c.1124C>T	p.Thr375Ile	exon 9/19	chr8	C	T	ENSG000000	rs156334946(	SNV Missense	MODERATE	Unknown	NA	ENST000004	0,0136	1106	T5	C8101.mFF202103
ADGRA2	c.1258A>G	p.Thr420Ala	exon 9/19	chr8	A	G	ENSG000000	NA	SNV Missense	MODERATE	Unknown	NA	ENST000004	0,0176	737	T5	C8101.mFF202103

ADGRA2	c.1853C>T	p.Ser618Phe	exon 13/19	chr8	C	T	ENSG00000(	NA	SNV Missense	MODERATE	Unknown	NA	ENST000004	0,0184	1362		T5	C8101.mFF202103
ADGRA2	c.1883C>T	p.Ser628Phe	exon 13/19	chr8	C	T	ENSG00000(	COSV59441(	SNV Missense	MODERATE	Unknown	NA	ENST000004	0,0108	1296		T5	C8101.mFF202103
KAT6A	c.4171G>A	p.Gly1391Arg	exon 18/18	chr8	C	T	ENSG00000(	rs123680977	SNV Missense	MODERATE	Unknown	NA	ENST000003	0,015	1469		T5	C8101.mFF202103
PRKDC	c.5119G>A	p.Glu1707Lys	exon 40/87	chr8	C	T	ENSG000002	NA	SNV Missense	MODERATE	Unknown	NA	ENST000003	0,0283	920		T5	C8101.mFF202103
PRKDC	c.676G>A	p.Gly226Arg	exon 7/87	chr8	C	T	ENSG000002	NA	SNV Missense	MODERATE	Unknown	NA	ENST000003	0,0121	1236		T5	C8101.mFF202103
PRKDC	c.199G>A	p.Val67Ile	exon 2/87	chr8	C	T	ENSG000002	NA	SNV Missense	MODERATE	Unknown	NA	ENST000003	0,0233	987		T5	C8101.mFF202103
SOX17	c.337G>A	p.Glu113Lys	exon 2/2	chr8	G	A	ENSG000001	NA	SNV Missense	MODERATE	Unknown	NA	ENST000002	0,0122	819		T5	C8101.mFF202103
PREX2	c.943G>A	p.Ala315Thr	exon 8/40	chr8	G	A	ENSG00000(	rs184081186,	SNV Missense	MODERATE	Unknown	NA	ENST000002	0,0169	1008		T5	C8101.mFF202103
MYC	c.505G>A	p.Ala169Thr	exon 2/3	chr8	G	A	ENSG000001	COSV523771	SNV Missense	MODERATE	Unknown	NA	ENST000003	0,017	1468		T5	C8101.mFF202103
RECQL4	c.2188C>T	p.Pro730Ser	exon 13/22	chr8	G	A	ENSG000001	rs120977690	SNV Missense	MODERATE	Unknown	NA	ENST000004	0,0163	1168		T5	C8101.mFF202103
RECQL4	c.1396C>T	p.Pro466Ser	exon 8/22	chr8	G	A	ENSG000001	rs562809072	SNV Missense	MODERATE	Unknown	NA	ENST000004	0,021	1382		T5	C8101.mFF202103
RECQL4	c.997G>A	p.Ala333Thr	exon 5/22	chr8	C	T	ENSG000001	NA	SNV Missense	MODERATE	Unknown	NA	ENST000004	0,0169	1717		T5	C8101.mFF202103
RECQL4	c.370G>A	p.Gly124Ser	exon 5/22	chr8	C	T	ENSG000001	NA	SNV Missense	MODERATE	Unknown	NA	ENST000004	0,027	1000		T5	C8101.mFF202103
JAK2	c.2041C>T	p.Leu681Phe	exon 16/25	chr9	C	T	ENSG00000(	NA	SNV Missense	MODERATE	Unknown	NA	ENST000003	0,0155	901		T5	C8101.mFF202103
PDCD1LG2	c.433G>A	p.Ala145Thr	exon 4/7	chr9	G	A	ENSG000001	NA	SNV Missense	MODERATE	Unknown	NA	ENST000003	0,0137	1171		T5	C8101.mFF202103
PTPRD	c.2833G>A	p.Ala945Thr	exon 25/43	chr9	C	T	ENSG000001	NA	SNV Missense	MODERATE	Unknown	NA	ENST000003	0,035	1545		T5	C8101.mFF202103
PAX5	c.934C>T	p.Leu312Phe	exon 8/10	chr9	G	A	ENSG000001	NA	SNV Missense	MODERATE	Unknown	NA	ENST000003	0,0105	1234		T5	C8101.mFF202103
HNRNPK	c.199G>A	p.Ala67Thr	exon 5/17	chr9	C	T	ENSG000001	NA	SNV Missense	MODERATE	Unknown	NA	ENST000003	0,0167	896		T5	C8101.mFF202103
NTRK2	c.2161G>A	p.Asp721Asn	exon 19/21	chr9	G	A	ENSG000001	rs156449958	SNV Missense	MODERATE	Unknown	NA	ENST000003	0,0115	1307		T5	C8101.mFF202103
SYK	c.1584C>T	p.Ala528=	exon 12/14	chr9	C	T	ENSG000001	NA	SNV Splice_Region	LOW	Unknown	NA	ENST000003	0,0127	867		T5	C8101.mFF202103
PTCH1	c.1921C>T	p.Pro641Ser	exon 14/24	chr9	G	A	ENSG000001	NA	SNV Missense	MODERATE	Unknown	NA	ENST000003	0,0159	1323		T5	C8101.mFF202103
TGFBR1	c.655G>A	p.Val219Ile	exon 4/9	chr9	G	A	ENSG000001	COSV666248	SNV Missense	MODERATE	Unknown	NA	ENST000003	0,0132	1062		T5	C8101.mFF202103
TGFBR1	c.928G>A	p.Ala310Thr	exon 5/9	chr9	G	A	ENSG000001	NA	SNV Missense	MODERATE	Unknown	NA	ENST000003	0,0135	1039		T5	C8101.mFF202103
KLF4	c.712C>T	p.Pro238Ser	exon 3/5	chr9	G	A	ENSG000001	rs200919263	SNV Missense	MODERATE	Unknown	NA	ENST000003	0,018	1055		T5	C8101.mFF202103
KLF4	c.52C>T	p.Pro18Ser	exon 2/5	chr9	G	A	ENSG000001	NA	SNV Missense	MODERATE	Unknown	NA	ENST000003	0,0338	1035		T5	C8101.mFF202103
PPP6C	c.76-1G>A	NA	exon NA	chr9	C	T	ENSG000001	NA	SNV Splice_Site	HIGH	Likely Loss-of-function	Likely Oncogenic	ENST000003	0,02	599		T5	C8101.mFF202103
ABL1	c.374A>G	p.His125Arg	exon 3/11	chr9	A	G	ENSG00000(	NA	SNV Missense	MODERATE	Unknown	NA	ENST000003	0,0152	1515		T5	C8101.mFF202103
ABL1	c.1313C>T	p.Ser438Phe	exon 8/11	chr9	C	T	ENSG00000(	COSV593373	SNV Missense	MODERATE	Unknown	NA	ENST000003	0,0357	1177		T5	C8101.mFF202103
ABL1	c.2474C>T	p.Ala825Val	exon 11/11	chr9	C	T	ENSG00000(	rs135226417	SNV Missense	MODERATE	Unknown	NA	ENST000003	0,0223	1076		T5	C8101.mFF202103
TSC1	c.2900G>A	p.Gly967Asp	exon 22/23	chr9	C	T	ENSG000001	NA	SNV Missense	MODERATE	Unknown	NA	ENST000002	0,0136	1174		T5	C8101.mFF202103
TSC1	c.1222C>T	p.His408Tyr	exon 12/23	chr9	G	A	ENSG000001	rs137424131	SNV Missense	MODERATE	Unknown	NA	ENST000002	0,019	1266		T5	C8101.mFF202103
NOTCH1	c.7007G>A	p.Ser2336Asn	exon 34/34	chr9	C	T	ENSG000001	rs136335108	SNV Missense	MODERATE	Unknown	NA	ENST000002	0,0181	1104		T5	C8101.mFF202103
NOTCH1	c.6280G>A	p.Asp2094Asn	exon 34/34	chr9	C	T	ENSG000001	NA	SNV Missense	MODERATE	Unknown	NA	ENST000002	0,0133	1274		T5	C8101.mFF202103
NOTCH1	c.4430G>A	p.Gly1477Asp	exon 25/34	chr9	C	T	ENSG000001	rs134692866	SNV Missense	MODERATE	Unknown	NA	ENST000002	0,012	1499		T5	C8101.mFF202103
NOTCH1	c.1519G>A	p.Asp507Asn	exon 9/34	chr9	C	T	ENSG000001	NA	SNV Missense	MODERATE	Unknown	NA	ENST000002	0,0113	1061		T5	C8101.mFF202103
EGFL7	c.152C>T	p.Pro51Leu	exon 3/9	chr9	C	T	ENSG000001	rs368404574	SNV Missense	MODERATE	Unknown	NA	ENST000003	0,0105	1044		T5	C8101.mFF202103
EGFL7	c.592G>A	p.Glu198Lys	exon 7/9	chr9	G	A	ENSG000001	NA	SNV Missense	MODERATE	Unknown	NA	ENST000003	0,0105	1148		T5	C8101.mFF202103
TRAF2	c.506C>T	p.Pro169Leu	exon 5/11	chr9	C	T	ENSG000001	NA	SNV Missense	MODERATE	Unknown	NA	ENST000002	0,0185	702		T5	C8101.mFF202103
TRAF2	c.530C>T	p.Ala177Val	exon 6/11	chr9	C	T	ENSG000001	rs367860949	SNV Missense	MODERATE	Unknown	NA	ENST000002	0,0137	1167		T5	C8101.mFF202103
CRLF2	c.236G>A	p.Gly79Asp	exon 3/6	chrX	C	T	ENSG000002	rs139135272	SNV Missense	MODERATE	Unknown	NA	ENST000003	0,0122	1148		T5	C8101.mFF202103
ZRSR2	c.649G>A	p.Asp217Asn	exon 8/11	chrX	G	A	ENSG000001	NA	SNV Missense	MODERATE	Unknown	NA	ENST000003	0,0175	1261		T5	C8101.mFF202103
ZRSR2	c.688G>A	p.Glu230Lys	exon 8/11	chrX	G	A	ENSG000001	NA	SNV Missense	MODERATE	Unknown	NA	ENST000003	0,0144	1249		T5	C8101.mFF202103
KDM6A	c.1666C>T	p.Pro556Ser	exon 16/29	chrX	C	T	ENSG000001	COSV65046(	SNV Missense	MODERATE	Unknown	NA	ENST000003	0,0106	1512		T5	C8101.mFF202103
KDM6A	c.1724G>A	p.Gly575Asp	exon 16/29	chrX	G	A	ENSG000001	NA	SNV Missense	MODERATE	Unknown	NA	ENST000003	0,0265	1508		T5	C8101.mFF202103
RBM10	c.1258G>A	p.Gly420Ser	exon 13/24	chrX	G	A	ENSG000001	NA	SNV Missense	MODERATE	Unknown	NA	ENST000003	0,0254	394		T5	C8101.mFF202103
RBM10	c.2083G>A	p.Ala695Thr	exon 18/24	chrX	G	A	ENSG000001	NA	SNV Missense	MODERATE	Unknown	NA	ENST000003	0,0118	1099		T5	C8101.mFF202103
RBM10	c.2150C>T	p.Ala717Val	exon 19/24	chrX	C	T	ENSG000001	rs868946632	SNV Missense	MODERATE	Unknown	NA	ENST000003	0,0108	1394		T5	C8101.mFF202103
RBM10	c.2573G>A	p.Ser858Asn	exon 23/24	chrX	G	A	ENSG000001	NA	SNV Missense	MODERATE	Unknown	NA	ENST000003	0,0114	1496		T5	C8101.mFF202103
KDM5C	c.4480G>A	p.Glu1494Lys	exon 26/26	chrX	C	T	ENSG000001	NA	SNV Missense	MODERATE	Unknown	NA	ENST000003	0,0148	1419		T5	C8101.mFF202103
KDM5C	c.4117+1G>A	NA	exon NA	chrX	C	T	ENSG000001	NA	SNV Splice_Site	HIGH	Likely Loss-of-function	Likely Oncogenic	ENST000003	0,013	1461		T5	C8101.mFF202103
KDM5C	c.3967G>A	p.Glu1323Lys	exon 23/26	chrX	C	T	ENSG000001	NA	SNV Missense	MODERATE	Unknown	NA	ENST000003	0,0125	1204		T5	C8101.mFF202103
KDM5C	c.3154G>A	p.Glu1052Lys	exon 21/26	chrX	C	T	ENSG000001	NA	SNV Missense	MODERATE	Unknown	NA	ENST000003	0,0119	1426		T5	C8101.mFF202103
KDM5C	c.2723G>A	p.Gly908Glu	exon 19/26	chrX	C	T	ENSG000001	rs782762372	SNV Missense	MODERATE	Unknown	NA	ENST000003	0,0214	1264		T5	C8101.mFF202103
KDM5C	c.739G>A	p.Gly247Ser	exon 6/26	chrX	C	T	ENSG000001	NA	SNV Missense	MODERATE	Unknown	NA	ENST000003	0,0253	1422		T5	C8101.mFF202103
SMC1A	c.3670C>T	p.Pro1224Ser	exon 25/25	chrX	G	A	ENSG00000(	NA	SNV Missense	MODERATE	Unknown	NA	ENST000003	0,0133	1275		T5	C8101.mFF202103
AMER1	c.1253G>A	p.Arg418Gln	exon 2/2	chrX	C	T	ENSG000001	rs758890390,	SNV Missense	MODERATE	Unknown	NA	ENST000003	0,0143	1675		T5	C8101.mFF202103
AR	c.1937C>T	p.Ala646Val	exon 4/8	chrX	C	T	ENSG000001	CM962533,C(	SNV Missense	MODERATE	Unknown	NA	ENST000003	0,0147	1290		T5	C8101.mFF202103
**MED12**	c.97G>A	p.Glu33Lys	exon 1/45	chrX	G	A	ENSG000001	rs769202858,	SNV Missense	MODERATE	Likely Loss-of-function	Oncogenic	ENST000003	0,0605	843		T2	C8101.mFF202103
MED12	c.1366G>A	p.Val456Ile	exon 10/45	chrX	G	A	ENSG000001	NA	SNV Missense	MODERATE	Unknown	NA	ENST000003	0,0225	1112		T5	C8101.mFF202103
**MED12**	c.3271G>A	p.Glu1091Lys	exon 23/45	chrX	G	A	ENSG000001	COSV61341(	SNV Missense	MODERATE	Unknown	NA	ENST000003	0,0503	1313		T3	C8101.mFF202103
TAF1	c.4985C>T	p.Pro1662Leu	exon 34/39	chrX	C	T	ENSG000001	NA	SNV Missense	MODERATE	Unknown	NA	ENST000004	0,0212	801		T5	C8101.mFF202103
ATRX	c.4351G>A	p.Glu1451Lys	exon 15/35	chrX	C	T	ENSG00000(	NA	SNV Missense	MODERATE	Unknown	NA	ENST000003	0,0215	651		T5	C8101.mFF202103
STAG2	c.2366C>T	p.Thr789Ile	exon 25/35	chrX	C	T	ENSG000001	NA	SNV Missense	MODERATE	Unknown	NA	ENST000002	0,0471	446		T5	C8101.mFF202103
STAG2	c.2456C>T	p.Ser819Phe	exon 25/35	chrX	C	T	ENSG000001	NA	SNV Missense	MODERATE	Unknown	NA	ENST000002	0,0158	758		T5	C8101.mFF202103
BCORL1	c.1187C>T	p.Pro396Leu	exon 3/12	chrX	C	T	ENSG00000(	NA	SNV Missense	MODERATE	Unknown	NA	ENST000005	0,0197	1119		T5	C8101.mFF202103
BCORL1	c.2653C>T	p.Pro885Ser	exon 3/12	chrX	C	T	ENSG00000(	rs371566085,	SNV Missense	MODERATE	Unknown	NA	ENST000005	0,0144	1323		T5	C8101.mFF202103
MDM4									CNV							1,534	T5	C5501.mFF202103
MYC									CNV							1,548	T5	C5501.mFF202103
MCL1	c.513_515del	p.Glu171del	exon 1/3	chr1	TCC	-	ENSG000001	rs759789515	SNV In_Frame_Del	MODERATE	Unknown	NA	ENST000003	0,0165	2424		T5	C5501.mFF202103
TET1	c.3053_3055deli	p.Asn1018_Lys1019	exon 4/12	chr10	ATA	GTG	ENSG000001	rs71483917	SNV Missense	MODERATE	Unknown	NA	ENST000003	0,1387	887		T4	C5501.mFF202103
SLX4	c.2854_2855deli	p.Ala952Met	exon 12/15	chr16	GC	AT	ENSG000001	rs863224277	SNV Missense	MODERATE	Unknown	NA	ENST000002	0,9545	549		T4	C5501.mFF202103
**SMAD4**	c.1082G>T	p.Arg361Leu	exon 9/12	chr18	G	T	ENSG000001	rs377767347,	SNV Missense	MODERATE	Likely Loss-of-function	Likely Oncogenic	ENST000003	0,6802	688		T2	C5501.mFF202103
ERCC2	c.260T>C	p.Leu87Pro	exon 5/23	chr19	A	G	ENSG000001	rs779462120	SNV Missense	MODERATE	Unknown	NA	ENST000003	0,4825	912		T3	C5501.mFF202103
BARD1	c.1518_1519inv	p.Val507Met	exon 6/11	chr2	CA	TG	ENSG000001	rs386654966	SNV Missense	MODERATE	Unknown	NA	ENST000002	1	1263		T4	C5501.mFF202103
**APC**	c.4459_4484del	p.Thr1487TyrfsTer1ε	exon 16/16	chr5	ACTTTATTA(	-	ENSG000001	NA	SNV Frame_Shift_D	HIGH	Likely Loss-of-function	Likely Oncogenic	ENST000002	0,6617	798		T2	C5501.mFF202103
FLT4	c.1927G>A	p.Val643Ile	exon 13/30	chr5	C	T	ENSG00000(	rs150093355	SNV Missense	MODERATE	Unknown	NA	ENST000002	0,4895	664		T4	C5501.mFF202103
CCND3	c.774_775delins	p.Ser259Ala	exon 5/5	chr6	AG	CA	ENSG000001	rs386700585	SNV Missense	MODERATE	Unknown	NA	ENST000003	0,4708	960		T4	C5501.mFF202103
PRDM1	c.2140C>T	p.Pro714Ser	exon 7/7	chr6	C	T	ENSG00000(	rs778760250	SNV Missense	MODERATE	Unknown	NA	ENST000003	0,4906	1537		T3	C5501.mFF202103
CUX1	c.4433G>A	p.Gly1478Asp	exon 24/24	chr7	G	A	ENSG000002	rs145457239	SNV Missense	MODERATE	Unknown	NA	ENST000003	0,4395	628		T3	C5501.mFF202103
PRKDC	c.2230G>A	p.Asp744Asn	exon 20/87	chr8	C	T	ENSG000002	rs35938758	SNV Missense	MODERATE	Unknown	NA	ENST000003	0,8137	687		T3	C5501.mFF202103
MDM4									CNV							1,499	T5	C3301.mFF202103

EMSY	c.3775G>A	p.Gly1259Ser	exon 20/21	chr11	G	A	ENSG000001	NA	SNV	Missense	MODERATE	Unknown	NA	ENST000005	0,4926	950		T3	C3301.mFF202103
**ERBB3**	c.1889T>C	p.Leu630Ser	exon 16/28	chr12	T	C	ENSG00000C	NA	SNV	Missense	MODERATE	Unknown	NA	ENST000002	0,4709	860		T3	C3301.mFF202103
CDK4	c.886C>T	p.His296Tyr	exon 8/8	chr12	G	A	ENSG000001	rs2227954	SNV	Missense	MODERATE	Unknown	NA	ENST000002	0,502	1016		T4	C3301.mFF202103
**RB1**	c.958C>T	p.Arg320Ter	exon 10/27	chr13	C	T	ENSG000001	rs121913300,	SNV	Nonsense	HIGH	Likely Loss-of-function	Likely Oncogenic	ENST000002	0,3625	309		T2	C3301.mFF202103
**RB1**	c.1190C>A	p.Ser397Ter	exon 12/27	chr13	C	A	ENSG000001	CM961227,C	SNV	Nonsense	HIGH	Likely Loss-of-function	Likely Oncogenic	ENST000002	0,3954	473		T2	C3301.mFF202103
IRS2	c.1291A>G	p.Met431Val	exon 1/2	chr13	T	C	ENSG000001	NA	SNV	Missense	MODERATE	Unknown	NA	ENST000003	0,4046	173		T3	C3301.mFF202103
**TP53**	c.976G>T	p.Glu326Ter	exon 9/11	chr17	C	A	ENSG000001	rs876659384,	SNV	Nonsense	HIGH	Likely Loss-of-function	Likely Oncogenic	ENST000002	0,9301	1001		T2	C3301.mFF202103
NF1	c.3662T>G	p.Leu1221Arg	exon 27/58	chr17	T	G	ENSG000001	CM111456	SNV	Missense	MODERATE	Unknown	NA	ENST000003	0,0169	1008		T5	C3301.mFF202103
SOX9	c.859dup	p.Glu287GlyfsTer9	exon 3/3	chr17	-	G	ENSG000001	NA	SNV	Frame_Shift_In	HIGH	Likely Loss-of-function	Likely Oncogenic	ENST000002	0,4278	1267		T3	C3301.mFF202103
BARD1	c.1518_1519inv	p.Val507Met	exon 6/11	chr2	CA	TG	ENSG000001	rs386654966	SNV	Missense	MODERATE	Unknown	NA	ENST000002	1	674		T4	C3301.mFF202103
FANCD2	c.2273G>C	p.Cys758Ser	exon 25/43	chr3	G	C	ENSG000001	rs540805431	SNV	Missense	MODERATE	Unknown	NA	ENST000002	0,4844	578		T4	C3301.mFF202103
**APC**	c.3295_3296del	p.Val1099PhefsTer1	exon 16/16	chr5	TG	-	ENSG000001	rs1064794228	SNV	Frame_Shift_D	HIGH	Likely Loss-of-function	Likely Oncogenic	ENST000002	0,4394	949		T2	C3301.mFF202103
**APC**	c.3942_3964del	p.Ala1316SerfsTer8	exon 16/16	chr5	GGTCAGCTC	-	ENSG000001	NA	SNV	Frame_Shift_D	HIGH	Likely Loss-of-function	Likely Oncogenic	ENST000002	0,4463	986		T2	C3301.mFF202103
NPM1	c.495C>G	p.Asp165Glu	exon 6/11	chr5	C	G	ENSG000001	rs146936311,	SNV	Missense	MODERATE	Unknown	NA	ENST000002	0,5027	549		T4	C3301.mFF202103
DDX41	c.138+5G>T	NA	exon NA	chr5	C	A	ENSG000001	rs187714514	SNV	Splice_Region	LOW	Unknown	NA	ENST000005	0,4917	901		T4	C3301.mFF202103
MDC1	c.3774_3775deli	p.Pro1259Ser	exon 10/15	chr6	GC	AT	ENSG000001	COSV645239	SNV	Missense	MODERATE	Unknown	NA	ENST000003	0,0156	962		T5	C3301.mFF202103
NOTCH4	c.2513del	p.Gly838GlufsTer7	exon 16/30	chr6	C	-	ENSG000002	NA	SNV	Frame_Shift_D	HIGH	Likely Loss-of-function	Likely Oncogenic	ENST000003	0,4655	999		T2	C3301.mFF202103
INHBA	c.1085C>T	p.Ser362Phe	exon 3/3	chr7	G	A	ENSG000001	COSV542239	SNV	Missense	MODERATE	Unknown	NA	ENST000002	0,448	1308		T3	C3301.mFF202103
PDGFRA									CNV								1,47	T5	C0701.mFF202103
ERBB2									CNV								1,772	T5	C0701.mFF202103
BRCA1									CNV								1,684	T5	C0701.mFF202103
RPS6KB1									CNV								1,546	T5	C0701.mFF202103
SPEN	c.6037G>A	p.Ala2013Thr	exon 11/15	chr1	G	A	ENSG00000C	rs766851755,	SNV	Missense	MODERATE	Unknown	NA	ENST000003	0,0469	1130		T5	C0701.mFF202103
ARID1A	c.5719A>T	p.Ile1907Phe	exon 20/20	chr1	A	T	ENSG000001	rs139230162,	SNV	Missense	MODERATE	Unknown	NA	ENST000003	0,0605	1439		T4	C0701.mFF202103
KMT2A	c.9947C>T	p.Ala3316Val	exon 27/36	chr11	C	T	ENSG000001	rs201447376	SNV	Missense	MODERATE	Unknown	NA	ENST000005	0,9381	1485		T4	C0701.mFF202103
CDK8	c.1096G>C	p.Asp366His	exon 11/13	chr13	G	C	ENSG000001	COSV674221	SNV	Missense	MODERATE	Unknown	NA	ENST000003	0,0362	636		T5	C0701.mFF202103
BRCA2	c.9613G>C	p.Ala3205Pro	exon 26/28	chr13	G	C	ENSG000001	rs528504546,	SNV	Missense	MODERATE	Unknown	Unknown	ENST000005	0,0178	1180		T5	C0701.mFF202103
TSHR	c.463A>T	p.Ile155Leu	exon 5/10	chr14	A	T	ENSG000001	rs141293178	SNV	Missense	MODERATE	Unknown	NA	ENST000002	0,4755	980		T4	C0701.mFF202103
NCOR1	c.2366G>C	p.Ser789Thr	exon 20/46	chr17	C	G	ENSG000001	rs61754982,C	SNV	Missense	MODERATE	Unknown	NA	ENST000002	0,3608	1929		T4	C0701.mFF202103
CD79B	c.323T>C	p.Leu108Pro	exon 3/6	chr17	A	G	ENSG00000C	NA	SNV	Missense	MODERATE	Unknown	NA	ENST000003	0,0088	2960		T5	C0701.mFF202103
EML4	c.2204A>G	p.Tyr735Cys	exon 20/23	chr2	A	G	ENSG000001	NA	SNV	Missense	MODERATE	Unknown	NA	ENST000003	0,0138	654		T5	C0701.mFF202103
BARD1	c.1518_1519inv	p.Val507Met	exon 6/11	chr2	CA	TG	ENSG000001	rs386654966	SNV	Missense	MODERATE	Unknown	NA	ENST000002	1	996		T4	C0701.mFF202103
PDGFRB	c.3251_3256del	p.Pro1084_Glu1085	exon 23/23	chr5	GCTCTG	-	ENSG000001	rs779686590	SNV	In_Frame_Del	MODERATE	Unknown	NA	ENST000002	0,0204	1764		T5	C0701.mFF202103
RECQL4	c.1892G>A	p.Arg631His	exon 12/22	chr8	C	T	ENSG000001	rs771179943	SNV	Missense	MODERATE	Unknown	NA	ENST000004	0,0271	776		T5	C0701.mFF202103
MLLT3	c.226T>C	p.Ser76Pro	exon 3/11	chr9	A	G	ENSG000001	NA	SNV	Missense	MODERATE	Unknown	NA	ENST000003	0,8339	596		T3	C0701.mFF202103
PDGFRA									CNV								1,531	T5	aP490.mFF202103
KIT									CNV								1,462	T5	aP490.mFF202103
CDK6									CNV								1,4	T5	aP490.mFF202103
FGFR1									CNV								1,361	T5	aP490.mFF202103
JAK2									CNV								1,49	T5	aP490.mFF202103
KRAS									CNV								1,379	T5	aP490.mFF202103
ERBB3									CNV								1,501	T5	aP490.mFF202103
CDK4									CNV								1,492	T5	aP490.mFF202103
MDM2									CNV								1,427	T5	aP490.mFF202103
ERBB2									CNV								1,521	T5	aP490.mFF202103
BRCA1									CNV								1,49	T5	aP490.mFF202103
RPS6KB1									CNV								1,479	T5	aP490.mFF202103
CCNE1									CNV								1,911	T5	aP490.mFF202103
AKT2									CNV								1,969	T5	aP490.mFF202103
ERCC2									CNV								1,591	T5	aP490.mFF202103
CSF3R	c.1260_1262deli	p.Pro421Leu	exon 10/17	chr1	GGA	AGG	ENSG000001	NA	SNV	Missense	MODERATE	Unknown	NA	ENST000003	0,0216	1020		T5	aP490.mFF202103
**PTEN**	c.253+1G>A	NA	exon NA	chr10	G	A	ENSG000001	rs587776667,	SNV	Splice_Site	HIGH	Likely Loss-of-function	Likely Oncogenic	ENST000003	0,6108	352		T2	aP490.mFF202103
**MEN1**	c.1331T>C	p.Leu444Pro	exon 9/10	chr11	A	G	ENSG000001	CM990850	SNV	Missense	MODERATE	Unknown	NA	ENST000003	0,6203	885		T2	aP490.mFF202103
FLT1	c.1294G>A	p.Glu432Lys	exon 10/30	chr13	C	T	ENSG000001	rs1473309404	SNV	Missense	MODERATE	Unknown	NA	ENST000002	0,6489	752		T3	aP490.mFF202103
FOXO1	c.153C>G	p.Asn51Lys	exon 1/3	chr13	G	C	ENSG000001	NA	SNV	Missense	MODERATE	Unknown	NA	ENST000003	0,8049	41		T3	aP490.mFF202103
MGA	c.2264C>T	p.Ala755Val	exon 6/24	chr15	C	T	ENSG000001	NA	SNV	Missense	MODERATE	Unknown	NA	ENST000002	0,6463	687		T3	aP490.mFF202103
B2M	c.112_123del	p.Gly38_Asn41del	exon 2/4	chr15	AATGGAAAC	-	ENSG000001	NA	SNV	In_Frame_Del	MODERATE	Unknown	NA	ENST000005	0,5877	764		T3	aP490.mFF202103
NF1	c.1891G>A	p.Gly631Arg	exon 17/58	chr17	G	A	ENSG000001	rs757424379,	SNV	Missense	MODERATE	Unknown	NA	ENST000003	0,62	621		T3	aP490.mFF202103
CIC	c.3862G>A	p.Glu1288Lys	exon 16/20	chr19	G	A	ENSG00000C	NA	SNV	Missense	MODERATE	Unknown	NA	ENST000005	0,2867	2403		T3	aP490.mFF202103
POLD1	c.49C>T	p.Arg17Trp	exon 2/27	chr19	C	T	ENSG00000C	rs570461545,	SNV	Missense	MODERATE	Unknown	NA	ENST000004	0,4049	1667		T4	aP490.mFF202103
**MSH6**	c.627+1G>A	NA	exon NA	chr2	G	A	ENSG000001	NA	SNV	Splice_Site	HIGH	Likely Loss-of-function	Likely Oncogenic	ENST000002	0,62	550		T2	aP490.mFF202103
BARD1	c.1518_1519inv	p.Val507Met	exon 6/11	chr2	CA	TG	ENSG000001	rs386654966	SNV	Missense	MODERATE	Unknown	NA	ENST000002	0,0862	1416		T4	aP490.mFF202103
EPHA3	c.919G>A	p.Glu307Lys	exon 4/17	chr3	G	A	ENSG00000C	rs148593404,	SNV	Missense	MODERATE	Unknown	NA	ENST000003	0,1472	1053		T4	aP490.mFF202103
**TET2**	c.4952_4961del	p.Pro1651ArgfsTer4	exon 11/11	chr4	CCCAGTCTC	-	ENSG000001	NA	SNV	Frame_Shift_D	HIGH	Likely Loss-of-function	Likely Oncogenic	ENST000003	0,6316	1064		T2	aP490.mFF202103
CARD11	c.1426G>A	p.Asp476Asn	exon 10/25	chr7	C	T	ENSG000001	rs131294317	SNV	Missense	MODERATE	Unknown	NA	ENST000003	0,3966	1591		T3	aP490.mFF202103
GRM3	c.1099C>T	p.Arg367Cys	exon 3/6	chr7	C	T	ENSG000001	NA	SNV	Missense	MODERATE	Unknown	NA	ENST000003	0,0172	2266		T5	aP490.mFF202103
PIK3CG	c.2630G>T	p.Gly877Val	exon 8/11	chr7	G	T	ENSG000001	NA	SNV	Missense	MODERATE	Unknown	NA	ENST000003	0,2089	1034		T3	aP490.mFF202103
CD274	c.286A>G	p.Asn96Asp	exon 3/7	chr9	A	G	ENSG000001	NA	SNV	Missense	MODERATE	Unknown	NA	ENST000003	0,3808	1946		T3	aP490.mFF202103
**TGFBR1**	c.671G>A	p.Trp224Ter	exon 4/9	chr9	G	A	ENSG000001	NA	SNV	Nonsense	HIGH	Likely Loss-of-function	Likely Oncogenic	ENST000003	0,3666	1451		T2	aP490.mFF202103
**TSC1**	c.663G>A	p.Lys221=	exon 7/23	chr9	C	T	ENSG000001	NA	SNV	Splice_Region	LOW	Likely Loss-of-function	Likely Oncogenic	ENST000002	0,4016	854		T2	aP490.mFF202103
NTRK1	c.236T>A	p.Leu79Gln	exon 2/17	chr1	T	A	ENSG000001	rs139140006	SNV	Missense	MODERATE	Unknown	NA	ENST000005	0,497	1519		T4	aP321.mFF202103
ATM	c.2932T>C	p.Ser978Pro	exon 20/63	chr11	T	C	ENSG000001	rs139552233,	SNV	Missense	MODERATE	Unknown	NA	ENST000002	0,456	647		T4	aP321.mFF202103
**KRAS**	c.34G>C	p.Gly12Arg	exon 2/6	chr12	C	G	ENSG000001	rs121913530,	SNV	Missense	MODERATE	Gain-of-function	Oncogenic	ENST000002	0,2112	1089		T2	aP321.mFF202103
TRAF7	c.253C>T	p.Arg85Cys	exon 5/21	chr16	C	T	ENSG000001	rs768139800	SNV	Missense	MODERATE	Unknown	NA	ENST000003	0,0443	1243		T5	aP321.mFF202103
SLX4	c.4682C>T	p.Thr1561Met	exon 13/15	chr16	G	A	ENSG000001	rs543847606,	SNV	Missense	MODERATE	Unknown	NA	ENST000002	0,4584	1562		T3	aP321.mFF202103
GRIN2A	c.82G>A	p.Glu28Lys	exon 2/13	chr16	C	T	ENSG000001	rs105705089	SNV	Missense	MODERATE	Unknown	NA	ENST000003	0,4889	1174		T3	aP321.mFF202103
TP53	c.920-3_920-1de	NA	exon NA	chr17	CTA	-	ENSG000001	NA	SNV	Splice_Site	HIGH	Likely Loss-of-function	Likely Oncogenic	ENST000002	0,0344	1483		T5	aP321.mFF202103
RNF43	c.1765C>G	p.Pro589Ala	exon 9/10	chr17	G	C	ENSG000001	COSV684595	SNV	Missense	MODERATE	Unknown	NA	ENST000005	0,4466	1863		T4	aP321.mFF202103
PDGFRB	c.3256_3257ins	p.Pro1084_Glu1085	exon 23/23	chr5	-	GCTCCG	ENSG000001	rs123869578	SNV	In_Frame_Ins	MODERATE	Unknown	NA	ENST000002	0,0097	1761		T5	aP321.mFF202103
ROS1	c.5348A>G	p.Tyr1783Cys	exon 32/43	chr6	T	C	ENSG00000C	rs125314958	SNV	Missense	MODERATE	Unknown	NA	ENST000003	0,467	893		T3	aP321.mFF202103

Table S3 MCB cytology

Sample_ID	Tumor_cells_content	Degenerated tumor cells [%]	Fibroblasts	Macrophages	Lymphocytes	Extracellular Matrix	Collagen	Nondistinctive Matrix	Comment
C9502m_MCB_D0_HE	high	2	no	no	no	no	no	no	necrosis in original tissue and MCBs
C9502m_MCB_D12_HE	high	2	no	no	no	focal	no	no	necrosis in original tissue and MCBs
C8802m_MCB_D0_HE	high	10	no	no	no	no	no	no	
C8802m_MCB_D12_HE	high	10	yes	yes	no	focal	no	yes	
C8101m_MCB_D0_HE	high	5	no	yes	no	no	no	no	necrosis in original tissue and MCBs
C8101m_MCB_D12_HE	high	2	no	no	no	no	no	no	necrosis in original tissue and MCBs
C5502m_MCB_D12_HE	high	5	no	yes	yes	focal	no	yes	necrosis in original tissue and MCBs
C3301m_MCB_D0_HE	high	2	no	no	no	no	no	no	
C3301m_MCB_D12_HE	high	2	no	no	no	no	no	no	
C0701m_MCB_D0_HE	high	0	no	no	no	no	no	no	necrosis in original tissue and MCBs
C0701m_MCB_D12_HE	high	0	no	no	no	no	no	no	necrosis in original tissue and MCBs
aP321_MCB_D12_HE_10	high	20	yes	no	no	focal	yes	no	necrosis in original tissue and MCBs
aP490m_MCB_D0_HE	high	0	no	no	no	no	no	no	necrosis in original tissue and MCBs
aP490m_MCB_D12_HE	high	0	no	no	no	no	no	no	necrosis in original tissue and MCBs

Supplementary Table 4: Differential expression original tumor tissue vs. PD tumroids

gene_id	log2baseM	log2FoldCh	pvalue	padj	stat	gene_type	gene_nam	gene_description	chr_scaffold
ENSG00000160211	6,314767	-2,569881	8,25E-10	1,04E-05	-12,07594	protein_coding	G6PD	glucose-6-phosphate dehydrogenase [Source:HGNC Symbol;Acc:HGNC:4057]	X
ENSG00000198431	7,462828	-2,683783	2,31E-09	1,04E-05	-11,29092	protein_coding	TXNRD1	thioredoxin reductase 1 [Source:HGNC Symbol;Acc:HGNC:12437]	12
ENSG00000005884	7,315545	-1,848754	2,57E-09	1,04E-05	-11,21304	protein_coding	ITGA3	integrin subunit alpha 3 [Source:HGNC Symbol;Acc:HGNC:6139]	17
ENSG00000066697	5,305379	-1,911781	3,34E-09	1,04E-05	-11,01966	protein_coding	MSANTD3	Myb/SANT DNA binding domain containing 3 [Source:HGNC Symbol;Acc:HGNC:23370]	9
ENSG00000181649	3,581229	-4,855776	3,84E-09	1,04E-05	-10,9193	protein_coding	PHLDA2	pleckstrin homology like domain family A member 2 [Source:HGNC Symbol;Acc:HGNC:12385]	11
ENSG00000145040	0,460229	-4,955782	1,65E-09	1,04E-05	-11,54333	protein_coding	UCN2	urocortin 2 [Source:HGNC Symbol;Acc:HGNC:18414]	3
ENSG00000161544	5,758284	-3,638556	6,27E-09	1,26E-05	-10,56885	protein_coding	CYGB	cytoglobin [Source:HGNC Symbol;Acc:HGNC:16505]	17
ENSG00000122641	7,056532	-2,993789	7,2E-09	1,26E-05	-10,47134	protein_coding	INHBA	inhibin subunit beta A [Source:HGNC Symbol;Acc:HGNC:6066]	7
ENSG00000181019	5,075154	-5,790874	5,5E-09	1,26E-05	-10,6617	protein_coding	NQO1	NAD(P)H quinone dehydrogenase 1 [Source:HGNC Symbol;Acc:HGNC:2874]	16
ENSG00000148834	5,743546	-1,67859	7,73E-09	1,26E-05	-10,42145	protein_coding	GSTO1	glutathione S-transferase omega 1 [Source:HGNC Symbol;Acc:HGNC:13312]	10
ENSG00000171067	5,644696	-2,12333	1,66E-08	2,33E-05	-9,90009	protein_coding	C11orf24	chromosome 11 open reading frame 24 [Source:HGNC Symbol;Acc:HGNC:1174]	11
ENSG00000196072	4,374352	-1,503467	1,94E-08	2,33E-05	-9,794075	protein_coding	BLOC1S2	biogenesis of lysosomal organelles complex 1 subunit 2 [Source:HGNC Symbol;Acc:HGNC:20984]	10
ENSG00000105329	7,61712	-2,94584	1,96E-08	2,33E-05	-9,786901	protein_coding	TGFB1	transforming growth factor beta 1 [Source:HGNC Symbol;Acc:HGNC:11766]	19
ENSG00000177628	5,477118	-1,828455	2,01E-08	2,33E-05	-9,77121	protein_coding	GBA	glucosylceramidase beta [Source:HGNC Symbol;Acc:HGNC:4177]	1
ENSG00000154127	5,204682	-3,254287	2,3E-08	2,36E-05	-9,681345	protein_coding	UBASH3B	ubiquitin associated and SH3 domain containing B [Source:HGNC Symbol;Acc:HGNC:29884]	11
ENSG00000137809	7,190484	-4,126736	2,32E-08	2,36E-05	-9,674103	protein_coding	ITGA11	integrin subunit alpha 11 [Source:HGNC Symbol;Acc:HGNC:6136]	15
ENSG00000023909	5,484389	-2,059232	2,48E-08	2,37E-05	-9,629934	protein_coding	GCLM	glutamate-cysteine ligase modifier subunit [Source:HGNC Symbol;Acc:HGNC:4312]	1
ENSG00000164171	6,885788	-4,901837	2,86E-08	2,59E-05	-9,536061	protein_coding	ITGA2	integrin subunit alpha 2 [Source:HGNC Symbol;Acc:HGNC:6137]	5
ENSG00000006327	4,881914	-3,196462	3,64E-08	3,1E-05	-9,380355	protein_coding	TNFRSF12A	TNF receptor superfamily member 12A [Source:HGNC Symbol;Acc:HGNC:18152]	16
ENSG00000117450	6,913436	-2,086219	3,81E-08	3,1E-05	-9,350553	protein_coding	PRDX1	peroxiredoxin 1 [Source:HGNC Symbol;Acc:HGNC:9352]	1
ENSG00000117385	6,452441	-1,956562	5,28E-08	3,51E-05	-9,141716	protein_coding	P3H1	prolyl 3-hydroxylase 1 [Source:HGNC Symbol;Acc:HGNC:19316]	1
ENSG00000113558	7,187025	-1,081492	5,6E-08	3,51E-05	-9,104693	protein_coding	SKP1	S-phase kinase associated protein 1 [Source:HGNC Symbol;Acc:HGNC:10899]	5
ENSG00000084112	7,075464	-1,784387	5,65E-08	3,51E-05	-9,098178	protein_coding	SSH1	slingshot protein phosphatase 1 [Source:HGNC Symbol;Acc:HGNC:30579]	12
ENSG00000057019	7,019116	-3,697916	5,8E-08	3,51E-05	-9,082004	protein_coding	DCBLD2	discoidin, CUB and LCCL domain containing 2 [Source:HGNC Symbol;Acc:HGNC:24627]	3
ENSG00000149948	3,006341	-4,801751	5,66E-08	3,51E-05	-9,097512	protein_coding	HMGA2	high mobility group AT-hook 2 [Source:HGNC Symbol;Acc:HGNC:5009]	12
ENSG00000018625	-0,78291	5,086617	5,83E-08	3,51E-05	9,078817	protein_coding	ATP1A2	ATPase Na+/K+ transporting subunit alpha 2 [Source:HGNC Symbol;Acc:HGNC:800]	1
ENSG00000166428	-1,991417	5,61104	4,62E-08	3,51E-05	9,227425	protein_coding	PLD4	phospholipase D family member 4 [Source:HGNC Symbol;Acc:HGNC:23792]	14
ENSG00000165476	6,092014	-1,570512	6,19E-08	3,58E-05	-9,041232	protein_coding	REEP3	receptor accessory protein 3 [Source:HGNC Symbol;Acc:HGNC:23711]	10
ENSG00000164935	0,468276	-4,209331	6,39E-08	3,58E-05	-9,020994	protein_coding	DCSTAMP	dendrocyte expressed seven transmembrane protein [Source:HGNC Symbol;Acc:HGNC:18549]	8
ENSG00000135318	6,160201	-4,77736	6,68E-08	3,6E-05	-8,992555	protein_coding	NT5E	5'-nucleotidase ecto [Source:HGNC Symbol;Acc:HGNC:8021]	6
ENSG00000213190	4,579577	-3,166269	7,02E-08	3,6E-05	-8,961404	protein_coding	MLLT11	MLLT11 transcription factor 7 cofactor [Source:HGNC Symbol;Acc:HGNC:16997]	1
ENSG00000167553	6,716972	-1,691344	7,09E-08	3,6E-05	-8,955727	protein_coding	TUBA1C	tubulin alpha 1c [Source:HGNC Symbol;Acc:HGNC:20768]	12
ENSG00000198768	3,166995	-8,240348	8,05E-08	3,96E-05	-8,876113	protein_coding	APCDD1L	APC down-regulated 1 like [Source:HGNC Symbol;Acc:HGNC:26892]	20
ENSG00000137331	6,438266	-3,891812	8,73E-08	4,01E-05	-8,825525	protein_coding	IER3	immediate early response 3 [Source:HGNC Symbol;Acc:HGNC:5392]	6
ENSG00000110092	7,887457	-4,491775	9,6E-08	4,01E-05	-8,766807	protein_coding	CCND1	cyclin D1 [Source:HGNC Symbol;Acc:HGNC:1582]	11
ENSG00000159228	5,888843	-1,545499	1,02E-07	4,01E-05	-8,730582	protein_coding	CBR1	carbonyl reductase 1 [Source:HGNC Symbol;Acc:HGNC:1548]	21
ENSG00000148572	4,317002	-1,538776	1,07E-07	4,01E-05	-8,697895	protein_coding	NRBF2	nuclear receptor binding factor 2 [Source:HGNC Symbol;Acc:HGNC:19692]	10
ENSG00000185651	5,668703	-1,203478	1,05E-07	4,01E-05	-8,710719	protein_coding	UBE2L3	ubiquitin conjugating enzyme E2 L3 [Source:HGNC Symbol;Acc:HGNC:12488]	22
ENSG00000151012	5,581649	-3,882208	1,1E-07	4,01E-05	-8,682579	protein_coding	SLC7A11	solute carrier family 7 member 11 [Source:HGNC Symbol;Acc:HGNC:11059]	4
ENSG00000144136	7,529056	-2,689024	1,06E-07	4,01E-05	-8,703773	protein_coding	SLC20A1	solute carrier family 20 member 1 [Source:HGNC Symbol;Acc:HGNC:10946]	2
ENSG00000161013	6,877946	-1,267444	1,06E-07	4,01E-05	-8,703626	protein_coding	MGAT4B	alpha-1,3-mannosyl-glycoprotein 4-beta-N-acetylglucosaminyltransferase B [Source:HGNC Symbol;Acc:HGNC:7048]	5
ENSG00000173457	6,023321	-1,80047	1,09E-07	4,01E-05	-8,690479	protein_coding	PPP1R14B	protein phosphatase 1 regulatory inhibitor subunit 14B [Source:HGNC Symbol;Acc:HGNC:9057]	11
ENSG00000203805	1,489824	-3,393087	1,11E-07	4,01E-05	-8,67674	protein_coding	PLPP4	phospholipid phosphatase 4 [Source:HGNC Symbol;Acc:HGNC:23531]	10
ENSG00000131055	-1,624917	5,111005	8,4E-08	4,01E-05	8,849222	protein_coding	COX4I2	cytochrome c oxidase subunit 4I2 [Source:HGNC Symbol;Acc:HGNC:16232]	20
ENSG00000131097	-2,2537	4,148625	1,08E-07	4,01E-05	8,696633	protein_coding	HIGD1B	HIG1 hypoxia inducible domain family member 1B [Source:HGNC Symbol;Acc:HGNC:24318]	17
ENSG00000069849	6,240078	-1,786075	1,23E-07	4,33E-05	-8,615845	protein_coding	ATP1B3	ATPase Na+/K+ transporting subunit beta 3 [Source:HGNC Symbol;Acc:HGNC:806]	3
ENSG00000148225	3,300889	2,421382	1,26E-07	4,36E-05	8,59975	protein_coding	WDR31	WD repeat domain 31 [Source:HGNC Symbol;Acc:HGNC:21421]	9
ENSG00000128595	8,545725	-2,191457	1,33E-07	4,52E-05	-8,564649	protein_coding	CALU	calumenin [Source:HGNC Symbol;Acc:HGNC:1458]	7
ENSG00000150093	9,601733	-2,231306	1,4E-07	4,66E-05	-8,533511	protein_coding	ITGB1	integrin subunit beta 1 [Source:HGNC Symbol;Acc:HGNC:6153]	10
ENSG00000164823	5,135988	-1,478025	1,58E-07	5,14E-05	-8,461065	protein_coding	OSGIN2	oxidative stress induced growth inhibitor family member 2 [Source:HGNC Symbol;Acc:HGNC:1355]	8

ENSG00000115233	5,825595	-1,270709	1,62E-07	5,15E-05	-8,448691	protein_coding	PSMD14	proteasome 26S subunit, non-ATPase 14 [Source:HGNC Symbol;Acc:HGNC:16889]	2
ENSG00000115963	6,273126	-3,139844	1,7E-07	5,31E-05	-8,418171	protein_coding	RND3	Rho family GTPase 3 [Source:HGNC Symbol;Acc:HGNC:671]	2
ENSG00000121310	4,906884	1,553143	1,82E-07	5,44E-05	8,377799	protein_coding	ECHDC2	enoyl-CoA hydratase domain containing 2 [Source:HGNC Symbol;Acc:HGNC:23408]	1
ENSG00000151176	7,239229	-1,384175	1,83E-07	5,44E-05	-8,374103	protein_coding	PLBD2	phospholipase B domain containing 2 [Source:HGNC Symbol;Acc:HGNC:27283]	12
ENSG00000166963	7,687929	-2,484327	1,86E-07	5,44E-05	-8,364158	protein_coding	MAP1A	microtubule associated protein 1A [Source:HGNC Symbol;Acc:HGNC:6835]	15
ENSG00000114270	7,047296	-3,984795	1,91E-07	5,44E-05	-8,348341	protein_coding	COL7A1	collagen type VII alpha 1 chain [Source:HGNC Symbol;Acc:HGNC:2214]	3
ENSG00000136026	7,879645	-2,09511	1,88E-07	5,44E-05	-8,358683	protein_coding	CKAP4	cytoskeleton associated protein 4 [Source:HGNC Symbol;Acc:HGNC:16991]	12
ENSG00000111328	6,284824	-2,0109	1,96E-07	5,5E-05	-8,331961	protein_coding	CDK2AP1	cyclin dependent kinase 2 associated protein 1 [Source:HGNC Symbol;Acc:HGNC:14002]	12
ENSG00000108094	5,344368	-1,013634	2,15E-07	5,82E-05	-8,277827	protein_coding	CUL2	cullin 2 [Source:HGNC Symbol;Acc:HGNC:2552]	10
ENSG00000115806	6,382739	-1,400553	2,14E-07	5,82E-05	-8,279422	protein_coding	GORASP2	golgi reassembly stacking protein 2 [Source:HGNC Symbol;Acc:HGNC:17500]	2
ENSG00000125868	8,374694	-1,374426	2,31E-07	6,15E-05	-8,235339	protein_coding	DSTN	destrin, actin depolymerizing factor [Source:HGNC Symbol;Acc:HGNC:15750]	20
ENSG00000102265	10,3167	-4,827212	2,38E-07	6,23E-05	-8,217779	protein_coding	TIMP1	TIMP metallopeptidase inhibitor 1 [Source:HGNC Symbol;Acc:HGNC:11820]	X
ENSG00000089063	6,190778	-1,140049	2,63E-07	6,67E-05	-8,158167	protein_coding	TMEM230	transmembrane protein 230 [Source:HGNC Symbol;Acc:HGNC:15876]	20
ENSG00000103313	-1,50383	4,893717	2,59E-07	6,67E-05	8,167154	protein_coding	MEFV	MEFV innate immuity regulator, pyrin [Source:HGNC Symbol;Acc:HGNC:6998]	16
ENSG00000164465	5,264915	-1,763247	2,69E-07	6,72E-05	-8,145069	protein_coding	DCBLD1	discoidin, CUB and LCCL domain containing 1 [Source:HGNC Symbol;Acc:HGNC:21479]	6
ENSG00000121807	-2,165983	5,28912	2,8E-07	6,89E-05	8,121327	protein_coding	CCR2	C-C motif chemokine receptor 2 [Source:HGNC Symbol;Acc:HGNC:1603]	3
ENSG00000147010	6,93766	-2,225847	3,07E-07	7,45E-05	-8,066498	protein_coding	SH3KBP1	SH3 domain containing kinase binding protein 1 [Source:HGNC Symbol;Acc:HGNC:13867]	X
ENSG00000164924	8,665336	-1,790179	3,19E-07	7,63E-05	-8,044026	protein_coding	YWHAZ	tyrosine 3-monooxygenase/tryptophan 5-monooxygenase activation protein zeta [Source:HGNC Symbol;Acc:HGNC:	8
ENSG00000142669	6,623383	-2,464485	3,27E-07	7,69E-05	-8,030299	protein_coding	SH3BGRL3	SH3 domain binding glutamate rich protein like 3 [Source:HGNC Symbol;Acc:HGNC:15568]	1
ENSG00000176170	4,230807	-2,959336	3,36E-07	7,79E-05	-8,014316	protein_coding	SPHK1	sphingosine kinase 1 [Source:HGNC Symbol;Acc:HGNC:11240]	17
ENSG00000065833	5,055899	-3,146153	3,42E-07	7,83E-05	-8,003666	protein_coding	ME1	malic enzyme 1 [Source:HGNC Symbol;Acc:HGNC:6983]	6
ENSG00000078098	5,003761	-4,058848	3,68E-07	8,23E-05	-7,961209	protein_coding	FAP	fibroblast activation protein alpha [Source:HGNC Symbol;Acc:HGNC:3590]	2
ENSG00000085063	7,547279	-1,862743	3,7E-07	8,23E-05	-7,958149	protein_coding	CD59	CD59 molecule (CD59 blood group) [Source:HGNC Symbol;Acc:HGNC:1689]	11
ENSG00000138604	5,134558	-1,808091	3,97E-07	8,62E-05	-7,916069	protein_coding	GLCE	glucuronic acid epimerase [Source:HGNC Symbol;Acc:HGNC:17855]	15
ENSG00000111252	6,865551	-2,679641	3,98E-07	8,62E-05	-7,915556	protein_coding	SH2B3	SH2B adaptor protein 3 [Source:HGNC Symbol;Acc:HGNC:29605]	12
ENSG00000133816	7,996177	-2,707112	4,11E-07	8,79E-05	-7,896523	protein_coding	MICAL2	microtubule associated monooxygenase, calponin and LIM domain containing 2 [Source:HGNC Symbol;Acc:HGNC:2	11
ENSG00000173530	4,171343	-3,151506	4,21E-07	8,88E-05	-7,883142	protein_coding	TNFRSF10D	TNF receptor superfamily member 10d [Source:HGNC Symbol;Acc:HGNC:11907]	8
ENSG00000166923	6,675977	-4,715745	4,53E-07	9,32E-05	-7,840215	protein_coding	GREM1	gremlin 1, DAN family BMP antagonist [Source:HGNC Symbol;Acc:HGNC:2001]	15
ENSG00000091490	7,068795	-1,620575	4,48E-07	9,32E-05	-7,846397	protein_coding	SEL1L3	SEL1L family member 3 [Source:HGNC Symbol;Acc:HGNC:29108]	4
ENSG00000172331	4,540481	-1,739695	4,67E-07	9,48E-05	-7,823466	protein_coding	BPGM	bisphosphoglycerate mutase [Source:HGNC Symbol;Acc:HGNC:1093]	7
ENSG00000103647	4,301217	-3,269928	4,85E-07	9,49E-05	-7,801302	protein_coding	CORO2B	coronin 2B [Source:HGNC Symbol;Acc:HGNC:2256]	15
ENSG00000185883	1,52442	-2,539678	4,78E-07	9,49E-05	-7,809391	protein_coding	ATP6V0C	ATPase H+ transporting V0 subunit c [Source:HGNC Symbol;Acc:HGNC:855]	16
ENSG00000278677	0,257483	3,045581	4,83E-07	9,49E-05	7,803738	protein_coding	H2AC17	H2A clustered histone 17 [Source:HGNC Symbol;Acc:HGNC:4735]	6
ENSG00000146242	4,911977	-3,077294	4,98E-07	9,63E-05	-7,786046	protein_coding	TPBG	trophoblast glycoprotein [Source:HGNC Symbol;Acc:HGNC:12004]	6
ENSG00000136810	5,907703	-1,72988	5,19E-07	9,93E-05	-7,761851	protein_coding	TXN	thioredoxin [Source:HGNC Symbol;Acc:HGNC:12435]	9
ENSG00000140945	5,560704	-4,469097	5,57E-07	0,000102	-7,721611	protein_coding	CDH13	cadherin 13 [Source:HGNC Symbol;Acc:HGNC:1753]	16
ENSG00000112031	4,396401	-1,606893	5,57E-07	0,000102	-7,721481	protein_coding	MTRF1L	mitochondrial translational release factor 1 like [Source:HGNC Symbol;Acc:HGNC:21051]	6
ENSG00000186660	6,146848	-1,133151	5,59E-07	0,000102	-7,719881	protein_coding	ZFP91	ZFP91 zinc finger protein, atypical E3 ubiquitin ligase [Source:HGNC Symbol;Acc:HGNC:14983]	11
ENSG00000147649	7,789286	-0,892181	5,56E-07	0,000102	-7,722367	protein_coding	MTDH	metadherin [Source:HGNC Symbol;Acc:HGNC:29608]	8
ENSG00000066322	4,936909	-1,235342	5,95E-07	0,000106	-7,684012	protein_coding	ELOVL1	ELOVL fatty acid elongase 1 [Source:HGNC Symbol;Acc:HGNC:14418]	1
ENSG00000139289	7,939989	-3,096603	5,92E-07	0,000106	-7,686595	protein_coding	PHLDA1	pleckstrin homology like domain family A member 1 [Source:HGNC Symbol;Acc:HGNC:8933]	12
ENSG00000197045	5,786797	-0,875818	6,14E-07	0,000108	-7,66579	protein_coding	GMFB	glia maturation factor beta [Source:HGNC Symbol;Acc:HGNC:4373]	14
ENSG00000054654	8,04205	2,30453	6,16E-07	0,000108	7,664228	protein_coding	SYNE2	spectrin repeat containing nuclear envelope protein 2 [Source:HGNC Symbol;Acc:HGNC:17084]	14
ENSG00000087263	5,070499	-1,101887	6,31E-07	0,000108	-7,650298	protein_coding	OGFOD1	2-oxoglutarate and iron dependent oxygenase domain containing 1 [Source:HGNC Symbol;Acc:HGNC:25585]	16
ENSG00000088827	1,735113	4,02254	6,31E-07	0,000108	7,650845	protein_coding	SIGLEC1	sialic acid binding Ig like lectin 1 [Source:HGNC Symbol;Acc:HGNC:11127]	20
ENSG00000131100	6,351847	-1,169008	6,62E-07	0,000112	-7,62311	protein_coding	ATP6V1E1	ATPase H+ transporting V1 subunit E1 [Source:HGNC Symbol;Acc:HGNC:857]	22
ENSG00000106080	4,865804	-1,842595	6,79E-07	0,000113	-7,608902	protein_coding	FKBP14	FKBP prolyl isomerase 14 [Source:HGNC Symbol;Acc:HGNC:18625]	7
ENSG00000103642	4,714029	-1,326532	6,85E-07	0,000113	-7,604292	protein_coding	LACTB	lactamase beta [Source:HGNC Symbol;Acc:HGNC:16468]	15
ENSG00000169826	6,331725	-2,190291	6,87E-07	0,000113	-7,60258	protein_coding	CSGALNAC	chondroitin sulfate N-acetylgalactosaminyltransferase 2 [Source:HGNC Symbol;Acc:HGNC:24292]	10
ENSG00000123094	5,295844	-2,693844	7,01E-07	0,000113	-7,590853	protein_coding	RASSF8	Ras association domain family member 8 [Source:HGNC Symbol;Acc:HGNC:13232]	12
ENSG00000113407	7,418496	-1,055183	6,98E-07	0,000113	-7,593677	protein_coding	TARS1	threonyl-tRNA synthetase 1 [Source:HGNC Symbol;Acc:HGNC:11572]	5

ENSG00000151650	-1,95223	4,31845	7,15E-07	0,000114	7,579562	protein_coding	VENTX	VENT homeobox [Source:HGNC Symbol;Acc:HGNC:13639]	10
ENSG00000124209	6,316343	-0,915981	7,44E-07	0,000117	-7,557066	protein_coding	RAB22A	RAB22A, member RAS oncogene family [Source:HGNC Symbol;Acc:HGNC:9764]	20
ENSG00000151388	6,569806	-3,744033	7,57E-07	0,000118	-7,547384	protein_coding	ADAMTS12	ADAM metallopeptidase with thrombospondin type 1 motif 12 [Source:HGNC Symbol;Acc:HGNC:14605]	5
ENSG00000271303	2,117927	-2,716929	7,8E-07	0,000121	-7,531004	protein_coding	SRXN1	sulfiredoxin 1 [Source:HGNC Symbol;Acc:HGNC:16132]	20
ENSG00000168040	4,646967	-1,398416	8,09E-07	0,000121	-7,510326	protein_coding	FADD	Fas associated via death domain [Source:HGNC Symbol;Acc:HGNC:3573]	11
ENSG00000152256	4,841552	1,745359	8,21E-07	0,000121	7,501751	protein_coding	PDK1	pyruvate dehydrogenase kinase 1 [Source:HGNC Symbol;Acc:HGNC:8809]	2
ENSG00000113361	6,353241	-3,762862	8,16E-07	0,000121	-7,505619	protein_coding	CDH6	cadherin 6 [Source:HGNC Symbol;Acc:HGNC:1765]	5
ENSG00000069956	6,538056	-1,177933	7,99E-07	0,000121	-7,517349	protein_coding	MAPK6	mitogen-activated protein kinase 6 [Source:HGNC Symbol;Acc:HGNC:6879]	15
ENSG00000166479	6,154775	-1,15103	8,17E-07	0,000121	-7,504848	protein_coding	TMX3	thioredoxin related transmembrane protein 3 [Source:HGNC Symbol;Acc:HGNC:24718]	18
ENSG00000155252	4,436291	-1,147223	8,77E-07	0,000127	-7,46457	protein_coding	PI4K2A	phosphatidylinositol 4-kinase type 2 alpha [Source:HGNC Symbol;Acc:HGNC:30031]	10
ENSG00000167280	4,623006	1,390303	8,99E-07	0,000127	7,450907	protein_coding	ENGASE	endo-beta-N-acetylglucosaminidase [Source:HGNC Symbol;Acc:HGNC:24622]	17
ENSG00000143106	5,662708	-1,375149	8,92E-07	0,000127	-7,455072	protein_coding	PSMA5	proteasome 20S subunit alpha 5 [Source:HGNC Symbol;Acc:HGNC:9534]	1
ENSG00000175592	3,133447	-5,371276	8,95E-07	0,000127	-7,453657	protein_coding	FOSL1	FOS like 1, AP-1 transcription factor subunit [Source:HGNC Symbol;Acc:HGNC:13718]	11
ENSG00000248483	1,467439	3,118285	8,87E-07	0,000127	7,458756	protein_coding	POU5F2	POU domain class 5, transcription factor 2 [Source:HGNC Symbol;Acc:HGNC:26367]	5
ENSG00000198792	6,953624	-1,108754	9,13E-07	0,000128	-7,442627	protein_coding	TMEM184B	transmembrane protein 184B [Source:HGNC Symbol;Acc:HGNC:1310]	22
ENSG00000134058	4,199016	-0,987955	9,23E-07	0,000128	-7,436028	protein_coding	CDK7	cyclin dependent kinase 7 [Source:HGNC Symbol;Acc:HGNC:1778]	5
ENSG00000168374	6,908107	-1,912949	9,32E-07	0,000128	-7,431044	protein_coding	ARF4	ADP ribosylation factor 4 [Source:HGNC Symbol;Acc:HGNC:655]	3
ENSG00000134668	3,015162	-2,866175	9,91E-07	0,000135	-7,396765	protein_coding	SPOCD1	SPOC domain containing 1 [Source:HGNC Symbol;Acc:HGNC:26338]	1
ENSG00000161057	6,368424	-1,00218	1,05E-06	0,000142	-7,36252	protein_coding	PSMC2	proteasome 26S subunit, ATPase 2 [Source:HGNC Symbol;Acc:HGNC:9548]	7
ENSG00000114999	6,577681	-1,279806	1,07E-06	0,000142	-7,355318	protein_coding	TTL	tubulin tyrosine ligase [Source:HGNC Symbol;Acc:HGNC:21586]	2
ENSG00000148680	2,049969	-4,688659	1,06E-06	0,000142	-7,357146	protein_coding	HTR7	5-hydroxytryptamine receptor 7 [Source:HGNC Symbol;Acc:HGNC:5302]	10
ENSG00000015479	3,982271	1,84472	1,23E-06	0,000162	7,278719	protein_coding	MATR3	matrin 3 [Source:HGNC Symbol;Acc:HGNC:6912]	5
ENSG00000132824	7,502006	-0,990789	1,26E-06	0,000165	-7,263486	protein_coding	SERINC3	serine incorporator 3 [Source:HGNC Symbol;Acc:HGNC:11699]	20
ENSG00000175348	4,540246	-1,045308	1,28E-06	0,000167	-7,252447	protein_coding	TMEM9B	TMEM9 domain family member B [Source:HGNC Symbol;Acc:HGNC:1168]	11
ENSG00000124783	7,819871	-1,044542	1,32E-06	0,00017	-7,237026	protein_coding	SSR1	signal sequence receptor subunit 1 [Source:HGNC Symbol;Acc:HGNC:11323]	6
ENSG00000071054	8,465439	-1,401726	1,34E-06	0,000171	-7,230972	protein_coding	MAP4K4	mitogen-activated protein kinase kinase kinase kinase 4 [Source:HGNC Symbol;Acc:HGNC:6866]	2
ENSG00000004779	5,091634	-1,140962	1,38E-06	0,000171	-7,213281	protein_coding	NDUFAB1	NADH:ubiquinone oxidoreductase subunit AB1 [Source:HGNC Symbol;Acc:HGNC:7694]	16
ENSG00000090020	5,564735	-1,538962	1,4E-06	0,000171	-7,204457	protein_coding	SLC9A1	solute carrier family 9 member A1 [Source:HGNC Symbol;Acc:HGNC:11071]	1
ENSG00000115170	5,684776	-1,401651	1,4E-06	0,000171	-7,204381	protein_coding	ACVR1	activin A receptor type 1 [Source:HGNC Symbol;Acc:HGNC:171]	2
ENSG00000163191	6,627011	-2,203011	1,4E-06	0,000171	-7,205325	protein_coding	S100A11	S100 calcium binding protein A11 [Source:HGNC Symbol;Acc:HGNC:10488]	1
ENSG00000161011	8,917739	-1,76694	1,43E-06	0,000171	-7,193082	protein_coding	SQSTM1	sequestosome 1 [Source:HGNC Symbol;Acc:HGNC:11280]	5
ENSG00000165678	6,626344	-1,237834	1,4E-06	0,000171	-7,20355	protein_coding	GHITM	growth hormone inducible transmembrane protein [Source:HGNC Symbol;Acc:HGNC:17281]	10
ENSG00000134007	-0,177412	3,801605	1,43E-06	0,000171	7,193416	protein_coding	ADAM20	ADAM metallopeptidase domain 20 [Source:HGNC Symbol;Acc:HGNC:199]	14
ENSG00000185640	-1,830861	-4,196842	1,43E-06	0,000171	-7,195118	protein_coding	KRT79	keratin 79 [Source:HGNC Symbol;Acc:HGNC:28930]	12
ENSG00000146285	-1,7391	4,633562	1,36E-06	0,000171	7,221769	protein_coding	SCML4	Scm polycomb group protein like 4 [Source:HGNC Symbol;Acc:HGNC:21397]	6
ENSG00000134108	5,925815	-0,960464	1,45E-06	0,000172	-7,185303	protein_coding	ARL8B	ADP ribosylation factor like GTPase 8B [Source:HGNC Symbol;Acc:HGNC:25564]	3
ENSG00000120708	8,645928	-3,008538	1,46E-06	0,000172	-7,181301	protein_coding	TGFBI	transforming growth factor beta induced [Source:HGNC Symbol;Acc:HGNC:11771]	5
ENSG00000170955	4,164916	-3,059246	1,54E-06	0,000173	-7,154644	protein_coding	CAVIN3	caveolae associated protein 3 [Source:HGNC Symbol;Acc:HGNC:9400]	11
ENSG00000111186	3,635518	-4,050803	1,54E-06	0,000173	-7,151742	protein_coding	WNT5B	Wnt family member 5B [Source:HGNC Symbol;Acc:HGNC:16265]	12
ENSG00000140157	5,17131	-1,224988	1,51E-06	0,000173	-7,164283	protein_coding	NIPA2	NIPA magnesium transporter 2 [Source:HGNC Symbol;Acc:HGNC:17044]	15
ENSG00000165891	5,338693	-3,146472	1,57E-06	0,000173	-7,140729	protein_coding	E2F7	E2F transcription factor 7 [Source:HGNC Symbol;Acc:HGNC:23820]	12
ENSG00000049130	4,923472	-1,902721	1,61E-06	0,000173	-7,130228	protein_coding	KITLG	KIT ligand [Source:HGNC Symbol;Acc:HGNC:6343]	12
ENSG00000124762	7,178058	-2,319914	1,5E-06	0,000173	-7,168736	protein_coding	CDKN1A	cyclin dependent kinase inhibitor 1A [Source:HGNC Symbol;Acc:HGNC:1784]	6
ENSG00000065154	5,857825	-1,531807	1,53E-06	0,000173	-7,157719	protein_coding	OAT	ornithine aminotransferase [Source:HGNC Symbol;Acc:HGNC:8091]	10
ENSG00000084073	5,876213	-1,25767	1,52E-06	0,000173	-7,158489	protein_coding	ZMPSTE24	zinc metallopeptidase STE24 [Source:HGNC Symbol;Acc:HGNC:12877]	1
ENSG00000186063	5,186251	-1,687491	1,6E-06	0,000173	-7,133282	protein_coding	AIDA	axin interactor, dorsalization associated [Source:HGNC Symbol;Acc:HGNC:25761]	1
ENSG00000164961	5,873519	-0,921888	1,54E-06	0,000173	-7,153306	protein_coding	WASHC5	WASH complex subunit 5 [Source:HGNC Symbol;Acc:HGNC:28984]	8
ENSG00000140941	6,116769	-1,51782	1,55E-06	0,000173	-7,148215	protein_coding	MAP1LC3B	microtubule associated protein 1 light chain 3 beta [Source:HGNC Symbol;Acc:HGNC:13352]	16
ENSG00000183291	5,888862	-1,279802	1,59E-06	0,000173	-7,136999	protein_coding	SELENOF	selenoprotein F [Source:HGNC Symbol;Acc:HGNC:17705]	1
ENSG00000104549	6,270637	-1,502908	1,6E-06	0,000173	-7,132325	protein_coding	SQLE	squalene epoxidase [Source:HGNC Symbol;Acc:HGNC:11279]	8
ENSG00000165169	5,783068	-2,048696	1,64E-06	0,000176	-7,117358	protein_coding	DYNLT3	dynein light chain Tctex-type 3 [Source:HGNC Symbol;Acc:HGNC:11694]	X

ENSG00000171421	3,578552	-1,402291	1,71E-06	0,000181	-7,096428	protein_coding	MRPL36	mitochondrial ribosomal protein L36 [Source:HGNC Symbol;Acc:HGNC:14490]	5
ENSG00000091317	6,494232	-1,145689	1,72E-06	0,000181	-7,093692	protein_coding	CMTM6	CKLF like MARVEL transmembrane domain containing 6 [Source:HGNC Symbol;Acc:HGNC:19177]	3
ENSG00000108010	5,044771	-0,99535	1,76E-06	0,000182	-7,080811	protein_coding	GLRX3	glutaredoxin 3 [Source:HGNC Symbol;Acc:HGNC:15987]	10
ENSG00000204291	7,268597	-2,791501	1,74E-06	0,000182	-7,085408	protein_coding	COL15A1	collagen type XV alpha 1 chain [Source:HGNC Symbol;Acc:HGNC:2192]	9
ENSG00000229117	7,349501	1,697446	1,75E-06	0,000182	7,083947	protein_coding	RPL41	ribosomal protein L41 [Source:HGNC Symbol;Acc:HGNC:10354]	12
ENSG00000132429	1,036634	-4,6911	1,77E-06	0,000182	-7,075808	protein_coding	POPDC3	popeye domain containing 3 [Source:HGNC Symbol;Acc:HGNC:17649]	6
ENSG00000134375	4,94732	-1,382175	1,8E-06	0,000183	-7,067136	protein_coding	TIMM17A	translocase of inner mitochondrial membrane 17A [Source:HGNC Symbol;Acc:HGNC:17315]	1
ENSG00000044115	8,372107	-1,203883	1,81E-06	0,000183	-7,064477	protein_coding	CTNNA1	catenin alpha 1 [Source:HGNC Symbol;Acc:HGNC:2509]	5
ENSG00000100243	7,542202	-1,303759	1,82E-06	0,000183	-7,060531	protein_coding	CYB5R3	cytochrome b5 reductase 3 [Source:HGNC Symbol;Acc:HGNC:2873]	22
ENSG00000103855	6,941459	-1,522264	1,83E-06	0,000183	-7,059481	protein_coding	CD276	CD276 molecule [Source:HGNC Symbol;Acc:HGNC:19137]	15
ENSG00000125148	6,624219	-3,798912	1,88E-06	0,000187	-7,045422	protein_coding	MT2A	metallothionein 2A [Source:HGNC Symbol;Acc:HGNC:7406]	16
ENSG00000082153	7,176528	-1,134743	1,96E-06	0,000194	-7,022383	protein_coding	BZW1	basic leucine zipper and W2 domains 1 [Source:HGNC Symbol;Acc:HGNC:18380]	2
ENSG00000166454	5,709816	-1,140765	2E-06	0,000195	-7,009801	protein_coding	ATMIN	ATM interactor [Source:HGNC Symbol;Acc:HGNC:29034]	16
ENSG00000197063	6,145913	-1,214829	1,99E-06	0,000195	-7,013465	protein_coding	MAFG	MAF bZIP transcription factor G [Source:HGNC Symbol;Acc:HGNC:6781]	17
ENSG00000171388	0,580063	4,386501	2E-06	0,000195	7,009951	protein_coding	APLN	apelin [Source:HGNC Symbol;Acc:HGNC:16665]	X
ENSG00000164251	4,527491	-3,74281	2,02E-06	0,000195	-7,006081	protein_coding	F2RL1	F2R like trypsin receptor 1 [Source:HGNC Symbol;Acc:HGNC:3538]	5
ENSG00000141458	6,151066	-1,219322	2,05E-06	0,000197	-6,998147	protein_coding	NPC1	NPC intracellular cholesterol transporter 1 [Source:HGNC Symbol;Acc:HGNC:7897]	18
ENSG00000101665	6,150258	-1,215958	2,06E-06	0,000197	-6,995147	protein_coding	SMAD7	SMAD family member 7 [Source:HGNC Symbol;Acc:HGNC:6773]	18
ENSG00000203852	-0,331182	3,772132	2,07E-06	0,000197	6,990853	protein_coding	H3C15	H3 clustered histone 15 [Source:HGNC Symbol;Acc:HGNC:20505]	1
ENSG00000136830	7,496179	-1,959292	2,1E-06	0,000198	-6,983949	protein_coding	NIBAN2	niban apoptosis regulator 2 [Source:HGNC Symbol;Acc:HGNC:25282]	9
ENSG00000174851	4,829672	-1,194516	2,11E-06	0,000199	-6,980588	protein_coding	YIF1A	Yip1 interacting factor homolog A, membrane trafficking protein [Source:HGNC Symbol;Acc:HGNC:16688]	11
ENSG00000125968	4,917052	-3,330632	2,19E-06	0,000204	-6,960553	protein_coding	ID1	inhibitor of DNA binding 1, HLH protein [Source:HGNC Symbol;Acc:HGNC:5360]	20
ENSG00000134970	5,960356	-1,424505	2,18E-06	0,000204	-6,963193	protein_coding	TMED7	transmembrane p24 trafficking protein 7 [Source:HGNC Symbol;Acc:HGNC:24253]	5
ENSG00000179933	4,330618	-1,188137	2,28E-06	0,000204	-6,939106	protein_coding	C14orf119	chromosome 14 open reading frame 119 [Source:HGNC Symbol;Acc:HGNC:20270]	14
ENSG00000169299	4,610526	-0,96965	2,26E-06	0,000204	-6,943489	protein_coding	PGM2	phosphoglucomutase 2 [Source:HGNC Symbol;Acc:HGNC:8906]	4
ENSG00000107789	5,075498	-1,12845	2,28E-06	0,000204	-6,939976	protein_coding	MINPP1	multiple inositol-polyphosphate phosphatase 1 [Source:HGNC Symbol;Acc:HGNC:7102]	10
ENSG00000109089	5,225365	-1,64563	2,28E-06	0,000204	-6,938729	protein_coding	CDR2L	cerebellar degeneration related protein 2 like [Source:HGNC Symbol;Acc:HGNC:29999]	17
ENSG00000186432	6,788106	-1,11891	2,24E-06	0,000204	-6,950046	protein_coding	KPNA4	karyopherin subunit alpha 4 [Source:HGNC Symbol;Acc:HGNC:6397]	3
ENSG00000061676	7,825709	-1,016287	2,25E-06	0,000204	-6,945764	protein_coding	NCKAP1	NCK associated protein 1 [Source:HGNC Symbol;Acc:HGNC:7666]	2
ENSG00000130706	6,458371	-1,000044	2,26E-06	0,000204	-6,943508	protein_coding	ADRM1	adhesion regulating molecule 1 [Source:HGNC Symbol;Acc:HGNC:15759]	20
ENSG00000116132	5,123079	-3,672513	2,31E-06	0,000205	-6,931989	protein_coding	PRRX1	paired related homeobox 1 [Source:HGNC Symbol;Acc:HGNC:9142]	1
ENSG00000115523	-1,649055	5,363834	2,32E-06	0,000205	6,931119	protein_coding	GNLY	granulysin [Source:HGNC Symbol;Acc:HGNC:4414]	2
ENSG00000166130	4,931698	-2,149942	2,48E-06	0,000217	-6,894767	protein_coding	IKBIP	IKBKB interacting protein [Source:HGNC Symbol;Acc:HGNC:26430]	12
ENSG00000140545	6,203328	-1,963268	2,48E-06	0,000217	-6,893765	protein_coding	MFGE8	milk fat globule EGF and factor V/VIII domain containing [Source:HGNC Symbol;Acc:HGNC:7036]	15
ENSG00000114251	5,721224	-3,459061	2,53E-06	0,00022	-6,883303	protein_coding	WNT5A	Wnt family member 5A [Source:HGNC Symbol;Acc:HGNC:12784]	3
ENSG00000157227	8,125155	-2,822669	2,56E-06	0,000221	-6,87798	protein_coding	MMP14	matrix metallopeptidase 14 [Source:HGNC Symbol;Acc:HGNC:7160]	14
ENSG00000129562	5,851881	-1,031732	2,59E-06	0,000223	-6,870918	protein_coding	DAD1	defender against cell death 1 [Source:HGNC Symbol;Acc:HGNC:2664]	14
ENSG00000168140	5,99466	-2,78196	2,65E-06	0,000226	-6,859256	protein_coding	VASN	vasorin [Source:HGNC Symbol;Acc:HGNC:18517]	16
ENSG00000181061	4,105429	-1,715068	2,71E-06	0,000229	-6,846799	protein_coding	HIGD1A	HIG1 hypoxia inducible domain family member 1A [Source:HGNC Symbol;Acc:HGNC:29527]	3
ENSG00000143753	5,662986	-1,460603	2,71E-06	0,000229	-6,846581	protein_coding	DEGS1	delta 4-desaturase, sphingolipid 1 [Source:HGNC Symbol;Acc:HGNC:13709]	1
ENSG00000159231	1,546921	-2,420821	2,78E-06	0,000234	-6,833678	protein_coding	CBR3	carbonyl reductase 3 [Source:HGNC Symbol;Acc:HGNC:1549]	21
ENSG00000007384	4,570988	-2,789361	2,81E-06	0,000235	-6,827899	protein_coding	RHBDF1	rhomboid 5 homolog 1 [Source:HGNC Symbol;Acc:HGNC:20561]	16
ENSG00000164332	4,539533	-1,308185	2,84E-06	0,000235	-6,821437	protein_coding	UBLCP1	ubiquitin like domain containing CTD phosphatase 1 [Source:HGNC Symbol;Acc:HGNC:28110]	5
ENSG00000182185	2,455338	1,961227	2,83E-06	0,000235	6,8228	protein_coding	RAD51B	RAD51 paralog B [Source:HGNC Symbol;Acc:HGNC:9822]	14
ENSG00000127364	1,047935	2,771467	2,85E-06	0,000235	6,819875	protein_coding	TAS2R4	taste 2 receptor member 4 [Source:HGNC Symbol;Acc:HGNC:14911]	7
ENSG00000127314	6,445707	-1,298081	2,87E-06	0,000235	-6,816267	protein_coding	RAP1B	RAP1B, member of RAS oncogene family [Source:HGNC Symbol;Acc:HGNC:9857]	12
ENSG00000136802	6,738303	-1,276382	2,93E-06	0,000239	-6,805497	protein_coding	LRRC8A	leucine rich repeat containing 8 VRAC subunit A [Source:HGNC Symbol;Acc:HGNC:19027]	9
ENSG00000173852	5,610066	-1,417585	2,95E-06	0,00024	-6,801873	protein_coding	DPY19L1	dpy-19 like C-mannosyltransferase 1 [Source:HGNC Symbol;Acc:HGNC:22205]	7
ENSG00000113721	8,498289	-2,007966	2,97E-06	0,00024	-6,798607	protein_coding	PDGFRB	platelet derived growth factor receptor beta [Source:HGNC Symbol;Acc:HGNC:8804]	5
ENSG00000105497	4,63787	-1,268371	3,09E-06	0,000248	-6,776652	protein_coding	ZNF175	zinc finger protein 175 [Source:HGNC Symbol;Acc:HGNC:12964]	19
ENSG00000077721	5,56618	-1,071138	3,1E-06	0,000248	-6,774469	protein_coding	UBE2A	ubiquitin conjugating enzyme E2 A [Source:HGNC Symbol;Acc:HGNC:12472]	X

ENSG00000181915	4,9435	-0,956405	3,16E-06	0,000251	-6,765444	protein_coding	ADO	2-aminoethanethiol dioxygenase [Source:HGNC Symbol;Acc:HGNC:23506]	10
ENSG00000143933	7,966441	-1,218152	3,2E-06	0,000254	-6,757536	protein_coding	CALM2	calmodulin 2 [Source:HGNC Symbol;Acc:HGNC:1445]	2
ENSG00000124074	3,485942	1,241315	3,25E-06	0,000255	6,749325	protein_coding	ENKD1	enkurin domain containing 1 [Source:HGNC Symbol;Acc:HGNC:25246]	16
ENSG00000230626	0,726262	1,921388	3,25E-06	0,000255	6,749346	protein_coding	AC011005.	novel protein similar to mitogen-activated protein kinase kinase 2 MAP2K2	7
ENSG00000214787	-1,666093	4,21705	3,27E-06	0,000256	6,746384	protein_coding	MS4A4E	membrane spanning 4-domains A4E [Source:HGNC Symbol;Acc:HGNC:14284]	11
ENSG00000135931	5,553002	-1,643135	3,36E-06	0,000261	-6,732571	protein_coding	ARMC9	armadillo repeat containing 9 [Source:HGNC Symbol;Acc:HGNC:20730]	2
ENSG00000166401	3,276784	-2,538664	3,4E-06	0,000263	-6,725882	protein_coding	SERPINB8	serpin family B member 8 [Source:HGNC Symbol;Acc:HGNC:8952]	18
ENSG00000179630	5,120587	-2,010841	3,43E-06	0,000263	-6,720807	protein_coding	LACC1	laccase domain containing 1 [Source:HGNC Symbol;Acc:HGNC:26789]	13
ENSG00000137055	5,496331	-0,908434	3,44E-06	0,000263	-6,719037	protein_coding	PLAA	phospholipase A2 activating protein [Source:HGNC Symbol;Acc:HGNC:9043]	9
ENSG00000122203	6,176193	-0,915584	3,44E-06	0,000263	-6,720055	protein_coding	KIAA1191	KIAA1191 [Source:HGNC Symbol;Acc:HGNC:29209]	5
ENSG00000011422	5,599648	-2,522079	3,48E-06	0,000264	-6,714269	protein_coding	PLAUR	plasminogen activator, urokinase receptor [Source:HGNC Symbol;Acc:HGNC:9053]	19
ENSG00000164294	4,971652	-3,232049	3,52E-06	0,000266	-6,707259	protein_coding	GPX8	glutathione peroxidase 8 (putative) [Source:HGNC Symbol;Acc:HGNC:33100]	5
ENSG00000156471	5,999614	-0,982676	3,56E-06	0,000268	-6,701479	protein_coding	PTDSS1	phosphatidylserine synthase 1 [Source:HGNC Symbol;Acc:HGNC:9587]	8
ENSG00000139324	5,834666	-1,339679	3,64E-06	0,000273	-6,689854	protein_coding	TMTC3	transmembrane O-mannosyltransferase targeting cadherins 3 [Source:HGNC Symbol;Acc:HGNC:26899]	12
ENSG00000067225	10,09992	-1,516906	3,73E-06	0,000278	-6,67745	protein_coding	PKM	pyruvate kinase M1/2 [Source:HGNC Symbol;Acc:HGNC:9021]	15
ENSG00000131127	4,879705	1,282249	3,77E-06	0,00028	6,671193	protein_coding	ZNF141	zinc finger protein 141 [Source:HGNC Symbol;Acc:HGNC:12926]	4
ENSG00000151465	5,387756	-1,094657	3,79E-06	0,00028	-6,668353	protein_coding	CDC123	cell division cycle 123 [Source:HGNC Symbol;Acc:HGNC:16827]	10
ENSG00000185475	4,155388	-1,000158	3,89E-06	0,000283	-6,654869	protein_coding	TMEM179I	transmembrane protein 179B [Source:HGNC Symbol;Acc:HGNC:33744]	11
ENSG00000165609	4,911617	-1,060123	3,89E-06	0,000283	-6,654827	protein_coding	NUDT5	nudix hydrolase 5 [Source:HGNC Symbol;Acc:HGNC:8052]	10
ENSG00000188529	5,091041	-0,97182	3,87E-06	0,000283	-6,657709	protein_coding	SRSF10	serine and arginine rich splicing factor 10 [Source:HGNC Symbol;Acc:HGNC:16713]	1
ENSG00000115520	5,122301	-1,383963	3,93E-06	0,000285	-6,648755	protein_coding	COQ10B	coenzyme Q10B [Source:HGNC Symbol;Acc:HGNC:25819]	2
ENSG00000148110	5,703179	-1,029734	3,94E-06	0,000285	-6,647439	protein_coding	MFSD14B	major facilitator superfamily domain containing 14B [Source:HGNC Symbol;Acc:HGNC:23376]	9
ENSG00000097021	5,132044	-1,798109	4,1E-06	0,000295	-6,626391	protein_coding	ACOT7	acyl-CoA thioesterase 7 [Source:HGNC Symbol;Acc:HGNC:24157]	1
ENSG00000240849	5,259636	-1,126354	4,14E-06	0,000295	-6,622053	protein_coding	PEDS1	plasmanylethanolamine desaturase 1 [Source:HGNC Symbol;Acc:HGNC:16735]	20
ENSG00000239704	-0,381348	3,942565	4,12E-06	0,000295	6,623808	protein_coding	CDRT4	CMT1A duplicated region transcript 4 [Source:HGNC Symbol;Acc:HGNC:14383]	17
ENSG00000188394	-0,515761	3,120832	4,16E-06	0,000295	6,619057	protein_coding	GPR21	G protein-coupled receptor 21 [Source:HGNC Symbol;Acc:HGNC:4476]	9
ENSG00000145817	5,981999	-1,724138	4,24E-06	0,0003	-6,609004	protein_coding	YIPF5	Yip1 domain family member 5 [Source:HGNC Symbol;Acc:HGNC:24877]	5
ENSG00000147592	4,126622	-1,290306	4,26E-06	0,0003	-6,606553	protein_coding	LACTB2	lactamase beta 2 [Source:HGNC Symbol;Acc:HGNC:18512]	8
ENSG00000276023	3,766179	-1,576358	4,35E-06	0,000301	-6,595441	protein_coding	DUSP14	dual specificity phosphatase 14 [Source:HGNC Symbol;Acc:HGNC:17007]	17
ENSG00000135723	4,994763	-1,390503	4,37E-06	0,000301	-6,593274	protein_coding	FHOD1	formin homology 2 domain containing 1 [Source:HGNC Symbol;Acc:HGNC:17905]	16
ENSG00000146414	5,922906	1,218518	4,41E-06	0,000301	6,589074	protein_coding	SHPRH	SNF2 histone linker PHD RING helicase [Source:HGNC Symbol;Acc:HGNC:19336]	6
ENSG00000080573	6,052567	-2,12858	4,41E-06	0,000301	-6,588368	protein_coding	COL5A3	collagen type V alpha 3 chain [Source:HGNC Symbol;Acc:HGNC:14864]	19
ENSG00000145740	5,889162	-0,877635	4,41E-06	0,000301	-6,588974	protein_coding	SLC30A5	solute carrier family 30 member 5 [Source:HGNC Symbol;Acc:HGNC:19089]	5
ENSG00000181104	6,988791	-2,979824	4,41E-06	0,000301	-6,58864	protein_coding	F2R	coagulation factor II thrombin receptor [Source:HGNC Symbol;Acc:HGNC:3537]	5
ENSG00000165280	8,243919	-0,920828	4,37E-06	0,000301	-6,593071	protein_coding	VCP	valosin containing protein [Source:HGNC Symbol;Acc:HGNC:12666]	9
ENSG00000061938	5,232478	1,251637	4,45E-06	0,000303	6,583667	protein_coding	TNK2	tyrosine kinase non receptor 2 [Source:HGNC Symbol;Acc:HGNC:19297]	3
ENSG00000174695	6,645577	-1,076045	4,52E-06	0,000306	-6,576145	protein_coding	TMEM167/	transmembrane protein 167A [Source:HGNC Symbol;Acc:HGNC:28330]	5
ENSG00000077157	6,057926	2,306111	4,55E-06	0,000306	6,57175	protein_coding	PPP1R12B	protein phosphatase 1 regulatory subunit 12B [Source:HGNC Symbol;Acc:HGNC:7619]	1
ENSG00000094631	5,740057	1,198207	4,58E-06	0,000306	6,569066	protein_coding	HDAC6	histone deacetylase 6 [Source:HGNC Symbol;Acc:HGNC:14064]	X
ENSG00000075785	8,030123	-1,062797	4,59E-06	0,000306	-6,567077	protein_coding	RAB7A	RAB7A, member RAS oncogene family [Source:HGNC Symbol;Acc:HGNC:9788]	3
ENSG00000198844	-0,110978	5,466204	4,59E-06	0,000306	6,56795	protein_coding	ARHGEF15	Rho guanine nucleotide exchange factor 15 [Source:HGNC Symbol;Acc:HGNC:15590]	17
ENSG00000135426	-1,940173	4,647008	4,63E-06	0,000307	6,562679	protein_coding	TESPA1	thymocyte expressed, positive selection associated 1 [Source:HGNC Symbol;Acc:HGNC:29109]	12
ENSG00000153989	5,218475	-1,130072	4,73E-06	0,000311	-6,551914	protein_coding	NUS1	NUS1 dehydrodolichyl diphosphate synthase subunit [Source:HGNC Symbol;Acc:HGNC:21042]	6
ENSG00000151835	6,561251	-1,584573	4,74E-06	0,000311	-6,550141	protein_coding	SACS	sacsin molecular chaperone [Source:HGNC Symbol;Acc:HGNC:10519]	13
ENSG00000131236	7,851718	-1,65629	4,75E-06	0,000311	-6,549771	protein_coding	CAP1	cyclase associated actin cytoskeleton regulatory protein 1 [Source:HGNC Symbol;Acc:HGNC:20040]	1
ENSG00000155380	4,932551	-1,455853	4,87E-06	0,000316	-6,536767	protein_coding	SLC16A1	solute carrier family 16 member 1 [Source:HGNC Symbol;Acc:HGNC:10922]	1
ENSG00000138600	6,060131	-1,12821	4,86E-06	0,000316	-6,537566	protein_coding	SPPL2A	signal peptide peptidase like 2A [Source:HGNC Symbol;Acc:HGNC:30227]	15
ENSG00000180398	6,992952	-1,493732	4,91E-06	0,000318	-6,532257	protein_coding	MCFD2	multiple coagulation factor deficiency 2, ER cargo receptor complex subunit [Source:HGNC Symbol;Acc:HGNC:18451	2
ENSG00000101928	3,402948	-1,572327	5E-06	0,000318	-6,523208	protein_coding	MOSPD1	motile sperm domain containing 1 [Source:HGNC Symbol;Acc:HGNC:25235]	X
ENSG00000163659	6,218398	-2,168275	4,97E-06	0,000318	-6,525972	protein_coding	TIPARP	TCDD inducible poly(ADP-ribose) polymerase [Source:HGNC Symbol;Acc:HGNC:23696]	3
ENSG00000100664	7,90515	-0,852352	5,01E-06	0,000318	-6,521915	protein_coding	EIF5	eukaryotic translation initiation factor 5 [Source:HGNC Symbol;Acc:HGNC:3299]	14

ENSG00000197965	6,962345	-1,687394	5E-06	0,000318	-6,522219	protein_coding	MPZL1	myelin protein zero like 1 [Source:HGNC Symbol;Acc:HGNC:7226]	1
ENSG00000168309	-1,685221	5,511774	4,93E-06	0,000318	6,529966	protein_coding	FAM107A	family with sequence similarity 107 member A [Source:HGNC Symbol;Acc:HGNC:30827]	3
ENSG00000121068	6,174805	-2,727492	5,11E-06	0,000321	-6,510984	protein_coding	TBX2	T-box transcription factor 2 [Source:HGNC Symbol;Acc:HGNC:11597]	17
ENSG00000182718	7,963937	-2,790726	5,1E-06	0,000321	-6,512213	protein_coding	ANXA2	annexin A2 [Source:HGNC Symbol;Acc:HGNC:537]	15
ENSG00000138107	6,509059	-0,839175	5,08E-06	0,000321	-6,514297	protein_coding	ACTR1A	actin related protein 1A [Source:HGNC Symbol;Acc:HGNC:167]	10
ENSG00000186010	1,800225	1,884569	5,17E-06	0,000322	6,505513	protein_coding	NDUFA13	NADH:ubiquinone oxidoreductase subunit A13 [Source:HGNC Symbol;Acc:HGNC:17194]	19
ENSG00000134308	7,511594	-1,099761	5,15E-06	0,000322	-6,506829	protein_coding	YWHAQ	tyrosine 3-monooxygenase/tryptophan 5-monooxygenase activation protein theta [Source:HGNC Symbol;Acc:HGN(	2
ENSG00000142627	4,634605	-3,285526	5,27E-06	0,000327	-6,495569	protein_coding	EPHA2	EPH receptor A2 [Source:HGNC Symbol;Acc:HGNC:3386]	1
ENSG00000137070	2,769158	1,591193	5,37E-06	0,000332	6,485127	protein_coding	IL11RA	interleukin 11 receptor subunit alpha [Source:HGNC Symbol;Acc:HGNC:5967]	9
ENSG00000121671	5,499589	1,510112	5,4E-06	0,000333	6,482205	protein_coding	CRY2	cryptochrome circadian regulator 2 [Source:HGNC Symbol;Acc:HGNC:2385]	11
ENSG00000115514	4,476283	-1,39085	5,49E-06	0,000333	-6,474036	protein_coding	TXNDC9	thioredoxin domain containing 9 [Source:HGNC Symbol;Acc:HGNC:24110]	2
ENSG00000124541	5,616611	-0,89478	5,43E-06	0,000333	-6,479347	protein_coding	RRP36	ribosomal RNA processing 36 [Source:HGNC Symbol;Acc:HGNC:21374]	6
ENSG00000182534	6,324782	-2,053649	5,5E-06	0,000333	-6,472495	protein_coding	MXRA7	matrix remodeling associated 7 [Source:HGNC Symbol;Acc:HGNC:7541]	17
ENSG00000198363	8,100026	-1,834344	5,55E-06	0,000333	-6,468223	protein_coding	ASPH	aspartate beta-hydroxylase [Source:HGNC Symbol;Acc:HGNC:757]	8
ENSG00000132341	7,340948	-0,932152	5,49E-06	0,000333	-6,474331	protein_coding	RAN	RAN, member RAS oncogene family [Source:HGNC Symbol;Acc:HGNC:9846]	12
ENSG00000212128	-0,356616	3,335884	5,55E-06	0,000333	6,468391	protein_coding	TAS2R13	taste 2 receptor member 13 [Source:HGNC Symbol;Acc:HGNC:14919]	12
ENSG00000277494	-1,376755	4,986346	5,51E-06	0,000333	6,471752	protein_coding	GPIHBP1	glycosylphosphatidylinositol anchored high density lipoprotein binding protein 1 [Source:HGNC Symbol;Acc:HGNC:2	8
ENSG00000176407	5,892715	-1,145289	5,57E-06	0,000333	-6,466095	protein_coding	KCMF1	potassium channel modulatory factor 1 [Source:HGNC Symbol;Acc:HGNC:20589]	2
ENSG00000013441	6,261024	1,244914	5,62E-06	0,000333	6,461769	protein_coding	CLK1	CDC like kinase 1 [Source:HGNC Symbol;Acc:HGNC:2068]	2
ENSG00000116237	6,305376	-1,151485	5,6E-06	0,000333	-6,463313	protein_coding	ICMT	isoprenylcysteine carboxyl methyltransferase [Source:HGNC Symbol;Acc:HGNC:5350]	1
ENSG00000215251	4,526896	-0,868798	5,73E-06	0,000337	-6,451831	protein_coding	FASTKD5	FAST kinase domains 5 [Source:HGNC Symbol;Acc:HGNC:25790]	20
ENSG00000174903	6,587308	-0,813718	5,73E-06	0,000337	-6,451841	protein_coding	RAB1B	RAB1B, member RAS oncogene family [Source:HGNC Symbol;Acc:HGNC:18370]	11
ENSG00000147099	3,978419	1,65191	5,76E-06	0,000338	6,44893	protein_coding	HDAC8	histone deacetylase 8 [Source:HGNC Symbol;Acc:HGNC:13315]	X
ENSG00000173692	7,029513	-0,943572	6,06E-06	0,000354	-6,422475	protein_coding	PSMD1	proteasome 26S subunit, non-ATPase 1 [Source:HGNC Symbol;Acc:HGNC:9554]	2
ENSG00000131871	5,773969	-1,199649	6,13E-06	0,000355	-6,416492	protein_coding	SELENOS	selenoprotein S [Source:HGNC Symbol;Acc:HGNC:30396]	15
ENSG00000011028	7,611999	-3,128081	6,14E-06	0,000355	-6,415484	protein_coding	MRC2	mannose receptor C type 2 [Source:HGNC Symbol;Acc:HGNC:16875]	17
ENSG00000188897	0,438435	2,318631	6,14E-06	0,000355	6,415685	protein_coding	AC099489.	novel lipoprotein amino terminal region containing protein	16
ENSG00000088826	3,891891	-1,898303	6,18E-06	0,000356	-6,412398	protein_coding	SMOX	spermine oxidase [Source:HGNC Symbol;Acc:HGNC:15862]	20
ENSG00000111711	5,274116	-1,373335	6,22E-06	0,000357	-6,408655	protein_coding	GOLT1B	golgi transport 1B [Source:HGNC Symbol;Acc:HGNC:20175]	12
ENSG00000166226	6,677403	-0,661152	6,32E-06	0,000362	-6,400378	protein_coding	CCT2	chaperonin containing TCP1 subunit 2 [Source:HGNC Symbol;Acc:HGNC:1615]	12
ENSG00000142552	6,151057	-2,706541	6,37E-06	0,000363	-6,39691	protein_coding	RCN3	reticulocalbin 3 [Source:HGNC Symbol;Acc:HGNC:21145]	19
ENSG00000156515	7,247171	-1,45804	6,47E-06	0,000368	-6,388599	protein_coding	HK1	hexokinase 1 [Source:HGNC Symbol;Acc:HGNC:4922]	10
ENSG00000126524	6,288792	-1,162087	6,5E-06	0,000368	-6,386211	protein_coding	SBDS	SBDS ribosome maturation factor [Source:HGNC Symbol;Acc:HGNC:19440]	7
ENSG00000221995	2,03557	2,205463	6,58E-06	0,00037	6,379868	protein_coding	TIAF1	TGFB1-induced anti-apoptotic factor 1 [Source:HGNC Symbol;Acc:HGNC:11803]	17
ENSG00000048544	4,726423	-0,974771	6,57E-06	0,00037	-6,380564	protein_coding	MRPS10	mitochondrial ribosomal protein S10 [Source:HGNC Symbol;Acc:HGNC:14502]	6
ENSG00000089775	4,54012	0,926141	6,61E-06	0,000371	6,377273	protein_coding	ZBTB25	zinc finger and BTB domain containing 25 [Source:HGNC Symbol;Acc:HGNC:13112]	14
ENSG00000114841	4,95275	1,746537	6,65E-06	0,000371	6,374628	protein_coding	DNAH1	dynein axonemal heavy chain 1 [Source:HGNC Symbol;Acc:HGNC:2940]	3
ENSG00000104974	-2,239272	4,188502	6,66E-06	0,000371	6,373649	protein_coding	LILRA1	leukocyte immunoglobulin like receptor A1 [Source:HGNC Symbol;Acc:HGNC:6602]	19
ENSG00000107175	5,354101	-1,021656	6,69E-06	0,000371	-6,371278	protein_coding	CREB3	cAMP responsive element binding protein 3 [Source:HGNC Symbol;Acc:HGNC:2347]	9
ENSG00000004897	6,509836	-0,873168	6,72E-06	0,000371	-6,369071	protein_coding	CDC27	cell division cycle 27 [Source:HGNC Symbol;Acc:HGNC:1728]	17
ENSG00000079332	6,24464	-1,070191	6,75E-06	0,000372	-6,366481	protein_coding	SAR1A	secretion associated Ras related GTPase 1A [Source:HGNC Symbol;Acc:HGNC:10534]	10
ENSG00000104814	0,253368	3,914858	6,78E-06	0,000372	6,364029	protein_coding	MAP4K1	mitogen-activated protein kinase kinase kinase kinase 1 [Source:HGNC Symbol;Acc:HGNC:6863]	19
ENSG00000165637	6,224992	-0,892155	6,81E-06	0,000373	-6,36196	protein_coding	VDAC2	voltage dependent anion channel 2 [Source:HGNC Symbol;Acc:HGNC:12672]	10
ENSG00000128805	4,311159	-2,418935	6,96E-06	0,000373	-6,350569	protein_coding	ARHGAP22	Rho GTPase activating protein 22 [Source:HGNC Symbol;Acc:HGNC:30320]	10
ENSG00000143369	4,906774	-2,775895	6,94E-06	0,000373	-6,352232	protein_coding	ECM1	extracellular matrix protein 1 [Source:HGNC Symbol;Acc:HGNC:3153]	1
ENSG00000171314	5,578103	-1,313606	6,94E-06	0,000373	-6,352281	protein_coding	PGAM1	phosphoglycerate mutase 1 [Source:HGNC Symbol;Acc:HGNC:8888]	10
ENSG00000104859	6,03827	1,15947	6,96E-06	0,000373	6,35041	protein_coding	CLASRP	CLK4 associating serine/arginine rich protein [Source:HGNC Symbol;Acc:HGNC:17731]	19
ENSG00000276231	-0,095448	3,988082	7E-06	0,000373	6,347829	protein_coding	PIK3R6	phosphoinositide-3-kinase regulatory subunit 6 [Source:HGNC Symbol;Acc:HGNC:27101]	17
ENSG00000130307	-1,022078	3,819007	6,94E-06	0,000373	6,352033	protein_coding	USHBP1	USH1 protein network component harmonin binding protein 1 [Source:HGNC Symbol;Acc:HGNC:24058]	19
ENSG00000198851	-1,2327	4,456849	6,98E-06	0,000373	6,349518	protein_coding	CD3E	CD3e molecule [Source:HGNC Symbol;Acc:HGNC:1674]	11
ENSG00000142178	-1,719009	3,992478	6,94E-06	0,000373	6,351978	protein_coding	SIK1	salt inducible kinase 1 [Source:HGNC Symbol;Acc:HGNC:11142]	21

ENSG00000115414	12,99756	-2,925598	7,12E-06	0,000376	-6,339132	protein_coding	FN1	fibronectin 1 [Source:HGNC Symbol;Acc:HGNC:3778]	2
ENSG00000175309	5,003502	1,582993	7,11E-06	0,000376	6,340074	protein_coding	PHYKPL	5-phosphohydroxy-L-lysine phospho-lyase [Source:HGNC Symbol;Acc:HGNC:28249]	5
ENSG00000133246	0,293859	2,932547	7,11E-06	0,000376	6,339803	protein_coding	PRAM1	PML-RARA regulated adaptor molecule 1 [Source:HGNC Symbol;Acc:HGNC:30091]	19
ENSG00000144635	5,552782	-0,985584	7,18E-06	0,000377	-6,334607	protein_coding	DYNC1LI1	dynein cytoplasmic 1 light intermediate chain 1 [Source:HGNC Symbol;Acc:HGNC:18745]	3
ENSG00000173546	6,015499	-1,702197	7,18E-06	0,000377	-6,334399	protein_coding	CSPG4	chondroitin sulfate proteoglycan 4 [Source:HGNC Symbol;Acc:HGNC:2466]	15
ENSG00000139631	3,879013	1,432107	7,37E-06	0,000384	6,32108	protein_coding	CSAD	cysteine sulfinic acid decarboxylase [Source:HGNC Symbol;Acc:HGNC:18966]	12
ENSG00000213949	8,010374	-1,592869	7,38E-06	0,000384	-6,320452	protein_coding	ITGA1	integrin subunit alpha 1 [Source:HGNC Symbol;Acc:HGNC:6134]	5
ENSG00000145241	5,143112	1,110872	7,45E-06	0,000385	6,315938	protein_coding	CENPC	centromere protein C [Source:HGNC Symbol;Acc:HGNC:1854]	4
ENSG00000129083	7,087192	-0,872465	7,43E-06	0,000385	-6,316818	protein_coding	COPB1	COPI coat complex subunit beta 1 [Source:HGNC Symbol;Acc:HGNC:2231]	11
ENSG00000140307	4,518745	-1,312701	7,55E-06	0,000389	-6,308957	protein_coding	GTF2A2	general transcription factor IIA subunit 2 [Source:HGNC Symbol;Acc:HGNC:4647]	15
ENSG00000139223	-2,512452	3,020911	7,59E-06	0,00039	6,305839	protein_coding	ANP32D	acidic nuclear phosphoprotein 32 family member D [Source:HGNC Symbol;Acc:HGNC:16676]	12
ENSG00000126903	4,251748	-1,355763	7,7E-06	0,000392	-6,298419	protein_coding	SLC10A3	solute carrier family 10 member 3 [Source:HGNC Symbol;Acc:HGNC:22979]	X
ENSG00000151116	4,528904	-1,007036	7,71E-06	0,000392	-6,297705	protein_coding	UEVLD	UEV and lactate/malate dehyrogenase domains [Source:HGNC Symbol;Acc:HGNC:30866]	11
ENSG00000177989	1,338447	2,874362	7,7E-06	0,000392	6,298524	protein_coding	ODF3B	outer dense fiber of sperm tails 3B [Source:HGNC Symbol;Acc:HGNC:34388]	22
ENSG00000075290	-3,021305	2,775846	7,73E-06	0,000392	6,296898	protein_coding	WNT8B	Wnt family member 8B [Source:HGNC Symbol;Acc:HGNC:12789]	10
ENSG00000175166	7,839329	-1,121789	7,75E-06	0,000392	-6,295208	protein_coding	PSMD2	proteasome 26S subunit, non-ATPase 2 [Source:HGNC Symbol;Acc:HGNC:9559]	3
ENSG00000174945	1,997478	-2,93405	7,79E-06	0,000393	-6,292573	protein_coding	AMZ1	archaelysin family metallopeptidase 1 [Source:HGNC Symbol;Acc:HGNC:22231]	7
ENSG00000188483	5,157558	-1,46757	7,98E-06	0,000399	-6,280173	protein_coding	IER5L	immediate early response 5 like [Source:HGNC Symbol;Acc:HGNC:23679]	9
ENSG00000101361	8,126484	1,718534	7,98E-06	0,000399	6,279953	protein_coding	NOP56	NOP56 ribonucleoprotein [Source:HGNC Symbol;Acc:HGNC:15911]	20
ENSG00000100941	7,676414	1,121537	7,95E-06	0,000399	6,282	protein_coding	PNN	pinin, desmosome associated protein [Source:HGNC Symbol;Acc:HGNC:9162]	14
ENSG00000182544	3,592538	-1,179259	8,15E-06	0,000405	-6,269446	protein_coding	MFSD5	major facilitator superfamily domain containing 5 [Source:HGNC Symbol;Acc:HGNC:28156]	12
ENSG00000118680	7,723763	-1,407298	8,15E-06	0,000405	-6,269271	protein_coding	MYL12B	myosin light chain 12B [Source:HGNC Symbol;Acc:HGNC:29827]	18
ENSG00000019549	4,805197	-2,990432	8,18E-06	0,000405	-6,267524	protein_coding	SNAI2	snail family transcriptional repressor 2 [Source:HGNC Symbol;Acc:HGNC:11094]	8
ENSG00000081087	5,165982	-1,430581	8,43E-06	0,000411	-6,252029	protein_coding	OSTM1	osteoclastogenesis associated transmembrane protein 1 [Source:HGNC Symbol;Acc:HGNC:21652]	6
ENSG00000120992	5,381562	-1,106939	8,38E-06	0,000411	-6,25519	protein_coding	LYPLA1	lysophospholipase 1 [Source:HGNC Symbol;Acc:HGNC:6737]	8
ENSG00000176155	5,70703	1,235575	8,34E-06	0,000411	6,257727	protein_coding	CCDC57	coiled-coil domain containing 57 [Source:HGNC Symbol;Acc:HGNC:27564]	17
ENSG00000048392	5,57426	-1,244148	8,42E-06	0,000411	-6,252455	protein_coding	RRM2B	ribonucleotide reductase regulatory TP53 inducible subunit M2B [Source:HGNC Symbol;Acc:HGNC:17296]	8
ENSG00000177156	6,656077	-1,01967	8,39E-06	0,000411	-6,254195	protein_coding	TALDO1	transaldolase 1 [Source:HGNC Symbol;Acc:HGNC:11559]	11
ENSG00000127870	6,436187	-0,92572	8,47E-06	0,000412	-6,249881	protein_coding	RNF6	ring finger protein 6 [Source:HGNC Symbol;Acc:HGNC:10069]	13
ENSG00000166200	6,005861	-0,921619	8,51E-06	0,000413	-6,247115	protein_coding	COPS2	COP9 signalosome subunit 2 [Source:HGNC Symbol;Acc:HGNC:30747]	15
ENSG00000114125	4,960048	-1,348235	8,58E-06	0,000413	-6,242822	protein_coding	RNF7	ring finger protein 7 [Source:HGNC Symbol;Acc:HGNC:10070]	3
ENSG00000074181	7,960943	-1,361424	8,6E-06	0,000413	-6,242079	protein_coding	NOTCH3	notch receptor 3 [Source:HGNC Symbol;Acc:HGNC:7883]	19
ENSG00000130340	7,08758	-1,386376	8,59E-06	0,000413	-6,242413	protein_coding	SNX9	sorting nexin 9 [Source:HGNC Symbol;Acc:HGNC:14973]	6
ENSG00000015568	0,323429	2,132334	8,63E-06	0,000414	6,240093	protein_coding	RGPD5	RANBP2 like and GRIP domain containing 5 [Source:HGNC Symbol;Acc:HGNC:32418]	2
ENSG00000160691	6,604206	-1,906979	8,73E-06	0,000416	-6,233921	protein_coding	SHC1	SHC adaptor protein 1 [Source:HGNC Symbol;Acc:HGNC:10840]	1
ENSG00000163606	-1,751904	3,367178	8,72E-06	0,000416	6,234454	protein_coding	CD200R1	CD200 receptor 1 [Source:HGNC Symbol;Acc:HGNC:24235]	3
ENSG00000174173	4,202767	-1,262426	8,77E-06	0,000417	-6,231943	protein_coding	TRMT10C	tRNA methyltransferase 10C, mitochondrial RNase P subunit [Source:HGNC Symbol;Acc:HGNC:26022]	3
ENSG00000155428	-1,907908	3,024646	8,85E-06	0,000419	6,227016	protein_coding	TRIM74	tripartite motif containing 74 [Source:HGNC Symbol;Acc:HGNC:17453]	7
ENSG00000135018	7,174777	-0,776473	8,89E-06	0,00042	-6,224831	protein_coding	UBQLN1	ubiquilin 1 [Source:HGNC Symbol;Acc:HGNC:12508]	9
ENSG00000203872	0,972753	2,191424	8,92E-06	0,00042	6,222932	protein_coding	C6orf163	chromosome 6 open reading frame 163 [Source:HGNC Symbol;Acc:HGNC:21403]	6
ENSG00000100558	3,518514	-2,374294	9,01E-06	0,000423	-6,217932	protein_coding	PLEK2	pleckstrin 2 [Source:HGNC Symbol;Acc:HGNC:19238]	14
ENSG00000214021	4,167358	1,397095	9,1E-06	0,000425	6,213134	protein_coding	TTLL3	tubulin tyrosine ligase like 3 [Source:HGNC Symbol;Acc:HGNC:24483]	3
ENSG00000198821	-0,951933	3,689927	9,08E-06	0,000425	6,213899	protein_coding	CD247	CD247 molecule [Source:HGNC Symbol;Acc:HGNC:1677]	1
ENSG00000188725	5,339924	-0,981382	9,2E-06	0,000428	-6,207353	protein_coding	SMIM15	small integral membrane protein 15 [Source:HGNC Symbol;Acc:HGNC:33861]	5
ENSG00000165457	-0,696442	5,920309	9,44E-06	0,000438	6,194128	protein_coding	FOLR2	folate receptor beta [Source:HGNC Symbol;Acc:HGNC:3793]	11
ENSG00000118579	5,783162	0,806427	9,52E-06	0,000441	6,189531	protein_coding	MED28	mediator complex subunit 28 [Source:HGNC Symbol;Acc:HGNC:24628]	4
ENSG00000221823	5,649237	-0,959233	9,56E-06	0,000441	-6,18749	protein_coding	PPP3R1	protein phosphatase 3 regulatory subunit B, alpha [Source:HGNC Symbol;Acc:HGNC:9317]	2
ENSG00000072401	3,822226	-1,221328	9,64E-06	0,000442	-6,183429	protein_coding	UBE2D1	ubiquitin conjugating enzyme E2 D1 [Source:HGNC Symbol;Acc:HGNC:12474]	10
ENSG00000237541	-1,834058	4,453781	9,62E-06	0,000442	6,184442	protein_coding	HLA-DQA2	major histocompatibility complex, class II, DQ alpha 2 [Source:HGNC Symbol;Acc:HGNC:4943]	6
ENSG00000206530	3,684273	1,487392	9,78E-06	0,000447	6,17594	protein_coding	CFAP44	cilia and flagella associated protein 44 [Source:HGNC Symbol;Acc:HGNC:25631]	3
ENSG00000203737	-0,841851	3,480537	9,81E-06	0,000447	6,174504	protein_coding	GPR52	G protein-coupled receptor 52 [Source:HGNC Symbol;Acc:HGNC:4508]	1

ENSG00000100191	-1,408018	3,848582	9,83E-06	0,000447	6,173368	protein_coding	SLC5A4	solute carrier family 5 member 4 [Source:HGNC Symbol;Acc:HGNC:11039]	22
ENSG00000166734	7,431815	-0,943613	9,96E-06	0,000451	-6,166963	protein_coding	GOLM2	golgi membrane protein 2 [Source:HGNC Symbol;Acc:HGNC:24892]	15
ENSG00000177688	-0,07873	2,590336	9,94E-06	0,000451	6,167566	protein_coding	SUMO4	small ubiquitin like modifier 4 [Source:HGNC Symbol;Acc:HGNC:21181]	6
ENSG00000177728	6,243595	0,907376	9,99E-06	0,000451	6,165168	protein_coding	TMEM94	transmembrane protein 94 [Source:HGNC Symbol;Acc:HGNC:28983]	17
ENSG00000162694	4,187729	-1,545391	1,01E-05	0,000455	-6,157471	protein_coding	EXTL2	exostosin like glycosyltransferase 2 [Source:HGNC Symbol;Acc:HGNC:3516]	1
ENSG00000100567	5,800136	-0,877858	1,01E-05	0,000455	-6,158596	protein_coding	PSMA3	proteasome 20S subunit alpha 3 [Source:HGNC Symbol;Acc:HGNC:9532]	14
ENSG00000108799	5,330955	1,01704	1,03E-05	0,00046	6,151268	protein_coding	EZH1	enhancer of zeste 1 polycomb repressive complex 2 subunit [Source:HGNC Symbol;Acc:HGNC:3526]	17
ENSG00000155876	5,750861	-1,02535	1,05E-05	0,000468	-6,140764	protein_coding	RRAGA	Ras related GTP binding A [Source:HGNC Symbol;Acc:HGNC:16963]	9
ENSG00000232593	3,682851	1,736305	1,07E-05	0,000469	6,128095	protein_coding	KANTR	KDM5C adjacent transcript [Source:HGNC Symbol;Acc:HGNC:49510]	X
ENSG00000182117	4,664975	-1,130842	1,07E-05	0,000469	-6,128477	protein_coding	NOP10	NOP10 ribonucleoprotein [Source:HGNC Symbol;Acc:HGNC:14378]	15
ENSG00000157593	5,032598	-0,907786	1,07E-05	0,000469	-6,129876	protein_coding	SLC35B2	solute carrier family 35 member B2 [Source:HGNC Symbol;Acc:HGNC:16872]	6
ENSG00000274070	0,822316	2,048606	1,08E-05	0,000469	6,126884	protein_coding	CASTOR2	cytosolic arginine sensor for mTORC1 subunit 2 [Source:HGNC Symbol;Acc:HGNC:37073]	7
ENSG00000123416	8,679882	-1,178616	1,07E-05	0,000469	-6,131485	protein_coding	TUBA1B	tubulin alpha 1b [Source:HGNC Symbol;Acc:HGNC:18809]	12
ENSG00000132963	5,769577	-1,008922	1,07E-05	0,000469	-6,128817	protein_coding	POMP	proteasome maturation protein [Source:HGNC Symbol;Acc:HGNC:20330]	13
ENSG00000067334	5,950096	-0,915633	1,07E-05	0,000469	-6,130822	protein_coding	DNTTIP2	deoxynucleotidyltransferase terminal interacting protein 2 [Source:HGNC Symbol;Acc:HGNC:24013]	1
ENSG00000122705	7,042796	-0,864498	1,06E-05	0,000469	-6,13272	protein_coding	CLTA	clathrin light chain A [Source:HGNC Symbol;Acc:HGNC:2090]	9
ENSG00000137747	-1,899134	3,906498	1,06E-05	0,000469	6,133428	protein_coding	TMPRSS13	transmembrane serine protease 13 [Source:HGNC Symbol;Acc:HGNC:29808]	11
ENSG00000084733	6,906604	-0,857986	1,08E-05	0,000471	-6,124009	protein_coding	RAB10	RAB10, member RAS oncogene family [Source:HGNC Symbol;Acc:HGNC:9759]	2
ENSG00000188603	0,369783	2,026013	1,09E-05	0,000472	6,121051	protein_coding	CLN3	CLN3 lysosomal/endosomal transmembrane protein, battenin [Source:HGNC Symbol;Acc:HGNC:2074]	16
ENSG00000185112	4,531338	-2,857149	1,1E-05	0,000473	-6,117613	protein_coding	FAM43A	family with sequence similarity 43 member A [Source:HGNC Symbol;Acc:HGNC:26888]	3
ENSG00000172757	8,5075	-1,007381	1,1E-05	0,000473	-6,118132	protein_coding	CFL1	cofilin 1 [Source:HGNC Symbol;Acc:HGNC:1874]	11
ENSG00000196209	-0,643854	4,532104	1,1E-05	0,000473	6,115104	protein_coding	SIRPB2	signal regulatory protein beta 2 [Source:HGNC Symbol;Acc:HGNC:16247]	20
ENSG00000005102	-2,0633	4,709597	1,1E-05	0,000473	6,114389	protein_coding	MEOX1	mesenchyme homeobox 1 [Source:HGNC Symbol;Acc:HGNC:7013]	17
ENSG00000099246	5,791617	-0,866442	1,11E-05	0,000476	-6,109615	protein_coding	RAB18	RAB18, member RAS oncogene family [Source:HGNC Symbol;Acc:HGNC:14244]	10
ENSG00000178971	5,182514	1,397111	1,12E-05	0,000477	6,104821	protein_coding	CTC1	CST telomere replication complex component 1 [Source:HGNC Symbol;Acc:HGNC:26169]	17
ENSG00000172380	6,101352	-1,755809	1,13E-05	0,000477	-6,102112	protein_coding	GNG12	G protein subunit gamma 12 [Source:HGNC Symbol;Acc:HGNC:19663]	1
ENSG00000087053	5,998097	-1,268523	1,13E-05	0,000477	-6,102091	protein_coding	MTMR2	myotubularin related protein 2 [Source:HGNC Symbol;Acc:HGNC:7450]	11
ENSG00000161217	6,051064	-0,936984	1,13E-05	0,000477	-6,102224	protein_coding	PCYT1A	phosphate cytidylyltransferase 1, choline, alpha [Source:HGNC Symbol;Acc:HGNC:8754]	3
ENSG00000119396	6,40491	-0,785168	1,12E-05	0,000477	-6,106005	protein_coding	RAB14	RAB14, member RAS oncogene family [Source:HGNC Symbol;Acc:HGNC:16524]	9
ENSG00000132603	3,779669	-1,230627	1,14E-05	0,00048	-6,096622	protein_coding	NIP7	nucleolar pre-rRNA processing protein NIP7 [Source:HGNC Symbol;Acc:HGNC:24328]	16
ENSG00000151917	1,525526	-3,5978	1,14E-05	0,00048	-6,097671	protein_coding	BEND6	BEN domain containing 6 [Source:HGNC Symbol;Acc:HGNC:20871]	6
ENSG00000051128	3,693234	-1,980141	1,15E-05	0,000483	-6,092599	protein_coding	HOMER3	homer scaffold protein 3 [Source:HGNC Symbol;Acc:HGNC:17514]	19
ENSG00000073150	2,32827	-2,217152	1,16E-05	0,000484	-6,088995	protein_coding	PANX2	pannexin 2 [Source:HGNC Symbol;Acc:HGNC:8600]	22
ENSG00000161203	7,993013	-1,123455	1,16E-05	0,000484	-6,088073	protein_coding	AP2M1	adaptor related protein complex 2 subunit mu 1 [Source:HGNC Symbol;Acc:HGNC:564]	3
ENSG00000185946	4,376101	1,097862	1,18E-05	0,000486	6,079925	protein_coding	RNPC3	RNA binding region (RNP1, RRM) containing 3 [Source:HGNC Symbol;Acc:HGNC:18666]	1
ENSG00000139278	4,91308	-3,127687	1,19E-05	0,000486	-6,077268	protein_coding	GLIPR1	GLI pathogenesis related 1 [Source:HGNC Symbol;Acc:HGNC:17001]	12
ENSG00000214160	4,950796	-1,131495	1,19E-05	0,000486	-6,076047	protein_coding	ALG3	ALG3 alpha-1,3- mannosyltransferase [Source:HGNC Symbol;Acc:HGNC:23056]	3
ENSG00000112659	6,04754	1,082544	1,2E-05	0,000486	6,071216	protein_coding	CUL9	cullin 9 [Source:HGNC Symbol;Acc:HGNC:15982]	6
ENSG00000162909	8,222491	-1,291683	1,19E-05	0,000486	-6,076121	protein_coding	CAPN2	calpain 2 [Source:HGNC Symbol;Acc:HGNC:1479]	1
ENSG00000175582	7,039813	-0,920302	1,17E-05	0,000486	-6,083449	protein_coding	RAB6A	RAB6A, member RAS oncogene family [Source:HGNC Symbol;Acc:HGNC:9786]	11
ENSG00000095139	7,915427	-1,154816	1,19E-05	0,000486	-6,076834	protein_coding	ARCN1	archain 1 [Source:HGNC Symbol;Acc:HGNC:649]	11
ENSG00000196141	6,928942	-1,698748	1,2E-05	0,000486	-6,073581	protein_coding	SPATS2L	spermatogenesis associated serine rich 2 like [Source:HGNC Symbol;Acc:HGNC:24574]	2
ENSG00000107959	6,693776	-0,805662	1,2E-05	0,000486	-6,071621	protein_coding	PITRM1	pitrilysin metallopeptidase 1 [Source:HGNC Symbol;Acc:HGNC:17663]	10
ENSG00000136758	6,998492	-0,665084	1,2E-05	0,000486	-6,072747	protein_coding	YME1L1	YME1 like 1 ATPase [Source:HGNC Symbol;Acc:HGNC:12843]	10
ENSG00000148357	0,215243	3,104725	1,18E-05	0,000486	6,079198	protein_coding	HMCN2	hemicentin 2 [Source:HGNC Symbol;Acc:HGNC:21293]	9
ENSG00000115008	-1,58612	-4,393136	1,18E-05	0,000486	-6,082355	protein_coding	IL1A	interleukin 1 alpha [Source:HGNC Symbol;Acc:HGNC:5991]	2
ENSG00000168268	6,441785	-1,559383	1,22E-05	0,000494	-6,061863	protein_coding	NT5DC2	5'-nucleotidase domain containing 2 [Source:HGNC Symbol;Acc:HGNC:25717]	3
ENSG00000100934	6,617294	-1,430497	1,24E-05	0,000496	-6,057232	protein_coding	SEC23A	Sec23 homolog A, COPII coat complex component [Source:HGNC Symbol;Acc:HGNC:10701]	14
ENSG00000130528	-0,399015	3,292915	1,23E-05	0,000496	6,057698	protein_coding	HRC	histidine rich calcium binding protein [Source:HGNC Symbol;Acc:HGNC:5178]	19
ENSG00000116221	5,285446	-0,925027	1,24E-05	0,000498	-6,053451	protein_coding	MRPL37	mitochondrial ribosomal protein L37 [Source:HGNC Symbol;Acc:HGNC:14034]	1
ENSG00000134460	-0,382823	5,871429	1,27E-05	0,000507	6,043776	protein_coding	IL2RA	interleukin 2 receptor subunit alpha [Source:HGNC Symbol;Acc:HGNC:6008]	10

ENSG00000139926	5,264862	-3,105615	1,28E-05	0,00051	-6,039225	protein_coding	FRMD6	FERM domain containing 6 [Source:HGNC Symbol;Acc:HGNC:19839]	14
ENSG00000100196	4,030366	-3,478275	1,29E-05	0,000513	-6,034764	protein_coding	KDELR3	KDEL endoplasmic reticulum protein retention receptor 3 [Source:HGNC Symbol;Acc:HGNC:6306]	22
ENSG00000019582	7,344274	2,968459	1,31E-05	0,000515	6,029378	protein_coding	CD74	CD74 molecule [Source:HGNC Symbol;Acc:HGNC:1697]	5
ENSG00000111412	5,843284	-1,408135	1,31E-05	0,000515	-6,029436	protein_coding	SPRING1	SREBF pathway regulator in golgi 1 [Source:HGNC Symbol;Acc:HGNC:26128]	12
ENSG00000109046	6,816439	0,971634	1,3E-05	0,000515	6,031503	protein_coding	WSB1	WD repeat and SOCS box containing 1 [Source:HGNC Symbol;Acc:HGNC:19221]	17
ENSG00000145354	4,829184	-1,130044	1,34E-05	0,000525	-6,016873	protein_coding	CISD2	CDGSH iron sulfur domain 2 [Source:HGNC Symbol;Acc:HGNC:24212]	4
ENSG00000161638	7,416117	-2,349351	1,33E-05	0,000525	-6,018008	protein_coding	ITGA5	integrin subunit alpha 5 [Source:HGNC Symbol;Acc:HGNC:6141]	12
ENSG00000173950	4,235475	-1,816159	1,34E-05	0,000526	-6,015285	protein_coding	XXYLT1	xyloside xylosyltransferase 1 [Source:HGNC Symbol;Acc:HGNC:26639]	3
ENSG00000185482	1,461964	1,983646	1,35E-05	0,000526	6,013677	protein_coding	STAC3	SH3 and cysteine rich domain 3 [Source:HGNC Symbol;Acc:HGNC:28423]	12
ENSG00000087245	7,558674	-3,383517	1,35E-05	0,000528	-6,010825	protein_coding	MMP2	matrix metallopeptidase 2 [Source:HGNC Symbol;Acc:HGNC:7166]	16
ENSG00000074047	4,946308	-2,810788	1,36E-05	0,000529	-6,00743	protein_coding	GLI2	GLI family zinc finger 2 [Source:HGNC Symbol;Acc:HGNC:4318]	2
ENSG00000108854	5,777201	-1,134109	1,37E-05	0,000529	-6,00556	protein_coding	SMURF2	SMAD specific E3 ubiquitin protein ligase 2 [Source:HGNC Symbol;Acc:HGNC:16809]	17
ENSG00000168329	-2,04683	3,491809	1,37E-05	0,000529	6,006346	protein_coding	CX3CR1	C-X3-C motif chemokine receptor 1 [Source:HGNC Symbol;Acc:HGNC:2558]	3
ENSG00000142512	0,869635	4,217733	1,38E-05	0,000534	6,00005	protein_coding	SIGLEC10	sialic acid binding Ig like lectin 10 [Source:HGNC Symbol;Acc:HGNC:15620]	19
ENSG00000196576	8,679602	-0,914016	1,39E-05	0,000534	-5,996515	protein_coding	PLXNB2	plexin B2 [Source:HGNC Symbol;Acc:HGNC:9104]	22
ENSG00000090054	6,062539	-0,835546	1,39E-05	0,000534	-5,997431	protein_coding	SPTLC1	serine palmitoyltransferase long chain base subunit 1 [Source:HGNC Symbol;Acc:HGNC:11277]	9
ENSG00000101608	7,569346	-1,812369	1,39E-05	0,000534	-5,997873	protein_coding	MYL12A	myosin light chain 12A [Source:HGNC Symbol;Acc:HGNC:16701]	18
ENSG00000155366	5,323608	-1,590808	1,41E-05	0,000536	-5,991988	protein_coding	RHOC	ras homolog family member C [Source:HGNC Symbol;Acc:HGNC:669]	1
ENSG00000167207	-0,18833	2,919475	1,41E-05	0,000536	5,992063	protein_coding	NOD2	nucleotide binding oligomerization domain containing 2 [Source:HGNC Symbol;Acc:HGNC:5331]	16
ENSG00000196597	3,892837	1,667491	1,41E-05	0,000538	5,989345	protein_coding	ZNF782	zinc finger protein 782 [Source:HGNC Symbol;Acc:HGNC:33110]	9
ENSG00000213593	5,241524	-0,664572	1,42E-05	0,00054	-5,986369	protein_coding	TMX2	thioredoxin related transmembrane protein 2 [Source:HGNC Symbol;Acc:HGNC:30739]	11
ENSG00000136895	2,581488	2,013792	1,43E-05	0,000542	5,981793	protein_coding	GARNL3	GTPase activating Rap/RanGAP domain like 3 [Source:HGNC Symbol;Acc:HGNC:25425]	9
ENSG00000143702	6,889551	-1,361811	1,43E-05	0,000542	-5,981571	protein_coding	CEP170	centrosomal protein 170 [Source:HGNC Symbol;Acc:HGNC:28920]	1
ENSG00000172315	4,187835	-1,050266	1,45E-05	0,000544	-5,977816	protein_coding	TP53RK	TP53 regulating kinase [Source:HGNC Symbol;Acc:HGNC:16197]	20
ENSG00000197081	8,859489	-0,99159	1,45E-05	0,000544	-5,97733	protein_coding	IGF2R	insulin like growth factor 2 receptor [Source:HGNC Symbol;Acc:HGNC:5467]	6
ENSG00000142867	4,312338	-1,286641	1,46E-05	0,000548	-5,973085	protein_coding	BCL10	BCL10 immune signaling adaptor [Source:HGNC Symbol;Acc:HGNC:989]	1
ENSG00000145020	-0,919138	3,178693	1,47E-05	0,00055	5,969548	protein_coding	AMT	aminomethyltransferase [Source:HGNC Symbol;Acc:HGNC:473]	3
ENSG00000173221	4,841194	-1,995574	1,49E-05	0,000554	-5,963624	protein_coding	GLRX	glutaredoxin [Source:HGNC Symbol;Acc:HGNC:4330]	5
ENSG00000117318	5,532301	-2,08645	1,48E-05	0,000554	-5,964412	protein_coding	ID3	inhibitor of DNA binding 3, HLH protein [Source:HGNC Symbol;Acc:HGNC:5362]	1
ENSG00000137831	8,41477	-2,07318	1,49E-05	0,000554	-5,962746	protein_coding	UACA	uveal autoantigen with coiled-coil domains and ankyrin repeats [Source:HGNC Symbol;Acc:HGNC:15947]	15
ENSG00000163617	3,266722	2,083212	1,51E-05	0,00056	5,95569	protein_coding	CCDC191	coiled-coil domain containing 191 [Source:HGNC Symbol;Acc:HGNC:29272]	3
ENSG00000132965	0,194149	3,50395	1,52E-05	0,000563	5,952053	protein_coding	ALOX5AP	arachidonate 5-lipoxygenase activating protein [Source:HGNC Symbol;Acc:HGNC:436]	13
ENSG00000099994	4,729162	-3,343657	1,55E-05	0,000573	-5,941915	protein_coding	SUSD2	sushi domain containing 2 [Source:HGNC Symbol;Acc:HGNC:30667]	22
ENSG00000103342	7,321802	-1,075034	1,56E-05	0,000576	-5,938678	protein_coding	GSPT1	G1 to S phase transition 1 [Source:HGNC Symbol;Acc:HGNC:4621]	16
ENSG00000166750	6,176348	-1,924114	1,59E-05	0,000582	-5,930294	protein_coding	SLFN5	schlafen family member 5 [Source:HGNC Symbol;Acc:HGNC:28286]	17
ENSG00000155096	7,424006	-0,794647	1,58E-05	0,000582	-5,932112	protein_coding	AZIN1	antizyme inhibitor 1 [Source:HGNC Symbol;Acc:HGNC:16432]	8
ENSG00000009790	-0,006787	4,207781	1,59E-05	0,000582	5,93107	protein_coding	TRAF3IP3	TRAF3 interacting protein 3 [Source:HGNC Symbol;Acc:HGNC:30766]	1
ENSG00000085433	4,64331	-0,904112	1,61E-05	0,000588	-5,923773	protein_coding	WDR47	WD repeat domain 47 [Source:HGNC Symbol;Acc:HGNC:29141]	1
ENSG00000198369	5,981214	-1,515348	1,62E-05	0,000588	-5,920157	protein_coding	SPRED2	sprouty related EVH1 domain containing 2 [Source:HGNC Symbol;Acc:HGNC:17722]	2
ENSG00000083444	7,426271	-1,799474	1,62E-05	0,000588	-5,921407	protein_coding	PLOD1	procollagen-lysine,2-oxoglutarate 5-dioxygenase 1 [Source:HGNC Symbol;Acc:HGNC:9081]	1
ENSG00000178809	-1,746175	3,392487	1,62E-05	0,000588	5,92041	protein_coding	TRIM73	tripartite motif containing 73 [Source:HGNC Symbol;Acc:HGNC:18162]	7
ENSG00000116729	6,359336	-2,549142	1,63E-05	0,00059	-5,91706	protein_coding	WLS	Wnt ligand secretion mediator [Source:HGNC Symbol;Acc:HGNC:30238]	1
ENSG00000065135	6,384225	-0,966737	1,66E-05	0,000601	-5,906879	protein_coding	GNAI3	G protein subunit alpha i3 [Source:HGNC Symbol;Acc:HGNC:4387]	1
ENSG00000141756	7,396002	-2,089302	1,67E-05	0,000602	-5,904923	protein_coding	FKBP10	FKBP prolyl isomerase 10 [Source:HGNC Symbol;Acc:HGNC:18169]	17
ENSG00000266173	3,471139	1,495577	1,68E-05	0,000603	5,901168	protein_coding	STRADA	STE20 related adaptor alpha [Source:HGNC Symbol;Acc:HGNC:30172]	17
ENSG00000158805	4,683309	0,904043	1,68E-05	0,000603	5,903097	protein_coding	ZNF276	zinc finger protein 276 [Source:HGNC Symbol;Acc:HGNC:23330]	16
ENSG00000049323	7,132259	-2,497134	1,68E-05	0,000603	-5,901776	protein_coding	LTBP1	latent transforming growth factor beta binding protein 1 [Source:HGNC Symbol;Acc:HGNC:6714]	2
ENSG00000164933	2,504702	-1,344066	1,7E-05	0,000607	-5,896382	protein_coding	SLC25A32	solute carrier family 25 member 32 [Source:HGNC Symbol;Acc:HGNC:29683]	8
ENSG00000149380	2,621358	-2,976404	1,71E-05	0,000608	-5,893146	protein_coding	P4HA3	prolyl 4-hydroxylase subunit alpha 3 [Source:HGNC Symbol;Acc:HGNC:30135]	11
ENSG00000165434	5,1598	-1,359367	1,71E-05	0,000608	-5,893243	protein_coding	PGM2L1	phosphoglucomutase 2 like 1 [Source:HGNC Symbol;Acc:HGNC:20898]	11
ENSG00000048828	7,700662	-0,698305	1,71E-05	0,000608	-5,892424	protein_coding	FAM120A	family with sequence similarity 120A [Source:HGNC Symbol;Acc:HGNC:13247]	9

ENSG00000196460	1,295419	-2,702861	1,72E-05	0,00061	-5,889296	protein_coding	RFX8	regulatory factor X8 [Source:HGNC Symbol;Acc:HGNC:37253]	2
ENSG00000105516	3,142508	1,85109	1,73E-05	0,000611	5,888066	protein_coding	DBP	D-box binding PAR bZIP transcription factor [Source:HGNC Symbol;Acc:HGNC:2697]	19
ENSG00000105122	0,724703	4,065692	1,74E-05	0,000613	5,885122	protein_coding	RASAL3	RAS protein activator like 3 [Source:HGNC Symbol;Acc:HGNC:26129]	19
ENSG00000104312	3,726902	-1,609587	1,76E-05	0,000614	-5,878636	protein_coding	RIPK2	receptor interacting serine/threonine kinase 2 [Source:HGNC Symbol;Acc:HGNC:10020]	8
ENSG00000183718	4,727339	1,18846	1,76E-05	0,000614	5,879118	protein_coding	TRIM52	tripartite motif containing 52 [Source:HGNC Symbol;Acc:HGNC:19024]	5
ENSG00000162148	0,376686	2,186863	1,76E-05	0,000614	5,879868	protein_coding	PPP1R32	protein phosphatase 1 regulatory subunit 32 [Source:HGNC Symbol;Acc:HGNC:28869]	11
ENSG00000100764	5,760917	-0,920629	1,75E-05	0,000614	-5,881505	protein_coding	PSMC1	proteasome 26S subunit, ATPase 1 [Source:HGNC Symbol;Acc:HGNC:9547]	14
ENSG00000115844	0,550603	-4,911994	1,75E-05	0,000614	-5,880458	protein_coding	DLX2	distal-less homeobox 2 [Source:HGNC Symbol;Acc:HGNC:2915]	2
ENSG00000180644	-1,454516	4,550307	1,77E-05	0,000615	5,876622	protein_coding	PRF1	perforin 1 [Source:HGNC Symbol;Acc:HGNC:9360]	10
ENSG00000023572	4,087497	-1,15415	1,8E-05	0,000621	-5,869019	protein_coding	GLRX2	glutaredoxin 2 [Source:HGNC Symbol;Acc:HGNC:16065]	1
ENSG00000197956	8,169083	-2,221646	1,8E-05	0,000621	-5,867999	protein_coding	S100A6	S100 calcium binding protein A6 [Source:HGNC Symbol;Acc:HGNC:10496]	1
ENSG00000140465	0,997635	-7,722236	1,8E-05	0,000621	-5,86868	protein_coding	CYP1A1	cytochrome P450 family 1 subfamily A member 1 [Source:HGNC Symbol;Acc:HGNC:2595]	15
ENSG00000147113	-0,117611	6,595419	1,79E-05	0,000621	5,869308	protein_coding	DIPK2B	divergent protein kinase domain 2B [Source:HGNC Symbol;Acc:HGNC:25866]	X
ENSG00000166073	4,707126	-2,448738	1,83E-05	0,000623	-5,860472	protein_coding	GPR176	G protein-coupled receptor 176 [Source:HGNC Symbol;Acc:HGNC:32370]	15
ENSG00000143977	4,682004	-1,106752	1,83E-05	0,000623	-5,858506	protein_coding	SNRPG	small nuclear ribonucleoprotein polypeptide G [Source:HGNC Symbol;Acc:HGNC:11163]	2
ENSG00000198018	5,243568	-1,246205	1,82E-05	0,000623	-5,863559	protein_coding	ENTPD7	ectonucleoside triphosphate diphosphohydrolase 7 [Source:HGNC Symbol;Acc:HGNC:19745]	10
ENSG00000132326	5,451465	1,468419	1,83E-05	0,000623	5,859427	protein_coding	PER2	period circadian regulator 2 [Source:HGNC Symbol;Acc:HGNC:8846]	2
ENSG00000135976	6,011505	2,198703	1,83E-05	0,000623	5,860603	protein_coding	ANKRD36	ankyrin repeat domain 36 [Source:HGNC Symbol;Acc:HGNC:24079]	2
ENSG00000133657	7,78533	-1,209921	1,81E-05	0,000623	-5,864255	protein_coding	ATP13A3	ATPase 13A3 [Source:HGNC Symbol;Acc:HGNC:24113]	3
ENSG00000107651	6,176633	-0,724357	1,83E-05	0,000623	-5,858879	protein_coding	SEC23IP	SEC23 interacting protein [Source:HGNC Symbol;Acc:HGNC:17018]	10
ENSG00000100439	4,666578	-1,007656	1,85E-05	0,000627	-5,853703	protein_coding	ABHD4	abhydrolase domain containing 4, N-acyl phospholipase B [Source:HGNC Symbol;Acc:HGNC:20154]	14
ENSG00000047617	-0,801414	4,032167	1,85E-05	0,000627	5,854743	protein_coding	ANO2	anoctamin 2 [Source:HGNC Symbol;Acc:HGNC:1183]	12
ENSG00000105339	4,977655	1,321416	1,86E-05	0,000627	5,852071	protein_coding	DENND3	DENN domain containing 3 [Source:HGNC Symbol;Acc:HGNC:29134]	8
ENSG00000166136	2,568118	1,176894	1,87E-05	0,00063	5,848601	protein_coding	NDUFB8	NADH:ubiquinone oxidoreductase subunit B8 [Source:HGNC Symbol;Acc:HGNC:7703]	10
ENSG00000157680	3,471317	-3,125616	1,88E-05	0,00063	-5,845264	protein_coding	DGKI	diacylglycerol kinase iota [Source:HGNC Symbol;Acc:HGNC:2855]	7
ENSG00000198843	5,703354	-1,007623	1,88E-05	0,00063	-5,846094	protein_coding	SELENOT	selenoprotein T [Source:HGNC Symbol;Acc:HGNC:18136]	3
ENSG00000056097	7,440984	-0,602605	1,87E-05	0,00063	-5,847583	protein_coding	ZFR	zinc finger RNA binding protein [Source:HGNC Symbol;Acc:HGNC:17277]	5
ENSG00000128383	-2,672227	3,693932	1,88E-05	0,00063	5,847192	protein_coding	APOBEC3A	apolipoprotein B mRNA editing enzyme catalytic subunit 3A [Source:HGNC Symbol;Acc:HGNC:17343]	22
ENSG00000105374	-1,64975	4,219734	1,89E-05	0,000632	5,842364	protein_coding	NKG7	natural killer cell granule protein 7 [Source:HGNC Symbol;Acc:HGNC:7830]	19
ENSG00000073605	3,021	1,908681	1,92E-05	0,00064	5,834984	protein_coding	GSDMB	gasdermin B [Source:HGNC Symbol;Acc:HGNC:23690]	17
ENSG00000037280	1,537426	5,436989	1,93E-05	0,000641	5,833207	protein_coding	FLT4	fms related receptor tyrosine kinase 4 [Source:HGNC Symbol;Acc:HGNC:3767]	5
ENSG00000108848	7,809269	1,236251	1,93E-05	0,000641	5,832466	protein_coding	LUC7L3	LUC7 like 3 pre-mRNA splicing factor [Source:HGNC Symbol;Acc:HGNC:24309]	17
ENSG00000109906	2,98333	6,46656	1,95E-05	0,000642	5,828173	protein_coding	ZBTB16	zinc finger and BTB domain containing 16 [Source:HGNC Symbol;Acc:HGNC:12930]	11
ENSG00000171865	4,458578	-1,0066	1,94E-05	0,000642	-5,829609	protein_coding	RNASEH1	ribonuclease H1 [Source:HGNC Symbol;Acc:HGNC:18466]	2
ENSG00000163466	7,49942	-1,125128	1,95E-05	0,000642	-5,827982	protein_coding	ARPC2	actin related protein 2/3 complex subunit 2 [Source:HGNC Symbol;Acc:HGNC:705]	2
ENSG00000110077	1,668335	4,583772	1,96E-05	0,000645	5,824917	protein_coding	MS4A6A	membrane spanning 4-domains A6A [Source:HGNC Symbol;Acc:HGNC:13375]	11
ENSG00000187678	6,177365	-2,261874	1,97E-05	0,000645	-5,822635	protein_coding	SPRY4	sprouty RTK signaling antagonist 4 [Source:HGNC Symbol;Acc:HGNC:15533]	5
ENSG00000204482	0,076104	3,497699	1,97E-05	0,000645	5,822974	protein_coding	LST1	leukocyte specific transcript 1 [Source:HGNC Symbol;Acc:HGNC:14189]	6
ENSG00000104731	4,917796	1,30851	1,98E-05	0,000648	5,819451	protein_coding	KLHDC4	kelch domain containing 4 [Source:HGNC Symbol;Acc:HGNC:25272]	16
ENSG00000171864	-2,659817	3,914314	1,99E-05	0,000648	5,818668	protein_coding	PRND	prion like protein doppel [Source:HGNC Symbol;Acc:HGNC:15748]	20
ENSG00000164305	4,503003	-1,105637	2E-05	0,00065	-5,815801	protein_coding	CASP3	caspase 3 [Source:HGNC Symbol;Acc:HGNC:1504]	4
ENSG00000150779	4,657713	-1,051068	2,01E-05	0,000652	-5,81382	protein_coding	TIMM8B	translocase of inner mitochondrial membrane 8 homolog B [Source:HGNC Symbol;Acc:HGNC:11818]	11
ENSG00000165886	3,913667	-1,472507	2,03E-05	0,000654	-5,806528	protein_coding	UBTD1	ubiquitin domain containing 1 [Source:HGNC Symbol;Acc:HGNC:25683]	10
ENSG00000171451	4,896394	-3,214342	2,04E-05	0,000654	-5,804549	protein_coding	DSEL	dermatan sulfate epimerase like [Source:HGNC Symbol;Acc:HGNC:18144]	18
ENSG00000173846	4,516826	-1,932499	2,04E-05	0,000654	-5,804766	protein_coding	PLK3	polo like kinase 3 [Source:HGNC Symbol;Acc:HGNC:2154]	1
ENSG00000138061	5,819999	-3,730628	2,02E-05	0,000654	-5,809299	protein_coding	CYP1B1	cytochrome P450 family 1 subfamily B member 1 [Source:HGNC Symbol;Acc:HGNC:2597]	2
ENSG00000138119	7,30926	-2,172985	2,03E-05	0,000654	-5,808019	protein_coding	MYOF	myoferlin [Source:HGNC Symbol;Acc:HGNC:3656]	10
ENSG00000197632	0,032173	-4,793565	2,04E-05	0,000654	-5,804544	protein_coding	SERPINB2	serpin family B member 2 [Source:HGNC Symbol;Acc:HGNC:8584]	18
ENSG00000143632	-0,68322	2,203635	2,03E-05	0,000654	5,80792	protein_coding	ACTA1	actin alpha 1, skeletal muscle [Source:HGNC Symbol;Acc:HGNC:129]	1
ENSG00000262484	-0,720543	3,110146	2,04E-05	0,000654	5,805812	protein_coding	CCER2	coiled-coil glutamate rich protein 2 [Source:HGNC Symbol;Acc:HGNC:44662]	19
ENSG00000155304	6,136878	-1,05026	2,05E-05	0,000654	-5,80341	protein_coding	HSPA13	heat shock protein family A (Hsp70) member 13 [Source:HGNC Symbol;Acc:HGNC:11375]	21

ENSG00000156599	6,781324	-0,769917	2,05E-05	0,000654	-5,802378	protein_coding	ZDHHC5	zinc finger DHHC-type palmitoyltransferase 5 [Source:HGNC Symbol;Acc:HGNC:18472]	11
ENSG00000142657	6,610522	-1,397731	2,08E-05	0,00066	-5,796813	protein_coding	PGD	phosphogluconate dehydrogenase [Source:HGNC Symbol;Acc:HGNC:8891]	1
ENSG00000174021	3,582467	-1,704538	2,09E-05	0,000664	-5,792596	protein_coding	GNG5	G protein subunit gamma 5 [Source:HGNC Symbol;Acc:HGNC:4408]	1
ENSG00000129204	-1,18135	3,742246	2,11E-05	0,000668	5,788961	protein_coding	USP6	ubiquitin specific peptidase 6 [Source:HGNC Symbol;Acc:HGNC:12629]	17
ENSG00000138641	0,509797	2,585852	2,12E-05	0,000668	5,786696	protein_coding	HERC3	HECT and RLD domain containing E3 ubiquitin protein ligase 3 [Source:HGNC Symbol;Acc:HGNC:4876]	4
ENSG00000138448	7,721683	-1,509792	2,11E-05	0,000668	-5,787413	protein_coding	ITGAV	integrin subunit alpha V [Source:HGNC Symbol;Acc:HGNC:6150]	2
ENSG00000160746	4,735707	-0,963289	2,13E-05	0,00067	-5,784145	protein_coding	ANO10	anoctamin 10 [Source:HGNC Symbol;Acc:HGNC:25519]	3
ENSG00000052802	5,114689	-1,898588	2,13E-05	0,00067	-5,783413	protein_coding	MSMO1	methylsterol monooxygenase 1 [Source:HGNC Symbol;Acc:HGNC:10545]	4
ENSG00000169660	4,42421	1,258084	2,14E-05	0,00067	5,781423	protein_coding	HEXD	hexosaminidase D [Source:HGNC Symbol;Acc:HGNC:26307]	17
ENSG00000176715	4,679949	0,975564	2,14E-05	0,00067	5,78143	protein_coding	ACSF3	acyl-CoA synthetase family member 3 [Source:HGNC Symbol;Acc:HGNC:27288]	16
ENSG00000279765	2,56344	2,609431	2,16E-05	0,000675	5,775955	protein_coding	AC013394.	novel protein	15
ENSG00000105483	4,994511	1,111152	2,16E-05	0,000675	5,775811	protein_coding	CARD8	caspase recruitment domain family member 8 [Source:HGNC Symbol;Acc:HGNC:17057]	19
ENSG00000025772	4,990796	-0,936657	2,17E-05	0,000675	-5,775159	protein_coding	TOMM34	translocase of outer mitochondrial membrane 34 [Source:HGNC Symbol;Acc:HGNC:15746]	20
ENSG00000244405	5,038117	-1,684249	2,18E-05	0,000675	-5,772789	protein_coding	ETV5	ETS variant transcription factor 5 [Source:HGNC Symbol;Acc:HGNC:3494]	3
ENSG00000150048	-1,916891	3,837758	2,18E-05	0,000675	5,77321	protein_coding	CLEC1A	C-type lectin domain family 1 member A [Source:HGNC Symbol;Acc:HGNC:24355]	12
ENSG00000265354	3,082295	-1,198799	2,2E-05	0,00068	-5,767386	protein_coding	TIMM23	translocase of inner mitochondrial membrane 23 [Source:HGNC Symbol;Acc:HGNC:17312]	10
ENSG00000213203	-0,158697	3,680995	2,2E-05	0,00068	5,767176	protein_coding	GIMAP1	GTPase, IMAP family member 1 [Source:HGNC Symbol;Acc:HGNC:23237]	7
ENSG00000183578	2,151822	-2,741639	2,22E-05	0,000683	-5,763116	protein_coding	TNFAIP8L3	TNF alpha induced protein 8 like 3 [Source:HGNC Symbol;Acc:HGNC:20620]	15
ENSG00000134294	7,860975	-1,776386	2,22E-05	0,000683	-5,763649	protein_coding	SLC38A2	solute carrier family 38 member 2 [Source:HGNC Symbol;Acc:HGNC:13448]	12
ENSG00000213977	2,649191	-1,614742	2,23E-05	0,000686	-5,759935	protein_coding	TAX1BP3	Tax1 binding protein 3 [Source:HGNC Symbol;Acc:HGNC:30684]	17
ENSG00000087157	4,798181	0,754534	2,24E-05	0,000686	5,759591	protein_coding	PGS1	phosphatidylglycerophosphate synthase 1 [Source:HGNC Symbol;Acc:HGNC:30029]	17
ENSG00000110218	5,333005	-1,07312	2,25E-05	0,000687	-5,7575	protein_coding	PANX1	pannexin 1 [Source:HGNC Symbol;Acc:HGNC:8599]	11
ENSG00000115762	6,608568	-0,87414	2,25E-05	0,000687	-5,756937	protein_coding	PLEKHB2	pleckstrin homology domain containing B2 [Source:HGNC Symbol;Acc:HGNC:19236]	2
ENSG00000137309	6,875367	-2,300721	2,25E-05	0,000687	-5,755867	protein_coding	HMGA1	high mobility group AT-hook 1 [Source:HGNC Symbol;Acc:HGNC:5010]	6
ENSG00000010327	5,180629	3,369467	2,27E-05	0,000688	5,751221	protein_coding	STAB1	stabilin 1 [Source:HGNC Symbol;Acc:HGNC:18628]	3
ENSG00000160799	4,805951	0,928662	2,27E-05	0,000688	5,751253	protein_coding	CCDC12	coiled-coil domain containing 12 [Source:HGNC Symbol;Acc:HGNC:28332]	3
ENSG00000169504	7,279272	-1,707846	2,26E-05	0,000688	-5,753988	protein_coding	CLIC4	chloride intracellular channel 4 [Source:HGNC Symbol;Acc:HGNC:13518]	1
ENSG00000174885	-2,32516	3,799395	2,27E-05	0,000688	5,752547	protein_coding	NLRP6	NLR family pyrin domain containing 6 [Source:HGNC Symbol;Acc:HGNC:22944]	11
ENSG00000198715	4,924216	-1,195677	2,28E-05	0,00069	-5,749102	protein_coding	GLMP	glycosylated lysosomal membrane protein [Source:HGNC Symbol;Acc:HGNC:29436]	1
ENSG00000168970	1,591902	2,196143	2,29E-05	0,000691	5,747237	protein_coding	JMJD7-PLA	JMJD7-PLA2G4B readthrough [Source:HGNC Symbol;Acc:HGNC:34449]	15
ENSG00000138468	4,786538	1,101024	2,3E-05	0,000692	5,745903	protein_coding	SENP7	SUMO specific peptidase 7 [Source:HGNC Symbol;Acc:HGNC:30402]	3
ENSG00000269743	2,951232	1,135832	2,31E-05	0,000695	5,742554	protein_coding	SLC25A53	solute carrier family 25 member 53 [Source:HGNC Symbol;Acc:HGNC:31894]	X
ENSG00000157193	4,918078	-1,70814	2,33E-05	0,000699	-5,738216	protein_coding	LRP8	LDL receptor related protein 8 [Source:HGNC Symbol;Acc:HGNC:6700]	1
ENSG00000256188	0,275804	2,938109	2,34E-05	0,000699	5,737116	protein_coding	TAS2R30	taste 2 receptor member 30 [Source:HGNC Symbol;Acc:HGNC:19112]	12
ENSG00000119535	0,850322	5,018888	2,35E-05	0,000699	5,735506	protein_coding	CSF3R	colony stimulating factor 3 receptor [Source:HGNC Symbol;Acc:HGNC:2439]	1
ENSG00000177311	7,224411	-1,300043	2,34E-05	0,000699	-5,737467	protein_coding	ZBTB38	zinc finger and BTB domain containing 38 [Source:HGNC Symbol;Acc:HGNC:26636]	3
ENSG00000171777	-1,298543	3,961089	2,35E-05	0,000699	5,735709	protein_coding	RASGRP4	RAS guanyl releasing protein 4 [Source:HGNC Symbol;Acc:HGNC:18958]	19
ENSG00000104783	1,523155	-2,86331	2,36E-05	0,000699	-5,732921	protein_coding	KCNN4	potassium calcium-activated channel subfamily N member 4 [Source:HGNC Symbol;Acc:HGNC:6293]	19
ENSG00000171150	5,671588	-1,532456	2,36E-05	0,000699	-5,733708	protein_coding	SOCS5	suppressor of cytokine signaling 5 [Source:HGNC Symbol;Acc:HGNC:16852]	2
ENSG00000243708	-0,47934	2,252442	2,36E-05	0,000699	5,73244	protein_coding	PLA2G4B	phospholipase A2 group IVB [Source:HGNC Symbol;Acc:HGNC:9036]	15
ENSG00000119185	4,802549	-0,849303	2,42E-05	0,000708	-5,721226	protein_coding	ITGB1BP1	integrin subunit beta 1 binding protein 1 [Source:HGNC Symbol;Acc:HGNC:23927]	2
ENSG00000155254	5,639368	-1,646447	2,42E-05	0,000708	-5,719729	protein_coding	MARVELD1	MARVEL domain containing 1 [Source:HGNC Symbol;Acc:HGNC:28674]	10
ENSG00000124225	5,915366	-2,13676	2,41E-05	0,000708	-5,72231	protein_coding	PMEPA1	prostate transmembrane protein, androgen induced 1 [Source:HGNC Symbol;Acc:HGNC:14107]	20
ENSG00000113583	5,775298	-1,013292	2,42E-05	0,000708	-5,719762	protein_coding	C5orf15	chromosome 5 open reading frame 15 [Source:HGNC Symbol;Acc:HGNC:20656]	5
ENSG00000197712	6,599538	-1,840176	2,4E-05	0,000708	-5,723875	protein_coding	FAM114A1	family with sequence similarity 114 member A1 [Source:HGNC Symbol;Acc:HGNC:25087]	4
ENSG00000176658	7,882385	-1,010297	2,4E-05	0,000708	-5,724546	protein_coding	MYO1D	myosin ID [Source:HGNC Symbol;Acc:HGNC:7598]	17
ENSG00000115310	8,066878	-1,192271	2,42E-05	0,000708	-5,719662	protein_coding	RTN4	reticulon 4 [Source:HGNC Symbol;Acc:HGNC:14085]	2
ENSG00000134987	5,724296	-0,753915	2,44E-05	0,000709	-5,717359	protein_coding	WDR36	WD repeat domain 36 [Source:HGNC Symbol;Acc:HGNC:30696]	5
ENSG00000162734	7,212665	-1,455144	2,44E-05	0,000709	-5,717125	protein_coding	PEA15	proliferation and apoptosis adaptor protein 15 [Source:HGNC Symbol;Acc:HGNC:8822]	1
ENSG00000008382	4,150366	1,166254	2,45E-05	0,000709	5,714714	protein_coding	MPND	MPN domain containing [Source:HGNC Symbol;Acc:HGNC:25934]	19
ENSG00000106853	4,877095	-2,601813	2,46E-05	0,000709	-5,713148	protein_coding	PTGR1	prostaglandin reductase 1 [Source:HGNC Symbol;Acc:HGNC:18429]	9

ENSG00000187554	0,845178	3,12911	2,44E-05	0,000709	5,71603	protein_coding	TLR5	toll like receptor 5 [Source:HGNC Symbol;Acc:HGNC:11851]	1
ENSG00000198856	5,59951	-1,456522	2,45E-05	0,000709	-5,714097	protein_coding	OSTC	oligosaccharyltransferase complex non-catalytic subunit [Source:HGNC Symbol;Acc:HGNC:24448]	4
ENSG00000151135	6,10839	-1,186967	2,46E-05	0,000709	-5,712677	protein_coding	TMEM263	transmembrane protein 263 [Source:HGNC Symbol;Acc:HGNC:28281]	12
ENSG00000164902	5,536257	-0,764546	2,48E-05	0,000713	-5,70792	protein_coding	PHAX	phosphorylated adaptor for RNA export [Source:HGNC Symbol;Acc:HGNC:10241]	5
ENSG00000106105	7,958701	-1,153985	2,48E-05	0,000713	-5,707668	protein_coding	GARS1	glycyl-tRNA synthetase 1 [Source:HGNC Symbol;Acc:HGNC:4162]	7
ENSG00000134571	-2,119068	2,911654	2,48E-05	0,000713	5,707783	protein_coding	MYBPC3	myosin binding protein C3 [Source:HGNC Symbol;Acc:HGNC:7551]	11
ENSG00000167491	7,280509	-0,807943	2,5E-05	0,000715	-5,705056	protein_coding	GATAD2A	GATA zinc finger domain containing 2A [Source:HGNC Symbol;Acc:HGNC:29989]	19
ENSG00000179144	0,616368	4,402967	2,51E-05	0,000719	5,702014	protein_coding	GIMAP7	GTPase, IMAP family member 7 [Source:HGNC Symbol;Acc:HGNC:22404]	7
ENSG00000134910	7,322524	-1,041332	2,57E-05	0,000734	-5,690878	protein_coding	STT3A	STT3 oligosaccharyltransferase complex catalytic subunit A [Source:HGNC Symbol;Acc:HGNC:6172]	11
ENSG00000068912	6,698178	-0,967571	2,58E-05	0,000735	-5,688874	protein_coding	ERLEC1	endoplasmic reticulum lectin 1 [Source:HGNC Symbol;Acc:HGNC:25222]	2
ENSG00000147883	5,506685	-2,714616	2,59E-05	0,000736	-5,687309	protein_coding	CDKN2B	cyclin dependent kinase inhibitor 2B [Source:HGNC Symbol;Acc:HGNC:1788]	9
ENSG00000163297	6,535709	-2,560036	2,62E-05	0,000743	-5,681066	protein_coding	ANTXR2	ANTXR cell adhesion molecule 2 [Source:HGNC Symbol;Acc:HGNC:21732]	4
ENSG00000146215	-0,40848	2,993849	2,62E-05	0,000743	5,681619	protein_coding	CRIP3	cysteine rich protein 3 [Source:HGNC Symbol;Acc:HGNC:17751]	6
ENSG00000105974	5,588205	-2,802303	2,62E-05	0,000743	-5,680189	protein_coding	CAV1	caveolin 1 [Source:HGNC Symbol;Acc:HGNC:1527]	7
ENSG00000102878	3,116442	2,488223	2,63E-05	0,000744	5,678521	protein_coding	HSF4	heat shock transcription factor 4 [Source:HGNC Symbol;Acc:HGNC:5227]	16
ENSG00000101182	6,907901	-0,733223	2,64E-05	0,000746	-5,676518	protein_coding	PSMA7	proteasome 20S subunit alpha 7 [Source:HGNC Symbol;Acc:HGNC:9536]	20
ENSG00000163806	0,666265	2,293594	2,66E-05	0,00075	5,672972	protein_coding	SPDYA	speedy/RINGO cell cycle regulator family member A [Source:HGNC Symbol;Acc:HGNC:30613]	2
ENSG00000138069	6,746417	-1,047525	2,67E-05	0,000751	-5,67161	protein_coding	RAB1A	RAB1A, member RAS oncogene family [Source:HGNC Symbol;Acc:HGNC:9758]	2
ENSG00000023734	6,526054	-0,873921	2,68E-05	0,000752	-5,669719	protein_coding	STRAP	serine/threonine kinase receptor associated protein [Source:HGNC Symbol;Acc:HGNC:30796]	12
ENSG00000197857	4,134362	1,401969	2,69E-05	0,000754	5,667815	protein_coding	ZNF44	zinc finger protein 44 [Source:HGNC Symbol;Acc:HGNC:13110]	19
ENSG00000142694	3,814482	-2,445046	2,7E-05	0,000755	-5,665503	protein_coding	EVA1B	eva-1 homolog B [Source:HGNC Symbol;Acc:HGNC:25558]	1
ENSG00000108518	7,985287	-1,02377	2,7E-05	0,000755	-5,665457	protein_coding	PFN1	profilin 1 [Source:HGNC Symbol;Acc:HGNC:8881]	17
ENSG00000118508	3,783492	-1,648792	2,74E-05	0,000765	-5,658204	protein_coding	RAB32	RAB32, member RAS oncogene family [Source:HGNC Symbol;Acc:HGNC:9772]	6
ENSG00000170035	5,358417	-2,125636	2,76E-05	0,000768	-5,655214	protein_coding	UBE2E3	ubiquitin conjugating enzyme E2 E3 [Source:HGNC Symbol;Acc:HGNC:12479]	2
ENSG00000075884	0,017913	4,266012	2,77E-05	0,000771	5,652814	protein_coding	ARHGAP15	Rho GTPase activating protein 15 [Source:HGNC Symbol;Acc:HGNC:21030]	2
ENSG00000100335	5,684589	-0,658019	2,79E-05	0,000774	-5,649564	protein_coding	MIEF1	mitochondrial elongation factor 1 [Source:HGNC Symbol;Acc:HGNC:25979]	22
ENSG00000064601	7,395444	-1,186519	2,82E-05	0,000782	-5,644159	protein_coding	CTSA	cathepsin A [Source:HGNC Symbol;Acc:HGNC:9251]	20
ENSG00000174059	1,507959	5,76398	2,83E-05	0,000782	5,643175	protein_coding	CD34	CD34 molecule [Source:HGNC Symbol;Acc:HGNC:1662]	1
ENSG00000101901	5,208219	1,168944	2,84E-05	0,000783	5,641911	protein_coding	ALG13	ALG13 UDP-N-acetylglucosaminyltransferase subunit [Source:HGNC Symbol;Acc:HGNC:30881]	X
ENSG00000104164	5,686419	-0,790221	2,86E-05	0,000787	-5,638576	protein_coding	BLOC1S6	biogenesis of lysosomal organelles complex 1 subunit 6 [Source:HGNC Symbol;Acc:HGNC:8549]	15
ENSG00000067560	8,250737	-0,936833	2,88E-05	0,000791	-5,634857	protein_coding	RHOA	ras homolog family member A [Source:HGNC Symbol;Acc:HGNC:667]	3
ENSG00000198932	4,406154	2,880269	2,89E-05	0,000792	5,633337	protein_coding	GPRASP1	G protein-coupled receptor associated sorting protein 1 [Source:HGNC Symbol;Acc:HGNC:24834]	X
ENSG00000082781	7,245971	-2,328354	2,91E-05	0,000796	-5,630078	protein_coding	ITGB5	integrin subunit beta 5 [Source:HGNC Symbol;Acc:HGNC:6160]	3
ENSG00000140598	5,542663	-0,976439	2,91E-05	0,000797	-5,628831	protein_coding	EFL1	elongation factor like GTPase 1 [Source:HGNC Symbol;Acc:HGNC:25789]	15
ENSG00000136827	5,488137	-0,849558	2,94E-05	0,000804	-5,623775	protein_coding	TOR1A	torsin family 1 member A [Source:HGNC Symbol;Acc:HGNC:3098]	9
ENSG00000119537	5,959797	-0,991205	2,96E-05	0,000808	-5,620445	protein_coding	KDSR	3-ketodihydrosphingosine reductase [Source:HGNC Symbol;Acc:HGNC:4021]	18
ENSG00000063660	6,558338	-1,801447	2,98E-05	0,00081	-5,617441	protein_coding	GPC1	glypican 1 [Source:HGNC Symbol;Acc:HGNC:4449]	2
ENSG00000114554	7,628388	-0,965346	2,98E-05	0,00081	-5,617516	protein_coding	PLXNA1	plexin A1 [Source:HGNC Symbol;Acc:HGNC:9099]	3
ENSG00000102931	4,616999	-1,248096	3E-05	0,000811	-5,61423	protein_coding	ARL2BP	ADP ribosylation factor like GTPase 2 binding protein [Source:HGNC Symbol;Acc:HGNC:17146]	16
ENSG00000137502	5,01278	-1,52495	3E-05	0,000811	-5,613523	protein_coding	RAB30	RAB30, member RAS oncogene family [Source:HGNC Symbol;Acc:HGNC:9770]	11
ENSG00000213699	6,044024	-0,769863	3E-05	0,000811	-5,61407	protein_coding	SLC35F6	solute carrier family 35 member F6 [Source:HGNC Symbol;Acc:HGNC:26055]	2
ENSG00000177697	7,0293	-1,283977	3E-05	0,000811	-5,614647	protein_coding	CD151	CD151 molecule (Raph blood group) [Source:HGNC Symbol;Acc:HGNC:1630]	11
ENSG00000163577	3,751486	-1,341315	3,02E-05	0,000813	-5,611578	protein_coding	EIF5A2	eukaryotic translation initiation factor 5A2 [Source:HGNC Symbol;Acc:HGNC:3301]	3
ENSG00000103353	5,945593	-0,819969	3,03E-05	0,000815	-5,609373	protein_coding	UBFD1	ubiquitin family domain containing 1 [Source:HGNC Symbol;Acc:HGNC:30565]	16
ENSG00000107864	4,120387	1,157931	3,05E-05	0,000819	5,60659	protein_coding	CPEB3	cytoplasmic polyadenylation element binding protein 3 [Source:HGNC Symbol;Acc:HGNC:21746]	10
ENSG00000241685	4,124381	-1,250887	3,05E-05	0,000819	-5,605726	protein_coding	ARPC1A	actin related protein 2/3 complex subunit 1A [Source:HGNC Symbol;Acc:HGNC:703]	7
ENSG00000033011	4,016596	-0,940023	3,07E-05	0,000821	-5,603517	protein_coding	ALG1	ALG1 chitobiosyldiphosphodolichol beta-mannosyltransferase [Source:HGNC Symbol;Acc:HGNC:18294]	16
ENSG00000110321	9,668559	-0,913603	3,08E-05	0,000823	-5,600934	protein_coding	EIF4G2	eukaryotic translation initiation factor 4 gamma 2 [Source:HGNC Symbol;Acc:HGNC:3297]	11
ENSG00000101082	-1,180959	3,123382	3,08E-05	0,000823	5,60079	protein_coding	SLA2	Src like adaptor 2 [Source:HGNC Symbol;Acc:HGNC:17329]	20
ENSG00000164163	5,990698	-0,919965	3,09E-05	0,000824	-5,599034	protein_coding	ABCE1	ATP binding cassette subfamily E member 1 [Source:HGNC Symbol;Acc:HGNC:69]	4
ENSG00000258890	5,333967	1,049178	3,12E-05	0,000825	5,594567	protein_coding	CEP95	centrosomal protein 95 [Source:HGNC Symbol;Acc:HGNC:25141]	17

ENSG00000213281	5,587296	-1,132537	3,11E-05	0,000825	-5,596845	protein_coding	NRAS	NRAS proto-oncogene, GTPase [Source:HGNC Symbol;Acc:HGNC:7989]	1
ENSG00000151327	6,369387	-0,928539	3,11E-05	0,000825	-5,596286	protein_coding	FAM177A1	family with sequence similarity 177 member A1 [Source:HGNC Symbol;Acc:HGNC:19829]	14
ENSG00000188820	-2,230008	3,752925	3,12E-05	0,000825	5,594953	protein_coding	CALHM6	calcium homeostasis modulator family member 6 [Source:HGNC Symbol;Acc:HGNC:33391]	6
ENSG00000140527	-2,491667	3,083529	3,12E-05	0,000825	5,594796	protein_coding	WDR93	WD repeat domain 93 [Source:HGNC Symbol;Acc:HGNC:26924]	15
ENSG00000270882	0,638327	1,94459	3,17E-05	0,000835	5,587881	protein_coding	H4C14	H4 clustered histone 14 [Source:HGNC Symbol;Acc:HGNC:4794]	1
ENSG00000187688	4,557275	-2,388746	3,19E-05	0,000837	-5,584779	protein_coding	TRPV2	transient receptor potential cation channel subfamily V member 2 [Source:HGNC Symbol;Acc:HGNC:18082]	17
ENSG00000153214	5,888201	-1,237444	3,19E-05	0,000837	-5,584124	protein_coding	TMEM87B	transmembrane protein 87B [Source:HGNC Symbol;Acc:HGNC:25913]	2
ENSG00000188536	-2,212708	4,33384	3,19E-05	0,000837	5,584702	protein_coding	HBA2	hemoglobin subunit alpha 2 [Source:HGNC Symbol;Acc:HGNC:4824]	16
ENSG00000198223	0,817591	3,68535	3,2E-05	0,00084	5,581987	protein_coding	CSF2RA	colony stimulating factor 2 receptor subunit alpha [Source:HGNC Symbol;Acc:HGNC:2435]	X
ENSG00000169756	6,618536	-1,76437	3,22E-05	0,00084	-5,580142	protein_coding	LIMS1	LIM zinc finger domain containing 1 [Source:HGNC Symbol;Acc:HGNC:6616]	2
ENSG00000175857	-2,384718	4,254009	3,21E-05	0,00084	5,580927	protein_coding	GAPT	GRB2 binding adaptor protein, transmembrane [Source:HGNC Symbol;Acc:HGNC:26588]	5
ENSG00000049245	5,652892	-1,008192	3,25E-05	0,000847	-5,57517	protein_coding	VAMP3	vesicle associated membrane protein 3 [Source:HGNC Symbol;Acc:HGNC:12644]	1
ENSG00000232629	-1,501978	4,540204	3,26E-05	0,000849	5,573396	protein_coding	HLA-DQB2	major histocompatibility complex, class II, DQ beta 2 [Source:HGNC Symbol;Acc:HGNC:4945]	6
ENSG00000259207	4,208798	-3,12218	3,29E-05	0,000855	-5,568666	protein_coding	ITGB3	integrin subunit beta 3 [Source:HGNC Symbol;Acc:HGNC:6156]	17
ENSG00000167977	4,66234	-0,924403	3,29E-05	0,000855	-5,568941	protein_coding	KCTD5	potassium channel tetramerization domain containing 5 [Source:HGNC Symbol;Acc:HGNC:21423]	16
ENSG00000119397	5,633835	1,611578	3,32E-05	0,00086	5,564873	protein_coding	CNTRL	centriolin [Source:HGNC Symbol;Acc:HGNC:1858]	9
ENSG00000196923	6,330032	-1,387681	3,32E-05	0,00086	-5,564108	protein_coding	PDLIM7	PDZ and LIM domain 7 [Source:HGNC Symbol;Acc:HGNC:22958]	5
ENSG00000131981	5,712048	-2,309076	3,33E-05	0,000861	-5,562824	protein_coding	LGALS3	galectin 3 [Source:HGNC Symbol;Acc:HGNC:6563]	14
ENSG00000033050	3,657674	-0,909217	3,34E-05	0,000862	-5,561367	protein_coding	ABCF2	ATP binding cassette subfamily F member 2 [Source:HGNC Symbol;Acc:HGNC:71]	7
ENSG00000130508	8,384453	-3,085412	3,35E-05	0,000862	-5,560693	protein_coding	PXDN	peroxidasin [Source:HGNC Symbol;Acc:HGNC:14966]	2
ENSG00000136738	4,919417	-0,761815	3,36E-05	0,000863	-5,559199	protein_coding	STAM	signal transducing adaptor molecule [Source:HGNC Symbol;Acc:HGNC:11357]	10
ENSG00000100528	6,00164	-0,925447	3,36E-05	0,000863	-5,558668	protein_coding	CNIH1	cornichon family AMPA receptor auxiliary protein 1 [Source:HGNC Symbol;Acc:HGNC:19431]	14
ENSG00000157353	3,290574	1,164338	3,37E-05	0,000863	5,556822	protein_coding	FCSK	fucose kinase [Source:HGNC Symbol;Acc:HGNC:29500]	16
ENSG00000126067	5,511487	-0,959656	3,37E-05	0,000863	-5,556456	protein_coding	PSMB2	proteasome 20S subunit beta 2 [Source:HGNC Symbol;Acc:HGNC:9539]	1
ENSG00000144566	5,85014	-0,784686	3,38E-05	0,000863	-5,556296	protein_coding	RAB5A	RAB5A, member RAS oncogene family [Source:HGNC Symbol;Acc:HGNC:9783]	3
ENSG00000167525	1,779025	2,124046	3,39E-05	0,000865	5,554358	protein_coding	PROCA1	protein interacting with cyclin A1 [Source:HGNC Symbol;Acc:HGNC:28600]	17
ENSG00000172667	5,98189	-1,356131	3,41E-05	0,000867	-5,551829	protein_coding	ZMAT3	zinc finger matrin-type 3 [Source:HGNC Symbol;Acc:HGNC:29983]	3
ENSG00000174788	-1,056486	3,386773	3,41E-05	0,000867	5,551283	protein_coding	PCP2	Purkinje cell protein 2 [Source:HGNC Symbol;Acc:HGNC:30209]	19
ENSG00000165507	4,983903	4,184496	3,44E-05	0,000869	5,546573	protein_coding	DEPP1	DEPP1 autophagy regulator [Source:HGNC Symbol;Acc:HGNC:23355]	10
ENSG00000142621	1,841707	3,534228	3,45E-05	0,000869	5,545223	protein_coding	FHAD1	forkhead associated phosphopeptide binding domain 1 [Source:HGNC Symbol;Acc:HGNC:29408]	1
ENSG00000172500	4,915492	-0,656701	3,43E-05	0,000869	-5,548015	protein_coding	FIBP	FGF1 intracellular binding protein [Source:HGNC Symbol;Acc:HGNC:3705]	11
ENSG00000240184	5,228048	-1,489835	3,43E-05	0,000869	-5,5488	protein_coding	PCDHGC3	protocadherin gamma subfamily C, 3 [Source:HGNC Symbol;Acc:HGNC:8716]	5
ENSG00000154864	6,117771	-3,028653	3,44E-05	0,000869	-5,54744	protein_coding	PIEZO2	piezo type mechanosensitive ion channel component 2 [Source:HGNC Symbol;Acc:HGNC:26270]	18
ENSG00000205730	6,638242	-1,708192	3,45E-05	0,000869	-5,546334	protein_coding	ITPRIPL2	ITPRIP like 2 [Source:HGNC Symbol;Acc:HGNC:27257]	16
ENSG00000116005	6,538529	-1,055166	3,46E-05	0,000869	-5,544818	protein_coding	PCYOX1	prenylcysteine oxidase 1 [Source:HGNC Symbol;Acc:HGNC:20588]	2
ENSG00000151239	6,460418	-0,966247	3,46E-05	0,000869	-5,543656	protein_coding	TWF1	twinfilin actin binding protein 1 [Source:HGNC Symbol;Acc:HGNC:9620]	12
ENSG00000162747	-1,880824	5,144624	3,46E-05	0,000869	5,543691	protein_coding	FCGR3B	Fc fragment of IgG receptor IIIb [Source:HGNC Symbol;Acc:HGNC:3620]	1
ENSG00000154451	0,580011	4,344511	3,48E-05	0,000872	5,54097	protein_coding	GBP5	guanylate binding protein 5 [Source:HGNC Symbol;Acc:HGNC:19895]	1
ENSG00000196305	7,656272	-1,099401	3,49E-05	0,000873	-5,539715	protein_coding	IARS1	isoleucyl-tRNA synthetase 1 [Source:HGNC Symbol;Acc:HGNC:5330]	9
ENSG00000204767	1,088715	-5,185684	3,52E-05	0,00088	-5,535153	protein_coding	INSYN2B	inhibitory synaptic factor family member 2B [Source:HGNC Symbol;Acc:HGNC:37271]	5
ENSG00000254986	5,319079	-0,739628	3,53E-05	0,00088	-5,534529	protein_coding	DPP3	dipeptidyl peptidase 3 [Source:HGNC Symbol;Acc:HGNC:3008]	11
ENSG00000162367	-0,991023	4,091083	3,56E-05	0,000886	5,530094	protein_coding	TAL1	TAL bHLH transcription factor 1, erythroid differentiation factor [Source:HGNC Symbol;Acc:HGNC:11556]	1
ENSG00000101000	3,451094	-2,794119	3,59E-05	0,000892	-5,525951	protein_coding	PROCR	protein C receptor [Source:HGNC Symbol;Acc:HGNC:9452]	20
ENSG00000171634	8,358715	0,640001	3,6E-05	0,000893	5,525093	protein_coding	BPTF	bromodomain PHD finger transcription factor [Source:HGNC Symbol;Acc:HGNC:3581]	17
ENSG00000095370	1,98504	3,319294	3,63E-05	0,000899	5,520393	protein_coding	SH2D3C	SH2 domain containing 3C [Source:HGNC Symbol;Acc:HGNC:16884]	9
ENSG00000198060	5,005562	-0,846524	3,64E-05	0,000899	-5,519909	protein_coding	MARCHF5	membrane associated ring-CH-type finger 5 [Source:HGNC Symbol;Acc:HGNC:26025]	10
ENSG00000188690	4,90361	-0,812974	3,65E-05	0,000901	-5,518169	protein_coding	UROS	uroporphyrinogen III synthase [Source:HGNC Symbol;Acc:HGNC:12592]	10
ENSG00000150630	4,284609	-3,128199	3,67E-05	0,000904	-5,515704	protein_coding	VEGFC	vascular endothelial growth factor C [Source:HGNC Symbol;Acc:HGNC:12682]	4
ENSG00000005238	4,253707	-1,285204	3,67E-05	0,000905	-5,514788	protein_coding	FAM214B	family with sequence similarity 214 member B [Source:HGNC Symbol;Acc:HGNC:25666]	9
ENSG00000170248	7,010828	-0,898281	3,69E-05	0,000906	-5,513403	protein_coding	PDCD6IP	programmed cell death 6 interacting protein [Source:HGNC Symbol;Acc:HGNC:8766]	3
ENSG00000113575	6,413076	-0,742906	3,69E-05	0,000906	-5,51261	protein_coding	PPP2CA	protein phosphatase 2 catalytic subunit alpha [Source:HGNC Symbol;Acc:HGNC:9299]	5

ENSG00000085265	-0,738362	5,635246	3,7E-05	0,000907	5,511346	protein_coding	FCN1	ficolin 1 [Source:HGNC Symbol;Acc:HGNC:3623]	9
ENSG00000136240	7,192544	-1,148346	3,73E-05	0,000912	-5,507857	protein_coding	KDELR2	KDEL endoplasmic reticulum protein retention receptor 2 [Source:HGNC Symbol;Acc:HGNC:6305]	7
ENSG00000162669	-0,242487	2,777219	3,74E-05	0,000912	5,50635	protein_coding	HFM1	helicase for meiosis 1 [Source:HGNC Symbol;Acc:HGNC:20193]	1
ENSG00000143742	6,465864	-1,189597	3,74E-05	0,000912	-5,505599	protein_coding	SRP9	signal recognition particle 9 [Source:HGNC Symbol;Acc:HGNC:11304]	1
ENSG00000067167	7,082907	-1,066671	3,74E-05	0,000912	-5,506045	protein_coding	TRAM1	translocation associated membrane protein 1 [Source:HGNC Symbol;Acc:HGNC:20568]	8
ENSG00000101236	5,571335	-1,546092	3,76E-05	0,000913	-5,503904	protein_coding	RNF24	ring finger protein 24 [Source:HGNC Symbol;Acc:HGNC:13779]	20
ENSG00000249751	-1,17011	4,528224	3,76E-05	0,000913	5,504141	protein_coding	ECSCR	endothelial cell surface expressed chemotaxis and apoptosis regulator [Source:HGNC Symbol;Acc:HGNC:35454]	5
ENSG00000114850	7,474617	-1,40186	3,78E-05	0,000918	-5,500468	protein_coding	SSR3	signal sequence receptor subunit 3 [Source:HGNC Symbol;Acc:HGNC:11325]	3
ENSG00000168615	7,596219	-1,384207	3,79E-05	0,000918	-5,499369	protein_coding	ADAM9	ADAM metallopeptidase domain 9 [Source:HGNC Symbol;Acc:HGNC:216]	8
ENSG00000157045	4,052519	-1,430232	3,81E-05	0,000921	-5,497425	protein_coding	NTAN1	N-terminal asparagine amidase [Source:HGNC Symbol;Acc:HGNC:29909]	16
ENSG00000130311	5,264105	-0,880475	3,82E-05	0,000921	-5,496455	protein_coding	DDA1	DET1 and DDB1 associated 1 [Source:HGNC Symbol;Acc:HGNC:28360]	19
ENSG00000110880	7,122452	-1,197569	3,85E-05	0,000928	-5,492069	protein_coding	CORO1C	coronin 1C [Source:HGNC Symbol;Acc:HGNC:2254]	12
ENSG00000130589	5,998115	0,760948	3,87E-05	0,000932	5,489183	protein_coding	HELZ2	helicase with zinc finger 2 [Source:HGNC Symbol;Acc:HGNC:30021]	20
ENSG00000135404	8,500371	-1,245318	3,89E-05	0,000935	-5,486977	protein_coding	CD63	CD63 molecule [Source:HGNC Symbol;Acc:HGNC:1692]	12
ENSG00000123815	4,667853	1,219022	3,9E-05	0,000936	5,485996	protein_coding	COQ8B	coenzyme Q8B [Source:HGNC Symbol;Acc:HGNC:19041]	19
ENSG00000123610	2,929726	-4,687747	3,91E-05	0,000937	-5,48454	protein_coding	TNFAIP6	TNF alpha induced protein 6 [Source:HGNC Symbol;Acc:HGNC:11898]	2
ENSG00000127947	6,618583	-1,044266	3,92E-05	0,000938	-5,483184	protein_coding	PTPN12	protein tyrosine phosphatase non-receptor type 12 [Source:HGNC Symbol;Acc:HGNC:9645]	7
ENSG00000165028	1,534299	1,987778	3,94E-05	0,000942	5,480665	protein_coding	NIPSNAP3E	nipsnap homolog 3B [Source:HGNC Symbol;Acc:HGNC:23641]	9
ENSG00000171105	7,247757	1,676001	3,96E-05	0,000944	5,478688	protein_coding	INSR	insulin receptor [Source:HGNC Symbol;Acc:HGNC:6091]	19
ENSG00000168288	5,387866	-1,194608	4,02E-05	0,000957	-5,471207	protein_coding	MMADHC	metabolism of cobalamin associated D [Source:HGNC Symbol;Acc:HGNC:25221]	2
ENSG00000136010	5,463925	-2,187868	4,04E-05	0,000962	-5,467955	protein_coding	ALDH1L2	aldehyde dehydrogenase 1 family member L2 [Source:HGNC Symbol;Acc:HGNC:26777]	12
ENSG00000132622	0,155899	5,043851	4,05E-05	0,000963	5,466717	protein_coding	HSPA12B	heat shock protein family A (Hsp70) member 12B [Source:HGNC Symbol;Acc:HGNC:16193]	20
ENSG00000204520	3,819237	-2,091891	4,07E-05	0,000964	-5,465407	protein_coding	MICA	MHC class I polypeptide-related sequence A [Source:HGNC Symbol;Acc:HGNC:7090]	6
ENSG00000082196	1,723355	2,982254	4,07E-05	0,000965	5,464573	protein_coding	C1QTNF3	C1q and TNF related 3 [Source:HGNC Symbol;Acc:HGNC:14326]	5
ENSG00000288642	1,412649	-3,856777	4,08E-05	0,000965	-5,463703	protein_coding	CDR1	cerebellar degeneration related protein 1 [Source:HGNC Symbol;Acc:HGNC:1798]	X
ENSG00000184113	-0,028269	5,447718	4,13E-05	0,000975	5,457892	protein_coding	CLDN5	claudin 5 [Source:HGNC Symbol;Acc:HGNC:2047]	22
ENSG00000108387	2,312185	2,630834	4,15E-05	0,000976	5,45497	protein_coding	SEPTIN4	septin 4 [Source:HGNC Symbol;Acc:HGNC:9165]	17
ENSG00000106803	5,556223	-0,999735	4,14E-05	0,000976	-5,456048	protein_coding	SEC61B	SEC61 translocon subunit beta [Source:HGNC Symbol;Acc:HGNC:16993]	9
ENSG00000017260	6,811543	-0,663584	4,16E-05	0,000976	-5,454451	protein_coding	ATP2C1	ATPase secretory pathway Ca2+ transporting 1 [Source:HGNC Symbol;Acc:HGNC:13211]	3
ENSG00000094963	-1,43263	6,048878	4,16E-05	0,000976	5,454628	protein_coding	FMO2	flavin containing dimethylaniline monoxygenase 2 [Source:HGNC Symbol;Acc:HGNC:3770]	1
ENSG00000137496	3,558528	1,523604	4,19E-05	0,000982	5,450434	protein_coding	IL18BP	interleukin 18 binding protein [Source:HGNC Symbol;Acc:HGNC:5987]	11
ENSG00000204305	1,454627	2,084166	4,19E-05	0,000982	5,450309	protein_coding	AGER	advanced glycosylation end-product specific receptor [Source:HGNC Symbol;Acc:HGNC:320]	6
ENSG00000167996	10,62426	-2,20048	4,23E-05	0,00099	-5,445537	protein_coding	FTH1	ferritin heavy chain 1 [Source:HGNC Symbol;Acc:HGNC:3976]	11
ENSG00000063438	0,652996	-2,961839	4,26E-05	0,000993	-5,442728	protein_coding	AHRR	aryl-hydrocarbon receptor repressor [Source:HGNC Symbol;Acc:HGNC:346]	5
ENSG00000116489	6,390868	-1,328347	4,26E-05	0,000993	-5,443029	protein_coding	CAPZA1	capping actin protein of muscle Z-line subunit alpha 1 [Source:HGNC Symbol;Acc:HGNC:1488]	1
ENSG00000179094	5,593631	2,36507	4,28E-05	0,000997	5,440196	protein_coding	PER1	period circadian regulator 1 [Source:HGNC Symbol;Acc:HGNC:8845]	17
ENSG00000085231	3,602888	-0,889928	4,29E-05	0,000997	-5,439235	protein_coding	AK6	adenylate kinase 6 [Source:HGNC Symbol;Acc:HGNC:49151]	5
ENSG00000106688	3,360802	-2,878888	4,3E-05	0,000997	-5,438498	protein_coding	SLC1A1	solute carrier family 1 member 1 [Source:HGNC Symbol;Acc:HGNC:10939]	9
ENSG00000112139	4,011421	-2,234959	4,32E-05	0,000998	-5,43558	protein_coding	MDGA1	MAM domain containing glycosylphosphatidylinositol anchor 1 [Source:HGNC Symbol;Acc:HGNC:19267]	6
ENSG00000104081	5,306759	-2,211324	4,32E-05	0,000998	-5,435766	protein_coding	BMF	Bcl2 modifying factor [Source:HGNC Symbol;Acc:HGNC:24132]	15
ENSG00000134590	6,066137	-1,114452	4,32E-05	0,000998	-5,435626	protein_coding	RTL8C	retrotransposon Gag like 8C [Source:HGNC Symbol;Acc:HGNC:2569]	X
ENSG00000100097	6,685876	-2,122869	4,31E-05	0,000998	-5,437371	protein_coding	LGALS1	galectin 1 [Source:HGNC Symbol;Acc:HGNC:6561]	22
ENSG00000114383	3,892688	-0,969956	4,34E-05	0,000999	-5,433971	protein_coding	TUSC2	tumor suppressor 2, mitochondrial calcium regulator [Source:HGNC Symbol;Acc:HGNC:17034]	3
ENSG00000143418	6,945806	-0,882219	4,34E-05	0,000999	-5,433381	protein_coding	CERS2	ceramide synthase 2 [Source:HGNC Symbol;Acc:HGNC:14076]	1
ENSG00000155099	3,935665	-1,174558	4,4E-05	0,001009	-5,427092	protein_coding	PIP4P2	phosphatidylinositol-4,5-bisphosphate 4-phosphatase 2 [Source:HGNC Symbol;Acc:HGNC:25452]	8
ENSG00000125870	5,28512	-0,845807	4,4E-05	0,001009	-5,427424	protein_coding	SNRPB2	small nuclear ribonucleoprotein polypeptide B2 [Source:HGNC Symbol;Acc:HGNC:11155]	20
ENSG00000148677	1,322954	-3,568095	4,42E-05	0,001011	-5,425074	protein_coding	ANKRD1	ankyrin repeat domain 1 [Source:HGNC Symbol;Acc:HGNC:15819]	10
ENSG00000169016	3,658676	-1,018544	4,43E-05	0,001011	-5,423707	protein_coding	E2F6	E2F transcription factor 6 [Source:HGNC Symbol;Acc:HGNC:3120]	2
ENSG00000186994	0,869948	3,319186	4,43E-05	0,001011	5,423751	protein_coding	KANK3	KN motif and ankyrin repeat domains 3 [Source:HGNC Symbol;Acc:HGNC:24796]	19
ENSG00000099985	-1,098345	4,397317	4,41E-05	0,001011	5,425248	protein_coding	OSM	oncostatin M [Source:HGNC Symbol;Acc:HGNC:8506]	22
ENSG00000004961	4,37977	-0,899648	4,44E-05	0,001011	-5,422906	protein_coding	HCCS	holocytochrome c synthase [Source:HGNC Symbol;Acc:HGNC:4837]	X

ENSG00000163110	7,409143	-1,343422	4,46E-05	0,001015	-5,420482	protein_coding	PDLIM5	PDZ and LIM domain 5 [Source:HGNC Symbol;Acc:HGNC:17468]	4
ENSG00000203883	0,709859	4,227911	4,49E-05	0,001018	5,41684	protein_coding	SOX18	SRY-box transcription factor 18 [Source:HGNC Symbol;Acc:HGNC:11194]	20
ENSG00000149929	4,322587	0,998882	4,51E-05	0,001018	5,415323	protein_coding	HIRIP3	HIRA interacting protein 3 [Source:HGNC Symbol;Acc:HGNC:4917]	16
ENSG00000113273	5,311471	-1,290846	4,51E-05	0,001018	-5,415023	protein_coding	ARSB	arylsulfatase B [Source:HGNC Symbol;Acc:HGNC:714]	5
ENSG00000109814	6,570495	-1,735324	4,5E-05	0,001018	-5,416154	protein_coding	UGDH	UDP-glucose 6-dehydrogenase [Source:HGNC Symbol;Acc:HGNC:12525]	4
ENSG00000105246	-2,120687	3,279192	4,49E-05	0,001018	5,416709	protein_coding	EBI3	Epstein-Barr virus induced 3 [Source:HGNC Symbol;Acc:HGNC:3129]	19
ENSG00000188282	-2,359772	3,53756	4,48E-05	0,001018	5,417726	protein_coding	RUFY4	RUN and FYVE domain containing 4 [Source:HGNC Symbol;Acc:HGNC:24804]	2
ENSG00000117395	5,713318	-0,795384	4,53E-05	0,001021	-5,412739	protein_coding	EBNA1BP2	EBNA1 binding protein 2 [Source:HGNC Symbol;Acc:HGNC:15531]	1
ENSG00000187513	0,123823	5,311914	4,54E-05	0,001022	5,411446	protein_coding	GJA4	gap junction protein alpha 4 [Source:HGNC Symbol;Acc:HGNC:4278]	1
ENSG00000172264	3,072134	2,562378	4,56E-05	0,001025	5,409304	protein_coding	MACROD2	mono-ADP ribosylhydrolase 2 [Source:HGNC Symbol;Acc:HGNC:16126]	20
ENSG00000100351	0,506793	2,795064	4,61E-05	0,001032	5,403953	protein_coding	GRAP2	GRB2 related adaptor protein 2 [Source:HGNC Symbol;Acc:HGNC:4563]	22
ENSG00000111145	5,569007	-1,770192	4,61E-05	0,001032	-5,404029	protein_coding	ELK3	ETS transcription factor ELK3 [Source:HGNC Symbol;Acc:HGNC:3325]	12
ENSG00000087116	7,167894	-1,908847	4,61E-05	0,001032	-5,404483	protein_coding	ADAMTS2	ADAM metallopeptidase with thrombospondin type 1 motif 2 [Source:HGNC Symbol;Acc:HGNC:218]	5
ENSG00000130653	4,278033	2,497416	4,62E-05	0,001033	5,402921	protein_coding	PNPLA7	patatin like phospholipase domain containing 7 [Source:HGNC Symbol;Acc:HGNC:24768]	9
ENSG00000206561	1,54191	2,18721	4,68E-05	0,001043	5,396578	protein_coding	COLQ	collagen like tail subunit of asymmetric acetylcholinesterase [Source:HGNC Symbol;Acc:HGNC:2226]	3
ENSG00000138381	3,515961	-0,957197	4,68E-05	0,001043	-5,396627	protein_coding	ASNSD1	asparagine synthetase domain containing 1 [Source:HGNC Symbol;Acc:HGNC:24910]	2
ENSG00000137101	0,706393	3,482203	4,69E-05	0,001043	5,39611	protein_coding	CD72	CD72 molecule [Source:HGNC Symbol;Acc:HGNC:1696]	9
ENSG00000100522	4,588554	-1,373958	4,69E-05	0,001043	-5,395518	protein_coding	GNPNAT1	glucosamine-phosphate N-acetyltransferase 1 [Source:HGNC Symbol;Acc:HGNC:19980]	14
ENSG00000147133	6,808508	1,047513	4,71E-05	0,001045	5,39401	protein_coding	TAF1	TATA-box binding protein associated factor 1 [Source:HGNC Symbol;Acc:HGNC:11535]	X
ENSG00000106952	-1,462918	4,325878	4,73E-05	0,001048	5,392226	protein_coding	TNFSF8	TNF superfamily member 8 [Source:HGNC Symbol;Acc:HGNC:11938]	9
ENSG00000124802	2,54825	-1,319428	4,82E-05	0,001066	-5,382094	protein_coding	EEF1E1	eukaryotic translation elongation factor 1 epsilon 1 [Source:HGNC Symbol;Acc:HGNC:3212]	6
ENSG00000013725	0,37172	3,448067	4,82E-05	0,001066	5,382526	protein_coding	CD6	CD6 molecule [Source:HGNC Symbol;Acc:HGNC:1691]	11
ENSG00000103035	6,518472	-0,797814	4,83E-05	0,001066	-5,381739	protein_coding	PSMD7	proteasome 26S subunit, non-ATPase 7 [Source:HGNC Symbol;Acc:HGNC:9565]	16
ENSG00000164761	4,687017	-5,038699	4,84E-05	0,001067	-5,380642	protein_coding	TNFRSF11E	TNF receptor superfamily member 11b [Source:HGNC Symbol;Acc:HGNC:11909]	8
ENSG00000110906	6,239974	-0,990135	4,87E-05	0,001073	-5,377432	protein_coding	KCTD10	potassium channel tetramerization domain containing 10 [Source:HGNC Symbol;Acc:HGNC:23236]	12
ENSG00000184313	-1,906201	3,520023	4,89E-05	0,001076	5,375092	protein_coding	MROH7	maestro heat like repeat family member 7 [Source:HGNC Symbol;Acc:HGNC:24802]	1
ENSG00000117152	5,135364	-2,849816	4,92E-05	0,001078	-5,372881	protein_coding	RGS4	regulator of G protein signaling 4 [Source:HGNC Symbol;Acc:HGNC:10000]	1
ENSG00000065308	6,554605	-1,425876	4,91E-05	0,001078	-5,373126	protein_coding	TRAM2	translocation associated membrane protein 2 [Source:HGNC Symbol;Acc:HGNC:16855]	6
ENSG00000142227	4,130895	-2,232978	4,93E-05	0,001079	-5,371751	protein_coding	EMP3	epithelial membrane protein 3 [Source:HGNC Symbol;Acc:HGNC:3335]	19
ENSG00000158186	4,98277	-2,235871	4,94E-05	0,00108	-5,370654	protein_coding	MRAS	muscle RAS oncogene homolog [Source:HGNC Symbol;Acc:HGNC:7227]	3
ENSG00000109063	1,847985	2,044651	4,96E-05	0,001083	5,368773	protein_coding	MYH3	myosin heavy chain 3 [Source:HGNC Symbol;Acc:HGNC:7573]	17
ENSG00000058453	5,584527	1,193706	5E-05	0,00109	5,364858	protein_coding	CROCC	ciliary rootlet coiled-coil, rootletin [Source:HGNC Symbol;Acc:HGNC:21299]	1
ENSG00000066056	0,915804	5,600794	5,09E-05	0,001108	5,356259	protein_coding	TIE1	tyrosine kinase with immunoglobulin like and EGF like domains 1 [Source:HGNC Symbol;Acc:HGNC:11809]	1
ENSG00000050165	6,413553	-2,246344	5,13E-05	0,001116	-5,352229	protein_coding	DKK3	dickkopf WNT signaling pathway inhibitor 3 [Source:HGNC Symbol;Acc:HGNC:2893]	11
ENSG00000196218	1,111069	4,416517	5,2E-05	0,00113	5,345512	protein_coding	RYR1	ryanodine receptor 1 [Source:HGNC Symbol;Acc:HGNC:10483]	19
ENSG00000041357	6,246649	-1,008735	5,22E-05	0,001133	-5,343564	protein_coding	PSMA4	proteasome 20S subunit alpha 4 [Source:HGNC Symbol;Acc:HGNC:9533]	15
ENSG00000204301	2,813923	3,670868	5,23E-05	0,001134	5,342721	protein_coding	NOTCH4	notch receptor 4 [Source:HGNC Symbol;Acc:HGNC:7884]	6
ENSG00000184730	2,331079	2,743006	5,24E-05	0,001134	5,34187	protein_coding	APOBR	apolipoprotein B receptor [Source:HGNC Symbol;Acc:HGNC:24087]	16
ENSG00000198380	7,874527	-0,898917	5,28E-05	0,001142	-5,337979	protein_coding	GFPT1	glutamine--fructose-6-phosphate transaminase 1 [Source:HGNC Symbol;Acc:HGNC:4241]	2
ENSG00000011258	4,919724	0,999298	5,3E-05	0,001144	5,336645	protein_coding	MBTD1	mbt domain containing 1 [Source:HGNC Symbol;Acc:HGNC:19866]	17
ENSG00000179271	6,164984	-0,758475	5,31E-05	0,001144	-5,335723	protein_coding	GADD45GI	GADD45G interacting protein 1 [Source:HGNC Symbol;Acc:HGNC:29996]	19
ENSG00000164244	6,308927	-0,747369	5,33E-05	0,001146	-5,334213	protein_coding	PRRC1	proline rich coiled-coil 1 [Source:HGNC Symbol;Acc:HGNC:28164]	5
ENSG00000150687	6,918879	-2,195744	5,34E-05	0,001147	-5,333127	protein_coding	PRSS23	serine protease 23 [Source:HGNC Symbol;Acc:HGNC:14370]	11
ENSG00000136250	1,206311	3,335278	5,35E-05	0,001148	5,332272	protein_coding	AOAH	acyloxyacyl hydrolase [Source:HGNC Symbol;Acc:HGNC:548]	7
ENSG00000112419	6,853085	-1,496018	5,38E-05	0,001153	-5,329454	protein_coding	PHACTR2	phosphatase and actin regulator 2 [Source:HGNC Symbol;Acc:HGNC:20956]	6
ENSG00000122376	4,822001	-0,903756	5,41E-05	0,001158	-5,326625	protein_coding	SHLD2	shieldin complex subunit 2 [Source:HGNC Symbol;Acc:HGNC:28773]	10
ENSG00000179295	7,783552	-0,8589	5,43E-05	0,00116	-5,325263	protein_coding	PTPN11	protein tyrosine phosphatase non-receptor type 11 [Source:HGNC Symbol;Acc:HGNC:9644]	12
ENSG00000213918	4,705552	1,32673	5,44E-05	0,001161	5,324177	protein_coding	DNASE1	deoxyribonuclease 1 [Source:HGNC Symbol;Acc:HGNC:2956]	16
ENSG00000184575	7,289658	-0,94317	5,47E-05	0,001166	-5,321358	protein_coding	XPOT	exportin for tRNA [Source:HGNC Symbol;Acc:HGNC:12826]	12
ENSG00000196405	6,046496	1,928087	5,49E-05	0,00117	5,319281	protein_coding	EVL	Enah/Vasp-like [Source:HGNC Symbol;Acc:HGNC:20234]	14
ENSG00000176853	6,651922	-1,023107	5,52E-05	0,001174	-5,316956	protein_coding	FAM91A1	family with sequence similarity 91 member A1 [Source:HGNC Symbol;Acc:HGNC:26306]	8

ENSG00000198908	-1,852259	2,790856	5,53E-05	0,001175	5,315898	protein_coding	BHLHB9	basic helix-loop-helix family member b9 [Source:HGNC Symbol;Acc:HGNC:29353]	X
ENSG00000115128	4,93322	-1,325471	5,57E-05	0,001181	-5,312804	protein_coding	SF3B6	splicing factor 3b subunit 6 [Source:HGNC Symbol;Acc:HGNC:30096]	2
ENSG00000260916	5,038578	-1,380818	5,59E-05	0,001183	-5,310777	protein_coding	CCPG1	cell cycle progression 1 [Source:HGNC Symbol;Acc:HGNC:24227]	15
ENSG00000255152	-1,636379	3,094715	5,58E-05	0,001183	5,311224	protein_coding	MSH5-SAP(	MSH5-SAPCD1 readthrough (NMD candidate) [Source:HGNC Symbol;Acc:HGNC:41994]	6
ENSG00000166913	7,986976	-0,704642	5,61E-05	0,001185	-5,309433	protein_coding	YWHAB	tyrosine 3-monooxygenase/tryptophan 5-monooxygenase activation protein beta [Source:HGNC Symbol;Acc:HGNC:	20
ENSG00000170681	2,044697	-1,917532	5,63E-05	0,001185	-5,307467	protein_coding	CAVIN4	caveolae associated protein 4 [Source:HGNC Symbol;Acc:HGNC:33742]	9
ENSG00000154265	6,082896	1,982542	5,62E-05	0,001185	5,307846	protein_coding	ABCA5	ATP binding cassette subfamily A member 5 [Source:HGNC Symbol;Acc:HGNC:35]	17
ENSG00000132514	-1,425498	4,73729	5,62E-05	0,001185	5,30825	protein_coding	CLEC10A	C-type lectin domain containing 10A [Source:HGNC Symbol;Acc:HGNC:16916]	17
ENSG00000131467	6,64772	-0,707932	5,65E-05	0,001187	-5,305873	protein_coding	PSME3	proteasome activator subunit 3 [Source:HGNC Symbol;Acc:HGNC:9570]	17
ENSG00000118496	5,309586	-0,958821	5,69E-05	0,001195	-5,302147	protein_coding	FBXO30	F-box protein 30 [Source:HGNC Symbol;Acc:HGNC:15600]	6
ENSG00000204310	6,166459	-0,875606	5,7E-05	0,001196	-5,301135	protein_coding	AGPAT1	1-acylglycerol-3-phosphate O-acyltransferase 1 [Source:HGNC Symbol;Acc:HGNC:324]	6
ENSG00000163564	-2,607407	3,600201	5,72E-05	0,001199	5,299263	protein_coding	PYHIN1	pyrin and HIN domain family member 1 [Source:HGNC Symbol;Acc:HGNC:28894]	1
ENSG00000154582	4,556013	-0,992878	5,76E-05	0,001204	-5,296598	protein_coding	ELOC	elongin C [Source:HGNC Symbol;Acc:HGNC:11617]	8
ENSG00000266412	7,021097	-0,98925	5,78E-05	0,001208	-5,294403	protein_coding	NCOA4	nuclear receptor coactivator 4 [Source:HGNC Symbol;Acc:HGNC:7671]	10
ENSG00000112599	0,100921	2,141308	5,79E-05	0,001208	5,293726	protein_coding	GUCA1B	guanylate cyclase activator 1B [Source:HGNC Symbol;Acc:HGNC:4679]	6
ENSG00000186635	7,049611	0,591279	5,8E-05	0,001208	5,293159	protein_coding	ARAP1	ArfGAP with RhoGAP domain, ankyrin repeat and PH domain 1 [Source:HGNC Symbol;Acc:HGNC:16925]	11
ENSG00000163814	6,268396	-1,532814	5,81E-05	0,001208	-5,292322	protein_coding	CDCP1	CUB domain containing protein 1 [Source:HGNC Symbol;Acc:HGNC:24357]	3
ENSG00000182712	-0,900345	-2,450296	5,83E-05	0,00121	-5,290499	protein_coding	CMC4	C-X9-C motif containing 4 [Source:HGNC Symbol;Acc:HGNC:35428]	X
ENSG00000105366	-2,059183	3,477308	5,83E-05	0,00121	5,29058	protein_coding	SIGLEC8	sialic acid binding Ig like lectin 8 [Source:HGNC Symbol;Acc:HGNC:10877]	19
ENSG00000119979	3,83455	-0,9041	5,85E-05	0,00121	-5,289175	protein_coding	DENND10	DENN domain containing 10 [Source:HGNC Symbol;Acc:HGNC:31793]	10
ENSG00000140090	-1,153402	3,735398	5,85E-05	0,00121	5,289003	protein_coding	SLC24A4	solute carrier family 24 member 4 [Source:HGNC Symbol;Acc:HGNC:10978]	14
ENSG00000124203	-1,059484	5,141928	5,86E-05	0,00121	5,288338	protein_coding	ZNF831	zinc finger protein 831 [Source:HGNC Symbol;Acc:HGNC:16167]	20
ENSG00000156642	7,170812	-0,856426	5,86E-05	0,00121	-5,288035	protein_coding	NPTN	neuroplastin [Source:HGNC Symbol;Acc:HGNC:17867]	15
ENSG00000042286	4,483234	-1,038761	5,88E-05	0,001213	-5,286174	protein_coding	AIFM2	apoptosis inducing factor mitochondria associated 2 [Source:HGNC Symbol;Acc:HGNC:21411]	10
ENSG00000146112	6,539542	-1,588556	5,92E-05	0,001219	-5,283287	protein_coding	PPP1R18	protein phosphatase 1 regulatory subunit 18 [Source:HGNC Symbol;Acc:HGNC:29413]	6
ENSG00000178562	-1,074911	4,855997	5,94E-05	0,001221	5,281718	protein_coding	CD28	CD28 molecule [Source:HGNC Symbol;Acc:HGNC:1653]	2
ENSG00000089847	1,381976	2,375745	5,97E-05	0,001226	5,279313	protein_coding	ANKRD24	ankyrin repeat domain 24 [Source:HGNC Symbol;Acc:HGNC:29424]	19
ENSG00000135046	6,541915	-2,665453	5,97E-05	0,001226	-5,278744	protein_coding	ANXA1	annexin A1 [Source:HGNC Symbol;Acc:HGNC:533]	9
ENSG00000050820	6,657849	-1,207287	6,03E-05	0,001236	-5,274206	protein_coding	BCAR1	BCAR1 scaffold protein, Cas family member [Source:HGNC Symbol;Acc:HGNC:971]	16
ENSG00000181847	-1,411861	4,109823	6,04E-05	0,001236	5,273248	protein_coding	TIGIT	T cell immunoreceptor with Ig and ITIM domains [Source:HGNC Symbol;Acc:HGNC:26838]	3
ENSG00000136986	5,626381	-0,913962	6,06E-05	0,001238	-5,271872	protein_coding	DERL1	derlin 1 [Source:HGNC Symbol;Acc:HGNC:28454]	8
ENSG00000155097	6,054615	-0,808105	6,07E-05	0,00124	-5,27085	protein_coding	ATP6V1C1	ATPase H+ transporting V1 subunit C1 [Source:HGNC Symbol;Acc:HGNC:856]	8
ENSG00000146192	1,772262	3,686921	6,11E-05	0,001243	5,268108	protein_coding	FGD2	FYVE, RhoGEF and PH domain containing 2 [Source:HGNC Symbol;Acc:HGNC:3664]	6
ENSG00000068831	0,590424	3,507797	6,11E-05	0,001243	5,268204	protein_coding	RASGRP2	RAS guanyl releasing protein 2 [Source:HGNC Symbol;Acc:HGNC:9879]	11
ENSG00000162144	4,75367	-1,375622	6,12E-05	0,001244	-5,267264	protein_coding	CYB561A3	cytochrome b561 family member A3 [Source:HGNC Symbol;Acc:HGNC:23014]	11
ENSG00000206172	-1,969522	4,078245	6,15E-05	0,001249	5,264892	protein_coding	HBA1	hemoglobin subunit alpha 1 [Source:HGNC Symbol;Acc:HGNC:4823]	16
ENSG00000184381	4,175253	1,792256	6,19E-05	0,001251	5,261702	protein_coding	PLA2G6	phospholipase A2 group VI [Source:HGNC Symbol;Acc:HGNC:9039]	22
ENSG00000116824	-1,032503	3,925425	6,19E-05	0,001251	5,261773	protein_coding	CD2	CD2 molecule [Source:HGNC Symbol;Acc:HGNC:1639]	1
ENSG00000105887	6,774758	-0,977703	6,19E-05	0,001251	-5,261541	protein_coding	MTPN	myotrophin [Source:HGNC Symbol;Acc:HGNC:15667]	7
ENSG00000172543	-2,087078	3,552686	6,18E-05	0,001251	5,262092	protein_coding	CTSW	cathepsin W [Source:HGNC Symbol;Acc:HGNC:2546]	11
ENSG00000111215	-1,358299	2,887878	6,21E-05	0,001253	5,259689	protein_coding	PRR4	proline rich 4 [Source:HGNC Symbol;Acc:HGNC:18020]	12
ENSG00000272333	7,215041	0,856911	6,22E-05	0,001253	5,25948	protein_coding	KMT2B	lysine methyltransferase 2B [Source:HGNC Symbol;Acc:HGNC:15840]	19
ENSG00000197019	3,653693	-1,583738	6,24E-05	0,001255	-5,257945	protein_coding	SERTAD1	SERTA domain containing 1 [Source:HGNC Symbol;Acc:HGNC:17932]	19
ENSG00000131355	-2,601201	3,203953	6,24E-05	0,001255	5,257504	protein_coding	ADGRE3	adhesion G protein-coupled receptor E3 [Source:HGNC Symbol;Acc:HGNC:23647]	19
ENSG00000185163	4,655748	0,866656	6,26E-05	0,001256	5,256218	protein_coding	DDX51	DEAD-box helicase 51 [Source:HGNC Symbol;Acc:HGNC:20082]	12
ENSG00000108829	7,260378	-1,072318	6,26E-05	0,001256	-5,25626	protein_coding	LRRC59	leucine rich repeat containing 59 [Source:HGNC Symbol;Acc:HGNC:28817]	17
ENSG00000147443	0,286082	3,243776	6,3E-05	0,001261	5,253097	protein_coding	DOK2	docking protein 2 [Source:HGNC Symbol;Acc:HGNC:2991]	8
ENSG00000196535	7,497022	1,158032	6,29E-05	0,001261	5,253683	protein_coding	MYO18A	myosin XVIIIA [Source:HGNC Symbol;Acc:HGNC:31104]	17
ENSG00000115944	5,273843	-0,76287	6,39E-05	0,001277	-5,246127	protein_coding	COX7A2L	cytochrome c oxidase subunit 7A2 like [Source:HGNC Symbol;Acc:HGNC:2289]	2
ENSG00000283632	0,699946	3,585549	6,4E-05	0,001278	5,245452	protein_coding	EXOC3L2	exocyst complex component 3 like 2 [Source:HGNC Symbol;Acc:HGNC:30162]	19
ENSG00000258102	0,901291	-1,71147	6,43E-05	0,00128	-5,242893	protein_coding	MAP1LC3B	microtubule associated protein 1 light chain 3 beta 2 [Source:HGNC Symbol;Acc:HGNC:34390]	12

ENSG00000171132	4,972008	1,163543	6,42E-05	0,00128	5,243842	protein_coding	PRKCE	protein kinase C epsilon [Source:HGNC Symbol;Acc:HGNC:9401]	2
ENSG00000160654	-1,466189	3,353868	6,44E-05	0,00128	5,242339	protein_coding	CD3G	CD3g molecule [Source:HGNC Symbol;Acc:HGNC:1675]	11
ENSG00000243156	6,292471	1,239157	6,43E-05	0,00128	5,243157	protein_coding	MICAL3	microtubule associated monooxygenase, calponin and LIM domain containing 3 [Source:HGNC Symbol;Acc:HGNC:2	22
ENSG00000213347	3,110037	1,92093	6,48E-05	0,001286	5,239289	protein_coding	MXD3	MAX dimerization protein 3 [Source:HGNC Symbol;Acc:HGNC:14008]	5
ENSG00000006007	5,692794	-1,208812	6,5E-05	0,001287	-5,238373	protein_coding	GDE1	glycerophosphodiester phosphodiesterase 1 [Source:HGNC Symbol;Acc:HGNC:29644]	16
ENSG00000138755	-1,43612	5,98861	6,5E-05	0,001287	5,237952	protein_coding	CXCL9	C-X-C motif chemokine ligand 9 [Source:HGNC Symbol;Acc:HGNC:7098]	4
ENSG00000167601	6,295411	-2,236306	6,52E-05	0,001288	-5,236619	protein_coding	AXL	AXL receptor tyrosine kinase [Source:HGNC Symbol;Acc:HGNC:905]	19
ENSG00000134539	-1,570839	3,414606	6,53E-05	0,001288	5,235625	protein_coding	KLRD1	killer cell lectin like receptor D1 [Source:HGNC Symbol;Acc:HGNC:6378]	12
ENSG00000125878	-2,014477	3,510116	6,53E-05	0,001288	5,235839	protein_coding	TCF15	transcription factor 15 [Source:HGNC Symbol;Acc:HGNC:11627]	20
ENSG00000113108	3,499215	1,1806	6,56E-05	0,001293	5,233265	protein_coding	APBB3	amyloid beta precursor protein binding family B member 3 [Source:HGNC Symbol;Acc:HGNC:20708]	5
ENSG00000122958	5,316955	-0,999478	6,57E-05	0,001293	-5,232609	protein_coding	VPS26A	VPS26, retromer complex component A [Source:HGNC Symbol;Acc:HGNC:12711]	10
ENSG00000173369	4,738825	3,392642	6,61E-05	0,001297	5,229604	protein_coding	C1QB	complement C1q B chain [Source:HGNC Symbol;Acc:HGNC:1242]	1
ENSG00000097033	7,09415	-1,120448	6,61E-05	0,001297	-5,229803	protein_coding	SH3GLB1	SH3 domain containing GRB2 like, endophilin B1 [Source:HGNC Symbol;Acc:HGNC:10833]	1
ENSG00000171049	-2,179666	3,931211	6,61E-05	0,001297	5,230005	protein_coding	FPR2	formyl peptide receptor 2 [Source:HGNC Symbol;Acc:HGNC:3827]	19
ENSG00000169230	6,191635	-1,233283	6,63E-05	0,001298	-5,228444	protein_coding	PRELID1	PRELI domain containing 1 [Source:HGNC Symbol;Acc:HGNC:30255]	5
ENSG00000066455	6,27954	-0,781824	6,64E-05	0,001299	-5,227589	protein_coding	GOLGA5	golgin A5 [Source:HGNC Symbol;Acc:HGNC:4428]	14
ENSG00000197119	4,986904	1,595673	6,69E-05	0,001306	5,224341	protein_coding	SLC25A29	solute carrier family 25 member 29 [Source:HGNC Symbol;Acc:HGNC:20116]	14
ENSG00000167632	6,27479	1,013283	6,74E-05	0,001313	5,220913	protein_coding	TRAPPC9	trafficking protein particle complex 9 [Source:HGNC Symbol;Acc:HGNC:30832]	8
ENSG00000136603	7,225285	-1,439347	6,74E-05	0,001313	-5,220662	protein_coding	SKIL	SKI like proto-oncogene [Source:HGNC Symbol;Acc:HGNC:10897]	3
ENSG00000282936	1,533382	2,09875	6,75E-05	0,001314	5,219606	protein_coding	AC004706.	novel protein	17
ENSG00000164587	7,604839	1,410023	6,76E-05	0,001314	5,218952	protein_coding	RPS14	ribosomal protein S14 [Source:HGNC Symbol;Acc:HGNC:10387]	5
ENSG00000181830	5,2044	-1,011804	6,79E-05	0,001318	-5,216999	protein_coding	SLC35C1	solute carrier family 35 member C1 [Source:HGNC Symbol;Acc:HGNC:20197]	11
ENSG00000108469	4,656268	1,045498	6,81E-05	0,00132	5,215889	protein_coding	RECQL5	RecQ like helicase 5 [Source:HGNC Symbol;Acc:HGNC:9950]	17
ENSG00000111845	4,181277	-1,061165	6,83E-05	0,001322	-5,214138	protein_coding	PAK1IP1	PAK1 interacting protein 1 [Source:HGNC Symbol;Acc:HGNC:20882]	6
ENSG00000152082	5,405447	0,876237	6,83E-05	0,001322	5,213845	protein_coding	MZT2B	mitotic spindle organizing protein 2B [Source:HGNC Symbol;Acc:HGNC:25886]	2
ENSG00000126458	4,138392	-1,746371	6,86E-05	0,001326	-5,211792	protein_coding	RRAS	RAS related [Source:HGNC Symbol;Acc:HGNC:10447]	19
ENSG00000136021	6,229044	-0,768939	6,89E-05	0,00133	-5,209802	protein_coding	SCYL2	SCY1 like pseudokinase 2 [Source:HGNC Symbol;Acc:HGNC:19286]	12
ENSG00000189409	-1,707494	3,351705	6,9E-05	0,001331	5,208937	protein_coding	MMP23B	matrix metallopeptidase 23B [Source:HGNC Symbol;Acc:HGNC:7171]	1
ENSG00000139211	5,186836	-2,612686	6,92E-05	0,001332	-5,208	protein_coding	AMIGO2	adhesion molecule with Ig like domain 2 [Source:HGNC Symbol;Acc:HGNC:24073]	12
ENSG00000109790	5,759122	-1,878082	6,93E-05	0,001332	-5,207534	protein_coding	KLHL5	kelch like family member 5 [Source:HGNC Symbol;Acc:HGNC:6356]	4
ENSG00000107863	6,81705	-1,16889	6,93E-05	0,001332	-5,206909	protein_coding	ARHGAP21	Rho GTPase activating protein 21 [Source:HGNC Symbol;Acc:HGNC:23725]	10
ENSG00000120949	-2,0149	3,633437	6,97E-05	0,001337	5,20445	protein_coding	TNFRSF8	TNF receptor superfamily member 8 [Source:HGNC Symbol;Acc:HGNC:11923]	1
ENSG00000198898	6,580896	-0,748914	6,99E-05	0,00134	-5,202951	protein_coding	CAPZA2	capping actin protein of muscle Z-line subunit alpha 2 [Source:HGNC Symbol;Acc:HGNC:1490]	7
ENSG00000119899	4,775728	-1,487879	7,02E-05	0,001343	-5,201213	protein_coding	SLC17A5	solute carrier family 17 member 5 [Source:HGNC Symbol;Acc:HGNC:10933]	6
ENSG00000249992	3,052992	-5,369457	7,1E-05	0,001357	-5,195807	protein_coding	TMEM158	transmembrane protein 158 [Source:HGNC Symbol;Acc:HGNC:30293]	3
ENSG00000198668	8,738282	-0,804749	7,1E-05	0,001357	-5,19524	protein_coding	CALM1	calmodulin 1 [Source:HGNC Symbol;Acc:HGNC:1442]	14
ENSG00000004700	6,17239	-1,227707	7,12E-05	0,001358	-5,19409	protein_coding	RECQL	RecQ like helicase [Source:HGNC Symbol;Acc:HGNC:9948]	12
ENSG00000206199	-2,7549	3,81482	7,19E-05	0,00137	5,189536	protein_coding	ANKUB1	ankyrin repeat and ubiquitin domain containing 1 [Source:HGNC Symbol;Acc:HGNC:29642]	3
ENSG00000173992	3,885293	1,014282	7,21E-05	0,00137	5,188276	protein_coding	CCS	copper chaperone for superoxide dismutase [Source:HGNC Symbol;Acc:HGNC:1613]	11
ENSG00000117090	-2,167981	3,43287	7,21E-05	0,00137	5,188366	protein_coding	SLAMF1	signaling lymphocytic activation molecule family member 1 [Source:HGNC Symbol;Acc:HGNC:10903]	1
ENSG00000102699	7,135219	-0,980905	7,23E-05	0,001372	-5,186772	protein_coding	PARP4	poly(ADP-ribose) polymerase family member 4 [Source:HGNC Symbol;Acc:HGNC:271]	13
ENSG00000163106	-2,289426	3,385427	7,24E-05	0,001372	5,186342	protein_coding	HPGDS	hematopoietic prostaglandin D synthase [Source:HGNC Symbol;Acc:HGNC:17890]	4
ENSG00000090659	-0,332364	4,217115	7,26E-05	0,001375	5,184992	protein_coding	CD209	CD209 molecule [Source:HGNC Symbol;Acc:HGNC:1641]	19
ENSG00000173269	1,997914	4,335047	7,31E-05	0,001383	5,181329	protein_coding	MMRN2	multimerin 2 [Source:HGNC Symbol;Acc:HGNC:19888]	10
ENSG00000188643	4,449779	-2,909756	7,36E-05	0,001392	-5,177971	protein_coding	S100A16	S100 calcium binding protein A16 [Source:HGNC Symbol;Acc:HGNC:20441]	1
ENSG00000133574	1,752521	4,326246	7,41E-05	0,001398	5,175281	protein_coding	GIMAP4	GTPase, IMAP family member 4 [Source:HGNC Symbol;Acc:HGNC:21872]	7
ENSG00000164932	5,491128	-2,206189	7,45E-05	0,001402	-5,172344	protein_coding	CTHRC1	collagen triple helix repeat containing 1 [Source:HGNC Symbol;Acc:HGNC:18831]	8
ENSG00000072110	9,003919	-1,029188	7,45E-05	0,001402	-5,172367	protein_coding	ACTN1	actinin alpha 1 [Source:HGNC Symbol;Acc:HGNC:163]	14
ENSG00000102024	7,010164	-1,550764	7,45E-05	0,001402	-5,172198	protein_coding	PLS3	plastin 3 [Source:HGNC Symbol;Acc:HGNC:9091]	X
ENSG00000131386	-0,126126	4,118348	7,48E-05	0,001405	5,170729	protein_coding	GALNT15	polypeptide N-acetylgalactosaminyltransferase 15 [Source:HGNC Symbol;Acc:HGNC:21531]	3
ENSG00000175505	2,097289	-2,794811	7,5E-05	0,001407	-5,169494	protein_coding	CLCF1	cardiotrophin like cytokine factor 1 [Source:HGNC Symbol;Acc:HGNC:17412]	11

ENSG00000067798	5,430596	-2,277467	7,51E-05	0,001407	-5,168698	protein_coding	NAV3	neuron navigator 3 [Source:HGNC Symbol;Acc:HGNC:15998]	12
ENSG00000125304	7,711538	-0,761365	7,58E-05	0,001419	-5,164042	protein_coding	TM9SF2	transmembrane 9 superfamily member 2 [Source:HGNC Symbol;Acc:HGNC:11865]	13
ENSG00000129515	5,889366	-0,790681	7,6E-05	0,001421	-5,162824	protein_coding	SNX6	sorting nexin 6 [Source:HGNC Symbol;Acc:HGNC:14970]	14
ENSG00000111615	6,252503	-0,683709	7,61E-05	0,001421	-5,162554	protein_coding	KRR1	KRR1 small subunit processome component homolog [Source:HGNC Symbol;Acc:HGNC:5176]	12
ENSG00000155307	1,509863	3,323211	7,71E-05	0,001437	5,155891	protein_coding	SAMSN1	SAM domain, SH3 domain and nuclear localization signals 1 [Source:HGNC Symbol;Acc:HGNC:10528]	21
ENSG00000070214	6,864866	-1,118831	7,71E-05	0,001437	-5,156075	protein_coding	SLC44A1	solute carrier family 44 member 1 [Source:HGNC Symbol;Acc:HGNC:18798]	9
ENSG00000151247	5,037227	-0,92913	7,78E-05	0,001449	-5,151495	protein_coding	EIF4E	eukaryotic translation initiation factor 4E [Source:HGNC Symbol;Acc:HGNC:3287]	4
ENSG00000115756	5,197307	-1,618242	7,8E-05	0,00145	-5,150442	protein_coding	HPCAL1	hippocalcin like 1 [Source:HGNC Symbol;Acc:HGNC:5145]	2
ENSG00000172943	5,988214	0,863315	7,82E-05	0,001453	5,14903	protein_coding	PHF8	PHD finger protein 8 [Source:HGNC Symbol;Acc:HGNC:20672]	X
ENSG00000136378	4,927047	-2,503082	7,84E-05	0,001454	-5,148202	protein_coding	ADAMTS7	ADAM metallopeptidase with thrombospondin type 1 motif 7 [Source:HGNC Symbol;Acc:HGNC:223]	15
ENSG00000149201	-0,325601	2,305032	7,88E-05	0,001458	5,145675	protein_coding	CCDC81	coiled-coil domain containing 81 [Source:HGNC Symbol;Acc:HGNC:26281]	11
ENSG00000189171	4,724729	-1,560771	7,88E-05	0,001458	-5,145455	protein_coding	S100A13	S100 calcium binding protein A13 [Source:HGNC Symbol;Acc:HGNC:10490]	1
ENSG00000023191	6,278072	-0,884999	7,88E-05	0,001458	-5,145249	protein_coding	RNH1	ribonuclease/angiogenin inhibitor 1 [Source:HGNC Symbol;Acc:HGNC:10074]	11
ENSG00000188869	-3,106857	2,431891	7,93E-05	0,001463	5,142814	protein_coding	TMC3	transmembrane channel like 3 [Source:HGNC Symbol;Acc:HGNC:22995]	15
ENSG00000213341	5,431614	-0,791457	7,94E-05	0,001464	-5,142101	protein_coding	CHUK	component of inhibitor of nuclear factor kappa B kinase complex [Source:HGNC Symbol;Acc:HGNC:1974]	10
ENSG00000133561	0,809623	4,405045	8E-05	0,001474	5,138262	protein_coding	GIMAP6	GTPase, IMAP family member 6 [Source:HGNC Symbol;Acc:HGNC:21918]	7
ENSG00000119681	8,429387	-2,672912	8,03E-05	0,001477	-5,136711	protein_coding	LTBP2	latent transforming growth factor beta binding protein 2 [Source:HGNC Symbol;Acc:HGNC:6715]	14
ENSG00000113384	6,880452	-0,692797	8,04E-05	0,001479	-5,135639	protein_coding	GOLPH3	golgi phosphoprotein 3 [Source:HGNC Symbol;Acc:HGNC:15452]	5
ENSG00000161940	0,878895	4,496361	8,06E-05	0,001479	5,134666	protein_coding	BCL6B	BCL6B transcription repressor [Source:HGNC Symbol;Acc:HGNC:1002]	17
ENSG00000141367	9,441858	-0,961712	8,06E-05	0,001479	-5,134484	protein_coding	CLTC	clathrin heavy chain [Source:HGNC Symbol;Acc:HGNC:2092]	17
ENSG00000147138	-2,107355	3,738371	8,11E-05	0,001485	5,131962	protein_coding	GPR174	G protein-coupled receptor 174 [Source:HGNC Symbol;Acc:HGNC:30245]	X
ENSG00000100150	3,911695	1,179354	8,14E-05	0,001485	5,130157	protein_coding	DEPDC5	DEP domain containing 5, GATOR1 subcomplex subunit [Source:HGNC Symbol;Acc:HGNC:18423]	22
ENSG00000163913	5,074255	1,000055	8,13E-05	0,001485	5,130293	protein_coding	IFT122	intraflagellar transport 122 [Source:HGNC Symbol;Acc:HGNC:13556]	3
ENSG00000171867	6,579166	-1,997777	8,14E-05	0,001485	-5,129794	protein_coding	PRNP	prion protein [Source:HGNC Symbol;Acc:HGNC:9449]	20
ENSG00000039560	7,027217	-1,195463	8,12E-05	0,001485	-5,131135	protein_coding	RAI14	retinoic acid induced 14 [Source:HGNC Symbol;Acc:HGNC:14873]	5
ENSG00000197006	6,080535	-0,925234	8,16E-05	0,001486	-5,128921	protein_coding	METTL9	methyltransferase like 9 [Source:HGNC Symbol;Acc:HGNC:24586]	16
ENSG00000064703	4,478855	-0,933302	8,17E-05	0,001487	-5,128078	protein_coding	DDX20	DEAD-box helicase 20 [Source:HGNC Symbol;Acc:HGNC:2743]	1
ENSG00000120509	3,968007	-0,926317	8,22E-05	0,001493	-5,125567	protein_coding	PDZD11	PDZ domain containing 11 [Source:HGNC Symbol;Acc:HGNC:28034]	X
ENSG00000214029	4,123093	1,599872	8,25E-05	0,001497	5,12373	protein_coding	ZNF891	zinc finger protein 891 [Source:HGNC Symbol;Acc:HGNC:38709]	12
ENSG00000138623	4,088378	-3,037056	8,27E-05	0,0015	-5,12242	protein_coding	SEMA7A	semaphorin 7A (John Milton Hagen blood group) [Source:HGNC Symbol;Acc:HGNC:10741]	15
ENSG00000196689	2,018424	1,58325	8,3E-05	0,001501	5,120937	protein_coding	TRPV1	transient receptor potential cation channel subfamily V member 1 [Source:HGNC Symbol;Acc:HGNC:12716]	17
ENSG00000204252	0,724195	4,656133	8,29E-05	0,001501	5,121302	protein_coding	HLA-DOA	major histocompatibility complex, class II, DO alpha [Source:HGNC Symbol;Acc:HGNC:4936]	6
ENSG00000197816	1,217615	2,111985	8,35E-05	0,001509	5,118064	protein_coding	CCDC180	coiled-coil domain containing 180 [Source:HGNC Symbol;Acc:HGNC:29303]	9
ENSG00000130684	4,84368	1,397066	8,4E-05	0,001516	5,115098	protein_coding	ZNF337	zinc finger protein 337 [Source:HGNC Symbol;Acc:HGNC:15809]	20
ENSG00000103510	4,875715	0,873408	8,45E-05	0,001524	5,112183	protein_coding	KAT8	lysine acetyltransferase 8 [Source:HGNC Symbol;Acc:HGNC:17933]	16
ENSG00000175352	2,643093	-2,39554	8,48E-05	0,001525	-5,110259	protein_coding	NRIP3	nuclear receptor interacting protein 3 [Source:HGNC Symbol;Acc:HGNC:1167]	11
ENSG00000119688	5,429848	0,960429	8,46E-05	0,001525	5,111304	protein_coding	ABCD4	ATP binding cassette subfamily D member 4 [Source:HGNC Symbol;Acc:HGNC:68]	14
ENSG00000115561	5,390742	-0,712943	8,48E-05	0,001525	-5,1105	protein_coding	CHMP3	charged multivesicular body protein 3 [Source:HGNC Symbol;Acc:HGNC:29865]	2
ENSG00000162695	5,898231	-1,040445	8,52E-05	0,00153	-5,10815	protein_coding	SLC30A7	solute carrier family 30 member 7 [Source:HGNC Symbol;Acc:HGNC:19306]	1
ENSG00000129235	3,962131	-0,923625	8,55E-05	0,001534	-5,106296	protein_coding	TXNDC17	thioredoxin domain containing 17 [Source:HGNC Symbol;Acc:HGNC:28218]	17
ENSG00000145649	-1,344081	3,815951	8,58E-05	0,001536	5,105053	protein_coding	GZMA	granzyme A [Source:HGNC Symbol;Acc:HGNC:4708]	5
ENSG00000103152	4,476277	-1,108096	8,62E-05	0,00154	-5,102656	protein_coding	MPG	N-methylpurine DNA glycosylase [Source:HGNC Symbol;Acc:HGNC:7211]	16
ENSG00000151929	5,836291	-1,403605	8,63E-05	0,00154	-5,102237	protein_coding	BAG3	BAG cochaperone 3 [Source:HGNC Symbol;Acc:HGNC:939]	10
ENSG00000159377	5,998504	-0,781112	8,61E-05	0,00154	-5,103067	protein_coding	PSMB4	proteasome 20S subunit beta 4 [Source:HGNC Symbol;Acc:HGNC:9541]	1
ENSG00000075213	4,947442	-2,14085	8,67E-05	0,001545	-5,099567	protein_coding	SEMA3A	semaphorin 3A [Source:HGNC Symbol;Acc:HGNC:10723]	7
ENSG00000285444	-1,696795	2,382577	8,68E-05	0,001545	5,099399	protein_coding	AL162377.:	novel transcript	13
ENSG00000107731	6,851408	-1,749271	8,67E-05	0,001545	-5,099614	protein_coding	UNC5B	unc-5 netrin receptor B [Source:HGNC Symbol;Acc:HGNC:12568]	10
ENSG00000178860	3,682884	-4,078944	8,72E-05	0,001547	-5,096922	protein_coding	MSC	musculin [Source:HGNC Symbol;Acc:HGNC:7321]	8
ENSG00000120265	5,693353	-0,920977	8,71E-05	0,001547	-5,097781	protein_coding	PCMT1	protein-L-isoaspartate (D-aspartate) O-methyltransferase [Source:HGNC Symbol;Acc:HGNC:8728]	6
ENSG00000136238	7,462949	-1,153403	8,71E-05	0,001547	-5,097361	protein_coding	RAC1	Rac family small GTPase 1 [Source:HGNC Symbol;Acc:HGNC:9801]	7
ENSG00000173588	4,413799	1,026729	8,74E-05	0,001548	5,095769	protein_coding	CEP83	centrosomal protein 83 [Source:HGNC Symbol;Acc:HGNC:17966]	12

ENSG00000142186	6,659035	-0,860798	8,74E-05	0,001548	-5,095826	protein_coding	SCYL1	SCY1 like pseudokinase 1 [Source:HGNC Symbol;Acc:HGNC:14372]	11
ENSG00000204136	-0,499605	3,830205	8,9E-05	0,001574	5,087335	protein_coding	GGTA1	glycoprotein alpha-galactosyltransferase 1 (inactive) [Source:HGNC Symbol;Acc:HGNC:4253]	9
ENSG00000171115	1,464682	3,80401	8,92E-05	0,001576	5,086132	protein_coding	GIMAP8	GTPase, IMAP family member 8 [Source:HGNC Symbol;Acc:HGNC:21792]	7
ENSG00000184922	3,990238	2,120071	8,96E-05	0,001581	5,084204	protein_coding	FMNL1	formin like 1 [Source:HGNC Symbol;Acc:HGNC:1212]	17
ENSG00000140961	2,69365	-1,820548	9,03E-05	0,001592	-5,080161	protein_coding	OSGIN1	oxidative stress induced growth inhibitor 1 [Source:HGNC Symbol;Acc:HGNC:30093]	16
ENSG00000141295	4,231545	1,262641	9,05E-05	0,001593	5,079281	protein_coding	SCRN2	secernin 2 [Source:HGNC Symbol;Acc:HGNC:30381]	17
ENSG00000163170	3,659884	-1,115581	9,08E-05	0,001596	-5,07796	protein_coding	BOLA3	bolA family member 3 [Source:HGNC Symbol;Acc:HGNC:24415]	2
ENSG00000127884	5,679248	-0,618635	9,12E-05	0,001601	-5,075885	protein_coding	ECHS1	enoyl-CoA hydratase, short chain 1 [Source:HGNC Symbol;Acc:HGNC:3151]	10
ENSG00000197170	6,415591	-0,694069	9,12E-05	0,001601	-5,075503	protein_coding	PSMD12	proteasome 26S subunit, non-ATPase 12 [Source:HGNC Symbol;Acc:HGNC:9557]	17
ENSG00000113263	-0,572135	3,426886	9,14E-05	0,001601	5,074379	protein_coding	ITK	IL2 inducible T cell kinase [Source:HGNC Symbol;Acc:HGNC:6171]	5
ENSG00000197461	5,761992	-2,018296	9,14E-05	0,001601	-5,074569	protein_coding	PDGFA	platelet derived growth factor subunit A [Source:HGNC Symbol;Acc:HGNC:8799]	7
ENSG00000184903	2,717587	1,721772	9,23E-05	0,001615	5,069697	protein_coding	IMMP2L	inner mitochondrial membrane peptidase subunit 2 [Source:HGNC Symbol;Acc:HGNC:14598]	7
ENSG00000099840	0,135652	2,257944	9,26E-05	0,001618	5,06843	protein_coding	IZUMO4	IZUMO family member 4 [Source:HGNC Symbol;Acc:HGNC:26950]	19
ENSG00000165527	6,895383	-0,677425	9,27E-05	0,001619	-5,067688	protein_coding	ARF6	ADP ribosylation factor 6 [Source:HGNC Symbol;Acc:HGNC:659]	14
ENSG00000167394	3,41636	-0,839735	9,3E-05	0,001622	-5,066091	protein_coding	ZNF668	zinc finger protein 668 [Source:HGNC Symbol;Acc:HGNC:25821]	16
ENSG00000067141	6,762408	-1,204475	9,35E-05	0,001628	-5,063934	protein_coding	NEO1	neogenin 1 [Source:HGNC Symbol;Acc:HGNC:7754]	15
ENSG00000103150	4,638217	1,155399	9,36E-05	0,001628	5,06334	protein_coding	MLYCD	malonyl-CoA decarboxylase [Source:HGNC Symbol;Acc:HGNC:7150]	16
ENSG00000072182	-1,356447	3,364224	9,47E-05	0,001645	5,057937	protein_coding	ASIC4	acid sensing ion channel subunit family member 4 [Source:HGNC Symbol;Acc:HGNC:21263]	2
ENSG00000119013	3,88011	-1,058709	9,51E-05	0,00165	-5,055875	protein_coding	NDUFB3	NADH:ubiquinone oxidoreductase subunit B3 [Source:HGNC Symbol;Acc:HGNC:7698]	2
ENSG00000127663	6,631611	0,953682	9,52E-05	0,001652	5,05502	protein_coding	KDM4B	lysine demethylase 4B [Source:HGNC Symbol;Acc:HGNC:29136]	19
ENSG00000176692	3,019302	-3,475695	9,66E-05	0,001673	-5,04832	protein_coding	FOXC2	forkhead box C2 [Source:HGNC Symbol;Acc:HGNC:3801]	16
ENSG00000100804	5,696628	-0,933427	9,68E-05	0,001675	-5,047221	protein_coding	PSMB5	proteasome 20S subunit beta 5 [Source:HGNC Symbol;Acc:HGNC:9542]	14
ENSG00000173113	5,11737	-1,040933	9,73E-05	0,001682	-5,044905	protein_coding	TRMT112	tRNA methyltransferase subunit 11-2 [Source:HGNC Symbol;Acc:HGNC:26940]	11
ENSG00000154813	4,7629	-1,02722	9,76E-05	0,001685	-5,043175	protein_coding	DPH3	diphthamide biosynthesis 3 [Source:HGNC Symbol;Acc:HGNC:27717]	3
ENSG00000198625	6,330796	1,295512	9,78E-05	0,001685	5,042428	protein_coding	MDM4	MDM4 regulator of p53 [Source:HGNC Symbol;Acc:HGNC:6974]	1
ENSG00000213719	7,491815	-1,283138	9,77E-05	0,001685	-5,042903	protein_coding	CLIC1	chloride intracellular channel 1 [Source:HGNC Symbol;Acc:HGNC:2062]	6
ENSG00000100385	0,563211	2,857825	9,79E-05	0,001686	5,041747	protein_coding	IL2RB	interleukin 2 receptor subunit beta [Source:HGNC Symbol;Acc:HGNC:6009]	22
ENSG00000123505	6,20863	-0,869152	9,85E-05	0,001694	-5,038902	protein_coding	AMD1	adenosylmethionine decarboxylase 1 [Source:HGNC Symbol;Acc:HGNC:457]	6
ENSG00000164111	7,872788	-1,564522	9,87E-05	0,001695	-5,038004	protein_coding	ANXA5	annexin A5 [Source:HGNC Symbol;Acc:HGNC:543]	4
ENSG00000173705	2,155699	-2,424161	9,89E-05	0,001697	-5,037011	protein_coding	SUSD5	sushi domain containing 5 [Source:HGNC Symbol;Acc:HGNC:29061]	3
ENSG00000163599	-1,494818	4,207309	9,91E-05	0,001699	5,035955	protein_coding	CTLA4	cytotoxic T-lymphocyte associated protein 4 [Source:HGNC Symbol;Acc:HGNC:2505]	2
ENSG00000178597	-2,479762	-3,432428	9,92E-05	0,001699	-5,03552	protein_coding	PSAPL1	prosaposin like 1 [Source:HGNC Symbol;Acc:HGNC:33131]	4
ENSG00000159685	3,25313	1,226086	9,96E-05	0,001702	5,03349	protein_coding	CHCHD6	coiled-coil-helix-coiled-coil-helix domain containing 6 [Source:HGNC Symbol;Acc:HGNC:28184]	3
ENSG00000100030	7,185251	-0,698209	9,96E-05	0,001702	-5,033576	protein_coding	MAPK1	mitogen-activated protein kinase 1 [Source:HGNC Symbol;Acc:HGNC:6871]	22
ENSG00000116649	5,874693	-1,231594	0,0001	0,00171	-5,030878	protein_coding	SRM	spermidine synthase [Source:HGNC Symbol;Acc:HGNC:11296]	1
ENSG00000070010	5,925147	-0,725022	0,000101	0,001725	-5,025919	protein_coding	UFD1	ubiquitin recognition factor in ER associated degradation 1 [Source:HGNC Symbol;Acc:HGNC:12520]	22
ENSG00000109572	6,686569	-1,292307	0,000101	0,001725	-5,02571	protein_coding	CLCN3	chloride voltage-gated channel 3 [Source:HGNC Symbol;Acc:HGNC:2021]	4
ENSG00000196449	3,447404	-1,199767	0,000102	0,001729	-5,023732	protein_coding	YRDC	yrdC N6-threonylcarbamoyltransferase domain containing [Source:HGNC Symbol;Acc:HGNC:28905]	1
ENSG00000074706	-0,69216	3,66949	0,000102	0,001729	5,024112	protein_coding	IPCEF1	interaction protein for cytohesin exchange factors 1 [Source:HGNC Symbol;Acc:HGNC:21204]	6
ENSG00000187634	3,049767	-3,026534	0,000103	0,001744	-5,019107	protein_coding	SAMD11	sterile alpha motif domain containing 11 [Source:HGNC Symbol;Acc:HGNC:28706]	1
ENSG00000106366	8,792585	-3,049617	0,000103	0,001744	-5,018577	protein_coding	SERPINE1	serpin family E member 1 [Source:HGNC Symbol;Acc:HGNC:8583]	7
ENSG00000065054	6,053951	-1,147922	0,000103	0,001752	-5,015726	protein_coding	SLC9A3R2	SLC9A3 regulator 2 [Source:HGNC Symbol;Acc:HGNC:11076]	16
ENSG00000166928	-0,635471	3,959818	0,000104	0,001755	5,01462	protein_coding	MS4A14	membrane spanning 4-domains A14 [Source:HGNC Symbol;Acc:HGNC:30706]	11
ENSG00000158683	0,010944	2,725886	0,000104	0,001755	5,013595	protein_coding	PKD1L1	polycystin 1 like 1, transient receptor potential channel interacting [Source:HGNC Symbol;Acc:HGNC:18053]	7
ENSG00000102978	5,290719	-0,658989	0,000104	0,001755	-5,013553	protein_coding	POLR2C	RNA polymerase II subunit C [Source:HGNC Symbol;Acc:HGNC:9189]	16
ENSG00000112276	2,367068	-1,933737	0,000105	0,001768	-5,008123	protein_coding	BVES	blood vessel epicardial substance [Source:HGNC Symbol;Acc:HGNC:1152]	6
ENSG00000155926	1,927244	4,444034	0,000105	0,001768	5,008517	protein_coding	SLA	Src like adaptor [Source:HGNC Symbol;Acc:HGNC:10902]	8
ENSG00000079150	3,576026	-2,104228	0,000105	0,001768	-5,00836	protein_coding	FKBP7	FKBP prolyl isomerase 7 [Source:HGNC Symbol;Acc:HGNC:3723]	2
ENSG00000151553	5,322529	-0,942088	0,000105	0,001768	-5,007583	protein_coding	FAM160B1	family with sequence similarity 160 member B1 [Source:HGNC Symbol;Acc:HGNC:29320]	10
ENSG00000024422	6,421953	-2,205595	0,000105	0,001768	-5,007539	protein_coding	EHD2	EH domain containing 2 [Source:HGNC Symbol;Acc:HGNC:3243]	19
ENSG00000175787	1,832504	1,425208	0,000106	0,001773	5,005756	protein_coding	ZNF169	zinc finger protein 169 [Source:HGNC Symbol;Acc:HGNC:12957]	9

ENSG00000141429	6,829716	-1,558057	0,000106	0,001777	-5,004296	protein_coding	GALNT1	polypeptide N-acetylgalactosaminyltransferase 1 [Source:HGNC Symbol;Acc:HGNC:4123]	18
ENSG00000151846	1,520175	1,7556	0,000107	0,001788	5,000456	protein_coding	PABPC3	poly(A) binding protein cytoplasmic 3 [Source:HGNC Symbol;Acc:HGNC:8556]	13
ENSG00000163516	5,382237	1,266895	0,000107	0,001788	5,000147	protein_coding	ANKZF1	ankyrin repeat and zinc finger peptidyl tRNA hydrolase 1 [Source:HGNC Symbol;Acc:HGNC:25527]	2
ENSG00000100744	4,046352	-1,065477	0,000107	0,00179	-4,998644	protein_coding	GSKIP	GSK3B interacting protein [Source:HGNC Symbol;Acc:HGNC:20343]	14
ENSG00000168282	5,709619	-0,725641	0,000107	0,00179	-4,999027	protein_coding	MGAT2	alpha-1,6-mannosyl-glycoprotein 2-beta-N-acetylglucosaminyltransferase [Source:HGNC Symbol;Acc:HGNC:7045]	14
ENSG00000152778	4,536068	-1,077237	0,000108	0,001808	-4,993408	protein_coding	IFIT5	interferon induced protein with tetratricopeptide repeats 5 [Source:HGNC Symbol;Acc:HGNC:13328]	10
ENSG00000167614	-1,810653	3,788957	0,000109	0,001818	4,990387	protein_coding	TTYH1	tweety family member 1 [Source:HGNC Symbol;Acc:HGNC:13476]	19
ENSG00000100346	-0,51759	3,710413	0,000109	0,00182	4,989419	protein_coding	CACNA1I	calcium voltage-gated channel subunit alpha1 I [Source:HGNC Symbol;Acc:HGNC:1396]	22
ENSG00000183813	-2,160481	4,414632	0,00011	0,001826	4,987232	protein_coding	CCR4	C-C motif chemokine receptor 4 [Source:HGNC Symbol;Acc:HGNC:1605]	3
ENSG00000170759	8,684066	-0,869239	0,00011	0,001826	-4,986772	protein_coding	KIF5B	kinesin family member 5B [Source:HGNC Symbol;Acc:HGNC:6324]	10
ENSG00000111271	4,589795	0,982144	0,00011	0,001827	4,986082	protein_coding	ACAD10	acyl-CoA dehydrogenase family member 10 [Source:HGNC Symbol;Acc:HGNC:21597]	12
ENSG00000104904	7,753547	-0,750729	0,00011	0,001829	-4,985202	protein_coding	OAZ1	ornithine decarboxylase antizyme 1 [Source:HGNC Symbol;Acc:HGNC:8095]	19
ENSG00000163636	5,963078	-0,843299	0,000111	0,001839	-4,981682	protein_coding	PSMD6	proteasome 26S subunit, non-ATPase 6 [Source:HGNC Symbol;Acc:HGNC:9564]	3
ENSG00000152672	-3,227913	3,126725	0,000111	0,001839	4,981433	protein_coding	CLEC4F	C-type lectin domain family 4 member F [Source:HGNC Symbol;Acc:HGNC:25357]	2
ENSG00000131019	2,353626	-1,903266	0,000112	0,001841	-4,978601	protein_coding	ULBP3	UL16 binding protein 3 [Source:HGNC Symbol;Acc:HGNC:14895]	6
ENSG00000113732	5,749886	-0,973504	0,000112	0,001841	-4,979147	protein_coding	ATP6V0E1	ATPase H+ transporting V0 subunit e1 [Source:HGNC Symbol;Acc:HGNC:863]	5
ENSG00000138834	6,550562	0,884744	0,000111	0,001841	4,980132	protein_coding	MAPK8IP3	mitogen-activated protein kinase 8 interacting protein 3 [Source:HGNC Symbol;Acc:HGNC:6884]	16
ENSG00000146830	6,786273	0,966353	0,000112	0,001841	4,978861	protein_coding	GIGYF1	GRB10 interacting GYF protein 1 [Source:HGNC Symbol;Acc:HGNC:9126]	7
ENSG00000099991	7,023386	0,685756	0,000112	0,001841	4,979893	protein_coding	CABIN1	calcineurin binding protein 1 [Source:HGNC Symbol;Acc:HGNC:24187]	22
ENSG00000178685	5,360788	0,765712	0,000112	0,001843	4,977677	protein_coding	PARP10	poly(ADP-ribose) polymerase family member 10 [Source:HGNC Symbol;Acc:HGNC:25895]	8
ENSG00000151491	6,9147	-1,725423	0,000112	0,001844	-4,976882	protein_coding	EPS8	epidermal growth factor receptor pathway substrate 8 [Source:HGNC Symbol;Acc:HGNC:3420]	12
ENSG00000083896	7,032519	0,548252	0,000112	0,001845	4,976118	protein_coding	YTHDC1	YTH domain containing 1 [Source:HGNC Symbol;Acc:HGNC:30626]	4
ENSG00000111897	7,514986	-0,796766	0,000113	0,001849	-4,974657	protein_coding	SERINC1	serine incorporator 1 [Source:HGNC Symbol;Acc:HGNC:13464]	6
ENSG00000164484	5,323526	-2,582375	0,000114	0,00186	-4,971445	protein_coding	TMEM200/	transmembrane protein 200A [Source:HGNC Symbol;Acc:HGNC:21075]	6
ENSG00000123329	1,212347	3,455523	0,000114	0,001864	4,969898	protein_coding	ARHGAP9	Rho GTPase activating protein 9 [Source:HGNC Symbol;Acc:HGNC:14130]	12
ENSG00000143479	5,114659	-1,174982	0,000114	0,001865	-4,968255	protein_coding	DYRK3	dual specificity tyrosine phosphorylation regulated kinase 3 [Source:HGNC Symbol;Acc:HGNC:3094]	1
ENSG00000160796	5,674616	1,634675	0,000114	0,001865	4,968349	protein_coding	NBEAL2	neurobeachin like 2 [Source:HGNC Symbol;Acc:HGNC:31928]	3
ENSG00000096060	5,946392	2,090245	0,000114	0,001865	4,968639	protein_coding	FKBP5	FKBP prolyl isomerase 5 [Source:HGNC Symbol;Acc:HGNC:3721]	6
ENSG00000080298	4,730329	1,449426	0,000115	0,001866	4,966063	protein_coding	RFX3	regulatory factor X3 [Source:HGNC Symbol;Acc:HGNC:9984]	9
ENSG00000172215	-1,971998	3,050732	0,000115	0,001866	4,967076	protein_coding	CXCR6	C-X-C motif chemokine receptor 6 [Source:HGNC Symbol;Acc:HGNC:16647]	3
ENSG00000020129	5,280475	-0,947863	0,000115	0,001866	-4,966674	protein_coding	NCDN	neurochondrin [Source:HGNC Symbol;Acc:HGNC:17597]	1
ENSG00000119912	5,700262	-0,933576	0,000115	0,001866	-4,966454	protein_coding	IDE	insulin degrading enzyme [Source:HGNC Symbol;Acc:HGNC:5381]	10
ENSG00000213625	5,648792	-1,051511	0,000115	0,001867	-4,965343	protein_coding	LEPROT	leptin receptor overlapping transcript [Source:HGNC Symbol;Acc:HGNC:29477]	1
ENSG00000087842	3,061675	-1,552712	0,000116	0,001868	-4,962288	protein_coding	PIR	pirin [Source:HGNC Symbol;Acc:HGNC:30048]	X
ENSG00000104972	1,045038	3,079816	0,000116	0,001868	4,961704	protein_coding	LILRB1	leukocyte immunoglobulin like receptor B1 [Source:HGNC Symbol;Acc:HGNC:6605]	19
ENSG00000112183	0,694384	-3,019206	0,000116	0,001868	-4,962394	protein_coding	RBM24	RNA binding motif protein 24 [Source:HGNC Symbol;Acc:HGNC:21539]	6
ENSG00000160801	0,056683	3,115845	0,000116	0,001868	4,963029	protein_coding	PTH1R	parathyroid hormone 1 receptor [Source:HGNC Symbol;Acc:HGNC:9608]	3
ENSG00000170801	5,148657	-2,608847	0,000116	0,001868	-4,961624	protein_coding	HTRA3	HtrA serine peptidase 3 [Source:HGNC Symbol;Acc:HGNC:30406]	4
ENSG00000168264	7,591292	0,930065	0,000115	0,001868	4,963491	protein_coding	IRF2BP2	interferon regulatory factor 2 binding protein 2 [Source:HGNC Symbol;Acc:HGNC:21729]	1
ENSG00000153914	7,254112	0,89313	0,000115	0,001868	4,963399	protein_coding	SREK1	splicing regulatory glutamic acid and lysine rich protein 1 [Source:HGNC Symbol;Acc:HGNC:17882]	5
ENSG00000196329	-1,749773	3,283712	0,000116	0,001869	4,960958	protein_coding	GIMAP5	GTPase, IMAP family member 5 [Source:HGNC Symbol;Acc:HGNC:18005]	7
ENSG00000176973	1,946475	-1,868858	0,000117	0,001875	-4,958917	protein_coding	FAM89B	family with sequence similarity 89 member B [Source:HGNC Symbol;Acc:HGNC:16708]	11
ENSG00000164253	5,218664	-0,888475	0,000117	0,001877	-4,957994	protein_coding	WDR41	WD repeat domain 41 [Source:HGNC Symbol;Acc:HGNC:25601]	5
ENSG00000100336	0,100079	3,403948	0,000117	0,001886	4,955279	protein_coding	APOL4	apolipoprotein L4 [Source:HGNC Symbol;Acc:HGNC:14867]	22
ENSG00000255374	-1,599226	3,278982	0,000118	0,001889	4,953356	protein_coding	TAS2R43	taste 2 receptor member 43 [Source:HGNC Symbol;Acc:HGNC:18875]	12
ENSG00000075240	6,085901	1,460585	0,000118	0,001889	4,953643	protein_coding	GRAMD4	GRAM domain containing 4 [Source:HGNC Symbol;Acc:HGNC:29113]	22
ENSG00000172531	6,361296	-0,668255	0,000118	0,001889	-4,953058	protein_coding	PPP1CA	protein phosphatase 1 catalytic subunit alpha [Source:HGNC Symbol;Acc:HGNC:9281]	11
ENSG00000187595	-0,22352	2,403671	0,000118	0,001892	4,95192	protein_coding	ZNF385C	zinc finger protein 385C [Source:HGNC Symbol;Acc:HGNC:33722]	17
ENSG00000081985	-1,486026	4,117751	0,000118	0,001892	4,951293	protein_coding	IL12RB2	interleukin 12 receptor subunit beta 2 [Source:HGNC Symbol;Acc:HGNC:5972]	1
ENSG00000104852	7,883459	1,008742	0,000119	0,001894	4,950439	protein_coding	SNRNP70	small nuclear ribonucleoprotein U1 subunit 70 [Source:HGNC Symbol;Acc:HGNC:11150]	19
ENSG00000074660	1,799233	2,602301	0,00012	0,00191	4,946082	protein_coding	SCARF1	scavenger receptor class F member 1 [Source:HGNC Symbol;Acc:HGNC:16820]	17

ENSG00000189283	1,542774	2,051174	0,00012	0,001913	4,944551	protein_coding	FHIT	fragile histidine triad diadenosine triphosphatase [Source:HGNC Symbol;Acc:HGNC:3701]	3
ENSG00000146386	3,206337	-2,014644	0,00012	0,001913	-4,943653	protein_coding	ABRACL	ABRA C-terminal like [Source:HGNC Symbol;Acc:HGNC:21230]	6
ENSG00000234616	5,123941	1,161003	0,00012	0,001913	4,94351	protein_coding	JRK	Jrk helix-turn-helix protein [Source:HGNC Symbol;Acc:HGNC:6199]	8
ENSG00000169251	5,917605	-0,887092	0,00012	0,001913	-4,944057	protein_coding	NMD3	NMD3 ribosome export adaptor [Source:HGNC Symbol;Acc:HGNC:24250]	3
ENSG00000112293	1,744786	1,654017	0,000121	0,001927	4,939533	protein_coding	GPLD1	glycosylphosphatidylinositol specific phospholipase D1 [Source:HGNC Symbol;Acc:HGNC:4459]	6
ENSG00000154188	3,543347	-2,599078	0,000122	0,001939	-4,936005	protein_coding	ANGPT1	angiopoietin 1 [Source:HGNC Symbol;Acc:HGNC:484]	8
ENSG00000214357	5,46719	2,12638	0,000123	0,001942	4,934773	protein_coding	NEURL1B	neuralized E3 ubiquitin protein ligase 1B [Source:HGNC Symbol;Acc:HGNC:35422]	5
ENSG00000152684	5,310822	-1,051805	0,000123	0,001944	-4,93388	protein_coding	PELO	pelota mRNA surveillance and ribosome rescue factor [Source:HGNC Symbol;Acc:HGNC:8829]	5
ENSG00000186153	3,963612	1,420106	0,000124	0,001954	4,930966	protein_coding	WWOX	WW domain containing oxidoreductase [Source:HGNC Symbol;Acc:HGNC:12799]	16
ENSG00000173372	3,194636	4,166987	0,000124	0,001957	4,929444	protein_coding	C1QA	complement C1q A chain [Source:HGNC Symbol;Acc:HGNC:1241]	1
ENSG00000145685	5,816647	-1,45907	0,000124	0,001957	-4,929781	protein_coding	LHFPL2	LHFPL tetraspan subfamily member 2 [Source:HGNC Symbol;Acc:HGNC:6588]	5
ENSG00000161664	-0,100037	3,054423	0,000124	0,001961	4,927921	protein_coding	ASB16	ankyrin repeat and SOCS box containing 16 [Source:HGNC Symbol;Acc:HGNC:19768]	17
ENSG00000244754	7,094472	0,921311	0,000125	0,001969	4,925613	protein_coding	N4BP2L2	NEDD4 binding protein 2 like 2 [Source:HGNC Symbol;Acc:HGNC:26916]	13
ENSG00000123091	6,369046	-1,075548	0,000125	0,001973	-4,924211	protein_coding	RNF11	ring finger protein 11 [Source:HGNC Symbol;Acc:HGNC:10056]	1
ENSG00000072818	0,435118	3,482998	0,000126	0,001979	4,922284	protein_coding	ACAP1	ArfGAP with coiled-coil, ankyrin repeat and PH domains 1 [Source:HGNC Symbol;Acc:HGNC:16467]	17
ENSG00000087494	2,402813	-3,523485	0,000126	0,00198	-4,921209	protein_coding	PTHLH	parathyroid hormone like hormone [Source:HGNC Symbol;Acc:HGNC:9607]	12
ENSG00000177963	6,2575	-0,60461	0,000126	0,00198	-4,921477	protein_coding	RIC8A	RIC8 guanine nucleotide exchange factor A [Source:HGNC Symbol;Acc:HGNC:29550]	11
ENSG00000148925	5,051939	-0,838486	0,000127	0,001995	-4,91707	protein_coding	BTBD10	BTB domain containing 10 [Source:HGNC Symbol;Acc:HGNC:21445]	11
ENSG00000166762	2,578743	2,489023	0,000128	0,001997	4,915295	protein_coding	CATSPER2	cation channel sperm associated 2 [Source:HGNC Symbol;Acc:HGNC:18810]	15
ENSG00000140575	8,842538	-1,212032	0,000128	0,001997	-4,915875	protein_coding	IQGAP1	IQ motif containing GTPase activating protein 1 [Source:HGNC Symbol;Acc:HGNC:6110]	15
ENSG00000142168	7,013169	-0,863674	0,000128	0,001997	-4,91522	protein_coding	SOD1	superoxide dismutase 1 [Source:HGNC Symbol;Acc:HGNC:11179]	21
ENSG00000178199	-0,24025	3,340244	0,000128	0,002004	4,913168	protein_coding	ZC3H12D	zinc finger CCCH-type containing 12D [Source:HGNC Symbol;Acc:HGNC:21175]	6
ENSG00000130254	7,160561	0,88549	0,000129	0,002009	4,911425	protein_coding	SAFB2	scaffold attachment factor B2 [Source:HGNC Symbol;Acc:HGNC:21605]	19
ENSG00000159189	4,73319	3,187222	0,000129	0,002015	4,909602	protein_coding	C1QC	complement C1q C chain [Source:HGNC Symbol;Acc:HGNC:1245]	1
ENSG00000198954	5,402379	-0,766582	0,000131	0,00204	-4,90338	protein_coding	KIFBP	kinesin family binding protein [Source:HGNC Symbol;Acc:HGNC:23419]	10
ENSG00000157884	-2,062718	3,29841	0,000131	0,00204	4,902748	protein_coding	CIB4	calcium and integrin binding family member 4 [Source:HGNC Symbol;Acc:HGNC:33703]	2
ENSG00000111796	-2,97382	3,256525	0,000131	0,002042	4,901928	protein_coding	KLRB1	killer cell lectin like receptor B1 [Source:HGNC Symbol;Acc:HGNC:6373]	12
ENSG00000168811	-0,590479	-1,737254	0,000132	0,002047	-4,900411	protein_coding	IL12A	interleukin 12A [Source:HGNC Symbol;Acc:HGNC:5969]	3
ENSG00000106991	7,227453	-2,417779	0,000132	0,002051	-4,898927	protein_coding	ENG	endoglin [Source:HGNC Symbol;Acc:HGNC:3349]	9
ENSG00000173762	-1,369295	3,63763	0,000133	0,002063	4,895404	protein_coding	CD7	CD7 molecule [Source:HGNC Symbol;Acc:HGNC:1695]	17
ENSG00000185024	5,563324	0,874547	0,000133	0,002063	4,895391	protein_coding	BRF1	BRF1 RNA polymerase III transcription initiation factor subunit [Source:HGNC Symbol;Acc:HGNC:11551]	14
ENSG00000146223	6,552473	-0,750361	0,000133	0,002063	-4,894751	protein_coding	RPL7L1	ribosomal protein L7 like 1 [Source:HGNC Symbol;Acc:HGNC:21370]	6
ENSG00000231389	4,616447	3,011841	0,000134	0,002071	4,892621	protein_coding	HLA-DPA1	major histocompatibility complex, class II, DP alpha 1 [Source:HGNC Symbol;Acc:HGNC:4938]	6
ENSG00000180776	6,359543	-0,956894	0,000134	0,002075	-4,891305	protein_coding	ZDHHC20	zinc finger DHHC-type palmitoyltransferase 20 [Source:HGNC Symbol;Acc:HGNC:20749]	13
ENSG00000197057	-2,052638	3,777254	0,000135	0,002084	4,888803	protein_coding	DTHD1	death domain containing 1 [Source:HGNC Symbol;Acc:HGNC:37261]	4
ENSG00000152377	6,522068	-3,148293	0,000135	0,002087	-4,887678	protein_coding	SPOCK1	SPARC (osteonectin), cwcv and kazal like domains proteoglycan 1 [Source:HGNC Symbol;Acc:HGNC:11251]	5
ENSG00000167004	8,664581	-0,959543	0,000136	0,002098	-4,884692	protein_coding	PDIA3	protein disulfide isomerase family A member 3 [Source:HGNC Symbol;Acc:HGNC:4606]	15
ENSG00000138495	5,003064	-0,848816	0,000137	0,002104	-4,882911	protein_coding	COX17	cytochrome c oxidase copper chaperone COX17 [Source:HGNC Symbol;Acc:HGNC:2264]	3
ENSG00000129925	6,350752	-0,989831	0,000137	0,002107	-4,881325	protein_coding	PGAP6	post-glycosylphosphatidylinositol attachment to proteins 6 [Source:HGNC Symbol;Acc:HGNC:17205]	16
ENSG00000131389	7,425045	-1,42712	0,000137	0,002107	-4,881616	protein_coding	SLC6A6	solute carrier family 6 member 6 [Source:HGNC Symbol;Acc:HGNC:11052]	3
ENSG00000182885	-1,648936	3,910626	0,000138	0,00211	4,880065	protein_coding	ADGRG3	adhesion G protein-coupled receptor G3 [Source:HGNC Symbol;Acc:HGNC:13728]	16
ENSG00000124440	-0,014149	4,696412	0,000138	0,002115	4,878505	protein_coding	HIF3A	hypoxia inducible factor 3 subunit alpha [Source:HGNC Symbol;Acc:HGNC:15825]	19
ENSG00000182197	6,595261	-1,727432	0,000138	0,002118	-4,877465	protein_coding	EXT1	exostosin glycosyltransferase 1 [Source:HGNC Symbol;Acc:HGNC:3512]	8
ENSG00000149634	0,209746	2,098606	0,000139	0,002122	4,875196	protein_coding	SPATA25	spermatogenesis associated 25 [Source:HGNC Symbol;Acc:HGNC:16158]	20
ENSG00000154556	6,083617	1,422487	0,000139	0,002122	4,876003	protein_coding	SORBS2	sorbin and SH3 domain containing 2 [Source:HGNC Symbol;Acc:HGNC:24098]	4
ENSG00000136003	5,883389	-1,028543	0,000139	0,002122	-4,875206	protein_coding	ISCU	iron-sulfur cluster assembly enzyme [Source:HGNC Symbol;Acc:HGNC:29882]	12
ENSG00000115486	5,407796	-0,929949	0,000139	0,002123	-4,874239	protein_coding	GGCX	gamma-glutamyl carboxylase [Source:HGNC Symbol;Acc:HGNC:4247]	2
ENSG00000005175	5,474412	-0,551286	0,000139	0,002123	-4,874204	protein_coding	RPAP3	RNA polymerase II associated protein 3 [Source:HGNC Symbol;Acc:HGNC:26151]	12
ENSG00000171928	4,782597	-1,128506	0,00014	0,002128	-4,872682	protein_coding	TVP23B	trans-golgi network vesicle protein 23 homolog B [Source:HGNC Symbol;Acc:HGNC:20399]	17
ENSG00000176153	4,203706	-3,385133	0,00014	0,002129	-4,871975	protein_coding	GPX2	glutathione peroxidase 2 [Source:HGNC Symbol;Acc:HGNC:4554]	14
ENSG00000168010	4,54771	1,512699	0,00014	0,00213	4,871261	protein_coding	ATG16L2	autophagy related 16 like 2 [Source:HGNC Symbol;Acc:HGNC:25464]	11

ENSG00000173812	8,132984	-0,712945	0,00014	0,00213	-4,870886	protein_coding	EIF1	eukaryotic translation initiation factor 1 [Source:HGNC Symbol;Acc:HGNC:3249]	17
ENSG00000119725	1,951351	1,235961	0,000141	0,002132	4,87002	protein_coding	ZNF410	zinc finger protein 410 [Source:HGNC Symbol;Acc:HGNC:20144]	14
ENSG00000122188	-1,804124	3,664191	0,000141	0,002132	4,8695	protein_coding	LAX1	lymphocyte transmembrane adaptor 1 [Source:HGNC Symbol;Acc:HGNC:26005]	1
ENSG00000166689	4,445415	1,717928	0,000141	0,002139	4,867407	protein_coding	PLEKHA7	pleckstrin homology domain containing A7 [Source:HGNC Symbol;Acc:HGNC:27049]	11
ENSG00000146574	3,826897	-1,130511	0,000142	0,002151	-4,864076	protein_coding	CCZ1B	CCZ1 homolog B, vacuolar protein trafficking and biogenesis associated [Source:HGNC Symbol;Acc:HGNC:21717]	7
ENSG00000095066	6,059207	1,455311	0,000142	0,002151	4,864141	protein_coding	HOOK2	hook microtubule tethering protein 2 [Source:HGNC Symbol;Acc:HGNC:19885]	19
ENSG00000067064	5,941143	-1,306453	0,000143	0,002159	-4,861881	protein_coding	IDI1	isopentenyl-diphosphate delta isomerase 1 [Source:HGNC Symbol;Acc:HGNC:5387]	10
ENSG00000186377	-2,148703	3,028948	0,000144	0,002166	4,859829	protein_coding	CYP4X1	cytochrome P450 family 4 subfamily X member 1 [Source:HGNC Symbol;Acc:HGNC:20244]	1
ENSG00000003436	5,661009	-2,37616	0,000144	0,002167	-4,858757	protein_coding	TFPI	tissue factor pathway inhibitor [Source:HGNC Symbol;Acc:HGNC:11760]	2
ENSG00000170027	8,232149	-0,814152	0,000144	0,002167	-4,858976	protein_coding	YWHAG	tyrosine 3-monooxygenase/tryptophan 5-monooxygenase activation protein gamma [Source:HGNC Symbol;Acc:HGI	7
ENSG00000103168	4,842033	0,638946	0,000144	0,002172	4,857289	protein_coding	TAF1C	TATA-box binding protein associated factor, RNA polymerase I subunit C [Source:HGNC Symbol;Acc:HGNC:11534]	16
ENSG00000143543	4,614438	-1,156619	0,000145	0,002175	-4,855767	protein_coding	JTB	jumping translocation breakpoint [Source:HGNC Symbol;Acc:HGNC:6201]	1
ENSG00000077147	7,83632	-0,891865	0,000145	0,002175	-4,855945	protein_coding	TM9SF3	transmembrane 9 superfamily member 3 [Source:HGNC Symbol;Acc:HGNC:21529]	10
ENSG00000188811	4,929582	0,963227	0,000145	0,002176	4,854849	protein_coding	NHLRC3	NHL repeat containing 3 [Source:HGNC Symbol;Acc:HGNC:33751]	13
ENSG00000079999	6,027499	-0,582233	0,000145	0,002176	-4,854688	protein_coding	KEAP1	kelch like ECH associated protein 1 [Source:HGNC Symbol;Acc:HGNC:23177]	19
ENSG00000133393	4,362447	-1,066716	0,000146	0,002187	-4,851029	protein_coding	CEP20	centrosomal protein 20 [Source:HGNC Symbol;Acc:HGNC:26435]	16
ENSG00000050426	4,901512	1,083965	0,000146	0,002187	4,850951	protein_coding	LETMD1	LETM1 domain containing 1 [Source:HGNC Symbol;Acc:HGNC:24241]	12
ENSG00000114480	5,903812	-0,884482	0,000146	0,002187	-4,851675	protein_coding	GBE1	1,4-alpha-glucan branching enzyme 1 [Source:HGNC Symbol;Acc:HGNC:4180]	3
ENSG00000116793	5,099091	-0,898648	0,000147	0,002191	-4,849616	protein_coding	PHTF1	putative homeodomain transcription factor 1 [Source:HGNC Symbol;Acc:HGNC:8939]	1
ENSG00000162702	5,664948	-1,065294	0,000147	0,002192	-4,848404	protein_coding	ZNF281	zinc finger protein 281 [Source:HGNC Symbol;Acc:HGNC:13075]	1
ENSG00000156030	6,999332	0,646095	0,000147	0,002192	4,848316	protein_coding	MIDEAS	mitotic deacetylase associated SANT domain protein [Source:HGNC Symbol;Acc:HGNC:19853]	14
ENSG00000112697	7,531398	-0,850608	0,000147	0,002192	-4,848163	protein_coding	TMEM30A	transmembrane protein 30A [Source:HGNC Symbol;Acc:HGNC:16667]	6
ENSG00000277203	2,319371	-1,977614	0,000147	0,002192	-4,847649	protein_coding	F8A1	coagulation factor VIII associated 1 [Source:HGNC Symbol;Acc:HGNC:3547]	X
ENSG00000148484	5,672381	-1,484714	0,000148	0,002197	-4,84608	protein_coding	RSU1	Ras suppressor protein 1 [Source:HGNC Symbol;Acc:HGNC:10464]	10
ENSG00000226761	-1,305028	2,898517	0,000148	0,002203	4,844347	protein_coding	TAS2R46	taste 2 receptor member 46 [Source:HGNC Symbol;Acc:HGNC:18877]	12
ENSG00000031698	7,283992	-0,747764	0,000149	0,002203	-4,844047	protein_coding	SARS1	seryl-tRNA synthetase 1 [Source:HGNC Symbol;Acc:HGNC:10537]	1
ENSG00000170545	3,032583	-1,419383	0,000149	0,002206	-4,842979	protein_coding	SMAGP	small cell adhesion glycoprotein [Source:HGNC Symbol;Acc:HGNC:26918]	12
ENSG00000101084	3,865127	-0,931656	0,000149	0,00221	-4,841714	protein_coding	RAB5IF	RAB5 interacting factor [Source:HGNC Symbol;Acc:HGNC:15870]	20
ENSG00000150961	6,906534	-1,551067	0,00015	0,002218	-4,839533	protein_coding	SEC24D	SEC24 homolog D, COPII coat complex component [Source:HGNC Symbol;Acc:HGNC:10706]	4
ENSG00000125652	4,332058	1,069927	0,000151	0,002233	4,836008	protein_coding	ALKBH7	alkB homolog 7 [Source:HGNC Symbol;Acc:HGNC:21306]	19
ENSG00000050130	4,831218	-0,90415	0,000152	0,002239	-4,833838	protein_coding	JKAMP	JNK1/MAPK8 associated membrane protein [Source:HGNC Symbol;Acc:HGNC:20184]	14
ENSG00000073614	7,225044	0,641324	0,000152	0,002239	4,834051	protein_coding	KDM5A	lysine demethylase 5A [Source:HGNC Symbol;Acc:HGNC:9886]	12
ENSG00000171790	-0,674292	2,669349	0,000153	0,002242	4,831377	protein_coding	SLFNL1	schlafen like 1 [Source:HGNC Symbol;Acc:HGNC:26313]	1
ENSG00000149218	5,310637	-2,064414	0,000153	0,002242	-4,831452	protein_coding	ENDOD1	endonuclease domain containing 1 [Source:HGNC Symbol;Acc:HGNC:29129]	11
ENSG00000107077	5,972178	1,03741	0,000152	0,002242	4,832729	protein_coding	KDM4C	lysine demethylase 4C [Source:HGNC Symbol;Acc:HGNC:17071]	9
ENSG00000099622	6,970646	1,010551	0,000153	0,002242	4,831071	protein_coding	CIRBP	cold inducible RNA binding protein [Source:HGNC Symbol;Acc:HGNC:1982]	19
ENSG00000125977	6,947862	-0,77895	0,000152	0,002242	-4,831794	protein_coding	EIF2S2	eukaryotic translation initiation factor 2 subunit beta [Source:HGNC Symbol;Acc:HGNC:3266]	20
ENSG00000133315	2,10609	1,942926	0,000153	0,002242	4,830587	protein_coding	MACROD1	mono-ADP ribosylhydrolase 1 [Source:HGNC Symbol;Acc:HGNC:29598]	11
ENSG00000154133	1,599515	3,617621	0,000153	0,002247	4,828652	protein_coding	ROBO4	roundabout guidance receptor 4 [Source:HGNC Symbol;Acc:HGNC:17985]	11
ENSG00000130595	-2,915423	3,33005	0,000153	0,002247	4,828936	protein_coding	TNNT3	troponin T3, fast skeletal type [Source:HGNC Symbol;Acc:HGNC:11950]	11
ENSG00000104043	1,946006	2,537667	0,000155	0,002249	4,825556	protein_coding	ATP8B4	ATPase phospholipid transporting 8B4 (putative) [Source:HGNC Symbol;Acc:HGNC:13536]	15
ENSG00000102445	0,972035	3,078321	0,000154	0,002249	4,827048	protein_coding	RUBCNL	rubicon like autophagy enhancer [Source:HGNC Symbol;Acc:HGNC:20420]	13
ENSG00000166452	3,806281	-0,780134	0,000155	0,002249	-4,824387	protein_coding	AKIP1	A-kinase interacting protein 1 [Source:HGNC Symbol;Acc:HGNC:1170]	11
ENSG00000161381	4,222569	1,303126	0,000154	0,002249	4,826175	protein_coding	PLXDC1	plexin domain containing 1 [Source:HGNC Symbol;Acc:HGNC:20945]	17
ENSG00000160271	4,408772	1,263332	0,000155	0,002249	4,824676	protein_coding	RALGDS	ral guanine nucleotide dissociation stimulator [Source:HGNC Symbol;Acc:HGNC:9842]	9
ENSG00000150681	-1,554736	3,52805	0,000154	0,002249	4,825705	protein_coding	RGS18	regulator of G protein signaling 18 [Source:HGNC Symbol;Acc:HGNC:14261]	1
ENSG00000163069	5,959665	-1,435824	0,000155	0,002249	-4,824996	protein_coding	SGCB	sarcoglycan beta [Source:HGNC Symbol;Acc:HGNC:10806]	4
ENSG00000157020	6,814868	-0,839961	0,000154	0,002249	-4,826934	protein_coding	SEC13	SEC13 homolog, nuclear pore and COPII coat complex component [Source:HGNC Symbol;Acc:HGNC:10697]	3
ENSG00000156261	6,787887	-0,986142	0,000155	0,002249	-4,825084	protein_coding	CCT8	chaperonin containing TCP1 subunit 8 [Source:HGNC Symbol;Acc:HGNC:1623]	21
ENSG00000116752	4,230051	-0,957328	0,000156	0,002258	-4,821727	protein_coding	BCAS2	BCAS2 pre-mRNA processing factor [Source:HGNC Symbol;Acc:HGNC:975]	1
ENSG00000156650	6,732232	0,655627	0,000156	0,002258	4,822018	protein_coding	KAT6B	lysine acetyltransferase 6B [Source:HGNC Symbol;Acc:HGNC:17582]	10

ENSG00000228474	5,546257	-1,232375	0,000156	0,002263	-4,820255	protein_coding	OST4	oligosaccharyltransferase complex subunit 4, non-catalytic [Source:HGNC Symbol;Acc:HGNC:32483]	2
ENSG00000170606	7,697816	-0,714494	0,000157	0,002273	-4,81775	protein_coding	HSPA4	heat shock protein family A (Hsp70) member 4 [Source:HGNC Symbol;Acc:HGNC:5237]	5
ENSG00000012660	6,640504	-0,879641	0,000158	0,002279	-4,816157	protein_coding	ELOVL5	ELOVL fatty acid elongase 5 [Source:HGNC Symbol;Acc:HGNC:21308]	6
ENSG00000169612	3,348078	-1,124835	0,000158	0,002283	-4,8148	protein_coding	RAMAC	RNA guanine-7 methyltransferase activating subunit [Source:HGNC Symbol;Acc:HGNC:31022]	15
ENSG00000115652	5,482441	-1,073945	0,000158	0,002285	-4,813766	protein_coding	UXS1	UDP-glucuronate decarboxylase 1 [Source:HGNC Symbol;Acc:HGNC:17729]	2
ENSG00000067248	6,211999	-0,554326	0,000158	0,002285	-4,813542	protein_coding	DHX29	DExH-box helicase 29 [Source:HGNC Symbol;Acc:HGNC:15815]	5
ENSG00000187116	-1,259818	3,248362	0,000159	0,002286	4,812915	protein_coding	LILRA5	leukocyte immunoglobulin like receptor A5 [Source:HGNC Symbol;Acc:HGNC:16309]	19
ENSG00000185291	-0,833432	3,719556	0,000159	0,002291	4,81154	protein_coding	IL3RA	interleukin 3 receptor subunit alpha [Source:HGNC Symbol;Acc:HGNC:6012]	X
ENSG00000188783	3,022391	4,396213	0,00016	0,002296	4,809363	protein_coding	PRELP	proline and arginine rich end leucine rich repeat protein [Source:HGNC Symbol;Acc:HGNC:9357]	1
ENSG00000131503	2,364445	1,53934	0,00016	0,002296	4,809274	protein_coding	ANKHD1	ankyrin repeat and KH domain containing 1 [Source:HGNC Symbol;Acc:HGNC:24714]	5
ENSG00000185009	5,328437	-0,680147	0,00016	0,002296	-4,808867	protein_coding	AP3M1	adaptor related protein complex 3 subunit mu 1 [Source:HGNC Symbol;Acc:HGNC:569]	10
ENSG00000131966	5,575237	-0,750533	0,00016	0,002296	-4,810001	protein_coding	ACTR10	actin related protein 10 [Source:HGNC Symbol;Acc:HGNC:17372]	14
ENSG00000109171	6,079313	-0,822156	0,000161	0,002309	-4,805339	protein_coding	SLAIN2	SLAIN motif family member 2 [Source:HGNC Symbol;Acc:HGNC:29282]	4
ENSG00000138071	7,583636	-1,066547	0,000161	0,002309	-4,805294	protein_coding	ACTR2	actin related protein 2 [Source:HGNC Symbol;Acc:HGNC:169]	2
ENSG00000246922	2,187998	2,02286	0,000162	0,002322	4,802257	protein_coding	UBAP1L	ubiquitin associated protein 1 like [Source:HGNC Symbol;Acc:HGNC:40028]	15
ENSG00000182870	1,617425	-3,129126	0,000163	0,002323	-4,801364	protein_coding	GALNT9	polypeptide N-acetylgalactosaminyltransferase 9 [Source:HGNC Symbol;Acc:HGNC:4131]	12
ENSG00000175324	4,708495	-0,900354	0,000163	0,002323	-4,801699	protein_coding	LSM1	LSM1 homolog, mRNA degradation associated [Source:HGNC Symbol;Acc:HGNC:20472]	8
ENSG00000121579	6,577429	-0,79708	0,000163	0,002323	-4,800826	protein_coding	NAA50	N-alpha-acetyltransferase 50, NatE catalytic subunit [Source:HGNC Symbol;Acc:HGNC:29533]	3
ENSG00000186575	6,641191	-0,649653	0,000163	0,002326	-4,79977	protein_coding	NF2	neurofibromin 2 [Source:HGNC Symbol;Acc:HGNC:7773]	22
ENSG00000153283	-0,720314	3,116439	0,000164	0,002337	4,797113	protein_coding	CD96	CD96 molecule [Source:HGNC Symbol;Acc:HGNC:16892]	3
ENSG00000165660	4,36595	-0,671523	0,000164	0,002337	-4,796719	protein_coding	ABRAXAS2	abraxas 2, BRISC complex subunit [Source:HGNC Symbol;Acc:HGNC:28975]	10
ENSG00000167286	-2,12093	3,436492	0,000165	0,002339	4,795572	protein_coding	CD3D	CD3d molecule [Source:HGNC Symbol;Acc:HGNC:1673]	11
ENSG00000213694	5,915812	-2,285293	0,000165	0,002339	-4,795848	protein_coding	S1PR3	sphingosine-1-phosphate receptor 3 [Source:HGNC Symbol;Acc:HGNC:3167]	9
ENSG00000182162	0,071238	4,314852	0,000167	0,002366	4,789701	protein_coding	P2RY8	P2Y receptor family member 8 [Source:HGNC Symbol;Acc:HGNC:15524]	X
ENSG00000198492	6,083871	-0,903997	0,000167	0,002367	-4,789138	protein_coding	YTHDF2	YTH N6-methyladenosine RNA binding protein 2 [Source:HGNC Symbol;Acc:HGNC:31675]	1
ENSG00000113712	7,446772	-0,592914	0,000168	0,002375	-4,787068	protein_coding	CSNK1A1	casein kinase 1 alpha 1 [Source:HGNC Symbol;Acc:HGNC:2451]	5
ENSG00000156222	-2,267975	2,735401	0,000168	0,002377	4,786339	protein_coding	SLC28A1	solute carrier family 28 member 1 [Source:HGNC Symbol;Acc:HGNC:11001]	15
ENSG00000249437	1,022956	2,185484	0,000168	0,002379	4,785581	protein_coding	NAIP	NLR family apoptosis inhibitory protein [Source:HGNC Symbol;Acc:HGNC:7634]	5
ENSG00000124772	0,210327	3,241421	0,000169	0,002383	4,784299	protein_coding	CPNE5	copine 5 [Source:HGNC Symbol;Acc:HGNC:2318]	6
ENSG00000090661	4,186136	2,098221	0,000169	0,002385	4,78345	protein_coding	CERS4	ceramide synthase 4 [Source:HGNC Symbol;Acc:HGNC:23747]	19
ENSG00000135070	4,916258	-0,882916	0,000169	0,002385	-4,783144	protein_coding	ISCA1	iron-sulfur cluster assembly 1 [Source:HGNC Symbol;Acc:HGNC:28660]	9
ENSG00000166780	4,799894	-1,758548	0,00017	0,00239	-4,781648	protein_coding	BMERB1	bMERB domain containing 1 [Source:HGNC Symbol;Acc:HGNC:19213]	16
ENSG00000106683	5,512032	-1,060403	0,00017	0,002392	-4,78091	protein_coding	LIMK1	LIM domain kinase 1 [Source:HGNC Symbol;Acc:HGNC:6613]	7
ENSG00000141985	6,342146	-0,767466	0,000171	0,002398	-4,778846	protein_coding	SH3GL1	SH3 domain containing GRB2 like 1, endophilin A2 [Source:HGNC Symbol;Acc:HGNC:10830]	19
ENSG00000116871	7,491876	-0,839512	0,00017	0,002398	-4,77923	protein_coding	MAP7D1	MAP7 domain containing 1 [Source:HGNC Symbol;Acc:HGNC:25514]	1
ENSG00000185787	7,655137	-0,901497	0,000171	0,002404	-4,777332	protein_coding	MORF4L1	mortality factor 4 like 1 [Source:HGNC Symbol;Acc:HGNC:16989]	15
ENSG00000175745	6,032554	-2,004521	0,000172	0,002409	-4,775937	protein_coding	NR2F1	nuclear receptor subfamily 2 group F member 1 [Source:HGNC Symbol;Acc:HGNC:7975]	5
ENSG00000102796	1,961857	1,719381	0,000172	0,002411	4,774719	protein_coding	DHRS12	dehydrogenase/reductase 12 [Source:HGNC Symbol;Acc:HGNC:25832]	13
ENSG00000256537	4,912202	-0,767431	0,000172	0,002411	-4,774376	protein_coding	SMIM10L1	small integral membrane protein 10 like 1 [Source:HGNC Symbol;Acc:HGNC:49847]	12
ENSG00000078369	8,588426	-0,987662	0,000172	0,002411	-4,775177	protein_coding	GNB1	G protein subunit beta 1 [Source:HGNC Symbol;Acc:HGNC:4396]	1
ENSG00000105321	5,58404	0,875964	0,000173	0,002414	4,773015	protein_coding	CCDC9	coiled-coil domain containing 9 [Source:HGNC Symbol;Acc:HGNC:24560]	19
ENSG00000164574	6,193211	-1,510439	0,000173	0,002414	-4,773168	protein_coding	GALNT10	polypeptide N-acetylgalactosaminyltransferase 10 [Source:HGNC Symbol;Acc:HGNC:19873]	5
ENSG00000103707	3,427612	-0,810235	0,000173	0,002416	-4,77169	protein_coding	MTFMT	mitochondrial methionyl-tRNA formyltransferase [Source:HGNC Symbol;Acc:HGNC:29666]	15
ENSG00000248592	-1,441264	1,924767	0,000173	0,002416	4,772102	protein_coding	STIMATE-M	STIMATE-MUSTN1 readthrough [Source:HGNC Symbol;Acc:HGNC:38834]	3
ENSG00000172239	6,369856	-0,890144	0,000173	0,002416	-4,771413	protein_coding	PAIP1	poly(A) binding protein interacting protein 1 [Source:HGNC Symbol;Acc:HGNC:16945]	5
ENSG00000160097	0,265849	2,83623	0,000174	0,002418	4,770557	protein_coding	FNDC5	fibronectin type III domain containing 5 [Source:HGNC Symbol;Acc:HGNC:20240]	1
ENSG00000148356	5,01702	0,971549	0,000174	0,002419	4,76999	protein_coding	LRSAM1	leucine rich repeat and sterile alpha motif containing 1 [Source:HGNC Symbol;Acc:HGNC:25135]	9
ENSG00000196126	4,409269	3,445415	0,000175	0,002426	4,768126	protein_coding	HLA-DRB1	major histocompatibility complex, class II, DR beta 1 [Source:HGNC Symbol;Acc:HGNC:4948]	6
ENSG00000106819	0,752333	5,05981	0,000175	0,002428	4,767312	protein_coding	ASPN	asporin [Source:HGNC Symbol;Acc:HGNC:14872]	9
ENSG00000114956	4,091903	-0,773501	0,000175	0,002431	-4,766515	protein_coding	DGUOK	deoxyguanosine kinase [Source:HGNC Symbol;Acc:HGNC:2858]	2
ENSG00000116954	3,558885	-1,015318	0,000175	0,002431	-4,765691	protein_coding	RRAGC	Ras related GTP binding C [Source:HGNC Symbol;Acc:HGNC:19902]	1

ENSG00000255837	-0,905357	3,011886	0,000175	0,002431	4,765746	protein_coding	TAS2R20	taste 2 receptor member 20 [Source:HGNC Symbol;Acc:HGNC:19109]	12
ENSG00000154822	4,239908	1,555289	0,000177	0,002453	4,760577	protein_coding	PLCL2	phospholipase C like 2 [Source:HGNC Symbol;Acc:HGNC:9064]	3
ENSG00000064726	6,27648	-0,990534	0,000177	0,002453	-4,760922	protein_coding	BTBD1	BTB domain containing 1 [Source:HGNC Symbol;Acc:HGNC:1120]	15
ENSG00000133106	3,539659	1,973905	0,000178	0,002461	4,758732	protein_coding	EPSTI1	epithelial stromal interaction 1 [Source:HGNC Symbol;Acc:HGNC:16465]	13
ENSG00000172062	1,973793	-1,233814	0,000179	0,002465	-4,757407	protein_coding	SMN1	survival of motor neuron 1, telomeric [Source:HGNC Symbol;Acc:HGNC:11117]	5
ENSG00000106615	5,72907	-0,718265	0,000179	0,002466	-4,756836	protein_coding	RHEB	Ras homolog, mTORC1 binding [Source:HGNC Symbol;Acc:HGNC:10011]	7
ENSG00000126870	5,784242	0,954137	0,00018	0,002477	4,754374	protein_coding	DYNC2I1	dynein 2 intermediate chain 1 [Source:HGNC Symbol;Acc:HGNC:21862]	7
ENSG00000115145	5,800463	-0,818437	0,00018	0,002481	-4,753213	protein_coding	STAM2	signal transducing adaptor molecule 2 [Source:HGNC Symbol;Acc:HGNC:11358]	2
ENSG00000166033	7,315449	-3,120156	0,000183	0,002512	-4,746984	protein_coding	HTRA1	HtrA serine peptidase 1 [Source:HGNC Symbol;Acc:HGNC:9476]	10
ENSG00000157570	1,383022	4,150221	0,000183	0,002513	4,746161	protein_coding	TSPAN18	tetraspanin 18 [Source:HGNC Symbol;Acc:HGNC:20660]	11
ENSG00000117569	4,023776	1,051785	0,000183	0,002513	4,745958	protein_coding	PTBP2	polypyrimidine tract binding protein 2 [Source:HGNC Symbol;Acc:HGNC:17662]	1
ENSG00000120049	1,090669	2,143299	0,000184	0,002525	4,743427	protein_coding	KCNIP2	potassium voltage-gated channel interacting protein 2 [Source:HGNC Symbol;Acc:HGNC:15522]	10
ENSG00000138138	5,477465	-0,692141	0,000185	0,002532	-4,741632	protein_coding	ATAD1	ATPase family AAA domain containing 1 [Source:HGNC Symbol;Acc:HGNC:25903]	10
ENSG00000010017	6,370611	-0,724699	0,000185	0,002538	-4,740166	protein_coding	RANBP9	RAN binding protein 9 [Source:HGNC Symbol;Acc:HGNC:13727]	6
ENSG00000082684	0,787811	4,382447	0,000186	0,002544	4,738631	protein_coding	SEMA5B	semaphorin 5B [Source:HGNC Symbol;Acc:HGNC:10737]	3
ENSG00000167461	6,1511	-0,622487	0,000187	0,00255	-4,736751	protein_coding	RAB8A	RAB8A, member RAS oncogene family [Source:HGNC Symbol;Acc:HGNC:7007]	19
ENSG00000078269	7,451411	-1,364521	0,000187	0,00255	-4,736853	protein_coding	SYNJ2	synaptojanin 2 [Source:HGNC Symbol;Acc:HGNC:11504]	6
ENSG00000171962	1,152897	2,774436	0,000188	0,002557	4,733782	protein_coding	DRC3	dynein regulatory complex subunit 3 [Source:HGNC Symbol;Acc:HGNC:25384]	17
ENSG00000019169	0,324304	4,093314	0,000188	0,002557	4,734002	protein_coding	MARCO	macrophage receptor with collagenous structure [Source:HGNC Symbol;Acc:HGNC:6895]	2
ENSG00000136816	5,153441	-1,112652	0,000188	0,002557	-4,733613	protein_coding	TOR1B	torsin family 1 member B [Source:HGNC Symbol;Acc:HGNC:11995]	9
ENSG00000176014	5,906475	-1,800042	0,000187	0,002557	-4,734971	protein_coding	TUBB6	tubulin beta 6 class V [Source:HGNC Symbol;Acc:HGNC:20776]	18
ENSG00000168092	7,056425	-0,876378	0,000188	0,002557	-4,734356	protein_coding	PAFAH1B2	platelet activating factor acetylhydrolase 1b catalytic subunit 2 [Source:HGNC Symbol;Acc:HGNC:8575]	11
ENSG00000143507	4,322913	-1,076279	0,000188	0,00256	-4,732552	protein_coding	DUSP10	dual specificity phosphatase 10 [Source:HGNC Symbol;Acc:HGNC:3065]	1
ENSG00000132840	2,367752	-3,278724	0,000189	0,00257	-4,730003	protein_coding	BHMT2	betaine--homocysteine S-methyltransferase 2 [Source:HGNC Symbol;Acc:HGNC:1048]	5
ENSG00000105383	-0,181541	2,931231	0,000189	0,00257	4,730206	protein_coding	CD33	CD33 molecule [Source:HGNC Symbol;Acc:HGNC:1659]	19
ENSG00000196139	4,778716	-2,134866	0,00019	0,002576	-4,72845	protein_coding	AKR1C3	aldo-keto reductase family 1 member C3 [Source:HGNC Symbol;Acc:HGNC:386]	10
ENSG00000267221	0,949562	2,165943	0,000191	0,002588	4,72595	protein_coding	C17orf113	chromosome 17 open reading frame 113 [Source:HGNC Symbol;Acc:HGNC:53437]	17
ENSG00000223865	4,38667	2,885466	0,000191	0,002589	4,725	protein_coding	HLA-DPB1	major histocompatibility complex, class II, DP beta 1 [Source:HGNC Symbol;Acc:HGNC:4940]	6
ENSG00000026508	7,685932	-2,495122	0,000191	0,002589	-4,725273	protein_coding	CD44	CD44 molecule (Indian blood group) [Source:HGNC Symbol;Acc:HGNC:1681]	11
ENSG00000100554	5,64279	-0,88866	0,000192	0,002593	-4,723813	protein_coding	ATP6V1D	ATPase H+ transporting V1 subunit D [Source:HGNC Symbol;Acc:HGNC:13527]	14
ENSG00000113068	4,956646	-0,621184	0,000193	0,002607	-4,720863	protein_coding	PFDN1	prefoldin subunit 1 [Source:HGNC Symbol;Acc:HGNC:8866]	5
ENSG00000166068	6,061292	-1,749626	0,000193	0,002607	-4,720567	protein_coding	SPRED1	sprouty related EVH1 domain containing 1 [Source:HGNC Symbol;Acc:HGNC:20249]	15
ENSG00000173674	5,65644	-0,911793	0,000194	0,002611	-4,719367	protein_coding	EIF1AX	eukaryotic translation initiation factor 1A X-linked [Source:HGNC Symbol;Acc:HGNC:3250]	X
ENSG00000155850	5,775709	-0,999874	0,000194	0,002612	-4,7189	protein_coding	SLC26A2	solute carrier family 26 member 2 [Source:HGNC Symbol;Acc:HGNC:10994]	5
ENSG00000081913	4,931483	0,961884	0,000194	0,002618	4,717319	protein_coding	PHLPP1	PH domain and leucine rich repeat protein phosphatase 1 [Source:HGNC Symbol;Acc:HGNC:20610]	18
ENSG00000198763	11,64424	-1,786933	0,000195	0,002622	-4,716205	protein_coding	MT-ND2	mitochondrially encoded NADH:ubiquinone oxidoreductase core subunit 2 [Source:HGNC Symbol;Acc:HGNC:7456]	MT
ENSG00000198892	3,196856	-1,660124	0,000196	0,002628	-4,714752	protein_coding	SHISA4	shisa family member 4 [Source:HGNC Symbol;Acc:HGNC:27139]	1
ENSG00000178927	5,47879	0,724774	0,000196	0,00263	4,714113	protein_coding	CYBC1	cytochrome b-245 chaperone 1 [Source:HGNC Symbol;Acc:HGNC:28672]	17
ENSG00000170017	7,220132	-1,135003	0,000197	0,002639	-4,712058	protein_coding	ALCAM	activated leukocyte cell adhesion molecule [Source:HGNC Symbol;Acc:HGNC:400]	3
ENSG00000173757	6,110563	0,773042	0,000198	0,002649	4,70991	protein_coding	STAT5B	signal transducer and activator of transcription 5B [Source:HGNC Symbol;Acc:HGNC:11367]	17
ENSG00000018408	6,674268	-2,418712	0,000198	0,002651	-4,709105	protein_coding	WWTR1	WW domain containing transcription regulator 1 [Source:HGNC Symbol;Acc:HGNC:24042]	3
ENSG00000130413	2,930699	2,455782	0,000199	0,00266	4,707102	protein_coding	STK33	serine/threonine kinase 33 [Source:HGNC Symbol;Acc:HGNC:14568]	11
ENSG00000122679	-1,555667	4,455252	0,000199	0,00266	4,706876	protein_coding	RAMP3	receptor activity modifying protein 3 [Source:HGNC Symbol;Acc:HGNC:9845]	7
ENSG00000100629	4,356176	1,520203	0,000199	0,002662	4,706045	protein_coding	CEP128	centrosomal protein 128 [Source:HGNC Symbol;Acc:HGNC:20359]	14
ENSG00000175538	1,561038	2,777284	0,0002	0,002663	4,703958	protein_coding	KCNE3	potassium voltage-gated channel subfamily E regulatory subunit 3 [Source:HGNC Symbol;Acc:HGNC:6243]	11
ENSG00000198520	-0,40631	2,09535	0,0002	0,002663	4,704417	protein_coding	ARMH1	armadillo like helical domain containing 1 [Source:HGNC Symbol;Acc:HGNC:34345]	1
ENSG00000153563	-0,94141	2,99963	0,0002	0,002663	4,705059	protein_coding	CD8A	CD8a molecule [Source:HGNC Symbol;Acc:HGNC:1706]	2
ENSG00000143546	-1,284322	4,367847	0,0002	0,002663	4,704397	protein_coding	S100A8	S100 calcium binding protein A8 [Source:HGNC Symbol;Acc:HGNC:10498]	1
ENSG00000102898	5,635834	-0,728039	0,0002	0,002663	-4,704185	protein_coding	NUTF2	nuclear transport factor 2 [Source:HGNC Symbol;Acc:HGNC:13722]	16
ENSG00000125810	3,370247	5,607099	0,0002	0,002664	4,703375	protein_coding	CD93	CD93 molecule [Source:HGNC Symbol;Acc:HGNC:15855]	20
ENSG00000173905	7,681396	-1,088809	0,000201	0,002668	-4,702376	protein_coding	GOLIM4	golgi integral membrane protein 4 [Source:HGNC Symbol;Acc:HGNC:15448]	3

ENSG00000196811	0,251168	7,236139	0,000201	0,002668	4,701885	protein_coding	CHRNG	cholinergic receptor nicotinic gamma subunit [Source:HGNC Symbol;Acc:HGNC:1967]	2
ENSG00000154447	5,894949	-0,960116	0,000202	0,002685	-4,698609	protein_coding	SH3RF1	SH3 domain containing ring finger 1 [Source:HGNC Symbol;Acc:HGNC:17650]	4
ENSG00000132879	3,577317	1,36612	0,000204	0,002708	4,694017	protein_coding	FBXO44	F-box protein 44 [Source:HGNC Symbol;Acc:HGNC:24847]	1
ENSG00000212993	-1,844913	2,583023	0,000204	0,002708	4,693751	protein_coding	POU5F1B	POU class 5 homeobox 1B [Source:HGNC Symbol;Acc:HGNC:9223]	8
ENSG00000136868	5,898134	-1,401992	0,000205	0,002708	-4,69343	protein_coding	SLC31A1	solute carrier family 31 member 1 [Source:HGNC Symbol;Acc:HGNC:11016]	9
ENSG00000188042	6,246795	-1,275398	0,000205	0,002713	-4,692111	protein_coding	ARL4C	ADP ribosylation factor like GTPase 4C [Source:HGNC Symbol;Acc:HGNC:698]	2
ENSG00000178188	5,33312	0,728675	0,000207	0,002735	4,688041	protein_coding	SH2B1	SH2B adaptor protein 1 [Source:HGNC Symbol;Acc:HGNC:30417]	16
ENSG00000146250	3,221046	-3,57628	0,000208	0,002744	-4,686084	protein_coding	PRSS35	serine protease 35 [Source:HGNC Symbol;Acc:HGNC:21387]	6
ENSG00000138193	4,623917	1,862312	0,000208	0,002747	4,685157	protein_coding	PLCE1	phospholipase C epsilon 1 [Source:HGNC Symbol;Acc:HGNC:17175]	10
ENSG00000214279	1,041138	1,79235	0,000209	0,00275	4,684248	protein_coding	SCART1	scavenger receptor family member expressed on T cells 1 [Source:HGNC Symbol;Acc:HGNC:32411]	10
ENSG00000204287	4,624026	3,952562	0,000209	0,002754	4,682959	protein_coding	HLA-DRA	major histocompatibility complex, class II, DR alpha [Source:HGNC Symbol;Acc:HGNC:4947]	6
ENSG00000108175	7,849797	-0,690595	0,000209	0,002754	-4,682805	protein_coding	ZMIZ1	zinc finger MIZ-type containing 1 [Source:HGNC Symbol;Acc:HGNC:16493]	10
ENSG00000204388	5,183833	1,687234	0,00021	0,002758	4,68178	protein_coding	HSPA1B	heat shock protein family A (Hsp70) member 1B [Source:HGNC Symbol;Acc:HGNC:5233]	6
ENSG00000171051	-0,410962	4,675043	0,000211	0,002767	4,679866	protein_coding	FPR1	formyl peptide receptor 1 [Source:HGNC Symbol;Acc:HGNC:3826]	19
ENSG00000171204	3,626772	-0,935545	0,000211	0,002775	-4,678204	protein_coding	TMEM126E	transmembrane protein 126B [Source:HGNC Symbol;Acc:HGNC:30883]	11
ENSG00000140319	7,464777	-0,994971	0,000212	0,00278	-4,677022	protein_coding	SRP14	signal recognition particle 14 [Source:HGNC Symbol;Acc:HGNC:11299]	15
ENSG00000138166	5,686257	-1,562349	0,000212	0,002784	-4,675961	protein_coding	DUSP5	dual specificity phosphatase 5 [Source:HGNC Symbol;Acc:HGNC:3071]	10
ENSG00000069482	-0,147313	-2,791106	0,000213	0,002791	-4,674322	protein_coding	GAL	galanin and GMAP prepropeptide [Source:HGNC Symbol;Acc:HGNC:4114]	11
ENSG00000239306	5,323951	1,009935	0,000214	0,002795	4,673239	protein_coding	RBM14	RNA binding motif protein 14 [Source:HGNC Symbol;Acc:HGNC:14219]	11
ENSG00000187446	5,93312	-1,016813	0,000214	0,002795	-4,67293	protein_coding	CHP1	calcineurin like EF-hand protein 1 [Source:HGNC Symbol;Acc:HGNC:17433]	15
ENSG00000163697	6,55929	-0,98158	0,000214	0,002796	-4,672342	protein_coding	APBB2	amyloid beta precursor protein binding family B member 2 [Source:HGNC Symbol;Acc:HGNC:582]	4
ENSG00000180739	1,454956	-1,520958	0,000215	0,002801	-4,671183	protein_coding	S1PR5	sphingosine-1-phosphate receptor 5 [Source:HGNC Symbol;Acc:HGNC:14299]	19
ENSG00000185522	1,348536	1,95219	0,000215	0,002801	4,670802	protein_coding	LMNTD2	lamin tail domain containing 2 [Source:HGNC Symbol;Acc:HGNC:28561]	11
ENSG00000178852	3,042887	1,291752	0,000215	0,002802	4,670315	protein_coding	EFCAB13	EF-hand calcium binding domain 13 [Source:HGNC Symbol;Acc:HGNC:26864]	17
ENSG00000183484	0,265129	2,65715	0,000217	0,002825	4,665785	protein_coding	GPR132	G protein-coupled receptor 132 [Source:HGNC Symbol;Acc:HGNC:17482]	14
ENSG00000004468	0,127587	3,279647	0,000218	0,002825	4,664848	protein_coding	CD38	CD38 molecule [Source:HGNC Symbol;Acc:HGNC:1667]	4
ENSG00000139372	4,958574	-0,892298	0,000217	0,002825	-4,665365	protein_coding	TDG	thymine DNA glycosylase [Source:HGNC Symbol;Acc:HGNC:11700]	12
ENSG00000134531	6,026141	-2,651654	0,000217	0,002825	-4,66522	protein_coding	EMP1	epithelial membrane protein 1 [Source:HGNC Symbol;Acc:HGNC:3333]	12
ENSG00000254521	-2,126834	3,391415	0,000219	0,002841	4,661514	protein_coding	SIGLEC12	sialic acid binding Ig like lectin 12 [Source:HGNC Symbol;Acc:HGNC:15482]	19
ENSG00000085733	8,105773	-0,946942	0,000219	0,002841	-4,661671	protein_coding	CTTN	cortactin [Source:HGNC Symbol;Acc:HGNC:3338]	11
ENSG00000140743	5,148262	-1,149928	0,000219	0,002844	-4,660618	protein_coding	CDR2	cerebellar degeneration related protein 2 [Source:HGNC Symbol;Acc:HGNC:1799]	16
ENSG00000205323	0,629491	1,555728	0,00022	0,002849	4,659504	protein_coding	SARNP	SAP domain containing ribonucleoprotein [Source:HGNC Symbol;Acc:HGNC:24432]	12
ENSG00000087086	10,16568	-1,62392	0,00022	0,00285	-4,658592	protein_coding	FTL	ferritin light chain [Source:HGNC Symbol;Acc:HGNC:3999]	19
ENSG00000198612	5,04083	-0,735288	0,00022	0,00285	-4,658542	protein_coding	COPS8	COP9 signalosome subunit 8 [Source:HGNC Symbol;Acc:HGNC:24335]	2
ENSG00000148411	5,377362	-0,896878	0,000221	0,002858	-4,656893	protein_coding	NACC2	NACC family member 2 [Source:HGNC Symbol;Acc:HGNC:23846]	9
ENSG00000103429	5,290207	-0,693557	0,000221	0,002858	-4,656467	protein_coding	BFAR	bifunctional apoptosis regulator [Source:HGNC Symbol;Acc:HGNC:17613]	16
ENSG00000160185	-1,936719	2,907073	0,000222	0,002861	4,655268	protein_coding	UBASH3A	ubiquitin associated and SH3 domain containing A [Source:HGNC Symbol;Acc:HGNC:12462]	21
ENSG00000164713	5,215212	-0,79813	0,000222	0,002861	-4,654921	protein_coding	BRI3	brain protein I3 [Source:HGNC Symbol;Acc:HGNC:1109]	7
ENSG00000134744	6,350442	0,644228	0,000222	0,002861	4,654957	protein_coding	TUT4	terminal uridylyl transferase 4 [Source:HGNC Symbol;Acc:HGNC:28981]	1
ENSG00000110786	-2,326201	3,498579	0,000223	0,002863	4,653944	protein_coding	PTPN5	protein tyrosine phosphatase non-receptor type 5 [Source:HGNC Symbol;Acc:HGNC:9657]	11
ENSG00000162604	4,790742	-0,694736	0,000223	0,002863	-4,653859	protein_coding	TM2D1	TM2 domain containing 1 [Source:HGNC Symbol;Acc:HGNC:24142]	1
ENSG00000069493	2,349954	2,098777	0,000223	0,002863	4,653232	protein_coding	CLEC2D	C-type lectin domain family 2 member D [Source:HGNC Symbol;Acc:HGNC:14351]	12
ENSG00000119772	6,178303	1,062799	0,000223	0,002863	4,653085	protein_coding	DNMT3A	DNA methyltransferase 3 alpha [Source:HGNC Symbol;Acc:HGNC:2978]	2
ENSG00000162931	-1,191177	2,771036	0,000226	0,002889	4,647776	protein_coding	TRIM17	tripartite motif containing 17 [Source:HGNC Symbol;Acc:HGNC:13430]	1
ENSG00000103365	6,048674	0,798133	0,000226	0,002889	4,647709	protein_coding	GGA2	golgi associated, gamma adaptin ear containing, ARF binding protein 2 [Source:HGNC Symbol;Acc:HGNC:16064]	16
ENSG00000114573	6,753605	-0,931091	0,000225	0,002889	-4,648001	protein_coding	ATP6V1A	ATPase H+ transporting V1 subunit A [Source:HGNC Symbol;Acc:HGNC:851]	3
ENSG00000100418	5,25007	-0,75203	0,000226	0,002894	-4,646552	protein_coding	DESI1	desumoylating isopeptidase 1 [Source:HGNC Symbol;Acc:HGNC:24577]	22
ENSG00000120071	6,744322	0,801325	0,000226	0,002895	4,64603	protein_coding	KANSL1	KAT8 regulatory NSL complex subunit 1 [Source:HGNC Symbol;Acc:HGNC:24565]	17
ENSG00000104951	1,621997	2,767842	0,000228	0,002904	4,6438	protein_coding	IL4I1	interleukin 4 induced 1 [Source:HGNC Symbol;Acc:HGNC:19094]	19
ENSG00000175283	3,871791	-1,079419	0,000227	0,002904	-4,643914	protein_coding	DOLK	dolichol kinase [Source:HGNC Symbol;Acc:HGNC:23406]	9
ENSG00000156253	3,594672	-1,243489	0,000228	0,002905	-4,642867	protein_coding	RWDD2B	RWD domain containing 2B [Source:HGNC Symbol;Acc:HGNC:1302]	21

ENSG00000167434	-2,663091	3,470557	0,000228	0,002905	4,643161	protein_coding	CA4	carbonic anhydrase 4 [Source:HGNC Symbol;Acc:HGNC:1375]	17
ENSG00000120705	6,980649	-0,632164	0,000229	0,002917	-4,640708	protein_coding	ETF1	eukaryotic translation termination factor 1 [Source:HGNC Symbol;Acc:HGNC:3477]	5
ENSG00000169446	4,97942	-0,890358	0,000229	0,002918	-4,639754	protein_coding	MMGT1	membrane magnesium transporter 1 [Source:HGNC Symbol;Acc:HGNC:28100]	X
ENSG00000132199	5,495855	1,326639	0,000229	0,002918	4,639961	protein_coding	ENOSF1	enolase superfamily member 1 [Source:HGNC Symbol;Acc:HGNC:30365]	18
ENSG00000166451	4,059924	-0,987141	0,00023	0,002923	-4,63864	protein_coding	CENPN	centromere protein N [Source:HGNC Symbol;Acc:HGNC:30873]	16
ENSG00000005844	1,454795	3,843267	0,000231	0,002929	4,636542	protein_coding	ITGAL	integrin subunit alpha L [Source:HGNC Symbol;Acc:HGNC:6148]	16
ENSG00000126062	5,221958	-0,640905	0,000231	0,002929	-4,636826	protein_coding	TMEM115	transmembrane protein 115 [Source:HGNC Symbol;Acc:HGNC:30055]	3
ENSG00000001497	5,566445	0,878511	0,000231	0,002929	4,636843	protein_coding	LAS1L	LAS1 like ribosome biogenesis factor [Source:HGNC Symbol;Acc:HGNC:25726]	X
ENSG00000184007	7,395555	-0,925034	0,000232	0,002936	-4,635001	protein_coding	PTP4A2	protein tyrosine phosphatase 4A2 [Source:HGNC Symbol;Acc:HGNC:9635]	1
ENSG00000123685	1,224358	1,588339	0,000232	0,002939	4,633871	protein_coding	BATF3	basic leucine zipper ATF-like transcription factor 3 [Source:HGNC Symbol;Acc:HGNC:28915]	1
ENSG00000163359	11,11395	-2,4899	0,000232	0,002939	-4,633762	protein_coding	COL6A3	collagen type VI alpha 3 chain [Source:HGNC Symbol;Acc:HGNC:2213]	2
ENSG00000151693	6,298525	-1,341201	0,000233	0,002939	-4,633554	protein_coding	ASAP2	ArfGAP with SH3 domain, ankyrin repeat and PH domain 2 [Source:HGNC Symbol;Acc:HGNC:2721]	2
ENSG00000130300	3,261176	5,489417	0,000234	0,002954	4,630128	protein_coding	PLVAP	plasmalemma vesicle associated protein [Source:HGNC Symbol;Acc:HGNC:13635]	19
ENSG00000150787	4,194858	-0,864735	0,000234	0,002954	-4,630702	protein_coding	PTS	6-pyruvoyltetrahydropterin synthase [Source:HGNC Symbol;Acc:HGNC:9689]	11
ENSG00000109919	5,627791	-0,695727	0,000234	0,002954	-4,630049	protein_coding	MTCH2	mitochondrial carrier 2 [Source:HGNC Symbol;Acc:HGNC:17587]	11
ENSG00000126698	5,794549	-0,735526	0,000235	0,002954	-4,629625	protein_coding	DNAJC8	DnaJ heat shock protein family (Hsp40) member C8 [Source:HGNC Symbol;Acc:HGNC:15470]	1
ENSG00000083312	7,529959	-0,690282	0,000235	0,002961	-4,628227	protein_coding	TNPO1	transportin 1 [Source:HGNC Symbol;Acc:HGNC:6401]	5
ENSG00000058085	5,555766	-2,646837	0,000235	0,002961	-4,627842	protein_coding	LAMC2	laminin subunit gamma 2 [Source:HGNC Symbol;Acc:HGNC:6493]	1
ENSG00000164808	5,424733	0,773464	0,000236	0,002962	4,62723	protein_coding	SPIDR	scaffold protein involved in DNA repair [Source:HGNC Symbol;Acc:HGNC:28971]	8
ENSG00000122884	6,773523	-1,079063	0,000236	0,00297	-4,625677	protein_coding	P4HA1	prolyl 4-hydroxylase subunit alpha 1 [Source:HGNC Symbol;Acc:HGNC:8546]	10
ENSG00000165915	5,691856	-0,855774	0,000238	0,002983	-4,62323	protein_coding	SLC39A13	solute carrier family 39 member 13 [Source:HGNC Symbol;Acc:HGNC:20859]	11
ENSG00000168026	2,959801	1,530601	0,000239	0,002991	4,621313	protein_coding	TTC21A	tetratricopeptide repeat domain 21A [Source:HGNC Symbol;Acc:HGNC:30761]	3
ENSG00000163762	-0,741439	3,28419	0,000239	0,002991	4,621374	protein_coding	TM4SF18	transmembrane 4 L six family member 18 [Source:HGNC Symbol;Acc:HGNC:25181]	3
ENSG00000025708	4,249923	1,871642	0,000239	0,002993	4,620282	protein_coding	TYMP	thymidine phosphorylase [Source:HGNC Symbol;Acc:HGNC:3148]	22
ENSG00000110768	5,478404	-0,765024	0,000239	0,002993	-4,62062	protein_coding	GTF2H1	general transcription factor IIH subunit 1 [Source:HGNC Symbol;Acc:HGNC:4655]	11
ENSG00000164989	3,392203	1,504922	0,000239	0,002993	4,61982	protein_coding	CCDC171	coiled-coil domain containing 171 [Source:HGNC Symbol;Acc:HGNC:29828]	9
ENSG00000167210	-0,402768	3,67043	0,00024	0,002999	4,618576	protein_coding	LOXHD1	lipoxygenase homology domains 1 [Source:HGNC Symbol;Acc:HGNC:26521]	18
ENSG00000183397	-0,364063	2,580317	0,000241	0,003002	4,617736	protein_coding	C19orf71	chromosome 19 open reading frame 71 [Source:HGNC Symbol;Acc:HGNC:34496]	19
ENSG00000116711	3,010811	-3,937614	0,000242	0,003012	-4,615554	protein_coding	PLA2G4A	phospholipase A2 group IVA [Source:HGNC Symbol;Acc:HGNC:9035]	1
ENSG00000132357	3,79114	-1,964995	0,000242	0,003012	-4,615761	protein_coding	CARD6	caspase recruitment domain family member 6 [Source:HGNC Symbol;Acc:HGNC:16394]	5
ENSG00000100836	5,676139	0,869386	0,000243	0,003019	4,613681	protein_coding	PABPN1	poly(A) binding protein nuclear 1 [Source:HGNC Symbol;Acc:HGNC:8565]	14
ENSG00000007202	8,210947	-0,601499	0,000243	0,003019	-4,613906	protein_coding	KIAA0100	KIAA0100 [Source:HGNC Symbol;Acc:HGNC:28960]	17
ENSG00000254732	-0,759433	1,898256	0,000243	0,003023	4,612484	protein_coding	AP001931.	novel protein, C11orf31-CTNND1 readthrough	11
ENSG00000065029	4,855908	0,885914	0,000243	0,003023	4,612322	protein_coding	ZNF76	zinc finger protein 76 [Source:HGNC Symbol;Acc:HGNC:13149]	6
ENSG00000181472	3,977731	-1,111347	0,000244	0,003026	-4,61161	protein_coding	ZBTB2	zinc finger and BTB domain containing 2 [Source:HGNC Symbol;Acc:HGNC:20868]	6
ENSG00000107551	4,126238	2,27042	0,000245	0,003029	4,610069	protein_coding	RASSF4	Ras association domain family member 4 [Source:HGNC Symbol;Acc:HGNC:20793]	10
ENSG00000110274	6,225013	0,804716	0,000244	0,003029	4,61071	protein_coding	CEP164	centrosomal protein 164 [Source:HGNC Symbol;Acc:HGNC:29182]	11
ENSG00000111229	6,771523	-1,067803	0,000244	0,003029	-4,610408	protein_coding	ARPC3	actin related protein 2/3 complex subunit 3 [Source:HGNC Symbol;Acc:HGNC:706]	12
ENSG00000162627	4,545366	-0,92373	0,000245	0,003033	-4,609087	protein_coding	SNX7	sorting nexin 7 [Source:HGNC Symbol;Acc:HGNC:14971]	1
ENSG00000141577	5,096755	1,019253	0,000246	0,003039	4,607827	protein_coding	CEP131	centrosomal protein 131 [Source:HGNC Symbol;Acc:HGNC:29511]	17
ENSG00000275464	-2,153421	2,234954	0,000246	0,003044	4,606658	protein_coding	FP565260.:	periodic tryptophan protein 2 homolog [Source:NCBI gene (formerly Entrezgene);Acc:102724159]	21
ENSG00000100307	4,525952	1,19875	0,000247	0,003051	4,605283	protein_coding	CBX7	chromobox 7 [Source:HGNC Symbol;Acc:HGNC:1557]	22
ENSG00000150551	2,699702	-3,171999	0,000248	0,003057	-4,603225	protein_coding	LYPD1	LY6/PLAUR domain containing 1 [Source:HGNC Symbol;Acc:HGNC:28431]	2
ENSG00000221869	5,437098	2,428066	0,000248	0,003057	4,603549	protein_coding	CEBPD	CCAAT enhancer binding protein delta [Source:HGNC Symbol;Acc:HGNC:1835]	8
ENSG00000136560	5,42493	-0,817739	0,000248	0,003057	-4,603798	protein_coding	TANK	TRAF family member associated NFKB activator [Source:HGNC Symbol;Acc:HGNC:11562]	2
ENSG00000255423	1,113588	1,624064	0,000249	0,003063	4,601886	protein_coding	EBLN2	endogenous Bornavirus like nucleoprotein 2 [Source:HGNC Symbol;Acc:HGNC:25493]	3
ENSG00000107779	5,4025	-1,011289	0,00025	0,003073	-4,600153	protein_coding	BMPR1A	bone morphogenetic protein receptor type 1A [Source:HGNC Symbol;Acc:HGNC:1076]	10
ENSG00000196937	5,629468	-0,846297	0,000251	0,003086	-4,597721	protein_coding	FAM3C	FAM3 metabolism regulating signaling molecule C [Source:HGNC Symbol;Acc:HGNC:18664]	7
ENSG00000133392	4,422957	5,248077	0,000251	0,003088	4,597116	protein_coding	MYH11	myosin heavy chain 11 [Source:HGNC Symbol;Acc:HGNC:7569]	16
ENSG00000130844	5,331422	1,445127	0,000252	0,003094	4,59581	protein_coding	ZNF331	zinc finger protein 331 [Source:HGNC Symbol;Acc:HGNC:15489]	19
ENSG00000231887	1,34499	1,645652	0,000253	0,003098	4,594494	protein_coding	PRH1	proline rich protein HaeIII subfamily 1 [Source:HGNC Symbol;Acc:HGNC:9366]	12

ENSG00000109971	9,881326	-0,832002	0,000253	0,003098	-4,59451	protein_coding	HSPA8	heat shock protein family A (Hsp70) member 8 [Source:HGNC Symbol;Acc:HGNC:5241]	11
ENSG00000204472	1,76672	3,938881	0,000253	0,003098	4,594108	protein_coding	AIF1	allograft inflammatory factor 1 [Source:HGNC Symbol;Acc:HGNC:352]	6
ENSG00000176435	0,840411	3,747802	0,000254	0,003105	4,592845	protein_coding	CLEC14A	C-type lectin domain containing 14A [Source:HGNC Symbol;Acc:HGNC:19832]	14
ENSG00000152894	6,696513	-1,811225	0,000254	0,003108	-4,591959	protein_coding	PTPRK	protein tyrosine phosphatase receptor type K [Source:HGNC Symbol;Acc:HGNC:9674]	6
ENSG00000178209	10,45118	-1,119418	0,000255	0,003114	-4,590731	protein_coding	PLEC	plectin [Source:HGNC Symbol;Acc:HGNC:9069]	8
ENSG00000167261	-0,70105	3,720482	0,000255	0,003116	4,590053	protein_coding	DPEP2	dipeptidase 2 [Source:HGNC Symbol;Acc:HGNC:23028]	16
ENSG00000204577	-1,377646	3,002074	0,000255	0,003117	4,589566	protein_coding	LILRB3	leukocyte immunoglobulin like receptor B3 [Source:HGNC Symbol;Acc:HGNC:6607]	19
ENSG00000006451	6,381892	-0,828532	0,000256	0,003121	-4,588569	protein_coding	RALA	RAS like proto-oncogene A [Source:HGNC Symbol;Acc:HGNC:9839]	7
ENSG00000091039	6,982236	-1,009038	0,000256	0,003121	-4,588292	protein_coding	OSBPL8	oxysterol binding protein like 8 [Source:HGNC Symbol;Acc:HGNC:16396]	12
ENSG00000288534	-0,384121	2,580318	0,000257	0,003124	4,58744	protein_coding	AP001931.	TMX2-CTNND1 readthrough (NMD candidate)	11
ENSG00000115091	7,548383	-1,073305	0,000257	0,003124	-4,587166	protein_coding	ACTR3	actin related protein 3 [Source:HGNC Symbol;Acc:HGNC:170]	2
ENSG00000187735	6,385446	-0,800581	0,000257	0,003125	-4,58663	protein_coding	TCEA1	transcription elongation factor A1 [Source:HGNC Symbol;Acc:HGNC:11612]	8
ENSG00000013523	5,260559	1,152422	0,000259	0,003144	4,583209	protein_coding	ANGEL1	angel homolog 1 [Source:HGNC Symbol;Acc:HGNC:19961]	14
ENSG00000113845	5,322611	-0,738881	0,000259	0,003144	-4,582703	protein_coding	TIMMDC1	translocase of inner mitochondrial membrane domain containing 1 [Source:HGNC Symbol;Acc:HGNC:1321]	3
ENSG00000171862	7,457446	-0,71546	0,000259	0,003144	-4,582972	protein_coding	PTEN	phosphatase and tensin homolog [Source:HGNC Symbol;Acc:HGNC:9588]	10
ENSG00000103091	4,836869	0,734703	0,00026	0,003148	4,581814	protein_coding	WDR59	WD repeat domain 59 [Source:HGNC Symbol;Acc:HGNC:25706]	16
ENSG00000142748	-2,432285	3,213205	0,00026	0,003154	4,580535	protein_coding	FCN3	ficolin 3 [Source:HGNC Symbol;Acc:HGNC:3625]	1
ENSG00000196664	-0,707516	3,573123	0,000261	0,003161	4,57916	protein_coding	TLR7	toll like receptor 7 [Source:HGNC Symbol;Acc:HGNC:15631]	X
ENSG00000188981	-0,04942	2,383449	0,000261	0,003161	4,578803	protein_coding	MSANTD1	Myb/SANT DNA binding domain containing 1 [Source:HGNC Symbol;Acc:HGNC:33741]	4
ENSG00000168283	4,58129	-0,897966	0,000262	0,003168	-4,577505	protein_coding	BMI1	BMI1 proto-oncogene, polycomb ring finger [Source:HGNC Symbol;Acc:HGNC:1066]	10
ENSG00000168487	5,816739	-1,482529	0,000263	0,00317	-4,576419	protein_coding	BMP1	bone morphogenetic protein 1 [Source:HGNC Symbol;Acc:HGNC:1067]	8
ENSG00000100234	8,278342	-2,588677	0,000263	0,00317	-4,576618	protein_coding	TIMP3	TIMP metallopeptidase inhibitor 3 [Source:HGNC Symbol;Acc:HGNC:11822]	22
ENSG00000100364	6,452239	-0,887499	0,000264	0,003177	-4,575131	protein_coding	KIAA0930	KIAA0930 [Source:HGNC Symbol;Acc:HGNC:1314]	22
ENSG00000110324	3,119798	3,138675	0,000264	0,003183	4,573811	protein_coding	IL10RA	interleukin 10 receptor subunit alpha [Source:HGNC Symbol;Acc:HGNC:5964]	11
ENSG00000186409	2,939924	1,929654	0,000265	0,003184	4,573388	protein_coding	CCDC30	coiled-coil domain containing 30 [Source:HGNC Symbol;Acc:HGNC:26103]	1
ENSG00000180817	5,331898	-0,935543	0,000265	0,003185	-4,572845	protein_coding	PPA1	inorganic pyrophosphatase 1 [Source:HGNC Symbol;Acc:HGNC:9226]	10
ENSG00000172575	0,348061	2,93454	0,000267	0,003203	4,569877	protein_coding	RASGRP1	RAS guanyl releasing protein 1 [Source:HGNC Symbol;Acc:HGNC:9878]	15
ENSG00000104388	7,044718	-0,827112	0,000268	0,003218	-4,567388	protein_coding	RAB2A	RAB2A, member RAS oncogene family [Source:HGNC Symbol;Acc:HGNC:9763]	8
ENSG00000160856	-2,313395	4,182137	0,000269	0,003221	4,566214	protein_coding	FCRL3	Fc receptor like 3 [Source:HGNC Symbol;Acc:HGNC:18506]	1
ENSG00000205339	7,616859	-0,813902	0,000268	0,003221	-4,566435	protein_coding	IPO7	importin 7 [Source:HGNC Symbol;Acc:HGNC:9852]	11
ENSG00000174840	5,320017	-0,590439	0,000269	0,003224	-4,565428	protein_coding	PDE12	phosphodiesterase 12 [Source:HGNC Symbol;Acc:HGNC:25386]	3
ENSG00000130985	7,969864	-0,618413	0,00027	0,003235	-4,563602	protein_coding	UBA1	ubiquitin like modifier activating enzyme 1 [Source:HGNC Symbol;Acc:HGNC:12469]	X
ENSG00000172172	4,112519	-0,837037	0,00027	0,003236	-4,563052	protein_coding	MRPL13	mitochondrial ribosomal protein L13 [Source:HGNC Symbol;Acc:HGNC:14278]	8
ENSG00000137996	5,486384	-0,743681	0,000271	0,00324	-4,562078	protein_coding	RTCA	RNA 3'-terminal phosphate cyclase [Source:HGNC Symbol;Acc:HGNC:17981]	1
ENSG00000004777	4,341673	2,344312	0,000271	0,003242	4,561534	protein_coding	ARHGAP33	Rho GTPase activating protein 33 [Source:HGNC Symbol;Acc:HGNC:23085]	19
ENSG00000079263	0,403556	3,709265	0,000274	0,003265	4,556436	protein_coding	SP140	SP140 nuclear body protein [Source:HGNC Symbol;Acc:HGNC:17133]	2
ENSG00000116815	2,705095	-1,269383	0,000274	0,003265	-4,556737	protein_coding	CD58	CD58 molecule [Source:HGNC Symbol;Acc:HGNC:1688]	1
ENSG00000277481	-1,681109	3,028029	0,000274	0,003265	4,557135	protein_coding	PKD1L3	polycystin 1 like 3, transient receptor potential channel interacting [Source:HGNC Symbol;Acc:HGNC:21716]	16
ENSG00000196262	8,334901	-0,957782	0,000274	0,003265	-4,55711	protein_coding	PPIA	peptidylprolyl isomerase A [Source:HGNC Symbol;Acc:HGNC:9253]	7
ENSG00000187555	7,339114	-0,595243	0,000274	0,003265	-4,556888	protein_coding	USP7	ubiquitin specific peptidase 7 [Source:HGNC Symbol;Acc:HGNC:12630]	16
ENSG00000152217	6,907228	1,450496	0,000275	0,003266	4,556047	protein_coding	SETBP1	SET binding protein 1 [Source:HGNC Symbol;Acc:HGNC:15573]	18
ENSG00000155659	0,888727	5,201194	0,000275	0,003269	4,554541	protein_coding	VSIG4	V-set and immunoglobulin domain containing 4 [Source:HGNC Symbol;Acc:HGNC:17032]	X
ENSG00000122861	5,798928	-3,805925	0,000275	0,003269	-4,5551	protein_coding	PLAU	plasminogen activator, urokinase [Source:HGNC Symbol;Acc:HGNC:9052]	10
ENSG00000001036	5,509805	-0,8826	0,000275	0,003269	-4,554514	protein_coding	FUCA2	alpha-L-fucosidase 2 [Source:HGNC Symbol;Acc:HGNC:4008]	6
ENSG00000091536	0,988235	1,94121	0,000279	0,003283	4,548873	protein_coding	MYO15A	myosin XVA [Source:HGNC Symbol;Acc:HGNC:7594]	17
ENSG00000179776	2,001757	5,731966	0,000279	0,003283	4,548377	protein_coding	CDH5	cadherin 5 [Source:HGNC Symbol;Acc:HGNC:1764]	16
ENSG00000163491	2,603211	1,513802	0,000278	0,003283	4,549647	protein_coding	NEK10	NIMA related kinase 10 [Source:HGNC Symbol;Acc:HGNC:18592]	3
ENSG00000187783	-1,193725	4,495101	0,000278	0,003283	4,549827	protein_coding	TMEM72	transmembrane protein 72 [Source:HGNC Symbol;Acc:HGNC:31658]	10
ENSG00000179403	4,573713	2,090794	0,000277	0,003283	4,551316	protein_coding	VWA1	von Willebrand factor A domain containing 1 [Source:HGNC Symbol;Acc:HGNC:30910]	1
ENSG00000108960	4,09194	-1,359078	0,000278	0,003283	-4,549406	protein_coding	MMD	monocyte to macrophage differentiation associated [Source:HGNC Symbol;Acc:HGNC:7153]	17
ENSG00000105607	4,284238	0,901976	0,000277	0,003283	4,551877	protein_coding	GCDH	glutaryl-CoA dehydrogenase [Source:HGNC Symbol;Acc:HGNC:4189]	19

ENSG00000112242	4,884168	-0,867951	0,000277	0,003283	-4,552165	protein_coding	E2F3	E2F transcription factor 3 [Source:HGNC Symbol;Acc:HGNC:3115]	6
ENSG00000183520	5,095562	-0,834677	0,000279	0,003283	-4,549334	protein_coding	UTP11	UTP11 small subunit processome component [Source:HGNC Symbol;Acc:HGNC:24329]	1
ENSG00000104142	5,241851	-0,792829	0,000277	0,003283	-4,551093	protein_coding	VPS18	VPS18 core subunit of CORVET and HOPS complexes [Source:HGNC Symbol;Acc:HGNC:15972]	15
ENSG00000149782	5,776121	-0,832596	0,000278	0,003283	-4,550112	protein_coding	PLCB3	phospholipase C beta 3 [Source:HGNC Symbol;Acc:HGNC:9056]	11
ENSG00000075618	6,239105	-1,807105	0,000279	0,003283	-4,548234	protein_coding	FSCN1	fascin actin-bundling protein 1 [Source:HGNC Symbol;Acc:HGNC:11148]	7
ENSG00000184432	7,837817	-0,88887	0,000279	0,003283	-4,548361	protein_coding	COPB2	COPI coat complex subunit beta 2 [Source:HGNC Symbol;Acc:HGNC:2232]	3
ENSG00000134202	5,010524	-1,127645	0,00028	0,003287	-4,547228	protein_coding	GSTM3	glutathione S-transferase mu 3 [Source:HGNC Symbol;Acc:HGNC:4635]	1
ENSG00000182578	4,371454	2,597497	0,00028	0,003288	4,54644	protein_coding	CSF1R	colony stimulating factor 1 receptor [Source:HGNC Symbol;Acc:HGNC:2433]	5
ENSG00000055070	6,77358	-0,860299	0,00028	0,003288	-4,546634	protein_coding	SZRD1	SUZ RNA binding domain containing 1 [Source:HGNC Symbol;Acc:HGNC:30232]	1
ENSG00000015285	1,75218	2,908386	0,000281	0,003292	4,545512	protein_coding	WAS	WASP actin nucleation promoting factor [Source:HGNC Symbol;Acc:HGNC:12731]	X
ENSG00000150477	4,312862	1,720214	0,000282	0,0033	4,544069	protein_coding	KIAA1328	KIAA1328 [Source:HGNC Symbol;Acc:HGNC:29248]	18
ENSG00000084623	6,48753	-0,953999	0,000284	0,003328	-4,539643	protein_coding	EIF3I	eukaryotic translation initiation factor 3 subunit I [Source:HGNC Symbol;Acc:HGNC:3272]	1
ENSG00000213585	7,017487	-0,756027	0,000284	0,003328	-4,539444	protein_coding	VDAC1	voltage dependent anion channel 1 [Source:HGNC Symbol;Acc:HGNC:12669]	5
ENSG00000198556	3,29039	1,458145	0,000285	0,003333	4,53843	protein_coding	ZNF789	zinc finger protein 789 [Source:HGNC Symbol;Acc:HGNC:27801]	7
ENSG00000162814	0,170248	2,024588	0,000286	0,003336	4,537713	protein_coding	SPATA17	spermatogenesis associated 17 [Source:HGNC Symbol;Acc:HGNC:25184]	1
ENSG00000136158	5,407266	-1,76574	0,000286	0,00334	-4,536512	protein_coding	SPRY2	sprouty RTK signaling antagonist 2 [Source:HGNC Symbol;Acc:HGNC:11270]	13
ENSG00000159840	6,952131	-1,710412	0,000286	0,00334	-4,536424	protein_coding	ZYX	zyxin [Source:HGNC Symbol;Acc:HGNC:13200]	7
ENSG00000119977	4,761589	-0,785393	0,000287	0,003341	-4,536031	protein_coding	TCTN3	tectonic family member 3 [Source:HGNC Symbol;Acc:HGNC:24519]	10
ENSG00000168439	7,051945	-0,656537	0,000288	0,00335	-4,534409	protein_coding	STIP1	stress induced phosphoprotein 1 [Source:HGNC Symbol;Acc:HGNC:11387]	11
ENSG00000146067	5,925444	1,037046	0,000288	0,003354	4,533485	protein_coding	FAM193B	family with sequence similarity 193 member B [Source:HGNC Symbol;Acc:HGNC:25524]	5
ENSG00000149357	5,401506	-0,605914	0,000289	0,003359	-4,532444	protein_coding	LAMTOR1	late endosomal/lysosomal adaptor, MAPK and MTOR activator 1 [Source:HGNC Symbol;Acc:HGNC:26068]	11
ENSG00000104129	4,196897	1,032502	0,000291	0,003379	4,529092	protein_coding	DNAJC17	DnaJ heat shock protein family (Hsp40) member C17 [Source:HGNC Symbol;Acc:HGNC:25556]	15
ENSG00000105971	5,366427	-1,907417	0,000291	0,003379	-4,529266	protein_coding	CAV2	caveolin 2 [Source:HGNC Symbol;Acc:HGNC:1528]	7
ENSG00000088808	5,243145	1,283893	0,000292	0,003391	4,52708	protein_coding	PPP1R13B	protein phosphatase 1 regulatory subunit 13B [Source:HGNC Symbol;Acc:HGNC:14950]	14
ENSG00000117505	6,162596	-0,845753	0,000293	0,003397	-4,525831	protein_coding	DR1	down-regulator of transcription 1 [Source:HGNC Symbol;Acc:HGNC:3017]	1
ENSG00000119446	4,84087	-0,768375	0,000293	0,003401	-4,525064	protein_coding	RBM18	RNA binding motif protein 18 [Source:HGNC Symbol;Acc:HGNC:28413]	9
ENSG00000270011	-2,096368	2,593921	0,000294	0,00341	4,523488	protein_coding	ZNF559-ZN	ZNF559-ZNF177 readthrough [Source:HGNC Symbol;Acc:HGNC:42964]	19
ENSG00000128000	5,138493	1,208835	0,000295	0,00342	4,521792	protein_coding	ZNF780B	zinc finger protein 780B [Source:HGNC Symbol;Acc:HGNC:33109]	19
ENSG00000114737	2,304647	2,576794	0,000296	0,003423	4,520299	protein_coding	CISH	cytokine inducible SH2 containing protein [Source:HGNC Symbol;Acc:HGNC:1984]	3
ENSG00000196616	-0,584064	5,588461	0,000296	0,003423	4,520903	protein_coding	ADH1B	alcohol dehydrogenase 1B (class I), beta polypeptide [Source:HGNC Symbol;Acc:HGNC:250]	4
ENSG00000110148	-1,355373	3,99987	0,000296	0,003423	4,520619	protein_coding	CCKBR	cholecystokinin B receptor [Source:HGNC Symbol;Acc:HGNC:1571]	11
ENSG00000144668	3,238411	3,709654	0,000297	0,003426	4,519585	protein_coding	ITGA9	integrin subunit alpha 9 [Source:HGNC Symbol;Acc:HGNC:6145]	3
ENSG00000145623	5,457019	-2,154113	0,000298	0,00344	-4,517398	protein_coding	OSMR	oncostatin M receptor [Source:HGNC Symbol;Acc:HGNC:8507]	5
ENSG00000155330	3,696994	-1,141713	0,000299	0,003444	-4,516521	protein_coding	C16orf87	chromosome 16 open reading frame 87 [Source:HGNC Symbol;Acc:HGNC:33754]	16
ENSG00000107201	1,694649	1,332166	0,0003	0,003448	4,51529	protein_coding	DDX58	DExD/H-box helicase 58 [Source:HGNC Symbol;Acc:HGNC:19102]	9
ENSG00000143847	3,06037	4,023333	0,0003	0,003448	4,515255	protein_coding	PPFIA4	PTPRF interacting protein alpha 4 [Source:HGNC Symbol;Acc:HGNC:9248]	1
ENSG00000126091	2,513899	1,770006	0,0003	0,00345	4,514719	protein_coding	ST3GAL3	ST3 beta-galactoside alpha-2,3-sialyltransferase 3 [Source:HGNC Symbol;Acc:HGNC:10866]	1
ENSG00000152213	-0,444634	3,275413	0,000301	0,003462	4,512784	protein_coding	ARL11	ADP ribosylation factor like GTPase 11 [Source:HGNC Symbol;Acc:HGNC:24046]	13
ENSG00000158077	-1,336978	2,941199	0,000303	0,003476	4,510513	protein_coding	NLRP14	NLR family pyrin domain containing 14 [Source:HGNC Symbol;Acc:HGNC:22939]	11
ENSG00000198899	11,69962	-1,636594	0,000303	0,003478	-4,509979	protein_coding	MT-ATP6	mitochondrially encoded ATP synthase membrane subunit 6 [Source:HGNC Symbol;Acc:HGNC:7414]	MT
ENSG00000163568	-2,499957	3,791482	0,000304	0,003483	4,50868	protein_coding	AIM2	absent in melanoma 2 [Source:HGNC Symbol;Acc:HGNC:357]	1
ENSG00000147874	4,937911	-0,700508	0,000304	0,003483	-4,508659	protein_coding	HAUS6	HAUS augmin like complex subunit 6 [Source:HGNC Symbol;Acc:HGNC:25948]	9
ENSG00000114735	4,412219	1,063969	0,000305	0,003494	4,50682	protein_coding	HEMK1	HemK methyltransferase family member 1 [Source:HGNC Symbol;Acc:HGNC:24923]	3
ENSG00000196693	4,565653	1,7056	0,000306	0,003506	4,504936	protein_coding	ZNF33B	zinc finger protein 33B [Source:HGNC Symbol;Acc:HGNC:13097]	10
ENSG00000033100	6,439234	-1,108427	0,000307	0,003508	-4,504317	protein_coding	CHPF2	chondroitin polymerizing factor 2 [Source:HGNC Symbol;Acc:HGNC:29270]	7
ENSG00000183726	5,344293	-0,966526	0,000309	0,003528	-4,501023	protein_coding	TMEM50A	transmembrane protein 50A [Source:HGNC Symbol;Acc:HGNC:30590]	1
ENSG00000129636	6,12253	-0,990462	0,000309	0,003528	-4,501016	protein_coding	ITFG1	integrin alpha FG-GAP repeat containing 1 [Source:HGNC Symbol;Acc:HGNC:30697]	16
ENSG00000164035	-0,302537	3,898763	0,00031	0,003537	4,49925	protein_coding	EMCN	endomucin [Source:HGNC Symbol;Acc:HGNC:16041]	4
ENSG00000125037	5,381411	-0,951491	0,00031	0,003537	-4,499126	protein_coding	EMC3	ER membrane protein complex subunit 3 [Source:HGNC Symbol;Acc:HGNC:23999]	3
ENSG00000121966	3,777057	3,363811	0,000311	0,003548	4,497388	protein_coding	CXCR4	C-X-C motif chemokine receptor 4 [Source:HGNC Symbol;Acc:HGNC:2561]	2
ENSG00000101557	6,487331	-0,869089	0,000314	0,003577	-4,493321	protein_coding	USP14	ubiquitin specific peptidase 14 [Source:HGNC Symbol;Acc:HGNC:12612]	18

ENSG00000163508	-1,846659	2,798416	0,000315	0,003587	4,491672	protein_coding	EOMES	eomesodermin [Source:HGNC Symbol;Acc:HGNC:3372]	3
ENSG00000178789	-1,007145	3,16089	0,000316	0,003596	4,48981	protein_coding	CD300LB	CD300 molecule like family member b [Source:HGNC Symbol;Acc:HGNC:30811]	17
ENSG00000269533	-1,682587	1,870639	0,000316	0,003596	4,489856	protein_coding	AC003002.	novel transcript	19
ENSG00000185896	7,990209	-0,820158	0,000317	0,0036	-4,489008	protein_coding	LAMP1	lysosomal associated membrane protein 1 [Source:HGNC Symbol;Acc:HGNC:6499]	13
ENSG00000104368	6,701344	-2,841071	0,000318	0,003604	-4,488207	protein_coding	PLAT	plasminogen activator, tissue type [Source:HGNC Symbol;Acc:HGNC:9051]	8
ENSG00000214194	3,952768	-1,01853	0,000318	0,003605	-4,487695	protein_coding	SMIM30	small integral membrane protein 30 [Source:HGNC Symbol;Acc:HGNC:48953]	7
ENSG00000115216	6,92025	-0,804628	0,000321	0,003633	-4,483753	protein_coding	NRBP1	nuclear receptor binding protein 1 [Source:HGNC Symbol;Acc:HGNC:7993]	2
ENSG00000099250	7,93469	-1,50251	0,000323	0,003655	-4,480707	protein_coding	NRP1	neuropilin 1 [Source:HGNC Symbol;Acc:HGNC:8004]	10
ENSG00000102128	-1,037917	2,359611	0,000323	0,003656	4,480181	protein_coding	RAB40AL	RAB40A like [Source:HGNC Symbol;Acc:HGNC:25410]	X
ENSG00000196878	4,761017	-2,386977	0,000324	0,003663	-4,478937	protein_coding	LAMB3	laminin subunit beta 3 [Source:HGNC Symbol;Acc:HGNC:6490]	1
ENSG00000139318	6,01812	-1,742344	0,000325	0,003669	-4,477963	protein_coding	DUSP6	dual specificity phosphatase 6 [Source:HGNC Symbol;Acc:HGNC:3072]	12
ENSG00000197580	0,699451	2,305003	0,000327	0,003688	4,475219	protein_coding	BCO2	beta-carotene oxygenase 2 [Source:HGNC Symbol;Acc:HGNC:18503]	11
ENSG00000133026	7,491992	1,138108	0,000327	0,003688	4,474792	protein_coding	MYH10	myosin heavy chain 10 [Source:HGNC Symbol;Acc:HGNC:7568]	17
ENSG00000140416	8,74942	-1,310454	0,000328	0,003696	-4,473564	protein_coding	TPM1	tropomyosin 1 [Source:HGNC Symbol;Acc:HGNC:12010]	15
ENSG00000169896	2,276212	2,855875	0,000328	0,003698	4,472888	protein_coding	ITGAM	integrin subunit alpha M [Source:HGNC Symbol;Acc:HGNC:6149]	16
ENSG00000162878	3,792431	2,245762	0,00033	0,003711	4,471025	protein_coding	PKDCC	protein kinase domain containing, cytoplasmic [Source:HGNC Symbol;Acc:HGNC:25123]	2
ENSG00000165996	1,401894	-2,330729	0,00033	0,003713	-4,470412	protein_coding	HACD1	3-hydroxyacyl-CoA dehydratase 1 [Source:HGNC Symbol;Acc:HGNC:9639]	10
ENSG00000006016	3,803309	-5,834999	0,000333	0,003742	-4,465846	protein_coding	CRLF1	cytokine receptor like factor 1 [Source:HGNC Symbol;Acc:HGNC:2364]	19
ENSG00000243279	3,052931	-1,233082	0,000333	0,003742	-4,466327	protein_coding	PRAF2	PRA1 domain family member 2 [Source:HGNC Symbol;Acc:HGNC:28911]	X
ENSG00000115993	6,815695	-1,037062	0,000333	0,003742	-4,466087	protein_coding	TRAK2	trafficking kinesin protein 2 [Source:HGNC Symbol;Acc:HGNC:13206]	2
ENSG00000124374	3,581487	2,122148	0,000334	0,003743	4,465406	protein_coding	PAIP2B	poly(A) binding protein interacting protein 2B [Source:HGNC Symbol;Acc:HGNC:29200]	2
ENSG00000197780	4,542824	-1,044173	0,000334	0,003746	-4,464704	protein_coding	TAF13	TATA-box binding protein associated factor 13 [Source:HGNC Symbol;Acc:HGNC:11546]	1
ENSG00000198870	-0,210277	1,464101	0,000334	0,003749	4,464067	protein_coding	STKLD1	serine/threonine kinase like domain containing 1 [Source:HGNC Symbol;Acc:HGNC:28669]	9
ENSG00000163406	0,723891	2,44509	0,000335	0,00375	4,463577	protein_coding	SLC15A2	solute carrier family 15 member 2 [Source:HGNC Symbol;Acc:HGNC:10921]	3
ENSG00000089876	5,473884	-0,947241	0,000336	0,003756	-4,462474	protein_coding	DHX32	DEAH-box helicase 32 (putative) [Source:HGNC Symbol;Acc:HGNC:16717]	10
ENSG00000274523	4,299115	-0,639859	0,000336	0,003762	-4,461497	protein_coding	RCC1L	RCC1 like [Source:HGNC Symbol;Acc:HGNC:14948]	7
ENSG00000169641	6,170069	-1,141426	0,000338	0,003776	-4,459402	protein_coding	LUZP1	leucine zipper protein 1 [Source:HGNC Symbol;Acc:HGNC:14985]	1
ENSG00000150753	7,865629	-0,876782	0,000339	0,003782	-4,458283	protein_coding	CCT5	chaperonin containing TCP1 subunit 5 [Source:HGNC Symbol;Acc:HGNC:1618]	5
ENSG00000175463	-0,594271	3,420751	0,00034	0,00379	4,456873	protein_coding	TBC1D10C	TBC1 domain family member 10C [Source:HGNC Symbol;Acc:HGNC:24702]	11
ENSG00000095739	4,933323	-1,566019	0,00034	0,00379	-4,456773	protein_coding	BAMBI	BMP and activin membrane bound inhibitor [Source:HGNC Symbol;Acc:HGNC:30251]	10
ENSG00000163812	5,6205	-0,646207	0,000341	0,003801	-4,455087	protein_coding	ZDHHC3	zinc finger DHHC-type palmitoyltransferase 3 [Source:HGNC Symbol;Acc:HGNC:18470]	3
ENSG00000259030	-1,683773	2,606574	0,000342	0,00381	4,45362	protein_coding	FPGT-TNNI	FPGT-TNNI3K readthrough [Source:HGNC Symbol;Acc:HGNC:42952]	1
ENSG00000187079	7,231418	-0,841399	0,000342	0,00381	-4,453304	protein_coding	TEAD1	TEA domain transcription factor 1 [Source:HGNC Symbol;Acc:HGNC:11714]	11
ENSG00000241404	-0,333154	1,823392	0,000343	0,003815	4,452427	protein_coding	EGFL8	EGF like domain multiple 8 [Source:HGNC Symbol;Acc:HGNC:13944]	6
ENSG00000142549	-0,700179	3,146309	0,000343	0,003816	4,451995	protein_coding	IGLON5	IgLON family member 5 [Source:HGNC Symbol;Acc:HGNC:34550]	19
ENSG00000186577	3,683949	-0,863627	0,000344	0,003819	-4,450957	protein_coding	SMIM29	small integral membrane protein 29 [Source:HGNC Symbol;Acc:HGNC:1340]	6
ENSG00000067208	6,025079	-0,861195	0,000344	0,003819	-4,451217	protein_coding	EVI5	ecotropic viral integration site 5 [Source:HGNC Symbol;Acc:HGNC:3501]	1
ENSG00000138685	3,868267	-3,845906	0,000345	0,003822	-4,449934	protein_coding	FGF2	fibroblast growth factor 2 [Source:HGNC Symbol;Acc:HGNC:3676]	4
ENSG00000135473	5,349241	1,217817	0,000345	0,003822	4,450223	protein_coding	PAN2	poly(A) specific ribonuclease subunit PAN2 [Source:HGNC Symbol;Acc:HGNC:20074]	12
ENSG00000152583	3,775736	5,066881	0,000345	0,003822	4,449613	protein_coding	SPARCL1	SPARC like 1 [Source:HGNC Symbol;Acc:HGNC:11220]	4
ENSG00000186407	-0,303742	3,981055	0,000346	0,003827	4,448648	protein_coding	CD300E	CD300e molecule [Source:HGNC Symbol;Acc:HGNC:28874]	17
ENSG00000143882	0,692631	-1,629164	0,000347	0,003828	-4,447596	protein_coding	ATP6V1C2	ATPase H+ transporting V1 subunit C2 [Source:HGNC Symbol;Acc:HGNC:18264]	2
ENSG00000069998	4,134534	0,95381	0,000347	0,003828	4,447173	protein_coding	HDHD5	haloacid dehalogenase like hydrolase domain containing 5 [Source:HGNC Symbol;Acc:HGNC:1843]	22
ENSG00000141480	4,744671	1,862406	0,000347	0,003828	4,447062	protein_coding	ARRB2	arrestin beta 2 [Source:HGNC Symbol;Acc:HGNC:712]	17
ENSG00000058729	4,611487	-0,648129	0,000346	0,003828	-4,447856	protein_coding	RIOK2	RIO kinase 2 [Source:HGNC Symbol;Acc:HGNC:18999]	5
ENSG00000103363	5,817638	-0,880493	0,000346	0,003828	-4,448073	protein_coding	ELOB	elongin B [Source:HGNC Symbol;Acc:HGNC:11619]	16
ENSG00000196230	9,007565	-1,016479	0,000348	0,00383	-4,445788	protein_coding	TUBB	tubulin beta class I [Source:HGNC Symbol;Acc:HGNC:20778]	6
ENSG00000134851	6,314698	-0,909385	0,000348	0,00383	-4,445769	protein_coding	TMEM165	transmembrane protein 165 [Source:HGNC Symbol;Acc:HGNC:30760]	4
ENSG00000114354	6,604718	-0,811227	0,000348	0,00383	-4,445802	protein_coding	TFG	trafficking from ER to golgi regulator [Source:HGNC Symbol;Acc:HGNC:11758]	3
ENSG00000125845	4,431546	-1,737849	0,000349	0,003836	-4,444799	protein_coding	BMP2	bone morphogenetic protein 2 [Source:HGNC Symbol;Acc:HGNC:1069]	20
ENSG00000265972	6,866088	2,054422	0,00035	0,003845	4,443375	protein_coding	TXNIP	thioredoxin interacting protein [Source:HGNC Symbol;Acc:HGNC:16952]	1

ENSG00000047849	8,139851	-0,941179	0,00035	0,003847	-4,442813	protein_coding	MAP4	microtubule associated protein 4 [Source:HGNC Symbol;Acc:HGNC:6862]	3
ENSG00000095261	5,266303	-0,573035	0,00035	0,003848	-4,442408	protein_coding	PSMD5	proteasome 26S subunit, non-ATPase 5 [Source:HGNC Symbol;Acc:HGNC:9563]	9
ENSG00000089169	-2,411046	2,851063	0,000352	0,00386	4,440583	protein_coding	RPH3A	rabphilin 3A [Source:HGNC Symbol;Acc:HGNC:17056]	12
ENSG00000244509	4,173478	-2,01435	0,000353	0,003867	-4,439413	protein_coding	APOBEC3C	apolipoprotein B mRNA editing enzyme catalytic subunit 3C [Source:HGNC Symbol;Acc:HGNC:17353]	22
ENSG00000251322	4,928576	1,980438	0,000353	0,003868	4,439011	protein_coding	SHANK3	SH3 and multiple ankyrin repeat domains 3 [Source:HGNC Symbol;Acc:HGNC:14294]	22
ENSG00000132424	7,42582	1,152242	0,000354	0,003874	4,438024	protein_coding	PNISR	PNN interacting serine and arginine rich protein [Source:HGNC Symbol;Acc:HGNC:21222]	6
ENSG00000256235	4,181384	-2,47332	0,000354	0,003876	-4,437489	protein_coding	SMIM3	small integral membrane protein 3 [Source:HGNC Symbol;Acc:HGNC:30248]	5
ENSG00000126247	7,40763	-0,783834	0,000354	0,003876	-4,437121	protein_coding	CAPNS1	calpain small subunit 1 [Source:HGNC Symbol;Acc:HGNC:1481]	19
ENSG00000106723	6,455125	-0,677299	0,000356	0,003888	-4,435428	protein_coding	SPIN1	spindlin 1 [Source:HGNC Symbol;Acc:HGNC:11243]	9
ENSG00000151881	3,127981	-0,954912	0,000357	0,00389	-4,434242	protein_coding	TMEM267	transmembrane protein 267 [Source:HGNC Symbol;Acc:HGNC:26139]	5
ENSG00000010278	6,921907	-3,023429	0,000356	0,00389	-4,434722	protein_coding	CD9	CD9 molecule [Source:HGNC Symbol;Acc:HGNC:1709]	12
ENSG00000137207	6,520193	-0,677039	0,000357	0,00389	-4,434306	protein_coding	YIPF3	Yip1 domain family member 3 [Source:HGNC Symbol;Acc:HGNC:21023]	6
ENSG00000172269	4,410999	-0,762109	0,000357	0,00389	-4,433898	protein_coding	DPAGT1	dolichyl-phosphate N-acetylglucosaminephosphotransferase 1 [Source:HGNC Symbol;Acc:HGNC:2995]	11
ENSG00000072694	0,363955	3,82979	0,000359	0,003893	4,431753	protein_coding	FCGR2B	Fc fragment of IgG receptor IIb [Source:HGNC Symbol;Acc:HGNC:3618]	1
ENSG00000189195	3,4986	1,086664	0,000359	0,003893	4,43173	protein_coding	BTBD8	BTB domain containing 8 [Source:HGNC Symbol;Acc:HGNC:21019]	1
ENSG00000152464	3,551536	-0,798338	0,000358	0,003893	-4,432298	protein_coding	RPP38	ribonuclease P/MRP subunit p38 [Source:HGNC Symbol;Acc:HGNC:30329]	10
ENSG00000104064	4,071221	-0,659701	0,000358	0,003893	-4,432332	protein_coding	GABPB1	GA binding protein transcription factor subunit beta 1 [Source:HGNC Symbol;Acc:HGNC:4074]	15
ENSG00000100023	5,582377	0,646439	0,000358	0,003893	4,432829	protein_coding	PPIL2	peptidylprolyl isomerase like 2 [Source:HGNC Symbol;Acc:HGNC:9261]	22
ENSG00000180370	6,990165	-0,914093	0,000358	0,003893	-4,432899	protein_coding	PAK2	p21 (RAC1) activated kinase 2 [Source:HGNC Symbol;Acc:HGNC:8591]	3
ENSG00000212126	-1,320642	2,704178	0,000359	0,003893	4,431307	protein_coding	TAS2R50	taste 2 receptor member 50 [Source:HGNC Symbol;Acc:HGNC:18882]	12
ENSG00000023892	2,590492	2,496484	0,00036	0,003896	4,430174	protein_coding	DEF6	DEF6 guanine nucleotide exchange factor [Source:HGNC Symbol;Acc:HGNC:2760]	6
ENSG00000239672	3,752389	-0,82083	0,00036	0,003896	-4,430015	protein_coding	NME1	NME/NM23 nucleoside diphosphate kinase 1 [Source:HGNC Symbol;Acc:HGNC:7849]	17
ENSG00000255529	4,034668	-0,934071	0,00036	0,003896	-4,429708	protein_coding	POLR2M	RNA polymerase II subunit M [Source:HGNC Symbol;Acc:HGNC:14862]	15
ENSG00000177889	5,788888	-0,719343	0,00036	0,003896	-4,43012	protein_coding	UBE2N	ubiquitin conjugating enzyme E2 N [Source:HGNC Symbol;Acc:HGNC:12492]	12
ENSG00000157764	6,355886	0,605228	0,00036	0,003896	4,429423	protein_coding	BRAF	B-Raf proto-oncogene, serine/threonine kinase [Source:HGNC Symbol;Acc:HGNC:1097]	7
ENSG00000169490	4,791899	-0,932804	0,000361	0,0039	-4,428471	protein_coding	TM2D2	TM2 domain containing 2 [Source:HGNC Symbol;Acc:HGNC:24127]	8
ENSG00000275023	7,326439	1,15335	0,000361	0,0039	4,428329	protein_coding	MLLT6	MLLT6, PHD finger containing [Source:HGNC Symbol;Acc:HGNC:7138]	17
ENSG00000175766	-3,02767	3,008364	0,000362	0,003907	4,42721	protein_coding	EIF4E1B	eukaryotic translation initiation factor 4E family member 1B [Source:HGNC Symbol;Acc:HGNC:33179]	5
ENSG00000046889	2,103298	3,112482	0,000363	0,00391	4,42654	protein_coding	PREX2	phosphatidylinositol-3,4,5-trisphosphate dependent Rac exchange factor 2 [Source:HGNC Symbol;Acc:HGNC:22950]	8
ENSG00000167770	5,34014	-0,558616	0,000363	0,00391	-4,426276	protein_coding	OTUB1	OTU deubiquitinase, ubiquitin aldehyde binding 1 [Source:HGNC Symbol;Acc:HGNC:23077]	11
ENSG00000007171	-1,329485	3,506111	0,000364	0,003922	4,424503	protein_coding	NOS2	nitric oxide synthase 2 [Source:HGNC Symbol;Acc:HGNC:7873]	17
ENSG00000205765	5,32823	-0,666975	0,000365	0,003924	-4,424006	protein_coding	C5orf51	chromosome 5 open reading frame 51 [Source:HGNC Symbol;Acc:HGNC:27750]	5
ENSG00000259332	-1,540221	2,77421	0,000365	0,003925	4,423505	protein_coding	ST20-MTHI	ST20-MTHFS readthrough [Source:HGNC Symbol;Acc:HGNC:44655]	15
ENSG00000138942	4,611612	-0,692511	0,000366	0,003934	-4,422186	protein_coding	RNF185	ring finger protein 185 [Source:HGNC Symbol;Acc:HGNC:26783]	22
ENSG00000110079	1,255685	3,31146	0,000368	0,003954	4,419557	protein_coding	MS4A4A	membrane spanning 4-domains A4A [Source:HGNC Symbol;Acc:HGNC:13371]	11
ENSG00000254415	-1,165511	2,817967	0,000369	0,003957	4,418834	protein_coding	SIGLEC14	sialic acid binding Ig like lectin 14 [Source:HGNC Symbol;Acc:HGNC:32926]	19
ENSG00000061656	2,265211	2,596421	0,000369	0,003961	4,418038	protein_coding	SPAG4	sperm associated antigen 4 [Source:HGNC Symbol;Acc:HGNC:11214]	20
ENSG00000105438	7,130457	-0,675019	0,000372	0,003983	-4,415207	protein_coding	KDELR1	KDEL endoplasmic reticulum protein retention receptor 1 [Source:HGNC Symbol;Acc:HGNC:6304]	19
ENSG00000187244	6,737694	1,784724	0,000372	0,003984	4,41481	protein_coding	BCAM	basal cell adhesion molecule (Lutheran blood group) [Source:HGNC Symbol;Acc:HGNC:6722]	19
ENSG00000273294	-1,558661	2,332481	0,000374	0,003999	4,412693	protein_coding	C1QTNF3-/	C1QTNF3-AMACR readthrough (NMD candidate) [Source:HGNC Symbol;Acc:HGNC:49198]	5
ENSG00000085721	5,146905	-0,640597	0,000374	0,004	-4,411991	protein_coding	RRN3	RRN3 homolog, RNA polymerase I transcription factor [Source:HGNC Symbol;Acc:HGNC:30346]	16
ENSG00000074319	5,250704	-0,755326	0,000374	0,004	-4,411989	protein_coding	TSG101	tumor susceptibility 101 [Source:HGNC Symbol;Acc:HGNC:15971]	11
ENSG00000177098	0,933529	2,096274	0,000375	0,00401	4,410597	protein_coding	SCN4B	sodium voltage-gated channel beta subunit 4 [Source:HGNC Symbol;Acc:HGNC:10592]	11
ENSG00000261408	-0,988647	1,919515	0,000377	0,00402	4,408769	protein_coding	TEN1-CDK3	TEN1-CDK3 readthrough (NMD candidate) [Source:HGNC Symbol;Acc:HGNC:44420]	17
ENSG00000095539	3,521786	1,499451	0,000377	0,00402	4,40902	protein_coding	SEMA4G	semaphorin 4G [Source:HGNC Symbol;Acc:HGNC:10735]	10
ENSG00000167604	2,928841	1,443725	0,000378	0,004024	4,407764	protein_coding	NFKBID	NFKB inhibitor delta [Source:HGNC Symbol;Acc:HGNC:15671]	19
ENSG00000128342	4,010881	-3,451824	0,000378	0,004024	-4,407742	protein_coding	LIF	LIF interleukin 6 family cytokine [Source:HGNC Symbol;Acc:HGNC:6596]	22
ENSG00000163645	-2,07546	2,350138	0,000379	0,004032	4,406359	protein_coding	ERICH6	glutamate rich 6 [Source:HGNC Symbol;Acc:HGNC:28602]	3
ENSG00000122644	4,237096	-1,193675	0,000379	0,004032	-4,406237	protein_coding	ARL4A	ADP ribosylation factor like GTPase 4A [Source:HGNC Symbol;Acc:HGNC:695]	7
ENSG00000135205	2,790843	1,671339	0,00038	0,004039	4,405102	protein_coding	CCDC146	coiled-coil domain containing 146 [Source:HGNC Symbol;Acc:HGNC:29296]	7
ENSG00000145777	0,865606	-3,6376	0,000381	0,004049	-4,403652	protein_coding	TSLP	thymic stromal lymphopoietin [Source:HGNC Symbol;Acc:HGNC:30743]	5

ENSG00000185088	5,599893	-1,057143	0,000382	0,004053	-4,402827	protein_coding	RPS27L	ribosomal protein S27 like [Source:HGNC Symbol;Acc:HGNC:18476]	15
ENSG00000203685	0,648046	2,72169	0,000382	0,004054	4,40218	protein_coding	STUM	stum, mechanosensory transduction mediator homolog [Source:HGNC Symbol;Acc:HGNC:30491]	1
ENSG00000117862	3,633083	-0,838972	0,000382	0,004054	-4,402398	protein_coding	TXNDC12	thioredoxin domain containing 12 [Source:HGNC Symbol;Acc:HGNC:24626]	1
ENSG00000101445	1,611807	4,121478	0,000383	0,004058	4,400804	protein_coding	PPP1R16B	protein phosphatase 1 regulatory subunit 16B [Source:HGNC Symbol;Acc:HGNC:15850]	20
ENSG00000255302	7,453434	-0,61095	0,000383	0,004058	-4,400955	protein_coding	EID1	EP300 interacting inhibitor of differentiation 1 [Source:HGNC Symbol;Acc:HGNC:1191]	15
ENSG00000140943	7,128384	-0,728353	0,000383	0,004058	-4,401098	protein_coding	MBTPS1	membrane bound transcription factor peptidase, site 1 [Source:HGNC Symbol;Acc:HGNC:15456]	16
ENSG00000095303	5,1637	-3,655761	0,000384	0,004059	-4,400322	protein_coding	PTGS1	prostaglandin-endoperoxide synthase 1 [Source:HGNC Symbol;Acc:HGNC:9604]	9
ENSG00000144043	5,46955	-0,57062	0,000384	0,004061	-4,399869	protein_coding	TEX261	testis expressed 261 [Source:HGNC Symbol;Acc:HGNC:30712]	2
ENSG00000114867	9,316359	-0,671835	0,000385	0,004063	-4,399024	protein_coding	EIF4G1	eukaryotic translation initiation factor 4 gamma 1 [Source:HGNC Symbol;Acc:HGNC:3296]	3
ENSG00000160209	6,924952	-0,610146	0,000385	0,004063	-4,399088	protein_coding	PDXK	pyridoxal kinase [Source:HGNC Symbol;Acc:HGNC:8819]	21
ENSG00000278637	2,816367	1,465917	0,000387	0,004082	4,396553	protein_coding	H4C1	H4 clustered histone 1 [Source:HGNC Symbol;Acc:HGNC:4781]	6
ENSG00000110844	4,964276	1,122728	0,000388	0,00409	4,395354	protein_coding	PRPF40B	pre-mRNA processing factor 40 homolog B [Source:HGNC Symbol;Acc:HGNC:25031]	12
ENSG00000108798	2,002949	2,339588	0,00039	0,004106	4,393262	protein_coding	ABI3	ABI family member 3 [Source:HGNC Symbol;Acc:HGNC:29859]	17
ENSG00000140386	5,630447	1,467002	0,000392	0,004128	4,390476	protein_coding	SCAPER	S-phase cyclin A associated protein in the ER [Source:HGNC Symbol;Acc:HGNC:13081]	15
ENSG00000147955	5,927224	-0,831729	0,000394	0,004142	-4,388613	protein_coding	SIGMAR1	sigma non-opioid intracellular receptor 1 [Source:HGNC Symbol;Acc:HGNC:8157]	9
ENSG00000117616	5,950399	0,906265	0,000396	0,004164	4,385835	protein_coding	RSRP1	arginine and serine rich protein 1 [Source:HGNC Symbol;Acc:HGNC:25234]	1
ENSG00000182180	5,438911	-0,5843	0,000396	0,004165	-4,385447	protein_coding	MRPS16	mitochondrial ribosomal protein S16 [Source:HGNC Symbol;Acc:HGNC:14048]	10
ENSG00000198929	3,19784	2,298859	0,000397	0,004167	4,384924	protein_coding	NOS1AP	nitric oxide synthase 1 adaptor protein [Source:HGNC Symbol;Acc:HGNC:16859]	1
ENSG00000198804	13,2875	-1,465198	0,000398	0,004176	-4,38355	protein_coding	MT-CO1	mitochondrially encoded cytochrome c oxidase I [Source:HGNC Symbol;Acc:HGNC:7419]	MT
ENSG00000161929	0,200848	3,448249	0,000398	0,004177	4,383125	protein_coding	SCIMP	SLP adaptor and CSK interacting membrane protein [Source:HGNC Symbol;Acc:HGNC:33504]	17
ENSG00000162373	0,889999	2,699356	0,000399	0,004179	4,382612	protein_coding	BEND5	BEN domain containing 5 [Source:HGNC Symbol;Acc:HGNC:25668]	1
ENSG00000163964	4,677183	-0,649058	0,000399	0,004183	-4,381945	protein_coding	PIGX	phosphatidylinositol glycan anchor biosynthesis class X [Source:HGNC Symbol;Acc:HGNC:26046]	3
ENSG00000186314	3,353773	1,455563	0,0004	0,004184	4,381325	protein_coding	PRELID2	PRELI domain containing 2 [Source:HGNC Symbol;Acc:HGNC:28306]	5
ENSG00000088387	6,176292	1,05059	0,0004	0,004184	4,381184	protein_coding	DOCK9	dedicator of cytokinesis 9 [Source:HGNC Symbol;Acc:HGNC:14132]	13
ENSG00000149925	5,6515	-1,221358	0,0004	0,004185	-4,380779	protein_coding	ALDOA	aldolase, fructose-bisphosphate A [Source:HGNC Symbol;Acc:HGNC:414]	16
ENSG00000012779	3,156069	2,112707	0,000401	0,004189	4,379975	protein_coding	ALOX5	arachidonate 5-lipoxygenase [Source:HGNC Symbol;Acc:HGNC:435]	10
ENSG00000177144	-1,911231	-2,685922	0,000402	0,004189	-4,37912	protein_coding	NUDT4B	nudix hydrolase 4B [Source:HGNC Symbol;Acc:HGNC:18012]	1
ENSG00000144815	4,911152	-0,98819	0,000401	0,004189	-4,379597	protein_coding	NXPE3	neurexophilin and PC-esterase domain family member 3 [Source:HGNC Symbol;Acc:HGNC:28238]	3
ENSG00000114857	6,910341	1,073323	0,000401	0,004189	4,37933	protein_coding	NKTR	natural killer cell triggering receptor [Source:HGNC Symbol;Acc:HGNC:7833]	3
ENSG00000243927	4,581507	-1,369664	0,000403	0,004201	-4,377527	protein_coding	MRPS6	mitochondrial ribosomal protein S6 [Source:HGNC Symbol;Acc:HGNC:14051]	21
ENSG00000238227	4,94557	-0,68409	0,000403	0,004202	-4,377153	protein_coding	TMEM250	transmembrane protein 250 [Source:HGNC Symbol;Acc:HGNC:31009]	9
ENSG00000198049	-2,058169	3,730146	0,000404	0,004206	4,376337	protein_coding	AVPR1B	arginine vasopressin receptor 1B [Source:HGNC Symbol;Acc:HGNC:896]	1
ENSG00000127249	-1,657833	3,226901	0,000406	0,004226	4,373888	protein_coding	ATP13A4	ATPase 13A4 [Source:HGNC Symbol;Acc:HGNC:25422]	3
ENSG00000166147	8,874734	-1,964369	0,000407	0,004228	-4,373415	protein_coding	FBN1	fibrillin 1 [Source:HGNC Symbol;Acc:HGNC:3603]	15
ENSG00000235387	-1,870445	3,077847	0,000408	0,004235	4,372065	protein_coding	SPAAR	small regulatory polypeptide of amino acid response [Source:HGNC Symbol;Acc:HGNC:27244]	9
ENSG00000204498	3,887268	0,739608	0,000408	0,004235	4,371948	protein_coding	NFKBIL1	NFKB inhibitor like 1 [Source:HGNC Symbol;Acc:HGNC:7800]	6
ENSG00000167615	7,995602	1,158655	0,00041	0,004254	4,369606	protein_coding	LENG8	leukocyte receptor cluster member 8 [Source:HGNC Symbol;Acc:HGNC:15500]	19
ENSG00000115306	10,47999	-1,011227	0,000411	0,004263	-4,368335	protein_coding	SPTBN1	spectrin beta, non-erythrocytic 1 [Source:HGNC Symbol;Acc:HGNC:11275]	2
ENSG00000179304	1,292704	1,799949	0,000412	0,004268	4,36727	protein_coding	FAM156B	family with sequence similarity 156 member B [Source:HGNC Symbol;Acc:HGNC:31962]	X
ENSG00000156398	2,816515	1,759179	0,000412	0,004268	4,367319	protein_coding	SFXN2	sideroflexin 2 [Source:HGNC Symbol;Acc:HGNC:16086]	10
ENSG00000243696	0,430514	1,769849	0,000413	0,004271	4,366594	protein_coding	AC006254.	novel MUSTN1-ITIH4 readthrough	3
ENSG00000197798	4,154086	-0,725269	0,000414	0,004282	-4,36513	protein_coding	FAM118B	family with sequence similarity 118 member B [Source:HGNC Symbol;Acc:HGNC:26110]	11
ENSG00000205413	5,954517	-1,084835	0,000415	0,00429	-4,363942	protein_coding	SAMD9	sterile alpha motif domain containing 9 [Source:HGNC Symbol;Acc:HGNC:1348]	7
ENSG00000115461	10,16597	-3,234188	0,000416	0,004298	-4,362753	protein_coding	IGFBP5	insulin like growth factor binding protein 5 [Source:HGNC Symbol;Acc:HGNC:5474]	2
ENSG00000137168	4,087716	-0,895041	0,000417	0,0043	-4,362262	protein_coding	PPIL1	peptidylprolyl isomerase like 1 [Source:HGNC Symbol;Acc:HGNC:9260]	6
ENSG00000160877	6,542808	-0,771494	0,000417	0,004303	-4,361663	protein_coding	NACC1	nucleus accumbens associated 1 [Source:HGNC Symbol;Acc:HGNC:20967]	19
ENSG00000092529	1,07258	1,842927	0,000418	0,004307	4,360902	protein_coding	CAPN3	calpain 3 [Source:HGNC Symbol;Acc:HGNC:1480]	15
ENSG00000197008	3,394543	1,25488	0,000419	0,004314	4,35935	protein_coding	ZNF138	zinc finger protein 138 [Source:HGNC Symbol;Acc:HGNC:12922]	7
ENSG00000204673	6,12273	-0,805084	0,000419	0,004314	-4,359378	protein_coding	AKT1S1	AKT1 substrate 1 [Source:HGNC Symbol;Acc:HGNC:28426]	19
ENSG00000068697	6,253402	-1,16199	0,000419	0,004314	-4,359526	protein_coding	LAPTM4A	lysosomal protein transmembrane 4 alpha [Source:HGNC Symbol;Acc:HGNC:6924]	2
ENSG00000242574	1,957955	3,152501	0,00042	0,004322	4,358148	protein_coding	HLA-DMB	major histocompatibility complex, class II, DM beta [Source:HGNC Symbol;Acc:HGNC:4935]	6

ENSG00000135862	8,881889	-1,600683	0,000422	0,004336	-4,356349	protein_coding	LAMC1	laminin subunit gamma 1 [Source:HGNC Symbol;Acc:HGNC:6492]	1
ENSG00000090006	6,204473	1,429945	0,000422	0,004338	4,35587	protein_coding	LTBP4	latent transforming growth factor beta binding protein 4 [Source:HGNC Symbol;Acc:HGNC:6717]	19
ENSG00000239264	5,384813	-1,223403	0,000423	0,004343	-4,355085	protein_coding	TXNDC5	thioredoxin domain containing 5 [Source:HGNC Symbol;Acc:HGNC:21073]	6
ENSG00000214193	3,817781	1,264018	0,000424	0,004348	4,35422	protein_coding	SH3D21	SH3 domain containing 21 [Source:HGNC Symbol;Acc:HGNC:26236]	1
ENSG00000147669	4,434674	-0,77766	0,000428	0,004385	-4,349987	protein_coding	POLR2K	RNA polymerase II, I and III subunit K [Source:HGNC Symbol;Acc:HGNC:9198]	8
ENSG00000070814	6,705433	0,621534	0,00043	0,004405	4,347588	protein_coding	TCOF1	treacle ribosome biogenesis factor 1 [Source:HGNC Symbol;Acc:HGNC:11654]	5
ENSG00000120279	-0,878407	4,156441	0,000432	0,004417	4,345714	protein_coding	MYCT1	MYC target 1 [Source:HGNC Symbol;Acc:HGNC:23172]	6
ENSG00000081320	6,266918	-1,836179	0,000432	0,004417	-4,345712	protein_coding	STK17B	serine/threonine kinase 17b [Source:HGNC Symbol;Acc:HGNC:11396]	2
ENSG00000049768	-0,626582	2,255889	0,000434	0,004435	4,343602	protein_coding	FOXP3	forkhead box P3 [Source:HGNC Symbol;Acc:HGNC:6106]	X
ENSG00000148672	6,762731	-0,747901	0,000434	0,004439	-4,342911	protein_coding	GLUD1	glutamate dehydrogenase 1 [Source:HGNC Symbol;Acc:HGNC:4335]	10
ENSG00000111452	2,363061	3,196189	0,000435	0,004444	4,341741	protein_coding	ADGRD1	adhesion G protein-coupled receptor D1 [Source:HGNC Symbol;Acc:HGNC:19893]	12
ENSG00000005075	4,993151	-0,850192	0,000435	0,004444	-4,342021	protein_coding	POLR2J	RNA polymerase II subunit J [Source:HGNC Symbol;Acc:HGNC:9197]	7
ENSG00000137103	4,132872	1,508971	0,000438	0,004464	4,339411	protein_coding	TMEM8B	transmembrane protein 8B [Source:HGNC Symbol;Acc:HGNC:21427]	9
ENSG00000105486	6,001321	1,553029	0,000438	0,004464	4,339139	protein_coding	LIG1	DNA ligase 1 [Source:HGNC Symbol;Acc:HGNC:6598]	19
ENSG00000197766	1,286599	3,289	0,00044	0,00448	4,337162	protein_coding	CFD	complement factor D [Source:HGNC Symbol;Acc:HGNC:2771]	19
ENSG00000285816	-1,546779	-2,40258	0,000441	0,004492	-4,335619	protein_coding	AP000944.	novel protein, POLA2-CDC42EP2 readthrough	11
ENSG00000125386	6,269562	0,613312	0,000442	0,004497	4,334797	protein_coding	FAM193A	family with sequence similarity 193 member A [Source:HGNC Symbol;Acc:HGNC:16822]	4
ENSG00000083857	8,771376	-1,596394	0,000443	0,004505	-4,333754	protein_coding	FAT1	FAT atypical cadherin 1 [Source:HGNC Symbol;Acc:HGNC:3595]	4
ENSG00000114859	3,176592	0,99261	0,000444	0,004517	4,332219	protein_coding	CLCN2	chloride voltage-gated channel 2 [Source:HGNC Symbol;Acc:HGNC:2020]	3
ENSG00000196914	8,724464	-0,647799	0,000446	0,004531	-4,330503	protein_coding	ARHGEF12	Rho guanine nucleotide exchange factor 12 [Source:HGNC Symbol;Acc:HGNC:14193]	11
ENSG00000011523	4,566726	1,21221	0,000447	0,004532	4,330055	protein_coding	CEP68	centrosomal protein 68 [Source:HGNC Symbol;Acc:HGNC:29076]	2
ENSG00000171456	7,116932	0,766235	0,000447	0,004532	4,329775	protein_coding	ASXL1	ASXL transcriptional regulator 1 [Source:HGNC Symbol;Acc:HGNC:18318]	20
ENSG00000005961	-0,197431	2,601062	0,000447	0,004536	4,329122	protein_coding	ITGA2B	integrin subunit alpha 2b [Source:HGNC Symbol;Acc:HGNC:6138]	17
ENSG00000141013	4,968674	1,033423	0,000448	0,004536	4,328796	protein_coding	GAS8	growth arrest specific 8 [Source:HGNC Symbol;Acc:HGNC:4166]	16
ENSG00000184378	1,014285	-1,700984	0,000452	0,004561	-4,324768	protein_coding	ACTRT3	actin related protein T3 [Source:HGNC Symbol;Acc:HGNC:24022]	3
ENSG00000140398	2,687329	1,974404	0,000452	0,004561	4,324506	protein_coding	NEIL1	nei like DNA glycosylase 1 [Source:HGNC Symbol;Acc:HGNC:18448]	15
ENSG00000268182	-1,166963	2,833547	0,000451	0,004561	4,325638	protein_coding	SMIM17	small integral membrane protein 17 [Source:HGNC Symbol;Acc:HGNC:27114]	19
ENSG00000181322	-1,322146	2,589099	0,000451	0,004561	4,325001	protein_coding	NME9	NME/NM23 family member 9 [Source:HGNC Symbol;Acc:HGNC:21343]	3
ENSG00000132122	3,480005	1,158939	0,000451	0,004561	4,325274	protein_coding	SPATA6	spermatogenesis associated 6 [Source:HGNC Symbol;Acc:HGNC:18309]	1
ENSG00000135148	5,068419	-0,580485	0,000451	0,004561	-4,325061	protein_coding	TRAFD1	TRAF-type zinc finger domain containing 1 [Source:HGNC Symbol;Acc:HGNC:24808]	12
ENSG00000182782	-2,41068	3,357776	0,000455	0,004588	4,321517	protein_coding	HCAR2	hydroxycarboxylic acid receptor 2 [Source:HGNC Symbol;Acc:HGNC:24827]	12
ENSG00000166181	6,334463	-0,678238	0,000456	0,004595	-4,320475	protein_coding	API5	apoptosis inhibitor 5 [Source:HGNC Symbol;Acc:HGNC:594]	11
ENSG00000100344	1,075326	-3,063382	0,000457	0,004596	-4,319818	protein_coding	PNPLA3	patatin like phospholipase domain containing 3 [Source:HGNC Symbol;Acc:HGNC:18590]	22
ENSG00000129128	6,565264	-1,003505	0,000456	0,004596	-4,319845	protein_coding	SPCS3	signal peptidase complex subunit 3 [Source:HGNC Symbol;Acc:HGNC:26212]	4
ENSG00000178175	0,570088	2,96894	0,000457	0,004603	4,318881	protein_coding	ZNF366	zinc finger protein 366 [Source:HGNC Symbol;Acc:HGNC:18316]	5
ENSG00000157514	5,149901	2,414527	0,000459	0,004612	4,317328	protein_coding	TSC22D3	TSC22 domain family member 3 [Source:HGNC Symbol;Acc:HGNC:3051]	X
ENSG00000108021	7,083127	-0,465332	0,000459	0,004612	-4,317501	protein_coding	TASOR2	transcription activation suppressor family member 2 [Source:HGNC Symbol;Acc:HGNC:23484]	10
ENSG00000127526	6,230095	-0,595461	0,00046	0,004615	-4,316786	protein_coding	SLC35E1	solute carrier family 35 member E1 [Source:HGNC Symbol;Acc:HGNC:20803]	19
ENSG00000112561	4,003939	1,395997	0,000461	0,004624	4,315574	protein_coding	TFEB	transcription factor EB [Source:HGNC Symbol;Acc:HGNC:11753]	6
ENSG00000130997	-0,180188	1,581269	0,000462	0,004634	4,314335	protein_coding	POLN	DNA polymerase nu [Source:HGNC Symbol;Acc:HGNC:18870]	4
ENSG00000105889	1,339218	-1,741571	0,000462	0,004634	-4,314031	protein_coding	STEAP1B	STEAP family member 1B [Source:HGNC Symbol;Acc:HGNC:41907]	7
ENSG00000100024	-0,799348	2,760109	0,000464	0,00465	4,312174	protein_coding	UPB1	beta-ureidopropionase 1 [Source:HGNC Symbol;Acc:HGNC:16297]	22
ENSG00000177575	4,422682	3,556344	0,000466	0,004668	4,310088	protein_coding	CD163	CD163 molecule [Source:HGNC Symbol;Acc:HGNC:1631]	12
ENSG00000131931	3,548864	-0,939393	0,000467	0,00467	-4,309483	protein_coding	THAP1	THAP domain containing 1 [Source:HGNC Symbol;Acc:HGNC:20856]	8
ENSG00000113643	5,935398	-0,658754	0,000467	0,00467	-4,309312	protein_coding	RARS1	arginyl-tRNA synthetase 1 [Source:HGNC Symbol;Acc:HGNC:9870]	5
ENSG00000148655	2,622072	1,963559	0,000468	0,004674	4,308649	protein_coding	LRMDA	leucine rich melanocyte differentiation associated [Source:HGNC Symbol;Acc:HGNC:23405]	10
ENSG00000025800	6,757719	-0,641374	0,000468	0,004674	-4,308326	protein_coding	KPNA6	karyopherin subunit alpha 6 [Source:HGNC Symbol;Acc:HGNC:6399]	1
ENSG00000164879	-1,876344	2,484732	0,000469	0,004682	4,307258	protein_coding	CA3	carbonic anhydrase 3 [Source:HGNC Symbol;Acc:HGNC:1374]	8
ENSG00000241489	1,006006	1,904241	0,000471	0,004694	4,305265	protein_coding	AC244197.	novel protein	X
ENSG00000181856	-0,823138	3,100855	0,000471	0,004694	4,305313	protein_coding	SLC2A4	solute carrier family 2 member 4 [Source:HGNC Symbol;Acc:HGNC:11009]	17
ENSG00000100376	4,601116	0,981151	0,000471	0,004694	4,305514	protein_coding	FAM118A	family with sequence similarity 118 member A [Source:HGNC Symbol;Acc:HGNC:1313]	22

ENSG00000131042	0,847402	3,377073	0,000472	0,004701	4,304276	protein_coding	LILRB2	leukocyte immunoglobulin like receptor B2 [Source:HGNC Symbol;Acc:HGNC:6606]	19
ENSG00000165916	6,755516	-0,748843	0,000473	0,004702	-4,303886	protein_coding	PSMC3	proteasome 26S subunit, ATPase 3 [Source:HGNC Symbol;Acc:HGNC:9549]	11
ENSG00000244734	0,02489	5,238278	0,000473	0,004703	4,303525	protein_coding	HBB	hemoglobin subunit beta [Source:HGNC Symbol;Acc:HGNC:4827]	11
ENSG00000110851	5,737426	-0,508604	0,000473	0,004703	-4,303222	protein_coding	PRDM4	PR/SET domain 4 [Source:HGNC Symbol;Acc:HGNC:9348]	12
ENSG00000057608	7,729925	-0,974177	0,000474	0,004704	-4,302876	protein_coding	GDI2	GDP dissociation inhibitor 2 [Source:HGNC Symbol;Acc:HGNC:4227]	10
ENSG00000178057	4,322897	-0,597888	0,000475	0,00472	-4,301011	protein_coding	NDUFAF3	NADH:ubiquinone oxidoreductase complex assembly factor 3 [Source:HGNC Symbol;Acc:HGNC:29918]	3
ENSG00000100644	8,228541	-1,346989	0,000476	0,004727	-4,300055	protein_coding	HIF1A	hypoxia inducible factor 1 subunit alpha [Source:HGNC Symbol;Acc:HGNC:4910]	14
ENSG00000156639	6,524518	-0,5274	0,00048	0,004757	-4,296818	protein_coding	ZFAND3	zinc finger AN1-type containing 3 [Source:HGNC Symbol;Acc:HGNC:18019]	6
ENSG00000110848	-1,324951	5,120488	0,000481	0,004765	4,29565	protein_coding	CD69	CD69 molecule [Source:HGNC Symbol;Acc:HGNC:1694]	12
ENSG00000117592	6,472819	-1,225111	0,000481	0,004765	-4,295469	protein_coding	PRDX6	peroxiredoxin 6 [Source:HGNC Symbol;Acc:HGNC:16753]	1
ENSG00000075826	3,149049	1,84425	0,000482	0,004767	4,295008	protein_coding	SEC31B	SEC31 homolog B, COPII coat complex component [Source:HGNC Symbol;Acc:HGNC:23197]	10
ENSG00000196735	1,818855	4,396169	0,000485	0,004774	4,292279	protein_coding	HLA-DQA1	major histocompatibility complex, class II, DQ alpha 1 [Source:HGNC Symbol;Acc:HGNC:4942]	6
ENSG00000167524	3,165465	1,415082	0,000485	0,004774	4,292189	protein_coding	RSKR	ribosomal protein S6 kinase related [Source:HGNC Symbol;Acc:HGNC:26314]	17
ENSG00000113073	-1,890492	2,640951	0,000484	0,004774	4,292416	protein_coding	SLC4A9	solute carrier family 4 member 9 [Source:HGNC Symbol;Acc:HGNC:11035]	5
ENSG00000228727	-1,924446	2,532884	0,000485	0,004774	4,292093	protein_coding	SAPCD1	suppressor APC domain containing 1 [Source:HGNC Symbol;Acc:HGNC:13938]	6
ENSG00000185621	3,937374	-0,737812	0,000483	0,004774	-4,293903	protein_coding	LMLN	leishmanolysin like peptidase [Source:HGNC Symbol;Acc:HGNC:15991]	3
ENSG00000069869	4,877388	-1,022308	0,000484	0,004774	-4,293215	protein_coding	NEDD4	NEDD4 E3 ubiquitin protein ligase [Source:HGNC Symbol;Acc:HGNC:7727]	15
ENSG00000174807	5,735616	-2,012412	0,000484	0,004774	-4,292483	protein_coding	CD248	CD248 molecule [Source:HGNC Symbol;Acc:HGNC:18219]	11
ENSG00000162576	5,79015	-1,878195	0,000483	0,004774	-4,293736	protein_coding	MXRA8	matrix remodeling associated 8 [Source:HGNC Symbol;Acc:HGNC:7542]	1
ENSG00000013364	6,809953	-1,054989	0,000487	0,00479	-4,29025	protein_coding	MVP	major vault protein [Source:HGNC Symbol;Acc:HGNC:7531]	16
ENSG00000179604	5,52104	1,021424	0,000487	0,004792	4,289779	protein_coding	CDC42EP4	CDC42 effector protein 4 [Source:HGNC Symbol;Acc:HGNC:17147]	17
ENSG00000160117	-0,290918	1,676419	0,000487	0,004792	4,289457	protein_coding	ANKLE1	ankyrin repeat and LEM domain containing 1 [Source:HGNC Symbol;Acc:HGNC:26812]	19
ENSG00000130368	-2,37376	3,130024	0,00049	0,004812	4,287245	protein_coding	MAS1	MAS1 proto-oncogene, G protein-coupled receptor [Source:HGNC Symbol;Acc:HGNC:6899]	6
ENSG00000196655	4,190408	-0,741025	0,00049	0,004816	-4,286624	protein_coding	TRAPPC4	trafficking protein particle complex 4 [Source:HGNC Symbol;Acc:HGNC:19943]	11
ENSG00000163563	0,357843	3,764024	0,000492	0,004826	4,285402	protein_coding	MNDA	myeloid cell nuclear differentiation antigen [Source:HGNC Symbol;Acc:HGNC:7183]	1
ENSG00000198342	1,840364	1,219299	0,000494	0,004841	4,283684	protein_coding	ZNF442	zinc finger protein 442 [Source:HGNC Symbol;Acc:HGNC:20877]	19
ENSG00000167193	6,130376	-0,750373	0,000495	0,004847	-4,28277	protein_coding	CRK	CRK proto-oncogene, adaptor protein [Source:HGNC Symbol;Acc:HGNC:2362]	17
ENSG00000114978	6,541308	-1,15752	0,000496	0,004854	-4,281864	protein_coding	MOB1A	MOB kinase activator 1A [Source:HGNC Symbol;Acc:HGNC:16015]	2
ENSG00000135956	6,643137	-0,665948	0,000496	0,004854	-4,281554	protein_coding	TMEM127	transmembrane protein 127 [Source:HGNC Symbol;Acc:HGNC:26038]	2
ENSG00000089693	6,681305	-0,619479	0,000496	0,004856	-4,281119	protein_coding	MLF2	myeloid leukemia factor 2 [Source:HGNC Symbol;Acc:HGNC:7126]	12
ENSG00000152291	8,295031	-0,666772	0,000497	0,004863	-4,28013	protein_coding	TGOLN2	trans-golgi network protein 2 [Source:HGNC Symbol;Acc:HGNC:15450]	2
ENSG00000165355	4,411618	-0,809991	0,000501	0,004894	-4,276974	protein_coding	FBXO33	F-box protein 33 [Source:HGNC Symbol;Acc:HGNC:19833]	14
ENSG00000143951	3,522868	0,913874	0,000502	0,004905	4,275667	protein_coding	WDPCP	WD repeat containing planar cell polarity effector [Source:HGNC Symbol;Acc:HGNC:28027]	2
ENSG00000152484	5,856311	-0,678156	0,000504	0,004923	-4,273626	protein_coding	USP12	ubiquitin specific peptidase 12 [Source:HGNC Symbol;Acc:HGNC:20485]	13
ENSG00000184203	5,528918	-0,793277	0,000506	0,004931	-4,272658	protein_coding	PPP1R2	protein phosphatase 1 regulatory inhibitor subunit 2 [Source:HGNC Symbol;Acc:HGNC:9288]	3
ENSG00000169599	4,419341	-0,709499	0,000506	0,004931	-4,272085	protein_coding	NFU1	NFU1 iron-sulfur cluster scaffold [Source:HGNC Symbol;Acc:HGNC:16287]	2
ENSG00000087302	6,283404	-0,720968	0,000506	0,004931	-4,272103	protein_coding	RTRAF	RNA transcription, translation and transport factor [Source:HGNC Symbol;Acc:HGNC:23169]	14
ENSG00000127311	3,043344	1,01611	0,000508	0,004943	4,270713	protein_coding	HELB	DNA helicase B [Source:HGNC Symbol;Acc:HGNC:17196]	12
ENSG00000040275	5,302358	-1,206125	0,000509	0,004949	-4,269846	protein_coding	SPDL1	spindle apparatus coiled-coil protein 1 [Source:HGNC Symbol;Acc:HGNC:26010]	5
ENSG00000101938	-0,208533	4,662413	0,000509	0,004952	4,269269	protein_coding	CHRDL1	chordin like 1 [Source:HGNC Symbol;Acc:HGNC:29861]	X
ENSG00000107957	8,246981	-1,026884	0,00051	0,004958	-4,268465	protein_coding	SH3PXD2A	SH3 and PX domains 2A [Source:HGNC Symbol;Acc:HGNC:23664]	10
ENSG00000165119	8,801015	-0,632796	0,000511	0,004962	-4,267814	protein_coding	HNRNPK	heterogeneous nuclear ribonucleoprotein K [Source:HGNC Symbol;Acc:HGNC:5044]	9
ENSG00000010322	6,783986	0,987122	0,000512	0,004973	4,266475	protein_coding	NISCH	nischarin [Source:HGNC Symbol;Acc:HGNC:18006]	3
ENSG00000142156	9,07322	-1,964335	0,000513	0,004975	-4,266001	protein_coding	COL6A1	collagen type VI alpha 1 chain [Source:HGNC Symbol;Acc:HGNC:2211]	21
ENSG00000169291	1,138629	3,513952	0,000514	0,004981	4,265202	protein_coding	SHE	Src homology 2 domain containing E [Source:HGNC Symbol;Acc:HGNC:27004]	1
ENSG00000112110	4,875621	-0,876906	0,000515	0,004986	-4,264442	protein_coding	MRPL18	mitochondrial ribosomal protein L18 [Source:HGNC Symbol;Acc:HGNC:14477]	6
ENSG00000124126	5,180983	2,058496	0,000516	0,004995	4,263314	protein_coding	PREX1	phosphatidylinositol-3,4,5-trisphosphate dependent Rac exchange factor 1 [Source:HGNC Symbol;Acc:HGNC:32594]	20
ENSG00000167984	0,125755	2,905504	0,000518	0,005011	4,261263	protein_coding	NLRC3	NLR family CARD domain containing 3 [Source:HGNC Symbol;Acc:HGNC:29889]	16
ENSG00000117151	3,969922	-1,040247	0,000518	0,005011	-4,261255	protein_coding	CTBS	chitobiase [Source:HGNC Symbol;Acc:HGNC:2496]	1
ENSG00000119729	5,607825	-1,225534	0,000518	0,005011	-4,261031	protein_coding	RHOQ	ras homolog family member Q [Source:HGNC Symbol;Acc:HGNC:17736]	2
ENSG00000186918	4,670992	2,122109	0,00052	0,005026	4,259362	protein_coding	ZNF395	zinc finger protein 395 [Source:HGNC Symbol;Acc:HGNC:18737]	8

ENSG00000180801	3,272793	-1,590588	0,000521	0,005032	-4,258322	protein_coding	ARSJ	arylsulfatase family member J [Source:HGNC Symbol;Acc:HGNC:26286]	4
ENSG00000119487	6,264624	-0,638587	0,000521	0,005032	-4,258525	protein_coding	MAPKAP1	MAPK associated protein 1 [Source:HGNC Symbol;Acc:HGNC:18752]	9
ENSG00000165629	6,201387	-0,609288	0,000523	0,005042	-4,257151	protein_coding	ATP5F1C	ATP synthase F1 subunit gamma [Source:HGNC Symbol;Acc:HGNC:833]	10
ENSG00000142065	3,612361	1,08285	0,000524	0,005048	4,256267	protein_coding	ZFP14	ZFP14 zinc finger protein [Source:HGNC Symbol;Acc:HGNC:29312]	19
ENSG00000166265	-0,179177	3,838292	0,000525	0,005056	4,255324	protein_coding	CYYR1	cysteine and tyrosine rich 1 [Source:HGNC Symbol;Acc:HGNC:16274]	21
ENSG00000182831	6,55014	-0,607101	0,000525	0,005056	-4,255037	protein_coding	C16orf72	chromosome 16 open reading frame 72 [Source:HGNC Symbol;Acc:HGNC:30103]	16
ENSG00000113749	0,687265	2,931866	0,000526	0,005063	4,254118	protein_coding	HRH2	histamine receptor H2 [Source:HGNC Symbol;Acc:HGNC:5183]	5
ENSG00000221866	4,605436	-2,956264	0,000527	0,005069	-4,253227	protein_coding	PLXNA4	plexin A4 [Source:HGNC Symbol;Acc:HGNC:9102]	7
ENSG00000170989	1,823123	3,819898	0,000528	0,00507	4,252881	protein_coding	S1PR1	sphingosine-1-phosphate receptor 1 [Source:HGNC Symbol;Acc:HGNC:3165]	1
ENSG00000100897	6,237378	0,765099	0,000528	0,00507	4,252679	protein_coding	DCAF11	DDB1 and CUL4 associated factor 11 [Source:HGNC Symbol;Acc:HGNC:20258]	14
ENSG00000179820	7,377831	-1,173256	0,000528	0,005072	-4,252152	protein_coding	MYADM	myeloid associated differentiation marker [Source:HGNC Symbol;Acc:HGNC:7544]	19
ENSG00000068383	4,076688	-0,881181	0,000529	0,005077	-4,25145	protein_coding	INPP5A	inositol polyphosphate-5-phosphatase A [Source:HGNC Symbol;Acc:HGNC:6076]	10
ENSG00000184206	-1,29864	2,426087	0,00053	0,005082	4,250707	protein_coding	GOLGA6L4	golgin A6 family like 4 [Source:HGNC Symbol;Acc:HGNC:27256]	15
ENSG00000187742	6,260789	0,807469	0,000531	0,005087	4,249959	protein_coding	SECISBP2	SECIS binding protein 2 [Source:HGNC Symbol;Acc:HGNC:30972]	9
ENSG00000223547	2,775454	0,990418	0,000532	0,005091	4,249385	protein_coding	ZNF844	zinc finger protein 844 [Source:HGNC Symbol;Acc:HGNC:25932]	19
ENSG00000174744	5,151537	-0,528596	0,000534	0,005107	-4,247626	protein_coding	BRMS1	BRMS1 transcriptional repressor and anoikis regulator [Source:HGNC Symbol;Acc:HGNC:17262]	11
ENSG00000113555	1,573525	3,516357	0,000535	0,005113	4,246062	protein_coding	PCDH12	protocadherin 12 [Source:HGNC Symbol;Acc:HGNC:8657]	5
ENSG00000212124	0,035635	1,736157	0,000536	0,005113	4,245647	protein_coding	TAS2R19	taste 2 receptor member 19 [Source:HGNC Symbol;Acc:HGNC:19108]	12
ENSG00000117751	4,95069	-0,742199	0,000535	0,005113	-4,246284	protein_coding	PPP1R8	protein phosphatase 1 regulatory subunit 8 [Source:HGNC Symbol;Acc:HGNC:9296]	1
ENSG00000013016	5,464542	-1,982541	0,000536	0,005113	-4,245644	protein_coding	EHD3	EH domain containing 3 [Source:HGNC Symbol;Acc:HGNC:3244]	2
ENSG00000167460	9,458254	-1,234962	0,000536	0,005113	-4,24521	protein_coding	TPM4	tropomyosin 4 [Source:HGNC Symbol;Acc:HGNC:12013]	19
ENSG00000084207	5,809419	-2,133861	0,000536	0,005113	-4,245675	protein_coding	GSTP1	glutathione S-transferase pi 1 [Source:HGNC Symbol;Acc:HGNC:4638]	11
ENSG00000107745	5,641325	-0,628608	0,000537	0,005113	-4,244921	protein_coding	MICU1	mitochondrial calcium uptake 1 [Source:HGNC Symbol;Acc:HGNC:1530]	10
ENSG00000109332	7,331071	-0,799894	0,000537	0,005113	-4,245067	protein_coding	UBE2D3	ubiquitin conjugating enzyme E2 D3 [Source:HGNC Symbol;Acc:HGNC:12476]	4
ENSG00000159674	5,854754	-1,619529	0,000537	0,005115	-4,244474	protein_coding	SPON2	spondin 2 [Source:HGNC Symbol;Acc:HGNC:11253]	4
ENSG00000139155	-1,840644	2,911849	0,000538	0,005123	4,243471	protein_coding	SLCO1C1	solute carrier organic anion transporter family member 1C1 [Source:HGNC Symbol;Acc:HGNC:13819]	12
ENSG00000167378	6,655662	-0,757828	0,000539	0,005125	-4,243027	protein_coding	IRGQ	immunity related GTPase Q [Source:HGNC Symbol;Acc:HGNC:24868]	19
ENSG00000164647	2,526159	-1,855766	0,00054	0,005135	-4,241818	protein_coding	STEAP1	STEAP family member 1 [Source:HGNC Symbol;Acc:HGNC:11378]	7
ENSG00000118855	5,242045	-1,059413	0,000541	0,005136	-4,241458	protein_coding	MFSD1	major facilitator superfamily domain containing 1 [Source:HGNC Symbol;Acc:HGNC:25874]	3
ENSG00000172020	2,902762	-2,936744	0,000542	0,005149	-4,240042	protein_coding	GAP43	growth associated protein 43 [Source:HGNC Symbol;Acc:HGNC:4140]	3
ENSG00000126464	6,890181	0,677038	0,000543	0,00515	4,23969	protein_coding	PRR12	proline rich 12 [Source:HGNC Symbol;Acc:HGNC:29217]	19
ENSG00000269858	3,78626	1,092882	0,000543	0,00515	4,239401	protein_coding	EGLN2	egl-9 family hypoxia inducible factor 2 [Source:HGNC Symbol;Acc:HGNC:14660]	19
ENSG00000130159	4,78219	0,741654	0,000547	0,005179	4,236601	protein_coding	ECSIT	ECSIT signaling integrator [Source:HGNC Symbol;Acc:HGNC:29548]	19
ENSG00000164086	4,607891	-1,337675	0,000547	0,00518	-4,236228	protein_coding	DUSP7	dual specificity phosphatase 7 [Source:HGNC Symbol;Acc:HGNC:3073]	3
ENSG00000137841	2,906946	2,729041	0,000547	0,00518	4,235911	protein_coding	PLCB2	phospholipase C beta 2 [Source:HGNC Symbol;Acc:HGNC:9055]	15
ENSG00000196517	2,977735	-1,402231	0,00055	0,005185	-4,233536	protein_coding	SLC6A9	solute carrier family 6 member 9 [Source:HGNC Symbol;Acc:HGNC:11056]	1
ENSG00000164934	5,522406	-0,76378	0,000549	0,005185	-4,23424	protein_coding	DCAF13	DDB1 and CUL4 associated factor 13 [Source:HGNC Symbol;Acc:HGNC:24535]	8
ENSG00000171310	5,736299	-0,983721	0,00055	0,005185	-4,233564	protein_coding	CHST11	carbohydrate sulfotransferase 11 [Source:HGNC Symbol;Acc:HGNC:17422]	12
ENSG00000116459	5,830551	-0,983316	0,000549	0,005185	-4,234159	protein_coding	ATP5PB	ATP synthase peripheral stalk-membrane subunit b [Source:HGNC Symbol;Acc:HGNC:840]	1
ENSG00000112514	5,837843	-0,707323	0,00055	0,005185	-4,233971	protein_coding	CUTA	cutA divalent cation tolerance homolog [Source:HGNC Symbol;Acc:HGNC:21101]	6
ENSG00000134001	6,188803	-0,679369	0,00055	0,005185	-4,233339	protein_coding	EIF2S1	eukaryotic translation initiation factor 2 subunit alpha [Source:HGNC Symbol;Acc:HGNC:3265]	14
ENSG00000070831	6,781446	-0,866837	0,000549	0,005185	-4,234623	protein_coding	CDC42	cell division cycle 42 [Source:HGNC Symbol;Acc:HGNC:1736]	1
ENSG00000110958	7,554504	-0,496065	0,00055	0,005185	-4,233657	protein_coding	PTGES3	prostaglandin E synthase 3 [Source:HGNC Symbol;Acc:HGNC:16049]	12
ENSG00000162378	5,759457	-0,72854	0,000552	0,005193	-4,232375	protein_coding	ZYG11B	zyg-11 family member B, cell cycle regulator [Source:HGNC Symbol;Acc:HGNC:25820]	1
ENSG00000135535	7,411409	-0,887049	0,000554	0,005209	-4,230727	protein_coding	CD164	CD164 molecule [Source:HGNC Symbol;Acc:HGNC:1632]	6
ENSG00000124635	5,071029	1,183725	0,000554	0,005209	4,23021	protein_coding	H2BC11	H2B clustered histone 11 [Source:HGNC Symbol;Acc:HGNC:4761]	6
ENSG00000153956	6,056557	-2,056039	0,000554	0,005209	-4,230154	protein_coding	CACNA2D1	calcium voltage-gated channel auxiliary subunit alpha2delta 1 [Source:HGNC Symbol;Acc:HGNC:1399]	7
ENSG00000205832	-2,230473	3,425464	0,000557	0,005236	4,227528	protein_coding	C16orf96	chromosome 16 open reading frame 96 [Source:HGNC Symbol;Acc:HGNC:40031]	16
ENSG00000179583	2,804658	3,892323	0,000558	0,005238	4,227053	protein_coding	CIITA	class II major histocompatibility complex transactivator [Source:HGNC Symbol;Acc:HGNC:7067]	16
ENSG00000035403	7,899364	-1,419971	0,000559	0,005243	-4,226344	protein_coding	VCL	vinculin [Source:HGNC Symbol;Acc:HGNC:12665]	10
ENSG00000166183	-1,29178	3,890187	0,00056	0,005248	4,225394	protein_coding	ASPG	asparaginase [Source:HGNC Symbol;Acc:HGNC:20123]	14

ENSG00000134352	8,625182	-2,004247	0,00056	0,005248	-4,225623	protein_coding	IL6ST	interleukin 6 signal transducer [Source:HGNC Symbol;Acc:HGNC:6021]	5
ENSG00000185950	6,663096	1,358103	0,000564	0,005279	4,22236	protein_coding	IRS2	insulin receptor substrate 2 [Source:HGNC Symbol;Acc:HGNC:6126]	13
ENSG00000188910	-1,554891	-3,275462	0,000565	0,005293	-4,220925	protein_coding	GJB3	gap junction protein beta 3 [Source:HGNC Symbol;Acc:HGNC:4285]	1
ENSG00000061936	6,425506	0,697216	0,000568	0,005316	4,218657	protein_coding	SFSWAP	splicing factor SWAP [Source:HGNC Symbol;Acc:HGNC:10790]	12
ENSG00000143368	5,58553	-1,027704	0,000569	0,005317	-4,21806	protein_coding	SF3B4	splicing factor 3b subunit 4 [Source:HGNC Symbol;Acc:HGNC:10771]	1
ENSG00000147065	8,346832	-1,722294	0,000569	0,005317	-4,217786	protein_coding	MSN	moesin [Source:HGNC Symbol;Acc:HGNC:7373]	X
ENSG00000137845	7,138022	-0,73293	0,000569	0,005317	-4,217747	protein_coding	ADAM10	ADAM metallopeptidase domain 10 [Source:HGNC Symbol;Acc:HGNC:188]	15
ENSG00000159433	5,330882	1,819944	0,00057	0,005323	4,216979	protein_coding	STARD9	StAR related lipid transfer domain containing 9 [Source:HGNC Symbol;Acc:HGNC:19162]	15
ENSG00000105851	2,040359	2,679131	0,000571	0,005328	4,21623	protein_coding	PIK3CG	phosphatidylinositol-4,5-bisphosphate 3-kinase catalytic subunit gamma [Source:HGNC Symbol;Acc:HGNC:8978]	7
ENSG00000164488	0,9412	2,677997	0,000574	0,005346	4,214189	protein_coding	DACT2	dishevelled binding antagonist of beta catenin 2 [Source:HGNC Symbol;Acc:HGNC:21231]	6
ENSG00000164050	6,866269	1,062062	0,000574	0,005346	4,214173	protein_coding	PLXNB1	plexin B1 [Source:HGNC Symbol;Acc:HGNC:9103]	3
ENSG00000154723	5,847085	-0,86458	0,000575	0,005357	-4,212954	protein_coding	ATP5PF	ATP synthase peripheral stalk subunit F6 [Source:HGNC Symbol;Acc:HGNC:847]	21
ENSG00000189129	1,894919	-2,228674	0,000577	0,005374	-4,211274	protein_coding	PLAC9	placenta associated 9 [Source:HGNC Symbol;Acc:HGNC:19255]	10
ENSG00000141682	4,207972	-1,990356	0,000578	0,005376	-4,210841	protein_coding	PMAIP1	phorbol-12-myristate-13-acetate-induced protein 1 [Source:HGNC Symbol;Acc:HGNC:9108]	18
ENSG00000196924	10,31098	-1,676919	0,000578	0,005377	-4,210437	protein_coding	FLNA	filamin A [Source:HGNC Symbol;Acc:HGNC:3754]	X
ENSG00000048707	7,398986	0,670239	0,000579	0,005377	4,2102	protein_coding	VPS13D	vacuolar protein sorting 13 homolog D [Source:HGNC Symbol;Acc:HGNC:23595]	1
ENSG00000160213	6,237454	-0,933907	0,000579	0,005377	-4,209918	protein_coding	CSTB	cystatin B [Source:HGNC Symbol;Acc:HGNC:2482]	21
ENSG00000182492	8,966453	-1,624706	0,000581	0,005392	-4,208083	protein_coding	BGN	biglycan [Source:HGNC Symbol;Acc:HGNC:1044]	X
ENSG00000158710	7,769625	-0,9939	0,000581	0,005392	-4,208345	protein_coding	TAGLN2	transgelin 2 [Source:HGNC Symbol;Acc:HGNC:11554]	1
ENSG00000131459	4,663956	-2,557056	0,000582	0,005395	-4,207586	protein_coding	GFPT2	glutamine-fructose-6-phosphate transaminase 2 [Source:HGNC Symbol;Acc:HGNC:4242]	5
ENSG00000146540	5,950559	1,254684	0,000583	0,0054	4,206938	protein_coding	C7orf50	chromosome 7 open reading frame 50 [Source:HGNC Symbol;Acc:HGNC:22421]	7
ENSG00000167468	7,33865	-0,717313	0,000584	0,005409	-4,205851	protein_coding	GPX4	glutathione peroxidase 4 [Source:HGNC Symbol;Acc:HGNC:4556]	19
ENSG00000035681	5,349235	-0,69949	0,000585	0,005411	-4,205411	protein_coding	NSMAF	neutral sphingomyelinase activation associated factor [Source:HGNC Symbol;Acc:HGNC:8017]	8
ENSG00000161249	3,225235	2,423789	0,000588	0,005436	4,20269	protein_coding	DMKN	dermokine [Source:HGNC Symbol;Acc:HGNC:25063]	19
ENSG00000138796	4,631431	0,811107	0,000588	0,005436	4,202516	protein_coding	HADH	hydroxyacyl-CoA dehydrogenase [Source:HGNC Symbol;Acc:HGNC:4799]	4
ENSG00000119318	7,966656	-0,479226	0,000588	0,005436	-4,202487	protein_coding	RAD23B	RAD23 homolog B, nucleotide excision repair protein [Source:HGNC Symbol;Acc:HGNC:9813]	9
ENSG00000198670	-2,3155	2,638112	0,000591	0,005452	4,200608	protein_coding	LPA	lipoprotein(a) [Source:HGNC Symbol;Acc:HGNC:6667]	6
ENSG00000111424	4,654735	-1,636753	0,000592	0,005452	-4,200098	protein_coding	VDR	vitamin D receptor [Source:HGNC Symbol;Acc:HGNC:12679]	12
ENSG00000004534	7,062437	1,065095	0,000591	0,005452	4,200188	protein_coding	RBM6	RNA binding motif protein 6 [Source:HGNC Symbol;Acc:HGNC:9903]	3
ENSG00000103335	7,073135	-1,154962	0,000591	0,005452	-4,20086	protein_coding	PIEZO1	piezo type mechanosensitive ion channel component 1 [Source:HGNC Symbol;Acc:HGNC:28993]	16
ENSG00000198467	7,922504	-1,711083	0,000593	0,005462	-4,199026	protein_coding	TPM2	tropomyosin 2 [Source:HGNC Symbol;Acc:HGNC:12011]	9
ENSG00000130037	-1,215834	5,593382	0,000594	0,005472	4,197919	protein_coding	KCNA5	potassium voltage-gated channel subfamily A member 5 [Source:HGNC Symbol;Acc:HGNC:6224]	12
ENSG00000162733	6,193654	-1,986096	0,000595	0,005477	-4,197242	protein_coding	DDR2	discoidin domain receptor tyrosine kinase 2 [Source:HGNC Symbol;Acc:HGNC:2731]	1
ENSG00000124333	5,218382	-0,750094	0,000597	0,005487	-4,195849	protein_coding	VAMP7	vesicle associated membrane protein 7 [Source:HGNC Symbol;Acc:HGNC:11486]	X
ENSG00000122591	6,066101	-1,363518	0,000597	0,005487	-4,195961	protein_coding	FAM126A	family with sequence similarity 126 member A [Source:HGNC Symbol;Acc:HGNC:24587]	7
ENSG00000258461	-2,065782	2,477767	0,000598	0,005496	4,194871	protein_coding	AC012651.	novel transcript	15
ENSG00000196123	4,714391	0,981351	0,0006	0,005504	4,193906	protein_coding	KIAA0895L	KIAA0895 like [Source:HGNC Symbol;Acc:HGNC:34408]	16
ENSG00000120280	-0,841629	3,298783	0,000602	0,005519	4,192378	protein_coding	TASL	TLR adaptor interacting with endolysosomal SLC15A4 [Source:HGNC Symbol;Acc:HGNC:25667]	X
ENSG00000179044	0,427328	2,952189	0,000603	0,005521	4,191018	protein_coding	EXOC3L1	exocyst complex component 3 like 1 [Source:HGNC Symbol;Acc:HGNC:27540]	16
ENSG00000120328	2,488491	1,501369	0,000604	0,005521	4,190755	protein_coding	PCDHB12	protocadherin beta 12 [Source:HGNC Symbol;Acc:HGNC:8683]	5
ENSG00000123545	3,650461	-0,993226	0,000604	0,005521	-4,190308	protein_coding	NDUFAF4	NADH:ubiquinone oxidoreductase complex assembly factor 4 [Source:HGNC Symbol;Acc:HGNC:21034]	6
ENSG00000107130	5,558196	-1,422204	0,000602	0,005521	-4,191914	protein_coding	NCS1	neuronal calcium sensor 1 [Source:HGNC Symbol;Acc:HGNC:3953]	9
ENSG00000103978	5,611819	-0,870915	0,000604	0,005521	-4,19055	protein_coding	TMEM87A	transmembrane protein 87A [Source:HGNC Symbol;Acc:HGNC:24522]	15
ENSG00000114902	5,660629	-0,712462	0,000603	0,005521	-4,191155	protein_coding	SPCS1	signal peptidase complex subunit 1 [Source:HGNC Symbol;Acc:HGNC:23401]	3
ENSG00000086061	7,784605	-0,70252	0,000604	0,005521	-4,19012	protein_coding	DNAJA1	DnaJ heat shock protein family (Hsp40) member A1 [Source:HGNC Symbol;Acc:HGNC:5229]	9
ENSG00000118200	6,942117	-1,082339	0,000603	0,005521	-4,191293	protein_coding	CAMSAP2	calmodulin regulated spectrin associated protein family member 2 [Source:HGNC Symbol;Acc:HGNC:29188]	1
ENSG00000198759	-1,126774	3,517491	0,000605	0,005528	4,18934	protein_coding	EGFL6	EGF like domain multiple 6 [Source:HGNC Symbol;Acc:HGNC:3235]	X
ENSG00000105479	0,418433	2,736886	0,000609	0,005553	4,186744	protein_coding	CCDC114	coiled-coil domain containing 114 [Source:HGNC Symbol;Acc:HGNC:26560]	19
ENSG00000069696	-0,683311	2,094019	0,000609	0,005553	4,18685	protein_coding	DRD4	dopamine receptor D4 [Source:HGNC Symbol;Acc:HGNC:3025]	11
ENSG00000068784	4,596641	0,615872	0,00061	0,005556	4,186163	protein_coding	SRBD1	S1 RNA binding domain 1 [Source:HGNC Symbol;Acc:HGNC:25521]	2
ENSG00000214534	-1,892802	2,184556	0,000611	0,005561	4,184947	protein_coding	ZNF705E	zinc finger protein 705E [Source:HGNC Symbol;Acc:HGNC:33203]	11

ENSG00000168038	3,892125	1,328655	0,000611	0,005561	4,184875	protein_coding	ULK4	unc-51 like kinase 4 [Source:HGNC Symbol;Acc:HGNC:15784]	3
ENSG00000198832	4,790837	-1,318137	0,000611	0,005561	-4,185326	protein_coding	SELENOM	selenoprotein M [Source:HGNC Symbol;Acc:HGNC:30397]	22
ENSG00000160145	5,609546	1,067144	0,000612	0,005561	4,184758	protein_coding	KALRN	kalirin RhoGEF kinase [Source:HGNC Symbol;Acc:HGNC:4814]	3
ENSG00000163512	5,39386	-0,644155	0,000613	0,005569	-4,183837	protein_coding	AZI2	5-azacytidine induced 2 [Source:HGNC Symbol;Acc:HGNC:24002]	3
ENSG00000139194	-0,070282	2,52033	0,000613	0,005572	4,183363	protein_coding	RBP5	retinol binding protein 5 [Source:HGNC Symbol;Acc:HGNC:15847]	12
ENSG00000254996	0,889665	1,382525	0,000614	0,005572	4,183102	protein_coding	ANKHD1-EI	ANKHD1-EIF4EBP3 readthrough [Source:HGNC Symbol;Acc:HGNC:33530]	5
ENSG00000171817	1,986326	1,709094	0,000614	0,005573	4,182767	protein_coding	ZNF540	zinc finger protein 540 [Source:HGNC Symbol;Acc:HGNC:25331]	19
ENSG00000168676	-1,440663	3,625748	0,000618	0,005601	4,179904	protein_coding	KCTD19	potassium channel tetramerization domain containing 19 [Source:HGNC Symbol;Acc:HGNC:24753]	16
ENSG00000224531	4,332529	-0,747787	0,000618	0,005601	-4,179905	protein_coding	SMIM13	small integral membrane protein 13 [Source:HGNC Symbol;Acc:HGNC:27356]	6
ENSG00000147614	0,884397	-2,645135	0,00062	0,005609	-4,178592	protein_coding	ATP6V0D2	ATPase H+ transporting V0 subunit d2 [Source:HGNC Symbol;Acc:HGNC:18266]	8
ENSG00000183671	0,260144	-2,819204	0,000619	0,005609	-4,178878	protein_coding	GPR1	G protein-coupled receptor 1 [Source:HGNC Symbol;Acc:HGNC:4463]	2
ENSG00000154016	0,219063	3,095014	0,00062	0,005609	4,178469	protein_coding	GRAP	GRB2 related adaptor protein [Source:HGNC Symbol;Acc:HGNC:4562]	17
ENSG00000128791	5,312145	-1,374412	0,000622	0,005625	-4,176876	protein_coding	TWSG1	twisted gastrulation BMP signaling modulator 1 [Source:HGNC Symbol;Acc:HGNC:12429]	18
ENSG00000186442	-1,745443	-3,771062	0,000626	0,00565	-4,174343	protein_coding	KRT3	keratin 3 [Source:HGNC Symbol;Acc:HGNC:6440]	12
ENSG00000111203	4,762409	0,937358	0,000625	0,00565	4,17459	protein_coding	ITFG2	integrin alpha FG-GAP repeat containing 2 [Source:HGNC Symbol;Acc:HGNC:30879]	12
ENSG00000180957	5,30319	-0,676469	0,000626	0,005655	-4,173648	protein_coding	PITPNB	phosphatidylinositol transfer protein beta [Source:HGNC Symbol;Acc:HGNC:9002]	22
ENSG00000102007	5,872291	-0,979938	0,000627	0,005658	-4,173175	protein_coding	PLP2	proteolipid protein 2 [Source:HGNC Symbol;Acc:HGNC:9087]	X
ENSG00000100300	3,520972	-1,465985	0,000629	0,005665	-4,171751	protein_coding	TSPO	translocator protein [Source:HGNC Symbol;Acc:HGNC:1158]	22
ENSG00000132478	5,600804	0,724164	0,000629	0,005665	4,17162	protein_coding	UNK	unk zinc finger [Source:HGNC Symbol;Acc:HGNC:29369]	17
ENSG00000168461	6,105838	-1,690144	0,000629	0,005665	-4,172075	protein_coding	RAB31	RAB31, member RAS oncogene family [Source:HGNC Symbol;Acc:HGNC:9771]	18
ENSG00000176915	7,050552	-0,608993	0,000629	0,005665	-4,172095	protein_coding	ANKLE2	ankyrin repeat and LEM domain containing 2 [Source:HGNC Symbol;Acc:HGNC:29101]	12
ENSG00000131711	10,17468	-1,389523	0,000631	0,005674	-4,170627	protein_coding	MAP1B	microtubule associated protein 1B [Source:HGNC Symbol;Acc:HGNC:6836]	5
ENSG00000106128	-2,298149	3,882781	0,000632	0,005682	4,169685	protein_coding	GHRHR	growth hormone releasing hormone receptor [Source:HGNC Symbol;Acc:HGNC:4266]	7
ENSG00000174791	2,704584	-1,680677	0,000632	0,005683	-4,169389	protein_coding	RIN1	Ras and Rab interactor 1 [Source:HGNC Symbol;Acc:HGNC:18749]	11
ENSG00000180044	2,658236	-3,383983	0,000633	0,005686	-4,168841	protein_coding	C3orf80	chromosome 3 open reading frame 80 [Source:HGNC Symbol;Acc:HGNC:40048]	3
ENSG00000022976	3,799382	0,922938	0,000634	0,005691	4,168232	protein_coding	ZNF839	zinc finger protein 839 [Source:HGNC Symbol;Acc:HGNC:20345]	14
ENSG00000137491	4,527922	2,576379	0,000634	0,005692	4,167906	protein_coding	SLCO2B1	solute carrier organic anion transporter family member 2B1 [Source:HGNC Symbol;Acc:HGNC:10962]	11
ENSG00000198888	11,63364	-1,550589	0,000635	0,005693	-4,167539	protein_coding	MT-ND1	mitochondrially encoded NADH:ubiquinone oxidoreductase core subunit 1 [Source:HGNC Symbol;Acc:HGNC:7455]	MT
ENSG00000007255	2,198087	1,300276	0,000637	0,005707	4,166181	protein_coding	TRAPPC6A	trafficking protein particle complex 6A [Source:HGNC Symbol;Acc:HGNC:23069]	19
ENSG00000196890	2,642693	2,065576	0,000641	0,00574	4,163206	protein_coding	H2BU1	H2B.U histone 1 [Source:HGNC Symbol;Acc:HGNC:20514]	1
ENSG00000282608	-1,661904	2,582984	0,000642	0,005749	4,162282	protein_coding	ADORA3	adenosine A3 receptor [Source:HGNC Symbol;Acc:HGNC:268]	1
ENSG00000104881	4,568128	0,992146	0,000646	0,005779	4,159631	protein_coding	PPP1R13L	protein phosphatase 1 regulatory subunit 13 like [Source:HGNC Symbol;Acc:HGNC:18838]	19
ENSG00000089177	6,204357	0,84172	0,000646	0,005779	4,159359	protein_coding	KIF16B	kinesin family member 16B [Source:HGNC Symbol;Acc:HGNC:15869]	20
ENSG00000159388	6,264569	2,049955	0,000648	0,00579	4,158254	protein_coding	BTG2	BTG anti-proliferation factor 2 [Source:HGNC Symbol;Acc:HGNC:1131]	1
ENSG00000186973	-1,056626	3,061757	0,000648	0,005791	4,157906	protein_coding	FAM183A	family with sequence similarity 183 member A [Source:HGNC Symbol;Acc:HGNC:34347]	1
ENSG00000132313	4,646272	-0,678925	0,000649	0,005793	-4,157446	protein_coding	MRPL35	mitochondrial ribosomal protein L35 [Source:HGNC Symbol;Acc:HGNC:14489]	2
ENSG00000198796	2,937113	-1,960284	0,000652	0,005814	-4,155134	protein_coding	ALPK2	alpha kinase 2 [Source:HGNC Symbol;Acc:HGNC:20565]	18
ENSG00000173436	3,565457	-0,948892	0,000652	0,005814	-4,155482	protein_coding	MICOS10	mitochondrial contact site and cristae organizing system subunit 10 [Source:HGNC Symbol;Acc:HGNC:32068]	1
ENSG00000134013	7,30739	-1,98297	0,000652	0,005814	-4,155068	protein_coding	LOXL2	lysyl oxidase like 2 [Source:HGNC Symbol;Acc:HGNC:6666]	8
ENSG00000213930	3,516193	0,866237	0,000654	0,00583	4,153561	protein_coding	GALT	galactose-1-phosphate uridylyltransferase [Source:HGNC Symbol;Acc:HGNC:4135]	9
ENSG00000100221	5,941205	-0,825742	0,000656	0,005834	-4,152712	protein_coding	JOSD1	Josephin domain containing 1 [Source:HGNC Symbol;Acc:HGNC:28953]	22
ENSG00000018510	6,412767	-0,750076	0,000655	0,005834	-4,152889	protein_coding	AGPS	alkylglycerone phosphate synthase [Source:HGNC Symbol;Acc:HGNC:327]	2
ENSG00000085998	5,815202	-0,677948	0,000657	0,005845	-4,151598	protein_coding	POMGNT1	protein O-linked mannose N-acetylglucosaminyltransferase 1 (beta 1,2-) [Source:HGNC Symbol;Acc:HGNC:19139]	1
ENSG00000173272	4,216557	0,912045	0,000662	0,00588	4,14843	protein_coding	MZT2A	mitotic spindle organizing protein 2A [Source:HGNC Symbol;Acc:HGNC:33187]	2
ENSG00000156671	5,6781	-0,718812	0,000662	0,00588	-4,148322	protein_coding	SAMD8	sterile alpha motif domain containing 8 [Source:HGNC Symbol;Acc:HGNC:26320]	10
ENSG00000187753	-1,026782	2,020945	0,000666	0,005912	4,145589	protein_coding	C9orf153	chromosome 9 open reading frame 153 [Source:HGNC Symbol;Acc:HGNC:31456]	9
ENSG00000166847	5,957877	-0,711606	0,000667	0,00592	-4,144738	protein_coding	DCTN5	dynactin subunit 5 [Source:HGNC Symbol;Acc:HGNC:24594]	16
ENSG00000127774	4,186208	-1,061812	0,000668	0,005922	-4,14403	protein_coding	EMC6	ER membrane protein complex subunit 6 [Source:HGNC Symbol;Acc:HGNC:28430]	17
ENSG00000152683	4,773959	-0,714599	0,000668	0,005922	-4,144244	protein_coding	SLC30A6	solute carrier family 30 member 6 [Source:HGNC Symbol;Acc:HGNC:19305]	2
ENSG00000162817	4,317282	1,509785	0,000671	0,005946	4,141766	protein_coding	C1orf115	chromosome 1 open reading frame 115 [Source:HGNC Symbol;Acc:HGNC:25873]	1
ENSG00000077380	6,754324	-0,792565	0,000671	0,005946	-4,141652	protein_coding	DYNC1I2	dynein cytoplasmic 1 intermediate chain 2 [Source:HGNC Symbol;Acc:HGNC:2964]	2

ENSG00000089159	6,784308	-1,101015	0,000672	0,005949	-4,141216	protein_coding	PXN	paxillin [Source:HGNC Symbol;Acc:HGNC:9718]	12
ENSG00000100055	2,777667	2,66615	0,000675	0,005972	4,139147	protein_coding	CYTH4	cytohesin 4 [Source:HGNC Symbol;Acc:HGNC:9505]	22
ENSG00000197061	5,392558	0,636179	0,000676	0,005973	4,138827	protein_coding	H4C3	H4 clustered histone 3 [Source:HGNC Symbol;Acc:HGNC:4787]	6
ENSG00000175376	4,559083	-0,576482	0,000676	0,005976	-4,13837	protein_coding	EIF1AD	eukaryotic translation initiation factor 1A domain containing [Source:HGNC Symbol;Acc:HGNC:28147]	11
ENSG00000109321	0,971479	-4,592225	0,000678	0,005992	-4,136902	protein_coding	AREG	amphiregulin [Source:HGNC Symbol;Acc:HGNC:651]	4
ENSG00000162526	-0,373933	1,623051	0,000679	0,005994	4,136475	protein_coding	TSSK3	testis specific serine kinase 3 [Source:HGNC Symbol;Acc:HGNC:15473]	1
ENSG00000205189	5,728949	0,914785	0,00068	0,006	4,135771	protein_coding	ZBTB10	zinc finger and BTB domain containing 10 [Source:HGNC Symbol;Acc:HGNC:30953]	8
ENSG00000082074	3,179721	2,896762	0,000682	0,00601	4,13451	protein_coding	FYB1	FYN binding protein 1 [Source:HGNC Symbol;Acc:HGNC:4036]	5
ENSG00000104320	5,8439	-0,685054	0,000682	0,00601	-4,13473	protein_coding	NBN	nibrin [Source:HGNC Symbol;Acc:HGNC:7652]	8
ENSG00000157224	5,304654	-0,888863	0,000683	0,006011	-4,13414	protein_coding	CLDN12	claudin 12 [Source:HGNC Symbol;Acc:HGNC:2034]	7
ENSG00000188687	-0,40633	2,210753	0,000684	0,00602	4,133261	protein_coding	SLC4A5	solute carrier family 4 member 5 [Source:HGNC Symbol;Acc:HGNC:18168]	2
ENSG00000071127	8,124239	-0,933234	0,000685	0,006028	-4,132364	protein_coding	WDR1	WD repeat domain 1 [Source:HGNC Symbol;Acc:HGNC:12754]	4
ENSG00000109854	5,167958	-1,128951	0,000686	0,006028	-4,1321	protein_coding	HTATIP2	HIV-1 Tat interactive protein 2 [Source:HGNC Symbol;Acc:HGNC:16637]	11
ENSG00000162542	3,44077	1,312646	0,000687	0,006035	4,131324	protein_coding	TMCO4	transmembrane and coiled-coil domains 4 [Source:HGNC Symbol;Acc:HGNC:27393]	1
ENSG00000157343	-2,074681	2,286103	0,000687	0,006036	4,131032	protein_coding	ARMC12	armadillo repeat containing 12 [Source:HGNC Symbol;Acc:HGNC:21099]	6
ENSG00000102390	4,974341	-0,611412	0,000688	0,006041	-4,130405	protein_coding	PBDC1	polysaccharide biosynthesis domain containing 1 [Source:HGNC Symbol;Acc:HGNC:28790]	X
ENSG00000105053	4,792823	0,800369	0,000691	0,006058	4,128727	protein_coding	VRK3	VRK serine/threonine kinase 3 [Source:HGNC Symbol;Acc:HGNC:18996]	19
ENSG00000138594	6,878286	-0,776549	0,000691	0,006058	-4,128616	protein_coding	TMOD3	tropomodulin 3 [Source:HGNC Symbol;Acc:HGNC:11873]	15
ENSG00000174483	1,576577	1,659896	0,000696	0,006103	4,124946	protein_coding	BBS1	Bardet-Biedl syndrome 1 [Source:HGNC Symbol;Acc:HGNC:966]	11
ENSG00000121594	-0,997776	2,929884	0,000697	0,006104	4,124583	protein_coding	CD80	CD80 molecule [Source:HGNC Symbol;Acc:HGNC:1700]	3
ENSG00000237172	4,394626	-0,945096	0,000698	0,006115	-4,123504	protein_coding	B3GNT9	UDP-GlcNAc:betaGal beta-1,3-N-acetylglucosaminyltransferase 9 [Source:HGNC Symbol;Acc:HGNC:28714]	16
ENSG00000204228	1,293562	1,967571	0,000699	0,006116	4,123193	protein_coding	HSD17B8	hydroxysteroid 17-beta dehydrogenase 8 [Source:HGNC Symbol;Acc:HGNC:3554]	6
ENSG00000187676	4,253038	-0,72448	0,000702	0,006139	-4,121248	protein_coding	B3GLCT	beta 3-glucosyltransferase [Source:HGNC Symbol;Acc:HGNC:20207]	13
ENSG00000109381	5,490758	0,553824	0,000703	0,006145	4,120544	protein_coding	ELF2	E74 like ETS transcription factor 2 [Source:HGNC Symbol;Acc:HGNC:3317]	4
ENSG00000144597	5,662807	-0,53563	0,000704	0,006148	-4,12005	protein_coding	EAF1	ELL associated factor 1 [Source:HGNC Symbol;Acc:HGNC:20907]	3
ENSG00000273841	4,489228	-0,753983	0,00071	0,006199	-4,116014	protein_coding	TAF9	TATA-box binding protein associated factor 9 [Source:HGNC Symbol;Acc:HGNC:11542]	5
ENSG00000183066	1,292565	2,04317	0,000711	0,006205	4,115084	protein_coding	WBP2NL	WBP2 N-terminal like [Source:HGNC Symbol;Acc:HGNC:28389]	22
ENSG00000076043	5,22074	-0,954183	0,000711	0,006205	-4,115091	protein_coding	REXO2	RNA exonuclease 2 [Source:HGNC Symbol;Acc:HGNC:17851]	11
ENSG00000159761	0,666043	1,483312	0,000713	0,00622	4,113719	protein_coding	C16orf86	chromosome 16 open reading frame 86 [Source:HGNC Symbol;Acc:HGNC:33755]	16
ENSG00000197375	4,355664	1,259757	0,000718	0,006255	4,11089	protein_coding	SLC22A5	solute carrier family 22 member 5 [Source:HGNC Symbol;Acc:HGNC:10969]	5
ENSG00000105355	5,742281	-1,431039	0,000718	0,006255	-4,110614	protein_coding	PLIN3	perilipin 3 [Source:HGNC Symbol;Acc:HGNC:16893]	19
ENSG00000133858	6,795688	0,675116	0,000719	0,00626	4,110011	protein_coding	ZFC3H1	zinc finger C3H1-type containing [Source:HGNC Symbol;Acc:HGNC:28328]	12
ENSG00000170477	-2,827274	-2,990675	0,00072	0,006262	-4,109429	protein_coding	KRT4	keratin 4 [Source:HGNC Symbol;Acc:HGNC:6441]	12
ENSG00000196526	6,222142	-1,255492	0,00072	0,006262	-4,10936	protein_coding	AFAP1	actin filament associated protein 1 [Source:HGNC Symbol;Acc:HGNC:24017]	4
ENSG00000112473	7,443744	-0,811712	0,000721	0,006262	-4,109143	protein_coding	SLC39A7	solute carrier family 39 member 7 [Source:HGNC Symbol;Acc:HGNC:4927]	6
ENSG00000144619	3,985758	-2,02541	0,000722	0,006269	-4,108139	protein_coding	CNTN4	contactin 4 [Source:HGNC Symbol;Acc:HGNC:2174]	3
ENSG00000197037	4,591939	0,892836	0,000722	0,006269	4,108184	protein_coding	ZSCAN25	zinc finger and SCAN domain containing 25 [Source:HGNC Symbol;Acc:HGNC:21961]	7
ENSG00000031081	5,482687	-1,59715	0,000724	0,006281	-4,106964	protein_coding	ARHGAP31	Rho GTPase activating protein 31 [Source:HGNC Symbol;Acc:HGNC:29216]	3
ENSG00000182552	3,489081	-0,978477	0,000725	0,006287	-4,106096	protein_coding	RWDD4	RWD domain containing 4 [Source:HGNC Symbol;Acc:HGNC:23750]	4
ENSG00000062194	6,363477	0,422419	0,000725	0,006287	4,106279	protein_coding	GPBP1	GC-rich promoter binding protein 1 [Source:HGNC Symbol;Acc:HGNC:29520]	5
ENSG00000124882	2,506591	-5,516654	0,000726	0,006288	-4,105755	protein_coding	EREG	epiregulin [Source:HGNC Symbol;Acc:HGNC:3443]	4
ENSG00000204764	4,023061	1,283356	0,000728	0,006305	4,10429	protein_coding	RANBP17	RAN binding protein 17 [Source:HGNC Symbol;Acc:HGNC:14428]	5
ENSG00000181090	6,768761	0,622195	0,000729	0,006309	4,103741	protein_coding	EHMT1	euchromatic histone lysine methyltransferase 1 [Source:HGNC Symbol;Acc:HGNC:24650]	9
ENSG00000186094	-0,053445	3,539767	0,000732	0,006333	4,10174	protein_coding	AGBL4	ATP/GTP binding protein like 4 [Source:HGNC Symbol;Acc:HGNC:25892]	1
ENSG00000175832	3,698485	-2,226989	0,000734	0,006343	-4,100761	protein_coding	ETV4	ETS variant transcription factor 4 [Source:HGNC Symbol;Acc:HGNC:3493]	17
ENSG00000182752	4,530334	-2,485513	0,000735	0,006347	-4,100237	protein_coding	PAPPA	pappalysin 1 [Source:HGNC Symbol;Acc:HGNC:8602]	9
ENSG00000162517	4,693956	-0,632888	0,000735	0,00635	-4,099737	protein_coding	PEF1	penta-EF-hand domain containing 1 [Source:HGNC Symbol;Acc:HGNC:30009]	1
ENSG00000182511	3,10412	1,71145	0,000736	0,006354	4,099223	protein_coding	FES	FES proto-oncogene, tyrosine kinase [Source:HGNC Symbol;Acc:HGNC:3657]	15
ENSG00000189269	-0,579589	1,743064	0,000738	0,006363	4,09831	protein_coding	DRICH1	aspartate rich 1 [Source:HGNC Symbol;Acc:HGNC:28031]	22
ENSG00000169429	4,613143	-2,832027	0,00074	0,006377	-4,096988	protein_coding	CXCL8	C-X-C motif chemokine ligand 8 [Source:HGNC Symbol;Acc:HGNC:6025]	4
ENSG00000135272	4,687096	-2,008025	0,00074	0,006377	-4,09685	protein_coding	MDFIC	MyoD family inhibitor domain containing [Source:HGNC Symbol;Acc:HGNC:28870]	7

ENSG00000178033	3,593474	-2,18549	0,000746	0,006422	-4,09336	protein_coding	CALHM5	calcium homeostasis modulator family member 5 [Source:HGNC Symbol;Acc:HGNC:21568]	6
ENSG00000167208	-0,266058	3,251101	0,000748	0,006435	4,091892	protein_coding	SNX20	sorting nexin 20 [Source:HGNC Symbol;Acc:HGNC:30390]	16
ENSG00000147027	4,868282	-1,785012	0,000748	0,006435	-4,091912	protein_coding	TMEM47	transmembrane protein 47 [Source:HGNC Symbol;Acc:HGNC:18515]	X
ENSG00000104870	5,378233	1,803841	0,000751	0,00645	4,090391	protein_coding	FCGRT	Fc fragment of IgG receptor and transporter [Source:HGNC Symbol;Acc:HGNC:3621]	19
ENSG00000078618	7,088437	-0,590555	0,00075	0,00645	-4,090607	protein_coding	NRDC	nardilysin convertase [Source:HGNC Symbol;Acc:HGNC:7995]	1
ENSG00000167157	1,012323	-4,036966	0,000753	0,006468	-4,088632	protein_coding	PRRX2	paired related homeobox 2 [Source:HGNC Symbol;Acc:HGNC:21338]	9
ENSG00000142892	5,024942	-0,838854	0,000753	0,006468	-4,0886	protein_coding	PIGK	phosphatidylinositol glycan anchor biosynthesis class K [Source:HGNC Symbol;Acc:HGNC:8965]	1
ENSG00000165732	7,199111	-0,645672	0,000754	0,00647	-4,088236	protein_coding	DDX21	DExD-box helicase 21 [Source:HGNC Symbol;Acc:HGNC:2744]	10
ENSG00000105675	-2,167642	3,572856	0,000757	0,00649	4,086534	protein_coding	ATP4A	ATPase H+/K+ transporting subunit alpha [Source:HGNC Symbol;Acc:HGNC:819]	19
ENSG00000101294	7,51192	-0,650393	0,000762	0,006528	-4,083639	protein_coding	HM13	histocompatibility minor 13 [Source:HGNC Symbol;Acc:HGNC:16435]	20
ENSG00000127084	2,003375	2,587972	0,000766	0,006561	4,081072	protein_coding	FGD3	FYVE, RhoGEF and PH domain containing 3 [Source:HGNC Symbol;Acc:HGNC:16027]	9
ENSG00000167674	6,526348	0,585933	0,000766	0,006561	4,080827	protein_coding	HDGFL2	HDGF like 2 [Source:HGNC Symbol;Acc:HGNC:14680]	19
ENSG00000169855	7,323825	-1,586459	0,000767	0,006566	-4,08021	protein_coding	ROBO1	roundabout guidance receptor 1 [Source:HGNC Symbol;Acc:HGNC:10249]	3
ENSG00000214456	-0,468065	4,208228	0,00077	0,006572	4,07884	protein_coding	PLIN5	perilipin 5 [Source:HGNC Symbol;Acc:HGNC:33196]	19
ENSG00000144959	4,371188	-1,405186	0,00077	0,006572	-4,078835	protein_coding	NCEH1	neutral cholesterol ester hydrolase 1 [Source:HGNC Symbol;Acc:HGNC:29260]	3
ENSG00000138002	4,945837	1,002641	0,000769	0,006572	4,079123	protein_coding	IFT172	intraflagellar transport 172 [Source:HGNC Symbol;Acc:HGNC:30391]	2
ENSG00000159593	5,099797	-0,553065	0,000769	0,006572	-4,078966	protein_coding	NAE1	NEDD8 activating enzyme E1 subunit 1 [Source:HGNC Symbol;Acc:HGNC:621]	16
ENSG00000138279	6,482078	-0,647098	0,00077	0,006572	-4,078584	protein_coding	ANXA7	annexin A7 [Source:HGNC Symbol;Acc:HGNC:545]	10
ENSG00000185737	2,676934	-2,384311	0,000772	0,006589	-4,077142	protein_coding	NRG3	neuregulin 3 [Source:HGNC Symbol;Acc:HGNC:7999]	10
ENSG00000197324	7,800116	-1,094102	0,000775	0,006609	-4,075548	protein_coding	LRP10	LDL receptor related protein 10 [Source:HGNC Symbol;Acc:HGNC:14553]	14
ENSG00000088832	6,084845	-0,93428	0,000778	0,006629	-4,073858	protein_coding	FKBP1A	FKBP prolyl isomerase 1A [Source:HGNC Symbol;Acc:HGNC:3711]	20
ENSG00000235631	-1,25633	2,712872	0,00078	0,006636	4,07269	protein_coding	RNF148	ring finger protein 148 [Source:HGNC Symbol;Acc:HGNC:22411]	7
ENSG00000267385	-2,424117	3,598805	0,000779	0,006636	4,072969	protein_coding	AC011498.	novel protein	19
ENSG00000198786	12,51183	-1,427613	0,00078	0,006636	-4,072843	protein_coding	MT-ND5	mitochondrially encoded NADH:ubiquinone oxidoreductase core subunit 5 [Source:HGNC Symbol;Acc:HGNC:7461]	MT
ENSG00000204086	-0,637658	2,073276	0,000784	0,006667	4,070067	protein_coding	RPA4	replication protein A4 [Source:HGNC Symbol;Acc:HGNC:30305]	X
ENSG00000105373	7,158827	1,323204	0,000784	0,006667	4,070105	protein_coding	NOP53	NOP53 ribosome biogenesis factor [Source:HGNC Symbol;Acc:HGNC:4333]	19
ENSG00000116641	6,334298	-0,617463	0,000785	0,006669	-4,069522	protein_coding	DOCK7	dedicator of cytokinesis 7 [Source:HGNC Symbol;Acc:HGNC:19190]	1
ENSG00000102580	7,210132	-0,853065	0,000786	0,006669	-4,069436	protein_coding	DNAJC3	DnaJ heat shock protein family (Hsp40) member C3 [Source:HGNC Symbol;Acc:HGNC:9439]	13
ENSG00000198727	11,98302	-1,383704	0,000788	0,006683	-4,06809	protein_coding	MT-CYB	mitochondrially encoded cytochrome b [Source:HGNC Symbol;Acc:HGNC:7427]	MT
ENSG00000144369	5,255579	-0,894068	0,000788	0,006683	-4,068022	protein_coding	FAM171B	family with sequence similarity 171 member B [Source:HGNC Symbol;Acc:HGNC:29412]	2
ENSG00000006025	4,177851	1,008734	0,000794	0,006732	4,06434	protein_coding	OSBPL7	oxysterol binding protein like 7 [Source:HGNC Symbol;Acc:HGNC:16387]	17
ENSG00000185164	5,258943	-1,163599	0,000795	0,006732	-4,063875	protein_coding	NOMO2	NODAL modulator 2 [Source:HGNC Symbol;Acc:HGNC:22652]	16
ENSG00000197694	9,387059	-0,63579	0,000795	0,006732	-4,064006	protein_coding	SPTAN1	spectrin alpha, non-erythrocytic 1 [Source:HGNC Symbol;Acc:HGNC:11273]	9
ENSG00000166333	6,47181	-0,800006	0,000797	0,006746	-4,062735	protein_coding	ILK	integrin linked kinase [Source:HGNC Symbol;Acc:HGNC:6040]	11
ENSG00000239887	2,556674	2,034887	0,000799	0,006761	4,061467	protein_coding	C1orf226	chromosome 1 open reading frame 226 [Source:HGNC Symbol;Acc:HGNC:34351]	1
ENSG00000103512	6,44791	-1,281622	0,000802	0,006778	-4,060057	protein_coding	NOMO1	NODAL modulator 1 [Source:HGNC Symbol;Acc:HGNC:30060]	16
ENSG00000203747	3,243867	3,704087	0,000802	0,006781	4,059606	protein_coding	FCGR3A	Fc fragment of IgG receptor IIIa [Source:HGNC Symbol;Acc:HGNC:3619]	1
ENSG00000115946	4,45721	-0,727542	0,000803	0,006783	-4,05924	protein_coding	PNO1	partner of NOB1 homolog [Source:HGNC Symbol;Acc:HGNC:32790]	2
ENSG00000101935	4,025004	-1,408468	0,000804	0,006784	-4,058939	protein_coding	AMMECR1	AMMECR nuclear protein 1 [Source:HGNC Symbol;Acc:HGNC:467]	X
ENSG00000100225	6,177169	-0,471379	0,000804	0,006787	-4,058517	protein_coding	FBXO7	F-box protein 7 [Source:HGNC Symbol;Acc:HGNC:13586]	22
ENSG00000127920	4,901247	-2,308901	0,000805	0,006788	-4,058203	protein_coding	GNG11	G protein subunit gamma 11 [Source:HGNC Symbol;Acc:HGNC:4403]	7
ENSG00000047249	5,124486	-0,815091	0,000808	0,006814	-4,056179	protein_coding	ATP6V1H	ATPase H+ transporting V1 subunit H [Source:HGNC Symbol;Acc:HGNC:18303]	8
ENSG00000212127	-0,24151	1,685067	0,000812	0,006839	4,054288	protein_coding	TAS2R14	taste 2 receptor member 14 [Source:HGNC Symbol;Acc:HGNC:14920]	12
ENSG00000204851	0,680873	2,852783	0,000813	0,006843	4,053642	protein_coding	PNMA8B	PNMA family member 8B [Source:HGNC Symbol;Acc:HGNC:29206]	19
ENSG00000147894	4,420572	0,846044	0,000813	0,006843	4,053517	protein_coding	C9orf72	C9orf72-SMCR8 complex subunit [Source:HGNC Symbol;Acc:HGNC:28337]	9
ENSG00000153294	-0,4623	-3,457758	0,000816	0,006861	-4,051885	protein_coding	ADGRF4	adhesion G protein-coupled receptor F4 [Source:HGNC Symbol;Acc:HGNC:19011]	6
ENSG00000204217	7,767175	-0,997529	0,000816	0,006861	-4,051813	protein_coding	BMPR2	bone morphogenetic protein receptor type 2 [Source:HGNC Symbol;Acc:HGNC:1078]	2
ENSG00000129197	5,364139	1,104364	0,00082	0,006888	4,049814	protein_coding	RPAIN	RPA interacting protein [Source:HGNC Symbol;Acc:HGNC:28641]	17
ENSG00000104953	0,421614	2,745042	0,000821	0,006892	4,049268	protein_coding	TLE6	TLE family member 6, subcortical maternal complex member [Source:HGNC Symbol;Acc:HGNC:30788]	19
ENSG00000170275	6,642184	-1,717471	0,000825	0,006927	-4,046728	protein_coding	CRTAP	cartilage associated protein [Source:HGNC Symbol;Acc:HGNC:2379]	3
ENSG00000114209	5,210621	-0,657251	0,000826	0,006928	-4,046433	protein_coding	PDCD10	programmed cell death 10 [Source:HGNC Symbol;Acc:HGNC:8761]	3

ENSG00000183918	-2,769565	2,953918	0,000827	0,006931	4,045784	protein_coding	SH2D1A	SH2 domain containing 1A [Source:HGNC Symbol;Acc:HGNC:10820]	X
ENSG00000064999	5,817146	0,767038	0,000827	0,006931	4,045711	protein_coding	ANKS1A	ankyrin repeat and sterile alpha motif domain containing 1A [Source:HGNC Symbol;Acc:HGNC:20961]	6
ENSG00000141696	5,543332	-1,3629	0,000828	0,006936	-4,045137	protein_coding	P3H4	prolyl 3-hydroxylase family member 4 (inactive) [Source:HGNC Symbol;Acc:HGNC:16946]	17
ENSG00000198074	1,101992	-5,257586	0,00083	0,006949	-4,044061	protein_coding	AKR1B10	aldo-keto reductase family 1 member B10 [Source:HGNC Symbol;Acc:HGNC:382]	7
ENSG00000167578	0,147614	1,945768	0,000831	0,006949	4,043577	protein_coding	RAB4B	RAB4B, member RAS oncogene family [Source:HGNC Symbol;Acc:HGNC:9782]	19
ENSG00000135241	5,354581	-0,556857	0,000831	0,006949	-4,043752	protein_coding	PNPLA8	patatin like phospholipase domain containing 8 [Source:HGNC Symbol;Acc:HGNC:28900]	7
ENSG00000181616	-2,963632	2,660406	0,000832	0,006955	4,042984	protein_coding	OR52H1	olfactory receptor family 52 subfamily H member 1 [Source:HGNC Symbol;Acc:HGNC:15218]	11
ENSG00000128052	2,540671	4,031852	0,000833	0,006959	4,04243	protein_coding	KDR	kinase insert domain receptor [Source:HGNC Symbol;Acc:HGNC:6307]	4
ENSG00000142046	1,897035	1,703957	0,000835	0,006972	4,041166	protein_coding	TMEM91	transmembrane protein 91 [Source:HGNC Symbol;Acc:HGNC:32393]	19
ENSG00000136930	5,876184	-0,579761	0,000835	0,006972	-4,041145	protein_coding	PSMB7	proteasome 20S subunit beta 7 [Source:HGNC Symbol;Acc:HGNC:9544]	9
ENSG00000173578	-2,702979	2,724944	0,000837	0,006981	4,040319	protein_coding	XCR1	X-C motif chemokine receptor 1 [Source:HGNC Symbol;Acc:HGNC:1625]	3
ENSG00000136244	3,200508	-4,202802	0,000838	0,006982	-4,039757	protein_coding	IL6	interleukin 6 [Source:HGNC Symbol;Acc:HGNC:6018]	7
ENSG00000123643	5,320998	-0,987746	0,000838	0,006982	-4,039741	protein_coding	SLC36A1	solute carrier family 36 member 1 [Source:HGNC Symbol;Acc:HGNC:18761]	5
ENSG00000145431	4,833005	-2,279522	0,000839	0,006989	-4,039065	protein_coding	PDGFC	platelet derived growth factor C [Source:HGNC Symbol;Acc:HGNC:8801]	4
ENSG00000198105	4,509801	0,935026	0,00084	0,00699	4,038523	protein_coding	ZNF248	zinc finger protein 248 [Source:HGNC Symbol;Acc:HGNC:13041]	10
ENSG00000153006	5,346355	-0,598856	0,000841	0,00699	-4,038294	protein_coding	SREK1IP1	SREK1 interacting protein 1 [Source:HGNC Symbol;Acc:HGNC:26716]	5
ENSG00000075151	7,832243	-0,783939	0,00084	0,00699	-4,038503	protein_coding	EIF4G3	eukaryotic translation initiation factor 4 gamma 3 [Source:HGNC Symbol;Acc:HGNC:3298]	1
ENSG00000165804	4,887622	0,689147	0,000843	0,006996	4,037016	protein_coding	ZNF219	zinc finger protein 219 [Source:HGNC Symbol;Acc:HGNC:13011]	14
ENSG00000144455	4,88406	-0,65598	0,000843	0,006996	-4,036991	protein_coding	SUMF1	sulfatase modifying factor 1 [Source:HGNC Symbol;Acc:HGNC:20376]	3
ENSG00000123213	5,202345	-0,834583	0,000842	0,006996	-4,037583	protein_coding	NLN	neurolysin [Source:HGNC Symbol;Acc:HGNC:16058]	5
ENSG00000099968	5,981361	-0,588325	0,000843	0,006996	-4,036943	protein_coding	BCL2L13	BCL2 like 13 [Source:HGNC Symbol;Acc:HGNC:17164]	22
ENSG00000112186	3,515026	-1,545289	0,000844	0,006999	-4,036533	protein_coding	CAP2	cyclase associated actin cytoskeleton regulatory protein 2 [Source:HGNC Symbol;Acc:HGNC:20039]	6
ENSG00000185728	6,577999	-0,57028	0,000844	0,007	-4,036227	protein_coding	YTHDF3	YTH N6-methyladenosine RNA binding protein 3 [Source:HGNC Symbol;Acc:HGNC:26465]	8
ENSG00000255274	-2,219294	3,501068	0,000847	0,007012	4,034848	protein_coding	SMIM35	small integral membrane protein 35 [Source:HGNC Symbol;Acc:HGNC:44179]	11
ENSG00000109220	4,599654	-0,855833	0,000846	0,007012	-4,035057	protein_coding	CHIC2	cysteine rich hydrophobic domain 2 [Source:HGNC Symbol;Acc:HGNC:1935]	4
ENSG00000084093	5,899297	-1,724857	0,000847	0,007012	-4,034761	protein_coding	REST	RE1 silencing transcription factor [Source:HGNC Symbol;Acc:HGNC:9966]	4
ENSG00000107937	6,010802	-0,645114	0,000848	0,007014	-4,034373	protein_coding	GTPBP4	GTP binding protein 4 [Source:HGNC Symbol;Acc:HGNC:21535]	10
ENSG00000158481	-2,957746	3,674727	0,000849	0,007023	4,033517	protein_coding	CD1C	CD1c molecule [Source:HGNC Symbol;Acc:HGNC:1636]	1
ENSG00000167851	2,500398	2,621579	0,000852	0,007026	4,032305	protein_coding	CD300A	CD300a molecule [Source:HGNC Symbol;Acc:HGNC:19319]	17
ENSG00000213088	-1,579323	4,067758	0,000852	0,007026	4,031909	protein_coding	ACKR1	atypical chemokine receptor 1 (Duffy blood group) [Source:HGNC Symbol;Acc:HGNC:4035]	1
ENSG00000184293	-2,646161	3,128879	0,000854	0,007026	4,031231	protein_coding	CLECL1	C-type lectin like 1 [Source:HGNC Symbol;Acc:HGNC:24462]	12
ENSG00000179240	3,433674	1,873058	0,000853	0,007026	4,031527	protein_coding	GVQW3	GVQW motif containing 3 [Source:HGNC Symbol;Acc:HGNC:51239]	11
ENSG00000119965	3,286343	-0,781049	0,000853	0,007026	-4,031286	protein_coding	C10orf88	chromosome 10 open reading frame 88 [Source:HGNC Symbol;Acc:HGNC:25822]	10
ENSG00000180626	4,238884	1,411332	0,000852	0,007026	4,032223	protein_coding	ZNF594	zinc finger protein 594 [Source:HGNC Symbol;Acc:HGNC:29392]	17
ENSG00000197860	4,739931	-0,821762	0,000852	0,007026	-4,032106	protein_coding	SGTB	small glutamine rich tetratricopeptide repeat containing beta [Source:HGNC Symbol;Acc:HGNC:23567]	5
ENSG00000138413	6,033711	-1,048784	0,000851	0,007026	-4,032723	protein_coding	IDH1	isocitrate dehydrogenase (NADP(+)) 1 [Source:HGNC Symbol;Acc:HGNC:5382]	2
ENSG00000186174	7,213648	-0,94385	0,00085	0,007026	-4,032904	protein_coding	BCL9L	BCL9 like [Source:HGNC Symbol;Acc:HGNC:23688]	11
ENSG00000141622	0,408407	3,072809	0,000855	0,007036	4,030332	protein_coding	RNF165	ring finger protein 165 [Source:HGNC Symbol;Acc:HGNC:31696]	18
ENSG00000256436	0,206227	2,172266	0,000857	0,007039	4,029457	protein_coding	TAS2R31	taste 2 receptor member 31 [Source:HGNC Symbol;Acc:HGNC:19113]	12
ENSG00000080546	4,230298	1,259448	0,000856	0,007039	4,029879	protein_coding	SESN1	sestrin 1 [Source:HGNC Symbol;Acc:HGNC:21595]	6
ENSG00000136888	6,525446	-0,716924	0,000856	0,007039	-4,029674	protein_coding	ATP6V1G1	ATPase H+ transporting V1 subunit G1 [Source:HGNC Symbol;Acc:HGNC:864]	9
ENSG00000144744	4,99362	-0,678244	0,000858	0,007044	-4,02865	protein_coding	UBA3	ubiquitin like modifier activating enzyme 3 [Source:HGNC Symbol;Acc:HGNC:12470]	3
ENSG00000103018	5,148506	-0,545405	0,000858	0,007044	-4,028711	protein_coding	CYB5B	cytochrome b5 type B [Source:HGNC Symbol;Acc:HGNC:24374]	16
ENSG00000147485	-0,131198	1,831713	0,00086	0,007049	4,027856	protein_coding	PXDNL	peroxidasin like [Source:HGNC Symbol;Acc:HGNC:26359]	8
ENSG00000161860	-0,423606	2,023501	0,00086	0,007049	4,027931	protein_coding	SYCE2	synaptonemal complex central element protein 2 [Source:HGNC Symbol;Acc:HGNC:27411]	19
ENSG00000177464	0,360267	3,521832	0,000862	0,007061	4,026676	protein_coding	GPR4	G protein-coupled receptor 4 [Source:HGNC Symbol;Acc:HGNC:4497]	19
ENSG00000158042	5,015623	-0,641137	0,000862	0,007061	-4,026632	protein_coding	MRPL17	mitochondrial ribosomal protein L17 [Source:HGNC Symbol;Acc:HGNC:14053]	11
ENSG00000054690	4,750768	1,633249	0,000866	0,007088	4,02467	protein_coding	PLEKHH1	pleckstrin homology, MyTH4 and FERM domain containing H1 [Source:HGNC Symbol;Acc:HGNC:17733]	14
ENSG00000206075	-0,775029	-3,191871	0,000866	0,007089	-4,024375	protein_coding	SERPINB5	serpin family B member 5 [Source:HGNC Symbol;Acc:HGNC:8949]	18
ENSG00000105877	1,043609	2,781158	0,000869	0,007104	4,023165	protein_coding	DNAH11	dynein axonemal heavy chain 11 [Source:HGNC Symbol;Acc:HGNC:2942]	7
ENSG00000143013	4,894112	-1,196507	0,00087	0,007108	-4,022448	protein_coding	LMO4	LIM domain only 4 [Source:HGNC Symbol;Acc:HGNC:6644]	1

ENSG00000174136	5,42319	-0,748469	0,00087	0,007108	-4,022192	protein_coding	RGMB	repulsive guidance molecule BMP co-receptor b [Source:HGNC Symbol;Acc:HGNC:26896]	5
ENSG00000182551	5,997472	-1,047181	0,000871	0,007108	-4,022178	protein_coding	ADI1	acireductone dioxygenase 1 [Source:HGNC Symbol;Acc:HGNC:30576]	2
ENSG00000187801	3,133193	-0,951972	0,000872	0,007118	-4,021323	protein_coding	ZFP69B	ZFP69 zinc finger protein B [Source:HGNC Symbol;Acc:HGNC:28053]	1
ENSG00000173039	6,13755	-0,686146	0,000874	0,007128	-4,020479	protein_coding	RELA	RELA proto-oncogene, NF-kB subunit [Source:HGNC Symbol;Acc:HGNC:9955]	11
ENSG00000137265	-0,429034	3,173312	0,000877	0,007148	4,018938	protein_coding	IRF4	interferon regulatory factor 4 [Source:HGNC Symbol;Acc:HGNC:6119]	6
ENSG00000186522	5,559216	-1,302847	0,000877	0,007148	-4,018687	protein_coding	SEPTIN10	septin 10 [Source:HGNC Symbol;Acc:HGNC:14349]	2
ENSG00000152133	4,597081	-0,756397	0,000879	0,007159	-4,017527	protein_coding	GPATCH11	G-patch domain containing 11 [Source:HGNC Symbol;Acc:HGNC:26768]	2
ENSG00000175115	6,873873	-0,899565	0,000879	0,007159	-4,017727	protein_coding	PACS1	phosphofurin acidic cluster sorting protein 1 [Source:HGNC Symbol;Acc:HGNC:30032]	11
ENSG00000154263	3,09096	2,288858	0,000882	0,007178	4,015985	protein_coding	ABCA10	ATP binding cassette subfamily A member 10 [Source:HGNC Symbol;Acc:HGNC:30]	17
ENSG00000212747	3,84941	-1,187709	0,000883	0,007178	-4,015833	protein_coding	RTL8B	retrotransposon Gag like 8B [Source:HGNC Symbol;Acc:HGNC:33156]	X
ENSG00000249115	4,475231	1,328157	0,000884	0,007186	4,01512	protein_coding	HAUS5	HAUS augmin like complex subunit 5 [Source:HGNC Symbol;Acc:HGNC:29130]	19
ENSG00000261652	0,539946	-1,387655	0,000885	0,007188	-4,014778	protein_coding	C15orf65	chromosome 15 open reading frame 65 [Source:HGNC Symbol;Acc:HGNC:44654]	15
ENSG00000111802	5,854497	-1,000283	0,000886	0,007192	-4,014249	protein_coding	TDP2	tyrosyl-DNA phosphodiesterase 2 [Source:HGNC Symbol;Acc:HGNC:17768]	6
ENSG00000163517	3,580293	1,471221	0,000892	0,007236	4,010932	protein_coding	HDAC11	histone deacetylase 11 [Source:HGNC Symbol;Acc:HGNC:19086]	3
ENSG00000212907	9,993852	-1,33371	0,000892	0,007236	-4,010898	protein_coding	MT-ND4L	mitochondrially encoded NADH:ubiquinone oxidoreductase core subunit 4L [Source:HGNC Symbol;Acc:HGNC:7460]	MT
ENSG00000173276	5,739985	-0,638436	0,000892	0,007236	-4,010764	protein_coding	ZBTB21	zinc finger and BTB domain containing 21 [Source:HGNC Symbol;Acc:HGNC:13083]	21
ENSG00000008018	6,016556	-0,770701	0,000893	0,00724	-4,010277	protein_coding	PSMB1	proteasome 20S subunit beta 1 [Source:HGNC Symbol;Acc:HGNC:9537]	6
ENSG00000178404	1,341819	2,205735	0,000895	0,007246	4,009544	protein_coding	CEP295NL	CEP295 N-terminal like [Source:HGNC Symbol;Acc:HGNC:44659]	17
ENSG00000116030	5,595635	-0,757093	0,000895	0,007246	-4,009475	protein_coding	SUMO1	small ubiquitin like modifier 1 [Source:HGNC Symbol;Acc:HGNC:12502]	2
ENSG00000102572	7,402172	-0,769106	0,000897	0,007257	-4,008538	protein_coding	STK24	serine/threonine kinase 24 [Source:HGNC Symbol;Acc:HGNC:11403]	13
ENSG00000188997	3,650008	-1,077981	0,000897	0,007257	-4,008285	protein_coding	KCTD21	potassium channel tetramerization domain containing 21 [Source:HGNC Symbol;Acc:HGNC:27452]	11
ENSG00000243749	-0,931647	-2,02463	0,000898	0,007258	-4,007974	protein_coding	TMEM35B	transmembrane protein 35B [Source:HGNC Symbol;Acc:HGNC:40021]	1
ENSG00000115084	7,088985	-0,677721	0,000899	0,007265	-4,007361	protein_coding	SLC35F5	solute carrier family 35 member F5 [Source:HGNC Symbol;Acc:HGNC:23617]	2
ENSG00000129535	0,879006	1,187619	0,000903	0,007292	4,005415	protein_coding	NRL	neural retina leucine zipper [Source:HGNC Symbol;Acc:HGNC:8002]	14
ENSG00000131400	-0,859738	1,917601	0,000905	0,007306	4,00432	protein_coding	NAPSA	napsin A aspartic peptidase [Source:HGNC Symbol;Acc:HGNC:13395]	19
ENSG00000139433	4,812414	-0,71505	0,000906	0,007313	-4,003641	protein_coding	GLTP	glycolipid transfer protein [Source:HGNC Symbol;Acc:HGNC:24867]	12
ENSG00000138587	2,982677	1,360409	0,00091	0,007335	4,001755	protein_coding	MNS1	meiosis specific nuclear structural 1 [Source:HGNC Symbol;Acc:HGNC:29636]	15
ENSG00000122696	3,690295	-0,723279	0,00091	0,007335	-4,001926	protein_coding	SLC25A51	solute carrier family 25 member 51 [Source:HGNC Symbol;Acc:HGNC:23323]	9
ENSG00000178921	4,920665	0,757648	0,000911	0,007337	4,001406	protein_coding	PFAS	phosphoribosylformylglycinamidine synthase [Source:HGNC Symbol;Acc:HGNC:8863]	17
ENSG00000184933	-2,246867	2,535777	0,000912	0,00734	4,00099	protein_coding	OR6A2	olfactory receptor family 6 subfamily A member 2 [Source:HGNC Symbol;Acc:HGNC:15301]	11
ENSG00000198939	0,499084	1,728532	0,000913	0,007348	4,000269	protein_coding	ZFP2	ZFP2 zinc finger protein [Source:HGNC Symbol;Acc:HGNC:26138]	5
ENSG00000141522	7,945862	-0,541807	0,000914	0,007353	-3,999742	protein_coding	ARHGDIA	Rho GDP dissociation inhibitor alpha [Source:HGNC Symbol;Acc:HGNC:678]	17
ENSG00000117133	4,610082	-0,861929	0,000918	0,00738	-3,997843	protein_coding	RPF1	ribosome production factor 1 homolog [Source:HGNC Symbol;Acc:HGNC:30350]	1
ENSG00000162623	4,843989	-0,64046	0,000919	0,007383	-3,997406	protein_coding	TYW3	tRNA-yW synthesizing protein 3 homolog [Source:HGNC Symbol;Acc:HGNC:24757]	1
ENSG00000152818	8,459795	1,175995	0,000921	0,007399	3,996172	protein_coding	UTRN	utrophin [Source:HGNC Symbol;Acc:HGNC:12635]	6
ENSG00000142583	1,706224	2,360027	0,000924	0,007419	3,99472	protein_coding	SLC2A5	solute carrier family 2 member 5 [Source:HGNC Symbol;Acc:HGNC:11010]	1
ENSG00000168003	7,18588	-0,953869	0,000924	0,007419	-3,994523	protein_coding	SLC3A2	solute carrier family 3 member 2 [Source:HGNC Symbol;Acc:HGNC:11026]	11
ENSG00000162896	-0,414608	3,859497	0,00093	0,007456	3,99201	protein_coding	PIGR	polymeric immunoglobulin receptor [Source:HGNC Symbol;Acc:HGNC:8968]	1
ENSG00000177879	5,455473	-0,913338	0,000931	0,007467	-3,991056	protein_coding	AP3S1	adaptor related protein complex 3 subunit sigma 1 [Source:HGNC Symbol;Acc:HGNC:2013]	5
ENSG00000101346	6,669184	-0,516689	0,000935	0,007495	-3,98914	protein_coding	POFUT1	protein O-fucosyltransferase 1 [Source:HGNC Symbol;Acc:HGNC:14988]	20
ENSG00000259431	2,906864	0,798963	0,000936	0,007498	3,988714	protein_coding	THTPA	thiamine triphosphatase [Source:HGNC Symbol;Acc:HGNC:18987]	14
ENSG00000170340	5,117376	-0,848205	0,000937	0,007501	-3,988183	protein_coding	B3GNT2	UDP-GlcNAc:betaGal beta-1,3-N-acetylglucosaminyltransferase 2 [Source:HGNC Symbol;Acc:HGNC:15629]	2
ENSG00000173210	5,444842	-1,59304	0,000938	0,007501	-3,988072	protein_coding	ABLIM3	actin binding LIM protein family member 3 [Source:HGNC Symbol;Acc:HGNC:29132]	5
ENSG00000189320	1,949925	-3,244794	0,000939	0,007504	-3,987209	protein_coding	FAM180A	family with sequence similarity 180 member A [Source:HGNC Symbol;Acc:HGNC:33773]	7
ENSG00000172650	-1,601727	1,910891	0,000939	0,007504	3,987254	protein_coding	AGAP5	ArfGAP with GTPase domain, ankyrin repeat and PH domain 5 [Source:HGNC Symbol;Acc:HGNC:23467]	10
ENSG00000163453	8,784193	-1,450348	0,000939	0,007504	-3,98755	protein_coding	IGFBP7	insulin like growth factor binding protein 7 [Source:HGNC Symbol;Acc:HGNC:5476]	4
ENSG00000010361	2,844009	0,966264	0,000941	0,00751	3,986609	protein_coding	FUZ	fuzzy planar cell polarity protein [Source:HGNC Symbol;Acc:HGNC:26219]	19
ENSG00000105792	2,063711	1,838448	0,000941	0,007511	3,986356	protein_coding	CFAP69	cilia and flagella associated protein 69 [Source:HGNC Symbol;Acc:HGNC:26107]	7
ENSG00000183908	-1,584817	3,405911	0,000942	0,007514	3,985951	protein_coding	LRRC55	leucine rich repeat containing 55 [Source:HGNC Symbol;Acc:HGNC:32324]	11
ENSG00000119242	5,891508	-1,362351	0,000946	0,007539	-3,984191	protein_coding	CCDC92	coiled-coil domain containing 92 [Source:HGNC Symbol;Acc:HGNC:29563]	12
ENSG00000068366	7,084094	-1,422507	0,000947	0,007544	-3,983665	protein_coding	ACSL4	acyl-CoA synthetase long chain family member 4 [Source:HGNC Symbol;Acc:HGNC:3571]	X

ENSG00000104714	5,240903	1,002921	0,000948	0,007545	3,983136	protein_coding	ERICH1	glutamate rich 1 [Source:HGNC Symbol;Acc:HGNC:27234]	8
ENSG00000164054	5,633716	-0,945376	0,000947	0,007545	-3,983349	protein_coding	SHISA5	shisa family member 5 [Source:HGNC Symbol;Acc:HGNC:30376]	3
ENSG00000185652	-0,467032	2,867185	0,000948	0,007546	3,982881	protein_coding	NTF3	neurotrophin 3 [Source:HGNC Symbol;Acc:HGNC:8023]	12
ENSG00000148688	3,914042	-0,647487	0,000949	0,007549	-3,982444	protein_coding	RPP30	ribonuclease P/MRP subunit p30 [Source:HGNC Symbol;Acc:HGNC:17688]	10
ENSG00000181031	5,003354	2,023104	0,000952	0,007571	3,980867	protein_coding	RPH3AL	rabphilin 3A like (without C2 domains) [Source:HGNC Symbol;Acc:HGNC:10296]	17
ENSG00000182768	5,36441	-1,019521	0,000955	0,007582	-3,979809	protein_coding	NGRN	neugrin, neurite outgrowth associated [Source:HGNC Symbol;Acc:HGNC:18077]	15
ENSG00000088986	6,96968	-0,868009	0,000955	0,007582	-3,979802	protein_coding	DYNLL1	dynein light chain LC8-type 1 [Source:HGNC Symbol;Acc:HGNC:15476]	12
ENSG00000133961	6,83362	-0,86788	0,000957	0,007598	-3,978556	protein_coding	NUMB	NUMB endocytic adaptor protein [Source:HGNC Symbol;Acc:HGNC:8060]	14
ENSG00000104442	5,071289	-0,568386	0,000959	0,007611	-3,977577	protein_coding	ARMC1	armadillo repeat containing 1 [Source:HGNC Symbol;Acc:HGNC:17684]	8
ENSG00000095059	1,982053	1,390492	0,00096	0,007613	3,97722	protein_coding	DHPS	deoxyhypusine synthase [Source:HGNC Symbol;Acc:HGNC:2869]	19
ENSG00000144118	5,814831	-0,869123	0,000962	0,007623	-3,97638	protein_coding	RALB	RAS like proto-oncogene B [Source:HGNC Symbol;Acc:HGNC:9840]	2
ENSG00000171246	5,395352	-3,664466	0,000965	0,007649	-3,974638	protein_coding	NPTX1	neuronal pentraxin 1 [Source:HGNC Symbol;Acc:HGNC:7952]	17
ENSG00000112304	4,442829	-0,946289	0,000967	0,007655	-3,973776	protein_coding	ACOT13	acyl-CoA thioesterase 13 [Source:HGNC Symbol;Acc:HGNC:20999]	6
ENSG00000178952	6,913196	-0,611284	0,000967	0,007655	-3,973965	protein_coding	TUFM	Tu translation elongation factor, mitochondrial [Source:HGNC Symbol;Acc:HGNC:12420]	16
ENSG00000178343	-1,984489	2,849839	0,000968	0,007658	3,973403	protein_coding	SHISA3	shisa family member 3 [Source:HGNC Symbol;Acc:HGNC:25159]	4
ENSG00000149257	7,92499	-1,655259	0,000969	0,00766	-3,972847	protein_coding	SERPINH1	serpin family H member 1 [Source:HGNC Symbol;Acc:HGNC:1546]	11
ENSG00000146731	7,48782	-0,839742	0,000969	0,00766	-3,97303	protein_coding	CCT6A	chaperonin containing TCP1 subunit 6A [Source:HGNC Symbol;Acc:HGNC:1620]	7
ENSG00000133597	3,980329	0,783656	0,000971	0,007669	3,972076	protein_coding	ADCK2	aarF domain containing kinase 2 [Source:HGNC Symbol;Acc:HGNC:19039]	7
ENSG00000113761	4,51632	0,859203	0,000972	0,007677	3,971389	protein_coding	ZNF346	zinc finger protein 346 [Source:HGNC Symbol;Acc:HGNC:16403]	5
ENSG00000138101	3,9228	1,842651	0,000973	0,007678	3,97107	protein_coding	DTNB	dystrobrevin beta [Source:HGNC Symbol;Acc:HGNC:3058]	2
ENSG00000099290	3,97985	-0,806547	0,000976	0,007703	-3,969381	protein_coding	WASHC2A	WASH complex subunit 2A [Source:HGNC Symbol;Acc:HGNC:23416]	10
ENSG00000165185	5,079142	1,142597	0,000978	0,00771	3,968753	protein_coding	KIAA1958	KIAA1958 [Source:HGNC Symbol;Acc:HGNC:23427]	9
ENSG00000153060	-3,179846	2,952175	0,000979	0,007714	3,968244	protein_coding	TEKT5	tektin 5 [Source:HGNC Symbol;Acc:HGNC:26554]	16
ENSG00000171163	4,196563	1,046492	0,000979	0,007715	3,967962	protein_coding	ZNF692	zinc finger protein 692 [Source:HGNC Symbol;Acc:HGNC:26049]	1
ENSG00000180448	3,698363	2,364523	0,000984	0,00775	3,965675	protein_coding	ARHGAP45	Rho GTPase activating protein 45 [Source:HGNC Symbol;Acc:HGNC:17102]	19
ENSG00000260007	-0,789115	1,83906	0,000986	0,007759	3,964932	protein_coding	AC107871.	novel protein	15
ENSG00000182774	-1,089436	-1,802395	0,000988	0,007765	-3,964145	protein_coding	RPS17	ribosomal protein S17 [Source:HGNC Symbol;Acc:HGNC:10397]	15
ENSG00000124608	4,879759	0,707566	0,000987	0,007765	3,964326	protein_coding	AARS2	alanyl-tRNA synthetase 2, mitochondrial [Source:HGNC Symbol;Acc:HGNC:21022]	6
ENSG00000149922	0,337911	1,848795	0,000992	0,007793	3,962265	protein_coding	TBX6	T-box transcription factor 6 [Source:HGNC Symbol;Acc:HGNC:11605]	16
ENSG00000196189	1,333188	2,189994	0,000993	0,007798	3,961504	protein_coding	SEMA4A	semaphorin 4A [Source:HGNC Symbol;Acc:HGNC:10729]	1
ENSG00000232112	6,429936	0,739416	0,000993	0,007798	3,961708	protein_coding	TMA7	translation machinery associated 7 homolog [Source:HGNC Symbol;Acc:HGNC:26932]	3
ENSG00000119596	8,015985	0,467928	0,000994	0,007803	3,960998	protein_coding	YLPM1	YLP motif containing 1 [Source:HGNC Symbol;Acc:HGNC:17798]	14
ENSG00000205937	6,781578	-0,558569	0,000995	0,007807	-3,960557	protein_coding	RNPS1	RNA binding protein with serine rich domain 1 [Source:HGNC Symbol;Acc:HGNC:10080]	16
ENSG00000138378	0,862778	2,527156	0,000996	0,007809	3,960199	protein_coding	STAT4	signal transducer and activator of transcription 4 [Source:HGNC Symbol;Acc:HGNC:11365]	2
ENSG00000169926	7,109989	-0,921236	0,000997	0,007809	-3,959994	protein_coding	KLF13	Kruppel like factor 13 [Source:HGNC Symbol;Acc:HGNC:13672]	15
ENSG00000213928	1,775201	1,150441	0,000998	0,007812	3,959166	protein_coding	IRF9	interferon regulatory factor 9 [Source:HGNC Symbol;Acc:HGNC:6131]	14
ENSG00000140992	5,767428	-0,560487	0,000998	0,007812	-3,95949	protein_coding	PDPK1	3-phosphoinositide dependent protein kinase 1 [Source:HGNC Symbol;Acc:HGNC:8816]	16
ENSG00000077232	7,654753	-0,743225	0,000999	0,007812	-3,959113	protein_coding	DNAJC10	DnaJ heat shock protein family (Hsp40) member C10 [Source:HGNC Symbol;Acc:HGNC:24637]	2
ENSG00000203710	0,28775	4,990953	0,001001	0,007823	3,958032	protein_coding	CR1	complement C3b/C4b receptor 1 (Knops blood group) [Source:HGNC Symbol;Acc:HGNC:2334]	1
ENSG00000121039	5,402576	-1,47056	0,001001	0,007823	-3,95811	protein_coding	RDH10	retinol dehydrogenase 10 [Source:HGNC Symbol;Acc:HGNC:19975]	8
ENSG00000081014	5,09598	-0,622752	0,001001	0,007824	-3,957772	protein_coding	AP4E1	adaptor related protein complex 4 subunit epsilon 1 [Source:HGNC Symbol;Acc:HGNC:573]	15
ENSG00000185453	3,549097	1,038373	0,001005	0,007841	3,95632	protein_coding	ZSWIM9	zinc finger SWIM-type containing 9 [Source:HGNC Symbol;Acc:HGNC:34495]	19
ENSG00000157654	7,943452	-1,502089	0,001005	0,007841	-3,956339	protein_coding	PALM2AKA	PALM2 and AKAP2 fusion [Source:HGNC Symbol;Acc:HGNC:33529]	9
ENSG00000139636	4,42013	0,763239	0,001006	0,007847	3,95575	protein_coding	LMBR1L	limb development membrane protein 1 like [Source:HGNC Symbol;Acc:HGNC:18268]	12
ENSG00000100359	2,007042	1,207082	0,001009	0,007866	3,954403	protein_coding	SGSM3	small G protein signaling modulator 3 [Source:HGNC Symbol;Acc:HGNC:25228]	22
ENSG00000213523	4,843613	-0,570505	0,00101	0,007872	-3,953655	protein_coding	SRA1	steroid receptor RNA activator 1 [Source:HGNC Symbol;Acc:HGNC:11281]	5
ENSG00000124383	5,616825	-0,858311	0,00101	0,007872	-3,953725	protein_coding	MPHOSPH:	M-phase phosphoprotein 10 [Source:HGNC Symbol;Acc:HGNC:7213]	2
ENSG00000177225	5,180979	0,854618	0,001013	0,007886	3,9526	protein_coding	GATD1	glutamine amidotransferase like class 1 domain containing 1 [Source:HGNC Symbol;Acc:HGNC:26616]	11
ENSG00000104883	0,409177	1,295361	0,001015	0,007903	3,951395	protein_coding	PEX11G	peroxisomal biogenesis factor 11 gamma [Source:HGNC Symbol;Acc:HGNC:20208]	19
ENSG00000162616	4,782311	-1,420006	0,001017	0,00791	-3,950768	protein_coding	DNAJB4	DnaJ heat shock protein family (Hsp40) member B4 [Source:HGNC Symbol;Acc:HGNC:14886]	1
ENSG00000196932	1,839783	-1,821497	0,001019	0,007924	-3,949745	protein_coding	TMEM26	transmembrane protein 26 [Source:HGNC Symbol;Acc:HGNC:28550]	10

ENSG00000108950	2,041266	3,200926	0,001024	0,007955	3,947517	protein_coding	FAM20A	FAM20A golgi associated secretory pathway pseudokinase [Source:HGNC Symbol;Acc:HGNC:23015]	17
ENSG00000014824	6,292783	-0,742179	0,001024	0,007955	-3,94756	protein_coding	SLC30A9	solute carrier family 30 member 9 [Source:HGNC Symbol;Acc:HGNC:1329]	4
ENSG00000112715	8,383218	1,215001	0,001028	0,007978	3,945749	protein_coding	VEGFA	vascular endothelial growth factor A [Source:HGNC Symbol;Acc:HGNC:12680]	6
ENSG00000198198	6,515449	0,638819	0,001028	0,007978	3,945955	protein_coding	SZT2	SZT2 subunit of KICSTOR complex [Source:HGNC Symbol;Acc:HGNC:29040]	1
ENSG00000115423	1,128641	4,131024	0,001033	0,007978	3,943321	protein_coding	DNAH6	dynein axonemal heavy chain 6 [Source:HGNC Symbol;Acc:HGNC:2951]	2
ENSG00000106336	-0,453548	1,659581	0,001034	0,007978	3,943308	protein_coding	FBXO24	F-box protein 24 [Source:HGNC Symbol;Acc:HGNC:13595]	7
ENSG00000172156	-2,272721	3,749357	0,001034	0,007978	3,943084	protein_coding	CCL11	C-C motif chemokine ligand 11 [Source:HGNC Symbol;Acc:HGNC:10610]	17
ENSG00000120915	3,820599	2,379393	0,001033	0,007978	3,943317	protein_coding	EPHX2	epoxide hydrolase 2 [Source:HGNC Symbol;Acc:HGNC:3402]	8
ENSG00000138172	3,784642	-1,331092	0,001034	0,007978	-3,943156	protein_coding	CALHM2	calcium homeostasis modulator family member 2 [Source:HGNC Symbol;Acc:HGNC:23493]	10
ENSG00000124813	3,818074	-1,551765	0,001033	0,007978	-3,943523	protein_coding	RUNX2	RUNX family transcription factor 2 [Source:HGNC Symbol;Acc:HGNC:10472]	6
ENSG00000197044	3,913674	0,858181	0,00103	0,007978	3,944707	protein_coding	ZNF441	zinc finger protein 441 [Source:HGNC Symbol;Acc:HGNC:20875]	19
ENSG00000109113	4,229798	-1,736337	0,001034	0,007978	-3,943144	protein_coding	RAB34	RAB34, member RAS oncogene family [Source:HGNC Symbol;Acc:HGNC:16519]	17
ENSG00000180376	4,628665	0,916463	0,00103	0,007978	3,944976	protein_coding	CCDC66	coiled-coil domain containing 66 [Source:HGNC Symbol;Acc:HGNC:27709]	3
ENSG00000115738	5,590084	-1,635133	0,001031	0,007978	-3,94449	protein_coding	ID2	inhibitor of DNA binding 2 [Source:HGNC Symbol;Acc:HGNC:5361]	2
ENSG00000107554	5,948802	-1,291442	0,001031	0,007978	-3,944298	protein_coding	DNMBP	dynamin binding protein [Source:HGNC Symbol;Acc:HGNC:30373]	10
ENSG00000005893	6,93845	-0,883051	0,001032	0,007978	-3,944128	protein_coding	LAMP2	lysosomal associated membrane protein 2 [Source:HGNC Symbol;Acc:HGNC:6501]	X
ENSG00000145700	0,153558	1,976408	0,001037	0,007992	3,941949	protein_coding	ANKRD31	ankyrin repeat domain 31 [Source:HGNC Symbol;Acc:HGNC:26853]	5
ENSG00000185340	4,663861	-1,279963	0,001037	0,007992	-3,941842	protein_coding	GAS2L1	growth arrest specific 2 like 1 [Source:HGNC Symbol;Acc:HGNC:16955]	22
ENSG00000170385	5,498288	-1,009466	0,001038	0,007999	-3,941232	protein_coding	SLC30A1	solute carrier family 30 member 1 [Source:HGNC Symbol;Acc:HGNC:11012]	1
ENSG00000119778	5,381292	0,747148	0,001039	0,008004	3,940735	protein_coding	ATAD2B	ATPase family AAA domain containing 2B [Source:HGNC Symbol;Acc:HGNC:29230]	2
ENSG00000136045	5,696197	-0,484225	0,001041	0,008013	-3,940032	protein_coding	PWP1	PWP1 homolog, endonuclein [Source:HGNC Symbol;Acc:HGNC:17015]	12
ENSG00000157741	6,505921	0,73397	0,001043	0,008029	3,9389	protein_coding	UBN2	ubinuclein 2 [Source:HGNC Symbol;Acc:HGNC:21931]	7
ENSG00000163874	3,551944	-1,255138	0,001044	0,00803	-3,938603	protein_coding	ZC3H12A	zinc finger CCCH-type containing 12A [Source:HGNC Symbol;Acc:HGNC:26259]	1
ENSG00000197785	5,066491	-0,708765	0,001045	0,00803	-3,938391	protein_coding	ATAD3A	ATPase family AAA domain containing 3A [Source:HGNC Symbol;Acc:HGNC:25567]	1
ENSG00000028839	3,492119	-1,010356	0,001048	0,008051	-3,93694	protein_coding	TBPL1	TATA-box binding protein like 1 [Source:HGNC Symbol;Acc:HGNC:11589]	6
ENSG00000177943	3,319218	1,627999	0,001052	0,008067	3,935152	protein_coding	MAMDC4	MAM domain containing 4 [Source:HGNC Symbol;Acc:HGNC:24083]	9
ENSG00000116521	5,626801	-0,889064	0,001051	0,008067	-3,935397	protein_coding	SCAMP3	secretory carrier membrane protein 3 [Source:HGNC Symbol;Acc:HGNC:10565]	1
ENSG00000128272	8,016564	-0,766853	0,001052	0,008067	-3,934981	protein_coding	ATF4	activating transcription factor 4 [Source:HGNC Symbol;Acc:HGNC:786]	22
ENSG00000184863	7,287679	0,658188	0,001052	0,008067	3,935364	protein_coding	RBM33	RNA binding motif protein 33 [Source:HGNC Symbol;Acc:HGNC:27223]	7
ENSG00000049860	6,853935	-0,896641	0,001052	0,008067	-3,93529	protein_coding	HEXB	hexosaminidase subunit beta [Source:HGNC Symbol;Acc:HGNC:4879]	5
ENSG00000180871	-2,298075	3,332086	0,001055	0,008085	3,933752	protein_coding	CXCR2	C-X-C motif chemokine receptor 2 [Source:HGNC Symbol;Acc:HGNC:6027]	2
ENSG00000145882	4,075971	1,262448	0,001058	0,008095	3,932741	protein_coding	PCYOX1L	prenylcysteine oxidase 1 like [Source:HGNC Symbol;Acc:HGNC:28477]	5
ENSG00000162714	5,584526	1,097363	0,001057	0,008095	3,932767	protein_coding	ZNF496	zinc finger protein 496 [Source:HGNC Symbol;Acc:HGNC:23713]	1
ENSG00000172717	-1,874228	2,048554	0,001058	0,008095	3,932447	protein_coding	FAM71D	family with sequence similarity 71 member D [Source:HGNC Symbol;Acc:HGNC:20101]	14
ENSG00000049192	4,061437	-1,353944	0,001059	0,008095	-3,932288	protein_coding	ADAMTS6	ADAM metallopeptidase with thrombospondin type 1 motif 6 [Source:HGNC Symbol;Acc:HGNC:222]	5
ENSG00000127914	8,520223	0,788819	0,00106	0,008105	3,93151	protein_coding	AKAP9	A-kinase anchoring protein 9 [Source:HGNC Symbol;Acc:HGNC:379]	7
ENSG00000073792	4,544552	-1,457419	0,001063	0,008123	-3,930292	protein_coding	IGF2BP2	insulin like growth factor 2 mRNA binding protein 2 [Source:HGNC Symbol;Acc:HGNC:28867]	3
ENSG00000284770	1,587936	1,43942	0,001064	0,008123	3,92991	protein_coding	TBCE	tubulin folding cofactor E [Source:HGNC Symbol;Acc:HGNC:11582]	1
ENSG00000076706	6,005394	1,188161	0,001064	0,008123	3,929852	protein_coding	MCAM	melanoma cell adhesion molecule [Source:HGNC Symbol;Acc:HGNC:6934]	11
ENSG00000135315	4,385771	0,859228	0,001068	0,008147	3,928291	protein_coding	CEP162	centrosomal protein 162 [Source:HGNC Symbol;Acc:HGNC:21107]	6
ENSG00000012822	6,472248	0,691373	0,00107	0,008157	3,927482	protein_coding	CALCOCO1	calcium binding and coiled-coil domain 1 [Source:HGNC Symbol;Acc:HGNC:29306]	12
ENSG00000164241	2,392593	1,663142	0,001072	0,008172	3,926443	protein_coding	C5orf63	chromosome 5 open reading frame 63 [Source:HGNC Symbol;Acc:HGNC:40051]	5
ENSG00000042445	4,977665	-0,822481	0,001073	0,008173	-3,926184	protein_coding	RETSAT	retinol saturase [Source:HGNC Symbol;Acc:HGNC:25991]	2
ENSG00000101955	3,340734	-2,154898	0,001074	0,00818	-3,925549	protein_coding	SRPX	sushi repeat containing protein X-linked [Source:HGNC Symbol;Acc:HGNC:11309]	X
ENSG00000141424	6,689969	-0,978805	0,001075	0,00818	-3,925354	protein_coding	SLC39A6	solute carrier family 39 member 6 [Source:HGNC Symbol;Acc:HGNC:18607]	18
ENSG00000100628	-1,069653	2,821015	0,001076	0,008182	3,924995	protein_coding	ASB2	ankyrin repeat and SOCS box containing 2 [Source:HGNC Symbol;Acc:HGNC:16012]	14
ENSG00000173988	-0,484633	2,630869	0,001077	0,008187	3,924541	protein_coding	LRRC63	leucine rich repeat containing 63 [Source:HGNC Symbol;Acc:HGNC:34296]	13
ENSG00000138757	7,334403	-0,769407	0,001078	0,008194	-3,923918	protein_coding	G3BP2	G3BP stress granule assembly factor 2 [Source:HGNC Symbol;Acc:HGNC:30291]	4
ENSG00000122122	1,54511	2,723698	0,001082	0,00821	3,922138	protein_coding	SASH3	SAM and SH3 domain containing 3 [Source:HGNC Symbol;Acc:HGNC:15975]	X
ENSG00000053524	2,239562	2,46705	0,001081	0,00821	3,922604	protein_coding	MCF2L2	MCF.2 cell line derived transforming sequence-like 2 [Source:HGNC Symbol;Acc:HGNC:30319]	3
ENSG00000171804	-2,109285	2,902768	0,001082	0,00821	3,922237	protein_coding	WDR87	WD repeat domain 87 [Source:HGNC Symbol;Acc:HGNC:29934]	19

ENSG00000100360	4,233878	0,747725	0,001082	0,00821	3,922349	protein_coding	IFT27	intraflagellar transport 27 [Source:HGNC Symbol;Acc:HGNC:18626]	22
ENSG00000137033	4,716916	-5,214758	0,001085	0,008219	-3,921	protein_coding	IL33	interleukin 33 [Source:HGNC Symbol;Acc:HGNC:16028]	9
ENSG00000116117	5,094161	1,396292	0,001085	0,008219	3,921033	protein_coding	PARD3B	par-3 family cell polarity regulator beta [Source:HGNC Symbol;Acc:HGNC:14446]	2
ENSG00000120437	4,914961	-1,323166	0,001085	0,008219	-3,92101	protein_coding	ACAT2	acetyl-CoA acetyltransferase 2 [Source:HGNC Symbol;Acc:HGNC:94]	6
ENSG00000144644	-2,814481	2,611458	0,001085	0,008219	3,920782	protein_coding	GADL1	glutamate decarboxylase like 1 [Source:HGNC Symbol;Acc:HGNC:27949]	3
ENSG00000068323	5,992897	-0,680035	0,001087	0,008224	-3,920332	protein_coding	TFE3	transcription factor binding to IGHM enhancer 3 [Source:HGNC Symbol;Acc:HGNC:11752]	X
ENSG00000132294	6,859911	-0,788389	0,001087	0,008226	-3,919983	protein_coding	EFR3A	EFR3 homolog A [Source:HGNC Symbol;Acc:HGNC:28970]	8
ENSG00000113119	3,125554	1,100911	0,001089	0,008229	3,919268	protein_coding	TMCO6	transmembrane and coiled-coil domains 6 [Source:HGNC Symbol;Acc:HGNC:28814]	5
ENSG00000180902	3,83943	1,253172	0,001089	0,008229	3,919322	protein_coding	D2HGDH	D-2-hydroxyglutarate dehydrogenase [Source:HGNC Symbol;Acc:HGNC:28358]	2
ENSG00000161654	4,612601	-0,689247	0,001089	0,008229	-3,919157	protein_coding	LSM12	LSM12 homolog [Source:HGNC Symbol;Acc:HGNC:26407]	17
ENSG00000175322	3,029187	1,583407	0,001093	0,008253	3,917613	protein_coding	ZNF519	zinc finger protein 519 [Source:HGNC Symbol;Acc:HGNC:30574]	18
ENSG00000163116	-0,764627	2,203692	0,001096	0,00827	3,916484	protein_coding	STPG2	sperm tail PG-rich repeat containing 2 [Source:HGNC Symbol;Acc:HGNC:28712]	4
ENSG00000198712	11,91126	-1,453535	0,001097	0,008274	-3,916011	protein_coding	MT-CO2	mitochondrially encoded cytochrome c oxidase II [Source:HGNC Symbol;Acc:HGNC:7421]	MT
ENSG00000205209	0,09331	1,699074	0,001099	0,008288	3,915014	protein_coding	SCGB2B2	secretoglobin family 2B member 2 [Source:HGNC Symbol;Acc:HGNC:27616]	19
ENSG00000116685	5,66464	-0,744634	0,001107	0,008341	-3,911904	protein_coding	KIAA2013	KIAA2013 [Source:HGNC Symbol;Acc:HGNC:28513]	1
ENSG00000169410	5,379219	-0,719767	0,001111	0,008374	-3,909899	protein_coding	PTPN9	protein tyrosine phosphatase non-receptor type 9 [Source:HGNC Symbol;Acc:HGNC:9661]	15
ENSG00000076351	4,491215	0,840774	0,001115	0,008393	3,908608	protein_coding	SLC46A1	solute carrier family 46 member 1 [Source:HGNC Symbol;Acc:HGNC:30521]	17
ENSG00000164104	6,146857	1,221907	0,001117	0,008408	3,907616	protein_coding	HMGB2	high mobility group box 2 [Source:HGNC Symbol;Acc:HGNC:5000]	4
ENSG00000100842	3,120789	-2,010999	0,001119	0,008418	-3,906844	protein_coding	EFS	embryonal Fyn-associated substrate [Source:HGNC Symbol;Acc:HGNC:16898]	14
ENSG00000240857	3,917443	-0,658849	0,001121	0,008432	-3,905836	protein_coding	RDH14	retinol dehydrogenase 14 [Source:HGNC Symbol;Acc:HGNC:19979]	2
ENSG00000172476	-1,590362	2,780529	0,001122	0,008432	3,90562	protein_coding	RAB40A	RAB40A, member RAS oncogene family [Source:HGNC Symbol;Acc:HGNC:18283]	X
ENSG00000135002	5,002491	-0,751779	0,001124	0,008443	-3,90481	protein_coding	RFK	riboflavin kinase [Source:HGNC Symbol;Acc:HGNC:30324]	9
ENSG00000174564	-0,252696	1,596958	0,001125	0,008447	3,904399	protein_coding	IL20RB	interleukin 20 receptor subunit beta [Source:HGNC Symbol;Acc:HGNC:6004]	3
ENSG00000140939	4,068201	1,088788	0,001132	0,0085	3,901306	protein_coding	NOL3	nucleolar protein 3 [Source:HGNC Symbol;Acc:HGNC:7869]	16
ENSG00000153786	5,418772	-0,707754	0,001134	0,008503	-3,900739	protein_coding	ZDHHC7	zinc finger DHHC-type palmitoyltransferase 7 [Source:HGNC Symbol;Acc:HGNC:18459]	16
ENSG00000160633	7,38873	0,573622	0,001134	0,008503	3,900729	protein_coding	SAFB	scaffold attachment factor B [Source:HGNC Symbol;Acc:HGNC:10520]	19
ENSG00000139304	-2,230658	2,454397	0,001135	0,008505	3,900201	protein_coding	PTPRQ	protein tyrosine phosphatase receptor type Q [Source:HGNC Symbol;Acc:HGNC:9679]	12
ENSG00000013561	5,964928	-0,61655	0,001135	0,008505	-3,900202	protein_coding	RNF14	ring finger protein 14 [Source:HGNC Symbol;Acc:HGNC:10058]	5
ENSG00000149658	6,395206	-0,564085	0,001136	0,00851	-3,89974	protein_coding	YTHDF1	YTH N6-methyladenosine RNA binding protein 1 [Source:HGNC Symbol;Acc:HGNC:15867]	20
ENSG00000176714	1,418053	1,164816	0,001137	0,008511	3,899434	protein_coding	CCDC121	coiled-coil domain containing 121 [Source:HGNC Symbol;Acc:HGNC:25833]	2
ENSG00000066382	0,21849	2,770628	0,001141	0,008531	3,898023	protein_coding	MPPED2	metallophosphoesterase domain containing 2 [Source:HGNC Symbol;Acc:HGNC:1180]	11
ENSG00000132507	7,634362	-0,677458	0,001141	0,008531	-3,897942	protein_coding	EIF5A	eukaryotic translation initiation factor 5A [Source:HGNC Symbol;Acc:HGNC:3300]	17
ENSG00000087206	5,482341	0,565485	0,001142	0,008536	3,897477	protein_coding	UIMC1	ubiquitin interaction motif containing 1 [Source:HGNC Symbol;Acc:HGNC:30298]	5
ENSG00000197265	4,878619	-0,678238	0,001143	0,008541	-3,897003	protein_coding	GTF2E2	general transcription factor IIE subunit 2 [Source:HGNC Symbol;Acc:HGNC:4651]	8
ENSG00000139193	-1,583957	3,387177	0,001147	0,008565	3,895508	protein_coding	CD27	CD27 molecule [Source:HGNC Symbol;Acc:HGNC:11922]	12
ENSG00000171488	4,38348	-1,229186	0,001149	0,008576	-3,894468	protein_coding	LRRC8C	leucine rich repeat containing 8 VRAC subunit C [Source:HGNC Symbol;Acc:HGNC:25075]	1
ENSG00000116133	6,99534	-1,6052	0,001149	0,008576	-3,894483	protein_coding	DHCR24	24-dehydrocholesterol reductase [Source:HGNC Symbol;Acc:HGNC:2859]	1
ENSG00000189144	2,966124	1,135543	0,001153	0,008602	3,892886	protein_coding	ZNF573	zinc finger protein 573 [Source:HGNC Symbol;Acc:HGNC:26420]	19
ENSG00000164211	5,349799	-1,205027	0,001155	0,008607	-3,892414	protein_coding	STARD4	StAR related lipid transfer domain containing 4 [Source:HGNC Symbol;Acc:HGNC:18058]	5
ENSG00000214595	4,359635	1,489478	0,001156	0,008616	3,891695	protein_coding	EML6	EMAP like 6 [Source:HGNC Symbol;Acc:HGNC:35412]	2
ENSG00000179218	10,32193	-0,75299	0,001158	0,008628	-3,89089	protein_coding	CALR	calreticulin [Source:HGNC Symbol;Acc:HGNC:1455]	19
ENSG00000204410	1,288487	1,697202	0,001159	0,008631	3,890497	protein_coding	MSH5	mutS homolog 5 [Source:HGNC Symbol;Acc:HGNC:7328]	6
ENSG00000179021	4,140963	-0,736735	0,001162	0,008642	-3,889687	protein_coding	C3orf38	chromosome 3 open reading frame 38 [Source:HGNC Symbol;Acc:HGNC:28384]	3
ENSG00000139505	5,517427	-0,619402	0,001163	0,008648	-3,889175	protein_coding	MTMR6	myotubularin related protein 6 [Source:HGNC Symbol;Acc:HGNC:7453]	13
ENSG00000204590	6,260662	0,565837	0,001164	0,00865	3,888834	protein_coding	GNL1	G protein nucleolar 1 (putative) [Source:HGNC Symbol;Acc:HGNC:4413]	6
ENSG00000100632	5,714005	-0,652306	0,001167	0,00867	-3,887609	protein_coding	ERH	ERH mRNA splicing and mitosis factor [Source:HGNC Symbol;Acc:HGNC:3447]	14
ENSG00000168785	4,993744	-1,540428	0,00117	0,008688	-3,886403	protein_coding	TSPAN5	tetraspanin 5 [Source:HGNC Symbol;Acc:HGNC:17753]	4
ENSG00000110799	4,813534	3,548431	0,001172	0,008695	3,885659	protein_coding	VWF	von Willebrand factor [Source:HGNC Symbol;Acc:HGNC:12726]	12
ENSG00000126883	6,769408	0,515087	0,001171	0,008695	3,88586	protein_coding	NUP214	nucleoporin 214 [Source:HGNC Symbol;Acc:HGNC:8064]	9
ENSG00000266967	0,8821	1,629105	0,001173	0,008702	3,88505	protein_coding	AARSD1	alanyl-tRNA synthetase domain containing 1 [Source:HGNC Symbol;Acc:HGNC:28417]	17
ENSG00000235272	-0,998295	1,735934	0,001175	0,008707	3,884358	protein_coding	RAMACL	RNA guanine-7 methyltransferase activating subunit like (pseudogene) [Source:HGNC Symbol;Acc:HGNC:21234]	6

ENSG00000166794	8,407573	-1,031774	0,001175	0,008707	-3,884398	protein_coding	PPIB	peptidylprolyl isomerase B [Source:HGNC Symbol;Acc:HGNC:9255]	15
ENSG00000131203	-1,682508	2,834309	0,001176	0,008709	3,884083	protein_coding	IDO1	indoleamine 2,3-dioxygenase 1 [Source:HGNC Symbol;Acc:HGNC:6059]	8
ENSG00000169604	7,607369	-2,104984	0,001179	0,008726	-3,88294	protein_coding	ANTXR1	ANTXR cell adhesion molecule 1 [Source:HGNC Symbol;Acc:HGNC:21014]	2
ENSG00000166532	4,457321	1,324013	0,00118	0,008733	3,882268	protein_coding	RIMKLB	ribosomal modification protein rimK like family member B [Source:HGNC Symbol;Acc:HGNC:29228]	12
ENSG00000198420	5,377516	-1,021591	0,001181	0,008733	-3,882144	protein_coding	TCAF1	TRPM8 channel associated factor 1 [Source:HGNC Symbol;Acc:HGNC:22201]	7
ENSG00000136897	4,492346	-0,587784	0,001182	0,008739	-3,881667	protein_coding	MRPL50	mitochondrial ribosomal protein L50 [Source:HGNC Symbol;Acc:HGNC:16654]	9
ENSG00000152952	7,237007	-1,679331	0,001183	0,008744	-3,881176	protein_coding	PLOD2	procollagen-lysine,2-oxoglutarate 5-dioxygenase 2 [Source:HGNC Symbol;Acc:HGNC:9082]	3
ENSG00000174175	-1,393203	3,544674	0,001184	0,008748	3,880742	protein_coding	SELP	selectin P [Source:HGNC Symbol;Acc:HGNC:10721]	1
ENSG00000065911	6,372369	-0,95254	0,001187	0,008761	-3,879891	protein_coding	MTHFD2	methylenetetrahydrofolate dehydrogenase (NADP+ dependent) 2, methenyltetrahydrofolate cyclohydrolase [Sourc	2
ENSG00000145715	6,29272	-0,576471	0,00119	0,008778	-3,878745	protein_coding	RASA1	RAS p21 protein activator 1 [Source:HGNC Symbol;Acc:HGNC:9871]	5
ENSG00000104472	4,782277	-0,594769	0,001201	0,008858	-3,874396	protein_coding	CHRAC1	chromatin accessibility complex subunit 1 [Source:HGNC Symbol;Acc:HGNC:13544]	8
ENSG00000125409	-1,337301	2,59189	0,001206	0,008895	3,872253	protein_coding	TEKT3	tektin 3 [Source:HGNC Symbol;Acc:HGNC:14293]	17
ENSG00000110047	5,30116	-1,239808	0,001207	0,008896	-3,872022	protein_coding	EHD1	EH domain containing 1 [Source:HGNC Symbol;Acc:HGNC:3242]	11
ENSG00000152422	3,269719	-1,407617	0,001209	0,008904	-3,871376	protein_coding	XRCC4	X-ray repair cross complementing 4 [Source:HGNC Symbol;Acc:HGNC:12831]	5
ENSG00000079459	6,644005	-0,900937	0,001211	0,008919	-3,870418	protein_coding	FDFT1	farnesyl-diphosphate farnesyltransferase 1 [Source:HGNC Symbol;Acc:HGNC:3629]	8
ENSG00000077420	2,097522	2,72557	0,001214	0,008932	3,869346	protein_coding	APBB1IP	amyloid beta precursor protein binding family B member 1 interacting protein [Source:HGNC Symbol;Acc:HGNC:173	10
ENSG00000011143	3,612765	1,083176	0,001214	0,008932	3,869516	protein_coding	MKS1	MKS transition zone complex subunit 1 [Source:HGNC Symbol;Acc:HGNC:7121]	17
ENSG00000163481	4,258656	-0,654149	0,001215	0,008936	-3,868912	protein_coding	RNF25	ring finger protein 25 [Source:HGNC Symbol;Acc:HGNC:14662]	2
ENSG00000119599	3,762039	0,72366	0,001218	0,008951	3,867959	protein_coding	DCAF4	DDB1 and CUL4 associated factor 4 [Source:HGNC Symbol;Acc:HGNC:20229]	14
ENSG00000127329	4,0659	1,91688	0,001219	0,008957	3,867422	protein_coding	PTPRB	protein tyrosine phosphatase receptor type B [Source:HGNC Symbol;Acc:HGNC:9665]	12
ENSG00000003756	6,752386	0,804396	0,001221	0,008963	3,866892	protein_coding	RBM5	RNA binding motif protein 5 [Source:HGNC Symbol;Acc:HGNC:9902]	3
ENSG00000148248	7,7752	-1,008153	0,001222	0,008971	-3,866301	protein_coding	SURF4	surfeit 4 [Source:HGNC Symbol;Acc:HGNC:11476]	9
ENSG00000185127	4,514669	-0,742721	0,001224	0,008978	-3,865736	protein_coding	C6orf120	chromosome 6 open reading frame 120 [Source:HGNC Symbol;Acc:HGNC:21247]	6
ENSG00000159788	5,513687	0,763454	0,001228	0,009001	3,864198	protein_coding	RGS12	regulator of G protein signaling 12 [Source:HGNC Symbol;Acc:HGNC:9994]	4
ENSG00000204262	8,734734	-1,823521	0,001228	0,009001	-3,864119	protein_coding	COL5A2	collagen type V alpha 2 chain [Source:HGNC Symbol;Acc:HGNC:2210]	2
ENSG00000187134	4,255287	-3,435149	0,001229	0,009003	-3,863807	protein_coding	AKR1C1	aldo-keto reductase family 1 member C1 [Source:HGNC Symbol;Acc:HGNC:384]	10
ENSG00000116044	6,742196	-1,031182	0,001231	0,009012	-3,863151	protein_coding	NFE2L2	nuclear factor, erythroid 2 like 2 [Source:HGNC Symbol;Acc:HGNC:7782]	2
ENSG00000125257	5,142512	-1,827955	0,001232	0,009017	-3,862713	protein_coding	ABCC4	ATP binding cassette subfamily C member 4 [Source:HGNC Symbol;Acc:HGNC:55]	13
ENSG00000271503	0,172547	2,767677	0,001233	0,009019	3,862383	protein_coding	CCL5	C-C motif chemokine ligand 5 [Source:HGNC Symbol;Acc:HGNC:10632]	17
ENSG00000235194	4,174234	1,380708	0,001235	0,009031	3,861599	protein_coding	PPP1R3E	protein phosphatase 1 regulatory subunit 3E [Source:HGNC Symbol;Acc:HGNC:14943]	14
ENSG00000205903	5,807722	0,769098	0,001242	0,009079	3,858932	protein_coding	ZNF316	zinc finger protein 316 [Source:HGNC Symbol;Acc:HGNC:13843]	7
ENSG00000168356	-1,443696	2,292937	0,001244	0,00908	3,85826	protein_coding	SCN11A	sodium voltage-gated channel alpha subunit 11 [Source:HGNC Symbol;Acc:HGNC:10583]	3
ENSG00000070729	-2,211023	3,334705	0,001244	0,00908	3,858274	protein_coding	CNGB1	cyclic nucleotide gated channel subunit beta 1 [Source:HGNC Symbol;Acc:HGNC:2151]	16
ENSG00000175106	3,165667	1,968786	0,001243	0,00908	3,858456	protein_coding	TVP23C	trans-golgi network vesicle protein 23 homolog C [Source:HGNC Symbol;Acc:HGNC:30453]	17
ENSG00000159708	-0,863192	3,277539	0,001246	0,009085	3,857414	protein_coding	LRRC36	leucine rich repeat containing 36 [Source:HGNC Symbol;Acc:HGNC:25615]	16
ENSG00000165410	4,524252	-1,008022	0,001246	0,009085	-3,857557	protein_coding	CFL2	cofilin 2 [Source:HGNC Symbol;Acc:HGNC:1875]	14
ENSG00000166173	4,861465	-1,529573	0,001245	0,009085	-3,857728	protein_coding	LARP6	La ribonucleoprotein 6, translational regulator [Source:HGNC Symbol;Acc:HGNC:24012]	15
ENSG00000185634	2,161927	-2,88735	0,001249	0,009098	-3,856524	protein_coding	SHC4	SHC adaptor protein 4 [Source:HGNC Symbol;Acc:HGNC:16743]	15
ENSG00000112280	-0,977114	2,488495	0,001257	0,009156	3,853416	protein_coding	COL9A1	collagen type IX alpha 1 chain [Source:HGNC Symbol;Acc:HGNC:2217]	6
ENSG00000206077	2,71815	2,501835	0,001258	0,009159	3,853057	protein_coding	ZDHHC11B	zinc finger DHHC-type containing 11B [Source:HGNC Symbol;Acc:HGNC:32962]	5
ENSG00000172819	4,113876	-1,193645	0,001259	0,00916	-3,852792	protein_coding	RARG	retinoic acid receptor gamma [Source:HGNC Symbol;Acc:HGNC:9866]	12
ENSG00000008394	3,616831	-3,106091	0,001264	0,009195	-3,850869	protein_coding	MGST1	microsomal glutathione S-transferase 1 [Source:HGNC Symbol;Acc:HGNC:7061]	12
ENSG00000175224	6,208392	-0,528021	0,001265	0,009198	-3,850465	protein_coding	ATG13	autophagy related 13 [Source:HGNC Symbol;Acc:HGNC:29091]	11
ENSG00000125817	7,015095	-0,498637	0,001266	0,009202	-3,850077	protein_coding	CENPB	centromere protein B [Source:HGNC Symbol;Acc:HGNC:1852]	20
ENSG00000119711	4,461858	1,396011	0,001267	0,009206	3,849697	protein_coding	ALDH6A1	aldehyde dehydrogenase 6 family member A1 [Source:HGNC Symbol;Acc:HGNC:7179]	14
ENSG00000198947	5,13163	1,737305	0,001274	0,009248	3,847187	protein_coding	DMD	dystrophin [Source:HGNC Symbol;Acc:HGNC:2928]	X
ENSG00000118705	8,472209	-0,733741	0,001274	0,009248	-3,84737	protein_coding	RPN2	ribophorin II [Source:HGNC Symbol;Acc:HGNC:10382]	20
ENSG00000198160	6,227324	-0,68996	0,001276	0,009258	-3,846476	protein_coding	MIER1	MIER1 transcriptional regulator [Source:HGNC Symbol;Acc:HGNC:29657]	1
ENSG00000100982	5,16031	0,64031	0,001279	0,009272	3,845563	protein_coding	PCIF1	phosphorylated CTD interacting factor 1 [Source:HGNC Symbol;Acc:HGNC:16200]	20
ENSG00000106636	6,669236	-0,791988	0,001283	0,009302	-3,843907	protein_coding	YKT6	YKT6 v-SNARE homolog [Source:HGNC Symbol;Acc:HGNC:16959]	7
ENSG00000186812	5,477274	0,935309	0,001286	0,009313	3,843127	protein_coding	ZNF397	zinc finger protein 397 [Source:HGNC Symbol;Acc:HGNC:18818]	18

ENSG00000119950	4,912781	0,895282	0,001287	0,009319	3,842655	protein_coding	MXI1	MAX interactor 1, dimerization protein [Source:HGNC Symbol;Acc:HGNC:7534]	10
ENSG00000114107	4,512163	1,169125	0,001289	0,00933	3,841911	protein_coding	CEP70	centrosomal protein 70 [Source:HGNC Symbol;Acc:HGNC:29972]	3
ENSG00000163600	-2,679418	2,664634	0,001292	0,00935	3,840671	protein_coding	ICOS	inducible T cell costimulator [Source:HGNC Symbol;Acc:HGNC:5351]	2
ENSG00000167981	2,626875	-1,607143	0,001293	0,00935	-3,840408	protein_coding	ZNF597	zinc finger protein 597 [Source:HGNC Symbol;Acc:HGNC:26573]	16
ENSG00000181929	5,005355	-0,604629	0,001294	0,00935	-3,840121	protein_coding	PRKAG1	protein kinase AMP-activated non-catalytic subunit gamma 1 [Source:HGNC Symbol;Acc:HGNC:9385]	12
ENSG00000113593	5,279743	0,662302	0,001293	0,00935	3,840302	protein_coding	PPWD1	peptidylprolyl isomerase domain and WD repeat containing 1 [Source:HGNC Symbol;Acc:HGNC:28954]	5
ENSG00000061794	5,462088	-0,682042	0,0013	0,009386	-3,838117	protein_coding	MRPS35	mitochondrial ribosomal protein S35 [Source:HGNC Symbol;Acc:HGNC:16635]	12
ENSG00000109861	6,306287	-1,223996	0,001307	0,009436	-3,835483	protein_coding	CTSC	cathepsin C [Source:HGNC Symbol;Acc:HGNC:2528]	11
ENSG00000117118	5,570544	-0,775943	0,001309	0,009444	-3,834918	protein_coding	SDHB	succinate dehydrogenase complex iron sulfur subunit B [Source:HGNC Symbol;Acc:HGNC:10681]	1
ENSG00000260729	-1,347466	1,876821	0,001309	0,009444	3,83471	protein_coding	AC009690.	novel protein	15
ENSG00000115607	-2,113498	3,182741	0,001314	0,00947	3,833128	protein_coding	IL18RAP	interleukin 18 receptor accessory protein [Source:HGNC Symbol;Acc:HGNC:5989]	2
ENSG00000090520	6,250592	-0,884102	0,001314	0,00947	-3,832933	protein_coding	DNAJB11	DnaJ heat shock protein family (Hsp40) member B11 [Source:HGNC Symbol;Acc:HGNC:14889]	3
ENSG00000146648	6,746377	-1,734021	0,001315	0,00947	-3,832802	protein_coding	EGFR	epidermal growth factor receptor [Source:HGNC Symbol;Acc:HGNC:3236]	7
ENSG00000010319	0,772558	3,345537	0,001316	0,009478	3,832214	protein_coding	SEMA3G	semaphorin 3G [Source:HGNC Symbol;Acc:HGNC:30400]	3
ENSG00000087303	6,369698	-2,028163	0,001324	0,009529	-3,829561	protein_coding	NID2	nidogen 2 [Source:HGNC Symbol;Acc:HGNC:13389]	14
ENSG00000151208	7,261442	-0,678441	0,001325	0,009535	-3,829093	protein_coding	DLG5	discs large MAGUK scaffold protein 5 [Source:HGNC Symbol;Acc:HGNC:2904]	10
ENSG00000182183	-1,743433	3,005598	0,001329	0,009537	3,827953	protein_coding	SHISAL2A	shisa like 2A [Source:HGNC Symbol;Acc:HGNC:28757]	1
ENSG00000196872	3,25524	3,125925	0,001327	0,009537	3,828484	protein_coding	CRACDL	CRACD like [Source:HGNC Symbol;Acc:HGNC:33454]	2
ENSG00000187764	4,643286	1,683844	0,001328	0,009537	3,828287	protein_coding	SEMA4D	semaphorin 4D [Source:HGNC Symbol;Acc:HGNC:10732]	9
ENSG00000170632	4,563186	-0,853786	0,001328	0,009537	-3,828137	protein_coding	ARMC10	armadillo repeat containing 10 [Source:HGNC Symbol;Acc:HGNC:21706]	7
ENSG00000155090	6,107531	-1,064418	0,001327	0,009537	-3,828395	protein_coding	KLF10	Kruppel like factor 10 [Source:HGNC Symbol;Acc:HGNC:11810]	8
ENSG00000205710	1,829407	2,10058	0,001331	0,009549	3,827205	protein_coding	C17orf107	chromosome 17 open reading frame 107 [Source:HGNC Symbol;Acc:HGNC:37238]	17
ENSG00000152104	6,023416	-1,75553	0,001333	0,009561	-3,826389	protein_coding	PTPN14	protein tyrosine phosphatase non-receptor type 14 [Source:HGNC Symbol;Acc:HGNC:9647]	1
ENSG00000165417	6,477468	-0,554754	0,001339	0,009597	-3,824493	protein_coding	GTF2A1	general transcription factor IIA subunit 1 [Source:HGNC Symbol;Acc:HGNC:4646]	14
ENSG00000162889	6,990455	-0,928416	0,001344	0,009632	-3,822609	protein_coding	MAPKAPK2	MAPK activated protein kinase 2 [Source:HGNC Symbol;Acc:HGNC:6887]	1
ENSG00000100802	3,116138	1,139119	0,001346	0,009637	3,821969	protein_coding	C14orf93	chromosome 14 open reading frame 93 [Source:HGNC Symbol;Acc:HGNC:20162]	14
ENSG00000116209	7,490649	-0,961794	0,001346	0,009637	-3,822017	protein_coding	TMEM59	transmembrane protein 59 [Source:HGNC Symbol;Acc:HGNC:1239]	1
ENSG00000147421	5,426381	0,747997	0,001347	0,009639	3,821683	protein_coding	HMBOX1	homeobox containing 1 [Source:HGNC Symbol;Acc:HGNC:26137]	8
ENSG00000091136	8,903919	-0,762343	0,001349	0,009651	-3,820908	protein_coding	LAMB1	laminin subunit beta 1 [Source:HGNC Symbol;Acc:HGNC:6486]	7
ENSG00000158428	-2,445805	2,624981	0,001351	0,009653	3,820227	protein_coding	CATIP	ciliogenesis associated TTC17 interacting protein [Source:HGNC Symbol;Acc:HGNC:25062]	2
ENSG00000135069	5,139767	-1,342947	0,001351	0,009653	-3,820478	protein_coding	PSAT1	phosphoserine aminotransferase 1 [Source:HGNC Symbol;Acc:HGNC:19129]	9
ENSG00000132432	5,632945	-0,864437	0,001351	0,009653	-3,820211	protein_coding	SEC61G	SEC61 translocon subunit gamma [Source:HGNC Symbol;Acc:HGNC:18277]	7
ENSG00000137462	1,930809	2,563698	0,001353	0,009653	3,819802	protein_coding	TLR2	toll like receptor 2 [Source:HGNC Symbol;Acc:HGNC:11848]	4
ENSG00000115641	5,173323	-2,12937	0,001353	0,009653	-3,819776	protein_coding	FHL2	four and a half LIM domains 2 [Source:HGNC Symbol;Acc:HGNC:3703]	2
ENSG00000284713	-2,11633	2,817003	0,001355	0,00966	3,818997	protein_coding	SMIM38	small integral membrane protein 38 [Source:HGNC Symbol;Acc:HGNC:54074]	11
ENSG00000107104	6,210434	0,864736	0,001355	0,00966	3,819026	protein_coding	KANK1	KN motif and ankyrin repeat domains 1 [Source:HGNC Symbol;Acc:HGNC:19309]	9
ENSG00000119707	7,793023	0,766094	0,001355	0,00966	3,818836	protein_coding	RBM25	RNA binding motif protein 25 [Source:HGNC Symbol;Acc:HGNC:23244]	14
ENSG00000085644	3,947261	-0,710721	0,001359	0,009682	-3,817543	protein_coding	ZNF213	zinc finger protein 213 [Source:HGNC Symbol;Acc:HGNC:13005]	16
ENSG00000154358	4,755005	2,359155	0,00136	0,009682	3,817276	protein_coding	OBSCN	obscurin, cytoskeletal calmodulin and titin-interacting RhoGEF [Source:HGNC Symbol;Acc:HGNC:15719]	1
ENSG00000171055	4,843575	-0,946765	0,00136	0,009682	-3,817221	protein_coding	FEZ2	fasciculation and elongation protein zeta 2 [Source:HGNC Symbol;Acc:HGNC:3660]	2
ENSG00000185811	1,799501	3,012387	0,001362	0,009694	3,816457	protein_coding	IKZF1	IKAROS family zinc finger 1 [Source:HGNC Symbol;Acc:HGNC:13176]	7
ENSG00000127954	-1,004994	4,542281	0,001364	0,009694	3,815792	protein_coding	STEAP4	STEAP4 metalloreductase [Source:HGNC Symbol;Acc:HGNC:21923]	7
ENSG00000256574	-2,340281	2,246317	0,001364	0,009694	3,816012	protein_coding	OR13A1	olfactory receptor family 13 subfamily A member 1 [Source:HGNC Symbol;Acc:HGNC:14772]	10
ENSG00000180934	-2,65192	2,948875	0,001366	0,009694	3,815276	protein_coding	OR56A1	olfactory receptor family 56 subfamily A member 1 [Source:HGNC Symbol;Acc:HGNC:14781]	11
ENSG00000108773	5,339921	0,970622	0,001366	0,009694	3,81523	protein_coding	KAT2A	lysine acetyltransferase 2A [Source:HGNC Symbol;Acc:HGNC:4201]	17
ENSG00000104067	7,816641	-0,827861	0,001365	0,009694	-3,815693	protein_coding	TJP1	tight junction protein 1 [Source:HGNC Symbol;Acc:HGNC:11827]	15
ENSG00000107862	7,386701	-0,466362	0,001365	0,009694	-3,815608	protein_coding	GBF1	golgi brefeldin A resistant guanine nucleotide exchange factor 1 [Source:HGNC Symbol;Acc:HGNC:4181]	10
ENSG00000131368	5,212724	0,537335	0,001368	0,009704	3,814555	protein_coding	MRPS25	mitochondrial ribosomal protein S25 [Source:HGNC Symbol;Acc:HGNC:14511]	3
ENSG00000110074	4,386625	1,022684	0,001369	0,009708	3,814161	protein_coding	FOXRED1	FAD dependent oxidoreductase domain containing 1 [Source:HGNC Symbol;Acc:HGNC:26927]	11
ENSG00000128271	-0,379915	1,867649	0,001371	0,00972	3,813427	protein_coding	ADORA2A	adenosine A2a receptor [Source:HGNC Symbol;Acc:HGNC:263]	22
ENSG00000136874	5,38765	0,530487	0,001375	0,009742	3,812148	protein_coding	STX17	syntaxin 17 [Source:HGNC Symbol;Acc:HGNC:11432]	9

ENSG00000138459	4,593456	-0,622524	0,001376	0,009746	-3,811782	protein_coding	SLC35A5	solute carrier family 35 member A5 [Source:HGNC Symbol;Acc:HGNC:20792]	3
ENSG00000117643	3,822345	2,424341	0,00138	0,009769	3,810485	protein_coding	MAN1C1	mannosidase alpha class 1C member 1 [Source:HGNC Symbol;Acc:HGNC:19080]	1
ENSG00000174501	5,771711	1,748663	0,001384	0,00979	3,809304	protein_coding	ANKRD36C	ankyrin repeat domain 36C [Source:HGNC Symbol;Acc:HGNC:32946]	2
ENSG00000060491	6,021537	0,681023	0,001385	0,009795	3,808862	protein_coding	OGFR	opioid growth factor receptor [Source:HGNC Symbol;Acc:HGNC:15768]	20
ENSG00000136040	5,400497	2,12202	0,001388	0,009807	3,807911	protein_coding	PLXNC1	plexin C1 [Source:HGNC Symbol;Acc:HGNC:9106]	12
ENSG00000138385	6,267046	-0,77956	0,001388	0,009807	-3,80793	protein_coding	SSB	small RNA binding exonuclease protection factor La [Source:HGNC Symbol;Acc:HGNC:11316]	2
ENSG00000158473	-0,138643	2,874172	0,001391	0,009819	3,807096	protein_coding	CD1D	CD1d molecule [Source:HGNC Symbol;Acc:HGNC:1637]	1
ENSG00000130962	3,751757	-1,767844	0,001391	0,009819	-3,806975	protein_coding	PRRG1	proline rich and Gla domain 1 [Source:HGNC Symbol;Acc:HGNC:9469]	X
ENSG00000242419	0,98396	2,685004	0,001393	0,009826	3,806132	protein_coding	PCDHGC4	protocadherin gamma subfamily C, 4 [Source:HGNC Symbol;Acc:HGNC:8717]	5
ENSG00000183638	-0,698282	2,761486	0,001393	0,009826	3,806186	protein_coding	RP1L1	RP1 like 1 [Source:HGNC Symbol;Acc:HGNC:15946]	8
ENSG00000181789	7,956305	-0,717734	0,001394	0,009826	-3,806032	protein_coding	COPG1	COPI coat complex subunit gamma 1 [Source:HGNC Symbol;Acc:HGNC:2236]	3
ENSG00000073711	4,053475	-1,226725	0,001395	0,009829	-3,805685	protein_coding	PPP2R3A	protein phosphatase 2 regulatory subunit B''alpha [Source:HGNC Symbol;Acc:HGNC:9307]	3
ENSG00000109685	7,17876	0,841991	0,001396	0,009834	3,805272	protein_coding	NSD2	nuclear receptor binding SET domain protein 2 [Source:HGNC Symbol;Acc:HGNC:12766]	4
ENSG00000172037	7,720812	-1,568915	0,001398	0,009844	-3,804603	protein_coding	LAMB2	laminin subunit beta 2 [Source:HGNC Symbol;Acc:HGNC:6487]	3
ENSG00000124380	5,13719	-0,520639	0,001399	0,009849	-3,804169	protein_coding	SNRNP27	small nuclear ribonucleoprotein U4/U6.U5 subunit 27 [Source:HGNC Symbol;Acc:HGNC:30240]	2
ENSG00000154930	3,894963	1,876023	0,001405	0,00988	3,802501	protein_coding	ACSS1	acyl-CoA synthetase short chain family member 1 [Source:HGNC Symbol;Acc:HGNC:16091]	20
ENSG00000196154	3,037132	-1,885305	0,001406	0,009886	-3,802067	protein_coding	S100A4	S100 calcium binding protein A4 [Source:HGNC Symbol;Acc:HGNC:10494]	1
ENSG00000014216	6,858471	-0,540158	0,00141	0,009908	-3,80083	protein_coding	CAPN1	calpain 1 [Source:HGNC Symbol;Acc:HGNC:1476]	11
ENSG00000109684	-1,386096	3,333639	0,001411	0,009912	3,800454	protein_coding	CLNK	cytokine dependent hematopoietic cell linker [Source:HGNC Symbol;Acc:HGNC:17438]	4
ENSG00000162746	-0,15525	-1,871345	0,001414	0,00993	-3,799405	protein_coding	FCRLB	Fc receptor like B [Source:HGNC Symbol;Acc:HGNC:26431]	1
ENSG00000165810	3,323217	3,166497	0,001417	0,009946	3,798473	protein_coding	BTNL9	butyrophilin like 9 [Source:HGNC Symbol;Acc:HGNC:24176]	5
ENSG00000116157	2,838779	-2,137684	0,001419	0,009958	-3,797703	protein_coding	GPX7	glutathione peroxidase 7 [Source:HGNC Symbol;Acc:HGNC:4559]	1
ENSG00000101282	-2,197715	2,793077	0,001422	0,009971	3,796884	protein_coding	RSPO4	R-spondin 4 [Source:HGNC Symbol;Acc:HGNC:16175]	20
ENSG00000108671	6,739087	-0,634715	0,001422	0,009971	-3,796739	protein_coding	PSMD11	proteasome 26S subunit, non-ATPase 11 [Source:HGNC Symbol;Acc:HGNC:9556]	17
ENSG00000160323	2,446877	1,738256	0,001423	0,009972	3,796503	protein_coding	ADAMTS13	ADAM metallopeptidase with thrombospondin type 1 motif 13 [Source:HGNC Symbol;Acc:HGNC:1366]	9
ENSG00000216490	-0,355761	2,059171	0,001427	0,009995	3,795145	protein_coding	IFI30	IFI30 lysosomal thiol reductase [Source:HGNC Symbol;Acc:HGNC:5398]	19
ENSG00000176986	6,97171	-0,477882	0,001428	0,009995	-3,79505	protein_coding	SEC24C	SEC24 homolog C, COPII coat complex component [Source:HGNC Symbol;Acc:HGNC:10705]	10
ENSG00000129951	-1,406578	2,716258	0,001429	0,009998	3,794685	protein_coding	PLPPR3	phospholipid phosphatase related 3 [Source:HGNC Symbol;Acc:HGNC:23497]	19
ENSG00000012504	-0,913921	3,113926	0,00143	0,010006	3,794134	protein_coding	NR1H4	nuclear receptor subfamily 1 group H member 4 [Source:HGNC Symbol;Acc:HGNC:7967]	12
ENSG00000086189	4,222773	-0,703108	0,001431	0,010006	-3,793953	protein_coding	DIMT1	DIMT1 rRNA methyltransferase and ribosome maturation factor [Source:HGNC Symbol;Acc:HGNC:30217]	5
ENSG00000285943	-0,404887	1,536816	0,001433	0,010008	3,793248	protein_coding	AC112128.	novel protein	3
ENSG00000138722	-0,177936	5,333636	0,001433	0,010008	3,793383	protein_coding	MMRN1	multimerin 1 [Source:HGNC Symbol;Acc:HGNC:7178]	4
ENSG00000087903	4,173683	1,242067	0,001433	0,010008	3,793458	protein_coding	RFX2	regulatory factor X2 [Source:HGNC Symbol;Acc:HGNC:9983]	19
ENSG00000105325	5,846205	0,763639	0,001436	0,010023	3,792374	protein_coding	FZR1	fizzy and cell division cycle 20 related 1 [Source:HGNC Symbol;Acc:HGNC:24824]	19
ENSG00000176871	6,871846	-1,016346	0,001437	0,010027	-3,791984	protein_coding	WSB2	WD repeat and SOCS box containing 2 [Source:HGNC Symbol;Acc:HGNC:19222]	12
ENSG00000046651	5,496547	0,786088	0,001438	0,010032	3,791565	protein_coding	OFD1	OFD1 centriole and centriolar satellite protein [Source:HGNC Symbol;Acc:HGNC:2567]	X
ENSG00000049246	5,525127	1,000388	0,001442	0,010047	3,790498	protein_coding	PER3	period circadian regulator 3 [Source:HGNC Symbol;Acc:HGNC:8847]	1
ENSG00000141556	6,633169	0,779102	0,001442	0,010047	3,790468	protein_coding	TBCD	tubulin folding cofactor D [Source:HGNC Symbol;Acc:HGNC:11581]	17
ENSG00000213983	6,193348	1,118254	0,001448	0,010083	3,78863	protein_coding	AP1G2	adaptor related protein complex 1 subunit gamma 2 [Source:HGNC Symbol;Acc:HGNC:556]	14
ENSG00000215717	4,655232	-0,713045	0,001451	0,010099	-3,787712	protein_coding	TMEM167B	transmembrane protein 167B [Source:HGNC Symbol;Acc:HGNC:30187]	1
ENSG00000164849	0,669569	1,612732	0,001455	0,010107	3,786398	protein_coding	GPR146	G protein-coupled receptor 146 [Source:HGNC Symbol;Acc:HGNC:21718]	7
ENSG00000163666	-0,645535	1,601455	0,001454	0,010107	3,786494	protein_coding	HESX1	HESX homeobox 1 [Source:HGNC Symbol;Acc:HGNC:4877]	3
ENSG00000064205	-1,673367	4,38235	0,001455	0,010107	3,786367	protein_coding	CCN5	cellular communication network factor 5 [Source:HGNC Symbol;Acc:HGNC:12770]	20
ENSG00000165156	5,910419	-0,592537	0,001453	0,010107	-3,786948	protein_coding	ZHX1	zinc fingers and homeoboxes 1 [Source:HGNC Symbol;Acc:HGNC:12871]	8
ENSG00000168298	7,897926	0,853174	0,001454	0,010107	3,786639	protein_coding	H1-4	H1.4 linker histone, cluster member [Source:HGNC Symbol;Acc:HGNC:4718]	6
ENSG00000123728	4,779994	-0,694768	0,001459	0,010133	-3,784981	protein_coding	RAP2C	RAP2C, member of RAS oncogene family [Source:HGNC Symbol;Acc:HGNC:21165]	X
ENSG00000153976	2,554127	-2,564141	0,00146	0,010136	-3,784686	protein_coding	HS3ST3A1	heparan sulfate-glucosamine 3-sulfotransferase 3A1 [Source:HGNC Symbol;Acc:HGNC:5196]	17
ENSG00000167094	-1,909763	2,65847	0,001468	0,010175	3,782311	protein_coding	TTC16	tetratricopeptide repeat domain 16 [Source:HGNC Symbol;Acc:HGNC:26536]	9
ENSG00000124216	2,882174	-1,365203	0,001468	0,010175	-3,782362	protein_coding	SNAI1	snail family transcriptional repressor 1 [Source:HGNC Symbol;Acc:HGNC:11128]	20
ENSG00000106799	6,471283	-0,649198	0,001467	0,010175	-3,782477	protein_coding	TGFBR1	transforming growth factor beta receptor 1 [Source:HGNC Symbol;Acc:HGNC:11772]	9
ENSG00000243317	4,97226	-0,792188	0,001469	0,010176	-3,782067	protein_coding	STMP1	short transmembrane mitochondrial protein 1 [Source:HGNC Symbol;Acc:HGNC:41909]	7

ENSG00000161692	3,708648	1,32662	0,001471	0,010188	3,781325	protein_coding	DBF4B	DBF4 zinc finger B [Source:HGNC Symbol;Acc:HGNC:17883]	17
ENSG00000197046	1,875942	-3,025995	0,001473	0,010197	-3,780756	protein_coding	SIGLEC15	sialic acid binding Ig like lectin 15 [Source:HGNC Symbol;Acc:HGNC:27596]	18
ENSG00000111981	0,884177	-2,515534	0,001475	0,010205	-3,780202	protein_coding	ULBP1	UL16 binding protein 1 [Source:HGNC Symbol;Acc:HGNC:14893]	6
ENSG00000185219	6,16979	0,571148	0,001475	0,010207	3,779914	protein_coding	ZNF445	zinc finger protein 445 [Source:HGNC Symbol;Acc:HGNC:21018]	3
ENSG00000171044	2,701826	1,233685	0,001476	0,01021	3,779588	protein_coding	XKR6	XK related 6 [Source:HGNC Symbol;Acc:HGNC:27806]	8
ENSG00000124491	1,881606	6,85213	0,001484	0,010249	3,777374	protein_coding	F13A1	coagulation factor XIII A chain [Source:HGNC Symbol;Acc:HGNC:3531]	6
ENSG00000187730	-1,015672	2,780092	0,001484	0,010249	3,777351	protein_coding	GABRD	gamma-aminobutyric acid type A receptor subunit delta [Source:HGNC Symbol;Acc:HGNC:4084]	1
ENSG00000142173	9,577859	-1,671836	0,001484	0,010249	-3,777259	protein_coding	COL6A2	collagen type VI alpha 2 chain [Source:HGNC Symbol;Acc:HGNC:2212]	21
ENSG00000100218	-1,12442	2,530761	0,001486	0,01025	3,776678	protein_coding	RSPH14	radial spoke head 14 homolog [Source:HGNC Symbol;Acc:HGNC:13437]	22
ENSG00000115350	3,090732	-1,253031	0,001485	0,01025	-3,776817	protein_coding	POLE4	DNA polymerase epsilon 4, accessory subunit [Source:HGNC Symbol;Acc:HGNC:18755]	2
ENSG00000103005	4,735065	-0,721106	0,001486	0,01025	-3,776599	protein_coding	USB1	U6 snRNA biogenesis phosphodiesterase 1 [Source:HGNC Symbol;Acc:HGNC:25792]	16
ENSG00000186517	2,744538	2,96131	0,001489	0,010266	3,775718	protein_coding	ARHGAP30	Rho GTPase activating protein 30 [Source:HGNC Symbol;Acc:HGNC:27414]	1
ENSG00000173641	1,746303	-2,735462	0,001491	0,010271	-3,775104	protein_coding	HSPB7	heat shock protein family B (small) member 7 [Source:HGNC Symbol;Acc:HGNC:5249]	1
ENSG00000151914	9,737527	-0,999765	0,001491	0,010271	-3,775155	protein_coding	DST	dystonin [Source:HGNC Symbol;Acc:HGNC:1090]	6
ENSG00000166523	-1,892231	3,111246	0,001493	0,010281	3,774464	protein_coding	CLEC4E	C-type lectin domain family 4 member E [Source:HGNC Symbol;Acc:HGNC:14555]	12
ENSG00000107890	5,23402	0,699095	0,001497	0,010304	3,773226	protein_coding	ANKRD26	ankyrin repeat domain 26 [Source:HGNC Symbol;Acc:HGNC:29186]	10
ENSG00000176105	6,384316	-0,803272	0,001504	0,010344	-3,771245	protein_coding	YES1	YES proto-oncogene 1, Src family tyrosine kinase [Source:HGNC Symbol;Acc:HGNC:12841]	18
ENSG00000158109	5,155496	-0,789584	0,001507	0,01036	-3,77035	protein_coding	TPRG1L	tumor protein p63 regulated 1 like [Source:HGNC Symbol;Acc:HGNC:27007]	1
ENSG00000135624	7,232184	-0,544139	0,001512	0,010391	-3,768769	protein_coding	CCT7	chaperonin containing TCP1 subunit 7 [Source:HGNC Symbol;Acc:HGNC:1622]	2
ENSG00000090674	4,744355	-0,668837	0,001516	0,010415	-3,76755	protein_coding	MCOLN1	mucolipin 1 [Source:HGNC Symbol;Acc:HGNC:13356]	19
ENSG00000162139	4,434345	0,863392	0,001517	0,010416	3,76728	protein_coding	NEU3	neuraminidase 3 [Source:HGNC Symbol;Acc:HGNC:7760]	11
ENSG00000166965	4,1376	1,291882	0,001518	0,010419	3,766961	protein_coding	RCCD1	RCC1 domain containing 1 [Source:HGNC Symbol;Acc:HGNC:30457]	15
ENSG00000138615	-0,616307	4,481551	0,001519	0,01042	3,76663	protein_coding	CILP	cartilage intermediate layer protein [Source:HGNC Symbol;Acc:HGNC:1980]	15
ENSG00000150672	2,494116	2,098221	0,001521	0,01042	3,765951	protein_coding	DLG2	discs large MAGUK scaffold protein 2 [Source:HGNC Symbol;Acc:HGNC:2901]	11
ENSG00000107099	4,616965	2,272323	0,00152	0,01042	3,766144	protein_coding	DOCK8	dedicator of cytokinesis 8 [Source:HGNC Symbol;Acc:HGNC:19191]	9
ENSG00000176641	5,778329	-1,296825	0,00152	0,01042	-3,766384	protein_coding	RNF152	ring finger protein 152 [Source:HGNC Symbol;Acc:HGNC:26811]	18
ENSG00000153936	5,690381	-0,787041	0,00152	0,01042	-3,766132	protein_coding	HS2ST1	heparan sulfate 2-O-sulfotransferase 1 [Source:HGNC Symbol;Acc:HGNC:5193]	1
ENSG00000164405	6,399826	-0,692436	0,001523	0,010429	-3,765386	protein_coding	UQCRQ	ubiquinol-cytochrome c reductase complex III subunit VII [Source:HGNC Symbol;Acc:HGNC:29594]	5
ENSG00000130309	6,936017	-0,751322	0,001527	0,010448	-3,764224	protein_coding	COLGALT1	collagen beta(1-O)galactosyltransferase 1 [Source:HGNC Symbol;Acc:HGNC:26182]	19
ENSG00000101367	7,294891	-0,464094	0,001527	0,010448	-3,764137	protein_coding	MAPRE1	microtubule associated protein RP/EB family member 1 [Source:HGNC Symbol;Acc:HGNC:6890]	20
ENSG00000277161	3,545974	-0,980875	0,001529	0,010457	-3,763554	protein_coding	PIGW	phosphatidylinositol glycan anchor biosynthesis class W [Source:HGNC Symbol;Acc:HGNC:23213]	17
ENSG00000197245	-2,250496	2,887801	0,001531	0,010464	3,763079	protein_coding	FAM110D	family with sequence similarity 110 member D [Source:HGNC Symbol;Acc:HGNC:25860]	1
ENSG00000196284	2,772216	1,038327	0,001534	0,010473	3,762095	protein_coding	SUPT3H	SPT3 homolog, SAGA and STAGA complex component [Source:HGNC Symbol;Acc:HGNC:11466]	6
ENSG00000184924	3,185405	-0,981857	0,001533	0,010473	-3,762249	protein_coding	PTRHD1	peptidyl-tRNA hydrolase domain containing 1 [Source:HGNC Symbol;Acc:HGNC:33782]	2
ENSG00000108100	6,287914	-0,611634	0,001534	0,010473	-3,76207	protein_coding	CCNY	cyclin Y [Source:HGNC Symbol;Acc:HGNC:23354]	10
ENSG00000196419	7,793515	-0,663935	0,001537	0,010491	-3,761101	protein_coding	XRCC6	X-ray repair cross complementing 6 [Source:HGNC Symbol;Acc:HGNC:4055]	22
ENSG00000163154	-0,921661	2,597814	0,001539	0,010499	3,760548	protein_coding	TNFAIP8L2	TNF alpha induced protein 8 like 2 [Source:HGNC Symbol;Acc:HGNC:26277]	1
ENSG00000168237	3,638468	1,067641	0,001544	0,010526	3,759177	protein_coding	GLYCTK	glycerate kinase [Source:HGNC Symbol;Acc:HGNC:24247]	3
ENSG00000173227	1,95649	-2,694445	0,001547	0,010543	-3,758274	protein_coding	SYT12	synaptotagmin 12 [Source:HGNC Symbol;Acc:HGNC:18381]	11
ENSG00000176018	5,145398	-0,628325	0,001548	0,010545	-3,757975	protein_coding	LYSMD3	LysM domain containing 3 [Source:HGNC Symbol;Acc:HGNC:26969]	5
ENSG00000008277	4,294028	1,823781	0,00155	0,010557	3,757251	protein_coding	ADAM22	ADAM metallopeptidase domain 22 [Source:HGNC Symbol;Acc:HGNC:201]	7
ENSG00000131148	4,249022	-0,498275	0,001556	0,010591	-3,755597	protein_coding	EMC8	ER membrane protein complex subunit 8 [Source:HGNC Symbol;Acc:HGNC:7864]	16
ENSG00000120341	-1,661429	1,835795	0,001564	0,010631	3,753281	protein_coding	SEC16B	SEC16 homolog B, endoplasmic reticulum export factor [Source:HGNC Symbol;Acc:HGNC:30301]	1
ENSG00000184983	5,602326	-0,714053	0,001563	0,010631	-3,753434	protein_coding	NDUFA6	NADH:ubiquinone oxidoreductase subunit A6 [Source:HGNC Symbol;Acc:HGNC:7690]	22
ENSG00000132646	6,225276	-0,993989	0,001563	0,010631	-3,753422	protein_coding	PCNA	proliferating cell nuclear antigen [Source:HGNC Symbol;Acc:HGNC:8729]	20
ENSG00000168763	5,344235	0,817198	0,001565	0,010633	3,753009	protein_coding	CNNM3	cyclin and CBS domain divalent metal cation transport mediator 3 [Source:HGNC Symbol;Acc:HGNC:104]	2
ENSG00000155275	3,748085	0,910259	0,001567	0,010644	3,752335	protein_coding	TRMT44	tRNA methyltransferase 44 homolog [Source:HGNC Symbol;Acc:HGNC:26653]	4
ENSG00000009844	5,260155	-0,749521	0,001569	0,010647	-3,75184	protein_coding	VTA1	vesicle trafficking 1 [Source:HGNC Symbol;Acc:HGNC:20954]	6
ENSG00000137076	9,446632	-0,723396	0,001568	0,010647	-3,751879	protein_coding	TLN1	talin 1 [Source:HGNC Symbol;Acc:HGNC:11845]	9
ENSG00000128591	6,079491	-1,593801	0,001575	0,010687	-3,749926	protein_coding	FLNC	filamin C [Source:HGNC Symbol;Acc:HGNC:3756]	7
ENSG00000108556	1,093274	1,966587	0,001577	0,010692	3,749341	protein_coding	CHRNE	cholinergic receptor nicotinic epsilon subunit [Source:HGNC Symbol;Acc:HGNC:1966]	17

ENSG00000175711	2,062868	1,508909	0,001577	0,010692	3,749356	protein_coding	B3GNTL1	UDP-GlcNAc:betaGal beta-1,3-N-acetylglucosaminyltransferase like 1 [Source:HGNC Symbol;Acc:HGNC:21727]	17
ENSG00000111670	6,24657	-0,777607	0,00158	0,010704	-3,748638	protein_coding	GNPTAB	N-acetylglucosamine-1-phosphate transferase subunits alpha and beta [Source:HGNC Symbol;Acc:HGNC:29670]	12
ENSG00000163354	-1,331767	2,190142	0,001583	0,01072	3,747571	protein_coding	DCST2	DC-STAMP domain containing 2 [Source:HGNC Symbol;Acc:HGNC:26562]	1
ENSG00000124588	4,170197	-1,11319	0,001583	0,01072	-3,747605	protein_coding	NQO2	N-ribosyldihydronicotinamide:quinone reductase 2 [Source:HGNC Symbol;Acc:HGNC:7856]	6
ENSG00000111057	7,619659	-0,989416	0,001587	0,010742	-3,746441	protein_coding	KRT18	keratin 18 [Source:HGNC Symbol;Acc:HGNC:6430]	12
ENSG00000128050	6,116277	-0,753681	0,001594	0,010781	-3,74457	protein_coding	PAICS	phosphoribosylaminoimidazole carboxylase and phosphoribosylaminoimidazolesuccinocarboxamide synthase [Sour	4
ENSG00000182916	1,27537	-2,111078	0,001595	0,010788	-3,744078	protein_coding	TCEAL7	transcription elongation factor A like 7 [Source:HGNC Symbol;Acc:HGNC:28336]	X
ENSG00000187713	3,937235	-0,699195	0,001597	0,01079	-3,743626	protein_coding	TMEM203	transmembrane protein 203 [Source:HGNC Symbol;Acc:HGNC:28217]	9
ENSG00000223496	5,668227	-0,681938	0,001596	0,01079	-3,743798	protein_coding	EXOSC6	exosome component 6 [Source:HGNC Symbol;Acc:HGNC:19055]	16
ENSG00000198841	1,484923	-1,090286	0,0016	0,010802	-3,742588	protein_coding	KTI12	KTI12 chromatin associated homolog [Source:HGNC Symbol;Acc:HGNC:25160]	1
ENSG00000196220	3,840183	1,867089	0,0016	0,010802	3,742686	protein_coding	SRGAP3	SLIT-ROBO Rho GTPase activating protein 3 [Source:HGNC Symbol;Acc:HGNC:19744]	3
ENSG00000196814	4,832229	1,365995	0,001601	0,010802	3,742549	protein_coding	MVB12B	multivesicular body subunit 12B [Source:HGNC Symbol;Acc:HGNC:23368]	9
ENSG00000176909	0,54668	2,224497	0,001603	0,010816	3,741759	protein_coding	MAMSTR	MEF2 activating motif and SAP domain containing transcriptional regulator [Source:HGNC Symbol;Acc:HGNC:26689]	19
ENSG00000138758	7,565333	-0,797495	0,001607	0,010834	-3,740791	protein_coding	SEPTIN11	septin 11 [Source:HGNC Symbol;Acc:HGNC:25589]	4
ENSG00000256525	3,769861	1,061484	0,001613	0,010871	3,739049	protein_coding	POLG2	DNA polymerase gamma 2, accessory subunit [Source:HGNC Symbol;Acc:HGNC:9180]	17
ENSG00000187642	-2,140863	2,266295	0,001616	0,010891	3,738023	protein_coding	PERM1	PPARGC1 and ESRR induced regulator, muscle 1 [Source:HGNC Symbol;Acc:HGNC:28208]	1
ENSG00000143226	3,293313	3,086826	0,001619	0,0109	3,737424	protein_coding	FCGR2A	Fc fragment of IgG receptor IIa [Source:HGNC Symbol;Acc:HGNC:3616]	1
ENSG00000135521	4,461349	-0,711892	0,001622	0,010917	-3,736528	protein_coding	LTV1	LTV1 ribosome biogenesis factor [Source:HGNC Symbol;Acc:HGNC:21173]	6
ENSG00000109511	0,085283	-4,176836	0,001626	0,010939	-3,735208	protein_coding	ANXA10	annexin A10 [Source:HGNC Symbol;Acc:HGNC:534]	4
ENSG00000109118	6,208772	0,55569	0,001626	0,010939	3,735208	protein_coding	PHF12	PHD finger protein 12 [Source:HGNC Symbol;Acc:HGNC:20816]	17
ENSG00000002822	1,043613	2,012892	0,001629	0,010948	3,734348	protein_coding	MAD1L1	mitotic arrest deficient 1 like 1 [Source:HGNC Symbol;Acc:HGNC:6762]	7
ENSG00000149557	3,785298	-1,794184	0,001629	0,010948	-3,734545	protein_coding	FEZ1	fasciculation and elongation protein zeta 1 [Source:HGNC Symbol;Acc:HGNC:3659]	11
ENSG00000038427	9,421592	-2,056818	0,00163	0,010948	-3,734263	protein_coding	VCAN	versican [Source:HGNC Symbol;Acc:HGNC:2464]	5
ENSG00000170209	-1,574705	2,272266	0,001631	0,010949	3,733873	protein_coding	ANKK1	ankyrin repeat and kinase domain containing 1 [Source:HGNC Symbol;Acc:HGNC:21027]	11
ENSG00000089351	6,551808	0,751073	0,001631	0,010949	3,733949	protein_coding	GRAMD1A	GRAM domain containing 1A [Source:HGNC Symbol;Acc:HGNC:29305]	19
ENSG00000196787	5,766382	1,20171	0,001632	0,010949	3,733663	protein_coding	H2AC11	H2A clustered histone 11 [Source:HGNC Symbol;Acc:HGNC:4737]	6
ENSG00000197223	3,363817	-0,717069	0,001636	0,010973	-3,732484	protein_coding	C1D	C1D nuclear receptor corepressor [Source:HGNC Symbol;Acc:HGNC:29911]	2
ENSG00000105655	4,919804	1,39922	0,001637	0,010975	3,732205	protein_coding	ISYNA1	inositol-3-phosphate synthase 1 [Source:HGNC Symbol;Acc:HGNC:29821]	19
ENSG00000101166	5,648221	-0,774562	0,001638	0,010975	-3,732021	protein_coding	PRELID3B	PRELI domain containing 3B [Source:HGNC Symbol;Acc:HGNC:15892]	20
ENSG00000105552	4,298318	1,267142	0,001639	0,01098	3,731605	protein_coding	BCAT2	branched chain amino acid transaminase 2 [Source:HGNC Symbol;Acc:HGNC:977]	19
ENSG00000101004	5,326465	1,283226	0,001644	0,011008	3,730243	protein_coding	NINL	ninein like [Source:HGNC Symbol;Acc:HGNC:29163]	20
ENSG00000132581	4,745229	-0,700294	0,001645	0,01101	-3,729966	protein_coding	SDF2	stromal cell derived factor 2 [Source:HGNC Symbol;Acc:HGNC:10675]	17
ENSG00000105697	-1,282749	2,004058	0,001647	0,011014	3,729438	protein_coding	HAMP	hepcidin antimicrobial peptide [Source:HGNC Symbol;Acc:HGNC:15598]	19
ENSG00000083290	5,298357	1,025754	0,001647	0,011014	3,729534	protein_coding	ULK2	unc-51 like autophagy activating kinase 2 [Source:HGNC Symbol;Acc:HGNC:13480]	17
ENSG00000198113	4,245163	-1,522905	0,001653	0,011043	-3,727859	protein_coding	TOR4A	torsin family 4 member A [Source:HGNC Symbol;Acc:HGNC:25981]	9
ENSG00000134278	6,210203	-0,981949	0,001652	0,011043	-3,728007	protein_coding	SPIRE1	spire type actin nucleation factor 1 [Source:HGNC Symbol;Acc:HGNC:30622]	18
ENSG00000065618	0,005496	-2,510318	0,001654	0,011049	-3,727404	protein_coding	COL17A1	collagen type XVII alpha 1 chain [Source:HGNC Symbol;Acc:HGNC:2194]	10
ENSG00000186074	-0,192393	2,007523	0,001658	0,011069	3,726388	protein_coding	CD300LF	CD300 molecule like family member f [Source:HGNC Symbol;Acc:HGNC:29883]	17
ENSG00000158715	2,401015	-1,36449	0,00166	0,011079	-3,72581	protein_coding	SLC45A3	solute carrier family 45 member 3 [Source:HGNC Symbol;Acc:HGNC:8642]	1
ENSG00000151474	6,117101	-1,325504	0,001661	0,011082	-3,725479	protein_coding	FRMD4A	FERM domain containing 4A [Source:HGNC Symbol;Acc:HGNC:25491]	10
ENSG00000104218	5,791569	0,816973	0,001667	0,011113	3,72401	protein_coding	CSPP1	centrosome and spindle pole associated protein 1 [Source:HGNC Symbol;Acc:HGNC:26193]	8
ENSG00000168291	5,656228	-0,660515	0,00167	0,011131	-3,723071	protein_coding	PDHB	pyruvate dehydrogenase E1 subunit beta [Source:HGNC Symbol;Acc:HGNC:8808]	3
ENSG00000162599	5,72589	1,409955	0,001672	0,011141	3,722488	protein_coding	NFIA	nuclear factor I A [Source:HGNC Symbol;Acc:HGNC:7784]	1
ENSG00000113645	5,861865	1,201785	0,001673	0,011145	3,722135	protein_coding	WWC1	WW and C2 domain containing 1 [Source:HGNC Symbol;Acc:HGNC:29435]	5
ENSG00000166477	5,864648	-0,784233	0,001685	0,011215	-3,719084	protein_coding	LEO1	LEO1 homolog, Paf1/RNA polymerase II complex component [Source:HGNC Symbol;Acc:HGNC:30401]	15
ENSG00000159917	3,278756	1,19983	0,001688	0,01122	3,718264	protein_coding	ZNF235	zinc finger protein 235 [Source:HGNC Symbol;Acc:HGNC:12866]	19
ENSG00000158545	6,192898	0,408757	0,001688	0,01122	3,718116	protein_coding	ZC3H18	zinc finger CCCH-type containing 18 [Source:HGNC Symbol;Acc:HGNC:25091]	16
ENSG00000085719	6,283318	-0,520775	0,001686	0,01122	-3,71858	protein_coding	CPNE3	copine 3 [Source:HGNC Symbol;Acc:HGNC:2316]	8
ENSG00000140391	6,919996	-0,64176	0,001687	0,01122	-3,718484	protein_coding	TSPAN3	tetraspanin 3 [Source:HGNC Symbol;Acc:HGNC:17752]	15
ENSG00000114503	5,673165	-0,604142	0,00169	0,01123	-3,717516	protein_coding	NCBP2	nuclear cap binding protein subunit 2 [Source:HGNC Symbol;Acc:HGNC:7659]	3
ENSG00000164691	0,657277	2,677168	0,001692	0,011236	3,717097	protein_coding	TAGAP	T cell activation RhoGTPase activating protein [Source:HGNC Symbol;Acc:HGNC:15669]	6

ENSG00000108691	4,954854	-2,860197	0,001693	0,011238	-3,716813	protein_coding	CCL2	C-C motif chemokine ligand 2 [Source:HGNC Symbol;Acc:HGNC:10618]	17
ENSG00000182132	-3,059691	2,853107	0,001694	0,011242	3,716489	protein_coding	KCNIP1	potassium voltage-gated channel interacting protein 1 [Source:HGNC Symbol;Acc:HGNC:15521]	5
ENSG00000134830	0,703515	1,63994	0,001697	0,011253	3,71571	protein_coding	C5AR2	complement component 5a receptor 2 [Source:HGNC Symbol;Acc:HGNC:4527]	19
ENSG00000186675	-2,829064	2,168612	0,001697	0,011253	3,715653	protein_coding	MAGEE2	MAGE family member E2 [Source:HGNC Symbol;Acc:HGNC:24935]	X
ENSG00000087589	0,717412	2,055665	0,001701	0,011275	3,714559	protein_coding	CASS4	Cas scaffold protein family member 4 [Source:HGNC Symbol;Acc:HGNC:15878]	20
ENSG00000187800	2,581961	2,052097	0,001703	0,011279	3,714044	protein_coding	PEAR1	platelet endothelial aggregation receptor 1 [Source:HGNC Symbol;Acc:HGNC:33631]	1
ENSG00000116704	4,544469	-1,173837	0,001703	0,011279	-3,7142	protein_coding	SLC35D1	solute carrier family 35 member D1 [Source:HGNC Symbol;Acc:HGNC:20800]	1
ENSG00000136104	4,888069	0,974146	0,001709	0,011311	3,712558	protein_coding	RNASEH2B	ribonuclease H2 subunit B [Source:HGNC Symbol;Acc:HGNC:25671]	13
ENSG00000106013	-1,765042	2,086482	0,001714	0,011327	3,711161	protein_coding	ANKRD7	ankyrin repeat domain 7 [Source:HGNC Symbol;Acc:HGNC:18588]	7
ENSG00000119714	1,906137	-1,971858	0,001715	0,011327	-3,710946	protein_coding	GPR68	G protein-coupled receptor 68 [Source:HGNC Symbol;Acc:HGNC:4519]	14
ENSG00000173110	2,735012	2,821901	0,001714	0,011327	3,711055	protein_coding	HSPA6	heat shock protein family A (Hsp70) member 6 [Source:HGNC Symbol;Acc:HGNC:5239]	1
ENSG00000117266	3,563143	1,533401	0,001714	0,011327	3,711132	protein_coding	CDK18	cyclin dependent kinase 18 [Source:HGNC Symbol;Acc:HGNC:8751]	1
ENSG00000176022	4,24812	-0,903391	0,001715	0,011327	-3,711006	protein_coding	B3GALT6	beta-1,3-galactosyltransferase 6 [Source:HGNC Symbol;Acc:HGNC:17978]	1
ENSG00000136048	4,198021	-1,538556	0,001716	0,011327	-3,710613	protein_coding	DRAM1	DNA damage regulated autophagy modulator 1 [Source:HGNC Symbol;Acc:HGNC:25645]	12
ENSG00000183853	6,372947	-1,555482	0,001716	0,011327	-3,710565	protein_coding	KIRREL1	kirre like nephrin family adhesion molecule 1 [Source:HGNC Symbol;Acc:HGNC:15734]	1
ENSG00000111639	5,446254	-0,539213	0,001718	0,011335	-3,710063	protein_coding	MRPL51	mitochondrial ribosomal protein L51 [Source:HGNC Symbol;Acc:HGNC:14044]	12
ENSG00000114771	-0,21598	-5,588728	0,001719	0,011336	-3,709867	protein_coding	AADAC	arylacetamide deacetylase [Source:HGNC Symbol;Acc:HGNC:17]	3
ENSG00000147162	7,63863	0,911602	0,001724	0,011368	3,708355	protein_coding	OGT	O-linked N-acetylglucosamine (GlcNAc) transferase [Source:HGNC Symbol;Acc:HGNC:8127]	X
ENSG00000144026	4,500497	1,180064	0,001734	0,01142	3,70588	protein_coding	ZNF514	zinc finger protein 514 [Source:HGNC Symbol;Acc:HGNC:25894]	2
ENSG00000204435	4,18934	-0,793455	0,001734	0,01142	-3,705801	protein_coding	CSNK2B	casein kinase 2 beta [Source:HGNC Symbol;Acc:HGNC:2460]	6
ENSG00000213339	4,531464	0,946983	0,001735	0,01142	3,705544	protein_coding	QTRT1	queuine tRNA-ribosyltransferase catalytic subunit 1 [Source:HGNC Symbol;Acc:HGNC:23797]	19
ENSG00000172766	4,705616	0,932021	0,001735	0,01142	3,705565	protein_coding	NAA16	N-alpha-acetyltransferase 16, NatA auxiliary subunit [Source:HGNC Symbol;Acc:HGNC:26164]	13
ENSG00000111358	4,076939	-0,553711	0,001739	0,011443	-3,704404	protein_coding	GTF2H3	general transcription factor IIH subunit 3 [Source:HGNC Symbol;Acc:HGNC:4657]	12
ENSG00000071967	5,96274	-2,122643	0,00174	0,011446	-3,704132	protein_coding	CYBRD1	cytochrome b reductase 1 [Source:HGNC Symbol;Acc:HGNC:20797]	2
ENSG00000137947	4,463277	-0,815713	0,001744	0,011463	-3,703246	protein_coding	GTF2B	general transcription factor IIB [Source:HGNC Symbol;Acc:HGNC:4648]	1
ENSG00000128606	3,792384	-3,046644	0,001749	0,01149	-3,701982	protein_coding	LRRC17	leucine rich repeat containing 17 [Source:HGNC Symbol;Acc:HGNC:16895]	7
ENSG00000175606	4,137467	-0,76312	0,00175	0,01149	-3,701624	protein_coding	TMEM70	transmembrane protein 70 [Source:HGNC Symbol;Acc:HGNC:26050]	8
ENSG00000162368	6,597522	-0,795129	0,00175	0,01149	-3,701641	protein_coding	CMPK1	cytidine/uridine monophosphate kinase 1 [Source:HGNC Symbol;Acc:HGNC:18170]	1
ENSG00000149483	4,099018	-0,65813	0,001753	0,011505	-3,700832	protein_coding	TMEM138	transmembrane protein 138 [Source:HGNC Symbol;Acc:HGNC:26944]	11
ENSG00000183833	-0,156198	3,565383	0,001759	0,01151	3,699302	protein_coding	CFAP91	cilia and flagella associated protein 91 [Source:HGNC Symbol;Acc:HGNC:24010]	3
ENSG00000066923	0,934103	2,024332	0,001755	0,01151	3,700333	protein_coding	STAG3	stromal antigen 3 [Source:HGNC Symbol;Acc:HGNC:11356]	7
ENSG00000164220	1,221195	-2,832358	0,001759	0,01151	-3,699244	protein_coding	F2RL2	coagulation factor II thrombin receptor like 2 [Source:HGNC Symbol;Acc:HGNC:3539]	5
ENSG00000174697	-2,911353	3,755638	0,001758	0,01151	3,699479	protein_coding	LEP	leptin [Source:HGNC Symbol;Acc:HGNC:6553]	7
ENSG00000092929	2,630222	1,795604	0,001758	0,01151	3,699402	protein_coding	UNC13D	unc-13 homolog D [Source:HGNC Symbol;Acc:HGNC:23147]	17
ENSG00000027075	3,371509	2,453015	0,00176	0,01151	3,698957	protein_coding	PRKCH	protein kinase C eta [Source:HGNC Symbol;Acc:HGNC:9403]	14
ENSG00000081721	4,047318	-0,748581	0,001759	0,01151	-3,699389	protein_coding	DUSP12	dual specificity phosphatase 12 [Source:HGNC Symbol;Acc:HGNC:3067]	1
ENSG00000166387	4,68666	1,331071	0,00176	0,01151	3,699101	protein_coding	PPFIBP2	PPFIA binding protein 2 [Source:HGNC Symbol;Acc:HGNC:9250]	11
ENSG00000104331	7,080972	-0,902919	0,001756	0,01151	-3,700119	protein_coding	BPNT2	3'(2'), 5'-bisphosphate nucleotidase 2 [Source:HGNC Symbol;Acc:HGNC:26019]	8
ENSG00000126001	7,38021	1,075244	0,001765	0,011535	3,697795	protein_coding	CEP250	centrosomal protein 250 [Source:HGNC Symbol;Acc:HGNC:1859]	20
ENSG00000165724	4,605924	-0,740413	0,001766	0,01154	-3,697409	protein_coding	ZMYND19	zinc finger MYND-type containing 19 [Source:HGNC Symbol;Acc:HGNC:21146]	9
ENSG00000064195	0,677353	-3,469217	0,001773	0,011573	-3,695543	protein_coding	DLX3	distal-less homeobox 3 [Source:HGNC Symbol;Acc:HGNC:2916]	17
ENSG00000204406	5,528573	0,643712	0,001773	0,011573	3,695635	protein_coding	MBD5	methyl-CpG binding domain protein 5 [Source:HGNC Symbol;Acc:HGNC:20444]	2
ENSG00000166398	5,889575	0,753679	0,001773	0,011573	3,695564	protein_coding	GARRE1	granule associated Rac and RHOG effector 1 [Source:HGNC Symbol;Acc:HGNC:29016]	19
ENSG00000127463	6,715678	-0,594822	0,001774	0,011573	-3,695342	protein_coding	EMC1	ER membrane protein complex subunit 1 [Source:HGNC Symbol;Acc:HGNC:28957]	1
ENSG00000165591	2,723458	1,711701	0,001778	0,01159	3,694417	protein_coding	FAAH2	fatty acid amide hydrolase 2 [Source:HGNC Symbol;Acc:HGNC:26440]	X
ENSG00000145911	3,442091	1,706578	0,001778	0,01159	3,69432	protein_coding	N4BP3	NEDD4 binding protein 3 [Source:HGNC Symbol;Acc:HGNC:29852]	5
ENSG00000159713	3,283178	1,976381	0,001785	0,011631	3,692413	protein_coding	TPPP3	tubulin polymerization promoting protein family member 3 [Source:HGNC Symbol;Acc:HGNC:24162]	16
ENSG00000030110	3,434825	-0,943781	0,001786	0,011631	-3,692316	protein_coding	BAK1	BCL2 antagonist/killer 1 [Source:HGNC Symbol;Acc:HGNC:949]	6
ENSG00000203734	-2,532192	2,365438	0,00179	0,011652	3,691333	protein_coding	ECT2L	epithelial cell transforming 2 like [Source:HGNC Symbol;Acc:HGNC:21118]	6
ENSG00000111799	8,591576	-2,503942	0,001792	0,011659	-3,690848	protein_coding	COL12A1	collagen type XII alpha 1 chain [Source:HGNC Symbol;Acc:HGNC:2188]	6
ENSG00000083807	2,210191	1,378642	0,001792	0,011659	3,69066	protein_coding	SLC27A5	solute carrier family 27 member 5 [Source:HGNC Symbol;Acc:HGNC:10999]	19

ENSG00000170855	3,888157	-0,900928	0,001802	0,011719	-3,688146	protein_coding	TRIAP1	TP53 regulated inhibitor of apoptosis 1 [Source:HGNC Symbol;Acc:HGNC:26937]	12
ENSG00000115307	6,39776	-0,705339	0,001804	0,011725	-3,68772	protein_coding	AUP1	AUP1 lipid droplet regulating VLDL assembly factor [Source:HGNC Symbol;Acc:HGNC:891]	2
ENSG00000083093	4,090319	-0,549936	0,001808	0,01174	-3,686572	protein_coding	PALB2	partner and localizer of BRCA2 [Source:HGNC Symbol;Acc:HGNC:26144]	16
ENSG00000134265	5,56154	-0,625488	0,001808	0,01174	-3,686687	protein_coding	NAPG	NSF attachment protein gamma [Source:HGNC Symbol;Acc:HGNC:7642]	18
ENSG00000119655	6,182946	-0,960007	0,001808	0,01174	-3,686667	protein_coding	NPC2	NPC intracellular cholesterol transporter 2 [Source:HGNC Symbol;Acc:HGNC:14537]	14
ENSG00000167355	-3,131024	3,03011	0,001822	0,011825	3,683081	protein_coding	OR51B5	olfactory receptor family 51 subfamily B member 5 [Source:HGNC Symbol;Acc:HGNC:19599]	11
ENSG00000170802	4,887115	-0,628456	0,001827	0,011854	-3,681777	protein_coding	FOXN2	forkhead box N2 [Source:HGNC Symbol;Acc:HGNC:5281]	2
ENSG00000181029	0,635766	1,22578	0,001829	0,011859	3,681396	protein_coding	TRAPPC5	trafficking protein particle complex 5 [Source:HGNC Symbol;Acc:HGNC:23067]	19
ENSG00000118690	2,495078	1,151382	0,00183	0,01186	3,68101	protein_coding	ARMC2	armadillo repeat containing 2 [Source:HGNC Symbol;Acc:HGNC:23045]	6
ENSG00000141759	5,858601	-0,796241	0,00183	0,01186	-3,681028	protein_coding	TXNL4A	thioredoxin like 4A [Source:HGNC Symbol;Acc:HGNC:30551]	18
ENSG00000165617	5,233737	-1,774515	0,001833	0,011872	-3,680346	protein_coding	DACT1	dishevelled binding antagonist of beta catenin 1 [Source:HGNC Symbol;Acc:HGNC:17748]	14
ENSG00000170961	3,606953	-2,390762	0,001838	0,011897	-3,679193	protein_coding	HAS2	hyaluronan synthase 2 [Source:HGNC Symbol;Acc:HGNC:4819]	8
ENSG00000100413	5,18532	-0,592042	0,001841	0,011914	-3,678346	protein_coding	POLR3H	RNA polymerase III subunit H [Source:HGNC Symbol;Acc:HGNC:30349]	22
ENSG00000160255	4,413211	2,449662	0,001842	0,011916	3,678086	protein_coding	ITGB2	integrin subunit beta 2 [Source:HGNC Symbol;Acc:HGNC:6155]	21
ENSG00000166816	-0,490348	3,15073	0,001846	0,011936	3,677143	protein_coding	LDHD	lactate dehydrogenase D [Source:HGNC Symbol;Acc:HGNC:19708]	16
ENSG00000124177	7,713921	0,795056	0,001847	0,011936	3,676959	protein_coding	CHD6	chromodomain helicase DNA binding protein 6 [Source:HGNC Symbol;Acc:HGNC:19057]	20
ENSG00000145391	6,723734	-1,211165	0,001849	0,011945	-3,676426	protein_coding	SETD7	SET domain containing 7, histone lysine methyltransferase [Source:HGNC Symbol;Acc:HGNC:30412]	4
ENSG00000085276	2,241381	4,061308	0,001863	0,012004	3,672809	protein_coding	MECOM	MDS1 and EVI1 complex locus [Source:HGNC Symbol;Acc:HGNC:3498]	3
ENSG00000132259	-2,057498	2,210366	0,001864	0,012004	3,672548	protein_coding	CNGA4	cyclic nucleotide gated channel subunit alpha 4 [Source:HGNC Symbol;Acc:HGNC:2152]	11
ENSG00000164049	-2,540307	3,412646	0,00186	0,012004	3,673748	protein_coding	FBXW12	F-box and WD repeat domain containing 12 [Source:HGNC Symbol;Acc:HGNC:20729]	3
ENSG00000137364	4,251073	-0,743933	0,001866	0,012004	-3,672173	protein_coding	TPMT	thiopurine S-methyltransferase [Source:HGNC Symbol;Acc:HGNC:12014]	6
ENSG00000135900	4,441451	-0,62845	0,001865	0,012004	-3,672333	protein_coding	MRPL44	mitochondrial ribosomal protein L44 [Source:HGNC Symbol;Acc:HGNC:16650]	2
ENSG00000174080	5,051375	0,842655	0,001865	0,012004	3,672354	protein_coding	CTSF	cathepsin F [Source:HGNC Symbol;Acc:HGNC:2531]	11
ENSG00000078177	5,376831	1,020094	0,001865	0,012004	3,672396	protein_coding	N4BP2	NEDD4 binding protein 2 [Source:HGNC Symbol;Acc:HGNC:29851]	4
ENSG00000029153	5,386769	-1,028872	0,001864	0,012004	-3,672641	protein_coding	ARNTL2	aryl hydrocarbon receptor nuclear translocator like 2 [Source:HGNC Symbol;Acc:HGNC:18984]	12
ENSG00000206560	6,34048	-0,724017	0,001861	0,012004	-3,673522	protein_coding	ANKRD28	ankyrin repeat domain 28 [Source:HGNC Symbol;Acc:HGNC:29024]	3
ENSG00000174197	7,222559	0,563116	0,00186	0,012004	3,673751	protein_coding	MGA	MAX dimerization protein MGA [Source:HGNC Symbol;Acc:HGNC:14010]	15
ENSG00000136731	7,580778	-0,595896	0,001861	0,012004	-3,673426	protein_coding	UGGT1	UDP-glucose glycoprotein glucosyltransferase 1 [Source:HGNC Symbol;Acc:HGNC:15663]	2
ENSG00000146021	3,631092	2,092429	0,00187	0,012028	3,671094	protein_coding	KLHL3	kelch like family member 3 [Source:HGNC Symbol;Acc:HGNC:6354]	5
ENSG00000163864	3,057891	2,121175	0,001872	0,012034	3,670652	protein_coding	NMNAT3	nicotinamide nucleotide adenylyltransferase 3 [Source:HGNC Symbol;Acc:HGNC:20989]	3
ENSG00000133800	-0,287323	5,006518	0,001875	0,012051	3,669857	protein_coding	LYVE1	lymphatic vessel endothelial hyaluronan receptor 1 [Source:HGNC Symbol;Acc:HGNC:14687]	11
ENSG00000188176	-2,193233	2,559486	0,001878	0,01206	3,669304	protein_coding	SMTNL2	smoothelin like 2 [Source:HGNC Symbol;Acc:HGNC:24764]	17
ENSG00000125846	4,225312	0,968466	0,001883	0,012091	3,667957	protein_coding	ZNF133	zinc finger protein 133 [Source:HGNC Symbol;Acc:HGNC:12917]	20
ENSG00000226321	-1,106646	3,759685	0,001887	0,012106	3,666939	protein_coding	CROCC2	ciliary rootlet coiled-coil, rootletin family member 2 [Source:HGNC Symbol;Acc:HGNC:51677]	2
ENSG00000079134	5,164355	0,845096	0,001888	0,012106	3,666847	protein_coding	THOC1	THO complex 1 [Source:HGNC Symbol;Acc:HGNC:19070]	18
ENSG00000128829	6,446245	-0,781289	0,001887	0,012106	-3,66698	protein_coding	EIF2AK4	eukaryotic translation initiation factor 2 alpha kinase 4 [Source:HGNC Symbol;Acc:HGNC:19687]	15
ENSG00000146005	-0,946105	1,733748	0,001889	0,012107	3,666612	protein_coding	PSD2	pleckstrin and Sec7 domain containing 2 [Source:HGNC Symbol;Acc:HGNC:19092]	5
ENSG00000142686	4,272924	-0,741792	0,001892	0,012125	-3,665747	protein_coding	C1orf216	chromosome 1 open reading frame 216 [Source:HGNC Symbol;Acc:HGNC:26800]	1
ENSG00000188229	7,896309	-0,683659	0,001893	0,012127	-3,665492	protein_coding	TUBB4B	tubulin beta 4B class IVb [Source:HGNC Symbol;Acc:HGNC:20771]	9
ENSG00000113615	6,135741	-0,783486	0,001897	0,012147	-3,664555	protein_coding	SEC24A	SEC24 homolog A, COPII coat complex component [Source:HGNC Symbol;Acc:HGNC:10703]	5
ENSG00000158987	2,984946	0,793118	0,001898	0,012148	3,664351	protein_coding	RAPGEF6	Rap guanine nucleotide exchange factor 6 [Source:HGNC Symbol;Acc:HGNC:20655]	5
ENSG00000162819	6,217542	-0,871778	0,0019	0,012156	-3,663871	protein_coding	BROX	BRO1 domain and CAAX motif containing [Source:HGNC Symbol;Acc:HGNC:26512]	1
ENSG00000239961	-3,482116	2,614532	0,001903	0,012165	3,663218	protein_coding	LILRA4	leukocyte immunoglobulin like receptor A4 [Source:HGNC Symbol;Acc:HGNC:15503]	19
ENSG00000143549	8,249158	-0,856477	0,001903	0,012165	-3,66317	protein_coding	TPM3	tropomyosin 3 [Source:HGNC Symbol;Acc:HGNC:12012]	1
ENSG00000139132	6,05219	0,749731	0,001904	0,012169	3,662852	protein_coding	FGD4	FYVE, RhoGEF and PH domain containing 4 [Source:HGNC Symbol;Acc:HGNC:19125]	12
ENSG00000277075	5,414151	1,688269	0,001907	0,012179	3,662284	protein_coding	H2AC8	H2A clustered histone 8 [Source:HGNC Symbol;Acc:HGNC:4724]	6
ENSG00000092200	-1,87582	2,290182	0,001915	0,012226	3,660334	protein_coding	RPGRIP1	RPGR interacting protein 1 [Source:HGNC Symbol;Acc:HGNC:13436]	14
ENSG00000239389	0,404318	2,308462	0,001918	0,012239	3,659665	protein_coding	PCDHA13	protocadherin alpha 13 [Source:HGNC Symbol;Acc:HGNC:8667]	5
ENSG00000278570	-2,40615	2,891528	0,00192	0,01225	3,659069	protein_coding	NR2E3	nuclear receptor subfamily 2 group E member 3 [Source:HGNC Symbol;Acc:HGNC:7974]	15
ENSG00000118557	1,805217	2,86247	0,001922	0,012259	3,65857	protein_coding	PMFBP1	polyamine modulated factor 1 binding protein 1 [Source:HGNC Symbol;Acc:HGNC:17728]	16
ENSG00000115183	6,486682	-1,0165	0,001925	0,012272	-3,657896	protein_coding	TANC1	tetratricopeptide repeat, ankyrin repeat and coiled-coil containing 1 [Source:HGNC Symbol;Acc:HGNC:29364]	2

ENSG00000149476	4,339058	0,82469	0,001932	0,012311	3,656262	protein_coding	TKFC	triokinase and FMN cyclase [Source:HGNC Symbol;Acc:HGNC:24552]	11
ENSG00000172139	-1,271476	2,676388	0,001934	0,012323	3,655643	protein_coding	SLC9C1	solute carrier family 9 member C1 [Source:HGNC Symbol;Acc:HGNC:31401]	3
ENSG00000184009	10,80677	-0,759584	0,001936	0,01233	-3,655176	protein_coding	ACTG1	actin gamma 1 [Source:HGNC Symbol;Acc:HGNC:144]	17
ENSG00000198886	13,11951	-1,27332	0,001937	0,012331	-3,654979	protein_coding	MT-ND4	mitochondrially encoded NADH:ubiquinone oxidoreductase core subunit 4 [Source:HGNC Symbol;Acc:HGNC:7459]	MT
ENSG00000205352	4,093188	-0,744407	0,001945	0,012374	-3,653203	protein_coding	PRR13	proline rich 13 [Source:HGNC Symbol;Acc:HGNC:24528]	12
ENSG00000169967	7,040166	-0,516968	0,001946	0,012377	-3,652881	protein_coding	MAP3K2	mitogen-activated protein kinase kinase kinase 2 [Source:HGNC Symbol;Acc:HGNC:6854]	2
ENSG00000213420	1,410495	1,754656	0,001948	0,012387	3,652344	protein_coding	GPC2	glypican 2 [Source:HGNC Symbol;Acc:HGNC:4450]	7
ENSG00000152402	4,227244	1,884565	0,001949	0,012389	3,652096	protein_coding	GUCY1A2	guanylate cyclase 1 soluble subunit alpha 2 [Source:HGNC Symbol;Acc:HGNC:4684]	11
ENSG00000266302	-1,249573	2,578125	0,001953	0,012406	3,651288	protein_coding	AC098850.	novel transcript	17
ENSG00000164144	4,732749	-0,6985	0,001956	0,012419	-3,65061	protein_coding	ARFIP1	ADP ribosylation factor interacting protein 1 [Source:HGNC Symbol;Acc:HGNC:21496]	4
ENSG00000174514	-0,480977	2,213254	0,001957	0,012424	3,65028	protein_coding	MFSD4A	major facilitator superfamily domain containing 4A [Source:HGNC Symbol;Acc:HGNC:25433]	1
ENSG00000119684	5,16222	0,662691	0,00196	0,012433	3,649733	protein_coding	MLH3	mutL homolog 3 [Source:HGNC Symbol;Acc:HGNC:7128]	14
ENSG00000181631	-1,249364	2,751865	0,001961	0,012438	3,649388	protein_coding	P2RY13	purinergic receptor P2Y13 [Source:HGNC Symbol;Acc:HGNC:4537]	3
ENSG00000138785	3,641379	-0,636343	0,001963	0,012444	-3,649004	protein_coding	INTS12	integrator complex subunit 12 [Source:HGNC Symbol;Acc:HGNC:25067]	4
ENSG00000103199	3,679125	0,713168	0,001968	0,01247	3,647861	protein_coding	ZNF500	zinc finger protein 500 [Source:HGNC Symbol;Acc:HGNC:23716]	16
ENSG00000100077	4,712215	1,095178	0,001973	0,012501	3,646547	protein_coding	GRK3	G protein-coupled receptor kinase 3 [Source:HGNC Symbol;Acc:HGNC:290]	22
ENSG00000130052	2,050228	2,710741	0,001977	0,012519	3,645704	protein_coding	STARD8	StAR related lipid transfer domain containing 8 [Source:HGNC Symbol;Acc:HGNC:19161]	X
ENSG00000196611	4,108406	-6,65606	0,001984	0,012557	-3,64414	protein_coding	MMP1	matrix metallopeptidase 1 [Source:HGNC Symbol;Acc:HGNC:7155]	11
ENSG00000165487	5,337887	-0,561387	0,001986	0,012567	-3,643562	protein_coding	MICU2	mitochondrial calcium uptake 2 [Source:HGNC Symbol;Acc:HGNC:31830]	13
ENSG00000169031	1,326904	3,391884	0,001988	0,012569	3,643223	protein_coding	COL4A3	collagen type IV alpha 3 chain [Source:HGNC Symbol;Acc:HGNC:2204]	2
ENSG00000134049	3,921772	-0,9092	0,001988	0,012569	-3,64316	protein_coding	IER3IP1	immediate early response 3 interacting protein 1 [Source:HGNC Symbol;Acc:HGNC:18550]	18
ENSG00000104853	7,387094	-0,679662	0,001989	0,012571	-3,64291	protein_coding	CLPTM1	CLPTM1 regulator of GABA type A receptor forward trafficking [Source:HGNC Symbol;Acc:HGNC:2087]	19
ENSG00000132522	3,145773	-0,852465	0,001992	0,012584	-3,642242	protein_coding	GPS2	G protein pathway suppressor 2 [Source:HGNC Symbol;Acc:HGNC:4550]	17
ENSG00000183023	4,2986	1,784201	0,001998	0,01261	3,640755	protein_coding	SLC8A1	solute carrier family 8 member A1 [Source:HGNC Symbol;Acc:HGNC:11068]	2
ENSG00000144535	5,197086	0,826017	0,001998	0,01261	3,640777	protein_coding	DIS3L2	DIS3 like 3'-5' exoribonuclease 2 [Source:HGNC Symbol;Acc:HGNC:28648]	2
ENSG00000143624	6,749534	0,867309	0,001998	0,01261	3,640754	protein_coding	INTS3	integrator complex subunit 3 [Source:HGNC Symbol;Acc:HGNC:26153]	1
ENSG00000132600	4,48513	0,649895	0,002002	0,012627	3,639967	protein_coding	PRMT7	protein arginine methyltransferase 7 [Source:HGNC Symbol;Acc:HGNC:25557]	16
ENSG00000167363	4,704627	1,582567	0,002004	0,012634	3,639547	protein_coding	FN3K	fructosamine 3 kinase [Source:HGNC Symbol;Acc:HGNC:24822]	17
ENSG00000100994	6,973475	-0,695439	0,002006	0,012645	-3,638972	protein_coding	PYGB	glycogen phosphorylase B [Source:HGNC Symbol;Acc:HGNC:9723]	20
ENSG00000205744	1,168332	2,522762	0,002008	0,012655	3,638418	protein_coding	DENND1C	DENN domain containing 1C [Source:HGNC Symbol;Acc:HGNC:26225]	19
ENSG00000160131	5,207762	-0,643874	0,00201	0,012657	-3,638152	protein_coding	VMA21	vacuolar ATPase assembly factor VMA21 [Source:HGNC Symbol;Acc:HGNC:22082]	X
ENSG00000161958	-2,047003	1,973474	0,002014	0,01268	3,637165	protein_coding	FGF11	fibroblast growth factor 11 [Source:HGNC Symbol;Acc:HGNC:3667]	17
ENSG00000172349	2,649861	2,203962	0,002018	0,012691	3,636283	protein_coding	IL16	interleukin 16 [Source:HGNC Symbol;Acc:HGNC:5980]	15
ENSG00000100902	4,026099	-0,580503	0,002017	0,012691	-3,636494	protein_coding	PSMA6	proteasome 20S subunit alpha 6 [Source:HGNC Symbol;Acc:HGNC:9535]	14
ENSG00000112234	5,241826	0,538132	0,002019	0,012691	3,636043	protein_coding	FBXL4	F-box and leucine rich repeat protein 4 [Source:HGNC Symbol;Acc:HGNC:13601]	6
ENSG00000153815	6,821317	1,021914	0,002019	0,012691	3,636078	protein_coding	CMIP	c-Maf inducing protein [Source:HGNC Symbol;Acc:HGNC:24319]	16
ENSG00000138311	0,235235	-2,663027	0,002023	0,012693	-3,635137	protein_coding	ZNF365	zinc finger protein 365 [Source:HGNC Symbol;Acc:HGNC:18194]	10
ENSG00000175489	0,662182	2,413183	0,002021	0,012693	3,635518	protein_coding	LRRC25	leucine rich repeat containing 25 [Source:HGNC Symbol;Acc:HGNC:29806]	19
ENSG00000166323	-1,234338	1,678246	0,002023	0,012693	3,635088	protein_coding	C11orf65	chromosome 11 open reading frame 65 [Source:HGNC Symbol;Acc:HGNC:28519]	11
ENSG00000167995	3,538303	-1,233092	0,002022	0,012693	-3,635326	protein_coding	BEST1	bestrophin 1 [Source:HGNC Symbol;Acc:HGNC:12703]	11
ENSG00000160584	6,53285	0,446885	0,002022	0,012693	3,635433	protein_coding	SIK3	SIK family kinase 3 [Source:HGNC Symbol;Acc:HGNC:29165]	11
ENSG00000136052	4,836841	-0,985667	0,002025	0,012695	-3,634666	protein_coding	SLC41A2	solute carrier family 41 member 2 [Source:HGNC Symbol;Acc:HGNC:31045]	12
ENSG00000198146	5,844356	-0,70554	0,002025	0,012695	-3,634741	protein_coding	ZNF770	zinc finger protein 770 [Source:HGNC Symbol;Acc:HGNC:26061]	15
ENSG00000141540	3,892922	1,67833	0,002026	0,0127	3,634314	protein_coding	TTYH2	tweety family member 2 [Source:HGNC Symbol;Acc:HGNC:13877]	17
ENSG00000128563	4,879498	0,797216	0,00203	0,012718	3,633477	protein_coding	PRKRIP1	PRKR interacting protein 1 [Source:HGNC Symbol;Acc:HGNC:21894]	7
ENSG00000131089	5,265007	1,047336	0,002031	0,012719	3,633248	protein_coding	ARHGEF9	Cdc42 guanine nucleotide exchange factor 9 [Source:HGNC Symbol;Acc:HGNC:14561]	X
ENSG00000166889	6,03071	-0,550334	0,002035	0,012736	-3,632468	protein_coding	PATL1	PAT1 homolog 1, processing body mRNA decay factor [Source:HGNC Symbol;Acc:HGNC:26721]	11
ENSG00000142233	-2,057991	3,074401	0,002038	0,012747	3,63173	protein_coding	NTN5	netrin 5 [Source:HGNC Symbol;Acc:HGNC:25208]	19
ENSG00000177873	3,421804	0,826882	0,002038	0,012747	3,631706	protein_coding	ZNF619	zinc finger protein 619 [Source:HGNC Symbol;Acc:HGNC:26910]	3
ENSG00000179954	5,347423	-1,86883	0,002041	0,012763	-3,630989	protein_coding	SSC5D	scavenger receptor cysteine rich family member with 5 domains [Source:HGNC Symbol;Acc:HGNC:26641]	19
ENSG00000152154	0,225449	2,019863	0,002046	0,01278	3,629814	protein_coding	TMEM178/	transmembrane protein 178A [Source:HGNC Symbol;Acc:HGNC:28517]	2

ENSG00000139549	-2,246776	2,837048	0,002046	0,01278	3,629879	protein_coding	DHH	desert hedgehog signaling molecule [Source:HGNC Symbol;Acc:HGNC:2865]	12
ENSG00000143622	5,29118	-0,648129	0,002045	0,01278	-3,630068	protein_coding	RIT1	Ras like without CAAX 1 [Source:HGNC Symbol;Acc:HGNC:10023]	1
ENSG00000032444	6,326178	-0,489607	0,002049	0,012789	-3,629322	protein_coding	PNPLA6	patatin like phospholipase domain containing 6 [Source:HGNC Symbol;Acc:HGNC:16268]	19
ENSG00000159596	3,334332	-0,791504	0,00205	0,01279	-3,629099	protein_coding	TMEM69	transmembrane protein 69 [Source:HGNC Symbol;Acc:HGNC:28035]	1
ENSG00000125753	6,474819	-0,876594	0,00205	0,01279	-3,628953	protein_coding	VASP	vasodilator stimulated phosphoprotein [Source:HGNC Symbol;Acc:HGNC:12652]	19
ENSG00000178935	3,002329	1,02466	0,002053	0,012799	3,628444	protein_coding	ZNF552	zinc finger protein 552 [Source:HGNC Symbol;Acc:HGNC:26135]	19
ENSG00000167986	7,964883	-0,511029	0,002054	0,012804	-3,628102	protein_coding	DDB1	damage specific DNA binding protein 1 [Source:HGNC Symbol;Acc:HGNC:2717]	11
ENSG00000123358	5,491673	2,124494	0,002059	0,012831	3,626962	protein_coding	NR4A1	nuclear receptor subfamily 4 group A member 1 [Source:HGNC Symbol;Acc:HGNC:7980]	12
ENSG00000197599	0,536454	1,719852	0,002061	0,012834	3,626668	protein_coding	CCDC154	coiled-coil domain containing 154 [Source:HGNC Symbol;Acc:HGNC:34454]	16
ENSG00000257093	6,227427	1,030753	0,002066	0,012861	3,62551	protein_coding	DENND11	DENN domain containing 11 [Source:HGNC Symbol;Acc:HGNC:29472]	7
ENSG00000273079	-0,134787	2,731191	0,002068	0,012862	3,624951	protein_coding	GRIN2B	glutamate ionotropic receptor NMDA type subunit 2B [Source:HGNC Symbol;Acc:HGNC:4586]	12
ENSG00000163075	-0,910711	3,727632	0,002072	0,012862	3,624194	protein_coding	CFAP221	cilia and flagella associated protein 221 [Source:HGNC Symbol;Acc:HGNC:33720]	2
ENSG00000163792	-2,453708	4,005089	0,002067	0,012862	3,62521	protein_coding	TCF23	transcription factor 23 [Source:HGNC Symbol;Acc:HGNC:18602]	2
ENSG00000183379	-2,625052	2,534949	0,00207	0,012862	3,624549	protein_coding	SYNDIG1L	synapse differentiation inducing 1 like [Source:HGNC Symbol;Acc:HGNC:32388]	14
ENSG00000161281	2,148386	-1,862253	0,002072	0,012862	-3,624077	protein_coding	COX7A1	cytochrome c oxidase subunit 7A1 [Source:HGNC Symbol;Acc:HGNC:2287]	19
ENSG00000276234	4,03199	0,799148	0,00207	0,012862	3,624641	protein_coding	TADA2A	transcriptional adaptor 2A [Source:HGNC Symbol;Acc:HGNC:11531]	17
ENSG00000067113	4,59488	-1,432131	0,00207	0,012862	-3,624539	protein_coding	PLPP1	phospholipid phosphatase 1 [Source:HGNC Symbol;Acc:HGNC:9228]	5
ENSG00000100813	8,189629	0,477288	0,002071	0,012862	3,624355	protein_coding	ACIN1	apoptotic chromatin condensation inducer 1 [Source:HGNC Symbol;Acc:HGNC:17066]	14
ENSG00000171180	-2,745049	3,15401	0,002079	0,012894	3,622551	protein_coding	OR2M4	olfactory receptor family 2 subfamily M member 4 [Source:HGNC Symbol;Acc:HGNC:8270]	1
ENSG00000251247	2,466484	1,082689	0,002079	0,012894	3,622649	protein_coding	ZNF345	zinc finger protein 345 [Source:HGNC Symbol;Acc:HGNC:16367]	19
ENSG00000050393	5,431739	-0,750065	0,00208	0,012894	-3,622426	protein_coding	MCUR1	mitochondrial calcium uniporter regulator 1 [Source:HGNC Symbol;Acc:HGNC:21097]	6
ENSG00000204084	4,911529	0,587493	0,002088	0,012942	3,620546	protein_coding	INPP5B	inositol polyphosphate-5-phosphatase B [Source:HGNC Symbol;Acc:HGNC:6077]	1
ENSG00000010671	0,692825	2,909046	0,002091	0,012954	3,619918	protein_coding	BTK	Bruton tyrosine kinase [Source:HGNC Symbol;Acc:HGNC:1133]	X
ENSG00000006468	5,484671	-1,727451	0,002093	0,012959	-3,6196	protein_coding	ETV1	ETS variant transcription factor 1 [Source:HGNC Symbol;Acc:HGNC:3490]	7
ENSG00000136231	2,973193	-4,307359	0,002094	0,01296	-3,619261	protein_coding	IGF2BP3	insulin like growth factor 2 mRNA binding protein 3 [Source:HGNC Symbol;Acc:HGNC:28868]	7
ENSG00000159069	6,451206	-0,664436	0,002094	0,01296	-3,619204	protein_coding	FBXW5	F-box and WD repeat domain containing 5 [Source:HGNC Symbol;Acc:HGNC:13613]	9
ENSG00000114784	4,702624	-0,712027	0,002103	0,013007	-3,617353	protein_coding	EIF1B	eukaryotic translation initiation factor 1B [Source:HGNC Symbol;Acc:HGNC:30792]	3
ENSG00000244617	-0,691748	1,880709	0,002107	0,013029	3,616414	protein_coding	ASPRV1	aspartic peptidase retroviral like 1 [Source:HGNC Symbol;Acc:HGNC:26321]	2
ENSG00000186431	-2,040254	2,764245	0,002109	0,013036	3,615989	protein_coding	FCAR	Fc fragment of IgA receptor [Source:HGNC Symbol;Acc:HGNC:3608]	19
ENSG00000188050	-1,373899	2,465647	0,002111	0,013039	3,615577	protein_coding	RNF133	ring finger protein 133 [Source:HGNC Symbol;Acc:HGNC:21154]	7
ENSG00000171307	4,874161	-0,511035	0,002111	0,013039	-3,61555	protein_coding	ZDHHC16	zinc finger DHHC-type palmitoyltransferase 16 [Source:HGNC Symbol;Acc:HGNC:20714]	10
ENSG00000108641	3,798317	1,654201	0,002116	0,013064	3,614394	protein_coding	B9D1	B9 domain containing 1 [Source:HGNC Symbol;Acc:HGNC:24123]	17
ENSG00000153317	6,920847	-0,989416	0,002117	0,013064	-3,614301	protein_coding	ASAP1	ArfGAP with SH3 domain, ankyrin repeat and PH domain 1 [Source:HGNC Symbol;Acc:HGNC:2720]	8
ENSG00000168658	-1,800897	2,780142	0,002121	0,013087	3,613335	protein_coding	VWA3B	von Willebrand factor A domain containing 3B [Source:HGNC Symbol;Acc:HGNC:28385]	2
ENSG00000126353	-1,742742	2,959706	0,002125	0,01309	3,612593	protein_coding	CCR7	C-C motif chemokine receptor 7 [Source:HGNC Symbol;Acc:HGNC:1608]	17
ENSG00000118946	2,783567	2,971386	0,002124	0,01309	3,612682	protein_coding	PCDH17	protocadherin 17 [Source:HGNC Symbol;Acc:HGNC:14267]	13
ENSG00000173275	3,372445	0,95316	0,002123	0,01309	3,61288	protein_coding	ZNF449	zinc finger protein 449 [Source:HGNC Symbol;Acc:HGNC:21039]	X
ENSG00000168938	5,494957	-1,412228	0,002125	0,01309	-3,612524	protein_coding	PPIC	peptidylprolyl isomerase C [Source:HGNC Symbol;Acc:HGNC:9256]	5
ENSG00000164591	-1,278239	2,363782	0,002129	0,013108	3,611715	protein_coding	MYOZ3	myozenin 3 [Source:HGNC Symbol;Acc:HGNC:18565]	5
ENSG00000091106	-0,519061	2,700069	0,00213	0,013112	3,611397	protein_coding	NLRC4	NLR family CARD domain containing 4 [Source:HGNC Symbol;Acc:HGNC:16412]	2
ENSG00000137575	6,69698	-0,892193	0,002131	0,013115	-3,611131	protein_coding	SDCBP	syndecan binding protein [Source:HGNC Symbol;Acc:HGNC:10662]	8
ENSG00000014641	6,256551	-0,578226	0,002134	0,013124	-3,610641	protein_coding	MDH1	malate dehydrogenase 1 [Source:HGNC Symbol;Acc:HGNC:6970]	2
ENSG00000164023	4,504834	-1,433312	0,002136	0,013133	-3,610162	protein_coding	SGMS2	sphingomyelin synthase 2 [Source:HGNC Symbol;Acc:HGNC:28395]	4
ENSG00000242372	5,174277	-0,539829	0,002139	0,01314	-3,609565	protein_coding	EIF6	eukaryotic translation initiation factor 6 [Source:HGNC Symbol;Acc:HGNC:6159]	20
ENSG00000197603	6,444792	0,65482	0,002139	0,01314	3,609561	protein_coding	CPLANE1	ciliogenesis and planar polarity effector 1 [Source:HGNC Symbol;Acc:HGNC:25801]	5
ENSG00000167552	7,536731	-1,332875	0,002144	0,013167	-3,608456	protein_coding	TUBA1A	tubulin alpha 1a [Source:HGNC Symbol;Acc:HGNC:20766]	12
ENSG00000120332	-0,560903	2,486803	0,002146	0,013169	3,608013	protein_coding	TNN	tenascin N [Source:HGNC Symbol;Acc:HGNC:22942]	1
ENSG00000165895	4,741536	-1,2191	0,002145	0,013169	-3,608162	protein_coding	ARHGAP42	Rho GTPase activating protein 42 [Source:HGNC Symbol;Acc:HGNC:26545]	11
ENSG00000123989	6,916313	-0,831466	0,002149	0,013185	-3,607294	protein_coding	CHPF	chondroitin polymerizing factor [Source:HGNC Symbol;Acc:HGNC:24291]	2
ENSG00000153046	5,739926	-0,509719	0,002154	0,013201	-3,606234	protein_coding	CDYL	chromodomain Y like [Source:HGNC Symbol;Acc:HGNC:1811]	6
ENSG00000160305	6,132658	0,686294	0,002154	0,013201	3,606349	protein_coding	DIP2A	disco interacting protein 2 homolog A [Source:HGNC Symbol;Acc:HGNC:17217]	21

ENSG00000197702	6,627355	-1,513872	0,002153	0,013201	-3,606559	protein_coding	PARVA	parvin alpha [Source:HGNC Symbol;Acc:HGNC:14652]	11
ENSG00000178202	5,445004	-1,135529	0,002156	0,013203	-3,605977	protein_coding	POGLUT3	protein O-glucosyltransferase 3 [Source:HGNC Symbol;Acc:HGNC:28496]	11
ENSG00000123130	4,756414	-1,225626	0,002159	0,013218	-3,605192	protein_coding	ACOT9	acyl-CoA thioesterase 9 [Source:HGNC Symbol;Acc:HGNC:17152]	X
ENSG00000173706	6,946044	-1,593161	0,00216	0,013218	-3,605101	protein_coding	HEG1	heart development protein with EGF like domains 1 [Source:HGNC Symbol;Acc:HGNC:29227]	3
ENSG00000167792	6,154471	0,488242	0,002161	0,013224	3,604726	protein_coding	NDUFV1	NADH:ubiquinone oxidoreductase core subunit V1 [Source:HGNC Symbol;Acc:HGNC:7716]	11
ENSG00000100410	4,123215	-0,699246	0,002167	0,013256	-3,603447	protein_coding	PHF5A	PHD finger protein 5A [Source:HGNC Symbol;Acc:HGNC:18000]	22
ENSG00000269313	1,832868	1,633477	0,002169	0,01326	3,60315	protein_coding	MAGIX	MAGI family member, X-linked [Source:HGNC Symbol;Acc:HGNC:30006]	X
ENSG00000136536	6,670007	-0,660556	0,002173	0,01328	-3,60227	protein_coding	MARCHF7	membrane associated ring-CH-type finger 7 [Source:HGNC Symbol;Acc:HGNC:17393]	2
ENSG00000127366	-0,735348	1,538609	0,002174	0,013282	3,602044	protein_coding	TAS2R5	taste 2 receptor member 5 [Source:HGNC Symbol;Acc:HGNC:14912]	7
ENSG00000143761	8,155417	-0,538247	0,002179	0,013304	-3,601111	protein_coding	ARF1	ADP ribosylation factor 1 [Source:HGNC Symbol;Acc:HGNC:652]	1
ENSG00000121716	3,35178	1,614183	0,002182	0,013319	3,600286	protein_coding	PILRB	paired immunoglobin like type 2 receptor beta [Source:HGNC Symbol;Acc:HGNC:18297]	7
ENSG00000169249	4,490283	0,720388	0,002183	0,013319	3,600246	protein_coding	ZRSR2	zinc finger CCCH-type, RNA binding motif and serine/arginine rich 2 [Source:HGNC Symbol;Acc:HGNC:23019]	X
ENSG00000111644	1,243195	1,56544	0,002184	0,013323	3,599919	protein_coding	ACRBP	acrosin binding protein [Source:HGNC Symbol;Acc:HGNC:17195]	12
ENSG00000183155	3,543892	-0,89087	0,002186	0,013329	-3,599546	protein_coding	RABIF	RAB interacting factor [Source:HGNC Symbol;Acc:HGNC:9797]	1
ENSG00000139537	-1,477925	2,935718	0,002193	0,013363	3,598019	protein_coding	CCDC65	coiled-coil domain containing 65 [Source:HGNC Symbol;Acc:HGNC:29937]	12
ENSG00000133247	3,216499	1,551413	0,002193	0,013363	3,598044	protein_coding	KMT5C	lysine methyltransferase 5C [Source:HGNC Symbol;Acc:HGNC:28405]	19
ENSG00000146072	6,346969	-0,78887	0,002195	0,013371	-3,597588	protein_coding	TNFRSF21	TNF receptor superfamily member 21 [Source:HGNC Symbol;Acc:HGNC:13469]	6
ENSG00000138315	-1,304037	2,267281	0,0022	0,013395	3,596602	protein_coding	OIT3	oncoprotein induced transcript 3 [Source:HGNC Symbol;Acc:HGNC:29953]	10
ENSG00000119866	3,610603	2,750821	0,002202	0,013399	3,596277	protein_coding	BCL11A	BAF chromatin remodeling complex subunit BCL11A [Source:HGNC Symbol;Acc:HGNC:13221]	2
ENSG00000130779	7,254513	-0,455317	0,002204	0,013407	-3,595848	protein_coding	CLIP1	CAP-Gly domain containing linker protein 1 [Source:HGNC Symbol;Acc:HGNC:10461]	12
ENSG00000083520	6,181658	-0,620799	0,00221	0,013439	-3,594578	protein_coding	DIS3	DIS3 homolog, exosome endoribonuclease and 3'-5' exoribonuclease [Source:HGNC Symbol;Acc:HGNC:20604]	13
ENSG00000070476	5,400503	0,752946	0,002213	0,013453	3,593925	protein_coding	ZXDC	ZXD family zinc finger C [Source:HGNC Symbol;Acc:HGNC:28160]	3
ENSG00000157916	5,931819	-0,739885	0,002214	0,013453	-3,593734	protein_coding	RER1	retention in endoplasmic reticulum sorting receptor 1 [Source:HGNC Symbol;Acc:HGNC:30309]	1
ENSG00000169418	1,08903	3,483408	0,002218	0,01347	3,59282	protein_coding	NPR1	natriuretic peptide receptor 1 [Source:HGNC Symbol;Acc:HGNC:7943]	1
ENSG00000204619	5,731475	-0,742349	0,002218	0,01347	-3,592837	protein_coding	PPP1R11	protein phosphatase 1 regulatory inhibitor subunit 11 [Source:HGNC Symbol;Acc:HGNC:9285]	6
ENSG00000117859	6,355213	-0,607007	0,00222	0,013476	-3,592462	protein_coding	OSBPL9	oxysterol binding protein like 9 [Source:HGNC Symbol;Acc:HGNC:16386]	1
ENSG00000176601	-3,32975	2,952754	0,002224	0,013494	3,591655	protein_coding	MAP3K19	mitogen-activated protein kinase kinase kinase 19 [Source:HGNC Symbol;Acc:HGNC:26249]	2
ENSG00000140105	6,869587	-0,760097	0,002227	0,013508	-3,59101	protein_coding	WARS1	tryptophanyl-tRNA synthetase 1 [Source:HGNC Symbol;Acc:HGNC:12729]	14
ENSG00000134884	7,600357	1,059476	0,002229	0,013514	3,590655	protein_coding	ARGLU1	arginine and glutamate rich 1 [Source:HGNC Symbol;Acc:HGNC:25482]	13
ENSG00000120438	7,001747	-0,946543	0,002233	0,013536	-3,589735	protein_coding	TCP1	t-complex 1 [Source:HGNC Symbol;Acc:HGNC:11655]	6
ENSG00000139714	-0,210244	2,538492	0,002238	0,013558	3,588791	protein_coding	MORN3	MORN repeat containing 3 [Source:HGNC Symbol;Acc:HGNC:29807]	12
ENSG00000172232	-3,489854	2,045265	0,002239	0,013558	3,588634	protein_coding	AZU1	azurocidin 1 [Source:HGNC Symbol;Acc:HGNC:913]	19
ENSG00000132688	6,868005	-1,570217	0,002241	0,013566	-3,588181	protein_coding	NES	nestin [Source:HGNC Symbol;Acc:HGNC:7756]	1
ENSG00000103769	6,790488	-0,688199	0,002242	0,013567	-3,587995	protein_coding	RAB11A	RAB11A, member RAS oncogene family [Source:HGNC Symbol;Acc:HGNC:9760]	15
ENSG00000134061	0,232596	2,549287	0,002246	0,01357	3,587035	protein_coding	CD180	CD180 molecule [Source:HGNC Symbol;Acc:HGNC:6726]	5
ENSG00000140931	4,029769	-1,328916	0,002246	0,01357	-3,587088	protein_coding	CMTM3	CKLF like MARVEL transmembrane domain containing 3 [Source:HGNC Symbol;Acc:HGNC:19174]	16
ENSG00000173214	4,795712	0,938193	0,002243	0,01357	3,587641	protein_coding	MFSD4B	major facilitator superfamily domain containing 4B [Source:HGNC Symbol;Acc:HGNC:21053]	6
ENSG00000165288	6,300066	0,857682	0,002245	0,01357	3,587334	protein_coding	BRWD3	bromodomain and WD repeat domain containing 3 [Source:HGNC Symbol;Acc:HGNC:17342]	X
ENSG00000143341	6,928406	-1,926583	0,002244	0,01357	-3,587494	protein_coding	HMCN1	hemicentin 1 [Source:HGNC Symbol;Acc:HGNC:19194]	1
ENSG00000213906	1,9237	1,368757	0,002247	0,013572	3,586801	protein_coding	LTB4R2	leukotriene B4 receptor 2 [Source:HGNC Symbol;Acc:HGNC:19260]	14
ENSG00000128011	1,170354	2,736848	0,00225	0,013579	3,586373	protein_coding	LRFN1	leucine rich repeat and fibronectin type III domain containing 1 [Source:HGNC Symbol;Acc:HGNC:29290]	19
ENSG00000173262	-2,248095	3,044078	0,002255	0,01359	3,585189	protein_coding	SLC2A14	solute carrier family 2 member 14 [Source:HGNC Symbol;Acc:HGNC:18301]	12
ENSG00000165495	2,681273	-2,162074	0,002254	0,01359	-3,585486	protein_coding	PKNOX2	PBX/knotted 1 homeobox 2 [Source:HGNC Symbol;Acc:HGNC:16714]	11
ENSG00000089094	5,403335	0,683034	0,002256	0,01359	3,585152	protein_coding	KDM2B	lysine demethylase 2B [Source:HGNC Symbol;Acc:HGNC:13610]	12
ENSG00000111843	5,331276	-0,611702	0,002256	0,01359	-3,585159	protein_coding	TMEM14C	transmembrane protein 14C [Source:HGNC Symbol;Acc:HGNC:20952]	6
ENSG00000179119	5,602311	-0,589362	0,002253	0,01359	-3,585612	protein_coding	SPTY2D1	SPT2 chromatin protein domain containing 1 [Source:HGNC Symbol;Acc:HGNC:26818]	11
ENSG00000126777	8,74509	-0,484399	0,002258	0,013602	-3,584593	protein_coding	KTN1	kinectin 1 [Source:HGNC Symbol;Acc:HGNC:6467]	14
ENSG00000198825	5,00578	-0,724016	0,002259	0,013603	-3,584366	protein_coding	INPP5F	inositol polyphosphate-5-phosphatase F [Source:HGNC Symbol;Acc:HGNC:17054]	10
ENSG00000132356	6,499477	-0,564303	0,00226	0,013603	-3,584204	protein_coding	PRKAA1	protein kinase AMP-activated catalytic subunit alpha 1 [Source:HGNC Symbol;Acc:HGNC:9376]	5
ENSG00000017427	0,803017	5,233572	0,002267	0,013631	3,58276	protein_coding	IGF1	insulin like growth factor 1 [Source:HGNC Symbol;Acc:HGNC:5464]	12
ENSG00000160808	-1,281844	2,191303	0,002267	0,013631	3,582911	protein_coding	MYL3	myosin light chain 3 [Source:HGNC Symbol;Acc:HGNC:7584]	3

ENSG00000100139	5,728822	-0,679654	0,002266	0,013631	-3,58307	protein_coding	MICALL1	MICAL like 1 [Source:HGNC Symbol;Acc:HGNC:29804]	22
ENSG00000276966	5,829707	0,790649	0,002268	0,013632	3,58255	protein_coding	H4C5	H4 clustered histone 5 [Source:HGNC Symbol;Acc:HGNC:4790]	6
ENSG00000185716	3,941079	-0,760971	0,002271	0,013644	-3,581969	protein_coding	MOSMO	modulator of smoothened [Source:HGNC Symbol;Acc:HGNC:27087]	16
ENSG00000119669	7,079868	-0,749825	0,002273	0,013651	-3,581586	protein_coding	IRF2BPL	interferon regulatory factor 2 binding protein like [Source:HGNC Symbol;Acc:HGNC:14282]	14
ENSG00000174944	-1,380927	2,734598	0,002275	0,013658	3,581171	protein_coding	P2RY14	purinergic receptor P2Y14 [Source:HGNC Symbol;Acc:HGNC:16442]	3
ENSG00000008282	4,886856	-0,867901	0,002277	0,013663	-3,5808	protein_coding	SYPL1	synaptophysin like 1 [Source:HGNC Symbol;Acc:HGNC:11507]	7
ENSG00000128463	5,492176	-0,634592	0,002278	0,013663	-3,58066	protein_coding	EMC4	ER membrane protein complex subunit 4 [Source:HGNC Symbol;Acc:HGNC:28032]	15
ENSG00000152784	1,838347	-2,12015	0,002286	0,013705	-3,578965	protein_coding	PRDM8	PR/SET domain 8 [Source:HGNC Symbol;Acc:HGNC:13993]	4
ENSG00000109929	5,861512	-0,843353	0,002286	0,013705	-3,578915	protein_coding	SC5D	sterol-C5-desaturase [Source:HGNC Symbol;Acc:HGNC:10547]	11
ENSG00000138964	2,614883	3,305607	0,002288	0,013706	3,578645	protein_coding	PARVG	parvin gamma [Source:HGNC Symbol;Acc:HGNC:14654]	22
ENSG00000086619	4,923056	1,622172	0,002288	0,013706	3,578558	protein_coding	ERO1B	endoplasmic reticulum oxidoreductase 1 beta [Source:HGNC Symbol;Acc:HGNC:14355]	1
ENSG00000205078	0,519897	1,973196	0,002296	0,013745	3,577076	protein_coding	SYCE1L	synaptonemal complex central element protein 1 like [Source:HGNC Symbol;Acc:HGNC:37236]	16
ENSG00000112837	1,252021	-2,060777	0,002296	0,013745	-3,576904	protein_coding	TBX18	T-box transcription factor 18 [Source:HGNC Symbol;Acc:HGNC:11595]	6
ENSG00000185989	5,82684	-1,213879	0,002298	0,013748	-3,576645	protein_coding	RASA3	RAS p21 protein activator 3 [Source:HGNC Symbol;Acc:HGNC:20331]	13
ENSG00000136940	5,272587	-0,435446	0,002303	0,013773	-3,575635	protein_coding	PDCL	phosducin like [Source:HGNC Symbol;Acc:HGNC:8770]	9
ENSG00000148719	5,591675	-0,450132	0,002312	0,013824	-3,573761	protein_coding	DNAJB12	DnaJ heat shock protein family (Hsp40) member B12 [Source:HGNC Symbol;Acc:HGNC:14891]	10
ENSG00000168679	2,114537	-2,416372	0,002324	0,013883	-3,571484	protein_coding	SLC16A4	solute carrier family 16 member 4 [Source:HGNC Symbol;Acc:HGNC:10925]	1
ENSG00000184838	2,921051	-2,33582	0,002324	0,013883	-3,571463	protein_coding	PRR16	proline rich 16 [Source:HGNC Symbol;Acc:HGNC:29654]	5
ENSG00000069345	6,352707	-0,644292	0,002325	0,013884	-3,571269	protein_coding	DNAJA2	DnaJ heat shock protein family (Hsp40) member A2 [Source:HGNC Symbol;Acc:HGNC:14884]	16
ENSG00000136908	4,403267	-0,727079	0,002335	0,01394	-3,569264	protein_coding	DPM2	dolichyl-phosphate mannosyltransferase subunit 2, regulatory [Source:HGNC Symbol;Acc:HGNC:3006]	9
ENSG00000168071	2,995667	3,028706	0,002336	0,013943	3,568991	protein_coding	CCDC88B	coiled-coil domain containing 88B [Source:HGNC Symbol;Acc:HGNC:26757]	11
ENSG00000183741	7,667178	-0,44209	0,002339	0,013954	-3,568445	protein_coding	CBX6	chromobox 6 [Source:HGNC Symbol;Acc:HGNC:1556]	22
ENSG00000163611	1,648769	1,120686	0,002343	0,013971	3,56774	protein_coding	SPICE1	spindle and centriole associated protein 1 [Source:HGNC Symbol;Acc:HGNC:25083]	3
ENSG00000197629	3,252856	2,889322	0,002347	0,013991	3,566912	protein_coding	MPEG1	macrophage expressed 1 [Source:HGNC Symbol;Acc:HGNC:29619]	11
ENSG00000033030	4,975583	0,562798	0,002349	0,013996	3,566565	protein_coding	ZCCHC8	zinc finger CCHC-type containing 8 [Source:HGNC Symbol;Acc:HGNC:25265]	12
ENSG00000164366	4,915163	-0,618361	0,002361	0,014062	-3,564237	protein_coding	CCDC127	coiled-coil domain containing 127 [Source:HGNC Symbol;Acc:HGNC:30520]	5
ENSG00000244687	2,616404	0,89108	0,002364	0,014071	3,563624	protein_coding	UBE2V1	ubiquitin conjugating enzyme E2 V1 [Source:HGNC Symbol;Acc:HGNC:12494]	20
ENSG00000120733	7,250817	0,447006	0,002363	0,014071	3,563784	protein_coding	KDM3B	lysine demethylase 3B [Source:HGNC Symbol;Acc:HGNC:1337]	5
ENSG00000244482	0,17572	2,157327	0,002367	0,014082	3,56293	protein_coding	LILRA6	leukocyte immunoglobulin like receptor A6 [Source:HGNC Symbol;Acc:HGNC:15495]	19
ENSG00000172578	2,291888	2,39407	0,002367	0,014082	3,563092	protein_coding	KLHL6	kelch like family member 6 [Source:HGNC Symbol;Acc:HGNC:18653]	3
ENSG00000105854	5,368382	-1,109853	0,002369	0,014088	-3,562565	protein_coding	PON2	paraoxonase 2 [Source:HGNC Symbol;Acc:HGNC:9205]	7
ENSG00000128656	4,614728	-1,106081	0,002378	0,014131	-3,56083	protein_coding	CHN1	chimerin 1 [Source:HGNC Symbol;Acc:HGNC:1943]	2
ENSG00000148730	7,189705	-0,836286	0,002377	0,014131	-3,560988	protein_coding	EIF4EBP2	eukaryotic translation initiation factor 4E binding protein 2 [Source:HGNC Symbol;Acc:HGNC:3289]	10
ENSG00000108465	3,808367	1,077925	0,00238	0,014135	3,560533	protein_coding	CDK5RAP3	CDK5 regulatory subunit associated protein 3 [Source:HGNC Symbol;Acc:HGNC:18673]	17
ENSG00000124614	2,26126	2,085759	0,002381	0,014137	3,560288	protein_coding	RPS10	ribosomal protein S10 [Source:HGNC Symbol;Acc:HGNC:10383]	6
ENSG00000117500	6,130574	-0,784119	0,002385	0,014152	-3,559616	protein_coding	TMED5	transmembrane p24 trafficking protein 5 [Source:HGNC Symbol;Acc:HGNC:24251]	1
ENSG00000164323	5,138573	-0,961613	0,002389	0,014173	-3,558766	protein_coding	CFAP97	cilia and flagella associated protein 97 [Source:HGNC Symbol;Acc:HGNC:29276]	4
ENSG00000143162	6,550879	-0,980622	0,002395	0,014203	-3,557639	protein_coding	CREG1	cellular repressor of E1A stimulated genes 1 [Source:HGNC Symbol;Acc:HGNC:2351]	1
ENSG00000117519	7,191537	-1,485372	0,002397	0,014212	-3,557186	protein_coding	CNN3	calponin 3 [Source:HGNC Symbol;Acc:HGNC:2157]	1
ENSG00000106554	5,905586	-0,467122	0,002403	0,01424	-3,556108	protein_coding	CHCHD3	coiled-coil-helix-coiled-coil-helix domain containing 3 [Source:HGNC Symbol;Acc:HGNC:21906]	7
ENSG00000174574	5,779869	-0,901232	0,002404	0,014242	-3,555884	protein_coding	AKIRIN1	akirin 1 [Source:HGNC Symbol;Acc:HGNC:25744]	1
ENSG00000253958	0,086478	-1,346075	0,002407	0,014255	-3,55525	protein_coding	CLDN23	claudin 23 [Source:HGNC Symbol;Acc:HGNC:17591]	8
ENSG00000135083	3,089219	-1,533448	0,002409	0,014255	-3,554908	protein_coding	CCNJL	cyclin J like [Source:HGNC Symbol;Acc:HGNC:25876]	5
ENSG00000177683	4,910742	-0,566122	0,00241	0,014255	-3,554792	protein_coding	THAP5	THAP domain containing 5 [Source:HGNC Symbol;Acc:HGNC:23188]	7
ENSG00000129187	5,241988	-0,802772	0,00241	0,014255	-3,554798	protein_coding	DCTD	dCMP deaminase [Source:HGNC Symbol;Acc:HGNC:2710]	4
ENSG00000181518	-3,160599	3,260414	0,002413	0,014267	3,554254	protein_coding	OR8D4	olfactory receptor family 8 subfamily D member 4 [Source:HGNC Symbol;Acc:HGNC:14840]	11
ENSG00000182631	-0,659506	-2,198361	0,002418	0,014294	-3,553199	protein_coding	RXFP3	relaxin family peptide receptor 3 [Source:HGNC Symbol;Acc:HGNC:24883]	5
ENSG00000274286	-0,791416	2,128482	0,002423	0,014317	3,552306	protein_coding	ADRA2B	adrenoceptor alpha 2B [Source:HGNC Symbol;Acc:HGNC:282]	2
ENSG00000142002	6,962767	0,731729	0,002424	0,014317	3,552139	protein_coding	DPP9	dipeptidyl peptidase 9 [Source:HGNC Symbol;Acc:HGNC:18648]	19
ENSG00000258429	1,866454	-0,815442	0,002429	0,014343	-3,551147	protein_coding	PDF	peptide deformylase, mitochondrial [Source:HGNC Symbol;Acc:HGNC:30012]	16
ENSG00000197586	6,067536	0,8016	0,002431	0,014352	3,550687	protein_coding	ENTPD6	ectonucleoside triphosphate diphosphohydrolase 6 [Source:HGNC Symbol;Acc:HGNC:3368]	20

ENSG00000100592	6,088322	0,632585	0,002433	0,014355	3,550425	protein_coding	DAAM1	dishevelled associated activator of morphogenesis 1 [Source:HGNC Symbol;Acc:HGNC:18142]	14
ENSG00000087076	2,665561	1,824231	0,002444	0,014413	3,54839	protein_coding	HSD17B14	hydroxysteroid 17-beta dehydrogenase 14 [Source:HGNC Symbol;Acc:HGNC:23238]	19
ENSG00000198589	7,486404	0,733756	0,002448	0,014432	3,547639	protein_coding	LRBA	LPS responsive beige-like anchor protein [Source:HGNC Symbol;Acc:HGNC:1742]	4
ENSG00000106665	7,251486	-0,868393	0,002451	0,014446	-3,546997	protein_coding	CLIP2	CAP-Gly domain containing linker protein 2 [Source:HGNC Symbol;Acc:HGNC:2586]	7
ENSG00000198925	5,0311	-0,569807	0,002452	0,014448	-3,546776	protein_coding	ATG9A	autophagy related 9A [Source:HGNC Symbol;Acc:HGNC:22408]	2
ENSG00000188001	1,00027	1,681014	0,002455	0,014455	3,546176	protein_coding	TPRG1	tumor protein p63 regulated 1 [Source:HGNC Symbol;Acc:HGNC:24759]	3
ENSG00000186130	4,075708	-0,715715	0,002456	0,014455	-3,54606	protein_coding	ZBTB6	zinc finger and BTB domain containing 6 [Source:HGNC Symbol;Acc:HGNC:16764]	9
ENSG00000068400	6,859852	0,679725	0,002455	0,014455	3,546269	protein_coding	GRIPAP1	GRIP1 associated protein 1 [Source:HGNC Symbol;Acc:HGNC:18706]	X
ENSG00000188785	4,195928	0,676427	0,002459	0,014464	3,545592	protein_coding	ZNF548	zinc finger protein 548 [Source:HGNC Symbol;Acc:HGNC:26561]	19
ENSG00000120832	3,611231	0,828162	0,002465	0,014496	3,544375	protein_coding	MTERF2	mitochondrial transcription termination factor 2 [Source:HGNC Symbol;Acc:HGNC:30779]	12
ENSG00000170619	4,017721	-0,521762	0,002466	0,014496	-3,544267	protein_coding	COMMD5	COMM domain containing 5 [Source:HGNC Symbol;Acc:HGNC:17902]	8
ENSG00000166471	5,027043	-0,582743	0,00247	0,014515	-3,543501	protein_coding	TMEM41B	transmembrane protein 41B [Source:HGNC Symbol;Acc:HGNC:28948]	11
ENSG00000067365	4,255914	0,57506	0,002472	0,014523	3,54308	protein_coding	METTL22	methyltransferase like 22 [Source:HGNC Symbol;Acc:HGNC:28368]	16
ENSG00000083097	5,628186	0,727144	0,002476	0,01454	3,542368	protein_coding	DOP1A	DOP1 leucine zipper like protein A [Source:HGNC Symbol;Acc:HGNC:21194]	6
ENSG00000121743	-0,750755	-2,183409	0,002482	0,01457	-3,541265	protein_coding	GJA3	gap junction protein alpha 3 [Source:HGNC Symbol;Acc:HGNC:4277]	13
ENSG00000100320	7,141509	-0,773502	0,002484	0,014576	-3,540902	protein_coding	RBFOX2	RNA binding fox-1 homolog 2 [Source:HGNC Symbol;Acc:HGNC:9906]	22
ENSG00000180509	-0,196582	2,090203	0,00249	0,014606	3,539801	protein_coding	KCNE1	potassium voltage-gated channel subfamily E regulatory subunit 1 [Source:HGNC Symbol;Acc:HGNC:6240]	21
ENSG00000135924	5,821805	0,740797	0,002498	0,014649	3,538277	protein_coding	DNAJB2	DnaJ heat shock protein family (Hsp40) member B2 [Source:HGNC Symbol;Acc:HGNC:5228]	2
ENSG00000134146	2,927991	1,096173	0,002499	0,014651	3,538049	protein_coding	DPH6	diphthamine biosynthesis 6 [Source:HGNC Symbol;Acc:HGNC:30543]	15
ENSG00000136286	2,29105	2,211672	0,002503	0,014666	3,53729	protein_coding	MYO1G	myosin IG [Source:HGNC Symbol;Acc:HGNC:13880]	7
ENSG00000123240	7,128857	-0,741455	0,002504	0,014666	-3,537233	protein_coding	OPTN	optineurin [Source:HGNC Symbol;Acc:HGNC:17142]	10
ENSG00000112308	6,676487	-0,67745	0,002505	0,014667	-3,537064	protein_coding	C6orf62	chromosome 6 open reading frame 62 [Source:HGNC Symbol;Acc:HGNC:20998]	6
ENSG00000154099	-0,241108	1,845466	0,002509	0,014686	3,536291	protein_coding	DNAAF1	dynein axonemal assembly factor 1 [Source:HGNC Symbol;Acc:HGNC:30539]	16
ENSG00000133398	4,706994	-0,680046	0,00251	0,014687	-3,536101	protein_coding	MED10	mediator complex subunit 10 [Source:HGNC Symbol;Acc:HGNC:28760]	5
ENSG00000237441	5,180389	0,711163	0,002514	0,014699	3,535392	protein_coding	RGL2	ral guanine nucleotide dissociation stimulator like 2 [Source:HGNC Symbol;Acc:HGNC:9769]	6
ENSG00000139697	7,180979	-0,519768	0,002514	0,014699	-3,535413	protein_coding	SBNO1	strawberry notch homolog 1 [Source:HGNC Symbol;Acc:HGNC:22973]	12
ENSG00000143155	5,491808	-0,810793	0,002518	0,014717	-3,534649	protein_coding	TIPRL	TOR signaling pathway regulator [Source:HGNC Symbol;Acc:HGNC:30231]	1
ENSG00000023041	4,854571	-0,550923	0,002525	0,014754	-3,533347	protein_coding	ZDHHC6	zinc finger DHHC-type palmitoyltransferase 6 [Source:HGNC Symbol;Acc:HGNC:19160]	10
ENSG00000163754	5,230857	-0,57828	0,002529	0,01477	-3,532662	protein_coding	GYG1	glycogenin 1 [Source:HGNC Symbol;Acc:HGNC:4699]	3
ENSG00000197771	5,733048	-0,698599	0,00253	0,014771	-3,532493	protein_coding	MCMBP	minichromosome maintenance complex binding protein [Source:HGNC Symbol;Acc:HGNC:25782]	10
ENSG00000065978	9,044918	-1,24581	0,002532	0,014781	-3,531992	protein_coding	YBX1	Y-box binding protein 1 [Source:HGNC Symbol;Acc:HGNC:8014]	1
ENSG00000089597	8,224004	-0,362106	0,002536	0,014797	-3,531343	protein_coding	GANAB	glucosidase II alpha subunit [Source:HGNC Symbol;Acc:HGNC:4138]	11
ENSG00000124214	7,351212	-0,51276	0,002539	0,014811	-3,530736	protein_coding	STAU1	staufen double-stranded RNA binding protein 1 [Source:HGNC Symbol;Acc:HGNC:11370]	20
ENSG00000112394	2,433511	3,069827	0,002543	0,014818	3,530074	protein_coding	SLC16A10	solute carrier family 16 member 10 [Source:HGNC Symbol;Acc:HGNC:17027]	6
ENSG00000077943	3,133873	-2,859245	0,002542	0,014818	-3,530203	protein_coding	ITGA8	integrin subunit alpha 8 [Source:HGNC Symbol;Acc:HGNC:6144]	10
ENSG00000175155	5,222762	0,999797	0,002543	0,014818	3,530033	protein_coding	YPEL2	yippee like 2 [Source:HGNC Symbol;Acc:HGNC:18326]	17
ENSG00000228198	-2,865561	3,365592	0,002548	0,01484	3,529201	protein_coding	OR2M3	olfactory receptor family 2 subfamily M member 3 [Source:HGNC Symbol;Acc:HGNC:8269]	1
ENSG00000185658	7,16721	0,69055	0,002553	0,014866	3,528217	protein_coding	BRWD1	bromodomain and WD repeat domain containing 1 [Source:HGNC Symbol;Acc:HGNC:12760]	21
ENSG00000136143	5,393394	-0,510682	0,002557	0,014881	-3,527578	protein_coding	SUCLA2	succinate-CoA ligase ADP-forming subunit beta [Source:HGNC Symbol;Acc:HGNC:11448]	13
ENSG00000163378	4,335148	-0,718317	0,002559	0,014886	-3,527281	protein_coding	EOGT	EGF domain specific O-linked N-acetylglucosamine transferase [Source:HGNC Symbol;Acc:HGNC:28526]	3
ENSG00000176678	2,703123	-3,596343	0,002566	0,014916	-3,526016	protein_coding	FOXL1	forkhead box L1 [Source:HGNC Symbol;Acc:HGNC:3817]	16
ENSG00000013275	6,821869	-0,702186	0,002565	0,014916	-3,526099	protein_coding	PSMC4	proteasome 26S subunit, ATPase 4 [Source:HGNC Symbol;Acc:HGNC:9551]	19
ENSG00000127472	0,226013	3,020767	0,002569	0,014929	3,525464	protein_coding	PLA2G5	phospholipase A2 group V [Source:HGNC Symbol;Acc:HGNC:9038]	1
ENSG00000204323	0,972716	2,934707	0,00259	0,015045	3,521741	protein_coding	SMIM5	small integral membrane protein 5 [Source:HGNC Symbol;Acc:HGNC:40030]	17
ENSG00000278023	-0,664913	2,490391	0,002593	0,015061	3,521086	protein_coding	RDM1	RAD52 motif containing 1 [Source:HGNC Symbol;Acc:HGNC:19950]	17
ENSG00000198673	1,464487	2,288351	0,0026	0,015096	3,519846	protein_coding	TAFA2	TAFA chemokine like family member 2 [Source:HGNC Symbol;Acc:HGNC:21589]	12
ENSG00000135945	5,349124	0,672766	0,002602	0,015102	3,519516	protein_coding	REV1	REV1 DNA directed polymerase [Source:HGNC Symbol;Acc:HGNC:14060]	2
ENSG00000187889	0,53954	2,914456	0,002604	0,015109	3,519113	protein_coding	FYB2	FYN binding protein 2 [Source:HGNC Symbol;Acc:HGNC:27295]	1
ENSG00000159450	2,364751	3,07392	0,002605	0,015109	3,518975	protein_coding	TCHH	trichohyalin [Source:HGNC Symbol;Acc:HGNC:11791]	1
ENSG00000165997	5,415505	-0,781419	0,00261	0,015129	-3,518192	protein_coding	ARL5B	ADP ribosylation factor like GTPase 5B [Source:HGNC Symbol;Acc:HGNC:23052]	10
ENSG00000205155	2,619658	-0,887708	0,002611	0,015134	-3,517883	protein_coding	PSENEN	presenilin enhancer, gamma-secretase subunit [Source:HGNC Symbol;Acc:HGNC:30100]	19

ENSG00000196912	4,677223	1,732052	0,002615	0,015148	3,51727	protein_coding	ANKRD36B	ankyrin repeat domain 36B [Source:HGNC Symbol;Acc:HGNC:29333]	2
ENSG00000093144	5,277768	-0,579171	0,00262	0,01517	-3,516447	protein_coding	ECHDC1	ethylmalonyl-CoA decarboxylase 1 [Source:HGNC Symbol;Acc:HGNC:21489]	6
ENSG00000163870	4,521528	-0,514612	0,002621	0,015172	-3,516229	protein_coding	TPRA1	transmembrane protein adipocyte associated 1 [Source:HGNC Symbol;Acc:HGNC:30413]	3
ENSG00000099365	0,881032	1,673292	0,002623	0,015173	3,515883	protein_coding	STX1B	syntaxin 1B [Source:HGNC Symbol;Acc:HGNC:18539]	16
ENSG00000175595	4,185676	-0,608143	0,002623	0,015173	-3,515868	protein_coding	ERCC4	ERCC excision repair 4, endonuclease catalytic subunit [Source:HGNC Symbol;Acc:HGNC:3436]	16
ENSG00000163576	-0,781782	2,197055	0,002625	0,015174	3,515504	protein_coding	EFHB	EF-hand domain family member B [Source:HGNC Symbol;Acc:HGNC:26330]	3
ENSG00000143727	5,907022	-0,530774	0,002625	0,015174	-3,515552	protein_coding	ACP1	acid phosphatase 1 [Source:HGNC Symbol;Acc:HGNC:122]	2
ENSG00000166341	4,812715	2,897491	0,002628	0,015187	3,514969	protein_coding	DCHS1	dachsous cadherin-related 1 [Source:HGNC Symbol;Acc:HGNC:13681]	11
ENSG00000114302	6,513187	-0,631678	0,002632	0,015202	-3,514329	protein_coding	PRKAR2A	protein kinase cAMP-dependent type II regulatory subunit alpha [Source:HGNC Symbol;Acc:HGNC:9391]	3
ENSG00000074966	-0,225079	1,96969	0,002633	0,015206	3,514061	protein_coding	TXK	TXK tyrosine kinase [Source:HGNC Symbol;Acc:HGNC:12434]	4
ENSG00000129625	6,723206	-0,877772	0,002641	0,015247	-3,512661	protein_coding	REEP5	receptor accessory protein 5 [Source:HGNC Symbol;Acc:HGNC:30077]	5
ENSG00000136051	6,373456	-0,657865	0,002643	0,015252	-3,512333	protein_coding	WASHC4	WASH complex subunit 4 [Source:HGNC Symbol;Acc:HGNC:29174]	12
ENSG00000205838	-0,077251	2,093982	0,002646	0,015262	3,51189	protein_coding	TTC23L	tetratricopeptide repeat domain 23 like [Source:HGNC Symbol;Acc:HGNC:26355]	5
ENSG00000113194	6,392673	-0,491121	0,002647	0,015266	-3,511585	protein_coding	FAF2	Fas associated factor family member 2 [Source:HGNC Symbol;Acc:HGNC:24666]	5
ENSG00000124212	3,739596	4,089222	0,002657	0,01531	3,509933	protein_coding	PTGIS	prostaglandin I2 synthase [Source:HGNC Symbol;Acc:HGNC:9603]	20
ENSG00000143164	6,278558	-0,721301	0,002656	0,01531	-3,510033	protein_coding	DCAF6	DDB1 and CUL4 associated factor 6 [Source:HGNC Symbol;Acc:HGNC:30002]	1
ENSG00000165409	-2,125536	2,833619	0,002658	0,015311	3,50974	protein_coding	TSHR	thyroid stimulating hormone receptor [Source:HGNC Symbol;Acc:HGNC:12373]	14
ENSG00000134697	6,087861	-0,64712	0,002677	0,015413	-3,506528	protein_coding	GNL2	G protein nucleolar 2 [Source:HGNC Symbol;Acc:HGNC:29925]	1
ENSG00000161267	4,283312	1,346565	0,002685	0,015457	3,50506	protein_coding	BDH1	3-hydroxybutyrate dehydrogenase 1 [Source:HGNC Symbol;Acc:HGNC:1027]	3
ENSG00000284526	0,456676	-1,34298	0,002691	0,015483	-3,504013	protein_coding	AC015802.	novel protein	17
ENSG00000136011	-2,043791	3,813415	0,002692	0,015483	3,503975	protein_coding	STAB2	stabilin 2 [Source:HGNC Symbol;Acc:HGNC:18629]	12
ENSG00000007314	-1,881262	3,227163	0,002693	0,015483	3,503798	protein_coding	SCN4A	sodium voltage-gated channel alpha subunit 4 [Source:HGNC Symbol;Acc:HGNC:10591]	17
ENSG00000042753	5,051841	-0,615737	0,002695	0,015491	-3,503414	protein_coding	AP2S1	adaptor related protein complex 2 subunit sigma 1 [Source:HGNC Symbol;Acc:HGNC:565]	19
ENSG00000142606	-1,606061	1,887608	0,002698	0,015491	3,502925	protein_coding	MMEL1	membrane metalloendopeptidase like 1 [Source:HGNC Symbol;Acc:HGNC:14668]	1
ENSG00000153107	5,010394	-0,796676	0,002697	0,015491	-3,503033	protein_coding	ANAPC1	anaphase promoting complex subunit 1 [Source:HGNC Symbol;Acc:HGNC:19988]	2
ENSG00000130787	6,457935	1,182196	0,002697	0,015491	3,503055	protein_coding	HIP1R	huntingtin interacting protein 1 related [Source:HGNC Symbol;Acc:HGNC:18415]	12
ENSG00000224383	-0,661184	3,098384	0,002708	0,01554	3,501153	protein_coding	PRR29	proline rich 29 [Source:HGNC Symbol;Acc:HGNC:25673]	17
ENSG00000077942	3,281863	3,236506	0,002708	0,01554	3,501203	protein_coding	FBLN1	fibulin 1 [Source:HGNC Symbol;Acc:HGNC:3600]	22
ENSG00000162704	6,999085	-0,694291	0,002711	0,015549	-3,500706	protein_coding	ARPC5	actin related protein 2/3 complex subunit 5 [Source:HGNC Symbol;Acc:HGNC:708]	1
ENSG00000086504	4,614611	-0,631902	0,002714	0,015564	-3,5001	protein_coding	MRPL28	mitochondrial ribosomal protein L28 [Source:HGNC Symbol;Acc:HGNC:14484]	16
ENSG00000013503	4,387329	-0,642806	0,002715	0,015565	-3,499919	protein_coding	POLR3B	RNA polymerase III subunit B [Source:HGNC Symbol;Acc:HGNC:30348]	12
ENSG00000214954	0,065279	1,337568	0,002721	0,015567	3,498918	protein_coding	LRRC69	leucine rich repeat containing 69 [Source:HGNC Symbol;Acc:HGNC:34303]	8
ENSG00000169413	0,897536	2,634405	0,002721	0,015567	3,499009	protein_coding	RNASE6	ribonuclease A family member k6 [Source:HGNC Symbol;Acc:HGNC:10048]	14
ENSG00000134245	1,804038	2,462988	0,002719	0,015567	3,499308	protein_coding	WNT2B	Wnt family member 2B [Source:HGNC Symbol;Acc:HGNC:12781]	1
ENSG00000169750	1,873952	1,597142	0,002717	0,015567	3,499632	protein_coding	RAC3	Rac family small GTPase 3 [Source:HGNC Symbol;Acc:HGNC:9803]	17
ENSG00000216937	3,52645	1,074656	0,002721	0,015567	3,499029	protein_coding	CCDC7	coiled-coil domain containing 7 [Source:HGNC Symbol;Acc:HGNC:26533]	10
ENSG00000144589	4,131264	0,765902	0,002722	0,015567	3,498875	protein_coding	STK11IP	serine/threonine kinase 11 interacting protein [Source:HGNC Symbol;Acc:HGNC:19184]	2
ENSG00000270106	-2,425535	2,252421	0,002725	0,015575	3,498334	protein_coding	TSNAX-DIS	TSNAX-DISC1 readthrough (NMD candidate) [Source:HGNC Symbol;Acc:HGNC:49177]	1
ENSG00000145782	5,658244	-0,549625	0,002724	0,015575	-3,498432	protein_coding	ATG12	autophagy related 12 [Source:HGNC Symbol;Acc:HGNC:588]	5
ENSG00000229474	0,920773	1,76404	0,002728	0,01559	3,497728	protein_coding	PATL2	PAT1 homolog 2 [Source:HGNC Symbol;Acc:HGNC:33630]	15
ENSG00000144229	-0,458895	3,937955	0,002732	0,015607	3,497064	protein_coding	THSD7B	thrombospondin type 1 domain containing 7B [Source:HGNC Symbol;Acc:HGNC:29348]	2
ENSG00000144559	3,430107	0,948444	0,002737	0,015624	3,496233	protein_coding	TAMM41	TAM41 mitochondrial translocator assembly and maintenance homolog [Source:HGNC Symbol;Acc:HGNC:25187]	3
ENSG00000105137	4,53287	-1,420919	0,002736	0,015624	-3,49638	protein_coding	SYDE1	synapse defective Rho GTPase homolog 1 [Source:HGNC Symbol;Acc:HGNC:25824]	19
ENSG00000079435	2,582492	2,034178	0,00274	0,015631	3,495857	protein_coding	LIPE	lipase E, hormone sensitive type [Source:HGNC Symbol;Acc:HGNC:6621]	19
ENSG00000198561	4,855403	0,883306	0,002742	0,015639	3,495469	protein_coding	CTNND1	catenin delta 1 [Source:HGNC Symbol;Acc:HGNC:2515]	11
ENSG00000137714	4,311774	-0,668065	0,002751	0,015679	-3,493976	protein_coding	FDX1	ferredoxin 1 [Source:HGNC Symbol;Acc:HGNC:3638]	11
ENSG00000105281	7,185124	-1,728021	0,002751	0,015679	-3,493982	protein_coding	SLC1A5	solute carrier family 1 member 5 [Source:HGNC Symbol;Acc:HGNC:10943]	19
ENSG00000100365	1,438116	2,173097	0,002757	0,015711	3,492886	protein_coding	NCF4	neutrophil cytosolic factor 4 [Source:HGNC Symbol;Acc:HGNC:7662]	22
ENSG00000204370	3,73353	-0,864694	0,002761	0,015718	-3,492283	protein_coding	SDHD	succinate dehydrogenase complex subunit D [Source:HGNC Symbol;Acc:HGNC:10683]	11
ENSG00000049449	5,561033	-1,518623	0,002762	0,015718	-3,492191	protein_coding	RCN1	reticulocalbin 1 [Source:HGNC Symbol;Acc:HGNC:9934]	11
ENSG00000173598	6,615431	-0,702606	0,00276	0,015718	-3,49242	protein_coding	NUDT4	nudix hydrolase 4 [Source:HGNC Symbol;Acc:HGNC:8051]	12

ENSG00000178719	6,824311	-0,653282	0,002764	0,015729	-3,491719	protein_coding	GRINA	glutamate ionotropic receptor NMDA type subunit associated protein 1 [Source:HGNC Symbol;Acc:HGNC:4589]	8
ENSG00000111011	7,094519	0,555459	0,002773	0,015772	3,490293	protein_coding	RSRC2	arginine and serine rich coiled-coil 2 [Source:HGNC Symbol;Acc:HGNC:30559]	12
ENSG00000163931	7,393324	-0,933468	0,002778	0,015794	-3,489504	protein_coding	TKT	transketolase [Source:HGNC Symbol;Acc:HGNC:11834]	3
ENSG00000166436	3,819709	1,518875	0,002781	0,015809	3,488895	protein_coding	TRIM66	tripartite motif containing 66 [Source:HGNC Symbol;Acc:HGNC:29005]	11
ENSG00000141258	6,124394	1,188341	0,002785	0,015824	3,488292	protein_coding	SGSM2	small G protein signaling modulator 2 [Source:HGNC Symbol;Acc:HGNC:29026]	17
ENSG00000172301	5,035439	-0,679192	0,002787	0,01583	-3,487967	protein_coding	COPRS	coordinator of PRMT5 and differentiation stimulator [Source:HGNC Symbol;Acc:HGNC:28848]	17
ENSG00000156603	3,833782	-0,531916	0,002789	0,01583	-3,487683	protein_coding	MED19	mediator complex subunit 19 [Source:HGNC Symbol;Acc:HGNC:29600]	11
ENSG00000100422	6,624317	-0,77101	0,002789	0,01583	-3,487628	protein_coding	CERK	ceramide kinase [Source:HGNC Symbol;Acc:HGNC:19256]	22
ENSG00000060558	1,51326	2,28383	0,002798	0,015869	3,486174	protein_coding	GNA15	G protein subunit alpha 15 [Source:HGNC Symbol;Acc:HGNC:4383]	19
ENSG00000081237	3,920961	2,829504	0,002798	0,015869	3,486202	protein_coding	PTPRC	protein tyrosine phosphatase receptor type C [Source:HGNC Symbol;Acc:HGNC:9666]	1
ENSG00000114013	2,005662	2,357555	0,002799	0,015871	3,485984	protein_coding	CD86	CD86 molecule [Source:HGNC Symbol;Acc:HGNC:1705]	3
ENSG00000053918	0,990465	3,905024	0,002809	0,015916	3,48436	protein_coding	KCNQ1	potassium voltage-gated channel subfamily Q member 1 [Source:HGNC Symbol;Acc:HGNC:6294]	11
ENSG00000095752	2,670891	-4,082654	0,002809	0,015916	-3,484393	protein_coding	IL11	interleukin 11 [Source:HGNC Symbol;Acc:HGNC:5966]	19
ENSG00000104967	1,584274	3,713878	0,002811	0,015921	3,484033	protein_coding	NOVA2	NOVA alternative splicing regulator 2 [Source:HGNC Symbol;Acc:HGNC:7887]	19
ENSG00000129480	2,977044	-0,857628	0,00282	0,01596	-3,482596	protein_coding	DTD2	D-aminoacyl-tRNA deacylase 2 [Source:HGNC Symbol;Acc:HGNC:20277]	14
ENSG00000142798	8,388786	1,349039	0,002819	0,01596	3,482705	protein_coding	HSPG2	heparan sulfate proteoglycan 2 [Source:HGNC Symbol;Acc:HGNC:5273]	1
ENSG00000173915	5,045832	-0,539367	0,002821	0,015963	-3,482357	protein_coding	ATP5MD	ATP synthase membrane subunit DAPIT [Source:HGNC Symbol;Acc:HGNC:30889]	10
ENSG00000118307	-0,435859	2,289001	0,002822	0,015964	3,482156	protein_coding	CFAP94	cilia and flagella associated protein 94 [Source:HGNC Symbol;Acc:HGNC:29599]	12
ENSG00000144451	4,227735	1,056873	0,002825	0,015976	3,481671	protein_coding	SPAG16	sperm associated antigen 16 [Source:HGNC Symbol;Acc:HGNC:23225]	2
ENSG00000127125	5,048697	-0,727424	0,002833	0,01601	-3,480514	protein_coding	PPCS	phosphopantothenoylcysteine synthetase [Source:HGNC Symbol;Acc:HGNC:25686]	1
ENSG00000242028	2,205884	0,989438	0,002839	0,016043	3,479414	protein_coding	HYPK	huntingtin interacting protein K [Source:HGNC Symbol;Acc:HGNC:18418]	15
ENSG00000131873	6,294455	-1,04262	0,002846	0,016077	-3,478286	protein_coding	CHSY1	chondroitin sulfate synthase 1 [Source:HGNC Symbol;Acc:HGNC:17198]	15
ENSG00000168175	7,143377	-0,54301	0,002848	0,016078	-3,478082	protein_coding	MAPK1IP1l	mitogen-activated protein kinase 1 interacting protein 1 like [Source:HGNC Symbol;Acc:HGNC:19840]	14
ENSG00000160593	1,634919	2,437983	0,002853	0,016105	3,477148	protein_coding	JAML	junction adhesion molecule like [Source:HGNC Symbol;Acc:HGNC:19084]	11
ENSG00000184489	4,983425	1,602512	0,002856	0,016117	3,476668	protein_coding	PTP4A3	protein tyrosine phosphatase 4A3 [Source:HGNC Symbol;Acc:HGNC:9636]	8
ENSG00000162928	4,79823	-0,51339	0,002861	0,016135	-3,475956	protein_coding	PEX13	peroxisomal biogenesis factor 13 [Source:HGNC Symbol;Acc:HGNC:8855]	2
ENSG00000170558	5,835334	-2,149434	0,002862	0,016135	-3,475833	protein_coding	CDH2	cadherin 2 [Source:HGNC Symbol;Acc:HGNC:1759]	18
ENSG00000173801	7,408655	-1,030694	0,002865	0,016147	-3,475329	protein_coding	JUP	junction plakoglobin [Source:HGNC Symbol;Acc:HGNC:6207]	17
ENSG00000105825	4,383706	-4,234059	0,002868	0,01616	-3,474788	protein_coding	TFPI2	tissue factor pathway inhibitor 2 [Source:HGNC Symbol;Acc:HGNC:11761]	7
ENSG00000179832	4,968734	0,926385	0,002872	0,016176	3,474186	protein_coding	MROH1	maestro heat like repeat family member 1 [Source:HGNC Symbol;Acc:HGNC:26958]	8
ENSG00000130164	7,340343	-1,132181	0,002874	0,016184	-3,4738	protein_coding	LDLR	low density lipoprotein receptor [Source:HGNC Symbol;Acc:HGNC:6547]	19
ENSG00000110330	5,912741	-0,773654	0,002896	0,016302	-3,470286	protein_coding	BIRC2	baculoviral IAP repeat containing 2 [Source:HGNC Symbol;Acc:HGNC:590]	11
ENSG00000185049	4,529036	0,539101	0,002897	0,016303	3,470113	protein_coding	NELFA	negative elongation factor complex member A [Source:HGNC Symbol;Acc:HGNC:12768]	4
ENSG00000169635	3,88332	0,749917	0,002902	0,016323	3,469375	protein_coding	HIC2	HIC ZBTB transcriptional repressor 2 [Source:HGNC Symbol;Acc:HGNC:18595]	22
ENSG00000080823	4,096281	1,010369	0,002904	0,016326	3,46914	protein_coding	MOK	MOK protein kinase [Source:HGNC Symbol;Acc:HGNC:9833]	14
ENSG00000158423	0,68437	1,789695	0,002908	0,016343	3,468508	protein_coding	RIBC1	RIB43A domain with coiled-coils 1 [Source:HGNC Symbol;Acc:HGNC:26537]	X
ENSG00000076555	5,107199	1,886727	0,00291	0,016353	3,468069	protein_coding	ACACB	acetyl-CoA carboxylase beta [Source:HGNC Symbol;Acc:HGNC:85]	12
ENSG00000188219	1,858045	2,608988	0,002912	0,016356	3,467815	protein_coding	POTEE	POTE ankyrin domain family member E [Source:HGNC Symbol;Acc:HGNC:33895]	2
ENSG00000198948	3,428799	-1,351948	0,002926	0,016431	-3,465548	protein_coding	MFAP3L	microfibril associated protein 3 like [Source:HGNC Symbol;Acc:HGNC:29083]	4
ENSG00000143297	-2,588535	3,798166	0,00293	0,016434	3,464984	protein_coding	FCRL5	Fc receptor like 5 [Source:HGNC Symbol;Acc:HGNC:18508]	1
ENSG00000168918	4,029775	2,443957	0,00293	0,016434	3,465007	protein_coding	INPP5D	inositol polyphosphate-5-phosphatase D [Source:HGNC Symbol;Acc:HGNC:6079]	2
ENSG00000068438	5,212697	-0,58863	0,00293	0,016434	-3,465003	protein_coding	FTSJ1	FtsJ RNA 2'-O-methyltransferase 1 [Source:HGNC Symbol;Acc:HGNC:13254]	X
ENSG00000007392	5,663202	0,839173	0,002931	0,016434	3,464852	protein_coding	LUC7L	LUC7 like [Source:HGNC Symbol;Acc:HGNC:6723]	16
ENSG00000147509	-1,188297	-1,8952	0,002936	0,016456	-3,464039	protein_coding	RGS20	regulator of G protein signaling 20 [Source:HGNC Symbol;Acc:HGNC:14600]	8
ENSG00000149090	2,951681	-2,900055	0,002937	0,016456	-3,463892	protein_coding	PAMR1	peptidase domain containing associated with muscle regeneration 1 [Source:HGNC Symbol;Acc:HGNC:24554]	11
ENSG00000177646	5,727967	0,730854	0,002938	0,016457	3,463716	protein_coding	ACAD9	acyl-CoA dehydrogenase family member 9 [Source:HGNC Symbol;Acc:HGNC:21497]	3
ENSG00000182329	-2,319959	3,293285	0,002947	0,016497	3,462312	protein_coding	KIAA2012	KIAA2012 [Source:HGNC Symbol;Acc:HGNC:51250]	2
ENSG00000086712	5,09356	0,571404	0,002947	0,016497	3,462296	protein_coding	TXLNG	taxilin gamma [Source:HGNC Symbol;Acc:HGNC:18578]	X
ENSG00000166704	3,61495	1,337467	0,002949	0,0165	3,462054	protein_coding	ZNF606	zinc finger protein 606 [Source:HGNC Symbol;Acc:HGNC:25879]	19
ENSG00000171813	4,101322	0,704492	0,002951	0,016507	3,461677	protein_coding	PWWP2B	PWWP domain containing 2B [Source:HGNC Symbol;Acc:HGNC:25150]	10
ENSG00000074356	6,289951	0,66661	0,002952	0,016507	3,461535	protein_coding	NCBP3	nuclear cap binding subunit 3 [Source:HGNC Symbol;Acc:HGNC:24612]	17

ENSG00000181513	3,184903	1,651984	0,002956	0,016521	3,460976	protein_coding	ACBD4	acyl-CoA binding domain containing 4 [Source:HGNC Symbol;Acc:HGNC:23337]	17
ENSG00000011021	4,710293	0,475183	0,00296	0,016539	3,460321	protein_coding	CLCN6	chloride voltage-gated channel 6 [Source:HGNC Symbol;Acc:HGNC:2024]	1
ENSG00000077684	5,616774	0,896721	0,002963	0,016553	3,459774	protein_coding	JADE1	jade family PHD finger 1 [Source:HGNC Symbol;Acc:HGNC:30027]	4
ENSG00000217930	1,285442	1,2755	0,002965	0,016554	3,459543	protein_coding	PAM16	presequence translocase associated motor 16 [Source:HGNC Symbol;Acc:HGNC:29679]	16
ENSG00000204859	3,932683	0,961908	0,002966	0,016554	3,459401	protein_coding	ZBTB48	zinc finger and BTB domain containing 48 [Source:HGNC Symbol;Acc:HGNC:4930]	1
ENSG00000198089	5,354124	1,023247	0,002967	0,016554	3,459269	protein_coding	SFI1	SFI1 centrin binding protein [Source:HGNC Symbol;Acc:HGNC:29064]	22
ENSG00000151687	2,497252	1,134543	0,002972	0,016574	3,458417	protein_coding	ANKAR	ankyrin and armadillo repeat containing [Source:HGNC Symbol;Acc:HGNC:26350]	2
ENSG00000119682	6,16324	-0,456437	0,002972	0,016574	-3,458457	protein_coding	AREL1	apoptosis resistant E3 ubiquitin protein ligase 1 [Source:HGNC Symbol;Acc:HGNC:20363]	14
ENSG00000135778	4,769613	0,768752	0,002982	0,016623	3,456901	protein_coding	NTPCR	nucleoside-triphosphatase, cancer-related [Source:HGNC Symbol;Acc:HGNC:28204]	1
ENSG00000103522	2,350173	-2,479901	0,002985	0,016633	-3,456457	protein_coding	IL21R	interleukin 21 receptor [Source:HGNC Symbol;Acc:HGNC:6006]	16
ENSG00000106125	1,520654	1,300872	0,002993	0,016665	3,455141	protein_coding	MINDY4	MINDY lysine 48 deubiquitinase 4 [Source:HGNC Symbol;Acc:HGNC:21916]	7
ENSG00000144061	3,068493	1,031074	0,002993	0,016665	3,455109	protein_coding	NPHP1	nephrocystin 1 [Source:HGNC Symbol;Acc:HGNC:7905]	2
ENSG00000128283	5,330213	-1,516954	0,002992	0,016665	-3,455286	protein_coding	CDC42EP1	CDC42 effector protein 1 [Source:HGNC Symbol;Acc:HGNC:17014]	22
ENSG00000181904	5,935224	-0,518707	0,002999	0,016685	-3,45423	protein_coding	C5orf24	chromosome 5 open reading frame 24 [Source:HGNC Symbol;Acc:HGNC:26746]	5
ENSG00000113580	6,64338	-0,996156	0,002999	0,016685	-3,454258	protein_coding	NR3C1	nuclear receptor subfamily 3 group C member 1 [Source:HGNC Symbol;Acc:HGNC:7978]	5
ENSG00000099341	7,188579	-0,801767	0,003006	0,016718	-3,453162	protein_coding	PSMD8	proteasome 26S subunit, non-ATPase 8 [Source:HGNC Symbol;Acc:HGNC:9566]	19
ENSG00000166343	0,81877	1,259226	0,00301	0,016735	3,452541	protein_coding	MSS51	MSS51 mitochondrial translational activator [Source:HGNC Symbol;Acc:HGNC:21000]	10
ENSG00000112977	7,49523	-0,821613	0,003012	0,01674	-3,45226	protein_coding	DAP	death associated protein [Source:HGNC Symbol;Acc:HGNC:2672]	5
ENSG00000163013	4,792615	1,29164	0,003018	0,016764	3,451422	protein_coding	FBXO41	F-box protein 41 [Source:HGNC Symbol;Acc:HGNC:29409]	2
ENSG00000254469	2,62858	1,375261	0,003046	0,016916	3,447109	protein_coding	AP002495.	XRCC1 N-terminal domain containing 1-like [Source:NCBI gene (formerly Entrezgene);Acc:100133315]	11
ENSG00000172014	-1,058195	2,513038	0,00305	0,016926	3,446535	protein_coding	ANKRD20A	ankyrin repeat domain 20 family member A4, pseudogene [Source:HGNC Symbol;Acc:HGNC:31982]	9
ENSG00000163751	-2,845181	2,813753	0,00305	0,016926	3,44653	protein_coding	CPA3	carboxypeptidase A3 [Source:HGNC Symbol;Acc:HGNC:2298]	3
ENSG00000183808	5,727319	0,652066	0,003052	0,016934	3,44615	protein_coding	RBM12B	RNA binding motif protein 12B [Source:HGNC Symbol;Acc:HGNC:32310]	8
ENSG00000070540	5,734251	-1,196734	0,003056	0,016948	-3,445627	protein_coding	WIPI1	WD repeat domain, phosphoinositide interacting 1 [Source:HGNC Symbol;Acc:HGNC:25471]	17
ENSG00000187187	2,560003	1,197436	0,003058	0,016954	3,445293	protein_coding	ZNF546	zinc finger protein 546 [Source:HGNC Symbol;Acc:HGNC:28671]	19
ENSG00000126759	-0,791625	1,466656	0,003064	0,016957	3,444442	protein_coding	CFP	complement factor properdin [Source:HGNC Symbol;Acc:HGNC:8864]	X
ENSG00000143110	1,816655	2,17891	0,003062	0,016957	3,444713	protein_coding	C1orf162	chromosome 1 open reading frame 162 [Source:HGNC Symbol;Acc:HGNC:28344]	1
ENSG00000100304	5,443987	-0,802674	0,003064	0,016957	-3,444426	protein_coding	TTLL12	tubulin tyrosine ligase like 12 [Source:HGNC Symbol;Acc:HGNC:28974]	22
ENSG00000188559	6,935592	0,978011	0,003061	0,016957	3,444789	protein_coding	RALGAPA2	Ral GTPase activating protein catalytic subunit alpha 2 [Source:HGNC Symbol;Acc:HGNC:16207]	20
ENSG00000075415	7,460851	-0,640866	0,00306	0,016957	-3,445041	protein_coding	SLC25A3	solute carrier family 25 member 3 [Source:HGNC Symbol;Acc:HGNC:10989]	12
ENSG00000185823	-1,38498	3,887038	0,003068	0,016975	3,443743	protein_coding	NPAP1	nuclear pore associated protein 1 [Source:HGNC Symbol;Acc:HGNC:1190]	15
ENSG00000162385	4,068118	-0,632184	0,00307	0,016975	-3,443493	protein_coding	MAGOH	mago homolog, exon junction complex subunit [Source:HGNC Symbol;Acc:HGNC:6815]	1
ENSG00000138246	6,884284	-0,681913	0,00307	0,016975	-3,443535	protein_coding	DNAJC13	DnaJ heat shock protein family (Hsp40) member C13 [Source:HGNC Symbol;Acc:HGNC:30343]	3
ENSG00000135047	7,411938	-1,345962	0,003075	0,016997	-3,442732	protein_coding	CTSL	cathepsin L [Source:HGNC Symbol;Acc:HGNC:2537]	9
ENSG00000122728	-1,195102	1,654228	0,003082	0,017025	3,44175	protein_coding	TAF1L	TATA-box binding protein associated factor 1 like [Source:HGNC Symbol;Acc:HGNC:18056]	9
ENSG00000189045	0,212912	1,755392	0,003082	0,017025	3,441645	protein_coding	ANKDD1B	ankyrin repeat and death domain containing 1B [Source:HGNC Symbol;Acc:HGNC:32525]	5
ENSG00000196155	3,912574	1,520264	0,00309	0,017057	3,440571	protein_coding	PLEKHG4	pleckstrin homology and RhoGEF domain containing G4 [Source:HGNC Symbol;Acc:HGNC:24501]	16
ENSG00000153250	5,764489	-1,236357	0,00309	0,017057	-3,440486	protein_coding	RBMS1	RNA binding motif single stranded interacting protein 1 [Source:HGNC Symbol;Acc:HGNC:9907]	2
ENSG00000007312	-2,110313	3,150596	0,0031	0,017105	3,439037	protein_coding	CD79B	CD79b molecule [Source:HGNC Symbol;Acc:HGNC:1699]	17
ENSG00000115257	0,780415	2,508745	0,003102	0,01711	3,438741	protein_coding	PCSK4	proprotein convertase subtilisin/kexin type 4 [Source:HGNC Symbol;Acc:HGNC:8746]	19
ENSG00000152518	6,080386	1,507363	0,003103	0,017111	3,43856	protein_coding	ZFP36L2	ZFP36 ring finger protein like 2 [Source:HGNC Symbol;Acc:HGNC:1108]	2
ENSG00000197467	3,430484	-2,641258	0,003104	0,017112	-3,438371	protein_coding	COL13A1	collagen type XIII alpha 1 chain [Source:HGNC Symbol;Acc:HGNC:2190]	10
ENSG00000163239	-1,763063	2,638913	0,003113	0,01713	3,437091	protein_coding	TDRD10	tudor domain containing 10 [Source:HGNC Symbol;Acc:HGNC:25316]	1
ENSG00000129467	2,282965	2,373655	0,003114	0,01713	3,436956	protein_coding	ADCY4	adenylate cyclase 4 [Source:HGNC Symbol;Acc:HGNC:235]	14
ENSG00000107821	2,732381	-1,322346	0,003112	0,01713	-3,437315	protein_coding	KAZALD1	Kazal type serine peptidase inhibitor domain 1 [Source:HGNC Symbol;Acc:HGNC:25460]	10
ENSG00000175854	3,921218	-0,620618	0,003114	0,01713	-3,437018	protein_coding	SWI5	SWI5 homologous recombination repair protein [Source:HGNC Symbol;Acc:HGNC:31412]	9
ENSG00000171723	4,875032	0,724007	0,003111	0,01713	3,437423	protein_coding	GPHN	gephyrin [Source:HGNC Symbol;Acc:HGNC:15465]	14
ENSG00000144746	5,317414	-0,945437	0,003112	0,01713	-3,437291	protein_coding	ARL6IP5	ADP ribosylation factor like GTPase 6 interacting protein 5 [Source:HGNC Symbol;Acc:HGNC:16937]	3
ENSG00000198502	2,419453	3,910583	0,003117	0,017133	3,436544	protein_coding	HLA-DRB5	major histocompatibility complex, class II, DR beta 5 [Source:HGNC Symbol;Acc:HGNC:4953]	6
ENSG00000088367	6,01334	0,648736	0,003118	0,017133	3,436415	protein_coding	EPB41L1	erythrocyte membrane protein band 4.1 like 1 [Source:HGNC Symbol;Acc:HGNC:3378]	20
ENSG00000159461	6,461477	-0,622777	0,003118	0,017133	-3,436406	protein_coding	AMFR	autocrine motility factor receptor [Source:HGNC Symbol;Acc:HGNC:463]	16

ENSG00000122873	4,059159	-0,836046	0,003119	0,017134	-3,436226	protein_coding	CISD1	CDGSH iron sulfur domain 1 [Source:HGNC Symbol;Acc:HGNC:30880]	10
ENSG00000055950	4,757046	-0,41963	0,003122	0,017143	-3,435835	protein_coding	MRPL43	mitochondrial ribosomal protein L43 [Source:HGNC Symbol;Acc:HGNC:14517]	10
ENSG00000102125	4,134136	0,93205	0,003124	0,017149	3,435514	protein_coding	TAZ	tafazzin [Source:HGNC Symbol;Acc:HGNC:11577]	X
ENSG00000198838	-1,067606	2,966001	0,003126	0,017153	3,435248	protein_coding	RYR3	ryanodine receptor 3 [Source:HGNC Symbol;Acc:HGNC:10485]	15
ENSG00000172824	2,080479	1,947133	0,003129	0,017168	3,434695	protein_coding	CES4A	carboxylesterase 4A [Source:HGNC Symbol;Acc:HGNC:26741]	16
ENSG00000132359	6,469372	1,672579	0,003135	0,017195	3,433802	protein_coding	RAP1GAP2	RAP1 GTPase activating protein 2 [Source:HGNC Symbol;Acc:HGNC:29176]	17
ENSG00000154258	3,349624	1,989283	0,003138	0,017202	3,433458	protein_coding	ABCA9	ATP binding cassette subfamily A member 9 [Source:HGNC Symbol;Acc:HGNC:39]	17
ENSG00000163945	5,330054	1,002962	0,003141	0,017206	3,433049	protein_coding	UVSSA	UV stimulated scaffold protein A [Source:HGNC Symbol;Acc:HGNC:29304]	4
ENSG00000134318	7,398705	-0,714658	0,00314	0,017206	-3,433121	protein_coding	ROCK2	Rho associated coiled-coil containing protein kinase 2 [Source:HGNC Symbol;Acc:HGNC:10252]	2
ENSG00000055483	6,635905	0,527566	0,003148	0,01724	3,431999	protein_coding	USP36	ubiquitin specific peptidase 36 [Source:HGNC Symbol;Acc:HGNC:20062]	17
ENSG00000143674	4,102374	1,125361	0,003151	0,017254	3,431461	protein_coding	MAP3K21	mitogen-activated protein kinase kinase kinase 21 [Source:HGNC Symbol;Acc:HGNC:29798]	1
ENSG00000008516	-0,298509	2,271332	0,003153	0,017259	3,431174	protein_coding	MMP25	matrix metallopeptidase 25 [Source:HGNC Symbol;Acc:HGNC:14246]	16
ENSG00000106686	2,883825	2,08547	0,003157	0,017271	3,430704	protein_coding	SPATA6L	spermatogenesis associated 6 like [Source:HGNC Symbol;Acc:HGNC:25472]	9
ENSG00000186810	-2,083044	2,36544	0,003159	0,017279	3,430341	protein_coding	CXCR3	C-X-C motif chemokine receptor 3 [Source:HGNC Symbol;Acc:HGNC:4540]	X
ENSG00000124610	0,932789	-2,410716	0,003161	0,017284	-3,430047	protein_coding	H1-1	H1.1 linker histone, cluster member [Source:HGNC Symbol;Acc:HGNC:4715]	6
ENSG00000141837	5,709345	2,899576	0,003163	0,017287	3,42981	protein_coding	CACNA1A	calcium voltage-gated channel subunit alpha1 A [Source:HGNC Symbol;Acc:HGNC:1388]	19
ENSG00000058262	8,329023	-0,660566	0,003164	0,017287	-3,429664	protein_coding	SEC61A1	SEC61 translocon subunit alpha 1 [Source:HGNC Symbol;Acc:HGNC:18276]	3
ENSG00000185085	4,07215	-0,506449	0,003169	0,017307	-3,428832	protein_coding	INTS5	integrator complex subunit 5 [Source:HGNC Symbol;Acc:HGNC:29352]	11
ENSG00000184634	6,579396	0,504207	0,00317	0,017307	3,428813	protein_coding	MED12	mediator complex subunit 12 [Source:HGNC Symbol;Acc:HGNC:11957]	X
ENSG00000108984	4,670589	1,590672	0,003172	0,017315	3,428454	protein_coding	MAP2K6	mitogen-activated protein kinase kinase 6 [Source:HGNC Symbol;Acc:HGNC:6846]	17
ENSG00000104237	-1,493939	2,795687	0,003174	0,017319	3,428185	protein_coding	RP1	RP1 axonemal microtubule associated [Source:HGNC Symbol;Acc:HGNC:10263]	8
ENSG00000104356	3,59546	-0,723724	0,003176	0,017325	-3,427875	protein_coding	POP1	POP1 homolog, ribonuclease P/MRP subunit [Source:HGNC Symbol;Acc:HGNC:30129]	8
ENSG00000148229	5,389937	-0,547963	0,003178	0,017329	-3,427597	protein_coding	POLE3	DNA polymerase epsilon 3, accessory subunit [Source:HGNC Symbol;Acc:HGNC:13546]	9
ENSG00000090104	4,028891	3,114533	0,003182	0,017346	3,42701	protein_coding	RGS1	regulator of G protein signaling 1 [Source:HGNC Symbol;Acc:HGNC:9991]	1
ENSG00000175267	-2,001365	2,756978	0,003187	0,017349	3,426307	protein_coding	VWA3A	von Willebrand factor A domain containing 3A [Source:HGNC Symbol;Acc:HGNC:27088]	16
ENSG00000221946	-2,45976	2,440921	0,003186	0,017349	3,426383	protein_coding	FXYD7	FXYD domain containing ion transport regulator 7 [Source:HGNC Symbol;Acc:HGNC:4034]	19
ENSG00000130244	3,724248	1,115722	0,003185	0,017349	3,426601	protein_coding	FAM98C	family with sequence similarity 98 member C [Source:HGNC Symbol;Acc:HGNC:27119]	19
ENSG00000108061	5,949612	-0,507122	0,003184	0,017349	-3,426674	protein_coding	SHOC2	SHOC2 leucine rich repeat scaffold protein [Source:HGNC Symbol;Acc:HGNC:15454]	10
ENSG00000167850	-0,580954	1,649156	0,00319	0,017363	3,425794	protein_coding	CD300C	CD300c molecule [Source:HGNC Symbol;Acc:HGNC:19320]	17
ENSG00000184185	0,555526	2,160287	0,003192	0,017366	3,425536	protein_coding	KCNJ12	potassium inwardly rectifying channel subfamily J member 12 [Source:HGNC Symbol;Acc:HGNC:6258]	17
ENSG00000196482	3,120667	2,695947	0,003195	0,017376	3,425136	protein_coding	ESRRG	estrogen related receptor gamma [Source:HGNC Symbol;Acc:HGNC:3474]	1
ENSG00000175040	4,000659	-1,319561	0,003197	0,017382	-3,424805	protein_coding	CHST2	carbohydrate sulfotransferase 2 [Source:HGNC Symbol;Acc:HGNC:1970]	3
ENSG00000138674	7,870397	-0,883761	0,0032	0,01739	-3,424462	protein_coding	SEC31A	SEC31 homolog A, COPII coat complex component [Source:HGNC Symbol;Acc:HGNC:17052]	4
ENSG00000122025	-2,864694	2,581304	0,003205	0,017412	3,423717	protein_coding	FLT3	fms related receptor tyrosine kinase 3 [Source:HGNC Symbol;Acc:HGNC:3765]	13
ENSG00000155530	0,824391	2,354921	0,003207	0,017419	3,423375	protein_coding	LRGUK	leucine rich repeats and guanylate kinase domain containing [Source:HGNC Symbol;Acc:HGNC:21964]	7
ENSG00000100368	2,004796	2,940627	0,00321	0,017428	3,422983	protein_coding	CSF2RB	colony stimulating factor 2 receptor subunit beta [Source:HGNC Symbol;Acc:HGNC:2436]	22
ENSG00000030419	2,937329	1,380429	0,00322	0,017452	3,421578	protein_coding	IKZF2	IKAROS family zinc finger 2 [Source:HGNC Symbol;Acc:HGNC:13177]	2
ENSG00000111737	5,489618	-0,576748	0,003216	0,017452	-3,422191	protein_coding	RAB35	RAB35, member RAS oncogene family [Source:HGNC Symbol;Acc:HGNC:9774]	12
ENSG00000198642	5,661818	-0,556803	0,003219	0,017452	-3,421758	protein_coding	KLHL9	kelch like family member 9 [Source:HGNC Symbol;Acc:HGNC:18732]	9
ENSG00000198563	5,980285	0,95987	0,003217	0,017452	3,421958	protein_coding	DDX39B	DExD-box helicase 39B [Source:HGNC Symbol;Acc:HGNC:13917]	6
ENSG00000198853	5,994832	-0,730253	0,003219	0,017452	-3,421688	protein_coding	RUSC2	RUN and SH3 domain containing 2 [Source:HGNC Symbol;Acc:HGNC:23625]	9
ENSG00000169891	3,521665	1,188138	0,003221	0,017452	3,421417	protein_coding	REPS2	RALBP1 associated Eps domain containing 2 [Source:HGNC Symbol;Acc:HGNC:9963]	X
ENSG00000085224	7,948371	0,50327	0,003223	0,01746	3,421066	protein_coding	ATRX	ATRX chromatin remodeler [Source:HGNC Symbol;Acc:HGNC:886]	X
ENSG00000239305	5,501333	-0,574734	0,003227	0,017476	-3,420502	protein_coding	RNF103	ring finger protein 103 [Source:HGNC Symbol;Acc:HGNC:12859]	2
ENSG00000130592	4,400135	1,943091	0,003239	0,017535	3,418773	protein_coding	LSP1	lymphocyte specific protein 1 [Source:HGNC Symbol;Acc:HGNC:6707]	11
ENSG00000175573	3,458543	-0,823697	0,003243	0,017548	-3,418292	protein_coding	C11orf68	chromosome 11 open reading frame 68 [Source:HGNC Symbol;Acc:HGNC:28801]	11
ENSG00000143028	0,964361	-2,034162	0,003247	0,017567	-3,417646	protein_coding	SYPL2	synaptophysin like 2 [Source:HGNC Symbol;Acc:HGNC:27638]	1
ENSG00000167272	3,36845	-0,581911	0,003253	0,01759	-3,416887	protein_coding	POP5	POP5 homolog, ribonuclease P/MRP subunit [Source:HGNC Symbol;Acc:HGNC:17689]	12
ENSG00000068976	-0,761464	1,967927	0,003269	0,01767	3,414651	protein_coding	PYGM	glycogen phosphorylase, muscle associated [Source:HGNC Symbol;Acc:HGNC:9726]	11
ENSG00000263465	5,136014	-0,632724	0,003271	0,017679	-3,414264	protein_coding	SRSF8	serine and arginine rich splicing factor 8 [Source:HGNC Symbol;Acc:HGNC:16988]	11
ENSG00000152102	6,791828	-0,562961	0,003274	0,017685	-3,413944	protein_coding	FAM168B	family with sequence similarity 168 member B [Source:HGNC Symbol;Acc:HGNC:27016]	2

ENSG00000123609	3,5366	-1,019902	0,003278	0,017705	-3,413274	protein_coding	NMI	N-myc and STAT interactor [Source:HGNC Symbol;Acc:HGNC:7854]	2
ENSG00000163430	8,088666	-1,583685	0,003287	0,017745	-3,412083	protein_coding	FSTL1	follistatin like 1 [Source:HGNC Symbol;Acc:HGNC:3972]	3
ENSG00000180185	4,446047	-0,697279	0,003291	0,017763	-3,41146	protein_coding	FAHD1	fumarylacetoacetate hydrolase domain containing 1 [Source:HGNC Symbol;Acc:HGNC:14169]	16
ENSG00000140678	3,880362	1,935858	0,003302	0,017815	3,409967	protein_coding	ITGAX	integrin subunit alpha X [Source:HGNC Symbol;Acc:HGNC:6152]	16
ENSG00000130751	1,256973	-1,978197	0,003306	0,017828	-3,409436	protein_coding	NPAS1	neuronal PAS domain protein 1 [Source:HGNC Symbol;Acc:HGNC:7894]	19
ENSG00000112983	6,432133	0,635105	0,003307	0,017828	3,409312	protein_coding	BRD8	bromodomain containing 8 [Source:HGNC Symbol;Acc:HGNC:19874]	5
ENSG00000144488	-0,555798	2,899271	0,003315	0,017866	3,408193	protein_coding	ESPNL	espin like [Source:HGNC Symbol;Acc:HGNC:27937]	2
ENSG00000109323	5,83036	-0,970969	0,003319	0,017882	-3,407623	protein_coding	MANBA	mannosidase beta [Source:HGNC Symbol;Acc:HGNC:6831]	4
ENSG00000183137	4,50513	0,769816	0,003322	0,017895	3,407145	protein_coding	CEP57L1	centrosomal protein 57 like 1 [Source:HGNC Symbol;Acc:HGNC:21561]	6
ENSG00000177469	7,983736	-1,573987	0,003329	0,017924	-3,406233	protein_coding	CAVIN1	caveolae associated protein 1 [Source:HGNC Symbol;Acc:HGNC:9688]	17
ENSG00000265107	0,207199	2,336875	0,003331	0,017931	3,405906	protein_coding	GJA5	gap junction protein alpha 5 [Source:HGNC Symbol;Acc:HGNC:4279]	1
ENSG00000073670	0,553958	2,218993	0,003339	0,017968	3,404792	protein_coding	ADAM11	ADAM metallopeptidase domain 11 [Source:HGNC Symbol;Acc:HGNC:189]	17
ENSG00000170881	4,405647	-0,710935	0,003344	0,017987	-3,404164	protein_coding	RNF139	ring finger protein 139 [Source:HGNC Symbol;Acc:HGNC:17023]	8
ENSG00000162413	4,876951	-1,016365	0,003347	0,017998	-3,403742	protein_coding	KLHL21	kelch like family member 21 [Source:HGNC Symbol;Acc:HGNC:29041]	1
ENSG00000145416	2,197796	2,87748	0,003349	0,018002	3,403484	protein_coding	MARCHF1	membrane associated ring-CH-type finger 1 [Source:HGNC Symbol;Acc:HGNC:26077]	4
ENSG00000166432	2,108089	3,174	0,003356	0,018034	3,402498	protein_coding	ZMAT1	zinc finger matrin-type 1 [Source:HGNC Symbol;Acc:HGNC:29377]	X
ENSG00000177283	4,642814	-2,053338	0,003362	0,01806	-3,401683	protein_coding	FZD8	frizzled class receptor 8 [Source:HGNC Symbol;Acc:HGNC:4046]	10
ENSG00000179344	4,273571	2,690871	0,003369	0,018091	3,400753	protein_coding	HLA-DQB1	major histocompatibility complex, class II, DQ beta 1 [Source:HGNC Symbol;Acc:HGNC:4944]	6
ENSG00000187260	-1,057055	3,010431	0,003371	0,018099	3,400389	protein_coding	WDR86	WD repeat domain 86 [Source:HGNC Symbol;Acc:HGNC:28020]	7
ENSG00000182256	-2,219004	2,806309	0,003376	0,018117	3,399786	protein_coding	GABRG3	gamma-aminobutyric acid type A receptor subunit gamma3 [Source:HGNC Symbol;Acc:HGNC:4088]	15
ENSG00000189184	4,922724	-1,734438	0,003379	0,018127	-3,399374	protein_coding	PCDH18	protocadherin 18 [Source:HGNC Symbol;Acc:HGNC:14268]	4
ENSG00000154122	7,1733	-0,878319	0,003381	0,018134	-3,399054	protein_coding	ANKH	ANKH inorganic pyrophosphate transport regulator [Source:HGNC Symbol;Acc:HGNC:15492]	5
ENSG00000161010	4,656744	0,840865	0,00339	0,018174	3,397881	protein_coding	MRNIP	MRN complex interacting protein [Source:HGNC Symbol;Acc:HGNC:30817]	5
ENSG00000181085	-0,167087	2,31727	0,003393	0,018184	3,397471	protein_coding	MAPK15	mitogen-activated protein kinase 15 [Source:HGNC Symbol;Acc:HGNC:24667]	8
ENSG00000205309	1,429811	2,15713	0,003406	0,018247	3,395663	protein_coding	NT5M	5',3'-nucleotidase, mitochondrial [Source:HGNC Symbol;Acc:HGNC:15769]	17
ENSG00000169564	7,444865	-0,67725	0,003407	0,018247	-3,395581	protein_coding	PCBP1	poly(rC) binding protein 1 [Source:HGNC Symbol;Acc:HGNC:8647]	2
ENSG00000258539	-1,048481	1,778974	0,003417	0,018293	3,394265	protein_coding	AC068896.	novel transcript, METTL10-FAM53B readthrough	10
ENSG00000113161	6,851685	-0,868688	0,00342	0,018304	-3,393838	protein_coding	HMGCR	3-hydroxy-3-methylglutaryl-CoA reductase [Source:HGNC Symbol;Acc:HGNC:5006]	5
ENSG00000232859	1,259974	1,343338	0,003424	0,01832	3,393292	protein_coding	LYRM9	LYR motif containing 9 [Source:HGNC Symbol;Acc:HGNC:27314]	17
ENSG00000005243	3,112436	-1,705278	0,003431	0,018345	-3,392358	protein_coding	COPZ2	COPI coat complex subunit zeta 2 [Source:HGNC Symbol;Acc:HGNC:19356]	17
ENSG00000185627	5,792877	-0,513784	0,00343	0,018345	-3,392496	protein_coding	PSMD13	proteasome 26S subunit, non-ATPase 13 [Source:HGNC Symbol;Acc:HGNC:9558]	11
ENSG00000205929	-3,168685	3,318045	0,003443	0,0184	3,39068	protein_coding	C21orf62	chromosome 21 open reading frame 62 [Source:HGNC Symbol;Acc:HGNC:1305]	21
ENSG00000169231	4,545489	1,013166	0,003443	0,0184	3,390665	protein_coding	THBS3	thrombospondin 3 [Source:HGNC Symbol;Acc:HGNC:11787]	1
ENSG00000161835	3,69531	2,271777	0,003445	0,018403	3,390454	protein_coding	TAMALIN	trafficking regulator and scaffold protein tamalin [Source:HGNC Symbol;Acc:HGNC:18707]	12
ENSG00000185739	-2,41616	2,983324	0,003451	0,018421	3,389604	protein_coding	SRL	sarcalumenin [Source:HGNC Symbol;Acc:HGNC:11295]	16
ENSG00000129646	3,496935	1,200598	0,003452	0,018421	3,389457	protein_coding	QRICH2	glutamine rich 2 [Source:HGNC Symbol;Acc:HGNC:25326]	17
ENSG00000163249	4,592964	-0,834995	0,003453	0,018421	-3,389394	protein_coding	CCNYL1	cyclin Y like 1 [Source:HGNC Symbol;Acc:HGNC:26868]	2
ENSG00000115364	5,365243	-0,548482	0,003452	0,018421	-3,389454	protein_coding	MRPL19	mitochondrial ribosomal protein L19 [Source:HGNC Symbol;Acc:HGNC:14052]	2
ENSG00000163815	-0,415615	2,520218	0,003458	0,018435	3,388743	protein_coding	CLEC3B	C-type lectin domain family 3 member B [Source:HGNC Symbol;Acc:HGNC:11891]	3
ENSG00000180008	5,894539	-0,564741	0,003458	0,018435	-3,388775	protein_coding	SOCS4	suppressor of cytokine signaling 4 [Source:HGNC Symbol;Acc:HGNC:19392]	14
ENSG00000127152	1,105969	2,973048	0,003461	0,018446	3,388324	protein_coding	BCL11B	BAF chromatin remodeling complex subunit BCL11B [Source:HGNC Symbol;Acc:HGNC:13222]	14
ENSG00000129667	4,486902	1,011971	0,003464	0,018448	3,38796	protein_coding	RHBDF2	rhomboid 5 homolog 2 [Source:HGNC Symbol;Acc:HGNC:20788]	17
ENSG00000143158	5,307068	-0,520201	0,003463	0,018448	-3,388094	protein_coding	MPC2	mitochondrial pyruvate carrier 2 [Source:HGNC Symbol;Acc:HGNC:24515]	1
ENSG00000111832	5,394083	-0,6636	0,003473	0,018493	-3,386697	protein_coding	RWDD1	RWD domain containing 1 [Source:HGNC Symbol;Acc:HGNC:20993]	6
ENSG00000115361	-2,326422	3,389974	0,003476	0,018497	3,386378	protein_coding	ACADL	acyl-CoA dehydrogenase long chain [Source:HGNC Symbol;Acc:HGNC:88]	2
ENSG00000086289	4,91077	-1,291234	0,003476	0,018497	-3,386289	protein_coding	EPDR1	ependymin related 1 [Source:HGNC Symbol;Acc:HGNC:17572]	7
ENSG00000163605	6,076143	-0,523725	0,003483	0,018529	-3,385349	protein_coding	PPP4R2	protein phosphatase 4 regulatory subunit 2 [Source:HGNC Symbol;Acc:HGNC:18296]	3
ENSG00000150625	-2,408195	3,116152	0,003486	0,018534	3,385046	protein_coding	GPM6A	glycoprotein M6A [Source:HGNC Symbol;Acc:HGNC:4460]	4
ENSG00000244731	1,407351	2,949251	0,003487	0,018534	3,38491	protein_coding	C4A	complement C4A (Rodgers blood group) [Source:HGNC Symbol;Acc:HGNC:1323]	6
ENSG00000158122	3,731371	-0,797769	0,003488	0,018536	-3,384718	protein_coding	PRXL2C	peroxiredoxin like 2C [Source:HGNC Symbol;Acc:HGNC:16881]	9
ENSG00000086205	0,753039	2,367725	0,003494	0,018553	3,383991	protein_coding	FOLH1	folate hydrolase 1 [Source:HGNC Symbol;Acc:HGNC:3788]	11

ENSG00000036530	1,397037	2,48595	0,003493	0,018553	3,384111	protein_coding	CYP46A1	cytochrome P450 family 46 subfamily A member 1 [Source:HGNC Symbol;Acc:HGNC:2641]	14
ENSG00000042781	0,456074	2,839641	0,0035	0,018578	3,383208	protein_coding	USH2A	usherin [Source:HGNC Symbol;Acc:HGNC:12601]	1
ENSG00000136522	4,255138	-0,6751	0,003502	0,018587	-3,382851	protein_coding	MRPL47	mitochondrial ribosomal protein L47 [Source:HGNC Symbol;Acc:HGNC:16652]	3
ENSG00000131471	2,795575	2,457262	0,003504	0,018589	3,38265	protein_coding	AOC3	amine oxidase copper containing 3 [Source:HGNC Symbol;Acc:HGNC:550]	17
ENSG00000093072	3,216408	2,164394	0,003506	0,018589	3,382355	protein_coding	ADA2	adenosine deaminase 2 [Source:HGNC Symbol;Acc:HGNC:1839]	22
ENSG00000108474	3,645644	1,096554	0,003505	0,018589	3,382436	protein_coding	PIGL	phosphatidylinositol glycan anchor biosynthesis class L [Source:HGNC Symbol;Acc:HGNC:8966]	17
ENSG00000165006	5,725687	-0,559256	0,003508	0,018594	-3,382068	protein_coding	UBAP1	ubiquitin associated protein 1 [Source:HGNC Symbol;Acc:HGNC:12461]	9
ENSG00000213316	-0,763256	2,041032	0,003517	0,01863	3,380934	protein_coding	LTC4S	leukotriene C4 synthase [Source:HGNC Symbol;Acc:HGNC:6719]	5
ENSG00000119541	5,932276	-0,628947	0,003517	0,01863	-3,380883	protein_coding	VPS4B	vacuolar protein sorting 4 homolog B [Source:HGNC Symbol;Acc:HGNC:10895]	18
ENSG00000104361	4,892495	-0,863329	0,003519	0,018633	-3,380646	protein_coding	NIPAL2	NIPA like domain containing 2 [Source:HGNC Symbol;Acc:HGNC:25854]	8
ENSG00000137077	-1,617895	6,329328	0,003522	0,018643	3,380262	protein_coding	CCL21	C-C motif chemokine ligand 21 [Source:HGNC Symbol;Acc:HGNC:10620]	9
ENSG00000100321	4,018486	2,342407	0,003525	0,018651	3,37992	protein_coding	SYNGR1	synaptogyrin 1 [Source:HGNC Symbol;Acc:HGNC:11498]	22
ENSG00000159216	7,031972	-1,017513	0,003528	0,018663	-3,379471	protein_coding	RUNX1	RUNX family transcription factor 1 [Source:HGNC Symbol;Acc:HGNC:10471]	21
ENSG00000166886	4,790873	-1,189856	0,00353	0,018663	-3,379283	protein_coding	NAB2	NGFI-A binding protein 2 [Source:HGNC Symbol;Acc:HGNC:7627]	12
ENSG00000125686	6,798187	-0,49585	0,003531	0,018663	-3,379153	protein_coding	MED1	mediator complex subunit 1 [Source:HGNC Symbol;Acc:HGNC:9234]	17
ENSG00000169139	5,33911	-0,565003	0,003533	0,018669	-3,378784	protein_coding	UBE2V2	ubiquitin conjugating enzyme E2 V2 [Source:HGNC Symbol;Acc:HGNC:12495]	8
ENSG00000137161	5,960402	0,632094	0,003534	0,018669	3,378722	protein_coding	CNPY3	canopy FGF signaling regulator 3 [Source:HGNC Symbol;Acc:HGNC:11968]	6
ENSG00000111218	0,186077	2,946917	0,003538	0,018685	3,378184	protein_coding	PRMT8	protein arginine methyltransferase 8 [Source:HGNC Symbol;Acc:HGNC:5188]	12
ENSG00000161999	4,952866	-0,592393	0,003541	0,018695	-3,377773	protein_coding	JMJD8	jumonji domain containing 8 [Source:HGNC Symbol;Acc:HGNC:14148]	16
ENSG00000134698	4,900773	0,660577	0,003543	0,018698	3,377549	protein_coding	AGO4	argonaute RISC component 4 [Source:HGNC Symbol;Acc:HGNC:18424]	1
ENSG00000151466	4,447354	0,755189	0,003553	0,018734	3,37623	protein_coding	SCLT1	sodium channel and clathrin linker 1 [Source:HGNC Symbol;Acc:HGNC:26406]	4
ENSG00000130513	7,304744	-2,044883	0,003552	0,018734	-3,376416	protein_coding	GDF15	growth differentiation factor 15 [Source:HGNC Symbol;Acc:HGNC:30142]	19
ENSG00000138593	6,945872	-0,47687	0,003553	0,018734	-3,376224	protein_coding	SECISBP2L	SECIS binding protein 2 like [Source:HGNC Symbol;Acc:HGNC:28997]	15
ENSG00000197885	3,628321	-0,566564	0,003561	0,018767	-3,37525	protein_coding	NKIRAS1	NFKB inhibitor interacting Ras like 1 [Source:HGNC Symbol;Acc:HGNC:17899]	3
ENSG00000161082	0,035128	3,99863	0,003564	0,018778	3,374838	protein_coding	CELF5	CUGBP Elav-like family member 5 [Source:HGNC Symbol;Acc:HGNC:14058]	19
ENSG00000104660	5,125421	-0,708586	0,003566	0,018784	-3,374551	protein_coding	LEPROTL1	leptin receptor overlapping transcript like 1 [Source:HGNC Symbol;Acc:HGNC:6555]	8
ENSG00000089692	0,693655	1,770752	0,003573	0,018814	3,373652	protein_coding	LAG3	lymphocyte activating 3 [Source:HGNC Symbol;Acc:HGNC:6476]	12
ENSG00000213780	-1,411661	1,641069	0,00358	0,018838	3,372689	protein_coding	GTF2H4	general transcription factor IIH subunit 4 [Source:HGNC Symbol;Acc:HGNC:4658]	6
ENSG00000114541	4,12732	1,635924	0,00358	0,018838	3,372761	protein_coding	FRMD4B	FERM domain containing 4B [Source:HGNC Symbol;Acc:HGNC:24886]	3
ENSG00000183648	5,232569	-0,704771	0,003581	0,018838	-3,372619	protein_coding	NDUFB1	NADH:ubiquinone oxidoreductase subunit B1 [Source:HGNC Symbol;Acc:HGNC:7695]	14
ENSG00000170004	7,818354	0,700048	0,00359	0,018878	3,371503	protein_coding	CHD3	chromodomain helicase DNA binding protein 3 [Source:HGNC Symbol;Acc:HGNC:1918]	17
ENSG00000099864	3,803976	2,281593	0,003601	0,018932	3,370039	protein_coding	PALM	paralemmin [Source:HGNC Symbol;Acc:HGNC:8594]	19
ENSG00000168395	5,354709	0,876721	0,003611	0,018977	3,368798	protein_coding	ING5	inhibitor of growth family member 5 [Source:HGNC Symbol;Acc:HGNC:19421]	2
ENSG00000139726	6,136861	-0,483888	0,003612	0,018978	-3,368605	protein_coding	DENR	density regulated re-initiation and release factor [Source:HGNC Symbol;Acc:HGNC:2769]	12
ENSG00000108932	3,234519	-4,0614	0,003615	0,018986	-3,368249	protein_coding	SLC16A6	solute carrier family 16 member 6 [Source:HGNC Symbol;Acc:HGNC:10927]	17
ENSG00000164077	3,56101	-0,628765	0,003617	0,018986	-3,368065	protein_coding	MON1A	MON1 homolog A, secretory trafficking associated [Source:HGNC Symbol;Acc:HGNC:28207]	3
ENSG00000135387	8,410331	-0,776981	0,003617	0,018986	-3,367975	protein_coding	CAPRIN1	cell cycle associated protein 1 [Source:HGNC Symbol;Acc:HGNC:6743]	11
ENSG00000121350	4,577155	-1,08391	0,00362	0,018994	-3,367619	protein_coding	PYROXD1	pyridine nucleotide-disulphide oxidoreductase domain 1 [Source:HGNC Symbol;Acc:HGNC:26162]	12
ENSG00000104894	1,383653	2,964744	0,003628	0,019007	3,366637	protein_coding	CD37	CD37 molecule [Source:HGNC Symbol;Acc:HGNC:1666]	19
ENSG00000135439	3,23167	1,940254	0,003629	0,019007	3,366418	protein_coding	AGAP2	ArfGAP with GTPase domain, ankyrin repeat and PH domain 2 [Source:HGNC Symbol;Acc:HGNC:16921]	12
ENSG00000111879	3,652074	1,446715	0,003626	0,019007	3,36683	protein_coding	FAM184A	family with sequence similarity 184 member A [Source:HGNC Symbol;Acc:HGNC:20991]	6
ENSG00000157350	5,397608	-0,740421	0,003629	0,019007	-3,366477	protein_coding	ST3GAL2	ST3 beta-galactoside alpha-2,3-sialyltransferase 2 [Source:HGNC Symbol;Acc:HGNC:10863]	16
ENSG00000211455	6,299942	-1,084969	0,003625	0,019007	-3,366936	protein_coding	STK38L	serine/threonine kinase 38 like [Source:HGNC Symbol;Acc:HGNC:17848]	12
ENSG00000160075	6,521493	-0,670213	0,003625	0,019007	-3,367018	protein_coding	SSU72	SSU72 homolog, RNA polymerase II CTD phosphatase [Source:HGNC Symbol;Acc:HGNC:25016]	1
ENSG00000125520	5,502205	0,831346	0,003634	0,019024	3,365867	protein_coding	SLC2A4RG	SLC2A4 regulator [Source:HGNC Symbol;Acc:HGNC:15930]	20
ENSG00000250067	2,755889	1,295509	0,003635	0,019024	3,365705	protein_coding	YJEFN3	YjeF N-terminal domain containing 3 [Source:HGNC Symbol;Acc:HGNC:24785]	19
ENSG00000140009	-0,405798	3,0802	0,003639	0,019038	3,36517	protein_coding	ESR2	estrogen receptor 2 [Source:HGNC Symbol;Acc:HGNC:3468]	14
ENSG00000204271	4,116374	0,87486	0,00364	0,019038	3,365076	protein_coding	SPIN3	spindlin family member 3 [Source:HGNC Symbol;Acc:HGNC:27272]	X
ENSG00000176531	4,754651	1,068151	0,003642	0,019043	3,364809	protein_coding	PHLDB3	pleckstrin homology like domain family B member 3 [Source:HGNC Symbol;Acc:HGNC:30499]	19
ENSG00000122550	5,089635	-0,623214	0,003653	0,01909	-3,363388	protein_coding	KLHL7	kelch like family member 7 [Source:HGNC Symbol;Acc:HGNC:15646]	7
ENSG00000087502	5,874811	-0,671366	0,003653	0,01909	-3,363497	protein_coding	ERGIC2	ERGIC and golgi 2 [Source:HGNC Symbol;Acc:HGNC:30208]	12

ENSG00000128274	3,400713	-1,523848	0,003658	0,019107	-3,362787	protein_coding	A4GALT	alpha 1,4-galactosyltransferase (P blood group) [Source:HGNC Symbol;Acc:HGNC:18149]	22
ENSG00000137474	3,919697	1,942951	0,003659	0,019107	3,362664	protein_coding	MYO7A	myosin VIIA [Source:HGNC Symbol;Acc:HGNC:7606]	11
ENSG00000135631	4,911849	-0,813059	0,003666	0,019129	-3,361854	protein_coding	RAB11FIP5	RAB11 family interacting protein 5 [Source:HGNC Symbol;Acc:HGNC:24845]	2
ENSG00000112033	5,494169	-0,908099	0,003665	0,019129	-3,361959	protein_coding	PPARD	peroxisome proliferator activated receptor delta [Source:HGNC Symbol;Acc:HGNC:9235]	6
ENSG00000157510	3,32973	1,883958	0,003668	0,019133	3,3616	protein_coding	AFAP1L1	actin filament associated protein 1 like 1 [Source:HGNC Symbol;Acc:HGNC:26714]	5
ENSG00000188878	2,423068	1,343853	0,003671	0,019146	3,361133	protein_coding	FBF1	Fas binding factor 1 [Source:HGNC Symbol;Acc:HGNC:24674]	17
ENSG00000241370	0,341326	1,296346	0,003675	0,019157	3,360722	protein_coding	RPP21	ribonuclease P/MRP subunit p21 [Source:HGNC Symbol;Acc:HGNC:21300]	6
ENSG00000185100	3,140951	1,638714	0,003676	0,01916	3,360504	protein_coding	ADSS1	adenylosuccinate synthase 1 [Source:HGNC Symbol;Acc:HGNC:20093]	14
ENSG00000085491	5,487581	-0,810186	0,003679	0,019169	-3,360149	protein_coding	SLC25A24	solute carrier family 25 member 24 [Source:HGNC Symbol;Acc:HGNC:20662]	1
ENSG00000011009	4,599457	-0,735667	0,00368	0,019169	-3,359997	protein_coding	LYPLA2	lysophospholipase 2 [Source:HGNC Symbol;Acc:HGNC:6738]	1
ENSG00000144712	2,493324	2,732172	0,003682	0,01917	3,359814	protein_coding	CAND2	cullin associated and neddylation dissociated 2 (putative) [Source:HGNC Symbol;Acc:HGNC:30689]	3
ENSG00000204653	-1,06427	3,29167	0,003688	0,019194	3,359095	protein_coding	ASPDH	aspartate dehydrogenase domain containing [Source:HGNC Symbol;Acc:HGNC:33856]	19
ENSG00000081791	5,44304	0,441682	0,003693	0,019217	3,358386	protein_coding	DELE1	DAP3 binding cell death enhancer 1 [Source:HGNC Symbol;Acc:HGNC:28969]	5
ENSG00000161558	2,446011	0,968205	0,003696	0,019219	3,358044	protein_coding	TMEM143	transmembrane protein 143 [Source:HGNC Symbol;Acc:HGNC:25603]	19
ENSG00000100106	7,021483	-0,675431	0,003696	0,019219	-3,358096	protein_coding	TRIOBP	TRIO and F-actin binding protein [Source:HGNC Symbol;Acc:HGNC:17009]	22
ENSG00000196421	0,934176	1,533071	0,003702	0,019246	3,35726	protein_coding	C20orf204	chromosome 20 open reading frame 204 [Source:HGNC Symbol;Acc:HGNC:27655]	20
ENSG00000198585	5,513427	0,55009	0,003708	0,019268	3,356583	protein_coding	NUDT16	nudix hydrolase 16 [Source:HGNC Symbol;Acc:HGNC:26442]	3
ENSG00000180592	-0,112338	1,836015	0,003712	0,019277	3,356074	protein_coding	SKIDA1	SKI/DACH domain containing 1 [Source:HGNC Symbol;Acc:HGNC:32697]	10
ENSG00000198356	5,258977	-0,542953	0,003711	0,019277	-3,356136	protein_coding	GET3	guided entry of tail-anchored proteins factor 3, ATPase [Source:HGNC Symbol;Acc:HGNC:752]	19
ENSG00000172339	2,767964	-0,684392	0,003715	0,019286	-3,355705	protein_coding	ALG14	ALG14 UDP-N-acetylglucosaminyltransferase subunit [Source:HGNC Symbol;Acc:HGNC:28287]	1
ENSG00000225828	1,881135	0,895504	0,003721	0,019315	3,354882	protein_coding	FAM229A	family with sequence similarity 229 member A [Source:HGNC Symbol;Acc:HGNC:44652]	1
ENSG00000124257	1,305958	1,25318	0,003724	0,019317	3,354532	protein_coding	NEURL2	neuralized E3 ubiquitin protein ligase 2 [Source:HGNC Symbol;Acc:HGNC:16156]	20
ENSG00000101558	7,086657	-0,615102	0,003724	0,019317	-3,354592	protein_coding	VAPA	VAMP associated protein A [Source:HGNC Symbol;Acc:HGNC:12648]	18
ENSG00000154589	1,494438	-1,576033	0,003726	0,019322	-3,354275	protein_coding	LY96	lymphocyte antigen 96 [Source:HGNC Symbol;Acc:HGNC:17156]	8
ENSG00000205592	-1,283475	3,030468	0,003735	0,019357	3,353194	protein_coding	MUC19	mucin 19, oligomeric [Source:HGNC Symbol;Acc:HGNC:14362]	12
ENSG00000174326	-0,111093	3,175663	0,003737	0,019357	3,352979	protein_coding	SLC16A11	solute carrier family 16 member 11 [Source:HGNC Symbol;Acc:HGNC:23093]	17
ENSG00000138175	4,758714	-0,670757	0,003737	0,019357	-3,353002	protein_coding	ARL3	ADP ribosylation factor like GTPase 3 [Source:HGNC Symbol;Acc:HGNC:694]	10
ENSG00000096093	4,592862	1,038912	0,003738	0,019359	3,352804	protein_coding	EFHC1	EF-hand domain containing 1 [Source:HGNC Symbol;Acc:HGNC:16406]	6
ENSG00000198626	2,69858	4,911806	0,00374	0,019359	3,352638	protein_coding	RYR2	ryanodine receptor 2 [Source:HGNC Symbol;Acc:HGNC:10484]	1
ENSG00000176533	1,054353	3,65185	0,003741	0,019362	3,352422	protein_coding	GNG7	G protein subunit gamma 7 [Source:HGNC Symbol;Acc:HGNC:4410]	19
ENSG00000107719	2,955273	2,567003	0,003746	0,019382	3,35181	protein_coding	PALD1	phosphatase domain containing paladin 1 [Source:HGNC Symbol;Acc:HGNC:23530]	10
ENSG00000151006	0,768963	1,061243	0,003754	0,019405	3,350885	protein_coding	PRSS53	serine protease 53 [Source:HGNC Symbol;Acc:HGNC:34407]	16
ENSG00000149292	3,180249	1,446085	0,003754	0,019405	3,350821	protein_coding	TTC12	tetratricopeptide repeat domain 12 [Source:HGNC Symbol;Acc:HGNC:23700]	11
ENSG00000002834	8,3702	-0,58019	0,003753	0,019405	-3,351043	protein_coding	LASP1	LIM and SH3 protein 1 [Source:HGNC Symbol;Acc:HGNC:6513]	17
ENSG00000178338	3,638782	0,735964	0,003756	0,019408	3,350607	protein_coding	ZNF354B	zinc finger protein 354B [Source:HGNC Symbol;Acc:HGNC:17197]	5
ENSG00000111530	7,376761	-0,49091	0,003759	0,019417	-3,350248	protein_coding	CAND1	cullin associated and neddylation dissociated 1 [Source:HGNC Symbol;Acc:HGNC:30688]	12
ENSG00000187498	10,41209	-1,417716	0,003762	0,019421	-3,349852	protein_coding	COL4A1	collagen type IV alpha 1 chain [Source:HGNC Symbol;Acc:HGNC:2202]	13
ENSG00000102359	5,180747	-1,772643	0,003762	0,019421	-3,349947	protein_coding	SRPX2	sushi repeat containing protein X-linked 2 [Source:HGNC Symbol;Acc:HGNC:30668]	X
ENSG00000164038	4,265256	-0,973431	0,003767	0,019434	-3,349248	protein_coding	SLC9B2	solute carrier family 9 member B2 [Source:HGNC Symbol;Acc:HGNC:25143]	4
ENSG00000163527	7,400931	-0,675539	0,003766	0,019434	-3,349357	protein_coding	STT3B	STT3 oligosaccharyltransferase complex catalytic subunit B [Source:HGNC Symbol;Acc:HGNC:30611]	3
ENSG00000106052	7,539717	-0,638996	0,003773	0,019457	-3,348557	protein_coding	TAX1BP1	Tax1 binding protein 1 [Source:HGNC Symbol;Acc:HGNC:11575]	7
ENSG00000161640	-1,833442	3,93685	0,003776	0,019467	3,348126	protein_coding	SIGLEC11	sialic acid binding Ig like lectin 11 [Source:HGNC Symbol;Acc:HGNC:15622]	19
ENSG00000170473	4,132686	-0,488922	0,003777	0,019467	-3,348031	protein_coding	PYM1	PYM homolog 1, exon junction complex associated factor [Source:HGNC Symbol;Acc:HGNC:30258]	12
ENSG00000169019	3,528995	-0,843339	0,003778	0,019467	-3,347878	protein_coding	COMMD8	COMM domain containing 8 [Source:HGNC Symbol;Acc:HGNC:26036]	4
ENSG00000129559	3,192756	-0,598497	0,003781	0,019476	-3,347534	protein_coding	NEDD8	NEDD8 ubiquitin like modifier [Source:HGNC Symbol;Acc:HGNC:7732]	14
ENSG00000183087	5,872717	1,407394	0,003784	0,019485	3,347157	protein_coding	GAS6	growth arrest specific 6 [Source:HGNC Symbol;Acc:HGNC:4168]	13
ENSG00000241644	1,631535	2,593038	0,003794	0,019508	3,346042	protein_coding	INMT	indolethylamine N-methyltransferase [Source:HGNC Symbol;Acc:HGNC:6069]	7
ENSG00000121864	4,549212	-0,555057	0,003793	0,019508	-3,346165	protein_coding	ZNF639	zinc finger protein 639 [Source:HGNC Symbol;Acc:HGNC:30950]	3
ENSG00000011295	5,390243	0,735278	0,003793	0,019508	3,346088	protein_coding	TTC19	tetratricopeptide repeat domain 19 [Source:HGNC Symbol;Acc:HGNC:26006]	17
ENSG00000198690	5,485445	0,75069	0,003793	0,019508	3,34606	protein_coding	FAN1	FANCD2 and FANCI associated nuclease 1 [Source:HGNC Symbol;Acc:HGNC:29170]	15
ENSG00000135048	7,102441	-1,087922	0,003796	0,019512	-3,345797	protein_coding	CEMIP2	cell migration inducing hyaluronidase 2 [Source:HGNC Symbol;Acc:HGNC:11869]	9

ENSG00000182704	4,448135	-1,053745	0,0038	0,019529	-3,345254	protein_coding	TSKU	tsukushi, small leucine rich proteoglycan [Source:HGNC Symbol;Acc:HGNC:28850]	11
ENSG00000173575	7,49264	0,655027	0,003802	0,019534	3,344989	protein_coding	CHD2	chromodomain helicase DNA binding protein 2 [Source:HGNC Symbol;Acc:HGNC:1917]	15
ENSG00000065613	7,385134	-0,486785	0,003806	0,019547	-3,34453	protein_coding	SLK	STE20 like kinase [Source:HGNC Symbol;Acc:HGNC:11088]	10
ENSG00000087237	-0,648456	2,738781	0,00381	0,01956	3,344085	protein_coding	CETP	cholesteryl ester transfer protein [Source:HGNC Symbol;Acc:HGNC:1869]	16
ENSG00000139437	5,256342	0,608233	0,003814	0,019577	3,343526	protein_coding	TCHP	trichoplein keratin filament binding [Source:HGNC Symbol;Acc:HGNC:28135]	12
ENSG00000161609	-1,785429	3,340122	0,003821	0,019606	3,342705	protein_coding	KASH5	KASH domain containing 5 [Source:HGNC Symbol;Acc:HGNC:26520]	19
ENSG00000083828	4,07477	1,111453	0,003823	0,019611	3,342436	protein_coding	ZNF586	zinc finger protein 586 [Source:HGNC Symbol;Acc:HGNC:25949]	19
ENSG00000254901	3,229749	0,805358	0,003846	0,0197	3,339682	protein_coding	BORCS8	BLOC-1 related complex subunit 8 [Source:HGNC Symbol;Acc:HGNC:37247]	19
ENSG00000178028	5,118206	0,656474	0,003847	0,0197	3,339621	protein_coding	DMAP1	DNA methyltransferase 1 associated protein 1 [Source:HGNC Symbol;Acc:HGNC:18291]	1
ENSG00000146063	5,289269	0,591876	0,003843	0,0197	3,340032	protein_coding	TRIM41	tripartite motif containing 41 [Source:HGNC Symbol;Acc:HGNC:19013]	5
ENSG00000100292	5,446355	-1,509234	0,003843	0,0197	-3,340076	protein_coding	HMOX1	heme oxygenase 1 [Source:HGNC Symbol;Acc:HGNC:5013]	22
ENSG00000137693	6,238377	-1,963914	0,003847	0,0197	-3,339637	protein_coding	YAP1	Yes1 associated transcriptional regulator [Source:HGNC Symbol;Acc:HGNC:16262]	11
ENSG00000197077	6,164727	0,647757	0,003848	0,019701	3,339471	protein_coding	KIAA1671	KIAA1671 [Source:HGNC Symbol;Acc:HGNC:29345]	22
ENSG00000129946	5,987266	1,218035	0,003852	0,019709	3,339043	protein_coding	SHC2	SHC adaptor protein 2 [Source:HGNC Symbol;Acc:HGNC:29869]	19
ENSG00000154380	7,843443	-0,84368	0,003852	0,019709	-3,338975	protein_coding	ENAH	ENAH actin regulator [Source:HGNC Symbol;Acc:HGNC:18271]	1
ENSG00000196670	5,063482	0,742699	0,003854	0,019714	3,338727	protein_coding	ZFP62	ZFP62 zinc finger protein [Source:HGNC Symbol;Acc:HGNC:23241]	5
ENSG00000103222	6,522973	-0,673555	0,003859	0,019731	-3,338169	protein_coding	ABCC1	ATP binding cassette subfamily C member 1 [Source:HGNC Symbol;Acc:HGNC:51]	16
ENSG00000040487	3,598433	-0,709433	0,003863	0,019749	-3,337625	protein_coding	SLC66A1	solute carrier family 66 member 1 [Source:HGNC Symbol;Acc:HGNC:26001]	1
ENSG00000196387	3,229741	-0,675516	0,003874	0,019794	-3,336423	protein_coding	ZNF140	zinc finger protein 140 [Source:HGNC Symbol;Acc:HGNC:12925]	12
ENSG00000101160	7,235115	-1,272486	0,003878	0,019809	-3,335914	protein_coding	CTSZ	cathepsin Z [Source:HGNC Symbol;Acc:HGNC:2547]	20
ENSG00000197535	6,808009	-0,652323	0,003881	0,019819	-3,335549	protein_coding	MYO5A	myosin VA [Source:HGNC Symbol;Acc:HGNC:7602]	15
ENSG00000171595	-1,239861	3,378468	0,003885	0,019825	3,335033	protein_coding	DNAI2	dynein axonemal intermediate chain 2 [Source:HGNC Symbol;Acc:HGNC:18744]	17
ENSG00000198547	-1,963194	2,651072	0,003886	0,019825	3,334971	protein_coding	C20orf203	chromosome 20 open reading frame 203 [Source:HGNC Symbol;Acc:HGNC:26592]	20
ENSG00000185551	6,787829	-1,369248	0,003885	0,019825	-3,335018	protein_coding	NR2F2	nuclear receptor subfamily 2 group F member 2 [Source:HGNC Symbol;Acc:HGNC:7976]	15
ENSG00000156313	4,052427	1,173153	0,003892	0,01985	3,334252	protein_coding	RPGR	retinitis pigmentosa GTPase regulator [Source:HGNC Symbol;Acc:HGNC:10295]	X
ENSG00000071994	5,364505	-0,751896	0,003905	0,019912	-3,332658	protein_coding	PDCD2	programmed cell death 2 [Source:HGNC Symbol;Acc:HGNC:8762]	6
ENSG00000100983	5,531496	-0,588161	0,00391	0,019927	-3,332112	protein_coding	GSS	glutathione synthetase [Source:HGNC Symbol;Acc:HGNC:4624]	20
ENSG00000233276	5,952322	-0,856518	0,003911	0,019927	-3,332017	protein_coding	GPX1	glutathione peroxidase 1 [Source:HGNC Symbol;Acc:HGNC:4553]	3
ENSG00000125910	-2,304626	2,35029	0,003916	0,019944	3,331412	protein_coding	S1PR4	sphingosine-1-phosphate receptor 4 [Source:HGNC Symbol;Acc:HGNC:3170]	19
ENSG00000187051	5,596026	-0,60464	0,003916	0,019944	-3,331343	protein_coding	RPS19BP1	ribosomal protein S19 binding protein 1 [Source:HGNC Symbol;Acc:HGNC:28749]	22
ENSG00000117069	1,499407	2,500446	0,003919	0,019945	3,331061	protein_coding	ST6GALNA	ST6 N-acetylgalactosaminide alpha-2,6-sialyltransferase 5 [Source:HGNC Symbol;Acc:HGNC:19342]	1
ENSG00000152904	2,942811	-0,946977	0,00392	0,019945	-3,330893	protein_coding	GGPS1	geranylgeranyl diphosphate synthase 1 [Source:HGNC Symbol;Acc:HGNC:4249]	1
ENSG00000108666	3,973048	0,83384	0,00392	0,019945	3,330918	protein_coding	C17orf75	chromosome 17 open reading frame 75 [Source:HGNC Symbol;Acc:HGNC:30173]	17
ENSG00000167701	-0,840388	1,596408	0,003922	0,019948	3,330684	protein_coding	GPT	glutamic--pyruvic transaminase [Source:HGNC Symbol;Acc:HGNC:4552]	8
ENSG00000149968	0,329304	-7,990673	0,003927	0,019959	-3,330134	protein_coding	MMP3	matrix metallopeptidase 3 [Source:HGNC Symbol;Acc:HGNC:7173]	11
ENSG00000142082	3,035332	0,876067	0,003926	0,019959	3,330251	protein_coding	SIRT3	sirtuin 3 [Source:HGNC Symbol;Acc:HGNC:14931]	11
ENSG00000121577	0,13848	1,47401	0,003931	0,019961	3,329647	protein_coding	POPDC2	popeye domain containing 2 [Source:HGNC Symbol;Acc:HGNC:17648]	3
ENSG00000130475	2,40888	1,583371	0,003928	0,019961	3,329931	protein_coding	FCHO1	FCH and mu domain containing endocytic adaptor 1 [Source:HGNC Symbol;Acc:HGNC:29002]	19
ENSG00000106609	6,8803	-0,525369	0,00393	0,019961	-3,329714	protein_coding	TMEM248	transmembrane protein 248 [Source:HGNC Symbol;Acc:HGNC:25476]	7
ENSG00000115687	4,78458	1,133386	0,003941	0,020009	3,328402	protein_coding	PASK	PAS domain containing serine/threonine kinase [Source:HGNC Symbol;Acc:HGNC:17270]	2
ENSG00000162994	2,851327	1,245508	0,003945	0,020018	3,328044	protein_coding	CLHC1	clathrin heavy chain linker domain containing 1 [Source:HGNC Symbol;Acc:HGNC:26453]	2
ENSG00000134042	-0,714266	2,845644	0,003946	0,020019	3,327881	protein_coding	MRO	maestro [Source:HGNC Symbol;Acc:HGNC:24121]	18
ENSG00000120129	6,063436	1,561285	0,003949	0,020028	3,327532	protein_coding	DUSP1	dual specificity phosphatase 1 [Source:HGNC Symbol;Acc:HGNC:3064]	5
ENSG00000026559	2,1762	-2,111929	0,003954	0,020043	-3,326904	protein_coding	KCNG1	potassium voltage-gated channel modifier subfamily G member 1 [Source:HGNC Symbol;Acc:HGNC:6248]	20
ENSG00000009307	8,715602	-0,795012	0,003953	0,020043	-3,327033	protein_coding	CSDE1	cold shock domain containing E1 [Source:HGNC Symbol;Acc:HGNC:29905]	1
ENSG00000170458	3,641983	2,466012	0,003957	0,020053	3,32653	protein_coding	CD14	CD14 molecule [Source:HGNC Symbol;Acc:HGNC:1628]	5
ENSG00000086967	-0,087014	3,464091	0,003966	0,020091	3,325505	protein_coding	MYBPC2	myosin binding protein C2 [Source:HGNC Symbol;Acc:HGNC:7550]	19
ENSG00000126264	-0,672992	1,831304	0,003976	0,020132	3,32438	protein_coding	HCST	hematopoietic cell signal transducer [Source:HGNC Symbol;Acc:HGNC:16977]	19
ENSG00000111540	6,684411	-0,489695	0,003977	0,020132	-3,324288	protein_coding	RAB5B	RAB5B, member RAS oncogene family [Source:HGNC Symbol;Acc:HGNC:9784]	12
ENSG00000110934	1,739845	2,134171	0,003981	0,020141	3,32382	protein_coding	BIN2	bridging integrator 2 [Source:HGNC Symbol;Acc:HGNC:1053]	12
ENSG00000145050	5,704889	-0,767543	0,003981	0,020141	-3,323785	protein_coding	MANF	mesencephalic astrocyte derived neurotrophic factor [Source:HGNC Symbol;Acc:HGNC:15461]	3

ENSG00000111490	2,159717	2,441586	0,003985	0,020155	3,323316	protein_coding	TBC1D30	TBC1 domain family member 30 [Source:HGNC Symbol;Acc:HGNC:29164]	12
ENSG00000005187	0,980178	3,843244	0,003987	0,02016	3,323058	protein_coding	ACSM3	acyl-CoA synthetase medium chain family member 3 [Source:HGNC Symbol;Acc:HGNC:10522]	16
ENSG00000152582	2,373447	2,453485	0,003991	0,020173	3,322603	protein_coding	SPEF2	sperm flagellar 2 [Source:HGNC Symbol;Acc:HGNC:26293]	5
ENSG00000166747	6,780838	-0,453638	0,003992	0,020173	-3,322483	protein_coding	AP1G1	adaptor related protein complex 1 subunit gamma 1 [Source:HGNC Symbol;Acc:HGNC:555]	16
ENSG00000123700	3,425983	1,836001	0,003996	0,020183	3,322096	protein_coding	KCNJ2	potassium inwardly rectifying channel subfamily J member 2 [Source:HGNC Symbol;Acc:HGNC:6263]	17
ENSG00000189134	0,263863	-1,565335	0,003999	0,020193	-3,321742	protein_coding	NKAPL	NFKB activating protein like [Source:HGNC Symbol;Acc:HGNC:21584]	6
ENSG00000171206	6,934944	-0,759381	0,004001	0,020198	-3,321468	protein_coding	TRIM8	tripartite motif containing 8 [Source:HGNC Symbol;Acc:HGNC:15579]	10
ENSG00000102001	-0,866535	2,705152	0,004003	0,020201	3,321274	protein_coding	CACNA1F	calcium voltage-gated channel subunit alpha1 F [Source:HGNC Symbol;Acc:HGNC:1393]	X
ENSG00000167178	1,904951	-1,37574	0,004008	0,020223	-3,320626	protein_coding	ISLR2	immunoglobulin superfamily containing leucine rich repeat 2 [Source:HGNC Symbol;Acc:HGNC:29286]	15
ENSG00000161905	-2,215414	3,335901	0,004014	0,02024	3,319981	protein_coding	ALOX15	arachidonate 15-lipoxygenase [Source:HGNC Symbol;Acc:HGNC:433]	17
ENSG00000100060	0,969952	1,789731	0,004014	0,02024	3,319941	protein_coding	MFNG	MFNG O-fucosylpeptide 3-beta-N-acetylglucosaminyltransferase [Source:HGNC Symbol;Acc:HGNC:7038]	22
ENSG00000197102	10,30673	-0,511341	0,004018	0,020254	-3,319484	protein_coding	DYNC1H1	dynein cytoplasmic 1 heavy chain 1 [Source:HGNC Symbol;Acc:HGNC:2961]	14
ENSG00000122367	0,326479	2,918514	0,004021	0,020261	3,319174	protein_coding	LDB3	LIM domain binding 3 [Source:HGNC Symbol;Acc:HGNC:15710]	10
ENSG00000111850	3,298701	0,861687	0,004024	0,020268	3,318879	protein_coding	SMIM8	small integral membrane protein 8 [Source:HGNC Symbol;Acc:HGNC:21401]	6
ENSG00000127362	-0,682856	1,540252	0,004028	0,020273	3,318346	protein_coding	TAS2R3	taste 2 receptor member 3 [Source:HGNC Symbol;Acc:HGNC:14910]	7
ENSG00000163040	3,452406	-1,411782	0,004028	0,020273	-3,318352	protein_coding	CCDC74A	coiled-coil domain containing 74A [Source:HGNC Symbol;Acc:HGNC:25197]	2
ENSG00000198740	6,926003	0,374444	0,004027	0,020273	3,31851	protein_coding	ZNF652	zinc finger protein 652 [Source:HGNC Symbol;Acc:HGNC:29147]	17
ENSG00000074964	6,030765	1,291922	0,004034	0,020293	3,317733	protein_coding	ARHGEF10	Rho guanine nucleotide exchange factor 10 like [Source:HGNC Symbol;Acc:HGNC:25540]	1
ENSG00000120903	-2,464133	3,028229	0,004037	0,020306	3,317297	protein_coding	CHRNA2	cholinergic receptor nicotinic alpha 2 subunit [Source:HGNC Symbol;Acc:HGNC:1956]	8
ENSG00000132849	6,366344	1,158937	0,00404	0,020312	3,317019	protein_coding	PATJ	PATJ crumbs cell polarity complex component [Source:HGNC Symbol;Acc:HGNC:28881]	1
ENSG00000146006	-0,168238	2,844369	0,004052	0,020366	3,315656	protein_coding	LRRTM2	leucine rich repeat transmembrane neuronal 2 [Source:HGNC Symbol;Acc:HGNC:19409]	5
ENSG00000115956	2,678383	2,206623	0,004056	0,020373	3,315199	protein_coding	PLEK	pleckstrin [Source:HGNC Symbol;Acc:HGNC:9070]	2
ENSG00000099940	5,816298	-0,566765	0,004056	0,020373	-3,315227	protein_coding	SNAP29	synaptosome associated protein 29 [Source:HGNC Symbol;Acc:HGNC:11133]	22
ENSG00000189157	0,961137	1,944966	0,004064	0,0204	3,314318	protein_coding	FAM47E	family with sequence similarity 47 member E [Source:HGNC Symbol;Acc:HGNC:34343]	4
ENSG00000160602	2,537407	0,911799	0,004062	0,0204	3,314458	protein_coding	NEK8	NIMA related kinase 8 [Source:HGNC Symbol;Acc:HGNC:13387]	17
ENSG00000178381	3,73895	-0,81932	0,004067	0,020408	-3,31399	protein_coding	ZFAND2A	zinc finger AN1-type containing 2A [Source:HGNC Symbol;Acc:HGNC:28073]	7
ENSG00000079246	8,033113	-0,604496	0,004075	0,020442	-3,313076	protein_coding	XRCC5	X-ray repair cross complementing 5 [Source:HGNC Symbol;Acc:HGNC:12833]	2
ENSG00000165555	-0,494065	1,360074	0,004084	0,020478	3,312025	protein_coding	NOXRED1	NADP dependent oxidoreductase domain containing 1 [Source:HGNC Symbol;Acc:HGNC:20487]	14
ENSG00000266714	6,147342	1,463762	0,004084	0,020478	3,311971	protein_coding	MYO15B	myosin XVB [Source:HGNC Symbol;Acc:HGNC:14083]	17
ENSG00000180479	2,718952	1,082387	0,00409	0,020498	3,31138	protein_coding	ZNF571	zinc finger protein 571 [Source:HGNC Symbol;Acc:HGNC:25000]	19
ENSG00000095970	0,802762	1,912204	0,004097	0,020527	3,310585	protein_coding	TREM2	triggering receptor expressed on myeloid cells 2 [Source:HGNC Symbol;Acc:HGNC:17761]	6
ENSG00000162461	0,988764	2,298087	0,004098	0,020529	3,310404	protein_coding	SLC25A34	solute carrier family 25 member 34 [Source:HGNC Symbol;Acc:HGNC:27653]	1
ENSG00000138036	3,80194	0,697299	0,0041	0,020532	3,310195	protein_coding	DYNC2LI1	dynein cytoplasmic 2 light intermediate chain 1 [Source:HGNC Symbol;Acc:HGNC:24595]	2
ENSG00000154262	3,397014	1,958577	0,004106	0,020549	3,309539	protein_coding	ABCA6	ATP binding cassette subfamily A member 6 [Source:HGNC Symbol;Acc:HGNC:36]	17
ENSG00000163872	6,40146	0,468404	0,004105	0,020549	3,309587	protein_coding	YEATS2	YEATS domain containing 2 [Source:HGNC Symbol;Acc:HGNC:25489]	3
ENSG00000115548	6,519865	0,653635	0,004113	0,020578	3,308732	protein_coding	KDM3A	lysine demethylase 3A [Source:HGNC Symbol;Acc:HGNC:20815]	2
ENSG00000110721	4,742782	0,934958	0,004121	0,020609	3,307892	protein_coding	CHKA	choline kinase alpha [Source:HGNC Symbol;Acc:HGNC:1937]	11
ENSG00000196136	-2,08411	4,136318	0,004125	0,020617	3,307426	protein_coding	SERPINA3	serpin family A member 3 [Source:HGNC Symbol;Acc:HGNC:16]	14
ENSG00000130414	6,544613	0,509062	0,004125	0,020617	3,307446	protein_coding	NDUFA10	NADH:ubiquinone oxidoreductase subunit A10 [Source:HGNC Symbol;Acc:HGNC:7684]	2
ENSG00000188517	-0,91461	2,510085	0,004129	0,02063	3,307006	protein_coding	COL25A1	collagen type XXV alpha 1 chain [Source:HGNC Symbol;Acc:HGNC:18603]	4
ENSG00000170476	-1,66611	3,100562	0,004132	0,020639	3,306671	protein_coding	MZB1	marginal zone B and B1 cell specific protein [Source:HGNC Symbol;Acc:HGNC:30125]	5
ENSG00000186205	2,009454	1,570718	0,004137	0,020655	3,306099	protein_coding	MTARC1	mitochondrial amidoxime reducing component 1 [Source:HGNC Symbol;Acc:HGNC:26189]	1
ENSG00000256269	4,023018	-0,622469	0,004137	0,020655	-3,306011	protein_coding	HMBS	hydroxymethylbilane synthase [Source:HGNC Symbol;Acc:HGNC:4982]	11
ENSG00000163807	4,804085	-0,754676	0,004141	0,020666	-3,305624	protein_coding	KIAA1143	KIAA1143 [Source:HGNC Symbol;Acc:HGNC:29198]	3
ENSG00000046647	3,323501	0,805756	0,004144	0,020675	3,305283	protein_coding	GEMIN8	gem nuclear organelle associated protein 8 [Source:HGNC Symbol;Acc:HGNC:26044]	X
ENSG00000133111	2,772653	0,937433	0,004146	0,020677	3,305107	protein_coding	RFXAP	regulatory factor X associated protein [Source:HGNC Symbol;Acc:HGNC:9988]	13
ENSG00000204257	2,566482	2,001991	0,004148	0,020685	3,304791	protein_coding	HLA-DMA	major histocompatibility complex, class II, DM alpha [Source:HGNC Symbol;Acc:HGNC:4934]	6
ENSG00000181409	2,713353	2,875909	0,004151	0,020691	3,3045	protein_coding	AATK	apoptosis associated tyrosine kinase [Source:HGNC Symbol;Acc:HGNC:21]	17
ENSG00000147383	4,117902	-0,731513	0,004157	0,020717	-3,303792	protein_coding	NSDHL	NAD(P) dependent steroid dehydrogenase-like [Source:HGNC Symbol;Acc:HGNC:13398]	X
ENSG00000111181	0,206052	2,523157	0,004165	0,02075	3,30291	protein_coding	SLC6A12	solute carrier family 6 member 12 [Source:HGNC Symbol;Acc:HGNC:11045]	12
ENSG00000078687	6,155058	0,759548	0,004174	0,020785	3,301989	protein_coding	TNRC6C	trinucleotide repeat containing adaptor 6C [Source:HGNC Symbol;Acc:HGNC:29318]	17

ENSG00000165923	0,618499	1,210754	0,00418	0,020808	3,301337	protein_coding	AGBL2	ATP/GTP binding protein like 2 [Source:HGNC Symbol;Acc:HGNC:26296]	11
ENSG00000163687	-0,957818	3,54709	0,004184	0,020813	3,300799	protein_coding	DNASE1L3	deoxyribonuclease 1 like 3 [Source:HGNC Symbol;Acc:HGNC:2959]	3
ENSG00000186827	-0,870839	2,795989	0,004183	0,020813	3,300907	protein_coding	TNFRSF4	TNF receptor superfamily member 4 [Source:HGNC Symbol;Acc:HGNC:11918]	1
ENSG00000283486	0,235472	2,423646	0,004183	0,020813	3,300901	protein_coding	FAM95C	family with sequence similarity 95 member C [Source:HGNC Symbol;Acc:HGNC:45272]	9
ENSG00000181481	2,76774	-1,17395	0,004206	0,020913	-3,298438	protein_coding	RNF135	ring finger protein 135 [Source:HGNC Symbol;Acc:HGNC:21158]	17
ENSG00000085449	5,195834	-0,720443	0,004212	0,020937	-3,297774	protein_coding	WDFY1	WD repeat and FYVE domain containing 1 [Source:HGNC Symbol;Acc:HGNC:20451]	2
ENSG00000103184	1,067607	2,125912	0,004218	0,020947	3,297126	protein_coding	SEC14L5	SEC14 like lipid binding 5 [Source:HGNC Symbol;Acc:HGNC:29032]	16
ENSG00000089820	3,905017	2,286181	0,004215	0,020947	3,297385	protein_coding	ARHGAP4	Rho GTPase activating protein 4 [Source:HGNC Symbol;Acc:HGNC:674]	X
ENSG00000119760	5,600659	-0,461883	0,004217	0,020947	-3,297182	protein_coding	SUPT7L	SPT7 like, STAGA complex subunit gamma [Source:HGNC Symbol;Acc:HGNC:30632]	2
ENSG00000161395	3,485195	0,842348	0,004221	0,020956	3,296731	protein_coding	PGAP3	post-GPI attachment to proteins phospholipase 3 [Source:HGNC Symbol;Acc:HGNC:23719]	17
ENSG00000148090	4,418385	0,61595	0,004222	0,020956	3,296661	protein_coding	AUH	AU RNA binding methylglutaconyl-CoA hydratase [Source:HGNC Symbol;Acc:HGNC:890]	9
ENSG00000109762	4,812279	-0,725958	0,004224	0,020958	-3,296467	protein_coding	SNX25	sorting nexin 25 [Source:HGNC Symbol;Acc:HGNC:21883]	4
ENSG00000180113	0,059185	2,339851	0,004228	0,020972	3,296014	protein_coding	TDRD6	tudor domain containing 6 [Source:HGNC Symbol;Acc:HGNC:21339]	6
ENSG00000147650	5,310697	-1,121758	0,004241	0,021031	-3,294579	protein_coding	LRP12	LDL receptor related protein 12 [Source:HGNC Symbol;Acc:HGNC:31708]	8
ENSG00000141527	-1,976782	1,796716	0,004255	0,021092	3,293099	protein_coding	CARD14	caspase recruitment domain family member 14 [Source:HGNC Symbol;Acc:HGNC:16446]	17
ENSG00000204442	3,290035	-1,863183	0,004257	0,021097	-3,292859	protein_coding	FAM155A	family with sequence similarity 155 member A [Source:HGNC Symbol;Acc:HGNC:33877]	13
ENSG00000106780	5,902497	-0,576235	0,00426	0,021104	-3,29255	protein_coding	MEGF9	multiple EGF like domains 9 [Source:HGNC Symbol;Acc:HGNC:3234]	9
ENSG00000273559	4,964989	0,558001	0,004264	0,021116	3,29215	protein_coding	CWC25	CWC25 spliceosome associated protein homolog [Source:HGNC Symbol;Acc:HGNC:25989]	17
ENSG00000106404	2,871049	0,801379	0,004266	0,021122	3,291875	protein_coding	CLDN15	claudin 15 [Source:HGNC Symbol;Acc:HGNC:2036]	7
ENSG00000150337	-0,762611	2,756099	0,004273	0,021152	3,291078	protein_coding	FCGR1A	Fc fragment of IgG receptor Ia [Source:HGNC Symbol;Acc:HGNC:3613]	1
ENSG00000142347	3,62812	1,894168	0,004278	0,021168	3,290587	protein_coding	MYO1F	myosin IF [Source:HGNC Symbol;Acc:HGNC:7600]	19
ENSG00000184702	1,825663	1,252907	0,004281	0,021169	3,290291	protein_coding	SEPTIN5	septin 5 [Source:HGNC Symbol;Acc:HGNC:9164]	22
ENSG00000154027	2,300518	-1,847431	0,00428	0,021169	-3,290367	protein_coding	AK5	adenylate kinase 5 [Source:HGNC Symbol;Acc:HGNC:365]	1
ENSG00000171045	4,443223	1,304705	0,004284	0,021177	3,289942	protein_coding	TSNARE1	t-SNARE domain containing 1 [Source:HGNC Symbol;Acc:HGNC:26437]	8
ENSG00000103249	6,250642	-0,480424	0,004285	0,021177	-3,289846	protein_coding	CLCN7	chloride voltage-gated channel 7 [Source:HGNC Symbol;Acc:HGNC:2025]	16
ENSG00000176387	1,335523	1,828947	0,004287	0,021183	3,289565	protein_coding	HSD11B2	hydroxysteroid 11-beta dehydrogenase 2 [Source:HGNC Symbol;Acc:HGNC:5209]	16
ENSG00000137834	3,889852	-1,916567	0,004291	0,021189	-3,289165	protein_coding	SMAD6	SMAD family member 6 [Source:HGNC Symbol;Acc:HGNC:6772]	15
ENSG00000062485	6,629416	-0,591095	0,00429	0,021189	-3,289274	protein_coding	CS	citrate synthase [Source:HGNC Symbol;Acc:HGNC:2422]	12
ENSG00000170876	5,672575	-0,78837	0,004294	0,021194	-3,288909	protein_coding	TMEM43	transmembrane protein 43 [Source:HGNC Symbol;Acc:HGNC:28472]	3
ENSG00000100416	5,070665	0,715213	0,004296	0,021199	3,288663	protein_coding	TRMU	tRNA mitochondrial 2-thiouridylase [Source:HGNC Symbol;Acc:HGNC:25481]	22
ENSG00000148481	4,929649	-0,682098	0,004299	0,021205	-3,288376	protein_coding	MINDY3	MINDY lysine 48 deubiquitinase 3 [Source:HGNC Symbol;Acc:HGNC:23578]	10
ENSG00000139263	4,141559	1,173054	0,004311	0,02126	3,287058	protein_coding	LRIG3	leucine rich repeats and immunoglobulin like domains 3 [Source:HGNC Symbol;Acc:HGNC:30991]	12
ENSG00000122140	4,448628	-0,581221	0,004312	0,02126	-3,286907	protein_coding	MRPS2	mitochondrial ribosomal protein S2 [Source:HGNC Symbol;Acc:HGNC:14495]	9
ENSG00000204272	4,086319	-1,09051	0,004316	0,021264	-3,286538	protein_coding	NBDY	negative regulator of P-body association [Source:HGNC Symbol;Acc:HGNC:50713]	X
ENSG00000140612	6,092489	-0,573507	0,004314	0,021264	-3,286671	protein_coding	SEC11A	SEC11 homolog A, signal peptidase complex subunit [Source:HGNC Symbol;Acc:HGNC:17718]	15
ENSG00000121318	-1,613006	1,96621	0,004322	0,021287	3,285904	protein_coding	TAS2R10	taste 2 receptor member 10 [Source:HGNC Symbol;Acc:HGNC:14918]	12
ENSG00000140543	0,538186	1,435397	0,004325	0,021289	3,285535	protein_coding	DET1	DET1 partner of COP1 E3 ubiquitin ligase [Source:HGNC Symbol;Acc:HGNC:25477]	15
ENSG00000277363	4,336808	2,217618	0,004326	0,021289	3,285431	protein_coding	SRCIN1	SRC kinase signaling inhibitor 1 [Source:HGNC Symbol;Acc:HGNC:29506]	17
ENSG00000186088	3,922452	1,239872	0,004325	0,021289	3,285565	protein_coding	GSAP	gamma-secretase activating protein [Source:HGNC Symbol;Acc:HGNC:28042]	7
ENSG00000172893	5,26815	-0,970278	0,00433	0,021304	-3,284967	protein_coding	DHCR7	7-dehydrocholesterol reductase [Source:HGNC Symbol;Acc:HGNC:2860]	11
ENSG00000005379	3,470537	4,623362	0,004337	0,021328	3,284308	protein_coding	TSPOAP1	TSPO associated protein 1 [Source:HGNC Symbol;Acc:HGNC:16831]	17
ENSG00000073756	4,270917	-4,150356	0,004339	0,021334	-3,284044	protein_coding	PTGS2	prostaglandin-endoperoxide synthase 2 [Source:HGNC Symbol;Acc:HGNC:9605]	1
ENSG00000012983	6,336595	-0,500843	0,004342	0,021343	-3,283706	protein_coding	MAP4K5	mitogen-activated protein kinase kinase kinase kinase 5 [Source:HGNC Symbol;Acc:HGNC:6867]	14
ENSG00000155666	2,256714	0,911748	0,004345	0,021352	3,283368	protein_coding	KDM8	lysine demethylase 8 [Source:HGNC Symbol;Acc:HGNC:25840]	16
ENSG00000124787	1,794936	-0,958964	0,004348	0,021359	-3,283081	protein_coding	RPP40	ribonuclease P/MRP subunit p40 [Source:HGNC Symbol;Acc:HGNC:20992]	6
ENSG00000153560	6,464485	-0,625722	0,004352	0,021371	-3,282696	protein_coding	UBP1	upstream binding protein 1 [Source:HGNC Symbol;Acc:HGNC:12507]	3
ENSG00000203499	1,234632	1,571449	0,00436	0,021399	3,281812	protein_coding	IQANK1	IQ motif and ankyrin repeat containing 1 [Source:HGNC Symbol;Acc:HGNC:49576]	8
ENSG00000254999	5,691417	-0,502869	0,004359	0,021399	-3,281882	protein_coding	BRK1	BRICK1 subunit of SCAR/WAVE actin nucleating complex [Source:HGNC Symbol;Acc:HGNC:23057]	3
ENSG00000124201	7,118223	-0,527051	0,004363	0,021407	-3,28149	protein_coding	ZNFX1	zinc finger NFX1-type containing 1 [Source:HGNC Symbol;Acc:HGNC:29271]	20
ENSG00000176225	4,615895	0,905868	0,004373	0,021447	3,280479	protein_coding	RTTN	rotatin [Source:HGNC Symbol;Acc:HGNC:18654]	18
ENSG00000149554	4,318168	-0,902928	0,004374	0,021449	-3,280314	protein_coding	CHEK1	checkpoint kinase 1 [Source:HGNC Symbol;Acc:HGNC:1925]	11

ENSG00000188266	1,885097	1,050617	0,00438	0,021472	3,279669	protein_coding	HYKK	hydroxylysine kinase [Source:HGNC Symbol;Acc:HGNC:34403]	15
ENSG00000044574	9,384238	-0,764589	0,004386	0,021487	-3,279067	protein_coding	HSPA5	heat shock protein family A (Hsp70) member 5 [Source:HGNC Symbol;Acc:HGNC:5238]	9
ENSG00000011243	6,644789	0,576437	0,004386	0,021487	3,279066	protein_coding	AKAP8L	A-kinase anchoring protein 8 like [Source:HGNC Symbol;Acc:HGNC:29857]	19
ENSG00000227345	3,135826	-0,584034	0,004392	0,021498	-3,278424	protein_coding	PARG	poly(ADP-ribose) glycohydrolase [Source:HGNC Symbol;Acc:HGNC:8605]	10
ENSG00000177707	3,993799	-0,966077	0,004392	0,021498	-3,278437	protein_coding	NECTIN3	nectin cell adhesion molecule 3 [Source:HGNC Symbol;Acc:HGNC:17664]	3
ENSG00000164951	4,918733	-0,824426	0,004392	0,021498	-3,278451	protein_coding	PDP1	pyruvate dehyrogenase phosphatase catalytic subunit 1 [Source:HGNC Symbol;Acc:HGNC:9279]	8
ENSG00000196684	1,564809	2,567351	0,004394	0,0215	3,278238	protein_coding	HSH2D	hematopoietic SH2 domain containing [Source:HGNC Symbol;Acc:HGNC:24920]	19
ENSG00000184925	0,476787	2,439323	0,004414	0,021567	3,276087	protein_coding	LCN12	lipocalin 12 [Source:HGNC Symbol;Acc:HGNC:28733]	9
ENSG00000135298	1,242311	2,428347	0,004412	0,021567	3,276358	protein_coding	ADGRB3	adhesion G protein-coupled receptor B3 [Source:HGNC Symbol;Acc:HGNC:945]	6
ENSG00000109771	1,365556	1,491245	0,004413	0,021567	3,276252	protein_coding	LRP2BP	LRP2 binding protein [Source:HGNC Symbol;Acc:HGNC:25434]	4
ENSG00000165113	3,308466	1,433524	0,004412	0,021567	3,276327	protein_coding	GKAP1	G kinase anchoring protein 1 [Source:HGNC Symbol;Acc:HGNC:17496]	9
ENSG00000168209	5,876634	1,642571	0,004414	0,021567	3,276117	protein_coding	DDIT4	DNA damage inducible transcript 4 [Source:HGNC Symbol;Acc:HGNC:24944]	10
ENSG00000134369	8,246723	-0,815555	0,004416	0,021567	-3,275969	protein_coding	NAV1	neuron navigator 1 [Source:HGNC Symbol;Acc:HGNC:15989]	1
ENSG00000175520	-2,336196	3,101024	0,004419	0,021575	3,275649	protein_coding	UBQLN3	ubiquilin 3 [Source:HGNC Symbol;Acc:HGNC:12510]	11
ENSG00000145888	-3,196161	2,103631	0,00443	0,021622	3,27443	protein_coding	GLRA1	glycine receptor alpha 1 [Source:HGNC Symbol;Acc:HGNC:4326]	5
ENSG00000159128	6,24588	-0,870397	0,004431	0,021622	-3,274377	protein_coding	IFNGR2	interferon gamma receptor 2 [Source:HGNC Symbol;Acc:HGNC:5440]	21
ENSG00000176974	4,029035	1,233137	0,004433	0,021625	3,274168	protein_coding	SHMT1	serine hydroxymethyltransferase 1 [Source:HGNC Symbol;Acc:HGNC:10850]	17
ENSG00000019144	7,888429	-1,014119	0,004435	0,021629	-3,273944	protein_coding	PHLDB1	pleckstrin homology like domain family B member 1 [Source:HGNC Symbol;Acc:HGNC:23697]	11
ENSG00000068885	4,316007	-0,685863	0,004439	0,021642	-3,273521	protein_coding	IFT80	intraflagellar transport 80 [Source:HGNC Symbol;Acc:HGNC:29262]	3
ENSG00000065518	4,892679	-0,608981	0,004443	0,021654	-3,273123	protein_coding	NDUFB4	NADH:ubiquinone oxidoreductase subunit B4 [Source:HGNC Symbol;Acc:HGNC:7699]	3
ENSG00000170522	4,361417	-0,754231	0,004446	0,021664	-3,272782	protein_coding	ELOVL6	ELOVL fatty acid elongase 6 [Source:HGNC Symbol;Acc:HGNC:15829]	4
ENSG00000161405	0,88026	3,149395	0,00445	0,021676	3,272383	protein_coding	IKZF3	IKAROS family zinc finger 3 [Source:HGNC Symbol;Acc:HGNC:13178]	17
ENSG00000124019	-1,638233	1,908848	0,004456	0,021698	3,271777	protein_coding	FAM124B	family with sequence similarity 124 member B [Source:HGNC Symbol;Acc:HGNC:26224]	2
ENSG00000162892	-0,672247	-6,883528	0,004459	0,021707	-3,271439	protein_coding	IL24	interleukin 24 [Source:HGNC Symbol;Acc:HGNC:11346]	1
ENSG00000181222	8,550914	-0,55017	0,004485	0,021829	-3,268721	protein_coding	POLR2A	RNA polymerase II subunit A [Source:HGNC Symbol;Acc:HGNC:9187]	17
ENSG00000048162	4,011768	-0,598123	0,00449	0,021847	-3,268203	protein_coding	NOP16	NOP16 nucleolar protein [Source:HGNC Symbol;Acc:HGNC:26934]	5
ENSG00000099954	0,020314	2,065344	0,004494	0,02186	3,267788	protein_coding	CECR2	CECR2 histone acetyl-lysine reader [Source:HGNC Symbol;Acc:HGNC:1840]	22
ENSG00000153291	3,557296	2,194053	0,004497	0,021865	3,267515	protein_coding	SLC25A27	solute carrier family 25 member 27 [Source:HGNC Symbol;Acc:HGNC:21065]	6
ENSG00000170037	5,532332	0,840848	0,004498	0,021865	3,267395	protein_coding	CNTROB	centrobin, centriole duplication and spindle assembly protein [Source:HGNC Symbol;Acc:HGNC:29616]	17
ENSG00000181035	3,193248	1,209287	0,004501	0,021871	3,267137	protein_coding	SLC25A42	solute carrier family 25 member 42 [Source:HGNC Symbol;Acc:HGNC:28380]	19
ENSG00000181458	3,287273	-1,066851	0,004505	0,021886	-3,266684	protein_coding	TMEM45A	transmembrane protein 45A [Source:HGNC Symbol;Acc:HGNC:25480]	3
ENSG00000118454	5,158646	-0,494859	0,004517	0,021935	-3,265506	protein_coding	ANKRD13C	ankyrin repeat domain 13C [Source:HGNC Symbol;Acc:HGNC:25374]	1
ENSG00000134070	3,242232	-1,239972	0,004521	0,021951	-3,265036	protein_coding	IRAK2	interleukin 1 receptor associated kinase 2 [Source:HGNC Symbol;Acc:HGNC:6113]	3
ENSG00000197647	2,132532	1,143582	0,004539	0,02203	3,263249	protein_coding	ZNF433	zinc finger protein 433 [Source:HGNC Symbol;Acc:HGNC:20811]	19
ENSG00000115540	3,952717	-0,634195	0,004543	0,022043	-3,262824	protein_coding	MOB4	MOB family member 4, phocein [Source:HGNC Symbol;Acc:HGNC:17261]	2
ENSG00000035687	5,367415	-0,743594	0,004545	0,022048	-3,262596	protein_coding	ADSS2	adenylosuccinate synthase 2 [Source:HGNC Symbol;Acc:HGNC:292]	1
ENSG00000141519	4,166664	1,427929	0,004559	0,022109	3,261178	protein_coding	CCDC40	coiled-coil domain containing 40 [Source:HGNC Symbol;Acc:HGNC:26090]	17
ENSG00000203943	-1,539149	1,784056	0,004566	0,022123	3,260489	protein_coding	SAMD13	sterile alpha motif domain containing 13 [Source:HGNC Symbol;Acc:HGNC:24582]	1
ENSG00000135919	6,444413	-1,841142	0,004566	0,022123	-3,26047	protein_coding	SERPINE2	serpin family E member 2 [Source:HGNC Symbol;Acc:HGNC:8951]	2
ENSG00000140937	6,697999	-1,885301	0,004566	0,022123	-3,260492	protein_coding	CDH11	cadherin 11 [Source:HGNC Symbol;Acc:HGNC:1750]	16
ENSG00000183876	0,870269	-2,360787	0,004568	0,022123	-3,260295	protein_coding	ARSI	arylsulfatase family member I [Source:HGNC Symbol;Acc:HGNC:32521]	5
ENSG00000188342	4,711044	-0,70434	0,004569	0,022123	-3,260186	protein_coding	GTF2F2	general transcription factor IIF subunit 2 [Source:HGNC Symbol;Acc:HGNC:4653]	13
ENSG00000117834	-2,040008	3,08428	0,004578	0,02213	3,259246	protein_coding	SLC5A9	solute carrier family 5 member 9 [Source:HGNC Symbol;Acc:HGNC:22146]	1
ENSG00000179921	0,219027	2,61917	0,004578	0,02213	3,259303	protein_coding	GPBAR1	G protein-coupled bile acid receptor 1 [Source:HGNC Symbol;Acc:HGNC:19680]	2
ENSG00000244165	2,542143	0,844986	0,004573	0,02213	3,259771	protein_coding	P2RY11	purinergic receptor P2Y11 [Source:HGNC Symbol;Acc:HGNC:8540]	19
ENSG00000156103	4,747501	-1,314521	0,004579	0,02213	-3,259215	protein_coding	MMP16	matrix metallopeptidase 16 [Source:HGNC Symbol;Acc:HGNC:7162]	8
ENSG00000145819	5,957474	0,975567	0,004576	0,02213	3,259464	protein_coding	ARHGAP26	Rho GTPase activating protein 26 [Source:HGNC Symbol;Acc:HGNC:17073]	5
ENSG00000163902	8,203986	-0,545281	0,004576	0,02213	-3,259439	protein_coding	RPN1	ribophorin I [Source:HGNC Symbol;Acc:HGNC:10381]	3
ENSG00000100345	11,35974	-0,895775	0,004581	0,022135	-3,258982	protein_coding	MYH9	myosin heavy chain 9 [Source:HGNC Symbol;Acc:HGNC:7579]	22
ENSG00000147166	0,41823	1,599079	0,004585	0,022146	3,258613	protein_coding	ITGB1BP2	integrin subunit beta 1 binding protein 2 [Source:HGNC Symbol;Acc:HGNC:6154]	X
ENSG00000162068	0,015018	2,380443	0,004588	0,022156	3,258259	protein_coding	NTN3	netrin 3 [Source:HGNC Symbol;Acc:HGNC:8030]	16

ENSG00000236383	-1,691579	1,637977	0,004593	0,022176	3,257716	protein_coding	CCDC200	coiled-coil domain containing 200 [Source:HGNC Symbol;Acc:HGNC:43658]	17
ENSG00000009335	7,143218	-0,562822	0,004599	0,022198	-3,25711	protein_coding	UBE3C	ubiquitin protein ligase E3C [Source:HGNC Symbol;Acc:HGNC:16803]	7
ENSG00000116497	4,524094	0,654504	0,004602	0,022203	3,25686	protein_coding	S100PBP	S100P binding protein [Source:HGNC Symbol;Acc:HGNC:25768]	1
ENSG00000270647	6,951586	0,446907	0,004611	0,022239	3,255982	protein_coding	TAF15	TATA-box binding protein associated factor 15 [Source:HGNC Symbol;Acc:HGNC:11547]	17
ENSG00000277157	4,040186	1,200294	0,004614	0,022247	3,255677	protein_coding	H4C4	H4 clustered histone 4 [Source:HGNC Symbol;Acc:HGNC:4782]	6
ENSG00000164074	3,921113	0,711399	0,004618	0,02226	3,255265	protein_coding	ABHD18	abhydrolase domain containing 18 [Source:HGNC Symbol;Acc:HGNC:26111]	4
ENSG00000178217	-1,728701	1,803953	0,004625	0,022282	3,254534	protein_coding	SH2D4B	SH2 domain containing 4B [Source:HGNC Symbol;Acc:HGNC:31440]	10
ENSG00000162711	1,667792	1,813392	0,004624	0,022282	3,254602	protein_coding	NLRP3	NLR family pyrin domain containing 3 [Source:HGNC Symbol;Acc:HGNC:16400]	1
ENSG00000163749	1,097979	1,883026	0,004637	0,02232	3,253372	protein_coding	CCDC158	coiled-coil domain containing 158 [Source:HGNC Symbol;Acc:HGNC:26374]	4
ENSG00000120889	5,442083	-1,404167	0,004634	0,02232	-3,253606	protein_coding	TNFRSF10B	TNF receptor superfamily member 10b [Source:HGNC Symbol;Acc:HGNC:11905]	8
ENSG00000129968	5,432853	-0,367215	0,004637	0,02232	-3,253349	protein_coding	ABHD17A	abhydrolase domain containing 17A, depalmitoylase [Source:HGNC Symbol;Acc:HGNC:28756]	19
ENSG00000178947	0,681212	2,680689	0,004648	0,022365	3,252278	protein_coding	SMIM10L2A	small integral membrane protein 10 like 2A [Source:HGNC Symbol;Acc:HGNC:34499]	X
ENSG00000226650	-1,65688	1,783745	0,00465	0,022369	3,252048	protein_coding	KIF4B	kinesin family member 4B [Source:HGNC Symbol;Acc:HGNC:6322]	5
ENSG00000174498	-1,400499	2,872472	0,004652	0,022371	3,251851	protein_coding	IGDCC3	immunoglobulin superfamily DCC subclass member 3 [Source:HGNC Symbol;Acc:HGNC:9700]	15
ENSG00000188649	-0,102023	1,603528	0,004654	0,022371	3,251657	protein_coding	CC2D2B	coiled-coil and C2 domain containing 2B [Source:HGNC Symbol;Acc:HGNC:31666]	10
ENSG00000116191	5,102263	0,945972	0,004655	0,022371	3,251608	protein_coding	RALGPS2	Ral GEF with PH domain and SH3 binding motif 2 [Source:HGNC Symbol;Acc:HGNC:30279]	1
ENSG00000267281	-1,367354	1,457917	0,004659	0,022384	3,251199	protein_coding	ATF7-NPFF	ATF7-NPFF readthrough [Source:HGNC Symbol;Acc:HGNC:55073]	12
ENSG00000242247	6,161328	-0,51601	0,00466	0,022384	-3,251065	protein_coding	ARFGAP3	ADP ribosylation factor GTPase activating protein 3 [Source:HGNC Symbol;Acc:HGNC:661]	22
ENSG00000176945	2,755271	1,593288	0,004664	0,022396	3,250671	protein_coding	MUC20	mucin 20, cell surface associated [Source:HGNC Symbol;Acc:HGNC:23282]	3
ENSG00000099901	6,223223	-0,666816	0,00467	0,022417	-3,250099	protein_coding	RANBP1	RAN binding protein 1 [Source:HGNC Symbol;Acc:HGNC:9847]	22
ENSG00000130313	4,722222	-0,577012	0,004672	0,022423	-3,249849	protein_coding	PGLS	6-phosphogluconolactonase [Source:HGNC Symbol;Acc:HGNC:8903]	19
ENSG00000188015	-0,587529	-3,053401	0,004675	0,022429	-3,249588	protein_coding	S100A3	S100 calcium binding protein A3 [Source:HGNC Symbol;Acc:HGNC:10493]	1
ENSG00000074527	5,101271	-1,839076	0,004678	0,022435	-3,249318	protein_coding	NTN4	netrin 4 [Source:HGNC Symbol;Acc:HGNC:13658]	12
ENSG00000080822	4,832916	-0,553992	0,004682	0,02245	-3,248876	protein_coding	CLDND1	claudin domain containing 1 [Source:HGNC Symbol;Acc:HGNC:1322]	3
ENSG00000168096	4,088235	0,61963	0,004685	0,022457	3,248598	protein_coding	ANKS3	ankyrin repeat and sterile alpha motif domain containing 3 [Source:HGNC Symbol;Acc:HGNC:29422]	16
ENSG00000173156	1,005174	-2,380536	0,00469	0,022471	-3,248048	protein_coding	RHOD	ras homolog family member D [Source:HGNC Symbol;Acc:HGNC:670]	11
ENSG00000177971	4,367726	-0,749112	0,00469	0,022471	-3,248069	protein_coding	IMP3	IMP U3 small nucleolar ribonucleoprotein 3 [Source:HGNC Symbol;Acc:HGNC:14497]	15
ENSG00000205038	0,79757	3,518749	0,004695	0,022484	3,247634	protein_coding	PKHD1L1	PKHD1 like 1 [Source:HGNC Symbol;Acc:HGNC:20313]	8
ENSG00000197646	1,132536	-1,79476	0,004702	0,022514	-3,246875	protein_coding	PDCD1LG2	programmed cell death 1 ligand 2 [Source:HGNC Symbol;Acc:HGNC:18731]	9
ENSG00000121104	3,922716	0,932444	0,004708	0,022524	3,246283	protein_coding	FAM117A	family with sequence similarity 117 member A [Source:HGNC Symbol;Acc:HGNC:24179]	17
ENSG00000183696	4,199716	-1,129646	0,00471	0,022524	-3,246127	protein_coding	UPP1	uridine phosphorylase 1 [Source:HGNC Symbol;Acc:HGNC:12576]	7
ENSG00000039523	6,459643	-0,590009	0,004708	0,022524	-3,246368	protein_coding	RIPOR1	RHO family interacting cell polarization regulator 1 [Source:HGNC Symbol;Acc:HGNC:25836]	16
ENSG00000186111	6,77308	-0,516407	0,004709	0,022524	-3,24622	protein_coding	PIP5K1C	phosphatidylinositol-4-phosphate 5-kinase type 1 gamma [Source:HGNC Symbol;Acc:HGNC:8996]	19
ENSG00000151617	4,350822	-1,26752	0,004714	0,022536	-3,245746	protein_coding	EDNRA	endothelin receptor type A [Source:HGNC Symbol;Acc:HGNC:3179]	4
ENSG00000164209	5,757258	-0,728556	0,004717	0,022544	-3,245442	protein_coding	SLC25A46	solute carrier family 25 member 46 [Source:HGNC Symbol;Acc:HGNC:25198]	5
ENSG00000048540	-0,825686	3,227622	0,004723	0,02256	3,244859	protein_coding	LMO3	LIM domain only 3 [Source:HGNC Symbol;Acc:HGNC:6643]	12
ENSG00000114654	1,15893	2,214781	0,004722	0,02256	3,244952	protein_coding	EFCC1	EF-hand and coiled-coil domain containing 1 [Source:HGNC Symbol;Acc:HGNC:25692]	3
ENSG00000110841	7,159682	-0,81026	0,004729	0,022582	-3,244258	protein_coding	PPFIBP1	PPFIA binding protein 1 [Source:HGNC Symbol;Acc:HGNC:9249]	12
ENSG00000112078	6,533844	-0,626245	0,004734	0,022601	-3,243748	protein_coding	KCTD20	potassium channel tetramerization domain containing 20 [Source:HGNC Symbol;Acc:HGNC:21052]	6
ENSG00000140848	4,39801	-0,703859	0,004741	0,022625	-3,24312	protein_coding	CPNE2	copine 2 [Source:HGNC Symbol;Acc:HGNC:2315]	16
ENSG00000114790	3,581548	2,111799	0,004746	0,022642	3,242633	protein_coding	ARHGEF26	Rho guanine nucleotide exchange factor 26 [Source:HGNC Symbol;Acc:HGNC:24490]	3
ENSG00000185130	3,431113	1,261127	0,004749	0,022642	3,242356	protein_coding	H2BC13	H2B clustered histone 13 [Source:HGNC Symbol;Acc:HGNC:4748]	6
ENSG00000151748	5,140607	-0,666005	0,004749	0,022642	-3,242361	protein_coding	SAV1	salvador family WW domain containing protein 1 [Source:HGNC Symbol;Acc:HGNC:17795]	14
ENSG00000107372	7,383787	-0,662798	0,004754	0,02266	-3,24186	protein_coding	ZFAND5	zinc finger AN1-type containing 5 [Source:HGNC Symbol;Acc:HGNC:13008]	9
ENSG00000155438	5,025197	-0,846357	0,00476	0,022683	-3,24124	protein_coding	NIFK	nucleolar protein interacting with the FHA domain of MKI67 [Source:HGNC Symbol;Acc:HGNC:17838]	2
ENSG00000173611	4,14186	0,786109	0,004772	0,022731	3,240127	protein_coding	SCAI	suppressor of cancer cell invasion [Source:HGNC Symbol;Acc:HGNC:26709]	9
ENSG00000174007	1,894342	-0,927568	0,00478	0,022758	-3,239337	protein_coding	CEP19	centrosomal protein 19 [Source:HGNC Symbol;Acc:HGNC:28209]	3
ENSG00000188747	2,525568	1,634233	0,00478	0,022758	3,239319	protein_coding	NOXA1	NADPH oxidase activator 1 [Source:HGNC Symbol;Acc:HGNC:10668]	9
ENSG00000285269	-2,180563	1,996022	0,004781	0,022758	3,239179	protein_coding	AL160269.1	solute carrier family 35 member D2 [Source:NCBI gene (formerly Entrezgene);Acc:11046]	9
ENSG00000179178	2,260241	1,75201	0,004789	0,022778	3,238437	protein_coding	TMEM125	transmembrane protein 125 [Source:HGNC Symbol;Acc:HGNC:28275]	1
ENSG00000019485	4,035473	0,837116	0,00479	0,022778	3,238376	protein_coding	PRDM11	PR/SET domain 11 [Source:HGNC Symbol;Acc:HGNC:13996]	11

ENSG00000120686	6,508534	-0,53253	0,004787	0,022778	-3,238631	protein_coding	UFM1	ubiquitin fold modifier 1 [Source:HGNC Symbol;Acc:HGNC:20597]	13
ENSG00000143553	3,58218	-0,749649	0,004794	0,02279	-3,237997	protein_coding	SNAPIN	SNAP associated protein [Source:HGNC Symbol;Acc:HGNC:17145]	1
ENSG00000143869	0,188642	2,087741	0,004798	0,022806	3,237531	protein_coding	GDF7	growth differentiation factor 7 [Source:HGNC Symbol;Acc:HGNC:4222]	2
ENSG00000100387	4,016044	-0,579364	0,004809	0,022846	-3,236553	protein_coding	RBX1	ring-box 1 [Source:HGNC Symbol;Acc:HGNC:9928]	22
ENSG00000177030	5,03319	0,38916	0,00481	0,022846	3,236437	protein_coding	DEAF1	DEAF1 transcription factor [Source:HGNC Symbol;Acc:HGNC:14677]	11
ENSG00000114670	2,855312	1,003264	0,004814	0,022862	3,235985	protein_coding	NEK11	NIMA related kinase 11 [Source:HGNC Symbol;Acc:HGNC:18593]	3
ENSG00000103888	6,164748	-2,631459	0,004832	0,02294	-3,234271	protein_coding	CEMIP	cell migration inducing hyaluronidase 1 [Source:HGNC Symbol;Acc:HGNC:29213]	15
ENSG00000183773	1,726203	2,468136	0,004841	0,022976	3,233415	protein_coding	AIFM3	apoptosis inducing factor mitochondria associated 3 [Source:HGNC Symbol;Acc:HGNC:26398]	22
ENSG00000213397	0,433023	1,289217	0,004846	0,022985	3,232971	protein_coding	HAUS7	HAUS augmin like complex subunit 7 [Source:HGNC Symbol;Acc:HGNC:32979]	X
ENSG00000162924	4,299088	1,206881	0,004845	0,022985	3,233023	protein_coding	REL	REL proto-oncogene, NF-kB subunit [Source:HGNC Symbol;Acc:HGNC:9954]	2
ENSG00000122877	4,179766	-1,786503	0,00486	0,023043	-3,231661	protein_coding	EGR2	early growth response 2 [Source:HGNC Symbol;Acc:HGNC:3239]	10
ENSG00000141428	3,12941	-0,757879	0,004878	0,023123	-3,229915	protein_coding	C18orf21	chromosome 18 open reading frame 21 [Source:HGNC Symbol;Acc:HGNC:28802]	18
ENSG00000150768	5,26036	-0,671942	0,004883	0,023142	-3,229411	protein_coding	DLAT	dihydrolipoamide S-acetyltransferase [Source:HGNC Symbol;Acc:HGNC:2896]	11
ENSG00000187510	-1,236933	2,944126	0,004892	0,023178	3,228555	protein_coding	PLEKHG7	pleckstrin homology and RhoGEF domain containing G7 [Source:HGNC Symbol;Acc:HGNC:33829]	12
ENSG00000105717	0,321764	2,364935	0,004901	0,023199	3,227728	protein_coding	PBX4	PBX homeobox 4 [Source:HGNC Symbol;Acc:HGNC:13403]	19
ENSG00000152207	1,07438	2,916451	0,004901	0,023199	3,227725	protein_coding	CYSLTR2	cysteinyl leukotriene receptor 2 [Source:HGNC Symbol;Acc:HGNC:18274]	13
ENSG00000170145	6,493434	0,503305	0,0049	0,023199	3,227814	protein_coding	SIK2	salt inducible kinase 2 [Source:HGNC Symbol;Acc:HGNC:21680]	11
ENSG00000267680	5,106129	0,724845	0,004911	0,023239	3,226797	protein_coding	ZNF224	zinc finger protein 224 [Source:HGNC Symbol;Acc:HGNC:13017]	19
ENSG00000159214	3,001361	1,256131	0,004915	0,023253	3,226391	protein_coding	CCDC24	coiled-coil domain containing 24 [Source:HGNC Symbol;Acc:HGNC:28688]	1
ENSG00000144671	-1,742031	1,883569	0,004917	0,023254	3,226226	protein_coding	SLC22A14	solute carrier family 22 member 14 [Source:HGNC Symbol;Acc:HGNC:8495]	3
ENSG00000135821	7,82887	1,087757	0,004921	0,023264	3,225892	protein_coding	GLUL	glutamate-ammonia ligase [Source:HGNC Symbol;Acc:HGNC:4341]	1
ENSG00000125510	0,758053	1,732844	0,004931	0,023301	3,224903	protein_coding	OPRL1	opioid related nociceptin receptor 1 [Source:HGNC Symbol;Acc:HGNC:8155]	20
ENSG00000100568	6,029575	-0,631177	0,00493	0,023301	-3,224978	protein_coding	VTI1B	vesicle transport through interaction with t-SNAREs 1B [Source:HGNC Symbol;Acc:HGNC:17793]	14
ENSG00000154359	4,470354	0,830621	0,004934	0,023307	3,224631	protein_coding	LONRF1	LON peptidase N-terminal domain and ring finger 1 [Source:HGNC Symbol;Acc:HGNC:26302]	8
ENSG00000110777	-0,176509	3,804563	0,004938	0,023318	3,224278	protein_coding	POU2AF1	POU class 2 homeobox associating factor 1 [Source:HGNC Symbol;Acc:HGNC:9211]	11
ENSG00000172613	3,488766	0,745462	0,004944	0,023343	3,223657	protein_coding	RAD9A	RAD9 checkpoint clamp component A [Source:HGNC Symbol;Acc:HGNC:9827]	11
ENSG00000136933	3,8511	-0,493318	0,004948	0,02335	-3,223294	protein_coding	RABEPK	Rab9 effector protein with kelch motifs [Source:HGNC Symbol;Acc:HGNC:16896]	9
ENSG00000116898	5,180283	-0,65949	0,00495	0,02335	-3,223112	protein_coding	MRPS15	mitochondrial ribosomal protein S15 [Source:HGNC Symbol;Acc:HGNC:14504]	1
ENSG00000171992	6,399702	1,527433	0,004949	0,02335	3,223241	protein_coding	SYNPO	synaptopodin [Source:HGNC Symbol;Acc:HGNC:30672]	5
ENSG00000163273	-0,111284	-2,647166	0,004952	0,023354	-3,222904	protein_coding	NPPC	natriuretic peptide C [Source:HGNC Symbol;Acc:HGNC:7941]	2
ENSG00000221968	5,078595	-1,021467	0,004956	0,023363	-3,222596	protein_coding	FADS3	fatty acid desaturase 3 [Source:HGNC Symbol;Acc:HGNC:3576]	11
ENSG00000131051	8,405089	0,573025	0,004966	0,023403	3,221668	protein_coding	RBM39	RNA binding motif protein 39 [Source:HGNC Symbol;Acc:HGNC:15923]	20
ENSG00000134815	5,011016	0,703655	0,004969	0,023414	3,221317	protein_coding	DHX34	DExH-box helicase 34 [Source:HGNC Symbol;Acc:HGNC:16719]	19
ENSG00000101336	2,780359	1,742238	0,004988	0,023492	3,219581	protein_coding	HCK	HCK proto-oncogene, Src family tyrosine kinase [Source:HGNC Symbol;Acc:HGNC:4840]	20
ENSG00000182173	4,61723	0,982493	0,004989	0,023492	3,219509	protein_coding	TSEN54	tRNA splicing endonuclease subunit 54 [Source:HGNC Symbol;Acc:HGNC:27561]	17
ENSG00000182308	-0,842769	1,779296	0,004993	0,023502	3,219157	protein_coding	DCAF4L1	DDB1 and CUL4 associated factor 4 like 1 [Source:HGNC Symbol;Acc:HGNC:27723]	4
ENSG00000197872	3,447452	1,455665	0,004994	0,023502	3,21904	protein_coding	CYRIA	CYFIP related Rac1 interactor A [Source:HGNC Symbol;Acc:HGNC:25373]	2
ENSG00000175265	4,900213	1,583168	0,004999	0,023518	3,218591	protein_coding	GOLGA8A	golgin A8 family member A [Source:HGNC Symbol;Acc:HGNC:31972]	15
ENSG00000143337	6,54716	-0,879769	0,005006	0,023546	-3,21791	protein_coding	TOR1AIP1	torsin 1A interacting protein 1 [Source:HGNC Symbol;Acc:HGNC:29456]	1
ENSG00000274290	3,631173	0,878886	0,005014	0,023576	3,217185	protein_coding	H2BC6	H2B clustered histone 6 [Source:HGNC Symbol;Acc:HGNC:4753]	6
ENSG00000189164	3,143755	0,783021	0,005022	0,023602	3,216408	protein_coding	ZNF527	zinc finger protein 527 [Source:HGNC Symbol;Acc:HGNC:29385]	19
ENSG00000119328	3,063296	-0,846448	0,005022	0,023602	-3,216405	protein_coding	ABITRAM	actin binding transcription modulator [Source:HGNC Symbol;Acc:HGNC:1364]	9
ENSG00000147257	1,080786	3,642123	0,005036	0,023661	3,215115	protein_coding	GPC3	glypican 3 [Source:HGNC Symbol;Acc:HGNC:4451]	X
ENSG00000088970	4,816807	0,977959	0,005043	0,023683	3,21455	protein_coding	KIZ	kizuna centrosomal protein [Source:HGNC Symbol;Acc:HGNC:15865]	20
ENSG00000174721	1,113089	1,268569	0,005045	0,023688	3,214307	protein_coding	FGFBP3	fibroblast growth factor binding protein 3 [Source:HGNC Symbol;Acc:HGNC:23428]	10
ENSG00000127083	-2,612929	3,58134	0,005057	0,023729	3,213252	protein_coding	OMD	osteomodulin [Source:HGNC Symbol;Acc:HGNC:8134]	9
ENSG00000136449	0,510785	2,088118	0,005056	0,023729	3,213309	protein_coding	MYCBPAP	MYCBP associated protein [Source:HGNC Symbol;Acc:HGNC:19677]	17
ENSG00000128641	7,141586	-0,879492	0,00506	0,023736	-3,21298	protein_coding	MYO1B	myosin IB [Source:HGNC Symbol;Acc:HGNC:7596]	2
ENSG00000196757	3,907095	0,691794	0,005063	0,023742	3,212728	protein_coding	ZNF700	zinc finger protein 700 [Source:HGNC Symbol;Acc:HGNC:25292]	19
ENSG00000146755	-2,881077	3,770741	0,005067	0,023758	3,212289	protein_coding	TRIM50	tripartite motif containing 50 [Source:HGNC Symbol;Acc:HGNC:19017]	7
ENSG00000079785	6,604105	-0,652557	0,005078	0,023801	-3,211314	protein_coding	DDX1	DEAD-box helicase 1 [Source:HGNC Symbol;Acc:HGNC:2734]	2

ENSG00000113312	5,649821	-0,478056	0,005083	0,023815	-3,210899	protein_coding	TTC1	tetratricopeptide repeat domain 1 [Source:HGNC Symbol;Acc:HGNC:12391]	5
ENSG00000139547	-1,457143	2,191824	0,005092	0,023853	3,210032	protein_coding	RDH16	retinol dehydrogenase 16 [Source:HGNC Symbol;Acc:HGNC:29674]	12
ENSG00000043039	-2,346895	2,537421	0,00511	0,02393	3,208404	protein_coding	BARX2	BARX homeobox 2 [Source:HGNC Symbol;Acc:HGNC:956]	11
ENSG00000146476	4,28091	-0,720648	0,005117	0,023957	-3,207744	protein_coding	ARMT1	acidic residue methyltransferase 1 [Source:HGNC Symbol;Acc:HGNC:17872]	6
ENSG00000185189	4,758839	0,696841	0,005121	0,023968	3,207401	protein_coding	NRBP2	nuclear receptor binding protein 2 [Source:HGNC Symbol;Acc:HGNC:19339]	8
ENSG00000181656	-2,791298	2,409057	0,00513	0,024003	3,206591	protein_coding	GPR88	G protein-coupled receptor 88 [Source:HGNC Symbol;Acc:HGNC:4539]	1
ENSG00000213903	3,279029	1,229362	0,005135	0,024018	3,206176	protein_coding	LTB4R	leukotriene B4 receptor [Source:HGNC Symbol;Acc:HGNC:6713]	14
ENSG00000105755	3,478884	-0,89007	0,00514	0,024034	-3,205728	protein_coding	ETHE1	ETHE1 persulfide dioxygenase [Source:HGNC Symbol;Acc:HGNC:23287]	19
ENSG00000119414	5,63975	-0,533918	0,005144	0,024045	-3,205378	protein_coding	PPP6C	protein phosphatase 6 catalytic subunit [Source:HGNC Symbol;Acc:HGNC:9323]	9
ENSG00000131725	5,426038	-0,618502	0,005149	0,024046	-3,204859	protein_coding	WDR44	WD repeat domain 44 [Source:HGNC Symbol;Acc:HGNC:30512]	X
ENSG00000123607	5,634904	0,587349	0,005147	0,024046	3,205062	protein_coding	TTC21B	tetratricopeptide repeat domain 21B [Source:HGNC Symbol;Acc:HGNC:25660]	2
ENSG00000163320	6,134878	-0,675232	0,005149	0,024046	-3,204843	protein_coding	CGGBP1	CGG triplet repeat binding protein 1 [Source:HGNC Symbol;Acc:HGNC:1888]	3
ENSG00000134109	6,346427	-0,682513	0,005148	0,024046	-3,205002	protein_coding	EDEM1	ER degradation enhancing alpha-mannosidase like protein 1 [Source:HGNC Symbol;Acc:HGNC:18967]	3
ENSG00000119777	6,416447	-0,612527	0,005164	0,024102	-3,203496	protein_coding	TMEM214	transmembrane protein 214 [Source:HGNC Symbol;Acc:HGNC:25983]	2
ENSG00000110917	7,844552	-0,454615	0,005164	0,024102	-3,203521	protein_coding	MLEC	malectin [Source:HGNC Symbol;Acc:HGNC:28973]	12
ENSG00000106785	4,684458	0,974047	0,005169	0,024115	3,203117	protein_coding	TRIM14	tripartite motif containing 14 [Source:HGNC Symbol;Acc:HGNC:16283]	9
ENSG00000150873	-1,502096	2,195518	0,005175	0,024136	3,202563	protein_coding	C2orf50	chromosome 2 open reading frame 50 [Source:HGNC Symbol;Acc:HGNC:26324]	2
ENSG00000182261	-2,275768	-2,386991	0,005189	0,024193	-3,201342	protein_coding	NLRP10	NLR family pyrin domain containing 10 [Source:HGNC Symbol;Acc:HGNC:21464]	11
ENSG00000285589	2,000107	0,983478	0,005192	0,024203	3,201028	protein_coding	AC010422.	novel transcript	19
ENSG00000145107	-1,815675	-2,395463	0,005194	0,024205	-3,200846	protein_coding	TM4SF19	transmembrane 4 L six family member 19 [Source:HGNC Symbol;Acc:HGNC:25167]	3
ENSG00000172771	0,231478	2,289215	0,0052	0,024221	3,200287	protein_coding	EFCAB12	EF-hand calcium binding domain 12 [Source:HGNC Symbol;Acc:HGNC:28061]	3
ENSG00000198042	5,183474	-0,641794	0,0052	0,024221	-3,200309	protein_coding	MAK16	MAK16 homolog [Source:HGNC Symbol;Acc:HGNC:13703]	8
ENSG00000132128	5,778539	-0,459665	0,005205	0,024234	-3,199901	protein_coding	LRRC41	leucine rich repeat containing 41 [Source:HGNC Symbol;Acc:HGNC:16917]	1
ENSG00000137507	6,056283	-1,119647	0,00521	0,024252	-3,199412	protein_coding	LRRC32	leucine rich repeat containing 32 [Source:HGNC Symbol;Acc:HGNC:4161]	11
ENSG00000141994	4,430666	0,608308	0,005216	0,024266	3,198928	protein_coding	DUS3L	dihydrouridine synthase 3 like [Source:HGNC Symbol;Acc:HGNC:26920]	19
ENSG00000186166	4,55048	0,943083	0,005216	0,024266	3,198894	protein_coding	CENATAC	centrosomal AT-AC splicing factor [Source:HGNC Symbol;Acc:HGNC:30460]	11
ENSG00000117425	1,537288	2,276043	0,005218	0,024266	3,19876	protein_coding	PTCH2	patched 2 [Source:HGNC Symbol;Acc:HGNC:9586]	1
ENSG00000166170	5,991724	-0,442762	0,005223	0,024282	-3,198316	protein_coding	BAG5	BAG cochaperone 5 [Source:HGNC Symbol;Acc:HGNC:941]	14
ENSG00000132510	7,075472	0,709893	0,005236	0,024338	3,197128	protein_coding	KDM6B	lysine demethylase 6B [Source:HGNC Symbol;Acc:HGNC:29012]	17
ENSG00000078808	7,197071	-0,723345	0,005259	0,024436	-3,195127	protein_coding	SDF4	stromal cell derived factor 4 [Source:HGNC Symbol;Acc:HGNC:24188]	1
ENSG00000181666	4,990356	0,635269	0,005262	0,024444	3,194833	protein_coding	ZNF875	zinc finger protein 875 [Source:HGNC Symbol;Acc:HGNC:4928]	19
ENSG00000188263	-2,757289	2,672155	0,005273	0,02448	3,193886	protein_coding	IL17REL	interleukin 17 receptor E like [Source:HGNC Symbol;Acc:HGNC:33808]	22
ENSG00000164180	4,318618	0,694613	0,005272	0,02448	3,193938	protein_coding	TMEM161I	transmembrane protein 161B [Source:HGNC Symbol;Acc:HGNC:28483]	5
ENSG00000183837	0,828622	3,759753	0,005282	0,024504	3,193064	protein_coding	PNMA3	PNMA family member 3 [Source:HGNC Symbol;Acc:HGNC:18742]	X
ENSG00000131477	1,788261	2,322962	0,005284	0,024504	3,192906	protein_coding	RAMP2	receptor activity modifying protein 2 [Source:HGNC Symbol;Acc:HGNC:9844]	17
ENSG00000105639	4,041297	1,638458	0,005282	0,024504	3,19306	protein_coding	JAK3	Janus kinase 3 [Source:HGNC Symbol;Acc:HGNC:6193]	19
ENSG00000140400	6,064487	0,694489	0,005283	0,024504	3,192966	protein_coding	MAN2C1	mannosidase alpha class 2C member 1 [Source:HGNC Symbol;Acc:HGNC:6827]	15
ENSG00000177051	4,702578	0,828784	0,005287	0,024514	3,192589	protein_coding	FBXO46	F-box protein 46 [Source:HGNC Symbol;Acc:HGNC:25069]	19
ENSG00000007129	-1,678409	2,393752	0,005293	0,024529	3,192118	protein_coding	CEACAM21	CEA cell adhesion molecule 21 [Source:HGNC Symbol;Acc:HGNC:28834]	19
ENSG00000126822	6,78797	1,064131	0,005294	0,024529	3,192031	protein_coding	PLEKHG3	pleckstrin homology and RhoGEF domain containing G3 [Source:HGNC Symbol;Acc:HGNC:20364]	14
ENSG00000168802	2,620386	-0,69082	0,005301	0,024554	-3,19144	protein_coding	CHTF8	chromosome transmission fidelity factor 8 [Source:HGNC Symbol;Acc:HGNC:24353]	16
ENSG00000164543	4,966903	-1,355632	0,00531	0,024591	-3,1906	protein_coding	STK17A	serine/threonine kinase 17a [Source:HGNC Symbol;Acc:HGNC:11395]	7
ENSG00000145979	3,633484	-0,720822	0,005313	0,024596	-3,190389	protein_coding	TBC1D7	TBC1 domain family member 7 [Source:HGNC Symbol;Acc:HGNC:21066]	6
ENSG00000197050	3,58615	0,630502	0,005321	0,024628	3,189641	protein_coding	ZNF420	zinc finger protein 420 [Source:HGNC Symbol;Acc:HGNC:20649]	19
ENSG00000110925	5,664614	-0,659826	0,005329	0,024649	-3,18898	protein_coding	CSRNP2	cysteine and serine rich nuclear protein 2 [Source:HGNC Symbol;Acc:HGNC:16006]	12
ENSG00000086598	8,102547	-0,642961	0,005328	0,024649	-3,189055	protein_coding	TMED2	transmembrane p24 trafficking protein 2 [Source:HGNC Symbol;Acc:HGNC:16996]	12
ENSG00000132874	-2,67055	2,143804	0,005349	0,024726	3,187251	protein_coding	SLC14A2	solute carrier family 14 member 2 [Source:HGNC Symbol;Acc:HGNC:10919]	18
ENSG00000116791	4,61233	-0,919694	0,00535	0,024726	-3,187153	protein_coding	CRYZ	crystallin zeta [Source:HGNC Symbol;Acc:HGNC:2419]	1
ENSG00000112210	4,752407	-0,863042	0,005348	0,024726	-3,187279	protein_coding	RAB23	RAB23, member RAS oncogene family [Source:HGNC Symbol;Acc:HGNC:14263]	6
ENSG00000055163	6,212024	0,998379	0,005354	0,024739	3,186763	protein_coding	CYFIP2	cytoplasmic FMR1 interacting protein 2 [Source:HGNC Symbol;Acc:HGNC:13760]	5
ENSG00000187257	5,46396	0,622531	0,005361	0,024761	3,18623	protein_coding	RSBN1L	round spermatid basic protein 1 like [Source:HGNC Symbol;Acc:HGNC:24765]	7

ENSG00000198933	3,637271	1,03399	0,005375	0,024822	3,184947	protein_coding	TBKBP1	TBK1 binding protein 1 [Source:HGNC Symbol;Acc:HGNC:30140]	17
ENSG00000197747	6,43196	-1,362234	0,005381	0,024843	-3,184433	protein_coding	S100A10	S100 calcium binding protein A10 [Source:HGNC Symbol;Acc:HGNC:10487]	1
ENSG00000155761	0,350727	2,32358	0,005391	0,02488	3,183611	protein_coding	SPAG17	sperm associated antigen 17 [Source:HGNC Symbol;Acc:HGNC:26620]	1
ENSG00000033122	-0,903073	3,065162	0,005404	0,024918	3,182476	protein_coding	LRRC7	leucine rich repeat containing 7 [Source:HGNC Symbol;Acc:HGNC:18531]	1
ENSG00000164898	-0,055798	1,052886	0,005401	0,024918	3,182756	protein_coding	FMC1	formation of mitochondrial complex V assembly factor 1 homolog [Source:HGNC Symbol;Acc:HGNC:26946]	7
ENSG00000176788	6,375987	-1,62614	0,005404	0,024918	-3,182491	protein_coding	BASP1	brain abundant membrane attached signal protein 1 [Source:HGNC Symbol;Acc:HGNC:957]	5
ENSG00000196367	7,874533	0,430644	0,005405	0,024918	3,182375	protein_coding	TRRAP	transformation/transcription domain associated protein [Source:HGNC Symbol;Acc:HGNC:12347]	7
ENSG00000198075	0,165339	3,103907	0,005412	0,024934	3,181807	protein_coding	SULT1C4	sulfotransferase family 1C member 4 [Source:HGNC Symbol;Acc:HGNC:11457]	2
ENSG00000142949	7,154031	0,959789	0,005411	0,024934	3,181862	protein_coding	PTPRF	protein tyrosine phosphatase receptor type F [Source:HGNC Symbol;Acc:HGNC:9670]	1
ENSG00000115129	4,512666	-1,04045	0,005415	0,02494	-3,181573	protein_coding	TP53I3	tumor protein p53 inducible protein 3 [Source:HGNC Symbol;Acc:HGNC:19373]	2
ENSG00000111834	0,008994	1,989469	0,005423	0,02497	3,180881	protein_coding	RSPH4A	radial spoke head component 4A [Source:HGNC Symbol;Acc:HGNC:21558]	6
ENSG00000137819	2,148411	-4,059603	0,005425	0,024973	-3,180699	protein_coding	PAQR5	progestin and adipoQ receptor family member 5 [Source:HGNC Symbol;Acc:HGNC:29645]	15
ENSG00000153944	6,79981	0,87015	0,005432	0,024999	3,180087	protein_coding	MSI2	musashi RNA binding protein 2 [Source:HGNC Symbol;Acc:HGNC:18585]	17
ENSG00000113716	6,668246	-0,466431	0,005441	0,025033	-3,179319	protein_coding	HMGXB3	HMG-box containing 3 [Source:HGNC Symbol;Acc:HGNC:28982]	5
ENSG00000141452	5,23962	-0,71504	0,005447	0,025053	-3,178813	protein_coding	RMC1	regulator of MON1-CCZ1 [Source:HGNC Symbol;Acc:HGNC:24326]	18
ENSG00000161618	4,280333	0,710546	0,005449	0,025057	3,178617	protein_coding	ALDH16A1	aldehyde dehydrogenase 16 family member A1 [Source:HGNC Symbol;Acc:HGNC:28114]	19
ENSG00000183160	3,282973	-2,521985	0,005457	0,025076	-3,178001	protein_coding	TMEM119	transmembrane protein 119 [Source:HGNC Symbol;Acc:HGNC:27884]	12
ENSG00000147852	5,873406	1,3215	0,005456	0,025076	3,178007	protein_coding	VLDLR	very low density lipoprotein receptor [Source:HGNC Symbol;Acc:HGNC:12698]	9
ENSG00000107758	5,569857	-0,469161	0,005465	0,025107	-3,177293	protein_coding	PPP3CB	protein phosphatase 3 catalytic subunit beta [Source:HGNC Symbol;Acc:HGNC:9315]	10
ENSG00000251201	-0,56453	-1,186407	0,00547	0,025124	-3,17685	protein_coding	TMED7-TIC	TMED7-TICAM2 readthrough [Source:HGNC Symbol;Acc:HGNC:33945]	5
ENSG00000141012	5,012988	-0,483029	0,005478	0,025154	-3,176171	protein_coding	GALNS	galactosamine (N-acetyl)-6-sulfatase [Source:HGNC Symbol;Acc:HGNC:4122]	16
ENSG00000013375	6,254759	-0,818821	0,005489	0,025198	-3,175226	protein_coding	PGM3	phosphoglucomutase 3 [Source:HGNC Symbol;Acc:HGNC:8907]	6
ENSG00000126351	6,084615	0,800655	0,005494	0,02521	3,17487	protein_coding	THRA	thyroid hormone receptor alpha [Source:HGNC Symbol;Acc:HGNC:11796]	17
ENSG00000176428	1,421197	1,293351	0,005496	0,025214	3,174675	protein_coding	VPS37D	VPS37D subunit of ESCRT-I [Source:HGNC Symbol;Acc:HGNC:18287]	7
ENSG00000152520	5,68874	0,677258	0,005505	0,02525	3,173869	protein_coding	PAN3	poly(A) specific ribonuclease subunit PAN3 [Source:HGNC Symbol;Acc:HGNC:29991]	13
ENSG00000112294	4,625503	1,096606	0,005509	0,02526	3,173567	protein_coding	ALDH5A1	aldehyde dehydrogenase 5 family member A1 [Source:HGNC Symbol;Acc:HGNC:408]	6
ENSG00000130032	-2,111587	2,957975	0,005516	0,025285	3,172967	protein_coding	PRRG3	proline rich and Gla domain 3 [Source:HGNC Symbol;Acc:HGNC:30798]	X
ENSG00000103710	1,704907	1,684315	0,005523	0,025308	3,172418	protein_coding	RASL12	RAS like family 12 [Source:HGNC Symbol;Acc:HGNC:30289]	15
ENSG00000161048	3,87391	0,819934	0,005541	0,025387	3,170848	protein_coding	NAPEPLD	N-acyl phosphatidylethanolamine phospholipase D [Source:HGNC Symbol;Acc:HGNC:21683]	7
ENSG00000147130	5,733747	0,616932	0,005544	0,025394	3,17059	protein_coding	ZMYM3	zinc finger MYM-type containing 3 [Source:HGNC Symbol;Acc:HGNC:13054]	X
ENSG00000062822	4,957473	0,837944	0,005548	0,0254	3,170317	protein_coding	POLD1	DNA polymerase delta 1, catalytic subunit [Source:HGNC Symbol;Acc:HGNC:9175]	19
ENSG00000107566	5,22169	-0,535766	0,005549	0,0254	-3,170221	protein_coding	ERLIN1	ER lipid raft associated 1 [Source:HGNC Symbol;Acc:HGNC:16947]	10
ENSG00000182853	-0,450573	2,088448	0,005552	0,025407	3,169953	protein_coding	VMO1	vitelline membrane outer layer 1 homolog [Source:HGNC Symbol;Acc:HGNC:30387]	17
ENSG00000222014	-0,142588	1,308021	0,005565	0,025462	3,168832	protein_coding	RAB6C	RAB6C, member RAS oncogene family [Source:HGNC Symbol;Acc:HGNC:16525]	2
ENSG00000177990	1,203262	2,517711	0,005579	0,025516	3,167709	protein_coding	DPY19L2	dpy-19 like 2 [Source:HGNC Symbol;Acc:HGNC:19414]	12
ENSG00000101204	-2,215684	2,728697	0,005581	0,025519	3,167527	protein_coding	CHRNA4	cholinergic receptor nicotinic alpha 4 subunit [Source:HGNC Symbol;Acc:HGNC:1958]	20
ENSG00000138760	8,391716	-0,925915	0,005587	0,025537	-3,16707	protein_coding	SCARB2	scavenger receptor class B member 2 [Source:HGNC Symbol;Acc:HGNC:1665]	4
ENSG00000138668	7,497851	0,401095	0,005591	0,025548	3,166737	protein_coding	HNRNPD	heterogeneous nuclear ribonucleoprotein D [Source:HGNC Symbol;Acc:HGNC:5036]	4
ENSG00000010256	6,735522	-0,505827	0,005604	0,025601	-3,165639	protein_coding	UQCRC1	ubiquinol-cytochrome c reductase core protein 1 [Source:HGNC Symbol;Acc:HGNC:12585]	3
ENSG00000059728	5,576431	-0,821291	0,005609	0,025615	-3,165257	protein_coding	MXD1	MAX dimerization protein 1 [Source:HGNC Symbol;Acc:HGNC:6761]	2
ENSG00000100105	4,803282	0,73041	0,005625	0,025681	3,163939	protein_coding	PATZ1	POZ/BTB and AT hook containing zinc finger 1 [Source:HGNC Symbol;Acc:HGNC:13071]	22
ENSG00000111875	3,972256	-1,111929	0,005644	0,025743	-3,162334	protein_coding	ASF1A	anti-silencing function 1A histone chaperone [Source:HGNC Symbol;Acc:HGNC:20995]	6
ENSG00000185477	5,046988	1,848231	0,005641	0,025743	3,1626	protein_coding	GPRIN3	GPRIN family member 3 [Source:HGNC Symbol;Acc:HGNC:27733]	4
ENSG00000198938	11,3018	-1,118587	0,005648	0,025743	-3,162035	protein_coding	MT-CO3	mitochondrially encoded cytochrome c oxidase III [Source:HGNC Symbol;Acc:HGNC:7422]	MT
ENSG00000035115	4,913089	0,908033	0,005645	0,025743	3,162283	protein_coding	SH3YL1	SH3 and SYLF domain containing 1 [Source:HGNC Symbol;Acc:HGNC:29546]	2
ENSG00000132467	5,124569	-0,649998	0,005647	0,025743	-3,162101	protein_coding	UTP3	UTP3 small subunit processome component [Source:HGNC Symbol;Acc:HGNC:24477]	4
ENSG00000197343	6,250001	0,517383	0,005644	0,025743	3,16233	protein_coding	ZNF655	zinc finger protein 655 [Source:HGNC Symbol;Acc:HGNC:30899]	7
ENSG00000122565	6,909886	-0,881723	0,005662	0,025802	-3,160846	protein_coding	CBX3	chromobox 3 [Source:HGNC Symbol;Acc:HGNC:1553]	7
ENSG00000273274	-0,499842	2,968793	0,005667	0,025817	3,160442	protein_coding	ZBTB8B	zinc finger and BTB domain containing 8B [Source:HGNC Symbol;Acc:HGNC:37057]	1
ENSG00000144355	-0,153277	-2,833991	0,005674	0,025842	-3,15987	protein_coding	DLX1	distal-less homeobox 1 [Source:HGNC Symbol;Acc:HGNC:2914]	2
ENSG00000163545	3,524371	1,340043	0,005691	0,02591	3,158511	protein_coding	NUAK2	NUAK family kinase 2 [Source:HGNC Symbol;Acc:HGNC:29558]	1

ENSG00000170485	5,068327	-1,704938	0,005699	0,025942	-3,157815	protein_coding	NPAS2	neuronal PAS domain protein 2 [Source:HGNC Symbol;Acc:HGNC:7895]	2
ENSG00000179698	1,478728	1,212086	0,005709	0,02598	3,157004	protein_coding	WDR97	WD repeat domain 97 [Source:HGNC Symbol;Acc:HGNC:26959]	8
ENSG00000044459	4,915295	1,076772	0,005722	0,026031	3,15596	protein_coding	CNTLN	centlein [Source:HGNC Symbol;Acc:HGNC:23432]	9
ENSG00000173868	-0,002784	-2,047066	0,005729	0,026058	-3,155358	protein_coding	PHOSPHO1	phosphoethanolamine/phosphocholine phosphatase 1 [Source:HGNC Symbol;Acc:HGNC:16815]	17
ENSG00000115419	7,006091	-0,668246	0,005732	0,02606	-3,15519	protein_coding	GLS	glutaminase [Source:HGNC Symbol;Acc:HGNC:4331]	2
ENSG00000163482	4,553569	0,754687	0,005737	0,026079	3,154719	protein_coding	STK36	serine/threonine kinase 36 [Source:HGNC Symbol;Acc:HGNC:17209]	2
ENSG00000119801	6,121048	-0,566671	0,00574	0,026085	-3,154481	protein_coding	YPEL5	yippee like 5 [Source:HGNC Symbol;Acc:HGNC:18329]	2
ENSG00000255330	-3,420937	-2,160445	0,005743	0,026089	-3,154279	protein_coding	AL096711.2	novel protein	6
ENSG00000099338	2,687959	1,996639	0,005747	0,0261	3,153962	protein_coding	CATSPERG	cation channel sperm associated auxiliary subunit gamma [Source:HGNC Symbol;Acc:HGNC:25243]	19
ENSG00000121879	5,686327	-0,725277	0,005753	0,026121	-3,153461	protein_coding	PIK3CA	phosphatidylinositol-4,5-bisphosphate 3-kinase catalytic subunit alpha [Source:HGNC Symbol;Acc:HGNC:8975]	3
ENSG00000224389	1,298599	3,409062	0,00576	0,026144	3,152912	protein_coding	C4B	complement C4B (Chido blood group) [Source:HGNC Symbol;Acc:HGNC:1324]	6
ENSG00000111321	6,588919	-0,864606	0,005765	0,026155	-3,152463	protein_coding	LTBR	lymphotoxin beta receptor [Source:HGNC Symbol;Acc:HGNC:6718]	12
ENSG00000120137	7,220343	-0,460456	0,005765	0,026155	-3,152467	protein_coding	PANK3	pantothenate kinase 3 [Source:HGNC Symbol;Acc:HGNC:19365]	5
ENSG00000100053	-1,463188	2,195502	0,005778	0,026207	3,151416	protein_coding	CRYBB3	crystallin beta B3 [Source:HGNC Symbol;Acc:HGNC:2400]	22
ENSG00000172927	-1,560364	-2,650035	0,005786	0,026233	-3,150813	protein_coding	MYEOV	myeloma overexpressed [Source:HGNC Symbol;Acc:HGNC:7563]	11
ENSG00000100170	-1,626833	3,000281	0,005796	0,026265	3,149988	protein_coding	SLC5A1	solute carrier family 5 member 1 [Source:HGNC Symbol;Acc:HGNC:11036]	22
ENSG00000181378	0,399167	1,869984	0,005795	0,026265	3,150097	protein_coding	CFAP65	cilia and flagella associated protein 65 [Source:HGNC Symbol;Acc:HGNC:25325]	2
ENSG00000125249	6,209945	-1,000375	0,005802	0,026282	-3,149559	protein_coding	RAP2A	RAP2A, member of RAS oncogene family [Source:HGNC Symbol;Acc:HGNC:9861]	13
ENSG00000053328	-0,553636	2,140516	0,005806	0,026292	3,14917	protein_coding	METTL24	methyltransferase like 24 [Source:HGNC Symbol;Acc:HGNC:21566]	6
ENSG00000100027	2,534661	1,792798	0,005807	0,026292	3,149134	protein_coding	YPEL1	yippee like 1 [Source:HGNC Symbol;Acc:HGNC:12845]	22
ENSG00000257335	0,419269	3,201785	0,005812	0,026307	3,148739	protein_coding	MGAM	maltase-glucoamylase [Source:HGNC Symbol;Acc:HGNC:7043]	7
ENSG00000099910	4,173752	0,774633	0,005815	0,026315	3,148467	protein_coding	KLHL22	kelch like family member 22 [Source:HGNC Symbol;Acc:HGNC:25888]	22
ENSG00000151893	6,087553	-0,360418	0,005827	0,026361	-3,147533	protein_coding	CACUL1	CDK2 associated cullin domain 1 [Source:HGNC Symbol;Acc:HGNC:23727]	10
ENSG00000141456	6,290804	0,642914	0,005833	0,026379	3,147074	protein_coding	PELP1	proline, glutamate and leucine rich protein 1 [Source:HGNC Symbol;Acc:HGNC:30134]	17
ENSG00000171659	0,335871	2,415622	0,005837	0,026389	3,146768	protein_coding	GPR34	G protein-coupled receptor 34 [Source:HGNC Symbol;Acc:HGNC:4490]	X
ENSG00000007520	5,003492	-0,517075	0,005847	0,026431	-3,145905	protein_coding	TSR3	TSR3 ribosome maturation factor [Source:HGNC Symbol;Acc:HGNC:14175]	16
ENSG00000215045	0,433509	1,960528	0,005853	0,026451	3,145422	protein_coding	GRID2IP	Grid2 interacting protein [Source:HGNC Symbol;Acc:HGNC:18464]	7
ENSG00000164663	3,831541	0,980135	0,005862	0,026476	3,144723	protein_coding	USP49	ubiquitin specific peptidase 49 [Source:HGNC Symbol;Acc:HGNC:20078]	6
ENSG00000134153	5,582625	-0,659432	0,005861	0,026476	-3,144836	protein_coding	EMC7	ER membrane protein complex subunit 7 [Source:HGNC Symbol;Acc:HGNC:24301]	15
ENSG00000146776	4,257628	0,509303	0,00587	0,026505	3,144092	protein_coding	ATXN7L1	ataxin 7 like 1 [Source:HGNC Symbol;Acc:HGNC:22210]	7
ENSG00000166317	-0,557715	-1,896866	0,005885	0,026521	-3,142964	protein_coding	SYNPO2L	synaptopodin 2 like [Source:HGNC Symbol;Acc:HGNC:23532]	10
ENSG00000198771	3,531855	2,196058	0,005885	0,026521	3,142902	protein_coding	RCSD1	RCSD domain containing 1 [Source:HGNC Symbol;Acc:HGNC:28310]	1
ENSG00000162341	3,302066	0,79287	0,005876	0,026521	3,143616	protein_coding	TPCN2	two pore segment channel 2 [Source:HGNC Symbol;Acc:HGNC:20820]	11
ENSG00000204267	3,995618	-0,874763	0,005883	0,026521	-3,14309	protein_coding	TAP2	transporter 2, ATP binding cassette subfamily B member [Source:HGNC Symbol;Acc:HGNC:44]	6
ENSG00000125863	4,188882	-0,598254	0,005884	0,026521	-3,143004	protein_coding	MKKS	McKusick-Kaufman syndrome [Source:HGNC Symbol;Acc:HGNC:7108]	20
ENSG00000106603	5,373022	0,721451	0,005878	0,026521	3,143474	protein_coding	COA1	cytochrome c oxidase assembly factor 1 homolog [Source:HGNC Symbol;Acc:HGNC:21868]	7
ENSG00000137200	6,00416	0,429763	0,005882	0,026521	3,14316	protein_coding	CMTR1	cap methyltransferase 1 [Source:HGNC Symbol;Acc:HGNC:21077]	6
ENSG00000069329	6,518957	-0,557066	0,005892	0,026546	-3,142341	protein_coding	VPS35	VPS35 retromer complex component [Source:HGNC Symbol;Acc:HGNC:13487]	16
ENSG00000258643	0,061867	-1,142775	0,005906	0,026556	-3,141275	protein_coding	BCL2L2-PA	BCL2L2-PABPN1 readthrough [Source:HGNC Symbol;Acc:HGNC:42959]	14
ENSG00000128815	3,191613	2,521483	0,005898	0,026556	3,141916	protein_coding	WDFY4	WDFY family member 4 [Source:HGNC Symbol;Acc:HGNC:29323]	10
ENSG00000121897	2,811728	0,795333	0,005899	0,026556	3,141805	protein_coding	LIAS	lipoic acid synthetase [Source:HGNC Symbol;Acc:HGNC:16429]	4
ENSG00000276045	2,96899	-0,899887	0,005906	0,026556	-3,141286	protein_coding	ORAI1	ORAI calcium release-activated calcium modulator 1 [Source:HGNC Symbol;Acc:HGNC:25896]	12
ENSG00000155975	5,880208	-0,608681	0,005902	0,026556	-3,141575	protein_coding	VPS37A	VPS37A subunit of ESCRT-I [Source:HGNC Symbol;Acc:HGNC:24928]	8
ENSG00000064651	6,364932	-0,610369	0,005904	0,026556	-3,141402	protein_coding	SLC12A2	solute carrier family 12 member 2 [Source:HGNC Symbol;Acc:HGNC:10911]	5
ENSG00000182934	7,47105	-0,648329	0,005903	0,026556	-3,141517	protein_coding	SRPRA	SRP receptor subunit alpha [Source:HGNC Symbol;Acc:HGNC:11307]	11
ENSG00000112038	-3,173728	1,842599	0,00592	0,02661	3,140199	protein_coding	OPRM1	opioid receptor mu 1 [Source:HGNC Symbol;Acc:HGNC:8156]	6
ENSG00000204175	1,896749	2,128475	0,005922	0,026615	3,139982	protein_coding	GPRIN2	G protein regulated inducer of neurite outgrowth 2 [Source:HGNC Symbol;Acc:HGNC:23730]	10
ENSG00000163050	0,919811	1,653238	0,005925	0,02662	3,139766	protein_coding	COQ8A	coenzyme Q8A [Source:HGNC Symbol;Acc:HGNC:16812]	1
ENSG00000165934	6,615249	-0,405892	0,005935	0,026658	-3,138978	protein_coding	CPSF2	cleavage and polyadenylation specific factor 2 [Source:HGNC Symbol;Acc:HGNC:2325]	14
ENSG00000132952	4,451146	0,575624	0,005945	0,026695	3,138193	protein_coding	USPL1	ubiquitin specific peptidase like 1 [Source:HGNC Symbol;Acc:HGNC:20294]	13
ENSG00000101331	1,308197	1,807087	0,005958	0,026738	3,137224	protein_coding	CCM2L	CCM2 like scaffold protein [Source:HGNC Symbol;Acc:HGNC:16153]	20

ENSG00000106397	6,407533	-0,790835	0,005958	0,026738	-3,137204	protein_coding	PLOD3	procollagen-lysine,2-oxoglutarate 5-dioxygenase 3 [Source:HGNC Symbol;Acc:HGNC:9083]	7
ENSG00000133773	4,389641	-0,584094	0,005972	0,026792	-3,136131	protein_coding	CCDC59	coiled-coil domain containing 59 [Source:HGNC Symbol;Acc:HGNC:25005]	12
ENSG00000117013	-1,171664	1,698339	0,00598	0,026813	3,135458	protein_coding	KCNQ4	potassium voltage-gated channel subfamily Q member 4 [Source:HGNC Symbol;Acc:HGNC:6298]	1
ENSG00000124593	0,757554	0,956563	0,005982	0,026813	3,135316	protein_coding	AL365205.:	novel protein	6
ENSG00000142765	3,358664	1,800051	0,005984	0,026813	3,13518	protein_coding	SYTL1	synaptotagmin like 1 [Source:HGNC Symbol;Acc:HGNC:15584]	1
ENSG00000152061	6,404164	1,026443	0,005985	0,026813	3,135127	protein_coding	RABGAP1L	RAB GTPase activating protein 1 like [Source:HGNC Symbol;Acc:HGNC:24663]	1
ENSG00000073921	7,138771	-0,693966	0,005984	0,026813	-3,135211	protein_coding	PICALM	phosphatidylinositol binding clathrin assembly protein [Source:HGNC Symbol;Acc:HGNC:15514]	11
ENSG00000124256	-1,123757	2,830912	0,005994	0,026823	3,134434	protein_coding	ZBP1	Z-DNA binding protein 1 [Source:HGNC Symbol;Acc:HGNC:16176]	20
ENSG00000119973	0,053399	3,447715	0,00599	0,026823	3,13468	protein_coding	PRLHR	prolactin releasing hormone receptor [Source:HGNC Symbol;Acc:HGNC:4464]	10
ENSG00000125505	5,897932	-0,532872	0,00599	0,026823	-3,134716	protein_coding	MBOAT7	membrane bound O-acyltransferase domain containing 7 [Source:HGNC Symbol;Acc:HGNC:15505]	19
ENSG00000038382	8,545734	-0,496765	0,005992	0,026823	-3,134545	protein_coding	TRIO	trio Rho guanine nucleotide exchange factor [Source:HGNC Symbol;Acc:HGNC:12303]	5
ENSG00000204516	2,875397	-1,263773	0,005999	0,026839	-3,134032	protein_coding	MICB	MHC class I polypeptide-related sequence B [Source:HGNC Symbol;Acc:HGNC:7091]	6
ENSG00000147041	3,407744	-2,561055	0,006002	0,026845	-3,133807	protein_coding	SYTL5	synaptotagmin like 5 [Source:HGNC Symbol;Acc:HGNC:15589]	X
ENSG00000197136	6,559193	-0,396317	0,006004	0,026848	-3,133621	protein_coding	PCNX3	pecanex 3 [Source:HGNC Symbol;Acc:HGNC:18760]	11
ENSG00000172939	6,088496	-0,453479	0,006008	0,026856	-3,133293	protein_coding	OXSR1	oxidative stress responsive kinase 1 [Source:HGNC Symbol;Acc:HGNC:8508]	3
ENSG00000173166	6,373719	-0,683683	0,006009	0,026856	-3,133233	protein_coding	RAPH1	Ras association (RalGDS/AF-6) and pleckstrin homology domains 1 [Source:HGNC Symbol;Acc:HGNC:14436]	2
ENSG00000161180	-1,46858	1,173127	0,006011	0,026858	3,133065	protein_coding	CCDC116	coiled-coil domain containing 116 [Source:HGNC Symbol;Acc:HGNC:26688]	22
ENSG00000143858	0,189134	2,889173	0,00603	0,026922	3,13164	protein_coding	SYT2	synaptotagmin 2 [Source:HGNC Symbol;Acc:HGNC:11510]	1
ENSG00000159958	0,397227	3,575109	0,006032	0,026922	3,131456	protein_coding	TNFRSF13C	TNF receptor superfamily member 13C [Source:HGNC Symbol;Acc:HGNC:17755]	22
ENSG00000134871	10,35167	-1,315862	0,00603	0,026922	-3,131604	protein_coding	COL4A2	collagen type IV alpha 2 chain [Source:HGNC Symbol;Acc:HGNC:2203]	13
ENSG00000135390	5,76627	0,390117	0,006031	0,026922	3,131567	protein_coding	ATP5MC2	ATP synthase membrane subunit c locus 2 [Source:HGNC Symbol;Acc:HGNC:842]	12
ENSG00000188290	3,491494	1,05394	0,006047	0,026981	3,130317	protein_coding	HES4	hes family bHLH transcription factor 4 [Source:HGNC Symbol;Acc:HGNC:24149]	1
ENSG00000129657	7,267407	0,549202	0,006056	0,027015	3,129598	protein_coding	SEC14L1	SEC14 like lipid binding 1 [Source:HGNC Symbol;Acc:HGNC:10698]	17
ENSG00000102981	2,527766	1,292802	0,006062	0,027028	3,129128	protein_coding	PARD6A	par-6 family cell polarity regulator alpha [Source:HGNC Symbol;Acc:HGNC:15943]	16
ENSG00000000419	4,899101	-0,551844	0,006063	0,027028	-3,129124	protein_coding	DPM1	dolichyl-phosphate mannosyltransferase subunit 1, catalytic [Source:HGNC Symbol;Acc:HGNC:3005]	20
ENSG00000165171	1,010773	1,590196	0,006069	0,027048	3,128653	protein_coding	METTL27	methyltransferase like 27 [Source:HGNC Symbol;Acc:HGNC:19068]	7
ENSG00000197616	-3,120808	2,59313	0,006077	0,027076	3,128039	protein_coding	MYH6	myosin heavy chain 6 [Source:HGNC Symbol;Acc:HGNC:7576]	14
ENSG00000136891	4,490219	-0,491837	0,006087	0,027115	-3,127245	protein_coding	TEX10	testis expressed 10 [Source:HGNC Symbol;Acc:HGNC:25988]	9
ENSG00000162739	-1,521309	2,768734	0,006093	0,027135	3,126768	protein_coding	SLAMF6	SLAM family member 6 [Source:HGNC Symbol;Acc:HGNC:21392]	1
ENSG00000204961	0,514359	2,390252	0,006097	0,027139	3,126454	protein_coding	PCDHA9	protocadherin alpha 9 [Source:HGNC Symbol;Acc:HGNC:8675]	5
ENSG00000144306	4,023393	-0,562448	0,006096	0,027139	-3,126545	protein_coding	SCRN3	secernin 3 [Source:HGNC Symbol;Acc:HGNC:30382]	2
ENSG00000102172	6,044329	-0,768082	0,006102	0,02715	-3,126133	protein_coding	SMS	spermine synthase [Source:HGNC Symbol;Acc:HGNC:11123]	X
ENSG00000186566	6,890581	0,357216	0,006105	0,027156	3,125902	protein_coding	GPATCH8	G-patch domain containing 8 [Source:HGNC Symbol;Acc:HGNC:29066]	17
ENSG00000161091	6,09477	-0,830439	0,006107	0,027158	-3,125745	protein_coding	MFSD12	major facilitator superfamily domain containing 12 [Source:HGNC Symbol;Acc:HGNC:28299]	19
ENSG00000181585	-1,183713	3,146935	0,006119	0,027204	3,124828	protein_coding	TMIE	transmembrane inner ear [Source:HGNC Symbol;Acc:HGNC:30800]	3
ENSG00000144580	5,618832	-0,443752	0,006122	0,027205	-3,124564	protein_coding	CNOT9	CCR4-NOT transcription complex subunit 9 [Source:HGNC Symbol;Acc:HGNC:10445]	2
ENSG00000141380	6,881424	-0,694491	0,006122	0,027205	-3,124594	protein_coding	SS18	SS18 subunit of BAF chromatin remodeling complex [Source:HGNC Symbol;Acc:HGNC:11340]	18
ENSG00000132330	2,334586	1,648508	0,006127	0,027218	3,124213	protein_coding	SCLY	selenocysteine lyase [Source:HGNC Symbol;Acc:HGNC:18161]	2
ENSG00000063127	1,024713	2,243823	0,006132	0,027233	3,123824	protein_coding	SLC6A16	solute carrier family 6 member 16 [Source:HGNC Symbol;Acc:HGNC:13622]	19
ENSG00000134376	-3,141942	2,649236	0,006141	0,027255	3,123131	protein_coding	CRB1	crumbs cell polarity complex component 1 [Source:HGNC Symbol;Acc:HGNC:2343]	1
ENSG00000136297	-3,245714	2,822721	0,006142	0,027255	3,123077	protein_coding	MMD2	monocyte to macrophage differentiation associated 2 [Source:HGNC Symbol;Acc:HGNC:30133]	7
ENSG00000196821	6,851586	-0,551701	0,006139	0,027255	-3,123274	protein_coding	ILRUN	inflammation and lipid regulator with UBA-like and NBR1-like domains [Source:HGNC Symbol;Acc:HGNC:21215]	6
ENSG00000134516	3,882732	1,897641	0,006151	0,027282	3,122389	protein_coding	DOCK2	dedicator of cytokinesis 2 [Source:HGNC Symbol;Acc:HGNC:2988]	5
ENSG00000164692	11,31191	-1,626485	0,006151	0,027282	-3,122361	protein_coding	COL1A2	collagen type I alpha 2 chain [Source:HGNC Symbol;Acc:HGNC:2198]	7
ENSG00000170956	-2,563281	2,329499	0,006156	0,027289	3,122046	protein_coding	CEACAM3	CEA cell adhesion molecule 3 [Source:HGNC Symbol;Acc:HGNC:1815]	19
ENSG00000167613	2,936902	1,95654	0,006157	0,027289	3,121907	protein_coding	LAIR1	leukocyte associated immunoglobulin like receptor 1 [Source:HGNC Symbol;Acc:HGNC:6477]	19
ENSG00000053372	4,726524	-0,598794	0,006158	0,027289	-3,121856	protein_coding	MRTO4	MRT4 homolog, ribosome maturation factor [Source:HGNC Symbol;Acc:HGNC:18477]	1
ENSG00000141506	2,740631	1,882084	0,006168	0,027326	3,121095	protein_coding	PIK3R5	phosphoinositide-3-kinase regulatory subunit 5 [Source:HGNC Symbol;Acc:HGNC:30035]	17
ENSG00000166377	5,662879	0,667241	0,006174	0,027347	3,120618	protein_coding	ATP9B	ATPase phospholipid transporting 9B (putative) [Source:HGNC Symbol;Acc:HGNC:13541]	18
ENSG00000197054	0,010911	1,228042	0,006184	0,027376	3,119885	protein_coding	ZNF763	zinc finger protein 763 [Source:HGNC Symbol;Acc:HGNC:27614]	19
ENSG00000105875	4,148375	0,965375	0,006185	0,027376	3,119865	protein_coding	WDR91	WD repeat domain 91 [Source:HGNC Symbol;Acc:HGNC:24997]	7

ENSG00000205356	5,332746	0,735271	0,006189	0,027391	3,119493	protein_coding	TECPR1	tectonin beta-propeller repeat containing 1 [Source:HGNC Symbol;Acc:HGNC:22214]	7
ENSG00000165948	2,968564	0,956914	0,006195	0,027407	3,119094	protein_coding	IFI27L1	interferon alpha inducible protein 27 like 1 [Source:HGNC Symbol;Acc:HGNC:19754]	14
ENSG00000131697	4,790276	1,035118	0,006198	0,027411	3,118889	protein_coding	NPHP4	nephrocystin 4 [Source:HGNC Symbol;Acc:HGNC:19104]	1
ENSG00000205560	-0,112903	1,703949	0,00621	0,027461	3,117918	protein_coding	CPT1B	carnitine palmitoyltransferase 1B [Source:HGNC Symbol;Acc:HGNC:2329]	22
ENSG00000172336	4,128281	-0,562339	0,006214	0,027468	-3,11768	protein_coding	POP7	POP7 homolog, ribonuclease P/MRP subunit [Source:HGNC Symbol;Acc:HGNC:19949]	7
ENSG00000227507	-0,418483	2,997743	0,006215	0,027468	3,117543	protein_coding	LTB	lymphotoxin beta [Source:HGNC Symbol;Acc:HGNC:6711]	6
ENSG00000095203	4,874614	1,457311	0,006218	0,027472	3,117365	protein_coding	EPB41L4B	erythrocyte membrane protein band 4.1 like 4B [Source:HGNC Symbol;Acc:HGNC:19818]	9
ENSG00000139746	6,565677	0,648165	0,006228	0,02751	3,116591	protein_coding	RBM26	RNA binding motif protein 26 [Source:HGNC Symbol;Acc:HGNC:20327]	13
ENSG00000226763	1,537653	1,038125	0,006233	0,027518	3,116209	protein_coding	SRRM5	serine/arginine repetitive matrix 5 [Source:HGNC Symbol;Acc:HGNC:37248]	19
ENSG00000278535	2,924725	1,045177	0,006233	0,027518	3,116201	protein_coding	DHRS11	dehydrogenase/reductase 11 [Source:HGNC Symbol;Acc:HGNC:28639]	17
ENSG00000157388	5,267751	1,835744	0,006237	0,027527	3,115919	protein_coding	CACNA1D	calcium voltage-gated channel subunit alpha1 D [Source:HGNC Symbol;Acc:HGNC:1391]	3
ENSG00000144847	-0,399108	2,844768	0,006247	0,027556	3,115173	protein_coding	IGSF11	immunoglobulin superfamily member 11 [Source:HGNC Symbol;Acc:HGNC:16669]	3
ENSG00000081154	6,123629	-0,451276	0,006247	0,027556	-3,115183	protein_coding	PCNP	PEST proteolytic signal containing nuclear protein [Source:HGNC Symbol;Acc:HGNC:30023]	3
ENSG00000108788	5,739793	-0,54599	0,006252	0,02757	-3,114812	protein_coding	MLX	MAX dimerization protein MLX [Source:HGNC Symbol;Acc:HGNC:11645]	17
ENSG00000111886	0,844317	1,794188	0,006259	0,027594	3,114286	protein_coding	GABRR2	gamma-aminobutyric acid type A receptor subunit rho2 [Source:HGNC Symbol;Acc:HGNC:4091]	6
ENSG00000123595	3,878033	-0,810867	0,006275	0,027658	-3,11309	protein_coding	RAB9A	RAB9A, member RAS oncogene family [Source:HGNC Symbol;Acc:HGNC:9792]	X
ENSG00000126088	5,015963	-0,837094	0,006281	0,027673	-3,112699	protein_coding	UROD	uroporphyrinogen decarboxylase [Source:HGNC Symbol;Acc:HGNC:12591]	1
ENSG00000113070	4,339805	-1,344052	0,006283	0,027677	-3,112507	protein_coding	HBEGF	heparin binding EGF like growth factor [Source:HGNC Symbol;Acc:HGNC:3059]	5
ENSG00000177303	5,126682	0,596018	0,006285	0,027677	3,112385	protein_coding	CASKIN2	CASK interacting protein 2 [Source:HGNC Symbol;Acc:HGNC:18200]	17
ENSG00000135362	3,145191	-1,064848	0,006299	0,027724	-3,111343	protein_coding	PRR5L	proline rich 5 like [Source:HGNC Symbol;Acc:HGNC:25878]	11
ENSG00000130396	7,679453	0,928505	0,006299	0,027724	3,111374	protein_coding	AFDN	afadin, adherens junction formation factor [Source:HGNC Symbol;Acc:HGNC:7137]	6
ENSG00000125755	6,813861	0,492433	0,006306	0,027749	3,110796	protein_coding	SYMPK	symplekin [Source:HGNC Symbol;Acc:HGNC:22935]	19
ENSG00000143502	2,395256	2,731364	0,006318	0,027776	3,109965	protein_coding	SUSD4	sushi domain containing 4 [Source:HGNC Symbol;Acc:HGNC:25470]	1
ENSG00000114698	4,380139	-1,538041	0,006316	0,027776	-3,110082	protein_coding	PLSCR4	phospholipid scramblase 4 [Source:HGNC Symbol;Acc:HGNC:16497]	3
ENSG00000132740	4,742539	0,52659	0,006315	0,027776	3,110153	protein_coding	IGHMBP2	immunoglobulin mu DNA binding protein 2 [Source:HGNC Symbol;Acc:HGNC:5542]	11
ENSG00000050767	0,654011	1,889051	0,006322	0,027782	3,109618	protein_coding	COL23A1	collagen type XXIII alpha 1 chain [Source:HGNC Symbol;Acc:HGNC:22990]	5
ENSG00000178951	6,858632	-0,561384	0,006322	0,027782	-3,109646	protein_coding	ZBTB7A	zinc finger and BTB domain containing 7A [Source:HGNC Symbol;Acc:HGNC:18078]	19
ENSG00000174405	4,831133	-0,664486	0,006327	0,027797	-3,109247	protein_coding	LIG4	DNA ligase 4 [Source:HGNC Symbol;Acc:HGNC:6601]	13
ENSG00000154743	3,81577	0,905105	0,006334	0,027819	3,108744	protein_coding	TSEN2	tRNA splicing endonuclease subunit 2 [Source:HGNC Symbol;Acc:HGNC:28422]	3
ENSG00000103489	4,991625	-1,163975	0,006337	0,027823	-3,108555	protein_coding	XYLT1	xylosyltransferase 1 [Source:HGNC Symbol;Acc:HGNC:15516]	16
ENSG00000283563	-1,224562	1,708904	0,006343	0,027837	3,108099	protein_coding	AC098650.	novel protein	3
ENSG00000187372	3,378291	1,345082	0,006343	0,027837	3,108075	protein_coding	PCDHB13	protocadherin beta 13 [Source:HGNC Symbol;Acc:HGNC:8684]	5
ENSG00000160226	3,717224	0,723422	0,006346	0,02784	3,107901	protein_coding	CFAP410	cilia and flagella associated protein 410 [Source:HGNC Symbol;Acc:HGNC:1260]	21
ENSG00000171566	5,220009	-0,584832	0,006353	0,027857	-3,10736	protein_coding	PLRG1	pleiotropic regulator 1 [Source:HGNC Symbol;Acc:HGNC:9089]	4
ENSG00000149503	6,246166	0,69698	0,006353	0,027857	3,1074	protein_coding	INCENP	inner centromere protein [Source:HGNC Symbol;Acc:HGNC:6058]	11
ENSG00000164099	2,12661	2,727007	0,00636	0,027881	3,106839	protein_coding	PRSS12	serine protease 12 [Source:HGNC Symbol;Acc:HGNC:9477]	4
ENSG00000154277	5,855458	-2,720318	0,006363	0,027884	-3,106659	protein_coding	UCHL1	ubiquitin C-terminal hydrolase L1 [Source:HGNC Symbol;Acc:HGNC:12513]	4
ENSG00000145365	3,634559	0,916821	0,006373	0,027921	3,105915	protein_coding	TIFA	TRAF interacting protein with forkhead associated domain [Source:HGNC Symbol;Acc:HGNC:19075]	4
ENSG00000075420	7,992355	-0,953707	0,006385	0,027966	-3,105035	protein_coding	FNDC3B	fibronectin type III domain containing 3B [Source:HGNC Symbol;Acc:HGNC:24670]	3
ENSG00000174776	0,366056	2,662949	0,006405	0,028039	3,103584	protein_coding	WDR49	WD repeat domain 49 [Source:HGNC Symbol;Acc:HGNC:26587]	3
ENSG00000143515	6,659451	-0,600197	0,006405	0,028039	-3,103605	protein_coding	ATP8B2	ATPase phospholipid transporting 8B2 [Source:HGNC Symbol;Acc:HGNC:13534]	1
ENSG00000174844	-0,38294	2,764606	0,006407	0,02804	3,103444	protein_coding	DNAH12	dynein axonemal heavy chain 12 [Source:HGNC Symbol;Acc:HGNC:2943]	3
ENSG00000189042	3,8914	0,615856	0,006412	0,028055	3,103062	protein_coding	ZNF567	zinc finger protein 567 [Source:HGNC Symbol;Acc:HGNC:28696]	19
ENSG00000130772	2,651439	-0,681617	0,00642	0,028084	-3,102464	protein_coding	MED18	mediator complex subunit 18 [Source:HGNC Symbol;Acc:HGNC:25944]	1
ENSG00000184216	7,270784	-0,63034	0,006422	0,028085	-3,10232	protein_coding	IRAK1	interleukin 1 receptor associated kinase 1 [Source:HGNC Symbol;Acc:HGNC:6112]	X
ENSG00000171056	-0,317299	1,808873	0,006429	0,028101	3,101802	protein_coding	SOX7	SRY-box transcription factor 7 [Source:HGNC Symbol;Acc:HGNC:18196]	8
ENSG00000104756	5,198281	-0,825169	0,006428	0,028101	-3,101918	protein_coding	KCTD9	potassium channel tetramerization domain containing 9 [Source:HGNC Symbol;Acc:HGNC:22401]	8
ENSG00000139597	3,091048	1,478708	0,006436	0,028124	3,101301	protein_coding	N4BP2L1	NEDD4 binding protein 2 like 1 [Source:HGNC Symbol;Acc:HGNC:25037]	13
ENSG00000166927	2,814118	2,453752	0,006445	0,028154	3,100665	protein_coding	MS4A7	membrane spanning 4-domains A7 [Source:HGNC Symbol;Acc:HGNC:13378]	11
ENSG00000172322	-1,473087	2,747792	0,00645	0,028155	3,100352	protein_coding	CLEC12A	C-type lectin domain family 12 member A [Source:HGNC Symbol;Acc:HGNC:31713]	12
ENSG00000165125	-1,31818	2,796062	0,006451	0,028155	3,100272	protein_coding	TRPV6	transient receptor potential cation channel subfamily V member 6 [Source:HGNC Symbol;Acc:HGNC:14006]	7

ENSG00000124786	4,454499	-0,655034	0,006452	0,028155	-3,10016	protein_coding	SLC35B3	solute carrier family 35 member B3 [Source:HGNC Symbol;Acc:HGNC:21601]	6
ENSG00000067182	5,837651	-1,269027	0,00645	0,028155	-3,100325	protein_coding	TNFRSF1A	TNF receptor superfamily member 1A [Source:HGNC Symbol;Acc:HGNC:11916]	12
ENSG00000152705	-1,167897	1,674546	0,00647	0,028224	3,098893	protein_coding	CATSPER3	cation channel sperm associated 3 [Source:HGNC Symbol;Acc:HGNC:20819]	5
ENSG00000275674	-1,746496	1,896914	0,006479	0,028257	3,098219	protein_coding	AC091167.	novel protein	15
ENSG00000235568	1,61406	2,00313	0,006484	0,028269	3,097909	protein_coding	NFAM1	NFAT activating protein with ITAM motif 1 [Source:HGNC Symbol;Acc:HGNC:29872]	22
ENSG00000129219	4,080026	0,836186	0,006499	0,028328	3,096806	protein_coding	PLD2	phospholipase D2 [Source:HGNC Symbol;Acc:HGNC:9068]	17
ENSG00000107738	3,44202	1,488329	0,006504	0,028343	3,096435	protein_coding	VSIR	V-set immunoregulatory receptor [Source:HGNC Symbol;Acc:HGNC:30085]	10
ENSG00000197283	5,545639	0,831034	0,006511	0,028366	3,095932	protein_coding	SYNGAP1	synaptic Ras GTPase activating protein 1 [Source:HGNC Symbol;Acc:HGNC:11497]	6
ENSG00000166501	2,278975	2,858114	0,00652	0,028396	3,095318	protein_coding	PRKCB	protein kinase C beta [Source:HGNC Symbol;Acc:HGNC:9395]	16
ENSG00000147437	0,41534	1,419144	0,006526	0,028416	3,094865	protein_coding	GNRH1	gonadotropin releasing hormone 1 [Source:HGNC Symbol;Acc:HGNC:4419]	8
ENSG00000089472	4,57691	-2,021386	0,00653	0,028425	-3,094588	protein_coding	HEPH	hephaestin [Source:HGNC Symbol;Acc:HGNC:4866]	X
ENSG00000243789	-0,393313	1,428224	0,006552	0,028513	3,093036	protein_coding	JMJD7	jumonji domain containing 7 [Source:HGNC Symbol;Acc:HGNC:34397]	15
ENSG00000184305	2,703458	2,26706	0,006556	0,028516	3,092732	protein_coding	CCSER1	coiled-coil serine rich protein 1 [Source:HGNC Symbol;Acc:HGNC:29349]	4
ENSG00000109339	4,672623	1,749251	0,006555	0,028516	3,092817	protein_coding	MAPK10	mitogen-activated protein kinase 10 [Source:HGNC Symbol;Acc:HGNC:6872]	4
ENSG00000174996	5,862858	-0,589641	0,006558	0,028517	-3,092598	protein_coding	KLC2	kinesin light chain 2 [Source:HGNC Symbol;Acc:HGNC:20716]	11
ENSG00000168806	3,55843	-0,69679	0,006584	0,028621	-3,090766	protein_coding	LCMT2	leucine carboxyl methyltransferase 2 [Source:HGNC Symbol;Acc:HGNC:17558]	15
ENSG00000169045	8,467588	0,66284	0,006588	0,028633	3,090455	protein_coding	HNRNPH1	heterogeneous nuclear ribonucleoprotein H1 [Source:HGNC Symbol;Acc:HGNC:5041]	5
ENSG00000113083	5,593409	-1,577502	0,006596	0,02866	-3,089881	protein_coding	LOX	lysyl oxidase [Source:HGNC Symbol;Acc:HGNC:6664]	5
ENSG00000172936	5,015286	-0,615749	0,006611	0,028717	-3,088833	protein_coding	MYD88	MYD88 innate immune signal transduction adaptor [Source:HGNC Symbol;Acc:HGNC:7562]	3
ENSG00000112159	7,466923	0,745283	0,006618	0,028741	3,088328	protein_coding	MDN1	midasin AAA ATPase 1 [Source:HGNC Symbol;Acc:HGNC:18302]	6
ENSG00000180917	4,41785	-0,482274	0,006623	0,028753	-3,088012	protein_coding	CMTR2	cap methyltransferase 2 [Source:HGNC Symbol;Acc:HGNC:25635]	16
ENSG00000178878	3,544326	2,268804	0,006628	0,028766	3,087669	protein_coding	APOLD1	apolipoprotein L domain containing 1 [Source:HGNC Symbol;Acc:HGNC:25268]	12
ENSG00000174123	-2,778132	2,832193	0,00664	0,028803	3,086831	protein_coding	TLR10	toll like receptor 10 [Source:HGNC Symbol;Acc:HGNC:15634]	4
ENSG00000119650	3,701978	0,659469	0,006639	0,028803	3,086903	protein_coding	IFT43	intraflagellar transport 43 [Source:HGNC Symbol;Acc:HGNC:29669]	14
ENSG00000138792	4,09539	1,856091	0,006643	0,02881	3,086583	protein_coding	ENPEP	glutamyl aminopeptidase [Source:HGNC Symbol;Acc:HGNC:3355]	4
ENSG00000283761	-0,586757	1,023368	0,006645	0,028811	3,086455	protein_coding	AC118553.	novel protein	1
ENSG00000149346	4,523654	0,621906	0,006648	0,028814	3,086276	protein_coding	SLX4IP	SLX4 interacting protein [Source:HGNC Symbol;Acc:HGNC:16225]	20
ENSG00000044446	5,385998	0,980558	0,006655	0,028838	3,085756	protein_coding	PHKA2	phosphorylase kinase regulatory subunit alpha 2 [Source:HGNC Symbol;Acc:HGNC:8926]	X
ENSG00000107771	6,534121	-0,675386	0,006668	0,028889	-3,084817	protein_coding	CCSER2	coiled-coil serine rich protein 2 [Source:HGNC Symbol;Acc:HGNC:29197]	10
ENSG00000121440	6,346777	1,443329	0,006701	0,029013	3,082578	protein_coding	PDZRN3	PDZ domain containing ring finger 3 [Source:HGNC Symbol;Acc:HGNC:17704]	3
ENSG00000111961	6,016139	-0,971666	0,0067	0,029013	-3,082652	protein_coding	SASH1	SAM and SH3 domain containing 1 [Source:HGNC Symbol;Acc:HGNC:19182]	6
ENSG00000041515	-0,938236	2,508584	0,006709	0,029028	3,081999	protein_coding	MYO16	myosin XVI [Source:HGNC Symbol;Acc:HGNC:29822]	13
ENSG00000165275	3,06575	1,003198	0,006709	0,029028	3,081967	protein_coding	TRMT10B	tRNA methyltransferase 10B [Source:HGNC Symbol;Acc:HGNC:26454]	9
ENSG00000107954	4,565818	1,98588	0,006709	0,029028	3,081992	protein_coding	NEURL1	neuralized E3 ubiquitin protein ligase 1 [Source:HGNC Symbol;Acc:HGNC:7761]	10
ENSG00000105829	3,553202	-0,669417	0,006718	0,029056	-3,081393	protein_coding	BET1	Bet1 golgi vesicular membrane trafficking protein [Source:HGNC Symbol;Acc:HGNC:14562]	7
ENSG00000118407	2,591147	1,588979	0,006722	0,029065	3,081128	protein_coding	FILIP1	filamin A interacting protein 1 [Source:HGNC Symbol;Acc:HGNC:21015]	6
ENSG00000080224	-0,089064	2,731729	0,006732	0,029084	3,080369	protein_coding	EPHA6	EPH receptor A6 [Source:HGNC Symbol;Acc:HGNC:19296]	3
ENSG00000278619	2,516374	0,872108	0,00673	0,029084	3,080513	protein_coding	MRM1	mitochondrial rRNA methyltransferase 1 [Source:HGNC Symbol;Acc:HGNC:26202]	17
ENSG00000180353	4,306759	1,646864	0,006733	0,029084	3,080317	protein_coding	HCLS1	hematopoietic cell-specific Lyn substrate 1 [Source:HGNC Symbol;Acc:HGNC:4844]	3
ENSG00000072803	5,929637	-0,417031	0,006729	0,029084	-3,080583	protein_coding	FBXW11	F-box and WD repeat domain containing 11 [Source:HGNC Symbol;Acc:HGNC:13607]	5
ENSG00000012174	4,699262	-0,643849	0,006735	0,029085	-3,080183	protein_coding	MBTPS2	membrane bound transcription factor peptidase, site 2 [Source:HGNC Symbol;Acc:HGNC:15455]	X
ENSG00000240505	-3,075967	2,97975	0,006755	0,0291	3,078833	protein_coding	TNFRSF13E	TNF receptor superfamily member 13B [Source:HGNC Symbol;Acc:HGNC:18153]	17
ENSG00000163221	-3,387709	2,798771	0,006751	0,0291	3,079067	protein_coding	S100A12	S100 calcium binding protein A12 [Source:HGNC Symbol;Acc:HGNC:10489]	1
ENSG00000213213	2,177286	1,671676	0,006747	0,0291	3,079392	protein_coding	CCDC183	coiled-coil domain containing 183 [Source:HGNC Symbol;Acc:HGNC:28236]	9
ENSG00000196639	2,602053	-1,407808	0,006746	0,0291	-3,079422	protein_coding	HRH1	histamine receptor H1 [Source:HGNC Symbol;Acc:HGNC:5182]	3
ENSG00000099998	3,762093	1,021758	0,006754	0,0291	3,078912	protein_coding	GGT5	gamma-glutamyltransferase 5 [Source:HGNC Symbol;Acc:HGNC:4260]	22
ENSG00000204681	4,630273	0,989534	0,006746	0,0291	3,079449	protein_coding	GABBR1	gamma-aminobutyric acid type B receptor subunit 1 [Source:HGNC Symbol;Acc:HGNC:4070]	6
ENSG00000196705	4,858755	0,680754	0,006751	0,0291	3,079083	protein_coding	ZNF431	zinc finger protein 431 [Source:HGNC Symbol;Acc:HGNC:20809]	19
ENSG00000189180	5,696231	0,779187	0,006749	0,0291	3,079197	protein_coding	ZNF33A	zinc finger protein 33A [Source:HGNC Symbol;Acc:HGNC:13096]	10
ENSG00000140367	5,623904	-0,455924	0,006741	0,0291	-3,079761	protein_coding	UBE2Q2	ubiquitin conjugating enzyme E2 Q2 [Source:HGNC Symbol;Acc:HGNC:19248]	15
ENSG00000185513	3,057108	1,861826	0,006758	0,029108	3,078579	protein_coding	L3MBTL1	L3MBTL histone methyl-lysine binding protein 1 [Source:HGNC Symbol;Acc:HGNC:15905]	20

ENSG00000158528	5,190825	1,471928	0,006768	0,029141	3,077919	protein_coding	PPP1R9A	protein phosphatase 1 regulatory subunit 9A [Source:HGNC Symbol;Acc:HGNC:14946]		7
ENSG00000082068	5,331833	0,488637	0,006779	0,02918	3,077178	protein_coding	WDR70	WD repeat domain 70 [Source:HGNC Symbol;Acc:HGNC:25495]		5
ENSG00000151348	6,555189	-0,522568	0,006784	0,029195	-3,076816	protein_coding	EXT2	exostosin glycosyltransferase 2 [Source:HGNC Symbol;Acc:HGNC:3513]		11
ENSG00000139914	-1,34823	1,808514	0,006795	0,029225	3,076042	protein_coding	FITM1	fat storage inducing transmembrane protein 1 [Source:HGNC Symbol;Acc:HGNC:33714]		14
ENSG00000026297	4,790926	1,433394	0,006798	0,029225	3,075849	protein_coding	RNASET2	ribonuclease T2 [Source:HGNC Symbol;Acc:HGNC:21686]		6
ENSG00000052723	5,341223	-0,533644	0,006798	0,029225	-3,075891	protein_coding	SIKE1	suppressor of IKBKE 1 [Source:HGNC Symbol;Acc:HGNC:26119]		1
ENSG00000164176	6,069074	-1,58916	0,006797	0,029225	-3,075956	protein_coding	EDIL3	EGF like repeats and discoidin domains 3 [Source:HGNC Symbol;Acc:HGNC:3173]		5
ENSG00000183439	-0,292796	-2,334486	0,006808	0,029252	-3,075176	protein_coding	TRIM61	tripartite motif containing 61 [Source:HGNC Symbol;Acc:HGNC:24339]		4
ENSG00000196277	0,020119	2,839684	0,006807	0,029252	3,07527	protein_coding	GRM7	glutamate metabotropic receptor 7 [Source:HGNC Symbol;Acc:HGNC:4599]		3
ENSG00000185803	5,045599	-0,602707	0,006847	0,029413	-3,07249	protein_coding	SLC52A2	solute carrier family 52 member 2 [Source:HGNC Symbol;Acc:HGNC:30224]		8
ENSG00000154545	0,248685	2,051717	0,006866	0,029477	3,071235	protein_coding	MAGED4	MAGE family member D4 [Source:HGNC Symbol;Acc:HGNC:23793]		X
ENSG00000139645	7,032332	-0,560894	0,006865	0,029477	-3,071279	protein_coding	ANKRD52	ankyrin repeat domain 52 [Source:HGNC Symbol;Acc:HGNC:26614]		12
ENSG00000148331	5,020399	-0,662773	0,006868	0,029477	-3,071104	protein_coding	ASB6	ankyrin repeat and SOCS box containing 6 [Source:HGNC Symbol;Acc:HGNC:17181]		9
ENSG00000134996	4,661567	-0,782233	0,006887	0,029552	-3,0698	protein_coding	OSTF1	osteoclast stimulating factor 1 [Source:HGNC Symbol;Acc:HGNC:8510]		9
ENSG00000134817	0,865891	3,537654	0,006892	0,029564	3,069451	protein_coding	APLNR	apelin receptor [Source:HGNC Symbol;Acc:HGNC:339]		11
ENSG00000196182	6,039994	-0,791836	0,006893	0,029564	-3,069372	protein_coding	STK40	serine/threonine kinase 40 [Source:HGNC Symbol;Acc:HGNC:21373]		1
ENSG00000130598	-1,024906	2,738676	0,006895	0,029564	3,069238	protein_coding	TNNI2	troponin I2, fast skeletal type [Source:HGNC Symbol;Acc:HGNC:11946]		11
ENSG00000075624	11,46173	-0,850079	0,006903	0,029591	-3,068691	protein_coding	ACTB	actin beta [Source:HGNC Symbol;Acc:HGNC:132]		7
ENSG00000172724	-2,971344	3,232705	0,006911	0,029616	3,068172	protein_coding	CCL19	C-C motif chemokine ligand 19 [Source:HGNC Symbol;Acc:HGNC:10617]		9
ENSG00000213741	5,863575	0,731988	0,006925	0,029666	3,067267	protein_coding	RPS29	ribosomal protein S29 [Source:HGNC Symbol;Acc:HGNC:10419]		14
ENSG00000063761	3,407712	0,833779	0,006932	0,029689	3,066779	protein_coding	ADCK1	aarF domain containing kinase 1 [Source:HGNC Symbol;Acc:HGNC:19038]		14
ENSG00000184752	4,881194	-0,680504	0,006937	0,029704	-3,066422	protein_coding	NDUFA12	NADH:ubiquinone oxidoreductase subunit A12 [Source:HGNC Symbol;Acc:HGNC:23987]		12
ENSG00000119203	5,332276	-0,535854	0,006941	0,029712	-3,06618	protein_coding	CPSF3	cleavage and polyadenylation specific factor 3 [Source:HGNC Symbol;Acc:HGNC:2326]		2
ENSG00000100767	2,73609	2,006974	0,006945	0,029725	3,065858	protein_coding	PAPLN	papilin, proteoglycan like sulfated glycoprotein [Source:HGNC Symbol;Acc:HGNC:19262]		14
ENSG00000174500	-2,659218	2,269071	0,006949	0,02973	3,065645	protein_coding	GCSAM	germinal center associated signaling and motility [Source:HGNC Symbol;Acc:HGNC:20253]		3
ENSG00000179796	-3,002181	3,225187	0,006956	0,029744	3,065148	protein_coding	LRRC3B	leucine rich repeat containing 3B [Source:HGNC Symbol;Acc:HGNC:28105]		3
ENSG00000162777	3,925715	1,106607	0,006957	0,029744	3,065058	protein_coding	DENND2D	DENN domain containing 2D [Source:HGNC Symbol;Acc:HGNC:26192]		1
ENSG00000205060	4,597423	-0,702248	0,006954	0,029744	-3,065295	protein_coding	SLC35B4	solute carrier family 35 member B4 [Source:HGNC Symbol;Acc:HGNC:20584]		7
ENSG00000112214	-1,6364	2,786728	0,006962	0,029753	3,064769	protein_coding	FHL5	four and a half LIM domains 5 [Source:HGNC Symbol;Acc:HGNC:17371]		6
ENSG00000108771	2,32433	0,945977	0,006965	0,029753	3,064574	protein_coding	DHX58	DExH-box helicase 58 [Source:HGNC Symbol;Acc:HGNC:29517]		17
ENSG00000197363	2,377171	0,921096	0,006965	0,029753	3,064549	protein_coding	ZNF517	zinc finger protein 517 [Source:HGNC Symbol;Acc:HGNC:27984]		8
ENSG00000179902	-1,946148	2,226294	0,006969	0,029761	3,064304	protein_coding	C1orf194	chromosome 1 open reading frame 194 [Source:HGNC Symbol;Acc:HGNC:32331]		1
ENSG00000157184	4,287487	-0,51099	0,006982	0,029809	-3,063437	protein_coding	CPT2	carnitine palmitoyltransferase 2 [Source:HGNC Symbol;Acc:HGNC:2330]		1
ENSG00000139187	-0,871002	1,484088	0,007009	0,029912	3,061625	protein_coding	KLRG1	killer cell lectin like receptor G1 [Source:HGNC Symbol;Acc:HGNC:6380]		12
ENSG00000127418	4,827479	-1,249997	0,00701	0,029912	-3,061574	protein_coding	FGFRL1	fibroblast growth factor receptor like 1 [Source:HGNC Symbol;Acc:HGNC:3693]		4
ENSG00000135094	1,807851	1,919034	0,007012	0,029916	3,061397	protein_coding	SDS	serine dehydratase [Source:HGNC Symbol;Acc:HGNC:10691]		12
ENSG00000026103	3,575885	-1,360119	0,007024	0,02995	-3,060626	protein_coding	FAS	Fas cell surface death receptor [Source:HGNC Symbol;Acc:HGNC:11920]		10
ENSG00000112578	3,889474	-0,566311	0,007023	0,02995	-3,060712	protein_coding	BYSL	bystin like [Source:HGNC Symbol;Acc:HGNC:1157]		6
ENSG00000163683	5,742194	-0,936612	0,007033	0,029982	-3,060002	protein_coding	SMIM14	small integral membrane protein 14 [Source:HGNC Symbol;Acc:HGNC:27321]		4
ENSG00000130035	-0,621705	3,050079	0,007039	0,029998	3,059636	protein_coding	GALNT8	polypeptide N-acetylgalactosaminyltransferase 8 [Source:HGNC Symbol;Acc:HGNC:4130]		12
ENSG00000130299	4,162553	0,770116	0,007047	0,030026	3,059068	protein_coding	GTPBP3	GTP binding protein 3, mitochondrial [Source:HGNC Symbol;Acc:HGNC:14880]		19
ENSG00000198723	-0,267425	1,956386	0,007063	0,030079	3,058007	protein_coding	TEX45	testis expressed 45 [Source:HGNC Symbol;Acc:HGNC:24745]		19
ENSG00000247315	5,166147	0,546455	0,007062	0,030079	3,05808	protein_coding	ZCCHC3	zinc finger CCHC-type containing 3 [Source:HGNC Symbol;Acc:HGNC:16230]		20
ENSG00000163661	2,675662	-3,085111	0,00707	0,030097	-3,057604	protein_coding	PTX3	pentraxin 3 [Source:HGNC Symbol;Acc:HGNC:9692]		3
ENSG00000088448	6,189848	0,579987	0,007077	0,03012	3,057124	protein_coding	ANKRD10	ankyrin repeat domain 10 [Source:HGNC Symbol;Acc:HGNC:20265]		13
ENSG00000265590	1,034014	1,085272	0,007081	0,03013	3,056827	protein_coding	CFAP298-T	CFAP298-TCP10L readthrough [Source:HGNC Symbol;Acc:HGNC:54636]		21
ENSG00000151689	4,304341	-0,912829	0,007083	0,03013	-3,056723	protein_coding	INPP1	inositol polyphosphate-1-phosphatase [Source:HGNC Symbol;Acc:HGNC:6071]		2
ENSG00000175262	0,440942	2,966283	0,007092	0,030162	3,056114	protein_coding	C1orf127	chromosome 1 open reading frame 127 [Source:HGNC Symbol;Acc:HGNC:26730]		1
ENSG00000140497	5,447999	-0,608258	0,007102	0,030194	-3,0555	protein_coding	SCAMP2	secretory carrier membrane protein 2 [Source:HGNC Symbol;Acc:HGNC:10564]		15
ENSG00000146350	3,325753	0,92289	0,00711	0,03022	3,054972	protein_coding	TBC1D32	TBC1 domain family member 32 [Source:HGNC Symbol;Acc:HGNC:21485]		6
ENSG00000102886	0,74366	1,265549	0,007118	0,030249	3,054399	protein_coding	GDPD3	glycerophosphodiester phosphodiesterase domain containing 3 [Source:HGNC Symbol;Acc:HGNC:28638]		16

ENSG00000170832	6,392477	-0,485031	0,007141	0,030336	-3,052937	protein_coding	USP32	ubiquitin specific peptidase 32 [Source:HGNC Symbol;Acc:HGNC:19143]	17
ENSG00000001461	5,53276	-0,747538	0,007145	0,030346	-3,052672	protein_coding	NIPAL3	NIPA like domain containing 3 [Source:HGNC Symbol;Acc:HGNC:25233]	1
ENSG00000141378	4,187928	-0,442162	0,007152	0,030368	-3,052214	protein_coding	PTRH2	peptidyl-tRNA hydrolase 2 [Source:HGNC Symbol;Acc:HGNC:24265]	17
ENSG00000012171	3,102951	1,466535	0,007189	0,030519	3,049774	protein_coding	SEMA3B	semaphorin 3B [Source:HGNC Symbol;Acc:HGNC:10724]	3
ENSG00000160791	0,360028	2,211175	0,007195	0,03052	3,049378	protein_coding	CCR5	C-C motif chemokine receptor 5 [Source:HGNC Symbol;Acc:HGNC:1606]	3
ENSG00000253767	1,681153	2,053307	0,007194	0,03052	3,049488	protein_coding	PCDHGA8	protocadherin gamma subfamily A, 8 [Source:HGNC Symbol;Acc:HGNC:8706]	5
ENSG00000108821	12,26022	-1,6825	0,007197	0,03052	-3,049269	protein_coding	COL1A1	collagen type I alpha 1 chain [Source:HGNC Symbol;Acc:HGNC:2197]	17
ENSG00000218739	4,694342	-0,495735	0,007194	0,03052	-3,049487	protein_coding	CEBPZOS	CEBPZ opposite strand [Source:HGNC Symbol;Acc:HGNC:49288]	2
ENSG00000163607	3,106328	-0,688814	0,007199	0,030521	-3,04914	protein_coding	GTPBP8	GTP binding protein 8 (putative) [Source:HGNC Symbol;Acc:HGNC:25007]	3
ENSG00000078747	6,899772	-0,376438	0,007205	0,030537	-3,048769	protein_coding	ITCH	itchy E3 ubiquitin protein ligase [Source:HGNC Symbol;Acc:HGNC:13890]	20
ENSG00000256029	-1,287142	1,760078	0,007212	0,030552	3,048296	protein_coding	AL590560.:	novel protein	1
ENSG00000137218	2,274264	0,746751	0,007212	0,030552	3,048335	protein_coding	FRS3	fibroblast growth factor receptor substrate 3 [Source:HGNC Symbol;Acc:HGNC:16970]	6
ENSG00000121644	5,497858	-0,695287	0,007229	0,030616	-3,047203	protein_coding	DESI2	desumoylating isopeptidase 2 [Source:HGNC Symbol;Acc:HGNC:24264]	1
ENSG00000091513	0,111564	3,785544	0,007236	0,030633	3,046746	protein_coding	TF	transferrin [Source:HGNC Symbol;Acc:HGNC:11740]	3
ENSG00000135446	6,012581	-0,516552	0,007237	0,030633	-3,046695	protein_coding	CDK4	cyclin dependent kinase 4 [Source:HGNC Symbol;Acc:HGNC:1773]	12
ENSG00000164949	5,688294	-1,374547	0,00724	0,03064	-3,046471	protein_coding	GEM	GTP binding protein overexpressed in skeletal muscle [Source:HGNC Symbol;Acc:HGNC:4234]	8
ENSG00000105549	-0,755398	2,823623	0,007247	0,030653	3,046025	protein_coding	THEG	theg spermatid protein [Source:HGNC Symbol;Acc:HGNC:13706]	19
ENSG00000134256	0,307759	1,724762	0,007247	0,030653	3,04604	protein_coding	CD101	CD101 molecule [Source:HGNC Symbol;Acc:HGNC:5949]	1
ENSG00000228486	1,389006	1,275169	0,00725	0,030657	3,045845	protein_coding	C2orf92	chromosome 2 open reading frame 92 [Source:HGNC Symbol;Acc:HGNC:49272]	2
ENSG00000173599	4,278631	-1,020705	0,007253	0,030663	-3,045632	protein_coding	PC	pyruvate carboxylase [Source:HGNC Symbol;Acc:HGNC:8636]	11
ENSG00000117245	1,062672	1,664492	0,007272	0,030694	3,044451	protein_coding	KIF17	kinesin family member 17 [Source:HGNC Symbol;Acc:HGNC:19167]	1
ENSG00000196338	1,298825	2,190641	0,007267	0,030694	3,044746	protein_coding	NLGN3	neuroligin 3 [Source:HGNC Symbol;Acc:HGNC:14289]	X
ENSG00000114686	5,546339	-0,518062	0,007272	0,030694	-3,044426	protein_coding	MRPL3	mitochondrial ribosomal protein L3 [Source:HGNC Symbol;Acc:HGNC:10379]	3
ENSG00000137216	5,714291	-0,64234	0,007274	0,030694	-3,044309	protein_coding	TMEM63B	transmembrane protein 63B [Source:HGNC Symbol;Acc:HGNC:17735]	6
ENSG00000250722	6,465625	1,599763	0,007265	0,030694	3,044905	protein_coding	SELENOP	selenoprotein P [Source:HGNC Symbol;Acc:HGNC:10751]	5
ENSG00000142166	6,46875	-0,575805	0,007274	0,030694	-3,044305	protein_coding	IFNAR1	interferon alpha and beta receptor subunit 1 [Source:HGNC Symbol;Acc:HGNC:5432]	21
ENSG00000143612	6,937502	-0,636564	0,007265	0,030694	-3,044902	protein_coding	C1orf43	chromosome 1 open reading frame 43 [Source:HGNC Symbol;Acc:HGNC:29876]	1
ENSG00000152592	-2,384804	1,918559	0,007281	0,030707	3,043868	protein_coding	DMP1	dentin matrix acidic phosphoprotein 1 [Source:HGNC Symbol;Acc:HGNC:2932]	4
ENSG00000286522	6,058783	1,02533	0,00728	0,030707	3,04391	protein_coding	H3C2	H3 clustered histone 2 [Source:HGNC Symbol;Acc:HGNC:4776]	6
ENSG00000143793	4,343695	0,733336	0,007286	0,030722	3,043528	protein_coding	C1orf35	chromosome 1 open reading frame 35 [Source:HGNC Symbol;Acc:HGNC:19032]	1
ENSG00000182158	7,13776	-1,072586	0,00729	0,030728	-3,043313	protein_coding	CREB3L2	cAMP responsive element binding protein 3 like 2 [Source:HGNC Symbol;Acc:HGNC:23720]	7
ENSG00000110455	3,127074	1,063208	0,007298	0,030755	3,042782	protein_coding	ACCS	1-aminocyclopropane-1-carboxylate synthase homolog (inactive) [Source:HGNC Symbol;Acc:HGNC:23989]	11
ENSG00000175793	1,490318	-3,952065	0,007307	0,030785	-3,042208	protein_coding	SFN	stratifin [Source:HGNC Symbol;Acc:HGNC:10773]	1
ENSG00000147606	-0,653228	2,860678	0,00731	0,030791	3,041992	protein_coding	SLC26A7	solute carrier family 26 member 7 [Source:HGNC Symbol;Acc:HGNC:14467]	8
ENSG00000130749	6,649352	0,578816	0,007319	0,030819	3,041446	protein_coding	ZC3H4	zinc finger CCCH-type containing 4 [Source:HGNC Symbol;Acc:HGNC:17808]	19
ENSG00000196715	5,91558	-0,604165	0,007321	0,030822	-3,041286	protein_coding	VKORC1L1	vitamin K epoxide reductase complex subunit 1 like 1 [Source:HGNC Symbol;Acc:HGNC:21492]	7
ENSG00000122965	6,148311	0,539944	0,007323	0,030822	3,041162	protein_coding	RBM19	RNA binding motif protein 19 [Source:HGNC Symbol;Acc:HGNC:29098]	12
ENSG00000115459	3,732406	0,615114	0,007325	0,030822	3,041033	protein_coding	ELMOD3	ELMO domain containing 3 [Source:HGNC Symbol;Acc:HGNC:26158]	2
ENSG00000181450	3,733382	0,83931	0,007342	0,030876	3,039979	protein_coding	ZNF678	zinc finger protein 678 [Source:HGNC Symbol;Acc:HGNC:28652]	1
ENSG00000033867	6,175136	-0,827956	0,00734	0,030876	-3,040082	protein_coding	SLC4A7	solute carrier family 4 member 7 [Source:HGNC Symbol;Acc:HGNC:11033]	3
ENSG00000130701	-0,27437	3,731475	0,00735	0,030885	3,039486	protein_coding	RBBP8NL	RBBP8 N-terminal like [Source:HGNC Symbol;Acc:HGNC:16144]	20
ENSG00000105609	0,58058	2,400018	0,007349	0,030885	3,039529	protein_coding	LILRB5	leukocyte immunoglobulin like receptor B5 [Source:HGNC Symbol;Acc:HGNC:6609]	19
ENSG00000085514	2,060614	1,317555	0,007348	0,030885	3,039559	protein_coding	PILRA	paired immunoglobin like type 2 receptor alpha [Source:HGNC Symbol;Acc:HGNC:20396]	7
ENSG00000170266	5,750093	-0,553652	0,007354	0,030894	-3,039218	protein_coding	GLB1	galactosidase beta 1 [Source:HGNC Symbol;Acc:HGNC:4298]	3
ENSG00000166266	5,765793	-0,524393	0,00736	0,030912	-3,038839	protein_coding	CUL5	cullin 5 [Source:HGNC Symbol;Acc:HGNC:2556]	11
ENSG00000196754	-0,83637	-2,132981	0,00738	0,03098	-3,037544	protein_coding	S100A2	S100 calcium binding protein A2 [Source:HGNC Symbol;Acc:HGNC:10492]	1
ENSG00000134086	6,068906	0,514105	0,00738	0,03098	3,037584	protein_coding	VHL	von Hippel-Lindau tumor suppressor [Source:HGNC Symbol;Acc:HGNC:12687]	3
ENSG00000188021	5,950232	-0,538026	0,007382	0,03098	-3,037446	protein_coding	UBQLN2	ubiquilin 2 [Source:HGNC Symbol;Acc:HGNC:12509]	X
ENSG00000162390	3,917149	1,162915	0,007384	0,030981	3,037313	protein_coding	ACOT11	acyl-CoA thioesterase 11 [Source:HGNC Symbol;Acc:HGNC:18156]	1
ENSG00000172915	6,185634	0,971317	0,007386	0,030982	3,037181	protein_coding	NBEA	neurobeachin [Source:HGNC Symbol;Acc:HGNC:7648]	13
ENSG00000139160	1,704289	0,997767	0,007391	0,030996	3,036835	protein_coding	ETFBKMT	electron transfer flavoprotein subunit beta lysine methyltransferase [Source:HGNC Symbol;Acc:HGNC:28739]	12

ENSG00000131015	1,619632	-1,695768	0,007415	0,031088	-3,035339	protein_coding	ULBP2	UL16 binding protein 2 [Source:HGNC Symbol;Acc:HGNC:14894]	6
ENSG00000152332	7,011833	-0,677953	0,007435	0,031164	-3,03408	protein_coding	UHMK1	U2AF homology motif kinase 1 [Source:HGNC Symbol;Acc:HGNC:19683]	1
ENSG00000205085	1,53046	1,362759	0,007439	0,031173	3,033829	protein_coding	FAM71F2	family with sequence similarity 71 member F2 [Source:HGNC Symbol;Acc:HGNC:27998]	7
ENSG00000148399	4,396114	0,766707	0,007443	0,031182	3,033563	protein_coding	DPH7	diphthamide biosynthesis 7 [Source:HGNC Symbol;Acc:HGNC:25199]	9
ENSG00000089234	5,224714	-0,401974	0,007449	0,031197	-3,03323	protein_coding	BRAP	BRCA1 associated protein [Source:HGNC Symbol;Acc:HGNC:1099]	12
ENSG00000166819	-0,495937	2,933436	0,007456	0,031219	3,032777	protein_coding	PLIN1	perilipin 1 [Source:HGNC Symbol;Acc:HGNC:9076]	15
ENSG00000086065	6,000242	-0,59971	0,007467	0,031259	-3,032061	protein_coding	CHMP5	charged multivesicular body protein 5 [Source:HGNC Symbol;Acc:HGNC:26942]	9
ENSG00000143252	4,385279	-0,478902	0,007483	0,031308	-3,031082	protein_coding	SDHC	succinate dehydrogenase complex subunit C [Source:HGNC Symbol;Acc:HGNC:10682]	1
ENSG00000271601	5,108966	-0,873351	0,007482	0,031308	-3,031158	protein_coding	LIX1L	limb and CNS expressed 1 like [Source:HGNC Symbol;Acc:HGNC:28715]	1
ENSG00000162869	4,835399	0,567814	0,007485	0,03131	3,030936	protein_coding	PPP1R21	protein phosphatase 1 regulatory subunit 21 [Source:HGNC Symbol;Acc:HGNC:30595]	2
ENSG00000109255	-0,96146	2,663183	0,007494	0,031337	3,030418	protein_coding	NMU	neuromedin U [Source:HGNC Symbol;Acc:HGNC:7859]	4
ENSG00000059588	4,945994	1,245156	0,007504	0,03137	3,029801	protein_coding	TARBP1	TAR (HIV-1) RNA binding protein 1 [Source:HGNC Symbol;Acc:HGNC:11568]	1
ENSG00000168517	1,793537	0,860409	0,007517	0,031413	3,028958	protein_coding	HEXIM2	HEXIM P-TEFb complex subunit 2 [Source:HGNC Symbol;Acc:HGNC:28591]	17
ENSG00000137409	6,87808	-0,404519	0,007518	0,031413	-3,028926	protein_coding	MTCH1	mitochondrial carrier 1 [Source:HGNC Symbol;Acc:HGNC:17586]	6
ENSG00000075043	-0,339217	3,512147	0,007521	0,031418	3,028727	protein_coding	KCNQ2	potassium voltage-gated channel subfamily Q member 2 [Source:HGNC Symbol;Acc:HGNC:6296]	20
ENSG00000175518	-1,433654	2,465394	0,007527	0,031435	3,028352	protein_coding	UBQLNL	ubiquilin like [Source:HGNC Symbol;Acc:HGNC:28294]	11
ENSG00000011590	-1,593458	2,566052	0,007538	0,031471	3,02765	protein_coding	ZBTB32	zinc finger and BTB domain containing 32 [Source:HGNC Symbol;Acc:HGNC:16763]	19
ENSG00000088766	3,686803	-0,550935	0,007539	0,031471	-3,027583	protein_coding	CRLS1	cardiolipin synthase 1 [Source:HGNC Symbol;Acc:HGNC:16148]	20
ENSG00000283154	2,576088	-0,828858	0,007547	0,03148	-3,027091	protein_coding	IQCJ-SCHIP	IQCJ-SCHIP1 readthrough [Source:HGNC Symbol;Acc:HGNC:38842]	3
ENSG00000102393	4,32241	-0,820656	0,007547	0,03148	-3,027145	protein_coding	GLA	galactosidase alpha [Source:HGNC Symbol;Acc:HGNC:4296]	X
ENSG00000115457	7,435135	1,509833	0,007546	0,03148	3,027175	protein_coding	IGFBP2	insulin like growth factor binding protein 2 [Source:HGNC Symbol;Acc:HGNC:5471]	2
ENSG00000170011	2,987192	2,119877	0,007551	0,031488	3,026848	protein_coding	MYRIP	myosin VIIA and Rab interacting protein [Source:HGNC Symbol;Acc:HGNC:19156]	3
ENSG00000196873	1,51761	0,961217	0,00756	0,031518	3,026285	protein_coding	CBWD3	COBW domain containing 3 [Source:HGNC Symbol;Acc:HGNC:18519]	9
ENSG00000102100	4,133387	-0,732992	0,007563	0,031521	-3,026117	protein_coding	SLC35A2	solute carrier family 35 member A2 [Source:HGNC Symbol;Acc:HGNC:11022]	X
ENSG00000137876	5,628838	-0,630606	0,007567	0,031525	-3,025883	protein_coding	RSL24D1	ribosomal L24 domain containing 1 [Source:HGNC Symbol;Acc:HGNC:18479]	15
ENSG00000163374	6,439702	0,735768	0,007568	0,031525	3,025824	protein_coding	YY1AP1	YY1 associated protein 1 [Source:HGNC Symbol;Acc:HGNC:30935]	1
ENSG00000135951	3,338976	1,276618	0,007573	0,031529	3,025516	protein_coding	TSGA10	testis specific 10 [Source:HGNC Symbol;Acc:HGNC:14927]	2
ENSG00000158169	4,167056	0,803359	0,007571	0,031529	3,025635	protein_coding	FANCC	FA complementation group C [Source:HGNC Symbol;Acc:HGNC:3584]	9
ENSG00000226124	0,929104	1,377065	0,007585	0,031573	3,024749	protein_coding	FTCDNL1	formiminotransferase cyclodeaminase N-terminal like [Source:HGNC Symbol;Acc:HGNC:48661]	2
ENSG00000161981	3,848877	0,679952	0,007591	0,031588	3,024414	protein_coding	SNRNP25	small nuclear ribonucleoprotein U11/U12 subunit 25 [Source:HGNC Symbol;Acc:HGNC:14161]	16
ENSG00000203663	-2,815807	2,233928	0,007597	0,031607	3,024008	protein_coding	OR2L2	olfactory receptor family 2 subfamily L member 2 [Source:HGNC Symbol;Acc:HGNC:8266]	1
ENSG00000149781	4,545951	-1,001196	0,007608	0,031645	-3,023334	protein_coding	FERMT3	fermitin family member 3 [Source:HGNC Symbol;Acc:HGNC:23151]	11
ENSG00000196724	2,592031	1,531701	0,007621	0,031688	3,02257	protein_coding	ZNF418	zinc finger protein 418 [Source:HGNC Symbol;Acc:HGNC:20647]	19
ENSG00000198919	5,189753	0,859246	0,007632	0,031717	3,021908	protein_coding	DZIP3	DAZ interacting zinc finger protein 3 [Source:HGNC Symbol;Acc:HGNC:30938]	3
ENSG00000197976	6,027267	0,702129	0,00763	0,031717	3,02202	protein_coding	AKAP17A	A-kinase anchoring protein 17A [Source:HGNC Symbol;Acc:HGNC:18783]	X
ENSG00000084754	7,240273	-0,652003	0,007639	0,03174	-3,021457	protein_coding	HADHA	hydroxyacyl-CoA dehydrogenase trifunctional multienzyme complex subunit alpha [Source:HGNC Symbol;Acc:HGNC	2
ENSG00000136379	3,637267	-1,102545	0,007644	0,031751	-3,021164	protein_coding	ABHD17C	abhydrolase domain containing 17C, depalmitoylase [Source:HGNC Symbol;Acc:HGNC:26925]	15
ENSG00000188735	4,793942	0,537785	0,007646	0,031753	3,021018	protein_coding	TMEM120[illegible]	transmembrane protein 120B [Source:HGNC Symbol;Acc:HGNC:32008]	12
ENSG00000116171	6,238996	-0,513402	0,00765	0,031759	-3,020812	protein_coding	SCP2	sterol carrier protein 2 [Source:HGNC Symbol;Acc:HGNC:10606]	1
ENSG00000143303	4,186253	0,723566	0,007652	0,031761	3,020671	protein_coding	RRNAD1	ribosomal RNA adenine dimethylase domain containing 1 [Source:HGNC Symbol;Acc:HGNC:24273]	1
ENSG00000255561	0,234528	-1,18524	0,007655	0,031767	-3,020459	protein_coding	FDXACB1	ferredoxin-fold anticodon binding domain containing 1 [Source:HGNC Symbol;Acc:HGNC:25110]	11
ENSG00000205670	-1,320289	1,350697	0,007661	0,031781	3,020091	protein_coding	SMIM11A	small integral membrane protein 11A [Source:HGNC Symbol;Acc:HGNC:1293]	21
ENSG00000105849	4,709508	-0,669603	0,007663	0,031781	-3,020013	protein_coding	POLR1F	RNA polymerase I subunit F [Source:HGNC Symbol;Acc:HGNC:18027]	7
ENSG00000164627	-0,06777	2,512642	0,00767	0,031802	3,019582	protein_coding	KIF6	kinesin family member 6 [Source:HGNC Symbol;Acc:HGNC:21202]	6
ENSG00000149043	-2,216825	2,439603	0,007673	0,031808	3,019375	protein_coding	SYT8	synaptotagmin 8 [Source:HGNC Symbol;Acc:HGNC:19264]	11
ENSG00000130640	6,018128	-0,383128	0,007708	0,031946	-3,017234	protein_coding	TUBGCP2	tubulin gamma complex associated protein 2 [Source:HGNC Symbol;Acc:HGNC:18599]	10
ENSG00000182400	5,403249	-0,399262	0,007728	0,03202	-3,016037	protein_coding	TRAPPC6B	trafficking protein particle complex 6B [Source:HGNC Symbol;Acc:HGNC:23066]	14
ENSG00000124215	0,344639	2,11207	0,007732	0,032022	3,015816	protein_coding	CDH26	cadherin 26 [Source:HGNC Symbol;Acc:HGNC:15902]	20
ENSG00000123338	3,609634	2,097689	0,007732	0,032022	3,015775	protein_coding	NCKAP1L	NCK associated protein 1 like [Source:HGNC Symbol;Acc:HGNC:4862]	12
ENSG00000015171	6,799023	-0,448941	0,007737	0,032032	-3,015502	protein_coding	ZMYND11	zinc finger MYND-type containing 11 [Source:HGNC Symbol;Acc:HGNC:16966]	10
ENSG00000100612	5,892498	-0,691847	0,007739	0,032034	-3,015354	protein_coding	DHRS7	dehydrogenase/reductase 7 [Source:HGNC Symbol;Acc:HGNC:21524]	14

ENSG00000180992	4,049326	-0,530823	0,00775	0,032069	-3,014732	protein_coding	MRPL14	mitochondrial ribosomal protein L14 [Source:HGNC Symbol;Acc:HGNC:14279]	6
ENSG00000187583	0,148178	-1,583953	0,007753	0,032075	-3,014518	protein_coding	PLEKHN1	pleckstrin homology domain containing N1 [Source:HGNC Symbol;Acc:HGNC:25284]	1
ENSG00000172889	4,163481	1,713418	0,007779	0,032172	3,012982	protein_coding	EGFL7	EGF like domain multiple 7 [Source:HGNC Symbol;Acc:HGNC:20594]	9
ENSG00000172738	0,061913	-1,355082	0,007785	0,032182	-3,01261	protein_coding	TMEM217	transmembrane protein 217 [Source:HGNC Symbol;Acc:HGNC:21238]	6
ENSG00000148926	4,906035	1,087349	0,007784	0,032182	3,012649	protein_coding	ADM	adrenomedullin [Source:HGNC Symbol;Acc:HGNC:259]	11
ENSG00000073067	-2,54285	2,111706	0,007792	0,032192	3,01222	protein_coding	CYP2W1	cytochrome P450 family 2 subfamily W member 1 [Source:HGNC Symbol;Acc:HGNC:20243]	7
ENSG00000101384	7,062975	-1,098301	0,007791	0,032192	-3,012265	protein_coding	JAG1	jagged canonical Notch ligand 1 [Source:HGNC Symbol;Acc:HGNC:6188]	20
ENSG00000185974	-2,119479	1,941275	0,007804	0,032234	3,011492	protein_coding	GRK1	G protein-coupled receptor kinase 1 [Source:HGNC Symbol;Acc:HGNC:10013]	13
ENSG00000186976	1,692321	1,490753	0,007815	0,032275	3,010786	protein_coding	EFCAB6	EF-hand calcium binding domain 6 [Source:HGNC Symbol;Acc:HGNC:24204]	22
ENSG00000174450	-3,296886	2,420222	0,007827	0,032298	3,010122	protein_coding	GOLGA6L2	golgin A6 family like 2 [Source:HGNC Symbol;Acc:HGNC:26695]	15
ENSG00000084453	0,032475	2,902433	0,007828	0,032298	3,010007	protein_coding	SLCO1A2	solute carrier organic anion transporter family member 1A2 [Source:HGNC Symbol;Acc:HGNC:10956]	12
ENSG00000111679	4,998127	1,249705	0,007825	0,032298	3,010203	protein_coding	PTPN6	protein tyrosine phosphatase non-receptor type 6 [Source:HGNC Symbol;Acc:HGNC:9658]	12
ENSG00000084090	6,987741	-0,494757	0,007829	0,032298	-3,009978	protein_coding	STARD7	StAR related lipid transfer domain containing 7 [Source:HGNC Symbol;Acc:HGNC:18063]	2
ENSG00000143756	5,060971	-0,562703	0,007832	0,032301	-3,00981	protein_coding	FBXO28	F-box protein 28 [Source:HGNC Symbol;Acc:HGNC:29046]	1
ENSG00000137078	-1,778202	2,938959	0,007864	0,032409	3,007885	protein_coding	SIT1	signaling threshold regulating transmembrane adaptor 1 [Source:HGNC Symbol;Acc:HGNC:17710]	9
ENSG00000099866	0,308421	1,311526	0,007866	0,032409	3,007784	protein_coding	MADCAM1	mucosal vascular addressin cell adhesion molecule 1 [Source:HGNC Symbol;Acc:HGNC:6765]	19
ENSG00000164107	3,073242	-1,984285	0,007864	0,032409	-3,007914	protein_coding	HAND2	heart and neural crest derivatives expressed 2 [Source:HGNC Symbol;Acc:HGNC:4808]	4
ENSG00000105221	6,789864	0,637611	0,007865	0,032409	3,007804	protein_coding	AKT2	AKT serine/threonine kinase 2 [Source:HGNC Symbol;Acc:HGNC:392]	19
ENSG00000166225	5,692606	-0,475667	0,007879	0,032454	-3,007017	protein_coding	FRS2	fibroblast growth factor receptor substrate 2 [Source:HGNC Symbol;Acc:HGNC:16971]	12
ENSG00000177189	6,74942	-0,850221	0,007887	0,032478	-3,006544	protein_coding	RPS6KA3	ribosomal protein S6 kinase A3 [Source:HGNC Symbol;Acc:HGNC:10432]	X
ENSG00000079257	3,713609	-1,554605	0,007905	0,03252	-3,005474	protein_coding	LXN	latexin [Source:HGNC Symbol;Acc:HGNC:13347]	3
ENSG00000100445	4,132801	0,635464	0,0079	0,03252	3,005771	protein_coding	SDR39U1	short chain dehydrogenase/reductase family 39U member 1 [Source:HGNC Symbol;Acc:HGNC:20275]	14
ENSG00000163156	4,166164	-0,663232	0,007903	0,03252	-3,00555	protein_coding	SCNM1	sodium channel modifier 1 [Source:HGNC Symbol;Acc:HGNC:23136]	1
ENSG00000115204	4,434753	-0,465765	0,007903	0,03252	-3,00558	protein_coding	MPV17	mitochondrial inner membrane protein MPV17 [Source:HGNC Symbol;Acc:HGNC:7224]	2
ENSG00000122224	-0,588403	2,31702	0,007907	0,03252	3,005351	protein_coding	LY9	lymphocyte antigen 9 [Source:HGNC Symbol;Acc:HGNC:6730]	1
ENSG00000160789	8,380776	-1,099153	0,007912	0,032531	-3,005068	protein_coding	LMNA	lamin A/C [Source:HGNC Symbol;Acc:HGNC:6636]	1
ENSG00000172661	5,061274	-0,417042	0,007928	0,03259	-3,004109	protein_coding	WASHC2C	WASH complex subunit 2C [Source:HGNC Symbol;Acc:HGNC:23414]	10
ENSG00000103544	5,015744	-0,889892	0,007934	0,032605	-3,003773	protein_coding	VPS35L	VPS35 endosomal protein sorting factor like [Source:HGNC Symbol;Acc:HGNC:24641]	16
ENSG00000124713	-0,358251	2,395225	0,007939	0,03261	3,003461	protein_coding	GNMT	glycine N-methyltransferase [Source:HGNC Symbol;Acc:HGNC:4415]	6
ENSG00000127191	4,433509	0,79617	0,007937	0,03261	3,003562	protein_coding	TRAF2	TNF receptor associated factor 2 [Source:HGNC Symbol;Acc:HGNC:12032]	9
ENSG00000177025	-0,521374	1,677726	0,007946	0,032628	3,003057	protein_coding	C19orf18	chromosome 19 open reading frame 18 [Source:HGNC Symbol;Acc:HGNC:28642]	19
ENSG00000113649	6,774651	0,47294	0,007947	0,032628	3,00298	protein_coding	TCERG1	transcription elongation regulator 1 [Source:HGNC Symbol;Acc:HGNC:15630]	5
ENSG00000122642	7,292937	-0,754981	0,007952	0,032638	-3,002711	protein_coding	FKBP9	FKBP prolyl isomerase 9 [Source:HGNC Symbol;Acc:HGNC:3725]	7
ENSG00000125246	1,922473	1,541855	0,007955	0,032645	3,0025	protein_coding	CLYBL	citramalyl-CoA lyase [Source:HGNC Symbol;Acc:HGNC:18355]	13
ENSG00000116205	3,904502	0,514339	0,007964	0,032671	3,002	protein_coding	TCEANC2	transcription elongation factor A N-terminal and central domain containing 2 [Source:HGNC Symbol;Acc:HGNC:264	1
ENSG00000083123	4,018778	0,694449	0,007971	0,032692	3,001584	protein_coding	BCKDHB	branched chain keto acid dehydrogenase E1 subunit beta [Source:HGNC Symbol;Acc:HGNC:987]	6
ENSG00000233954	2,374706	0,853234	0,007988	0,032735	3,000606	protein_coding	UQCRHL	ubiquinol-cytochrome c reductase hinge protein like [Source:HGNC Symbol;Acc:HGNC:51714]	1
ENSG00000106100	3,720398	1,326363	0,007989	0,032735	3,000492	protein_coding	NOD1	nucleotide binding oligomerization domain containing 1 [Source:HGNC Symbol;Acc:HGNC:16390]	7
ENSG00000126391	5,349939	-0,661598	0,007988	0,032735	-3,000571	protein_coding	FRMD8	FERM domain containing 8 [Source:HGNC Symbol;Acc:HGNC:25462]	11
ENSG00000182054	6,665565	0,995307	0,007984	0,032735	3,000784	protein_coding	IDH2	isocitrate dehydrogenase (NADP(+)) 2 [Source:HGNC Symbol;Acc:HGNC:5383]	15
ENSG00000107159	1,330979	2,848674	0,007993	0,032742	3,000275	protein_coding	CA9	carbonic anhydrase 9 [Source:HGNC Symbol;Acc:HGNC:1383]	9
ENSG00000198824	4,947952	-0,65523	0,008	0,032763	-2,999866	protein_coding	CHAMP1	chromosome alignment maintaining phosphoprotein 1 [Source:HGNC Symbol;Acc:HGNC:20311]	13
ENSG00000112667	3,835744	0,776322	0,008013	0,032807	2,999114	protein_coding	DNPH1	2'-deoxynucleoside 5'-phosphate N-hydrolase 1 [Source:HGNC Symbol;Acc:HGNC:21218]	6
ENSG00000115085	0,652278	2,566165	0,008023	0,032842	2,998506	protein_coding	ZAP70	zeta chain of T cell receptor associated protein kinase 70 [Source:HGNC Symbol;Acc:HGNC:12858]	2
ENSG00000092850	-0,963768	3,029033	0,008029	0,032856	2,998189	protein_coding	TEKT2	tektin 2 [Source:HGNC Symbol;Acc:HGNC:11725]	1
ENSG00000111962	3,570164	-1,268449	0,008041	0,032896	-2,997501	protein_coding	UST	uronyl 2-sulfotransferase [Source:HGNC Symbol;Acc:HGNC:17223]	6
ENSG00000104938	-2,91739	2,4546	0,00805	0,032917	2,99696	protein_coding	CLEC4M	C-type lectin domain family 4 member M [Source:HGNC Symbol;Acc:HGNC:13523]	19
ENSG00000111667	6,293678	-0,418307	0,00805	0,032917	-2,996983	protein_coding	USP5	ubiquitin specific peptidase 5 [Source:HGNC Symbol;Acc:HGNC:12628]	12
ENSG00000239920	-1,132019	2,243393	0,008055	0,032929	2,996678	protein_coding	AC104389.	novel transcript	11
ENSG00000166682	-0,165222	1,361627	0,008058	0,032932	2,99651	protein_coding	TMPRSS5	transmembrane serine protease 5 [Source:HGNC Symbol;Acc:HGNC:14908]	11
ENSG00000185278	5,28411	0,815061	0,008065	0,032954	2,99609	protein_coding	ZBTB37	zinc finger and BTB domain containing 37 [Source:HGNC Symbol;Acc:HGNC:28365]	1

ENSG00000162813	4,228035	-0,597188	0,008071	0,032968	-2,995765	protein_coding	BPNT1	3'(2'), 5'-bisphosphate nucleotidase 1 [Source:HGNC Symbol;Acc:HGNC:1096]	1
ENSG00000023902	5,638517	-0,941413	0,008073	0,032969	-2,99563	protein_coding	PLEKHO1	pleckstrin homology domain containing O1 [Source:HGNC Symbol;Acc:HGNC:24310]	1
ENSG00000158113	0,038599	2,30451	0,008079	0,032977	2,995298	protein_coding	LRRC43	leucine rich repeat containing 43 [Source:HGNC Symbol;Acc:HGNC:28562]	12
ENSG00000164296	3,211471	-0,555278	0,008079	0,032977	-2,995292	protein_coding	TIGD6	tigger transposable element derived 6 [Source:HGNC Symbol;Acc:HGNC:18332]	5
ENSG00000185432	4,096613	2,302289	0,008088	0,032988	2,994785	protein_coding	METTL7A	methyltransferase like 7A [Source:HGNC Symbol;Acc:HGNC:24550]	12
ENSG00000139233	3,537762	-0,648561	0,008087	0,032988	-2,994818	protein_coding	LLPH	LLP homolog, long-term synaptic facilitation factor [Source:HGNC Symbol;Acc:HGNC:28229]	12
ENSG00000111726	4,974163	-0,835188	0,008085	0,032988	-2,99493	protein_coding	CMAS	cytidine monophosphate N-acetylneuraminic acid synthetase [Source:HGNC Symbol;Acc:HGNC:18290]	12
ENSG00000178802	4,497119	0,561801	0,008091	0,032994	2,994576	protein_coding	MPI	mannose phosphate isomerase [Source:HGNC Symbol;Acc:HGNC:7216]	15
ENSG00000139684	5,055963	-0,520431	0,008097	0,033012	-2,994211	protein_coding	ESD	esterase D [Source:HGNC Symbol;Acc:HGNC:3465]	13
ENSG00000120656	3,907066	-0,700404	0,008103	0,033027	-2,993872	protein_coding	TAF12	TATA-box binding protein associated factor 12 [Source:HGNC Symbol;Acc:HGNC:11545]	1
ENSG00000156508	10,64947	-1,301251	0,008106	0,033029	-2,993727	protein_coding	EEF1A1	eukaryotic translation elongation factor 1 alpha 1 [Source:HGNC Symbol;Acc:HGNC:3189]	6
ENSG00000151632	2,636517	-4,033176	0,008132	0,033126	-2,992242	protein_coding	AKR1C2	aldo-keto reductase family 1 member C2 [Source:HGNC Symbol;Acc:HGNC:385]	10
ENSG00000095637	5,790004	1,415473	0,008136	0,033127	2,991985	protein_coding	SORBS1	sorbin and SH3 domain containing 1 [Source:HGNC Symbol;Acc:HGNC:14565]	10
ENSG00000080503	7,610212	0,569982	0,008136	0,033127	2,991999	protein_coding	SMARCA2	SWI/SNF related, matrix associated, actin dependent regulator of chromatin, subfamily a, member 2 [Source:HGNC	9
ENSG00000226174	0,644103	1,077407	0,00814	0,033134	2,99178	protein_coding	TEX22	testis expressed 22 [Source:HGNC Symbol;Acc:HGNC:40026]	14
ENSG00000171236	0,162959	2,649754	0,008167	0,033238	2,99019	protein_coding	LRG1	leucine rich alpha-2-glycoprotein 1 [Source:HGNC Symbol;Acc:HGNC:29480]	19
ENSG00000166743	-2,743192	2,979312	0,008182	0,033282	2,989345	protein_coding	ACSM1	acyl-CoA synthetase medium chain family member 1 [Source:HGNC Symbol;Acc:HGNC:18049]	16
ENSG00000153246	4,060692	-1,106579	0,008182	0,033282	-2,989349	protein_coding	PLA2R1	phospholipase A2 receptor 1 [Source:HGNC Symbol;Acc:HGNC:9042]	2
ENSG00000184465	4,404641	1,184952	0,008187	0,033292	2,989059	protein_coding	WDR27	WD repeat domain 27 [Source:HGNC Symbol;Acc:HGNC:21248]	6
ENSG00000116266	5,248071	-0,489202	0,008189	0,033292	-2,988963	protein_coding	STXBP3	syntaxin binding protein 3 [Source:HGNC Symbol;Acc:HGNC:11446]	1
ENSG00000138640	5,577574	0,985772	0,008199	0,033324	2,988399	protein_coding	FAM13A	family with sequence similarity 13 member A [Source:HGNC Symbol;Acc:HGNC:19367]	4
ENSG00000188404	0,321926	2,614304	0,008213	0,033375	2,987571	protein_coding	SELL	selectin L [Source:HGNC Symbol;Acc:HGNC:10720]	1
ENSG00000158517	0,351255	2,126679	0,008218	0,033377	2,987296	protein_coding	NCF1	neutrophil cytosolic factor 1 [Source:HGNC Symbol;Acc:HGNC:7660]	7
ENSG00000170113	5,273664	-0,870778	0,008218	0,033377	-2,987291	protein_coding	NIPA1	NIPA magnesium transporter 1 [Source:HGNC Symbol;Acc:HGNC:17043]	15
ENSG00000197912	6,718345	0,426543	0,00822	0,033377	2,987179	protein_coding	SPG7	SPG7 matrix AAA peptidase subunit, paraplegin [Source:HGNC Symbol;Acc:HGNC:11237]	16
ENSG00000174307	4,131687	-1,351473	0,008223	0,033383	-2,986989	protein_coding	PHLDA3	pleckstrin homology like domain family A member 3 [Source:HGNC Symbol;Acc:HGNC:8934]	1
ENSG00000141497	1,161442	2,387778	0,008242	0,033433	2,985943	protein_coding	ZMYND15	zinc finger MYND-type containing 15 [Source:HGNC Symbol;Acc:HGNC:20997]	17
ENSG00000151338	4,16452	1,063068	0,008238	0,033433	2,986161	protein_coding	MIPOL1	mirror-image polydactyly 1 [Source:HGNC Symbol;Acc:HGNC:21460]	14
ENSG00000074695	8,089281	-0,670609	0,008242	0,033433	-2,985931	protein_coding	LMAN1	lectin, mannose binding 1 [Source:HGNC Symbol;Acc:HGNC:6631]	18
ENSG00000173482	6,522135	-1,041705	0,008247	0,033444	-2,985665	protein_coding	PTPRM	protein tyrosine phosphatase receptor type M [Source:HGNC Symbol;Acc:HGNC:9675]	18
ENSG00000072134	5,355066	0,683285	0,008257	0,033476	2,985096	protein_coding	EPN2	epsin 2 [Source:HGNC Symbol;Acc:HGNC:18639]	17
ENSG00000112137	3,377565	1,571692	0,008267	0,033511	2,984498	protein_coding	PHACTR1	phosphatase and actin regulator 1 [Source:HGNC Symbol;Acc:HGNC:20990]	6
ENSG00000196730	5,544768	1,503858	0,00827	0,033513	2,984344	protein_coding	DAPK1	death associated protein kinase 1 [Source:HGNC Symbol;Acc:HGNC:2674]	9
ENSG00000106331	-3,304608	3,118897	0,008304	0,033638	2,982442	protein_coding	PAX4	paired box 4 [Source:HGNC Symbol;Acc:HGNC:8618]	7
ENSG00000140451	2,460331	1,368683	0,008307	0,033638	2,982262	protein_coding	PIF1	PIF1 5'-to-3' DNA helicase [Source:HGNC Symbol;Acc:HGNC:26220]	15
ENSG00000128016	6,529232	1,428911	0,008306	0,033638	2,982312	protein_coding	ZFP36	ZFP36 ring finger protein [Source:HGNC Symbol;Acc:HGNC:12862]	19
ENSG00000183077	4,014978	1,117806	0,008311	0,033647	2,982009	protein_coding	AFMID	arylformamidase [Source:HGNC Symbol;Acc:HGNC:20910]	17
ENSG00000173208	-1,266982	1,982597	0,008315	0,033651	2,981834	protein_coding	ABCD2	ATP binding cassette subfamily D member 2 [Source:HGNC Symbol;Acc:HGNC:66]	12
ENSG00000185361	3,80812	1,001581	0,008325	0,033686	2,981234	protein_coding	TNFAIP8L1	TNF alpha induced protein 8 like 1 [Source:HGNC Symbol;Acc:HGNC:28279]	19
ENSG00000144320	5,976109	-0,388046	0,008327	0,033686	-2,981115	protein_coding	LNPK	lunapark, ER junction formation factor [Source:HGNC Symbol;Acc:HGNC:21610]	2
ENSG00000204536	4,574938	0,984286	0,008332	0,03369	2,980851	protein_coding	CCHCR1	coiled-coil alpha-helical rod protein 1 [Source:HGNC Symbol;Acc:HGNC:13930]	6
ENSG00000183955	5,322356	-0,584301	0,008332	0,03369	-2,980826	protein_coding	KMT5A	lysine methyltransferase 5A [Source:HGNC Symbol;Acc:HGNC:29489]	12
ENSG00000118308	1,056376	2,553463	0,008342	0,033719	2,980316	protein_coding	IRAG2	inositol 1,4,5-triphosphate receptor associated 2 [Source:HGNC Symbol;Acc:HGNC:6690]	12
ENSG00000070444	5,753737	0,489933	0,008346	0,033728	2,980074	protein_coding	MNT	MAX network transcriptional repressor [Source:HGNC Symbol;Acc:HGNC:7188]	17
ENSG00000124092	-2,733944	2,506427	0,008368	0,033809	2,978827	protein_coding	CTCFL	CCCTC-binding factor like [Source:HGNC Symbol;Acc:HGNC:16234]	20
ENSG00000198538	4,906682	-0,753831	0,008387	0,033876	-2,977788	protein_coding	ZNF28	zinc finger protein 28 [Source:HGNC Symbol;Acc:HGNC:13073]	19
ENSG00000185379	4,142146	0,808417	0,008392	0,033888	2,977509	protein_coding	RAD51D	RAD51 paralog D [Source:HGNC Symbol;Acc:HGNC:9823]	17
ENSG00000116288	6,331496	-0,551989	0,008402	0,033922	-2,976925	protein_coding	PARK7	Parkinsonism associated deglycase [Source:HGNC Symbol;Acc:HGNC:16369]	1
ENSG00000197496	3,869016	-1,85765	0,008406	0,033926	-2,976688	protein_coding	SLC2A10	solute carrier family 2 member 10 [Source:HGNC Symbol;Acc:HGNC:13444]	20
ENSG00000162775	5,746202	0,624095	0,008407	0,033926	2,976635	protein_coding	RBM15	RNA binding motif protein 15 [Source:HGNC Symbol;Acc:HGNC:14959]	1
ENSG00000182199	6,676711	-0,674174	0,008414	0,033946	-2,976241	protein_coding	SHMT2	serine hydroxymethyltransferase 2 [Source:HGNC Symbol;Acc:HGNC:10852]	12

ENSG00000137731	0,772866	2,851203	0,008417	0,033948	2,976099	protein_coding	FXYD2	FXYD domain containing ion transport regulator 2 [Source:HGNC Symbol;Acc:HGNC:4026]	11
ENSG00000181826	5,548827	-0,860598	0,008429	0,033988	-2,975427	protein_coding	RELL1	RELT like 1 [Source:HGNC Symbol;Acc:HGNC:27379]	4
ENSG00000145545	4,750082	-0,826505	0,008437	0,03401	-2,975012	protein_coding	SRD5A1	steroid 5 alpha-reductase 1 [Source:HGNC Symbol;Acc:HGNC:11284]	5
ENSG00000184144	-1,601529	2,627772	0,008439	0,034011	2,974875	protein_coding	CNTN2	contactin 2 [Source:HGNC Symbol;Acc:HGNC:2172]	1
ENSG00000165238	5,881084	1,909024	0,008462	0,034094	2,973616	protein_coding	WNK2	WNK lysine deficient protein kinase 2 [Source:HGNC Symbol;Acc:HGNC:14542]	9
ENSG00000121022	5,522807	-0,415442	0,008464	0,034094	-2,973503	protein_coding	COPS5	COP9 signalosome subunit 5 [Source:HGNC Symbol;Acc:HGNC:2240]	8
ENSG00000161714	4,207842	-1,431056	0,008469	0,034108	-2,973196	protein_coding	PLCD3	phospholipase C delta 3 [Source:HGNC Symbol;Acc:HGNC:9061]	17
ENSG00000069509	3,32018	-0,51483	0,008482	0,034151	-2,972489	protein_coding	FUNDC1	FUN14 domain containing 1 [Source:HGNC Symbol;Acc:HGNC:28746]	X
ENSG00000110811	5,315556	-1,312939	0,008502	0,03422	-2,971383	protein_coding	P3H3	prolyl 3-hydroxylase 3 [Source:HGNC Symbol;Acc:HGNC:19318]	12
ENSG00000118482	7,709497	0,559559	0,008503	0,03422	2,971316	protein_coding	PHF3	PHD finger protein 3 [Source:HGNC Symbol;Acc:HGNC:8921]	6
ENSG00000055955	-1,130976	1,436245	0,008519	0,034248	2,970473	protein_coding	ITIH4	inter-alpha-trypsin inhibitor heavy chain 4 [Source:HGNC Symbol;Acc:HGNC:6169]	3
ENSG00000107984	0,802392	-3,514732	0,008513	0,034248	-2,970759	protein_coding	DKK1	dickkopf WNT signaling pathway inhibitor 1 [Source:HGNC Symbol;Acc:HGNC:2891]	10
ENSG00000148288	1,31827	1,221319	0,008517	0,034248	2,970583	protein_coding	GBGT1	globoside alpha-1,3-N-acetylgalactosaminyltransferase 1 (FORS blood group) [Source:HGNC Symbol;Acc:HGNC:2046	9
ENSG00000167536	3,504204	1,540261	0,008517	0,034248	2,97055	protein_coding	DHRS13	dehydrogenase/reductase 13 [Source:HGNC Symbol;Acc:HGNC:28326]	17
ENSG00000117114	5,625393	-1,532899	0,008529	0,03428	-2,969911	protein_coding	ADGRL2	adhesion G protein-coupled receptor L2 [Source:HGNC Symbol;Acc:HGNC:18582]	1
ENSG00000138614	4,731578	-0,600082	0,008558	0,034389	-2,968309	protein_coding	INTS14	integrator complex subunit 14 [Source:HGNC Symbol;Acc:HGNC:25372]	15
ENSG00000136715	5,171371	-0,47518	0,008562	0,034396	-2,968096	protein_coding	SAP130	Sin3A associated protein 130 [Source:HGNC Symbol;Acc:HGNC:29813]	2
ENSG00000074842	6,171496	-0,903385	0,008582	0,034468	-2,967	protein_coding	MYDGF	myeloid derived growth factor [Source:HGNC Symbol;Acc:HGNC:16948]	19
ENSG00000110090	6,592141	-0,657427	0,008585	0,034472	-2,966832	protein_coding	CPT1A	carnitine palmitoyltransferase 1A [Source:HGNC Symbol;Acc:HGNC:2328]	11
ENSG00000106809	-1,716844	3,567824	0,008597	0,034497	2,966179	protein_coding	OGN	osteoglycin [Source:HGNC Symbol;Acc:HGNC:8126]	9
ENSG00000197479	3,462168	1,251467	0,008598	0,034497	2,966148	protein_coding	PCDHB11	protocadherin beta 11 [Source:HGNC Symbol;Acc:HGNC:8682]	5
ENSG00000124406	4,117386	2,23022	0,008598	0,034497	2,966156	protein_coding	ATP8A1	ATPase phospholipid transporting 8A1 [Source:HGNC Symbol;Acc:HGNC:13531]	4
ENSG00000171119	0,123485	1,82797	0,00861	0,034538	2,96547	protein_coding	NRTN	neurturin [Source:HGNC Symbol;Acc:HGNC:8007]	19
ENSG00000124688	3,680134	-0,55917	0,008619	0,034566	-2,964984	protein_coding	MAD2L1BP	MAD2L1 binding protein [Source:HGNC Symbol;Acc:HGNC:21059]	6
ENSG00000242498	4,802103	0,658459	0,008642	0,03465	2,963728	protein_coding	ARPIN	actin related protein 2/3 complex inhibitor [Source:HGNC Symbol;Acc:HGNC:28782]	15
ENSG00000168569	2,937537	-0,644006	0,008661	0,034689	-2,962736	protein_coding	TMEM223	transmembrane protein 223 [Source:HGNC Symbol;Acc:HGNC:28464]	11
ENSG00000161996	4,775437	0,945673	0,00866	0,034689	2,962787	protein_coding	WDR90	WD repeat domain 90 [Source:HGNC Symbol;Acc:HGNC:26960]	16
ENSG00000122882	5,065473	-0,407493	0,008659	0,034689	-2,962804	protein_coding	ECD	ecdysoneless cell cycle regulator [Source:HGNC Symbol;Acc:HGNC:17029]	10
ENSG00000125447	5,56662	0,392942	0,008657	0,034689	2,962938	protein_coding	GGA3	golgi associated, gamma adaptin ear containing, ARF binding protein 3 [Source:HGNC Symbol;Acc:HGNC:17079]	17
ENSG00000189292	-1,768293	3,258231	0,008675	0,034737	2,961975	protein_coding	ALKAL2	ALK and LTK ligand 2 [Source:HGNC Symbol;Acc:HGNC:27683]	2
ENSG00000102890	3,328158	1,067174	0,008685	0,034771	2,961396	protein_coding	ELMO3	engulfment and cell motility 3 [Source:HGNC Symbol;Acc:HGNC:17289]	16
ENSG00000116406	6,923479	-0,698094	0,008703	0,034834	-2,960436	protein_coding	EDEM3	ER degradation enhancing alpha-mannosidase like protein 3 [Source:HGNC Symbol;Acc:HGNC:16787]	1
ENSG00000170242	6,854402	-0,423343	0,008706	0,034836	-2,96029	protein_coding	USP47	ubiquitin specific peptidase 47 [Source:HGNC Symbol;Acc:HGNC:20076]	11
ENSG00000260456	-0,141256	1,110327	0,008729	0,034899	2,959067	protein_coding	C16orf95	chromosome 16 open reading frame 95 [Source:HGNC Symbol;Acc:HGNC:40033]	16
ENSG00000126583	0,892502	2,603157	0,008729	0,034899	2,959057	protein_coding	PRKCG	protein kinase C gamma [Source:HGNC Symbol;Acc:HGNC:9402]	19
ENSG00000203791	3,372995	0,775581	0,008732	0,034899	2,958872	protein_coding	EEF1AKMT	EEF1A lysine methyltransferase 2 [Source:HGNC Symbol;Acc:HGNC:33787]	10
ENSG00000116667	4,393088	1,042551	0,008732	0,034899	2,958869	protein_coding	C1orf21	chromosome 1 open reading frame 21 [Source:HGNC Symbol;Acc:HGNC:15494]	1
ENSG00000092094	4,603825	0,561858	0,008732	0,034899	2,958891	protein_coding	OSGEP	O-sialoglycoprotein endopeptidase [Source:HGNC Symbol;Acc:HGNC:18028]	14
ENSG00000196456	2,93303	0,959748	0,008742	0,034928	2,958367	protein_coding	ZNF775	zinc finger protein 775 [Source:HGNC Symbol;Acc:HGNC:28501]	7
ENSG00000176248	5,210227	0,538929	0,00876	0,034994	2,95736	protein_coding	ANAPC2	anaphase promoting complex subunit 2 [Source:HGNC Symbol;Acc:HGNC:19989]	9
ENSG00000179111	-0,868851	1,782363	0,00877	0,03502	2,956837	protein_coding	HES7	hes family bHLH transcription factor 7 [Source:HGNC Symbol;Acc:HGNC:15977]	17
ENSG00000137766	0,006426	3,043772	0,008771	0,03502	2,956789	protein_coding	UNC13C	unc-13 homolog C [Source:HGNC Symbol;Acc:HGNC:23149]	15
ENSG00000100220	5,803387	-0,399453	0,008779	0,035042	-2,956377	protein_coding	RTCB	RNA 2',3'-cyclic phosphate and 5'-OH ligase [Source:HGNC Symbol;Acc:HGNC:26935]	22
ENSG00000158062	4,328239	0,676833	0,008788	0,035062	2,955873	protein_coding	UBXN11	UBX domain protein 11 [Source:HGNC Symbol;Acc:HGNC:30600]	1
ENSG00000023516	7,551681	-0,403404	0,008788	0,035062	-2,9559	protein_coding	AKAP11	A-kinase anchoring protein 11 [Source:HGNC Symbol;Acc:HGNC:369]	13
ENSG00000248144	-2,335105	2,64862	0,008806	0,035118	2,954906	protein_coding	ADH1C	alcohol dehydrogenase 1C (class I), gamma polypeptide [Source:HGNC Symbol;Acc:HGNC:251]	4
ENSG00000132677	-1,817976	3,388942	0,008806	0,035118	2,954951	protein_coding	RHBG	Rh family B glycoprotein [Source:HGNC Symbol;Acc:HGNC:14572]	1
ENSG00000198265	7,055408	0,384106	0,00881	0,035124	2,954705	protein_coding	HELZ	helicase with zinc finger [Source:HGNC Symbol;Acc:HGNC:16878]	17
ENSG00000261609	5,224901	-0,483216	0,008818	0,035146	-2,954291	protein_coding	GAN	gigaxonin [Source:HGNC Symbol;Acc:HGNC:4137]	16
ENSG00000162496	4,397641	1,808201	0,008836	0,03521	2,953324	protein_coding	DHRS3	dehydrogenase/reductase 3 [Source:HGNC Symbol;Acc:HGNC:17693]	1
ENSG00000178177	5,322113	0,775596	0,008843	0,035229	2,952963	protein_coding	LCORL	ligand dependent nuclear receptor corepressor like [Source:HGNC Symbol;Acc:HGNC:30776]	4

ENSG00000168710	7,256008	-0,638909	0,008852	0,035255	-2,952506	protein_coding	AHCYL1	adenosylhomocysteinase like 1 [Source:HGNC Symbol;Acc:HGNC:344]	1
ENSG00000172409	3,202803	-0,577771	0,008879	0,035356	-2,951045	protein_coding	CLP1	cleavage factor polyribonucleotide kinase subunit 1 [Source:HGNC Symbol;Acc:HGNC:16999]	11
ENSG00000227124	1,824815	0,879838	0,008899	0,035426	2,949998	protein_coding	ZNF717	zinc finger protein 717 [Source:HGNC Symbol;Acc:HGNC:29448]	3
ENSG00000183230	-0,528436	2,389461	0,008913	0,035437	2,949282	protein_coding	CTNNA3	catenin alpha 3 [Source:HGNC Symbol;Acc:HGNC:2511]	10
ENSG00000251664	0,391479	2,249839	0,008907	0,035437	2,949603	protein_coding	PCDHA12	protocadherin alpha 12 [Source:HGNC Symbol;Acc:HGNC:8666]	5
ENSG00000171174	0,851562	1,204102	0,008908	0,035437	2,949553	protein_coding	RBKS	ribokinase [Source:HGNC Symbol;Acc:HGNC:30325]	2
ENSG00000220205	5,056066	0,820974	0,008909	0,035437	2,949474	protein_coding	VAMP2	vesicle associated membrane protein 2 [Source:HGNC Symbol;Acc:HGNC:12643]	17
ENSG00000174891	5,663882	0,588847	0,008912	0,035437	2,949322	protein_coding	RSRC1	arginine and serine rich coiled-coil 1 [Source:HGNC Symbol;Acc:HGNC:24152]	3
ENSG00000128789	4,534896	-0,485817	0,008918	0,03545	-2,948999	protein_coding	PSMG2	proteasome assembly chaperone 2 [Source:HGNC Symbol;Acc:HGNC:24929]	18
ENSG00000174109	2,271918	-0,748507	0,008925	0,03546	-2,948629	protein_coding	C16orf91	chromosome 16 open reading frame 91 [Source:HGNC Symbol;Acc:HGNC:27558]	16
ENSG00000105879	5,193269	-0,437159	0,008924	0,03546	-2,948709	protein_coding	CBLL1	Cbl proto-oncogene like 1 [Source:HGNC Symbol;Acc:HGNC:21225]	7
ENSG00000198718	5,534163	0,447396	0,008936	0,035493	2,948078	protein_coding	TOGARAM	TOG array regulator of axonemal microtubules 1 [Source:HGNC Symbol;Acc:HGNC:19959]	14
ENSG00000149654	-0,648061	2,467141	0,008944	0,035519	2,947617	protein_coding	CDH22	cadherin 22 [Source:HGNC Symbol;Acc:HGNC:13251]	20
ENSG00000132570	4,189826	0,813434	0,008963	0,035575	2,946652	protein_coding	PCBD2	pterin-4 alpha-carbinolamine dehydratase 2 [Source:HGNC Symbol;Acc:HGNC:24474]	5
ENSG00000123636	7,077112	0,631262	0,008962	0,035575	2,9467	protein_coding	BAZ2B	bromodomain adjacent to zinc finger domain 2B [Source:HGNC Symbol;Acc:HGNC:963]	2
ENSG00000183255	7,647403	-0,48861	0,008972	0,035601	-2,946194	protein_coding	PTTG1IP	PTTG1 interacting protein [Source:HGNC Symbol;Acc:HGNC:13524]	21
ENSG00000159202	6,631111	-0,466729	0,008986	0,035652	-2,945414	protein_coding	UBE2Z	ubiquitin conjugating enzyme E2 Z [Source:HGNC Symbol;Acc:HGNC:25847]	17
ENSG00000080709	0,068915	2,583154	0,008996	0,035673	2,944905	protein_coding	KCNN2	potassium calcium-activated channel subfamily N member 2 [Source:HGNC Symbol;Acc:HGNC:6291]	5
ENSG00000184347	3,846028	2,610522	0,008996	0,035673	2,944918	protein_coding	SLIT3	slit guidance ligand 3 [Source:HGNC Symbol;Acc:HGNC:11087]	5
ENSG00000164776	0,521846	1,122594	0,009012	0,035727	2,944075	protein_coding	PHKG1	phosphorylase kinase catalytic subunit gamma 1 [Source:HGNC Symbol;Acc:HGNC:8930]	7
ENSG00000178700	3,417367	-0,660618	0,009015	0,03573	-2,943933	protein_coding	DHFR2	dihydrofolate reductase 2 [Source:HGNC Symbol;Acc:HGNC:27309]	3
ENSG00000162543	1,262338	1,478913	0,009021	0,035745	2,943612	protein_coding	UBXN10	UBX domain protein 10 [Source:HGNC Symbol;Acc:HGNC:26354]	1
ENSG00000152404	4,927852	0,6703	0,009025	0,035754	2,943389	protein_coding	CWF19L2	CWF19 like cell cycle control factor 2 [Source:HGNC Symbol;Acc:HGNC:26508]	11
ENSG00000123146	4,9195	-1,060177	0,009054	0,035859	-2,941892	protein_coding	ADGRE5	adhesion G protein-coupled receptor E5 [Source:HGNC Symbol;Acc:HGNC:1711]	19
ENSG00000126246	-0,881062	1,457175	0,009078	0,035934	2,940654	protein_coding	IGFLR1	IGF like family receptor 1 [Source:HGNC Symbol;Acc:HGNC:23620]	19
ENSG00000107338	4,115604	-0,984116	0,00908	0,035934	-2,940567	protein_coding	SHB	SH2 domain containing adaptor protein B [Source:HGNC Symbol;Acc:HGNC:10838]	9
ENSG00000156931	5,9086	0,399991	0,009077	0,035934	2,940684	protein_coding	VPS8	VPS8 subunit of CORVET complex [Source:HGNC Symbol;Acc:HGNC:29122]	3
ENSG00000115904	6,544951	-0,494552	0,009085	0,035946	-2,940303	protein_coding	SOS1	SOS Ras/Rac guanine nucleotide exchange factor 1 [Source:HGNC Symbol;Acc:HGNC:11187]	2
ENSG00000232070	-1,862028	1,459881	0,0091	0,035994	2,939531	protein_coding	TMEM253	transmembrane protein 253 [Source:HGNC Symbol;Acc:HGNC:32545]	14
ENSG00000162444	-0,554656	2,805968	0,009103	0,035994	2,939373	protein_coding	RBP7	retinol binding protein 7 [Source:HGNC Symbol;Acc:HGNC:30316]	1
ENSG00000167685	4,72765	0,77836	0,009104	0,035994	2,939336	protein_coding	ZNF444	zinc finger protein 444 [Source:HGNC Symbol;Acc:HGNC:16052]	19
ENSG00000267127	-0,733972	1,404619	0,009108	0,036001	2,939131	protein_coding	AC090360.	novel protein	18
ENSG00000113240	4,542545	0,659925	0,009112	0,036011	2,93888	protein_coding	CLK4	CDC like kinase 4 [Source:HGNC Symbol;Acc:HGNC:13659]	5
ENSG00000137944	3,820989	-0,821312	0,009116	0,036014	-2,938723	protein_coding	KYAT3	kynurenine aminotransferase 3 [Source:HGNC Symbol;Acc:HGNC:33238]	1
ENSG00000146281	4,455418	1,130737	0,009129	0,03605	2,938031	protein_coding	PM20D2	peptidase M20 domain containing 2 [Source:HGNC Symbol;Acc:HGNC:21408]	6
ENSG00000188706	5,610817	-0,626897	0,009127	0,03605	-2,938118	protein_coding	ZDHHC9	zinc finger DHHC-type palmitoyltransferase 9 [Source:HGNC Symbol;Acc:HGNC:18475]	X
ENSG00000112339	6,064483	-0,525099	0,009136	0,036068	-2,937684	protein_coding	HBS1L	HBS1 like translational GTPase [Source:HGNC Symbol;Acc:HGNC:4834]	6
ENSG00000132670	6,836709	-0,334194	0,009143	0,036088	-2,937301	protein_coding	PTPRA	protein tyrosine phosphatase receptor type A [Source:HGNC Symbol;Acc:HGNC:9664]	20
ENSG00000023287	6,983378	-0,465176	0,009149	0,036104	-2,936985	protein_coding	RB1CC1	RB1 inducible coiled-coil 1 [Source:HGNC Symbol;Acc:HGNC:15574]	8
ENSG00000136153	7,786873	-0,932708	0,00916	0,036136	-2,936452	protein_coding	LMO7	LIM domain 7 [Source:HGNC Symbol;Acc:HGNC:6646]	13
ENSG00000143178	1,433968	1,194479	0,009173	0,03618	2,935771	protein_coding	TBX19	T-box transcription factor 19 [Source:HGNC Symbol;Acc:HGNC:11596]	1
ENSG00000273154	-0,751301	1,373489	0,009188	0,036232	2,934984	protein_coding	AL121845.:	novel protein, ZGPAT-LIME1 readthrough	20
ENSG00000120324	3,079627	1,129381	0,009192	0,036236	2,934812	protein_coding	PCDHB10	protocadherin beta 10 [Source:HGNC Symbol;Acc:HGNC:8681]	5
ENSG00000185344	5,650002	-0,532402	0,0092	0,036262	-2,934371	protein_coding	ATP6V0A2	ATPase H+ transporting V0 subunit a2 [Source:HGNC Symbol;Acc:HGNC:18481]	12
ENSG00000214944	5,821693	1,210608	0,009206	0,036276	2,934073	protein_coding	ARHGEF28	Rho guanine nucleotide exchange factor 28 [Source:HGNC Symbol;Acc:HGNC:30322]	5
ENSG00000154814	4,157637	0,685284	0,009215	0,036301	2,93363	protein_coding	OXNAD1	oxidoreductase NAD binding domain containing 1 [Source:HGNC Symbol;Acc:HGNC:25128]	3
ENSG00000188996	-0,598331	1,371985	0,009245	0,036411	2,932103	protein_coding	HUS1B	HUS1 checkpoint clamp component B [Source:HGNC Symbol;Acc:HGNC:16485]	6
ENSG00000164024	5,150753	-0,483725	0,009253	0,036433	-2,9317	protein_coding	METAP1	methionyl aminopeptidase 1 [Source:HGNC Symbol;Acc:HGNC:15789]	4
ENSG00000176956	0,749231	3,440191	0,009268	0,036464	2,930956	protein_coding	LY6H	lymphocyte antigen 6 family member H [Source:HGNC Symbol;Acc:HGNC:6728]	8
ENSG00000158457	2,809303	1,678376	0,009265	0,036464	2,931072	protein_coding	TSPAN33	tetraspanin 33 [Source:HGNC Symbol;Acc:HGNC:28743]	7
ENSG00000178252	6,920679	0,527162	0,009267	0,036464	2,930988	protein_coding	WDR6	WD repeat domain 6 [Source:HGNC Symbol;Acc:HGNC:12758]	3

ENSG00000188763	-0,241438	-2,795948	0,009279	0,036501	-2,930375	protein_coding	FZD9	frizzled class receptor 9 [Source:HGNC Symbol;Acc:HGNC:4047]	7
ENSG00000161955	0,946638	1,344721	0,009286	0,03652	2,930018	protein_coding	TNFSF13	TNF superfamily member 13 [Source:HGNC Symbol;Acc:HGNC:11928]	17
ENSG00000157483	6,186381	-0,679252	0,009289	0,036521	-2,929891	protein_coding	MYO1E	myosin IE [Source:HGNC Symbol;Acc:HGNC:7599]	15
ENSG00000142405	-1,631508	1,817579	0,009296	0,036532	2,929539	protein_coding	NLRP12	NLR family pyrin domain containing 12 [Source:HGNC Symbol;Acc:HGNC:22938]	19
ENSG00000213901	-0,589351	1,335901	0,009296	0,036532	2,929513	protein_coding	SLC23A3	solute carrier family 23 member 3 [Source:HGNC Symbol;Acc:HGNC:20601]	2
ENSG00000197162	3,797359	0,674167	0,009303	0,036549	2,929187	protein_coding	ZNF785	zinc finger protein 785 [Source:HGNC Symbol;Acc:HGNC:26496]	16
ENSG00000215912	0,785224	1,530822	0,00931	0,036569	2,928814	protein_coding	TTC34	tetratricopeptide repeat domain 34 [Source:HGNC Symbol;Acc:HGNC:34297]	1
ENSG00000144048	4,012912	-0,630881	0,009316	0,036583	-2,928527	protein_coding	DUSP11	dual specificity phosphatase 11 [Source:HGNC Symbol;Acc:HGNC:3066]	2
ENSG00000042062	2,027351	-2,199795	0,009326	0,036615	-2,928002	protein_coding	RIPOR3	RIPOR family member 3 [Source:HGNC Symbol;Acc:HGNC:16168]	20
ENSG00000117625	5,291611	0,719281	0,00934	0,036662	2,927283	protein_coding	RCOR3	REST corepressor 3 [Source:HGNC Symbol;Acc:HGNC:25594]	1
ENSG00000203722	-1,63163	-1,622692	0,00935	0,036689	-2,926817	protein_coding	RAET1G	retinoic acid early transcript 1G [Source:HGNC Symbol;Acc:HGNC:16795]	6
ENSG00000162069	2,794797	2,229521	0,009354	0,036697	2,926603	protein_coding	BICDL2	BICD family like cargo adaptor 2 [Source:HGNC Symbol;Acc:HGNC:33584]	16
ENSG00000160908	4,840617	0,589557	0,009361	0,036707	2,926258	protein_coding	ZNF394	zinc finger protein 394 [Source:HGNC Symbol;Acc:HGNC:18832]	7
ENSG00000077235	7,553016	-0,406596	0,009358	0,036707	-2,926371	protein_coding	GTF3C1	general transcription factor IIIC subunit 1 [Source:HGNC Symbol;Acc:HGNC:4664]	16
ENSG00000163739	2,098035	-1,846388	0,009366	0,036718	-2,926004	protein_coding	CXCL1	C-X-C motif chemokine ligand 1 [Source:HGNC Symbol;Acc:HGNC:4602]	4
ENSG00000133454	0,094408	2,785394	0,00938	0,036747	2,925293	protein_coding	MYO18B	myosin XVIIIB [Source:HGNC Symbol;Acc:HGNC:18150]	22
ENSG00000084444	4,10346	1,152756	0,009377	0,036747	2,92543	protein_coding	FAM234B	family with sequence similarity 234 member B [Source:HGNC Symbol;Acc:HGNC:29288]	12
ENSG00000144659	4,6169	-0,415825	0,00938	0,036747	-2,925307	protein_coding	SLC25A38	solute carrier family 25 member 38 [Source:HGNC Symbol;Acc:HGNC:26054]	3
ENSG00000166557	6,722412	-0,71378	0,009389	0,036771	-2,924865	protein_coding	TMED3	transmembrane p24 trafficking protein 3 [Source:HGNC Symbol;Acc:HGNC:28889]	15
ENSG00000106546	5,781922	-1,097101	0,009405	0,036826	-2,924057	protein_coding	AHR	aryl hydrocarbon receptor [Source:HGNC Symbol;Acc:HGNC:348]	7
ENSG00000169252	0,325931	2,08477	0,009439	0,036949	2,92234	protein_coding	ADRB2	adrenoceptor beta 2 [Source:HGNC Symbol;Acc:HGNC:286]	5
ENSG00000167641	2,494057	2,021363	0,009441	0,036949	2,922255	protein_coding	PPP1R14A	protein phosphatase 1 regulatory inhibitor subunit 14A [Source:HGNC Symbol;Acc:HGNC:14871]	19
ENSG00000101916	-0,178532	2,078214	0,009445	0,036955	2,922033	protein_coding	TLR8	toll like receptor 8 [Source:HGNC Symbol;Acc:HGNC:15632]	X
ENSG00000170873	6,236462	0,677865	0,009447	0,036955	2,921958	protein_coding	MTSS1	MTSS I-BAR domain containing 1 [Source:HGNC Symbol;Acc:HGNC:20443]	8
ENSG00000185324	5,443098	0,571759	0,009457	0,036985	2,921464	protein_coding	CDK10	cyclin dependent kinase 10 [Source:HGNC Symbol;Acc:HGNC:1770]	16
ENSG00000078596	0,037386	3,04703	0,009464	0,037004	2,921114	protein_coding	ITM2A	integral membrane protein 2A [Source:HGNC Symbol;Acc:HGNC:6173]	X
ENSG00000074603	5,997286	-0,49748	0,009471	0,037015	-2,920741	protein_coding	DPP8	dipeptidyl peptidase 8 [Source:HGNC Symbol;Acc:HGNC:16490]	15
ENSG00000169714	7,679655	-0,593206	0,009471	0,037015	-2,920781	protein_coding	CNBP	CCHC-type zinc finger nucleic acid binding protein [Source:HGNC Symbol;Acc:HGNC:13164]	3
ENSG00000124507	1,519528	2,221668	0,009488	0,037072	2,919911	protein_coding	PACSIN1	protein kinase C and casein kinase substrate in neurons 1 [Source:HGNC Symbol;Acc:HGNC:8570]	6
ENSG00000143382	1,847152	1,690817	0,009508	0,037132	2,918918	protein_coding	ADAMTSL4	ADAMTS like 4 [Source:HGNC Symbol;Acc:HGNC:19706]	1
ENSG00000125730	5,94257	3,087288	0,009507	0,037132	2,918975	protein_coding	C3	complement C3 [Source:HGNC Symbol;Acc:HGNC:1318]	19
ENSG00000101407	5,375159	0,54526	0,009518	0,037161	2,918437	protein_coding	TTI1	TELO2 interacting protein 1 [Source:HGNC Symbol;Acc:HGNC:29029]	20
ENSG00000114656	1,238671	1,573358	0,009524	0,037169	2,918118	protein_coding	CFAP92	cilia and flagella associated protein 92 (putative) [Source:HGNC Symbol;Acc:HGNC:29231]	3
ENSG00000165959	5,251021	1,220068	0,009524	0,037169	2,918151	protein_coding	CLMN	calmin [Source:HGNC Symbol;Acc:HGNC:19972]	14
ENSG00000139144	-2,198599	2,518643	0,009538	0,037212	2,917453	protein_coding	PIK3C2G	phosphatidylinositol-4-phosphate 3-kinase catalytic subunit type 2 gamma [Source:HGNC Symbol;Acc:HGNC:8973]	12
ENSG00000189410	-0,306239	-1,51211	0,009544	0,037228	-2,917141	protein_coding	SH2D5	SH2 domain containing 5 [Source:HGNC Symbol;Acc:HGNC:28819]	1
ENSG00000174137	0,028283	1,011353	0,009556	0,037264	2,916581	protein_coding	FAM53A	family with sequence similarity 53 member A [Source:HGNC Symbol;Acc:HGNC:31860]	4
ENSG00000002016	3,375101	0,798032	0,009565	0,037291	2,916122	protein_coding	RAD52	RAD52 homolog, DNA repair protein [Source:HGNC Symbol;Acc:HGNC:9824]	12
ENSG00000171951	7,063893	-2,264536	0,009592	0,037388	-2,914783	protein_coding	SCG2	secretogranin II [Source:HGNC Symbol;Acc:HGNC:10575]	2
ENSG00000144868	1,902127	1,927832	0,009603	0,037424	2,914229	protein_coding	TMEM108	transmembrane protein 108 [Source:HGNC Symbol;Acc:HGNC:28451]	3
ENSG00000166342	-0,386	-2,806138	0,009616	0,037464	-2,913603	protein_coding	NETO1	neuropilin and tolloid like 1 [Source:HGNC Symbol;Acc:HGNC:13823]	18
ENSG00000118113	-1,173107	-2,806292	0,009625	0,037484	-2,913168	protein_coding	MMP8	matrix metallopeptidase 8 [Source:HGNC Symbol;Acc:HGNC:7175]	11
ENSG00000139405	3,942631	-0,651602	0,009626	0,037484	-2,913128	protein_coding	RITA1	RBPJ interacting and tubulin associated 1 [Source:HGNC Symbol;Acc:HGNC:25925]	12
ENSG00000120057	-1,638893	3,318883	0,009632	0,037494	2,912855	protein_coding	SFRP5	secreted frizzled related protein 5 [Source:HGNC Symbol;Acc:HGNC:10779]	10
ENSG00000136813	7,264485	-0,406915	0,009633	0,037494	-2,912776	protein_coding	ECPAS	Ecm29 proteasome adaptor and scaffold [Source:HGNC Symbol;Acc:HGNC:29020]	9
ENSG00000110013	4,63367	-0,861762	0,009652	0,03755	-2,911854	protein_coding	SIAE	sialic acid acetylesterase [Source:HGNC Symbol;Acc:HGNC:18187]	11
ENSG00000165689	5,22385	-0,481421	0,009651	0,03755	-2,911893	protein_coding	ENTR1	endosome associated trafficking regulator 1 [Source:HGNC Symbol;Acc:HGNC:10667]	9
ENSG00000140057	1,45241	1,728177	0,009672	0,037611	2,910862	protein_coding	AK7	adenylate kinase 7 [Source:HGNC Symbol;Acc:HGNC:20091]	14
ENSG00000139793	6,710563	-0,953811	0,009671	0,037611	-2,910917	protein_coding	MBNL2	muscleblind like splicing regulator 2 [Source:HGNC Symbol;Acc:HGNC:16746]	13
ENSG00000130304	3,633705	1,128846	0,009677	0,037619	2,910648	protein_coding	SLC27A1	solute carrier family 27 member 1 [Source:HGNC Symbol;Acc:HGNC:10995]	19
ENSG00000165806	4,753423	-0,835207	0,009682	0,037631	-2,910395	protein_coding	CASP7	caspase 7 [Source:HGNC Symbol;Acc:HGNC:1508]	10

ENSG00000114026	4,288926	0,749807	0,009687	0,03764	2,910165	protein_coding	OGG1	8-oxoguanine DNA glycosylase [Source:HGNC Symbol;Acc:HGNC:8125]	3
ENSG00000138496	5,921182	1,092672	0,009702	0,037691	2,909415	protein_coding	PARP9	poly(ADP-ribose) polymerase family member 9 [Source:HGNC Symbol;Acc:HGNC:24118]	3
ENSG00000174453	-2,223504	2,362398	0,009718	0,037735	2,908645	protein_coding	VWC2L	von Willebrand factor C domain containing 2 like [Source:HGNC Symbol;Acc:HGNC:37203]	2
ENSG00000112936	2,319201	6,215822	0,009716	0,037735	2,908735	protein_coding	C7	complement C7 [Source:HGNC Symbol;Acc:HGNC:1346]	5
ENSG00000169064	-1,42995	2,486752	0,009724	0,037738	2,908383	protein_coding	ZBBX	zinc finger B-box domain containing [Source:HGNC Symbol;Acc:HGNC:26245]	3
ENSG00000089250	-0,057771	2,966298	0,009724	0,037738	2,908385	protein_coding	NOS1	nitric oxide synthase 1 [Source:HGNC Symbol;Acc:HGNC:7872]	12
ENSG00000102781	5,402936	-0,790115	0,009733	0,037766	-2,907919	protein_coding	KATNAL1	katanin catalytic subunit A1 like 1 [Source:HGNC Symbol;Acc:HGNC:28361]	13
ENSG00000183628	1,724929	0,961755	0,009739	0,037769	2,907662	protein_coding	DGCR6	DiGeorge syndrome critical region gene 6 [Source:HGNC Symbol;Acc:HGNC:2846]	22
ENSG00000073060	5,766055	0,969556	0,009739	0,037769	2,90766	protein_coding	SCARB1	scavenger receptor class B member 1 [Source:HGNC Symbol;Acc:HGNC:1664]	12
ENSG00000183605	3,331688	0,639908	0,009748	0,037795	2,907225	protein_coding	SFXN4	sideroflexin 4 [Source:HGNC Symbol;Acc:HGNC:16088]	10
ENSG00000043462	3,577821	1,879307	0,009754	0,037809	2,906935	protein_coding	LCP2	lymphocyte cytosolic protein 2 [Source:HGNC Symbol;Acc:HGNC:6529]	5
ENSG00000157423	2,223733	2,43793	0,009756	0,037809	2,906822	protein_coding	HYDIN	HYDIN axonemal central pair apparatus protein [Source:HGNC Symbol;Acc:HGNC:19368]	16
ENSG00000070047	6,985783	0,445316	0,009767	0,037842	2,906297	protein_coding	PHRF1	PHD and ring finger domains 1 [Source:HGNC Symbol;Acc:HGNC:24351]	11
ENSG00000205923	-2,031023	1,778451	0,009782	0,037874	2,905568	protein_coding	CEMP1	cementum protein 1 [Source:HGNC Symbol;Acc:HGNC:32553]	16
ENSG00000146733	4,599204	-0,693656	0,009779	0,037874	-2,905717	protein_coding	PSPH	phosphoserine phosphatase [Source:HGNC Symbol;Acc:HGNC:9577]	7
ENSG00000172809	7,792043	0,682377	0,00978	0,037874	2,905645	protein_coding	RPL38	ribosomal protein L38 [Source:HGNC Symbol;Acc:HGNC:10349]	17
ENSG00000197858	6,14779	-0,400941	0,009791	0,037898	-2,905154	protein_coding	GPAA1	glycosylphosphatidylinositol anchor attachment 1 [Source:HGNC Symbol;Acc:HGNC:4446]	8
ENSG00000112851	7,515286	-0,393277	0,009811	0,03797	-2,904151	protein_coding	ERBIN	erbb2 interacting protein [Source:HGNC Symbol;Acc:HGNC:15842]	5
ENSG00000197256	7,406036	-1,023606	0,009837	0,038061	-2,90292	protein_coding	KANK2	KN motif and ankyrin repeat domains 2 [Source:HGNC Symbol;Acc:HGNC:29300]	19
ENSG00000197826	-2,621509	2,17568	0,009843	0,038062	2,902653	protein_coding	CFAP299	cilia and flagella associated protein 299 [Source:HGNC Symbol;Acc:HGNC:28554]	4
ENSG00000174442	4,066625	-0,865066	0,009845	0,038062	-2,902564	protein_coding	ZWILCH	zwilch kinetochore protein [Source:HGNC Symbol;Acc:HGNC:25468]	15
ENSG00000057757	5,196932	-0,49167	0,009842	0,038062	-2,90269	protein_coding	PITHD1	PITH domain containing 1 [Source:HGNC Symbol;Acc:HGNC:25022]	1
ENSG00000125375	3,680646	0,732718	0,009849	0,038069	2,902375	protein_coding	DMAC2L	distal membrane arm assembly complex 2 like [Source:HGNC Symbol;Acc:HGNC:18799]	14
ENSG00000253485	2,462366	1,898896	0,009874	0,038156	2,901187	protein_coding	PCDHGA5	protocadherin gamma subfamily A, 5 [Source:HGNC Symbol;Acc:HGNC:8703]	5
ENSG00000135898	-2,188685	2,419448	0,009884	0,038171	2,900666	protein_coding	GPR55	G protein-coupled receptor 55 [Source:HGNC Symbol;Acc:HGNC:4511]	2
ENSG00000188321	3,651236	0,624017	0,009883	0,038171	2,900753	protein_coding	ZNF559	zinc finger protein 559 [Source:HGNC Symbol;Acc:HGNC:28197]	19
ENSG00000106635	5,86501	-0,499944	0,009884	0,038171	-2,900698	protein_coding	BCL7B	BAF chromatin remodeling complex subunit BCL7B [Source:HGNC Symbol;Acc:HGNC:1005]	7
ENSG00000087301	4,748143	0,797232	0,009889	0,03818	2,900435	protein_coding	TXNDC16	thioredoxin domain containing 16 [Source:HGNC Symbol;Acc:HGNC:19965]	14
ENSG00000187164	5,899726	0,991982	0,009893	0,038187	2,900236	protein_coding	SHTN1	shootin 1 [Source:HGNC Symbol;Acc:HGNC:29319]	10
ENSG00000146151	-1,519528	2,354696	0,009914	0,038248	2,899265	protein_coding	HMGCLL1	3-hydroxymethyl-3-methylglutaryl-CoA lyase like 1 [Source:HGNC Symbol;Acc:HGNC:21359]	6
ENSG00000144063	0,028455	-2,832142	0,009913	0,038248	-2,899315	protein_coding	MALL	mal, T cell differentiation protein like [Source:HGNC Symbol;Acc:HGNC:6818]	2
ENSG00000103154	-0,834873	2,895682	0,00992	0,038259	2,898992	protein_coding	NECAB2	N-terminal EF-hand calcium binding protein 2 [Source:HGNC Symbol;Acc:HGNC:23746]	16
ENSG00000121005	1,279032	2,444958	0,009921	0,038259	2,898909	protein_coding	CRISPLD1	cysteine rich secretory protein LCCL domain containing 1 [Source:HGNC Symbol;Acc:HGNC:18206]	8
ENSG00000178222	0,082135	2,976571	0,009925	0,038262	2,89876	protein_coding	RNF212	ring finger protein 212 [Source:HGNC Symbol;Acc:HGNC:27729]	4
ENSG00000155959	5,047253	-0,469049	0,009927	0,038264	-2,898628	protein_coding	VBP1	VHL binding protein 1 [Source:HGNC Symbol;Acc:HGNC:12662]	X
ENSG00000178904	5,524696	-0,566773	0,00993	0,038264	-2,898511	protein_coding	DPY19L3	dpy-19 like C-mannosyltransferase 3 [Source:HGNC Symbol;Acc:HGNC:27120]	19
ENSG00000171121	2,077302	1,279786	0,009949	0,038329	2,8976	protein_coding	KCNMB3	potassium calcium-activated channel subfamily M regulatory beta subunit 3 [Source:HGNC Symbol;Acc:HGNC:6287]	3
ENSG00000167861	5,591534	1,281161	0,009967	0,03839	2,896745	protein_coding	HID1	HID1 domain containing [Source:HGNC Symbol;Acc:HGNC:15736]	17
ENSG00000214212	-0,48676	1,500109	0,009973	0,038399	2,896466	protein_coding	C19orf38	chromosome 19 open reading frame 38 [Source:HGNC Symbol;Acc:HGNC:34073]	19
ENSG00000174780	6,812431	-0,586469	0,009974	0,038399	-2,89641	protein_coding	SRP72	signal recognition particle 72 [Source:HGNC Symbol;Acc:HGNC:11303]	4
ENSG00000255398	-2,620255	2,395504	0,009986	0,038435	2,895851	protein_coding	HCAR3	hydroxycarboxylic acid receptor 3 [Source:HGNC Symbol;Acc:HGNC:16824]	12
ENSG00000171316	7,211242	1,241572	0,009998	0,038473	2,895285	protein_coding	CHD7	chromodomain helicase DNA binding protein 7 [Source:HGNC Symbol;Acc:HGNC:20626]	8
ENSG00000274997	4,458356	0,952145	0,010012	0,038518	2,894619	protein_coding	H2AC12	H2A clustered histone 12 [Source:HGNC Symbol;Acc:HGNC:13671]	6
ENSG00000085377	5,35084	-0,671462	0,010015	0,038518	-2,894509	protein_coding	PREP	prolyl endopeptidase [Source:HGNC Symbol;Acc:HGNC:9358]	6
ENSG00000141753	7,90048	-1,320049	0,010032	0,038576	-2,893683	protein_coding	IGFBP4	insulin like growth factor binding protein 4 [Source:HGNC Symbol;Acc:HGNC:5473]	17
ENSG00000186265	-2,273831	2,24867	0,01004	0,038597	2,893314	protein_coding	BTLA	B and T lymphocyte associated [Source:HGNC Symbol;Acc:HGNC:21087]	3
ENSG00000132518	-0,765936	2,428154	0,010062	0,038667	2,892292	protein_coding	GUCY2D	guanylate cyclase 2D, retinal [Source:HGNC Symbol;Acc:HGNC:4689]	17
ENSG00000173933	3,739827	0,791849	0,010063	0,038667	2,892247	protein_coding	RBM4	RNA binding motif protein 4 [Source:HGNC Symbol;Acc:HGNC:9901]	11
ENSG00000165240	5,372985	-0,687556	0,010066	0,038671	-2,89209	protein_coding	ATP7A	ATPase copper transporting alpha [Source:HGNC Symbol;Acc:HGNC:869]	X
ENSG00000167281	-1,944594	2,587002	0,010069	0,038672	2,891954	protein_coding	RBFOX3	RNA binding fox-1 homolog 3 [Source:HGNC Symbol;Acc:HGNC:27097]	17
ENSG00000197565	0,095977	-2,94598	0,010076	0,038682	-2,891611	protein_coding	COL4A6	collagen type IV alpha 6 chain [Source:HGNC Symbol;Acc:HGNC:2208]	X

ENSG00000038002	3,271124	-0,944047	0,010076	0,038682	-2,891631	protein_coding	AGA	aspartylglucosaminidase [Source:HGNC Symbol;Acc:HGNC:318]	4
ENSG00000125351	5,111719	0,866111	0,010082	0,038695	2,891352	protein_coding	UPF3B	UPF3B regulator of nonsense mediated mRNA decay [Source:HGNC Symbol;Acc:HGNC:20439]	X
ENSG00000130487	0,024338	1,415219	0,01009	0,038716	2,890985	protein_coding	KLHDC7B	kelch domain containing 7B [Source:HGNC Symbol;Acc:HGNC:25145]	22
ENSG00000204815	2,044971	1,597257	0,010117	0,03881	2,889722	protein_coding	TTC25	tetratricopeptide repeat domain 25 [Source:HGNC Symbol;Acc:HGNC:25280]	17
ENSG00000137760	3,689281	-0,562194	0,010128	0,038843	-2,889212	protein_coding	ALKBH8	alkB homolog 8, tRNA methyltransferase [Source:HGNC Symbol;Acc:HGNC:25189]	11
ENSG00000172247	-1,027531	1,861963	0,01014	0,038881	2,888645	protein_coding	C1QTNF4	C1q and TNF related 4 [Source:HGNC Symbol;Acc:HGNC:14346]	11
ENSG00000179846	-0,771773	1,564686	0,010154	0,038897	2,888009	protein_coding	NKPD1	NTPase KAP family P-loop domain containing 1 [Source:HGNC Symbol;Acc:HGNC:24739]	19
ENSG00000196378	3,020332	0,813723	0,010151	0,038897	2,888124	protein_coding	ZNF34	zinc finger protein 34 [Source:HGNC Symbol;Acc:HGNC:13098]	8
ENSG00000172890	5,825266	0,487659	0,010151	0,038897	2,888119	protein_coding	NADSYN1	NAD synthetase 1 [Source:HGNC Symbol;Acc:HGNC:29832]	11
ENSG00000150593	6,633835	1,007462	0,01015	0,038897	2,8882	protein_coding	PDCD4	programmed cell death 4 [Source:HGNC Symbol;Acc:HGNC:8763]	10
ENSG00000167964	1,644483	2,487737	0,010162	0,038908	2,887635	protein_coding	RAB26	RAB26, member RAS oncogene family [Source:HGNC Symbol;Acc:HGNC:14259]	16
ENSG00000188818	2,447198	1,524642	0,010162	0,038908	2,887611	protein_coding	ZDHHC11	zinc finger DHHC-type containing 11 [Source:HGNC Symbol;Acc:HGNC:19158]	5
ENSG00000131669	4,967992	-0,86138	0,010164	0,038908	-2,887536	protein_coding	NINJ1	ninjurin 1 [Source:HGNC Symbol;Acc:HGNC:7824]	9
ENSG00000147394	2,792211	1,830471	0,010174	0,038927	2,887087	protein_coding	ZNF185	zinc finger protein 185 with LIM domain [Source:HGNC Symbol;Acc:HGNC:12976]	X
ENSG00000133943	4,984988	0,866405	0,010172	0,038927	2,88716	protein_coding	DGLUCY	D-glutamate cyclase [Source:HGNC Symbol;Acc:HGNC:20498]	14
ENSG00000130287	-0,840195	2,427308	0,010189	0,038978	2,886359	protein_coding	NCAN	neurocan [Source:HGNC Symbol;Acc:HGNC:2465]	19
ENSG00000115165	2,061413	2,171684	0,010199	0,039007	2,885898	protein_coding	CYTIP	cytohesin 1 interacting protein [Source:HGNC Symbol;Acc:HGNC:9506]	2
ENSG00000022840	7,18596	-0,390293	0,010217	0,039067	-2,885071	protein_coding	RNF10	ring finger protein 10 [Source:HGNC Symbol;Acc:HGNC:10055]	12
ENSG00000140368	1,450804	2,71349	0,010223	0,039077	2,884831	protein_coding	PSTPIP1	proline-serine-threonine phosphatase interacting protein 1 [Source:HGNC Symbol;Acc:HGNC:9580]	15
ENSG00000146828	4,595427	0,673495	0,010226	0,03908	2,884682	protein_coding	SLC12A9	solute carrier family 12 member 9 [Source:HGNC Symbol;Acc:HGNC:17435]	7
ENSG00000182389	0,99633	1,809588	0,010239	0,039121	2,884078	protein_coding	CACNB4	calcium voltage-gated channel auxiliary subunit beta 4 [Source:HGNC Symbol;Acc:HGNC:1404]	2
ENSG00000171587	1,445355	3,469234	0,010244	0,039132	2,883838	protein_coding	DSCAM	DS cell adhesion molecule [Source:HGNC Symbol;Acc:HGNC:3039]	21
ENSG00000090432	4,684364	-0,594794	0,010255	0,039155	-2,88334	protein_coding	MUL1	mitochondrial E3 ubiquitin protein ligase 1 [Source:HGNC Symbol;Acc:HGNC:25762]	1
ENSG00000152409	6,140367	0,638422	0,010253	0,039155	2,883423	protein_coding	JMY	junction mediating and regulatory protein, p53 cofactor [Source:HGNC Symbol;Acc:HGNC:28916]	5
ENSG00000164889	7,126288	-0,465859	0,010261	0,039168	-2,883075	protein_coding	SLC4A2	solute carrier family 4 member 2 [Source:HGNC Symbol;Acc:HGNC:11028]	7
ENSG00000119121	-0,322549	2,06318	0,010269	0,039175	2,882688	protein_coding	TRPM6	transient receptor potential cation channel subfamily M member 6 [Source:HGNC Symbol;Acc:HGNC:17995]	9
ENSG00000081870	3,787157	-0,510771	0,01027	0,039175	-2,882658	protein_coding	HSPB11	heat shock protein family B (small) member 11 [Source:HGNC Symbol;Acc:HGNC:25019]	1
ENSG00000106123	4,24513	-2,196021	0,010268	0,039175	-2,882738	protein_coding	EPHB6	EPH receptor B6 [Source:HGNC Symbol;Acc:HGNC:3396]	7
ENSG00000204248	1,651421	1,316817	0,010278	0,039196	2,882301	protein_coding	COL11A2	collagen type XI alpha 2 chain [Source:HGNC Symbol;Acc:HGNC:2187]	6
ENSG00000038210	4,62131	-0,543439	0,010286	0,039218	-2,881926	protein_coding	PI4K2B	phosphatidylinositol 4-kinase type 2 beta [Source:HGNC Symbol;Acc:HGNC:18215]	4
ENSG00000104381	5,375166	1,152375	0,010289	0,039222	2,881767	protein_coding	GDAP1	ganglioside induced differentiation associated protein 1 [Source:HGNC Symbol;Acc:HGNC:15968]	8
ENSG00000136770	5,719175	-0,532158	0,010296	0,039233	-2,881459	protein_coding	DNAJC1	DnaJ heat shock protein family (Hsp40) member C1 [Source:HGNC Symbol;Acc:HGNC:20090]	10
ENSG00000075413	7,011556	0,435459	0,010297	0,039233	2,881407	protein_coding	MARK3	microtubule affinity regulating kinase 3 [Source:HGNC Symbol;Acc:HGNC:6897]	14
ENSG00000159588	0,759648	1,100898	0,010309	0,03927	2,880853	protein_coding	CCDC17	coiled-coil domain containing 17 [Source:HGNC Symbol;Acc:HGNC:26574]	1
ENSG00000174099	5,072639	-1,230411	0,01033	0,03933	-2,879913	protein_coding	MSRB3	methionine sulfoxide reductase B3 [Source:HGNC Symbol;Acc:HGNC:27375]	12
ENSG00000137497	8,954132	0,521748	0,010328	0,03933	2,880009	protein_coding	NUMA1	nuclear mitotic apparatus protein 1 [Source:HGNC Symbol;Acc:HGNC:8059]	11
ENSG00000160883	2,161038	1,549591	0,010348	0,039382	2,8791	protein_coding	HK3	hexokinase 3 [Source:HGNC Symbol;Acc:HGNC:4925]	5
ENSG00000004660	3,745535	1,356389	0,010348	0,039382	2,879069	protein_coding	CAMKK1	calcium/calmodulin dependent protein kinase kinase 1 [Source:HGNC Symbol;Acc:HGNC:1469]	17
ENSG00000121064	5,59292	-0,837741	0,010366	0,03944	-2,878267	protein_coding	SCPEP1	serine carboxypeptidase 1 [Source:HGNC Symbol;Acc:HGNC:29507]	17
ENSG00000140287	-3,121509	2,93543	0,010386	0,039506	2,87737	protein_coding	HDC	histidine decarboxylase [Source:HGNC Symbol;Acc:HGNC:4855]	15
ENSG00000178917	3,475941	0,748783	0,01039	0,039513	2,87718	protein_coding	ZNF852	zinc finger protein 852 [Source:HGNC Symbol;Acc:HGNC:27713]	3
ENSG00000286264	-0,800574	1,300655	0,010395	0,039523	2,876944	protein_coding	AP001453.	Uncharacterized protein LOC114841035 [Source:NCBI gene (formerly Entrezgene);Acc:114841035]	11
ENSG00000185158	3,809826	0,713234	0,010402	0,039539	2,876648	protein_coding	LRRC37B	leucine rich repeat containing 37B [Source:HGNC Symbol;Acc:HGNC:29070]	17
ENSG00000179431	3,392954	-1,236694	0,010416	0,039583	-2,876014	protein_coding	FJX1	four-jointed box kinase 1 [Source:HGNC Symbol;Acc:HGNC:17166]	11
ENSG00000061987	6,572217	0,384234	0,010422	0,039598	2,875727	protein_coding	MON2	MON2 homolog, regulator of endosome-to-Golgi trafficking [Source:HGNC Symbol;Acc:HGNC:29177]	12
ENSG00000138617	3,164358	0,609917	0,010436	0,039641	2,875095	protein_coding	PARP16	poly(ADP-ribose) polymerase family member 16 [Source:HGNC Symbol;Acc:HGNC:26040]	15
ENSG00000198246	3,159264	-0,679734	0,010439	0,039644	-2,87495	protein_coding	SLC29A3	solute carrier family 29 member 3 [Source:HGNC Symbol;Acc:HGNC:23096]	10
ENSG00000147570	-2,153553	2,224527	0,010462	0,039721	2,873929	protein_coding	DNAJC5B	DnaJ heat shock protein family (Hsp40) member C5 beta [Source:HGNC Symbol;Acc:HGNC:24138]	8
ENSG00000214290	1,627955	1,940232	0,010482	0,039788	2,873028	protein_coding	COLCA2	colorectal cancer associated 2 [Source:HGNC Symbol;Acc:HGNC:26978]	11
ENSG00000096872	4,404163	0,530132	0,0105	0,039848	2,872206	protein_coding	IFT74	intraflagellar transport 74 [Source:HGNC Symbol;Acc:HGNC:21424]	9
ENSG00000257950	-0,403843	-1,293207	0,010516	0,039896	-2,871515	protein_coding	P2RX5-TAX	P2RX5-TAX1BP3 readthrough (NMD candidate) [Source:HGNC Symbol;Acc:HGNC:49191]	17

ENSG00000168564	3,839929	-0,487405	0,010519	0,039896	-2,871369	protein_coding	CDKN2AIP	CDKN2A interacting protein [Source:HGNC Symbol;Acc:HGNC:24325]	4
ENSG00000108828	7,244622	-0,678218	0,01052	0,039896	-2,871304	protein_coding	VAT1	vesicle amine transport 1 [Source:HGNC Symbol;Acc:HGNC:16919]	17
ENSG00000176895	-3,001414	2,789437	0,010539	0,03996	2,870446	protein_coding	OR51A7	olfactory receptor family 51 subfamily A member 7 [Source:HGNC Symbol;Acc:HGNC:15188]	11
ENSG00000221843	-1,715068	1,687136	0,010548	0,039984	2,870052	protein_coding	C2orf16	chromosome 2 open reading frame 16 [Source:HGNC Symbol;Acc:HGNC:25275]	2
ENSG00000168389	2,4233	-1,057772	0,010559	0,040011	-2,869581	protein_coding	MFSD2A	major facilitator superfamily domain containing 2A [Source:HGNC Symbol;Acc:HGNC:25897]	1
ENSG00000178741	5,806502	-0,641931	0,01056	0,040011	-2,869511	protein_coding	COX5A	cytochrome c oxidase subunit 5A [Source:HGNC Symbol;Acc:HGNC:2267]	15
ENSG00000112378	5,835524	-1,179458	0,010585	0,040094	-2,868428	protein_coding	PERP	p53 apoptosis effector related to PMP22 [Source:HGNC Symbol;Acc:HGNC:17637]	6
ENSG00000205403	-0,056671	2,412075	0,01059	0,040103	2,868207	protein_coding	CFI	complement factor I [Source:HGNC Symbol;Acc:HGNC:5394]	4
ENSG00000081148	0,747427	1,385377	0,010606	0,040157	2,867464	protein_coding	IMPG2	interphotoreceptor matrix proteoglycan 2 [Source:HGNC Symbol;Acc:HGNC:18362]	3
ENSG00000179859	2,224386	1,199283	0,010616	0,040178	2,86704	protein_coding	RNF227	ring finger protein 227 [Source:HGNC Symbol;Acc:HGNC:27571]	17
ENSG00000100201	9,124526	0,55634	0,010617	0,040178	2,866993	protein_coding	DDX17	DEAD-box helicase 17 [Source:HGNC Symbol;Acc:HGNC:2740]	22
ENSG00000102057	2,439117	1,100617	0,010625	0,040201	2,866615	protein_coding	KCND1	potassium voltage-gated channel subfamily D member 1 [Source:HGNC Symbol;Acc:HGNC:6237]	X
ENSG00000183386	3,54532	-1,050442	0,01063	0,040209	-2,866413	protein_coding	FHL3	four and a half LIM domains 3 [Source:HGNC Symbol;Acc:HGNC:3704]	1
ENSG00000186952	0,814186	1,655333	0,010636	0,040221	2,866168	protein_coding	TMEM232	transmembrane protein 232 [Source:HGNC Symbol;Acc:HGNC:37270]	5
ENSG00000280670	1,144322	1,120973	0,01064	0,040227	2,865981	protein_coding	CCDC163	coiled-coil domain containing 163 [Source:HGNC Symbol;Acc:HGNC:27003]	1
ENSG00000164236	1,647162	1,381156	0,010648	0,04025	2,865602	protein_coding	ANKRD33B	ankyrin repeat domain 33B [Source:HGNC Symbol;Acc:HGNC:35240]	5
ENSG00000130332	4,293987	0,52385	0,010654	0,040263	2,86534	protein_coding	LSM7	LSM7 homolog, U6 small nuclear RNA and mRNA degradation associated [Source:HGNC Symbol;Acc:HGNC:20470]	19
ENSG00000137710	7,076443	-0,566851	0,010667	0,040303	-2,86476	protein_coding	RDX	radixin [Source:HGNC Symbol;Acc:HGNC:9944]	11
ENSG00000215375	2,987155	0,640992	0,010674	0,040311	2,864449	protein_coding	MYL5	myosin light chain 5 [Source:HGNC Symbol;Acc:HGNC:7586]	4
ENSG00000104518	4,774699	0,993257	0,010674	0,040311	2,864474	protein_coding	GSDMD	gasdermin D [Source:HGNC Symbol;Acc:HGNC:25697]	8
ENSG00000101096	4,962579	1,295215	0,010684	0,040338	2,864023	protein_coding	NFATC2	nuclear factor of activated T cells 2 [Source:HGNC Symbol;Acc:HGNC:7776]	20
ENSG00000117122	4,729078	-1,464749	0,010688	0,040345	-2,863837	protein_coding	MFAP2	microfibril associated protein 2 [Source:HGNC Symbol;Acc:HGNC:7033]	1
ENSG00000043093	5,216276	-0,536427	0,010706	0,040403	-2,863042	protein_coding	DCUN1D1	defective in cullin neddylation 1 domain containing 1 [Source:HGNC Symbol;Acc:HGNC:18184]	3
ENSG00000054803	-1,142874	3,527805	0,010717	0,040424	2,862586	protein_coding	CBLN4	cerebellin 4 precursor [Source:HGNC Symbol;Acc:HGNC:16231]	20
ENSG00000109927	1,557319	1,131363	0,010714	0,040424	2,862687	protein_coding	TECTA	tectorin alpha [Source:HGNC Symbol;Acc:HGNC:11720]	11
ENSG00000122691	1,977357	-1,645015	0,010725	0,040436	-2,862228	protein_coding	TWIST1	twist family bHLH transcription factor 1 [Source:HGNC Symbol;Acc:HGNC:12428]	7
ENSG00000132286	4,697195	-0,528875	0,010723	0,040436	-2,862307	protein_coding	TIMM10B	translocase of inner mitochondrial membrane 10B [Source:HGNC Symbol;Acc:HGNC:4022]	11
ENSG00000159784	1,299382	-1,975159	0,010737	0,040472	-2,861698	protein_coding	FAM131B	family with sequence similarity 131 member B [Source:HGNC Symbol;Acc:HGNC:22202]	7
ENSG00000164463	5,410686	0,772309	0,010761	0,040555	2,860617	protein_coding	CREBRF	CREB3 regulatory factor [Source:HGNC Symbol;Acc:HGNC:24050]	5
ENSG00000111554	3,801646	0,593845	0,010769	0,040566	2,86027	protein_coding	MDM1	Mdm1 nuclear protein [Source:HGNC Symbol;Acc:HGNC:29917]	12
ENSG00000162736	6,625103	-0,56037	0,010767	0,040566	-2,860368	protein_coding	NCSTN	nicastrin [Source:HGNC Symbol;Acc:HGNC:17091]	1
ENSG00000151023	-1,199738	2,085685	0,010777	0,040587	2,859923	protein_coding	ENKUR	enkurin, TRPC channel interacting protein [Source:HGNC Symbol;Acc:HGNC:28388]	10
ENSG00000126215	3,247437	1,144395	0,010784	0,040604	2,859615	protein_coding	XRCC3	X-ray repair cross complementing 3 [Source:HGNC Symbol;Acc:HGNC:12830]	14
ENSG00000136938	7,214131	0,508873	0,010787	0,040604	2,859507	protein_coding	ANP32B	acidic nuclear phosphoprotein 32 family member B [Source:HGNC Symbol;Acc:HGNC:16677]	9
ENSG00000284691	2,668184	0,986179	0,010793	0,040616	2,859253	protein_coding	AC073111.	novel zinc finger protein	7
ENSG00000139865	1,791577	2,015728	0,0108	0,040634	2,858939	protein_coding	TTC6	tetratricopeptide repeat domain 6 [Source:HGNC Symbol;Acc:HGNC:19739]	14
ENSG00000144827	4,693988	-0,553837	0,010808	0,040653	-2,8586	protein_coding	ABHD10	abhydrolase domain containing 10, depalmitoylase [Source:HGNC Symbol;Acc:HGNC:25656]	3
ENSG00000249158	1,542451	2,623157	0,010822	0,040699	2,857963	protein_coding	PCDHA11	protocadherin alpha 11 [Source:HGNC Symbol;Acc:HGNC:8665]	5
ENSG00000132773	3,122116	-0,672225	0,010825	0,040699	-2,857854	protein_coding	TOE1	target of EGR1, exonuclease [Source:HGNC Symbol;Acc:HGNC:15954]	1
ENSG00000065427	6,332962	-0,440841	0,010828	0,0407	-2,857729	protein_coding	KARS1	lysyl-tRNA synthetase 1 [Source:HGNC Symbol;Acc:HGNC:6215]	16
ENSG00000206190	4,195318	-0,891875	0,010837	0,040725	-2,857328	protein_coding	ATP10A	ATPase phospholipid transporting 10A (putative) [Source:HGNC Symbol;Acc:HGNC:13542]	15
ENSG00000162976	2,829648	-1,173139	0,010847	0,040755	-2,856878	protein_coding	SLC66A3	solute carrier family 66 member 3 [Source:HGNC Symbol;Acc:HGNC:28503]	2
ENSG00000140682	4,860956	-1,18688	0,010859	0,04078	-2,856374	protein_coding	TGFB1I1	transforming growth factor beta 1 induced transcript 1 [Source:HGNC Symbol;Acc:HGNC:11767]	16
ENSG00000115234	5,900208	-0,489173	0,010858	0,04078	-2,856416	protein_coding	SNX17	sorting nexin 17 [Source:HGNC Symbol;Acc:HGNC:14979]	2
ENSG00000215712	3,367248	-0,455971	0,010876	0,040835	-2,855623	protein_coding	TMEM242	transmembrane protein 242 [Source:HGNC Symbol;Acc:HGNC:17206]	6
ENSG00000114805	1,404954	2,000682	0,010885	0,040848	2,85521	protein_coding	PLCH1	phospholipase C eta 1 [Source:HGNC Symbol;Acc:HGNC:29185]	3
ENSG00000169862	4,578826	2,648553	0,010886	0,040848	2,855176	protein_coding	CTNND2	catenin delta 2 [Source:HGNC Symbol;Acc:HGNC:2516]	5
ENSG00000060982	5,948469	-1,549509	0,010887	0,040848	-2,855148	protein_coding	BCAT1	branched chain amino acid transaminase 1 [Source:HGNC Symbol;Acc:HGNC:976]	12
ENSG00000118514	-0,188143	2,005419	0,01091	0,040924	2,854157	protein_coding	ALDH8A1	aldehyde dehydrogenase 8 family member A1 [Source:HGNC Symbol;Acc:HGNC:15471]	6
ENSG00000137486	5,067056	1,058279	0,010918	0,040946	2,853793	protein_coding	ARRB1	arrestin beta 1 [Source:HGNC Symbol;Acc:HGNC:711]	11
ENSG00000128245	6,851558	-0,375267	0,010948	0,041049	-2,8525	protein_coding	YWHAH	tyrosine 3-monooxygenase/tryptophan 5-monooxygenase activation protein eta [Source:HGNC Symbol;Acc:HGNC:1	22

ENSG00000044524	4,483396	-1,819482	0,010953	0,041056	-2,852306	protein_coding	EPHA3	EPH receptor A3 [Source:HGNC Symbol;Acc:HGNC:3387]	3
ENSG00000144233	5,022129	-0,469838	0,010955	0,041056	-2,852208	protein_coding	AMMECR1	AMMECR1 like [Source:HGNC Symbol;Acc:HGNC:28658]	2
ENSG00000169918	3,212827	0,947935	0,01096	0,041064	2,851999	protein_coding	OTUD7A	OTU deubiquitinase 7A [Source:HGNC Symbol;Acc:HGNC:20718]	15
ENSG00000015475	4,273135	-0,515659	0,010963	0,041067	-2,851865	protein_coding	BID	BH3 interacting domain death agonist [Source:HGNC Symbol;Acc:HGNC:1050]	22
ENSG00000067842	-0,094882	2,823347	0,01097	0,041085	2,851545	protein_coding	ATP2B3	ATPase plasma membrane Ca2+ transporting 3 [Source:HGNC Symbol;Acc:HGNC:816]	X
ENSG00000179593	0,021495	2,316252	0,010974	0,04109	2,85138	protein_coding	ALOX15B	arachidonate 15-lipoxygenase type B [Source:HGNC Symbol;Acc:HGNC:434]	17
ENSG00000150637	1,207299	1,558862	0,010979	0,041097	2,851187	protein_coding	CD226	CD226 molecule [Source:HGNC Symbol;Acc:HGNC:16961]	18
ENSG00000140948	6,488884	-0,409795	0,010982	0,041101	-2,851032	protein_coding	ZCCHC14	zinc finger CCHC-type containing 14 [Source:HGNC Symbol;Acc:HGNC:24134]	16
ENSG00000104903	1,547981	1,287408	0,010987	0,041111	2,850814	protein_coding	LYL1	LYL1 basic helix-loop-helix family member [Source:HGNC Symbol;Acc:HGNC:6734]	19
ENSG00000172243	1,677011	1,79933	0,010993	0,041121	2,850588	protein_coding	CLEC7A	C-type lectin domain containing 7A [Source:HGNC Symbol;Acc:HGNC:14558]	12
ENSG00000135111	4,690447	-1,547957	0,011007	0,041166	-2,849962	protein_coding	TBX3	T-box transcription factor 3 [Source:HGNC Symbol;Acc:HGNC:11602]	12
ENSG00000112164	-0,753735	2,760416	0,011029	0,041239	2,849015	protein_coding	GLP1R	glucagon like peptide 1 receptor [Source:HGNC Symbol;Acc:HGNC:4324]	6
ENSG00000215440	2,932595	0,831999	0,01105	0,041307	2,848133	protein_coding	NPEPL1	aminopeptidase like 1 [Source:HGNC Symbol;Acc:HGNC:16244]	20
ENSG00000153558	2,652089	0,91647	0,011056	0,04132	2,847875	protein_coding	FBXL2	F-box and leucine rich repeat protein 2 [Source:HGNC Symbol;Acc:HGNC:13598]	3
ENSG00000119943	1,762091	1,234456	0,011064	0,041331	2,847532	protein_coding	PYROXD2	pyridine nucleotide-disulphide oxidoreductase domain 2 [Source:HGNC Symbol;Acc:HGNC:23517]	10
ENSG00000138363	5,791073	-0,648686	0,011064	0,041331	-2,847536	protein_coding	ATIC	5-aminoimidazole-4-carboxamide ribonucleotide formyltransferase/IMP cyclohydrolase [Source:HGNC Symbol;Acc:I	2
ENSG00000161653	0,267781	1,260451	0,011077	0,041345	2,846966	protein_coding	NAGS	N-acetylglutamate synthase [Source:HGNC Symbol;Acc:HGNC:17996]	17
ENSG00000175182	3,881139	1,384563	0,011078	0,041345	2,846933	protein_coding	FAM131A	family with sequence similarity 131 member A [Source:HGNC Symbol;Acc:HGNC:28308]	3
ENSG00000169118	5,09582	-0,452684	0,011076	0,041345	-2,847007	protein_coding	CSNK1G1	casein kinase 1 gamma 1 [Source:HGNC Symbol;Acc:HGNC:2454]	15
ENSG00000179632	6,135034	-0,46723	0,011075	0,041345	-2,84706	protein_coding	MAF1	MAF1 homolog, negative regulator of RNA polymerase III [Source:HGNC Symbol;Acc:HGNC:24966]	8
ENSG00000158201	5,220285	-1,11101	0,011097	0,041406	-2,846132	protein_coding	ABHD3	abhydrolase domain containing 3, phospholipase [Source:HGNC Symbol;Acc:HGNC:18718]	18
ENSG00000091181	-1,925799	1,727236	0,011104	0,041425	2,84581	protein_coding	IL5RA	interleukin 5 receptor subunit alpha [Source:HGNC Symbol;Acc:HGNC:6017]	3
ENSG00000108688	-1,790515	-1,943681	0,011112	0,041445	-2,845471	protein_coding	CCL7	C-C motif chemokine ligand 7 [Source:HGNC Symbol;Acc:HGNC:10634]	17
ENSG00000165973	1,067503	3,366233	0,01112	0,041465	2,845133	protein_coding	NELL1	neural EGFL like 1 [Source:HGNC Symbol;Acc:HGNC:7750]	11
ENSG00000101868	5,362383	0,797548	0,011128	0,041476	2,844817	protein_coding	POLA1	DNA polymerase alpha 1, catalytic subunit [Source:HGNC Symbol;Acc:HGNC:9173]	X
ENSG00000010292	6,635688	0,413397	0,011128	0,041476	2,844787	protein_coding	NCAPD2	non-SMC condensin I complex subunit D2 [Source:HGNC Symbol;Acc:HGNC:24305]	12
ENSG00000154429	4,410423	0,625481	0,011135	0,041492	2,844507	protein_coding	CCSAP	centriole, cilia and spindle associated protein [Source:HGNC Symbol;Acc:HGNC:29578]	1
ENSG00000162755	0,858928	1,347116	0,011155	0,041544	2,843648	protein_coding	KLHDC9	kelch domain containing 9 [Source:HGNC Symbol;Acc:HGNC:28489]	1
ENSG00000179598	2,019659	1,734741	0,011156	0,041544	2,843625	protein_coding	PLD6	phospholipase D family member 6 [Source:HGNC Symbol;Acc:HGNC:30447]	17
ENSG00000175334	4,501727	-0,50381	0,011157	0,041544	-2,843582	protein_coding	BANF1	BAF nuclear assembly factor 1 [Source:HGNC Symbol;Acc:HGNC:17397]	11
ENSG00000179041	4,30759	-0,527727	0,011167	0,041563	-2,843149	protein_coding	RRS1	ribosome biogenesis regulator 1 homolog [Source:HGNC Symbol;Acc:HGNC:17083]	8
ENSG00000149485	7,020727	-0,755731	0,011166	0,041563	-2,843213	protein_coding	FADS1	fatty acid desaturase 1 [Source:HGNC Symbol;Acc:HGNC:3574]	11
ENSG00000165966	-1,226304	2,513974	0,01117	0,041564	2,843035	protein_coding	PDZRN4	PDZ domain containing ring finger 4 [Source:HGNC Symbol;Acc:HGNC:30552]	12
ENSG00000102984	3,261964	0,69708	0,011179	0,041587	2,842664	protein_coding	ZNF821	zinc finger protein 821 [Source:HGNC Symbol;Acc:HGNC:28043]	16
ENSG00000121101	-0,672767	2,255823	0,011188	0,041614	2,842245	protein_coding	TEX14	testis expressed 14, intercellular bridge forming factor [Source:HGNC Symbol;Acc:HGNC:11737]	17
ENSG00000103042	4,748918	-0,547689	0,011191	0,041615	-2,842128	protein_coding	SLC38A7	solute carrier family 38 member 7 [Source:HGNC Symbol;Acc:HGNC:25582]	16
ENSG00000105642	0,097589	2,693433	0,011205	0,041655	2,841551	protein_coding	KCNN1	potassium calcium-activated channel subfamily N member 1 [Source:HGNC Symbol;Acc:HGNC:6290]	19
ENSG00000102096	3,481675	0,946864	0,01121	0,041655	2,841354	protein_coding	PIM2	Pim-2 proto-oncogene, serine/threonine kinase [Source:HGNC Symbol;Acc:HGNC:8987]	X
ENSG00000132024	6,383114	0,752679	0,011208	0,041655	2,841423	protein_coding	CC2D1A	coiled-coil and C2 domain containing 1A [Source:HGNC Symbol;Acc:HGNC:30237]	19
ENSG00000112799	-0,564949	2,51638	0,011218	0,041666	2,841012	protein_coding	LY86	lymphocyte antigen 86 [Source:HGNC Symbol;Acc:HGNC:16837]	6
ENSG00000104976	3,47116	-0,583941	0,011216	0,041666	-2,841086	protein_coding	SNAPC2	small nuclear RNA activating complex polypeptide 2 [Source:HGNC Symbol;Acc:HGNC:11135]	19
ENSG00000150636	5,321523	-1,385652	0,011221	0,04167	-2,840863	protein_coding	CCDC102B	coiled-coil domain containing 102B [Source:HGNC Symbol;Acc:HGNC:26295]	18
ENSG00000160179	4,793326	1,256584	0,011228	0,041675	2,840583	protein_coding	ABCG1	ATP binding cassette subfamily G member 1 [Source:HGNC Symbol;Acc:HGNC:73]	21
ENSG00000101773	5,923352	-0,652344	0,011227	0,041675	-2,840639	protein_coding	RBBP8	RB binding protein 8, endonuclease [Source:HGNC Symbol;Acc:HGNC:9891]	18
ENSG00000242852	-0,669819	1,362958	0,011238	0,041687	2,840179	protein_coding	ZNF709	zinc finger protein 709 [Source:HGNC Symbol;Acc:HGNC:20629]	19
ENSG00000198216	0,803798	3,227423	0,011239	0,041687	2,840127	protein_coding	CACNA1E	calcium voltage-gated channel subunit alpha1 E [Source:HGNC Symbol;Acc:HGNC:1392]	1
ENSG00000125450	5,024007	0,56164	0,011235	0,041687	2,840303	protein_coding	NUP85	nucleoporin 85 [Source:HGNC Symbol;Acc:HGNC:8734]	17
ENSG00000164142	4,231001	1,639281	0,011244	0,041697	2,839905	protein_coding	FAM160A1	family with sequence similarity 160 member A1 [Source:HGNC Symbol;Acc:HGNC:34237]	4
ENSG00000039537	0,285814	5,485144	0,011251	0,041713	2,839614	protein_coding	C6	complement C6 [Source:HGNC Symbol;Acc:HGNC:1339]	5
ENSG00000148488	-1,117244	1,934046	0,011255	0,041716	2,839444	protein_coding	ST8SIA6	ST8 alpha-N-acetyl-neuraminide alpha-2,8-sialyltransferase 6 [Source:HGNC Symbol;Acc:HGNC:23317]	10
ENSG00000124120	4,581371	-0,522283	0,011257	0,041716	-2,839369	protein_coding	TTPAL	alpha tocopherol transfer protein like [Source:HGNC Symbol;Acc:HGNC:16114]	20

ENSG00000169093	4,205577	0,779019	0,011275	0,041773	2,838613	protein_coding	ASMTL	acetylserotonin O-methyltransferase like [Source:HGNC Symbol;Acc:HGNC:751]	X
ENSG00000142235	1,927343	2,976655	0,011285	0,041801	2,838188	protein_coding	LMTK3	lemur tyrosine kinase 3 [Source:HGNC Symbol;Acc:HGNC:19295]	19
ENSG00000196968	5,405741	-0,480995	0,011289	0,041806	-2,838029	protein_coding	FUT11	fucosyltransferase 11 [Source:HGNC Symbol;Acc:HGNC:19233]	10
ENSG00000197140	1,265217	1,076764	0,011298	0,041822	2,837628	protein_coding	ADAM32	ADAM metallopeptidase domain 32 [Source:HGNC Symbol;Acc:HGNC:15479]	8
ENSG00000082397	5,842022	1,545034	0,011297	0,041822	2,837695	protein_coding	EPB41L3	erythrocyte membrane protein band 4.1 like 3 [Source:HGNC Symbol;Acc:HGNC:3380]	18
ENSG00000136828	4,969856	1,2888	0,01132	0,041892	2,836725	protein_coding	RALGPS1	Ral GEF with PH domain and SH3 binding motif 1 [Source:HGNC Symbol;Acc:HGNC:16851]	9
ENSG00000138698	5,082796	-0,522925	0,011324	0,041896	-2,836575	protein_coding	RAP1GDS1	Rap1 GTPase-GDP dissociation stimulator 1 [Source:HGNC Symbol;Acc:HGNC:9859]	4
ENSG00000151612	5,503309	0,544189	0,011329	0,041907	2,836347	protein_coding	ZNF827	zinc finger protein 827 [Source:HGNC Symbol;Acc:HGNC:27193]	4
ENSG00000197753	1,519692	1,92928	0,011334	0,041916	2,836133	protein_coding	LHFPL5	LHFPL tetraspan subfamily member 5 [Source:HGNC Symbol;Acc:HGNC:21253]	6
ENSG00000173914	3,701393	-0,615917	0,011339	0,041926	-2,835919	protein_coding	RBM4B	RNA binding motif protein 4B [Source:HGNC Symbol;Acc:HGNC:28842]	11
ENSG00000150457	4,980079	-1,326706	0,011348	0,041949	-2,835549	protein_coding	LATS2	large tumor suppressor kinase 2 [Source:HGNC Symbol;Acc:HGNC:6515]	13
ENSG00000144199	1,981356	1,330915	0,011369	0,042009	2,834664	protein_coding	FAHD2B	fumarylacetoacetate hydrolase domain containing 2B [Source:HGNC Symbol;Acc:HGNC:25318]	2
ENSG00000035862	7,677132	-1,326764	0,011369	0,042009	-2,834691	protein_coding	TIMP2	TIMP metallopeptidase inhibitor 2 [Source:HGNC Symbol;Acc:HGNC:11821]	17
ENSG00000102967	2,726978	0,859587	0,011386	0,042059	2,833986	protein_coding	DHODH	dihydroorotate dehydrogenase (quinone) [Source:HGNC Symbol;Acc:HGNC:2867]	16
ENSG00000131269	4,821815	0,491224	0,011392	0,042074	2,833713	protein_coding	ABCB7	ATP binding cassette subfamily B member 7 [Source:HGNC Symbol;Acc:HGNC:48]	X
ENSG00000162241	2,650309	0,997529	0,011402	0,0421	2,833314	protein_coding	SLC25A45	solute carrier family 25 member 45 [Source:HGNC Symbol;Acc:HGNC:27442]	11
ENSG00000121933	-0,781919	2,094748	0,011406	0,042105	2,833154	protein_coding	TMIGD3	transmembrane and immunoglobulin domain containing 3 [Source:HGNC Symbol;Acc:HGNC:51375]	1
ENSG00000167191	3,204879	1,424067	0,011416	0,042131	2,832749	protein_coding	GPRC5B	G protein-coupled receptor class C group 5 member B [Source:HGNC Symbol;Acc:HGNC:13308]	16
ENSG00000187391	4,563448	1,337838	0,01146	0,042286	2,83091	protein_coding	MAGI2	membrane associated guanylate kinase, WW and PDZ domain containing 2 [Source:HGNC Symbol;Acc:HGNC:18957]	7
ENSG00000100749	4,752311	0,866852	0,011472	0,042319	2,830427	protein_coding	VRK1	VRK serine/threonine kinase 1 [Source:HGNC Symbol;Acc:HGNC:12718]	14
ENSG00000163644	4,722619	1,379168	0,011495	0,042389	2,829461	protein_coding	PPM1K	protein phosphatase, Mg2+/Mn2+ dependent 1K [Source:HGNC Symbol;Acc:HGNC:25415]	4
ENSG00000113387	6,58643	-0,452311	0,011496	0,042389	-2,829439	protein_coding	SUB1	SUB1 regulator of transcription [Source:HGNC Symbol;Acc:HGNC:19985]	5
ENSG00000065060	5,778941	0,455125	0,011518	0,042459	2,828543	protein_coding	UHRF1BP1	UHRF1 binding protein 1 [Source:HGNC Symbol;Acc:HGNC:21216]	6
ENSG00000134684	6,405518	-0,593287	0,011537	0,04252	-2,827767	protein_coding	YARS1	tyrosyl-tRNA synthetase 1 [Source:HGNC Symbol;Acc:HGNC:12840]	1
ENSG00000177483	0,598612	0,993239	0,011544	0,042537	2,827465	protein_coding	RBM44	RNA binding motif protein 44 [Source:HGNC Symbol;Acc:HGNC:24756]	2
ENSG00000110876	1,913631	1,595716	0,011569	0,042621	2,826427	protein_coding	SELPLG	selectin P ligand [Source:HGNC Symbol;Acc:HGNC:10722]	12
ENSG00000175938	2,223808	-0,715579	0,011577	0,042638	-2,826126	protein_coding	ORAI3	ORAI calcium release-activated calcium modulator 3 [Source:HGNC Symbol;Acc:HGNC:28185]	16
ENSG00000100519	6,233155	-0,468428	0,011586	0,042663	-2,825744	protein_coding	PSMC6	proteasome 26S subunit, ATPase 6 [Source:HGNC Symbol;Acc:HGNC:9553]	14
ENSG00000139641	7,481429	-0,579473	0,01161	0,042743	-2,82475	protein_coding	ESYT1	extended synaptotagmin 1 [Source:HGNC Symbol;Acc:HGNC:29534]	12
ENSG00000166974	6,828917	-0,578146	0,011616	0,042754	-2,824523	protein_coding	MAPRE2	microtubule associated protein RP/EB family member 2 [Source:HGNC Symbol;Acc:HGNC:6891]	18
ENSG00000078589	-2,611547	2,959737	0,011628	0,042782	2,824015	protein_coding	P2RY10	P2Y receptor family member 10 [Source:HGNC Symbol;Acc:HGNC:19906]	X
ENSG00000143641	7,350567	-0,918762	0,011629	0,042782	-2,824002	protein_coding	GALNT2	polypeptide N-acetylgalactosaminyltransferase 2 [Source:HGNC Symbol;Acc:HGNC:4124]	1
ENSG00000152926	4,123759	0,832145	0,011632	0,042786	2,823853	protein_coding	ZNF117	zinc finger protein 117 [Source:HGNC Symbol;Acc:HGNC:12897]	7
ENSG00000125912	6,356455	-0,503066	0,011651	0,042843	-2,823109	protein_coding	NCLN	nicalin [Source:HGNC Symbol;Acc:HGNC:26923]	19
ENSG00000101400	4,330289	0,935158	0,011656	0,042852	2,822903	protein_coding	SNTA1	syntrophin alpha 1 [Source:HGNC Symbol;Acc:HGNC:11167]	20
ENSG00000139192	3,941252	1,196567	0,011661	0,042862	2,822685	protein_coding	TAPBPL	TAP binding protein like [Source:HGNC Symbol;Acc:HGNC:30683]	12
ENSG00000284686	-1,532316	1,763755	0,011678	0,042901	2,822012	protein_coding	AC119674.	novel transcript	1
ENSG00000137558	1,009692	1,952542	0,011679	0,042901	2,821977	protein_coding	PI15	peptidase inhibitor 15 [Source:HGNC Symbol;Acc:HGNC:8946]	8
ENSG00000145022	4,674657	-0,550522	0,01168	0,042901	-2,821938	protein_coding	TCTA	T cell leukemia translocation altered [Source:HGNC Symbol;Acc:HGNC:11692]	3
ENSG00000070886	-1,652058	2,864458	0,011688	0,042921	2,821614	protein_coding	EPHA8	EPH receptor A8 [Source:HGNC Symbol;Acc:HGNC:3391]	1
ENSG00000198695	8,335471	-0,71784	0,011703	0,042967	-2,820993	protein_coding	MT-ND6	mitochondrially encoded NADH:ubiquinone oxidoreductase core subunit 6 [Source:HGNC Symbol;Acc:HGNC:7462]	MT
ENSG00000103381	4,633382	-0,510802	0,01171	0,042983	-2,820715	protein_coding	CPPED1	calcineurin like phosphoesterase domain containing 1 [Source:HGNC Symbol;Acc:HGNC:25632]	16
ENSG00000129103	6,567839	-0,533867	0,011716	0,042995	-2,820471	protein_coding	SUMF2	sulfatase modifying factor 2 [Source:HGNC Symbol;Acc:HGNC:20415]	7
ENSG00000149182	5,852831	0,482498	0,011734	0,043051	2,819752	protein_coding	ARFGAP2	ADP ribosylation factor GTPase activating protein 2 [Source:HGNC Symbol;Acc:HGNC:13504]	11
ENSG00000100784	3,500718	1,515261	0,011749	0,043096	2,819144	protein_coding	RPS6KA5	ribosomal protein S6 kinase A5 [Source:HGNC Symbol;Acc:HGNC:10434]	14
ENSG00000036828	-1,311129	3,798424	0,011767	0,043154	2,818402	protein_coding	CASR	calcium sensing receptor [Source:HGNC Symbol;Acc:HGNC:1514]	3
ENSG00000101782	6,348511	-0,560172	0,011774	0,04317	-2,818125	protein_coding	RIOK3	RIO kinase 3 [Source:HGNC Symbol;Acc:HGNC:11451]	18
ENSG00000241484	-0,42817	2,18595	0,011785	0,043198	2,817705	protein_coding	ARHGAP8	Rho GTPase activating protein 8 [Source:HGNC Symbol;Acc:HGNC:677]	22
ENSG00000138085	5,320394	-0,408385	0,011796	0,043232	-2,817233	protein_coding	ATRAID	all-trans retinoic acid induced differentiation factor [Source:HGNC Symbol;Acc:HGNC:24090]	2
ENSG00000224877	4,467325	-0,556377	0,011801	0,043238	-2,817058	protein_coding	NDUFAF8	NADH:ubiquinone oxidoreductase complex assembly factor 8 [Source:HGNC Symbol;Acc:HGNC:33551]	17
ENSG00000198182	3,237052	0,79874	0,011808	0,04325	2,816781	protein_coding	ZNF607	zinc finger protein 607 [Source:HGNC Symbol;Acc:HGNC:28192]	19

ENSG00000161914	3,25143	0,688654	0,011809	0,04325	2,81671	protein_coding	ZNF653	zinc finger protein 653 [Source:HGNC Symbol;Acc:HGNC:25196]	19
ENSG00000244486	3,931523	-1,177133	0,011834	0,043328	-2,815726	protein_coding	SCARF2	scavenger receptor class F member 2 [Source:HGNC Symbol;Acc:HGNC:19869]	22
ENSG00000026950	4,501465	0,697155	0,011836	0,043328	2,815651	protein_coding	BTN3A1	butyrophilin subfamily 3 member A1 [Source:HGNC Symbol;Acc:HGNC:1138]	6
ENSG00000203772	-0,587881	2,202522	0,011849	0,043346	2,815117	protein_coding	SPRN	shadow of prion protein [Source:HGNC Symbol;Acc:HGNC:16871]	10
ENSG00000136936	4,405936	0,453299	0,011857	0,043346	2,814812	protein_coding	XPA	XPA, DNA damage recognition and repair factor [Source:HGNC Symbol;Acc:HGNC:12814]	9
ENSG00000110057	5,015108	0,6238	0,011855	0,043346	2,814872	protein_coding	UNC93B1	unc-93 homolog B1, TLR signaling regulator [Source:HGNC Symbol;Acc:HGNC:13481]	11
ENSG00000126217	6,197843	1,81701	0,011844	0,043346	2,815336	protein_coding	MCF2L	MCF.2 cell line derived transforming sequence like [Source:HGNC Symbol;Acc:HGNC:14576]	13
ENSG00000198791	5,875343	-0,504981	0,011851	0,043346	-2,815044	protein_coding	CNOT7	CCR4-NOT transcription complex subunit 7 [Source:HGNC Symbol;Acc:HGNC:14101]	8
ENSG00000153827	8,442144	-0,579908	0,011852	0,043346	-2,814995	protein_coding	TRIP12	thyroid hormone receptor interactor 12 [Source:HGNC Symbol;Acc:HGNC:12306]	2
ENSG00000026025	9,942408	-1,619741	0,011866	0,04337	-2,814439	protein_coding	VIM	vimentin [Source:HGNC Symbol;Acc:HGNC:12692]	10
ENSG00000136950	5,328144	-0,548406	0,011915	0,043538	-2,81251	protein_coding	ARPC5L	actin related protein 2/3 complex subunit 5 like [Source:HGNC Symbol;Acc:HGNC:23366]	9
ENSG00000196497	0,08892	1,029322	0,011924	0,043563	2,812129	protein_coding	IPO4	importin 4 [Source:HGNC Symbol;Acc:HGNC:19426]	14
ENSG00000166669	1,194608	1,758965	0,01196	0,043655	2,810706	protein_coding	ATF7IP2	activating transcription factor 7 interacting protein 2 [Source:HGNC Symbol;Acc:HGNC:20397]	16
ENSG00000152270	5,26226	1,238976	0,011957	0,043655	2,810844	protein_coding	PDE3B	phosphodiesterase 3B [Source:HGNC Symbol;Acc:HGNC:8779]	11
ENSG00000181396	5,054474	0,455772	0,011958	0,043655	2,81078	protein_coding	OGFOD3	2-oxoglutarate and iron dependent oxygenase domain containing 3 [Source:HGNC Symbol;Acc:HGNC:26174]	17
ENSG00000165733	6,679289	-0,303792	0,011954	0,043655	-2,810937	protein_coding	BMS1	BMS1 ribosome biogenesis factor [Source:HGNC Symbol;Acc:HGNC:23505]	10
ENSG00000187808	-0,859526	1,729759	0,011978	0,04369	2,810008	protein_coding	SOWAHD	sosondowah ankyrin repeat domain family member D [Source:HGNC Symbol;Acc:HGNC:32960]	X
ENSG00000128833	5,175925	1,3932	0,011977	0,04369	2,81005	protein_coding	MYO5C	myosin VC [Source:HGNC Symbol;Acc:HGNC:7604]	15
ENSG00000109920	6,914285	0,683213	0,011978	0,04369	2,810012	protein_coding	FNBP4	formin binding protein 4 [Source:HGNC Symbol;Acc:HGNC:19752]	11
ENSG00000145604	4,913671	0,861244	0,011988	0,043717	2,809611	protein_coding	SKP2	S-phase kinase associated protein 2 [Source:HGNC Symbol;Acc:HGNC:10901]	5
ENSG00000116127	6,534575	0,721802	0,012003	0,043764	2,80899	protein_coding	ALMS1	ALMS1 centrosome and basal body associated protein [Source:HGNC Symbol;Acc:HGNC:428]	2
ENSG00000137393	3,159591	1,384624	0,012022	0,043813	2,808248	protein_coding	RNF144B	ring finger protein 144B [Source:HGNC Symbol;Acc:HGNC:21578]	6
ENSG00000138768	7,008894	-0,668944	0,01202	0,043813	-2,808351	protein_coding	USO1	USO1 vesicle transport factor [Source:HGNC Symbol;Acc:HGNC:30904]	4
ENSG00000140299	6,048324	-0,809174	0,012029	0,043817	-2,807998	protein_coding	BNIP2	BCL2 interacting protein 2 [Source:HGNC Symbol;Acc:HGNC:1083]	15
ENSG00000162434	7,752598	-0,731689	0,012027	0,043817	-2,808074	protein_coding	JAK1	Janus kinase 1 [Source:HGNC Symbol;Acc:HGNC:6190]	1
ENSG00000134242	0,707011	1,452437	0,01205	0,043883	2,807173	protein_coding	PTPN22	protein tyrosine phosphatase non-receptor type 22 [Source:HGNC Symbol;Acc:HGNC:9652]	1
ENSG00000137720	1,897486	-0,832741	0,012054	0,043889	-2,807007	protein_coding	C11orf1	chromosome 11 open reading frame 1 [Source:HGNC Symbol;Acc:HGNC:1163]	11
ENSG00000139329	6,033586	-2,024939	0,012057	0,043889	-2,8069	protein_coding	LUM	lumican [Source:HGNC Symbol;Acc:HGNC:6724]	12
ENSG00000157933	7,212522	-0,68708	0,012081	0,043966	-2,805957	protein_coding	SKI	SKI proto-oncogene [Source:HGNC Symbol;Acc:HGNC:10896]	1
ENSG00000106018	-0,220866	2,864816	0,0121	0,044026	2,805213	protein_coding	VIPR2	vasoactive intestinal peptide receptor 2 [Source:HGNC Symbol;Acc:HGNC:12695]	7
ENSG00000139651	5,390418	0,522535	0,012113	0,044066	2,80467	protein_coding	ZNF740	zinc finger protein 740 [Source:HGNC Symbol;Acc:HGNC:27465]	12
ENSG00000112787	6,93779	0,496945	0,01213	0,044116	2,804031	protein_coding	FBRSL1	fibrosin like 1 [Source:HGNC Symbol;Acc:HGNC:29308]	12
ENSG00000163884	1,976217	2,869328	0,012145	0,044152	2,803427	protein_coding	KLF15	Kruppel like factor 15 [Source:HGNC Symbol;Acc:HGNC:14536]	3
ENSG00000138658	3,723337	0,896727	0,012145	0,044152	2,803428	protein_coding	ZGRF1	zinc finger GRF-type containing 1 [Source:HGNC Symbol;Acc:HGNC:25654]	4
ENSG00000137414	5,25814	-0,575868	0,012151	0,044164	-2,803198	protein_coding	FAM8A1	family with sequence similarity 8 member A1 [Source:HGNC Symbol;Acc:HGNC:16372]	6
ENSG00000186532	4,049661	0,618673	0,012158	0,044178	2,802936	protein_coding	SMYD4	SET and MYND domain containing 4 [Source:HGNC Symbol;Acc:HGNC:21067]	17
ENSG00000149289	4,749257	-0,799053	0,012195	0,044302	-2,801509	protein_coding	ZC3H12C	zinc finger CCCH-type containing 12C [Source:HGNC Symbol;Acc:HGNC:29362]	11
ENSG00000143224	3,120409	0,876212	0,012214	0,044363	2,800744	protein_coding	PPOX	protoporphyrinogen oxidase [Source:HGNC Symbol;Acc:HGNC:9280]	1
ENSG00000010072	4,107427	-0,476515	0,012234	0,044415	-2,799982	protein_coding	SPRTN	SprT-like N-terminal domain [Source:HGNC Symbol;Acc:HGNC:25356]	1
ENSG00000188807	4,482428	0,602016	0,012232	0,044415	2,800039	protein_coding	TMEM201	transmembrane protein 201 [Source:HGNC Symbol;Acc:HGNC:33719]	1
ENSG00000197721	-3,000257	2,091874	0,012243	0,044428	2,799632	protein_coding	CR1L	complement C3b/C4b receptor 1 like [Source:HGNC Symbol;Acc:HGNC:2335]	1
ENSG00000146197	4,826715	-1,76811	0,012243	0,044428	-2,79964	protein_coding	SCUBE3	signal peptide, CUB domain and EGF like domain containing 3 [Source:HGNC Symbol;Acc:HGNC:13655]	6
ENSG00000159625	-0,432197	1,937398	0,012256	0,044465	2,799128	protein_coding	DRC7	dynein regulatory complex subunit 7 [Source:HGNC Symbol;Acc:HGNC:25289]	16
ENSG00000139644	8,557263	-0,658903	0,012282	0,044541	-2,798111	protein_coding	TMBIM6	transmembrane BAX inhibitor motif containing 6 [Source:HGNC Symbol;Acc:HGNC:11723]	12
ENSG00000167123	6,011076	-1,141814	0,012282	0,044541	-2,798108	protein_coding	CERCAM	cerebral endothelial cell adhesion molecule [Source:HGNC Symbol;Acc:HGNC:23723]	9
ENSG00000143851	-0,183909	3,085247	0,012288	0,044552	2,797882	protein_coding	PTPN7	protein tyrosine phosphatase non-receptor type 7 [Source:HGNC Symbol;Acc:HGNC:9659]	1
ENSG00000222038	-1,757338	2,485513	0,012314	0,044631	2,796908	protein_coding	POTEJ	POTE ankyrin domain family member J [Source:HGNC Symbol;Acc:HGNC:37094]	2
ENSG00000125875	6,227094	-0,434303	0,012315	0,044631	-2,796834	protein_coding	TBC1D20	TBC1 domain family member 20 [Source:HGNC Symbol;Acc:HGNC:16133]	20
ENSG00000037241	3,690845	-0,797553	0,012326	0,044652	-2,796429	protein_coding	RPL26L1	ribosomal protein L26 like 1 [Source:HGNC Symbol;Acc:HGNC:17050]	5
ENSG00000100138	5,773304	-0,368262	0,012327	0,044652	-2,796406	protein_coding	SNU13	small nuclear ribonucleoprotein 13 [Source:HGNC Symbol;Acc:HGNC:7819]	22
ENSG00000158571	-1,119675	1,596929	0,012352	0,044724	2,795421	protein_coding	PFKFB1	6-phosphofructo-2-kinase/fructose-2,6-biphosphatase 1 [Source:HGNC Symbol;Acc:HGNC:8872]	X

ENSG00000162836	3,780314	1,01453	0,012352	0,044724	2,795442	protein_coding	ACP6	acid phosphatase 6, lysophosphatidic [Source:HGNC Symbol;Acc:HGNC:29609]	1
ENSG00000151967	0,640783	1,164856	0,012361	0,044736	2,795091	protein_coding	SCHIP1	schwannomin interacting protein 1 [Source:HGNC Symbol;Acc:HGNC:15678]	3
ENSG00000184743	6,697207	-1,145108	0,012361	0,044736	-2,795089	protein_coding	ATL3	atlastin GTPase 3 [Source:HGNC Symbol;Acc:HGNC:24526]	11
ENSG00000275302	-0,813825	1,956269	0,012369	0,044754	2,794794	protein_coding	CCL4	C-C motif chemokine ligand 4 [Source:HGNC Symbol;Acc:HGNC:10630]	17
ENSG00000198917	-1,059493	1,085608	0,012384	0,044799	2,794209	protein_coding	SPOUT1	SPOUT domain containing methyltransferase 1 [Source:HGNC Symbol;Acc:HGNC:26933]	9
ENSG00000149084	5,206267	-0,994037	0,012388	0,044803	-2,794066	protein_coding	HSD17B12	hydroxysteroid 17-beta dehydrogenase 12 [Source:HGNC Symbol;Acc:HGNC:18646]	11
ENSG00000121858	3,219438	1,404995	0,012412	0,044882	2,793125	protein_coding	TNFSF10	TNF superfamily member 10 [Source:HGNC Symbol;Acc:HGNC:11925]	3
ENSG00000115677	9,849042	-0,820069	0,012416	0,044886	-2,792969	protein_coding	HDLBP	high density lipoprotein binding protein [Source:HGNC Symbol;Acc:HGNC:4857]	2
ENSG00000205045	-1,406846	1,46556	0,012441	0,044966	2,79202	protein_coding	SLFN12L	schlafen family member 12 like [Source:HGNC Symbol;Acc:HGNC:33920]	17
ENSG00000143183	6,102372	-1,147398	0,012463	0,045035	-2,791189	protein_coding	TMCO1	transmembrane and coiled-coil domains 1 [Source:HGNC Symbol;Acc:HGNC:18188]	1
ENSG00000163558	5,330697	-0,541296	0,012479	0,045081	-2,790598	protein_coding	PRKCI	protein kinase C iota [Source:HGNC Symbol;Acc:HGNC:9404]	3
ENSG00000001084	5,64986	-0,918814	0,012481	0,045081	-2,790501	protein_coding	GCLC	glutamate-cysteine ligase catalytic subunit [Source:HGNC Symbol;Acc:HGNC:4311]	6
ENSG00000198142	4,360401	-0,80142	0,012498	0,045131	-2,789864	protein_coding	SOWAHC	sosondowah ankyrin repeat domain family member C [Source:HGNC Symbol;Acc:HGNC:26149]	2
ENSG00000167968	-0,245065	1,564426	0,012516	0,045167	2,789165	protein_coding	DNASE1L2	deoxyribonuclease 1 like 2 [Source:HGNC Symbol;Acc:HGNC:2958]	16
ENSG00000103449	1,073673	-3,32294	0,012513	0,045167	-2,789302	protein_coding	SALL1	spalt like transcription factor 1 [Source:HGNC Symbol;Acc:HGNC:10524]	16
ENSG00000110900	2,260146	-1,906485	0,012516	0,045167	-2,789181	protein_coding	TSPAN11	tetraspanin 11 [Source:HGNC Symbol;Acc:HGNC:30795]	12
ENSG00000151413	3,631678	0,611486	0,012534	0,0452	2,788506	protein_coding	NUBPL	nucleotide binding protein like [Source:HGNC Symbol;Acc:HGNC:20278]	14
ENSG00000169242	4,021214	1,508679	0,012532	0,0452	2,78857	protein_coding	EFNA1	ephrin A1 [Source:HGNC Symbol;Acc:HGNC:3221]	1
ENSG00000130024	5,700838	0,535289	0,01253	0,0452	2,788636	protein_coding	PHF10	PHD finger protein 10 [Source:HGNC Symbol;Acc:HGNC:18250]	6
ENSG00000162591	4,088177	1,679893	0,012548	0,045242	2,787964	protein_coding	MEGF6	multiple EGF like domains 6 [Source:HGNC Symbol;Acc:HGNC:3232]	1
ENSG00000189221	3,615601	2,182206	0,012555	0,045259	2,787683	protein_coding	MAOA	monoamine oxidase A [Source:HGNC Symbol;Acc:HGNC:6833]	X
ENSG00000165115	3,794675	1,066336	0,012563	0,045276	2,787392	protein_coding	KIF27	kinesin family member 27 [Source:HGNC Symbol;Acc:HGNC:18632]	9
ENSG00000162851	3,626519	-0,666784	0,01257	0,045292	-2,787124	protein_coding	TFB2M	transcription factor B2, mitochondrial [Source:HGNC Symbol;Acc:HGNC:18559]	1
ENSG00000140262	6,761924	-0,614946	0,012573	0,045292	-2,787016	protein_coding	TCF12	transcription factor 12 [Source:HGNC Symbol;Acc:HGNC:11623]	15
ENSG00000258839	2,696372	1,089753	0,012597	0,045367	2,786125	protein_coding	MC1R	melanocortin 1 receptor [Source:HGNC Symbol;Acc:HGNC:6929]	16
ENSG00000164708	-0,605207	1,889133	0,012654	0,045556	2,78397	protein_coding	PGAM2	phosphoglycerate mutase 2 [Source:HGNC Symbol;Acc:HGNC:8889]	7
ENSG00000182310	2,996508	0,908559	0,012655	0,045556	2,783945	protein_coding	SPACA6	sperm acrosome associated 6 [Source:HGNC Symbol;Acc:HGNC:27113]	19
ENSG00000221994	2,624767	0,981349	0,012674	0,045617	2,783207	protein_coding	ZNF630	zinc finger protein 630 [Source:HGNC Symbol;Acc:HGNC:28855]	X
ENSG00000070190	-0,140349	1,955771	0,012688	0,045654	2,782718	protein_coding	DAPP1	dual adaptor of phosphotyrosine and 3-phosphoinositides 1 [Source:HGNC Symbol;Acc:HGNC:16500]	4
ENSG00000206432	2,217175	-1,867416	0,012704	0,045704	-2,782091	protein_coding	TMEM200(	transmembrane protein 200C [Source:HGNC Symbol;Acc:HGNC:37208]	18
ENSG00000239998	-1,059082	2,122403	0,012714	0,045728	2,78174	protein_coding	LILRA2	leukocyte immunoglobulin like receptor A2 [Source:HGNC Symbol;Acc:HGNC:6603]	19
ENSG00000162654	2,777443	2,216816	0,012724	0,04575	2,781367	protein_coding	GBP4	guanylate binding protein 4 [Source:HGNC Symbol;Acc:HGNC:20480]	1
ENSG00000133460	3,940028	0,956913	0,012726	0,04575	2,781296	protein_coding	SLC2A11	solute carrier family 2 member 11 [Source:HGNC Symbol;Acc:HGNC:14239]	22
ENSG00000177105	4,087893	-0,659894	0,01274	0,045761	-2,780763	protein_coding	RHOG	ras homolog family member G [Source:HGNC Symbol;Acc:HGNC:672]	11
ENSG00000125967	4,773954	0,779413	0,012732	0,045761	2,781047	protein_coding	NECAB3	N-terminal EF-hand calcium binding protein 3 [Source:HGNC Symbol;Acc:HGNC:15851]	20
ENSG00000138442	4,859922	-0,487006	0,012736	0,045761	-2,780916	protein_coding	WDR12	WD repeat domain 12 [Source:HGNC Symbol;Acc:HGNC:14098]	2
ENSG00000109133	6,159835	-0,670396	0,012737	0,045761	-2,780864	protein_coding	TMEM33	transmembrane protein 33 [Source:HGNC Symbol;Acc:HGNC:25541]	4
ENSG00000137959	2,049433	2,200466	0,012751	0,045789	2,780365	protein_coding	IFI44L	interferon induced protein 44 like [Source:HGNC Symbol;Acc:HGNC:17817]	1
ENSG00000162601	5,713906	0,408612	0,012757	0,045803	2,780123	protein_coding	MYSM1	Myb like, SWIRM and MPN domains 1 [Source:HGNC Symbol;Acc:HGNC:29401]	1
ENSG00000219607	-0,014989	1,09546	0,012764	0,045818	2,779857	protein_coding	PPP1R3G	protein phosphatase 1 regulatory subunit 3G [Source:HGNC Symbol;Acc:HGNC:14945]	6
ENSG00000100650	7,339576	0,502798	0,012768	0,045821	2,779726	protein_coding	SRSF5	serine and arginine rich splicing factor 5 [Source:HGNC Symbol;Acc:HGNC:10787]	14
ENSG00000179918	5,801404	-0,804162	0,012778	0,045847	-2,779351	protein_coding	SEPHS2	selenophosphate synthetase 2 [Source:HGNC Symbol;Acc:HGNC:19686]	16
ENSG00000018280	3,187373	2,23972	0,012801	0,045902	2,778494	protein_coding	SLC11A1	solute carrier family 11 member 1 [Source:HGNC Symbol;Acc:HGNC:10907]	2
ENSG00000119640	3,342186	1,13156	0,012801	0,045902	2,778487	protein_coding	ACYP1	acylphosphatase 1 [Source:HGNC Symbol;Acc:HGNC:179]	14
ENSG00000064115	6,12954	-0,907553	0,012802	0,045902	-2,778469	protein_coding	TM7SF3	transmembrane 7 superfamily member 3 [Source:HGNC Symbol;Acc:HGNC:23049]	12
ENSG00000170236	-1,34158	1,377874	0,012812	0,045927	2,778102	protein_coding	USP50	ubiquitin specific peptidase 50 [Source:HGNC Symbol;Acc:HGNC:20079]	15
ENSG00000171860	1,344138	1,810705	0,012818	0,045932	2,777858	protein_coding	C3AR1	complement C3a receptor 1 [Source:HGNC Symbol;Acc:HGNC:1319]	12
ENSG00000100442	5,487409	-0,541611	0,012819	0,045932	-2,777842	protein_coding	FKBP3	FKBP prolyl isomerase 3 [Source:HGNC Symbol;Acc:HGNC:3719]	14
ENSG00000175564	0,6236	0,940803	0,012838	0,045981	2,777124	protein_coding	UCP3	uncoupling protein 3 [Source:HGNC Symbol;Acc:HGNC:12519]	11
ENSG00000177427	3,423986	0,934988	0,012837	0,045981	2,777165	protein_coding	MIEF2	mitochondrial elongation factor 2 [Source:HGNC Symbol;Acc:HGNC:17920]	17
ENSG00000167994	2,965189	1,06842	0,012844	0,045992	2,776907	protein_coding	RAB3IL1	RAB3A interacting protein like 1 [Source:HGNC Symbol;Acc:HGNC:9780]	11

ENSG00000166025	7,183379	-0,655791	0,012848	0,045999	-2,776734	protein_coding	AMOTL1	angiomotin like 1 [Source:HGNC Symbol;Acc:HGNC:17811]	11
ENSG00000066735	3,133184	2,696244	0,01286	0,046024	2,776304	protein_coding	KIF26A	kinesin family member 26A [Source:HGNC Symbol;Acc:HGNC:20226]	14
ENSG00000171291	3,800627	0,65857	0,012861	0,046024	2,776261	protein_coding	ZNF439	zinc finger protein 439 [Source:HGNC Symbol;Acc:HGNC:20873]	19
ENSG00000170469	0,960642	1,018797	0,012867	0,046026	2,776034	protein_coding	SPATA24	spermatogenesis associated 24 [Source:HGNC Symbol;Acc:HGNC:27322]	5
ENSG00000180787	4,146883	0,916504	0,012867	0,046026	2,776049	protein_coding	ZFP3	ZFP3 zinc finger protein [Source:HGNC Symbol;Acc:HGNC:12861]	17
ENSG00000144331	1,346461	2,023935	0,012873	0,046033	2,775811	protein_coding	ZNF385B	zinc finger protein 385B [Source:HGNC Symbol;Acc:HGNC:26332]	2
ENSG00000067191	3,775323	0,971214	0,012875	0,046033	2,775756	protein_coding	CACNB1	calcium voltage-gated channel auxiliary subunit beta 1 [Source:HGNC Symbol;Acc:HGNC:1401]	17
ENSG00000135407	3,018354	1,360234	0,01289	0,046072	2,775186	protein_coding	AVIL	advillin [Source:HGNC Symbol;Acc:HGNC:14188]	12
ENSG00000167637	3,879177	0,535535	0,012895	0,046072	2,775033	protein_coding	ZNF283	zinc finger protein 283 [Source:HGNC Symbol;Acc:HGNC:13077]	19
ENSG00000113810	7,314238	-0,736189	0,012893	0,046072	-2,775108	protein_coding	SMC4	structural maintenance of chromosomes 4 [Source:HGNC Symbol;Acc:HGNC:14013]	3
ENSG00000104957	4,96371	0,558767	0,012929	0,046173	2,773784	protein_coding	CCDC130	coiled-coil domain containing 130 [Source:HGNC Symbol;Acc:HGNC:28118]	19
ENSG00000175073	6,069677	-0,434597	0,012928	0,046173	-2,773808	protein_coding	VCPIP1	valosin containing protein interacting protein 1 [Source:HGNC Symbol;Acc:HGNC:30897]	8
ENSG00000140259	5,83395	-0,458413	0,012944	0,046212	-2,773213	protein_coding	MFAP1	microfibril associated protein 1 [Source:HGNC Symbol;Acc:HGNC:7032]	15
ENSG00000090863	8,308809	-0,484268	0,012945	0,046212	-2,773182	protein_coding	GLG1	golgi glycoprotein 1 [Source:HGNC Symbol;Acc:HGNC:4316]	16
ENSG00000156875	3,990447	0,513619	0,01299	0,046363	2,771529	protein_coding	MFSD14A	major facilitator superfamily domain containing 14A [Source:HGNC Symbol;Acc:HGNC:23363]	1
ENSG00000164117	3,505244	-0,677355	0,013003	0,0464	-2,771041	protein_coding	FBXO8	F-box protein 8 [Source:HGNC Symbol;Acc:HGNC:13587]	4
ENSG00000156110	4,473628	-0,559479	0,013035	0,046494	-2,769873	protein_coding	ADK	adenosine kinase [Source:HGNC Symbol;Acc:HGNC:257]	10
ENSG00000101577	6,64301	-0,471023	0,013034	0,046494	-2,769926	protein_coding	LPIN2	lipin 2 [Source:HGNC Symbol;Acc:HGNC:14450]	18
ENSG00000180096	1,545363	1,293813	0,013042	0,046506	2,769647	protein_coding	SEPTIN1	septin 1 [Source:HGNC Symbol;Acc:HGNC:2879]	16
ENSG00000176485	4,496806	1,318255	0,013078	0,046606	2,768317	protein_coding	PLAAT3	phospholipase A and acyltransferase 3 [Source:HGNC Symbol;Acc:HGNC:17825]	11
ENSG00000103202	5,247732	-0,645973	0,013075	0,046606	-2,76842	protein_coding	NME4	NME/NM23 nucleoside diphosphate kinase 4 [Source:HGNC Symbol;Acc:HGNC:7852]	16
ENSG00000189403	7,20485	-0,583122	0,013076	0,046606	-2,768386	protein_coding	HMGB1	high mobility group box 1 [Source:HGNC Symbol;Acc:HGNC:4983]	13
ENSG00000167676	0,466695	4,500785	0,013095	0,046656	2,767701	protein_coding	PLIN4	perilipin 4 [Source:HGNC Symbol;Acc:HGNC:29393]	19
ENSG00000204632	1,691194	1,834603	0,013104	0,046656	2,767379	protein_coding	HLA-G	major histocompatibility complex, class I, G [Source:HGNC Symbol;Acc:HGNC:4964]	6
ENSG00000166263	4,496419	0,382746	0,013105	0,046656	2,767336	protein_coding	STXBP4	syntaxin binding protein 4 [Source:HGNC Symbol;Acc:HGNC:19694]	17
ENSG00000152413	4,585781	-0,864828	0,013103	0,046656	-2,76742	protein_coding	HOMER1	homer scaffold protein 1 [Source:HGNC Symbol;Acc:HGNC:17512]	5
ENSG00000145246	5,755234	-0,730772	0,013107	0,046656	-2,767278	protein_coding	ATP10D	ATPase phospholipid transporting 10D (putative) [Source:HGNC Symbol;Acc:HGNC:13549]	4
ENSG00000169857	4,155522	-0,683781	0,013121	0,046696	-2,766772	protein_coding	AVEN	apoptosis and caspase activation inhibitor [Source:HGNC Symbol;Acc:HGNC:13509]	15
ENSG00000145113	-0,813791	2,099912	0,013131	0,046722	2,766403	protein_coding	MUC4	mucin 4, cell surface associated [Source:HGNC Symbol;Acc:HGNC:7514]	3
ENSG00000138606	2,403908	1,587779	0,013141	0,046748	2,766035	protein_coding	SHF	Src homology 2 domain containing F [Source:HGNC Symbol;Acc:HGNC:25116]	15
ENSG00000272916	-1,128001	1,721251	0,013155	0,046785	2,765554	protein_coding	AC022400.	novel transcript	10
ENSG00000057704	3,764817	1,450604	0,013158	0,046787	2,765428	protein_coding	TMCC3	transmembrane and coiled-coil domain family 3 [Source:HGNC Symbol;Acc:HGNC:29199]	12
ENSG00000173818	3,569706	0,705016	0,013167	0,046808	2,765118	protein_coding	ENDOV	endonuclease V [Source:HGNC Symbol;Acc:HGNC:26640]	17
ENSG00000164430	2,315356	-1,372889	0,01317	0,04681	-2,764992	protein_coding	CGAS	cyclic GMP-AMP synthase [Source:HGNC Symbol;Acc:HGNC:21367]	6
ENSG00000074657	6,542148	-0,998559	0,013174	0,046815	-2,764833	protein_coding	ZNF532	zinc finger protein 532 [Source:HGNC Symbol;Acc:HGNC:30940]	18
ENSG00000139182	6,151727	0,967203	0,013181	0,046821	2,764603	protein_coding	CLSTN3	calsyntenin 3 [Source:HGNC Symbol;Acc:HGNC:18371]	12
ENSG00000185963	6,508571	-0,45009	0,013182	0,046821	-2,76457	protein_coding	BICD2	BICD cargo adaptor 2 [Source:HGNC Symbol;Acc:HGNC:17208]	9
ENSG00000186642	2,642187	1,789875	0,013185	0,046821	2,764457	protein_coding	PDE2A	phosphodiesterase 2A [Source:HGNC Symbol;Acc:HGNC:8777]	11
ENSG00000125775	2,824824	-1,552833	0,013197	0,046854	-2,764025	protein_coding	SDCBP2	syndecan binding protein 2 [Source:HGNC Symbol;Acc:HGNC:15756]	20
ENSG00000103194	5,61198	-0,344578	0,013216	0,046913	-2,763322	protein_coding	USP10	ubiquitin specific peptidase 10 [Source:HGNC Symbol;Acc:HGNC:12608]	16
ENSG00000156042	2,496871	2,449724	0,013227	0,046941	2,762935	protein_coding	CFAP70	cilia and flagella associated protein 70 [Source:HGNC Symbol;Acc:HGNC:30726]	10
ENSG00000136877	5,083274	0,483413	0,01323	0,046941	2,762831	protein_coding	FPGS	folylpolyglutamate synthase [Source:HGNC Symbol;Acc:HGNC:3824]	9
ENSG00000101825	6,865486	-2,139399	0,013242	0,046973	-2,7624	protein_coding	MXRA5	matrix remodeling associated 5 [Source:HGNC Symbol;Acc:HGNC:7539]	X
ENSG00000204161	0,561274	1,938808	0,013264	0,047041	2,76161	protein_coding	TMEM273	transmembrane protein 273 [Source:HGNC Symbol;Acc:HGNC:27274]	10
ENSG00000166573	-0,960385	4,0012	0,013281	0,047089	2,761021	protein_coding	GALR1	galanin receptor 1 [Source:HGNC Symbol;Acc:HGNC:4132]	18
ENSG00000125531	0,178993	1,342345	0,013299	0,047143	2,760358	protein_coding	FNDC11	fibronectin type III domain containing 11 [Source:HGNC Symbol;Acc:HGNC:28764]	20
ENSG00000167088	5,791282	-0,548917	0,013302	0,047143	-2,760272	protein_coding	SNRPD1	small nuclear ribonucleoprotein D1 polypeptide [Source:HGNC Symbol;Acc:HGNC:11158]	18
ENSG00000198863	5,767419	0,615764	0,013309	0,047159	2,760005	protein_coding	RUNDC1	RUN domain containing 1 [Source:HGNC Symbol;Acc:HGNC:25418]	17
ENSG00000278318	2,865991	-0,938897	0,013318	0,047171	-2,75968	protein_coding	ZNF229	zinc finger protein 229 [Source:HGNC Symbol;Acc:HGNC:13022]	19
ENSG00000160216	6,639232	-0,517085	0,013317	0,047171	-2,759721	protein_coding	AGPAT3	1-acylglycerol-3-phosphate O-acyltransferase 3 [Source:HGNC Symbol;Acc:HGNC:326]	21
ENSG00000136237	5,73617	1,257839	0,013345	0,047254	2,758737	protein_coding	RAPGEF5	Rap guanine nucleotide exchange factor 5 [Source:HGNC Symbol;Acc:HGNC:16862]	7

ENSG00000104671	3,746972	-0,632197	0,013349	0,04726	-2,75857	protein_coding	DCTN6	dynactin subunit 6 [Source:HGNC Symbol;Acc:HGNC:16964]	8
ENSG00000171208	4,487567	-1,262941	0,013391	0,047399	-2,757071	protein_coding	NETO2	neuropilin and tolloid like 2 [Source:HGNC Symbol;Acc:HGNC:14644]	16
ENSG00000154511	3,052704	-1,239312	0,013417	0,047471	-2,756148	protein_coding	DIPK1A	divergent protein kinase domain 1A [Source:HGNC Symbol;Acc:HGNC:32213]	1
ENSG00000073350	6,455332	1,429855	0,013417	0,047471	2,756168	protein_coding	LLGL2	LLGL scribble cell polarity complex component 2 [Source:HGNC Symbol;Acc:HGNC:6629]	17
ENSG00000155849	4,454229	1,78473	0,01343	0,047506	2,755686	protein_coding	ELMO1	engulfment and cell motility 1 [Source:HGNC Symbol;Acc:HGNC:16286]	7
ENSG00000102595	6,061958	-0,561367	0,013436	0,047517	-2,755477	protein_coding	UGGT2	UDP-glucose glycoprotein glucosyltransferase 2 [Source:HGNC Symbol;Acc:HGNC:15664]	13
ENSG00000143554	4,377888	0,733927	0,013454	0,04757	2,754839	protein_coding	SLC27A3	solute carrier family 27 member 3 [Source:HGNC Symbol;Acc:HGNC:10997]	1
ENSG00000032219	6,002798	0,530524	0,013459	0,047575	2,754693	protein_coding	ARID4A	AT-rich interaction domain 4A [Source:HGNC Symbol;Acc:HGNC:9885]	14
ENSG00000171155	4,381417	-0,877153	0,013471	0,047609	-2,754246	protein_coding	C1GALT1C:	C1GALT1 specific chaperone 1 [Source:HGNC Symbol;Acc:HGNC:24338]	X
ENSG00000163220	1,547006	2,245952	0,013479	0,047627	2,753964	protein_coding	S100A9	S100 calcium binding protein A9 [Source:HGNC Symbol;Acc:HGNC:10499]	1
ENSG00000107404	5,557303	-0,604423	0,013487	0,047633	-2,753695	protein_coding	DVL1	dishevelled segment polarity protein 1 [Source:HGNC Symbol;Acc:HGNC:3084]	1
ENSG00000067704	6,491584	-0,49217	0,013486	0,047633	-2,753708	protein_coding	IARS2	isoleucyl-tRNA synthetase 2, mitochondrial [Source:HGNC Symbol;Acc:HGNC:29685]	1
ENSG00000176953	5,28557	0,575786	0,013501	0,047674	2,753184	protein_coding	NFATC2IP	nuclear factor of activated T cells 2 interacting protein [Source:HGNC Symbol;Acc:HGNC:25906]	16
ENSG00000085741	2,32994	2,094128	0,01351	0,047677	2,752874	protein_coding	WNT11	Wnt family member 11 [Source:HGNC Symbol;Acc:HGNC:12776]	11
ENSG00000054116	4,699124	-0,467609	0,013505	0,047677	-2,753044	protein_coding	TRAPPC3	trafficking protein particle complex 3 [Source:HGNC Symbol;Acc:HGNC:19942]	1
ENSG00000166946	5,314992	-0,624195	0,013511	0,047677	-2,752844	protein_coding	CCNDBP1	cyclin D1 binding protein 1 [Source:HGNC Symbol;Acc:HGNC:1587]	15
ENSG00000275066	6,360298	0,476837	0,013534	0,047748	2,752035	protein_coding	SYNRG	synergin gamma [Source:HGNC Symbol;Acc:HGNC:557]	17
ENSG00000167930	5,972925	-0,680888	0,013554	0,047808	-2,751333	protein_coding	FAM234A	family with sequence similarity 234 member A [Source:HGNC Symbol;Acc:HGNC:14163]	16
ENSG00000171815	-1,247043	1,880817	0,013566	0,04783	2,75091	protein_coding	PCDHB1	protocadherin beta 1 [Source:HGNC Symbol;Acc:HGNC:8680]	5
ENSG00000159658	7,047135	-0,411424	0,013564	0,04783	-2,750967	protein_coding	EFCAB14	EF-hand calcium binding domain 14 [Source:HGNC Symbol;Acc:HGNC:29051]	1
ENSG00000168256	5,711863	-0,40115	0,013574	0,047838	-2,750624	protein_coding	NKIRAS2	NFKB inhibitor interacting Ras like 2 [Source:HGNC Symbol;Acc:HGNC:17898]	17
ENSG00000244038	6,800689	-0,502713	0,013572	0,047838	-2,750697	protein_coding	DDOST	dolichyl-diphosphooligosaccharide--protein glycosyltransferase non-catalytic subunit [Source:HGNC Symbol;Acc:HG	1
ENSG00000157873	5,254042	0,968109	0,013586	0,047871	2,750192	protein_coding	TNFRSF14	TNF receptor superfamily member 14 [Source:HGNC Symbol;Acc:HGNC:11912]	1
ENSG00000175931	6,416708	0,636598	0,013607	0,047926	2,749472	protein_coding	UBE2O	ubiquitin conjugating enzyme E2 O [Source:HGNC Symbol;Acc:HGNC:29554]	17
ENSG00000068305	6,548782	-0,505431	0,013608	0,047926	-2,749433	protein_coding	MEF2A	myocyte enhancer factor 2A [Source:HGNC Symbol;Acc:HGNC:6993]	15
ENSG00000173391	0,768533	2,639901	0,013616	0,047943	2,74917	protein_coding	OLR1	oxidized low density lipoprotein receptor 1 [Source:HGNC Symbol;Acc:HGNC:8133]	12
ENSG00000101542	-2,728846	1,950618	0,013622	0,047943	2,748945	protein_coding	CDH20	cadherin 20 [Source:HGNC Symbol;Acc:HGNC:1760]	18
ENSG00000172382	-0,306749	1,09848	0,013631	0,047943	2,748646	protein_coding	PRSS27	serine protease 27 [Source:HGNC Symbol;Acc:HGNC:15475]	16
ENSG00000133256	1,230545	2,208256	0,013629	0,047943	2,748703	protein_coding	PDE6B	phosphodiesterase 6B [Source:HGNC Symbol;Acc:HGNC:8786]	4
ENSG00000156298	2,630309	1,770895	0,013629	0,047943	2,748698	protein_coding	TSPAN7	tetraspanin 7 [Source:HGNC Symbol;Acc:HGNC:11854]	X
ENSG00000183044	4,463241	1,843649	0,013625	0,047943	2,748835	protein_coding	ABAT	4-aminobutyrate aminotransferase [Source:HGNC Symbol;Acc:HGNC:23]	16
ENSG00000095627	-0,821656	-1,584356	0,013661	0,048038	-2,747603	protein_coding	TDRD1	tudor domain containing 1 [Source:HGNC Symbol;Acc:HGNC:11712]	10
ENSG00000135679	7,515743	-0,523884	0,013668	0,048052	-2,747359	protein_coding	MDM2	MDM2 proto-oncogene [Source:HGNC Symbol;Acc:HGNC:6973]	12
ENSG00000182272	2,813938	2,552623	0,013697	0,048146	2,746328	protein_coding	B4GALNT4	beta-1,4-N-acetyl-galactosaminyltransferase 4 [Source:HGNC Symbol;Acc:HGNC:26315]	11
ENSG00000144908	2,278966	1,850108	0,013726	0,048236	2,745341	protein_coding	ALDH1L1	aldehyde dehydrogenase 1 family member L1 [Source:HGNC Symbol;Acc:HGNC:3978]	3
ENSG00000010270	5,065067	-0,511944	0,013733	0,048251	-2,745093	protein_coding	STARD3NL	STARD3 N-terminal like [Source:HGNC Symbol;Acc:HGNC:19169]	7
ENSG00000175756	5,737355	-0,484576	0,013738	0,048257	-2,744926	protein_coding	AURKAIP1	aurora kinase A interacting protein 1 [Source:HGNC Symbol;Acc:HGNC:24114]	1
ENSG00000116299	6,416301	1,621945	0,013747	0,048279	2,744608	protein_coding	ELAPOR1	endosome-lysosome associated apoptosis and autophagy regulator 1 [Source:HGNC Symbol;Acc:HGNC:29618]	1
ENSG00000215252	3,922603	1,747813	0,013757	0,048304	2,744261	protein_coding	GOLGA8B	golgin A8 family member B [Source:HGNC Symbol;Acc:HGNC:31973]	15
ENSG00000095015	5,433814	0,755804	0,013764	0,048317	2,744028	protein_coding	MAP3K1	mitogen-activated protein kinase kinase kinase 1 [Source:HGNC Symbol;Acc:HGNC:6848]	5
ENSG00000026036	1,928054	1,146801	0,013777	0,048353	2,743571	protein_coding	RTEL1-TNF	RTEL1-TNFRSF6B readthrough (NMD candidate) [Source:HGNC Symbol;Acc:HGNC:44095]	20
ENSG00000147118	3,623439	0,531619	0,013795	0,048407	2,742933	protein_coding	ZNF182	zinc finger protein 182 [Source:HGNC Symbol;Acc:HGNC:13001]	X
ENSG00000148848	6,072986	-1,772977	0,013807	0,048437	-2,742533	protein_coding	ADAM12	ADAM metallopeptidase domain 12 [Source:HGNC Symbol;Acc:HGNC:190]	10
ENSG00000275221	2,800922	0,582617	0,013818	0,048445	2,742151	protein_coding	H2AC15	H2A clustered histone 15 [Source:HGNC Symbol;Acc:HGNC:4726]	6
ENSG00000143436	5,095018	-0,701035	0,013815	0,048445	-2,742269	protein_coding	MRPL9	mitochondrial ribosomal protein L9 [Source:HGNC Symbol;Acc:HGNC:14277]	1
ENSG00000124767	5,478667	-0,575797	0,013816	0,048445	-2,742222	protein_coding	GLO1	glyoxalase I [Source:HGNC Symbol;Acc:HGNC:4323]	6
ENSG00000135077	2,226108	1,540882	0,013821	0,048447	2,74203	protein_coding	HAVCR2	hepatitis A virus cellular receptor 2 [Source:HGNC Symbol;Acc:HGNC:18437]	5
ENSG00000172795	5,360631	0,5851	0,013828	0,048459	2,741809	protein_coding	DCP2	decapping mRNA 2 [Source:HGNC Symbol;Acc:HGNC:24452]	5
ENSG00000235098	1,156807	2,499071	0,01385	0,048526	2,741047	protein_coding	ANKRD65	ankyrin repeat domain 65 [Source:HGNC Symbol;Acc:HGNC:42950]	1
ENSG00000070193	-1,979608	2,826853	0,013891	0,048645	2,739658	protein_coding	FGF10	fibroblast growth factor 10 [Source:HGNC Symbol;Acc:HGNC:3666]	5
ENSG00000183474	2,600171	-0,574183	0,013893	0,048645	-2,739576	protein_coding	GTF2H2C	GTF2H2 family member C [Source:HGNC Symbol;Acc:HGNC:31394]	5

ENSG00000106948	5,60185	1,123155	0,01389	0,048645	2,739661	protein_coding	AKNA	AT-hook transcription factor [Source:HGNC Symbol;Acc:HGNC:24108]	9
ENSG00000265808	5,639944	-0,702405	0,0139	0,048648	-2,73934	protein_coding	SEC22B	SEC22 homolog B, vesicle trafficking protein [Source:HGNC Symbol;Acc:HGNC:10700]	1
ENSG00000140990	5,821018	-0,548499	0,0139	0,048648	-2,739349	protein_coding	NDUFB10	NADH:ubiquinone oxidoreductase subunit B10 [Source:HGNC Symbol;Acc:HGNC:7696]	16
ENSG00000164129	-2,256787	-3,038576	0,01393	0,04868	-2,738301	protein_coding	NPY5R	neuropeptide Y receptor Y5 [Source:HGNC Symbol;Acc:HGNC:7958]	4
ENSG00000183117	-0,324374	2,829483	0,013933	0,04868	2,738202	protein_coding	CSMD1	CUB and Sushi multiple domains 1 [Source:HGNC Symbol;Acc:HGNC:14026]	8
ENSG00000069431	4,346042	1,226202	0,013924	0,04868	2,738511	protein_coding	ABCC9	ATP binding cassette subfamily C member 9 [Source:HGNC Symbol;Acc:HGNC:60]	12
ENSG00000163257	5,50371	0,636116	0,013926	0,04868	2,738444	protein_coding	DCAF16	DDB1 and CUL4 associated factor 16 [Source:HGNC Symbol;Acc:HGNC:25987]	4
ENSG00000211456	5,538336	-0,383083	0,013933	0,04868	-2,738209	protein_coding	SACM1L	SAC1 like phosphatidylinositide phosphatase [Source:HGNC Symbol;Acc:HGNC:17059]	3
ENSG00000090061	6,046846	-0,404941	0,013921	0,04868	-2,738605	protein_coding	CCNK	cyclin K [Source:HGNC Symbol;Acc:HGNC:1596]	14
ENSG00000141503	7,495283	0,640096	0,013915	0,04868	2,738804	protein_coding	MINK1	misshapen like kinase 1 [Source:HGNC Symbol;Acc:HGNC:17565]	17
ENSG00000177119	6,776822	-1,049774	0,013915	0,04868	-2,738823	protein_coding	ANO6	anoctamin 6 [Source:HGNC Symbol;Acc:HGNC:25240]	12
ENSG00000156234	-2,433269	3,719024	0,013964	0,048778	2,737142	protein_coding	CXCL13	C-X-C motif chemokine ligand 13 [Source:HGNC Symbol;Acc:HGNC:10639]	4
ENSG00000148541	2,747367	1,489723	0,013985	0,048836	2,736413	protein_coding	FAM13C	family with sequence similarity 13 member C [Source:HGNC Symbol;Acc:HGNC:19371]	10
ENSG00000108963	4,160267	0,69672	0,013987	0,048836	2,736377	protein_coding	DPH1	diphthamide biosynthesis 1 [Source:HGNC Symbol;Acc:HGNC:3003]	17
ENSG00000172461	0,11589	3,302125	0,013992	0,048846	2,736176	protein_coding	FUT9	fucosyltransferase 9 [Source:HGNC Symbol;Acc:HGNC:4020]	6
ENSG00000060718	2,150573	5,355964	0,014017	0,048902	2,735343	protein_coding	COL11A1	collagen type XI alpha 1 chain [Source:HGNC Symbol;Acc:HGNC:2186]	1
ENSG00000176386	3,506666	-0,555453	0,014018	0,048902	-2,735323	protein_coding	CDC26	cell division cycle 26 [Source:HGNC Symbol;Acc:HGNC:17839]	9
ENSG00000065717	4,148884	1,909287	0,014012	0,048902	2,735514	protein_coding	TLE2	TLE family member 2, transcriptional corepressor [Source:HGNC Symbol;Acc:HGNC:11838]	19
ENSG00000065675	-0,274793	3,160879	0,014036	0,048934	2,734687	protein_coding	PRKCQ	protein kinase C theta [Source:HGNC Symbol;Acc:HGNC:9410]	10
ENSG00000178226	-0,438263	1,433656	0,014036	0,048934	2,734696	protein_coding	PRSS36	serine protease 36 [Source:HGNC Symbol;Acc:HGNC:26906]	16
ENSG00000182667	4,395403	-1,761365	0,014038	0,048934	-2,734624	protein_coding	NTM	neurotrimin [Source:HGNC Symbol;Acc:HGNC:17941]	11
ENSG00000055130	6,238745	-0,382813	0,014039	0,048934	-2,734596	protein_coding	CUL1	cullin 1 [Source:HGNC Symbol;Acc:HGNC:2551]	7
ENSG00000137473	-2,79115	2,309722	0,014061	0,049	2,733859	protein_coding	TTC29	tetratricopeptide repeat domain 29 [Source:HGNC Symbol;Acc:HGNC:29936]	4
ENSG00000070961	7,334847	-0,895112	0,014069	0,049017	-2,733592	protein_coding	ATP2B1	ATPase plasma membrane Ca2+ transporting 1 [Source:HGNC Symbol;Acc:HGNC:814]	12
ENSG00000104983	3,334641	0,819508	0,014085	0,049044	2,733025	protein_coding	CCDC61	coiled-coil domain containing 61 [Source:HGNC Symbol;Acc:HGNC:33629]	19
ENSG00000177595	3,980016	0,866828	0,014079	0,049044	2,733225	protein_coding	PIDD1	p53-induced death domain protein 1 [Source:HGNC Symbol;Acc:HGNC:16491]	11
ENSG00000174437	8,556888	-0,432254	0,014083	0,049044	-2,733119	protein_coding	ATP2A2	ATPase sarcoplasmic/endoplasmic reticulum Ca2+ transporting 2 [Source:HGNC Symbol;Acc:HGNC:812]	12
ENSG00000205423	3,263911	-0,5433	0,014101	0,049084	-2,732486	protein_coding	CNEP1R1	CTD nuclear envelope phosphatase 1 regulatory subunit 1 [Source:HGNC Symbol;Acc:HGNC:26759]	16
ENSG00000104687	6,424501	-0,84959	0,014103	0,049084	-2,732428	protein_coding	GSR	glutathione-disulfide reductase [Source:HGNC Symbol;Acc:HGNC:4623]	8
ENSG00000154845	6,693027	-0,500677	0,014111	0,0491	-2,732172	protein_coding	PPP4R1	protein phosphatase 4 regulatory subunit 1 [Source:HGNC Symbol;Acc:HGNC:9320]	18
ENSG00000169758	-0,518309	2,541253	0,014132	0,049165	2,731443	protein_coding	TMEM266	transmembrane protein 266 [Source:HGNC Symbol;Acc:HGNC:26763]	15
ENSG00000182923	5,391137	0,408285	0,014146	0,049201	2,730988	protein_coding	CEP63	centrosomal protein 63 [Source:HGNC Symbol;Acc:HGNC:25815]	3
ENSG00000173531	2,566669	1,500913	0,014159	0,049238	2,730523	protein_coding	MST1	macrophage stimulating 1 [Source:HGNC Symbol;Acc:HGNC:7380]	3
ENSG00000071282	5,10093	0,997351	0,014174	0,049277	2,730049	protein_coding	LMCD1	LIM and cysteine rich domains 1 [Source:HGNC Symbol;Acc:HGNC:6633]	3
ENSG00000138639	4,55654	-1,231423	0,014182	0,049295	-2,729775	protein_coding	ARHGAP24	Rho GTPase activating protein 24 [Source:HGNC Symbol;Acc:HGNC:25361]	4
ENSG00000148296	5,637113	0,40738	0,014191	0,049315	2,729472	protein_coding	SURF6	surfeit 6 [Source:HGNC Symbol;Acc:HGNC:11478]	9
ENSG00000179364	6,558174	0,422263	0,014212	0,04938	2,72875	protein_coding	PACS2	phosphofurin acidic cluster sorting protein 2 [Source:HGNC Symbol;Acc:HGNC:23794]	14
ENSG00000124523	3,543916	0,645464	0,01423	0,04943	2,728163	protein_coding	SIRT5	sirtuin 5 [Source:HGNC Symbol;Acc:HGNC:14933]	6
ENSG00000121073	5,033604	-0,575712	0,014241	0,049452	-2,727785	protein_coding	SLC35B1	solute carrier family 35 member B1 [Source:HGNC Symbol;Acc:HGNC:20798]	17
ENSG00000181555	7,44213	0,409087	0,014242	0,049452	2,72775	protein_coding	SETD2	SET domain containing 2, histone lysine methyltransferase [Source:HGNC Symbol;Acc:HGNC:18420]	3
ENSG00000166823	0,438344	1,732185	0,014248	0,04946	2,727568	protein_coding	MESP1	mesoderm posterior bHLH transcription factor 1 [Source:HGNC Symbol;Acc:HGNC:29658]	15
ENSG00000091164	6,354876	-0,492247	0,014255	0,049477	-2,727302	protein_coding	TXNL1	thioredoxin like 1 [Source:HGNC Symbol;Acc:HGNC:12436]	18
ENSG00000155085	3,454135	0,855639	0,014278	0,049544	2,726555	protein_coding	AK9	adenylate kinase 9 [Source:HGNC Symbol;Acc:HGNC:33814]	6
ENSG00000105650	-1,185129	2,675856	0,014296	0,049598	2,725937	protein_coding	PDE4C	phosphodiesterase 4C [Source:HGNC Symbol;Acc:HGNC:8782]	19
ENSG00000117480	3,87722	1,016166	0,014306	0,049619	2,725627	protein_coding	FAAH	fatty acid amide hydrolase [Source:HGNC Symbol;Acc:HGNC:3553]	1
ENSG00000198598	3,164321	-1,474405	0,014312	0,049631	-2,725415	protein_coding	MMP17	matrix metallopeptidase 17 [Source:HGNC Symbol;Acc:HGNC:7163]	12
ENSG00000157315	-1,381424	2,048603	0,01432	0,049635	2,725143	protein_coding	TMED6	transmembrane p24 trafficking protein 6 [Source:HGNC Symbol;Acc:HGNC:28331]	16
ENSG00000164011	2,446219	0,592355	0,014323	0,049635	2,725065	protein_coding	ZNF691	zinc finger protein 691 [Source:HGNC Symbol;Acc:HGNC:28028]	1
ENSG00000119705	5,186454	-0,4795	0,014318	0,049635	-2,725226	protein_coding	SLIRP	SRA stem-loop interacting RNA binding protein [Source:HGNC Symbol;Acc:HGNC:20495]	14
ENSG00000160472	-1,67235	2,65805	0,014351	0,049723	2,724122	protein_coding	TMEM190	transmembrane protein 190 [Source:HGNC Symbol;Acc:HGNC:29632]	19
ENSG00000179673	-0,199754	2,619313	0,014394	0,049861	2,722699	protein_coding	RPRML	reprimo like [Source:HGNC Symbol;Acc:HGNC:32422]	17

ENSG00000149823	5,949523	0,523572	0,014401	0,049877	2,722448	protein_coding	VPS51	VPS51 subunit of GARP complex [Source:HGNC Symbol;Acc:HGNC:1172]	11
ENSG00000121351	-3,228113	2,396019	0,014415	0,049913	2,721999	protein_coding	IAPP	islet amyloid polypeptide [Source:HGNC Symbol;Acc:HGNC:5329]	12
ENSG00000141668	-0,416075	-2,988438	0,014419	0,049914	-2,721848	protein_coding	CBLN2	cerebellin 2 precursor [Source:HGNC Symbol;Acc:HGNC:1544]	18
ENSG00000171448	3,527379	0,67669	0,014421	0,049914	2,721791	protein_coding	ZBTB26	zinc finger and BTB domain containing 26 [Source:HGNC Symbol;Acc:HGNC:23383]	9
ENSG00000135722	1,944833	0,747508	0,014426	0,049918	2,721643	protein_coding	FBXL8	F-box and leucine rich repeat protein 8 [Source:HGNC Symbol;Acc:HGNC:17875]	16
ENSG00000146243	3,082261	1,127368	0,014432	0,049926	2,721437	protein_coding	IRAK1BP1	interleukin 1 receptor associated kinase 1 binding protein 1 [Source:HGNC Symbol;Acc:HGNC:17368]	6
ENSG00000076928	6,666465	0,656588	0,014434	0,049926	2,721372	protein_coding	ARHGEF1	Rho guanine nucleotide exchange factor 1 [Source:HGNC Symbol;Acc:HGNC:681]	19
ENSG00000055609	8,47536	0,494809	0,01444	0,049937	2,721164	protein_coding	KMT2C	lysine methyltransferase 2C [Source:HGNC Symbol;Acc:HGNC:13726]	7
ENSG00000039650	5,524932	0,566438	0,014453	0,04997	2,720743	protein_coding	PNKP	polynucleotide kinase 3'-phosphatase [Source:HGNC Symbol;Acc:HGNC:9154]	19
ENSG00000178965	1,765081	2,84724	0,014457	0,049975	2,7206	protein_coding	ERICH3	glutamate rich 3 [Source:HGNC Symbol;Acc:HGNC:25346]	1
ENSG00000182446	7,283503	-0,472592	0,014471	0,050012	-2,720143	protein_coding	NPLOC4	NPL4 homolog, ubiquitin recognition factor [Source:HGNC Symbol;Acc:HGNC:18261]	17
ENSG00000131771	0,89174	3,557601	0,014477	0,050023	2,719935	protein_coding	PPP1R1B	protein phosphatase 1 regulatory inhibitor subunit 1B [Source:HGNC Symbol;Acc:HGNC:9287]	17
ENSG00000110975	-1,916906	3,146615	0,014493	0,050066	2,719425	protein_coding	SYT10	synaptotagmin 10 [Source:HGNC Symbol;Acc:HGNC:19266]	12
ENSG00000162706	0,633388	3,622946	0,014502	0,050086	2,719128	protein_coding	CADM3	cell adhesion molecule 3 [Source:HGNC Symbol;Acc:HGNC:17601]	1
ENSG00000172086	4,277051	-1,092901	0,01451	0,050105	-2,718853	protein_coding	KRCC1	lysine rich coiled-coil 1 [Source:HGNC Symbol;Acc:HGNC:28039]	2
ENSG00000107736	1,566103	2,682536	0,014539	0,050181	2,717925	protein_coding	CDH23	cadherin related 23 [Source:HGNC Symbol;Acc:HGNC:13733]	10
ENSG00000178397	3,42065	-0,603031	0,014538	0,050181	-2,717944	protein_coding	FAM220A	family with sequence similarity 220 member A [Source:HGNC Symbol;Acc:HGNC:22422]	7
ENSG00000188215	4,558887	-0,621619	0,014557	0,050224	-2,717316	protein_coding	DCUN1D3	defective in cullin neddylation 1 domain containing 3 [Source:HGNC Symbol;Acc:HGNC:28734]	16
ENSG00000115286	5,194733	0,52766	0,014556	0,050224	2,71734	protein_coding	NDUFS7	NADH:ubiquinone oxidoreductase core subunit S7 [Source:HGNC Symbol;Acc:HGNC:7714]	19
ENSG00000116095	5,104197	-0,477613	0,014563	0,050233	-2,717122	protein_coding	PLEKHA3	pleckstrin homology domain containing A3 [Source:HGNC Symbol;Acc:HGNC:14338]	2
ENSG00000196961	6,936213	-0,423474	0,014586	0,050302	-2,716369	protein_coding	AP2A1	adaptor related protein complex 2 subunit alpha 1 [Source:HGNC Symbol;Acc:HGNC:561]	19
ENSG00000138080	7,120313	-1,353083	0,01459	0,050306	-2,716229	protein_coding	EMILIN1	elastin microfibril interfacer 1 [Source:HGNC Symbol;Acc:HGNC:19880]	2
ENSG00000239779	2,193687	0,735968	0,014608	0,050356	2,715653	protein_coding	WBP1	WW domain binding protein 1 [Source:HGNC Symbol;Acc:HGNC:12737]	2
ENSG00000174473	-1,718185	2,473047	0,014618	0,050379	2,71534	protein_coding	GALNTL6	polypeptide N-acetylgalactosaminyltransferase like 6 [Source:HGNC Symbol;Acc:HGNC:33844]	4
ENSG00000109265	5,32224	-1,065139	0,014639	0,050429	-2,714645	protein_coding	CRACD	capping protein inhibiting regulator of actin dynamics [Source:HGNC Symbol;Acc:HGNC:29219]	4
ENSG00000163629	5,68839	-1,32136	0,014642	0,050429	-2,714558	protein_coding	PTPN13	protein tyrosine phosphatase non-receptor type 13 [Source:HGNC Symbol;Acc:HGNC:9646]	4
ENSG00000173889	6,95502	0,421412	0,014637	0,050429	2,714715	protein_coding	PHC3	polyhomeotic homolog 3 [Source:HGNC Symbol;Acc:HGNC:15682]	3
ENSG00000019991	3,417471	-3,228816	0,014656	0,050469	-2,714084	protein_coding	HGF	hepatocyte growth factor [Source:HGNC Symbol;Acc:HGNC:4893]	7
ENSG00000186510	-1,198701	2,204369	0,014661	0,050476	2,713916	protein_coding	CLCNKA	chloride voltage-gated channel Ka [Source:HGNC Symbol;Acc:HGNC:2026]	1
ENSG00000104321	1,374394	-5,796455	0,014672	0,050501	-2,713573	protein_coding	TRPA1	transient receptor potential cation channel subfamily A member 1 [Source:HGNC Symbol;Acc:HGNC:497]	8
ENSG00000078487	1,789233	0,998723	0,014682	0,050505	2,713233	protein_coding	ZCWPW1	zinc finger CW-type and PWWP domain containing 1 [Source:HGNC Symbol;Acc:HGNC:23486]	7
ENSG00000160961	4,068332	0,715547	0,014678	0,050505	2,71338	protein_coding	ZNF333	zinc finger protein 333 [Source:HGNC Symbol;Acc:HGNC:15624]	19
ENSG00000006625	3,951323	-0,776493	0,014682	0,050505	-2,713237	protein_coding	GGCT	gamma-glutamylcyclotransferase [Source:HGNC Symbol;Acc:HGNC:21705]	7
ENSG00000241106	-1,980828	1,884406	0,014689	0,050518	2,713006	protein_coding	HLA-DOB	major histocompatibility complex, class II, DO beta [Source:HGNC Symbol;Acc:HGNC:4937]	6
ENSG00000180447	1,1574	2,69131	0,014723	0,050614	2,711907	protein_coding	GAS1	growth arrest specific 1 [Source:HGNC Symbol;Acc:HGNC:4165]	9
ENSG00000089041	1,994662	1,52167	0,014722	0,050614	2,711942	protein_coding	P2RX7	purinergic receptor P2X 7 [Source:HGNC Symbol;Acc:HGNC:8537]	12
ENSG00000204060	1,823028	1,475685	0,014744	0,050675	2,711224	protein_coding	FOXO6	forkhead box O6 [Source:HGNC Symbol;Acc:HGNC:24814]	1
ENSG00000099974	-0,079299	1,700957	0,014771	0,050755	2,710368	protein_coding	DDTL	D-dopachrome tautomerase like [Source:HGNC Symbol;Acc:HGNC:33446]	22
ENSG00000185885	2,320149	2,22898	0,014776	0,050764	2,710188	protein_coding	IFITM1	interferon induced transmembrane protein 1 [Source:HGNC Symbol;Acc:HGNC:5412]	11
ENSG00000140564	7,440755	-0,891867	0,014795	0,050817	-2,709592	protein_coding	FURIN	furin, paired basic amino acid cleaving enzyme [Source:HGNC Symbol;Acc:HGNC:8568]	15
ENSG00000119147	-2,834414	2,128098	0,014808	0,050852	2,709163	protein_coding	ECRG4	ECRG4 augurin precursor [Source:HGNC Symbol;Acc:HGNC:24642]	2
ENSG00000164414	1,720613	-0,763176	0,014812	0,050853	-2,709027	protein_coding	SLC35A1	solute carrier family 35 member A1 [Source:HGNC Symbol;Acc:HGNC:11021]	6
ENSG00000113140	10,19538	-1,25758	0,014815	0,050853	-2,708944	protein_coding	SPARC	secreted protein acidic and cysteine rich [Source:HGNC Symbol;Acc:HGNC:11219]	5
ENSG00000147576	-0,318096	1,311582	0,01482	0,05086	2,708776	protein_coding	ADHFE1	alcohol dehydrogenase iron containing 1 [Source:HGNC Symbol;Acc:HGNC:16354]	8
ENSG00000185245	-2,904525	2,22121	0,014826	0,05087	2,708587	protein_coding	GP1BA	glycoprotein Ib platelet subunit alpha [Source:HGNC Symbol;Acc:HGNC:4439]	17
ENSG00000077713	3,619134	-0,901392	0,014829	0,05087	-2,708485	protein_coding	SLC25A43	solute carrier family 25 member 43 [Source:HGNC Symbol;Acc:HGNC:30557]	X
ENSG00000105767	4,113351	1,200219	0,014855	0,05095	2,707633	protein_coding	CADM4	cell adhesion molecule 4 [Source:HGNC Symbol;Acc:HGNC:30825]	19
ENSG00000158813	2,679814	1,582053	0,014877	0,050984	2,706946	protein_coding	EDA	ectodysplasin A [Source:HGNC Symbol;Acc:HGNC:3157]	X
ENSG00000103241	3,320813	-3,502307	0,014878	0,050984	-2,706907	protein_coding	FOXF1	forkhead box F1 [Source:HGNC Symbol;Acc:HGNC:3809]	16
ENSG00000132938	3,740721	2,417559	0,014881	0,050984	2,706815	protein_coding	MTUS2	microtubule associated scaffold protein 2 [Source:HGNC Symbol;Acc:HGNC:20595]	13
ENSG00000133641	3,843929	-0,651081	0,014874	0,050984	-2,707052	protein_coding	C12orf29	chromosome 12 open reading frame 29 [Source:HGNC Symbol;Acc:HGNC:25322]	12

ENSG00000158526	5,279017	0,409475	0,014874	0,050984	2,707036	protein_coding	TSR2	TSR2 ribosome maturation factor [Source:HGNC Symbol;Acc:HGNC:25455]	X
ENSG00000110583	4,807008	0,514097	0,014901	0,051042	2,706172	protein_coding	NAA40	N-alpha-acetyltransferase 40, NatD catalytic subunit [Source:HGNC Symbol;Acc:HGNC:25845]	11
ENSG00000167220	3,562042	0,607147	0,014934	0,051143	2,705126	protein_coding	HDHD2	haloacid dehalogenase like hydrolase domain containing 2 [Source:HGNC Symbol;Acc:HGNC:25364]	18
ENSG00000171793	5,4639	-0,598878	0,014938	0,051148	-2,704975	protein_coding	CTPS1	CTP synthase 1 [Source:HGNC Symbol;Acc:HGNC:2519]	1
ENSG00000183615	0,047299	1,335598	0,01497	0,051245	2,703975	protein_coding	FAM167B	family with sequence similarity 167 member B [Source:HGNC Symbol;Acc:HGNC:28133]	1
ENSG00000142449	-0,009909	3,650132	0,014975	0,051251	2,703811	protein_coding	FBN3	fibrillin 3 [Source:HGNC Symbol;Acc:HGNC:18794]	19
ENSG00000011007	6,598482	-0,499589	0,014991	0,051296	-2,703294	protein_coding	ELOA	elongin A [Source:HGNC Symbol;Acc:HGNC:11620]	1
ENSG00000162779	-1,207239	1,531714	0,015007	0,05133	2,702794	protein_coding	AXDND1	axonemal dynein light chain domain containing 1 [Source:HGNC Symbol;Acc:HGNC:26564]	1
ENSG00000157856	-0,804573	2,127989	0,015007	0,05133	2,70278	protein_coding	DRC1	dynein regulatory complex subunit 1 [Source:HGNC Symbol;Acc:HGNC:24245]	2
ENSG00000122735	0,022861	2,884891	0,01502	0,051363	2,702372	protein_coding	DNAI1	dynein axonemal intermediate chain 1 [Source:HGNC Symbol;Acc:HGNC:2954]	9
ENSG00000100100	4,602783	1,103696	0,015023	0,051363	2,702265	protein_coding	PIK3IP1	phosphoinositide-3-kinase interacting protein 1 [Source:HGNC Symbol;Acc:HGNC:24942]	22
ENSG00000162882	0,928413	1,394001	0,015043	0,051414	2,701638	protein_coding	HAAO	3-hydroxyanthranilate 3,4-dioxygenase [Source:HGNC Symbol;Acc:HGNC:4796]	2
ENSG00000167711	1,558462	3,138022	0,015045	0,051414	2,701589	protein_coding	SERPINF2	serpin family F member 2 [Source:HGNC Symbol;Acc:HGNC:9075]	17
ENSG00000143771	4,334817	-0,497094	0,015081	0,051515	-2,700451	protein_coding	CNIH4	cornichon family AMPA receptor auxiliary protein 4 [Source:HGNC Symbol;Acc:HGNC:25013]	1
ENSG00000112679	4,667998	1,048723	0,01508	0,051515	2,700464	protein_coding	DUSP22	dual specificity phosphatase 22 [Source:HGNC Symbol;Acc:HGNC:16077]	6
ENSG00000134824	6,643915	-1,317295	0,015112	0,051613	-2,699444	protein_coding	FADS2	fatty acid desaturase 2 [Source:HGNC Symbol;Acc:HGNC:3575]	11
ENSG00000118503	5,065969	1,171971	0,015116	0,051614	2,699337	protein_coding	TNFAIP3	TNF alpha induced protein 3 [Source:HGNC Symbol;Acc:HGNC:11896]	6
ENSG00000138777	5,237419	-0,481377	0,015127	0,051643	-2,698968	protein_coding	PPA2	inorganic pyrophosphatase 2 [Source:HGNC Symbol;Acc:HGNC:28883]	4
ENSG00000102098	2,768251	1,345234	0,01514	0,051674	2,69858	protein_coding	SCML2	Scm polycomb group protein like 2 [Source:HGNC Symbol;Acc:HGNC:10581]	X
ENSG00000188672	-1,876563	1,481942	0,015168	0,051745	2,697696	protein_coding	RHCE	Rh blood group CcEe antigens [Source:HGNC Symbol;Acc:HGNC:10008]	1
ENSG00000172673	-1,225074	2,247611	0,01517	0,051745	2,697621	protein_coding	THEMIS	thymocyte selection associated [Source:HGNC Symbol;Acc:HGNC:21569]	6
ENSG00000185864	3,628799	0,795373	0,01517	0,051745	2,69763	protein_coding	NPIPB4	nuclear pore complex interacting protein family member B4 [Source:HGNC Symbol;Acc:HGNC:41985]	16
ENSG00000085662	5,356468	-1,349961	0,015176	0,051754	-2,697444	protein_coding	AKR1B1	aldo-keto reductase family 1 member B [Source:HGNC Symbol;Acc:HGNC:381]	7
ENSG00000130939	6,273383	-0,390205	0,015183	0,051769	-2,697204	protein_coding	UBE4B	ubiquitination factor E4B [Source:HGNC Symbol;Acc:HGNC:12500]	1
ENSG00000183426	2,532391	-0,722315	0,01519	0,05178	-2,697004	protein_coding	NPIPA1	nuclear pore complex interacting protein family member A1 [Source:HGNC Symbol;Acc:HGNC:7909]	16
ENSG00000099849	3,816268	1,011536	0,0152	0,051802	2,696692	protein_coding	RASSF7	Ras association domain family member 7 [Source:HGNC Symbol;Acc:HGNC:1166]	11
ENSG00000121067	5,600399	0,405323	0,015216	0,051846	2,69619	protein_coding	SPOP	speckle type BTB/POZ protein [Source:HGNC Symbol;Acc:HGNC:11254]	17
ENSG00000132002	7,075835	0,855299	0,015225	0,051869	2,695882	protein_coding	DNAJB1	DnaJ heat shock protein family (Hsp40) member B1 [Source:HGNC Symbol;Acc:HGNC:5270]	19
ENSG00000110536	2,893775	-0,509753	0,015234	0,051888	-2,695605	protein_coding	PTPMT1	protein tyrosine phosphatase mitochondrial 1 [Source:HGNC Symbol;Acc:HGNC:26965]	11
ENSG00000136883	3,256376	3,190509	0,015254	0,051943	2,695	protein_coding	KIF12	kinesin family member 12 [Source:HGNC Symbol;Acc:HGNC:21495]	9
ENSG00000188039	-1,29272	3,089096	0,015265	0,051971	2,694638	protein_coding	NWD1	NACHT and WD repeat domain containing 1 [Source:HGNC Symbol;Acc:HGNC:27619]	19
ENSG00000071626	6,557949	0,350571	0,01527	0,051977	2,694482	protein_coding	DAZAP1	DAZ associated protein 1 [Source:HGNC Symbol;Acc:HGNC:2683]	19
ENSG00000125901	5,704072	0,5165	0,015274	0,051981	2,694348	protein_coding	MRPS26	mitochondrial ribosomal protein S26 [Source:HGNC Symbol;Acc:HGNC:14045]	20
ENSG00000066654	5,577555	-0,463385	0,015278	0,051981	-2,694243	protein_coding	THUMPD1	THUMP domain containing 1 [Source:HGNC Symbol;Acc:HGNC:23807]	16
ENSG00000173218	4,214599	-1,144205	0,015288	0,051996	-2,693909	protein_coding	VANGL1	VANGL planar cell polarity protein 1 [Source:HGNC Symbol;Acc:HGNC:15512]	1
ENSG00000073464	4,471902	0,912735	0,015286	0,051996	2,693976	protein_coding	CLCN4	chloride voltage-gated channel 4 [Source:HGNC Symbol;Acc:HGNC:2022]	X
ENSG00000126070	5,998712	0,468617	0,015294	0,052003	2,693745	protein_coding	AGO3	argonaute RISC catalytic component 3 [Source:HGNC Symbol;Acc:HGNC:18421]	1
ENSG00000114353	7,886648	-0,485997	0,015305	0,052032	-2,693377	protein_coding	GNAI2	G protein subunit alpha i2 [Source:HGNC Symbol;Acc:HGNC:4385]	3
ENSG00000185988	-1,880651	2,085481	0,015318	0,052064	2,692983	protein_coding	PLK5	polo like kinase 5 (inactive) [Source:HGNC Symbol;Acc:HGNC:27001]	19
ENSG00000126500	-2,300281	2,21746	0,015354	0,052148	2,69185	protein_coding	FLRT1	fibronectin leucine rich transmembrane protein 1 [Source:HGNC Symbol;Acc:HGNC:3760]	11
ENSG00000115850	-0,746865	1,6359	0,015359	0,052148	2,691713	protein_coding	LCT	lactase [Source:HGNC Symbol;Acc:HGNC:6530]	2
ENSG00000222036	0,081097	2,713374	0,01535	0,052148	2,691998	protein_coding	POTEM	POTE ankyrin domain family member M [Source:HGNC Symbol;Acc:HGNC:37096]	14
ENSG00000116014	0,100891	2,603977	0,015358	0,052148	2,691752	protein_coding	KISS1R	KISS1 receptor [Source:HGNC Symbol;Acc:HGNC:4510]	19
ENSG00000197584	0,447929	1,530242	0,01535	0,052148	2,691982	protein_coding	KCNMB2	potassium calcium-activated channel subfamily M regulatory beta subunit 2 [Source:HGNC Symbol;Acc:HGNC:6286]	3
ENSG00000166272	5,69617	-0,669728	0,015378	0,05219	-2,69113	protein_coding	WBP1L	WW domain binding protein 1 like [Source:HGNC Symbol;Acc:HGNC:23510]	10
ENSG00000069020	6,610624	0,646362	0,015376	0,05219	2,691182	protein_coding	MAST4	microtubule associated serine/threonine kinase family member 4 [Source:HGNC Symbol;Acc:HGNC:19037]	5
ENSG00000131044	-0,868697	2,269722	0,015388	0,052204	2,690803	protein_coding	TTLL9	tubulin tyrosine ligase like 9 [Source:HGNC Symbol;Acc:HGNC:16118]	20
ENSG00000155542	2,024103	-0,767364	0,015387	0,052204	-2,690825	protein_coding	SETD9	SET domain containing 9 [Source:HGNC Symbol;Acc:HGNC:28508]	5
ENSG00000169884	-0,562345	2,043207	0,015409	0,05222	2,690152	protein_coding	WNT10B	Wnt family member 10B [Source:HGNC Symbol;Acc:HGNC:12775]	12
ENSG00000137273	1,634841	-1,302989	0,015408	0,05222	-2,690176	protein_coding	FOXF2	forkhead box F2 [Source:HGNC Symbol;Acc:HGNC:3810]	6
ENSG00000175581	3,908822	-0,583632	0,015404	0,05222	-2,690318	protein_coding	MRPL48	mitochondrial ribosomal protein L48 [Source:HGNC Symbol;Acc:HGNC:16653]	11

ENSG00000171298	6,256495	0,624031	0,015401	0,05222	2,690414	protein_coding	GAA	glucosidase alpha, acid [Source:HGNC Symbol;Acc:HGNC:4065]	17
ENSG00000166411	6,153411	-0,580698	0,015407	0,05222	-2,69022	protein_coding	IDH3A	isocitrate dehydrogenase (NAD(+)) 3 catalytic subunit alpha [Source:HGNC Symbol;Acc:HGNC:5384]	15
ENSG00000187867	-0,073755	1,67926	0,015438	0,052307	2,689256	protein_coding	PALM3	paralemmin 3 [Source:HGNC Symbol;Acc:HGNC:33274]	19
ENSG00000101162	-0,039107	2,85468	0,015445	0,052319	2,68905	protein_coding	TUBB1	tubulin beta 1 class VI [Source:HGNC Symbol;Acc:HGNC:16257]	20
ENSG00000103066	4,133506	-0,762943	0,015452	0,052325	-2,688829	protein_coding	PLA2G15	phospholipase A2 group XV [Source:HGNC Symbol;Acc:HGNC:17163]	16
ENSG00000146963	4,630599	0,564956	0,015453	0,052325	2,688798	protein_coding	LUC7L2	LUC7 like 2, pre-mRNA splicing factor [Source:HGNC Symbol;Acc:HGNC:21608]	7
ENSG00000243725	1,699085	-0,702992	0,015459	0,052334	-2,688611	protein_coding	TTC4	tetratricopeptide repeat domain 4 [Source:HGNC Symbol;Acc:HGNC:12394]	1
ENSG00000130822	-0,498582	3,592691	0,01547	0,052362	2,688258	protein_coding	PNCK	pregnancy up-regulated nonubiquitous CaM kinase [Source:HGNC Symbol;Acc:HGNC:13415]	X
ENSG00000018610	4,353959	-0,463388	0,015475	0,052366	-2,688119	protein_coding	CXorf56	chromosome X open reading frame 56 [Source:HGNC Symbol;Acc:HGNC:26239]	X
ENSG00000188868	2,061507	0,678492	0,015488	0,052402	2,687696	protein_coding	ZNF563	zinc finger protein 563 [Source:HGNC Symbol;Acc:HGNC:30498]	19
ENSG00000154721	1,12216	2,056063	0,015497	0,052408	2,687441	protein_coding	JAM2	junctional adhesion molecule 2 [Source:HGNC Symbol;Acc:HGNC:14686]	21
ENSG00000131788	5,338228	-0,481005	0,015494	0,052408	-2,687523	protein_coding	PIAS3	protein inhibitor of activated STAT 3 [Source:HGNC Symbol;Acc:HGNC:16861]	1
ENSG00000176697	2,076311	-1,900544	0,015505	0,052413	-2,68719	protein_coding	BDNF	brain derived neurotrophic factor [Source:HGNC Symbol;Acc:HGNC:1033]	11
ENSG00000134440	7,675781	-0,63227	0,015504	0,052413	-2,687213	protein_coding	NARS1	asparaginyl-tRNA synthetase 1 [Source:HGNC Symbol;Acc:HGNC:7643]	18
ENSG00000122678	4,373258	0,768631	0,01552	0,052453	2,686733	protein_coding	POLM	DNA polymerase mu [Source:HGNC Symbol;Acc:HGNC:9185]	7
ENSG00000188766	4,119627	-1,105429	0,015525	0,052456	-2,686575	protein_coding	SPRED3	sprouty related EVH1 domain containing 3 [Source:HGNC Symbol;Acc:HGNC:31041]	19
ENSG00000074416	5,574528	-1,130465	0,015527	0,052456	-2,686508	protein_coding	MGLL	monoglyceride lipase [Source:HGNC Symbol;Acc:HGNC:17038]	3
ENSG00000105202	6,021599	0,832358	0,015539	0,052487	2,686122	protein_coding	FBL	fibrillarin [Source:HGNC Symbol;Acc:HGNC:3599]	19
ENSG00000135916	6,44827	-0,813158	0,015556	0,052531	-2,685624	protein_coding	ITM2C	integral membrane protein 2C [Source:HGNC Symbol;Acc:HGNC:6175]	2
ENSG00000106245	5,680735	-0,455648	0,015565	0,052553	-2,685323	protein_coding	BUD31	BUD31 homolog [Source:HGNC Symbol;Acc:HGNC:29629]	7
ENSG00000100979	4,799104	1,630827	0,015608	0,052686	2,684016	protein_coding	PLTP	phospholipid transfer protein [Source:HGNC Symbol;Acc:HGNC:9093]	20
ENSG00000173261	-1,029591	1,424874	0,015612	0,052689	2,683884	protein_coding	PLAC8L1	PLAC8 like 1 [Source:HGNC Symbol;Acc:HGNC:31746]	5
ENSG00000206559	-0,057352	1,088566	0,01562	0,052691	2,683657	protein_coding	ZCWPW2	zinc finger CW-type and PWWP domain containing 2 [Source:HGNC Symbol;Acc:HGNC:23574]	3
ENSG00000124067	5,919186	-0,952668	0,015616	0,052691	-2,683757	protein_coding	SLC12A4	solute carrier family 12 member 4 [Source:HGNC Symbol;Acc:HGNC:10913]	16
ENSG00000063978	5,936618	-0,442224	0,015622	0,052691	-2,683573	protein_coding	RNF4	ring finger protein 4 [Source:HGNC Symbol;Acc:HGNC:10067]	4
ENSG00000182481	6,453549	-0,626939	0,015642	0,052746	-2,68297	protein_coding	KPNA2	karyopherin subunit alpha 2 [Source:HGNC Symbol;Acc:HGNC:6395]	17
ENSG00000155066	0,149428	1,678099	0,015649	0,052758	2,682761	protein_coding	PROM2	prominin 2 [Source:HGNC Symbol;Acc:HGNC:20685]	2
ENSG00000187672	2,893723	2,30076	0,015664	0,052797	2,682307	protein_coding	ERC2	ELKS/RAB6-interacting/CAST family member 2 [Source:HGNC Symbol;Acc:HGNC:31922]	3
ENSG00000105695	-2,65795	3,234852	0,015693	0,052853	2,681427	protein_coding	MAG	myelin associated glycoprotein [Source:HGNC Symbol;Acc:HGNC:6783]	19
ENSG00000171885	-1,458604	2,645098	0,015693	0,052853	2,68141	protein_coding	AQP4	aquaporin 4 [Source:HGNC Symbol;Acc:HGNC:637]	18
ENSG00000120526	4,670721	-0,448838	0,015691	0,052853	-2,681461	protein_coding	NUDCD1	NudC domain containing 1 [Source:HGNC Symbol;Acc:HGNC:24306]	8
ENSG00000047578	5,259991	0,350944	0,015691	0,052853	2,681489	protein_coding	KATNIP	katanin interacting protein [Source:HGNC Symbol;Acc:HGNC:29068]	16
ENSG00000158711	6,392142	-0,653847	0,015709	0,052895	-2,680923	protein_coding	ELK4	ETS transcription factor ELK4 [Source:HGNC Symbol;Acc:HGNC:3326]	1
ENSG00000262209	2,489027	1,630901	0,015732	0,05296	2,680242	protein_coding	PCDHGB3	protocadherin gamma subfamily B, 3 [Source:HGNC Symbol;Acc:HGNC:8710]	5
ENSG00000215475	-1,597871	2,292005	0,015741	0,052971	2,679943	protein_coding	SIAH3	siah E3 ubiquitin protein ligase family member 3 [Source:HGNC Symbol;Acc:HGNC:30553]	13
ENSG00000181626	-0,625176	2,953365	0,015745	0,052971	2,679846	protein_coding	ANKRD62	ankyrin repeat domain 62 [Source:HGNC Symbol;Acc:HGNC:35241]	18
ENSG00000105677	4,70648	-0,537339	0,015745	0,052971	-2,679842	protein_coding	TMEM147	transmembrane protein 147 [Source:HGNC Symbol;Acc:HGNC:30414]	19
ENSG00000154727	5,336816	-0,647456	0,015804	0,053161	-2,67803	protein_coding	GABPA	GA binding protein transcription factor subunit alpha [Source:HGNC Symbol;Acc:HGNC:4071]	21
ENSG00000196678	3,497194	-0,628711	0,015833	0,053243	-2,677151	protein_coding	ERI2	ERI1 exoribonuclease family member 2 [Source:HGNC Symbol;Acc:HGNC:30541]	16
ENSG00000156928	4,172573	-0,519981	0,015835	0,053243	-2,67709	protein_coding	MALSU1	mitochondrial assembly of ribosomal large subunit 1 [Source:HGNC Symbol;Acc:HGNC:21721]	7
ENSG00000135926	6,210977	-0,918322	0,015845	0,053264	-2,676802	protein_coding	TMBIM1	transmembrane BAX inhibitor motif containing 1 [Source:HGNC Symbol;Acc:HGNC:23410]	2
ENSG00000197746	10,06216	-0,53051	0,015855	0,053286	-2,676512	protein_coding	PSAP	prosaposin [Source:HGNC Symbol;Acc:HGNC:9498]	10
ENSG00000076067	5,561572	-0,992891	0,015861	0,053298	-2,676306	protein_coding	RBMS2	RNA binding motif single stranded interacting protein 2 [Source:HGNC Symbol;Acc:HGNC:9909]	12
ENSG00000155011	1,875493	-2,652851	0,015882	0,053355	-2,675695	protein_coding	DKK2	dickkopf WNT signaling pathway inhibitor 2 [Source:HGNC Symbol;Acc:HGNC:2892]	4
ENSG00000107443	3,396637	-0,658228	0,015892	0,053378	-2,675387	protein_coding	CCNJ	cyclin J [Source:HGNC Symbol;Acc:HGNC:23434]	10
ENSG00000140481	0,605792	2,161949	0,015909	0,053425	2,674867	protein_coding	CCDC33	coiled-coil domain containing 33 [Source:HGNC Symbol;Acc:HGNC:26552]	15
ENSG00000205436	0,590965	2,139369	0,015931	0,053477	2,674206	protein_coding	EXOC3L4	exocyst complex component 3 like 4 [Source:HGNC Symbol;Acc:HGNC:20120]	14
ENSG00000169733	4,404411	0,424116	0,015931	0,053477	2,674219	protein_coding	RFNG	RFNG O-fucosylpeptide 3-beta-N-acetylglucosaminyltransferase [Source:HGNC Symbol;Acc:HGNC:9974]	17
ENSG00000121361	2,388896	1,997372	0,015943	0,053505	2,673855	protein_coding	KCNJ8	potassium inwardly rectifying channel subfamily J member 8 [Source:HGNC Symbol;Acc:HGNC:6269]	12
ENSG00000188375	-1,133015	1,22771	0,01595	0,053519	2,673631	protein_coding	H3-5	H3.5 histone [Source:HGNC Symbol;Acc:HGNC:33164]	12
ENSG00000037757	4,610343	0,56344	0,015961	0,053536	2,673304	protein_coding	MRI1	methylthioribose-1-phosphate isomerase 1 [Source:HGNC Symbol;Acc:HGNC:28469]	19

ENSG00000211584	5,085284	-0,657845	0,015962	0,053536	-2,67328	protein_coding	SLC48A1	solute carrier family 48 member 1 [Source:HGNC Symbol;Acc:HGNC:26035]	12
ENSG00000136542	3,629518	-2,257112	0,015971	0,053555	-2,673011	protein_coding	GALNT5	polypeptide N-acetylgalactosaminyltransferase 5 [Source:HGNC Symbol;Acc:HGNC:4127]	2
ENSG00000171643	-1,047054	2,239599	0,016004	0,053632	2,672029	protein_coding	S100Z	S100 calcium binding protein Z [Source:HGNC Symbol;Acc:HGNC:30367]	5
ENSG00000168490	0,61193	1,237425	0,016003	0,053632	2,672061	protein_coding	PHYHIP	phytanoyl-CoA 2-hydroxylase interacting protein [Source:HGNC Symbol;Acc:HGNC:16865]	8
ENSG00000128159	6,081634	0,567009	0,016	0,053632	2,672153	protein_coding	TUBGCP6	tubulin gamma complex associated protein 6 [Source:HGNC Symbol;Acc:HGNC:18127]	22
ENSG00000163909	5,32151	0,73551	0,016015	0,053657	2,671703	protein_coding	HEYL	hes related family bHLH transcription factor with YRPW motif like [Source:HGNC Symbol;Acc:HGNC:4882]	1
ENSG00000120685	5,617283	-0,612585	0,016029	0,053694	-2,671278	protein_coding	PROSER1	proline and serine rich 1 [Source:HGNC Symbol;Acc:HGNC:20291]	13
ENSG00000132334	5,713201	-0,855732	0,016045	0,053738	-2,670781	protein_coding	PTPRE	protein tyrosine phosphatase receptor type E [Source:HGNC Symbol;Acc:HGNC:9669]	10
ENSG00000076344	2,102417	1,895597	0,016049	0,05374	2,67067	protein_coding	RGS11	regulator of G protein signaling 11 [Source:HGNC Symbol;Acc:HGNC:9993]	16
ENSG00000083937	5,518997	-0,637124	0,016055	0,053748	-2,670501	protein_coding	CHMP2B	charged multivesicular body protein 2B [Source:HGNC Symbol;Acc:HGNC:24537]	3
ENSG00000159479	4,890286	-0,438058	0,016062	0,053759	-2,670296	protein_coding	MED8	mediator complex subunit 8 [Source:HGNC Symbol;Acc:HGNC:19971]	1
ENSG00000090539	3,4164	1,559371	0,016072	0,053784	2,669975	protein_coding	CHRD	chordin [Source:HGNC Symbol;Acc:HGNC:1949]	3
ENSG00000188636	6,168932	-0,493324	0,016088	0,053826	-2,669507	protein_coding	RTL6	retrotransposon Gag like 6 [Source:HGNC Symbol;Acc:HGNC:13343]	22
ENSG00000004838	0,086638	2,020542	0,016101	0,053847	2,669117	protein_coding	ZMYND10	zinc finger MYND-type containing 10 [Source:HGNC Symbol;Acc:HGNC:19412]	3
ENSG00000171492	4,734621	-0,590289	0,0161	0,053847	-2,669161	protein_coding	LRRC8D	leucine rich repeat containing 8 VRAC subunit D [Source:HGNC Symbol;Acc:HGNC:16992]	1
ENSG00000189319	4,80094	0,699728	0,016113	0,053875	2,668776	protein_coding	FAM53B	family with sequence similarity 53 member B [Source:HGNC Symbol;Acc:HGNC:28968]	10
ENSG00000139445	-1,579662	3,284957	0,016127	0,053899	2,66836	protein_coding	FOXN4	forkhead box N4 [Source:HGNC Symbol;Acc:HGNC:21399]	12
ENSG00000167447	4,535753	-0,602526	0,016126	0,053899	-2,668368	protein_coding	SMG8	SMG8 nonsense mediated mRNA decay factor [Source:HGNC Symbol;Acc:HGNC:25551]	17
ENSG00000171533	3,587407	1,603352	0,016146	0,053938	2,667772	protein_coding	MAP6	microtubule associated protein 6 [Source:HGNC Symbol;Acc:HGNC:6868]	11
ENSG00000228253	10,27834	-1,021495	0,016148	0,053938	-2,667717	protein_coding	MT-ATP8	mitochondrially encoded ATP synthase membrane subunit 8 [Source:HGNC Symbol;Acc:HGNC:7415]	MT
ENSG00000123395	5,11671	-0,541977	0,016145	0,053938	-2,667813	protein_coding	ATG101	autophagy related 101 [Source:HGNC Symbol;Acc:HGNC:25679]	12
ENSG00000180245	-1,426814	1,347336	0,016169	0,053997	2,667095	protein_coding	RRH	retinal pigment epithelium-derived rhodopsin homolog [Source:HGNC Symbol;Acc:HGNC:10450]	4
ENSG00000162300	4,131501	-0,378134	0,016192	0,054039	-2,666429	protein_coding	ZFPL1	zinc finger protein like 1 [Source:HGNC Symbol;Acc:HGNC:12868]	11
ENSG00000187210	4,646302	1,308829	0,016189	0,054039	2,666515	protein_coding	GCNT1	glucosaminyl (N-acetyl) transferase 1 [Source:HGNC Symbol;Acc:HGNC:4203]	9
ENSG00000129473	5,592181	0,53128	0,016192	0,054039	2,666428	protein_coding	BCL2L2	BCL2 like 2 [Source:HGNC Symbol;Acc:HGNC:995]	14
ENSG00000100151	4,540854	0,595968	0,016221	0,054127	2,665553	protein_coding	PICK1	protein interacting with PRKCA 1 [Source:HGNC Symbol;Acc:HGNC:9394]	22
ENSG00000042317	3,335265	0,815965	0,016228	0,054138	2,665355	protein_coding	SPATA7	spermatogenesis associated 7 [Source:HGNC Symbol;Acc:HGNC:20423]	14
ENSG00000146966	3,363132	1,965904	0,016235	0,054149	2,665158	protein_coding	DENND2A	DENN domain containing 2A [Source:HGNC Symbol;Acc:HGNC:22212]	7
ENSG00000128285	-0,940033	1,582062	0,016239	0,054152	2,665033	protein_coding	MCHR1	melanin concentrating hormone receptor 1 [Source:HGNC Symbol;Acc:HGNC:4479]	22
ENSG00000118655	3,348391	-0,614143	0,016245	0,054161	-2,664854	protein_coding	DCLRE1B	DNA cross-link repair 1B [Source:HGNC Symbol;Acc:HGNC:17641]	1
ENSG00000186496	1,809725	0,956358	0,016263	0,05421	2,664327	protein_coding	ZNF396	zinc finger protein 396 [Source:HGNC Symbol;Acc:HGNC:18824]	18
ENSG00000182040	-3,187843	1,893738	0,01628	0,054254	2,663822	protein_coding	USH1G	USH1 protein network component sans [Source:HGNC Symbol;Acc:HGNC:16356]	17
ENSG00000273899	1,478717	0,770933	0,016283	0,054254	2,663742	protein_coding	NOL12	nucleolar protein 12 [Source:HGNC Symbol;Acc:HGNC:28585]	22
ENSG00000070985	-0,285111	2,198247	0,016311	0,054335	2,662923	protein_coding	TRPM5	transient receptor potential cation channel subfamily M member 5 [Source:HGNC Symbol;Acc:HGNC:14323]	11
ENSG00000161970	6,852089	1,003586	0,016317	0,054346	2,662733	protein_coding	RPL26	ribosomal protein L26 [Source:HGNC Symbol;Acc:HGNC:10327]	17
ENSG00000155093	6,699987	1,270338	0,016344	0,054424	2,661947	protein_coding	PTPRN2	protein tyrosine phosphatase receptor type N2 [Source:HGNC Symbol;Acc:HGNC:9677]	7
ENSG00000143799	7,626356	0,727723	0,016351	0,054437	2,661731	protein_coding	PARP1	poly(ADP-ribose) polymerase 1 [Source:HGNC Symbol;Acc:HGNC:270]	1
ENSG00000167895	1,824425	2,493831	0,016365	0,054471	2,661336	protein_coding	TMC8	transmembrane channel like 8 [Source:HGNC Symbol;Acc:HGNC:20474]	17
ENSG00000183508	4,776587	1,889537	0,016371	0,054481	2,661152	protein_coding	TENT5C	terminal nucleotidyltransferase 5C [Source:HGNC Symbol;Acc:HGNC:24712]	1
ENSG00000148362	4,219896	0,720365	0,0164	0,054567	2,660292	protein_coding	PAXX	PAXX non-homologous end joining factor [Source:HGNC Symbol;Acc:HGNC:27849]	9
ENSG00000232388	2,64548	-0,620336	0,016435	0,05467	-2,659295	protein_coding	SMIM26	small integral membrane protein 26 [Source:HGNC Symbol;Acc:HGNC:43430]	20
ENSG00000152049	4,9037	-1,069435	0,016444	0,054689	-2,65903	protein_coding	KCNE4	potassium voltage-gated channel subfamily E regulatory subunit 4 [Source:HGNC Symbol;Acc:HGNC:6244]	2
ENSG00000166169	4,285989	0,443427	0,016465	0,05475	2,658394	protein_coding	POLL	DNA polymerase lambda [Source:HGNC Symbol;Acc:HGNC:9184]	10
ENSG00000159167	6,071411	-2,101693	0,016483	0,054799	-2,657869	protein_coding	STC1	stanniocalcin 1 [Source:HGNC Symbol;Acc:HGNC:11373]	8
ENSG00000111652	5,37232	-0,454607	0,016537	0,054954	-2,656306	protein_coding	COPS7A	COP9 signalosome subunit 7A [Source:HGNC Symbol;Acc:HGNC:16758]	12
ENSG00000105397	6,500347	0,459296	0,01654	0,054954	2,656222	protein_coding	TYK2	tyrosine kinase 2 [Source:HGNC Symbol;Acc:HGNC:12440]	19
ENSG00000171604	6,507756	-0,68015	0,016538	0,054954	-2,656278	protein_coding	CXXC5	CXXC finger protein 5 [Source:HGNC Symbol;Acc:HGNC:26943]	5
ENSG00000153157	-1,210863	2,36065	0,01655	0,054971	2,655949	protein_coding	SYCP2L	synaptonemal complex protein 2 like [Source:HGNC Symbol;Acc:HGNC:21537]	6
ENSG00000116983	0,512623	2,205222	0,016553	0,054971	2,655854	protein_coding	HPCAL4	hippocalcin like 4 [Source:HGNC Symbol;Acc:HGNC:18212]	1
ENSG00000049089	1,80309	1,028169	0,016556	0,054971	2,655778	protein_coding	COL9A2	collagen type IX alpha 2 chain [Source:HGNC Symbol;Acc:HGNC:2218]	1
ENSG00000100591	6,750726	-0,374493	0,016579	0,055039	-2,655089	protein_coding	AHSA1	activator of HSP90 ATPase activity 1 [Source:HGNC Symbol;Acc:HGNC:1189]	14

ENSG00000072135	6,310741	0,5529	0,016584	0,055043	2,654956	protein_coding	PTPN18	protein tyrosine phosphatase non-receptor type 18 [Source:HGNC Symbol;Acc:HGNC:9649]	2
ENSG00000176563	-0,951425	1,44385	0,016588	0,055044	2,654847	protein_coding	CNTD1	cyclin N-terminal domain containing 1 [Source:HGNC Symbol;Acc:HGNC:26847]	17
ENSG00000111817	5,725613	-1,017571	0,016592	0,055047	-2,654725	protein_coding	DSE	dermatan sulfate epimerase [Source:HGNC Symbol;Acc:HGNC:21144]	6
ENSG00000173226	4,372135	0,717823	0,016612	0,055101	2,654151	protein_coding	IQCB1	IQ motif containing B1 [Source:HGNC Symbol;Acc:HGNC:28949]	3
ENSG00000108830	0,435934	2,648863	0,016632	0,055152	2,653566	protein_coding	RND2	Rho family GTPase 2 [Source:HGNC Symbol;Acc:HGNC:18315]	17
ENSG00000011198	4,764446	-0,536005	0,016634	0,055152	-2,653516	protein_coding	ABHD5	abhydrolase domain containing 5, lysophosphatidic acid acyltransferase [Source:HGNC Symbol;Acc:HGNC:21396]	3
ENSG00000147121	3,295968	0,584136	0,016642	0,055157	2,653276	protein_coding	KRBOX4	KRAB box domain containing 4 [Source:HGNC Symbol;Acc:HGNC:26007]	X
ENSG00000127586	4,22438	0,935758	0,01664	0,055157	2,653344	protein_coding	CHTF18	chromosome transmission fidelity factor 18 [Source:HGNC Symbol;Acc:HGNC:18435]	16
ENSG00000164031	5,489945	-0,452674	0,016654	0,055184	-2,65294	protein_coding	DNAJB14	DnaJ heat shock protein family (Hsp40) member B14 [Source:HGNC Symbol;Acc:HGNC:25881]	4
ENSG00000136869	5,203981	-0,856628	0,016673	0,055237	-2,652383	protein_coding	TLR4	toll like receptor 4 [Source:HGNC Symbol;Acc:HGNC:11850]	9
ENSG00000029559	0,857745	2,2379	0,016695	0,055277	2,651745	protein_coding	IBSP	integrin binding sialoprotein [Source:HGNC Symbol;Acc:HGNC:5341]	4
ENSG00000186352	2,568604	0,979061	0,016694	0,055277	2,651778	protein_coding	ANKRD37	ankyrin repeat domain 37 [Source:HGNC Symbol;Acc:HGNC:29593]	4
ENSG00000105072	3,418789	0,823918	0,016691	0,055277	2,651877	protein_coding	C19orf44	chromosome 19 open reading frame 44 [Source:HGNC Symbol;Acc:HGNC:26141]	19
ENSG00000177272	0,213707	2,432326	0,0167	0,055282	2,651599	protein_coding	KCNA3	potassium voltage-gated channel subfamily A member 3 [Source:HGNC Symbol;Acc:HGNC:6221]	1
ENSG00000158050	1,287331	1,61849	0,016731	0,055363	2,650711	protein_coding	DUSP2	dual specificity phosphatase 2 [Source:HGNC Symbol;Acc:HGNC:3068]	2
ENSG00000176208	4,356983	1,031713	0,016732	0,055363	2,650701	protein_coding	ATAD5	ATPase family AAA domain containing 5 [Source:HGNC Symbol;Acc:HGNC:25752]	17
ENSG00000186567	2,881341	1,788414	0,016747	0,05538	2,650267	protein_coding	CEACAM19	CEA cell adhesion molecule 19 [Source:HGNC Symbol;Acc:HGNC:31951]	19
ENSG00000175782	4,95622	0,536101	0,016747	0,05538	2,650259	protein_coding	SLC35E3	solute carrier family 35 member E3 [Source:HGNC Symbol;Acc:HGNC:20864]	12
ENSG00000112655	7,073818	-1,108239	0,016741	0,05538	-2,650428	protein_coding	PTK7	protein tyrosine kinase 7 (inactive) [Source:HGNC Symbol;Acc:HGNC:9618]	6
ENSG00000176884	1,673652	2,500245	0,016777	0,055435	2,649393	protein_coding	GRIN1	glutamate ionotropic receptor NMDA type subunit 1 [Source:HGNC Symbol;Acc:HGNC:4584]	9
ENSG00000100003	3,30673	-0,943299	0,016775	0,055435	-2,649467	protein_coding	SEC14L2	SEC14 like lipid binding 2 [Source:HGNC Symbol;Acc:HGNC:10699]	22
ENSG00000130775	4,378746	1,280153	0,016777	0,055435	2,649407	protein_coding	THEMIS2	thymocyte selection associated family member 2 [Source:HGNC Symbol;Acc:HGNC:16839]	1
ENSG00000126456	5,304183	0,573169	0,016771	0,055435	2,649565	protein_coding	IRF3	interferon regulatory factor 3 [Source:HGNC Symbol;Acc:HGNC:6118]	19
ENSG00000148516	6,159037	-0,862486	0,016788	0,055459	-2,649088	protein_coding	ZEB1	zinc finger E-box binding homeobox 1 [Source:HGNC Symbol;Acc:HGNC:11642]	10
ENSG00000187942	-0,225013	1,483771	0,016794	0,055467	2,64892	protein_coding	LDLRAD2	low density lipoprotein receptor class A domain containing 2 [Source:HGNC Symbol;Acc:HGNC:32071]	1
ENSG00000187522	3,523732	-0,623121	0,016804	0,055489	-2,648637	protein_coding	HSPA14	heat shock protein family A (Hsp70) member 14 [Source:HGNC Symbol;Acc:HGNC:29526]	10
ENSG00000167380	4,608618	0,576255	0,016817	0,055522	2,648254	protein_coding	ZNF226	zinc finger protein 226 [Source:HGNC Symbol;Acc:HGNC:13019]	19
ENSG00000166311	5,301256	-0,919242	0,016822	0,055526	-2,648123	protein_coding	SMPD1	sphingomyelin phosphodiesterase 1 [Source:HGNC Symbol;Acc:HGNC:11120]	11
ENSG00000087250	-0,19341	3,135155	0,016829	0,055539	2,647909	protein_coding	MT3	metallothionein 3 [Source:HGNC Symbol;Acc:HGNC:7408]	16
ENSG00000160949	4,663407	0,805048	0,016838	0,055548	2,647653	protein_coding	TONSL	tonsoku like, DNA repair protein [Source:HGNC Symbol;Acc:HGNC:7801]	8
ENSG00000117906	6,124757	-0,716103	0,016839	0,055548	-2,647639	protein_coding	RCN2	reticulocalbin 2 [Source:HGNC Symbol;Acc:HGNC:9935]	15
ENSG00000074803	-2,264849	2,08075	0,01686	0,055579	2,647035	protein_coding	SLC12A1	solute carrier family 12 member 1 [Source:HGNC Symbol;Acc:HGNC:10910]	15
ENSG00000154710	2,636855	0,692978	0,016862	0,055579	2,646981	protein_coding	RABGEF1	RAB guanine nucleotide exchange factor 1 [Source:HGNC Symbol;Acc:HGNC:17676]	7
ENSG00000116691	3,529305	0,743382	0,016854	0,055579	2,647214	protein_coding	MIIP	migration and invasion inhibitory protein [Source:HGNC Symbol;Acc:HGNC:25715]	1
ENSG00000198833	6,408735	-0,658592	0,016858	0,055579	-2,647101	protein_coding	UBE2J1	ubiquitin conjugating enzyme E2 J1 [Source:HGNC Symbol;Acc:HGNC:17598]	6
ENSG00000168952	3,413969	1,6513	0,016882	0,055635	2,646397	protein_coding	STXBP6	syntaxin binding protein 6 [Source:HGNC Symbol;Acc:HGNC:19666]	14
ENSG00000089356	-0,224403	2,874978	0,016892	0,055654	2,646134	protein_coding	FXYD3	FXYD domain containing ion transport regulator 3 [Source:HGNC Symbol;Acc:HGNC:4027]	19
ENSG00000164338	4,469152	-0,476845	0,016897	0,055655	-2,645974	protein_coding	UTP15	UTP15 small subunit processome component [Source:HGNC Symbol;Acc:HGNC:25758]	5
ENSG00000160917	4,62752	0,586728	0,016905	0,055655	2,64576	protein_coding	CPSF4	cleavage and polyadenylation specific factor 4 [Source:HGNC Symbol;Acc:HGNC:2327]	7
ENSG00000173456	5,185617	-0,429374	0,016906	0,055655	-2,645736	protein_coding	RNF26	ring finger protein 26 [Source:HGNC Symbol;Acc:HGNC:14646]	11
ENSG00000186340	8,70216	-1,289172	0,016902	0,055655	-2,645842	protein_coding	THBS2	thrombospondin 2 [Source:HGNC Symbol;Acc:HGNC:11786]	6
ENSG00000167483	-0,144506	2,616421	0,016916	0,055656	2,645438	protein_coding	NIBAN3	niban apoptosis regulator 3 [Source:HGNC Symbol;Acc:HGNC:24130]	19
ENSG00000006210	1,865744	1,676327	0,016911	0,055656	2,645592	protein_coding	CX3CL1	C-X3-C motif chemokine ligand 1 [Source:HGNC Symbol;Acc:HGNC:10647]	16
ENSG00000089091	2,709196	1,014479	0,016913	0,055656	2,645527	protein_coding	DZANK1	double zinc ribbon and ankyrin repeat domains 1 [Source:HGNC Symbol;Acc:HGNC:15858]	20
ENSG00000115841	2,767978	-0,888628	0,016932	0,055696	-2,644997	protein_coding	RMDN2	regulator of microtubule dynamics 2 [Source:HGNC Symbol;Acc:HGNC:26567]	2
ENSG00000116786	6,488305	-0,434913	0,016939	0,055709	-2,644784	protein_coding	PLEKHM2	pleckstrin homology and RUN domain containing M2 [Source:HGNC Symbol;Acc:HGNC:29131]	1
ENSG00000233608	1,332996	-1,80636	0,016947	0,055725	-2,644555	protein_coding	TWIST2	twist family bHLH transcription factor 2 [Source:HGNC Symbol;Acc:HGNC:20670]	2
ENSG00000162572	0,878257	1,016546	0,01697	0,055787	2,643917	protein_coding	SCNN1D	sodium channel epithelial 1 subunit delta [Source:HGNC Symbol;Acc:HGNC:10601]	1
ENSG00000128059	4,003982	-0,450945	0,017005	0,055894	-2,642907	protein_coding	PPAT	phosphoribosyl pyrophosphate amidotransferase [Source:HGNC Symbol;Acc:HGNC:9238]	4
ENSG00000154783	3,535424	1,578119	0,017012	0,055906	2,642708	protein_coding	FGD5	FYVE, RhoGEF and PH domain containing 5 [Source:HGNC Symbol;Acc:HGNC:19117]	3
ENSG00000137500	5,307951	-0,486981	0,017017	0,05591	-2,642569	protein_coding	CCDC90B	coiled-coil domain containing 90B [Source:HGNC Symbol;Acc:HGNC:28108]	11

ENSG00000073584	4,041944	0,492812	0,017029	0,055939	2,64223	protein_coding	SMARCE1	SWI/SNF related, matrix associated, actin dependent regulator of chromatin, subfamily e, member 1 [Source:HGNC	17
ENSG00000166986	7,130312	-0,516434	0,01705	0,055995	-2,64165	protein_coding	MARS1	methionyl-tRNA synthetase 1 [Source:HGNC Symbol;Acc:HGNC:6898]	12
ENSG00000136819	6,160177	-0,376841	0,017072	0,056055	-2,641038	protein_coding	C9orf78	chromosome 9 open reading frame 78 [Source:HGNC Symbol;Acc:HGNC:24932]	9
ENSG00000165269	0,613143	2,765498	0,017097	0,056128	2,640317	protein_coding	AQP7	aquaporin 7 [Source:HGNC Symbol;Acc:HGNC:640]	9
ENSG00000118420	3,054606	0,89776	0,01713	0,056223	2,639408	protein_coding	UBE3D	ubiquitin protein ligase E3D [Source:HGNC Symbol;Acc:HGNC:21381]	6
ENSG00000138738	3,350158	1,086695	0,017191	0,056406	2,637681	protein_coding	PRDM5	PR/SET domain 5 [Source:HGNC Symbol;Acc:HGNC:9349]	4
ENSG00000188283	3,586284	0,481985	0,017192	0,056406	2,637652	protein_coding	ZNF383	zinc finger protein 383 [Source:HGNC Symbol;Acc:HGNC:18609]	19
ENSG00000174292	2,181056	1,069943	0,017231	0,056521	2,636578	protein_coding	TNK1	tyrosine kinase non receptor 1 [Source:HGNC Symbol;Acc:HGNC:11940]	17
ENSG00000173198	-1,221814	2,128261	0,017239	0,056536	2,636353	protein_coding	CYSLTR1	cysteinyl leukotriene receptor 1 [Source:HGNC Symbol;Acc:HGNC:17451]	X
ENSG00000092140	4,639014	0,443926	0,017277	0,056648	2,635304	protein_coding	G2E3	G2/M-phase specific E3 ubiquitin protein ligase [Source:HGNC Symbol;Acc:HGNC:20338]	14
ENSG00000109189	4,302189	-0,525414	0,017314	0,05676	-2,634255	protein_coding	USP46	ubiquitin specific peptidase 46 [Source:HGNC Symbol;Acc:HGNC:20075]	4
ENSG00000129355	3,579642	1,03962	0,017332	0,056795	2,633766	protein_coding	CDKN2D	cyclin dependent kinase inhibitor 2D [Source:HGNC Symbol;Acc:HGNC:1790]	19
ENSG00000243147	4,204517	-0,442864	0,01733	0,056795	-2,633824	protein_coding	MRPL33	mitochondrial ribosomal protein L33 [Source:HGNC Symbol;Acc:HGNC:14487]	2
ENSG00000117215	-3,442491	2,683598	0,017349	0,056828	2,633294	protein_coding	PLA2G2D	phospholipase A2 group IID [Source:HGNC Symbol;Acc:HGNC:9033]	1
ENSG00000081052	3,422025	1,89309	0,017347	0,056828	2,633339	protein_coding	COL4A4	collagen type IV alpha 4 chain [Source:HGNC Symbol;Acc:HGNC:2206]	2
ENSG00000153774	5,246079	0,462047	0,01736	0,056841	2,632991	protein_coding	CFDP1	craniofacial development protein 1 [Source:HGNC Symbol;Acc:HGNC:1873]	16
ENSG00000101439	8,253817	-0,987989	0,017359	0,056841	-2,633019	protein_coding	CST3	cystatin C [Source:HGNC Symbol;Acc:HGNC:2475]	20
ENSG00000182685	1,198906	1,03592	0,01738	0,056895	2,632433	protein_coding	BRICD5	BRICHOS domain containing 5 [Source:HGNC Symbol;Acc:HGNC:28309]	16
ENSG00000232040	0,758712	2,037904	0,017384	0,056896	2,632325	protein_coding	ZBED9	zinc finger BED-type containing 9 [Source:HGNC Symbol;Acc:HGNC:13851]	6
ENSG00000247595	1,142209	1,102665	0,017389	0,0569	2,632196	protein_coding	SPTY2D1OS	SPTY2D1 opposite strand [Source:HGNC Symbol;Acc:HGNC:44122]	11
ENSG00000185681	-3,053149	2,263335	0,017397	0,056916	2,631968	protein_coding	MORN5	MORN repeat containing 5 [Source:HGNC Symbol;Acc:HGNC:17841]	9
ENSG00000158402	2,381085	1,316114	0,01741	0,056947	2,631608	protein_coding	CDC25C	cell division cycle 25C [Source:HGNC Symbol;Acc:HGNC:1727]	5
ENSG00000106571	4,223957	-1,230699	0,017429	0,056996	-2,631097	protein_coding	GLI3	GLI family zinc finger 3 [Source:HGNC Symbol;Acc:HGNC:4319]	7
ENSG00000148950	2,311918	0,79357	0,017443	0,057031	2,630708	protein_coding	IMMP1L	inner mitochondrial membrane peptidase subunit 1 [Source:HGNC Symbol;Acc:HGNC:26317]	11
ENSG00000197776	2,01506	0,925363	0,017456	0,057052	2,630338	protein_coding	KLHDC1	kelch domain containing 1 [Source:HGNC Symbol;Acc:HGNC:19836]	14
ENSG00000011566	5,895991	-0,467723	0,017456	0,057052	-2,630339	protein_coding	MAP4K3	mitogen-activated protein kinase kinase kinase kinase 3 [Source:HGNC Symbol;Acc:HGNC:6865]	2
ENSG00000174348	3,643611	1,996115	0,017493	0,057161	2,629313	protein_coding	PODN	podocan [Source:HGNC Symbol;Acc:HGNC:23174]	1
ENSG00000070087	6,021637	-0,679892	0,017497	0,057161	-2,62922	protein_coding	PFN2	profilin 2 [Source:HGNC Symbol;Acc:HGNC:8882]	3
ENSG00000183856	5,180111	1,230912	0,017602	0,057491	2,626328	protein_coding	IQGAP3	IQ motif containing GTPase activating protein 3 [Source:HGNC Symbol;Acc:HGNC:20669]	1
ENSG00000154767	5,977847	0,436479	0,017606	0,057491	2,626225	protein_coding	XPC	XPC complex subunit, DNA damage recognition and repair factor [Source:HGNC Symbol;Acc:HGNC:12816]	3
ENSG00000135932	6,414014	-0,623755	0,017608	0,057491	-2,626166	protein_coding	CAB39	calcium binding protein 39 [Source:HGNC Symbol;Acc:HGNC:20292]	2
ENSG00000131558	6,494642	0,515016	0,017615	0,057503	2,625971	protein_coding	EXOC4	exocyst complex component 4 [Source:HGNC Symbol;Acc:HGNC:30389]	7
ENSG00000081377	4,305448	-0,697047	0,017624	0,05752	-2,625726	protein_coding	CDC14B	cell division cycle 14B [Source:HGNC Symbol;Acc:HGNC:1719]	9
ENSG00000135605	2,221656	0,841971	0,017632	0,057523	2,625507	protein_coding	TEC	tec protein tyrosine kinase [Source:HGNC Symbol;Acc:HGNC:11719]	4
ENSG00000146535	6,884063	-0,440456	0,017629	0,057523	-2,625591	protein_coding	GNA12	G protein subunit alpha 12 [Source:HGNC Symbol;Acc:HGNC:4380]	7
ENSG00000132182	6,081052	1,593132	0,017636	0,057525	2,625399	protein_coding	NUP210	nucleoporin 210 [Source:HGNC Symbol;Acc:HGNC:30052]	3
ENSG00000179915	2,037357	3,195913	0,017651	0,057548	2,625009	protein_coding	NRXN1	neurexin 1 [Source:HGNC Symbol;Acc:HGNC:8008]	2
ENSG00000272391	6,055189	-0,440483	0,017653	0,057548	-2,62494	protein_coding	POM121C	POM121 transmembrane nucleoporin C [Source:HGNC Symbol;Acc:HGNC:34005]	7
ENSG00000068878	6,792896	-0,420139	0,017654	0,057548	-2,624917	protein_coding	PSME4	proteasome activator subunit 4 [Source:HGNC Symbol;Acc:HGNC:20635]	2
ENSG00000198218	6,654416	-0,332271	0,017665	0,057574	-2,624604	protein_coding	QRICH1	glutamine rich 1 [Source:HGNC Symbol;Acc:HGNC:24713]	3
ENSG00000129084	3,262425	-0,471891	0,017679	0,057606	-2,624238	protein_coding	PSMA1	proteasome 20S subunit alpha 1 [Source:HGNC Symbol;Acc:HGNC:9530]	11
ENSG00000136367	4,344812	0,845116	0,017703	0,057673	2,623583	protein_coding	ZFHX2	zinc finger homeobox 2 [Source:HGNC Symbol;Acc:HGNC:20152]	14
ENSG00000203896	1,381838	1,039777	0,017707	0,057674	2,62348	protein_coding	LIME1	Lck interacting transmembrane adaptor 1 [Source:HGNC Symbol;Acc:HGNC:26016]	20
ENSG00000143119	2,717058	1,981001	0,017718	0,057699	2,623169	protein_coding	CD53	CD53 molecule [Source:HGNC Symbol;Acc:HGNC:1686]	1
ENSG00000119703	2,018562	1,251658	0,017736	0,057734	2,622689	protein_coding	ZC2HC1C	zinc finger C2HC-type containing 1C [Source:HGNC Symbol;Acc:HGNC:20354]	14
ENSG00000083099	5,139849	-0,57507	0,017733	0,057734	-2,622756	protein_coding	LYRM2	LYR motif containing 2 [Source:HGNC Symbol;Acc:HGNC:25229]	6
ENSG00000165995	2,735772	2,121973	0,017751	0,05777	2,622289	protein_coding	CACNB2	calcium voltage-gated channel auxiliary subunit beta 2 [Source:HGNC Symbol;Acc:HGNC:1402]	10
ENSG00000078549	-1,287505	3,329383	0,017757	0,05778	2,622115	protein_coding	ADCYAP1R	ADCYAP receptor type I [Source:HGNC Symbol;Acc:HGNC:242]	7
ENSG00000150867	5,439542	-0,679184	0,017764	0,057791	-2,621925	protein_coding	PIP4K2A	phosphatidylinositol-5-phosphate 4-kinase type 2 alpha [Source:HGNC Symbol;Acc:HGNC:8997]	10
ENSG00000205129	-0,283506	1,2538	0,017774	0,057799	2,621661	protein_coding	C4orf47	chromosome 4 open reading frame 47 [Source:HGNC Symbol;Acc:HGNC:34346]	4
ENSG00000128917	5,046852	1,419127	0,017772	0,057799	2,621716	protein_coding	DLL4	delta like canonical Notch ligand 4 [Source:HGNC Symbol;Acc:HGNC:2910]	15

ENSG00000126561	4,20453	0,746024	0,017802	0,05788	2,620894	protein_coding	STAT5A	signal transducer and activator of transcription 5A [Source:HGNC Symbol;Acc:HGNC:11366]	17
ENSG00000249773	-2,376133	1,594406	0,017835	0,057968	2,620004	protein_coding	AC092647.	novel zinc finger protein 713 (ZNF713) and mitochondrial ribosomal protein S17 (MRPS17) protein	7
ENSG00000070778	5,546117	-0,851631	0,017836	0,057968	-2,61997	protein_coding	PTPN21	protein tyrosine phosphatase non-receptor type 21 [Source:HGNC Symbol;Acc:HGNC:9651]	14
ENSG00000197837	3,760372	0,724449	0,017848	0,057994	2,61966	protein_coding	H4-16	H4 histone 16 [Source:HGNC Symbol;Acc:HGNC:20510]	12
ENSG00000164040	5,852061	-0,521054	0,017862	0,058029	-2,619271	protein_coding	PGRMC2	progesterone receptor membrane component 2 [Source:HGNC Symbol;Acc:HGNC:16089]	4
ENSG00000133962	0,893933	2,165172	0,017898	0,058122	2,61832	protein_coding	CATSPERB	cation channel sperm associated auxiliary subunit beta [Source:HGNC Symbol;Acc:HGNC:20500]	14
ENSG00000091428	5,060437	1,095761	0,017898	0,058122	2,618308	protein_coding	RAPGEF4	Rap guanine nucleotide exchange factor 4 [Source:HGNC Symbol;Acc:HGNC:16626]	2
ENSG00000215883	2,253066	0,783341	0,017919	0,058177	2,617756	protein_coding	CYB5RL	cytochrome b5 reductase like [Source:HGNC Symbol;Acc:HGNC:32220]	1
ENSG00000152944	4,754777	-0,443896	0,017926	0,058188	-2,617568	protein_coding	MED21	mediator complex subunit 21 [Source:HGNC Symbol;Acc:HGNC:11473]	12
ENSG00000085978	5,124992	-0,557824	0,017996	0,058405	-2,615681	protein_coding	ATG16L1	autophagy related 16 like 1 [Source:HGNC Symbol;Acc:HGNC:21498]	2
ENSG00000236609	3,7814	1,40423	0,01801	0,058425	2,615302	protein_coding	ZNF853	zinc finger protein 853 [Source:HGNC Symbol;Acc:HGNC:21767]	7
ENSG00000006042	4,815028	-1,069933	0,018013	0,058425	-2,615224	protein_coding	TMEM98	transmembrane protein 98 [Source:HGNC Symbol;Acc:HGNC:24529]	17
ENSG00000126107	5,773314	-0,552824	0,018011	0,058425	-2,615284	protein_coding	HECTD3	HECT domain E3 ubiquitin protein ligase 3 [Source:HGNC Symbol;Acc:HGNC:26117]	1
ENSG00000170482	-1,688548	1,923576	0,018019	0,058435	2,615051	protein_coding	SLC23A1	solute carrier family 23 member 1 [Source:HGNC Symbol;Acc:HGNC:10974]	5
ENSG00000285437	2,173563	0,997559	0,018032	0,058455	2,614706	protein_coding	POLR2J3	RNA polymerase II subunit J3 [Source:HGNC Symbol;Acc:HGNC:33853]	7
ENSG00000058866	2,363735	-1,072727	0,018033	0,058455	-2,614694	protein_coding	DGKG	diacylglycerol kinase gamma [Source:HGNC Symbol;Acc:HGNC:2853]	3
ENSG00000102802	2,739523	-2,42048	0,018048	0,058482	-2,614279	protein_coding	MEDAG	mesenteric estrogen dependent adipogenesis [Source:HGNC Symbol;Acc:HGNC:25926]	13
ENSG00000108883	6,550571	-0,314763	0,018046	0,058482	-2,614356	protein_coding	EFTUD2	elongation factor Tu GTP binding domain containing 2 [Source:HGNC Symbol;Acc:HGNC:30858]	17
ENSG00000120054	-3,11635	1,722445	0,018064	0,058522	2,613855	protein_coding	CPN1	carboxypeptidase N subunit 1 [Source:HGNC Symbol;Acc:HGNC:2312]	10
ENSG00000163618	6,256522	1,303651	0,01808	0,05856	2,613444	protein_coding	CADPS	calcium dependent secretion activator [Source:HGNC Symbol;Acc:HGNC:1426]	3
ENSG00000011114	6,156442	-0,337237	0,018089	0,058577	-2,613209	protein_coding	BTBD7	BTB domain containing 7 [Source:HGNC Symbol;Acc:HGNC:18269]	14
ENSG00000196834	-0,17604	2,902282	0,018097	0,058592	2,612988	protein_coding	POTEI	POTE ankyrin domain family member I [Source:HGNC Symbol;Acc:HGNC:37093]	2
ENSG00000160505	-2,549387	2,071648	0,018129	0,058685	2,612126	protein_coding	NLRP4	NLR family pyrin domain containing 4 [Source:HGNC Symbol;Acc:HGNC:22943]	19
ENSG00000141959	7,076238	0,513141	0,018138	0,058701	2,611901	protein_coding	PFKL	phosphofructokinase, liver type [Source:HGNC Symbol;Acc:HGNC:8876]	21
ENSG00000163803	1,70972	1,504395	0,018166	0,058768	2,611157	protein_coding	PLB1	phospholipase B1 [Source:HGNC Symbol;Acc:HGNC:30041]	2
ENSG00000149547	6,393486	-0,478319	0,018164	0,058768	-2,611191	protein_coding	EI24	EI24 autophagy associated transmembrane protein [Source:HGNC Symbol;Acc:HGNC:13276]	11
ENSG00000128573	1,891932	2,335295	0,018205	0,05888	2,61012	protein_coding	FOXP2	forkhead box P2 [Source:HGNC Symbol;Acc:HGNC:13875]	7
ENSG00000189007	2,640786	0,800641	0,018207	0,05888	2,610052	protein_coding	ADAT2	adenosine deaminase tRNA specific 2 [Source:HGNC Symbol;Acc:HGNC:21172]	6
ENSG00000166793	0,473439	1,727263	0,018249	0,058996	2,608964	protein_coding	YPEL4	yippee like 4 [Source:HGNC Symbol;Acc:HGNC:18328]	11
ENSG00000089123	4,25105	0,547607	0,018251	0,058996	2,608909	protein_coding	TASP1	taspase 1 [Source:HGNC Symbol;Acc:HGNC:15859]	20
ENSG00000278195	0,389191	2,682841	0,018258	0,059008	2,608714	protein_coding	SSTR3	somatostatin receptor 3 [Source:HGNC Symbol;Acc:HGNC:11332]	22
ENSG00000101457	4,88243	-0,459336	0,01827	0,059027	-2,608394	protein_coding	DNTTIP1	deoxynucleotidyltransferase terminal interacting protein 1 [Source:HGNC Symbol;Acc:HGNC:16160]	20
ENSG00000055211	5,342831	-0,567565	0,018271	0,059027	-2,608365	protein_coding	GINM1	glycoprotein integral membrane 1 [Source:HGNC Symbol;Acc:HGNC:21074]	6
ENSG00000169902	5,524522	-1,100127	0,018275	0,059027	-2,608278	protein_coding	TPST1	tyrosylprotein sulfotransferase 1 [Source:HGNC Symbol;Acc:HGNC:12020]	7
ENSG00000173614	2,83497	-0,608514	0,018282	0,059029	-2,608076	protein_coding	NMNAT1	nicotinamide nucleotide adenylyltransferase 1 [Source:HGNC Symbol;Acc:HGNC:17877]	1
ENSG00000184619	4,005834	1,148613	0,018286	0,059029	2,607984	protein_coding	KRBA2	KRAB-A domain containing 2 [Source:HGNC Symbol;Acc:HGNC:26989]	17
ENSG00000160325	4,47921	1,033154	0,018286	0,059029	2,607971	protein_coding	CACFD1	calcium channel flower domain containing 1 [Source:HGNC Symbol;Acc:HGNC:1365]	9
ENSG00000146013	-0,958625	2,779693	0,018308	0,059082	2,60741	protein_coding	GFRA3	GDNF family receptor alpha 3 [Source:HGNC Symbol;Acc:HGNC:4245]	5
ENSG00000198829	1,133348	-1,265699	0,01831	0,059082	-2,60735	protein_coding	SUCNR1	succinate receptor 1 [Source:HGNC Symbol;Acc:HGNC:4542]	3
ENSG00000106351	4,737516	1,095042	0,018359	0,059229	2,606055	protein_coding	AGFG2	ArfGAP with FG repeats 2 [Source:HGNC Symbol;Acc:HGNC:5177]	7
ENSG00000167978	9,886793	0,371772	0,018367	0,059242	2,60585	protein_coding	SRRM2	serine/arginine repetitive matrix 2 [Source:HGNC Symbol;Acc:HGNC:16639]	16
ENSG00000091592	4,396068	1,139479	0,018389	0,059301	2,605274	protein_coding	NLRP1	NLR family pyrin domain containing 1 [Source:HGNC Symbol;Acc:HGNC:14374]	17
ENSG00000108091	6,233602	-0,500586	0,018407	0,059349	-2,604791	protein_coding	CCDC6	coiled-coil domain containing 6 [Source:HGNC Symbol;Acc:HGNC:18782]	10
ENSG00000079841	2,094722	2,261498	0,018464	0,059512	2,60331	protein_coding	RIMS1	regulating synaptic membrane exocytosis 1 [Source:HGNC Symbol;Acc:HGNC:17282]	6
ENSG00000146038	2,998904	1,450841	0,018469	0,059512	2,60318	protein_coding	DCDC2	doublecortin domain containing 2 [Source:HGNC Symbol;Acc:HGNC:18141]	6
ENSG00000005206	5,985266	0,900546	0,018468	0,059512	2,603214	protein_coding	SPPL2B	signal peptide peptidase like 2B [Source:HGNC Symbol;Acc:HGNC:30627]	19
ENSG00000010932	-2,622662	2,895869	0,018488	0,059563	2,602674	protein_coding	FMO1	flavin containing dimethylaniline monoxygenase 1 [Source:HGNC Symbol;Acc:HGNC:3769]	1
ENSG00000154803	5,616496	0,714725	0,018499	0,059573	2,602402	protein_coding	FLCN	folliculin [Source:HGNC Symbol;Acc:HGNC:27310]	17
ENSG00000100258	5,882508	-0,612817	0,018496	0,059573	-2,602475	protein_coding	LMF2	lipase maturation factor 2 [Source:HGNC Symbol;Acc:HGNC:25096]	22
ENSG00000124479	-2,606267	-2,674007	0,018517	0,059597	-2,601919	protein_coding	NDP	norrin cystine knot growth factor NDP [Source:HGNC Symbol;Acc:HGNC:7678]	X
ENSG00000124743	-0,226578	1,770948	0,018519	0,059597	2,601885	protein_coding	KLHL31	kelch like family member 31 [Source:HGNC Symbol;Acc:HGNC:21353]	6

ENSG00000205268	4,317077	0,903785	0,01852	0,059597	2,601841	protein_coding	PDE7A	phosphodiesterase 7A [Source:HGNC Symbol;Acc:HGNC:8791]	8
ENSG00000144909	4,52556	-0,452112	0,018521	0,059597	-2,601823	protein_coding	OSBPL11	oxysterol binding protein like 11 [Source:HGNC Symbol;Acc:HGNC:16397]	3
ENSG00000123154	3,321637	0,575532	0,018535	0,059632	2,601445	protein_coding	WDR83	WD repeat domain 83 [Source:HGNC Symbol;Acc:HGNC:32672]	19
ENSG00000138675	0,218842	-2,145027	0,018546	0,059633	-2,601178	protein_coding	FGF5	fibroblast growth factor 5 [Source:HGNC Symbol;Acc:HGNC:3683]	4
ENSG00000115226	2,683667	-1,815074	0,018546	0,059633	-2,601179	protein_coding	FNDC4	fibronectin type III domain containing 4 [Source:HGNC Symbol;Acc:HGNC:20239]	2
ENSG00000117226	4,712611	-1,769573	0,018547	0,059633	-2,601149	protein_coding	GBP3	guanylate binding protein 3 [Source:HGNC Symbol;Acc:HGNC:4184]	1
ENSG00000122435	3,797983	0,492161	0,018553	0,05964	2,600998	protein_coding	TRMT13	tRNA methyltransferase 13 homolog [Source:HGNC Symbol;Acc:HGNC:25502]	1
ENSG00000135074	5,820068	-1,120583	0,018564	0,059666	-2,600692	protein_coding	ADAM19	ADAM metallopeptidase domain 19 [Source:HGNC Symbol;Acc:HGNC:197]	5
ENSG00000204219	4,203415	1,970107	0,018594	0,059737	2,599928	protein_coding	TCEA3	transcription elongation factor A3 [Source:HGNC Symbol;Acc:HGNC:11615]	1
ENSG00000215301	8,657442	-0,50126	0,018594	0,059737	-2,599937	protein_coding	DDX3X	DEAD-box helicase 3 X-linked [Source:HGNC Symbol;Acc:HGNC:2745]	X
ENSG00000257727	3,224172	-0,517299	0,018614	0,05979	-2,599407	protein_coding	CNPY2	canopy FGF signaling regulator 2 [Source:HGNC Symbol;Acc:HGNC:13529]	12
ENSG00000056586	7,222731	-0,423474	0,018624	0,05981	-2,599147	protein_coding	RC3H2	ring finger and CCCH-type domains 2 [Source:HGNC Symbol;Acc:HGNC:21461]	9
ENSG00000206538	4,740211	-1,505603	0,018628	0,059812	-2,599042	protein_coding	VGLL3	vestigial like family member 3 [Source:HGNC Symbol;Acc:HGNC:24327]	3
ENSG00000206418	5,687344	-0,622167	0,018639	0,059835	-2,598754	protein_coding	RAB12	RAB12, member RAS oncogene family [Source:HGNC Symbol;Acc:HGNC:31332]	18
ENSG00000102312	2,805291	-0,613166	0,018685	0,05997	-2,597577	protein_coding	PORCN	porcupine O-acyltransferase [Source:HGNC Symbol;Acc:HGNC:17652]	X
ENSG00000106443	6,975735	0,811687	0,018712	0,060045	2,596875	protein_coding	PHF14	PHD finger protein 14 [Source:HGNC Symbol;Acc:HGNC:22203]	7
ENSG00000139637	-0,30141	-1,232671	0,018718	0,060054	-2,596713	protein_coding	MYG1	MYG1 exonuclease [Source:HGNC Symbol;Acc:HGNC:17590]	12
ENSG00000170185	5,374377	-0,603694	0,018741	0,060115	-2,596125	protein_coding	USP38	ubiquitin specific peptidase 38 [Source:HGNC Symbol;Acc:HGNC:20067]	4
ENSG00000182247	3,973082	-0,711008	0,018754	0,060144	-2,595797	protein_coding	UBE2E2	ubiquitin conjugating enzyme E2 E2 [Source:HGNC Symbol;Acc:HGNC:12478]	3
ENSG00000178828	-0,482612	2,807544	0,018773	0,060194	2,595299	protein_coding	RNF186	ring finger protein 186 [Source:HGNC Symbol;Acc:HGNC:25978]	1
ENSG00000160282	0,130532	2,972117	0,018777	0,060194	2,595204	protein_coding	FTCD	formimidoyltransferase cyclodeaminase [Source:HGNC Symbol;Acc:HGNC:3974]	21
ENSG00000178607	6,697973	0,814742	0,01879	0,060223	2,594873	protein_coding	ERN1	endoplasmic reticulum to nucleus signaling 1 [Source:HGNC Symbol;Acc:HGNC:3449]	17
ENSG00000204869	-1,907772	2,074427	0,018806	0,06025	2,594467	protein_coding	IGFL4	IGF like family member 4 [Source:HGNC Symbol;Acc:HGNC:32931]	19
ENSG00000114933	6,323648	0,318098	0,018803	0,06025	2,59454	protein_coding	INO80D	INO80 complex subunit D [Source:HGNC Symbol;Acc:HGNC:25997]	2
ENSG00000197894	6,013744	-0,733955	0,018832	0,060323	-2,593788	protein_coding	ADH5	alcohol dehydrogenase 5 (class III), chi polypeptide [Source:HGNC Symbol;Acc:HGNC:253]	4
ENSG00000136156	8,386445	-0,591946	0,018852	0,060377	-2,593266	protein_coding	ITM2B	integral membrane protein 2B [Source:HGNC Symbol;Acc:HGNC:6174]	13
ENSG00000165566	-0,958989	3,717527	0,018862	0,060382	2,59303	protein_coding	AMER2	APC membrane recruitment protein 2 [Source:HGNC Symbol;Acc:HGNC:26360]	13
ENSG00000133619	3,488753	1,063738	0,018865	0,060382	2,592937	protein_coding	KRBA1	KRAB-A domain containing 1 [Source:HGNC Symbol;Acc:HGNC:22228]	7
ENSG00000143786	3,48452	-1,297623	0,018864	0,060382	-2,592972	protein_coding	CNIH3	cornichon family AMPA receptor auxiliary protein 3 [Source:HGNC Symbol;Acc:HGNC:26802]	1
ENSG00000101079	5,739022	-0,443928	0,01887	0,060384	-2,592824	protein_coding	NDRG3	NDRG family member 3 [Source:HGNC Symbol;Acc:HGNC:14462]	20
ENSG00000159339	-2,575009	2,624183	0,018875	0,06039	2,592683	protein_coding	PADI4	peptidyl arginine deiminase 4 [Source:HGNC Symbol;Acc:HGNC:18368]	1
ENSG00000272305	-1,362024	1,571431	0,018881	0,060397	2,592536	protein_coding	AC096887.	novel transcript	3
ENSG00000128294	5,17101	-1,011375	0,018913	0,060488	-2,591709	protein_coding	TPST2	tyrosylprotein sulfotransferase 2 [Source:HGNC Symbol;Acc:HGNC:12021]	22
ENSG00000148908	3,519858	-1,354576	0,018922	0,060503	-2,591496	protein_coding	RGS10	regulator of G protein signaling 10 [Source:HGNC Symbol;Acc:HGNC:9992]	10
ENSG00000165233	4,182412	-0,453949	0,018936	0,060536	-2,591138	protein_coding	CARD19	caspase recruitment domain family member 19 [Source:HGNC Symbol;Acc:HGNC:28148]	9
ENSG00000065320	2,557317	1,716569	0,018986	0,060662	2,589854	protein_coding	NTN1	netrin 1 [Source:HGNC Symbol;Acc:HGNC:8029]	17
ENSG00000182287	4,487878	-0,560436	0,018983	0,060662	-2,589929	protein_coding	AP1S2	adaptor related protein complex 1 subunit sigma 2 [Source:HGNC Symbol;Acc:HGNC:560]	X
ENSG00000108669	6,139363	0,514976	0,018981	0,060662	2,589973	protein_coding	CYTH1	cytohesin 1 [Source:HGNC Symbol;Acc:HGNC:9501]	17
ENSG00000120149	-0,003268	-2,619843	0,018995	0,060679	-2,58962	protein_coding	MSX2	msh homeobox 2 [Source:HGNC Symbol;Acc:HGNC:7392]	5
ENSG00000156531	5,281211	-0,457014	0,019005	0,060698	-2,589374	protein_coding	PHF6	PHD finger protein 6 [Source:HGNC Symbol;Acc:HGNC:18145]	X
ENSG00000075340	3,145314	3,129665	0,01901	0,060702	2,58925	protein_coding	ADD2	adducin 2 [Source:HGNC Symbol;Acc:HGNC:244]	2
ENSG00000198743	6,318284	-0,783231	0,019021	0,060724	-2,58898	protein_coding	SLC5A3	solute carrier family 5 member 3 [Source:HGNC Symbol;Acc:HGNC:11038]	21
ENSG00000076321	4,724566	-0,617132	0,019031	0,060744	-2,588723	protein_coding	KLHL20	kelch like family member 20 [Source:HGNC Symbol;Acc:HGNC:25056]	1
ENSG00000274641	4,488939	0,955693	0,019037	0,060752	2,588565	protein_coding	H2BC17	H2B clustered histone 17 [Source:HGNC Symbol;Acc:HGNC:4758]	6
ENSG00000180822	3,994968	0,590602	0,019045	0,060768	2,588347	protein_coding	PSMG4	proteasome assembly chaperone 4 [Source:HGNC Symbol;Acc:HGNC:21108]	6
ENSG00000148841	5,120486	-0,972085	0,019053	0,06078	-2,588156	protein_coding	ITPRIP	inositol 1,4,5-trisphosphate receptor interacting protein [Source:HGNC Symbol;Acc:HGNC:29370]	10
ENSG00000177455	-2,609718	2,808703	0,019076	0,060821	2,587583	protein_coding	CD19	CD19 molecule [Source:HGNC Symbol;Acc:HGNC:1633]	16
ENSG00000164039	2,937164	-1,01863	0,019077	0,060821	-2,587545	protein_coding	BDH2	3-hydroxybutyrate dehydrogenase 2 [Source:HGNC Symbol;Acc:HGNC:32389]	4
ENSG00000141040	3,297594	0,841326	0,019073	0,060821	2,58766	protein_coding	ZNF287	zinc finger protein 287 [Source:HGNC Symbol;Acc:HGNC:13502]	17
ENSG00000153208	4,04981	1,143251	0,019081	0,060822	2,58744	protein_coding	MERTK	MER proto-oncogene, tyrosine kinase [Source:HGNC Symbol;Acc:HGNC:7027]	2
ENSG00000038274	5,29817	-0,612315	0,019088	0,060831	-2,587274	protein_coding	MAT2B	methionine adenosyltransferase 2B [Source:HGNC Symbol;Acc:HGNC:6905]	5

Supplementary Table 5: GSEA of original tumor tissue vs PD tumoroids

ID	Description	setSize	enrichmen	NES	pvalue	p.adjust	qvalues	rank	leading_edge
GO:004368	post-translational protein modification	337	-0,44869	-2,31851	5,72E-17	6,69E-14	3,13E-14	2678	tags=36%, list=16%, signal=30%
GO:000688	endoplasmic reticulum to Golgi vesicle-mediated transport	191	-0,52011	-2,51605	1,47E-15	6,51E-13	3,05E-13	2869	tags=42%, list=18%, signal=35%
GO:004819	Golgi vesicle transport	352	-0,43592	-2,25862	1,67E-15	6,51E-13	3,05E-13	2963	tags=36%, list=18%, signal=30%
GO:003497	response to endoplasmic reticulum stress	288	-0,45732	-2,32746	4,89E-15	1,43E-12	6,69E-13	3823	tags=43%, list=24%, signal=34%
GO:000225	adaptive immune response	353	0,391787	2,20227	7,99E-15	1,87E-12	8,75E-13	2791	tags=36%, list=17%, signal=30%
GO:003298	protein-containing complex disassembly	316	-0,4356	-2,23253	3,14E-14	5,22E-12	2,44E-12	3954	tags=44%, list=24%, signal=34%
GO:004522	external encapsulating structure organization	364	-0,42013	-2,18369	3,25E-14	5,22E-12	2,44E-12	2899	tags=35%, list=18%, signal=30%
GO:004306	extracellular structure organization	364	-0,41996	-2,18281	3,57E-14	5,22E-12	2,44E-12	2899	tags=35%, list=18%, signal=30%
GO:003019	extracellular matrix organization	363	-0,42181	-2,18995	1E-13	1,21E-11	5,66E-12	2899	tags=36%, list=18%, signal=30%
GO:000334	cilium movement	120	0,544839	2,621434	1,03E-13	1,21E-11	5,66E-12	4589	tags=65%, list=28%, signal=47%
GO:004362	cellular protein complex disassembly	215	-0,47886	-2,3552	2,56E-13	2,73E-11	1,28E-11	3782	tags=46%, list=23%, signal=35%
GO:001623	macroautophagy	302	-0,42245	-2,15471	1,08E-12	1,05E-10	4,91E-11	3791	tags=42%, list=23%, signal=33%
GO:000641	translational termination	104	-0,57743	-2,55606	2,36E-12	2,12E-10	9,93E-11	4202	tags=59%, list=26%, signal=44%
GO:006007	Wnt signaling pathway, planar cell polarity pathway	105	-0,57029	-2,52772	4,98E-12	3,9E-10	1,82E-10	2632	tags=45%, list=16%, signal=38%
GO:000717	transforming growth factor beta receptor signaling pathway	177	-0,48934	-2,33879	5E-12	3,9E-10	1,82E-10	3040	tags=38%, list=19%, signal=31%
GO:003596	response to topologically incorrect protein	197	-0,47542	-2,31054	5,87E-12	4,1E-10	1,92E-10	3992	tags=48%, list=25%, signal=37%
GO:009017	regulation of establishment of planar polarity	109	-0,5566	-2,48575	5,96E-12	4,1E-10	1,92E-10	2632	tags=43%, list=16%, signal=36%
GO:004362	regulation of DNA-templated transcription in response to stress	111	-0,5561	-2,48322	6,89E-12	4,25E-10	1,99E-10	2661	tags=44%, list=16%, signal=37%
GO:001605	vesicle organization	324	-0,4035	-2,07669	6,9E-12	4,25E-10	1,99E-10	4233	tags=44%, list=26%, signal=34%
GO:190384	regulation of cellular response to transforming growth factor beta stimulus	114	-0,54954	-2,47507	1,23E-11	7,22E-10	3,38E-10	3040	tags=42%, list=19%, signal=34%
GO:004361	regulation of transcription from RNA polymerase II promoter in response to stress	105	-0,5628	-2,4945	1,46E-11	8,11E-10	3,79E-10	2661	tags=45%, list=16%, signal=38%
GO:001701	regulation of transforming growth factor beta receptor signaling pathway	112	-0,55041	-2,46315	1,6E-11	8,53E-10	3,99E-10	3040	tags=42%, list=19%, signal=34%
GO:000698	response to unfolded protein	176	-0,47392	-2,26338	3,99E-11	2,03E-09	9,5E-10	3992	tags=47%, list=25%, signal=36%
GO:000173	morphogenesis of a polarized epithelium	141	-0,50534	-2,34072	7,93E-11	3,83E-09	1,79E-09	2632	tags=40%, list=16%, signal=34%
GO:007155	response to transforming growth factor beta	228	-0,44335	-2,19018	8,18E-11	3,83E-09	1,79E-09	3085	tags=36%, list=19%, signal=29%
GO:003556	non-canonical Wnt signaling pathway	147	-0,4992	-2,32392	1,18E-10	5,2E-09	2,43E-09	2632	tags=39%, list=16%, signal=33%
GO:003596	cellular response to topologically incorrect protein	162	-0,48996	-2,3054	1,2E-10	5,2E-09	2,43E-09	3604	tags=46%, list=22%, signal=36%
GO:009719	extrinsic apoptotic signaling pathway	202	-0,4482	-2,18394	1,3E-10	5,41E-09	2,53E-09	3077	tags=35%, list=19%, signal=28%
GO:003254	mitochondrial translation	133	-0,51132	-2,36279	1,96E-10	7,89E-09	3,69E-09	5137	tags=62%, list=32%, signal=43%
GO:007134	cellular response to interleukin-1	161	-0,47588	-2,23716	3,35E-10	1,21E-08	5,65E-09	2632	tags=50%, list=16%, signal=42%
GO:000173	establishment of planar polarity	120	-0,52373	-2,37166	3,4E-10	1,21E-08	5,65E-09	2632	tags=41%, list=16%, signal=34%
GO:000716	establishment of tissue polarity	120	-0,52373	-2,37166	3,4E-10	1,21E-08	5,65E-09	2632	tags=41%, list=16%, signal=34%
GO:003017	positive regulation of Wnt signaling pathway	172	-0,47059	-2,23657	3,41E-10	1,21E-08	5,65E-09	4198	tags=48%, list=26%, signal=36%
GO:000910	glycoprotein metabolic process	385	-0,37396	-1,95514	4,24E-10	1,46E-08	6,82E-09	3311	tags=33%, list=20%, signal=27%
GO:003238	regulation of intracellular transport	332	-0,38745	-1,99971	5,64E-10	1,82E-08	8,5E-09	4021	tags=40%, list=25%, signal=31%
GO:000150	ossification	358	-0,3784	-1,96283	5,71E-10	1,82E-08	8,5E-09	3117	tags=32%, list=19%, signal=27%
GO:007156	cellular response to transforming growth factor beta stimulus	222	-0,43997	-2,16878	5,75E-10	1,82E-08	8,5E-09	3085	tags=35%, list=19%, signal=28%
GO:014005	mitochondrial gene expression	164	-0,46554	-2,19633	7,58E-10	2,31E-08	1,08E-08	5137	tags=55%, list=32%, signal=38%
GO:000247	antigen processing and presentation of exogenous peptide antigen	167	-0,4628	-2,18822	7,71E-10	2,31E-08	1,08E-08	2632	tags=40%, list=16%, signal=34%
GO:200002	regulation of animal organ morphogenesis	169	-0,46389	-2,20403	7,96E-10	2,33E-08	1,09E-08	2632	tags=42%, list=16%, signal=36%
GO:006113	regulation of proteasomal protein catabolic process	176	-0,45312	-2,16403	9,31E-10	2,66E-08	1,24E-08	3878	tags=45%, list=24%, signal=35%
GO:015011	cell-substrate junction organization	103	-0,53418	-2,35798	1,03E-09	2,82E-08	1,32E-08	2915	tags=44%, list=18%, signal=36%
GO:200123	regulation of apoptotic signaling pathway	322	-0,38541	-1,98029	1,04E-09	2,82E-08	1,32E-08	3248	tags=31%, list=20%, signal=25%
GO:007055	response to interleukin-1	182	-0,45257	-2,17036	1,22E-09	3,22E-08	1,51E-08	2632	tags=34%, list=16%, signal=29%
GO:200123	regulation of extrinsic apoptotic signaling pathway	142	-0,48031	-2,23018	1,26E-09	3,22E-08	1,51E-08	3077	tags=38%, list=19%, signal=31%
GO:009026	positive regulation of canonical Wnt signaling pathway	140	-0,491	-2,27437	1,27E-09	3,22E-08	1,51E-08	2726	tags=36%, list=17%, signal=31%
GO:000645	protein folding	203	-0,43947	-2,1438	1,46E-09	3,63E-08	1,7E-08	3838	tags=47%, list=24%, signal=36%
GO:003011	regulation of Wnt signaling pathway	348	-0,37341	-1,93036	2,15E-09	5,25E-08	2,46E-08	4198	tags=39%, list=26%, signal=29%

GO:005121	cartilage development	167	-0,45428	-2,14793	2,66E-09	6,35E-08	2,97E-08	3096	tags=37%, list=19%, signal=30%
GO:009010	negative regulation of transmembrane receptor protein serine/threonine kinase signaling pathway	119	-0,50948	-2,30356	2,82E-09	6,6E-08	3,09E-08	3234	tags=42%, list=20%, signal=34%
GO:004348	regulation of mRNA stability	180	-0,44983	-2,15942	3,02E-09	6,94E-08	3,25E-08	3686	tags=42%, list=23%, signal=33%
GO:004348	regulation of RNA stability	190	-0,43802	-2,11543	3,18E-09	7,16E-08	3,35E-08	3705	tags=40%, list=23%, signal=31%
GO:190275	negative regulation of cell cycle G2/M phase transition	105	-0,52218	-2,31448	3,72E-09	8,09E-08	3,78E-08	2632	tags=40%, list=16%, signal=34%
GO:000717	transmembrane receptor protein serine/threonine kinase signaling pathway	316	-0,38174	-1,95651	3,73E-09	8,09E-08	3,78E-08	3044	tags=34%, list=19%, signal=28%
GO:000164	osteoblast differentiation	197	-0,4348	-2,11313	3,82E-09	8,13E-08	3,81E-08	3117	tags=34%, list=19%, signal=28%
GO:003462	cellular response to unfolded protein	143	-0,48079	-2,23523	5,01E-09	1,05E-07	4,9E-08	3992	tags=47%, list=25%, signal=36%
GO:003096	endoplasmic reticulum unfolded protein response	123	-0,49953	-2,27344	5,34E-09	1,1E-07	5,13E-08	3992	tags=46%, list=25%, signal=35%
GO:003158	cell-substrate adhesion	341	-0,37238	-1,92804	5,96E-09	1,2E-07	5,6E-08	3009	tags=33%, list=19%, signal=28%
GO:000193	negative regulation of protein phosphorylation	332	-0,37418	-1,93123	6,03E-09	1,2E-07	5,6E-08	3156	tags=31%, list=19%, signal=26%
GO:009028	regulation of cellular response to growth factor stimulus	249	-0,40031	-1,9982	6,23E-09	1,21E-07	5,68E-08	3663	tags=35%, list=23%, signal=27%
GO:004217	regulation of protein catabolic process	368	-0,36709	-1,91206	6,46E-09	1,24E-07	5,8E-08	3895	tags=38%, list=24%, signal=29%
GO:000020	protein polyubiquitination	321	-0,37457	-1,92268	8,24E-09	1,55E-07	7,27E-08	4211	tags=42%, list=26%, signal=32%
GO:004232	negative regulation of phosphorylation	370	-0,36069	-1,87866	8,42E-09	1,56E-07	7,32E-08	3212	tags=31%, list=20%, signal=25%
GO:004665	lymphocyte proliferation	240	0,367915	1,9719	8,65E-09	1,58E-07	7,39E-08	2737	tags=35%, list=17%, signal=29%
GO:001988	antigen processing and presentation of exogenous antigen	172	-0,44724	-2,12561	9,67E-09	1,74E-07	8,13E-08	2632	tags=39%, list=16%, signal=33%
GO:003294	mononuclear cell proliferation	243	0,364654	1,959168	9,8E-09	1,74E-07	8,13E-08	2737	tags=34%, list=17%, signal=29%
GO:005165	establishment of vesicle localization	202	-0,42283	-2,06033	1,07E-08	1,87E-07	8,74E-08	2987	tags=46%, list=18%, signal=38%
GO:006219	cellular response to chemical stress	318	-0,37632	-1,9289	1,48E-08	2,55E-07	1,19E-07	3729	tags=37%, list=23%, signal=29%
GO:190382	positive regulation of cellular protein localization	287	-0,38781	-1,9691	1,57E-08	2,66E-07	1,24E-07	3085	tags=33%, list=19%, signal=27%
GO:000183	epithelial to mesenchymal transition	141	-0,46679	-2,16216	1,85E-08	3,02E-07	1,41E-07	3309	tags=38%, list=20%, signal=30%
GO:006007	canonical Wnt signaling pathway	316	-0,37277	-1,91055	1,85E-08	3,02E-07	1,41E-07	4119	tags=39%, list=25%, signal=29%
GO:200123	negative regulation of apoptotic signaling pathway	208	-0,42089	-2,05359	1,86E-08	3,02E-07	1,41E-07	3077	tags=34%, list=19%, signal=28%
GO:007064	protein modification by small protein removal	271	-0,3918	-1,9755	2,09E-08	3,34E-07	1,56E-07	3281	tags=34%, list=20%, signal=28%
GO:007048	response to oxygen levels	360	-0,35642	-1,85122	2,34E-08	3,7E-07	1,73E-07	2981	tags=29%, list=18%, signal=24%
GO:003809	Fc-epsilon receptor signaling pathway	106	-0,51394	-2,2762	2,37E-08	3,7E-07	1,73E-07	2632	tags=44%, list=16%, signal=37%
GO:190336	regulation of cellular protein catabolic process	242	-0,40186	-1,99698	2,45E-08	3,74E-07	1,75E-07	3895	tags=42%, list=24%, signal=32%
GO:000641	translational elongation	130	-0,4713	-2,16752	2,47E-08	3,74E-07	1,75E-07	3465	tags=50%, list=21%, signal=40%
GO:200073	regulation of stem cell differentiation	106	-0,51329	-2,27332	2,5E-08	3,74E-07	1,75E-07	3048	tags=43%, list=19%, signal=35%
GO:190199	negative regulation of mitotic cell cycle phase transition	224	-0,40687	-2,00897	2,53E-08	3,74E-07	1,75E-07	3538	tags=37%, list=22%, signal=29%
GO:003017	negative regulation of Wnt signaling pathway	202	-0,41493	-2,02182	2,94E-08	4,3E-07	2,01E-07	3009	tags=31%, list=19%, signal=26%
GO:004876	mesenchymal cell differentiation	214	-0,41087	-2,02043	3,15E-08	4,53E-07	2,12E-07	3309	tags=34%, list=20%, signal=27%
GO:009009	regulation of transmembrane receptor protein serine/threonine kinase signaling pathway	219	-0,40876	-2,00919	3,17E-08	4,53E-07	2,12E-07	3663	tags=36%, list=23%, signal=28%
GO:004886	stem cell differentiation	237	-0,40224	-1,99757	3,97E-08	5,59E-07	2,62E-07	2632	tags=30%, list=16%, signal=25%
GO:000679	sulfur compound metabolic process	342	-0,35651	-1,84516	4,79E-08	6,67E-07	3,12E-07	3502	tags=34%, list=22%, signal=27%
GO:001657	protein deubiquitination	254	-0,39257	-1,96793	5,15E-08	7,08E-07	3,31E-07	2863	tags=31%, list=18%, signal=26%
GO:006101	regulation of mRNA catabolic process	201	-0,41108	-2,00626	5,47E-08	7,45E-07	3,48E-07	3686	tags=39%, list=23%, signal=30%
GO:190305	regulation of proteolysis involved in cellular protein catabolic process	207	-0,41342	-2,02139	5,96E-08	8,01E-07	3,75E-07	3878	tags=42%, list=24%, signal=32%
GO:001619	endosomal transport	234	-0,39526	-1,95751	6,67E-08	8,86E-07	4,15E-07	4023	tags=38%, list=25%, signal=29%
GO:004800	antigen processing and presentation of peptide antigen	184	-0,42589	-2,0457	6,99E-08	9,19E-07	4,3E-07	2632	tags=37%, list=16%, signal=31%
GO:003243	regulation of proteasomal ubiquitin-dependent protein catabolic process	130	-0,46138	-2,12191	7,48E-08	9,72E-07	4,55E-07	3878	tags=46%, list=24%, signal=35%
GO:000170	formation of primary germ layer	102	-0,50356	-2,21479	8,3E-08	1,07E-06	4,99E-07	3143	tags=44%, list=19%, signal=36%
GO:005164	vesicle localization	217	-0,4052	-1,99199	8,68E-08	1,1E-06	5,13E-07	4215	tags=45%, list=26%, signal=34%
GO:003238	positive regulation of intracellular transport	208	-0,41109	-2,00578	8,71E-08	1,1E-06	5,13E-07	4113	tags=43%, list=25%, signal=33%
GO:003806	NIK/NF-kappaB signaling	161	-0,43761	-2,05728	8,93E-08	1,11E-06	5,2E-07	2890	tags=40%, list=18%, signal=34%
GO:001624	regulation of macroautophagy	156	-0,43994	-2,06413	9,08E-08	1,12E-06	5,23E-07	3588	tags=44%, list=22%, signal=35%
GO:003294	regulation of mononuclear cell proliferation	199	0,375939	1,973716	9,31E-08	1,12E-06	5,25E-07	2709	tags=35%, list=17%, signal=30%
GO:009009	negative regulation of canonical Wnt signaling pathway	170	-0,42691	-2,02494	9,33E-08	1,12E-06	5,25E-07	3009	tags=34%, list=19%, signal=28%

GO:190198	negative regulation of cell cycle phase transition	237	-0,39758	-1,97446	9,39E-08	1,12E-06	5,25E-07	3538	tags=36%, list=22%, signal=28%
GO:000646	negative regulation of protein kinase activity	226	-0,39745	-1,95911	1,01E-07	1,2E-06	5,59E-07	3148	tags=32%, list=19%, signal=26%
GO:006082	regulation of canonical Wnt signaling pathway	272	-0,37997	-1,91628	1,12E-07	1,31E-06	6,11E-07	4232	tags=40%, list=26%, signal=30%
GO:000222	stimulatory C-type lectin receptor signaling pathway	103	-0,4961	-2,18986	1,24E-07	1,44E-06	6,72E-07	2700	tags=40%, list=17%, signal=33%
GO:000910	glycoprotein biosynthetic process	318	-0,36263	-1,85874	1,43E-07	1,65E-06	7,7E-07	3528	tags=33%, list=22%, signal=26%
GO:006144	connective tissue development	222	-0,40333	-1,9882	1,53E-07	1,74E-06	8,13E-07	3544	tags=37%, list=22%, signal=30%
GO:001821	protein phosphopantetheinylation	291	-0,36143	-1,84447	1,6E-07	1,8E-06	8,43E-07	3353	tags=35%, list=21%, signal=28%
GO:005067	regulation of lymphocyte proliferation	197	0,377581	1,982095	1,69E-07	1,88E-06	8,8E-07	2709	tags=36%, list=17%, signal=30%
GO:001988	antigen processing and presentation	220	-0,39799	-1,95791	1,75E-07	1,93E-06	9,02E-07	2636	tags=35%, list=16%, signal=30%
GO:000683	mitochondrial transport	258	-0,37534	-1,88579	1,84E-07	2,01E-06	9,42E-07	3829	tags=37%, list=24%, signal=29%
GO:000690	vesicle budding from membrane	106	-0,49313	-2,18402	1,96E-07	2,12E-06	9,94E-07	3966	tags=58%, list=24%, signal=44%
GO:007145	cellular response to oxygen levels	212	-0,3945	-1,93671	1,98E-07	2,13E-06	9,94E-07	2930	tags=33%, list=18%, signal=28%
GO:006048	mesenchyme development	259	-0,37128	-1,86673	2,02E-07	2,13E-06	9,97E-07	3309	tags=34%, list=20%, signal=28%
GO:007058	protein localization to mitochondrion	143	-0,45043	-2,09409	2,02E-07	2,13E-06	9,97E-07	4151	tags=46%, list=26%, signal=35%
GO:190382	negative regulation of cellular protein localization	108	-0,48214	-2,14687	2,08E-07	2,17E-06	1,02E-06	4526	tags=56%, list=28%, signal=40%
GO:004225	ribosome biogenesis	295	-0,3641	-1,85717	2,36E-07	2,44E-06	1,14E-06	4312	tags=41%, list=27%, signal=31%
GO:190547	regulation of protein localization to membrane	185	-0,41052	-1,97677	2,56E-07	2,63E-06	1,23E-06	2825	tags=32%, list=17%, signal=27%
GO:000166	ameboidal-type cell migration	382	-0,3381	-1,76691	2,89E-07	2,94E-06	1,38E-06	4333	tags=39%, list=27%, signal=29%
GO:003315	regulation of intracellular protein transport	239	-0,38229	-1,89888	3,1E-07	3,12E-06	1,46E-06	4217	tags=42%, list=26%, signal=31%
GO:004218	cellular ketone metabolic process	227	-0,38548	-1,9017	3,25E-07	3,25E-06	1,52E-06	2632	tags=30%, list=16%, signal=25%
GO:007265	establishment of protein localization to mitochondrion	139	-0,44413	-2,05996	3,31E-07	3,28E-06	1,54E-06	4151	tags=45%, list=26%, signal=34%
GO:004593	negative regulation of mitotic cell cycle	288	-0,36362	-1,85057	3,37E-07	3,31E-06	1,55E-06	3754	tags=34%, list=23%, signal=27%
GO:000716	cell-matrix adhesion	217	-0,39375	-1,9357	3,57E-07	3,48E-06	1,63E-06	3009	tags=35%, list=19%, signal=29%
GO:000736	gastrulation	162	-0,43173	-2,03141	3,8E-07	3,65E-06	1,71E-06	3143	tags=35%, list=19%, signal=28%
GO:000244	lymphocyte mediated immunity	213	0,354211	1,867853	3,81E-07	3,65E-06	1,71E-06	2791	tags=32%, list=17%, signal=27%
GO:001056	regulation of cellular ketone metabolic process	165	-0,42297	-1,99757	3,98E-07	3,79E-06	1,77E-06	2632	tags=33%, list=16%, signal=28%
GO:003809	Fc receptor signaling pathway	170	-0,41415	-1,96442	5,03E-07	4,75E-06	2,22E-06	2939	tags=40%, list=18%, signal=33%
GO:000275	innate immune response-activating signal transduction	107	-0,46716	-2,07671	5,25E-07	4,91E-06	2,3E-06	2700	tags=38%, list=17%, signal=32%
GO:000666	sphingolipid metabolic process	154	-0,42629	-2,00195	5,36E-07	4,98E-06	2,33E-06	3108	tags=34%, list=19%, signal=28%
GO:004410	cellular amine metabolic process	140	-0,44116	-2,04352	5,98E-07	5,51E-06	2,58E-06	2632	tags=32%, list=16%, signal=27%
GO:007066	leukocyte proliferation	267	0,331532	1,784361	6,41E-07	5,86E-06	2,74E-06	2737	tags=33%, list=17%, signal=28%
GO:003367	negative regulation of kinase activity	247	-0,37585	-1,87455	7,93E-07	7,19E-06	3,36E-06	3212	tags=32%, list=20%, signal=26%
GO:003459	cellular response to oxidative stress	272	-0,36563	-1,84394	1,02E-06	9,17E-06	4,29E-06	2542	tags=26%, list=16%, signal=22%
GO:000930	amine metabolic process	145	-0,43239	-2,01952	1,06E-06	9,49E-06	4,44E-06	2632	tags=32%, list=16%, signal=27%
GO:001063	epithelial cell migration	276	-0,36062	-1,82407	1,14E-06	1,01E-05	4,71E-06	2528	tags=26%, list=16%, signal=23%
GO:005134	negative regulation of transferase activity	275	-0,36634	-1,85256	1,22E-06	1,07E-05	5,03E-06	3212	tags=30%, list=20%, signal=25%
GO:003629	response to decreased oxygen levels	337	-0,34213	-1,7679	1,27E-06	1,11E-05	5,19E-06	2981	tags=28%, list=18%, signal=24%
GO:000222	innate immune response activating cell surface receptor signaling pathway	106	-0,47393	-2,099	1,45E-06	1,26E-05	5,88E-06	2700	tags=39%, list=17%, signal=32%
GO:003444	substrate adhesion-dependent cell spreading	106	-0,47386	-2,0987	1,48E-06	1,27E-05	5,95E-06	2080	tags=35%, list=13%, signal=31%
GO:007066	regulation of leukocyte proliferation	214	0,350424	1,849459	1,58E-06	1,35E-05	6,3E-06	2709	tags=34%, list=17%, signal=29%
GO:001050	regulation of autophagy	319	-0,34944	-1,79074	1,59E-06	1,35E-05	6,31E-06	3588	tags=37%, list=22%, signal=29%
GO:009013	epithelium migration	278	-0,35942	-1,81926	1,63E-06	1,37E-05	6,43E-06	2528	tags=26%, list=16%, signal=23%
GO:004209	T cell proliferation	175	0,37484	1,925995	1,69E-06	1,41E-05	6,6E-06	2311	tags=33%, list=14%, signal=28%
GO:190300	organelle disassembly	105	-0,46886	-2,07815	1,79E-06	1,49E-05	6,96E-06	3842	tags=46%, list=24%, signal=35%
GO:004212	regulation of T cell proliferation	148	0,381147	1,904911	1,84E-06	1,52E-05	7,1E-06	2311	tags=33%, list=14%, signal=29%
GO:000270	regulation of leukocyte mediated immunity	181	0,363787	1,891453	1,88E-06	1,54E-05	7,2E-06	2777	tags=33%, list=17%, signal=28%
GO:009031	positive regulation of intracellular protein transport	172	-0,40952	-1,94635	1,93E-06	1,57E-05	7,35E-06	4113	tags=44%, list=25%, signal=33%
GO:003450	protein localization to nucleus	266	-0,35836	-1,81007	1,95E-06	1,57E-05	7,37E-06	4182	tags=42%, list=26%, signal=32%
GO:007265	protein localization to plasma membrane	266	-0,35828	-1,80965	1,98E-06	1,58E-05	7,41E-06	2738	tags=28%, list=17%, signal=24%

GO:000611	oxidative phosphorylation	139	-0,42872	-1,98849	2,1E-06	1,67E-05	7,82E-06	3855	tags=47%, list=24%, signal=36%
GO:000224	hematopoietic progenitor cell differentiation	152	-0,40998	-1,91897	2,32E-06	1,84E-05	8,59E-06	3048	tags=36%, list=19%, signal=29%
GO:009015	establishment of protein localization to membrane	333	-0,34194	-1,76464	2,38E-06	1,87E-05	8,73E-06	4068	tags=35%, list=25%, signal=27%
GO:007008	glycosylation	248	-0,36546	-1,82483	2,47E-06	1,92E-05	9E-06	3857	tags=35%, list=24%, signal=27%
GO:200005	regulation of ubiquitin-dependent protein catabolic process	157	-0,41098	-1,9257	2,5E-06	1,94E-05	9,06E-06	4151	tags=45%, list=26%, signal=34%
GO:009013	tissue migration	282	-0,35427	-1,79449	2,52E-06	1,94E-05	9,07E-06	2528	tags=26%, list=16%, signal=23%
GO:000664	membrane lipid metabolic process	198	-0,38419	-1,8705	2,56E-06	1,96E-05	9,17E-06	4176	tags=39%, list=26%, signal=30%
GO:005090	detection of stimulus involved in sensory perception	100	0,449116	2,093802	2,6E-06	1,97E-05	9,23E-06	4510	tags=54%, list=28%, signal=39%
GO:004478	cilium organization	382	0,293149	1,653045	2,61E-06	1,97E-05	9,23E-06	3561	tags=33%, list=22%, signal=26%
GO:000695	humoral immune response	180	0,369288	1,910405	2,66E-06	1,99E-05	9,33E-06	3860	tags=39%, list=24%, signal=30%
GO:000166	response to hypoxia	326	-0,33901	-1,74525	2,84E-06	2,12E-05	9,92E-06	2981	tags=28%, list=18%, signal=23%
GO:003629	cellular response to decreased oxygen levels	197	-0,38904	-1,89072	2,96E-06	2,19E-05	1,03E-05	2930	tags=33%, list=18%, signal=27%
GO:007135	cellular response to tumor necrosis factor	263	-0,35275	-1,78004	3,31E-06	2,44E-05	1,14E-05	3943	tags=39%, list=24%, signal=30%
GO:005067	positive regulation of lymphocyte proliferation	119	0,406485	1,948888	3,59E-06	2,63E-05	1,23E-05	2311	tags=35%, list=14%, signal=30%
GO:000722	integrin-mediated signaling pathway	102	-0,4643	-2,04214	3,64E-06	2,64E-05	1,24E-05	3051	tags=41%, list=19%, signal=34%
GO:003461	response to tumor necrosis factor	283	-0,34695	-1,75543	3,71E-06	2,68E-05	1,25E-05	3943	tags=39%, list=24%, signal=30%
GO:004603	ATP metabolic process	292	-0,33994	-1,73471	4,16E-06	2,98E-05	1,4E-05	3864	tags=37%, list=24%, signal=29%
GO:000246	adaptive immune response based on somatic recombination of immune receptors built from immunoglobulin supe	230	0,332642	1,779357	4,19E-06	2,99E-05	1,4E-05	2791	tags=31%, list=17%, signal=26%
GO:000640	mRNA catabolic process	364	-0,32905	-1,7103	4,5E-06	3,19E-05	1,49E-05	3696	tags=32%, list=23%, signal=25%
GO:190331	regulation of mRNA metabolic process	320	-0,33284	-1,7048	4,96E-06	3,49E-05	1,63E-05	3879	tags=35%, list=24%, signal=27%
GO:001094	negative regulation of cell cycle process	319	-0,34109	-1,74796	5,11E-06	3,58E-05	1,67E-05	3538	tags=31%, list=22%, signal=25%
GO:003295	regulation of actin cytoskeleton organization	328	-0,32942	-1,69836	5,16E-06	3,59E-05	1,68E-05	3801	tags=35%, list=23%, signal=28%
GO:007145	cellular response to hypoxia	189	-0,38906	-1,87952	5,22E-06	3,62E-05	1,69E-05	2930	tags=33%, list=18%, signal=27%
GO:190351	mucopolysaccharide metabolic process	113	-0,43611	-1,95939	5,94E-06	4,09E-05	1,91E-05	2687	tags=35%, list=17%, signal=30%
GO:011005	regulation of actin filament organization	257	-0,34637	-1,74005	6,02E-06	4,12E-05	1,93E-05	2915	tags=30%, list=18%, signal=25%
GO:000269	regulation of immune effector process	350	0,294308	1,648575	6,11E-06	4,15E-05	1,94E-05	2777	tags=29%, list=17%, signal=24%
GO:006027	cilium assembly	363	0,293403	1,652488	6,59E-06	4,46E-05	2,08E-05	3561	tags=33%, list=22%, signal=27%
GO:009719	intrinsic apoptotic signaling pathway	269	-0,34607	-1,744	6,66E-06	4,48E-05	2,09E-05	4812	tags=45%, list=30%, signal=32%
GO:006201	regulation of small molecule metabolic process	392	-0,31705	-1,65848	7,18E-06	4,8E-05	2,24E-05	3791	tags=35%, list=23%, signal=27%
GO:000703	Golgi organization	133	-0,41507	-1,91803	7,55E-06	5,02E-05	2,35E-05	3186	tags=34%, list=20%, signal=27%
GO:199077	protein localization to cell periphery	318	-0,33595	-1,72194	7,94E-06	5,25E-05	2,46E-05	2010	tags=22%, list=12%, signal=19%
GO:004566	regulation of osteoblast differentiation	108	-0,43865	-1,95324	8,19E-06	5,38E-05	2,52E-05	2466	tags=31%, list=15%, signal=26%
GO:003294	positive regulation of mononuclear cell proliferation	120	0,404703	1,947183	8,25E-06	5,39E-05	2,52E-05	2311	tags=35%, list=14%, signal=30%
GO:004586	positive regulation of proteolysis	340	-0,32721	-1,69417	9,29E-06	6,04E-05	2,82E-05	3804	tags=36%, list=23%, signal=28%
GO:190336	positive regulation of cellular protein catabolic process	146	-0,40657	-1,89609	9,72E-06	6,22E-05	2,91E-05	3804	tags=43%, list=23%, signal=33%
GO:000648	protein glycosylation	237	-0,36093	-1,79245	9,73E-06	6,22E-05	2,91E-05	3311	tags=31%, list=20%, signal=25%
GO:004341	macromolecule glycosylation	237	-0,36093	-1,79245	9,73E-06	6,22E-05	2,91E-05	3311	tags=31%, list=20%, signal=25%
GO:004573	positive regulation of protein catabolic process	216	-0,3677	-1,80754	9,85E-06	6,26E-05	2,93E-05	3895	tags=39%, list=24%, signal=30%
GO:000270	regulation of lymphocyte mediated immunity	130	0,382366	1,862852	1E-05	6,32E-05	2,96E-05	2777	tags=35%, list=17%, signal=29%
GO:190332	regulation of protein modification by small protein conjugation or removal	230	-0,35493	-1,75616	1E-05	6,32E-05	2,96E-05	4198	tags=41%, list=26%, signal=31%
GO:005160	detection of stimulus	243	0,320167	1,720154	1,12E-05	6,98E-05	3,27E-05	3677	tags=35%, list=23%, signal=28%
GO:003432	cell junction assembly	394	-0,31168	-1,6278	1,13E-05	7,02E-05	3,29E-05	2915	tags=26%, list=18%, signal=22%
GO:190332	positive regulation of protein modification by small protein conjugation or removal	132	-0,42096	-1,94583	1,19E-05	7,35E-05	3,44E-05	3786	tags=44%, list=23%, signal=34%
GO:190290	regulation of supramolecular fiber organization	347	-0,32384	-1,67496	1,22E-05	7,54E-05	3,53E-05	3808	tags=36%, list=23%, signal=28%
GO:007066	positive regulation of leukocyte proliferation	129	0,387916	1,88269	1,23E-05	7,54E-05	3,53E-05	2311	tags=34%, list=14%, signal=29%
GO:000602	aminoglycan biosynthetic process	113	-0,42788	-1,92244	1,24E-05	7,55E-05	3,53E-05	3459	tags=45%, list=21%, signal=36%
GO:000863	apoptotic mitochondrial changes	112	-0,43755	-1,95808	1,26E-05	7,61E-05	3,56E-05	4151	tags=39%, list=26%, signal=29%
GO:005507	transition metal ion homeostasis	122	-0,41698	-1,89586	1,26E-05	7,61E-05	3,56E-05	4365	tags=48%, list=27%, signal=35%
GO:190199	regulation of mitotic cell cycle phase transition	398	-0,31377	-1,64514	1,3E-05	7,81E-05	3,65E-05	3538	tags=31%, list=22%, signal=25%

GO:000602	glycosaminoglycan biosynthetic process	107	-0,43235	-1,92197	1,4E-05	8,34E-05	3,9E-05	3459	tags=39%, list=21%, signal=31%
GO:003032	lung development	154	-0,39427	-1,85157	1,54E-05	9,14E-05	4,28E-05	3966	tags=41%, list=24%, signal=31%
GO:004427	sulfur compound biosynthetic process	180	-0,38713	-1,85846	1,59E-05	9,4E-05	4,4E-05	3502	tags=39%, list=22%, signal=31%
GO:190180	positive regulation of proteasomal protein catabolic process	106	-0,44876	-1,98751	1,67E-05	9,79E-05	4,58E-05	3804	tags=46%, list=23%, signal=36%
GO:000700	mitochondrial membrane organization	139	-0,40957	-1,89966	1,73E-05	0,000101	4,73E-05	4143	tags=42%, list=25%, signal=31%
GO:190495	positive regulation of establishment of protein localization	315	-0,32853	-1,68426	1,74E-05	0,000101	4,73E-05	3116	tags=30%, list=19%, signal=25%
GO:004358	skin development	239	-0,35051	-1,741	1,83E-05	0,000106	4,97E-05	3307	tags=28%, list=20%, signal=23%
GO:003032	respiratory tube development	158	-0,3903	-1,83111	2,09E-05	0,00012	5,63E-05	3966	tags=41%, list=24%, signal=31%
GO:003139	positive regulation of protein ubiquitination	113	-0,4204	-1,88883	2,63E-05	0,000151	7,05E-05	4198	tags=44%, list=26%, signal=33%
GO:000705	cell cycle arrest	219	-0,35826	-1,76095	2,65E-05	0,000151	7,08E-05	3226	tags=28%, list=20%, signal=23%
GO:003164	regulation of protein stability	276	-0,33579	-1,6985	2,79E-05	0,000158	7,41E-05	3558	tags=35%, list=22%, signal=28%
GO:007190	negative regulation of protein serine/threonine kinase activity	130	-0,40391	-1,8576	2,93E-05	0,000166	7,75E-05	3148	tags=32%, list=19%, signal=26%
GO:005087	positive regulation of T cell activation	193	0,336679	1,759549	2,97E-05	0,000167	7,82E-05	2311	tags=30%, list=14%, signal=26%
GO:000004	transition metal ion transport	115	-0,42854	-1,92773	3,11E-05	0,000174	8,15E-05	1388	tags=25%, list=9%, signal=23%
GO:000602	aminoglycan metabolic process	163	-0,38658	-1,82327	3,16E-05	0,000176	8,22E-05	4242	tags=42%, list=26%, signal=32%
GO:000703	vacuole organization	168	-0,37589	-1,78142	3,18E-05	0,000176	8,22E-05	4448	tags=48%, list=27%, signal=35%
GO:000008	G2/M transition of mitotic cell cycle	250	-0,34479	-1,72348	3,18E-05	0,000176	8,22E-05	2716	tags=27%, list=17%, signal=23%
GO:007149	cellular response to external stimulus	286	-0,33435	-1,69877	3,33E-05	0,000183	8,55E-05	2951	tags=29%, list=18%, signal=24%
GO:005124	regulation of lymphocyte activation	391	0,273615	1,54945	3,35E-05	0,000183	8,57E-05	2709	tags=28%, list=17%, signal=24%
GO:005109	regulation of binding	343	-0,31641	-1,63807	3,46E-05	0,000189	8,82E-05	3520	tags=34%, list=22%, signal=28%
GO:001063	regulation of epithelial cell migration	216	-0,35612	-1,75061	3,55E-05	0,000192	9E-05	2468	tags=25%, list=15%, signal=21%
GO:001038	regulation of G2/M transition of mitotic cell cycle	196	-0,36616	-1,78028	3,65E-05	0,000197	9,21E-05	2632	tags=29%, list=16%, signal=24%
GO:190305	positive regulation of proteolysis involved in cellular protein catabolic process	123	-0,4154	-1,89056	3,67E-05	0,000197	9,23E-05	3804	tags=44%, list=23%, signal=34%
GO:003253	regulation of cellular component size	355	-0,31154	-1,61573	3,83E-05	0,000204	9,55E-05	3791	tags=34%, list=23%, signal=26%
GO:004691	cellular transition metal ion homeostasis	102	-0,43672	-1,92084	3,84E-05	0,000204	9,55E-05	2890	tags=36%, list=18%, signal=30%
GO:004533	cellular respiration	178	-0,37336	-1,78406	4E-05	0,000212	9,91E-05	3855	tags=41%, list=24%, signal=32%
GO:003020	glycosaminoglycan metabolic process	151	-0,39141	-1,82816	4,02E-05	0,000212	9,92E-05	4242	tags=43%, list=26%, signal=32%
GO:003424	regulation of cellular amide metabolic process	397	-0,30581	-1,60379	4,1E-05	0,000215	0,000101	3593	tags=33%, list=22%, signal=26%
GO:003133	positive regulation of cellular catabolic process	365	-0,31292	-1,62903	4,18E-05	0,000218	0,000102	3804	tags=36%, list=23%, signal=28%
GO:004877	tissue remodeling	148	-0,38765	-1,8061	4,22E-05	0,00022	0,000103	2701	tags=30%, list=17%, signal=26%
GO:000221	activation of innate immune response	135	-0,39939	-1,84386	4,5E-05	0,000232	0,000109	2700	tags=35%, list=17%, signal=29%
GO:190274	regulation of cell cycle G2/M phase transition	212	-0,34825	-1,70964	4,51E-05	0,000232	0,000109	2632	tags=27%, list=16%, signal=23%
GO:001063	positive regulation of epithelial cell migration	142	-0,38824	-1,80267	4,52E-05	0,000232	0,000109	1995	tags=27%, list=12%, signal=24%
GO:190303	positive regulation of leukocyte cell-cell adhesion	211	0,317874	1,67374	4,55E-05	0,000232	0,000109	2311	tags=29%, list=14%, signal=25%
GO:000636	transcription initiation from RNA polymerase II promoter	182	-0,36613	-1,75584	4,86E-05	0,000247	0,000116	3582	tags=33%, list=22%, signal=26%
GO:004354	endothelial cell migration	204	-0,34871	-1,70344	5,03E-05	0,000255	0,000119	2160	tags=25%, list=13%, signal=22%
GO:000715	leukocyte cell-cell adhesion	321	0,27966	1,555917	5,44E-05	0,000274	0,000128	2718	tags=29%, list=17%, signal=24%
GO:003320	tumor necrosis factor-mediated signaling pathway	160	-0,37837	-1,78317	5,72E-05	0,000287	0,000134	2697	tags=32%, list=17%, signal=27%
GO:000716	establishment or maintenance of cell polarity	209	-0,35289	-1,72604	5,89E-05	0,000294	0,000138	2446	tags=27%, list=15%, signal=23%
GO:190547	positive regulation of protein localization to membrane	123	-0,41059	-1,86865	5,94E-05	0,000296	0,000138	3085	tags=33%, list=19%, signal=27%
GO:005086	regulation of T cell activation	291	0,292803	1,587005	6,07E-05	0,000301	0,000141	2311	tags=26%, list=14%, signal=23%
GO:006054	respiratory system development	177	-0,36172	-1,72883	6,16E-05	0,000304	0,000142	3966	tags=39%, list=24%, signal=30%
GO:003297	regulation of actin filament-based process	364	-0,30923	-1,60726	6,25E-05	0,000307	0,000144	3156	tags=29%, list=19%, signal=24%
GO:000720	positive regulation of cytosolic calcium ion concentration	266	0,299613	1,614313	6,39E-05	0,000313	0,000146	3577	tags=35%, list=22%, signal=27%
GO:009878	ncRNA transcription	107	-0,41487	-1,84426	6,49E-05	0,000316	0,000148	4272	tags=48%, list=26%, signal=35%
GO:004616	alcohol biosynthetic process	140	-0,38795	-1,79703	7,48E-05	0,000363	0,00017	3937	tags=39%, list=24%, signal=29%
GO:190260	proton transmembrane transport	136	-0,39657	-1,83081	7,64E-05	0,000369	0,000173	3816	tags=43%, list=23%, signal=33%
GO:005116	nuclear transport	327	-0,31232	-1,60831	8,02E-05	0,000386	0,000181	4435	tags=39%, list=27%, signal=29%
GO:190437	regulation of protein localization to cell periphery	114	-0,41398	-1,86451	8,59E-05	0,000412	0,000193	2825	tags=39%, list=17%, signal=32%

GO:190018	regulation of protein localization to nucleus	127	-0,40048	-1,83954	9,01E-05	0,00043	0,000201	4182	tags=45%, list=26%, signal=34%
GO:009875	detoxification	116	-0,4122	-1,8593	9,2E-05	0,000437	0,000205	2927	tags=34%, list=18%, signal=28%
GO:200124	regulation of intrinsic apoptotic signaling pathway	150	-0,3755	-1,75059	9,34E-05	0,000442	0,000207	4478	tags=42%, list=28%, signal=31%
GO:001969	ribose phosphate metabolic process	396	-0,29619	-1,5526	9,49E-05	0,000448	0,000209	3864	tags=33%, list=24%, signal=25%
GO:002290	electron transport chain	169	-0,37042	-1,75993	0,000103	0,000483	0,000226	4038	tags=43%, list=25%, signal=33%
GO:000170	in utero embryonic development	321	-0,30995	-1,59102	0,000104	0,000486	0,000227	3832	tags=31%, list=24%, signal=24%
GO:003050	BMP signaling pathway	135	-0,38879	-1,79496	0,000108	0,000505	0,000236	3371	tags=38%, list=21%, signal=30%
GO:000717	epidermal growth factor receptor signaling pathway	117	-0,40457	-1,8228	0,000109	0,000505	0,000236	2172	tags=26%, list=13%, signal=23%
GO:004483	cell cycle G2/M phase transition	268	-0,32727	-1,65301	0,000109	0,000505	0,000236	2716	tags=26%, list=17%, signal=22%
GO:005125	positive regulation of lymphocyte activation	259	0,291771	1,572811	0,000116	0,000533	0,000249	2311	tags=27%, list=14%, signal=24%
GO:006034	bone development	178	-0,36187	-1,72917	0,000116	0,000533	0,00025	2576	tags=30%, list=16%, signal=26%
GO:003133	positive regulation of protein-containing complex assembly	241	-0,33284	-1,65248	0,000117	0,000535	0,00025	3741	tags=36%, list=23%, signal=28%
GO:003812	ERBB signaling pathway	137	-0,39383	-1,82062	0,000124	0,000561	0,000262	2172	tags=26%, list=13%, signal=22%
GO:000641	regulation of translation	337	-0,30677	-1,58518	0,000124	0,000561	0,000262	3593	tags=33%, list=22%, signal=26%
GO:007177	response to BMP	147	-0,38145	-1,77576	0,000138	0,00062	0,00029	3371	tags=37%, list=21%, signal=29%
GO:007177	cellular response to BMP stimulus	147	-0,38145	-1,77576	0,000138	0,00062	0,00029	3371	tags=37%, list=21%, signal=29%
GO:000915	purine ribonucleotide metabolic process	372	-0,29547	-1,53913	0,00014	0,000627	0,000293	3864	tags=33%, list=24%, signal=26%
GO:004324	regulation of protein-containing complex disassembly	114	-0,40571	-1,82725	0,000142	0,000627	0,000293	3782	tags=39%, list=23%, signal=31%
GO:000759	blood coagulation	300	-0,31271	-1,5962	0,000142	0,000627	0,000293	2146	tags=21%, list=13%, signal=19%
GO:007121	cellular response to abiotic stimulus	300	-0,31267	-1,596	0,000142	0,000627	0,000293	4115	tags=39%, list=25%, signal=30%
GO:010400	cellular response to environmental stimulus	300	-0,31267	-1,596	0,000142	0,000627	0,000293	4115	tags=39%, list=25%, signal=30%
GO:190290	positive regulation of supramolecular fiber organization	192	-0,35164	-1,701	0,00015	0,000661	0,000309	1939	tags=38%, list=12%, signal=33%
GO:000647	protein dephosphorylation	302	-0,3114	-1,58831	0,000154	0,000673	0,000315	3070	tags=27%, list=19%, signal=22%
GO:005081	coagulation	302	-0,31097	-1,58611	0,000164	0,000714	0,000334	2146	tags=21%, list=13%, signal=19%
GO:000641	translational initiation	186	-0,35212	-1,69557	0,000165	0,000719	0,000337	4053	tags=34%, list=25%, signal=26%
GO:005148	regulation of cytosolic calcium ion concentration	297	0,28652	1,565337	0,000167	0,000724	0,000339	3577	tags=34%, list=22%, signal=27%
GO:000963	response to toxic substance	214	-0,33738	-1,65905	0,000169	0,000731	0,000342	2927	tags=27%, list=18%, signal=22%
GO:000691	nucleocytoplasmic transport	324	-0,30785	-1,58441	0,000171	0,000734	0,000344	4435	tags=39%, list=27%, signal=29%
GO:000854	epidermis development	272	-0,32473	-1,63768	0,000171	0,000734	0,000344	3068	tags=26%, list=19%, signal=22%
GO:004639	ribose phosphate biosynthetic process	179	-0,36189	-1,73171	0,000177	0,000756	0,000354	3537	tags=38%, list=22%, signal=30%
GO:000635	DNA-templated transcription, initiation	238	-0,3256	-1,61888	0,000185	0,000783	0,000366	3739	tags=32%, list=23%, signal=25%
GO:001648	cytosolic transport	163	-0,36563	-1,72445	0,000185	0,000783	0,000366	2788	tags=28%, list=17%, signal=24%
GO:001972	B cell mediated immunity	106	0,377987	1,781373	0,000185	0,000783	0,000366	2737	tags=33%, list=17%, signal=28%
GO:005126	protein depolymerization	107	-0,39795	-1,76903	0,000194	0,000817	0,000382	3782	tags=39%, list=23%, signal=30%
GO:004572	positive regulation of translation	121	-0,39375	-1,78787	0,0002	0,000837	0,000391	3543	tags=47%, list=22%, signal=37%
GO:001081	regulation of cell-substrate adhesion	207	-0,34376	-1,68078	0,000201	0,000838	0,000392	3335	tags=31%, list=21%, signal=25%
GO:004249	response to drug	329	-0,30781	-1,58566	0,000208	0,000867	0,000406	3391	tags=27%, list=21%, signal=22%
GO:001810	peptidyl-threonine phosphorylation	109	-0,39382	-1,75878	0,000215	0,000892	0,000417	4151	tags=41%, list=26%, signal=31%
GO:009723	cellular response to toxic substance	105	-0,4077	-1,80707	0,000219	0,000904	0,000423	2709	tags=33%, list=17%, signal=28%
GO:005086	positive regulation of cell activation	307	0,276847	1,523518	0,000221	0,000911	0,000426	2311	tags=27%, list=14%, signal=23%
GO:005117	import into nucleus	154	-0,36372	-1,70811	0,000223	0,000914	0,000428	4540	tags=44%, list=28%, signal=32%
GO:000756	aging	265	-0,31834	-1,60596	0,00024	0,000983	0,00046	2353	tags=22%, list=14%, signal=19%
GO:005082	protein stabilization	177	-0,34758	-1,66126	0,000252	0,001029	0,000481	3818	tags=38%, list=23%, signal=29%
GO:200006	positive regulation of ubiquitin-dependent protein catabolic process	101	-0,41076	-1,8115	0,000262	0,001064	0,000498	3804	tags=44%, list=23%, signal=34%
GO:000257	platelet degranulation	120	-0,39318	-1,78047	0,000269	0,001089	0,00051	3252	tags=37%, list=20%, signal=30%
GO:005122	positive regulation of protein transport	299	-0,31207	-1,59084	0,000272	0,001096	0,000513	3116	tags=29%, list=19%, signal=24%
GO:190303	regulation of leukocyte cell-cell adhesion	287	0,28298	1,537042	0,00028	0,001124	0,000526	2311	tags=25%, list=14%, signal=22%
GO:000759	hemostasis	303	-0,31079	-1,58663	0,000282	0,001128	0,000527	2146	tags=21%, list=13%, signal=19%
GO:003425	positive regulation of cellular amide metabolic process	148	-0,36576	-1,70408	0,000282	0,001128	0,000527	3543	tags=36%, list=22%, signal=29%

GO:000925	ribonucleotide metabolic process	387	-0,28883	-1,51048	0,00029	0,001155	0,00054	3864	tags=33%, list=24%, signal=25%
GO:000926	ribonucleotide biosynthetic process	173	-0,35281	-1,67838	0,000303	0,001203	0,000563	3537	tags=38%, list=22%, signal=30%
GO:190161	organic hydroxy compound biosynthetic process	220	-0,33375	-1,64191	0,000305	0,001207	0,000564	3857	tags=33%, list=24%, signal=25%
GO:001082	regulation of mitochondrion organization	148	-0,3641	-1,69634	0,000314	0,001238	0,000579	3588	tags=36%, list=22%, signal=29%
GO:004312	positive regulation of I-kappaB kinase/NF-kappaB signaling	174	-0,35148	-1,67783	0,000323	0,001266	0,000592	3754	tags=38%, list=23%, signal=29%
GO:001905	viral life cycle	322	-0,30274	-1,5555	0,000324	0,001266	0,000592	3937	tags=35%, list=24%, signal=27%
GO:001821	peptidyl-threonine modification	118	-0,38727	-1,74796	0,000325	0,001268	0,000593	4151	tags=41%, list=26%, signal=31%
GO:000915	purine ribonucleotide biosynthetic process	161	-0,35601	-1,67365	0,000337	0,001311	0,000613	3537	tags=39%, list=22%, signal=30%
GO:000703	vacuolar transport	149	-0,36334	-1,69408	0,00034	0,001316	0,000616	3966	tags=39%, list=24%, signal=30%
GO:000269	positive regulation of immune effector process	188	0,311782	1,625056	0,000341	0,001318	0,000617	2777	tags=31%, list=17%, signal=26%
GO:000914	nucleoside triphosphate metabolic process	101	-0,40624	-1,79155	0,000362	0,001394	0,000652	3913	tags=43%, list=24%, signal=33%
GO:000606	alcohol metabolic process	336	-0,29304	-1,51185	0,000373	0,001429	0,000669	3284	tags=27%, list=20%, signal=22%
GO:001606	immunoglobulin mediated immune response	103	0,36998	1,726753	0,000386	0,001475	0,00069	2145	tags=29%, list=13%, signal=25%
GO:003139	regulation of protein ubiquitination	198	-0,33723	-1,64188	0,000406	0,001546	0,000723	4198	tags=39%, list=26%, signal=29%
GO:001607	rRNA metabolic process	228	-0,32443	-1,6027	0,00041	0,001557	0,000729	4620	tags=40%, list=28%, signal=29%
GO:200123	positive regulation of apoptotic signaling pathway	113	-0,38125	-1,71293	0,000436	0,001651	0,000772	3247	tags=31%, list=20%, signal=25%
GO:190188	regulation of cell junction assembly	183	-0,3382	-1,62278	0,000438	0,001652	0,000773	2915	tags=28%, list=18%, signal=23%
GO:000652	cellular amino acid metabolic process	311	-0,30908	-1,57887	0,00045	0,001693	0,000792	2692	tags=26%, list=17%, signal=22%
GO:004851	spermatid differentiation	108	0,352949	1,660687	0,000453	0,0017	0,000795	3561	tags=36%, list=22%, signal=28%
GO:000270	positive regulation of leukocyte mediated immunity	106	0,364027	1,71558	0,000479	0,001791	0,000838	2777	tags=35%, list=17%, signal=29%
GO:005254	regulation of peptidase activity	371	-0,28954	-1,51011	0,000488	0,001817	0,00085	3085	tags=28%, list=19%, signal=23%
GO:002290	respiratory electron transport chain	112	-0,3876	-1,73458	0,000495	0,00184	0,000861	4038	tags=47%, list=25%, signal=36%
GO:004434	cellular response to fibroblast growth factor stimulus	124	-0,37095	-1,68846	0,000505	0,001869	0,000874	4265	tags=43%, list=26%, signal=32%
GO:001598	energy derivation by oxidation of organic compounds	261	-0,31802	-1,60061	0,000531	0,00196	0,000917	4258	tags=40%, list=26%, signal=30%
GO:005105	negative regulation of transport	363	-0,29557	-1,53458	0,000541	0,001992	0,000932	2911	tags=26%, list=18%, signal=22%
GO:004521	cell-cell junction organization	185	-0,33998	-1,6371	0,000548	0,002008	0,000939	3099	tags=30%, list=19%, signal=24%
GO:005149	positive regulation of cytoskeleton organization	208	-0,3329	-1,62429	0,000552	0,002018	0,000944	1939	tags=23%, list=12%, signal=21%
GO:003134	positive regulation of cell projection organization	326	-0,2965	-1,52641	0,000587	0,002138	0,001	3319	tags=27%, list=20%, signal=22%
GO:003033	DNA damage response, signal transduction by p53 class mediator	105	-0,39091	-1,73263	0,00059	0,002143	0,001003	2405	tags=27%, list=15%, signal=23%
GO:000636	rRNA processing	219	-0,32886	-1,61644	0,00062	0,002244	0,00105	4620	tags=42%, list=28%, signal=30%
GO:001072	positive regulation of cell development	269	-0,30373	-1,53064	0,00064	0,00231	0,001081	1977	tags=20%, list=12%, signal=18%
GO:190495	negative regulation of establishment of protein localization	109	-0,37735	-1,68524	0,000643	0,002313	0,001082	3359	tags=34%, list=21%, signal=27%
GO:000664	phospholipid metabolic process	389	-0,28827	-1,5056	0,00065	0,002334	0,001092	3708	tags=32%, list=23%, signal=25%
GO:000728	spermatid development	102	0,364497	1,702484	0,000722	0,002584	0,001209	3561	tags=37%, list=22%, signal=29%
GO:004211	B cell activation	221	0,282687	1,500723	0,00076	0,002712	0,001269	2118	tags=24%, list=13%, signal=22%
GO:000190	cell killing	125	0,339808	1,643231	0,000772	0,002746	0,001285	2791	tags=34%, list=17%, signal=28%
GO:000669	steroid biosynthetic process	161	-0,34591	-1,62615	0,000794	0,002816	0,001318	4262	tags=40%, list=26%, signal=30%
GO:001703	protein import	184	-0,33308	-1,5999	0,00083	0,002935	0,001373	4182	tags=38%, list=26%, signal=28%
GO:006104	regulation of wound healing	117	-0,376	-1,69406	0,000853	0,003002	0,001404	3335	tags=32%, list=21%, signal=25%
GO:003021	T cell differentiation	224	0,290775	1,541548	0,000854	0,003002	0,001404	2888	tags=29%, list=18%, signal=25%
GO:002240	positive regulation of cell-cell adhesion	251	0,27829	1,499964	0,000867	0,003038	0,001421	2373	tags=26%, list=15%, signal=22%
GO:000269	positive regulation of leukocyte activation	297	0,270283	1,476629	0,000873	0,003048	0,001426	2311	tags=26%, list=14%, signal=23%
GO:003300	muscle cell proliferation	166	-0,34393	-1,62541	0,000878	0,003056	0,00143	2779	tags=26%, list=17%, signal=22%
GO:000616	purine nucleotide metabolic process	396	-0,27684	-1,4512	0,000883	0,003066	0,001434	3864	tags=32%, list=24%, signal=25%
GO:000008	G1/S transition of mitotic cell cycle	240	-0,31048	-1,54366	0,000887	0,003069	0,001436	4374	tags=37%, list=27%, signal=27%
GO:000940	response to heat	154	-0,34616	-1,62563	0,000971	0,003353	0,001569	4512	tags=43%, list=28%, signal=31%
GO:005067	epithelial cell proliferation	353	-0,2846	-1,47358	0,001048	0,003606	0,001687	3307	tags=27%, list=20%, signal=22%
GO:004327	negative regulation of ion transport	259	-0,30191	-1,51795	0,001052	0,00361	0,001689	4093	tags=35%, list=25%, signal=27%
GO:003526	organ growth	140	-0,35103	-1,626	0,001061	0,00363	0,001698	1905	tags=36%, list=12%, signal=32%

GO:003264	tumor necrosis factor production	129	0,331485	1,60881	0,00114	0,00389	0,00182	2687	tags=34%, list=17%, signal=29%
GO:007097	protein localization to endoplasmic reticulum	141	-0,35091	-1,62544	0,00115	0,00391	0,001829	3972	tags=34%, list=24%, signal=26%
GO:001921	regulation of lipid metabolic process	366	-0,28418	-1,48029	0,001154	0,003913	0,001831	2787	tags=26%, list=17%, signal=22%
GO:005507	calcium ion homeostasis	396	0,246048	1,395771	0,001157	0,003913	0,001831	3577	tags=31%, list=22%, signal=24%
GO:004353	blood vessel endothelial cell migration	109	-0,36943	-1,64985	0,001171	0,003947	0,001846	1840	tags=24%, list=11%, signal=21%
GO:000701	microtubule-based movement	321	0,255481	1,421396	0,001225	0,004112	0,001924	2906	tags=28%, list=18%, signal=23%
GO:004663	alpha-beta T cell activation	131	0,33208	1,617099	0,001227	0,004112	0,001924	2673	tags=31%, list=16%, signal=26%
GO:000195	regulation of cell-matrix adhesion	118	-0,37046	-1,67207	0,001239	0,004133	0,001933	3335	tags=32%, list=21%, signal=26%
GO:006053	muscle tissue development	320	-0,28538	-1,46171	0,00124	0,004133	0,001933	2335	tags=29%, list=14%, signal=25%
GO:004689	regulation of lipid biosynthetic process	177	-0,32947	-1,57469	0,001258	0,004183	0,001957	4718	tags=46%, list=29%, signal=33%
GO:005086	regulation of B cell activation	112	0,34372	1,632655	0,001298	0,004303	0,002013	2709	tags=35%, list=17%, signal=29%
GO:003109	regeneration	172	-0,33633	-1,59851	0,001321	0,004366	0,002042	3288	tags=33%, list=20%, signal=27%
GO:190355	regulation of tumor necrosis factor superfamily cytokine production	131	0,330095	1,607434	0,001375	0,004532	0,00212	2687	tags=34%, list=17%, signal=29%
GO:007177	response to fibroblast growth factor	129	-0,35931	-1,65378	0,00138	0,004534	0,002121	4320	tags=42%, list=27%, signal=31%
GO:003009	lymphocyte differentiation	313	0,256626	1,416302	0,001468	0,004812	0,002251	2759	tags=27%, list=17%, signal=23%
GO:001003	response to metal ion	322	-0,28805	-1,48003	0,00148	0,004837	0,002263	3475	tags=29%, list=21%, signal=23%
GO:003133	negative regulation of protein-containing complex assembly	130	-0,35261	-1,62169	0,001489	0,004852	0,00227	2974	tags=32%, list=18%, signal=27%
GO:00640	RNA localization	223	-0,30287	-1,4929	0,001519	0,004938	0,00231	4472	tags=38%, list=28%, signal=28%
GO:003268	regulation of tumor necrosis factor production	127	0,336778	1,626474	0,001528	0,004952	0,002317	2687	tags=35%, list=17%, signal=29%
GO:005127	negative regulation of cellular component movement	265	-0,30154	-1,52122	0,001574	0,005086	0,002379	1826	tags=20%, list=11%, signal=18%
GO:003083	regulation of actin filament length	176	-0,32831	-1,56797	0,001599	0,005155	0,002412	3791	tags=38%, list=23%, signal=29%
GO:200014	negative regulation of cell motility	258	-0,29708	-1,49263	0,001608	0,005167	0,002417	1826	tags=19%, list=11%, signal=17%
GO:190303	regulation of response to wounding	146	-0,34342	-1,60158	0,001617	0,005182	0,002424	3335	tags=30%, list=21%, signal=24%
GO:000806	regulation of actin polymerization or depolymerization	175	-0,33124	-1,58118	0,001712	0,00546	0,002554	3791	tags=38%, list=23%, signal=29%
GO:000687	cellular calcium ion homeostasis	384	0,24796	1,396615	0,001713	0,00546	0,002554	3577	tags=31%, list=22%, signal=25%
GO:000283	positive regulation of response to biotic stimulus	223	-0,30105	-1,48393	0,001742	0,005539	0,002592	2700	tags=28%, list=17%, signal=24%
GO:000865	phospholipid biosynthetic process	257	-0,29613	-1,48764	0,001765	0,005595	0,002617	3940	tags=34%, list=24%, signal=26%
GO:007252	purine-containing compound biosynthetic process	187	-0,31689	-1,52853	0,001817	0,005747	0,002688	3537	tags=35%, list=22%, signal=28%
GO:005109	positive regulation of binding	168	-0,33037	-1,56571	0,001866	0,005884	0,002753	3520	tags=40%, list=22%, signal=32%
GO:003166	cellular response to extracellular stimulus	222	-0,31417	-1,54868	0,001962	0,006172	0,002887	2951	tags=27%, list=18%, signal=22%
GO:003286	cellular response to insulin stimulus	212	-0,30797	-1,51189	0,001978	0,006195	0,002898	3822	tags=34%, list=24%, signal=27%
GO:004339	regulation of protein binding	189	-0,32007	-1,54622	0,00198	0,006195	0,002898	3507	tags=30%, list=22%, signal=24%
GO:004311	receptor metabolic process	158	-0,33856	-1,58838	0,002036	0,006352	0,002972	2825	tags=28%, list=17%, signal=24%
GO:003021	keratinocyte differentiation	130	-0,34763	-1,59876	0,002049	0,006369	0,002979	4240	tags=37%, list=26%, signal=28%
GO:004873	cardiac muscle tissue development	175	-0,32758	-1,56373	0,002052	0,006369	0,002979	1861	tags=20%, list=11%, signal=18%
GO:005254	regulation of endopeptidase activity	345	-0,27622	-1,42931	0,002112	0,006538	0,003059	3247	tags=27%, list=20%, signal=22%
GO:005076	regulation of neurogenesis	317	-0,28852	-1,47867	0,002135	0,006592	0,003084	2651	tags=22%, list=16%, signal=19%
GO:002261	gland morphogenesis	106	-0,38101	-1,68746	0,002207	0,006795	0,003179	3226	tags=29%, list=20%, signal=24%
GO:007170	tumor necrosis factor superfamily cytokine production	133	0,325042	1,585734	0,002223	0,006826	0,003194	2687	tags=34%, list=17%, signal=28%
GO:003016	platelet activation	144	-0,33833	-1,57723	0,00225	0,006885	0,003221	2484	tags=28%, list=15%, signal=24%
GO:003010	regulation of endocytosis	192	-0,31936	-1,54484	0,002254	0,006885	0,003221	2855	tags=27%, list=18%, signal=23%
GO:001470	striated muscle tissue development	302	-0,28561	-1,45675	0,002293	0,006987	0,003269	2335	tags=20%, list=14%, signal=18%
GO:003027	regulation of ossification	100	-0,37909	-1,66256	0,002368	0,007195	0,003366	2652	tags=29%, list=16%, signal=24%
GO:004269	muscle cell differentiation	302	-0,28538	-1,45558	0,002374	0,007195	0,003366	3628	tags=31%, list=22%, signal=24%
GO:190313	mononuclear cell differentiation	360	0,247706	1,392476	0,002412	0,007291	0,003411	2759	tags=26%, list=17%, signal=22%
GO:000276	immune response-regulating signaling pathway	364	0,243733	1,370422	0,002444	0,007369	0,003447	2737	tags=29%, list=17%, signal=25%
GO:004312	regulation of I-kappaB kinase/NF-kappaB signaling	230	-0,30235	-1,49599	0,002452	0,007376	0,003451	3050	tags=29%, list=19%, signal=24%
GO:006102	membrane fusion	154	-0,3329	-1,56336	0,002531	0,007593	0,003552	3023	tags=30%, list=19%, signal=25%
GO:004484	cell cycle G1/S phase transition	257	-0,29129	-1,46335	0,002546	0,007618	0,003564	4374	tags=35%, list=27%, signal=26%

GO:00519€	regulation of nervous system development	386	-0,27657	-1,44621	0,002559	0,007636	0,003573	2651	tags=22%, list=16%, signal=19%
GO:00195	regulation of cytokine-mediated signaling pathway	148	-0,33677	-1,56901	0,00261	0,007769	0,003635	2268	tags=25%, list=14%, signal=22%
GO:00704	lysosomal transport	108	-0,35836	-1,59571	0,002669	0,007927	0,003709	2562	tags=28%, list=16%, signal=24%
GO:00450	positive regulation of innate immune response	197	-0,31335	-1,52285	0,002706	0,008017	0,00375	3098	tags=32%, list=19%, signal=27%
GO:00017€	morphogenesis of a branching structure	176	-0,32051	-1,53073	0,002873	0,008461	0,003958	3730	tags=30%, list=23%, signal=23%
GO:00003(	response to reactive oxygen species	205	-0,30728	-1,50257	0,002877	0,008461	0,003958	2374	tags=22%, list=15%, signal=19%
GO:00301	sphingolipid biosynthetic process	103	-0,37277	-1,64546	0,002878	0,008461	0,003958	2906	tags=30%, list=18%, signal=25%
GO:00464€	membrane lipid biosynthetic process	139	-0,33885	-1,57166	0,00298	0,008739	0,004088	2906	tags=27%, list=18%, signal=22%
GO:00512	negative regulation of lymphocyte activation	135	0,307643	1,507335	0,002997	0,008764	0,0041	2687	tags=32%, list=17%, signal=27%
GO:00066(	protein import into nucleus	134	-0,33951	-1,56864	0,003004	0,008764	0,0041	4540	tags=43%, list=28%, signal=31%
GO:00487	gland development	387	-0,26791	-1,40107	0,003065	0,008921	0,004174	3711	tags=28%, list=23%, signal=22%
GO:00161	sterol metabolic process	147	-0,33716	-1,56955	0,003081	0,008946	0,004185	3682	tags=35%, list=23%, signal=27%
GO:00482	organelle fusion	141	-0,33774	-1,56442	0,003125	0,009051	0,004234	3822	tags=36%, list=24%, signal=28%
GO:00512	negative regulation of protein transport	105	-0,36253	-1,60686	0,003142	0,009078	0,004247	3609	tags=35%, list=22%, signal=28%
GO:00521	movement in host environment	160	-0,32657	-1,53907	0,003184	0,009175	0,004292	3904	tags=39%, list=24%, signal=30%
GO:00020	sprouting angiogenesis	116	-0,361	-1,62835	0,003196	0,009186	0,004297	1809	tags=24%, list=11%, signal=22%
GO:00074	mesoderm development	105	-0,3617	-1,60316	0,003203	0,009186	0,004297	2227	tags=24%, list=14%, signal=21%
GO:00032(	cardiac chamber development	142	-0,33363	-1,54912	0,003212	0,009187	0,004298	2446	tags=26%, list=15%, signal=22%
GO:00081	actin polymerization or depolymerization	204	-0,29939	-1,46251	0,003258	0,009297	0,00435	3791	tags=35%, list=23%, signal=27%
GO:00082(	cholesterol metabolic process	135	-0,3366	-1,55399	0,003321	0,009455	0,004423	3937	tags=35%, list=24%, signal=27%
GO:00316€	cellular response to nutrient levels	199	-0,3064	-1,49293	0,003467	0,009845	0,004606	2848	tags=27%, list=18%, signal=22%
GO:00075€	female pregnancy	148	-0,33201	-1,54683	0,003564	0,010098	0,004724	4333	tags=40%, list=27%, signal=30%
GO:00094	response to UV	138	-0,33551	-1,55302	0,003587	0,010137	0,004742	3981	tags=36%, list=24%, signal=28%
GO:00303	negative regulation of cell migration	246	-0,29878	-1,4899	0,003615	0,010192	0,004768	1826	tags=20%, list=11%, signal=18%
GO:00072€	Ras protein signal transduction	324	-0,27653	-1,42322	0,003688	0,010371	0,004852	2358	tags=22%, list=15%, signal=19%
GO:00511	striated muscle cell differentiation	224	-0,30435	-1,50273	0,003771	0,010579	0,004949	2531	tags=25%, list=16%, signal=21%
GO:00901	organelle membrane fusion	107	-0,35227	-1,56596	0,003784	0,01059	0,004954	3023	tags=36%, list=19%, signal=29%
GO:00456	regulation of lymphocyte differentiation	158	0,298781	1,515034	0,003829	0,010691	0,005002	1887	tags=25%, list=12%, signal=22%
GO:00510	actin filament bundle assembly	146	-0,32755	-1,52756	0,003939	0,010974	0,005134	2915	tags=29%, list=18%, signal=24%
GO:00300(	cellular monovalent inorganic cation homeostasis	102	-0,36817	-1,61932	0,003975	0,011046	0,005168	1196	tags=21%, list=7%, signal=19%
GO:00063	nucleosome assembly	116	0,320228	1,527524	0,004028	0,011162	0,005222	4364	tags=33%, list=27%, signal=24%
GO:00065	cellular modified amino acid metabolic process	179	-0,31994	-1,53098	0,004036	0,011162	0,005222	2399	tags=23%, list=15%, signal=20%
GO:00026	negative regulation of leukocyte activation	160	0,292022	1,481144	0,004131	0,011399	0,005333	2736	tags=30%, list=17%, signal=25%
GO:00300	actin filament-based movement	134	0,308348	1,505838	0,004142	0,011403	0,005335	3388	tags=34%, list=21%, signal=27%
GO:00105	positive regulation of endothelial cell migration	103	-0,36416	-1,60749	0,004193	0,011517	0,005388	1995	tags=42%, list=12%, signal=37%
GO:00463	carboxylic acid catabolic process	231	0,262535	1,406743	0,004254	0,011657	0,005454	4573	tags=35%, list=28%, signal=25%
GO:00434(	regulation of MAP kinase activity	285	-0,28105	-1,42656	0,004415	0,01207	0,005647	3738	tags=32%, list=23%, signal=25%
GO:00518	regulation of protein kinase B signaling	203	-0,30203	-1,47333	0,004454	0,012148	0,005683	3793	tags=32%, list=23%, signal=25%
GO:00611	morphogenesis of a branching epithelium	163	-0,32121	-1,51498	0,004541	0,012357	0,005781	3730	tags=30%, list=23%, signal=23%
GO:00082(	steroid metabolic process	278	-0,28463	-1,44072	0,004663	0,012644	0,005915	4321	tags=34%, list=27%, signal=25%
GO:00028	regulation of adaptive immune response based on somatic recombination of immune receptors built from immuno	136	0,304755	1,494758	0,004668	0,012644	0,005915	2375	tags=27%, list=15%, signal=23%
GO:00032(	cardiac chamber morphogenesis	106	-0,36461	-1,61482	0,004684	0,012656	0,005921	2381	tags=26%, list=15%, signal=23%
GO:00063	chromatin assembly or disassembly	188	0,284243	1,481517	0,004725	0,012739	0,00596	4276	tags=30%, list=26%, signal=23%
GO:00061€	purine nucleotide biosynthetic process	176	-0,3148	-1,50344	0,00475	0,012747	0,005964	3537	tags=35%, list=22%, signal=28%
GO:00063	tRNA metabolic process	176	-0,31449	-1,50196	0,00475	0,012747	0,005964	3222	tags=28%, list=20%, signal=23%
GO:00159	nucleobase-containing compound transport	236	-0,29142	-1,44525	0,004769	0,012768	0,005973	4504	tags=38%, list=28%, signal=28%
GO:00507€	positive regulation of neurogenesis	203	-0,2991	-1,45903	0,004959	0,013245	0,006197	3826	tags=32%, list=24%, signal=24%
GO:00709	neuron death	319	-0,28166	-1,44339	0,005008	0,013301	0,006223	3108	tags=26%, list=19%, signal=21%
GO:00974	neuron projection guidance	262	-0,28504	-1,4353	0,005008	0,013301	0,006223	2911	tags=24%, list=18%, signal=20%

GO:004558	regulation of T cell differentiation	133	0,308181	1,503472	0,005023	0,013301	0,006223	4427	tags=41%, list=27%, signal=30%
GO:003447	ncRNA processing	378	-0,26871	-1,40214	0,005025	0,013301	0,006223	4705	tags=38%, list=29%, signal=27%
GO:004682	regulation of nucleocytoplasmic transport	100	-0,36588	-1,60462	0,005067	0,013382	0,006261	4534	tags=47%, list=28%, signal=34%
GO:000276	immune response-regulating cell surface receptor signaling pathway	361	0,239543	1,349182	0,005097	0,013432	0,006284	2737	tags=29%, list=17%, signal=24%
GO:000181	positive regulation of cytokine production	380	0,242069	1,36465	0,00525	0,013802	0,006457	2778	tags=26%, list=17%, signal=22%
GO:001059	regulation of endothelial cell migration	159	-0,31154	-1,46473	0,005261	0,013802	0,006457	2438	tags=38%, list=15%, signal=32%
GO:000633	chromatin remodeling	195	0,281763	1,477405	0,005354	0,014014	0,006556	4038	tags=30%, list=25%, signal=23%
GO:004870	skeletal system morphogenesis	197	-0,30423	-1,47856	0,00544	0,014192	0,00664	4452	tags=36%, list=27%, signal=26%
GO:006075	regulation of response to cytokine stimulus	158	-0,32225	-1,51186	0,005446	0,014192	0,00664	2268	tags=24%, list=14%, signal=21%
GO:004277	signal transduction in response to DNA damage	127	-0,33883	-1,55636	0,00547	0,014223	0,006654	2470	tags=24%, list=15%, signal=21%
GO:004470	multi-multicellular organism process	168	-0,31282	-1,48251	0,005669	0,014663	0,00686	4333	tags=38%, list=27%, signal=28%
GO:003083	regulation of actin filament polymerization	159	-0,30969	-1,45601	0,005673	0,014663	0,00686	3791	tags=36%, list=23%, signal=28%
GO:004860	reproductive structure development	358	-0,26433	-1,37113	0,005682	0,014663	0,00686	3711	tags=27%, list=23%, signal=21%
GO:190165	cellular response to peptide	352	-0,271	-1,40409	0,00569	0,014663	0,00686	3822	tags=33%, list=24%, signal=26%
GO:000661	protein export from nucleus	177	-0,30836	-1,47379	0,005709	0,014679	0,006868	4472	tags=40%, list=28%, signal=29%
GO:190265	secondary alcohol metabolic process	143	-0,3389	-1,57557	0,005855	0,015023	0,007028	3937	tags=35%, list=24%, signal=27%
GO:004866	regulation of smooth muscle cell proliferation	117	-0,34073	-1,53517	0,005903	0,015112	0,00707	2393	tags=26%, list=15%, signal=22%
GO:007137	cellular response to peptide hormone stimulus	293	-0,27654	-1,40816	0,005979	0,015273	0,007145	3822	tags=32%, list=24%, signal=25%
GO:003070	cytoskeleton-dependent intracellular transport	189	-0,30162	-1,45711	0,006071	0,015474	0,007239	2192	tags=22%, list=13%, signal=19%
GO:006145	reproductive system development	361	-0,26215	-1,3609	0,006125	0,015578	0,007288	3711	tags=26%, list=23%, signal=21%
GO:006157	actin filament bundle organization	149	-0,32472	-1,51401	0,006242	0,015842	0,007411	2915	tags=30%, list=18%, signal=24%
GO:000150	action potential	122	0,30741	1,481387	0,006303	0,015962	0,007467	3375	tags=33%, list=21%, signal=26%
GO:000281	regulation of adaptive immune response	150	0,293187	1,470267	0,00661	0,016681	0,007804	2375	tags=27%, list=15%, signal=23%
GO:004508	regulation of innate immune response	277	-0,28534	-1,44526	0,006615	0,016681	0,007804	2636	tags=27%, list=16%, signal=23%
GO:000716	negative regulation of cell adhesion	256	-0,2802	-1,40559	0,006662	0,016762	0,007842	1341	tags=16%, list=8%, signal=15%
GO:004206	gliogenesis	253	-0,28342	-1,41985	0,006702	0,016826	0,007872	2499	tags=27%, list=15%, signal=23%
GO:200037	regulation of reactive oxygen species metabolic process	168	-0,31081	-1,47296	0,006836	0,017127	0,008012	3850	tags=36%, list=24%, signal=28%
GO:003434	response to interferon-gamma	170	0,283684	1,461663	0,007235	0,018088	0,008462	3820	tags=34%, list=24%, signal=26%
GO:007259	reactive oxygen species metabolic process	240	-0,28487	-1,41633	0,00731	0,018235	0,008531	3577	tags=32%, list=22%, signal=25%
GO:001021	response to ionizing radiation	140	-0,32164	-1,48989	0,007561	0,018822	0,008805	3959	tags=39%, list=24%, signal=29%
GO:005189	positive regulation of protein kinase B signaling	152	-0,3125	-1,46272	0,007642	0,018959	0,00887	3793	tags=35%, list=23%, signal=27%
GO:004865	smooth muscle cell proliferation	119	-0,33727	-1,52496	0,007648	0,018959	0,00887	2779	tags=28%, list=17%, signal=23%
GO:005068	negative regulation of epithelial cell proliferation	114	-0,34089	-1,5353	0,007889	0,0195	0,009123	2160	tags=39%, list=13%, signal=34%
GO:004274	defense response to bacterium	155	0,282231	1,418935	0,0079	0,0195	0,009123	3797	tags=36%, list=23%, signal=28%
GO:000916	nucleotide biosynthetic process	238	-0,28581	-1,42107	0,007955	0,019596	0,009167	3537	tags=32%, list=22%, signal=26%
GO:000283	regulation of response to biotic stimulus	367	-0,26597	-1,38626	0,008288	0,020371	0,00953	3098	tags=28%, list=19%, signal=23%
GO:004001	negative regulation of locomotion	288	-0,27695	-1,40948	0,008359	0,020504	0,009593	1826	tags=18%, list=11%, signal=17%
GO:000694	striated muscle contraction	149	0,290933	1,455132	0,008394	0,020546	0,009612	3432	tags=34%, list=21%, signal=27%
GO:000726	Rho protein signal transduction	125	-0,33985	-1,55231	0,00845	0,020641	0,009657	1966	tags=22%, list=12%, signal=20%
GO:004254	response to hydrogen peroxide	125	-0,33953	-1,55088	0,00857	0,02089	0,009773	2374	tags=25%, list=15%, signal=21%
GO:190370	regulation of hemopoiesis	374	0,234501	1,322065	0,008821	0,021457	0,010038	3475	tags=28%, list=21%, signal=23%
GO:190129	nucleoside phosphate biosynthetic process	241	-0,28282	-1,40413	0,00884	0,021458	0,010039	3537	tags=32%, list=22%, signal=25%
GO:000941	response to xenobiotic stimulus	109	-0,33349	-1,48934	0,009136	0,02213	0,010353	1903	tags=18%, list=12%, signal=16%
GO:000741	axon guidance	261	-0,28662	-1,44255	0,009318	0,022508	0,01053	2911	tags=25%, list=18%, signal=20%
GO:004343	response to peptide hormone	393	-0,26237	-1,37061	0,00933	0,022508	0,01053	4930	tags=41%, list=30%, signal=29%
GO:000991	epidermal cell differentiation	181	-0,2992	-1,43327	0,009449	0,022749	0,010643	4240	tags=34%, list=26%, signal=26%
GO:003286	response to insulin	257	-0,27568	-1,3849	0,009522	0,022809	0,010671	4331	tags=37%, list=27%, signal=27%
GO:004340	negative regulation of MAPK cascade	158	-0,31383	-1,47235	0,009526	0,022809	0,010671	2607	tags=23%, list=16%, signal=20%
GO:000206	epithelial cell development	191	-0,29884	-1,44566	0,009533	0,022809	0,010671	4351	tags=41%, list=27%, signal=31%

GO:005170	biological process involved in interaction with host	198	-0,29121	-1,41781	0,010012	0,023905	0,011184	4143	tags=37%, list=25%, signal=28%
GO:001097	positive regulation of neuron projection development	144	-0,31153	-1,45226	0,010129	0,024101	0,011275	3290	tags=28%, list=20%, signal=23%
GO:005116	nuclear export	192	-0,30116	-1,45681	0,010135	0,024101	0,011275	4472	tags=39%, list=28%, signal=29%
GO:005085	T cell receptor signaling pathway	184	-0,30117	-1,44663	0,010313	0,024474	0,01145	1701	tags=22%, list=10%, signal=20%
GO:001605	organic acid catabolic process	246	0,254473	1,366923	0,01035	0,024512	0,011468	4573	tags=34%, list=28%, signal=25%
GO:000155	regulation of cell growth	381	-0,25878	-1,35185	0,010378	0,02453	0,011476	3901	tags=29%, list=24%, signal=22%
GO:004592	negative regulation of growth	226	-0,28883	-1,42372	0,010448	0,024646	0,01153	3627	tags=26%, list=22%, signal=21%
GO:000926	cellular response to starvation	150	-0,31268	-1,45774	0,010609	0,024963	0,011679	3044	tags=29%, list=19%, signal=24%
GO:003461	cellular response to reactive oxygen species	141	-0,31845	-1,47505	0,010625	0,024963	0,011679	1547	tags=32%, list=10%, signal=29%
GO:005128	regulation of sequestering of calcium ion	107	0,303888	1,428548	0,0107	0,025089	0,011737	3432	tags=36%, list=21%, signal=28%
GO:003085	regulation of epithelial cell differentiation	125	-0,33596	-1,53453	0,010848	0,025384	0,011875	3068	tags=32%, list=19%, signal=26%
GO:000242	immune response-activating cell surface receptor signaling pathway	334	0,234065	1,305913	0,010934	0,025483	0,011922	2737	tags=28%, list=17%, signal=24%
GO:000275	immune response-activating signal transduction	334	0,234065	1,305913	0,010934	0,025483	0,011922	2737	tags=28%, list=17%, signal=24%
GO:004576	positive regulation of angiogenesis	138	-0,31698	-1,46724	0,011077	0,025716	0,012031	2779	tags=28%, list=17%, signal=23%
GO:190401	positive regulation of vasculature development	138	-0,31698	-1,46724	0,011077	0,025716	0,012031	2779	tags=28%, list=17%, signal=23%
GO:190165	response to ketone	173	-0,30131	-1,43336	0,011107	0,025734	0,012039	2387	tags=21%, list=15%, signal=18%
GO:000091	cytokinesis	160	-0,30765	-1,44986	0,011447	0,026437	0,012368	1840	tags=28%, list=11%, signal=25%
GO:200004	regulation of G1/S transition of mitotic cell cycle	142	-0,31251	-1,45105	0,011456	0,026437	0,012368	4374	tags=38%, list=27%, signal=28%
GO:003313	regulation of peptidyl-serine phosphorylation	117	-0,32815	-1,47848	0,011503	0,026492	0,012394	3238	tags=34%, list=20%, signal=28%
GO:003472	nucleosome organization	149	0,284479	1,422849	0,011554	0,026558	0,012425	3040	tags=23%, list=19%, signal=19%
GO:003149	chromatin assembly	164	0,279509	1,428606	0,01197	0,027461	0,012847	4364	tags=31%, list=27%, signal=23%
GO:003134	positive regulation of defense response	312	-0,27193	-1,39179	0,012051	0,027593	0,012909	2636	tags=26%, list=16%, signal=22%
GO:005196	positive regulation of nervous system development	246	-0,28087	-1,40057	0,012152	0,027768	0,012991	2882	tags=24%, list=18%, signal=20%
GO:003460	cellular response to heat	116	-0,33485	-1,51039	0,012427	0,028343	0,01326	4472	tags=43%, list=28%, signal=31%
GO:190121	regulation of neuron death	281	-0,27276	-1,38067	0,012504	0,028462	0,013315	3108	tags=25%, list=19%, signal=20%
GO:005076	negative regulation of neurogenesis	118	-0,32474	-1,46575	0,012709	0,028874	0,013508	2616	tags=24%, list=16%, signal=20%
GO:003030	negative regulation of cell growth	173	-0,29816	-1,41838	0,013208	0,029948	0,014011	3627	tags=27%, list=22%, signal=21%
GO:005140	neuron apoptotic process	216	-0,28704	-1,41105	0,013321	0,030147	0,014104	3108	tags=25%, list=19%, signal=20%
GO:002153	telencephalon development	215	-0,27678	-1,3613	0,013611	0,030743	0,014383	3013	tags=23%, list=19%, signal=19%
GO:005090	leukocyte migration	390	-0,25409	-1,32765	0,013742	0,030978	0,014493	2370	tags=20%, list=15%, signal=18%
GO:000165	eye development	313	-0,26222	-1,344	0,013865	0,031145	0,014571	3822	tags=31%, list=24%, signal=24%
GO:000724	I-kappaB kinase/NF-kappaB signaling	265	-0,27601	-1,39242	0,013869	0,031145	0,014571	3050	tags=28%, list=19%, signal=23%
GO:007025	actin-mediated cell contraction	105	0,307641	1,440582	0,014039	0,031467	0,014722	3388	tags=34%, list=21%, signal=27%
GO:005073	regulation of peptidyl-tyrosine phosphorylation	228	-0,27904	-1,37846	0,014125	0,031555	0,014762	1373	tags=17%, list=8%, signal=16%
GO:000632	DNA packaging	199	0,255238	1,340024	0,014132	0,031555	0,014762	4276	tags=29%, list=26%, signal=21%
GO:000165	urogenital system development	302	-0,26349	-1,34397	0,014264	0,031788	0,014872	3769	tags=28%, list=23%, signal=22%
GO:007146	cellular response to xenobiotic stimulus	103	-0,34237	-1,51129	0,014466	0,032178	0,015054	1439	tags=17%, list=9%, signal=15%
GO:190379	negative regulation of anion transport	177	-0,29677	-1,41844	0,014571	0,032349	0,015134	3979	tags=34%, list=24%, signal=26%
GO:004301	camera-type eye development	268	-0,27703	-1,39923	0,014678	0,032525	0,015217	3822	tags=32%, list=24%, signal=25%
GO:009730	response to alcohol	209	-0,28387	-1,38848	0,01478	0,03269	0,015293	2463	tags=20%, list=15%, signal=17%
GO:001972	calcium-mediated signaling	174	0,275671	1,415366	0,015077	0,033275	0,015567	4687	tags=44%, list=29%, signal=32%
GO:000760	sensory perception of sound	134	0,287027	1,401716	0,01511	0,033275	0,015567	5596	tags=49%, list=34%, signal=32%
GO:000676	vitamin metabolic process	117	-0,32282	-1,45446	0,01513	0,033275	0,015567	2135	tags=21%, list=13%, signal=18%
GO:001072	negative regulation of cell development	154	-0,30614	-1,43771	0,015618	0,034284	0,016039	2616	tags=24%, list=16%, signal=20%
GO:002260	regulation of cell morphogenesis	297	-0,26751	-1,36312	0,015697	0,034392	0,01609	2925	tags=25%, list=18%, signal=21%
GO:004349	protein kinase B signaling	229	-0,28326	-1,40124	0,016159	0,035337	0,016532	3793	tags=31%, list=23%, signal=24%
GO:005128	negative regulation of sequestering of calcium ion	105	0,304915	1,427819	0,016301	0,035518	0,016617	3432	tags=36%, list=21%, signal=29%
GO:004390	regulation of biological process involved in symbiotic interaction	179	-0,29908	-1,43114	0,016302	0,035518	0,016617	3904	tags=34%, list=24%, signal=26%
GO:004586	negative regulation of proteolysis	269	-0,26475	-1,33422	0,017026	0,037026	0,017322	3524	tags=29%, list=22%, signal=23%

GO:00353(	regulation of dephosphorylation	194	-0,29035	-1,41173	0,017088	0,037093	0,017354	3062	tags=25%, list=19%, signal=21%
GO:00083€	regulation of cell size	168	-0,29299	-1,38855	0,017262	0,037402	0,017498	3680	tags=32%, list=23%, signal=25%
GO:00511(	regulation of DNA binding	115	-0,32729	-1,47226	0,017637	0,038142	0,017844	3013	tags=26%, list=19%, signal=21%
GO:000661	protein targeting to membrane	192	-0,29231	-1,414	0,017838	0,038506	0,018014	4068	tags=31%, list=25%, signal=23%
GO:00068S	post-Golgi vesicle-mediated transport	103	-0,33936	-1,49802	0,018175	0,039163	0,018322	4529	tags=45%, list=28%, signal=32%
GO:190303	negative regulation of leukocyte cell-cell adhesion	114	0,285391	1,357272	0,018318	0,039398	0,018432	2594	tags=27%, list=16%, signal=23%
GO:001921	regulation of steroid metabolic process	106	-0,33903	-1,50152	0,01867	0,04008	0,018751	4368	tags=42%, list=27%, signal=31%
GO:005123	maintenance of location	297	-0,26405	-1,34547	0,019377	0,041523	0,019426	1868	tags=17%, list=11%, signal=15%
GO:001095	positive regulation of peptidase activity	181	-0,28684	-1,37405	0,02039	0,043606	0,0204	3085	tags=28%, list=19%, signal=23%
GO:000323	cardiac ventricle development	106	-0,33526	-1,48485	0,020424	0,043606	0,0204	2990	tags=31%, list=18%, signal=26%
GO:00310S	stress-activated protein kinase signaling cascade	260	-0,26715	-1,34371	0,02073	0,044179	0,020668	3763	tags=32%, list=23%, signal=25%
GO:00069S	nucleus organization	114	-0,31969	-1,43982	0,02094	0,044545	0,02084	5405	tags=51%, list=33%, signal=34%
GO:00488E	sensory system development	321	-0,25328	-1,30012	0,021759	0,046147	0,021589	3822	tags=30%, list=24%, signal=24%
GO:003505	cardiocyte differentiation	115	-0,32386	-1,45686	0,021772	0,046147	0,021589	2335	tags=33%, list=14%, signal=28%
GO:003121	biomineral tissue development	139	-0,30093	-1,39576	0,021857	0,046243	0,021634	2652	tags=24%, list=16%, signal=21%
GO:00070⁄	cell-cell junction assembly	125	-0,31877	-1,45604	0,022044	0,046554	0,02178	2010	tags=23%, list=12%, signal=20%
GO:001097	transport along microtubule	153	-0,30062	-1,40787	0,0225	0,047432	0,022191	1997	tags=20%, list=12%, signal=18%
GO:005067	regulation of epithelial cell proliferation	305	-0,25615	-1,30781	0,022945	0,048285	0,022589	3307	tags=25%, list=20%, signal=20%
GO:005065	nucleic acid transport	187	-0,28037	-1,35237	0,023219	0,048685	0,022776	4472	tags=37%, list=28%, signal=27%
GO:005065	RNA transport	187	-0,28037	-1,35237	0,023219	0,048685	0,022776	4472	tags=37%, list=28%, signal=27%
GO:190165	glycosyl compound metabolic process	118	-0,31249	-1,41042	0,023361	0,048895	0,022875	2608	tags=25%, list=16%, signal=21%
GO:004215	lipoprotein metabolic process	124	-0,31036	-1,41267	0,023565	0,049234	0,023033	3844	tags=31%, list=24%, signal=24%
GO:004352	regulation of neuron apoptotic process	185	-0,28369	-1,36606	0,023679	0,049383	0,023103	2147	tags=18%, list=13%, signal=16%
GO:00716S	protein localization to extracellular region	343	-0,25324	-1,31104	0,023794	0,049536	0,023175	2911	tags=23%, list=18%, signal=19%
GO:00508€	negative regulation of T cell activation	103	0,298597	1,393598	0,02393	0,04973	0,023265	2594	tags=29%, list=16%, signal=25%

Supplementary Table 6: GRmetrics

aPmP_num	time	agent	concentrat	treatment_ID	mean_cell_	SEM_cell_c	mean_RLU	SEM_RLU_	cell_count_	cell_count_	ctrl_cell_do	mean_rel_	SEM_rel_co	GR_AOC
aP321m	96	Cisplatin	0,106667	CPT 0.11uM	1,40186	0,140315	130,3583	13,04783	1,075643	1	0,1052	1	0	0,338108
aP321m	96	Cisplatin	0,533333	CPT 0.53uM	1,112147	0,065731	103,4179	6,112283	1,075643	1	0,1052	0,967131	0,019915	0,338108
aP321m	96	Cisplatin	2,666667	CPT 2.67uM	1,298487	0,164462	120,7456	15,29327	1,075643	1	0,1052	0,967795	0,032205	0,338108
aP321m	96	Cisplatin	13,33333	CPT 13.33uM	1,383813	0,045983	128,68	4,275922	1,075643	1	0,1052	1	0	0,338108
aP321m	96	Cisplatin	66,66667	CPT 66.67uM	0,939524	0,095564	87,36583	8,886438	1,075643	1	0,1052	0,873453	0,088843	0,338108
aP321m	96	Temozolomide	0,46	TEM 0.46uM	0,652495	0,049823	60,67521	4,633053	1,075643	1	0,1052	0,606609	0,04632	1,652121
aP321m	96	Temozolomide	2,3	TEM 2.30uM	0,750113	0,060499	69,75261	5,625788	1,075643	1	0,1052	0,697362	0,056245	1,652121
aP321m	96	Temozolomide	11,52	TEM 11.52uM	0,570604	0,047074	53,06014	4,377367	1,075643	1	0,1052	0,530477	0,043763	1,652121
aP321m	96	Temozolomide	57,6	TEM 57.60uM	0,993818	0,062451	92,41463	5,80727	1,075643	1	0,1052	0,918835	0,055401	1,652121
aP321m	96	Temozolomide	288	TEM 288.00uM	0,629529	0,035515	58,53959	3,302517	1,075643	1	0,1052	0,585258	0,033017	1,652121
aP321m	168	Cisplatin	0,106667	CPT 0.11uM	1,570853	0,048214	118,501	3,637164	1,290008	1	0,36738	1	0	0,297699
aP321m	168	Cisplatin	0,533333	CPT 0.53uM	1,183337	0,054291	89,26785	4,095601	1,290008	1	0,36738	0,91731	0,042086	0,297699
aP321m	168	Cisplatin	2,666667	CPT 2.67uM	1,258774	0,138378	94,95867	10,43889	1,290008	1	0,36738	0,920803	0,079197	0,297699
aP321m	168	Cisplatin	13,33333	CPT 13.33uM	1,391371	0,014447	104,9614	1,089838	1,290008	1	0,36738	1	0	0,297699
aP321m	168	Cisplatin	66,66667	CPT 66.67uM	1,007848	0,112138	76,02946	8,459389	1,290008	1	0,36738	0,781273	0,086928	0,297699
aP321m	168	Temozolomide	0,46	TEM 0.46uM	0,665095	0,055278	50,173	4,170029	1,290008	1	0,36738	0,515574	0,042851	1,485616
aP321m	168	Temozolomide	2,3	TEM 2.30uM	0,713024	0,064227	53,7887	4,845096	1,290008	1	0,36738	0,552729	0,049788	1,485616
aP321m	168	Temozolomide	11,52	TEM 11.52uM	0,56374	0,031407	42,52705	2,369281	1,290008	1	0,36738	0,437005	0,024347	1,485616
aP321m	168	Temozolomide	57,6	TEM 57.60uM	1,005659	0,087174	75,86428	6,576155	1,290008	1	0,36738	0,779576	0,067576	1,485616
aP321m	168	Temozolomide	288	TEM 288.00uM	0,678026	0,057328	51,14849	4,324665	1,290008	1	0,36738	0,525598	0,04444	1,485616
aP490m	96	Cisplatin	0,106667	CPT 0.11uM	1,746474	0,216731	112,4468	13,95422	1,532408	1	0,6158	0,965875	0,034125	0,266454
aP490m	96	Cisplatin	0,533333	CPT 0.53uM	1,724501	0,17352	111,032	11,17208	1,532408	1	0,6158	0,957467	0,042533	0,266454
aP490m	96	Cisplatin	2,666667	CPT 2.67uM	1,719438	0,221872	110,7061	14,28527	1,532408	1	0,6158	0,933541	0,066459	0,266454
aP490m	96	Cisplatin	13,33333	CPT 13.33uM	1,70985	0,253978	110,0887	16,35236	1,532408	1	0,6158	0,931699	0,04051	0,266454
aP490m	96	Cisplatin	66,66667	CPT 66.67uM	1,028135	0,073074	66,1965	4,704872	1,532408	1	0,6158	0,670928	0,047686	0,266454
aP490m	96	Temozolomide	0,46	TEM 0.46uM	1,177591	0,105759	75,81928	6,809327	1,532408	1	0,6158	0,768458	0,069015	0,718847
aP490m	96	Temozolomide	2,3	TEM 2.30uM	1,248269	0,191138	80,36983	12,30644	1,532408	1	0,6158	0,807093	0,118564	0,718847
aP490m	96	Temozolomide	11,52	TEM 11.52uM	1,012507	0,115666	65,1903	7,447155	1,532408	1	0,6158	0,66073	0,07548	0,718847
aP490m	96	Temozolomide	57,6	TEM 57.60uM	1,130248	0,108528	72,77108	6,987603	1,532408	1	0,6158	0,737564	0,070822	0,718847
aP490m	96	Temozolomide	288	TEM 288.00uM	1,283722	0,128496	82,65249	8,273237	1,532408	1	0,6158	0,837716	0,083853	0,718847
aP490m	168	Cisplatin	0,106667	CPT 0.11uM	1,778674	0,18396	105,8049	10,94293	1,691237	1	0,758079	0,942461	0,057161	0,485406
aP490m	168	Cisplatin	0,533333	CPT 0.53uM	1,740798	0,163254	103,5519	9,711236	1,691237	1	0,758079	0,932019	0,039971	0,485406
aP490m	168	Cisplatin	2,666667	CPT 2.67uM	1,395636	0,113376	83,01984	6,7442	1,691237	1	0,758079	0,825216	0,067037	0,485406
aP490m	168	Cisplatin	13,33333	CPT 13.33uM	1,249164	0,157183	74,3069	9,350079	1,691237	1	0,758079	0,73861	0,09294	0,485406
aP490m	168	Cisplatin	66,66667	CPT 66.67uM	0,832725	0,052848	49,53486	3,143702	1,691237	1	0,758079	0,492376	0,031248	0,485406
aP490m	168	Temozolomide	0,46	TEM 0.46uM	1,302661	0,135842	77,48918	8,080572	1,691237	1	0,758079	0,770242	0,080321	0,742522
aP490m	168	Temozolomide	2,3	TEM 2.30uM	1,279007	0,177788	76,08211	10,57578	1,691237	1	0,758079	0,756255	0,105123	0,742522

aP490m	168	Temozolomide	11,52	TEM 11.52uM	1,073125	0,175128	63,83517	10,41754	1,691237	1	0,758079	0,634521	0,10355	0,742522
aP490m	168	Temozolomide	57,6	TEM 57.60uM	1,170525	0,206018	69,62901	12,25506	1,691237	1	0,758079	0,692112	0,121815	0,742522
aP490m	168	Temozolomide	288	TEM 288.00uM	1,095783	0,140905	65,18298	8,381782	1,691237	1	0,758079	0,647918	0,083315	0,742522
C0701m	96	Cisplatin	0,106667	CPT 0.11uM	1,907578	0,212363	123,1148	13,70585	1,487453	1	0,572844	0,98509	0,01491	0,441121
C0701m	96	Cisplatin	0,533333	CPT 0.53uM	1,835637	0,149619	118,4718	9,656421	1,487453	1	0,572844	0,990041	0,009959	0,441121
C0701m	96	Cisplatin	2,666667	CPT 2.67uM	1,796616	0,082362	115,9534	5,315632	1,487453	1	0,572844	1	0	0,441121
C0701m	96	Cisplatin	13,33333	CPT 13.33uM	1,082358	0,16623	69,85526	10,72846	1,487453	1	0,572844	0,727659	0,111755	0,441121
C0701m	96	Cisplatin	66,66667	CPT 66.67uM	0,335533	0,126031	21,65526	8,134042	1,487453	1	0,572844	0,225576	0,08473	0,441121
C0701m	96	Temozolomide	0,46	TEM 0.46uM	1,961322	0,048636	126,5835	3,138988	1,487453	1	0,572844	1	0	0,0868
C0701m	96	Temozolomide	2,3	TEM 2.30uM	1,721877	0,109619	111,1298	7,074792	1,487453	1	0,572844	0,994572	0,005428	0,0868
C0701m	96	Temozolomide	11,52	TEM 11.52uM	1,671758	0,06217	107,8951	4,012434	1,487453	1	0,572844	1	0	0,0868
C0701m	96	Temozolomide	57,6	TEM 57.60uM	1,534027	0,065316	99,00591	4,215486	1,487453	1	0,572844	0,988626	0,009394	0,0868
C0701m	96	Temozolomide	288	TEM 288.00uM	1,28049	0,162473	82,64264	10,48599	1,487453	1	0,572844	0,816247	0,066898	0,0868
C0701m	168	Cisplatin	0,106667	CPT 0.11uM	2,937058	0,211724	134,2114	9,67491	2,083683	1	1,059136	1	0	0,408215
C0701m	168	Cisplatin	0,533333	CPT 0.53uM	2,449889	0,241038	111,9498	11,01444	2,083683	1	1,059136	0,968929	0,031071	0,408215
C0701m	168	Cisplatin	2,666667	CPT 2.67uM	2,36763	0,118119	108,1909	5,397563	2,083683	1	1,059136	1	0	0,408215
C0701m	168	Cisplatin	13,33333	CPT 13.33uM	1,241711	0,187499	56,74105	8,567913	2,083683	1	1,059136	0,595921	0,089984	0,408215
C0701m	168	Cisplatin	66,66667	CPT 66.67uM	0,386329	0,151949	17,65364	6,943431	2,083683	1	1,059136	0,185407	0,072923	0,408215
C0701m	168	Temozolomide	0,46	TEM 0.46uM	2,482911	0,054117	113,4587	2,472914	2,083683	1	1,059136	1	0	0,108475
C0701m	168	Temozolomide	2,3	TEM 2.30uM	2,261039	0,091088	103,3201	4,162323	2,083683	1	1,059136	1	0	0,108475
C0701m	168	Temozolomide	11,52	TEM 11.52uM	2,248726	0,113201	102,7575	5,172825	2,083683	1	1,059136	0,996201	0,003799	0,108475
C0701m	168	Temozolomide	57,6	TEM 57.60uM	1,872324	0,10564	85,55744	4,827327	2,083683	1	1,059136	0,892818	0,046117	0,108475
C0701m	168	Temozolomide	288	TEM 288.00uM	1,692493	0,273746	77,33991	12,50906	2,083683	1	1,059136	0,765888	0,088723	0,108475
C3301m	96	Cisplatin	0,106667	CPT 0.11uM	1,132272	0,095048	94,47864	7,930925	1,180554	1	0,239463	0,916383	0,047704	1,223286
C3301m	96	Cisplatin	0,533333	CPT 0.53uM	1,012034	0,004011	84,44583	0,334726	1,180554	1	0,239463	0,857254	0,003398	1,223286
C3301m	96	Cisplatin	2,666667	CPT 2.67uM	0,962235	0,063208	80,29051	5,274216	1,180554	1	0,239463	0,815071	0,053541	1,223286
C3301m	96	Cisplatin	13,33333	CPT 13.33uM	0,793132	0,037755	66,18024	3,150313	1,180554	1	0,239463	0,671831	0,03198	1,223286
C3301m	96	Cisplatin	66,66667	CPT 66.67uM	0,246412	0,018275	20,56099	1,524932	1,180554	1	0,239463	0,208725	0,01548	1,223286
C3301m	96	Temozolomide	0,46	TEM 0.46uM	1,113725	0,043351	92,93104	3,61731	1,180554	1	0,239463	0,94026	0,033934	1,024945
C3301m	96	Temozolomide	2,3	TEM 2.30uM	0,979541	0,06349	81,73453	5,297688	1,180554	1	0,239463	0,82973	0,05378	1,024945
C3301m	96	Temozolomide	11,52	TEM 11.52uM	0,983755	0,105495	82,08616	8,802701	1,180554	1	0,239463	0,80811	0,064253	1,024945
C3301m	96	Temozolomide	57,6	TEM 57.60uM	0,932016	0,094917	77,769	7,920068	1,180554	1	0,239463	0,789474	0,080401	1,024945
C3301m	96	Temozolomide	288	TEM 288.00uM	0,835061	0,085019	69,67889	7,094122	1,180554	1	0,239463	0,707347	0,072016	1,024945
C3301m	168	Cisplatin	0,106667	CPT 0.11uM	1,025345	0,074748	96,31204	7,021208	1,055361	1	0,077737	0,927884	0,031768	1,484359
C3301m	168	Cisplatin	0,533333	CPT 0.53uM	0,969246	0,040339	91,04259	3,789083	1,055361	1	0,077737	0,911792	0,032071	1,484359
C3301m	168	Cisplatin	2,666667	CPT 2.67uM	0,921734	0,066703	86,57966	6,265478	1,055361	1	0,077737	0,865062	0,056365	1,484359
C3301m	168	Cisplatin	13,33333	CPT 13.33uM	0,789096	0,026753	74,1208	2,512963	1,055361	1	0,077737	0,747702	0,02535	1,484359
C3301m	168	Cisplatin	66,66667	CPT 66.67uM	0,279086	0,03985	26,21488	3,7432	1,055361	1	0,077737	0,264446	0,03776	1,484359

C3301m	168	Temozolomide	0,46	TEM 0.46uM	1,05504	0,072878	99,10129	6,845573	1,055361	1	0,077737	0,956668	0,043332	1,197946
C3301m	168	Temozolomide	2,3	TEM 2.30uM	0,910873	0,048975	85,55953	4,600311	1,055361	1	0,077737	0,863092	0,046406	1,197946
C3301m	168	Temozolomide	11,52	TEM 11.52uM	0,93512	0,104308	87,83704	9,797757	1,055361	1	0,077737	0,843949	0,060109	1,197946
C3301m	168	Temozolomide	57,6	TEM 57.60uM	0,995951	0,053793	93,55102	5,052841	1,055361	1	0,077737	0,929328	0,043013	1,197946
C3301m	168	Temozolomide	288	TEM 288.00uM	0,884929	0,052532	83,12258	4,93437	1,055361	1	0,077737	0,838509	0,049776	1,197946
C5501m	96	Cisplatin	0,106667	CPT 0.11uM	1,551644	0,051989	100,3869	3,363507	1,593305	1	0,672022	0,970612	0,029388	0,226293
C5501m	96	Cisplatin	0,533333	CPT 0.53uM	1,802106	0,083928	116,591	5,429868	1,593305	1	0,672022	1	0	0,226293
C5501m	96	Cisplatin	2,666667	CPT 2.67uM	1,818828	0,149247	117,6729	9,65582	1,593305	1	0,672022	1	0	0,226293
C5501m	96	Cisplatin	13,33333	CPT 13.33uM	1,657114	0,05807	107,2105	3,756962	1,593305	1	0,672022	1	0	0,226293
C5501m	96	Cisplatin	66,66667	CPT 66.67uM	0,420353	0,028999	27,19564	1,876154	1,593305	1	0,672022	0,263825	0,018201	0,226293
C5501m	168	Cisplatin	0,106667	CPT 0.11uM	1,3682	0,025592	104,2684	1,950353	1,296484	1	0,374604	1	0	0,305987
C5501m	168	Cisplatin	0,533333	CPT 0.53uM	1,441465	0,0684	109,8517	5,212661	1,296484	1	0,374604	1	0	0,305987
C5501m	168	Cisplatin	2,666667	CPT 2.67uM	1,500459	0,116912	114,3476	8,909674	1,296484	1	0,374604	1	0	0,305987
C5501m	168	Cisplatin	13,33333	CPT 13.33uM	1,323411	0,153649	100,855	11,70938	1,296484	1	0,374604	0,951128	0,048872	0,305987
C5501m	168	Cisplatin	66,66667	CPT 66.67uM	0,276423	0,001346	21,06578	0,102614	1,296484	1	0,374604	0,21321	0,001039	0,305987
C8101m	96	Cisplatin	0,106667	CPT 0.11uM	1,20727	0,079997	92,15986	6,106788	1,301813	1	0,380522	0,911962	0,046706	0,339438
C8101m	96	Cisplatin	0,533333	CPT 0.53uM	1,405727	0,078597	107,3095	5,999913	1,301813	1	0,380522	0,996879	0,003121	0,339438
C8101m	96	Cisplatin	2,666667	CPT 2.67uM	1,496634	0,072694	114,2492	5,549286	1,301813	1	0,380522	1	0	0,339438
C8101m	96	Cisplatin	13,33333	CPT 13.33uM	1,304285	0,159884	99,5657	12,20513	1,301813	1	0,380522	0,908943	0,091057	0,339438
C8101m	96	Cisplatin	66,66667	CPT 66.67uM	0,718367	0,033738	54,83823	2,575468	1,301813	1	0,380522	0,55182	0,025916	0,339438
C8101m	96	Temozolomide	0,46	TEM 0.46uM	1,066452	0,057461	81,41017	4,386413	1,301813	1	0,380522	0,819206	0,044139	0,373987
C8101m	96	Temozolomide	2,3	TEM 2.30uM	1,277442	0,116353	97,5166	8,88207	1,301813	1	0,380522	0,915518	0,050524	0,373987
C8101m	96	Temozolomide	11,52	TEM 11.52uM	1,194287	0,054252	91,16875	4,141437	1,301813	1	0,380522	0,909365	0,034445	0,373987
C8101m	96	Temozolomide	57,6	TEM 57.60uM	1,485435	0,209147	113,3942	15,96572	1,301813	1	0,380522	0,930262	0,069738	0,373987
C8101m	96	Temozolomide	288	TEM 288.00uM	1,539298	0,047104	117,506	3,595823	1,301813	1	0,380522	1	0	0,373987
C8101m	168	Cisplatin	0,106667	CPT 0.11uM	0,834727	0,072881	70,68911	6,171985	1,146216	1	0,196879	0,728246	0,063584	1,061712
C8101m	168	Cisplatin	0,533333	CPT 0.53uM	1,026609	0,028591	86,93874	2,421272	1,146216	1	0,196879	0,895651	0,024944	1,061712
C8101m	168	Cisplatin	2,666667	CPT 2.67uM	1,035517	0,036684	87,6931	3,106555	1,146216	1	0,196879	0,903423	0,032004	1,061712
C8101m	168	Cisplatin	13,33333	CPT 13.33uM	0,974323	0,063414	82,51081	5,370272	1,146216	1	0,196879	0,850034	0,055325	1,061712
C8101m	168	Cisplatin	66,66667	CPT 66.67uM	0,752882	0,030513	63,75809	2,584026	1,146216	1	0,196879	0,656842	0,026621	1,061712
C8101m	168	Temozolomide	0,46	TEM 0.46uM	0,859581	0,045471	72,79385	3,850706	1,146216	1	0,196879	0,749929	0,03967	1,039962
C8101m	168	Temozolomide	2,3	TEM 2.30uM	0,898734	0,039194	76,10956	3,319179	1,146216	1	0,196879	0,784088	0,034195	1,039962
C8101m	168	Temozolomide	11,52	TEM 11.52uM	0,84206	0,019068	71,31014	1,614751	1,146216	1	0,196879	0,734644	0,016635	1,039962
C8101m	168	Temozolomide	57,6	TEM 57.60uM	1,150267	0,088707	97,41076	7,512149	1,146216	1	0,196879	0,94631	0,039706	1,039962
C8101m	168	Temozolomide	288	TEM 288.00uM	1,154809	0,019732	97,79538	1,671051	1,146216	1	0,196879	0,991505	0,008495	1,039962
C8802p	96	Cisplatin	0,106667	CPT 0.11uM	1,016268	0,084412	98,62567	8,191945	1,048468	1	0,068282	0,923784	0,058986	1,075221
C8802p	96	Cisplatin	0,533333	CPT 0.53uM	0,960805	0,075692	93,24314	7,345638	1,048468	1	0,068282	0,898281	0,061195	1,075221
C8802p	96	Cisplatin	2,666667	CPT 2.67uM	0,942932	0,077663	91,50867	7,536908	1,048468	1	0,068282	0,891337	0,067473	1,075221

C8802p	96	Cisplatin	13,33333	CPT 13.33uM	1,03014	0,081442	99,97196	7,90372	1,048468	1	0,068282	0,935406	0,045056	1,075221
C8802p	96	Cisplatin	66,66667	CPT 66.67uM	0,570941	0,06567	55,40805	6,373095	1,048468	1	0,068282	0,544548	0,062634	1,075221
C8802p	96	Temozolomide	0,46	TEM 0.46uM	0,992529	0,198976	96,32187	19,30998	1,048468	1	0,068282	0,832356	0,100894	0,632048
C8802p	96	Temozolomide	2,3	TEM 2.30uM	0,97466	0,168339	94,5878	16,33676	1,048468	1	0,068282	0,839015	0,088599	0,632048
C8802p	96	Temozolomide	11,52	TEM 11.52uM	1,155262	0,139372	112,1146	13,52566	1,048468	1	0,068282	0,96846	0,03154	0,632048
C8802p	96	Temozolomide	57,6	TEM 57.60uM	1,440888	0,131465	139,8338	12,75831	1,048468	1	0,068282	1	0	0,632048
C8802p	96	Temozolomide	288	TEM 288.00uM	1,08024	0,051068	104,834	4,955971	1,048468	1	0,068282	0,979676	0,01888	0,632048
C8802p	168	Cisplatin	0,106667	CPT 0.11uM	1,163186	0,078401	104,6333	7,052453	1,134365	1	0,181885	0,956899	0,043101	1,14341
C8802p	168	Cisplatin	0,533333	CPT 0.53uM	1,005804	0,102818	90,47619	9,248925	1,134365	1	0,181885	0,868084	0,080288	1,14341
C8802p	168	Cisplatin	2,666667	CPT 2.67uM	0,825564	0,101621	74,26283	9,141263	1,134365	1	0,181885	0,727776	0,089584	1,14341
C8802p	168	Cisplatin	13,33333	CPT 13.33uM	0,938829	0,070274	84,4515	6,321429	1,134365	1	0,181885	0,825023	0,059393	1,14341
C8802p	168	Cisplatin	66,66667	CPT 66.67uM	0,609279	0,088091	54,80713	7,924165	1,134365	1	0,181885	0,53711	0,077657	1,14341
C8802p	168	Temozolomide	0,46	TEM 0.46uM	0,97346	0,115453	87,56665	10,38549	1,134365	1	0,181885	0,844128	0,093298	0,395648
C8802p	168	Temozolomide	2,3	TEM 2.30uM	1,13861	0,154967	102,4226	13,93988	1,134365	1	0,181885	0,894095	0,063403	0,395648
C8802p	168	Temozolomide	11,52	TEM 11.52uM	1,290364	0,163233	116,0735	14,68346	1,134365	1	0,181885	0,953765	0,046235	0,395648
C8802p	168	Temozolomide	57,6	TEM 57.60uM	1,719437	0,15448	154,6704	13,89614	1,134365	1	0,181885	1	0	0,395648
C8802p	168	Temozolomide	288	TEM 288.00uM	1,271307	0,089901	114,3592	8,086975	1,134365	1	0,181885	0,993382	0,006618	0,395648
C9502m	96	Cisplatin	0,106667	CPT 0.11uM	1,091612	0,199415	89,25023	16,30416	1,197884	1	0,260488	0,859479	0,140521	1,120708
C9502m	96	Cisplatin	0,533333	CPT 0.53uM	0,850246	0,114984	69,51615	9,401115	1,197884	1	0,260488	0,70979	0,09599	1,120708
C9502m	96	Cisplatin	2,666667	CPT 2.67uM	1,024818	0,094281	83,78907	7,708387	1,197884	1	0,260488	0,855523	0,078706	1,120708
C9502m	96	Cisplatin	13,33333	CPT 13.33uM	0,928783	0,080206	75,93728	6,557609	1,197884	1	0,260488	0,775353	0,066956	1,120708
C9502m	96	Cisplatin	66,66667	CPT 66.67uM	0,347544	0,020417	28,41517	1,669283	1,197884	1	0,260488	0,290131	0,017044	1,120708
C9502m	96	Temozolomide	0,46	TEM 0.46uM	0,998943	0,114026	81,6736	9,322769	1,197884	1	0,260488	0,833713	0,095005	0,744423
C9502m	96	Temozolomide	2,3	TEM 2.30uM	1,156069	0,064982	94,52017	5,312954	1,197884	1	0,260488	0,946345	0,039818	0,744423
C9502m	96	Temozolomide	11,52	TEM 11.52uM	0,999822	0,108579	81,74542	8,877393	1,197884	1	0,260488	0,834657	0,090642	0,744423
C9502m	96	Temozolomide	57,6	TEM 57.60uM	1,095017	0,109292	89,52854	8,935689	1,197884	1	0,260488	0,879494	0,071078	0,744423
C9502m	96	Temozolomide	288	TEM 288.00uM	0,832875	0,05805	68,09582	4,746173	1,197884	1	0,260488	0,695288	0,048461	0,744423
C9502m	168	Cisplatin	0,106667	CPT 0.11uM	1,134032	0,193595	93,18434	15,90786	1,204152	1	0,268018	0,873642	0,126358	1,149122
C9502m	168	Cisplatin	0,533333	CPT 0.53uM	0,852019	0,119697	70,01113	9,835632	1,204152	1	0,268018	0,707568	0,099404	1,149122
C9502m	168	Cisplatin	2,666667	CPT 2.67uM	1,013747	0,131339	83,30048	10,79224	1,204152	1	0,268018	0,836441	0,106181	1,149122
C9502m	168	Cisplatin	13,33333	CPT 13.33uM	0,873088	0,083374	71,74237	6,850878	1,204152	1	0,268018	0,725064	0,069238	1,149122
C9502m	168	Cisplatin	66,66667	CPT 66.67uM	0,363241	0,021744	29,84782	1,786684	1,204152	1	0,268018	0,301657	0,018057	1,149122
C9502m	168	Temozolomide	0,46	TEM 0.46uM	1,020647	0,092527	83,86745	7,603046	1,204152	1	0,268018	0,847606	0,07684	0,914281
C9502m	168	Temozolomide	2,3	TEM 2.30uM	1,095032	0,125444	89,97968	10,30788	1,204152	1	0,268018	0,875655	0,074309	0,914281
C9502m	168	Temozolomide	11,52	TEM 11.52uM	0,946593	0,104786	77,78239	8,610356	1,204152	1	0,268018	0,786108	0,087021	0,914281
C9502m	168	Temozolomide	57,6	TEM 57.60uM	1,069847	0,13375	87,91026	10,99035	1,204152	1	0,268018	0,848722	0,087369	0,914281
C9502m	168	Temozolomide	288	TEM 288.00uM	0,789738	0,049913	64,89347	4,101356	1,204152	1	0,268018	0,655846	0,04145	0,914281

Supplementary Table 7: Differential expression of drug treatment vs DMSO in PD tumoroids

gene_id	baseMean	log2FoldCh	pvalue	padj	stat	lfcStandard	lfcNotShru	gene_type	gene_name	gene_description	chr_scaffold
ENSG00000198952	2330,49	-0,14023	6,31E-06	0,014526	-4,51563	0,036863	-0,15778	protein_coding	SMG5	SMG5 nonsense mediated mRNA decay factor [Source:HGNC Symbol;Acc:HGNC:24644]	1
ENSG00000131016	7296,143	0,247638	3,66E-05	0,018946	4,127781	0,076789	0,291375	protein_coding	AKAP12	A-kinase anchoring protein 12 [Source:HGNC Symbol;Acc:HGNC:370]	6
ENSG00000187134	1641,267	-0,3721	4,12E-05	0,018946	-4,10081	0,118092	-0,44122	protein_coding	AKR1C1	aldo-keto reductase family 1 member C1 [Source:HGNC Symbol;Acc:HGNC:384]	10
ENSG00000103888	3243,696	-0,36708	3,97E-05	0,018946	-4,10903	0,112841	-0,42526	protein_coding	CEMIP	cell migration inducing hyaluronidase 1 [Source:HGNC Symbol;Acc:HGNC:29213]	15
ENSG00000011422	2195,383	0,21957	2,9E-05	0,018946	4,181197	0,063961	0,248819	protein_coding	PLAUR	plasminogen activator, urokinase receptor [Source:HGNC Symbol;Acc:HGNC:9053]	19
ENSG00000164932	2149,945	0,212254	6,12E-05	0,023456	4,008275	0,066122	0,24422	protein_coding	CTHRC1	collagen triple helix repeat containing 1 [Source:HGNC Symbol;Acc:HGNC:18831]	8
ENSG00000163479	1856,007	0,121282	0,000111	0,024348	3,864725	0,038606	0,138876	protein_coding	SSR2	signal sequence receptor subunit 2 [Source:HGNC Symbol;Acc:HGNC:11324]	1
ENSG00000034510	9688,807	0,191899	0,000122	0,024348	3,84108	0,065543	0,228749	protein_coding	TMSB10	thymosin beta 10 [Source:HGNC Symbol;Acc:HGNC:11879]	2
ENSG00000057608	4815,689	0,10882	0,000127	0,024348	3,832244	0,035865	0,127935	protein_coding	GDI2	GDP dissociation inhibitor 2 [Source:HGNC Symbol;Acc:HGNC:4227]	10
ENSG00000092841	6752,458	0,148644	9,75E-05	0,024348	3,896736	0,048177	0,173152	protein_coding	MYL6	myosin light chain 6 [Source:HGNC Symbol;Acc:HGNC:7587]	12
ENSG00000182718	12623,74	0,152171	8,63E-05	0,024348	3,926067	0,048831	0,176894	protein_coding	ANXA2	annexin A2 [Source:HGNC Symbol;Acc:HGNC:537]	15
ENSG00000002586	3864,326	0,14394	0,000115	0,024348	3,855993	0,046488	0,165562	protein_coding	CD99	CD99 molecule (Xg blood group) [Source:HGNC Symbol;Acc:HGNC:7082]	X
ENSG00000113083	1679,566	0,245426	0,000172	0,026114	3,757393	0,087133	0,29432	protein_coding	LOX	lysyl oxidase [Source:HGNC Symbol;Acc:HGNC:6664]	5
ENSG00000181163	7356,004	0,107076	0,00016	0,026114	3,774902	0,039189	0,13583	protein_coding	NPM1	nucleophosmin 1 [Source:HGNC Symbol;Acc:HGNC:7910]	5
ENSG00000137154	9801,567	0,101184	0,000193	0,026114	3,728092	0,034462	0,119435	protein_coding	RPS6	ribosomal protein S6 [Source:HGNC Symbol;Acc:HGNC:10429]	9
ENSG00000149806	2158,156	0,104866	0,000182	0,026114	3,742912	0,03569	0,123969	protein_coding	FAU	FAU ubiquitin like and ribosomal protein S30 fusion [Source:HGNC Symbol;Acc:HGNC:3597]	11
ENSG00000147065	11283,63	0,151775	0,000153	0,026114	3,785378	0,051993	0,179845	protein_coding	MSN	moesin [Source:HGNC Symbol;Acc:HGNC:7373]	X
ENSG00000049860	2755,121	0,097831	0,000218	0,027836	3,697482	0,03546	0,121082	protein_coding	HEXB	hexosaminidase subunit beta [Source:HGNC Symbol;Acc:HGNC:4879]	5
ENSG00000074800	10066,74	0,09221	0,000267	0,029234	3,645565	0,032028	0,108815	protein_coding	ENO1	enolase 1 [Source:HGNC Symbol;Acc:HGNC:3350]	1
ENSG00000188846	4775,617	0,089913	0,000266	0,029234	3,646074	0,031129	0,105989	protein_coding	RPL14	ribosomal protein L14 [Source:HGNC Symbol;Acc:HGNC:10305]	3
ENSG00000105355	1572,041	0,153241	0,00026	0,029234	3,652302	0,052062	0,173988	protein_coding	PLIN3	perilipin 3 [Source:HGNC Symbol;Acc:HGNC:16893]	19
ENSG00000143549	7030,469	0,117191	0,00029	0,030363	3,623802	0,042405	0,140531	protein_coding	TPM3	tropomyosin 3 [Source:HGNC Symbol;Acc:HGNC:12012]	1
ENSG00000142871	3101,715	0,293348	0,000338	0,031109	3,584292	0,115221	0,362048	protein_coding	CCN1	cellular communication network factor 1 [Source:HGNC Symbol;Acc:HGNC:2654]	1
ENSG00000069275	6083,549	0,117212	0,00033	0,031109	3,590522	0,045885	0,148595	protein_coding	NUCKS1	nuclear casein kinase and cyclin dependent kinase substrate 1 [Source:HGNC Symbol;Acc:HGNC:29923]	1
ENSG00000157613	2390,468	0,195146	0,000317	0,031109	3,601001	0,073222	0,235373	protein_coding	CREB3L1	cAMP responsive element binding protein 3 like 1 [Source:HGNC Symbol;Acc:HGNC:18856]	11
ENSG00000125691	5223,264	0,116082	0,000402	0,034961	3,538771	0,043667	0,140479	protein_coding	RPL23	ribosomal protein L23 [Source:HGNC Symbol;Acc:HGNC:10316]	17
ENSG00000131469	5012,824	0,098207	0,00041	0,034961	3,53341	0,03633	0,118113	protein_coding	RPL27	ribosomal protein L27 [Source:HGNC Symbol;Acc:HGNC:10328]	17
ENSG00000004799	4668,591	-0,27354	0,000435	0,035743	-3,51792	0,11118	-0,34123	protein_coding	PDK4	pyruvate dehydrogenase kinase 4 [Source:HGNC Symbol;Acc:HGNC:8812]	7
ENSG00000198755	2655,091	0,11098	0,000453	0,035818	3,506956	0,043192	0,137195	protein_coding	RPL10A	ribosomal protein L10a [Source:HGNC Symbol;Acc:HGNC:10299]	6
ENSG00000086598	5480,238	0,082383	0,000467	0,035818	3,499014	0,030665	0,099936	protein_coding	TMED2	transmembrane p24 trafficking protein 2 [Source:HGNC Symbol;Acc:HGNC:16996]	12
ENSG00000122406	7721,28	0,095087	0,000527	0,037861	3,466884	0,036451	0,115913	protein_coding	RPL5	ribosomal protein L5 [Source:HGNC Symbol;Acc:HGNC:10360]	1
ENSG00000163191	3626,099	0,127094	0,000517	0,037861	3,471677	0,051049	0,158417	protein_coding	S100A11	S100 calcium binding protein A11 [Source:HGNC Symbol;Acc:HGNC:10488]	1
ENSG00000172757	8075,467	0,113851	0,000578	0,040277	3,441902	0,045854	0,141857	protein_coding	CFL1	cofilin 1 [Source:HGNC Symbol;Acc:HGNC:1874]	11
ENSG00000116288	1679,778	0,135797	0,000644	0,041387	3,412227	0,055928	0,169106	protein_coding	PARK7	Parkinsonism associated deglycase [Source:HGNC Symbol;Acc:HGNC:16369]	1
ENSG00000142676	4009,009	0,101512	0,000658	0,041387	3,406612	0,041767	0,128272	protein_coding	RPL11	ribosomal protein L11 [Source:HGNC Symbol;Acc:HGNC:10301]	1
ENSG00000115091	4715,178	0,127091	0,000683	0,041387	3,396118	0,052703	0,158963	protein_coding	ACTR3	actin related protein 3 [Source:HGNC Symbol;Acc:HGNC:170]	2
ENSG00000108518	5984,85	0,12191	0,00067	0,041387	3,401684	0,050328	0,152482	protein_coding	PFN1	profilin 1 [Source:HGNC Symbol;Acc:HGNC:8881]	17
ENSG00000100345	65539,97	0,125181	0,000657	0,041387	3,406843	0,051417	0,155967	protein_coding	MYH9	myosin heavy chain 9 [Source:HGNC Symbol;Acc:HGNC:7579]	22
ENSG00000131236	7209,277	0,11321	0,000737	0,041978	3,375569	0,046688	0,14107	protein_coding	CAP1	cyclase associated actin cytoskeleton regulatory protein 1 [Source:HGNC Symbol;Acc:HGNC:20040]	1
ENSG00000277443	5202,902	0,093829	0,000736	0,041978	3,375937	0,038103	0,117026	protein_coding	MARCKS	myristoylated alanine rich protein kinase C substrate [Source:HGNC Symbol;Acc:HGNC:6759]	6
ENSG00000136830	6721,196	0,111398	0,000748	0,041978	3,371362	0,047373	0,142122	protein_coding	NIBAN2	niban apoptosis regulator 2 [Source:HGNC Symbol;Acc:HGNC:25282]	9
ENSG00000176014	2056,091	0,191321	0,00077	0,042186	3,363352	0,085759	0,247042	protein_coding	TUBB6	tubulin beta 6 class V [Source:HGNC Symbol;Acc:HGNC:20776]	18
ENSG00000138326	4263,507	0,0965	0,000803	0,042968	3,351773	0,040872	0,123377	protein_coding	RPS24	ribosomal protein S24 [Source:HGNC Symbol;Acc:HGNC:10411]	10
ENSG00000164715	1890,351	-0,09063	0,000918	0,047998	-3,31458	0,037982	-0,1141	protein_coding	LMTK2	lemur tyrosine kinase 2 [Source:HGNC Symbol;Acc:HGNC:17880]	7
ENSG00000173064	2313,477	-0,09455	0,000941	0,048129	-3,30752	0,039838	-0,11893	protein_coding	HECTD4	HECT domain E3 ubiquitin protein ligase 4 [Source:HGNC Symbol;Acc:HGNC:26611]	12
ENSG00000065978	14189,31	0,088667	0,001021	0,048938	3,284712	0,034499	0,104145	protein_coding	YBX1	Y-box binding protein 1 [Source:HGNC Symbol;Acc:HGNC:8014]	1
ENSG00000064666	1705,564	0,179342	0,001005	0,048938	3,289231	0,083556	0,233768	protein_coding	CNN2	calponin 2 [Source:HGNC Symbol;Acc:HGNC:2156]	19
ENSG00000100097	4456,5	0,165148	0,000982	0,048938	3,295725	0,077177	0,216635	protein_coding	LGALS1	galectin 1 [Source:HGNC Symbol;Acc:HGNC:6561]	22
ENSG00000084207	1736,076	0,114824	0,001172	0,053095	3,245584	0,052004	0,147876	protein_coding	GSTP1	glutathione S-transferase pi 1 [Source:HGNC Symbol;Acc:HGNC:4638]	11
ENSG00000132341	3710,83	0,108504	0,001177	0,053095	3,244442	0,050955	0,144155	protein_coding	RAN	RAN, member RAS oncogene family [Source:HGNC Symbol;Acc:HGNC:9846]	12
ENSG00000171246	5840,087	-0,20194	0,001142	0,053095	-3,25309	0,097779	-0,26634	protein_coding	NPTX1	neuronal pentraxin 1 [Source:HGNC Symbol;Acc:HGNC:7952]	17

ENSG00000089009	6305,996	0,09307	0,001358	0,060112	3,203332	0,041428	0,118745	protein_coding	RPL6	ribosomal protein L6 [Source:HGNC Symbol;Acc:HGNC:10362]	12
ENSG00000170515	1793,081	0,09825	0,001424	0,060578	3,18983	0,044898	0,126706	protein_coding	PA2G4	proliferation-associated 2G4 [Source:HGNC Symbol;Acc:HGNC:8550]	12
ENSG00000092199	4361,239	0,074334	0,001448	0,060578	3,184912	0,031619	0,092863	protein_coding	HNRNPC	heterogeneous nuclear ribonucleoprotein C [Source:HGNC Symbol;Acc:HGNC:5035]	14
ENSG00000167004	9114,043	0,087552	0,001405	0,060578	3,193553	0,038843	0,111701	protein_coding	PDIA3	protein disulfide isomerase family A member 3 [Source:HGNC Symbol;Acc:HGNC:4606]	15
ENSG00000115594	2743,184	-0,16791	0,00158	0,064923	-3,15956	0,085279	-0,22398	protein_coding	IL1R1	interleukin 1 receptor type 1 [Source:HGNC Symbol;Acc:HGNC:5993]	2
ENSG00000150093	31326,25	0,107273	0,001623	0,065506	3,151794	0,051166	0,140285	protein_coding	ITGB1	integrin subunit beta 1 [Source:HGNC Symbol;Acc:HGNC:6153]	10
ENSG00000179010	4738,358	0,079681	0,001659	0,065836	3,145242	0,034903	0,100014	protein_coding	MRFAP1	Morf4 family associated protein 1 [Source:HGNC Symbol;Acc:HGNC:24549]	4
ENSG00000135316	3814,116	0,077008	0,001706	0,066515	3,137233	0,033061	0,09519	protein_coding	SYNCRIP	synaptotagmin binding cytoplasmic RNA interacting protein [Source:HGNC Symbol;Acc:HGNC:16918]	6
ENSG00000143183	1971,719	0,106982	0,001878	0,066569	3,108816	0,052741	0,141587	protein_coding	TMCO1	transmembrane and coiled-coil domains 1 [Source:HGNC Symbol;Acc:HGNC:18188]	1
ENSG00000106211	3890,497	0,090005	0,001872	0,066569	3,109756	0,041597	0,115225	protein_coding	HSPB1	heat shock protein family B (small) member 1 [Source:HGNC Symbol;Acc:HGNC:5246]	7
ENSG00000165119	9096,921	0,075822	0,001867	0,066569	3,110554	0,033357	0,094984	protein_coding	HNRNPK	heterogeneous nuclear ribonucleoprotein K [Source:HGNC Symbol;Acc:HGNC:5044]	9
ENSG00000136942	4750,281	0,107849	0,001754	0,066569	3,129024	0,053033	0,143087	protein_coding	RPL35	ribosomal protein L35 [Source:HGNC Symbol;Acc:HGNC:10344]	9
ENSG00000171456	1624,008	-0,09537	0,001801	0,066569	-3,12117	0,045318	-0,12446	protein_coding	ASXL1	ASXL transcriptional regulator 1 [Source:HGNC Symbol;Acc:HGNC:18318]	20
ENSG00000198646	2084,171	-0,0753	0,00188	0,066569	-3,10849	0,035814	-0,1006	protein_coding	NCOA6	nuclear receptor coactivator 6 [Source:HGNC Symbol;Acc:HGNC:15936]	20
ENSG00000110514	1911,839	-0,08915	0,001945	0,067825	-3,09844	0,042459	-0,11652	protein_coding	MADD	MAP kinase activating death domain [Source:HGNC Symbol;Acc:HGNC:6766]	11
ENSG00000135074	2046,508	0,221919	0,002055	0,070588	3,082113	0,126607	0,307443	protein_coding	ADAM19	ADAM metallopeptidase domain 19 [Source:HGNC Symbol;Acc:HGNC:197]	5
ENSG00000150347	2380,409	0,098583	0,002087	0,070609	3,077613	0,048969	0,130907	protein_coding	ARID5B	AT-rich interaction domain 5B [Source:HGNC Symbol;Acc:HGNC:17362]	10
ENSG00000171863	3538,095	0,096485	0,00212	0,070688	3,072925	0,047711	0,127703	protein_coding	RPS7	ribosomal protein S7 [Source:HGNC Symbol;Acc:HGNC:10440]	2
ENSG00000064393	8150,72	-0,08234	0,002162	0,07106	-3,06706	0,038145	-0,10511	protein_coding	HIPK2	homeodomain interacting protein kinase 2 [Source:HGNC Symbol;Acc:HGNC:14402]	7
ENSG00000026025	35674,74	0,16515	0,002353	0,076267	3,041591	0,096589	0,23273	protein_coding	VIM	vimentin [Source:HGNC Symbol;Acc:HGNC:12692]	10
ENSG00000070831	2377,125	0,09635	0,00251	0,078058	3,022092	0,050558	0,131133	protein_coding	CDC42	cell division cycle 42 [Source:HGNC Symbol;Acc:HGNC:1736]	1
ENSG00000035403	7094,688	0,100893	0,002468	0,078058	3,027229	0,052703	0,13637	protein_coding	VCL	vinculin [Source:HGNC Symbol;Acc:HGNC:12665]	10
ENSG00000196547	1647,555	-0,11291	0,002501	0,078058	-3,02324	0,060857	-0,1541	protein_coding	MAN2A2	mannosidase alpha class 2A member 2 [Source:HGNC Symbol;Acc:HGNC:6825]	15
ENSG00000143933	6521,752	0,122222	0,002586	0,078793	3,013043	0,069598	0,171421	protein_coding	CALM2	calmodulin 2 [Source:HGNC Symbol;Acc:HGNC:1445]	2
ENSG00000189403	3156,267	0,118146	0,002602	0,078793	3,011167	0,070315	0,170795	protein_coding	HMGB1	high mobility group box 1 [Source:HGNC Symbol;Acc:HGNC:4983]	13
ENSG00000196923	2284,998	0,101221	0,002679	0,079177	3,002388	0,054394	0,138482	protein_coding	PDLIM7	PDZ and LIM domain 7 [Source:HGNC Symbol;Acc:HGNC:22958]	5
ENSG00000075624	64907,69	0,104897	0,002684	0,079177	3,001791	0,056859	0,14382	protein_coding	ACTB	actin beta [Source:HGNC Symbol;Acc:HGNC:132]	7
ENSG00000179218	26695,27	0,086002	0,00281	0,081839	2,987817	0,050014	0,126471	protein_coding	CALR	calreticulin [Source:HGNC Symbol;Acc:HGNC:1455]	19
ENSG00000145495	2628,821	-0,06762	0,002961	0,084926	-2,97176	0,033064	-0,08968	protein_coding	MARCHF6	membrane associated ring-CH-type finger 6 [Source:HGNC Symbol;Acc:HGNC:30550]	5
ENSG00000140416	12518,16	0,161921	0,00299	0,084926	2,968808	0,104281	0,236433	protein_coding	TPM1	tropomyosin 1 [Source:HGNC Symbol;Acc:HGNC:12010]	15
ENSG00000115216	2608,854	0,088694	0,003058	0,08581	2,961849	0,04704	0,12041	protein_coding	NRBP1	nuclear receptor binding protein 1 [Source:HGNC Symbol;Acc:HGNC:7993]	2
ENSG00000177469	8908,613	0,14015	0,003164	0,087702	2,951385	0,089099	0,203962	protein_coding	CAVIN1	caveolae associated protein 1 [Source:HGNC Symbol;Acc:HGNC:9688]	17
ENSG00000166794	8218,314	0,098908	0,003412	0,093457	2,92798	0,057925	0,140533	protein_coding	PPIB	peptidylprolyl isomerase B [Source:HGNC Symbol;Acc:HGNC:9255]	15
ENSG00000075618	2491,29	0,115665	0,003835	0,095065	2,891416	0,07697	0,17278	protein_coding	FSCN1	fascin actin-bundling protein 1 [Source:HGNC Symbol;Acc:HGNC:11148]	7
ENSG00000136938	2066,842	0,090843	0,003597	0,095065	2,911483	0,060235	0,141339	protein_coding	ANP32B	acidic nuclear phosphoprotein 32 family member B [Source:HGNC Symbol;Acc:HGNC:16677]	9
ENSG00000111669	6193,909	0,074364	0,003801	0,095065	2,894205	0,039145	0,100502	protein_coding	TPI1	triosephosphate isomerase 1 [Source:HGNC Symbol;Acc:HGNC:12009]	12
ENSG00000135486	7152,07	0,081971	0,003696	0,095065	2,903009	0,043828	0,110902	protein_coding	HNRNPA1	heterogeneous nuclear ribonucleoprotein A1 [Source:HGNC Symbol;Acc:HGNC:5031]	12
ENSG00000197728	2145,195	0,089579	0,003825	0,095065	2,892234	0,050248	0,123487	protein_coding	RPS26	ribosomal protein S26 [Source:HGNC Symbol;Acc:HGNC:10414]	12
ENSG00000140264	4509,336	0,09106	0,003724	0,095065	2,90063	0,053176	0,129384	protein_coding	SERF2	small EDRK-rich factor 2 [Source:HGNC Symbol;Acc:HGNC:10757]	15
ENSG00000103855	3565,815	0,078865	0,003619	0,095065	2,909585	0,044965	0,112911	protein_coding	CD276	CD276 molecule [Source:HGNC Symbol;Acc:HGNC:19137]	15
ENSG00000103187	1879,073	0,096072	0,00368	0,095065	2,904402	0,056583	0,136485	protein_coding	COTL1	coactosin like F-actin binding protein 1 [Source:HGNC Symbol;Acc:HGNC:18304]	16
ENSG00000125991	2964,483	0,066836	0,003842	0,095065	2,890829	0,034175	0,089655	protein_coding	ERGIC3	ERGIC and golgi 3 [Source:HGNC Symbol;Acc:HGNC:15927]	20
ENSG00000169641	1815,77	0,097802	0,003938	0,096047	2,883108	0,054958	0,1332	protein_coding	LUZP1	leucine zipper protein 1 [Source:HGNC Symbol;Acc:HGNC:14985]	1
ENSG00000147162	2073,213	-0,09457	0,003965	0,096047	-2,8809	0,056209	-0,13427	protein_coding	OGT	O-linked N-acetylglucosamine (GlcNAc) transferase [Source:HGNC Symbol;Acc:HGNC:8127]	X
ENSG00000142669	4010,299	0,076153	0,004378	0,098766	2,849546	0,04324	0,10712	protein_coding	SH3BGRL3	SH3 domain binding glutamate rich protein like 3 [Source:HGNC Symbol;Acc:HGNC:15568]	1
ENSG00000145715	1701,054	0,098825	0,004245	0,098766	2,859368	0,059357	0,139597	protein_coding	RASA1	RAS p21 protein activator 1 [Source:HGNC Symbol;Acc:HGNC:9871]	5
ENSG00000072682	3171,195	0,105674	0,004296	0,098766	2,855591	0,068199	0,154827	protein_coding	P4HA2	prolyl 4-hydroxylase subunit alpha 2 [Source:HGNC Symbol;Acc:HGNC:8547]	5
ENSG00000197958	5038,86	0,068111	0,004165	0,098766	2,865419	0,0387	0,098283	protein_coding	RPL12	ribosomal protein L12 [Source:HGNC Symbol;Acc:HGNC:10302]	9
ENSG00000196531	4537,521	0,066297	0,004303	0,098766	2,855021	0,03515	0,090617	protein_coding	NACA	nascent polypeptide associated complex subunit alpha [Source:HGNC Symbol;Acc:HGNC:7629]	12
ENSG00000101608	6006,132	0,083342	0,004192	0,098766	2,863344	0,049896	0,120595	protein_coding	MYL12A	myosin light chain 12A [Source:HGNC Symbol;Acc:HGNC:16701]	18
ENSG00000189060	3426,168	-0,08647	0,004338	0,098766	-2,85247	0,057271	-0,1327	protein_coding	H1-0	H1.0 linker histone [Source:HGNC Symbol;Acc:HGNC:4714]	22
ENSG00000113504	1701,509	-0,10952	0,004527	0,099899	-2,83887	0,074583	-0,16405	protein_coding	SLC12A7	solute carrier family 12 member 7 [Source:HGNC Symbol;Acc:HGNC:10915]	5

ENSG00000065833	1814,176	-0,09628	0,004539	0,099899	-2,83805	0,072198	-0,15578	protein_coding	ME1	malic enzyme 1 [Source:HGNC Symbol;Acc:HGNC:6983]	6
ENSG00000110696	2611,304	0,074365	0,004602	0,099899	2,833645	0,040758	0,101587	protein_coding	C11orf58	chromosome 11 open reading frame 58 [Source:HGNC Symbol;Acc:HGNC:16990]	11
ENSG00000174444	11781,18	0,073792	0,004591	0,099899	2,834408	0,04085	0,101751	protein_coding	RPL4	ribosomal protein L4 [Source:HGNC Symbol;Acc:HGNC:10353]	15
ENSG00000137563	2209,614	0,125975	0,004653	0,100071	2,830093	0,095013	0,196048	protein_coding	GGH	gamma-glutamyl hydrolase [Source:HGNC Symbol;Acc:HGNC:4248]	8
ENSG00000168653	1774,291	0,082859	0,004818	0,101258	2,818975	0,050631	0,119899	protein_coding	NDUFS5	NADH:ubiquinone oxidoreductase subunit S5 [Source:HGNC Symbol;Acc:HGNC:7712]	1
ENSG00000136068	6562,111	0,102489	0,004797	0,101258	2,820391	0,069632	0,153742	protein_coding	FLNB	filamin B [Source:HGNC Symbol;Acc:HGNC:3755]	3
ENSG00000231500	5114,161	0,084995	0,00493	0,101258	2,811551	0,059398	0,133546	protein_coding	RPS18	ribosomal protein S18 [Source:HGNC Symbol;Acc:HGNC:10401]	6
ENSG00000156508	43671,63	0,088986	0,00492	0,101258	2,812256	0,056257	0,129902	protein_coding	EEF1A1	eukaryotic translation elongation factor 1 alpha 1 [Source:HGNC Symbol;Acc:HGNC:3189]	6
ENSG00000149273	6154,415	0,067323	0,004982	0,101258	2,808184	0,036835	0,092564	protein_coding	RPS3	ribosomal protein S3 [Source:HGNC Symbol;Acc:HGNC:10420]	11
ENSG00000167553	3263,062	0,100713	0,005017	0,101258	2,805959	0,069605	0,152294	protein_coding	TUBA1C	tubulin alpha 1c [Source:HGNC Symbol;Acc:HGNC:20768]	12
ENSG00000108829	3760,299	0,084239	0,004985	0,101258	2,807985	0,050152	0,118865	protein_coding	LRRC59	leucine rich repeat containing 59 [Source:HGNC Symbol;Acc:HGNC:28817]	17
ENSG00000038427	30174,9	0,084655	0,005198	0,102221	2,794521	0,053109	0,123232	protein_coding	VCAN	versican [Source:HGNC Symbol;Acc:HGNC:2464]	5
ENSG00000120875	1916,582	-0,08241	0,005121	0,102221	-2,79932	0,056556	-0,12817	protein_coding	DUSP4	dual specificity phosphatase 4 [Source:HGNC Symbol;Acc:HGNC:3070]	8
ENSG00000148303	6503,91	0,067568	0,005193	0,102221	2,794797	0,037019	0,092494	protein_coding	RPL7A	ribosomal protein L7a [Source:HGNC Symbol;Acc:HGNC:10364]	9
ENSG00000138668	2548,924	0,084612	0,00535	0,103973	2,785176	0,055241	0,126096	protein_coding	HNRNPD	heterogeneous nuclear ribonucleoprotein D [Source:HGNC Symbol;Acc:HGNC:5036]	4
ENSG00000134333	6809,632	0,06762	0,005377	0,103973	2,783528	0,040722	0,099289	protein_coding	LDHA	lactate dehydrogenase A [Source:HGNC Symbol;Acc:HGNC:6535]	11
ENSG00000118680	5522,898	0,083977	0,005507	0,105599	2,77577	0,054081	0,123715	protein_coding	MYL12B	myosin light chain 12B [Source:HGNC Symbol;Acc:HGNC:29827]	18
ENSG00000110841	3044,023	0,071642	0,005755	0,10944	2,761423	0,043064	0,103045	protein_coding	PPFIBP1	PPFIA binding protein 1 [Source:HGNC Symbol;Acc:HGNC:9249]	12
ENSG00000187109	7160,734	0,057986	0,00581	0,109576	2,758329	0,040244	0,09784	protein_coding	NAP1L1	nucleosome assembly protein 1 like 1 [Source:HGNC Symbol;Acc:HGNC:7637]	12
ENSG00000165916	2364,111	0,068907	0,006025	0,112705	2,746437	0,040831	0,098243	protein_coding	PSMC3	proteasome 26S subunit, ATPase 3 [Source:HGNC Symbol;Acc:HGNC:9549]	11
ENSG00000138385	1701,23	0,072824	0,006359	0,113959	2,728662	0,046737	0,108258	protein_coding	SSB	small RNA binding exonuclease protection factor La [Source:HGNC Symbol;Acc:HGNC:11316]	2
ENSG00000082153	3501,498	0,074524	0,006389	0,113959	2,727128	0,048754	0,111633	protein_coding	BZW1	basic leucine zipper and W2 domains 1 [Source:HGNC Symbol;Acc:HGNC:18380]	2
ENSG00000114942	3939,651	0,070277	0,006262	0,113959	2,733728	0,050695	0,114416	protein_coding	EEF1B2	eukaryotic translation elongation factor 1 beta 2 [Source:HGNC Symbol;Acc:HGNC:3208]	2
ENSG00000131149	2252,898	-0,09867	0,006197	0,113959	-2,73717	0,081103	-0,16108	protein_coding	GSE1	Gse1 coiled-coil protein [Source:HGNC Symbol;Acc:HGNC:28979]	16
ENSG00000108953	5490,713	0,064611	0,00627	0,113959	2,733298	0,044379	0,10392	protein_coding	YWHAE	tyrosine 3-monooxygenase/tryptophan 5-monooxygenase activation protein epsilon [Source:HGNC Symbol;Acc:HGNC:	17
ENSG00000102024	4091,019	0,09524	0,006344	0,113959	2,729425	0,074088	0,151567	protein_coding	PLS3	plastin 3 [Source:HGNC Symbol;Acc:HGNC:9091]	X
ENSG00000149257	8240,279	0,119282	0,006458	0,113982	2,723566	0,131933	0,212801	protein_coding	SERPINH1	serpin family H member 1 [Source:HGNC Symbol;Acc:HGNC:1546]	11
ENSG00000166598	20613,27	0,078461	0,006539	0,113982	2,719466	0,052182	0,117304	protein_coding	HSP90B1	heat shock protein 90 beta family member 1 [Source:HGNC Symbol;Acc:HGNC:12028]	12
ENSG00000125503	1738,154	-0,06763	0,006506	0,113982	-2,72112	0,04088	-0,09735	protein_coding	PPP1R12C	protein phosphatase 1 regulatory subunit 12C [Source:HGNC Symbol;Acc:HGNC:14947]	19
ENSG00000144713	4140,851	0,069767	0,006854	0,115801	2,703843	0,049597	0,111533	protein_coding	RPL32	ribosomal protein L32 [Source:HGNC Symbol;Acc:HGNC:10336]	3
ENSG00000148834	1612,275	0,077589	0,007096	0,115801	2,692303	0,059174	0,125952	protein_coding	GSTO1	glutathione S-transferase omega 1 [Source:HGNC Symbol;Acc:HGNC:13312]	10
ENSG00000109861	2293,715	0,08603	0,007083	0,115801	2,692928	0,065909	0,136653	protein_coding	CTSC	cathepsin C [Source:HGNC Symbol;Acc:HGNC:2528]	11
ENSG00000211455	2149,15	0,080225	0,006805	0,115801	2,706222	0,075799	0,146479	protein_coding	STK38L	serine/threonine kinase 38 like [Source:HGNC Symbol;Acc:HGNC:17848]	12
ENSG00000110906	1802,518	0,079423	0,007023	0,115801	2,695756	0,056112	0,122262	protein_coding	KCTD10	potassium channel tetramerization domain containing 10 [Source:HGNC Symbol;Acc:HGNC:23236]	12
ENSG00000166710	17144,53	0,077419	0,007066	0,115801	2,693723	0,053127	0,117442	protein_coding	B2M	beta-2-microglobulin [Source:HGNC Symbol;Acc:HGNC:914]	15
ENSG00000137818	7906,447	0,071771	0,007071	0,115801	2,693483	0,058551	0,124081	protein_coding	RPLP1	ribosomal protein lateral stalk subunit P1 [Source:HGNC Symbol;Acc:HGNC:10372]	15
ENSG00000079432	2765,465	-0,06583	0,00691	0,115801	-2,70114	0,041317	-0,09736	protein_coding	CIC	capicua transcriptional repressor [Source:HGNC Symbol;Acc:HGNC:14214]	19
ENSG00000101182	2540,416	0,070935	0,006871	0,115801	2,703032	0,045861	0,105563	protein_coding	PSMA7	proteasome 20S subunit alpha 7 [Source:HGNC Symbol;Acc:HGNC:9536]	20
ENSG00000197043	3581,775	0,105454	0,007339	0,116461	2,681061	0,125729	0,194931	protein_coding	ANXA6	annexin A6 [Source:HGNC Symbol;Acc:HGNC:544]	5
ENSG00000107581	9455,075	0,05045	0,007312	0,116461	2,682282	0,02743	0,069638	protein_coding	EIF3A	eukaryotic translation initiation factor 3 subunit A [Source:HGNC Symbol;Acc:HGNC:3271]	10
ENSG00000170558	2592,205	0,108625	0,007227	0,116461	2,686192	0,192987	0,220431	protein_coding	CDH2	cadherin 2 [Source:HGNC Symbol;Acc:HGNC:1759]	18
ENSG00000100106	2763,532	0,079407	0,007276	0,116461	2,683927	0,072559	0,141763	protein_coding	TRIOBP	TRIO and F-actin binding protein [Source:HGNC Symbol;Acc:HGNC:17009]	22
ENSG00000168268	2659,337	0,087913	0,00759	0,119628	2,669764	0,101115	0,168027	protein_coding	NT5DC2	5'-nucleotidase domain containing 2 [Source:HGNC Symbol;Acc:HGNC:25717]	3
ENSG00000180900	1831,046	-0,07263	0,007739	0,121145	-2,66323	0,047911	-0,10742	protein_coding	SCRIB	scribble planar cell polarity protein [Source:HGNC Symbol;Acc:HGNC:30377]	8
ENSG00000074842	1636,597	0,088115	0,00809	0,124093	2,64831	0,065128	0,13409	protein_coding	MYDGF	myeloid derived growth factor [Source:HGNC Symbol;Acc:HGNC:16948]	19
ENSG00000064607	1637,369	-0,0675	0,008057	0,124093	-2,64969	0,04443	-0,10084	protein_coding	SUGP2	SURP and G-patch domain containing 2 [Source:HGNC Symbol;Acc:HGNC:18641]	19
ENSG00000196924	39210,91	0,089462	0,007991	0,124093	2,652458	0,082174	0,153152	protein_coding	FLNA	filamin A [Source:HGNC Symbol;Acc:HGNC:3754]	X
ENSG00000153187	8948,153	0,056431	0,008332	0,125304	2,638327	0,032507	0,078721	protein_coding	HNRNPU	heterogeneous nuclear ribonucleoprotein U [Source:HGNC Symbol;Acc:HGNC:5048]	1
ENSG00000065548	1598,362	0,070639	0,008332	0,125304	2,63832	0,052597	0,113429	protein_coding	ZC3H15	zinc finger CCCH-type containing 15 [Source:HGNC Symbol;Acc:HGNC:29528]	2
ENSG00000179820	4388,495	0,080548	0,008322	0,125304	2,638713	0,074575	0,141811	protein_coding	MYADM	myeloid associated differentiation marker [Source:HGNC Symbol;Acc:HGNC:7544]	19
ENSG00000170889	2680,514	0,061453	0,008548	0,12689	2,62964	0,039447	0,091464	protein_coding	RPS9	ribosomal protein S9 [Source:HGNC Symbol;Acc:HGNC:10442]	19
ENSG00000099901	1562,208	0,084458	0,008518	0,12689	2,630822	0,065992	0,133079	protein_coding	RANBP1	RAN binding protein 1 [Source:HGNC Symbol;Acc:HGNC:9847]	22

ENSG00000067560	6987,937	0,068956	0,009149	0,134088	2,606436	0,052802	0,112138	protein_coding	RHOA	ras homolog family member A [Source:HGNC Symbol;Acc:HGNC:667]	3
ENSG00000041357	1902,313	0,06973	0,009132	0,134088	2,607073	0,053566	0,113328	protein_coding	PSMA4	proteasome 20S subunit alpha 4 [Source:HGNC Symbol;Acc:HGNC:9533]	15
ENSG00000197451	3166,502	0,067865	0,009272	0,135036	2,601846	0,054329	0,113913	protein_coding	HNRNPAB	heterogeneous nuclear ribonucleoprotein A/B [Source:HGNC Symbol;Acc:HGNC:5034]	5
ENSG00000135046	4583,096	0,078351	0,009474	0,136251	2,594452	0,115835	0,165807	protein_coding	ANXA1	annexin A1 [Source:HGNC Symbol;Acc:HGNC:533]	9
ENSG00000177731	4016,989	-0,05971	0,00947	0,136251	-2,59461	0,037571	-0,08696	protein_coding	FLII	FLII actin remodeling protein [Source:HGNC Symbol;Acc:HGNC:3750]	17
ENSG00000174748	5875,474	0,069465	0,009624	0,137547	2,58905	0,052299	0,110682	protein_coding	RPL15	ribosomal protein L15 [Source:HGNC Symbol;Acc:HGNC:10306]	3
ENSG00000115484	2438,96	0,065037	0,009855	0,138403	2,580887	0,046904	0,102156	protein_coding	CCT4	chaperonin containing TCP1 subunit 4 [Source:HGNC Symbol;Acc:HGNC:1617]	2
ENSG00000078098	2701,946	0,035285	0,00979	0,138403	2,583147	0,077516	0,224205	protein_coding	FAP	fibroblast activation protein alpha [Source:HGNC Symbol;Acc:HGNC:3590]	2
ENSG00000104112	4148,212	0,024459	0,009915	0,138403	2,578787	0,04411	0,239831	protein_coding	SCG3	secretogranin III [Source:HGNC Symbol;Acc:HGNC:13707]	15
ENSG00000140988	5866,417	0,06628	0,009925	0,138403	2,578445	0,045342	0,099591	protein_coding	RPS2	ribosomal protein S2 [Source:HGNC Symbol;Acc:HGNC:10404]	16
ENSG00000198467	8741,763	0,079777	0,010016	0,138773	2,575285	0,135931	0,172728	protein_coding	TPM2	tropomyosin 2 [Source:HGNC Symbol;Acc:HGNC:12011]	9
ENSG00000182831	1881,221	0,060272	0,010072	0,138773	2,573356	0,04093	0,092197	protein_coding	C16orf72	chromosome 16 open reading frame 72 [Source:HGNC Symbol;Acc:HGNC:30103]	16
ENSG00000011007	1950,937	0,060849	0,01033	0,139001	2,564584	0,040631	0,091317	protein_coding	ELOA	elongin A [Source:HGNC Symbol;Acc:HGNC:11620]	1
ENSG00000198830	2268,555	0,072951	0,010281	0,139001	2,566244	0,063677	0,1244	protein_coding	HMGN2	high mobility group nucleosomal binding domain 2 [Source:HGNC Symbol;Acc:HGNC:4986]	1
ENSG00000058668	3115,223	0,029209	0,010219	0,139001	2,568331	0,053496	0,214647	protein_coding	ATP2B4	ATPase plasma membrane Ca2+ transporting 4 [Source:HGNC Symbol;Acc:HGNC:817]	1
ENSG00000145592	4982,232	0,06846	0,010249	0,139001	2,567325	0,054751	0,113004	protein_coding	RPL37	ribosomal protein L37 [Source:HGNC Symbol;Acc:HGNC:10347]	5
ENSG00000132694	2349,105	-0,06768	0,010803	0,14379	-2,54902	0,054241	-0,11143	protein_coding	ARHGEF11	Rho guanine nucleotide exchange factor 11 [Source:HGNC Symbol;Acc:HGNC:14580]	1
ENSG00000105401	3404,774	0,060263	0,010811	0,14379	2,548756	0,040462	0,090461	protein_coding	CDC37	cell division cycle 37, HSP90 cochaperone [Source:HGNC Symbol;Acc:HGNC:1735]	19
ENSG00000079785	2122,201	0,058698	0,011047	0,146084	2,541216	0,038797	0,087407	protein_coding	DDX1	DEAD-box helicase 1 [Source:HGNC Symbol;Acc:HGNC:2734]	2
ENSG00000142937	6216,675	0,062717	0,011191	0,146423	2,536675	0,045518	0,098236	protein_coding	RPS8	ribosomal protein S8 [Source:HGNC Symbol;Acc:HGNC:10441]	1
ENSG00000140443	2385,976	0,072733	0,0112	0,146423	2,536407	0,067368	0,127097	protein_coding	IGF1R	insulin like growth factor 1 receptor [Source:HGNC Symbol;Acc:HGNC:5465]	15
ENSG00000116489	2289,024	0,062719	0,011807	0,14971	2,51785	0,071236	0,128918	protein_coding	CAPZA1	capping actin protein of muscle Z-line subunit alpha 1 [Source:HGNC Symbol;Acc:HGNC:1488]	1
ENSG00000177600	7194,222	0,059859	0,011841	0,14971	2,516835	0,042548	0,092825	protein_coding	RPLP2	ribosomal protein lateral stalk subunit P2 [Source:HGNC Symbol;Acc:HGNC:10377]	11
ENSG00000110422	2905,889	-0,06111	0,011797	0,14971	-2,51815	0,036063	-0,08162	protein_coding	HIPK3	homeodomain interacting protein kinase 3 [Source:HGNC Symbol;Acc:HGNC:4915]	11
ENSG00000050405	2052,792	0,073318	0,011543	0,14971	2,525829	0,07812	0,136684	protein_coding	LIMA1	LIM domain and actin binding 1 [Source:HGNC Symbol;Acc:HGNC:24636]	12
ENSG00000141756	6245,341	0,057465	0,011594	0,14971	2,52426	0,045675	0,098344	protein_coding	FKBP10	FKBP prolyl isomerase 10 [Source:HGNC Symbol;Acc:HGNC:18169]	17
ENSG00000125977	2653,1	0,055496	0,011742	0,14971	2,519799	0,035568	0,081101	protein_coding	EIF2S2	eukaryotic translation initiation factor 2 subunit beta [Source:HGNC Symbol;Acc:HGNC:3266]	20
ENSG00000086015	1991,931	-0,05765	0,012091	0,150816	-2,50947	0,039779	-0,08809	protein_coding	MAST2	microtubule associated serine/threonine kinase 2 [Source:HGNC Symbol;Acc:HGNC:19035]	1
ENSG00000134250	4577,244	0,061321	0,012388	0,150816	2,500901	0,046547	0,098457	protein_coding	NOTCH2	notch receptor 2 [Source:HGNC Symbol;Acc:HGNC:7882]	1
ENSG00000008988	5040,011	0,060035	0,01236	0,150816	2,501683	0,043988	0,094624	protein_coding	RPS20	ribosomal protein S20 [Source:HGNC Symbol;Acc:HGNC:10405]	8
ENSG00000168439	2661,933	0,063739	0,012181	0,150816	2,506846	0,049231	0,102605	protein_coding	STIP1	stress induced phosphoprotein 1 [Source:HGNC Symbol;Acc:HGNC:11387]	11
ENSG00000134954	1614,696	0,064551	0,012057	0,150816	2,510477	0,050359	0,104404	protein_coding	ETS1	ETS proto-oncogene 1, transcription factor [Source:HGNC Symbol;Acc:HGNC:3488]	11
ENSG00000006327	1749,234	0,069661	0,012367	0,150816	2,501484	0,073006	0,130205	protein_coding	TNFRSF12A	TNF receptor superfamily member 12A [Source:HGNC Symbol;Acc:HGNC:18152]	16
ENSG00000126247	3632,482	0,054095	0,012319	0,150816	2,502882	0,037988	0,085212	protein_coding	CAPNS1	calpain small subunit 1 [Source:HGNC Symbol;Acc:HGNC:1481]	19
ENSG00000163682	1729,825	0,064236	0,012735	0,153661	2,49109	0,09774	0,146636	protein_coding	RPL9	ribosomal protein L9 [Source:HGNC Symbol;Acc:HGNC:10369]	4
ENSG00000148516	1933,837	0,049353	0,012755	0,153661	2,490537	0,120731	0,168526	protein_coding	ZEB1	zinc finger E-box binding homeobox 1 [Source:HGNC Symbol;Acc:HGNC:11642]	10
ENSG00000143322	1936,855	0,060328	0,013008	0,155756	2,483558	0,05018	0,103206	protein_coding	ABL2	ABL proto-oncogene 2, non-receptor tyrosine kinase [Source:HGNC Symbol;Acc:HGNC:77]	1
ENSG00000114353	4551,426	0,050027	0,013274	0,155756	2,476324	0,032517	0,074699	protein_coding	GNAI2	G protein subunit alpha i2 [Source:HGNC Symbol;Acc:HGNC:4385]	3
ENSG00000149218	1691,404	0,073271	0,013335	0,155756	2,474693	0,115129	0,155371	protein_coding	ENDOD1	endonuclease domain containing 1 [Source:HGNC Symbol;Acc:HGNC:29129]	11
ENSG00000167526	5924,003	0,056111	0,01318	0,155756	2,478866	0,03443	0,077628	protein_coding	RPL13	ribosomal protein L13 [Source:HGNC Symbol;Acc:HGNC:10303]	16
ENSG00000167460	19226,91	0,025109	0,013213	0,155756	2,477964	0,044634	0,214118	protein_coding	TPM4	tropomyosin 4 [Source:HGNC Symbol;Acc:HGNC:12013]	19
ENSG00000142168	2873,852	0,074096	0,013139	0,155756	2,479986	0,059676	0,115821	protein_coding	SOD1	superoxide dismutase 1 [Source:HGNC Symbol;Acc:HGNC:11179]	21
ENSG00000099814	1825,831	-0,06613	0,013413	0,15588	-2,4726	0,053703	-0,10734	protein_coding	CEP170B	centrosomal protein 170B [Source:HGNC Symbol;Acc:HGNC:20362]	14
ENSG00000255302	3667,877	0,060526	0,013619	0,15669	2,467152	0,048112	0,099509	protein_coding	EID1	EP300 interacting inhibitor of differentiation 1 [Source:HGNC Symbol;Acc:HGNC:1191]	15
ENSG00000172809	2941,071	0,064288	0,013574	0,15669	2,46835	0,059049	0,113763	protein_coding	RPL38	ribosomal protein L38 [Source:HGNC Symbol;Acc:HGNC:10349]	17
ENSG00000173457	2011,609	0,059785	0,013814	0,156922	2,462076	0,101323	0,147092	protein_coding	PPP1R14B	protein phosphatase 1 regulatory inhibitor subunit 14B [Source:HGNC Symbol;Acc:HGNC:9057]	11
ENSG00000140391	2461,865	0,058032	0,013844	0,156922	2,461285	0,051181	0,103941	protein_coding	TSPAN3	tetraspanin 3 [Source:HGNC Symbol;Acc:HGNC:17752]	15
ENSG00000132507	4118,229	0,060523	0,013709	0,156922	2,464811	0,053926	0,107398	protein_coding	EIF5A	eukaryotic translation initiation factor 5A [Source:HGNC Symbol;Acc:HGNC:3300]	17
ENSG00000155368	1608,703	0,068281	0,013921	0,157023	2,45929	0,083985	0,136518	protein_coding	DBI	diazepam binding inhibitor, acyl-CoA binding protein [Source:HGNC Symbol;Acc:HGNC:2690]	2
ENSG00000114391	3005,079	0,062876	0,014012	0,157279	2,456949	0,059475	0,113728	protein_coding	RPL24	ribosomal protein L24 [Source:HGNC Symbol;Acc:HGNC:10325]	3
ENSG00000101150	2178,047	0,055921	0,014842	0,165782	2,436216	0,042503	0,090227	protein_coding	TPD52L2	TPD52 like 2 [Source:HGNC Symbol;Acc:HGNC:12007]	20
ENSG00000170144	3377,417	0,052323	0,015294	0,167634	2,425348	0,037609	0,082156	protein_coding	HNRNPA3	heterogeneous nuclear ribonucleoprotein A3 [Source:HGNC Symbol;Acc:HGNC:24941]	2

ENSG00000096384	19229,85	0,052809	0,015299	0,167634	2,425221	0,037408	0,081706	protein_coding	HSP90AB1	heat shock protein 90 alpha family class B member 1 [Source:HGNC Symbol;Acc:HGNC:5258]	6
ENSG00000130164	3497,89	-0,05789	0,01519	0,167634	-2,42782	0,049874	-0,10063	protein_coding	LDLR	low density lipoprotein receptor [Source:HGNC Symbol;Acc:HGNC:6547]	19
ENSG00000132669	1676,679	0,061336	0,015085	0,167634	2,430343	0,061585	0,114911	protein_coding	RIN2	Ras and Rab interactor 2 [Source:HGNC Symbol;Acc:HGNC:18750]	20
ENSG00000151690	2064,569	-0,05859	0,015655	0,170722	-2,41686	0,057682	-0,11014	protein_coding	MFSD6	major facilitator superfamily domain containing 6 [Source:HGNC Symbol;Acc:HGNC:24711]	2
ENSG00000166913	5212,698	0,051422	0,015781	0,171287	2,413934	0,035941	0,07896	protein_coding	YWHAB	tyrosine 3-monooxygenase/tryptophan 5-monooxygenase activation protein beta [Source:HGNC Symbol;Acc:HGNC:12{	20
ENSG00000071054	9969,342	0,045037	0,015861	0,171345	2,412094	0,081206	0,147131	protein_coding	MAP4K4	mitogen-activated protein kinase kinase kinase kinase 4 [Source:HGNC Symbol;Acc:HGNC:6866]	2
ENSG00000100316	10704,41	0,056115	0,015965	0,171664	2,409709	0,045627	0,094019	protein_coding	RPL3	ribosomal protein L3 [Source:HGNC Symbol;Acc:HGNC:10332]	22
ENSG00000139211	1703,429	0,039796	0,016197	0,173348	2,404441	0,073175	0,155564	protein_coding	AMIGO2	adhesion molecule with Ig like domain 2 [Source:HGNC Symbol;Acc:HGNC:24073]	12
ENSG00000063660	3148,014	0,042757	0,016475	0,175507	2,398214	0,082265	0,154493	protein_coding	GPC1	glypican 1 [Source:HGNC Symbol;Acc:HGNC:4449]	2
ENSG00000109472	3857,986	-0,03243	0,016606	0,176088	-2,39531	0,056842	-0,16792	protein_coding	CPE	carboxypeptidase E [Source:HGNC Symbol;Acc:HGNC:2303]	4
ENSG00000241973	2245,577	-0,06657	0,016829	0,177629	-2,39043	0,045967	-0,09327	protein_coding	PI4KA	phosphatidylinositol 4-kinase alpha [Source:HGNC Symbol;Acc:HGNC:8983]	22
ENSG00000106366	25962,54	0,034611	0,016936	0,177946	2,388089	0,062689	0,166658	protein_coding	SERPINE1	serpin family E member 1 [Source:HGNC Symbol;Acc:HGNC:8583]	7
ENSG00000078808	3189,21	0,05245	0,017332	0,17964	2,379596	0,035906	0,077578	protein_coding	SDF4	stromal cell derived factor 4 [Source:HGNC Symbol;Acc:HGNC:24188]	1
ENSG00000147604	4302,262	0,053644	0,017272	0,17964	2,380865	0,047181	0,09571	protein_coding	RPL7	ribosomal protein L7 [Source:HGNC Symbol;Acc:HGNC:10363]	8
ENSG00000044574	14372,43	0,051411	0,017178	0,17964	2,382869	0,038134	0,08183	protein_coding	HSPA5	heat shock protein family A (Hsp70) member 5 [Source:HGNC Symbol;Acc:HGNC:5238]	9
ENSG00000145425	4017,195	0,053464	0,017621	0,181816	2,373497	0,055212	0,106506	protein_coding	RPS3A	ribosomal protein S3A [Source:HGNC Symbol;Acc:HGNC:10421]	4
ENSG00000152377	6507,346	0,018748	0,01831	0,186427	2,359279	0,035539	0,23181	protein_coding	SPOCK1	SPARC (osteonectin), cwcv and kazal like domains proteoglycan 1 [Source:HGNC Symbol;Acc:HGNC:11251]	5
ENSG00000119335	6785,296	0,051582	0,018176	0,186427	2,362007	0,037578	0,080088	protein_coding	SET	SET nuclear proto-oncogene [Source:HGNC Symbol;Acc:HGNC:10760]	9
ENSG00000076108	3526,893	-0,05253	0,018239	0,186427	-2,36073	0,043976	-0,09035	protein_coding	BAZ2A	bromodomain adjacent to zinc finger domain 2A [Source:HGNC Symbol;Acc:HGNC:962]	12
ENSG00000166825	2992,015	0,016685	0,018412	0,186638	2,357219	0,033503	0,254918	protein_coding	ANPEP	alanyl aminopeptidase, membrane [Source:HGNC Symbol;Acc:HGNC:500]	15
ENSG00000117020	2226,151	0,054857	0,018629	0,18801	2,352865	0,065126	0,116401	protein_coding	AKT3	AKT serine/threonine kinase 3 [Source:HGNC Symbol;Acc:HGNC:393]	1
ENSG00000163466	4609,879	0,056985	0,019105	0,190304	2,343479	0,047208	0,093641	protein_coding	ARPC2	actin related protein 2/3 complex subunit 2 [Source:HGNC Symbol;Acc:HGNC:705]	2
ENSG00000113448	1581,877	0,057051	0,019071	0,190304	2,344143	0,094863	0,137925	protein_coding	PDE4D	phosphodiesterase 4D [Source:HGNC Symbol;Acc:HGNC:8783]	5
ENSG00000112306	5118,155	0,053873	0,018982	0,190304	2,345882	0,049646	0,097778	protein_coding	RPS12	ribosomal protein S12 [Source:HGNC Symbol;Acc:HGNC:10385]	6
ENSG00000149091	1564,982	-0,05376	0,01931	0,191521	-2,33949	0,050728	-0,09898	protein_coding	DGKZ	diacylglycerol kinase zeta [Source:HGNC Symbol;Acc:HGNC:2857]	11
ENSG00000132688	4526,961	0,038595	0,019523	0,192074	2,335382	0,068335	0,148644	protein_coding	NES	nestin [Source:HGNC Symbol;Acc:HGNC:7756]	1
ENSG00000160213	1627,846	0,051354	0,019533	0,192074	2,3352	0,042614	0,087633	protein_coding	CSTB	cystatin B [Source:HGNC Symbol;Acc:HGNC:2482]	21
ENSG00000137076	15428,9	0,05364	0,019751	0,192573	2,331042	0,051577	0,099851	protein_coding	TLN1	talin 1 [Source:HGNC Symbol;Acc:HGNC:11845]	9
ENSG00000214655	1679,579	-0,05387	0,019694	0,192573	-2,33212	0,053754	-0,10263	protein_coding	ZSWIM8	zinc finger SWIM-type containing 8 [Source:HGNC Symbol;Acc:HGNC:23528]	10
ENSG00000073756	4679,334	-0,00883	0,020318	0,193192	-2,32042	0,02807	-0,42504	protein_coding	PTGS2	prostaglandin-endoperoxide synthase 2 [Source:HGNC Symbol;Acc:HGNC:9605]	1
ENSG00000143774	1772,669	0,046926	0,020279	0,193192	2,32114	0,041441	0,086778	protein_coding	GUK1	guanylate kinase 1 [Source:HGNC Symbol;Acc:HGNC:4693]	1
ENSG00000184840	3505,581	0,054088	0,020055	0,193192	2,325324	0,052761	0,101018	protein_coding	TMED9	transmembrane p24 trafficking protein 9 [Source:HGNC Symbol;Acc:HGNC:24878]	5
ENSG00000131459	1588,641	0,019476	0,020004	0,193192	2,326272	0,036241	0,218315	protein_coding	GFPT2	glutamine-fructose-6-phosphate transaminase 2 [Source:HGNC Symbol;Acc:HGNC:4242]	5
ENSG00000073712	2106,149	0,048209	0,020287	0,193192	2,320988	0,087267	0,140335	protein_coding	FERMT2	fermitin family member 2 [Source:HGNC Symbol;Acc:HGNC:15767]	14
ENSG00000230989	1575,852	0,051684	0,020131	0,193192	2,323891	0,056551	0,106645	protein_coding	HSBP1	heat shock factor binding protein 1 [Source:HGNC Symbol;Acc:HGNC:5203]	16
ENSG00000143669	1856,481	-0,05072	0,020691	0,195365	-2,31358	0,078909	-0,12984	protein_coding	LYST	lysosomal trafficking regulator [Source:HGNC Symbol;Acc:HGNC:1968]	1
ENSG00000108107	5871,337	0,043029	0,020717	0,195365	2,313109	0,029682	0,065934	protein_coding	RPL28	ribosomal protein L28 [Source:HGNC Symbol;Acc:HGNC:10330]	19
ENSG00000166226	2097,466	0,052362	0,020946	0,19672	2,308959	0,051728	0,099503	protein_coding	CCT2	chaperonin containing TCP1 subunit 2 [Source:HGNC Symbol;Acc:HGNC:1615]	12
ENSG00000187514	15371,54	0,047307	0,021182	0,197325	2,304729	0,045779	0,093009	protein_coding	PTMA	prothymosin alpha [Source:HGNC Symbol;Acc:HGNC:9623]	2
ENSG00000085733	6315,119	0,053163	0,021151	0,197325	2,30527	0,043524	0,087418	protein_coding	CTTN	cortactin [Source:HGNC Symbol;Acc:HGNC:3338]	11
ENSG00000168028	4198,47	0,050626	0,021346	0,197914	2,3018	0,043232	0,087533	protein_coding	RPSA	ribosomal protein SA [Source:HGNC Symbol;Acc:HGNC:6502]	3
ENSG00000029363	3242,445	0,047466	0,021417	0,197914	2,300551	0,037349	0,078748	protein_coding	BCLAF1	BCL2 associated transcription factor 1 [Source:HGNC Symbol;Acc:HGNC:16863]	6
ENSG00000166068	2130,104	0,045258	0,021664	0,199391	2,296216	0,065411	0,122455	protein_coding	SPRED1	sprouty related EVH1 domain containing 1 [Source:HGNC Symbol;Acc:HGNC:20249]	15
ENSG00000186468	4951,011	0,048712	0,02194	0,200645	2,291401	0,041706	0,085367	protein_coding	RPS23	ribosomal protein S23 [Source:HGNC Symbol;Acc:HGNC:10410]	5
ENSG00000105738	3303,733	-0,05254	0,021974	0,200645	-2,29081	0,063146	-0,11231	protein_coding	SIPA1L3	signal induced proliferation associated 1 like 3 [Source:HGNC Symbol;Acc:HGNC:23801]	19
ENSG00000077380	2398,1	0,049502	0,022071	0,20073	2,289149	0,049232	0,096444	protein_coding	DYNC1I2	dynein cytoplasmic 1 intermediate chain 2 [Source:HGNC Symbol;Acc:HGNC:2964]	2
ENSG00000131389	4308,662	-0,05577	0,022171	0,200846	-2,28743	0,108346	-0,1415	protein_coding	SLC6A6	solute carrier family 6 member 6 [Source:HGNC Symbol;Acc:HGNC:11052]	3
ENSG00000167085	1709,383	0,049861	0,022304	0,201259	2,285155	0,04666	0,092375	protein_coding	PHB	prohibitin [Source:HGNC Symbol;Acc:HGNC:8912]	17
ENSG00000176619	2166,683	0,046876	0,022422	0,20154	2,283134	0,062522	0,115869	protein_coding	LMNB2	lamin B2 [Source:HGNC Symbol;Acc:HGNC:6638]	19
ENSG00000077549	2802,002	0,055845	0,022875	0,201722	2,275509	0,043524	0,085839	protein_coding	CAPZB	capping actin protein of muscle Z-line subunit beta [Source:HGNC Symbol;Acc:HGNC:1491]	1
ENSG00000130294	4878,331	-0,01863	0,022647	0,201722	-2,27934	0,035233	-0,21748	protein_coding	KIF1A	kinesin family member 1A [Source:HGNC Symbol;Acc:HGNC:888]	2
ENSG00000197702	3006,689	0,049167	0,022646	0,201722	2,279359	0,046731	0,092574	protein_coding	PARVA	parvin alpha [Source:HGNC Symbol;Acc:HGNC:14652]	11

ENSG00000131873	2073,899	0,049579	0,022881	0,201722	2,275415	0,061383	0,111476	protein_coding	CHSY1	chondroitin sulfate synthase 1 [Source:HGNC Symbol;Acc:HGNC:17198]	15
ENSG00000132475	4271,275	0,051762	0,022769	0,201722	2,277294	0,043022	0,085756	protein_coding	H3-3B	H3.3 histone B [Source:HGNC Symbol;Acc:HGNC:4765]	17
ENSG00000109475	1891,576	0,048748	0,023171	0,203502	2,270596	0,050863	0,0984	protein_coding	RPL34	ribosomal protein L34 [Source:HGNC Symbol;Acc:HGNC:10340]	4
ENSG00000129657	2050,986	-0,05908	0,023436	0,205039	-2,26626	0,046557	-0,08939	protein_coding	SEC14L1	SEC14 like lipid binding 1 [Source:HGNC Symbol;Acc:HGNC:10698]	17
ENSG00000196419	4582,773	0,045718	0,023594	0,205645	2,263673	0,044038	0,089568	protein_coding	XRCC6	X-ray repair cross complementing 6 [Source:HGNC Symbol;Acc:HGNC:4055]	22
ENSG00000170275	3271,079	0,048038	0,023693	0,205726	2,262073	0,059417	0,109771	protein_coding	CRTAP	cartilage associated protein [Source:HGNC Symbol;Acc:HGNC:2379]	3
ENSG00000167615	2259,132	-0,02524	0,024035	0,207914	-2,25656	0,043342	-0,17289	protein_coding	LENG8	leukocyte receptor cluster member 8 [Source:HGNC Symbol;Acc:HGNC:15500]	19
ENSG00000142864	4799,153	0,04624	0,02482	0,209414	2,244193	0,036585	0,075853	protein_coding	SERBP1	SERPINE1 mRNA binding protein 1 [Source:HGNC Symbol;Acc:HGNC:17860]	1
ENSG00000138071	4632,01	0,040257	0,024779	0,209414	2,244835	0,049883	0,104609	protein_coding	ACTR2	actin related protein 2 [Source:HGNC Symbol;Acc:HGNC:169]	2
ENSG00000164924	12500,42	0,047733	0,024883	0,209414	2,243207	0,053927	0,102074	protein_coding	YWHAZ	tyrosine 3-monooxygenase/tryptophan 5-monooxygenase activation protein zeta [Source:HGNC Symbol;Acc:HGNC:128	8
ENSG00000133816	13253,37	0,046317	0,024944	0,209414	2,242268	0,056494	0,106799	protein_coding	MICAL2	microtubule associated monooxygenase, calponin and LIM domain containing 2 [Source:HGNC Symbol;Acc:HGNC:2469	11
ENSG00000172531	1600,635	0,046525	0,024561	0,209414	2,248236	0,053243	0,102318	protein_coding	PPP1CA	protein phosphatase 1 catalytic subunit alpha [Source:HGNC Symbol;Acc:HGNC:9281]	11
ENSG00000215021	1566,817	0,048007	0,025028	0,209414	2,240974	0,04555	0,089798	protein_coding	PHB2	prohibitin 2 [Source:HGNC Symbol;Acc:HGNC:30306]	12
ENSG00000123416	10531,38	0,043176	0,024765	0,209414	2,245047	0,061632	0,118145	protein_coding	TUBA1B	tubulin alpha 1b [Source:HGNC Symbol;Acc:HGNC:18809]	12
ENSG00000122026	2840,336	0,050123	0,02471	0,209414	2,245913	0,062539	0,110915	protein_coding	RPL21	ribosomal protein L21 [Source:HGNC Symbol;Acc:HGNC:10313]	13
ENSG00000078699	1604,705	-0,03052	0,024546	0,209414	-2,24847	0,050088	-0,14655	protein_coding	CBFA2T2	CBFA2/RUNX1 partner transcriptional co-repressor 2 [Source:HGNC Symbol;Acc:HGNC:1536]	20
ENSG00000130522	3153,186	-0,05007	0,025617	0,213568	-2,23197	0,07688	-0,12419	protein_coding	JUND	JunD proto-oncogene, AP-1 transcription factor subunit [Source:HGNC Symbol;Acc:HGNC:6206]	19
ENSG00000213719	4424,329	0,046986	0,02606	0,215695	2,225322	0,047452	0,092597	protein_coding	CLIC1	chloride intracellular channel 1 [Source:HGNC Symbol;Acc:HGNC:2062]	6
ENSG00000184009	37322,56	0,042521	0,026058	0,215695	2,225339	0,061156	0,117747	protein_coding	ACTG1	actin gamma 1 [Source:HGNC Symbol;Acc:HGNC:144]	17
ENSG00000168036	8648,49	0,050245	0,02619	0,215999	2,223379	0,043078	0,08447	protein_coding	CTNNB1	catenin beta 1 [Source:HGNC Symbol;Acc:HGNC:2514]	3
ENSG00000117592	2270,399	0,043126	0,026517	0,217149	2,218549	0,043962	0,089528	protein_coding	PRDX6	peroxiredoxin 6 [Source:HGNC Symbol;Acc:HGNC:16753]	1
ENSG00000163527	3482,743	0,043564	0,026518	0,217149	2,218535	0,035444	0,074063	protein_coding	STT3B	STT3 oligosaccharyltransferase complex catalytic subunit B [Source:HGNC Symbol;Acc:HGNC:30611]	3
ENSG00000158710	5230,418	0,046291	0,02679	0,218593	2,214567	0,054584	0,102862	protein_coding	TAGLN2	transgelin 2 [Source:HGNC Symbol;Acc:HGNC:11554]	1
ENSG00000265241	1578,706	0,045858	0,027086	0,218686	2,210272	0,05135	0,098491	protein_coding	RBM8A	RNA binding motif protein 8A [Source:HGNC Symbol;Acc:HGNC:9905]	1
ENSG00000162704	2746,049	0,045303	0,027068	0,218686	2,210541	0,049536	0,096295	protein_coding	ARPC5	actin related protein 2/3 complex subunit 5 [Source:HGNC Symbol;Acc:HGNC:708]	1
ENSG00000160014	3348,393	0,049223	0,026989	0,218686	2,211682	0,046892	0,089831	protein_coding	CALM3	calmodulin 3 [Source:HGNC Symbol;Acc:HGNC:1449]	19
ENSG00000125944	2932,09	0,04597	0,027478	0,220336	2,204663	0,044798	0,088316	protein_coding	HNRNPR	heterogeneous nuclear ribonucleoprotein R [Source:HGNC Symbol;Acc:HGNC:5047]	1
ENSG00000173692	2948,584	0,040191	0,027578	0,220336	2,203238	0,029465	0,06337	protein_coding	PSMD1	proteasome 26S subunit, non-ATPase 1 [Source:HGNC Symbol;Acc:HGNC:9554]	2
ENSG00000013364	2858,957	0,045502	0,02754	0,220336	2,203773	0,051522	0,098819	protein_coding	MVP	major vault protein [Source:HGNC Symbol;Acc:HGNC:7531]	16
ENSG00000196230	12250,98	0,043228	0,028056	0,223377	2,196509	0,052987	0,103032	protein_coding	TUBB	tubulin beta class I [Source:HGNC Symbol;Acc:HGNC:20778]	6
ENSG00000113140	39908,68	0,021227	0,028876	0,229119	2,18517	0,037695	0,179015	protein_coding	SPARC	secreted protein acidic and cysteine rich [Source:HGNC Symbol;Acc:HGNC:11219]	5
ENSG00000154127	2095,58	0,032749	0,029288	0,23159	2,179583	0,049161	0,122387	protein_coding	UBASH3B	ubiquitin associated and SH3 domain containing B [Source:HGNC Symbol;Acc:HGNC:29884]	11
ENSG00000117519	4474,614	0,040345	0,029841	0,232047	2,172198	0,05767	0,114329	protein_coding	CNN3	calponin 3 [Source:HGNC Symbol;Acc:HGNC:2157]	1
ENSG00000071127	6597,04	0,04281	0,029603	0,232047	2,175368	0,046031	0,091527	protein_coding	WDR1	WD repeat domain 1 [Source:HGNC Symbol;Acc:HGNC:12754]	4
ENSG00000145555	11026,71	0,047372	0,02948	0,232047	2,177011	0,053168	0,098309	protein_coding	MYO10	myosin X [Source:HGNC Symbol;Acc:HGNC:7593]	5
ENSG00000161638	6341,217	0,023041	0,029718	0,232047	2,173833	0,039876	0,169267	protein_coding	ITGA5	integrin subunit alpha 5 [Source:HGNC Symbol;Acc:HGNC:6141]	12
ENSG00000165891	2168,005	0,03049	0,02985	0,232047	2,17207	0,049221	0,137741	protein_coding	E2F7	E2F transcription factor 7 [Source:HGNC Symbol;Acc:HGNC:23820]	12
ENSG00000072110	13018,13	0,037406	0,030951	0,239793	2,1577	0,050141	0,106799	protein_coding	ACTN1	actinin alpha 1 [Source:HGNC Symbol;Acc:HGNC:163]	14
ENSG00000185989	1614,934	0,037971	0,031091	0,240069	2,155904	0,049118	0,103254	protein_coding	RASA3	RAS p21 protein activator 3 [Source:HGNC Symbol;Acc:HGNC:20331]	13
ENSG00000160075	1887,764	0,040553	0,031802	0,240802	2,146889	0,035354	0,073058	protein_coding	SSU72	SSU72 homolog, RNA polymerase II CTD phosphatase [Source:HGNC Symbol;Acc:HGNC:25016]	1
ENSG00000116729	4037,796	0,035601	0,031654	0,240802	2,148747	0,053509	0,119666	protein_coding	WLS	Wnt ligand secretion mediator [Source:HGNC Symbol;Acc:HGNC:30238]	1
ENSG00000065135	1903,415	0,045904	0,031814	0,240802	2,146741	0,053665	0,098839	protein_coding	GNAI3	G protein subunit alpha i3 [Source:HGNC Symbol;Acc:HGNC:4387]	1
ENSG00000138674	5488,109	0,041264	0,031715	0,240802	2,147988	0,036478	0,074796	protein_coding	SEC31A	SEC31 homolog A, COPII coat complex component [Source:HGNC Symbol;Acc:HGNC:17052]	4
ENSG00000145741	2871,925	0,041774	0,031689	0,240802	2,148317	0,045818	0,091059	protein_coding	BTF3	basic transcription factor 3 [Source:HGNC Symbol;Acc:HGNC:1125]	5
ENSG00000070756	14234,8	0,042225	0,031611	0,240802	2,149297	0,028536	0,059323	protein_coding	PABPC1	poly(A) binding protein cytoplasmic 1 [Source:HGNC Symbol;Acc:HGNC:8554]	8
ENSG00000108298	10247,58	0,049626	0,032345	0,244022	2,140116	0,035505	0,069627	protein_coding	RPL19	ribosomal protein L19 [Source:HGNC Symbol;Acc:HGNC:10312]	17
ENSG00000163468	4178,462	0,040603	0,033099	0,247275	2,130881	0,036402	0,074475	protein_coding	CCT3	chaperonin containing TCP1 subunit 3 [Source:HGNC Symbol;Acc:HGNC:1616]	1
ENSG00000099783	3862,234	0,04095	0,033086	0,247275	2,131035	0,04581	0,091234	protein_coding	HNRNPM	heterogeneous nuclear ribonucleoprotein M [Source:HGNC Symbol;Acc:HGNC:5046]	19
ENSG00000102144	2874,64	0,044089	0,032886	0,247275	2,133475	0,037849	0,07521	protein_coding	PGK1	phosphoglycerate kinase 1 [Source:HGNC Symbol;Acc:HGNC:8896]	X
ENSG00000136010	1877,563	0,015591	0,033267	0,247727	2,128844	0,032158	0,215091	protein_coding	ALDH1L2	aldehyde dehydrogenase 1 family member L2 [Source:HGNC Symbol;Acc:HGNC:26777]	12
ENSG00000026508	10232,27	0,029127	0,033416	0,248031	2,127053	0,046539	0,133965	protein_coding	CD44	CD44 molecule (Indian blood group) [Source:HGNC Symbol;Acc:HGNC:1681]	11
ENSG00000089597	5343,619	0,036418	0,033801	0,250082	2,12244	0,025117	0,053475	protein_coding	GANAB	glucosidase II alpha subunit [Source:HGNC Symbol;Acc:HGNC:4138]	11

ENSG00000060982	2132,764	0,014625	0,034141	0,250988	2,118399	0,031405	0,224962	protein_coding	BCAT1	branched chain amino acid transaminase 1 [Source:HGNC Symbol;Acc:HGNC:976]	12
ENSG00000229117	1625,936	0,02156	0,034095	0,250988	2,118952	0,038009	0,170617	protein_coding	RPL41	ribosomal protein L41 [Source:HGNC Symbol;Acc:HGNC:10354]	12
ENSG00000102189	2211,857	0,028952	0,03431	0,251426	2,116407	0,046201	0,133163	protein_coding	EEA1	early endosome antigen 1 [Source:HGNC Symbol;Acc:HGNC:3185]	12
ENSG00000164587	1935,153	0,027813	0,034617	0,252803	2,112808	0,042401	0,12243	protein_coding	RPS14	ribosomal protein S14 [Source:HGNC Symbol;Acc:HGNC:10387]	5
ENSG00000175220	2226,662	0,03819	0,034718	0,252803	2,111635	0,048999	0,100508	protein_coding	ARHGAP1	Rho GTPase activating protein 1 [Source:HGNC Symbol;Acc:HGNC:673]	11
ENSG00000116199	1576,131	0,038229	0,034963	0,253067	2,108784	0,050823	0,103867	protein_coding	FAM20B	FAM20B glycosaminoglycan xylosylkinase [Source:HGNC Symbol;Acc:HGNC:23017]	1
ENSG00000164828	2404,204	-0,04117	0,034974	0,253067	-2,10866	0,038825	-0,07778	protein_coding	SUN1	Sad1 and UNC84 domain containing 1 [Source:HGNC Symbol;Acc:HGNC:18587]	7
ENSG00000108424	6664,723	0,039755	0,035095	0,253147	2,107259	0,04058	0,082098	protein_coding	KPNB1	karyopherin subunit beta 1 [Source:HGNC Symbol;Acc:HGNC:6400]	17
ENSG00000110321	17434,44	0,040253	0,035234	0,253352	2,105662	0,034705	0,070638	protein_coding	EIF4G2	eukaryotic translation initiation factor 4 gamma 2 [Source:HGNC Symbol;Acc:HGNC:3297]	11
ENSG00000113387	1770,419	0,040335	0,036271	0,256014	2,093871	0,044442	0,088041	protein_coding	SUB1	SUB1 regulator of transcription [Source:HGNC Symbol;Acc:HGNC:19985]	5
ENSG00000130396	2642,462	-0,04055	0,036178	0,256014	-2,09492	0,039756	-0,07939	protein_coding	AFDN	afadin, adherens junction formation factor [Source:HGNC Symbol;Acc:HGNC:7137]	6
ENSG00000105193	4360,39	0,042028	0,036028	0,256014	2,096614	0,042526	0,083154	protein_coding	RPS16	ribosomal protein S16 [Source:HGNC Symbol;Acc:HGNC:10396]	19
ENSG00000101384	3918,033	0,030367	0,035903	0,256014	2,09802	0,048719	0,131725	protein_coding	JAG1	jagged canonical Notch ligand 1 [Source:HGNC Symbol;Acc:HGNC:6188]	20
ENSG00000124225	2535,349	0,025528	0,036101	0,256014	2,095785	0,041946	0,141937	protein_coding	PMEPA1	prostate transmembrane protein, androgen induced 1 [Source:HGNC Symbol;Acc:HGNC:14107]	20
ENSG00000130741	2279,852	0,040576	0,035992	0,256014	2,09702	0,037201	0,074795	protein_coding	EIF2S3	eukaryotic translation initiation factor 2 subunit gamma [Source:HGNC Symbol;Acc:HGNC:3267]	X
ENSG00000198959	2384,808	0,017496	0,036637	0,257804	2,089785	0,033654	0,18792	protein_coding	TGM2	transglutaminase 2 [Source:HGNC Symbol;Acc:HGNC:11778]	20
ENSG00000143870	5409,658	0,040247	0,036794	0,258116	2,088047	0,043828	0,086753	protein_coding	PDIA6	protein disulfide isomerase family A member 6 [Source:HGNC Symbol;Acc:HGNC:30168]	2
ENSG00000071894	1724,162	-0,03172	0,037003	0,258793	-2,08574	0,036085	-0,08229	protein_coding	CPSF1	cleavage and polyadenylation specific factor 1 [Source:HGNC Symbol;Acc:HGNC:2324]	8
ENSG00000168374	3918,122	0,03773	0,037469	0,260577	2,08062	0,042876	0,087914	protein_coding	ARF4	ADP ribosylation factor 4 [Source:HGNC Symbol;Acc:HGNC:655]	3
ENSG00000165458	2178,96	-0,03706	0,037484	0,260577	-2,08045	0,028209	-0,0586	protein_coding	INPPL1	inositol polyphosphate phosphatase like 1 [Source:HGNC Symbol;Acc:HGNC:6080]	11
ENSG00000169116	1891,571	-0,018	0,037941	0,262168	-2,07549	0,033194	-0,15618	protein_coding	PARM1	prostate androgen-regulated mucin-like protein 1 [Source:HGNC Symbol;Acc:HGNC:24536]	4
ENSG00000125826	1814,641	-0,03821	0,037843	0,262168	-2,07655	0,045008	-0,0911	protein_coding	RBCK1	RANBP2-type and C3HC4-type zinc finger containing 1 [Source:HGNC Symbol;Acc:HGNC:15864]	20
ENSG00000091986	2200,851	0,017786	0,038539	0,265505	2,069074	0,033831	0,181131	protein_coding	CCDC80	coiled-coil domain containing 80 [Source:HGNC Symbol;Acc:HGNC:30649]	3
ENSG00000186340	15575,21	0,032898	0,038758	0,266137	2,066745	0,047947	0,111739	protein_coding	THBS2	thrombospondin 2 [Source:HGNC Symbol;Acc:HGNC:11786]	6
ENSG00000148702	1876,885	0,00737	0,039033	0,266137	2,063835	0,027402	0,395659	protein_coding	HABP2	hyaluronan binding protein 2 [Source:HGNC Symbol;Acc:HGNC:4798]	10
ENSG00000173801	4022,84	-0,03029	0,039094	0,266137	-2,0632	0,042959	-0,10703	protein_coding	JUP	junction plakoglobin [Source:HGNC Symbol;Acc:HGNC:6207]	17
ENSG00000147274	2806,57	0,038718	0,038914	0,266137	2,065097	0,035266	0,071335	protein_coding	RBMX	RNA binding motif protein X-linked [Source:HGNC Symbol;Acc:HGNC:9910]	X
ENSG00000161016	6653,768	0,039352	0,039464	0,267866	2,059316	0,038137	0,076075	protein_coding	RPL8	ribosomal protein L8 [Source:HGNC Symbol;Acc:HGNC:10368]	8
ENSG00000088986	2805,905	0,037314	0,039641	0,268278	2,057468	0,04662	0,095052	protein_coding	DYNLL1	dynein light chain LC8-type 1 [Source:HGNC Symbol;Acc:HGNC:15476]	12
ENSG00000156467	2228,115	0,037475	0,040446	0,272922	2,049163	0,044278	0,08975	protein_coding	UQCRB	ubiquinol-cytochrome c reductase binding protein [Source:HGNC Symbol;Acc:HGNC:12582]	8
ENSG00000196562	2245,657	0,010545	0,040934	0,275406	2,0442	0,028772	0,275145	protein_coding	SULF2	sulfatase 2 [Source:HGNC Symbol;Acc:HGNC:20392]	20
ENSG00000031698	3363,355	0,036122	0,041373	0,277547	2,039775	0,03266	0,067163	protein_coding	SARS1	seryl-tRNA synthetase 1 [Source:HGNC Symbol;Acc:HGNC:10537]	1
ENSG00000183431	1575,256	0,036683	0,041782	0,279481	2,035681	0,045102	0,092195	protein_coding	SF3A3	splicing factor 3a subunit 3 [Source:HGNC Symbol;Acc:HGNC:10767]	1
ENSG00000118181	2666,282	0,03477	0,042544	0,283748	2,028163	0,047098	0,100767	protein_coding	RPS25	ribosomal protein S25 [Source:HGNC Symbol;Acc:HGNC:10413]	11
ENSG00000100353	2274,592	0,037008	0,0427	0,283964	2,026637	0,033952	0,068695	protein_coding	EIF3D	eukaryotic translation initiation factor 3 subunit D [Source:HGNC Symbol;Acc:HGNC:3278]	22
ENSG00000076201	1739,061	-0,03718	0,043116	0,285844	-2,02259	0,040586	-0,08164	protein_coding	PTPN23	protein tyrosine phosphatase non-receptor type 23 [Source:HGNC Symbol;Acc:HGNC:14406]	3
ENSG00000122359	2150,208	0,037965	0,043231	0,285844	2,021475	0,035001	0,069908	protein_coding	ANXA11	annexin A11 [Source:HGNC Symbol;Acc:HGNC:535]	10
ENSG00000166441	3780,716	0,037595	0,04337	0,285943	2,02013	0,036521	0,073106	protein_coding	RPL27A	ribosomal protein L27a [Source:HGNC Symbol;Acc:HGNC:10329]	11
ENSG00000139926	2415,711	0,028663	0,043855	0,288313	2,015477	0,04338	0,115448	protein_coding	FRMD6	FERM domain containing 6 [Source:HGNC Symbol;Acc:HGNC:19839]	14
ENSG00000160691	3285,693	0,033376	0,044072	0,288916	2,013406	0,025009	0,051674	protein_coding	SHC1	SHC adaptor protein 1 [Source:HGNC Symbol;Acc:HGNC:10840]	1
ENSG00000120705	2584,224	0,037041	0,044351	0,289918	2,010761	0,040048	0,080319	protein_coding	ETF1	eukaryotic translation termination factor 1 [Source:HGNC Symbol;Acc:HGNC:3477]	5
ENSG00000063046	6582,844	0,033917	0,045089	0,293906	2,003827	0,030951	0,064118	protein_coding	EIF4B	eukaryotic translation initiation factor 4B [Source:HGNC Symbol;Acc:HGNC:3285]	12
ENSG00000110958	3472,197	0,037677	0,045259	0,294181	2,002243	0,03911	0,077417	protein_coding	PTGES3	prostaglandin E synthase 3 [Source:HGNC Symbol;Acc:HGNC:16049]	12
ENSG00000095139	5724,892	0,033314	0,045478	0,294772	2,00021	0,026815	0,055286	protein_coding	ARCN1	archain 1 [Source:HGNC Symbol;Acc:HGNC:649]	11
ENSG00000198722	1613,501	-0,03332	0,046921	0,303271	-1,98701	0,037109	-0,0783	protein_coding	UNC13B	unc-13 homolog B [Source:HGNC Symbol;Acc:HGNC:12566]	9
ENSG00000083444	5676,694	0,025515	0,047405	0,305544	1,982662	0,038572	0,110572	protein_coding	PLOD1	procollagen-lysine,2-oxoglutarate 5-dioxygenase 1 [Source:HGNC Symbol;Acc:HGNC:9081]	1
ENSG00000079246	5468,979	0,034331	0,04754	0,305557	1,981458	0,03605	0,074202	protein_coding	XRCC5	X-ray repair cross complementing 5 [Source:HGNC Symbol;Acc:HGNC:12833]	2
ENSG00000151474	2018,486	0,023499	0,047705	0,305763	1,979989	0,038572	0,130465	protein_coding	FRMD4A	FERM domain containing 4A [Source:HGNC Symbol;Acc:HGNC:25491]	10
ENSG00000090621	2315,792	0,036221	0,048014	0,306887	1,977248	0,035387	0,070565	protein_coding	PABPC4	poly(A) binding protein cytoplasmic 4 [Source:HGNC Symbol;Acc:HGNC:8557]	1
ENSG00000168137	3195,528	-0,03398	0,048275	0,307702	-1,97494	0,028186	-0,05715	protein_coding	SETD5	SET domain containing 5 [Source:HGNC Symbol;Acc:HGNC:25566]	3
ENSG00000119669	2763,798	-0,03593	0,048463	0,308045	-1,97329	0,038726	-0,07755	protein_coding	IRF2BPL	interferon regulatory factor 2 binding protein like [Source:HGNC Symbol;Acc:HGNC:14282]	14
ENSG00000116641	1695,725	0,031243	0,048734	0,308067	1,970914	0,041798	0,094967	protein_coding	DOCK7	dedicator of cytokinesis 7 [Source:HGNC Symbol;Acc:HGNC:19190]	1

ENSG00000111640	28821,36	0,033931	0,04869	0,308067	1,9713	0,027155	0,054883	protein_coding	GAPDH	glyceraldehyde-3-phosphate dehydrogenase [Source:HGNC Symbol;Acc:HGNC:4141]	12
ENSG00000176788	2364,914	0,029913	0,049204	0,308496	1,966821	0,046773	0,118758	protein_coding	BASP1	brain abundant membrane attached signal protein 1 [Source:HGNC Symbol;Acc:HGNC:957]	5
ENSG00000166716	1598,812	-0,03569	0,049023	0,308496	-1,96839	0,035345	-0,07065	protein_coding	ZNF592	zinc finger protein 592 [Source:HGNC Symbol;Acc:HGNC:28986]	15
ENSG00000101367	3024,052	0,04158	0,049101	0,308496	1,967715	0,039508	0,073348	protein_coding	MAPRE1	microtubule associated protein RP/EB family member 1 [Source:HGNC Symbol;Acc:HGNC:6890]	20
ENSG00000117298	5874,251	0,032311	0,051473	0,309241	1,947516	0,033872	0,070595	protein_coding	ECE1	endothelin converting enzyme 1 [Source:HGNC Symbol;Acc:HGNC:3146]	1
ENSG00000162889	2672,111	-0,03377	0,049807	0,309241	-1,96162	0,04001	-0,08334	protein_coding	MAPKAPK2	MAPK activated protein kinase 2 [Source:HGNC Symbol;Acc:HGNC:6887]	1
ENSG00000071082	3559,614	0,032214	0,050666	0,309241	1,954294	0,046782	0,105033	protein_coding	RPL31	ribosomal protein L31 [Source:HGNC Symbol;Acc:HGNC:10334]	2
ENSG00000197756	4031,084	0,031787	0,050851	0,309241	1,952734	0,042123	0,093234	protein_coding	RPL37A	ribosomal protein L37a [Source:HGNC Symbol;Acc:HGNC:10348]	2
ENSG00000065534	3231,712	0,026001	0,049821	0,309241	1,961498	0,039598	0,11181	protein_coding	MYLK	myosin light chain kinase [Source:HGNC Symbol;Acc:HGNC:7590]	3
ENSG00000127184	2527,1	0,036675	0,05105	0,309241	1,951057	0,034199	0,066885	protein_coding	COX7C	cytochrome c oxidase subunit 7C [Source:HGNC Symbol;Acc:HGNC:2292]	5
ENSG00000146278	1563,952	-0,02928	0,049603	0,309241	-1,96337	0,043186	-0,10785	protein_coding	PNRC1	proline rich nuclear receptor coactivator 1 [Source:HGNC Symbol;Acc:HGNC:17278]	6
ENSG00000122545	4441,586	0,038252	0,051036	0,309241	1,951177	0,044077	0,083929	protein_coding	SEPTIN7	septin 7 [Source:HGNC Symbol;Acc:HGNC:1717]	7
ENSG00000122515	2053,781	-0,03375	0,051042	0,309241	-1,95113	0,043016	-0,08995	protein_coding	ZMIZ2	zinc finger MIZ-type containing 2 [Source:HGNC Symbol;Acc:HGNC:22229]	7
ENSG00000122861	8409,768	0,016513	0,050533	0,309241	1,955424	0,032474	0,16968	protein_coding	PLAU	plasminogen activator, urokinase [Source:HGNC Symbol;Acc:HGNC:9052]	10
ENSG00000251562	74164,61	-0,0159	0,049933	0,309241	-1,96054	0,032018	-0,17546	lncRNA	MALAT1	metastasis associated lung adenocarcinoma transcript 1 [Source:HGNC Symbol;Acc:HGNC:29665]	11
ENSG00000111142	1746,63	0,03415	0,05109	0,309241	1,950727	0,039699	0,081437	protein_coding	METAP2	methionyl aminopeptidase 2 [Source:HGNC Symbol;Acc:HGNC:16672]	12
ENSG00000131143	3069,582	0,035688	0,050295	0,309241	1,957447	0,031029	0,061493	protein_coding	COX4I1	cytochrome c oxidase subunit 4I1 [Source:HGNC Symbol;Acc:HGNC:2265]	16
ENSG00000198242	3912,416	0,03465	0,050082	0,309241	1,95926	0,03942	0,080349	protein_coding	RPL23A	ribosomal protein L23a [Source:HGNC Symbol;Acc:HGNC:10317]	17
ENSG00000101335	5634,51	0,01353	0,051457	0,309241	1,947653	0,030398	0,201151	protein_coding	MYL9	myosin light chain 9 [Source:HGNC Symbol;Acc:HGNC:15754]	20
ENSG00000166681	1696,118	0,027293	0,051444	0,309241	1,947761	0,040197	0,105436	protein_coding	BEX3	brain expressed X-linked 3 [Source:HGNC Symbol;Acc:HGNC:13388]	X
ENSG00000180398	3722,765	0,032228	0,051682	0,309688	1,945774	0,04507	0,099751	protein_coding	MCFD2	multiple coagulation factor deficiency 2, ER cargo receptor complex subunit [Source:HGNC Symbol;Acc:HGNC:18451]	2
ENSG00000162734	4293,269	0,034896	0,051858	0,309938	1,944308	0,042247	0,085487	protein_coding	PEA15	proliferation and apoptosis adaptor protein 15 [Source:HGNC Symbol;Acc:HGNC:8822]	1
ENSG00000248527	24333,64	-0,01709	0,052324	0,311721	-1,94046	0,031473	-0,12887	unprocessed_pseu	MTATP6P1	MT-ATP6 pseudogene 1 [Source:HGNC Symbol;Acc:HGNC:44575]	1
ENSG00000142552	3616,878	0,023821	0,052428	0,311721	1,939605	0,038791	0,125678	protein_coding	RCN3	reticulocalbin 3 [Source:HGNC Symbol;Acc:HGNC:21145]	19
ENSG00000142599	4533,997	-0,042	0,052924	0,313055	-1,93554	0,029192	-0,05463	protein_coding	RERE	arginine-glutamic acid dipeptide repeats [Source:HGNC Symbol;Acc:HGNC:9965]	1
ENSG00000091136	10396,18	0,039057	0,052793	0,313055	1,936611	0,054714	0,100136	protein_coding	LAMB1	laminin subunit beta 1 [Source:HGNC Symbol;Acc:HGNC:6486]	7
ENSG00000091039	3065,143	0,032088	0,053093	0,313251	1,93416	0,042635	0,092836	protein_coding	OSBPL8	oxysterol binding protein like 8 [Source:HGNC Symbol;Acc:HGNC:16396]	12
ENSG00000152795	3582,178	0,032657	0,053296	0,313346	1,932517	0,03432	0,070616	protein_coding	HNRNPDL	heterogeneous nuclear ribonucleoprotein D like [Source:HGNC Symbol;Acc:HGNC:5037]	4
ENSG00000106299	1807,508	-0,03449	0,053927	0,313346	-1,92743	0,032697	-0,06487	protein_coding	WASL	WASP like actin nucleation promoting factor [Source:HGNC Symbol;Acc:HGNC:12735]	7
ENSG00000205339	4160,396	0,034236	0,053524	0,313346	1,930672	0,039469	0,079953	protein_coding	IPO7	importin 7 [Source:HGNC Symbol;Acc:HGNC:9852]	11
ENSG00000182985	2231,796	0,023186	0,053812	0,313346	1,928348	0,037805	0,124083	protein_coding	CADM1	cell adhesion molecule 1 [Source:HGNC Symbol;Acc:HGNC:5951]	11
ENSG00000123562	3147,584	0,035594	0,053404	0,313346	1,931643	0,033384	0,0655	protein_coding	MORF4L2	mortality factor 4 like 2 [Source:HGNC Symbol;Acc:HGNC:16849]	X
ENSG00000071859	1756,16	0,035027	0,053707	0,313346	1,929196	0,036402	0,072154	protein_coding	FAM50A	family with sequence similarity 50 member A [Source:HGNC Symbol;Acc:HGNC:18786]	X
ENSG00000188042	2014,485	0,037935	0,054849	0,313507	1,920071	0,043304	0,081639	protein_coding	ARL4C	ADP ribosylation factor like GTPase 4C [Source:HGNC Symbol;Acc:HGNC:698]	2
ENSG00000091527	3195,765	0,03255	0,054959	0,313507	1,9192	0,029202	0,058679	protein_coding	CDV3	CDV3 homolog [Source:HGNC Symbol;Acc:HGNC:26928]	3
ENSG00000038219	2699,797	0,03536	0,054763	0,313507	1,920753	0,034941	0,068478	protein_coding	BOD1L1	biorientation of chromosomes in cell division 1 like 1 [Source:HGNC Symbol;Acc:HGNC:31792]	4
ENSG00000164111	8062,963	0,033181	0,054918	0,313507	1,919523	0,040678	0,084267	protein_coding	ANXA5	annexin A5 [Source:HGNC Symbol;Acc:HGNC:543]	4
ENSG00000146112	2689,384	0,038265	0,054151	0,313507	1,925625	0,03869	0,073106	protein_coding	PPP1R18	protein phosphatase 1 regulatory subunit 18 [Source:HGNC Symbol;Acc:HGNC:29413]	6
ENSG00000147649	4900,284	0,034185	0,055044	0,313507	1,918526	0,032053	0,063504	protein_coding	MTDH	metadherin [Source:HGNC Symbol;Acc:HGNC:29608]	8
ENSG00000159461	1767,393	0,033642	0,054757	0,313507	1,9208	0,034265	0,068873	protein_coding	AMFR	autocrine motility factor receptor [Source:HGNC Symbol;Acc:HGNC:463]	16
ENSG00000091542	2349,973	-0,03354	0,054975	0,313507	-1,91907	0,040138	-0,08222	protein_coding	ALKBH5	alkB homolog 5, RNA demethylase [Source:HGNC Symbol;Acc:HGNC:25996]	17
ENSG00000168090	1834,29	0,033454	0,055186	0,313536	1,917412	0,042426	0,087746	protein_coding	COPS6	COP9 signalosome subunit 6 [Source:HGNC Symbol;Acc:HGNC:21749]	7
ENSG00000050165	3533,82	0,016021	0,05594	0,315484	1,911504	0,032	0,166253	protein_coding	DKK3	dickkopf WNT signaling pathway inhibitor 3 [Source:HGNC Symbol;Acc:HGNC:2893]	11
ENSG00000170581	1590,936	-0,03237	0,055851	0,315484	-1,9122	0,043563	-0,09342	protein_coding	STAT2	signal transducer and activator of transcription 2 [Source:HGNC Symbol;Acc:HGNC:11363]	12
ENSG00000105063	3486,333	-0,0336	0,055781	0,315484	-1,91275	0,032086	-0,06383	protein_coding	PPP6R1	protein phosphatase 6 regulatory subunit 1 [Source:HGNC Symbol;Acc:HGNC:29195]	19
ENSG00000124198	2928,902	-0,03492	0,056078	0,31549	-1,91043	0,031739	-0,06201	protein_coding	ARFGEF2	ADP ribosylation factor guanine nucleotide exchange factor 2 [Source:HGNC Symbol;Acc:HGNC:15853]	20
ENSG00000121774	2889,472	0,031794	0,058128	0,316496	1,894733	0,034893	0,071902	protein_coding	KHDRBS1	KH RNA binding domain containing, signal transduction associated 1 [Source:HGNC Symbol;Acc:HGNC:18116]	1
ENSG00000162244	2420,433	0,030262	0,057121	0,316496	1,902386	0,041269	0,093317	protein_coding	RPL29	ribosomal protein L29 [Source:HGNC Symbol;Acc:HGNC:10331]	3
ENSG00000114416	1983,837	0,030762	0,058671	0,316496	1,890649	0,036068	0,076391	protein_coding	FXR1	FMR1 autosomal homolog 1 [Source:HGNC Symbol;Acc:HGNC:4023]	3
ENSG00000138758	4438,323	0,022497	0,058288	0,316496	1,893523	0,036865	0,120943	protein_coding	SEPTIN11	septin 11 [Source:HGNC Symbol;Acc:HGNC:25589]	4
ENSG00000145901	2879,995	0,03244	0,05887	0,316496	1,889162	0,033554	0,067524	protein_coding	TNIP1	TNFAIP3 interacting protein 1 [Source:HGNC Symbol;Acc:HGNC:16903]	5
ENSG00000112081	2632,985	0,031598	0,056721	0,316496	1,905452	0,041144	0,088864	protein_coding	SRSF3	serine and arginine rich splicing factor 3 [Source:HGNC Symbol;Acc:HGNC:10785]	6

ENSG00000122705	2916,314	0,035249	0,057697	0,316496	1,897994	0,035302	0,068498	protein_coding	CLTA	clathrin light chain A [Source:HGNC Symbol;Acc:HGNC:2090]	9
ENSG00000196205	1755,706	0,013251	0,057509	0,316496	1,899423	0,029734	0,168756	processed_pseudog	EEF1A1P5	eukaryotic translation elongation factor 1 alpha 1 pseudogene 5 [Source:HGNC Symbol;Acc:HGNC:3200]	9
ENSG00000184743	2749,587	0,027115	0,056444	0,316496	1,907591	0,042017	0,113675	protein_coding	ATL3	atlastin GTPase 3 [Source:HGNC Symbol;Acc:HGNC:24526]	11
ENSG00000174903	1932,109	0,034416	0,058563	0,316496	1,891458	0,034829	0,068169	protein_coding	RAB1B	RAB1B, member RAS oncogene family [Source:HGNC Symbol;Acc:HGNC:18370]	11
ENSG00000166750	2643,123	0,024914	0,057741	0,316496	1,89766	0,039894	0,119902	protein_coding	SLFN5	schlafen family member 5 [Source:HGNC Symbol;Acc:HGNC:28286]	17
ENSG00000108819	2591,694	0,032874	0,057927	0,316496	1,896252	0,032445	0,064808	protein_coding	PPP1R9B	protein phosphatase 1 regulatory subunit 9B [Source:HGNC Symbol;Acc:HGNC:9298]	17
ENSG00000108821	233768	0,011659	0,057865	0,316496	1,896717	0,029237	0,216999	protein_coding	COL1A1	collagen type I alpha 1 chain [Source:HGNC Symbol;Acc:HGNC:2197]	17
ENSG00000089057	1660,6	-0,03427	0,057743	0,316496	-1,89764	0,041573	-0,08318	protein_coding	SLC23A2	solute carrier family 23 member 2 [Source:HGNC Symbol;Acc:HGNC:10973]	20
ENSG00000125968	1919,039	-0,03177	0,058845	0,316496	-1,88935	0,03841	-0,08035	protein_coding	ID1	inhibitor of DNA binding 1, HLH protein [Source:HGNC Symbol;Acc:HGNC:5360]	20
ENSG00000078814	2293,073	-0,00201	0,057717	0,316496	-1,89784	0,026175	-1,09303	protein_coding	MYH7B	myosin heavy chain 7B [Source:HGNC Symbol;Acc:HGNC:15906]	20
ENSG00000172216	1725,957	-0,02815	0,058312	0,316496	-1,89335	0,041352	-0,1028	protein_coding	CEBPB	CCAAT enhancer binding protein beta [Source:HGNC Symbol;Acc:HGNC:1834]	20
ENSG00000182944	3400,331	0,035026	0,057165	0,316496	1,902046	0,037497	0,073373	protein_coding	EWSR1	EWS RNA binding protein 1 [Source:HGNC Symbol;Acc:HGNC:3508]	22
ENSG00000198034	7408,892	0,031268	0,058851	0,316496	1,889304	0,030852	0,062635	protein_coding	RPS4X	ribosomal protein S4 X-linked [Source:HGNC Symbol;Acc:HGNC:10424]	X
ENSG00000173020	1820,078	-0,02588	0,059424	0,318729	-1,88504	0,033199	-0,07884	protein_coding	GRK2	G protein-coupled receptor kinase 2 [Source:HGNC Symbol;Acc:HGNC:289]	11
ENSG00000068366	4158,378	-0,03537	0,059606	0,318962	-1,8837	0,039134	-0,07567	protein_coding	ACSL4	acyl-CoA synthetase long chain family member 4 [Source:HGNC Symbol;Acc:HGNC:3571]	X
ENSG00000160789	8864,046	0,026237	0,060961	0,323485	1,873776	0,037989	0,097151	protein_coding	LMNA	lamin A/C [Source:HGNC Symbol;Acc:HGNC:6636]	1
ENSG00000133121	1621,982	0,02565	0,060968	0,323485	1,873726	0,038632	0,10396	protein_coding	STARD13	StAR related lipid transfer domain containing 13 [Source:HGNC Symbol;Acc:HGNC:19164]	13
ENSG00000141503	2225,276	-0,03008	0,06096	0,323485	-1,87379	0,037645	-0,08165	protein_coding	MINK1	misshapen like kinase 1 [Source:HGNC Symbol;Acc:HGNC:17565]	17
ENSG00000101210	1581,364	-0,01322	0,061014	0,323485	-1,8734	0,030077	-0,18756	protein_coding	EEF1A2	eukaryotic translation elongation factor 1 alpha 2 [Source:HGNC Symbol;Acc:HGNC:3192]	20
ENSG00000186566	1628,692	-0,03239	0,062132	0,328656	-1,86535	0,031982	-0,06322	protein_coding	GPATCH8	G-patch domain containing 8 [Source:HGNC Symbol;Acc:HGNC:29066]	17
ENSG00000099250	6925,561	0,033862	0,063033	0,332659	1,858957	0,039908	0,078588	protein_coding	NRP1	neuropilin 1 [Source:HGNC Symbol;Acc:HGNC:8004]	10
ENSG00000171150	1592,922	-0,02432	0,063646	0,334692	-1,85465	0,035251	-0,09264	protein_coding	SOCS5	suppressor of cytokine signaling 5 [Source:HGNC Symbol;Acc:HGNC:16852]	2
ENSG00000112773	1594,372	0,022021	0,063855	0,334692	1,853193	0,035905	0,114485	protein_coding	TENT5A	terminal nucleotidyltransferase 5A [Source:HGNC Symbol;Acc:HGNC:18345]	6
ENSG00000133636	4975,911	0,00329	0,063771	0,334692	1,853774	0,026341	0,730818	protein_coding	NTS	neurotensin [Source:HGNC Symbol;Acc:HGNC:8038]	12
ENSG00000122641	6318,537	0,017486	0,064072	0,335065	1,851681	0,033449	0,160248	protein_coding	INHBA	inhibin subunit beta A [Source:HGNC Symbol;Acc:HGNC:6066]	7
ENSG00000141458	1721,64	-0,02601	0,064267	0,335327	-1,85032	0,03932	-0,10393	protein_coding	NPC1	NPC intracellular cholesterol transporter 1 [Source:HGNC Symbol;Acc:HGNC:7897]	18
ENSG00000196367	3053,436	-0,03344	0,064586	0,336227	-1,84811	0,031772	-0,06119	protein_coding	TRRAP	transformation/transcription domain associated protein [Source:HGNC Symbol;Acc:HGNC:12347]	7
ENSG00000196591	2605,63	0,031916	0,065025	0,33699	1,845083	0,031167	0,06121	protein_coding	HDAC2	histone deacetylase 2 [Source:HGNC Symbol;Acc:HGNC:4853]	6
ENSG00000173120	2499,569	-0,0317	0,064944	0,33699	-1,84565	0,030546	-0,06005	protein_coding	KDM2A	lysine demethylase 2A [Source:HGNC Symbol;Acc:HGNC:13606]	11
ENSG00000196526	2273,389	0,023422	0,065421	0,337599	1,842373	0,036316	0,104204	protein_coding	AFAP1	actin filament associated protein 1 [Source:HGNC Symbol;Acc:HGNC:24017]	4
ENSG00000164692	102953,2	0,018721	0,06577	0,337599	1,839991	0,033656	0,132555	protein_coding	COL1A2	collagen type I alpha 2 chain [Source:HGNC Symbol;Acc:HGNC:2198]	7
ENSG00000128595	14553,8	0,028705	0,065877	0,337599	1,839263	0,038115	0,085459	protein_coding	CALU	calumenin [Source:HGNC Symbol;Acc:HGNC:1458]	7
ENSG00000179889	2883,957	-0,03012	0,065852	0,337599	-1,83943	0,034061	-0,07022	protein_coding	PDXDC1	pyridoxal dependent decarboxylase domain containing 1 [Source:HGNC Symbol;Acc:HGNC:28995]	16
ENSG00000198804	276943,6	-0,0213	0,065719	0,337599	-1,84034	0,035444	-0,11754	protein_coding	MT-CO1	mitochondrially encoded cytochrome c oxidase I [Source:HGNC Symbol;Acc:HGNC:7419]	MT
ENSG00000184863	1820,599	-0,03171	0,066167	0,33808	-1,83729	0,03779	-0,07669	protein_coding	RBM33	RNA binding motif protein 33 [Source:HGNC Symbol;Acc:HGNC:27223]	7
ENSG00000130811	1747,387	0,031814	0,066264	0,33808	1,836632	0,037411	0,075563	protein_coding	EIF3G	eukaryotic translation initiation factor 3 subunit G [Source:HGNC Symbol;Acc:HGNC:3274]	19
ENSG00000006451	1800,049	0,029681	0,066697	0,339534	1,833712	0,038727	0,084034	protein_coding	RALA	RAS like proto-oncogene A [Source:HGNC Symbol;Acc:HGNC:9839]	7
ENSG00000090520	1726,177	0,030093	0,067043	0,340541	1,831388	0,037147	0,078287	protein_coding	DNAJB11	DnaJ heat shock protein family (Hsp40) member B11 [Source:HGNC Symbol;Acc:HGNC:14889]	3
ENSG00000115677	20209,91	0,029264	0,067968	0,344479	1,825221	0,023765	0,046138	protein_coding	HDLBP	high density lipoprotein binding protein [Source:HGNC Symbol;Acc:HGNC:4857]	2
ENSG00000113758	4245,056	0,030692	0,068486	0,346344	1,821795	0,041992	0,090016	protein_coding	DBN1	drebrin 1 [Source:HGNC Symbol;Acc:HGNC:2695]	5
ENSG00000138119	6338,037	0,026785	0,068877	0,347559	1,819223	0,039445	0,098022	protein_coding	MYOF	myoferlin [Source:HGNC Symbol;Acc:HGNC:3656]	10
ENSG00000225630	2542,586	-0,01858	0,069155	0,348195	-1,81741	0,034046	-0,14166	unprocessed_pseu	MTND2P28	MT-ND2 pseudogene 28 [Source:HGNC Symbol;Acc:HGNC:42129]	1
ENSG00000104529	3751,997	0,032008	0,069655	0,349947	1,814149	0,037525	0,074558	protein_coding	EEF1D	eukaryotic translation elongation factor 1 delta [Source:HGNC Symbol;Acc:HGNC:3211]	8
ENSG00000167548	5841,274	-0,02625	0,070055	0,350427	-1,81156	0,03947	-0,10082	protein_coding	KMT2D	lysine methyltransferase 2D [Source:HGNC Symbol;Acc:HGNC:7133]	12
ENSG00000174437	6887,801	0,034089	0,069948	0,350427	1,81225	0,028016	0,052042	protein_coding	ATP2A2	ATPase sarcoplasmic/endoplasmic reticulum Ca2+ transporting 2 [Source:HGNC Symbol;Acc:HGNC:812]	12
ENSG00000126012	2862,7	-0,02934	0,071101	0,354886	-1,80483	0,025791	-0,05005	protein_coding	KDM5C	lysine demethylase 5C [Source:HGNC Symbol;Acc:HGNC:11114]	X
ENSG00000087365	5688,443	0,032483	0,071397	0,355574	1,802945	0,034102	0,065613	protein_coding	SF3B2	splicing factor 3b subunit 2 [Source:HGNC Symbol;Acc:HGNC:10769]	11
ENSG00000105372	4930,842	0,02915	0,071547	0,355574	1,801987	0,038041	0,082099	protein_coding	RPS19	ribosomal protein S19 [Source:HGNC Symbol;Acc:HGNC:10402]	19
ENSG00000198668	8710,754	0,029264	0,072078	0,357436	1,798628	0,0355	0,074172	protein_coding	CALM1	calmodulin 1 [Source:HGNC Symbol;Acc:HGNC:1442]	14
ENSG00000169100	2789,688	0,030276	0,072253	0,357537	1,797519	0,033282	0,066485	protein_coding	SLC25A6	solute carrier family 25 member 6 [Source:HGNC Symbol;Acc:HGNC:10992]	X
ENSG00000198363	8168,234	0,022938	0,072778	0,359359	1,794223	0,035931	0,102613	protein_coding	ASPH	aspartate beta-hydroxylase [Source:HGNC Symbol;Acc:HGNC:757]	8
ENSG00000110880	3424,041	0,022525	0,073147	0,360408	1,791914	0,035694	0,104217	protein_coding	CORO1C	coronin 1C [Source:HGNC Symbol;Acc:HGNC:2254]	12

ENSG00000047410	5865,652	0,026576	0,073409	0,360927	1,79028	0,028515	0,058922	protein_coding	TPR	translocated promoter region, nuclear basket protein [Source:HGNC Symbol;Acc:HGNC:12017]	1
ENSG00000119777	1740,441	0,030862	0,073774	0,361949	1,788013	0,030685	0,059309	protein_coding	TMEM214	transmembrane protein 214 [Source:HGNC Symbol;Acc:HGNC:25983]	2
ENSG00000185896	5334,924	-0,02802	0,074675	0,365587	-1,78246	0,022755	-0,04346	protein_coding	LAMP1	lysosomal associated membrane protein 1 [Source:HGNC Symbol;Acc:HGNC:6499]	13
ENSG00000105939	2145,517	0,029297	0,075176	0,36726	1,77939	0,032992	0,066533	protein_coding	ZC3HAV1	zinc finger CCCH-type containing, antiviral 1 [Source:HGNC Symbol;Acc:HGNC:23721]	7
ENSG00000143761	5410,821	0,02919	0,075788	0,368684	1,775667	0,02669	0,051351	protein_coding	ARF1	ADP ribosylation factor 1 [Source:HGNC Symbol;Acc:HGNC:652]	1
ENSG00000058272	2522,588	0,027552	0,075665	0,368684	1,776411	0,033543	0,071338	protein_coding	PPP1R12A	protein phosphatase 1 regulatory subunit 12A [Source:HGNC Symbol;Acc:HGNC:7618]	12
ENSG00000030582	5097,303	-0,02912	0,076253	0,370165	-1,77285	0,039596	-0,08568	protein_coding	GRN	granulin precursor [Source:HGNC Symbol;Acc:HGNC:4601]	17
ENSG00000105993	1716,863	0,02869	0,076517	0,370663	1,771265	0,035905	0,075562	protein_coding	DNAJB6	DnaJ heat shock protein family (Hsp40) member B6 [Source:HGNC Symbol;Acc:HGNC:14888]	7
ENSG00000198727	114344,2	-0,01816	0,076847	0,37148	-1,76928	0,032865	-0,12385	protein_coding	MT-CYB	mitochondrially encoded cytochrome b [Source:HGNC Symbol;Acc:HGNC:7427]	MT
ENSG00000117450	4202,262	0,030175	0,077121	0,371839	1,767641	0,031279	0,060722	protein_coding	PRDX1	peroxiredoxin 1 [Source:HGNC Symbol;Acc:HGNC:9352]	1
ENSG00000135052	3101,97	0,018401	0,077244	0,371839	1,766904	0,032671	0,11666	protein_coding	GOLM1	golgi membrane protein 1 [Source:HGNC Symbol;Acc:HGNC:15451]	9
ENSG00000161203	6333,408	0,027106	0,077434	0,371973	1,765775	0,036062	0,080321	protein_coding	AP2M1	adaptor related protein complex 2 subunit mu 1 [Source:HGNC Symbol;Acc:HGNC:564]	3
ENSG00000135829	3677,326	0,02851	0,078354	0,373278	1,760316	0,03311	0,067557	protein_coding	DHX9	DExH-box helicase 9 [Source:HGNC Symbol;Acc:HGNC:2750]	1
ENSG00000115053	12484,04	0,030473	0,078043	0,373278	1,762155	0,03758	0,075644	protein_coding	NCL	nucleolin [Source:HGNC Symbol;Acc:HGNC:7667]	2
ENSG00000113719	2168,985	0,029036	0,078003	0,373278	1,762394	0,035016	0,07182	protein_coding	ERGIC1	endoplasmic reticulum-golgi intermediate compartment 1 [Source:HGNC Symbol;Acc:HGNC:29205]	5
ENSG00000110700	1932,067	0,026109	0,078352	0,373278	1,76033	0,036822	0,086768	protein_coding	RPS13	ribosomal protein S13 [Source:HGNC Symbol;Acc:HGNC:10386]	11
ENSG00000107796	2356,894	0,007165	0,078547	0,373425	1,759177	0,027227	0,293799	protein_coding	ACTA2	actin alpha 2, smooth muscle [Source:HGNC Symbol;Acc:HGNC:130]	10
ENSG00000172037	6881,229	-0,02904	0,079369	0,375779	-1,75436	0,028339	-0,05462	protein_coding	LAMB2	laminin subunit beta 2 [Source:HGNC Symbol;Acc:HGNC:6487]	3
ENSG00000177697	3252,863	0,025759	0,079207	0,375779	1,755305	0,036075	0,084866	protein_coding	CD151	CD151 molecule (Raph blood group) [Source:HGNC Symbol;Acc:HGNC:1630]	11
ENSG00000159840	3668,77	0,027806	0,079915	0,377132	1,751181	0,039527	0,08964	protein_coding	ZYX	zyxin [Source:HGNC Symbol;Acc:HGNC:13200]	7
ENSG00000167291	2494,406	-0,02455	0,079983	0,377132	-1,75079	0,034958	-0,0849	protein_coding	TBC1D16	TBC1 domain family member 16 [Source:HGNC Symbol;Acc:HGNC:28356]	17
ENSG00000214022	2097,223	-0,0249	0,081306	0,381372	-1,74316	0,033705	-0,07781	protein_coding	REPIN1	replication initiator 1 [Source:HGNC Symbol;Acc:HGNC:17922]	7
ENSG00000133112	23707,88	0,028739	0,081379	0,381372	1,742739	0,029503	0,057303	protein_coding	TPT1	tumor protein, translationally-controlled 1 [Source:HGNC Symbol;Acc:HGNC:12022]	13
ENSG00000101152	1864,383	-0,02841	0,081228	0,381372	-1,74361	0,03407	-0,06969	protein_coding	DNAJC5	DnaJ heat shock protein family (Hsp40) member C5 [Source:HGNC Symbol;Acc:HGNC:16235]	20
ENSG00000144381	4300,961	0,028023	0,08207	0,383829	1,738798	0,031778	0,063923	protein_coding	HSPD1	heat shock protein family D (Hsp60) member 1 [Source:HGNC Symbol;Acc:HGNC:5261]	2
ENSG00000221983	3702,514	0,031476	0,082605	0,385545	1,735769	0,030805	0,057238	protein_coding	UBA52	ubiquitin A-52 residue ribosomal protein fusion product 1 [Source:HGNC Symbol;Acc:HGNC:12458]	19
ENSG00000204843	3926,436	0,027807	0,083027	0,38673	1,733388	0,026937	0,0518	protein_coding	DCTN1	dynactin subunit 1 [Source:HGNC Symbol;Acc:HGNC:2711]	2
ENSG00000137331	5544,346	-0,02139	0,083531	0,388294	-1,73055	0,034341	-0,09971	protein_coding	IER3	immediate early response 3 [Source:HGNC Symbol;Acc:HGNC:5392]	6
ENSG00000163297	4392,092	0,01978	0,084811	0,393448	1,723429	0,033495	0,106988	protein_coding	ANTXR2	ANTXR cell adhesion molecule 2 [Source:HGNC Symbol;Acc:HGNC:21732]	4
ENSG00000159335	4455,318	0,023095	0,085345	0,395129	1,72048	0,034513	0,088492	protein_coding	PTMS	parathymosin [Source:HGNC Symbol;Acc:HGNC:9629]	12
ENSG00000008710	2980,177	-0,02219	0,085566	0,395355	-1,71927	0,034633	-0,09501	protein_coding	PKD1	polycystin 1, transient receptor potential channel interacting [Source:HGNC Symbol;Acc:HGNC:9008]	16
ENSG00000169045	4367,216	0,028157	0,085874	0,395476	1,717576	0,030042	0,058478	protein_coding	HNRNPH1	heterogeneous nuclear ribonucleoprotein H1 [Source:HGNC Symbol;Acc:HGNC:5041]	5
ENSG00000245532	4809,174	-0,00783	0,085936	0,395476	-1,71724	0,027421	-0,2551	lncRNA	NEAT1	nuclear paraspeckle assembly transcript 1 [Source:HGNC Symbol;Acc:HGNC:30815]	11
ENSG00000134107	4187,337	0,026964	0,086419	0,396906	1,714598	0,038578	0,087948	protein_coding	BHLHE40	basic helix-loop-helix family member e40 [Source:HGNC Symbol;Acc:HGNC:1046]	3
ENSG00000135404	9110,566	0,029696	0,086855	0,398115	1,712225	0,033477	0,064628	protein_coding	CD63	CD63 molecule [Source:HGNC Symbol;Acc:HGNC:1692]	12
ENSG00000120885	3881,148	0,013392	0,087698	0,398876	1,707668	0,030374	0,181558	protein_coding	CLU	clusterin [Source:HGNC Symbol;Acc:HGNC:2095]	8
ENSG00000124942	24570,95	0,017327	0,087715	0,398876	1,707579	0,032038	0,119242	protein_coding	AHNAK	AHNAK nucleoprotein [Source:HGNC Symbol;Acc:HGNC:347]	11
ENSG00000187555	3116,641	0,026902	0,087378	0,398876	1,709394	0,030686	0,061728	protein_coding	USP7	ubiquitin specific peptidase 7 [Source:HGNC Symbol;Acc:HGNC:12630]	16
ENSG00000132646	2001,056	0,022923	0,08739	0,398876	1,709331	0,034636	0,089625	protein_coding	PCNA	proliferating cell nuclear antigen [Source:HGNC Symbol;Acc:HGNC:8729]	20
ENSG00000132549	1659,126	-0,02504	0,088412	0,401252	-1,70384	0,034475	-0,0786	protein_coding	VPS13B	vacuolar protein sorting 13 homolog B [Source:HGNC Symbol;Acc:HGNC:2183]	8
ENSG00000178952	2397,391	0,027737	0,088866	0,402521	1,70141	0,03038	0,059325	protein_coding	TUFM	Tu translation elongation factor, mitochondrial [Source:HGNC Symbol;Acc:HGNC:12420]	16
ENSG00000130175	5449,092	0,020927	0,089172	0,403112	1,699783	0,026391	0,057726	protein_coding	PRKCSH	protein kinase C substrate 80K-H [Source:HGNC Symbol;Acc:HGNC:9411]	19
ENSG00000092201	3322,683	0,027441	0,08943	0,403488	1,698411	0,030837	0,060835	protein_coding	SUPT16H	SPT16 homolog, facilitates chromatin remodeling subunit [Source:HGNC Symbol;Acc:HGNC:11465]	14
ENSG00000147548	2126,32	-0,02481	0,089742	0,4041	-1,69676	0,030717	-0,06519	protein_coding	NSD3	nuclear receptor binding SET domain protein 3 [Source:HGNC Symbol;Acc:HGNC:12767]	8
ENSG00000023734	2007,928	0,028351	0,090607	0,405613	1,692203	0,029496	0,056071	protein_coding	STRAP	serine/threonine kinase receptor associated protein [Source:HGNC Symbol;Acc:HGNC:30796]	12
ENSG00000102606	1644,547	-0,02718	0,090783	0,405613	-1,69128	0,032428	-0,06539	protein_coding	ARHGEF7	Rho guanine nucleotide exchange factor 7 [Source:HGNC Symbol;Acc:HGNC:15607]	13
ENSG00000074755	2130,507	-0,02218	0,090507	0,405613	-1,69273	0,025869	-0,0539	protein_coding	ZZEF1	zinc finger ZZ-type and EF-hand domain containing 1 [Source:HGNC Symbol;Acc:HGNC:29027]	17
ENSG00000160007	4550,994	-0,0276	0,090326	0,405613	-1,69368	0,030185	-0,05872	protein_coding	ARHGAP35	Rho GTPase activating protein 35 [Source:HGNC Symbol;Acc:HGNC:4591]	19
ENSG00000204628	7576,745	0,022448	0,090984	0,405727	1,690228	0,023769	0,047325	protein_coding	RACK1	receptor for activated C kinase 1 [Source:HGNC Symbol;Acc:HGNC:4399]	5
ENSG00000169230	1764,594	0,02573	0,09144	0,406971	1,687849	0,035943	0,081169	protein_coding	PRELID1	PRELI domain containing 1 [Source:HGNC Symbol;Acc:HGNC:30255]	5
ENSG00000089157	12099,7	0,027724	0,091657	0,407148	1,686722	0,031787	0,062481	protein_coding	RPLP0	ribosomal protein lateral stalk subunit P0 [Source:HGNC Symbol;Acc:HGNC:10371]	12
ENSG00000141568	1682,881	-0,0217	0,092033	0,408031	-1,68477	0,030088	-0,07032	protein_coding	FOXK2	forkhead box K2 [Source:HGNC Symbol;Acc:HGNC:6036]	17

ENSG00000178719	2125,736	-0,02455	0,092721	0,410291	-1,68122	0,034392	-0,07902	protein_coding	GRINA	glutamate ionotropic receptor NMDA type subunit associated protein 1 [Source:HGNC Symbol;Acc:HGNC:4589]	8
ENSG00000164484	2128,253	0,016451	0,093221	0,410922	1,678647	0,030668	0,107491	protein_coding	TMEM200/	transmembrane protein 200A [Source:HGNC Symbol;Acc:HGNC:21075]	6
ENSG00000011426	1596,216	0,007801	0,093049	0,410922	1,679529	0,027386	0,2457	protein_coding	ANLN	anillin actin binding protein [Source:HGNC Symbol;Acc:HGNC:14082]	7
ENSG00000005007	2656,084	-0,02756	0,093859	0,412942	-1,67539	0,033266	-0,0664	protein_coding	UPF1	UPF1 RNA helicase and ATPase [Source:HGNC Symbol;Acc:HGNC:9962]	19
ENSG00000127463	2120,221	0,026308	0,095052	0,413773	1,669329	0,030916	0,061853	protein_coding	EMC1	ER membrane protein complex subunit 1 [Source:HGNC Symbol;Acc:HGNC:28957]	1
ENSG00000084623	2066,648	0,027009	0,094908	0,413773	1,670055	0,029549	0,057089	protein_coding	EIF3I	eukaryotic translation initiation factor 3 subunit I [Source:HGNC Symbol;Acc:HGNC:3272]	1
ENSG00000143742	2183,535	0,025054	0,095838	0,413773	1,665374	0,035442	0,080742	protein_coding	SRP9	signal recognition particle 9 [Source:HGNC Symbol;Acc:HGNC:11304]	1
ENSG00000114573	2353,42	-0,0264	0,095846	0,413773	-1,66534	0,030781	-0,06115	protein_coding	ATP6V1A	ATPase H+ transporting V1 subunit A [Source:HGNC Symbol;Acc:HGNC:851]	3
ENSG00000187837	2931,971	0,019312	0,095501	0,413773	1,667067	0,032742	0,100413	protein_coding	H1-2	H1.2 linker histone, cluster member [Source:HGNC Symbol;Acc:HGNC:4716]	6
ENSG00000111912	1595,4	-0,02505	0,095632	0,413773	-1,66641	0,033821	-0,07432	protein_coding	NCOA7	nuclear receptor coactivator 7 [Source:HGNC Symbol;Acc:HGNC:21081]	6
ENSG00000111676	3948,183	-0,02719	0,095257	0,413773	-1,6683	0,032004	-0,06328	protein_coding	ATN1	atrophin 1 [Source:HGNC Symbol;Acc:HGNC:3033]	12
ENSG00000166949	2427,734	0,023652	0,094747	0,413773	1,67087	0,033754	0,079349	protein_coding	SMAD3	SMAD family member 3 [Source:HGNC Symbol;Acc:HGNC:6769]	15
ENSG00000169710	4927,74	-0,02433	0,095093	0,413773	-1,66912	0,033267	-0,07465	protein_coding	FASN	fatty acid synthase [Source:HGNC Symbol;Acc:HGNC:3594]	17
ENSG00000212907	27670,91	-0,0163	0,095523	0,413773	-1,66696	0,031257	-0,11918	protein_coding	MT-ND4L	mitochondrially encoded NADH:ubiquinone oxidoreductase core subunit 4L [Source:HGNC Symbol;Acc:HGNC:7460]	MT
ENSG00000183963	2260,225	0,018964	0,096444	0,415577	1,662343	0,032769	0,1042	protein_coding	SMTN	smoothelin [Source:HGNC Symbol;Acc:HGNC:11126]	22
ENSG00000049323	6751,796	0,017142	0,096773	0,415682	1,660704	0,031667	0,112613	protein_coding	LTBP1	latent transforming growth factor beta binding protein 1 [Source:HGNC Symbol;Acc:HGNC:6714]	2
ENSG00000113558	3357,172	0,025779	0,09683	0,415682	1,66042	0,029993	0,059769	protein_coding	SKP1	S-phase kinase associated protein 1 [Source:HGNC Symbol;Acc:HGNC:10899]	5
ENSG00000168298	2936,146	0,019033	0,097047	0,415839	1,659341	0,032559	0,10082	protein_coding	H1-4	H1.4 linker histone, cluster member [Source:HGNC Symbol;Acc:HGNC:4718]	6
ENSG00000237973	3464,84	-0,01132	0,099832	0,417049	-1,64567	0,028731	-0,16546	unprocessed_pseu	MTCO1P12	MT-CO1 pseudogene 12 [Source:HGNC Symbol;Acc:HGNC:52014]	1
ENSG00000143621	2098,183	0,025941	0,098883	0,417049	1,650292	0,030942	0,061861	protein_coding	ILF2	interleukin enhancer binding factor 2 [Source:HGNC Symbol;Acc:HGNC:6037]	1
ENSG00000076356	2013,168	-0,01967	0,09999	0,417049	-1,6449	0,032764	-0,09593	protein_coding	PLXNA2	plexin A2 [Source:HGNC Symbol;Acc:HGNC:9100]	1
ENSG00000136731	3706,318	0,026463	0,097684	0,417049	1,656186	0,028373	0,054277	protein_coding	UGGT1	UDP-glucose glycoprotein glucosyltransferase 1 [Source:HGNC Symbol;Acc:HGNC:15663]	2
ENSG00000173473	3225,216	0,026146	0,100154	0,417049	1,644107	0,024063	0,043892	protein_coding	SMARCC1	SWI/SNF related, matrix associated, actin dependent regulator of chromatin subfamily c member 1 [Source:HGNC Symb	3
ENSG00000169429	1721,824	-0,00541	0,099832	0,417049	-1,64567	0,026704	-0,33261	protein_coding	CXCL8	C-X-C motif chemokine ligand 8 [Source:HGNC Symbol;Acc:HGNC:6025]	4
ENSG00000124795	3107,902	0,02501	0,098161	0,417049	1,653833	0,035558	0,08077	protein_coding	DEK	DEK proto-oncogene [Source:HGNC Symbol;Acc:HGNC:2768]	6
ENSG00000122565	3050,139	0,024175	0,098775	0,417049	1,650823	0,03084	0,065277	protein_coding	CBX3	chromobox 3 [Source:HGNC Symbol;Acc:HGNC:1553]	7
ENSG00000106244	2287,355	0,023002	0,098364	0,417049	1,652837	0,032572	0,075717	protein_coding	PDAP1	PDGFA associated protein 1 [Source:HGNC Symbol;Acc:HGNC:14634]	7
ENSG00000122884	2609,191	0,020925	0,099113	0,417049	1,649169	0,033445	0,091748	protein_coding	P4HA1	prolyl 4-hydroxylase subunit alpha 1 [Source:HGNC Symbol;Acc:HGNC:8546]	10
ENSG00000109971	20691,06	0,030566	0,100229	0,417049	1,643742	0,033455	0,060775	protein_coding	HSPA8	heat shock protein family A (Hsp70) member 8 [Source:HGNC Symbol;Acc:HGNC:5241]	11
ENSG00000117906	1578,585	0,024583	0,099874	0,417049	1,645466	0,032982	0,071652	protein_coding	RCN2	reticulocalbin 2 [Source:HGNC Symbol;Acc:HGNC:9935]	15
ENSG00000108691	2468,004	-0,01065	0,099229	0,417049	-1,6486	0,028448	-0,1765	protein_coding	CCL2	C-C motif chemokine ligand 2 [Source:HGNC Symbol;Acc:HGNC:10618]	17
ENSG00000024422	3671,502	0,014393	0,098777	0,417049	1,650812	0,03	0,125708	protein_coding	EHD2	EH domain containing 2 [Source:HGNC Symbol;Acc:HGNC:3243]	19
ENSG00000156261	2521,491	0,026639	0,098101	0,417049	1,65413	0,03086	0,060572	protein_coding	CCT8	chaperonin containing TCP1 subunit 8 [Source:HGNC Symbol;Acc:HGNC:1623]	21
ENSG00000198786	158608,8	-0,01659	0,099357	0,417049	-1,64798	0,031302	-0,11385	protein_coding	MT-ND5	mitochondrially encoded NADH:ubiquinone oxidoreductase core subunit 5 [Source:HGNC Symbol;Acc:HGNC:7461]	MT
ENSG00000143947	4233,632	0,023041	0,100466	0,417281	1,642596	0,032982	0,07704	protein_coding	RPS27A	ribosomal protein S27a [Source:HGNC Symbol;Acc:HGNC:10417]	2
ENSG00000165092	3223,952	0,007173	0,100737	0,417649	1,641293	0,02717	0,252971	protein_coding	ALDH1A1	aldehyde dehydrogenase 1 family member A1 [Source:HGNC Symbol;Acc:HGNC:402]	9
ENSG00000143641	3918,446	0,025103	0,101679	0,420798	1,636767	0,034083	0,073793	protein_coding	GALNT2	polypeptide N-acetylgalactosaminyltransferase 2 [Source:HGNC Symbol;Acc:HGNC:4124]	1
ENSG00000188612	2133,188	0,033078	0,102158	0,422022	1,634479	0,035704	0,061879	protein_coding	SUMO2	small ubiquitin like modifier 2 [Source:HGNC Symbol;Acc:HGNC:11125]	17
ENSG00000118200	3219,143	0,026554	0,102634	0,42247	1,632217	0,033573	0,06785	protein_coding	CAMSAP2	calmodulin regulated spectrin associated protein family member 2 [Source:HGNC Symbol;Acc:HGNC:29188]	1
ENSG00000168487	1905,127	0,013945	0,10249	0,42247	1,632902	0,029931	0,132563	protein_coding	BMP1	bone morphogenetic protein 1 [Source:HGNC Symbol;Acc:HGNC:1067]	8
ENSG00000109814	2843,309	0,022997	0,103767	0,424486	1,626858	0,032179	0,072779	protein_coding	UGDH	UDP-glucose 6-dehydrogenase [Source:HGNC Symbol;Acc:HGNC:12525]	4
ENSG00000067082	2267,166	0,016307	0,103498	0,424486	1,628127	0,031108	0,113628	protein_coding	KLF6	Kruppel like factor 6 [Source:HGNC Symbol;Acc:HGNC:2235]	10
ENSG00000065613	3163,52	0,023078	0,103862	0,424486	1,626414	0,03128	0,06876	protein_coding	SLK	STE20 like kinase [Source:HGNC Symbol;Acc:HGNC:11088]	10
ENSG00000101558	2509,397	0,025944	0,103826	0,424486	1,626583	0,028701	0,054814	protein_coding	VAPA	VAMP associated protein A [Source:HGNC Symbol;Acc:HGNC:12648]	18
ENSG00000106538	1736,1	-0,00452	0,104548	0,426533	-1,62319	0,026514	-0,38717	protein_coding	RARRES2	retinoic acid receptor responder 2 [Source:HGNC Symbol;Acc:HGNC:9868]	7
ENSG00000185787	4653,743	0,02359	0,104903	0,427225	1,621534	0,031425	0,067645	protein_coding	MORF4L1	mortality factor 4 like 1 [Source:HGNC Symbol;Acc:HGNC:16989]	15
ENSG00000141522	4806,778	0,023764	0,105462	0,427375	1,618931	0,032879	0,072748	protein_coding	ARHGDIA	Rho GDP dissociation inhibitor alpha [Source:HGNC Symbol;Acc:HGNC:678]	17
ENSG00000115268	3558,686	0,024182	0,105338	0,427375	1,61951	0,031096	0,064797	protein_coding	RPS15	ribosomal protein S15 [Source:HGNC Symbol;Acc:HGNC:10388]	19
ENSG00000198792	2826,176	-0,02458	0,105497	0,427375	-1,61877	0,030718	-0,06265	protein_coding	TMEM184I	transmembrane protein 184B [Source:HGNC Symbol;Acc:HGNC:1310]	22
ENSG00000155660	5885,47	0,022931	0,105755	0,427666	1,617572	0,03272	0,075017	protein_coding	PDIA4	protein disulfide isomerase family A member 4 [Source:HGNC Symbol;Acc:HGNC:30167]	7
ENSG00000138095	2507,972	0,02689	0,106825	0,42898	1,612628	0,027575	0,050462	protein_coding	LRPPRC	leucine rich pentatricopeptide repeat containing [Source:HGNC Symbol;Acc:HGNC:15714]	2
ENSG00000164405	1733,68	0,022961	0,106321	0,42898	1,614953	0,033077	0,076429	protein_coding	UQCRQ	ubiquinol-cytochrome c reductase complex III subunit VII [Source:HGNC Symbol;Acc:HGNC:29594]	5

ENSG00000122966	2151,471	0,015075	0,106814	0,42898	1,612679	0,030412	0,119732	protein_coding	CIT	citron rho-interacting serine/threonine kinase [Source:HGNC Symbol;Acc:HGNC:1985]	12
ENSG00000100380	4122,351	0,023899	0,106698	0,42898	1,613215	0,028613	0,057112	protein_coding	ST13	ST13 Hsp70 interacting protein [Source:HGNC Symbol;Acc:HGNC:11343]	22
ENSG00000113810	3577,641	0,019456	0,107321	0,429014	1,610352	0,032561	0,094003	protein_coding	SMC4	structural maintenance of chromosomes 4 [Source:HGNC Symbol;Acc:HGNC:14013]	3
ENSG00000112335	2169,712	0,023465	0,107379	0,429014	1,610085	0,031949	0,069558	protein_coding	SNX3	sorting nexin 3 [Source:HGNC Symbol;Acc:HGNC:11174]	6
ENSG00000142541	14121,28	0,02459	0,107393	0,429014	1,610021	0,026747	0,050752	protein_coding	RPL13A	ribosomal protein L13a [Source:HGNC Symbol;Acc:HGNC:10304]	19
ENSG00000077097	3073,193	0,024119	0,10793	0,429664	1,607569	0,031606	0,06633	protein_coding	TOP2B	DNA topoisomerase II beta [Source:HGNC Symbol;Acc:HGNC:11990]	3
ENSG00000118898	2008,988	-0,01931	0,107873	0,429664	-1,60783	0,032176	-0,09175	protein_coding	PPL	periplakin [Source:HGNC Symbol;Acc:HGNC:9273]	16
ENSG00000091129	1630,363	-0,02034	0,108179	0,429914	-1,60643	0,034113	-0,10022	protein_coding	NRCAM	neuronal cell adhesion molecule [Source:HGNC Symbol;Acc:HGNC:7994]	7
ENSG00000133606	1947,755	0,02551	0,108392	0,430015	1,605465	0,031333	0,062151	protein_coding	MKRN1	makorin ring finger protein 1 [Source:HGNC Symbol;Acc:HGNC:7112]	7
ENSG00000169223	2469,624	0,021719	0,1087	0,430189	1,604062	0,032985	0,081253	protein_coding	LMAN2	lectin, mannose binding 2 [Source:HGNC Symbol;Acc:HGNC:16986]	5
ENSG00000198898	1935,375	0,023186	0,109104	0,430189	1,602232	0,032696	0,073266	protein_coding	CAPZA2	capping actin protein of muscle Z-line subunit alpha 2 [Source:HGNC Symbol;Acc:HGNC:1490]	7
ENSG00000004700	2047,324	0,022619	0,109183	0,430189	1,601875	0,032378	0,073992	protein_coding	RECQL	RecQ like helicase [Source:HGNC Symbol;Acc:HGNC:9948]	12
ENSG00000133961	2581,923	0,027475	0,109102	0,430189	1,602241	0,026746	0,047724	protein_coding	NUMB	NUMB endocytic adaptor protein [Source:HGNC Symbol;Acc:HGNC:8060]	14
ENSG00000123684	1893,717	0,025754	0,109572	0,430982	1,600122	0,030378	0,058706	protein_coding	LPGAT1	lysophosphatidylglycerol acyltransferase 1 [Source:HGNC Symbol;Acc:HGNC:28985]	1
ENSG00000172380	2264,625	0,01959	0,109795	0,431122	1,599117	0,03236	0,090455	protein_coding	GNG12	G protein subunit gamma 12 [Source:HGNC Symbol;Acc:HGNC:19663]	1
ENSG00000138069	2476,173	0,018706	0,11066	0,432133	1,595236	0,028777	0,070199	protein_coding	RAB1A	RAB1A, member RAS oncogene family [Source:HGNC Symbol;Acc:HGNC:9758]	2
ENSG00000138434	6387,574	-0,02734	0,110803	0,432133	-1,59459	0,030207	-0,05572	protein_coding	ITPRID2	ITPR interacting domain containing 2 [Source:HGNC Symbol;Acc:HGNC:11319]	2
ENSG00000009413	1750,424	-0,02227	0,110551	0,432133	-1,59572	0,033458	-0,08071	protein_coding	REV3L	REV3 like, DNA directed polymerase zeta catalytic subunit [Source:HGNC Symbol;Acc:HGNC:9968]	6
ENSG00000198542	2979,344	0,011372	0,110684	0,432133	1,595128	0,028671	0,15448	protein_coding	ITGBL1	integrin subunit beta like 1 [Source:HGNC Symbol;Acc:HGNC:6164]	13
ENSG00000134419	1662,104	0,022154	0,111468	0,433989	1,591631	0,032018	0,07363	protein_coding	RPS15A	ribosomal protein S15a [Source:HGNC Symbol;Acc:HGNC:10389]	16
ENSG00000133030	7386,995	0,022282	0,111914	0,434989	1,58965	0,034335	0,085366	protein_coding	MPRIP	myosin phosphatase Rho interacting protein [Source:HGNC Symbol;Acc:HGNC:30321]	17
ENSG00000081320	3200,633	0,018897	0,112514	0,435213	1,586994	0,03179	0,090218	protein_coding	STK17B	serine/threonine kinase 17b [Source:HGNC Symbol;Acc:HGNC:11396]	2
ENSG00000106261	3906,752	-0,02772	0,112539	0,435213	-1,58688	0,032522	-0,06065	protein_coding	ZKSCAN1	zinc finger with KRAB and SCAN domains 1 [Source:HGNC Symbol;Acc:HGNC:13101]	7
ENSG00000125844	10303,83	0,022359	0,112465	0,435213	1,587212	0,032844	0,076578	protein_coding	RRBP1	ribosome binding protein 1 [Source:HGNC Symbol;Acc:HGNC:10448]	20
ENSG00000169504	5055,863	0,020485	0,115723	0,436932	1,572984	0,032122	0,081263	protein_coding	CLIC4	chloride intracellular channel 4 [Source:HGNC Symbol;Acc:HGNC:13518]	1
ENSG00000163659	2738,143	-0,01998	0,114233	0,436932	-1,57945	0,030226	-0,07264	protein_coding	TIPARP	TCDD inducible poly(ADP-ribose) polymerase [Source:HGNC Symbol;Acc:HGNC:23696]	3
ENSG00000136527	1945,71	0,024121	0,116021	0,436932	1,571695	0,028795	0,055839	protein_coding	TRA2B	transformer 2 beta homolog [Source:HGNC Symbol;Acc:HGNC:10781]	3
ENSG00000039560	3284,726	0,017491	0,114415	0,436932	1,578657	0,030821	0,09227	protein_coding	RAI14	retinoic acid induced 14 [Source:HGNC Symbol;Acc:HGNC:14873]	5
ENSG00000135318	7079,128	0,014983	0,115986	0,436932	1,571847	0,030183	0,113108	protein_coding	NT5E	5'-nucleotidase ecto [Source:HGNC Symbol;Acc:HGNC:8021]	6
ENSG00000127920	1619,988	0,015407	0,115058	0,436932	1,575861	0,030392	0,110694	protein_coding	GNG11	G protein subunit gamma 11 [Source:HGNC Symbol;Acc:HGNC:4403]	7
ENSG00000187079	3507,701	0,025952	0,11591	0,436932	1,572176	0,02901	0,053823	protein_coding	TEAD1	TEA domain transcription factor 1 [Source:HGNC Symbol;Acc:HGNC:11714]	11
ENSG00000011405	1666,599	-0,02395	0,113614	0,436932	-1,58216	0,030574	-0,06216	protein_coding	PIK3C2A	phosphatidylinositol-4-phosphate 3-kinase catalytic subunit type 2 alpha [Source:HGNC Symbol;Acc:HGNC:8971]	11
ENSG00000100503	3002,434	0,019176	0,113309	0,436932	1,583494	0,032035	0,090063	protein_coding	NIN	ninein [Source:HGNC Symbol;Acc:HGNC:14906]	14
ENSG00000007202	5749,259	-0,02303	0,113978	0,436932	-1,58056	0,0213	-0,0379	protein_coding	KIAA0100	KIAA0100 [Source:HGNC Symbol;Acc:HGNC:28960]	17
ENSG00000002834	6672,334	0,021088	0,114219	0,436932	1,579512	0,030722	0,070819	protein_coding	LASP1	LIM and SH3 protein 1 [Source:HGNC Symbol;Acc:HGNC:6513]	17
ENSG00000005884	5091,507	0,01426	0,113473	0,436932	1,582773	0,030126	0,126937	protein_coding	ITGA3	integrin subunit alpha 3 [Source:HGNC Symbol;Acc:HGNC:6139]	17
ENSG00000035862	6296,286	0,017687	0,114763	0,436932	1,577142	0,02871	0,073124	protein_coding	TIMP2	TIMP metallopeptidase inhibitor 2 [Source:HGNC Symbol;Acc:HGNC:11821]	17
ENSG00000101421	2579,907	0,024748	0,115407	0,436932	1,574346	0,027378	0,051086	protein_coding	CHMP4B	charged multivesicular body protein 4B [Source:HGNC Symbol;Acc:HGNC:16171]	20
ENSG00000142156	21262,72	0,014245	0,115511	0,436932	1,573897	0,029865	0,119291	protein_coding	COL6A1	collagen type VI alpha 1 chain [Source:HGNC Symbol;Acc:HGNC:2211]	21
ENSG00000147403	4356,952	0,024572	0,115059	0,436932	1,575858	0,02766	0,052117	protein_coding	RPL10	ribosomal protein L10 [Source:HGNC Symbol;Acc:HGNC:10298]	X
ENSG00000115993	2968,908	0,018509	0,117055	0,43724	1,567257	0,030247	0,079308	protein_coding	TRAK2	trafficking kinesin protein 2 [Source:HGNC Symbol;Acc:HGNC:13206]	2
ENSG00000075785	6026,679	0,024059	0,116707	0,43724	1,568745	0,024629	0,044686	protein_coding	RAB7A	RAB7A, member RAS oncogene family [Source:HGNC Symbol;Acc:HGNC:9788]	3
ENSG00000170759	9015,592	0,022233	0,117433	0,43724	1,565638	0,031443	0,069503	protein_coding	KIF5B	kinesin family member 5B [Source:HGNC Symbol;Acc:HGNC:6324]	10
ENSG00000085063	6251,957	0,016353	0,11726	0,43724	1,56638	0,030736	0,102594	protein_coding	CD59	CD59 molecule (CD59 blood group) [Source:HGNC Symbol;Acc:HGNC:1689]	11
ENSG00000151239	2025,267	0,022713	0,116513	0,43724	1,569578	0,032092	0,070875	protein_coding	TWF1	twinfilin actin binding protein 1 [Source:HGNC Symbol;Acc:HGNC:9620]	12
ENSG00000139990	1560,971	-0,02383	0,117181	0,43724	-1,56672	0,029852	-0,05941	protein_coding	DCAF5	DDB1 and CUL4 associated factor 5 [Source:HGNC Symbol;Acc:HGNC:20224]	14
ENSG00000123144	2096,228	0,025463	0,11721	0,43724	1,566592	0,033288	0,066798	protein_coding	TRIR	telomerase RNA component interacting RNase [Source:HGNC Symbol;Acc:HGNC:28424]	19
ENSG00000185222	1893,267	0,019122	0,118709	0,441275	1,560215	0,031484	0,084524	protein_coding	TCEAL9	transcription elongation factor A like 9 [Source:HGNC Symbol;Acc:HGNC:30084]	X
ENSG00000108312	2483,314	0,021892	0,119332	0,442877	1,557582	0,029028	0,060376	protein_coding	UBTF	upstream binding transcription factor [Source:HGNC Symbol;Acc:HGNC:12511]	17
ENSG00000087074	3160,957	0,014539	0,119603	0,443168	1,55644	0,029508	0,105274	protein_coding	PPP1R15A	protein phosphatase 1 regulatory subunit 15A [Source:HGNC Symbol;Acc:HGNC:14375]	19
ENSG00000178913	1567,921	0,0203	0,120019	0,443912	1,554694	0,027729	0,058933	protein_coding	TAF7	TATA-box binding protein associated factor 7 [Source:HGNC Symbol;Acc:HGNC:11541]	5
ENSG00000105810	2481,799	0,018286	0,120377	0,443912	1,553195	0,03128	0,08861	protein_coding	CDK6	cyclin dependent kinase 6 [Source:HGNC Symbol;Acc:HGNC:1777]	7

ENSG00000150687	4972,842	0,013268	0,120769	0,443912	1,551555	0,029362	0,123944	protein_coding	PRSS23	serine protease 23 [Source:HGNC Symbol;Acc:HGNC:14370]	11
ENSG00000111229	2520,301	0,020035	0,120475	0,443912	1,552781	0,03104	0,075825	protein_coding	ARPC3	actin related protein 2/3 complex subunit 3 [Source:HGNC Symbol;Acc:HGNC:706]	12
ENSG00000183255	3832,105	0,022645	0,120732	0,443912	1,55171	0,027993	0,05512	protein_coding	PTTG1IP	PTTG1 interacting protein [Source:HGNC Symbol;Acc:HGNC:13524]	21
ENSG00000144028	6552,072	0,024047	0,122875	0,445347	1,542816	0,021595	0,037135	protein_coding	SNRNP200	small nuclear ribonucleoprotein U5 subunit 200 [Source:HGNC Symbol;Acc:HGNC:30859]	2
ENSG00000128641	3304,441	0,016949	0,122077	0,445347	1,546113	0,030964	0,097376	protein_coding	MYO1B	myosin IB [Source:HGNC Symbol;Acc:HGNC:7596]	2
ENSG00000154122	3800,166	-0,02232	0,122605	0,445347	-1,54393	0,030677	-0,06505	protein_coding	ANKH	ANKH inorganic pyrophosphate transport regulator [Source:HGNC Symbol;Acc:HGNC:15492]	5
ENSG00000113161	2239,541	-0,02258	0,124405	0,445347	-1,53655	0,03053	-0,06348	protein_coding	HMGCR	3-hydroxy-3-methylglutaryl-CoA reductase [Source:HGNC Symbol;Acc:HGNC:5006]	5
ENSG00000113621	1567,128	0,019999	0,124819	0,445347	1,534856	0,031067	0,07529	protein_coding	TXNDC15	thioredoxin domain containing 15 [Source:HGNC Symbol;Acc:HGNC:20652]	5
ENSG00000113657	2782,446	0,011521	0,121983	0,445347	1,546505	0,028621	0,141194	protein_coding	DPYSL3	dihydropyrimidinase like 3 [Source:HGNC Symbol;Acc:HGNC:3015]	5
ENSG00000106682	3477,122	0,022388	0,121667	0,445347	1,547813	0,025436	0,0478	protein_coding	EIF4H	eukaryotic translation initiation factor 4H [Source:HGNC Symbol;Acc:HGNC:12741]	7
ENSG00000055609	4204,777	-0,02185	0,124741	0,445347	-1,53517	0,030724	-0,06628	protein_coding	KMT2C	lysine methyltransferase 2C [Source:HGNC Symbol;Acc:HGNC:13726]	7
ENSG00000107331	2208,923	-0,0249	0,123414	0,445347	-1,5406	0,038847	-0,09117	protein_coding	ABCA2	ATP binding cassette subfamily A member 2 [Source:HGNC Symbol;Acc:HGNC:32]	9
ENSG00000155254	1564,373	0,020394	0,123163	0,445347	1,541632	0,031777	0,077915	protein_coding	MARVELD1	MARVEL domain containing 1 [Source:HGNC Symbol;Acc:HGNC:28674]	10
ENSG00000175582	2976,081	0,024382	0,123436	0,445347	1,540511	0,028629	0,053848	protein_coding	RAB6A	RAB6A, member RAS oncogene family [Source:HGNC Symbol;Acc:HGNC:9786]	11
ENSG00000151491	3869,578	0,021985	0,124836	0,445347	1,534786	0,032086	0,071951	protein_coding	EPS8	epidermal growth factor receptor pathway substrate 8 [Source:HGNC Symbol;Acc:HGNC:3420]	12
ENSG00000080824	28884,38	0,021464	0,122123	0,445347	1,545923	0,03125	0,070498	protein_coding	HSP90AA1	heat shock protein 90 alpha family class A member 1 [Source:HGNC Symbol;Acc:HGNC:5253]	14
ENSG00000067225	30421,83	0,025961	0,122943	0,445347	1,542537	0,025105	0,043555	protein_coding	PKM	pyruvate kinase M1/2 [Source:HGNC Symbol;Acc:HGNC:9021]	15
ENSG00000133026	1827,405	-0,01505	0,123911	0,445347	-1,53856	0,029942	-0,10517	protein_coding	MYH10	myosin heavy chain 10 [Source:HGNC Symbol;Acc:HGNC:7568]	17
ENSG00000175061	2315,27	0,020239	0,124744	0,445347	1,535161	0,029188	0,064515	lncRNA	SNHG29	small nucleolar RNA host gene 29 [Source:HGNC Symbol;Acc:HGNC:28619]	17
ENSG00000182473	2991,428	-0,01558	0,123905	0,445347	-1,53859	0,023294	-0,04929	protein_coding	EXOC7	exocyst complex component 7 [Source:HGNC Symbol;Acc:HGNC:23214]	17
ENSG00000264462	12394,4	-0,01813	0,124364	0,445347	-1,53671	0,033721	-0,12295	miRNA	MIR3648-2	microRNA 3648-2 [Source:HGNC Symbol;Acc:HGNC:50843]	21
ENSG00000100422	2332,055	0,01794	0,123359	0,445347	1,540828	0,031061	0,088624	protein_coding	CERK	ceramide kinase [Source:HGNC Symbol;Acc:HGNC:19256]	22
ENSG00000137710	2854,681	0,018957	0,125289	0,446269	1,532947	0,030568	0,077275	protein_coding	RDX	radixin [Source:HGNC Symbol;Acc:HGNC:9944]	11
ENSG00000114270	9219,914	0,012072	0,125617	0,446744	1,531619	0,028816	0,132613	protein_coding	COL7A1	collagen type VII alpha 1 chain [Source:HGNC Symbol;Acc:HGNC:2214]	3
ENSG00000146676	1632,033	0,020776	0,126225	0,448216	1,529159	0,031891	0,076036	protein_coding	PURB	purine rich element binding protein B [Source:HGNC Symbol;Acc:HGNC:9702]	7
ENSG00000101665	1794,846	0,022561	0,127023	0,450354	1,525947	0,030935	0,064626	protein_coding	SMAD7	SMAD family member 7 [Source:HGNC Symbol;Acc:HGNC:6773]	18
ENSG00000141429	3248,783	0,020667	0,12769	0,452023	1,523274	0,031452	0,073742	protein_coding	GALNT1	polypeptide N-acetylgalactosaminyltransferase 1 [Source:HGNC Symbol;Acc:HGNC:4123]	18
ENSG00000105974	2530,286	0,013319	0,12791	0,452104	1,522397	0,029228	0,116811	protein_coding	CAV1	caveolin 1 [Source:HGNC Symbol;Acc:HGNC:1527]	7
ENSG00000152104	2137,043	0,017821	0,128807	0,454578	1,518824	0,031109	0,088953	protein_coding	PTPN14	protein tyrosine phosphatase non-receptor type 14 [Source:HGNC Symbol;Acc:HGNC:9647]	1
ENSG00000107223	2136,946	0,019039	0,130001	0,458091	1,514097	0,031004	0,078973	protein_coding	EDF1	endothelial differentiation related factor 1 [Source:HGNC Symbol;Acc:HGNC:3164]	9
ENSG00000165757	1919,477	0,013757	0,130514	0,459195	1,512076	0,02935	0,110981	protein_coding	JCAD	junctional cadherin 5 associated [Source:HGNC Symbol;Acc:HGNC:29283]	10
ENSG00000159592	1641,347	-0,02321	0,130747	0,45931	-1,51116	0,027751	-0,05208	protein_coding	GPBP1L1	GC-rich promoter binding protein 1 like 1 [Source:HGNC Symbol;Acc:HGNC:28843]	1
ENSG00000135047	5002,68	0,016805	0,131194	0,460179	1,50941	0,030459	0,090315	protein_coding	CTSL	cathepsin L [Source:HGNC Symbol;Acc:HGNC:2537]	9
ENSG00000153560	1748,363	0,022901	0,131754	0,461439	1,507222	0,028389	0,054345	protein_coding	UBP1	upstream binding protein 1 [Source:HGNC Symbol;Acc:HGNC:12507]	3
ENSG00000175216	3909,377	0,018404	0,132123	0,462028	1,505783	0,027283	0,059821	protein_coding	CKAP5	cytoskeleton associated protein 5 [Source:HGNC Symbol;Acc:HGNC:28959]	11
ENSG00000111786	1876,304	0,022711	0,132601	0,462296	1,503923	0,029132	0,056921	protein_coding	SRSF9	serine and arginine rich splicing factor 9 [Source:HGNC Symbol;Acc:HGNC:10791]	12
ENSG00000100714	1776,01	0,02106	0,132581	0,462296	1,504	0,029528	0,062288	protein_coding	MTHFD1	methylenetetrahydrofolate dehydrogenase, cyclohydrolase and formyltetrahydrofolate synthetase 1 [Source:HGNC Syr	14
ENSG00000179051	1923,459	0,030213	0,132938	0,462767	1,502618	0,029088	0,04733	protein_coding	RCC2	regulator of chromosome condensation 2 [Source:HGNC Symbol;Acc:HGNC:30297]	1
ENSG00000134308	4340,234	0,021878	0,133296	0,462842	1,501229	0,030779	0,064994	protein_coding	YWHAQ	tyrosine 3-monooxygenase/tryptophan 5-monooxygenase activation protein theta [Source:HGNC Symbol;Acc:HGNC:12	2
ENSG00000148773	5108,008	0,012739	0,133562	0,462842	1,500201	0,028986	0,119824	protein_coding	MKI67	marker of proliferation Ki-67 [Source:HGNC Symbol;Acc:HGNC:7107]	10
ENSG00000127314	2191,237	0,021501	0,133426	0,462842	1,500729	0,0298	0,062068	protein_coding	RAP1B	RAP1B, member of RAS oncogene family [Source:HGNC Symbol;Acc:HGNC:9857]	12
ENSG00000179222	4971,95	0,022331	0,133768	0,462858	1,499407	0,023976	0,04257	protein_coding	MAGED1	MAGE family member D1 [Source:HGNC Symbol;Acc:HGNC:6813]	X
ENSG00000084733	2602,294	0,023131	0,134767	0,464914	1,495567	0,029019	0,055443	protein_coding	RAB10	RAB10, member RAS oncogene family [Source:HGNC Symbol;Acc:HGNC:9759]	2
ENSG00000149136	3362,318	0,023142	0,134599	0,464914	1,49621	0,029469	0,056876	protein_coding	SSRP1	structure specific recognition protein 1 [Source:HGNC Symbol;Acc:HGNC:11327]	11
ENSG00000115355	2159,359	0,021665	0,135102	0,465121	1,494281	0,029371	0,059731	protein_coding	CCDC88A	coiled-coil domain containing 88A [Source:HGNC Symbol;Acc:HGNC:25523]	2
ENSG00000128585	1742,406	-0,01813	0,135231	0,465121	-1,49379	0,029852	-0,07488	protein_coding	MKLN1	muskelin 1 [Source:HGNC Symbol;Acc:HGNC:7109]	7
ENSG00000134884	2106,118	-0,02141	0,136215	0,467808	-1,49003	0,02907	-0,05901	protein_coding	ARGLU1	arginine and glutamate rich 1 [Source:HGNC Symbol;Acc:HGNC:25482]	13
ENSG00000153147	3111,691	0,022927	0,136649	0,468597	1,488387	0,025391	0,04527	protein_coding	SMARCA5	SWI/SNF related, matrix associated, actin dependent regulator of chromatin, subfamily a, member 5 [Source:HGNC Syn	4
ENSG00000116016	3905,759	-0,01376	0,137834	0,469167	-1,4839	0,029294	-0,1076	protein_coding	EPAS1	endothelial PAS domain protein 1 [Source:HGNC Symbol;Acc:HGNC:3374]	2
ENSG00000135048	3712,257	0,02111	0,137233	0,469167	1,486175	0,028874	0,05881	protein_coding	CEMIP2	cell migration inducing hyaluronidase 2 [Source:HGNC Symbol;Acc:HGNC:11869]	9
ENSG00000130255	2604,651	0,016909	0,137809	0,469167	1,484001	0,030148	0,084739	protein_coding	RPL36	ribosomal protein L36 [Source:HGNC Symbol;Acc:HGNC:13631]	19
ENSG00000105640	3901,999	0,019061	0,137793	0,469167	1,48406	0,030439	0,073463	protein_coding	RPL18A	ribosomal protein L18a [Source:HGNC Symbol;Acc:HGNC:10311]	19

ENSG00000205542	6015,985	0,020085	0,137401	0,469167	1,485538	0,032288	0,080162	protein_coding	TMSB4X	thymosin beta 4 X-linked [Source:HGNC Symbol;Acc:HGNC:11881]	X
ENSG00000099995	2449,088	0,022591	0,138129	0,469475	1,482795	0,027919	0,052531	protein_coding	SF3A1	splicing factor 3a subunit 1 [Source:HGNC Symbol;Acc:HGNC:10765]	22
ENSG00000175166	5575,695	0,020913	0,138422	0,469621	1,481692	0,031745	0,072286	protein_coding	PSMD2	proteasome 26S subunit, non-ATPase 2 [Source:HGNC Symbol;Acc:HGNC:9559]	3
ENSG00000165417	1681,333	0,021205	0,138784	0,469621	1,480334	0,030009	0,062899	protein_coding	GTF2A1	general transcription factor IIA subunit 1 [Source:HGNC Symbol;Acc:HGNC:4646]	14
ENSG00000137845	2935,186	0,020749	0,13872	0,469621	1,480576	0,02909	0,060394	protein_coding	ADAM10	ADAM metallopeptidase domain 10 [Source:HGNC Symbol;Acc:HGNC:188]	15
ENSG00000119242	1616,879	0,020414	0,13914	0,470135	1,479	0,031079	0,070722	protein_coding	CCDC92	coiled-coil domain containing 92 [Source:HGNC Symbol;Acc:HGNC:29563]	12
ENSG00000116044	2610,936	0,021916	0,140139	0,471263	1,475274	0,028628	0,055729	protein_coding	NFE2L2	nuclear factor, erythroid 2 like 2 [Source:HGNC Symbol;Acc:HGNC:7782]	2
ENSG00000130147	2763,938	0,022151	0,141734	0,471263	1,469364	0,0274	0,051198	protein_coding	SH3BP4	SH3 domain binding protein 4 [Source:HGNC Symbol;Acc:HGNC:10826]	2
ENSG00000122068	1612,882	0,018305	0,141057	0,471263	1,471868	0,030039	0,074122	protein_coding	FYTTD1	forty-two-three domain containing 1 [Source:HGNC Symbol;Acc:HGNC:25407]	3
ENSG00000111885	4142,412	-0,01689	0,1422	0,471263	-1,46765	0,030112	-0,08356	protein_coding	MAN1A1	mannosidase alpha class 1A member 1 [Source:HGNC Symbol;Acc:HGNC:6821]	6
ENSG00000172354	1686,941	-0,01167	0,141095	0,471263	-1,47172	0,024742	-0,06325	protein_coding	GNB2	G protein subunit beta 2 [Source:HGNC Symbol;Acc:HGNC:4398]	7
ENSG00000197969	1986,105	-0,0163	0,142193	0,471263	-1,46768	0,029922	-0,08643	protein_coding	VPS13A	vacuolar protein sorting 13 homolog A [Source:HGNC Symbol;Acc:HGNC:1908]	9
ENSG00000023191	1712,685	0,021602	0,141515	0,471263	1,470173	0,028925	0,057277	protein_coding	RNH1	ribonuclease/angiogenin inhibitor 1 [Source:HGNC Symbol;Acc:HGNC:10074]	11
ENSG00000179195	3212,977	-0,02197	0,140259	0,471263	-1,47483	0,025763	-0,04693	protein_coding	ZNF664	zinc finger protein 664 [Source:HGNC Symbol;Acc:HGNC:25406]	12
ENSG00000054654	2069,417	-0,01321	0,142301	0,471263	-1,46728	0,029039	-0,10965	protein_coding	SYNE2	spectrin repeat containing nuclear envelope protein 2 [Source:HGNC Symbol;Acc:HGNC:17084]	14
ENSG00000140319	4278,137	0,02352	0,140503	0,471263	1,47392	0,030345	0,058111	protein_coding	SRP14	signal recognition particle 14 [Source:HGNC Symbol;Acc:HGNC:11299]	15
ENSG00000180357	2010,428	-0,02255	0,14247	0,471263	-1,46665	0,032249	-0,06737	protein_coding	ZNF609	zinc finger protein 609 [Source:HGNC Symbol;Acc:HGNC:29003]	15
ENSG00000125148	7124,597	0,016329	0,140521	0,471263	1,473854	0,029921	0,08645	protein_coding	MT2A	metallothionein 2A [Source:HGNC Symbol;Acc:HGNC:7406]	16
ENSG00000183530	1772,49	0,021483	0,141157	0,471263	1,471497	0,027278	0,051967	protein_coding	PRR14L	proline rich 14 like [Source:HGNC Symbol;Acc:HGNC:28738]	22
ENSG00000130827	2085,239	-0,02021	0,142546	0,471263	-1,46637	0,032895	-0,08259	protein_coding	PLXNA3	plexin A3 [Source:HGNC Symbol;Acc:HGNC:9101]	X
ENSG00000198888	109310,7	-0,06761	0,141526	0,471263	-1,47013	0,216354	-0,10226	protein_coding	MT-ND1	mitochondrially encoded NADH:ubiquinone oxidoreductase core subunit 1 [Source:HGNC Symbol;Acc:HGNC:7455]	MT
ENSG00000119655	1730,954	0,010233	0,143294	0,473054	1,463635	0,026238	0,08712	protein_coding	NPC2	NPC intracellular cholesterol transporter 2 [Source:HGNC Symbol;Acc:HGNC:14537]	14
ENSG00000110092	18025,54	0,015629	0,143712	0,473077	1,462107	0,029791	0,090777	protein_coding	CCND1	cyclin D1 [Source:HGNC Symbol;Acc:HGNC:1582]	11
ENSG00000134910	3633,853	0,023398	0,143688	0,473077	1,462194	0,032155	0,06417	protein_coding	STT3A	STT3 oligosaccharyltransferase complex catalytic subunit A [Source:HGNC Symbol;Acc:HGNC:6172]	11
ENSG00000117724	3016,436	0,009635	0,144483	0,47426	1,459297	0,02778	0,148965	protein_coding	CENPF	centromere protein F [Source:HGNC Symbol;Acc:HGNC:1857]	1
ENSG00000146731	4306,307	0,021576	0,144438	0,47426	1,459462	0,027785	0,053031	protein_coding	CCT6A	chaperonin containing TCP1 subunit 6A [Source:HGNC Symbol;Acc:HGNC:1620]	7
ENSG00000145907	3507,306	0,021918	0,145492	0,474281	1,45564	0,025503	0,045639	protein_coding	G3BP1	G3BP stress granule assembly factor 1 [Source:HGNC Symbol;Acc:HGNC:30292]	5
ENSG00000126524	1941,981	0,021301	0,14552	0,474281	1,455538	0,028908	0,05732	protein_coding	SBDS	SBDS ribosome maturation factor [Source:HGNC Symbol;Acc:HGNC:19440]	7
ENSG00000136810	1888,933	0,02046	0,14504	0,474281	1,457277	0,030569	0,066924	protein_coding	TXN	thioredoxin [Source:HGNC Symbol;Acc:HGNC:12435]	9
ENSG00000149930	1830,51	-0,02117	0,145292	0,474281	-1,45636	0,028619	-0,05657	protein_coding	TAOK2	TAO kinase 2 [Source:HGNC Symbol;Acc:HGNC:16835]	16
ENSG00000205336	1735,453	-0,01721	0,144835	0,474281	-1,45802	0,030775	-0,08712	protein_coding	ADGRG1	adhesion G protein-coupled receptor G1 [Source:HGNC Symbol;Acc:HGNC:4512]	16
ENSG00000025800	2105,863	-0,02141	0,149426	0,476995	-1,44156	0,027713	-0,05242	protein_coding	KPNA6	karyopherin subunit alpha 6 [Source:HGNC Symbol;Acc:HGNC:6399]	1
ENSG00000177954	3447,874	0,01868	0,148781	0,476995	1,443852	0,029951	0,070314	protein_coding	RPS27	ribosomal protein S27 [Source:HGNC Symbol;Acc:HGNC:10416]	1
ENSG00000136536	2076,983	0,021348	0,148781	0,476995	1,443849	0,027048	0,050512	protein_coding	MARCHF7	membrane associated ring-CH-type finger 7 [Source:HGNC Symbol;Acc:HGNC:17393]	2
ENSG00000118523	9434,145	0,011352	0,149115	0,476995	1,442663	0,028321	0,123533	protein_coding	CCN2	cellular communication network factor 2 [Source:HGNC Symbol;Acc:HGNC:2500]	6
ENSG00000131018	3857,004	-0,0146	0,148528	0,476995	-1,44475	0,029379	-0,09486	protein_coding	SYNE1	spectrin repeat containing nuclear envelope protein 1 [Source:HGNC Symbol;Acc:HGNC:17089]	6
ENSG00000129103	1935,744	0,021204	0,147607	0,476995	1,448037	0,028566	0,055969	protein_coding	SUMF2	sulfatase modifying factor 2 [Source:HGNC Symbol;Acc:HGNC:20415]	7
ENSG00000107164	1570,517	0,020259	0,147283	0,476995	1,449194	0,027654	0,054719	protein_coding	FUBP3	far upstream element binding protein 3 [Source:HGNC Symbol;Acc:HGNC:4005]	9
ENSG00000107957	8330,085	0,018433	0,149434	0,476995	1,441532	0,029823	0,070507	protein_coding	SH3PXD2A	SH3 and PX domains 2A [Source:HGNC Symbol;Acc:HGNC:23664]	10
ENSG00000167996	54148,45	-0,01585	0,147962	0,476995	-1,44677	0,0297	-0,08643	protein_coding	FTH1	ferritin heavy chain 1 [Source:HGNC Symbol;Acc:HGNC:3976]	11
ENSG00000149591	3627,207	0,004945	0,148635	0,476995	1,44437	0,026527	0,268182	protein_coding	TAGLN	transgelin [Source:HGNC Symbol;Acc:HGNC:11553]	11
ENSG00000069956	2290,751	0,021357	0,148302	0,476995	1,445556	0,029671	0,059663	protein_coding	MAPK6	mitogen-activated protein kinase 6 [Source:HGNC Symbol;Acc:HGNC:6879]	15
ENSG00000131747	2931,695	0,006783	0,148325	0,476995	1,445474	0,026949	0,208114	protein_coding	TOP2A	DNA topoisomerase II alpha [Source:HGNC Symbol;Acc:HGNC:11989]	17
ENSG00000167601	3898,183	0,011189	0,149463	0,476995	1,441432	0,02826	0,124925	protein_coding	AXL	AXL receptor tyrosine kinase [Source:HGNC Symbol;Acc:HGNC:905]	19
ENSG00000063177	3501,794	0,022792	0,14821	0,476995	1,445884	0,032721	0,067501	protein_coding	RPL18	ribosomal protein L18 [Source:HGNC Symbol;Acc:HGNC:10310]	19
ENSG00000142173	27583,58	0,011614	0,149419	0,476995	1,441586	0,028409	0,12062	protein_coding	COL6A2	collagen type VI alpha 2 chain [Source:HGNC Symbol;Acc:HGNC:2212]	21
ENSG00000128567	1917,168	0,009969	0,150281	0,47889	1,438541	0,027855	0,14001	protein_coding	PODXL	podocalyxin like [Source:HGNC Symbol;Acc:HGNC:9171]	7
ENSG00000164761	3854,944	-0,00768	0,150473	0,47889	-1,43786	0,027176	-0,18218	protein_coding	TNFRSF11E	TNF receptor superfamily member 11b [Source:HGNC Symbol;Acc:HGNC:11909]	8
ENSG00000151693	2258,877	0,016951	0,150953	0,479454	1,436175	0,029157	0,073151	protein_coding	ASAP2	ArfGAP with SH3 domain, ankyrin repeat and PH domain 2 [Source:HGNC Symbol;Acc:HGNC:2721]	2
ENSG00000141551	1797,579	-0,02105	0,151067	0,479454	-1,43577	0,027879	-0,05341	protein_coding	CSNK1D	casein kinase 1 delta [Source:HGNC Symbol;Acc:HGNC:2452]	17
ENSG00000084754	2995,775	0,022742	0,151527	0,479709	1,43416	0,026395	0,046376	protein_coding	HADHA	hydroxyacyl-CoA dehydrogenase trifunctional multienzyme complex subunit alpha [Source:HGNC Symbol;Acc:HGNC:48	2
ENSG00000174780	2269,063	0,021039	0,151564	0,479709	1,434029	0,028043	0,05396	protein_coding	SRP72	signal recognition particle 72 [Source:HGNC Symbol;Acc:HGNC:11303]	4

ENSG00000197321	4254,73	0,015709	0,153651	0,485464	1,426755	0,029358	0,082648	protein_coding	SVIL	supervillin [Source:HGNC Symbol;Acc:HGNC:11480]	10
ENSG00000166557	2191,598	0,021218	0,154153	0,485464	1,425014	0,030958	0,064534	protein_coding	TMED3	transmembrane p24 trafficking protein 3 [Source:HGNC Symbol;Acc:HGNC:28889]	15
ENSG00000196557	1971,565	-0,01578	0,153828	0,485464	-1,42614	0,029521	-0,08379	protein_coding	CACNA1H	calcium voltage-gated channel subunit alpha1 H [Source:HGNC Symbol;Acc:HGNC:1395]	16
ENSG00000103549	2068,547	0,020043	0,154226	0,485464	1,424762	0,027424	0,053413	protein_coding	RNF40	ring finger protein 40 [Source:HGNC Symbol;Acc:HGNC:16867]	16
ENSG00000102572	3608,484	0,019728	0,154999	0,48723	1,422095	0,029187	0,061233	protein_coding	STK24	serine/threonine kinase 24 [Source:HGNC Symbol;Acc:HGNC:11403]	13
ENSG00000196535	2001,414	-0,01572	0,15578	0,489018	-1,41941	0,029725	-0,08629	protein_coding	MYO18A	myosin XVIIIA [Source:HGNC Symbol;Acc:HGNC:31104]	17
ENSG00000075415	3369,404	0,021048	0,156039	0,489163	1,41852	0,027572	0,051816	protein_coding	SLC25A3	solute carrier family 25 member 3 [Source:HGNC Symbol;Acc:HGNC:10989]	12
ENSG00000068912	2396,763	0,020299	0,157747	0,490785	1,41269	0,028065	0,054768	protein_coding	ERLEC1	endoplasmic reticulum lectin 1 [Source:HGNC Symbol;Acc:HGNC:25222]	2
ENSG00000091490	3585,686	-0,02046	0,156851	0,490785	-1,41574	0,027674	-0,05313	protein_coding	SEL1L3	SEL1L family member 3 [Source:HGNC Symbol;Acc:HGNC:29108]	4
ENSG00000113360	1791,551	0,01794	0,158049	0,490785	1,411663	0,026301	0,052944	protein_coding	DROSHA	drosha ribonuclease III [Source:HGNC Symbol;Acc:HGNC:17904]	5
ENSG00000089220	2023,321	0,021054	0,157621	0,490785	1,413118	0,028995	0,056486	protein_coding	PEBP1	phosphatidylethanolamine binding protein 1 [Source:HGNC Symbol;Acc:HGNC:8630]	12
ENSG00000092148	5704,717	-0,02014	0,158035	0,490785	-1,41171	0,019966	-0,03243	protein_coding	HECTD1	HECT domain E3 ubiquitin protein ligase 1 [Source:HGNC Symbol;Acc:HGNC:20157]	14
ENSG00000105698	1883,539	0,02089	0,157932	0,490785	1,412061	0,027191	0,050561	protein_coding	USF2	upstream transcription factor 2, c-fos interacting [Source:HGNC Symbol;Acc:HGNC:12594]	19
ENSG00000126461	1847,697	-0,02087	0,157457	0,490785	-1,41368	0,026446	-0,04831	protein_coding	SCAF1	SR-related CTD associated factor 1 [Source:HGNC Symbol;Acc:HGNC:30403]	19
ENSG00000120708	22102,55	0,005966	0,158409	0,491239	1,410442	0,026741	0,225192	protein_coding	TGFBI	transforming growth factor beta induced [Source:HGNC Symbol;Acc:HGNC:11771]	5
ENSG00000132824	4281,217	0,020362	0,158942	0,492229	1,408637	0,028191	0,054957	protein_coding	SERINC3	serine incorporator 3 [Source:HGNC Symbol;Acc:HGNC:11699]	20
ENSG00000106565	1621,936	0,009431	0,159582	0,492882	1,40648	0,027628	0,140575	protein_coding	TMEM176I	transmembrane protein 176B [Source:HGNC Symbol;Acc:HGNC:29596]	7
ENSG00000167106	1570,416	-0,01017	0,159373	0,492882	-1,40718	0,026807	-0,09504	protein_coding	FAM102A	family with sequence similarity 102 member A [Source:HGNC Symbol;Acc:HGNC:31419]	9
ENSG00000104321	1972,061	-0,00503	0,16041	0,494114	-1,40369	0,026548	-0,26142	protein_coding	TRPA1	transient receptor potential cation channel subfamily A member 1 [Source:HGNC Symbol;Acc:HGNC:497]	8
ENSG00000148700	2737,074	0,019597	0,160388	0,494114	1,403768	0,026333	0,049619	protein_coding	ADD3	adducin 3 [Source:HGNC Symbol;Acc:HGNC:245]	10
ENSG00000150753	5569,519	0,019186	0,161181	0,495824	1,401112	0,024823	0,045269	protein_coding	CCT5	chaperonin containing TCP1 subunit 5 [Source:HGNC Symbol;Acc:HGNC:1618]	5
ENSG00000089199	7677,899	0,015063	0,161765	0,496958	1,399159	0,029069	0,083101	protein_coding	CHGB	chromogranin B [Source:HGNC Symbol;Acc:HGNC:1930]	20
ENSG00000010017	1636,382	-0,0187	0,162353	0,4981	-1,3972	0,026636	-0,05216	protein_coding	RANBP9	RAN binding protein 9 [Source:HGNC Symbol;Acc:HGNC:13727]	6
ENSG00000150961	3559,87	0,021827	0,16427	0,501985	1,390852	0,028434	0,052094	protein_coding	SEC24D	SEC24 homolog D, COPII coat complex component [Source:HGNC Symbol;Acc:HGNC:10706]	4
ENSG00000140575	12553,38	0,021846	0,164274	0,501985	1,39084	0,029513	0,05563	protein_coding	IQGAP1	IQ motif containing GTPase activating protein 1 [Source:HGNC Symbol;Acc:HGNC:6110]	15
ENSG00000109072	1660,206	0,00446	0,164063	0,501985	1,391536	0,026444	0,285604	protein_coding	VTN	vitronectin [Source:HGNC Symbol;Acc:HGNC:12724]	17
ENSG00000078618	2698,095	0,019697	0,167609	0,502653	1,379928	0,026899	0,050562	protein_coding	NRDC	nardilysin convertase [Source:HGNC Symbol;Acc:HGNC:7995]	1
ENSG00000196504	2610,221	0,019867	0,168256	0,502653	1,377828	0,028455	0,055909	protein_coding	PRPF40A	pre-mRNA processing factor 40 homolog A [Source:HGNC Symbol;Acc:HGNC:16463]	2
ENSG00000198677	2487,269	0,020938	0,167926	0,502653	1,378898	0,026168	0,046236	protein_coding	TTC37	tetratricopeptide repeat domain 37 [Source:HGNC Symbol;Acc:HGNC:23639]	5
ENSG00000122566	11863,57	0,02007	0,167685	0,502653	1,379679	0,026973	0,05012	protein_coding	HNRNPA2E	heterogeneous nuclear ribonucleoprotein A2/B1 [Source:HGNC Symbol;Acc:HGNC:5033]	7
ENSG00000186591	2241,833	-0,01974	0,166729	0,502653	-1,38279	0,023616	-0,04063	protein_coding	UBE2H	ubiquitin conjugating enzyme E2 H [Source:HGNC Symbol;Acc:HGNC:12484]	7
ENSG00000033100	1976,898	-0,01993	0,165866	0,502653	-1,38561	0,02492	-0,04413	protein_coding	CHPF2	chondroitin polymerizing factor 2 [Source:HGNC Symbol;Acc:HGNC:29270]	7
ENSG00000186480	1670,392	-0,01514	0,165125	0,502653	-1,38804	0,029113	-0,08235	protein_coding	INSIG1	insulin induced gene 1 [Source:HGNC Symbol;Acc:HGNC:6083]	7
ENSG00000148730	3114,895	0,021434	0,168211	0,502653	1,377975	0,025053	0,042554	protein_coding	EIF4EBP2	eukaryotic translation initiation factor 4E binding protein 2 [Source:HGNC Symbol;Acc:HGNC:3289]	10
ENSG00000107862	3001,535	-0,02529	0,168237	0,502653	-1,37789	0,02899	-0,04783	protein_coding	GBF1	golgi brefeldin A resistant guanine nucleotide exchange factor 1 [Source:HGNC Symbol;Acc:HGNC:4181]	10
ENSG00000175274	2434,734	-0,00896	0,168216	0,502653	-1,37796	0,02734	-0,13498	protein_coding	TP53I11	tumor protein p53 inducible protein 11 [Source:HGNC Symbol;Acc:HGNC:16842]	11
ENSG00000207445	1713,513	-0,00628	0,165258	0,502653	-1,3876	0,026789	-0,20552	snoRNA	SNORD15B	small nucleolar RNA, C/D box 15B [Source:HGNC Symbol;Acc:HGNC:16649]	11
ENSG00000084112	4100,518	-0,02243	0,167302	0,502653	-1,38093	0,02961	-0,05431	protein_coding	SSH1	slingshot protein phosphatase 1 [Source:HGNC Symbol;Acc:HGNC:30579]	12
ENSG00000090863	6161,246	0,020568	0,167495	0,502653	1,380299	0,027016	0,049413	protein_coding	GLG1	golgi glycoprotein 1 [Source:HGNC Symbol;Acc:HGNC:4316]	16
ENSG00000130402	10090,27	0,020196	0,168425	0,502653	1,377283	0,024919	0,043549	protein_coding	ACTN4	actinin alpha 4 [Source:HGNC Symbol;Acc:HGNC:166]	19
ENSG00000088325	1674,845	0,009868	0,167222	0,502653	1,381186	0,027798	0,134326	protein_coding	TPX2	TPX2 microtubule nucleation factor [Source:HGNC Symbol;Acc:HGNC:1249]	20
ENSG00000264063	4675,406	-0,01201	0,165598	0,502653	-1,38649	0,028424	-0,10821	miRNA	CR392039.1		21
ENSG00000159228	1661,967	0,019319	0,16637	0,502653	1,383961	0,02855	0,057986	protein_coding	CBR1	carbonyl reductase 1 [Source:HGNC Symbol;Acc:HGNC:1548]	21
ENSG00000099917	1611,941	0,020201	0,166215	0,502653	1,384468	0,02653	0,048603	protein_coding	MED15	mediator complex subunit 15 [Source:HGNC Symbol;Acc:HGNC:14248]	22
ENSG00000115758	2641,541	0,017076	0,169039	0,50318	1,375299	0,028881	0,067409	protein_coding	ODC1	ornithine decarboxylase 1 [Source:HGNC Symbol;Acc:HGNC:8109]	2
ENSG00000114978	2283,186	0,016627	0,168892	0,50318	1,375772	0,029141	0,071667	protein_coding	MOB1A	MOB kinase activator 1A [Source:HGNC Symbol;Acc:HGNC:16015]	2
ENSG00000114850	4778,236	0,016599	0,172543	0,503195	1,364078	0,028595	0,067235	protein_coding	SSR3	signal sequence receptor subunit 3 [Source:HGNC Symbol;Acc:HGNC:11325]	3
ENSG00000113407	4087,354	0,02218	0,170381	0,503195	1,370982	0,02707	0,046811	protein_coding	TARS1	threonyl-tRNA synthetase 1 [Source:HGNC Symbol;Acc:HGNC:11572]	5
ENSG00000122642	3524,829	0,018626	0,170885	0,503195	1,369367	0,028351	0,058455	protein_coding	FKBP9	FKBP prolyl isomerase 9 [Source:HGNC Symbol;Acc:HGNC:3725]	7
ENSG00000146648	4247,116	0,015509	0,169817	0,503195	1,372792	0,029042	0,077797	protein_coding	EGFR	epidermal growth factor receptor [Source:HGNC Symbol;Acc:HGNC:3236]	7
ENSG00000182197	3317,417	0,017676	0,172416	0,503195	1,364481	0,029165	0,066103	protein_coding	EXT1	exostosin glycosyltransferase 1 [Source:HGNC Symbol;Acc:HGNC:3512]	8
ENSG00000179950	1831,394	0,018039	0,17061	0,503195	1,370247	0,028771	0,062569	protein_coding	PUF60	poly(U) binding splicing factor 60 [Source:HGNC Symbol;Acc:HGNC:17042]	8

ENSG00000147883	2472,677	-0,01087	0,171105	0,503195	-1,36866	0,027958	-0,11416	protein_coding	CDKN2B	cyclin dependent kinase inhibitor 2B [Source:HGNC Symbol;Acc:HGNC:1788]	9
ENSG00000148396	4123,917	-0,01761	0,171266	0,503195	-1,36815	0,029277	-0,06723	protein_coding	SEC16A	SEC16 homolog A, endoplasmic reticulum export factor [Source:HGNC Symbol;Acc:HGNC:29006]	9
ENSG00000151929	1648,888	0,020099	0,170766	0,503195	1,369745	0,030749	0,065128	protein_coding	BAG3	BAG cochaperone 3 [Source:HGNC Symbol;Acc:HGNC:939]	10
ENSG00000110651	2831,239	0,019265	0,170077	0,503195	1,371955	0,028313	0,056603	protein_coding	CD81	CD81 molecule [Source:HGNC Symbol;Acc:HGNC:1701]	11
ENSG00000166025	3164,437	0,018235	0,17241	0,503195	1,3645	0,030015	0,068938	protein_coding	AMOTL1	angiomotin like 1 [Source:HGNC Symbol;Acc:HGNC:17811]	11
ENSG00000151135	1654,492	0,014386	0,17196	0,503195	1,365934	0,028437	0,079265	protein_coding	TMEM263	transmembrane protein 263 [Source:HGNC Symbol;Acc:HGNC:28281]	12
ENSG00000100934	2922,234	0,012527	0,171283	0,503195	1,368092	0,028095	0,092039	protein_coding	SEC23A	Sec23 homolog A, COPII coat complex component [Source:HGNC Symbol;Acc:HGNC:10701]	14
ENSG00000074657	2189,61	0,01895	0,172103	0,503195	1,365478	0,027993	0,05582	protein_coding	ZNF532	zinc finger protein 532 [Source:HGNC Symbol;Acc:HGNC:30940]	18
ENSG00000185236	1648,98	-0,01884	0,171808	0,503195	-1,36642	0,026511	-0,05025	protein_coding	RAB11B	RAB11B, member RAS oncogene family [Source:HGNC Symbol;Acc:HGNC:9761]	19
ENSG00000101191	2727,524	-0,02014	0,17104	0,503195	-1,36887	0,026762	-0,04891	protein_coding	DIDO1	death inducer-obliterator 1 [Source:HGNC Symbol;Acc:HGNC:2680]	20
ENSG00000173702	5985,551	0,005758	0,173089	0,50415	1,362344	0,026667	0,216086	protein_coding	MUC13	mucin 13, cell surface associated [Source:HGNC Symbol;Acc:HGNC:7511]	3
ENSG00000071537	4511,352	-0,0182	0,173529	0,504791	-1,36095	0,023926	-0,04246	protein_coding	SEL1L	SEL1L adaptor subunit of ERAD E3 ubiquitin ligase [Source:HGNC Symbol;Acc:HGNC:10717]	14
ENSG00000108557	1792,718	-0,01634	0,174168	0,50601	-1,35893	0,028611	-0,06835	protein_coding	RAI1	retinoic acid induced 1 [Source:HGNC Symbol;Acc:HGNC:9834]	17
ENSG00000133657	5549,421	0,019922	0,174986	0,507746	1,356357	0,028076	0,053461	protein_coding	ATP13A3	ATPase 13A3 [Source:HGNC Symbol;Acc:HGNC:24113]	3
ENSG00000128272	4936,966	-0,0217	0,175278	0,507952	-1,35544	0,03201	-0,06428	protein_coding	ATF4	activating transcription factor 4 [Source:HGNC Symbol;Acc:HGNC:786]	22
ENSG00000196141	4102,775	0,020563	0,175767	0,508731	1,353903	0,024947	0,042462	protein_coding	SPATS2L	spermatogenesis associated serine rich 2 like [Source:HGNC Symbol;Acc:HGNC:24574]	2
ENSG00000158417	3187,286	0,018826	0,17627	0,509448	1,35233	0,028069	0,055928	protein_coding	EIF5B	eukaryotic translation initiation factor 5B [Source:HGNC Symbol;Acc:HGNC:30793]	2
ENSG00000174695	2280,972	0,019777	0,176458	0,509448	1,351741	0,026122	0,046826	protein_coding	TMEM167/	transmembrane protein 167A [Source:HGNC Symbol;Acc:HGNC:28330]	5
ENSG00000067057	2315,31	0,014511	0,17686	0,509587	1,350487	0,028737	0,08086	protein_coding	PFKP	phosphofructokinase, platelet [Source:HGNC Symbol;Acc:HGNC:8878]	10
ENSG00000142230	1736,024	0,019422	0,176949	0,509587	1,350209	0,027541	0,052282	protein_coding	SAE1	SUMO1 activating enzyme subunit 1 [Source:HGNC Symbol;Acc:HGNC:30660]	19
ENSG00000225733	1872,393	0,016614	0,178704	0,51144	1,344756	0,028646	0,066564	lncRNA	FGD5-AS1	FGD5 antisense RNA 1 [Source:HGNC Symbol;Acc:HGNC:40410]	3
ENSG00000145817	2181,245	0,017257	0,178413	0,51144	1,345657	0,028553	0,063226	protein_coding	YIPF5	Yip1 domain family member 5 [Source:HGNC Symbol;Acc:HGNC:24877]	5
ENSG00000105887	2519,978	0,021634	0,178635	0,51144	1,34497	0,030416	0,057543	protein_coding	MTPN	myotrophin [Source:HGNC Symbol;Acc:HGNC:15667]	7
ENSG00000166333	2015,435	0,016711	0,178385	0,51144	1,345745	0,028706	0,066551	protein_coding	ILK	integrin linked kinase [Source:HGNC Symbol;Acc:HGNC:6040]	11
ENSG00000154845	1878,722	0,018084	0,17814	0,51144	1,346503	0,027309	0,054324	protein_coding	PPP4R1	protein phosphatase 4 regulatory subunit 1 [Source:HGNC Symbol;Acc:HGNC:9320]	18
ENSG00000168092	2847,919	0,018281	0,179258	0,512388	1,343044	0,027596	0,054874	protein_coding	PAFAH1B2	platelet activating factor acetylhydrolase 1b catalytic subunit 2 [Source:HGNC Symbol;Acc:HGNC:8575]	11
ENSG00000173402	3081,649	-0,0202	0,17996	0,512485	-1,34088	0,028128	-0,0524	protein_coding	DAG1	dystroglycan 1 [Source:HGNC Symbol;Acc:HGNC:2666]	3
ENSG00000161011	14566,68	-0,01452	0,17991	0,512485	-1,34103	0,028644	-0,07903	protein_coding	SQSTM1	sequestosome 1 [Source:HGNC Symbol;Acc:HGNC:11280]	5
ENSG00000136026	7971,2	0,016936	0,179566	0,512485	1,342091	0,028778	0,065811	protein_coding	CKAP4	cytoskeleton associated protein 4 [Source:HGNC Symbol;Acc:HGNC:16991]	12
ENSG00000135679	3441,647	0,014687	0,180575	0,5136	1,338988	0,028622	0,077425	protein_coding	MDM2	MDM2 proto-oncogene [Source:HGNC Symbol;Acc:HGNC:6973]	12
ENSG00000153201	7278,269	0,021478	0,180974	0,513665	1,337761	0,023772	0,03819	protein_coding	RANBP2	RAN binding protein 2 [Source:HGNC Symbol;Acc:HGNC:9848]	2
ENSG00000163513	2507,843	0,016014	0,181267	0,513665	1,336863	0,028892	0,071085	protein_coding	TGFBR2	transforming growth factor beta receptor 2 [Source:HGNC Symbol;Acc:HGNC:11773]	3
ENSG00000163625	2585,788	-0,02278	0,181239	0,513665	-1,33695	0,030639	-0,05512	protein_coding	WDFY3	WD repeat and FYVE domain containing 3 [Source:HGNC Symbol;Acc:HGNC:20751]	4
ENSG00000090273	2415,509	0,017518	0,182732	0,514362	1,33239	0,028302	0,06015	protein_coding	NUDC	nuclear distribution C, dynein complex regulator [Source:HGNC Symbol;Acc:HGNC:8045]	1
ENSG00000057019	8283,054	0,013818	0,182411	0,514362	1,333368	0,02852	0,083135	protein_coding	DCBLD2	discoidin, CUB and LCCL domain containing 2 [Source:HGNC Symbol;Acc:HGNC:24627]	3
ENSG00000145391	2743,799	0,018652	0,18276	0,514362	1,332304	0,027252	0,052159	protein_coding	SETD7	SET domain containing 7, histone lysine methyltransferase [Source:HGNC Symbol;Acc:HGNC:30412]	4
ENSG00000006468	1645,41	0,017727	0,182855	0,514362	1,332017	0,028054	0,05816	protein_coding	ETV1	ETS variant transcription factor 1 [Source:HGNC Symbol;Acc:HGNC:3490]	7
ENSG00000119487	1597,84	0,019108	0,182536	0,514362	1,332986	0,026927	0,050034	protein_coding	MAPKAP1	MAPK associated protein 1 [Source:HGNC Symbol;Acc:HGNC:18752]	9
ENSG00000087303	3073,16	0,009033	0,182681	0,514362	1,332544	0,027396	0,131617	protein_coding	NID2	nidogen 2 [Source:HGNC Symbol;Acc:HGNC:13389]	14
ENSG00000116698	2582,174	-0,01959	0,184776	0,518201	-1,32619	0,025639	-0,04476	protein_coding	SMG7	SMG7 nonsense mediated mRNA decay factor [Source:HGNC Symbol;Acc:HGNC:16792]	1
ENSG00000115524	6234,5	0,019441	0,18557	0,518201	1,323797	0,021587	0,034242	protein_coding	SF3B1	splicing factor 3b subunit 1 [Source:HGNC Symbol;Acc:HGNC:10768]	2
ENSG00000104765	1571,902	0,01501	0,185458	0,518201	1,324136	0,02855	0,073581	protein_coding	BNIP3L	BCL2 interacting protein 3 like [Source:HGNC Symbol;Acc:HGNC:1085]	8
ENSG00000102580	3102,457	-0,0176	0,185345	0,518201	-1,32448	0,024577	-0,04413	protein_coding	DNAJC3	DnaJ heat shock protein family (Hsp40) member C3 [Source:HGNC Symbol;Acc:HGNC:9439]	13
ENSG00000129003	4666,002	-0,02002	0,184446	0,518201	-1,32719	0,026688	-0,04738	protein_coding	VPS13C	vacuolar protein sorting 13 homolog C [Source:HGNC Symbol;Acc:HGNC:23594]	15
ENSG00000169895	1762,673	0,017286	0,185051	0,518201	1,325361	0,02811	0,059629	protein_coding	SYAP1	synapse associated protein 1 [Source:HGNC Symbol;Acc:HGNC:16273]	X
ENSG00000165527	2457,185	0,017213	0,186094	0,519033	1,322223	0,027348	0,055843	protein_coding	ARF6	ADP ribosylation factor 6 [Source:HGNC Symbol;Acc:HGNC:659]	14
ENSG00000167772	1773,542	0,010902	0,186409	0,519283	1,321276	0,027839	0,105871	protein_coding	ANGPTL4	angiopoietin like 4 [Source:HGNC Symbol;Acc:HGNC:16039]	19
ENSG00000137309	4650,452	0,015415	0,187126	0,519393	1,319129	0,028422	0,069663	protein_coding	HMGA1	high mobility group AT-hook 1 [Source:HGNC Symbol;Acc:HGNC:5010]	6
ENSG00000275023	1828,538	-0,01803	0,186913	0,519393	-1,31977	0,02784	-0,05568	protein_coding	MLLT6	MLLT6, PHD finger containing [Source:HGNC Symbol;Acc:HGNC:7138]	17
ENSG00000197256	4947,457	0,018593	0,186787	0,519393	1,320145	0,027058	0,051063	protein_coding	KANK2	KN motif and ankyrin repeat domains 2 [Source:HGNC Symbol;Acc:HGNC:29300]	19
ENSG00000069188	1709,843	0,01276	0,187887	0,520877	1,316856	0,028191	0,087599	protein_coding	SDK2	sidekick cell adhesion molecule 2 [Source:HGNC Symbol;Acc:HGNC:19308]	17
ENSG00000143702	3279,192	0,012406	0,190202	0,526026	1,309983	0,027574	0,080812	protein_coding	CEP170	centrosomal protein 170 [Source:HGNC Symbol;Acc:HGNC:28920]	1

ENSG00000196305	4988,657	0,019453	0,190042	0,526026	1,310453	0,024263	0,040472	protein_coding	IARS1	isoleucyl-tRNA synthetase 1 [Source:HGNC Symbol;Acc:HGNC:5330]	9
ENSG00000115977	2971,911	0,018659	0,191359	0,527958	1,306569	0,023273	0,038507	protein_coding	AAK1	AP2 associated kinase 1 [Source:HGNC Symbol;Acc:HGNC:19679]	2
ENSG00000136802	2795,856	0,019312	0,191163	0,527958	1,307147	0,027743	0,051629	protein_coding	LRRC8A	leucine rich repeat containing 8 VRAC subunit A [Source:HGNC Symbol;Acc:HGNC:19027]	9
ENSG00000122591	2036,467	0,018139	0,191638	0,528095	1,305749	0,026998	0,051265	protein_coding	FAM126A	family with sequence similarity 126 member A [Source:HGNC Symbol;Acc:HGNC:24587]	7
ENSG00000184432	5099,952	0,019725	0,193215	0,530535	1,301126	0,024713	0,041104	protein_coding	COPB2	COPI coat complex subunit beta 2 [Source:HGNC Symbol;Acc:HGNC:2232]	3
ENSG00000164919	1812,677	0,016894	0,193151	0,530535	1,301314	0,02775	0,057951	protein_coding	COX6C	cytochrome c oxidase subunit 6C [Source:HGNC Symbol;Acc:HGNC:2285]	8
ENSG00000167978	12268,25	-0,01871	0,19279	0,530535	-1,30237	0,027768	-0,0529	protein_coding	SRRM2	serine/arginine repetitive matrix 2 [Source:HGNC Symbol;Acc:HGNC:16639]	16
ENSG00000159352	2507,344	0,019533	0,194113	0,532366	1,298507	0,02762	0,050309	protein_coding	PSMD4	proteasome 26S subunit, non-ATPase 4 [Source:HGNC Symbol;Acc:HGNC:9561]	1
ENSG00000186432	2677,067	0,015557	0,19496	0,534052	1,296044	0,027587	0,061637	protein_coding	KPNA4	karyopherin subunit alpha 4 [Source:HGNC Symbol;Acc:HGNC:6397]	3
ENSG00000143337	2336,532	-0,01714	0,195373	0,534545	-1,29485	0,027836	-0,05733	protein_coding	TOR1AIP1	torsin 1A interacting protein 1 [Source:HGNC Symbol;Acc:HGNC:29456]	1
ENSG00000131100	1957,363	0,019211	0,196448	0,536849	1,291737	0,0279	0,051826	protein_coding	ATP6V1E1	ATPase H+ transporting V1 subunit E1 [Source:HGNC Symbol;Acc:HGNC:857]	22
ENSG00000130635	27838,43	0,006761	0,196849	0,536902	1,29058	0,026818	0,165431	protein_coding	COL5A1	collagen type V alpha 1 chain [Source:HGNC Symbol;Acc:HGNC:2209]	9
ENSG00000133639	1945,525	-0,01153	0,196934	0,536902	-1,29034	0,024494	-0,05424	protein_coding	BTG1	BTG anti-proliferation factor 1 [Source:HGNC Symbol;Acc:HGNC:1130]	12
ENSG00000131981	1664,515	0,015131	0,198046	0,539293	1,28714	0,028311	0,068634	protein_coding	LGALS3	galectin 3 [Source:HGNC Symbol;Acc:HGNC:6563]	14
ENSG00000135862	14004,8	0,01455	0,199113	0,540185	1,284084	0,028171	0,070656	protein_coding	LAMC1	laminin subunit gamma 1 [Source:HGNC Symbol;Acc:HGNC:6492]	1
ENSG00000113441	1703,909	0,017996	0,199429	0,540185	1,283179	0,026651	0,04936	protein_coding	LNPEP	leucyl and cystinyl aminopeptidase [Source:HGNC Symbol;Acc:HGNC:6656]	5
ENSG00000107263	2172,822	-0,02479	0,199362	0,540185	-1,28337	0,029646	-0,0467	protein_coding	RAPGEF1	Rap guanine nucleotide exchange factor 1 [Source:HGNC Symbol;Acc:HGNC:4568]	9
ENSG00000196914	8086,904	-0,01762	0,199169	0,540185	-1,28392	0,02638	-0,04908	protein_coding	ARHGEF12	Rho guanine nucleotide exchange factor 12 [Source:HGNC Symbol;Acc:HGNC:14193]	11
ENSG00000123143	3124,216	-0,01681	0,199547	0,540185	-1,28284	0,027641	-0,05679	protein_coding	PKN1	protein kinase N1 [Source:HGNC Symbol;Acc:HGNC:9405]	19
ENSG00000115241	2196,386	0,016923	0,200073	0,540337	1,281344	0,028266	0,059783	protein_coding	PPM1G	protein phosphatase, Mg2+/Mn2+ dependent 1G [Source:HGNC Symbol;Acc:HGNC:9278]	2
ENSG00000197903	1662,964	0,014054	0,199967	0,540337	1,281645	0,028367	0,075982	protein_coding	H2BC12	H2B clustered histone 12 [Source:HGNC Symbol;Acc:HGNC:13954]	6
ENSG00000100664	5068,818	0,017836	0,201104	0,542485	1,278413	0,027344	0,052344	protein_coding	EIF5	eukaryotic translation initiation factor 5 [Source:HGNC Symbol;Acc:HGNC:3299]	14
ENSG00000197747	2270,537	0,010917	0,202766	0,545133	1,27371	0,027684	0,097525	protein_coding	S100A10	S100 calcium binding protein A10 [Source:HGNC Symbol;Acc:HGNC:10487]	1
ENSG00000075711	1798,671	0,016901	0,202796	0,545133	1,273625	0,028527	0,061078	protein_coding	DLG1	discs large MAGUK scaffold protein 1 [Source:HGNC Symbol;Acc:HGNC:2900]	3
ENSG00000204569	1738,621	-0,01857	0,202406	0,545133	-1,27473	0,025987	-0,04557	protein_coding	PPP1R10	protein phosphatase 1 regulatory subunit 10 [Source:HGNC Symbol;Acc:HGNC:9284]	6
ENSG00000134684	1874,853	0,01767	0,204706	0,548985	1,268256	0,027081	0,051196	protein_coding	YARS1	tyrosyl-tRNA synthetase 1 [Source:HGNC Symbol;Acc:HGNC:12840]	1
ENSG00000167552	4907,257	0,011441	0,204644	0,548985	1,268432	0,027578	0,087658	protein_coding	TUBA1A	tubulin alpha 1a [Source:HGNC Symbol;Acc:HGNC:20766]	12
ENSG00000134324	1620,271	-0,01643	0,205748	0,550258	-1,26534	0,027307	-0,05549	protein_coding	LPIN1	lipin 1 [Source:HGNC Symbol;Acc:HGNC:13345]	2
ENSG00000163171	1570,902	0,014685	0,205617	0,550258	1,265711	0,027997	0,067445	protein_coding	CDC42EP3	CDC42 effector protein 3 [Source:HGNC Symbol;Acc:HGNC:16943]	2
ENSG00000087302	1683,858	0,018269	0,205898	0,550258	1,264925	0,023977	0,039544	protein_coding	RTRAF	RNA transcription, translation and transport factor [Source:HGNC Symbol;Acc:HGNC:23169]	14
ENSG00000103657	2983,37	-0,01844	0,20616	0,550318	-1,2642	0,025283	-0,04315	protein_coding	HERC1	HECT and RLD domain containing E3 ubiquitin protein ligase family member 1 [Source:HGNC Symbol;Acc:HGNC:4867]	15
ENSG00000162430	1577,926	0,016141	0,206435	0,550415	1,263428	0,02754	0,057598	protein_coding	SELENON	selenoprotein N [Source:HGNC Symbol;Acc:HGNC:15999]	1
ENSG00000126432	1604,666	0,013309	0,207261	0,551337	1,261134	0,028211	0,078899	protein_coding	PRDX5	peroxiredoxin 5 [Source:HGNC Symbol;Acc:HGNC:9355]	11
ENSG00000141905	4949,708	0,015877	0,207238	0,551337	1,261196	0,026897	0,054802	protein_coding	NFIC	nuclear factor I C [Source:HGNC Symbol;Acc:HGNC:7786]	19
ENSG00000198734	3053,91	0,009292	0,208403	0,553099	1,257968	0,027291	0,112088	protein_coding	F5	coagulation factor V [Source:HGNC Symbol;Acc:HGNC:3542]	1
ENSG00000111341	5492,272	-0,00315	0,208296	0,553099	-1,25826	0,026239	-0,32535	protein_coding	MGP	matrix Gla protein [Source:HGNC Symbol;Acc:HGNC:7060]	12
ENSG00000159216	3129,039	0,016275	0,208786	0,553266	1,256911	0,027137	0,054708	protein_coding	RUNX1	RUNX family transcription factor 1 [Source:HGNC Symbol;Acc:HGNC:10471]	21
ENSG00000147140	6371,409	0,016599	0,208948	0,553266	1,256464	0,02187	0,035147	protein_coding	NONO	non-POU domain containing octamer binding [Source:HGNC Symbol;Acc:HGNC:7871]	X
ENSG00000117152	1698,539	0,00775	0,209448	0,553337	1,255085	0,026907	0,130437	protein_coding	RGS4	regulator of G protein signaling 4 [Source:HGNC Symbol;Acc:HGNC:10000]	1
ENSG00000089280	2724,809	0,018251	0,209696	0,553337	1,254403	0,02604	0,045564	protein_coding	FUS	FUS RNA binding protein [Source:HGNC Symbol;Acc:HGNC:4010]	16
ENSG00000141753	7068,465	-0,01363	0,209641	0,553337	-1,25455	0,027891	-0,07208	protein_coding	IGFBP4	insulin like growth factor binding protein 4 [Source:HGNC Symbol;Acc:HGNC:5473]	17
ENSG00000138592	2026,353	0,016501	0,209951	0,553375	1,253701	0,025735	0,047619	protein_coding	USP8	ubiquitin specific peptidase 8 [Source:HGNC Symbol;Acc:HGNC:12631]	15
ENSG00000157227	13928,13	0,010562	0,210526	0,553622	1,252121	0,027539	0,097276	protein_coding	MMP14	matrix metallopeptidase 14 [Source:HGNC Symbol;Acc:HGNC:7160]	14
ENSG00000173482	2312,986	-0,01355	0,210319	0,553622	-1,25269	0,027012	-0,06402	protein_coding	PTPRM	protein tyrosine phosphatase receptor type M [Source:HGNC Symbol;Acc:HGNC:9675]	18
ENSG00000004487	1696,874	0,015784	0,212396	0,554108	1,247004	0,02581	0,049231	protein_coding	KDM1A	lysine demethylase 1A [Source:HGNC Symbol;Acc:HGNC:29079]	1
ENSG00000143384	4801,607	0,017081	0,212274	0,554108	1,247336	0,026092	0,047648	protein_coding	MCL1	MCL1 apoptosis regulator, BCL2 family member [Source:HGNC Symbol;Acc:HGNC:6943]	1
ENSG00000036257	2092,213	0,016928	0,211733	0,554108	1,248814	0,025883	0,047197	protein_coding	CUL3	cullin 3 [Source:HGNC Symbol;Acc:HGNC:2553]	2
ENSG00000134853	2518,953	-0,01259	0,211046	0,554108	-1,25069	0,027834	-0,07947	protein_coding	PDGFRA	platelet derived growth factor receptor alpha [Source:HGNC Symbol;Acc:HGNC:8803]	4
ENSG00000164741	3313,456	0,008045	0,211835	0,554108	1,248534	0,027025	0,129137	protein_coding	DLC1	DLC1 Rho GTPase activating protein [Source:HGNC Symbol;Acc:HGNC:2897]	8
ENSG00000148848	3679,236	0,008416	0,211796	0,554108	1,248642	0,027094	0,12251	protein_coding	ADAM12	ADAM metallopeptidase domain 12 [Source:HGNC Symbol;Acc:HGNC:190]	10
ENSG00000100401	1876,975	-0,01731	0,212173	0,554108	-1,24761	0,028362	-0,05735	protein_coding	RANGAP1	Ran GTPase activating protein 1 [Source:HGNC Symbol;Acc:HGNC:9854]	22
ENSG00000117859	1604,116	0,015962	0,213436	0,554927	1,244172	0,027487	0,057048	protein_coding	OSBPL9	oxysterol binding protein like 9 [Source:HGNC Symbol;Acc:HGNC:16386]	1

ENSG00000047849	6882,643	0,017752	0,213675	0,554927	1,243524	0,027199	0,050498	protein_coding	MAP4	microtubule associated protein 4 [Source:HGNC Symbol;Acc:HGNC:6862]	3
ENSG00000127947	2293,921	0,016604	0,2135	0,554927	1,243999	0,027349	0,054255	protein_coding	PTPN12	protein tyrosine phosphatase non-receptor type 12 [Source:HGNC Symbol;Acc:HGNC:9645]	7
ENSG00000019991	2076,148	-0,00369	0,213418	0,554927	-1,24422	0,026296	-0,27933	protein_coding	HGF	hepatocyte growth factor [Source:HGNC Symbol;Acc:HGNC:4893]	7
ENSG00000164300	1713,337	0,011255	0,214574	0,556008	1,241086	0,026772	0,074238	protein_coding	SERINC5	serine incorporator 5 [Source:HGNC Symbol;Acc:HGNC:18825]	5
ENSG00000067182	1626,905	0,014905	0,214341	0,556008	1,241717	0,027635	0,062034	protein_coding	TNFRSF1A	TNF receptor superfamily member 1A [Source:HGNC Symbol;Acc:HGNC:11916]	12
ENSG00000048707	2016,251	-0,01268	0,215363	0,557204	-1,23895	0,024788	-0,05096	protein_coding	VPS13D	vacuolar protein sorting 13 homolog D [Source:HGNC Symbol;Acc:HGNC:23595]	1
ENSG00000120802	2109,914	0,012424	0,21552	0,557204	1,238529	0,027734	0,078511	protein_coding	TMPO	thymopoietin [Source:HGNC Symbol;Acc:HGNC:11875]	12
ENSG00000110955	5286,183	0,020414	0,216157	0,557548	1,236813	0,022089	0,032468	protein_coding	ATP5F1B	ATP synthase F1 subunit beta [Source:HGNC Symbol;Acc:HGNC:830]	12
ENSG00000136478	1612,035	-0,01416	0,21638	0,557548	-1,23621	0,028054	-0,06931	protein_coding	TEX2	testis expressed 2 [Source:HGNC Symbol;Acc:HGNC:30884]	17
ENSG00000101350	2464,528	-0,0162	0,216225	0,557548	-1,23663	0,027127	-0,054	protein_coding	KIF3B	kinesin family member 3B [Source:HGNC Symbol;Acc:HGNC:6320]	20
ENSG00000117523	12820,79	0,017354	0,216722	0,557805	1,235291	0,020489	0,03076	protein_coding	PRRC2C	proline rich coiled-coil 2C [Source:HGNC Symbol;Acc:HGNC:24903]	1
ENSG00000132463	1978,033	0,017676	0,217665	0,558359	1,23276	0,024889	0,041936	protein_coding	GRSF1	G-rich RNA sequence binding factor 1 [Source:HGNC Symbol;Acc:HGNC:4610]	4
ENSG00000169926	3276,3	0,016706	0,217361	0,558359	1,233576	0,026802	0,050898	protein_coding	KLF13	Kruppel like factor 13 [Source:HGNC Symbol;Acc:HGNC:13672]	15
ENSG00000105438	2732,854	0,017608	0,217601	0,558359	1,232932	0,024294	0,040189	protein_coding	KDELR1	KDEL endoplasmic reticulum protein retention receptor 1 [Source:HGNC Symbol;Acc:HGNC:6304]	19
ENSG00000106397	1882,041	0,015111	0,21859	0,559392	1,230285	0,027151	0,057406	protein_coding	PLOD3	procollagen-lysine,2-oxoglutarate 5-dioxygenase 3 [Source:HGNC Symbol;Acc:HGNC:9083]	7
ENSG00000196233	1868,127	-0,02364	0,218347	0,559392	-1,23093	0,031604	-0,05177	protein_coding	LCOR	ligand dependent nuclear receptor corepressor [Source:HGNC Symbol;Acc:HGNC:29503]	10
ENSG00000166510	2570,613	-0,00815	0,218797	0,559392	-1,22973	0,027004	-0,12257	protein_coding	CCDC68	coiled-coil domain containing 68 [Source:HGNC Symbol;Acc:HGNC:24350]	18
ENSG00000075151	4769,478	-0,01746	0,219492	0,560545	-1,22788	0,024005	-0,03936	protein_coding	EIF4G3	eukaryotic translation initiation factor 4 gamma 3 [Source:HGNC Symbol;Acc:HGNC:3298]	1
ENSG00000188157	2134,122	0,011719	0,222067	0,560641	1,221051	0,027637	0,082431	protein_coding	AGRN	agrin [Source:HGNC Symbol;Acc:HGNC:329]	1
ENSG00000159674	2346,02	0,008337	0,2211	0,560641	1,223609	0,027033	0,118842	protein_coding	SPON2	spondin 2 [Source:HGNC Symbol;Acc:HGNC:11253]	4
ENSG00000171560	1826,084	0,002385	0,221517	0,560641	1,222503	0,026176	0,418247	protein_coding	FGA	fibrinogen alpha chain [Source:HGNC Symbol;Acc:HGNC:3661]	4
ENSG00000156639	1681,881	-0,01636	0,222453	0,560641	-1,22003	0,024267	-0,04128	protein_coding	ZFAND3	zinc finger AN1-type containing 3 [Source:HGNC Symbol;Acc:HGNC:18019]	6
ENSG00000105953	2900,985	-0,01457	0,221955	0,560641	-1,22134	0,025549	-0,04972	protein_coding	OGDH	oxoglutarate dehydrogenase [Source:HGNC Symbol;Acc:HGNC:8124]	7
ENSG00000106853	1576,222	0,0079	0,221999	0,560641	1,22123	0,026942	0,124387	protein_coding	PTGR1	prostaglandin reductase 1 [Source:HGNC Symbol;Acc:HGNC:18429]	9
ENSG00000156030	1576,906	-0,0153	0,221926	0,560641	-1,22142	0,026671	-0,05358	protein_coding	MIDEAS	mitotic deacetylase associated SANT domain protein [Source:HGNC Symbol;Acc:HGNC:19853]	14
ENSG00000182534	2715,036	0,014582	0,221369	0,560641	1,222895	0,027389	0,060737	protein_coding	MXRA7	matrix remodeling associated 7 [Source:HGNC Symbol;Acc:HGNC:7541]	17
ENSG00000130203	1837,028	0,004156	0,221739	0,560641	1,221917	0,026341	0,235297	protein_coding	APOE	apolipoprotein E [Source:HGNC Symbol;Acc:HGNC:613]	19
ENSG00000100242	1796,528	0,015426	0,222307	0,560641	1,220416	0,027135	0,055759	protein_coding	SUN2	Sad1 and UNC84 domain containing 2 [Source:HGNC Symbol;Acc:HGNC:14210]	22
ENSG00000005893	2756,652	0,015566	0,22151	0,560641	1,222522	0,027006	0,054656	protein_coding	LAMP2	lysosomal associated membrane protein 2 [Source:HGNC Symbol;Acc:HGNC:6501]	X
ENSG00000160211	3167,679	-0,01711	0,221894	0,560641	-1,22151	0,027921	-0,05471	protein_coding	G6PD	glucose-6-phosphate dehydrogenase [Source:HGNC Symbol;Acc:HGNC:4057]	X
ENSG00000127481	8659,811	-0,01119	0,222954	0,560675	-1,21871	0,023683	-0,04735	protein_coding	UBR4	ubiquitin protein ligase E3 component n-recognin 4 [Source:HGNC Symbol;Acc:HGNC:30313]	1
ENSG00000111328	2647,326	0,014236	0,222739	0,560675	1,219277	0,026622	0,056689	protein_coding	CDK2AP1	cyclin dependent kinase 2 associated protein 1 [Source:HGNC Symbol;Acc:HGNC:14002]	12
ENSG00000067798	1987,641	0,012017	0,223338	0,561027	1,2177	0,027628	0,079361	protein_coding	NAV3	neuron navigator 3 [Source:HGNC Symbol;Acc:HGNC:15998]	12
ENSG00000120438	3113,481	0,017037	0,224277	0,562771	1,215234	0,027084	0,050565	protein_coding	TCP1	t-complex 1 [Source:HGNC Symbol;Acc:HGNC:11655]	6
ENSG00000148337	2667,093	-0,0166	0,224631	0,563046	-1,21431	0,025949	-0,04677	protein_coding	CIZ1	CDKN1A interacting zinc finger protein 1 [Source:HGNC Symbol;Acc:HGNC:16744]	9
ENSG00000157985	2369,338	-0,01606	0,225236	0,563948	-1,21272	0,026314	-0,04941	protein_coding	AGAP1	ArfGAP with GTPase domain, ankyrin repeat and PH domain 1 [Source:HGNC Symbol;Acc:HGNC:16922]	2
ENSG00000144711	2239,12	-0,00954	0,226089	0,56449	-1,21049	0,026742	-0,08693	protein_coding	IQSEC1	IQ motif and Sec7 domain ArfGEF 1 [Source:HGNC Symbol;Acc:HGNC:29112]	3
ENSG00000055208	1876,135	0,017409	0,226189	0,56449	1,210236	0,027093	0,049497	protein_coding	TAB2	TGF-beta activated kinase 1 (MAP3K7) binding protein 2 [Source:HGNC Symbol;Acc:HGNC:17075]	6
ENSG00000088247	4574,087	0,017437	0,226098	0,56449	1,21047	0,023167	0,036485	protein_coding	KHSRP	KH-type splicing regulatory protein [Source:HGNC Symbol;Acc:HGNC:6316]	19
ENSG00000115415	3791,604	-0,01472	0,22693	0,565727	-1,2083	0,029689	-0,08229	protein_coding	STAT1	signal transducer and activator of transcription 1 [Source:HGNC Symbol;Acc:HGNC:11362]	2
ENSG00000143569	3623,926	0,017975	0,227881	0,566833	1,205836	0,02465	0,039909	protein_coding	UBAP2L	ubiquitin associated protein 2 like [Source:HGNC Symbol;Acc:HGNC:29877]	1
ENSG00000162736	2031,967	0,015841	0,228535	0,566833	1,20414	0,026687	0,051387	protein_coding	NCSTN	nicastrin [Source:HGNC Symbol;Acc:HGNC:17091]	1
ENSG00000168385	5256,995	0,01696	0,228292	0,566833	1,204771	0,024934	0,042138	protein_coding	SEPTIN2	septin 2 [Source:HGNC Symbol;Acc:HGNC:7729]	2
ENSG00000088888	2766,725	-0,01786	0,228605	0,566833	-1,20396	0,024586	-0,03977	protein_coding	MAVS	mitochondrial antiviral signaling protein [Source:HGNC Symbol;Acc:HGNC:29233]	20
ENSG00000141959	1663,382	0,016551	0,227813	0,566833	1,206011	0,026511	0,048867	protein_coding	PFKL	phosphofructokinase, liver type [Source:HGNC Symbol;Acc:HGNC:8876]	21
ENSG00000142534	6060,125	0,013674	0,229226	0,56776	1,202355	0,02647	0,056903	protein_coding	RPS11	ribosomal protein S11 [Source:HGNC Symbol;Acc:HGNC:10384]	19
ENSG00000234745	12791	0,015072	0,229546	0,56794	1,20153	0,028027	0,062264	protein_coding	HLA-B	major histocompatibility complex, class I, B [Source:HGNC Symbol;Acc:HGNC:4932]	6
ENSG00000017797	2319,231	0,015014	0,229856	0,568099	1,200729	0,02694	0,055126	protein_coding	RALBP1	ralA binding protein 1 [Source:HGNC Symbol;Acc:HGNC:9841]	18
ENSG00000039068	2221,253	0,014468	0,230256	0,568475	1,1997	0,026989	0,057325	protein_coding	CDH1	cadherin 1 [Source:HGNC Symbol;Acc:HGNC:1748]	16
ENSG00000156482	2675,214	0,01247	0,23062	0,568764	1,198763	0,02768	0,074935	protein_coding	RPL30	ribosomal protein L30 [Source:HGNC Symbol;Acc:HGNC:10333]	8
ENSG00000162521	2421,719	-0,01692	0,231691	0,570062	-1,19601	0,024326	-0,03992	protein_coding	RBBP4	RB binding protein 4, chromatin remodeling factor [Source:HGNC Symbol;Acc:HGNC:9887]	1
ENSG00000135624	2981,031	0,015826	0,231501	0,570062	1,196502	0,026502	0,050199	protein_coding	CCT7	chaperonin containing TCP1 subunit 7 [Source:HGNC Symbol;Acc:HGNC:1622]	2

ENSG00000115307	1740,744	0,016468	0,23303	0,570062	1,192589	0,023965	0,039276	protein_coding	AUP1	AUP1 lipid droplet regulating VLDL assembly factor [Source:HGNC Symbol;Acc:HGNC:891]	2
ENSG00000138398	1695,425	0,013263	0,232208	0,570062	1,19469	0,025686	0,052769	protein_coding	PPIG	peptidylprolyl isomerase G [Source:HGNC Symbol;Acc:HGNC:14650]	2
ENSG00000129116	6120,581	0,010674	0,233086	0,570062	1,192447	0,027319	0,085841	protein_coding	PALLD	palladin, cytoskeletal associated protein [Source:HGNC Symbol;Acc:HGNC:17068]	4
ENSG00000112531	1978,166	0,016512	0,232131	0,570062	1,194888	0,02803	0,055916	protein_coding	QKI	QKI, KH domain containing RNA binding [Source:HGNC Symbol;Acc:HGNC:21100]	6
ENSG00000178209	33859,17	0,012799	0,233129	0,570062	1,192339	0,027416	0,068807	protein_coding	PLEC	plectin [Source:HGNC Symbol;Acc:HGNC:9069]	8
ENSG00000205581	1878,313	0,015945	0,232737	0,570062	1,193339	0,02735	0,054004	protein_coding	HMGN1	high mobility group nucleosome binding domain 1 [Source:HGNC Symbol;Acc:HGNC:4984]	21
ENSG00000145920	2227,188	0,010094	0,233567	0,570528	1,191221	0,027701	0,106434	protein_coding	CPLX2	complexin 2 [Source:HGNC Symbol;Acc:HGNC:2310]	5
ENSG00000165934	1807,062	0,016473	0,234475	0,572138	1,188911	0,025844	0,045647	protein_coding	CPSF2	cleavage and polyadenylation specific factor 2 [Source:HGNC Symbol;Acc:HGNC:2325]	14
ENSG00000069329	1751,361	0,014251	0,235541	0,574132	1,186205	0,027324	0,059917	protein_coding	VPS35	VPS35 retromer complex component [Source:HGNC Symbol;Acc:HGNC:13487]	16
ENSG00000165671	2681,028	0,018254	0,236257	0,575268	1,184394	0,02379	0,036556	protein_coding	NSD1	nuclear receptor binding SET domain protein 1 [Source:HGNC Symbol;Acc:HGNC:14234]	5
ENSG00000168906	2342,647	-0,01533	0,236664	0,575648	-1,18337	0,02686	-0,05284	protein_coding	MAT2A	methionine adenosyltransferase 2A [Source:HGNC Symbol;Acc:HGNC:6904]	2
ENSG00000152102	2184,144	0,01831	0,237015	0,575893	1,182481	0,025566	0,041391	protein_coding	FAM168B	family with sequence similarity 168 member B [Source:HGNC Symbol;Acc:HGNC:27016]	2
ENSG00000134318	3322,108	0,013563	0,237554	0,576595	1,181123	0,026214	0,054591	protein_coding	ROCK2	Rho associated coiled-coil containing protein kinase 2 [Source:HGNC Symbol;Acc:HGNC:10252]	2
ENSG00000104408	2562,223	0,014279	0,238227	0,577619	1,17943	0,02607	0,051494	protein_coding	EIF3E	eukaryotic translation initiation factor 3 subunit E [Source:HGNC Symbol;Acc:HGNC:3277]	8
ENSG00000139278	1688,128	0,008347	0,238871	0,578571	1,177813	0,02694	0,109499	protein_coding	GLIPR1	GLI pathogenesis related 1 [Source:HGNC Symbol;Acc:HGNC:17001]	12
ENSG00000015171	2092,34	0,01671	0,239158	0,578656	1,177095	0,025241	0,042573	protein_coding	ZMYND11	zinc finger MYND-type containing 11 [Source:HGNC Symbol;Acc:HGNC:16966]	10
ENSG00000116871	3998,6	0,01715	0,240146	0,579875	1,174622	0,025129	0,04143	protein_coding	MAP7D1	MAP7 domain containing 1 [Source:HGNC Symbol;Acc:HGNC:25514]	1
ENSG00000143321	4925,284	0,016431	0,240417	0,579875	1,173944	0,025084	0,042325	protein_coding	HDGF	heparin binding growth factor [Source:HGNC Symbol;Acc:HGNC:4856]	1
ENSG00000257923	3200,62	-0,01829	0,240327	0,579875	-1,17417	0,02483	-0,03896	protein_coding	CUX1	cut like homeobox 1 [Source:HGNC Symbol;Acc:HGNC:2557]	7
ENSG00000136861	1990,788	0,012058	0,240917	0,580471	1,172698	0,027281	0,07107	protein_coding	CDK5RAP2	CDK5 regulatory subunit associated protein 2 [Source:HGNC Symbol;Acc:HGNC:18672]	9
ENSG00000181019	4313,33	-0,00878	0,241833	0,582068	-1,17042	0,026058	-0,07703	protein_coding	NQO1	NAD(P)H quinone dehydrogenase 1 [Source:HGNC Symbol;Acc:HGNC:2874]	16
ENSG00000119681	19479,37	0,010629	0,242268	0,582506	1,169337	0,027213	0,082481	protein_coding	LTBP2	latent transforming growth factor beta binding protein 2 [Source:HGNC Symbol;Acc:HGNC:6715]	14
ENSG00000097033	3403,549	0,018364	0,24322	0,582656	1,166976	0,027051	0,045576	protein_coding	SH3GLB1	SH3 domain containing GRB2 like, endophilin B1 [Source:HGNC Symbol;Acc:HGNC:10833]	1
ENSG00000169756	3457,536	0,014096	0,243789	0,582656	1,165569	0,026851	0,056162	protein_coding	LIMS1	LIM zinc finger domain containing 1 [Source:HGNC Symbol;Acc:HGNC:6616]	2
ENSG00000133706	2360,917	0,016132	0,24378	0,582656	1,165591	0,024384	0,040142	protein_coding	LARS1	leucyl-tRNA synthetase 1 [Source:HGNC Symbol;Acc:HGNC:6512]	5
ENSG00000110367	3606,52	0,015192	0,243284	0,582656	1,166817	0,026484	0,050538	protein_coding	DDX6	DEAD-box helicase 6 [Source:HGNC Symbol;Acc:HGNC:2747]	11
ENSG00000100815	2318,333	0,015952	0,243849	0,582656	1,165419	0,024858	0,042017	protein_coding	TRIP11	thyroid hormone receptor interactor 11 [Source:HGNC Symbol;Acc:HGNC:12305]	14
ENSG00000124164	1663,312	0,01593	0,243814	0,582656	1,165506	0,025581	0,044745	protein_coding	VAPB	VAMP associated protein B and C [Source:HGNC Symbol;Acc:HGNC:12649]	20
ENSG00000068650	3017,558	-0,01637	0,244434	0,582977	-1,16398	0,0283	-0,05651	protein_coding	ATP11A	ATPase phospholipid transporting 11A [Source:HGNC Symbol;Acc:HGNC:13552]	13
ENSG00000102241	2001,39	0,014623	0,244491	0,582977	1,163835	0,026667	0,05308	protein_coding	HTATSF1	HIV-1 Tat specific factor 1 [Source:HGNC Symbol;Acc:HGNC:5276]	X
ENSG00000161057	1963,576	0,015769	0,244764	0,583024	1,163162	0,025853	0,046099	protein_coding	PSMC2	proteasome 26S subunit, ATPase 2 [Source:HGNC Symbol;Acc:HGNC:9548]	7
ENSG00000022840	2505,262	-0,01636	0,245097	0,583215	-1,16234	0,023209	-0,03619	protein_coding	RNF10	ring finger protein 10 [Source:HGNC Symbol;Acc:HGNC:10055]	12
ENSG00000184897	1658,191	-0,01599	0,245554	0,583699	-1,16122	0,027559	-0,05356	protein_coding	H1-10	H1.10 linker histone [Source:HGNC Symbol;Acc:HGNC:4722]	3
ENSG00000116161	1831,427	0,015357	0,245916	0,583955	1,160326	0,028442	0,061736	protein_coding	CACYBP	calcyclin binding protein [Source:HGNC Symbol;Acc:HGNC:30423]	1
ENSG00000168488	2938,487	-0,01629	0,2465	0,584136	-1,15889	0,025006	-0,04176	protein_coding	ATXN2L	ataxin 2 like [Source:HGNC Symbol;Acc:HGNC:31326]	16
ENSG00000108848	2220,171	-0,01421	0,246425	0,584136	-1,15908	0,025538	-0,04793	protein_coding	LUC7L3	LUC7 like 3 pre-mRNA splicing factor [Source:HGNC Symbol;Acc:HGNC:24309]	17
ENSG00000129083	2920,987	0,014392	0,246852	0,58437	1,158029	0,026508	0,052649	protein_coding	COPB1	COPI coat complex subunit beta 1 [Source:HGNC Symbol;Acc:HGNC:2231]	11
ENSG00000136238	4558,196	0,01457	0,248666	0,587454	1,153595	0,02554	0,04696	protein_coding	RAC1	Rac family small GTPase 1 [Source:HGNC Symbol;Acc:HGNC:9801]	7
ENSG00000124181	1760,063	-0,01688	0,248555	0,587454	-1,15387	0,027666	-0,05099	protein_coding	PLCG1	phospholipase C gamma 1 [Source:HGNC Symbol;Acc:HGNC:9065]	20
ENSG00000070961	3717,501	-0,01455	0,249032	0,587715	-1,1527	0,025919	-0,04879	protein_coding	ATP2B1	ATPase plasma membrane Ca2+ transporting 1 [Source:HGNC Symbol;Acc:HGNC:814]	12
ENSG00000186575	1996,458	0,01339	0,250868	0,591441	1,148245	0,026927	0,05872	protein_coding	NF2	neurofibromin 2 [Source:HGNC Symbol;Acc:HGNC:7773]	22
ENSG00000048649	2124,789	0,017839	0,251226	0,591679	1,147377	0,025212	0,039751	protein_coding	RSF1	remodeling and spacing factor 1 [Source:HGNC Symbol;Acc:HGNC:18118]	11
ENSG00000113716	1848,345	0,015973	0,251727	0,592253	1,146165	0,024722	0,040815	protein_coding	HMGXB3	HMG-box containing 3 [Source:HGNC Symbol;Acc:HGNC:28982]	5
ENSG00000137801	42813,93	0,015064	0,252391	0,593209	1,144561	0,025831	0,046792	protein_coding	THBS1	thrombospondin 1 [Source:HGNC Symbol;Acc:HGNC:11785]	15
ENSG00000213585	2592,831	0,014351	0,252989	0,594007	1,143121	0,023526	0,038862	protein_coding	VDAC1	voltage dependent anion channel 1 [Source:HGNC Symbol;Acc:HGNC:12669]	5
ENSG00000054118	3646,491	0,016181	0,253753	0,594071	1,141281	0,021747	0,031909	protein_coding	THRAP3	thyroid hormone receptor associated protein 3 [Source:HGNC Symbol;Acc:HGNC:22964]	1
ENSG00000145362	1669,456	-0,0101	0,253553	0,594071	-1,14176	0,027031	-0,08231	protein_coding	ANK2	ankyrin 2 [Source:HGNC Symbol;Acc:HGNC:493]	4
ENSG00000065883	2039,535	-0,01561	0,25379	0,594071	-1,14119	0,024431	-0,04017	protein_coding	CDK13	cyclin dependent kinase 13 [Source:HGNC Symbol;Acc:HGNC:1733]	7
ENSG00000103035	1977,895	0,016474	0,254587	0,595331	1,139278	0,023707	0,036765	protein_coding	PSMD7	proteasome 26S subunit, non-ATPase 7 [Source:HGNC Symbol;Acc:HGNC:9565]	16
ENSG00000151012	2935,809	-0,01108	0,254931	0,595529	-1,13845	0,026092	-0,06141	protein_coding	SLC7A11	solute carrier family 7 member 11 [Source:HGNC Symbol;Acc:HGNC:11059]	4
ENSG00000102401	1584,958	-0,01315	0,255607	0,596502	-1,13684	0,02679	-0,05807	protein_coding	ARMCX3	armadillo repeat containing X-linked 3 [Source:HGNC Symbol;Acc:HGNC:24065]	X
ENSG00000198793	1974,747	-0,01482	0,257855	0,597766	-1,13148	0,025827	-0,04679	protein_coding	MTOR	mechanistic target of rapamycin kinase [Source:HGNC Symbol;Acc:HGNC:3942]	1

ENSG00000169213	2593,577	-0,01172	0,258162	0,597766	-1,13075	0,026686	-0,06386	protein_coding	RAB3B	RAB3B, member RAS oncogene family [Source:HGNC Symbol;Acc:HGNC:9778]	1
ENSG00000122218	5460,616	0,014613	0,258227	0,597766	1,130593	0,020868	0,030555	protein_coding	COPA	COPI coat complex subunit alpha [Source:HGNC Symbol;Acc:HGNC:2230]	1
ENSG00000137033	5803,289	-0,00577	0,256926	0,597766	-1,13369	0,026492	-0,14828	protein_coding	IL33	interleukin 33 [Source:HGNC Symbol;Acc:HGNC:16028]	9
ENSG00000099194	8551,276	-0,01469	0,256653	0,597766	-1,13434	0,027748	-0,0584	protein_coding	SCD	stearoyl-CoA desaturase [Source:HGNC Symbol;Acc:HGNC:10571]	10
ENSG00000168003	3184,958	-0,01468	0,258037	0,597766	-1,13104	0,027873	-0,05919	protein_coding	SLC3A2	solute carrier family 3 member 2 [Source:HGNC Symbol;Acc:HGNC:11026]	11
ENSG00000166848	1764,449	-0,01554	0,257239	0,597766	-1,13294	0,025675	-0,04464	protein_coding	TERF2IP	TERF2 interacting protein [Source:HGNC Symbol;Acc:HGNC:19246]	16
ENSG00000100201	6806,587	-0,01346	0,257344	0,597766	-1,13269	0,021857	-0,03412	protein_coding	DDX17	DEAD-box helicase 17 [Source:HGNC Symbol;Acc:HGNC:2740]	22
ENSG00000171467	1571,254	-0,01368	0,258601	0,598031	-1,1297	0,026247	-0,05201	protein_coding	ZNF318	zinc finger protein 318 [Source:HGNC Symbol;Acc:HGNC:13578]	6
ENSG00000115461	141086	-0,00425	0,258974	0,598292	-1,12882	0,026313	-0,19961	protein_coding	IGFBP5	insulin like growth factor binding protein 5 [Source:HGNC Symbol;Acc:HGNC:5474]	2
ENSG00000141867	4396,56	-0,0161	0,259283	0,598405	-1,12809	0,02589	-0,04416	protein_coding	BRD4	bromodomain containing 4 [Source:HGNC Symbol;Acc:HGNC:13575]	19
ENSG00000167658	31997,19	0,017159	0,259697	0,598761	1,127107	0,023738	0,035716	protein_coding	EEF2	eukaryotic translation elongation factor 2 [Source:HGNC Symbol;Acc:HGNC:3214]	19
ENSG00000134686	2429,008	-0,02091	0,260079	0,599041	-1,1262	0,027112	-0,04013	protein_coding	PHC2	polyhomeotic homolog 2 [Source:HGNC Symbol;Acc:HGNC:3183]	1
ENSG00000008441	1893,484	0,010904	0,26097	0,600492	1,124102	0,027019	0,073231	protein_coding	NFIX	nuclear factor I X [Source:HGNC Symbol;Acc:HGNC:7788]	19
ENSG00000003402	1583,789	-0,01368	0,261546	0,601216	-1,12274	0,026396	-0,05261	protein_coding	CFLAR	CASP8 and FADD like apoptosis regulator [Source:HGNC Symbol;Acc:HGNC:1876]	2
ENSG00000104388	2635,021	0,016012	0,262196	0,601509	1,121215	0,023563	0,03627	protein_coding	RAB2A	RAB2A, member RAS oncogene family [Source:HGNC Symbol;Acc:HGNC:9763]	8
ENSG00000123908	3159,83	-0,0141	0,262037	0,601509	-1,12159	0,025826	-0,04814	protein_coding	AGO2	argonaute RISC catalytic component 2 [Source:HGNC Symbol;Acc:HGNC:3263]	8
ENSG00000206503	18133,31	0,007964	0,264075	0,605215	1,116812	0,023948	0,051736	protein_coding	HLA-A	major histocompatibility complex, class I, A [Source:HGNC Symbol;Acc:HGNC:4931]	6
ENSG00000104635	2642,473	-0,01298	0,264782	0,60563	-1,11516	0,026593	-0,05619	protein_coding	SLC39A14	solute carrier family 39 member 14 [Source:HGNC Symbol;Acc:HGNC:20858]	8
ENSG00000067167	3209,121	0,014138	0,264782	0,60563	1,115159	0,026512	0,051517	protein_coding	TRAM1	translocation associated membrane protein 1 [Source:HGNC Symbol;Acc:HGNC:20568]	8
ENSG00000104368	8235,274	-0,00429	0,265545	0,606772	-1,11338	0,02631	-0,19362	protein_coding	PLAT	plasminogen activator, tissue type [Source:HGNC Symbol;Acc:HGNC:9051]	8
ENSG00000127022	18866,79	0,015881	0,26593	0,60705	1,112483	0,023495	0,03594	protein_coding	CANX	calnexin [Source:HGNC Symbol;Acc:HGNC:1473]	5
ENSG00000116786	1628,038	-0,01632	0,267272	0,607497	-1,10937	0,025469	-0,04149	protein_coding	PLEKHM2	pleckstrin homology and RUN domain containing M2 [Source:HGNC Symbol;Acc:HGNC:29131]	1
ENSG00000181104	8657,138	0,015181	0,267808	0,607497	1,108125	0,024447	0,039669	protein_coding	F2R	coagulation factor II thrombin receptor [Source:HGNC Symbol;Acc:HGNC:3537]	5
ENSG00000138279	1746,434	0,014013	0,267842	0,607497	1,108047	0,025672	0,046989	protein_coding	ANXA7	annexin A7 [Source:HGNC Symbol;Acc:HGNC:545]	10
ENSG00000060237	7326,931	-0,01633	0,267545	0,607497	-1,10873	0,023544	-0,03546	protein_coding	WNK1	WNK lysine deficient protein kinase 1 [Source:HGNC Symbol;Acc:HGNC:14540]	12
ENSG00000166147	19184,36	0,005586	0,267417	0,607497	1,109031	0,02645	0,147106	protein_coding	FBN1	fibrillin 1 [Source:HGNC Symbol;Acc:HGNC:3603]	15
ENSG00000159140	7999,644	-0,01289	0,267545	0,607497	-1,10873	0,02158	-0,03311	protein_coding	SON	SON DNA and RNA binding protein [Source:HGNC Symbol;Acc:HGNC:11183]	21
ENSG00000100243	4836,432	0,013133	0,267975	0,607497	1,107739	0,02596	0,050964	protein_coding	CYB5R3	cytochrome b5 reductase 3 [Source:HGNC Symbol;Acc:HGNC:2873]	22
ENSG00000198589	2101,483	-0,01303	0,268343	0,607734	-1,10689	0,026311	-0,05357	protein_coding	LRBA	LPS responsive beige-like anchor protein [Source:HGNC Symbol;Acc:HGNC:1742]	4
ENSG00000186472	2358,573	-0,00933	0,27098	0,609114	-1,10081	0,026837	-0,08391	protein_coding	PCLO	piccolo presynaptic cytomatrix protein [Source:HGNC Symbol;Acc:HGNC:13406]	7
ENSG00000168615	5334,034	0,012537	0,27107	0,609114	1,100601	0,026581	0,057208	protein_coding	ADAM9	ADAM metallopeptidase domain 9 [Source:HGNC Symbol;Acc:HGNC:216]	8
ENSG00000188229	5179,715	0,01127	0,269639	0,609114	1,103895	0,026538	0,06321	protein_coding	TUBB4B	tubulin beta 4B class IVb [Source:HGNC Symbol;Acc:HGNC:20771]	9
ENSG00000156599	2273,106	0,014627	0,270153	0,609114	1,102709	0,026111	0,047399	protein_coding	ZDHHC5	zinc finger DHHC-type palmitoyltransferase 5 [Source:HGNC Symbol;Acc:HGNC:18472]	11
ENSG00000151835	3020,741	0,008142	0,270302	0,609114	1,102367	0,026731	0,097495	protein_coding	SACS	sacsin molecular chaperone [Source:HGNC Symbol;Acc:HGNC:10519]	13
ENSG00000065150	3356,008	0,014792	0,269591	0,609114	1,104005	0,025071	0,042511	protein_coding	IPO5	importin 5 [Source:HGNC Symbol;Acc:HGNC:6402]	13
ENSG00000067900	3614,791	0,014552	0,269948	0,609114	1,103181	0,025934	0,04675	protein_coding	ROCK1	Rho associated coiled-coil containing protein kinase 1 [Source:HGNC Symbol;Acc:HGNC:10251]	18
ENSG00000147044	1836,319	0,015269	0,270616	0,609114	1,101646	0,023926	0,037607	protein_coding	CASK	calcium/calmodulin dependent serine protein kinase [Source:HGNC Symbol;Acc:HGNC:1497]	X
ENSG00000170035	1594,263	0,014286	0,272283	0,609457	1,097821	0,024975	0,042814	protein_coding	UBE2E3	ubiquitin conjugating enzyme E2 E3 [Source:HGNC Symbol;Acc:HGNC:12479]	2
ENSG00000138448	6457,96	0,011695	0,27218	0,609457	1,098057	0,026904	0,064527	protein_coding	ITGAV	integrin subunit alpha V [Source:HGNC Symbol;Acc:HGNC:6150]	2
ENSG00000164292	1716,639	-0,01449	0,271685	0,609457	-1,09919	0,022951	-0,03539	protein_coding	RHOBTB3	Rho related BTB domain containing 3 [Source:HGNC Symbol;Acc:HGNC:18757]	5
ENSG00000197063	1705,13	-0,01328	0,271958	0,609457	-1,09856	0,026202	-0,05157	protein_coding	MAFG	MAF bZIP transcription factor G [Source:HGNC Symbol;Acc:HGNC:6781]	17
ENSG00000204525	11103,18	0,013587	0,272939	0,609541	1,096319	0,027348	0,058394	protein_coding	HLA-C	major histocompatibility complex, class I, C [Source:HGNC Symbol;Acc:HGNC:4933]	6
ENSG00000134531	3130,924	0,011971	0,272972	0,609541	1,096244	0,026675	0,060479	protein_coding	EMP1	epithelial membrane protein 1 [Source:HGNC Symbol;Acc:HGNC:3333]	12
ENSG00000253719	1644,957	0,015232	0,273115	0,609541	1,095918	0,02379	0,037024	protein_coding	ATXN7L3B	ataxin 7 like 3B [Source:HGNC Symbol;Acc:HGNC:37931]	12
ENSG00000052841	1610,347	-0,01404	0,273589	0,610009	-1,09483	0,025543	-0,04572	protein_coding	TTC17	tetratricopeptide repeat domain 17 [Source:HGNC Symbol;Acc:HGNC:25596]	11
ENSG00000104738	1855,283	-0,01358	0,27401	0,61033	-1,09387	0,026651	-0,05326	protein_coding	MCM4	minichromosome maintenance complex component 4 [Source:HGNC Symbol;Acc:HGNC:6947]	8
ENSG00000138107	1957,128	0,014749	0,274529	0,61033	1,092692	0,023774	0,037476	protein_coding	ACTR1A	actin related protein 1A [Source:HGNC Symbol;Acc:HGNC:167]	10
ENSG00000181222	7032,815	0,014206	0,274331	0,61033	1,093142	0,026929	0,05281	protein_coding	POLR2A	RNA polymerase II subunit A [Source:HGNC Symbol;Acc:HGNC:9187]	17
ENSG00000154229	1711,169	0,010988	0,275277	0,611402	1,090991	0,026814	0,067581	protein_coding	PRKCA	protein kinase C alpha [Source:HGNC Symbol;Acc:HGNC:9393]	17
ENSG00000163359	117156,8	0,005921	0,275558	0,611435	1,090353	0,026467	0,133705	protein_coding	COL6A3	collagen type VI alpha 3 chain [Source:HGNC Symbol;Acc:HGNC:2213]	2
ENSG00000129128	2233,424	0,013227	0,276167	0,612198	1,08897	0,026393	0,052553	protein_coding	SPCS3	signal peptidase complex subunit 3 [Source:HGNC Symbol;Acc:HGNC:26212]	4
ENSG00000080845	3910,177	-0,01394	0,276564	0,612487	-1,08807	0,025604	-0,04598	protein_coding	DLGAP4	DLG associated protein 4 [Source:HGNC Symbol;Acc:HGNC:24476]	20

ENSG00000116747	2082,706	0,015744	0,276964	0,612783	1,087164	0,024306	0,037696	protein_coding	RO60	Ro60, Y RNA binding protein [Source:HGNC Symbol;Acc:HGNC:11313]	1
ENSG00000116604	1845,234	-0,01424	0,278163	0,614684	-1,08446	0,025448	-0,04441	protein_coding	MEF2D	myocyte enhancer factor 2D [Source:HGNC Symbol;Acc:HGNC:6997]	1
ENSG00000102531	3381,364	0,014732	0,278357	0,614684	1,084017	0,024772	0,040686	protein_coding	FNDC3A	fibronectin type III domain containing 3A [Source:HGNC Symbol;Acc:HGNC:20296]	13
ENSG00000106771	1724,737	-0,01344	0,279639	0,615741	-1,08113	0,025854	-0,04826	protein_coding	TMEM245	transmembrane protein 245 [Source:HGNC Symbol;Acc:HGNC:1363]	9
ENSG00000169813	3706,899	0,015566	0,279573	0,615741	1,08128	0,023667	0,035751	protein_coding	HNRNPF	heterogeneous nuclear ribonucleoprotein F [Source:HGNC Symbol;Acc:HGNC:5039]	10
ENSG00000005810	3603,881	-0,02273	0,279496	0,615741	-1,08145	0,029292	-0,04129	protein_coding	MYCBP2	MYC binding protein 2 [Source:HGNC Symbol;Acc:HGNC:23386]	13
ENSG00000183495	2970,007	-0,01487	0,280105	0,616177	-1,08008	0,025329	-0,04246	protein_coding	EP400	E1A binding protein p400 [Source:HGNC Symbol;Acc:HGNC:11958]	12
ENSG00000087087	2287,111	0,014743	0,280849	0,617224	1,078414	0,025374	0,0428	protein_coding	SRRT	serrate, RNA effector molecule [Source:HGNC Symbol;Acc:HGNC:24101]	7
ENSG00000130338	3003,191	-0,01463	0,281307	0,61764	-1,07739	0,02411	-0,03821	protein_coding	TULP4	TUB like protein 4 [Source:HGNC Symbol;Acc:HGNC:15530]	6
ENSG00000044115	7681,888	0,01469	0,28205	0,617724	1,075725	0,024145	0,038199	protein_coding	CTNNA1	catenin alpha 1 [Source:HGNC Symbol;Acc:HGNC:2509]	5
ENSG00000159069	1701,343	-0,01416	0,281711	0,617724	-1,07648	0,025281	-0,04352	protein_coding	FBXW5	F-box and WD repeat domain containing 5 [Source:HGNC Symbol;Acc:HGNC:13613]	9
ENSG00000160299	2269,195	-0,01519	0,28215	0,617724	-1,0755	0,025485	-0,04229	protein_coding	PCNT	pericentrin [Source:HGNC Symbol;Acc:HGNC:16068]	21
ENSG00000127616	5270,611	0,014859	0,283121	0,61926	1,073334	0,024322	0,038476	protein_coding	SMARCA4	SWI/SNF related, matrix associated, actin dependent regulator of chromatin, subfamily a, member 4 [Source:HGNC Sym	19
ENSG00000104687	1748,625	-0,01472	0,283464	0,619421	-1,07257	0,025849	-0,04467	protein_coding	GSR	glutathione-disulfide reductase [Source:HGNC Symbol;Acc:HGNC:4623]	8
ENSG00000131626	2237,694	0,014793	0,283737	0,619429	1,071963	0,023419	0,035601	protein_coding	PPFIA1	PTPRF interacting protein alpha 1 [Source:HGNC Symbol;Acc:HGNC:9245]	11
ENSG00000079459	1968,088	-0,01348	0,28431	0,620091	-1,07069	0,02566	-0,04663	protein_coding	FDFT1	farnesyl-diphosphate farnesyltransferase 1 [Source:HGNC Symbol;Acc:HGNC:3629]	8
ENSG00000164733	19357,27	0,007984	0,28495	0,620312	1,069266	0,026615	0,092293	protein_coding	CTSB	cathepsin B [Source:HGNC Symbol;Acc:HGNC:2527]	8
ENSG00000150760	2703,942	0,013224	0,284875	0,620312	1,069433	0,025885	0,048499	protein_coding	DOCK1	dedicator of cytokinesis 1 [Source:HGNC Symbol;Acc:HGNC:2987]	10
ENSG00000130382	1745,907	-0,01215	0,285562	0,620604	-1,06791	0,026312	-0,05493	protein_coding	MLLT1	MLLT1 super elongation complex subunit [Source:HGNC Symbol;Acc:HGNC:7134]	19
ENSG00000185825	1853,683	0,014672	0,285624	0,620604	1,067771	0,023863	0,036995	protein_coding	BCAP31	B cell receptor associated protein 31 [Source:HGNC Symbol;Acc:HGNC:16695]	X
ENSG00000161791	1722,708	0,011031	0,286366	0,621433	1,066127	0,027693	0,080261	protein_coding	FMNL3	formin like 3 [Source:HGNC Symbol;Acc:HGNC:23698]	12
ENSG00000176871	2687,723	0,013027	0,286601	0,621433	1,065609	0,026326	0,051674	protein_coding	WSB2	WD repeat and SOCS box containing 2 [Source:HGNC Symbol;Acc:HGNC:19222]	12
ENSG00000196576	8735,825	0,014595	0,286815	0,621433	1,065134	0,023843	0,036913	protein_coding	PLXNB2	plexin B2 [Source:HGNC Symbol;Acc:HGNC:9104]	22
ENSG00000198682	1789,034	0,009836	0,287454	0,621647	1,063723	0,02666	0,072256	protein_coding	PAPSS2	3'-phosphoadenosine 5'-phosphosulfate synthase 2 [Source:HGNC Symbol;Acc:HGNC:8604]	10
ENSG00000102225	1713,611	-0,01467	0,287437	0,621647	-1,06376	0,023634	-0,03613	protein_coding	CDK16	cyclin dependent kinase 16 [Source:HGNC Symbol;Acc:HGNC:8749]	X
ENSG00000124177	2456,535	-0,0114	0,288462	0,62324	-1,0615	0,025954	-0,05465	protein_coding	CHD6	chromodomain helicase DNA binding protein 6 [Source:HGNC Symbol;Acc:HGNC:19057]	20
ENSG00000167123	2080,32	0,012653	0,288774	0,62333	1,060815	0,026539	0,05431	protein_coding	CERCAM	cerebral endothelial cell adhesion molecule [Source:HGNC Symbol;Acc:HGNC:23723]	9
ENSG00000106636	2244,662	0,018993	0,290209	0,625839	1,057664	0,024024	0,032538	protein_coding	YKT6	YKT6 v-SNARE homolog [Source:HGNC Symbol;Acc:HGNC:16959]	7
ENSG00000157657	1846,385	-0,01293	0,290837	0,626607	-1,05629	0,025677	-0,04753	protein_coding	ZNF618	zinc finger protein 618 [Source:HGNC Symbol;Acc:HGNC:29416]	9
ENSG00000136628	4108,433	0,014297	0,291965	0,627275	1,053821	0,024763	0,040231	protein_coding	EPRS1	glutamyl-prolyl-tRNA synthetase 1 [Source:HGNC Symbol;Acc:HGNC:3418]	1
ENSG00000115904	1645,303	-0,01401	0,291704	0,627275	-1,05439	0,02563	-0,04452	protein_coding	SOS1	SOS Ras/Rac guanine nucleotide exchange factor 1 [Source:HGNC Symbol;Acc:HGNC:11187]	2
ENSG00000148248	5156,518	0,015238	0,291691	0,627275	1,054418	0,025278	0,040582	protein_coding	SURF4	surfeit 4 [Source:HGNC Symbol;Acc:HGNC:11476]	9
ENSG00000112769	2834,191	0,007995	0,292675	0,627628	1,052271	0,026575	0,089567	protein_coding	LAMA4	laminin subunit alpha 4 [Source:HGNC Symbol;Acc:HGNC:6484]	6
ENSG00000079332	1796,164	0,012891	0,292418	0,627628	1,052833	0,025851	0,048516	protein_coding	SAR1A	secretion associated Ras related GTPase 1A [Source:HGNC Symbol;Acc:HGNC:10534]	10
ENSG00000080345	2634,518	0,014314	0,293329	0,628446	1,050846	0,024575	0,039398	protein_coding	RIF1	replication timing regulatory factor 1 [Source:HGNC Symbol;Acc:HGNC:23207]	2
ENSG00000137573	9955,636	0,00754	0,293775	0,628816	1,049876	0,02654	0,094978	protein_coding	SULF1	sulfatase 1 [Source:HGNC Symbol;Acc:HGNC:20391]	8
ENSG00000018408	4715,825	0,011036	0,294294	0,629065	1,048747	0,02619	0,057683	protein_coding	WWTR1	WW domain containing transcription regulator 1 [Source:HGNC Symbol;Acc:HGNC:24042]	3
ENSG00000113361	7296,912	0,009428	0,294439	0,629065	1,048434	0,026593	0,073513	protein_coding	CDH6	cadherin 6 [Source:HGNC Symbol;Acc:HGNC:1765]	5
ENSG00000079819	2375,068	0,012695	0,295865	0,630952	1,045341	0,026304	0,051663	protein_coding	EPB41L2	erythrocyte membrane protein band 4.1 like 2 [Source:HGNC Symbol;Acc:HGNC:3379]	6
ENSG00000147677	2269,149	0,014439	0,295904	0,630952	1,045257	0,024132	0,037402	protein_coding	EIF3H	eukaryotic translation initiation factor 3 subunit H [Source:HGNC Symbol;Acc:HGNC:3273]	8
ENSG00000198695	6644,825	-0,00504	0,296144	0,630952	-1,04474	0,026324	-0,14284	protein_coding	MT-ND6	mitochondrially encoded NADH:ubiquinone oxidoreductase core subunit 6 [Source:HGNC Symbol;Acc:HGNC:7462]	MT
ENSG00000143164	1649,826	0,013491	0,298538	0,633121	1,039573	0,024926	0,041818	protein_coding	DCAF6	DDB1 and CUL4 associated factor 6 [Source:HGNC Symbol;Acc:HGNC:30002]	1
ENSG00000129625	2331,669	0,014863	0,297871	0,633121	1,04101	0,023107	0,033588	protein_coding	REEP5	receptor accessory protein 5 [Source:HGNC Symbol;Acc:HGNC:30077]	5
ENSG00000164244	1627,938	0,013171	0,297896	0,633121	1,040956	0,025146	0,043534	protein_coding	PRRC1	proline rich coiled-coil 1 [Source:HGNC Symbol;Acc:HGNC:28164]	5
ENSG00000189241	2170,845	0,01212	0,298487	0,633121	1,039683	0,024745	0,043749	protein_coding	TSPYL1	TSPY like 1 [Source:HGNC Symbol;Acc:HGNC:12382]	6
ENSG00000106153	2199,504	0,012792	0,298267	0,633121	1,040156	0,026191	0,050327	protein_coding	CHCHD2	coiled-coil-helix-coiled-coil-helix domain containing 2 [Source:HGNC Symbol;Acc:HGNC:21645]	7
ENSG00000156515	4066,651	0,012304	0,298982	0,633478	1,03862	0,026014	0,050777	protein_coding	HK1	hexokinase 1 [Source:HGNC Symbol;Acc:HGNC:4922]	10
ENSG00000079308	9334,584	0,005359	0,299924	0,634307	1,036595	0,02635	0,133618	protein_coding	TNS1	tensin 1 [Source:HGNC Symbol;Acc:HGNC:11973]	2
ENSG00000163939	2514,829	0,014353	0,299668	0,634307	1,037146	0,022652	0,032686	protein_coding	PBRM1	polybromo 1 [Source:HGNC Symbol;Acc:HGNC:30064]	3
ENSG00000141985	1757,724	0,013969	0,300647	0,635251	1,035048	0,02508	0,041351	protein_coding	SH3GL1	SH3 domain containing GRB2 like 1, endophilin A2 [Source:HGNC Symbol;Acc:HGNC:10830]	19
ENSG00000170017	3856,511	0,012176	0,302248	0,637229	1,031625	0,026192	0,052078	protein_coding	ALCAM	activated leukocyte cell adhesion molecule [Source:HGNC Symbol;Acc:HGNC:400]	3
ENSG00000149547	1581,828	0,013385	0,302022	0,637229	1,032107	0,025082	0,042437	protein_coding	EI24	EI24 autophagy associated transmembrane protein [Source:HGNC Symbol;Acc:HGNC:13276]	11

ENSG00000101439	8585,707	-0,0105	0,302414	0,637229	-1,03127	0,024059	-0,04314	protein_coding	CST3	cystatin C [Source:HGNC Symbol;Acc:HGNC:2475]	20
ENSG00000130508	21972,87	0,008272	0,303875	0,639042	1,028159	0,026511	0,082027	protein_coding	PXDN	peroxidasin [Source:HGNC Symbol;Acc:HGNC:14966]	2
ENSG00000058262	6342,859	0,014079	0,303676	0,639042	1,028583	0,023049	0,033828	protein_coding	SEC61A1	SEC61 translocon subunit alpha 1 [Source:HGNC Symbol;Acc:HGNC:18276]	3
ENSG00000077238	1933,277	0,011979	0,304107	0,639042	1,027666	0,026269	0,053193	protein_coding	IL4R	interleukin 4 receptor [Source:HGNC Symbol;Acc:HGNC:6015]	16
ENSG00000184007	3774,565	0,013638	0,305523	0,639186	1,024662	0,024663	0,039851	protein_coding	PTP4A2	protein tyrosine phosphatase 4A2 [Source:HGNC Symbol;Acc:HGNC:9635]	1
ENSG00000198162	2974,808	0,01378	0,30612	0,639186	1,023397	0,024127	0,03754	protein_coding	MAN1A2	mannosidase alpha class 1A member 2 [Source:HGNC Symbol;Acc:HGNC:6822]	1
ENSG00000068654	1943,915	0,010042	0,305608	0,639186	1,024482	0,024028	0,043605	protein_coding	POLR1A	RNA polymerase I subunit A [Source:HGNC Symbol;Acc:HGNC:17264]	2
ENSG00000114867	12730,35	0,013643	0,305681	0,639186	1,024328	0,023448	0,035419	protein_coding	EIF4G1	eukaryotic translation initiation factor 4 gamma 1 [Source:HGNC Symbol;Acc:HGNC:3296]	3
ENSG00000165219	2277,65	-0,01356	0,306023	0,639186	-1,0236	0,025019	-0,04143	protein_coding	GAPVD1	GTPase activating protein and VPS9 domains 1 [Source:HGNC Symbol;Acc:HGNC:23375]	9
ENSG00000104067	5099,257	0,015675	0,305285	0,639186	1,025165	0,021746	0,029007	protein_coding	TJP1	tight junction protein 1 [Source:HGNC Symbol;Acc:HGNC:11827]	15
ENSG00000135720	2874,4	0,014162	0,304656	0,639186	1,0265	0,023411	0,034763	protein_coding	DYNC1LI2	dynein cytoplasmic 1 light intermediate chain 2 [Source:HGNC Symbol;Acc:HGNC:2966]	16
ENSG00000116580	1640,486	-0,01068	0,306499	0,639397	-1,0226	0,024142	-0,04287	protein_coding	GON4L	gon-4 like [Source:HGNC Symbol;Acc:HGNC:25973]	1
ENSG00000115457	1748,266	-0,00867	0,308103	0,640421	-1,01921	0,026467	-0,07591	protein_coding	IGFBP2	insulin like growth factor binding protein 2 [Source:HGNC Symbol;Acc:HGNC:5471]	2
ENSG00000136295	2387,875	-0,00796	0,307852	0,640421	-1,01974	0,023798	-0,04624	protein_coding	TTYH3	tweety family member 3 [Source:HGNC Symbol;Acc:HGNC:22222]	7
ENSG00000134001	1566,412	0,012555	0,30809	0,640421	1,019237	0,026032	0,049135	protein_coding	EIF2S1	eukaryotic translation initiation factor 2 subunit alpha [Source:HGNC Symbol;Acc:HGNC:3265]	14
ENSG00000100354	2763,327	-0,0136	0,307876	0,640421	-1,01969	0,026745	-0,04999	protein_coding	TNRC6B	trinucleotide repeat containing adaptor 6B [Source:HGNC Symbol;Acc:HGNC:29190]	22
ENSG00000136603	4163,866	0,01242	0,309422	0,640914	1,016436	0,025904	0,048563	protein_coding	SKIL	SKI like proto-oncogene [Source:HGNC Symbol;Acc:HGNC:10897]	3
ENSG00000120594	2257,481	-0,00858	0,309577	0,640914	-1,01611	0,026628	-0,08071	protein_coding	PLXDC2	plexin domain containing 2 [Source:HGNC Symbol;Acc:HGNC:21013]	10
ENSG00000128989	3043,619	0,012573	0,309421	0,640914	1,016438	0,025227	0,044243	protein_coding	ARPP19	cAMP regulated phosphoprotein 19 [Source:HGNC Symbol;Acc:HGNC:16967]	15
ENSG00000185359	2562,751	-0,01284	0,309733	0,640914	-1,01578	0,024887	-0,04194	protein_coding	HGS	hepatocyte growth factor-regulated tyrosine kinase substrate [Source:HGNC Symbol;Acc:HGNC:4897]	17
ENSG00000125753	1876,463	0,014494	0,309596	0,640914	1,016071	0,024236	0,036636	protein_coding	VASP	vasodilator stimulated phosphoprotein [Source:HGNC Symbol;Acc:HGNC:12652]	19
ENSG00000106263	4053,828	-0,01283	0,310039	0,640971	-1,01514	0,019533	-0,02544	protein_coding	EIF3B	eukaryotic translation initiation factor 3 subunit B [Source:HGNC Symbol;Acc:HGNC:3280]	7
ENSG00000168610	3665,196	-0,01288	0,31109	0,642566	-1,01294	0,024949	-0,04201	protein_coding	STAT3	signal transducer and activator of transcription 3 [Source:HGNC Symbol;Acc:HGNC:11364]	17
ENSG00000116260	5045,912	0,013481	0,313746	0,643704	1,007393	0,02523	0,04181	protein_coding	QSOX1	quiescin sulfhydryl oxidase 1 [Source:HGNC Symbol;Acc:HGNC:9756]	1
ENSG00000182827	2147,82	0,013234	0,313513	0,643704	1,007878	0,024223	0,038138	protein_coding	ACBD3	acyl-CoA binding domain containing 3 [Source:HGNC Symbol;Acc:HGNC:15453]	1
ENSG00000138768	2761,779	0,013592	0,313879	0,643704	1,007116	0,022253	0,031327	protein_coding	USO1	USO1 vesicle transport factor [Source:HGNC Symbol;Acc:HGNC:30904]	4
ENSG00000138688	2657,963	-0,02154	0,312456	0,643704	-1,01008	0,031513	-0,04678	protein_coding	KIAA1109	KIAA1109 [Source:HGNC Symbol;Acc:HGNC:26953]	4
ENSG00000065154	1746,131	0,0125	0,313326	0,643704	1,008268	0,025463	0,045336	protein_coding	OAT	ornithine aminotransferase [Source:HGNC Symbol;Acc:HGNC:8091]	10
ENSG00000185567	1638,789	0,005913	0,312688	0,643704	1,009598	0,026348	0,113187	protein_coding	AHNAK2	AHNAK nucleoprotein 2 [Source:HGNC Symbol;Acc:HGNC:20125]	14
ENSG00000087191	2042,792	0,012419	0,31371	0,643704	1,007468	0,025238	0,044255	protein_coding	PSMC5	proteasome 26S subunit, ATPase 5 [Source:HGNC Symbol;Acc:HGNC:9552]	17
ENSG00000118705	7246,671	0,013761	0,313042	0,643704	1,00886	0,023314	0,034322	protein_coding	RPN2	ribophorin II [Source:HGNC Symbol;Acc:HGNC:10382]	20
ENSG00000096696	20175,52	0,011018	0,315081	0,645214	1,004617	0,026039	0,054013	protein_coding	DSP	desmoplakin [Source:HGNC Symbol;Acc:HGNC:3052]	6
ENSG00000019144	6597,328	0,013143	0,315176	0,645214	1,00442	0,026162	0,047321	protein_coding	PHLDB1	pleckstrin homology like domain family B member 1 [Source:HGNC Symbol;Acc:HGNC:23697]	11
ENSG00000168461	2160,523	0,011017	0,315853	0,646024	1,003017	0,026243	0,05581	protein_coding	RAB31	RAB31, member RAS oncogene family [Source:HGNC Symbol;Acc:HGNC:9771]	18
ENSG00000169439	2522,355	0,009059	0,316811	0,64626	1,001034	0,026281	0,06768	protein_coding	SDC2	syndecan 2 [Source:HGNC Symbol;Acc:HGNC:10659]	8
ENSG00000106723	1803,458	0,013473	0,316402	0,64626	1,001879	0,023622	0,035424	protein_coding	SPIN1	spindlin 1 [Source:HGNC Symbol;Acc:HGNC:11243]	9
ENSG00000170348	4919,962	0,014335	0,316559	0,64626	1,001554	0,023515	0,034033	protein_coding	TMED10	transmembrane p24 trafficking protein 10 [Source:HGNC Symbol;Acc:HGNC:16998]	14
ENSG00000160294	2355,438	-0,01269	0,317247	0,646578	-1,00013	0,024874	-0,04153	protein_coding	MCM3AP	minichromosome maintenance complex component 3 associated protein [Source:HGNC Symbol;Acc:HGNC:6946]	21
ENSG00000121741	1674,319	-0,0138	0,317962	0,646888	-0,99865	0,024754	-0,03892	protein_coding	ZMYM2	zinc finger MYM-type containing 2 [Source:HGNC Symbol;Acc:HGNC:12989]	13
ENSG00000140948	1598,461	-0,01322	0,317747	0,646888	-0,9991	0,024287	-0,03806	protein_coding	ZCCHC14	zinc finger CCHC-type containing 14 [Source:HGNC Symbol;Acc:HGNC:24134]	16
ENSG00000116560	6056,843	0,013467	0,318562	0,647537	0,997416	0,023117	0,033645	protein_coding	SFPQ	splicing factor proline and glutamine rich [Source:HGNC Symbol;Acc:HGNC:10774]	1
ENSG00000091317	2152,01	0,011383	0,32018	0,650252	0,994087	0,025747	0,049736	protein_coding	CMTM6	CKLF like MARVEL transmembrane domain containing 6 [Source:HGNC Symbol;Acc:HGNC:19177]	3
ENSG00000055070	2356,819	0,012681	0,321165	0,650625	0,992067	0,023363	0,035208	protein_coding	SZRD1	SUZ RNA binding domain containing 1 [Source:HGNC Symbol;Acc:HGNC:30232]	1
ENSG00000115414	479894,7	0,007175	0,322297	0,650625	0,989749	0,026367	0,088393	protein_coding	FN1	fibronectin 1 [Source:HGNC Symbol;Acc:HGNC:3778]	2
ENSG00000163110	4729,367	0,012832	0,322017	0,650625	0,990322	0,024688	0,040034	protein_coding	PDLIM5	PDZ and LIM domain 5 [Source:HGNC Symbol;Acc:HGNC:17468]	4
ENSG00000064651	1571,326	0,010735	0,322235	0,650625	0,989875	0,026031	0,054329	protein_coding	SLC12A2	solute carrier family 12 member 2 [Source:HGNC Symbol;Acc:HGNC:10911]	5
ENSG00000111799	23945,9	-0,00764	0,32198	0,650625	-0,9904	0,026365	-0,08217	protein_coding	COL12A1	collagen type XII alpha 1 chain [Source:HGNC Symbol;Acc:HGNC:2188]	6
ENSG00000164916	2779,628	-0,01376	0,322909	0,650625	-0,9885	0,022066	-0,0301	protein_coding	FOXK1	forkhead box K1 [Source:HGNC Symbol;Acc:HGNC:23480]	7
ENSG00000165476	1993,876	0,011203	0,32282	0,650625	0,988678	0,025883	0,051147	protein_coding	REEP3	receptor accessory protein 3 [Source:HGNC Symbol;Acc:HGNC:23711]	10
ENSG00000178695	2477,775	-0,0111	0,322798	0,650625	-0,98872	0,0258	-0,05082	protein_coding	KCTD12	potassium channel tetramerization domain containing 12 [Source:HGNC Symbol;Acc:HGNC:14678]	13
ENSG00000065000	6552,052	-0,01252	0,320914	0,650625	-0,99258	0,02465	-0,04054	protein_coding	AP3D1	adaptor related protein complex 3 subunit delta 1 [Source:HGNC Symbol;Acc:HGNC:568]	19
ENSG00000117632	1834,934	0,009874	0,323457	0,650872	0,987379	0,026182	0,060031	protein_coding	STMN1	stathmin 1 [Source:HGNC Symbol;Acc:HGNC:6510]	1

ENSG00000165733	1813,378	0,012461	0,323597	0,650872	0,987093	0,024802	0,041127	protein_coding	BMS1	BMS1 ribosome biogenesis factor [Source:HGNC Symbol;Acc:HGNC:23505]	10
ENSG00000197461	2057,853	0,006421	0,324221	0,650989	0,98582	0,026335	0,099022	protein_coding	PDGFA	platelet derived growth factor subunit A [Source:HGNC Symbol;Acc:HGNC:8799]	7
ENSG00000090615	5819,469	-0,01276	0,323998	0,650989	-0,98628	0,022005	-0,03069	protein_coding	GOLGA3	golgin A3 [Source:HGNC Symbol;Acc:HGNC:4426]	12
ENSG00000124201	2613,101	-0,01253	0,324796	0,651574	-0,98465	0,022856	-0,03339	protein_coding	ZNFX1	zinc finger NFX1-type containing 1 [Source:HGNC Symbol;Acc:HGNC:29271]	20
ENSG00000129292	1858,931	0,012899	0,32591	0,652587	0,982386	0,024117	0,037293	protein_coding	PHF20L1	PHD finger protein 20 like 1 [Source:HGNC Symbol;Acc:HGNC:24280]	8
ENSG00000011485	1564,436	0,012866	0,326068	0,652587	0,982064	0,024677	0,039575	protein_coding	PPP5C	protein phosphatase 5 catalytic subunit [Source:HGNC Symbol;Acc:HGNC:9322]	19
ENSG00000100241	2758,667	-0,0199	0,326151	0,652587	-0,9819	0,02686	-0,03561	protein_coding	SBF1	SET binding factor 1 [Source:HGNC Symbol;Acc:HGNC:10542]	22
ENSG00000113575	1714,319	0,010588	0,328272	0,656259	0,9776	0,025917	0,053269	protein_coding	PPP2CA	protein phosphatase 2 catalytic subunit alpha [Source:HGNC Symbol;Acc:HGNC:9299]	5
ENSG00000197712	3457,616	0,012596	0,329226	0,656454	0,975675	0,024415	0,038702	protein_coding	FAM114A1	family with sequence similarity 114 member A1 [Source:HGNC Symbol;Acc:HGNC:25087]	4
ENSG00000186174	3418,594	0,013075	0,328769	0,656454	0,976597	0,023845	0,03581	protein_coding	BCL9L	BCL9 like [Source:HGNC Symbol;Acc:HGNC:23688]	11
ENSG00000137642	1693,09	0,005714	0,328967	0,656454	0,976196	0,026293	0,110634	protein_coding	SORL1	sortilin related receptor 1 [Source:HGNC Symbol;Acc:HGNC:11185]	11
ENSG00000134970	1634,64	0,009587	0,331507	0,659892	0,971083	0,026469	0,064861	protein_coding	TMED7	transmembrane p24 trafficking protein 7 [Source:HGNC Symbol;Acc:HGNC:24253]	5
ENSG00000177119	2551,222	0,009697	0,331523	0,659892	0,971051	0,025896	0,056665	protein_coding	ANO6	anoctamin 6 [Source:HGNC Symbol;Acc:HGNC:25240]	12
ENSG00000144357	1565,894	0,012343	0,33276	0,661476	0,96857	0,024525	0,03935	protein_coding	UBR3	ubiquitin protein ligase E3 component n-recognin 3 [Source:HGNC Symbol;Acc:HGNC:30467]	2
ENSG00000060339	2175,506	0,01274	0,332894	0,661476	0,968301	0,024119	0,036991	protein_coding	CCAR1	cell division cycle and apoptosis regulator 1 [Source:HGNC Symbol;Acc:HGNC:24236]	10
ENSG00000113013	7069,789	-0,01434	0,333236	0,661585	-0,96762	0,021587	-0,0279	protein_coding	HSPA9	heat shock protein family A (Hsp70) member 9 [Source:HGNC Symbol;Acc:HGNC:5244]	5
ENSG00000087274	3925,166	0,012863	0,335138	0,662532	0,963817	0,023916	0,035882	protein_coding	ADD1	adducin 1 [Source:HGNC Symbol;Acc:HGNC:243]	4
ENSG00000117868	2631,487	0,013634	0,335153	0,662532	0,963786	0,023066	0,032174	protein_coding	ESYT2	extended synaptotagmin 2 [Source:HGNC Symbol;Acc:HGNC:22211]	7
ENSG00000170421	10741,71	0,006925	0,334502	0,662532	0,965085	0,026517	0,096539	protein_coding	KRT8	keratin 8 [Source:HGNC Symbol;Acc:HGNC:6446]	12
ENSG00000197249	22071,95	0,002929	0,335017	0,662532	0,964057	0,026143	0,20948	protein_coding	SERPINA1	serpin family A member 1 [Source:HGNC Symbol;Acc:HGNC:8941]	14
ENSG00000166562	2661,654	0,010121	0,335085	0,662532	0,963923	0,025924	0,054505	protein_coding	SEC11C	SEC11 homolog C, signal peptidase complex subunit [Source:HGNC Symbol;Acc:HGNC:23400]	18
ENSG00000121892	2977,811	-0,0127	0,335771	0,662865	-0,96255	0,022786	-0,03227	protein_coding	PDS5A	PDS5 cohesin associated factor A [Source:HGNC Symbol;Acc:HGNC:29088]	4
ENSG00000103335	3295,259	0,010474	0,335897	0,662865	0,962303	0,02597	0,053371	protein_coding	PIEZO1	piezo type mechanosensitive ion channel component 1 [Source:HGNC Symbol;Acc:HGNC:28993]	16
ENSG00000196569	2416,411	0,006305	0,338207	0,664573	0,957714	0,02627	0,094886	protein_coding	LAMA2	laminin subunit alpha 2 [Source:HGNC Symbol;Acc:HGNC:6482]	6
ENSG00000196262	7808,71	0,01349	0,337934	0,664573	0,958255	0,021175	0,027238	protein_coding	PPIA	peptidylprolyl isomerase A [Source:HGNC Symbol;Acc:HGNC:9253]	7
ENSG00000149418	1696,751	0,007134	0,337678	0,664573	0,958762	0,026278	0,083079	protein_coding	ST14	ST14 transmembrane serine protease matriptase [Source:HGNC Symbol;Acc:HGNC:11344]	11
ENSG00000197324	5538,731	0,011134	0,338014	0,664573	0,958096	0,022066	0,031389	protein_coding	LRP10	LDL receptor related protein 10 [Source:HGNC Symbol;Acc:HGNC:14553]	14
ENSG00000172534	3756,738	-0,01221	0,337546	0,664573	-0,95903	0,024782	-0,04035	protein_coding	HCFC1	host cell factor C1 [Source:HGNC Symbol;Acc:HGNC:4839]	X
ENSG00000066279	1580,471	0,005632	0,342765	0,666133	0,948716	0,02624	0,105291	protein_coding	ASPM	assembly factor for spindle microtubules [Source:HGNC Symbol;Acc:HGNC:19048]	1
ENSG00000204262	17870,43	0,007588	0,342469	0,666133	0,949299	0,026231	0,075586	protein_coding	COL5A2	collagen type V alpha 2 chain [Source:HGNC Symbol;Acc:HGNC:2210]	2
ENSG00000144674	5462,46	0,01389	0,341531	0,666133	0,951144	0,022933	0,031141	protein_coding	GOLGA4	golgin A4 [Source:HGNC Symbol;Acc:HGNC:4427]	3
ENSG00000170801	2270,654	0,005667	0,340129	0,666133	0,95391	0,026246	0,105232	protein_coding	HTRA3	HtrA serine peptidase 3 [Source:HGNC Symbol;Acc:HGNC:30406]	4
ENSG00000124762	5464,373	0,00751	0,341634	0,666133	0,950941	0,026498	0,085064	protein_coding	CDKN1A	cyclin dependent kinase inhibitor 1A [Source:HGNC Symbol;Acc:HGNC:1784]	6
ENSG00000135218	2972,533	0,003508	0,340296	0,666133	0,95358	0,026159	0,170237	protein_coding	CD36	CD36 molecule [Source:HGNC Symbol;Acc:HGNC:1663]	7
ENSG00000118971	2421,584	0,00672	0,34015	0,666133	0,95387	0,026262	0,087524	protein_coding	CCND2	cyclin D2 [Source:HGNC Symbol;Acc:HGNC:1583]	12
ENSG00000066455	1624,651	0,012472	0,341183	0,666133	0,951831	0,023887	0,035906	protein_coding	GOLGA5	golgin A5 [Source:HGNC Symbol;Acc:HGNC:4428]	14
ENSG00000090060	3751,375	-0,01178	0,342613	0,666133	-0,94901	0,025165	-0,04283	protein_coding	PAPOLA	poly(A) polymerase alpha [Source:HGNC Symbol;Acc:HGNC:14981]	14
ENSG00000005339	3383,094	-0,01259	0,34219	0,666133	-0,94985	0,023748	-0,03513	protein_coding	CREBBP	CREB binding protein [Source:HGNC Symbol;Acc:HGNC:2348]	16
ENSG00000178951	2084,082	-0,01139	0,341102	0,666133	-0,95199	0,024033	-0,03814	protein_coding	ZBTB7A	zinc finger and BTB domain containing 7A [Source:HGNC Symbol;Acc:HGNC:18078]	19
ENSG00000105664	2994,399	-0,0015	0,340254	0,666133	-0,95366	0,026095	-0,37799	protein_coding	COMP	cartilage oligomeric matrix protein [Source:HGNC Symbol;Acc:HGNC:2227]	19
ENSG00000198886	245579,2	-0,00539	0,341014	0,666133	-0,95216	0,026232	-0,1106	protein_coding	MT-ND4	mitochondrially encoded NADH:ubiquinone oxidoreductase core subunit 4 [Source:HGNC Symbol;Acc:HGNC:7459]	MT
ENSG00000139842	1928,139	-0,01283	0,344414	0,668774	-0,94548	0,023042	-0,03234	protein_coding	CUL4A	cullin 4A [Source:HGNC Symbol;Acc:HGNC:2554]	13
ENSG00000118058	2601,591	-0,01223	0,3448	0,668959	-0,94472	0,024527	-0,03859	protein_coding	KMT2A	lysine methyltransferase 2A [Source:HGNC Symbol;Acc:HGNC:7132]	11
ENSG00000170606	4297,609	0,012631	0,345302	0,669368	0,943741	0,023249	0,033158	protein_coding	HSPA4	heat shock protein family A (Hsp70) member 4 [Source:HGNC Symbol;Acc:HGNC:5237]	5
ENSG00000173546	2198,183	0,002523	0,345595	0,669372	0,943168	0,026125	0,2362	protein_coding	CSPG4	chondroitin sulfate proteoglycan 4 [Source:HGNC Symbol;Acc:HGNC:2466]	15
ENSG00000145632	1661,519	-0,00924	0,346148	0,669879	-0,94209	0,025976	-0,05814	protein_coding	PLK2	polo like kinase 2 [Source:HGNC Symbol;Acc:HGNC:19699]	5
ENSG00000128591	2564,641	0,007187	0,347124	0,671203	0,940183	0,026212	0,078593	protein_coding	FLNC	filamin C [Source:HGNC Symbol;Acc:HGNC:3756]	7
ENSG00000105329	10523,34	0,008695	0,34786	0,672063	0,938747	0,026067	0,062418	protein_coding	TGFB1	transforming growth factor beta 1 [Source:HGNC Symbol;Acc:HGNC:11766]	19
ENSG00000100644	7467,295	-0,01362	0,34851	0,672189	-0,93748	0,026143	-0,0428	protein_coding	HIF1A	hypoxia inducible factor 1 subunit alpha [Source:HGNC Symbol;Acc:HGNC:4910]	14
ENSG00000125817	2500,781	0,012062	0,348455	0,672189	0,93759	0,024622	0,038995	protein_coding	CENPB	centromere protein B [Source:HGNC Symbol;Acc:HGNC:1852]	20
ENSG00000161202	2788,117	-0,01225	0,349242	0,672473	-0,93606	0,023605	-0,03455	protein_coding	DVL3	dishevelled segment polarity protein 3 [Source:HGNC Symbol;Acc:HGNC:3087]	3
ENSG00000165732	2951,307	0,01446	0,349052	0,672473	0,93643	0,024944	0,036114	protein_coding	DDX21	DExD-box helicase 21 [Source:HGNC Symbol;Acc:HGNC:2744]	10

ENSG00000163399	7797,606	-0,0127	0,349894	0,673165	-0,9348	0,022465	-0,0304	protein_coding	ATP1A1	ATPase Na+/K+ transporting subunit alpha 1 [Source:HGNC Symbol;Acc:HGNC:799]	1
ENSG00000140836	3921,32	-0,01113	0,350807	0,673795	-0,93303	0,024787	-0,04152	protein_coding	ZFHX3	zinc finger homeobox 3 [Source:HGNC Symbol;Acc:HGNC:777]	16
ENSG00000131051	4252,894	0,013238	0,350521	0,673795	0,933579	0,022156	0,029055	protein_coding	RBM39	RNA binding motif protein 39 [Source:HGNC Symbol;Acc:HGNC:15923]	20
ENSG00000182481	1887,459	0,007976	0,351244	0,674072	0,932179	0,026072	0,067198	protein_coding	KPNA2	karyopherin subunit alpha 2 [Source:HGNC Symbol;Acc:HGNC:6395]	17
ENSG00000139329	3288,608	-0,00781	0,352744	0,676019	-0,92928	0,026123	-0,06938	protein_coding	LUM	lumican [Source:HGNC Symbol;Acc:HGNC:6724]	12
ENSG00000126777	8489,982	0,011292	0,352846	0,676019	0,929083	0,02544	0,04477	protein_coding	KTN1	kinectin 1 [Source:HGNC Symbol;Acc:HGNC:6467]	14
ENSG00000116731	1641	0,011745	0,359261	0,678168	0,916774	0,024054	0,036246	protein_coding	PRDM2	PR/SET domain 2 [Source:HGNC Symbol;Acc:HGNC:9347]	1
ENSG00000132718	1963,542	-0,01028	0,354303	0,678168	-0,92628	0,025728	-0,05008	protein_coding	SYT11	synaptotagmin 11 [Source:HGNC Symbol;Acc:HGNC:19239]	1
ENSG00000143248	2299,754	-0,00307	0,358751	0,678168	-0,91775	0,026126	-0,18126	protein_coding	RGS5	regulator of G protein signaling 5 [Source:HGNC Symbol;Acc:HGNC:10001]	1
ENSG00000173706	4125,847	0,009895	0,360009	0,678168	0,915348	0,025547	0,049287	protein_coding	HEG1	heart development protein with EGF like domains 1 [Source:HGNC Symbol;Acc:HGNC:29227]	3
ENSG00000163714	1978,165	0,01033	0,356679	0,678168	0,921712	0,025028	0,044375	protein_coding	U2SURP	U2 snRNP associated SURP domain containing [Source:HGNC Symbol;Acc:HGNC:30855]	3
ENSG00000113141	2425,539	0,011457	0,355052	0,678168	0,924835	0,024591	0,03948	protein_coding	IK	IK cytokine [Source:HGNC Symbol;Acc:HGNC:5958]	5
ENSG00000182095	4209,929	0,012066	0,356794	0,678168	0,921491	0,024886	0,039524	protein_coding	TNRC18	trinucleotide repeat containing 18 [Source:HGNC Symbol;Acc:HGNC:11962]	7
ENSG00000134013	6896,452	0,008001	0,359185	0,678168	0,91692	0,026057	0,065683	protein_coding	LOXL2	lysyl oxidase like 2 [Source:HGNC Symbol;Acc:HGNC:6666]	8
ENSG00000104331	2776,222	0,012535	0,358604	0,678168	0,918029	0,023804	0,034225	protein_coding	BPNT2	3'(2'), 5'-bisphosphate nucleotidase 2 [Source:HGNC Symbol;Acc:HGNC:26019]	8
ENSG00000095303	4405,897	-0,00456	0,359894	0,678168	-0,91557	0,026161	-0,12116	protein_coding	PTGS1	prostaglandin-endoperoxide synthase 1 [Source:HGNC Symbol;Acc:HGNC:9604]	9
ENSG00000095787	2984,8	0,012009	0,359768	0,678168	0,915807	0,0236	0,034109	protein_coding	WAC	WW domain containing adaptor with coiled-coil [Source:HGNC Symbol;Acc:HGNC:17327]	10
ENSG00000110048	1803,816	-0,01178	0,35965	0,678168	-0,91603	0,025542	-0,04338	protein_coding	OSBP	oxysterol binding protein [Source:HGNC Symbol;Acc:HGNC:8503]	11
ENSG00000127334	1654,339	-0,01072	0,355329	0,678168	-0,9243	0,024967	-0,04314	protein_coding	DYRK2	dual specificity tyrosine phosphorylation regulated kinase 2 [Source:HGNC Symbol;Acc:HGNC:3093]	12
ENSG00000139697	2691,524	0,012145	0,357375	0,678168	0,920379	0,024304	0,036805	protein_coding	SBNO1	strawberry notch homolog 1 [Source:HGNC Symbol;Acc:HGNC:22973]	12
ENSG00000140262	2266,072	-0,01173	0,358268	0,678168	-0,91867	0,023575	-0,03449	protein_coding	TCF12	transcription factor 12 [Source:HGNC Symbol;Acc:HGNC:11623]	15
ENSG00000129925	1750,769	-0,01167	0,355617	0,678168	-0,92375	0,024017	-0,03651	protein_coding	PGAP6	post-glycosylphosphatidylinositol attachment to proteins 6 [Source:HGNC Symbol;Acc:HGNC:17205]	16
ENSG00000103222	1780,337	-0,01189	0,360157	0,678168	-0,91507	0,025506	-0,04285	protein_coding	ABCC1	ATP binding cassette subfamily C member 1 [Source:HGNC Symbol;Acc:HGNC:51]	16
ENSG00000130309	2685,768	0,011135	0,3571	0,678168	0,920904	0,024917	0,041674	protein_coding	COLGALT1	collagen beta(1-O)galactosyltransferase 1 [Source:HGNC Symbol;Acc:HGNC:26182]	19
ENSG00000196961	2297,061	0,012047	0,355586	0,678168	0,923808	0,023799	0,035101	protein_coding	AP2A1	adaptor related protein complex 2 subunit alpha 1 [Source:HGNC Symbol;Acc:HGNC:561]	19
ENSG00000183337	1778,904	-0,01035	0,359608	0,678168	-0,91611	0,02496	-0,04364	protein_coding	BCOR	BCL6 corepressor [Source:HGNC Symbol;Acc:HGNC:20893]	X
ENSG00000198938	73121,88	-0,00604	0,357904	0,678168	-0,91937	0,025381	-0,06569	protein_coding	MT-CO3	mitochondrially encoded cytochrome c oxidase III [Source:HGNC Symbol;Acc:HGNC:7422]	MT
ENSG00000204120	2776,923	-0,01195	0,362364	0,68086	-0,91087	0,022891	-0,0316	protein_coding	GIGYF2	GRB10 interacting GYF protein 2 [Source:HGNC Symbol;Acc:HGNC:11960]	2
ENSG00000109606	2373,603	0,012189	0,361941	0,68086	0,911674	0,022706	0,030828	protein_coding	DHX15	DEAH-box helicase 15 [Source:HGNC Symbol;Acc:HGNC:2738]	4
ENSG00000137809	10867,13	-0,00676	0,362474	0,68086	-0,91066	0,026125	-0,07808	protein_coding	ITGA11	integrin subunit alpha 11 [Source:HGNC Symbol;Acc:HGNC:6136]	15
ENSG00000088930	2740,448	0,010505	0,363357	0,681961	0,908987	0,022244	0,030915	protein_coding	XRN2	5'-3' exoribonuclease 2 [Source:HGNC Symbol;Acc:HGNC:12836]	20
ENSG00000171951	11177,71	-0,00841	0,363666	0,681984	-0,9084	0,025925	-0,06003	protein_coding	SCG2	secretogranin II [Source:HGNC Symbol;Acc:HGNC:10575]	2
ENSG00000082781	6839,48	0,007881	0,364111	0,682043	0,90756	0,025681	0,059453	protein_coding	ITGB5	integrin subunit beta 5 [Source:HGNC Symbol;Acc:HGNC:6160]	3
ENSG00000166986	2603,261	-0,01087	0,36429	0,682043	-0,90722	0,024283	-0,03835	protein_coding	MARS1	methionyl-tRNA synthetase 1 [Source:HGNC Symbol;Acc:HGNC:6898]	12
ENSG00000139352	2250,145	-0,00353	0,364634	0,682055	-0,90657	0,026132	-0,1543	protein_coding	ASCL1	achaete-scute family bHLH transcription factor 1 [Source:HGNC Symbol;Acc:HGNC:738]	12
ENSG00000100811	2281,441	-0,01155	0,364889	0,682055	-0,90609	0,022098	-0,02944	protein_coding	YY1	YY1 transcription factor [Source:HGNC Symbol;Acc:HGNC:12856]	14
ENSG00000182871	11063,51	0,00488	0,365568	0,68277	0,904806	0,026157	0,110747	protein_coding	COL18A1	collagen type XVIII alpha 1 chain [Source:HGNC Symbol;Acc:HGNC:2195]	21
ENSG00000144810	3202,699	-0,00679	0,366953	0,683527	-0,9022	0,025327	-0,05949	protein_coding	COL8A1	collagen type VIII alpha 1 chain [Source:HGNC Symbol;Acc:HGNC:2215]	3
ENSG00000151914	18487,1	-0,00413	0,368053	0,683527	-0,90013	0,023436	-0,04482	protein_coding	DST	dystonin [Source:HGNC Symbol;Acc:HGNC:1090]	6
ENSG00000134294	6749,885	-0,00989	0,366954	0,683527	-0,90219	0,023697	-0,03704	protein_coding	SLC38A2	solute carrier family 38 member 2 [Source:HGNC Symbol;Acc:HGNC:13448]	12
ENSG00000103994	2744,205	-0,01164	0,367285	0,683527	-0,90157	0,02302	-0,03205	protein_coding	ZNF106	zinc finger protein 106 [Source:HGNC Symbol;Acc:HGNC:12886]	15
ENSG00000140807	5541,498	0,00343	0,367132	0,683527	0,901858	0,026128	0,157294	protein_coding	NKD1	NKD inhibitor of WNT signaling pathway 1 [Source:HGNC Symbol;Acc:HGNC:17045]	16
ENSG00000173812	5896,072	0,012748	0,36777	0,683527	0,900659	0,021523	0,026854	protein_coding	EIF1	eukaryotic translation initiation factor 1 [Source:HGNC Symbol;Acc:HGNC:3249]	17
ENSG00000100234	16659,4	-0,00456	0,367542	0,683527	-0,90109	0,026143	-0,11713	protein_coding	TIMP3	TIMP metallopeptidase inhibitor 3 [Source:HGNC Symbol;Acc:HGNC:11822]	22
ENSG00000144224	2986,041	0,012325	0,369993	0,683636	0,896486	0,02256	0,029763	protein_coding	UBXN4	UBX domain protein 4 [Source:HGNC Symbol;Acc:HGNC:14860]	2
ENSG00000181789	5171,927	0,011873	0,369165	0,683636	0,898039	0,021715	0,027875	protein_coding	COPG1	COPI coat complex subunit gamma 1 [Source:HGNC Symbol;Acc:HGNC:2236]	3
ENSG00000112977	4088,202	-0,00264	0,370319	0,683636	-0,89588	0,021608	-0,03401	protein_coding	DAP	death associated protein [Source:HGNC Symbol;Acc:HGNC:2672]	5
ENSG00000112701	1821,106	0,011137	0,370399	0,683636	0,895725	0,023624	0,034638	protein_coding	SENP6	SUMO specific peptidase 6 [Source:HGNC Symbol;Acc:HGNC:20944]	6
ENSG00000136888	1891,322	0,01083	0,368992	0,683636	0,898363	0,023418	0,03434	protein_coding	ATP6V1G1	ATPase H+ transporting V1 subunit G1 [Source:HGNC Symbol;Acc:HGNC:864]	9
ENSG00000135480	1645,029	0,003107	0,369426	0,683636	0,897549	0,026117	0,17098	protein_coding	KRT7	keratin 7 [Source:HGNC Symbol;Acc:HGNC:6445]	12
ENSG00000177200	2467,937	0,010466	0,370489	0,683636	0,895558	0,024865	0,041793	protein_coding	CHD9	chromodomain helicase DNA binding protein 9 [Source:HGNC Symbol;Acc:HGNC:25701]	16
ENSG00000105669	1599,352	0,010513	0,36937	0,683636	0,897654	0,024449	0,039439	protein_coding	COPE	COPI coat complex subunit epsilon [Source:HGNC Symbol;Acc:HGNC:2234]	19

ENSG00000113194	1601,996	0,011122	0,371694	0,684734	0,893304	0,023424	0,033787	protein_coding	FAF2	Fas associated factor family member 2 [Source:HGNC Symbol;Acc:HGNC:24666]	5
ENSG00000152894	3735,456	0,010233	0,371976	0,684734	0,892778	0,024857	0,042171	protein_coding	PTPRK	protein tyrosine phosphatase receptor type K [Source:HGNC Symbol;Acc:HGNC:9674]	6
ENSG00000174231	7200,269	0,011547	0,371869	0,684734	0,892979	0,024742	0,038659	protein_coding	PRPF8	pre-mRNA processing factor 8 [Source:HGNC Symbol;Acc:HGNC:17340]	17
ENSG00000188554	3179,026	-0,00718	0,372674	0,685469	-0,89148	0,02322	-0,03831	protein_coding	NBR1	NBR1 autophagy cargo receptor [Source:HGNC Symbol;Acc:HGNC:6746]	17
ENSG00000263740	12381,09	-0,00477	0,37545	0,685659	-0,88631	0,026096	-0,10555	misc_RNA	RN7SL4P	RNA, 7SL, cytoplasmic 4, pseudogene [Source:HGNC Symbol;Acc:HGNC:10039]	3
ENSG00000184014	1786,646	-0,01008	0,375459	0,685659	-0,88629	0,024613	-0,04077	protein_coding	DENND5A	DENN domain containing 5A [Source:HGNC Symbol;Acc:HGNC:19344]	11
ENSG00000152558	2742,301	0,010486	0,373521	0,685659	0,889898	0,024857	0,041419	protein_coding	TMEM123	transmembrane protein 123 [Source:HGNC Symbol;Acc:HGNC:30138]	11
ENSG00000197930	1641,487	0,010619	0,374579	0,685659	0,88793	0,024869	0,041103	protein_coding	ERO1A	endoplasmic reticulum oxidoreductase 1 alpha [Source:HGNC Symbol;Acc:HGNC:13280]	14
ENSG00000078304	2102,603	0,011059	0,374747	0,685659	0,887617	0,02478	0,039633	protein_coding	PPP2R5C	protein phosphatase 2 regulatory subunit B'gamma [Source:HGNC Symbol;Acc:HGNC:9311]	14
ENSG00000067141	2940,246	-0,01084	0,374604	0,685659	-0,88788	0,021822	-0,02865	protein_coding	NEO1	neogenin 1 [Source:HGNC Symbol;Acc:HGNC:7754]	15
ENSG00000170832	1625,187	-0,01971	0,375441	0,685659	-0,88633	0,027127	-0,03298	protein_coding	USP32	ubiquitin specific peptidase 32 [Source:HGNC Symbol;Acc:HGNC:19143]	17
ENSG00000184640	7404,808	0,010691	0,374153	0,685659	0,888722	0,024715	0,040103	protein_coding	SEPTIN9	septin 9 [Source:HGNC Symbol;Acc:HGNC:7323]	17
ENSG00000105281	6116,939	0,011526	0,37367	0,685659	0,88962	0,02357	0,033638	protein_coding	SLC1A5	solute carrier family 1 member 5 [Source:HGNC Symbol;Acc:HGNC:10943]	19
ENSG00000137177	1940,696	0,008957	0,375811	0,685758	0,885641	0,025568	0,05147	protein_coding	KIF13A	kinesin family member 13A [Source:HGNC Symbol;Acc:HGNC:14566]	6
ENSG00000058063	1728,669	0,009089	0,376584	0,686468	0,884207	0,024298	0,040972	protein_coding	ATP11B	ATPase phospholipid transporting 11B (putative) [Source:HGNC Symbol;Acc:HGNC:13553]	3
ENSG00000101745	2252,006	-0,01142	0,376797	0,686468	-0,88381	0,023648	-0,0339	protein_coding	ANKRD12	ankyrin repeat domain 12 [Source:HGNC Symbol;Acc:HGNC:29135]	18
ENSG00000173905	5180,461	0,01044	0,378298	0,686581	0,881036	0,024695	0,040205	protein_coding	GOLIM4	golgi integral membrane protein 4 [Source:HGNC Symbol;Acc:HGNC:15448]	3
ENSG00000165678	2484,006	0,010286	0,377425	0,686581	0,882651	0,024907	0,041866	protein_coding	GHITM	growth hormone inducible transmembrane protein [Source:HGNC Symbol;Acc:HGNC:17281]	10
ENSG00000135387	6934,744	0,012032	0,378309	0,686581	0,881017	0,019957	0,023274	protein_coding	CAPRIN1	cell cycle associated protein 1 [Source:HGNC Symbol;Acc:HGNC:6743]	11
ENSG00000123384	22439,73	0,008067	0,378153	0,686581	0,881304	0,025851	0,059188	protein_coding	LRP1	LDL receptor related protein 1 [Source:HGNC Symbol;Acc:HGNC:6692]	12
ENSG00000259001	62966,04	-0,00669	0,37835	0,686581	-0,88094	0,026034	-0,07358	lncRNA	AL355075.4	ribonuclease P RNA component H1	14
ENSG00000204469	8287,168	-0,01197	0,379765	0,686665	-0,87833	0,02153	-0,02671	protein_coding	PRRC2A	proline rich coiled-coil 2A [Source:HGNC Symbol;Acc:HGNC:13918]	6
ENSG00000166033	6648,303	0,008202	0,37949	0,686665	0,878837	0,025802	0,057515	protein_coding	HTRA1	HtrA serine peptidase 1 [Source:HGNC Symbol;Acc:HGNC:9476]	10
ENSG00000123104	3893,823	-0,00828	0,379889	0,686665	-0,8781	0,022006	-0,03105	protein_coding	ITPR2	inositol 1,4,5-trisphosphate receptor type 2 [Source:HGNC Symbol;Acc:HGNC:6181]	12
ENSG00000086548	6697,436	0,002188	0,37936	0,686665	0,879076	0,026099	0,237332	protein_coding	CEACAM6	CEA cell adhesion molecule 6 [Source:HGNC Symbol;Acc:HGNC:1818]	19
ENSG00000167378	2070,348	-0,01043	0,379158	0,686665	-0,87945	0,024351	-0,03832	protein_coding	IRGQ	immunity related GTPase Q [Source:HGNC Symbol;Acc:HGNC:24868]	19
ENSG00000100614	1615,916	0,011714	0,380498	0,687226	0,876979	0,023105	0,031365	protein_coding	PPM1A	protein phosphatase, Mg2+/Mn2+ dependent 1A [Source:HGNC Symbol;Acc:HGNC:9275]	14
ENSG00000146223	1919,162	0,010657	0,38082	0,687269	0,876386	0,024072	0,036447	protein_coding	RPL7L1	ribosomal protein L7 like 1 [Source:HGNC Symbol;Acc:HGNC:21370]	6
ENSG00000155304	1564,557	-0,00965	0,381496	0,687949	-0,87514	0,023732	-0,03649	protein_coding	HSPA13	heat shock protein family A (Hsp70) member 13 [Source:HGNC Symbol;Acc:HGNC:11375]	21
ENSG00000104371	4021,874	-0,00052	0,384568	0,692945	-0,86951	0,026083	-0,82503	protein_coding	DKK4	dickkopf WNT signaling pathway inhibitor 4 [Source:HGNC Symbol;Acc:HGNC:2894]	8
ENSG00000112118	1863,224	-0,00925	0,386687	0,69513	-0,86564	0,024131	-0,0388	protein_coding	MCM3	minichromosome maintenance complex component 3 [Source:HGNC Symbol;Acc:HGNC:6945]	6
ENSG00000012660	2142,699	0,01036	0,386371	0,69513	0,866217	0,02415	0,036897	protein_coding	ELOVL5	ELOVL fatty acid elongase 5 [Source:HGNC Symbol;Acc:HGNC:21308]	6
ENSG00000161671	1956,27	-0,01129	0,386566	0,69513	-0,86586	0,02443	-0,03654	protein_coding	EMC10	ER membrane protein complex subunit 10 [Source:HGNC Symbol;Acc:HGNC:27609]	19
ENSG00000174807	2135,39	0,002979	0,387413	0,695519	0,864319	0,026102	0,168345	protein_coding	CD248	CD248 molecule [Source:HGNC Symbol;Acc:HGNC:18219]	11
ENSG00000111530	3067,767	0,011386	0,387508	0,695519	0,864145	0,023088	0,031207	protein_coding	CAND1	cullin associated and neddylation dissociated 1 [Source:HGNC Symbol;Acc:HGNC:30688]	12
ENSG00000197622	1594,91	0,0101	0,389555	0,696175	0,860425	0,024555	0,039227	protein_coding	CDC42SE1	CDC42 small effector 1 [Source:HGNC Symbol;Acc:HGNC:17719]	1
ENSG00000173193	1785,942	-0,00872	0,39062	0,696175	-0,85849	0,025489	-0,05002	protein_coding	PARP14	poly(ADP-ribose) polymerase family member 14 [Source:HGNC Symbol;Acc:HGNC:29232]	3
ENSG00000084093	1966,155	0,009542	0,390704	0,696175	0,858342	0,025082	0,043779	protein_coding	REST	RE1 silencing transcription factor [Source:HGNC Symbol;Acc:HGNC:9966]	4
ENSG00000171564	2019,041	0,002283	0,388211	0,696175	0,862867	0,026116	0,233773	protein_coding	FGB	fibrinogen beta chain [Source:HGNC Symbol;Acc:HGNC:3662]	4
ENSG00000087116	5167,893	0,004279	0,390899	0,696175	0,857989	0,026095	0,113947	protein_coding	ADAMTS2	ADAM metallopeptidase with thrombospondin type 1 motif 2 [Source:HGNC Symbol;Acc:HGNC:218]	5
ENSG00000001629	1876,283	-0,01436	0,390247	0,696175	-0,85917	0,024999	-0,03348	protein_coding	ANKIB1	ankyrin repeat and IBR domain containing 1 [Source:HGNC Symbol;Acc:HGNC:22215]	7
ENSG00000096746	2126,43	0,009124	0,389057	0,696175	0,861328	0,025153	0,045711	protein_coding	HNRNPH3	heterogeneous nuclear ribonucleoprotein H3 [Source:HGNC Symbol;Acc:HGNC:5043]	10
ENSG00000139218	2826,847	0,011859	0,388891	0,696175	0,86163	0,021452	0,026098	protein_coding	SCAF11	SR-related CTD associated factor 11 [Source:HGNC Symbol;Acc:HGNC:10784]	12
ENSG00000168175	2714,585	-0,01094	0,38913	0,696175	-0,8612	0,023703	-0,03386	protein_coding	MAPK1IP1L	mitogen-activated protein kinase 1 interacting protein 1 like [Source:HGNC Symbol;Acc:HGNC:19840]	14
ENSG00000183762	2034,141	-0,00712	0,389874	0,696175	-0,85985	0,025916	-0,06549	protein_coding	KREMEN1	kringle containing transmembrane protein 1 [Source:HGNC Symbol;Acc:HGNC:17550]	22
ENSG00000198176	1839,681	0,009837	0,392662	0,698775	0,854799	0,024271	0,037958	protein_coding	TFDP1	transcription factor Dp-1 [Source:HGNC Symbol;Acc:HGNC:11749]	13
ENSG00000108100	1590,298	0,010596	0,394295	0,701138	0,851854	0,024305	0,036607	protein_coding	CCNY	cyclin Y [Source:HGNC Symbol;Acc:HGNC:23354]	10
ENSG00000128731	3320,008	-0,01009	0,394857	0,701596	-0,85084	0,023908	-0,03556	protein_coding	HERC2	HECT and RLD domain containing E3 ubiquitin protein ligase 2 [Source:HGNC Symbol;Acc:HGNC:4868]	15
ENSG00000175203	1782,812	-0,01082	0,395434	0,702079	-0,8498	0,023611	-0,03323	protein_coding	DCTN2	dynactin subunit 2 [Source:HGNC Symbol;Acc:HGNC:2712]	12
ENSG00000144747	2038,59	-0,0135	0,397774	0,703066	-0,8456	0,023139	-0,02856	protein_coding	TMF1	TATA element modulatory factor 1 [Source:HGNC Symbol;Acc:HGNC:11870]	3
ENSG00000106624	9379,437	0,004538	0,39792	0,703066	0,845341	0,026074	0,103994	protein_coding	AEBP1	AE binding protein 1 [Source:HGNC Symbol;Acc:HGNC:303]	7
ENSG00000128564	5978,68	0,005731	0,397423	0,703066	0,846232	0,026009	0,080332	protein_coding	VGF	VGF nerve growth factor inducible [Source:HGNC Symbol;Acc:HGNC:12684]	7

ENSG00000078674	3907,781	0,012013	0,397781	0,703066	0,84559	0,020011	0,022422	protein_coding	PCM1	pericentriolar material 1 [Source:HGNC Symbol;Acc:HGNC:8727]	8
ENSG00000174243	1998,723	-0,00986	0,397911	0,703066	-0,84536	0,024728	-0,04002	protein_coding	DDX23	DEAD-box helicase 23 [Source:HGNC Symbol;Acc:HGNC:17347]	12
ENSG00000165502	2203,027	0,010599	0,398129	0,703066	0,844967	0,023825	0,034199	protein_coding	RPL36AL	ribosomal protein L36a like [Source:HGNC Symbol;Acc:HGNC:10346]	14
ENSG00000198840	12150,77	-0,0063	0,397395	0,703066	-0,84628	0,025957	-0,07238	protein_coding	MT-ND3	mitochondrially encoded NADH:ubiquinone oxidoreductase core subunit 3 [Source:HGNC Symbol;Acc:HGNC:7458]	MT
ENSG00000112308	2015,519	0,010356	0,399058	0,703888	0,843304	0,024465	0,037466	protein_coding	C6orf62	chromosome 6 open reading frame 62 [Source:HGNC Symbol;Acc:HGNC:20998]	6
ENSG00000103342	3479,024	-0,01225	0,399303	0,703888	-0,84287	0,022906	-0,029	protein_coding	GSPT1	G1 to S phase transition 1 [Source:HGNC Symbol;Acc:HGNC:4621]	16
ENSG00000170004	2851,658	0,008969	0,399512	0,703888	0,842493	0,025411	0,047429	protein_coding	CHD3	chromodomain helicase DNA binding protein 3 [Source:HGNC Symbol;Acc:HGNC:1918]	17
ENSG00000173821	9125,488	-0,01089	0,401224	0,706363	-0,83944	0,023377	-0,03185	protein_coding	RNF213	ring finger protein 213 [Source:HGNC Symbol;Acc:HGNC:14539]	17
ENSG00000172493	2436,037	-0,0116	0,402156	0,707462	-0,83778	0,024908	-0,03693	protein_coding	AFF1	AF4/FMR2 family member 1 [Source:HGNC Symbol;Acc:HGNC:7135]	4
ENSG00000134851	1875,207	0,007931	0,402926	0,707736	0,836406	0,025326	0,05014	protein_coding	TMEM165	transmembrane protein 165 [Source:HGNC Symbol;Acc:HGNC:30760]	4
ENSG00000132294	2391,149	-0,01286	0,402785	0,707736	-0,83666	0,025194	-0,03573	protein_coding	EFR3A	EFR3 homolog A [Source:HGNC Symbol;Acc:HGNC:28970]	8
ENSG00000221978	1857,213	-0,00859	0,404166	0,708082	-0,8342	0,025477	-0,04897	protein_coding	CCNL2	cyclin L2 [Source:HGNC Symbol;Acc:HGNC:20570]	1
ENSG00000082898	2804,944	0,009337	0,405278	0,708082	0,832233	0,025328	0,044913	protein_coding	XPO1	exportin 1 [Source:HGNC Symbol;Acc:HGNC:12825]	2
ENSG00000163069	1893,223	0,008203	0,404358	0,708082	0,833863	0,024641	0,042879	protein_coding	SGCB	sarcoglycan beta [Source:HGNC Symbol;Acc:HGNC:10806]	4
ENSG00000083168	2682,604	-0,01033	0,40505	0,708082	-0,83264	0,023163	-0,03148	protein_coding	KAT6A	lysine acetyltransferase 6A [Source:HGNC Symbol;Acc:HGNC:13013]	8
ENSG00000056586	2720,512	-0,01065	0,404607	0,708082	-0,83342	0,023107	-0,03097	protein_coding	RC3H2	ring finger and CCCH-type domains 2 [Source:HGNC Symbol;Acc:HGNC:21461]	9
ENSG00000123240	2994,73	0,011552	0,405007	0,708082	0,832712	0,025447	0,039517	protein_coding	OPTN	optineurin [Source:HGNC Symbol;Acc:HGNC:17142]	10
ENSG00000205730	3333,716	0,007497	0,405057	0,708082	0,832624	0,024488	0,043431	protein_coding	ITPRIPL2	ITPRIP like 2 [Source:HGNC Symbol;Acc:HGNC:27257]	16
ENSG00000270103	1910,851	-0,00201	0,405991	0,70879	-0,83097	0,026081	-0,22519	lncRNA	AL360012.	RNA, U11 small nuclear	1
ENSG00000163814	2270,068	-0,00759	0,406524	0,708824	-0,83003	0,025044	-0,04808	protein_coding	CDCP1	CUB domain containing protein 1 [Source:HGNC Symbol;Acc:HGNC:24357]	3
ENSG00000131507	1929,719	0,010542	0,406774	0,708824	0,829583	0,022949	0,030413	protein_coding	NDFIP1	Nedd4 family interacting protein 1 [Source:HGNC Symbol;Acc:HGNC:17592]	5
ENSG00000151348	1811,107	0,010216	0,406934	0,708824	0,829301	0,024812	0,038957	protein_coding	EXT2	exostosin glycosyltransferase 2 [Source:HGNC Symbol;Acc:HGNC:3513]	11
ENSG00000104517	3458,24	-0,0117	0,408788	0,711513	-0,82603	0,021234	-0,02462	protein_coding	UBR5	ubiquitin protein ligase E3 component n-recognin 5 [Source:HGNC Symbol;Acc:HGNC:16806]	8
ENSG00000137507	2173,39	0,00506	0,409098	0,711516	0,825481	0,026011	0,087616	protein_coding	LRRC32	leucine rich repeat containing 32 [Source:HGNC Symbol;Acc:HGNC:4161]	11
ENSG00000069849	2115,447	-0,0104	0,410786	0,713912	-0,82251	0,023113	-0,03087	protein_coding	ATP1B3	ATPase Na+/K+ transporting subunit beta 3 [Source:HGNC Symbol;Acc:HGNC:806]	3
ENSG00000127603	20674,2	-0,01482	0,413086	0,715124	-0,81848	0,024608	-0,03016	protein_coding	MACF1	microtubule actin crosslinking factor 1 [Source:HGNC Symbol;Acc:HGNC:13664]	1
ENSG00000009307	8949,246	0,008955	0,413034	0,715124	0,818567	0,021181	0,025964	protein_coding	CSDE1	cold shock domain containing E1 [Source:HGNC Symbol;Acc:HGNC:29905]	1
ENSG00000198836	1942,479	0,011435	0,412827	0,715124	0,818929	0,023473	0,030772	protein_coding	OPA1	OPA1 mitochondrial dynamin like GTPase [Source:HGNC Symbol;Acc:HGNC:8140]	3
ENSG00000172466	2052,598	-0,01035	0,413349	0,715124	-0,81801	0,023393	-0,03177	protein_coding	ZNF24	zinc finger protein 24 [Source:HGNC Symbol;Acc:HGNC:13032]	18
ENSG00000105388	15117,1	0,002518	0,412781	0,715124	0,819009	0,026073	0,17107	protein_coding	CEACAM5	CEA cell adhesion molecule 5 [Source:HGNC Symbol;Acc:HGNC:1817]	19
ENSG00000101825	7893,642	-0,00723	0,411963	0,715124	-0,82044	0,025677	-0,05713	protein_coding	MXRA5	matrix remodeling associated 5 [Source:HGNC Symbol;Acc:HGNC:7539]	X
ENSG00000136153	5216,429	0,00817	0,413724	0,715152	0,817359	0,025038	0,045345	protein_coding	LMO7	LIM domain 7 [Source:HGNC Symbol;Acc:HGNC:6646]	13
ENSG00000063245	2522,121	-0,009	0,413986	0,715152	-0,8169	0,023345	-0,03326	protein_coding	EPN1	epsin 1 [Source:HGNC Symbol;Acc:HGNC:21604]	19
ENSG00000151327	1807,926	0,010797	0,415066	0,716479	0,815011	0,023145	0,030259	protein_coding	FAM177A1	family with sequence similarity 177 member A1 [Source:HGNC Symbol;Acc:HGNC:19829]	14
ENSG00000116209	4063,655	-0,01016	0,416074	0,716722	-0,81325	0,020558	-0,02356	protein_coding	TMEM59	transmembrane protein 59 [Source:HGNC Symbol;Acc:HGNC:1239]	1
ENSG00000164506	2038,768	-0,0094	0,416472	0,716722	-0,81256	0,023824	-0,03467	protein_coding	STXBP5	syntaxin binding protein 5 [Source:HGNC Symbol;Acc:HGNC:19665]	6
ENSG00000186660	1646,142	0,009435	0,415901	0,716722	0,813554	0,024336	0,037216	protein_coding	ZFP91	ZFP91 zinc finger protein, atypical E3 ubiquitin ligase [Source:HGNC Symbol;Acc:HGNC:14983]	11
ENSG00000066084	1748,019	-0,01061	0,417076	0,716722	-0,81151	0,023084	-0,03012	protein_coding	DIP2B	disco interacting protein 2 homolog B [Source:HGNC Symbol;Acc:HGNC:29284]	12
ENSG00000182253	1887,067	0,005825	0,416705	0,716722	0,812151	0,025775	0,068337	protein_coding	SYNM	synemin [Source:HGNC Symbol;Acc:HGNC:24466]	15
ENSG00000105323	6204,293	0,010659	0,416819	0,716722	0,811953	0,02259	0,028486	protein_coding	HNRNPUL1	heterogeneous nuclear ribonucleoprotein U like 1 [Source:HGNC Symbol;Acc:HGNC:17011]	19
ENSG00000087053	1634,76	0,008977	0,417828	0,71748	0,810195	0,024848	0,041211	protein_coding	MTMR2	myotubularin related protein 2 [Source:HGNC Symbol;Acc:HGNC:7450]	11
ENSG00000137312	2180,961	-0,01017	0,418931	0,718301	-0,80828	0,023076	-0,03045	protein_coding	FLOT1	flotillin 1 [Source:HGNC Symbol;Acc:HGNC:3757]	6
ENSG00000110075	2141,919	0,013681	0,418902	0,718301	0,808327	0,022593	0,025841	protein_coding	PPP6R3	protein phosphatase 6 regulatory subunit 3 [Source:HGNC Symbol;Acc:HGNC:1173]	11
ENSG00000133226	2789,235	-0,00983	0,420496	0,719509	-0,80556	0,023928	-0,03417	protein_coding	SRRM1	serine and arginine repetitive matrix 1 [Source:HGNC Symbol;Acc:HGNC:16638]	1
ENSG00000162434	4711,217	0,008797	0,420573	0,719509	0,805427	0,02404	0,036444	protein_coding	JAK1	Janus kinase 1 [Source:HGNC Symbol;Acc:HGNC:6190]	1
ENSG00000152332	2786,484	0,00857	0,420226	0,719509	0,80603	0,025047	0,043599	protein_coding	UHMK1	U2AF homology motif kinase 1 [Source:HGNC Symbol;Acc:HGNC:19683]	1
ENSG00000047315	1990,39	0,011348	0,421346	0,720296	0,804088	0,023739	0,031227	protein_coding	POLR2B	RNA polymerase II subunit B [Source:HGNC Symbol;Acc:HGNC:9188]	4
ENSG00000205413	1587,313	0,008499	0,423296	0,722555	0,800716	0,025109	0,044044	protein_coding	SAMD9	sterile alpha motif domain containing 9 [Source:HGNC Symbol;Acc:HGNC:1348]	7
ENSG00000119396	1772,508	0,009019	0,423242	0,722555	0,80081	0,023861	0,034938	protein_coding	RAB14	RAB14, member RAS oncogene family [Source:HGNC Symbol;Acc:HGNC:16524]	9
ENSG00000135968	1818,016	0,010386	0,42587	0,722661	0,796278	0,024584	0,035889	protein_coding	GCC2	GRIP and coiled-coil domain containing 2 [Source:HGNC Symbol;Acc:HGNC:23218]	2
ENSG00000082701	2031,554	0,008572	0,425778	0,722661	0,796437	0,023633	0,034354	protein_coding	GSK3B	glycogen synthase kinase 3 beta [Source:HGNC Symbol;Acc:HGNC:4617]	3
ENSG00000185728	1743,441	-0,00444	0,425384	0,722661	-0,79712	0,021437	-0,02875	protein_coding	YTHDF3	YTH N6-methyladenosine RNA binding protein 3 [Source:HGNC Symbol;Acc:HGNC:26465]	8

ENSG00000120694	3567,096	0,00902	0,424771	0,722661	0,798172	0,025063	0,042005	protein_coding	HSPH1	heat shock protein family H (Hsp110) member 1 [Source:HGNC Symbol;Acc:HGNC:16969]	13
ENSG00000100852	4700,918	0,010114	0,424902	0,722661	0,797946	0,024269	0,034947	protein_coding	ARHGAP5	Rho GTPase activating protein 5 [Source:HGNC Symbol;Acc:HGNC:675]	14
ENSG00000124422	6068,047	-0,00749	0,425654	0,722661	-0,79665	0,019471	-0,02181	protein_coding	USP22	ubiquitin specific peptidase 22 [Source:HGNC Symbol;Acc:HGNC:12621]	17
ENSG00000105223	2849,505	-0,00783	0,424823	0,722661	-0,79808	0,025313	-0,04805	protein_coding	PLD3	phospholipase D family member 3 [Source:HGNC Symbol;Acc:HGNC:17158]	19
ENSG00000101161	2394,945	0,009451	0,42512	0,722661	0,79757	0,022121	0,027632	protein_coding	PRPF6	pre-mRNA processing factor 6 [Source:HGNC Symbol;Acc:HGNC:15860]	20
ENSG00000186815	1765,362	-0,0089	0,426615	0,72339	-0,795	0,025863	-0,04945	protein_coding	TPCN1	two pore segment channel 1 [Source:HGNC Symbol;Acc:HGNC:18182]	12
ENSG00000173575	2210,52	-0,01025	0,427344	0,724094	-0,79374	0,022749	-0,02873	protein_coding	CHD2	chromodomain helicase DNA binding protein 2 [Source:HGNC Symbol;Acc:HGNC:1917]	15
ENSG00000025796	3032,74	0,00973	0,427928	0,72455	0,792742	0,022609	0,028741	protein_coding	SEC63	SEC63 homolog, protein translocation regulator [Source:HGNC Symbol;Acc:HGNC:21082]	6
ENSG00000054356	1942,196	-0,0072	0,428871	0,725079	-0,79113	0,025515	-0,05288	protein_coding	PTPRN	protein tyrosine phosphatase receptor type N [Source:HGNC Symbol;Acc:HGNC:9676]	2
ENSG00000146535	2312,314	0,009443	0,428662	0,725079	0,791483	0,022426	0,028384	protein_coding	GNA12	G protein subunit alpha 12 [Source:HGNC Symbol;Acc:HGNC:4380]	7
ENSG00000213639	3258,762	0,010071	0,429333	0,725167	0,790334	0,023265	0,030562	protein_coding	PPP1CB	protein phosphatase 1 catalytic subunit beta [Source:HGNC Symbol;Acc:HGNC:9282]	2
ENSG00000148180	5585,961	0,009866	0,429553	0,725167	0,789956	0,021961	0,026561	protein_coding	GSN	gelsolin [Source:HGNC Symbol;Acc:HGNC:4620]	9
ENSG00000135506	6334,984	0,010359	0,430274	0,725851	0,788722	0,022142	0,026641	protein_coding	OS9	OS9 endoplasmic reticulum lectin [Source:HGNC Symbol;Acc:HGNC:16994]	12
ENSG00000171862	3751,397	0,011886	0,431299	0,726729	0,78697	0,026515	0,042681	protein_coding	PTEN	phosphatase and tensin homolog [Source:HGNC Symbol;Acc:HGNC:9588]	10
ENSG00000138600	1648,768	0,008977	0,431426	0,726729	0,786753	0,024723	0,039193	protein_coding	SPPL2A	signal peptide peptidase like 2A [Source:HGNC Symbol;Acc:HGNC:30227]	15
ENSG00000150403	2720,332	0,010269	0,432173	0,727454	0,785478	0,022524	0,027731	protein_coding	TMCO3	transmembrane and coiled-coil domains 3 [Source:HGNC Symbol;Acc:HGNC:20329]	13
ENSG00000139645	2467,762	0,004987	0,4325	0,727473	0,784921	0,023	0,035146	protein_coding	ANKRD52	ankyrin repeat domain 52 [Source:HGNC Symbol;Acc:HGNC:26614]	12
ENSG00000158711	1820,205	0,009059	0,434202	0,729802	0,782021	0,024385	0,036755	protein_coding	ELK4	ETS transcription factor ELK4 [Source:HGNC Symbol;Acc:HGNC:3326]	1
ENSG00000265735	1787,233	-0,00588	0,435639	0,731149	-0,77958	0,025478	-0,05838	misc_RNA	RN7SL5P	RNA, 7SL, cytoplasmic 5, pseudogene [Source:HGNC Symbol;Acc:HGNC:10040]	9
ENSG00000124155	2812,314	-0,00975	0,435505	0,731149	-0,77981	0,023392	-0,03098	protein_coding	PIGT	phosphatidylinositol glycan anchor biosynthesis class T [Source:HGNC Symbol;Acc:HGNC:14938]	20
ENSG00000211445	5761,427	-0,00812	0,436409	0,731374	-0,77827	0,025428	-0,04695	protein_coding	GPX3	glutathione peroxidase 3 [Source:HGNC Symbol;Acc:HGNC:4555]	5
ENSG00000198712	110511,9	-0,00683	0,436106	0,731374	-0,77879	0,025545	-0,05427	protein_coding	MT-CO2	mitochondrially encoded cytochrome c oxidase II [Source:HGNC Symbol;Acc:HGNC:7421]	MT
ENSG00000270066	1902,6	-0,00691	0,436873	0,731619	-0,77748	0,025677	-0,05592	lncRNA	AL356488.:	small Cajal body-specific RNA 2	1
ENSG00000257103	2260,17	0,009561	0,437465	0,732078	0,77648	0,023576	0,03183	protein_coding	LSM14A	LSM14A mRNA processing body assembly factor [Source:HGNC Symbol;Acc:HGNC:24489]	19
ENSG00000010818	2425,922	-0,00921	0,438474	0,7327	-0,77477	0,023661	-0,03261	protein_coding	HIVEP2	HIVEP zinc finger 2 [Source:HGNC Symbol;Acc:HGNC:4921]	6
ENSG00000169871	1648,072	-0,00796	0,438323	0,7327	-0,77503	0,024286	-0,03797	protein_coding	TRIM56	tripartite motif containing 56 [Source:HGNC Symbol;Acc:HGNC:19028]	7
ENSG00000157654	7735,876	0,007662	0,438928	0,732927	0,774005	0,025217	0,046269	protein_coding	PALM2AKA	PALM2 and AKAP2 fusion [Source:HGNC Symbol;Acc:HGNC:33529]	9
ENSG00000074054	2074,735	-0,01099	0,44132	0,733569	-0,76997	0,023612	-0,0299	protein_coding	CLASP1	cytoplasmic linker associated protein 1 [Source:HGNC Symbol;Acc:HGNC:17088]	2
ENSG00000120742	1884,094	0,009837	0,441386	0,733569	0,769856	0,022945	0,028916	protein_coding	SERP1	stress associated endoplasmic reticulum protein 1 [Source:HGNC Symbol;Acc:HGNC:10759]	3
ENSG00000112851	3267,974	0,009042	0,441485	0,733569	0,769688	0,02432	0,035866	protein_coding	ERBIN	erbb2 interacting protein [Source:HGNC Symbol;Acc:HGNC:15842]	5
ENSG00000188647	1677,924	0,009259	0,44064	0,733569	0,771112	0,022899	0,029408	protein_coding	PTAR1	protein prenyltransferase alpha subunit repeat containing 1 [Source:HGNC Symbol;Acc:HGNC:30449]	9
ENSG00000137693	2668,829	0,009205	0,441544	0,733569	0,769589	0,02449	0,036462	protein_coding	YAP1	Yes1 associated transcriptional regulator [Source:HGNC Symbol;Acc:HGNC:16262]	11
ENSG00000007168	2771,308	0,009551	0,440847	0,733569	0,770764	0,023056	0,029649	protein_coding	PAFAH1B1	platelet activating factor acetylhydrolase 1b regulatory subunit 1 [Source:HGNC Symbol;Acc:HGNC:8574]	17
ENSG00000101337	2003,47	-0,01051	0,440988	0,733569	-0,77053	0,021151	-0,02345	protein_coding	TM9SF4	transmembrane 9 superfamily member 4 [Source:HGNC Symbol;Acc:HGNC:30797]	20
ENSG00000163931	3706,087	0,009582	0,443239	0,735741	0,766736	0,023095	0,029577	protein_coding	TKT	transketolase [Source:HGNC Symbol;Acc:HGNC:11834]	3
ENSG00000179134	2205,459	-0,00983	0,443491	0,735741	-0,76631	0,022294	-0,02674	protein_coding	SAMD4B	sterile alpha motif domain containing 4B [Source:HGNC Symbol;Acc:HGNC:25492]	19
ENSG00000160633	2108,667	-0,00991	0,444545	0,736958	-0,76454	0,024056	-0,03284	protein_coding	SAFB	scaffold attachment factor B [Source:HGNC Symbol;Acc:HGNC:10520]	19
ENSG00000131711	30999,3	-0,00873	0,44529	0,737662	-0,76329	0,02435	-0,0363	protein_coding	MAP1B	microtubule associated protein 1B [Source:HGNC Symbol;Acc:HGNC:6836]	5
ENSG00000182446	2951,981	-0,01522	0,445673	0,737766	-0,76265	0,022873	-0,02388	protein_coding	NPLOC4	NPL4 homolog, ubiquitin recognition factor [Source:HGNC Symbol;Acc:HGNC:18261]	17
ENSG00000164151	1891,097	-0,0103	0,446118	0,737971	-0,7619	0,021214	-0,02346	protein_coding	ICE1	interactor of little elongation complex ELL subunit 1 [Source:HGNC Symbol;Acc:HGNC:29154]	5
ENSG00000198887	1644,699	0,010111	0,447297	0,738877	0,75993	0,023873	0,031612	protein_coding	SMC5	structural maintenance of chromosomes 5 [Source:HGNC Symbol;Acc:HGNC:20465]	9
ENSG00000167468	3191,582	0,009455	0,447551	0,738877	0,759505	0,025339	0,040692	protein_coding	GPX4	glutathione peroxidase 4 [Source:HGNC Symbol;Acc:HGNC:4556]	19
ENSG00000186111	1913,118	-0,00932	0,447629	0,738877	-0,75937	0,024427	-0,03541	protein_coding	PIP5K1C	phosphatidylinositol-4-phosphate 5-kinase type 1 gamma [Source:HGNC Symbol;Acc:HGNC:8996]	19
ENSG00000151176	3952,509	0,008558	0,44854	0,73985	0,757851	0,024406	0,036703	protein_coding	PLBD2	phospholipase B domain containing 2 [Source:HGNC Symbol;Acc:HGNC:27283]	12
ENSG00000270647	1740,301	0,006734	0,448954	0,740003	0,757159	0,024343	0,040034	protein_coding	TAF15	TATA-box binding protein associated factor 15 [Source:HGNC Symbol;Acc:HGNC:11547]	17
ENSG00000162613	1945,872	0,008427	0,449604	0,740013	0,756076	0,02459	0,03803	protein_coding	FUBP1	far upstream element binding protein 1 [Source:HGNC Symbol;Acc:HGNC:4004]	1
ENSG00000168140	3397,459	0,007811	0,449354	0,740013	0,756493	0,024969	0,042423	protein_coding	VASN	vasorin [Source:HGNC Symbol;Acc:HGNC:18517]	16
ENSG00000136279	2216,835	-0,00986	0,451179	0,742076	-0,75345	0,021807	-0,02492	protein_coding	DBNL	drebrin like [Source:HGNC Symbol;Acc:HGNC:2696]	7
ENSG00000119888	4050,445	0,005952	0,452254	0,742938	0,751663	0,025498	0,056426	protein_coding	EPCAM	epithelial cell adhesion molecule [Source:HGNC Symbol;Acc:HGNC:11529]	2
ENSG00000198961	3078,572	0,010437	0,452743	0,742938	0,75085	0,020811	0,022152	protein_coding	PJA2	praja ring finger ubiquitin ligase 2 [Source:HGNC Symbol;Acc:HGNC:17481]	5
ENSG00000120137	2566,933	-0,00993	0,452995	0,742938	-0,75043	0,021756	-0,02466	protein_coding	PANK3	pantothenate kinase 3 [Source:HGNC Symbol;Acc:HGNC:19365]	5
ENSG00000072501	3799,591	0,008165	0,452643	0,742938	0,751017	0,023892	0,034229	protein_coding	SMC1A	structural maintenance of chromosomes 1A [Source:HGNC Symbol;Acc:HGNC:11111]	X

ENSG00000224078	2998,955	-0,00521	0,453318	0,742939	-0,74989	0,022853	-0,03259	lncRNA	SNHG14	small nucleolar RNA host gene 14 [Source:HGNC Symbol;Acc:HGNC:37462]	15
ENSG00000174282	1965,178	-0,00955	0,453818	0,743227	-0,74907	0,022775	-0,02788	protein_coding	ZBTB4	zinc finger and BTB domain containing 4 [Source:HGNC Symbol;Acc:HGNC:23847]	17
ENSG00000163125	1801,066	0,009669	0,457293	0,74616	0,743312	0,021764	0,024604	protein_coding	RPRD2	regulation of nuclear pre-mRNA domain containing 2 [Source:HGNC Symbol;Acc:HGNC:29039]	1
ENSG00000169967	2485,416	0,010255	0,458067	0,74616	0,742034	0,0225	0,026145	protein_coding	MAP3K2	mitogen-activated protein kinase kinase kinase 2 [Source:HGNC Symbol;Acc:HGNC:6854]	2
ENSG00000196549	2924,253	0,00267	0,457096	0,74616	0,743638	0,026029	0,137762	protein_coding	MME	membrane metalloendopeptidase [Source:HGNC Symbol;Acc:HGNC:7154]	3
ENSG00000124783	4975,746	0,00967	0,456996	0,74616	0,743803	0,022693	0,027288	protein_coding	SSR1	signal sequence receptor subunit 1 [Source:HGNC Symbol;Acc:HGNC:11323]	6
ENSG00000096070	1812,093	-0,00872	0,458003	0,74616	-0,74214	0,024043	-0,03369	protein_coding	BRPF3	bromodomain and PHD finger containing 3 [Source:HGNC Symbol;Acc:HGNC:14256]	6
ENSG00000139613	2979,832	-0,00943	0,45872	0,74616	-0,74096	0,022548	-0,02693	protein_coding	SMARCC2	SWI/SNF related, matrix associated, actin dependent regulator of chromatin subfamily c member 2 [Source:HGNC Symb	12
ENSG00000140941	1933,084	0,008653	0,458641	0,74616	0,741087	0,023963	0,033374	protein_coding	MAP1LC3B	microtubule associated protein 1 light chain 3 beta [Source:HGNC Symbol;Acc:HGNC:13352]	16
ENSG00000134440	3913,294	0,008363	0,456338	0,74616	0,744891	0,022002	0,026289	protein_coding	NARS1	asparaginyl-tRNA synthetase 1 [Source:HGNC Symbol;Acc:HGNC:7643]	18
ENSG00000101361	2425,446	0,010393	0,457029	0,74616	0,743748	0,026224	0,04352	protein_coding	NOP56	NOP56 ribonucleoprotein [Source:HGNC Symbol;Acc:HGNC:15911]	20
ENSG00000101160	4028,191	0,006581	0,458851	0,74616	0,740739	0,025407	0,05077	protein_coding	CTSZ	cathepsin Z [Source:HGNC Symbol;Acc:HGNC:2547]	20
ENSG00000162368	2072,253	0,009044	0,460186	0,747274	0,738541	0,023105	0,029115	protein_coding	CMPK1	cytidine/uridine monophosphate kinase 1 [Source:HGNC Symbol;Acc:HGNC:18170]	1
ENSG00000186635	1624,197	-0,00902	0,459976	0,747274	-0,73889	0,023417	-0,03034	protein_coding	ARAP1	ArfGAP with RhoGAP domain, ankyrin repeat and PH domain 1 [Source:HGNC Symbol;Acc:HGNC:16925]	11
ENSG00000049239	1659,26	0,00763	0,460644	0,747491	0,737787	0,024811	0,040582	protein_coding	H6PD	hexose-6-phosphate dehydrogenase/glucose 1-dehydrogenase [Source:HGNC Symbol;Acc:HGNC:4795]	1
ENSG00000116237	1975,269	0,005535	0,462323	0,747614	0,735027	0,02093	0,024397	protein_coding	ICMT	isoprenylcysteine carboxyl methyltransferase [Source:HGNC Symbol;Acc:HGNC:5350]	1
ENSG00000143418	2678,676	-0,00827	0,463319	0,747614	-0,73339	0,021354	-0,0241	protein_coding	CERS2	ceramide synthase 2 [Source:HGNC Symbol;Acc:HGNC:14076]	1
ENSG00000198087	1834,804	0,009091	0,462412	0,747614	0,734882	0,025024	0,038216	protein_coding	CD2AP	CD2 associated protein [Source:HGNC Symbol;Acc:HGNC:14258]	6
ENSG00000174238	1600,901	-0,00869	0,46325	0,747614	-0,73351	0,023423	-0,03056	protein_coding	PITPNA	phosphatidylinositol transfer protein alpha [Source:HGNC Symbol;Acc:HGNC:9001]	17
ENSG00000131467	2083,163	0,013089	0,463253	0,747614	0,733501	0,022589	0,023849	protein_coding	PSME3	proteasome activator subunit 3 [Source:HGNC Symbol;Acc:HGNC:9570]	17
ENSG00000177885	2203,452	-0,00923	0,461526	0,747614	-0,73634	0,023043	-0,0286	protein_coding	GRB2	growth factor receptor bound protein 2 [Source:HGNC Symbol;Acc:HGNC:4566]	17
ENSG00000130726	4357,657	0,009723	0,46259	0,747614	0,734588	0,021326	0,023195	protein_coding	TRIM28	tripartite motif containing 28 [Source:HGNC Symbol;Acc:HGNC:16384]	19
ENSG00000100030	2885,734	0,009833	0,46238	0,747614	0,734934	0,021532	0,023649	protein_coding	MAPK1	mitogen-activated protein kinase 1 [Source:HGNC Symbol;Acc:HGNC:6871]	22
ENSG00000136240	3632,57	0,008444	0,463913	0,748047	0,73242	0,023952	0,033254	protein_coding	KDELR2	KDEL endoplasmic reticulum protein retention receptor 2 [Source:HGNC Symbol;Acc:HGNC:6305]	7
ENSG00000164889	2489,129	0,008898	0,464937	0,749174	0,730742	0,024264	0,034008	protein_coding	SLC4A2	solute carrier family 4 member 2 [Source:HGNC Symbol;Acc:HGNC:11028]	7
ENSG00000148400	2117,794	0,007773	0,466025	0,749876	0,728962	0,024774	0,039476	protein_coding	NOTCH1	notch receptor 1 [Source:HGNC Symbol;Acc:HGNC:7881]	9
ENSG00000141425	1785,419	-0,00858	0,465781	0,749876	-0,72936	0,023886	-0,03258	protein_coding	RPRD1A	regulation of nuclear pre-mRNA domain containing 1A [Source:HGNC Symbol;Acc:HGNC:25560]	18
ENSG00000091409	4453,183	0,005547	0,467806	0,751053	0,726054	0,02565	0,060208	protein_coding	ITGA6	integrin subunit alpha 6 [Source:HGNC Symbol;Acc:HGNC:6142]	2
ENSG00000204217	5542,148	0,008288	0,468715	0,751053	0,724572	0,024758	0,03785	protein_coding	BMPR2	bone morphogenetic protein receptor type 2 [Source:HGNC Symbol;Acc:HGNC:1078]	2
ENSG00000164294	1877,919	0,006036	0,468527	0,751053	0,724877	0,024968	0,046136	protein_coding	GPX8	glutathione peroxidase 8 (putative) [Source:HGNC Symbol;Acc:HGNC:33100]	5
ENSG00000119318	4779,094	0,009628	0,467731	0,751053	0,726175	0,021262	0,022838	protein_coding	RAD23B	RAD23 homolog B, nucleotide excision repair protein [Source:HGNC Symbol;Acc:HGNC:9813]	9
ENSG00000185551	3165,172	0,007614	0,468124	0,751053	0,725535	0,024754	0,039471	protein_coding	NR2F2	nuclear receptor subfamily 2 group F member 2 [Source:HGNC Symbol;Acc:HGNC:7976]	15
ENSG00000104853	3278,304	-0,00972	0,467292	0,751053	-0,72689	0,02091	-0,022	protein_coding	CLPTM1	CLPTM1 regulator of GABA type A receptor forward trafficking [Source:HGNC Symbol;Acc:HGNC:2087]	19
ENSG00000115306	31637,37	0,009241	0,470494	0,752332	0,721676	0,023889	0,031273	protein_coding	SPTBN1	spectrin beta, non-erythrocytic 1 [Source:HGNC Symbol;Acc:HGNC:11275]	2
ENSG00000197111	2234,483	0,008634	0,47033	0,752332	0,721942	0,023644	0,031077	protein_coding	PCBP2	poly(rC) binding protein 2 [Source:HGNC Symbol;Acc:HGNC:8648]	12
ENSG00000172270	8353,768	0,009526	0,469901	0,752332	0,72264	0,021713	0,023879	protein_coding	BSG	basigin (Ok blood group) [Source:HGNC Symbol;Acc:HGNC:1116]	19
ENSG00000065526	3752,306	-0,00652	0,472878	0,753478	-0,7178	0,022672	-0,02917	protein_coding	SPEN	spen family transcriptional repressor [Source:HGNC Symbol;Acc:HGNC:17575]	1
ENSG00000172239	1813,632	0,008094	0,473502	0,753478	0,716792	0,022999	0,028849	protein_coding	PAIP1	poly(A) binding protein interacting protein 1 [Source:HGNC Symbol;Acc:HGNC:16945]	5
ENSG00000213949	8176,98	0,004971	0,473348	0,753478	0,717042	0,025738	0,066194	protein_coding	ITGA1	integrin subunit alpha 1 [Source:HGNC Symbol;Acc:HGNC:6134]	5
ENSG00000106443	1695,368	0,008647	0,472289	0,753478	0,71876	0,024003	0,032549	protein_coding	PHF14	PHD finger protein 14 [Source:HGNC Symbol;Acc:HGNC:22203]	7
ENSG00000110713	2096,263	-0,00916	0,471553	0,753478	-0,71995	0,021848	-0,0244	protein_coding	NUP98	nucleoporin 98 and 96 precursor [Source:HGNC Symbol;Acc:HGNC:8068]	11
ENSG00000184575	3414,568	-0,01052	0,472088	0,753478	-0,71909	0,020888	-0,02133	protein_coding	XPOT	exportin for tRNA [Source:HGNC Symbol;Acc:HGNC:12826]	12
ENSG00000104824	2294,597	0,008836	0,473044	0,753478	0,717535	0,023359	0,029464	protein_coding	HNRNPL	heterogeneous nuclear ribonucleoprotein L [Source:HGNC Symbol;Acc:HGNC:5045]	19
ENSG00000196642	1980,789	-0,01036	0,474366	0,754331	-0,71539	0,023853	-0,02936	protein_coding	RABL6	RAB, member RAS oncogene family like 6 [Source:HGNC Symbol;Acc:HGNC:24703]	9
ENSG00000175130	2190,122	0,00637	0,478783	0,756454	0,708262	0,024556	0,03997	protein_coding	MARCKSL1	MARCKS like 1 [Source:HGNC Symbol;Acc:HGNC:7142]	1
ENSG00000116133	3444,616	-0,00811	0,477073	0,756454	-0,71102	0,024728	-0,03735	protein_coding	DHCR24	24-dehydrocholesterol reductase [Source:HGNC Symbol;Acc:HGNC:2859]	1
ENSG00000170248	2730,631	0,009237	0,479318	0,756454	0,7074	0,023183	0,027971	protein_coding	PDCD6IP	programmed cell death 6 interacting protein [Source:HGNC Symbol;Acc:HGNC:8766]	3
ENSG00000259956	1840,601	0,008283	0,479297	0,756454	0,707434	0,022933	0,02805	protein_coding	RBM15B	RNA binding motif protein 15B [Source:HGNC Symbol;Acc:HGNC:24303]	3
ENSG00000075420	6369,506	0,009069	0,477814	0,756454	0,709823	0,022569	0,026185	protein_coding	FNDC3B	fibronectin type III domain containing 3B [Source:HGNC Symbol;Acc:HGNC:24670]	3
ENSG00000114331	1697,639	-0,00855	0,477291	0,756454	-0,71067	0,023309	-0,02933	protein_coding	ACAP2	ArfGAP with coiled-coil, ankyrin repeat and PH domains 2 [Source:HGNC Symbol;Acc:HGNC:16469]	3
ENSG00000140332	2068,381	0,007624	0,477135	0,756454	0,710919	0,02458	0,037437	protein_coding	TLE3	TLE family member 3, transcriptional corepressor [Source:HGNC Symbol;Acc:HGNC:11839]	15
ENSG00000196628	3348,117	0,005981	0,478357	0,756454	0,708947	0,025431	0,051751	protein_coding	TCF4	transcription factor 4 [Source:HGNC Symbol;Acc:HGNC:11634]	18

ENSG00000125827	3220,117	-0,01023	0,477107	0,756454	-0,71096	0,024062	-0,03018	protein_coding	TMX4	thioredoxin related transmembrane protein 4 [Source:HGNC Symbol;Acc:HGNC:25237]	20
ENSG00000131263	1942,511	0,008569	0,478467	0,756454	0,708771	0,021215	0,022765	protein_coding	RLIM	ring finger protein, LIM domain interacting [Source:HGNC Symbol;Acc:HGNC:13429]	X
ENSG00000198763	108599,1	-0,00378	0,478057	0,756454	-0,70943	0,025899	-0,08702	protein_coding	MT-ND2	mitochondrially encoded NADH:ubiquinone oxidoreductase core subunit 2 [Source:HGNC Symbol;Acc:HGNC:7456]	MT
ENSG00000188895	1888,765	-0,00869	0,480179	0,757294	-0,70601	0,022806	-0,02715	protein_coding	MSL1	MSL complex subunit 1 [Source:HGNC Symbol;Acc:HGNC:27905]	17
ENSG00000197535	2358,162	0,006923	0,480826	0,7574	0,704974	0,024988	0,042288	protein_coding	MYO5A	myosin VA [Source:HGNC Symbol;Acc:HGNC:7602]	15
ENSG00000103241	1569,355	-0,00439	0,480905	0,7574	-0,70485	0,025833	-0,07474	protein_coding	FOXF1	forkhead box F1 [Source:HGNC Symbol;Acc:HGNC:3809]	16
ENSG00000142657	2509,537	-0,01335	0,482575	0,75797	-0,70217	0,025807	-0,03129	protein_coding	PGD	phosphogluconate dehydrogenase [Source:HGNC Symbol;Acc:HGNC:8891]	1
ENSG00000176986	2258,755	-0,0085	0,482913	0,75797	-0,70162	0,023336	-0,02911	protein_coding	SEC24C	SEC24 homolog C, COPII coat complex component [Source:HGNC Symbol;Acc:HGNC:10705]	10
ENSG00000150991	13275,81	-0,01136	0,482622	0,75797	-0,70209	0,024026	-0,02818	protein_coding	UBC	ubiquitin C [Source:HGNC Symbol;Acc:HGNC:12468]	12
ENSG00000142166	1680,554	-0,0084	0,482623	0,75797	-0,70209	0,023571	-0,03021	protein_coding	IFNAR1	interferon alpha and beta receptor subunit 1 [Source:HGNC Symbol;Acc:HGNC:5432]	21
ENSG00000085224	3258,153	-0,0075	0,481829	0,75797	-0,70336	0,023192	-0,02974	protein_coding	ATRX	ATRX chromatin remodeler [Source:HGNC Symbol;Acc:HGNC:886]	X
ENSG00000204574	2428,644	0,014696	0,484121	0,75883	0,69969	0,024941	0,026717	protein_coding	ABCF1	ATP binding cassette subfamily F member 1 [Source:HGNC Symbol;Acc:HGNC:70]	6
ENSG00000166833	1707,471	0,007053	0,483981	0,75883	0,699914	0,024857	0,040395	protein_coding	NAV2	neuron navigator 2 [Source:HGNC Symbol;Acc:HGNC:15997]	11
ENSG00000197965	3689,492	-0,00952	0,486711	0,762371	-0,69555	0,020286	-0,01989	protein_coding	MPZL1	myelin protein zero like 1 [Source:HGNC Symbol;Acc:HGNC:7226]	1
ENSG00000197136	1604,737	-0,00867	0,487717	0,763426	-0,69394	0,023398	-0,02881	protein_coding	PCNX3	pecanex 3 [Source:HGNC Symbol;Acc:HGNC:18760]	11
ENSG00000104904	4432,978	0,008247	0,488497	0,763608	0,692701	0,021486	0,023088	protein_coding	OAZ1	ornithine decarboxylase antizyme 1 [Source:HGNC Symbol;Acc:HGNC:8095]	19
ENSG00000198911	3308,251	-0,00865	0,488263	0,763608	-0,69307	0,024171	-0,03219	protein_coding	SREBF2	sterol regulatory element binding transcription factor 2 [Source:HGNC Symbol;Acc:HGNC:11290]	22
ENSG00000154277	3395,326	-0,00662	0,488883	0,763693	-0,69209	0,025483	-0,04832	protein_coding	UCHL1	ubiquitin C-terminal hydrolase L1 [Source:HGNC Symbol;Acc:HGNC:12513]	4
ENSG00000118785	2228,679	0,001686	0,49028	0,765355	0,689864	0,026042	0,180067	protein_coding	SPP1	secreted phosphoprotein 1 [Source:HGNC Symbol;Acc:HGNC:11255]	4
ENSG00000198815	1636,442	-0,00843	0,49274	0,765561	-0,68596	0,022765	-0,02649	protein_coding	FOXJ3	forkhead box J3 [Source:HGNC Symbol;Acc:HGNC:29178]	1
ENSG00000169554	3849,196	0,006078	0,49128	0,765561	0,688274	0,02527	0,047423	protein_coding	ZEB2	zinc finger E-box binding homeobox 2 [Source:HGNC Symbol;Acc:HGNC:14881]	2
ENSG00000135926	1815,921	0,005901	0,492356	0,765561	0,686566	0,022608	0,028161	protein_coding	TMBIM1	transmembrane BAX inhibitor motif containing 1 [Source:HGNC Symbol;Acc:HGNC:23410]	2
ENSG00000163902	5514,323	0,013678	0,4913	0,765561	0,688242	0,020165	0,017905	protein_coding	RPN1	ribophorin I [Source:HGNC Symbol;Acc:HGNC:10381]	3
ENSG00000159842	2374,08	-0,00167	0,491727	0,765561	-0,68756	0,02116	-0,02473	protein_coding	ABR	ABR activator of RhoGEF and GTPase [Source:HGNC Symbol;Acc:HGNC:81]	17
ENSG00000125651	2023,179	-0,00817	0,492446	0,765561	-0,68642	0,023478	-0,02942	protein_coding	GTF2F1	general transcription factor IIF subunit 1 [Source:HGNC Symbol;Acc:HGNC:4652]	19
ENSG00000083845	3151,379	0,008622	0,492375	0,765561	0,686537	0,023302	0,028214	protein_coding	RPS5	ribosomal protein S5 [Source:HGNC Symbol;Acc:HGNC:10426]	19
ENSG00000158195	2472,58	0,008888	0,493164	0,765664	0,685286	0,021856	0,023414	protein_coding	WASF2	WASP family member 2 [Source:HGNC Symbol;Acc:HGNC:12733]	1
ENSG00000233927	1659,74	0,006819	0,493472	0,765664	0,684796	0,023572	0,031527	protein_coding	RPS28	ribosomal protein S28 [Source:HGNC Symbol;Acc:HGNC:10418]	19
ENSG00000135837	3629,829	-0,00748	0,493958	0,765902	-0,68403	0,02246	-0,02619	protein_coding	CEP350	centrosomal protein 350 [Source:HGNC Symbol;Acc:HGNC:24238]	1
ENSG00000170315	9412,936	0,007653	0,494308	0,765927	0,683473	0,023584	0,030401	protein_coding	UBB	ubiquitin B [Source:HGNC Symbol;Acc:HGNC:12463]	17
ENSG00000138081	1626,853	0,007804	0,498213	0,77094	0,677305	0,023209	0,028374	protein_coding	FBXO11	F-box protein 11 [Source:HGNC Symbol;Acc:HGNC:13590]	2
ENSG00000140564	3985,228	0,012714	0,497944	0,77094	0,677728	0,02509	0,0288	protein_coding	FURIN	furin, paired basic amino acid cleaving enzyme [Source:HGNC Symbol;Acc:HGNC:8568]	15
ENSG00000090372	2269,523	-0,00926	0,498609	0,771035	-0,67668	0,021587	-0,02222	protein_coding	STRN4	striatin 4 [Source:HGNC Symbol;Acc:HGNC:15721]	19
ENSG00000143341	4852,567	-0,00421	0,501209	0,77243	-0,67259	0,025744	-0,06872	protein_coding	HMCN1	hemicentin 1 [Source:HGNC Symbol;Acc:HGNC:19194]	1
ENSG00000153113	4442,754	0,008046	0,502324	0,77243	0,670838	0,022828	0,026455	protein_coding	CAST	calpastatin [Source:HGNC Symbol;Acc:HGNC:1515]	5
ENSG00000112715	3174,932	-0,00621	0,502533	0,77243	-0,67051	0,024823	-0,04064	protein_coding	VEGFA	vascular endothelial growth factor A [Source:HGNC Symbol;Acc:HGNC:12680]	6
ENSG00000078269	5185,548	-0,00841	0,50212	0,77243	-0,67116	0,018923	-0,01714	protein_coding	SYNJ2	synaptojanin 2 [Source:HGNC Symbol;Acc:HGNC:11504]	6
ENSG00000122786	19452,51	0,005155	0,500197	0,77243	0,674179	0,02553	0,055477	protein_coding	CALD1	caldesmon 1 [Source:HGNC Symbol;Acc:HGNC:1441]	7
ENSG00000177156	2171,838	-0,00776	0,501427	0,77243	-0,67225	0,023851	-0,03102	protein_coding	TALDO1	transaldolase 1 [Source:HGNC Symbol;Acc:HGNC:11559]	11
ENSG00000128829	1930,749	0,007278	0,501731	0,77243	0,671768	0,023561	0,030271	protein_coding	EIF2AK4	eukaryotic translation initiation factor 2 alpha kinase 4 [Source:HGNC Symbol;Acc:HGNC:19687]	15
ENSG00000141424	2313,232	0,007702	0,50142	0,77243	0,672256	0,02431	0,033584	protein_coding	SLC39A6	solute carrier family 39 member 6 [Source:HGNC Symbol;Acc:HGNC:18607]	18
ENSG00000099341	3082,693	0,008532	0,500545	0,77243	0,673632	0,022265	0,024381	protein_coding	PSMD8	proteasome 26S subunit, non-ATPase 8 [Source:HGNC Symbol;Acc:HGNC:9566]	19
ENSG00000006125	4204,266	0,011385	0,504179	0,773927	0,667929	0,021457	0,020492	protein_coding	AP2B1	adaptor related protein complex 2 subunit beta 1 [Source:HGNC Symbol;Acc:HGNC:563]	17
ENSG00000086758	11490,82	-0,00947	0,503992	0,773927	-0,66822	0,019027	-0,01696	protein_coding	HUWE1	HECT, UBA and WWE domain containing E3 ubiquitin protein ligase 1 [Source:HGNC Symbol;Acc:HGNC:30892]	X
ENSG00000197386	2622,499	-0,00749	0,504766	0,774311	-0,66701	0,024105	-0,03251	protein_coding	HTT	huntingtin [Source:HGNC Symbol;Acc:HGNC:4851]	4
ENSG00000163956	2703,503	0,008188	0,505623	0,775109	0,665669	0,023017	0,026759	protein_coding	LRPAP1	LDL receptor related protein associated protein 1 [Source:HGNC Symbol;Acc:HGNC:6701]	4
ENSG00000173273	1635,404	0,008028	0,506346	0,775184	0,664538	0,023465	0,028629	protein_coding	TNKS	tankyrase [Source:HGNC Symbol;Acc:HGNC:11941]	8
ENSG00000125249	2120,988	0,006391	0,506112	0,775184	0,664904	0,024234	0,03507	protein_coding	RAP2A	RAP2A, member of RAS oncogene family [Source:HGNC Symbol;Acc:HGNC:9861]	13
ENSG00000109332	3483,003	0,007989	0,507942	0,776094	0,662045	0,023739	0,02973	protein_coding	UBE2D3	ubiquitin conjugating enzyme E2 D3 [Source:HGNC Symbol;Acc:HGNC:12476]	4
ENSG00000100813	3717,427	-0,00845	0,507952	0,776094	-0,66203	0,022784	-0,02559	protein_coding	ACIN1	apoptotic chromatin condensation inducer 1 [Source:HGNC Symbol;Acc:HGNC:17066]	14
ENSG00000159202	1855,797	-0,00808	0,507941	0,776094	-0,66205	0,022684	-0,02559	protein_coding	UBE2Z	ubiquitin conjugating enzyme E2 Z [Source:HGNC Symbol;Acc:HGNC:25847]	17
ENSG00000170027	6591,229	0,010291	0,510559	0,778585	0,657968	0,019713	0,017553	protein_coding	YWHAG	tyrosine 3-monooxygenase/tryptophan 5-monooxygenase activation protein gamma [Source:HGNC Symbol;Acc:HGNC:	7

ENSG00000184374	4279,249	-0,00204	0,510029	0,778585	-0,65879	0,026013	-0,14276	protein_coding	COLEC10	collectin subfamily member 10 [Source:HGNC Symbol;Acc:HGNC:2220]	8
ENSG00000105220	3492,334	-0,00908	0,510598	0,778585	-0,65791	0,022651	-0,0245	protein_coding	GPI	glucose-6-phosphate isomerase [Source:HGNC Symbol;Acc:HGNC:4458]	19
ENSG00000130770	1622,642	0,01036	0,51155	0,779521	0,656426	0,023238	0,025009	protein_coding	ATP5IF1	ATP synthase inhibitory factor subunit 1 [Source:HGNC Symbol;Acc:HGNC:871]	1
ENSG00000070367	2050,182	0,008133	0,511927	0,779579	0,65584	0,023215	0,027163	protein_coding	EXOC5	exocyst complex component 5 [Source:HGNC Symbol;Acc:HGNC:10696]	14
ENSG00000164880	3074,319	-0,0082	0,5128	0,780392	-0,65448	0,022708	-0,02528	protein_coding	INTS1	integrator complex subunit 1 [Source:HGNC Symbol;Acc:HGNC:24555]	7
ENSG00000100591	2000,327	0,00886	0,513559	0,781031	0,653305	0,023002	0,025601	protein_coding	AHSA1	activator of HSP90 ATPase activity 1 [Source:HGNC Symbol;Acc:HGNC:1189]	14
ENSG00000143442	1606,676	-0,00821	0,517323	0,783648	-0,64748	0,023143	-0,02646	protein_coding	POGZ	pogo transposable element derived with ZNF domain [Source:HGNC Symbol;Acc:HGNC:18801]	1
ENSG00000136193	3177,176	-0,00713	0,516753	0,783648	-0,64836	0,024136	-0,03236	protein_coding	SCRN1	secernin 1 [Source:HGNC Symbol;Acc:HGNC:22192]	7
ENSG00000119314	2915,902	0,00766	0,516971	0,783648	0,648021	0,023345	0,027856	protein_coding	PTBP3	polypyrimidine tract binding protein 3 [Source:HGNC Symbol;Acc:HGNC:10253]	9
ENSG00000107959	2142,914	0,00895	0,517238	0,783648	0,64761	0,021963	0,022356	protein_coding	PITRM1	pitrilysin metallopeptidase 1 [Source:HGNC Symbol;Acc:HGNC:17663]	10
ENSG00000107863	2542,669	0,006897	0,517061	0,783648	0,647883	0,023633	0,030009	protein_coding	ARHGAP21	Rho GTPase activating protein 21 [Source:HGNC Symbol;Acc:HGNC:23725]	10
ENSG00000168056	2176,946	0,006957	0,516553	0,783648	0,648668	0,024991	0,038793	protein_coding	LTBP3	latent transforming growth factor beta binding protein 3 [Source:HGNC Symbol;Acc:HGNC:6716]	11
ENSG00000162923	2928,694	0,007919	0,519278	0,785575	0,644459	0,021282	0,021176	protein_coding	WDR26	WD repeat domain 26 [Source:HGNC Symbol;Acc:HGNC:21208]	1
ENSG00000152492	2464,153	0,00718	0,519015	0,785575	0,644865	0,024281	0,032954	protein_coding	CCDC50	coiled-coil domain containing 50 [Source:HGNC Symbol;Acc:HGNC:18111]	3
ENSG00000143612	2562,809	-0,00821	0,520041	0,786209	-0,64328	0,02127	-0,02095	protein_coding	C1orf43	chromosome 1 open reading frame 43 [Source:HGNC Symbol;Acc:HGNC:29876]	1
ENSG00000143515	2093,917	-0,0057	0,521748	0,786209	-0,64065	0,022855	-0,02742	protein_coding	ATP8B2	ATPase phospholipid transporting 8B2 [Source:HGNC Symbol;Acc:HGNC:13534]	1
ENSG00000175426	4220,107	0,003083	0,520998	0,786209	0,641807	0,025883	0,087782	protein_coding	PCSK1	proprotein convertase subtilisin/kexin type 1 [Source:HGNC Symbol;Acc:HGNC:8743]	5
ENSG00000269900	47046,78	-0,00566	0,521348	0,786209	-0,64127	0,025128	-0,04386	lncRNA	RMRP	RNA component of mitochondrial RNA processing endoribonuclease [Source:HGNC Symbol;Acc:HGNC:10031]	9
ENSG00000135677	4492,773	-0,00958	0,521445	0,786209	-0,64112	0,022469	-0,02299	protein_coding	GNS	glucosamine (N-acetyl)-6-sulfatase [Source:HGNC Symbol;Acc:HGNC:4422]	12
ENSG00000089053	1769,949	-0,00693	0,521456	0,786209	-0,6411	0,024123	-0,03224	protein_coding	ANAPC5	anaphase promoting complex subunit 5 [Source:HGNC Symbol;Acc:HGNC:15713]	12
ENSG00000067369	2810,786	-0,00821	0,522113	0,786244	-0,64009	0,022705	-0,02471	protein_coding	TP53BP1	tumor protein p53 binding protein 1 [Source:HGNC Symbol;Acc:HGNC:11999]	15
ENSG00000065911	1830,063	0,006617	0,523166	0,787316	0,638472	0,024444	0,034704	protein_coding	MTHFD2	methylenetetrahydrofolate dehydrogenase (NADP+ dependent) 2, methenyltetrahydrofolate cyclohydrolase [Source:H	2
ENSG00000075568	2415,882	-0,00764	0,524214	0,788377	-0,63686	0,022007	-0,02294	protein_coding	TMEM131	transmembrane protein 131 [Source:HGNC Symbol;Acc:HGNC:30366]	2
ENSG00000143970	2203,63	0,007922	0,524793	0,788731	0,635975	0,022665	0,024652	protein_coding	ASXL2	ASXL transcriptional regulator 2 [Source:HGNC Symbol;Acc:HGNC:23805]	2
ENSG00000164574	2340,133	0,005631	0,525465	0,789226	0,634944	0,025111	0,0433	protein_coding	GALNT10	polypeptide N-acetylgalactosaminyltransferase 10 [Source:HGNC Symbol;Acc:HGNC:19873]	5
ENSG00000242125	5433,4	-0,00224	0,526403	0,790119	-0,63351	0,025743	-0,0853	lncRNA	SNHG3	small nucleolar RNA host gene 3 [Source:HGNC Symbol;Acc:HGNC:10118]	1
ENSG00000115963	4158,873	-0,00665	0,52828	0,79242	-0,63063	0,024356	-0,03366	protein_coding	RND3	Rho family GTPase 3 [Source:HGNC Symbol;Acc:HGNC:671]	2
ENSG00000155096	3487,627	0,007609	0,528736	0,792587	0,629937	0,023816	0,029135	protein_coding	AZIN1	antizyme inhibitor 1 [Source:HGNC Symbol;Acc:HGNC:16432]	8
ENSG00000083312	3554,165	0,007217	0,529519	0,793244	0,62874	0,023571	0,028462	protein_coding	TNPO1	transportin 1 [Source:HGNC Symbol;Acc:HGNC:6401]	5
ENSG00000038382	6811,84	-0,0079	0,531083	0,794361	-0,62635	0,022268	-0,02313	protein_coding	TRIO	trio Rho guanine nucleotide exchange factor [Source:HGNC Symbol;Acc:HGNC:12303]	5
ENSG00000134982	2621,32	-0,00715	0,531785	0,794361	-0,62528	0,024334	-0,03232	protein_coding	APC	APC regulator of WNT signaling pathway [Source:HGNC Symbol;Acc:HGNC:583]	5
ENSG00000196498	7295,989	-0,00734	0,5319	0,794361	-0,62511	0,020764	-0,01964	protein_coding	NCOR2	nuclear receptor corepressor 2 [Source:HGNC Symbol;Acc:HGNC:7673]	12
ENSG00000171490	1822,45	0,007853	0,532681	0,794361	0,623918	0,022858	0,024875	protein_coding	RSL1D1	ribosomal L1 domain containing 1 [Source:HGNC Symbol;Acc:HGNC:24534]	16
ENSG00000101294	3448,311	-0,00763	0,532479	0,794361	-0,62423	0,023345	-0,02683	protein_coding	HM13	histocompatibility minor 13 [Source:HGNC Symbol;Acc:HGNC:16435]	20
ENSG00000124209	1780,715	0,006548	0,532679	0,794361	0,623922	0,024379	0,033598	protein_coding	RAB22A	RAB22A, member RAS oncogene family [Source:HGNC Symbol;Acc:HGNC:9764]	20
ENSG00000100320	3065,299	0,00835	0,531127	0,794361	0,626286	0,021999	0,022083	protein_coding	RBFOX2	RNA binding fox-1 homolog 2 [Source:HGNC Symbol;Acc:HGNC:9906]	22
ENSG00000164171	13427,28	-0,01036	0,534499	0,794555	-0,62115	0,024282	-0,0269	protein_coding	ITGA2	integrin subunit alpha 2 [Source:HGNC Symbol;Acc:HGNC:6137]	5
ENSG00000092439	2024,707	-0,00686	0,533785	0,794555	-0,62224	0,023166	-0,02685	protein_coding	TRPM7	transient receptor potential cation channel subfamily M member 7 [Source:HGNC Symbol;Acc:HGNC:17994]	15
ENSG00000103257	2872,213	0,005504	0,534458	0,794555	0,621215	0,025072	0,042254	protein_coding	SLC7A5	solute carrier family 7 member 5 [Source:HGNC Symbol;Acc:HGNC:11063]	16
ENSG00000166479	1726,001	0,007235	0,534538	0,794555	0,621094	0,023491	0,027749	protein_coding	TMX3	thioredoxin related transmembrane protein 3 [Source:HGNC Symbol;Acc:HGNC:24718]	18
ENSG00000100280	1998,114	-0,00832	0,533464	0,794555	-0,62273	0,023905	-0,02824	protein_coding	AP1B1	adaptor related protein complex 1 subunit beta 1 [Source:HGNC Symbol;Acc:HGNC:554]	22
ENSG00000162733	2819,028	0,004017	0,5363	0,795897	0,618417	0,025643	0,06119	protein_coding	DDR2	discoidin domain receptor tyrosine kinase 2 [Source:HGNC Symbol;Acc:HGNC:2731]	1
ENSG00000134369	6968,449	0,004878	0,536901	0,795897	0,617506	0,0236	0,03088	protein_coding	NAV1	neuron navigator 1 [Source:HGNC Symbol;Acc:HGNC:15989]	1
ENSG00000111897	4106,598	0,007846	0,53717	0,795897	0,617098	0,022373	0,023117	protein_coding	SERINC1	serine incorporator 1 [Source:HGNC Symbol;Acc:HGNC:13464]	6
ENSG00000139318	2163,915	0,004887	0,536581	0,795897	0,617991	0,025319	0,047945	protein_coding	DUSP6	dual specificity phosphatase 6 [Source:HGNC Symbol;Acc:HGNC:3072]	12
ENSG00000139514	2299,838	-0,00687	0,536605	0,795897	-0,61795	0,023254	-0,027	protein_coding	SLC7A1	solute carrier family 7 member 1 [Source:HGNC Symbol;Acc:HGNC:11057]	13
ENSG00000143153	2738,383	-0,00226	0,540325	0,800056	-0,61232	0,021723	-0,02372	protein_coding	ATP1B1	ATPase Na+/K+ transporting subunit beta 1 [Source:HGNC Symbol;Acc:HGNC:804]	1
ENSG00000244038	2120,181	0,006984	0,541943	0,801146	0,609878	0,023654	0,028244	protein_coding	DDOST	dolichyl-diphosphooligosaccharide--protein glycosyltransferase non-catalytic subunit [Source:HGNC Symbol;Acc:HGNC:	1
ENSG00000172053	1973,812	0,006391	0,541659	0,801146	0,610307	0,021907	0,022455	protein_coding	QARS1	glutaminyl-tRNA synthetase 1 [Source:HGNC Symbol;Acc:HGNC:9751]	3
ENSG00000173598	1858,217	0,006375	0,542105	0,801146	0,609632	0,024386	0,033208	protein_coding	NUDT4	nudix hydrolase 4 [Source:HGNC Symbol;Acc:HGNC:8051]	12
ENSG00000162337	2755,75	-0,00464	0,543441	0,802282	-0,60762	0,02538	-0,04944	protein_coding	LRP5	LDL receptor related protein 5 [Source:HGNC Symbol;Acc:HGNC:6697]	11
ENSG00000278540	1655,485	-0,00821	0,543717	0,802282	-0,6072	0,02168	-0,02072	protein_coding	ACACA	acetyl-CoA carboxylase alpha [Source:HGNC Symbol;Acc:HGNC:84]	17

ENSG00000105429	1588,697	-0,00644	0,54392	0,802282	-0,6069	0,024237	-0,03194	protein_coding	MEGF8	multiple EGF like domains 8 [Source:HGNC Symbol;Acc:HGNC:3233]	19
ENSG00000175115	2569,441	0,006428	0,544933	0,803261	0,605371	0,023477	0,027867	protein_coding	PACS1	phosphofurin acidic cluster sorting protein 1 [Source:HGNC Symbol;Acc:HGNC:30032]	11
ENSG00000104419	2108,675	-0,00494	0,545569	0,803545	-0,60441	0,02523	-0,04521	protein_coding	NDRG1	N-myc downstream regulated 1 [Source:HGNC Symbol;Acc:HGNC:7679]	8
ENSG00000147872	2210,524	0,006302	0,546173	0,803545	0,603505	0,024548	0,034126	protein_coding	PLIN2	perilipin 2 [Source:HGNC Symbol;Acc:HGNC:248]	9
ENSG00000099994	1829,089	0,003171	0,546077	0,803545	0,603649	0,026129	0,115695	protein_coding	SUSD2	sushi domain containing 2 [Source:HGNC Symbol;Acc:HGNC:30667]	22
ENSG00000108679	5401,279	0,005669	0,547258	0,804112	0,601875	0,024374	0,033962	protein_coding	LGALS3BP	galectin 3 binding protein [Source:HGNC Symbol;Acc:HGNC:6564]	17
ENSG00000157637	1956,922	0,006769	0,547232	0,804112	0,601913	0,023599	0,027876	protein_coding	SLC38A10	solute carrier family 38 member 10 [Source:HGNC Symbol;Acc:HGNC:28237]	17
ENSG00000083223	1593,438	0,007192	0,548073	0,804797	0,60065	0,022968	0,024883	protein_coding	TUT7	terminal uridylyl transferase 7 [Source:HGNC Symbol;Acc:HGNC:25817]	9
ENSG00000074370	2409,564	-0,00434	0,548762	0,805294	-0,59962	0,025467	-0,05217	protein_coding	ATP2A3	ATPase sarcoplasmic/endoplasmic reticulum Ca2+ transporting 3 [Source:HGNC Symbol;Acc:HGNC:813]	17
ENSG00000275052	1642,232	-0,00739	0,551717	0,806111	-0,59519	0,021719	-0,02084	protein_coding	PPP4R3B	protein phosphatase 4 regulatory subunit 3B [Source:HGNC Symbol;Acc:HGNC:29267]	2
ENSG00000163166	1582,362	0,006851	0,551151	0,806111	0,596037	0,023394	0,026628	protein_coding	IWS1	interacts with SUPT6H, CTD assembly factor 1 [Source:HGNC Symbol;Acc:HGNC:25467]	2
ENSG00000159692	2025,016	-0,0072	0,555625	0,806111	-0,58935	0,022438	-0,0227	protein_coding	CTBP1	C-terminal binding protein 1 [Source:HGNC Symbol;Acc:HGNC:2494]	4
ENSG00000138760	8350,571	0,007299	0,552354	0,806111	0,594237	0,019186	0,015837	protein_coding	SCARB2	scavenger receptor class B member 2 [Source:HGNC Symbol;Acc:HGNC:1665]	4
ENSG00000112972	2126,299	-0,00444	0,551987	0,806111	-0,59478	0,023687	-0,03074	protein_coding	HMGCS1	3-hydroxy-3-methylglutaryl-CoA synthase 1 [Source:HGNC Symbol;Acc:HGNC:5007]	5
ENSG00000146072	1591,986	-0,00833	0,555123	0,806111	-0,5901	0,025838	-0,03849	protein_coding	TNFRSF21	TNF receptor superfamily member 21 [Source:HGNC Symbol;Acc:HGNC:13469]	6
ENSG00000146433	1658,688	0,00748	0,551122	0,806111	0,59608	0,022454	0,022818	protein_coding	TMEM181	transmembrane protein 181 [Source:HGNC Symbol;Acc:HGNC:20958]	6
ENSG00000106052	3856,529	0,007481	0,555513	0,806111	0,589519	0,022179	0,021788	protein_coding	TAX1BP1	Tax1 binding protein 1 [Source:HGNC Symbol;Acc:HGNC:11575]	7
ENSG00000119402	1594,125	-0,00677	0,553186	0,806111	-0,59299	0,023095	-0,02539	protein_coding	FBXW2	F-box and WD repeat domain containing 2 [Source:HGNC Symbol;Acc:HGNC:13608]	9
ENSG00000136854	1823,577	-0,00729	0,552188	0,806111	-0,59448	0,022641	-0,02345	protein_coding	STXBP1	syntaxin binding protein 1 [Source:HGNC Symbol;Acc:HGNC:11444]	9
ENSG00000185650	3860,436	-0,00576	0,555275	0,806111	-0,58987	0,024389	-0,03321	protein_coding	ZFP36L1	ZFP36 ring finger protein like 1 [Source:HGNC Symbol;Acc:HGNC:1107]	14
ENSG00000137831	15667,8	0,003012	0,5552	0,806111	0,589985	0,02581	0,075925	protein_coding	UACA	uveal autoantigen with coiled-coil domains and ankyrin repeats [Source:HGNC Symbol;Acc:HGNC:15947]	15
ENSG00000102908	1957,505	-0,00536	0,552645	0,806111	-0,5938	0,024962	-0,03959	protein_coding	NFAT5	nuclear factor of activated T cells 5 [Source:HGNC Symbol;Acc:HGNC:7774]	16
ENSG00000062716	3321,73	0,008078	0,551314	0,806111	0,595792	0,023725	0,026586	protein_coding	VMP1	vacuole membrane protein 1 [Source:HGNC Symbol;Acc:HGNC:29559]	17
ENSG00000108654	12053,63	0,007916	0,550303	0,806111	0,597306	0,01828	0,014441	protein_coding	DDX5	DEAD-box helicase 5 [Source:HGNC Symbol;Acc:HGNC:2746]	17
ENSG00000141380	2424,513	0,006412	0,553706	0,806111	0,592216	0,023741	0,028536	protein_coding	SS18	SS18 subunit of BAF chromatin remodeling complex [Source:HGNC Symbol;Acc:HGNC:11340]	18
ENSG00000130702	3325,065	-0,00153	0,553403	0,806111	-0,59267	0,025309	-0,05939	protein_coding	LAMA5	laminin subunit alpha 5 [Source:HGNC Symbol;Acc:HGNC:6485]	20
ENSG00000100403	2395,493	-0,0079	0,554106	0,806111	-0,59162	0,021691	-0,02037	protein_coding	ZC3H7B	zinc finger CCCH-type containing 7B [Source:HGNC Symbol;Acc:HGNC:30869]	22
ENSG00000160551	4370,992	0,011252	0,555995	0,80614	0,588801	0,022443	0,019978	protein_coding	TAOK1	TAO kinase 1 [Source:HGNC Symbol;Acc:HGNC:29259]	17
ENSG00000010278	8819,624	-0,00452	0,556704	0,806152	-0,58774	0,025334	-0,04756	protein_coding	CD9	CD9 molecule [Source:HGNC Symbol;Acc:HGNC:1709]	12
ENSG00000090861	3105,217	-0,00766	0,556605	0,806152	-0,58789	0,020945	-0,01872	protein_coding	AARS1	alanyl-tRNA synthetase 1 [Source:HGNC Symbol;Acc:HGNC:20]	16
ENSG00000077232	4252,923	0,007055	0,557999	0,806505	0,585816	0,022964	0,024366	protein_coding	DNAJC10	DnaJ heat shock protein family (Hsp40) member C10 [Source:HGNC Symbol;Acc:HGNC:24637]	2
ENSG00000121579	2010,336	0,007505	0,557884	0,806505	0,585987	0,021506	0,019957	protein_coding	NAA50	N-alpha-acetyltransferase 50, NatE catalytic subunit [Source:HGNC Symbol;Acc:HGNC:29533]	3
ENSG00000046604	2713,317	0,003422	0,557362	0,806505	0,586765	0,025711	0,065576	protein_coding	DSG2	desmoglein 2 [Source:HGNC Symbol;Acc:HGNC:3049]	18
ENSG00000212232	2298,222	-0,00353	0,558441	0,806637	-0,58516	0,025664	-0,06223	snoRNA	SNORD17	small nucleolar RNA, C/D box 17 [Source:HGNC Symbol;Acc:HGNC:32713]	20
ENSG00000111252	5344,899	0,004578	0,55896	0,80688	0,584387	0,0253	0,046401	protein_coding	SH2B3	SH2B adaptor protein 3 [Source:HGNC Symbol;Acc:HGNC:29605]	12
ENSG00000200087	6051,69	-0,00366	0,559471	0,807112	-0,58363	0,025624	-0,05954	snoRNA	SNORA73B	small nucleolar RNA, H/ACA box 73B [Source:HGNC Symbol;Acc:HGNC:10116]	1
ENSG00000013375	1735,889	0,005159	0,560863	0,808106	0,58156	0,023592	0,028726	protein_coding	PGM3	phosphoglucomutase 3 [Source:HGNC Symbol;Acc:HGNC:8907]	6
ENSG00000023516	3400,229	-0,00701	0,560822	0,808106	-0,58162	0,022849	-0,02384	protein_coding	AKAP11	A-kinase anchoring protein 11 [Source:HGNC Symbol;Acc:HGNC:369]	13
ENSG00000123200	3345,338	0,00699	0,561849	0,808514	0,580098	0,022759	0,02348	protein_coding	ZC3H13	zinc finger CCCH-type containing 13 [Source:HGNC Symbol;Acc:HGNC:20368]	13
ENSG00000105357	1767,33	0,00424	0,561654	0,808514	0,580387	0,025418	0,049901	protein_coding	MYH14	myosin heavy chain 14 [Source:HGNC Symbol;Acc:HGNC:23212]	19
ENSG00000198752	5596,429	-0,00867	0,562741	0,809292	0,578775	0,023872	0,025709	protein_coding	CDC42BPB	CDC42 binding protein kinase beta [Source:HGNC Symbol;Acc:HGNC:1738]	14
ENSG00000204291	6479,215	0,001924	0,563705	0,809666	0,577348	0,025971	0,115928	protein_coding	COL15A1	collagen type XV alpha 1 chain [Source:HGNC Symbol;Acc:HGNC:2192]	9
ENSG00000198408	1955,424	0,005785	0,563508	0,809666	0,577639	0,023886	0,029362	protein_coding	OGA	O-GlcNAcase [Source:HGNC Symbol;Acc:HGNC:7056]	10
ENSG00000120451	2379,507	-0,00764	0,564481	0,810276	-0,5762	0,021645	-0,01988	protein_coding	SNX19	sorting nexin 19 [Source:HGNC Symbol;Acc:HGNC:21532]	11
ENSG00000171223	2769,42	0,004841	0,56488	0,810343	0,575608	0,025002	0,040292	protein_coding	JUNB	JunB proto-oncogene, AP-1 transcription factor subunit [Source:HGNC Symbol;Acc:HGNC:6205]	19
ENSG00000078369	8541,158	0,007141	0,568775	0,810875	0,569856	0,020649	0,017755	protein_coding	GNB1	G protein subunit beta 1 [Source:HGNC Symbol;Acc:HGNC:4396]	1
ENSG00000116406	2412,796	0,007232	0,566191	0,810875	0,57367	0,022142	0,021248	protein_coding	EDEM3	ER degradation enhancing alpha-mannosidase like protein 3 [Source:HGNC Symbol;Acc:HGNC:16787]	1
ENSG00000154305	3931,945	-0,00625	0,568409	0,810875	-0,5704	0,023108	-0,02491	protein_coding	MIA3	MIA SH3 domain ER export factor 3 [Source:HGNC Symbol;Acc:HGNC:24008]	1
ENSG00000150938	2782,659	0,004417	0,567227	0,810875	0,57214	0,025307	0,046193	protein_coding	CRIM1	cysteine rich transmembrane BMP regulator 1 [Source:HGNC Symbol;Acc:HGNC:2359]	2
ENSG00000206560	1714,194	0,005898	0,566677	0,810875	0,572952	0,024294	0,031437	protein_coding	ANKRD28	ankyrin repeat domain 28 [Source:HGNC Symbol;Acc:HGNC:29024]	3
ENSG00000177565	2796,379	0,007719	0,568567	0,810875	0,570162	0,022468	0,021697	protein_coding	TBL1XR1	TBL1X receptor 1 [Source:HGNC Symbol;Acc:HGNC:29529]	3
ENSG00000083857	12300,74	0,005219	0,568105	0,810875	0,570845	0,025089	0,039952	protein_coding	FAT1	FAT atypical cadherin 1 [Source:HGNC Symbol;Acc:HGNC:3595]	4

ENSG00000083642	1952,891	0,00699	0,566514	0,810875	0,573193	0,022829	0,023442	protein_coding	PDS5B	PDS5 cohesin associated factor B [Source:HGNC Symbol;Acc:HGNC:20418]	13
ENSG00000100697	2306,249	0,005554	0,567297	0,810875	0,572037	0,023409	0,026941	protein_coding	DICER1	dicer 1, ribonuclease III [Source:HGNC Symbol;Acc:HGNC:17098]	14
ENSG00000198899	109104,9	-0,00301	0,568174	0,810875	-0,57074	0,025764	-0,07037	protein_coding	MT-ATP6	mitochondrially encoded ATP synthase membrane subunit 6 [Source:HGNC Symbol;Acc:HGNC:7414]	MT
ENSG00000108510	3311,814	-0,00768	0,570553	0,812906	-0,56724	0,020472	-0,01715	protein_coding	MED13	mediator complex subunit 13 [Source:HGNC Symbol;Acc:HGNC:22474]	17
ENSG00000113384	2335,957	-0,00723	0,572843	0,813649	-0,56387	0,020918	-0,01806	protein_coding	GOLPH3	golgi phosphoprotein 3 [Source:HGNC Symbol;Acc:HGNC:15452]	5
ENSG00000070614	2711,699	0,006679	0,572793	0,813649	0,563942	0,023096	0,024241	protein_coding	NDST1	N-deacetylase and N-sulfotransferase 1 [Source:HGNC Symbol;Acc:HGNC:7680]	5
ENSG00000107771	1973,493	0,006133	0,572014	0,813649	0,565088	0,02394	0,028577	protein_coding	CCSER2	coiled-coil serine rich protein 2 [Source:HGNC Symbol;Acc:HGNC:29197]	10
ENSG00000140105	2621,35	0,007765	0,571493	0,813649	0,565854	0,024062	0,027007	protein_coding	WARS1	tryptophanyl-tRNA synthetase 1 [Source:HGNC Symbol;Acc:HGNC:12729]	14
ENSG00000114062	2165,324	0,006456	0,572561	0,813649	0,564284	0,023177	0,024753	protein_coding	UBE3A	ubiquitin protein ligase E3A [Source:HGNC Symbol;Acc:HGNC:12496]	15
ENSG00000168264	2152,684	0,006166	0,574728	0,81532	0,561102	0,022683	0,023058	protein_coding	IRF2BP2	interferon regulatory factor 2 binding protein 2 [Source:HGNC Symbol;Acc:HGNC:21729]	1
ENSG00000004399	3948,966	0,007057	0,574567	0,81532	0,561339	0,022421	0,021666	protein_coding	PLXND1	plexin D1 [Source:HGNC Symbol;Acc:HGNC:9107]	3
ENSG00000117139	1969,155	0,005455	0,578541	0,820224	0,555517	0,022254	0,021929	protein_coding	KDM5B	lysine demethylase 5B [Source:HGNC Symbol;Acc:HGNC:18039]	1
ENSG00000147010	5147,973	0,005987	0,579537	0,82113	0,554061	0,023937	0,028182	protein_coding	SH3KBP1	SH3 domain containing kinase binding protein 1 [Source:HGNC Symbol;Acc:HGNC:13867]	X
ENSG00000134243	2281,928	0,009345	0,580255	0,821334	0,553012	0,024124	0,024656	protein_coding	SORT1	sortilin 1 [Source:HGNC Symbol;Acc:HGNC:11186]	1
ENSG00000065308	2342,739	0,006895	0,580405	0,821334	0,552793	0,023832	0,026482	protein_coding	TRAM2	translocation associated membrane protein 2 [Source:HGNC Symbol;Acc:HGNC:16855]	6
ENSG00000049618	3163,983	-0,00747	0,580752	0,821334	-0,55229	0,020193	-0,01628	protein_coding	ARID1B	AT-rich interaction domain 1B [Source:HGNC Symbol;Acc:HGNC:18040]	6
ENSG00000174705	1848,195	0,006711	0,581935	0,821492	0,550561	0,02246	0,021586	protein_coding	SH3PXD2B	SH3 and PX domains 2B [Source:HGNC Symbol;Acc:HGNC:29242]	5
ENSG00000185591	2356,329	0,006832	0,581411	0,821492	0,551324	0,021898	0,020016	protein_coding	SP1	Sp1 transcription factor [Source:HGNC Symbol;Acc:HGNC:11205]	12
ENSG00000139874	1681,34	-0,00371	0,581621	0,821492	-0,55102	0,025506	-0,05246	protein_coding	SSTR1	somatostatin receptor 1 [Source:HGNC Symbol;Acc:HGNC:11330]	14
ENSG00000176903	1863,304	0,006723	0,583085	0,822611	0,548884	0,022283	0,021011	protein_coding	PNMA1	PNMA family member 1 [Source:HGNC Symbol;Acc:HGNC:9158]	14
ENSG00000120948	1924,498	0,006676	0,585104	0,824853	0,545944	0,022572	0,021748	protein_coding	TARDBP	TAR DNA binding protein [Source:HGNC Symbol;Acc:HGNC:11571]	1
ENSG00000115760	4750,615	-0,00735	0,585391	0,824853	-0,54553	0,021584	-0,01882	protein_coding	BIRC6	baculoviral IAP repeat containing 6 [Source:HGNC Symbol;Acc:HGNC:13516]	2
ENSG00000204463	3469,896	-0,0067	0,586116	0,825293	-0,54447	0,02189	-0,01982	protein_coding	BAG6	BAG cochaperone 6 [Source:HGNC Symbol;Acc:HGNC:13919]	6
ENSG00000140945	4824,542	-0,00414	0,586421	0,825293	-0,54403	0,025271	-0,04432	protein_coding	CDH13	cadherin 13 [Source:HGNC Symbol;Acc:HGNC:1753]	16
ENSG00000117713	3960,216	-0,00651	0,586853	0,825397	-0,5434	0,023198	-0,02387	protein_coding	ARID1A	AT-rich interaction domain 1A [Source:HGNC Symbol;Acc:HGNC:11110]	1
ENSG00000160752	1886,543	-0,00621	0,587602	0,825946	-0,54231	0,023613	-0,02577	protein_coding	FDPS	farnesyl diphosphate synthase [Source:HGNC Symbol;Acc:HGNC:3631]	1
ENSG00000106991	7133,446	0,005384	0,588854	0,826883	0,540498	0,024396	0,031142	protein_coding	ENG	endoglin [Source:HGNC Symbol;Acc:HGNC:3349]	9
ENSG00000073921	2829,18	0,007566	0,590066	0,826883	0,538741	0,023546	0,023943	protein_coding	PICALM	phosphatidylinositol binding clathrin assembly protein [Source:HGNC Symbol;Acc:HGNC:15514]	11
ENSG00000130779	2825,72	0,009284	0,58952	0,826883	0,539532	0,021813	0,01816	protein_coding	CLIP1	CAP-Gly domain containing linker protein 1 [Source:HGNC Symbol;Acc:HGNC:10461]	12
ENSG00000103769	2354,405	0,006036	0,589732	0,826883	0,539224	0,022686	0,022274	protein_coding	RAB11A	RAB11A, member RAS oncogene family [Source:HGNC Symbol;Acc:HGNC:9760]	15
ENSG00000125868	8758,581	0,007224	0,589517	0,826883	0,539536	0,024079	0,026543	protein_coding	DSTN	destrin, actin depolymerizing factor [Source:HGNC Symbol;Acc:HGNC:15750]	20
ENSG00000171316	1925,549	-0,00597	0,590538	0,82704	-0,53806	0,023969	-0,02759	protein_coding	CHD7	chromodomain helicase DNA binding protein 7 [Source:HGNC Symbol;Acc:HGNC:20626]	8
ENSG00000131446	2117,246	0,008312	0,594422	0,831558	0,532438	0,021763	0,018293	protein_coding	MGAT1	alpha-1,3-mannosyl-glycoprotein 2-beta-N-acetylglucosaminyltransferase [Source:HGNC Symbol;Acc:HGNC:7044]	5
ENSG00000157540	2127,08	-0,00764	0,594486	0,831558	-0,53235	0,02123	-0,0175	protein_coding	DYRK1A	dual specificity tyrosine phosphorylation regulated kinase 1A [Source:HGNC Symbol;Acc:HGNC:3091]	21
ENSG00000114251	2904,873	0,002484	0,595688	0,832227	0,530612	0,025321	0,050657	protein_coding	WNT5A	Wnt family member 5A [Source:HGNC Symbol;Acc:HGNC:12784]	3
ENSG00000149212	1595,707	0,004717	0,595665	0,832227	0,530645	0,024902	0,036378	protein_coding	SESN3	sestrin 3 [Source:HGNC Symbol;Acc:HGNC:23060]	11
ENSG00000136758	2577,492	0,006206	0,59616	0,832382	0,52993	0,023017	0,022872	protein_coding	YME1L1	YME1 like 1 ATPase [Source:HGNC Symbol;Acc:HGNC:12843]	10
ENSG00000135744	1587,748	-0,00227	0,597424	0,832739	-0,52811	0,02586	-0,08194	protein_coding	AGT	angiotensinogen [Source:HGNC Symbol;Acc:HGNC:333]	1
ENSG00000169567	1824,085	0,006512	0,596796	0,832739	0,529013	0,022835	0,021999	protein_coding	HINT1	histidine triad nucleotide binding protein 1 [Source:HGNC Symbol;Acc:HGNC:4912]	5
ENSG00000136205	6960,552	-0,00571	0,597502	0,832739	-0,528	0,023806	-0,02649	protein_coding	TNS3	tensin 3 [Source:HGNC Symbol;Acc:HGNC:21616]	7
ENSG00000116005	2214,104	0,005375	0,599113	0,833398	0,525676	0,024091	0,028369	protein_coding	PCYOX1	prenylcysteine oxidase 1 [Source:HGNC Symbol;Acc:HGNC:20588]	2
ENSG00000163430	9923,695	0,002622	0,598362	0,833398	0,526757	0,025778	0,069745	protein_coding	FSTL1	follistatin like 1 [Source:HGNC Symbol;Acc:HGNC:3972]	3
ENSG00000112378	1670,876	0,00366	0,599737	0,833398	0,524779	0,024513	0,034092	protein_coding	PERP	p53 apoptosis effector related to PMP22 [Source:HGNC Symbol;Acc:HGNC:17637]	6
ENSG00000159167	4407,32	0,00128	0,600009	0,833398	0,524388	0,026013	0,143214	protein_coding	STC1	stanniocalcin 1 [Source:HGNC Symbol;Acc:HGNC:11373]	8
ENSG00000094916	5121,274	0,009041	0,600148	0,833398	0,524188	0,023044	0,020508	protein_coding	CBX5	chromobox 5 [Source:HGNC Symbol;Acc:HGNC:1555]	12
ENSG00000074696	2198,298	0,006607	0,599851	0,833398	0,524614	0,021069	0,01732	protein_coding	HACD3	3-hydroxyacyl-CoA dehydratase 3 [Source:HGNC Symbol;Acc:HGNC:24175]	15
ENSG00000196586	2130,239	-0,00596	0,601075	0,834182	-0,52286	0,021828	-0,01923	protein_coding	MYO6	myosin VI [Source:HGNC Symbol;Acc:HGNC:7605]	6
ENSG00000095319	1769,976	-0,00829	0,601777	0,834357	-0,52185	0,022382	-0,01929	protein_coding	NUP188	nucleoporin 188 [Source:HGNC Symbol;Acc:HGNC:17859]	9
ENSG00000161544	3850,034	0,002255	0,601927	0,834357	0,521632	0,025855	0,080037	protein_coding	CYGB	cytoglobin [Source:HGNC Symbol;Acc:HGNC:16505]	17
ENSG00000162402	1792,913	-0,00636	0,602389	0,834495	-0,52097	0,022392	-0,02044	protein_coding	USP24	ubiquitin specific peptidase 24 [Source:HGNC Symbol;Acc:HGNC:12623]	1
ENSG00000163349	2085,378	-0,00643	0,607865	0,835778	-0,51312	0,021895	-0,01882	protein_coding	HIPK1	homeodomain interacting protein kinase 1 [Source:HGNC Symbol;Acc:HGNC:19006]	1
ENSG00000116584	2951,033	-0,00589	0,60974	0,835778	-0,51044	0,022331	-0,02012	protein_coding	ARHGEF2	Rho/Rac guanine nucleotide exchange factor 2 [Source:HGNC Symbol;Acc:HGNC:682]	1

ENSG00000116132	2222,516	0,002343	0,610216	0,835778	0,509766	0,025816	0,073441	protein_coding	PRRX1	paired related homeobox 1 [Source:HGNC Symbol;Acc:HGNC:9142]	1
ENSG00000117335	3550,625	-0,00605	0,606922	0,835778	-0,51447	0,023113	-0,02268	protein_coding	CD46	CD46 molecule [Source:HGNC Symbol;Acc:HGNC:6953]	1
ENSG00000169604	9310,75	-0,00533	0,607562	0,835778	-0,51356	0,02407	-0,02764	protein_coding	ANTXR1	ANTXR cell adhesion molecule 1 [Source:HGNC Symbol;Acc:HGNC:21014]	2
ENSG00000115762	2067,632	-0,00491	0,607955	0,835778	-0,513	0,022503	-0,02129	protein_coding	PLEKHB2	pleckstrin homology domain containing B2 [Source:HGNC Symbol;Acc:HGNC:19236]	2
ENSG00000115806	2130,259	0,006134	0,605547	0,835778	0,516439	0,02173	0,018676	protein_coding	GORASP2	golgi reassembly stacking protein 2 [Source:HGNC Symbol;Acc:HGNC:17500]	2
ENSG00000145730	8247,521	-0,00537	0,60871	0,835778	-0,51192	0,021721	-0,01883	protein_coding	PAM	peptidylglycine alpha-amidating monooxygenase [Source:HGNC Symbol;Acc:HGNC:8596]	5
ENSG00000187678	2881,555	-0,00604	0,608958	0,835778	-0,51156	0,022908	-0,02184	protein_coding	SPRY4	sprouty RTK signaling antagonist 4 [Source:HGNC Symbol;Acc:HGNC:15533]	5
ENSG00000113742	2025,207	-0,00473	0,607018	0,835778	-0,51433	0,023557	-0,02562	protein_coding	CPEB4	cytoplasmic polyadenylation element binding protein 4 [Source:HGNC Symbol;Acc:HGNC:21747]	5
ENSG00000136235	3058,607	0,003525	0,609709	0,835778	0,510488	0,025404	0,046569	protein_coding	GPNMB	glycoprotein nmb [Source:HGNC Symbol;Acc:HGNC:4462]	7
ENSG00000106609	2221,457	-0,00688	0,609422	0,835778	-0,5109	0,02048	-0,0157	protein_coding	TMEM248	transmembrane protein 248 [Source:HGNC Symbol;Acc:HGNC:25476]	7
ENSG00000196313	1599,004	-0,00536	0,609937	0,835778	-0,51016	0,023711	-0,02553	protein_coding	POM121	POM121 transmembrane nucleoporin [Source:HGNC Symbol;Acc:HGNC:19702]	7
ENSG00000148358	2609,041	-0,00718	0,604244	0,835778	-0,51831	0,020627	-0,0161	protein_coding	GPR107	G protein-coupled receptor 107 [Source:HGNC Symbol;Acc:HGNC:17830]	9
ENSG00000107731	4517,991	-0,00478	0,607376	0,835778	-0,51382	0,024617	-0,03227	protein_coding	UNC5B	unc-5 netrin receptor B [Source:HGNC Symbol;Acc:HGNC:12568]	10
ENSG00000111605	1634,937	0,005992	0,608967	0,835778	0,511549	0,021722	0,018552	protein_coding	CPSF6	cleavage and polyadenylation specific factor 6 [Source:HGNC Symbol;Acc:HGNC:13871]	12
ENSG00000198431	7311,573	-0,01118	0,606416	0,835778	-0,5152	0,025115	-0,02396	protein_coding	TXNRD1	thioredoxin reductase 1 [Source:HGNC Symbol;Acc:HGNC:12437]	12
ENSG00000170776	3974,955	0,00635	0,607459	0,835778	0,513704	0,02241	0,020217	protein_coding	AKAP13	A-kinase anchoring protein 13 [Source:HGNC Symbol;Acc:HGNC:371]	15
ENSG00000134590	1645,729	0,005735	0,606482	0,835778	0,515101	0,023566	0,024748	protein_coding	RTL8C	retrotransposon Gag like 8C [Source:HGNC Symbol;Acc:HGNC:2569]	X
ENSG00000124782	1892,657	-0,00554	0,612385	0,837751	-0,50667	0,022945	-0,0221	protein_coding	RREB1	ras responsive element binding protein 1 [Source:HGNC Symbol;Acc:HGNC:10449]	6
ENSG00000092820	2664,722	0,005907	0,612189	0,837751	0,506951	0,023578	0,024244	protein_coding	EZR	ezrin [Source:HGNC Symbol;Acc:HGNC:12691]	6
ENSG00000101596	2719,348	0,00652	0,613246	0,838431	0,505445	0,02109	0,016771	protein_coding	SMCHD1	structural maintenance of chromosomes flexible hinge domain containing 1 [Source:HGNC Symbol;Acc:HGNC:29090]	18
ENSG00000135956	2022,207	0,00618	0,616877	0,839415	0,500282	0,02171	0,018037	protein_coding	TMEM127	transmembrane protein 127 [Source:HGNC Symbol;Acc:HGNC:26038]	2
ENSG00000114354	2018,466	0,006138	0,615938	0,839415	0,501615	0,022241	0,019407	protein_coding	TFG	trafficking from ER to golgi regulator [Source:HGNC Symbol;Acc:HGNC:11758]	3
ENSG00000155506	7678,359	0,005601	0,614931	0,839415	0,503047	0,021176	0,017167	protein_coding	LARP1	La ribonucleoprotein 1, translational regulator [Source:HGNC Symbol;Acc:HGNC:29531]	5
ENSG00000153956	2822,255	0,004526	0,615261	0,839415	0,502578	0,024745	0,033278	protein_coding	CACNA2D1	calcium voltage-gated channel auxiliary subunit alpha2delta 1 [Source:HGNC Symbol;Acc:HGNC:1399]	7
ENSG00000187735	1722,57	0,006465	0,617979	0,839415	0,498717	0,020908	0,016213	protein_coding	TCEA1	transcription elongation factor A1 [Source:HGNC Symbol;Acc:HGNC:11612]	8
ENSG00000104549	2062,943	-0,00564	0,616377	0,839415	-0,50099	0,023474	-0,02377	protein_coding	SQLE	squalene epoxidase [Source:HGNC Symbol;Acc:HGNC:11279]	8
ENSG00000072042	1690,692	-0,00656	0,616586	0,839415	-0,50069	0,021544	-0,01752	protein_coding	RDH11	retinol dehydrogenase 11 [Source:HGNC Symbol;Acc:HGNC:17964]	14
ENSG00000069345	1598,506	0,005588	0,617952	0,839415	0,498755	0,02215	0,01933	protein_coding	DNAJA2	DnaJ heat shock protein family (Hsp40) member A2 [Source:HGNC Symbol;Acc:HGNC:14884]	16
ENSG00000108946	5579,732	-0,00695	0,616367	0,839415	-0,50101	0,020762	-0,01586	protein_coding	PRKAR1A	protein kinase cAMP-dependent type I regulatory subunit alpha [Source:HGNC Symbol;Acc:HGNC:9388]	17
ENSG00000124172	2772,149	0,005768	0,61719	0,839415	0,499837	0,023389	0,023261	protein_coding	ATP5F1E	ATP synthase F1 subunit epsilon [Source:HGNC Symbol;Acc:HGNC:838]	20
ENSG00000253352	2934,298	-0,00615	0,61762	0,839415	-0,49923	0,022429	-0,01981	protein_coding	TUG1	taurine up-regulated 1 [Source:HGNC Symbol;Acc:HGNC:26066]	22
ENSG00000167258	2300,875	0,006401	0,618669	0,839856	0,497738	0,02104	0,01645	protein_coding	CDK12	cyclin dependent kinase 12 [Source:HGNC Symbol;Acc:HGNC:24224]	17
ENSG00000135919	2861,89	-0,00244	0,619188	0,840066	-0,497	0,025752	-0,06528	protein_coding	SERPINE2	serpin family E member 2 [Source:HGNC Symbol;Acc:HGNC:8951]	2
ENSG00000101346	1945,003	0,005487	0,621134	0,84221	0,494243	0,023526	0,023813	protein_coding	POFUT1	protein O-fucosyltransferase 1 [Source:HGNC Symbol;Acc:HGNC:14988]	20
ENSG00000166974	2213,485	-0,00693	0,621797	0,842611	-0,49331	0,021586	-0,0172	protein_coding	MAPRE2	microtubule associated protein RP/EB family member 2 [Source:HGNC Symbol;Acc:HGNC:6891]	18
ENSG00000147526	3419,327	0,004855	0,622479	0,84304	0,492339	0,024324	0,028688	protein_coding	TACC1	transforming acidic coiled-coil containing protein 1 [Source:HGNC Symbol;Acc:HGNC:11522]	8
ENSG00000156453	2029,21	0,003908	0,623567	0,843455	0,490801	0,024782	0,034099	protein_coding	PCDH1	protocadherin 1 [Source:HGNC Symbol;Acc:HGNC:8655]	5
ENSG00000140545	2892,59	0,005405	0,623321	0,843455	0,491149	0,024095	0,026465	protein_coding	MFGE8	milk fat globule EGF and factor V/VIII domain containing [Source:HGNC Symbol;Acc:HGNC:7036]	15
ENSG00000104805	4089,25	0,007911	0,623885	0,843455	0,490351	0,024081	0,023311	protein_coding	NUCB1	nucleobindin 1 [Source:HGNC Symbol;Acc:HGNC:8043]	19
ENSG00000039523	1723,51	-0,00547	0,624782	0,843676	-0,48908	0,023549	-0,02367	protein_coding	RIPOR1	RHO family interacting cell polarization regulator 1 [Source:HGNC Symbol;Acc:HGNC:25836]	16
ENSG00000154856	2174,483	-0,00124	0,624682	0,843676	-0,48923	0,025999	-0,12824	protein_coding	APCDD1	APC down-regulated 1 [Source:HGNC Symbol;Acc:HGNC:15718]	18
ENSG00000050820	2729,079	0,00557	0,62522	0,843772	0,488466	0,023249	0,022356	protein_coding	BCAR1	BCAR1 scaffold protein, Cas family member [Source:HGNC Symbol;Acc:HGNC:971]	16
ENSG00000167110	2939,14	-0,00562	0,626165	0,844453	-0,48713	0,021933	-0,01832	protein_coding	GOLGA2	golgin A2 [Source:HGNC Symbol;Acc:HGNC:4425]	9
ENSG00000154096	2227,632	0,001665	0,626459	0,844453	0,486717	0,024308	0,031918	protein_coding	THY1	Thy-1 cell surface antigen [Source:HGNC Symbol;Acc:HGNC:11801]	11
ENSG00000197555	1671,453	-0,00599	0,628566	0,845543	-0,48375	0,02209	-0,01841	protein_coding	SIPA1L1	signal induced proliferation associated 1 like 1 [Source:HGNC Symbol;Acc:HGNC:20284]	14
ENSG00000129636	1684,32	-0,00532	0,628737	0,845543	-0,48351	0,023544	-0,02351	protein_coding	ITFG1	integrin alpha FG-GAP repeat containing 1 [Source:HGNC Symbol;Acc:HGNC:30697]	16
ENSG00000108256	3147,368	-0,00823	0,628418	0,845543	-0,48396	0,021997	-0,01712	protein_coding	NUFIP2	nuclear FMR1 interacting protein 2 [Source:HGNC Symbol;Acc:HGNC:17634]	17
ENSG00000171345	3525,377	0,001437	0,62772	0,845543	0,484938	0,025968	0,108378	protein_coding	KRT19	keratin 19 [Source:HGNC Symbol;Acc:HGNC:6436]	17
ENSG00000100731	2445,255	-0,00635	0,629945	0,846672	-0,4818	0,02159	-0,01704	protein_coding	PCNX1	pecanex 1 [Source:HGNC Symbol;Acc:HGNC:19740]	14
ENSG00000123636	1773,206	-0,00148	0,631982	0,846937	-0,47894	0,022427	-0,02086	protein_coding	BAZ2B	bromodomain adjacent to zinc finger domain 2B [Source:HGNC Symbol;Acc:HGNC:963]	2
ENSG00000114439	2090,795	-0,00578	0,630922	0,846937	-0,48043	0,022266	-0,01883	protein_coding	BBX	BBX high mobility group box domain containing [Source:HGNC Symbol;Acc:HGNC:14422]	3
ENSG00000167671	1646,231	0,005936	0,631791	0,846937	0,479208	0,02227	0,018711	protein_coding	UBXN6	UBX domain protein 6 [Source:HGNC Symbol;Acc:HGNC:14928]	19

ENSG00000104852	2442,23	-0,00421	0,631717	0,846937	-0,47931	0,022888	-0,02154	protein_coding	SNRNP70	small nuclear ribonucleoprotein U1 subunit 70 [Source:HGNC Symbol;Acc:HGNC:11150]	19
ENSG00000130066	2083,608	0,004417	0,631017	0,846937	0,480296	0,024299	0,028408	protein_coding	SAT1	spermidine/spermine N1-acetyltransferase 1 [Source:HGNC Symbol;Acc:HGNC:10540]	X
ENSG00000010256	1985,602	0,006141	0,633147	0,847018	0,477302	0,022186	0,018326	protein_coding	UQCRC1	ubiquinol-cytochrome c reductase core protein 1 [Source:HGNC Symbol;Acc:HGNC:12585]	3
ENSG00000120733	1964,459	-0,00618	0,632797	0,847018	-0,47779	0,022009	-0,01789	protein_coding	KDM3B	lysine demethylase 3B [Source:HGNC Symbol;Acc:HGNC:1337]	5
ENSG00000164754	3774,626	0,005826	0,633111	0,847018	0,477353	0,022707	0,019907	protein_coding	RAD21	RAD21 cohesin complex component [Source:HGNC Symbol;Acc:HGNC:9811]	8
ENSG00000008952	3342,008	0,005632	0,634772	0,847713	0,475021	0,022784	0,020161	protein_coding	SEC62	SEC62 homolog, preprotein translocation factor [Source:HGNC Symbol;Acc:HGNC:11846]	3
ENSG00000138757	3292,672	0,006063	0,634528	0,847713	0,475364	0,02091	0,015583	protein_coding	G3BP2	G3BP stress granule assembly factor 2 [Source:HGNC Symbol;Acc:HGNC:30291]	4
ENSG00000118482	2714,65	0,006183	0,634712	0,847713	0,475105	0,020579	0,014948	protein_coding	PHF3	PHD finger protein 3 [Source:HGNC Symbol;Acc:HGNC:8921]	6
ENSG00000013016	1732,153	0,005257	0,635957	0,848085	0,473359	0,023459	0,022725	protein_coding	EHD3	EH domain containing 3 [Source:HGNC Symbol;Acc:HGNC:3244]	2
ENSG00000169855	5287,105	0,004652	0,636524	0,848085	0,472564	0,023397	0,022908	protein_coding	ROBO1	roundabout guidance receptor 1 [Source:HGNC Symbol;Acc:HGNC:10249]	3
ENSG00000054965	2368,377	-0,00593	0,635689	0,848085	-0,47374	0,02271	-0,0197	protein_coding	FAM168A	family with sequence similarity 168 member A [Source:HGNC Symbol;Acc:HGNC:28999]	11
ENSG00000089159	2795,909	0,005663	0,636517	0,848085	0,472574	0,022603	0,019513	protein_coding	PXN	paxillin [Source:HGNC Symbol;Acc:HGNC:9718]	12
ENSG00000100941	2193,171	0,005523	0,637326	0,848661	0,471441	0,023238	0,021576	protein_coding	PNN	pinin, desmosome associated protein [Source:HGNC Symbol;Acc:HGNC:9162]	14
ENSG00000196235	3835,769	-0,00574	0,637719	0,848694	-0,47089	0,022207	-0,01831	protein_coding	SUPT5H	SPT5 homolog, DSIF elongation factor subunit [Source:HGNC Symbol;Acc:HGNC:11469]	19
ENSG00000124766	4679,501	0,005536	0,638826	0,849676	0,46934	0,022609	0,019452	protein_coding	SOX4	SRY-box transcription factor 4 [Source:HGNC Symbol;Acc:HGNC:11200]	6
ENSG00000130985	4621,274	0,005988	0,640087	0,850861	0,467577	0,021381	0,016243	protein_coding	UBA1	ubiquitin like modifier activating enzyme 1 [Source:HGNC Symbol;Acc:HGNC:12469]	X
ENSG00000092847	1637,695	-0,00496	0,643494	0,853087	-0,46282	0,023741	-0,02369	protein_coding	AGO1	argonaute RISC component 1 [Source:HGNC Symbol;Acc:HGNC:3262]	1
ENSG00000117385	2915,025	0,00556	0,642296	0,853087	0,464491	0,022322	0,018455	protein_coding	P3H1	prolyl 3-hydroxylase 1 [Source:HGNC Symbol;Acc:HGNC:19316]	1
ENSG00000169905	4146,291	0,006156	0,642608	0,853087	0,464056	0,022328	0,018169	protein_coding	TOR1AIP2	torsin 1A interacting protein 2 [Source:HGNC Symbol;Acc:HGNC:24055]	1
ENSG00000023228	2941,299	0,002641	0,643615	0,853087	0,46265	0,019642	0,01368	protein_coding	NDUFS1	NADH:ubiquinone oxidoreductase core subunit S1 [Source:HGNC Symbol;Acc:HGNC:7707]	2
ENSG00000063244	2545,13	-0,0058	0,643206	0,853087	-0,46322	0,021708	-0,01684	protein_coding	U2AF2	U2 small nuclear RNA auxiliary factor 2 [Source:HGNC Symbol;Acc:HGNC:23156]	19
ENSG00000166326	3229,731	0,006002	0,644761	0,853131	0,461053	0,021489	0,016226	protein_coding	TRIM44	tripartite motif containing 44 [Source:HGNC Symbol;Acc:HGNC:19016]	11
ENSG00000187498	48291,72	0,001753	0,644446	0,853131	0,461491	0,025887	0,080969	protein_coding	COL4A1	collagen type IV alpha 1 chain [Source:HGNC Symbol;Acc:HGNC:2202]	13
ENSG00000105568	2574,155	0,005984	0,644491	0,853131	0,461429	0,020932	0,015186	protein_coding	PPP2R1A	protein phosphatase 2 scaffold subunit Aalpha [Source:HGNC Symbol;Acc:HGNC:9302]	19
ENSG00000107372	3269,119	-0,0055	0,6473	0,855999	-0,45752	0,021918	-0,01722	protein_coding	ZFAND5	zinc finger AN1-type containing 5 [Source:HGNC Symbol;Acc:HGNC:13008]	9
ENSG00000108344	2090,746	0,005573	0,647929	0,856338	0,456641	0,021601	0,016454	protein_coding	PSMD3	proteasome 26S subunit, non-ATPase 3 [Source:HGNC Symbol;Acc:HGNC:9560]	17
ENSG00000071564	1574,873	0,005508	0,64845	0,856535	0,455916	0,023005	0,020093	protein_coding	TCF3	transcription factor 3 [Source:HGNC Symbol;Acc:HGNC:11633]	19
ENSG00000075292	2746,246	-0,00559	0,650294	0,857173	-0,45335	0,022	-0,01721	protein_coding	ZNF638	zinc finger protein 638 [Source:HGNC Symbol;Acc:HGNC:17894]	2
ENSG00000107554	1709,809	0,004688	0,650424	0,857173	0,453174	0,023951	0,024486	protein_coding	DNMBP	dynamin binding protein [Source:HGNC Symbol;Acc:HGNC:30373]	10
ENSG00000140937	3803,438	0,003491	0,649884	0,857173	0,453924	0,02512	0,036547	protein_coding	CDH11	cadherin 11 [Source:HGNC Symbol;Acc:HGNC:1750]	16
ENSG00000136448	2264,091	-0,00579	0,649895	0,857173	-0,45391	0,021636	-0,01635	protein_coding	NMT1	N-myristoyltransferase 1 [Source:HGNC Symbol;Acc:HGNC:7857]	17
ENSG00000139641	3427,529	0,005594	0,65218	0,857594	0,450736	0,022077	0,017293	protein_coding	ESYT1	extended synaptotagmin 1 [Source:HGNC Symbol;Acc:HGNC:29534]	12
ENSG00000156642	3077,918	-0,00524	0,651214	0,857594	-0,45208	0,021951	-0,0172	protein_coding	NPTN	neuroplastin [Source:HGNC Symbol;Acc:HGNC:17867]	15
ENSG00000149658	1591,893	-0,00529	0,65168	0,857594	-0,45143	0,022805	-0,01942	protein_coding	YTHDF1	YTH N6-methyladenosine RNA binding protein 1 [Source:HGNC Symbol;Acc:HGNC:15867]	20
ENSG00000185658	1616,135	-0,00472	0,652234	0,857594	-0,45066	0,024268	-0,02609	protein_coding	BRWD1	bromodomain and WD repeat domain containing 1 [Source:HGNC Symbol;Acc:HGNC:12760]	21
ENSG00000196821	2154,693	-0,00539	0,653495	0,857802	-0,44891	0,022613	-0,01869	protein_coding	ILRUN	inflammation and lipid regulator with UBA-like and NBR1-like domains [Source:HGNC Symbol;Acc:HGNC:21215]	6
ENSG00000197157	4873,592	0,005615	0,65351	0,857802	0,448891	0,020367	0,013923	protein_coding	SND1	staphylococcal nuclease and tudor domain containing 1 [Source:HGNC Symbol;Acc:HGNC:30646]	7
ENSG00000157106	2754,522	-0,0056	0,652868	0,857802	-0,44978	0,023635	-0,02199	protein_coding	SMG1	SMG1 nonsense mediated mRNA decay associated PI3K related kinase [Source:HGNC Symbol;Acc:HGNC:30045]	16
ENSG00000009335	2698,149	-0,00563	0,654312	0,858365	-0,44778	0,021574	-0,01607	protein_coding	UBE3C	ubiquitin protein ligase E3C [Source:HGNC Symbol;Acc:HGNC:16803]	7
ENSG00000134313	3266,299	0,005317	0,655872	0,859562	0,445619	0,022328	0,01784	protein_coding	KIDINS220	kinase D interacting substrate 220 [Source:HGNC Symbol;Acc:HGNC:29508]	2
ENSG00000153914	1660,704	-0,00475	0,655972	0,859562	-0,44548	0,023701	-0,0228	protein_coding	SREK1	splicing regulatory glutamic acid and lysine rich protein 1 [Source:HGNC Symbol;Acc:HGNC:17882]	5
ENSG00000012983	1572,651	0,004427	0,657119	0,86043	0,443894	0,023683	0,022924	protein_coding	MAP4K5	mitogen-activated protein kinase kinase kinase kinase 5 [Source:HGNC Symbol;Acc:HGNC:6867]	14
ENSG00000102226	1909,557	-0,00456	0,657382	0,86043	-0,44353	0,023271	-0,02108	protein_coding	USP11	ubiquitin specific peptidase 11 [Source:HGNC Symbol;Acc:HGNC:12609]	X
ENSG00000072364	5615,307	-0,00584	0,65787	0,86058	-0,44286	0,020397	-0,01374	protein_coding	AFF4	AF4/FMR2 family member 4 [Source:HGNC Symbol;Acc:HGNC:17869]	5
ENSG00000112249	2484,932	0,005321	0,660895	0,861806	0,438678	0,022466	0,017917	protein_coding	ASCC3	activating signal cointegrator 1 complex subunit 3 [Source:HGNC Symbol;Acc:HGNC:18697]	6
ENSG00000169398	2836,953	-0,00577	0,661429	0,861806	-0,43794	0,021627	-0,01577	protein_coding	PTK2	protein tyrosine kinase 2 [Source:HGNC Symbol;Acc:HGNC:9611]	8
ENSG00000182087	2368,516	-0,00567	0,660078	0,861806	-0,43981	0,021671	-0,01595	protein_coding	TMEM259	transmembrane protein 259 [Source:HGNC Symbol;Acc:HGNC:17039]	19
ENSG00000080573	2573,567	0,001494	0,661346	0,861806	0,438056	0,02592	0,086299	protein_coding	COL5A3	collagen type V alpha 3 chain [Source:HGNC Symbol;Acc:HGNC:14864]	19
ENSG00000171867	3685,138	-0,00362	0,659864	0,861806	-0,4401	0,024908	-0,03241	protein_coding	PRNP	prion protein [Source:HGNC Symbol;Acc:HGNC:9449]	20
ENSG00000171858	2426,741	0,005429	0,660699	0,861806	0,438948	0,02356	0,021325	protein_coding	RPS21	ribosomal protein S21 [Source:HGNC Symbol;Acc:HGNC:10409]	20
ENSG00000142192	18831,96	0,009269	0,66069	0,861806	0,438961	0,022082	0,015215	protein_coding	APP	amyloid beta precursor protein [Source:HGNC Symbol;Acc:HGNC:620]	21
ENSG00000138080	4797,909	0,003757	0,663409	0,863896	0,435212	0,024762	0,030267	protein_coding	EMILIN1	elastin microfibril interfacer 1 [Source:HGNC Symbol;Acc:HGNC:19880]	2

ENSG00000252316	3301,059	-0,00109	0,664023	0,864206	-0,43437	0,02599	-0,11376	misc_RNA	RNY4	RNA, Ro60-associated Y4 [Source:HGNC Symbol;Acc:HGNC:10244]	7
ENSG00000151779	1619,045	0,003911	0,665703	0,865414	0,432053	0,021389	0,01563	protein_coding	NBAS	NBAS subunit of NRZ tethering complex [Source:HGNC Symbol;Acc:HGNC:15625]	2
ENSG00000183741	3644,471	-0,00736	0,665613	0,865414	-0,43218	0,022309	-0,01629	protein_coding	CBX6	chromobox 6 [Source:HGNC Symbol;Acc:HGNC:1556]	22
ENSG00000198218	1750,04	0,004672	0,66697	0,866201	0,43031	0,022222	0,017223	protein_coding	QRICH1	glutamine rich 1 [Source:HGNC Symbol;Acc:HGNC:24713]	3
ENSG00000139644	7442,496	0,005014	0,667061	0,866201	0,430185	0,019405	0,012097	protein_coding	TMBIM6	transmembrane BAX inhibitor motif containing 6 [Source:HGNC Symbol;Acc:HGNC:11723]	12
ENSG00000152601	2917,507	0,004093	0,668629	0,866769	0,428031	0,023771	0,022775	protein_coding	MBNL1	muscleblind like splicing regulator 1 [Source:HGNC Symbol;Acc:HGNC:6923]	3
ENSG00000111321	2262,999	-0,00534	0,668411	0,866769	-0,42833	0,023674	-0,02131	protein_coding	LTBR	lymphotoxin beta receptor [Source:HGNC Symbol;Acc:HGNC:6718]	12
ENSG00000100412	1806,676	0,006014	0,667941	0,866769	0,428976	0,020601	0,013576	protein_coding	ACO2	aconitase 2 [Source:HGNC Symbol;Acc:HGNC:118]	22
ENSG00000138061	4941,202	-0,00374	0,670501	0,867242	-0,42546	0,024789	-0,02986	protein_coding	CYP1B1	cytochrome P450 family 1 subfamily B member 1 [Source:HGNC Symbol;Acc:HGNC:2597]	2
ENSG00000197226	3208,436	-0,00964	0,67043	0,867242	-0,42556	0,022651	-0,01555	protein_coding	TBC1D9B	TBC1 domain family member 9B [Source:HGNC Symbol;Acc:HGNC:29097]	5
ENSG00000242265	7240,402	-0,00314	0,66986	0,867242	-0,42634	0,025167	-0,0359	protein_coding	PEG10	paternally expressed 10 [Source:HGNC Symbol;Acc:HGNC:14005]	7
ENSG00000070081	2130,249	0,004514	0,669388	0,867242	0,426988	0,023861	0,022801	protein_coding	NUCB2	nucleobindin 2 [Source:HGNC Symbol;Acc:HGNC:8044]	11
ENSG00000145012	3054,444	0,003559	0,672854	0,867447	0,422235	0,024824	0,030371	protein_coding	LPP	LIM domain containing preferred translocation partner in lipoma [Source:HGNC Symbol;Acc:HGNC:6679]	3
ENSG00000117984	3951,391	-0,00384	0,672329	0,867447	-0,42295	0,024535	-0,02735	protein_coding	CTSD	cathepsin D [Source:HGNC Symbol;Acc:HGNC:2529]	11
ENSG00000166181	1640,472	0,005905	0,672566	0,867447	0,42263	0,022613	0,01735	protein_coding	API5	apoptosis inhibitor 5 [Source:HGNC Symbol;Acc:HGNC:594]	11
ENSG00000100888	3293,636	-0,00935	0,671367	0,867447	-0,42427	0,019939	-0,01177	protein_coding	CHD8	chromodomain helicase DNA binding protein 8 [Source:HGNC Symbol;Acc:HGNC:20153]	14
ENSG00000108828	3226,293	-0,00217	0,671842	0,867447	-0,42362	0,021609	-0,01616	protein_coding	VAT1	vesicle amine transport 1 [Source:HGNC Symbol;Acc:HGNC:16919]	17
ENSG00000158290	1612,346	0,005203	0,672922	0,867447	0,422142	0,021952	0,016072	protein_coding	CUL4B	cullin 4B [Source:HGNC Symbol;Acc:HGNC:2555]	X
ENSG00000061676	5260,285	0,005289	0,673638	0,867651	0,42116	0,022467	0,017221	protein_coding	NCKAP1	NCK associated protein 1 [Source:HGNC Symbol;Acc:HGNC:7666]	2
ENSG00000005700	1740,206	0,005031	0,674211	0,867651	0,420376	0,021973	0,01612	protein_coding	IBTK	inhibitor of Bruton tyrosine kinase [Source:HGNC Symbol;Acc:HGNC:17853]	6
ENSG00000101972	2351,14	0,00509	0,674085	0,867651	0,420548	0,022371	0,017039	protein_coding	STAG2	stromal antigen 2 [Source:HGNC Symbol;Acc:HGNC:11355]	X
ENSG00000169564	3501,474	0,005459	0,675726	0,869103	0,418302	0,021794	0,015494	protein_coding	PCBP1	poly(rC) binding protein 1 [Source:HGNC Symbol;Acc:HGNC:8647]	2
ENSG00000134824	2504,869	0,004332	0,676095	0,869103	0,417798	0,024659	0,027274	protein_coding	FADS2	fatty acid desaturase 2 [Source:HGNC Symbol;Acc:HGNC:3575]	11
ENSG00000079805	2718,84	-0,00538	0,676574	0,869234	-0,41714	0,021073	-0,01412	protein_coding	DNM2	dynamin 2 [Source:HGNC Symbol;Acc:HGNC:2974]	19
ENSG00000179262	2548,882	-0,00494	0,677086	0,869406	-0,41644	0,022199	-0,0165	protein_coding	RAD23A	RAD23 homolog A, nucleotide excision repair protein [Source:HGNC Symbol;Acc:HGNC:9812]	19
ENSG00000115084	2861,046	0,003863	0,679849	0,870649	0,412669	0,023226	0,019873	protein_coding	SLC35F5	solute carrier family 35 member F5 [Source:HGNC Symbol;Acc:HGNC:23617]	2
ENSG00000138443	1572,902	0,006519	0,679946	0,870649	0,412536	0,022774	0,017013	protein_coding	ABI2	abl interactor 2 [Source:HGNC Symbol;Acc:HGNC:24011]	2
ENSG00000122203	1568,282	0,005416	0,67905	0,870649	0,41376	0,022718	0,017494	protein_coding	KIAA1191	KIAA1191 [Source:HGNC Symbol;Acc:HGNC:29209]	5
ENSG00000019505	2655,755	0,002999	0,679659	0,870649	0,412928	0,02501	0,032911	protein_coding	SYT13	synaptotagmin 13 [Source:HGNC Symbol;Acc:HGNC:14962]	11
ENSG00000102910	1668,557	0,00466	0,678565	0,870649	0,414423	0,0213	0,014626	protein_coding	LONP2	lon peptidase 2, peroxisomal [Source:HGNC Symbol;Acc:HGNC:20598]	16
ENSG00000135821	2705,277	-0,00477	0,682804	0,870998	-0,40864	0,023929	-0,02189	protein_coding	GLUL	glutamate-ammonia ligase [Source:HGNC Symbol;Acc:HGNC:4341]	1
ENSG00000118257	2442,131	-0,0024	0,680714	0,870998	-0,41149	0,024216	-0,0258	protein_coding	NRP2	neuropilin 2 [Source:HGNC Symbol;Acc:HGNC:8005]	2
ENSG00000108055	2309,25	0,005167	0,68198	0,870998	0,409762	0,021553	0,014804	protein_coding	SMC3	structural maintenance of chromosomes 3 [Source:HGNC Symbol;Acc:HGNC:2468]	10
ENSG00000167522	5787,798	0,005764	0,682587	0,870998	0,408936	0,022987	0,017833	protein_coding	ANKRD11	ankyrin repeat domain 11 [Source:HGNC Symbol;Acc:HGNC:21316]	16
ENSG00000197879	4298,144	0,006494	0,681538	0,870998	0,410366	0,02375	0,019722	protein_coding	MYO1C	myosin IC [Source:HGNC Symbol;Acc:HGNC:7597]	17
ENSG00000118271	4008,218	0,000554	0,681249	0,870998	0,410759	0,026067	0,246031	protein_coding	TTR	transthyretin [Source:HGNC Symbol;Acc:HGNC:12405]	18
ENSG00000182670	6816,319	0,004477	0,682869	0,870998	0,408552	0,022147	0,016245	protein_coding	TTC3	tetratricopeptide repeat domain 3 [Source:HGNC Symbol;Acc:HGNC:12393]	21
ENSG00000198380	4946,527	-0,00527	0,685072	0,873324	-0,40555	0,020767	-0,01326	protein_coding	GFPT1	glutamine--fructose-6-phosphate transaminase 1 [Source:HGNC Symbol;Acc:HGNC:4241]	2
ENSG00000136383	1961,643	-0,00292	0,686564	0,874741	-0,40352	0,024681	-0,02856	protein_coding	ALPK3	alpha kinase 3 [Source:HGNC Symbol;Acc:HGNC:17574]	15
ENSG00000108175	4391,556	-0,00529	0,686989	0,874799	-0,40294	0,020507	-0,01277	protein_coding	ZMIZ1	zinc finger MIZ-type containing 1 [Source:HGNC Symbol;Acc:HGNC:16493]	10
ENSG00000200156	1677,746	-0,00082	0,688834	0,876269	-0,40044	0,026017	-0,12689	snRNA	RNU5B-1	RNA, U5B small nuclear 1 [Source:HGNC Symbol;Acc:HGNC:10212]	15
ENSG00000072778	5021,338	-0,00506	0,688905	0,876269	-0,40034	0,020554	-0,0128	protein_coding	ACADVL	acyl-CoA dehydrogenase very long chain [Source:HGNC Symbol;Acc:HGNC:92]	17
ENSG00000108021	2525,172	0,005259	0,689569	0,876362	0,39944	0,019777	0,011662	protein_coding	TASOR2	transcription activation suppressor family member 2 [Source:HGNC Symbol;Acc:HGNC:23484]	10
ENSG00000166197	2115,434	0,004662	0,69042	0,876362	0,398285	0,021233	0,013931	protein_coding	NOLC1	nucleolar and coiled-body phosphoprotein 1 [Source:HGNC Symbol;Acc:HGNC:15608]	10
ENSG00000100650	2037,387	0,00478	0,690502	0,876362	0,398174	0,022471	0,016494	protein_coding	SRSF5	serine and arginine rich splicing factor 5 [Source:HGNC Symbol;Acc:HGNC:10787]	14
ENSG00000260032	9035,159	0,00333	0,690124	0,876362	0,398687	0,02156	0,014881	lncRNA	NORAD	non-coding RNA activated by DNA damage [Source:HGNC Symbol;Acc:HGNC:44311]	20
ENSG00000143369	1614,817	0,002244	0,691675	0,877362	0,396583	0,025515	0,04367	protein_coding	ECM1	extracellular matrix protein 1 [Source:HGNC Symbol;Acc:HGNC:3153]	1
ENSG00000163453	15620,21	0,001929	0,692052	0,877362	0,396072	0,025711	0,053375	protein_coding	IGFBP7	insulin like growth factor binding protein 7 [Source:HGNC Symbol;Acc:HGNC:5476]	4
ENSG00000084674	2466,386	0,000464	0,692723	0,87767	0,395162	0,026063	0,234149	protein_coding	APOB	apolipoprotein B [Source:HGNC Symbol;Acc:HGNC:603]	2
ENSG00000086061	4465,616	0,004494	0,693058	0,87767	0,394708	0,021879	0,0151	protein_coding	DNAJA1	DnaJ heat shock protein family (Hsp40) member A1 [Source:HGNC Symbol;Acc:HGNC:5229]	9
ENSG00000129250	3581,833	0,006176	0,693992	0,877966	0,393443	0,021712	0,014188	protein_coding	KIF1C	kinesin family member 1C [Source:HGNC Symbol;Acc:HGNC:6317]	17
ENSG00000152234	4218,409	0,004133	0,694055	0,877966	0,393358	0,022447	0,016489	protein_coding	ATP5F1A	ATP synthase F1 subunit alpha [Source:HGNC Symbol;Acc:HGNC:823]	18

ENSG00000104723	1565,504	0,005392	0,694556	0,878117	0,39268	0,024211	0,021668	protein_coding	TUSC3	tumor suppressor candidate 3 [Source:HGNC Symbol;Acc:HGNC:30242]	8
ENSG00000068878	2048,278	0,004327	0,696366	0,879522	0,390231	0,021015	0,013356	protein_coding	PSME4	proteasome activator subunit 4 [Source:HGNC Symbol;Acc:HGNC:20635]	2
ENSG00000108671	2141,971	-0,00478	0,696431	0,879522	-0,39014	0,021757	-0,0146	protein_coding	PSMD11	proteasome 26S subunit, non-ATPase 11 [Source:HGNC Symbol;Acc:HGNC:9556]	17
ENSG00000142798	3199,887	-0,00342	0,697296	0,879622	-0,38897	0,024164	-0,02321	protein_coding	HSPG2	heparan sulfate proteoglycan 2 [Source:HGNC Symbol;Acc:HGNC:5273]	1
ENSG00000168710	3161,494	-0,00483	0,697658	0,879622	-0,38848	0,021583	-0,01418	protein_coding	AHCYL1	adenosylhomocysteinase like 1 [Source:HGNC Symbol;Acc:HGNC:344]	1
ENSG00000073614	1763,335	-0,00421	0,697152	0,879622	-0,38917	0,023437	-0,01929	protein_coding	KDM5A	lysine demethylase 5A [Source:HGNC Symbol;Acc:HGNC:9886]	12
ENSG00000151388	7812,39	0,002614	0,699427	0,88018	0,386094	0,025302	0,03605	protein_coding	ADAMTS12	ADAM metallopeptidase with thrombospondin type 1 motif 12 [Source:HGNC Symbol;Acc:HGNC:14605]	5
ENSG00000149115	3424,732	0,003682	0,699543	0,88018	0,385938	0,02321	0,01861	protein_coding	TNKS1BP1	tankyrase 1 binding protein 1 [Source:HGNC Symbol;Acc:HGNC:19081]	11
ENSG00000013275	2287,597	0,004443	0,69963	0,88018	0,38582	0,021032	0,013206	protein_coding	PSMC4	proteasome 26S subunit, ATPase 4 [Source:HGNC Symbol;Acc:HGNC:9551]	19
ENSG00000078747	2062,228	-0,00478	0,698983	0,88018	-0,38669	0,02202	-0,015	protein_coding	ITCH	itchy E3 ubiquitin protein ligase [Source:HGNC Symbol;Acc:HGNC:13890]	20
ENSG00000131504	3361,276	-0,00438	0,700756	0,880633	-0,3843	0,022941	-0,01735	protein_coding	DIAPH1	diaphanous related formin 1 [Source:HGNC Symbol;Acc:HGNC:2876]	5
ENSG00000125686	2157,342	0,00451	0,700662	0,880633	0,384426	0,02189	0,01473	protein_coding	MED1	mediator complex subunit 1 [Source:HGNC Symbol;Acc:HGNC:9234]	17
ENSG00000010244	1613,697	-0,00436	0,701951	0,881653	-0,38269	0,022905	-0,01718	protein_coding	ZNF207	zinc finger protein 207 [Source:HGNC Symbol;Acc:HGNC:12998]	17
ENSG00000203879	2423,09	0,004616	0,703506	0,883124	0,380592	0,022404	0,015675	protein_coding	GDI1	GDP dissociation inhibitor 1 [Source:HGNC Symbol;Acc:HGNC:4226]	X
ENSG00000168386	2140,273	-0,00276	0,706234	0,884796	-0,37692	0,025125	-0,03199	protein_coding	FILIP1L	filamin A interacting protein 1 like [Source:HGNC Symbol;Acc:HGNC:24589]	3
ENSG00000119707	2524,347	0,004672	0,705645	0,884796	0,377712	0,021583	0,013829	protein_coding	RBM25	RNA binding motif protein 25 [Source:HGNC Symbol;Acc:HGNC:23244]	14
ENSG00000138593	2305,578	-0,00572	0,706375	0,884796	-0,37673	0,020768	-0,01222	protein_coding	SECISBP2L	SECIS binding protein 2 like [Source:HGNC Symbol;Acc:HGNC:28997]	15
ENSG00000140526	10044,48	-0,00437	0,705231	0,884796	-0,37827	0,019476	-0,0108	protein_coding	ABHD2	abhydrolase domain containing 2, acylglycerol lipase [Source:HGNC Symbol;Acc:HGNC:18717]	15
ENSG00000234741	2026,44	0,001458	0,706777	0,884817	0,376188	0,025845	0,064235	lncRNA	GAS5	growth arrest specific 5 [Source:HGNC Symbol;Acc:HGNC:16355]	1
ENSG00000182326	3122,262	-0,00261	0,707179	0,884839	-0,37565	0,025237	-0,03405	protein_coding	C1S	complement C1s [Source:HGNC Symbol;Acc:HGNC:1247]	12
ENSG00000121068	4548,002	0,003411	0,707903	0,885264	0,374674	0,02446	0,024226	protein_coding	TBX2	T-box transcription factor 2 [Source:HGNC Symbol;Acc:HGNC:11597]	17
ENSG00000060749	1599,487	0,003946	0,70853	0,885566	0,373831	0,023382	0,018494	protein_coding	QSER1	glutamine and serine rich 1 [Source:HGNC Symbol;Acc:HGNC:26154]	11
ENSG00000143924	1682,818	0,004468	0,712999	0,885858	0,367832	0,022356	0,015084	protein_coding	EML4	EMAP like 4 [Source:HGNC Symbol;Acc:HGNC:1316]	2
ENSG00000163697	2241,913	-0,00278	0,709641	0,885858	-0,37234	0,022143	-0,01527	protein_coding	APBB2	amyloid beta precursor protein binding family B member 2 [Source:HGNC Symbol;Acc:HGNC:582]	4
ENSG00000163631	4301,059	0,000329	0,712034	0,885858	0,369126	0,026075	0,329796	protein_coding	ALB	albumin [Source:HGNC Symbol;Acc:HGNC:399]	4
ENSG00000231925	2761,743	0,004344	0,712972	0,885858	0,367867	0,022806	0,016244	protein_coding	TAPBP	TAP binding protein [Source:HGNC Symbol;Acc:HGNC:11566]	6
ENSG00000006715	1934,089	-0,00358	0,709207	0,885858	-0,37292	0,022339	-0,01556	protein_coding	VPS41	VPS41 subunit of HOPS complex [Source:HGNC Symbol;Acc:HGNC:12713]	7
ENSG00000023287	2338,01	-0,01134	0,711804	0,885858	0,369435	0,024972	0,016682	protein_coding	RB1CC1	RB1 inducible coiled-coil 1 [Source:HGNC Symbol;Acc:HGNC:15574]	8
ENSG00000151208	3200,07	-0,0059	0,711756	0,885858	-0,3695	0,022771	-0,01552	protein_coding	DLG5	discs large MAGUK scaffold protein 5 [Source:HGNC Symbol;Acc:HGNC:2904]	10
ENSG00000129167	2864,421	-0,00074	0,71247	0,885858	-0,36854	0,026017	-0,12082	protein_coding	TPH1	tryptophan hydroxylase 1 [Source:HGNC Symbol;Acc:HGNC:12008]	11
ENSG00000171067	1670,332	0,004627	0,710311	0,885858	0,371438	0,021842	0,014104	protein_coding	C11orf24	chromosome 11 open reading frame 24 [Source:HGNC Symbol;Acc:HGNC:1174]	11
ENSG00000123066	3222,706	-0,00482	0,712887	0,885858	-0,36798	0,019895	-0,01095	protein_coding	MED13L	mediator complex subunit 13L [Source:HGNC Symbol;Acc:HGNC:22962]	12
ENSG00000138594	2655,371	0,005688	0,711515	0,885858	0,369822	0,022754	0,015624	protein_coding	TMOD3	tropomodulin 3 [Source:HGNC Symbol;Acc:HGNC:11873]	15
ENSG00000168066	3656,511	0,008836	0,713599	0,886125	0,367027	0,022015	0,012789	protein_coding	SF1	splicing factor 1 [Source:HGNC Symbol;Acc:HGNC:12950]	11
ENSG00000175745	2610,289	0,003313	0,715141	0,887561	0,364961	0,02446	0,023712	protein_coding	NR2F1	nuclear receptor subfamily 2 group F member 1 [Source:HGNC Symbol;Acc:HGNC:7975]	5
ENSG00000196776	1654,118	0,00397	0,715962	0,887614	0,363861	0,023265	0,017603	protein_coding	CD47	CD47 molecule [Source:HGNC Symbol;Acc:HGNC:1682]	3
ENSG00000172667	1761,157	0,003333	0,716341	0,887614	0,363353	0,024516	0,02399	protein_coding	ZMAT3	zinc finger matrin-type 3 [Source:HGNC Symbol;Acc:HGNC:29983]	3
ENSG00000197081	10690,74	0,004605	0,716965	0,887614	0,362518	0,021127	0,012529	protein_coding	IGF2R	insulin like growth factor 2 receptor [Source:HGNC Symbol;Acc:HGNC:5467]	6
ENSG00000139793	2707,477	-0,00396	0,717498	0,887614	-0,3618	0,023586	-0,01862	protein_coding	MBNL2	muscleblind like splicing regulator 2 [Source:HGNC Symbol;Acc:HGNC:16746]	13
ENSG00000273749	2740,023	0,001928	0,717191	0,887614	-0,36222	0,019843	-0,01101	protein_coding	CYFIP1	cytoplasmic FMR1 interacting protein 1 [Source:HGNC Symbol;Acc:HGNC:13759]	15
ENSG00000199568	2873,345	-0,00077	0,715985	0,887614	-0,36383	0,026008	-0,11099	snRNA	RNU5A-1	RNA, U5A small nuclear 1 [Source:HGNC Symbol;Acc:HGNC:10211]	15
ENSG00000142208	2621,407	0,004161	0,718716	0,888644	0,360175	0,022553	0,015343	protein_coding	AKT1	AKT serine/threonine kinase 1 [Source:HGNC Symbol;Acc:HGNC:391]	14
ENSG00000082641	6170,59	-0,00493	0,719469	0,889097	-0,35917	0,0218	-0,01348	protein_coding	NFE2L1	nuclear factor, erythroid 2 like 1 [Source:HGNC Symbol;Acc:HGNC:7781]	17
ENSG00000158258	1725,876	-0,00193	0,722559	0,892436	-0,35504	0,025198	-0,03282	protein_coding	CLSTN2	calsyntenin 2 [Source:HGNC Symbol;Acc:HGNC:17448]	3
ENSG00000152952	4676,449	0,003143	0,725681	0,894521	0,350877	0,023858	0,019654	protein_coding	PLOD2	procollagen-lysine,2-oxoglutarate 5-dioxygenase 2 [Source:HGNC Symbol;Acc:HGNC:9082]	3
ENSG00000113648	4159,591	0,002333	0,726114	0,894521	0,3503	0,020127	0,010988	protein_coding	MACROH2A	macroH2A.1 histone [Source:HGNC Symbol;Acc:HGNC:4740]	5
ENSG00000113658	1585,98	-0,00377	0,725357	0,894521	-0,35131	0,02343	-0,01763	protein_coding	SMAD5	SMAD family member 5 [Source:HGNC Symbol;Acc:HGNC:6771]	5
ENSG00000125107	3799,234	-0,00444	0,725488	0,894521	-0,35113	0,020487	-0,01124	protein_coding	CNOT1	CCR4-NOT transcription complex subunit 1 [Source:HGNC Symbol;Acc:HGNC:7877]	16
ENSG00000131473	6025,875	0,004272	0,726191	0,894521	0,350197	0,02195	0,013597	protein_coding	ACLY	ATP citrate lyase [Source:HGNC Symbol;Acc:HGNC:115]	17
ENSG00000115183	2075,186	-0,00368	0,730842	0,896572	-0,34401	0,021166	-0,01212	protein_coding	TANC1	tetratricopeptide repeat, ankyrin repeat and coiled-coil containing 1 [Source:HGNC Symbol;Acc:HGNC:29364]	2
ENSG00000112096	2596,489	-0,00336	0,729736	0,896572	-0,34548	0,02408	-0,02023	protein_coding	SOD2	superoxide dismutase 2 [Source:HGNC Symbol;Acc:HGNC:11180]	6
ENSG00000137497	6445,107	-0,00436	0,730418	0,896572	-0,34457	0,021406	-0,01239	protein_coding	NUMA1	nuclear mitotic apparatus protein 1 [Source:HGNC Symbol;Acc:HGNC:8059]	11

ENSG00000111540	1893,381	-0,00523	0,728615	0,896572	-0,34697	0,022297	-0,01385	protein_coding	RAB5B	RAB5B, member RAS oncogene family [Source:HGNC Symbol;Acc:HGNC:9784]	12
ENSG00000075413	1666,666	0,004373	0,730973	0,896572	0,343831	0,021872	0,013177	protein_coding	MARK3	microtubule affinity regulating kinase 3 [Source:HGNC Symbol;Acc:HGNC:6897]	14
ENSG00000100991	1676,064	-0,00439	0,730299	0,896572	-0,34473	0,020878	-0,01159	protein_coding	TRPC4AP	transient receptor potential cation channel subfamily C member 4 associated protein [Source:HGNC Symbol;Acc:HGNC:	20
ENSG00000124151	2114,723	-0,00389	0,730552	0,896572	-0,34439	0,023006	-0,01593	protein_coding	NCOA3	nuclear receptor coactivator 3 [Source:HGNC Symbol;Acc:HGNC:7670]	20
ENSG00000160216	1834,877	0,004031	0,730808	0,896572	0,344051	0,021727	0,013004	protein_coding	AGPAT3	1-acylglycerol-3-phosphate O-acyltransferase 3 [Source:HGNC Symbol;Acc:HGNC:326]	21
ENSG00000018510	1700,045	-0,00379	0,733718	0,898514	-0,34018	0,023274	-0,01656	protein_coding	AGPS	alkylglycerone phosphate synthase [Source:HGNC Symbol;Acc:HGNC:327]	2
ENSG00000120738	4156,53	0,001341	0,733436	0,898514	0,340558	0,02583	0,05774	protein_coding	EGR1	early growth response 1 [Source:HGNC Symbol;Acc:HGNC:3238]	5
ENSG00000091879	2206,785	0,001088	0,733728	0,898514	0,34017	0,025914	0,070262	protein_coding	ANGPT2	angiopoietin 2 [Source:HGNC Symbol;Acc:HGNC:485]	8
ENSG00000118217	1787,704	-0,00315	0,736396	0,899388	-0,33663	0,021654	-0,01277	protein_coding	ATF6	activating transcription factor 6 [Source:HGNC Symbol;Acc:HGNC:791]	1
ENSG00000144724	1618,998	0,003542	0,736045	0,899388	0,337096	0,023587	0,017568	protein_coding	PTPRG	protein tyrosine phosphatase receptor type G [Source:HGNC Symbol;Acc:HGNC:9671]	3
ENSG00000263001	2489,592	-0,00413	0,735445	0,899388	-0,33789	0,022478	-0,01424	protein_coding	GTF2I	general transcription factor IIi [Source:HGNC Symbol;Acc:HGNC:4659]	7
ENSG00000080503	2341,195	-0,0044	0,735245	0,899388	-0,33816	0,021822	-0,01286	protein_coding	SMARCA2	SWI/SNF related, matrix associated, actin dependent regulator of chromatin, subfamily a, member 2 [Source:HGNC Syn	9
ENSG00000182220	1920,735	0,004544	0,735866	0,899388	0,337333	0,022498	0,014126	protein_coding	ATP6AP2	ATPase H+ transporting accessory protein 2 [Source:HGNC Symbol;Acc:HGNC:18305]	X
ENSG00000072840	1601,612	-0,00138	0,73708	0,899746	-0,33572	0,024632	-0,0247	protein_coding	EVC	EvC ciliary complex subunit 1 [Source:HGNC Symbol;Acc:HGNC:3497]	4
ENSG00000116679	2780,743	-0,00385	0,738705	0,900019	-0,33357	0,022682	-0,01463	protein_coding	IVNS1ABP	influenza virus NS1A binding protein [Source:HGNC Symbol;Acc:HGNC:16951]	1
ENSG00000135018	3022,54	0,004276	0,739203	0,900019	0,332909	0,020759	0,011048	protein_coding	UBQLN1	ubiquilin 1 [Source:HGNC Symbol;Acc:HGNC:12508]	9
ENSG00000171988	2713,374	-0,00414	0,740433	0,900019	-0,33128	0,021807	-0,01264	protein_coding	JMJD1C	jumonji domain containing 1C [Source:HGNC Symbol;Acc:HGNC:12313]	10
ENSG00000182934	3558,584	-0,00388	0,74021	0,900019	-0,33158	0,020148	-0,01027	protein_coding	SRPRA	SRP receptor subunit alpha [Source:HGNC Symbol;Acc:HGNC:11307]	11
ENSG00000175899	3011,229	0,001077	0,738325	0,900019	0,334072	0,025911	0,068787	protein_coding	A2M	alpha-2-macroglobulin [Source:HGNC Symbol;Acc:HGNC:7]	12
ENSG00000029725	2355,24	0,004148	0,738788	0,900019	0,333458	0,021495	0,012186	protein_coding	RABEP1	rabaptin, RAB GTPase binding effector protein 1 [Source:HGNC Symbol;Acc:HGNC:17677]	17
ENSG00000175387	2065,3	0,002784	0,739837	0,900019	0,332069	0,022118	0,01357	protein_coding	SMAD2	SMAD family member 2 [Source:HGNC Symbol;Acc:HGNC:6768]	18
ENSG00000064601	4035,155	0,004022	0,739406	0,900019	0,33264	0,022528	0,014158	protein_coding	CTSA	cathepsin A [Source:HGNC Symbol;Acc:HGNC:9251]	20
ENSG00000112078	1887,714	0,004254	0,741367	0,900203	0,330044	0,021961	0,012834	protein_coding	KCTD20	potassium channel tetramerization domain containing 20 [Source:HGNC Symbol;Acc:HGNC:21052]	6
ENSG00000170471	2269,214	0,003284	0,741317	0,900203	0,33011	0,021928	0,013008	protein_coding	RALGAPB	Ral GTPase activating protein non-catalytic subunit beta [Source:HGNC Symbol;Acc:HGNC:29221]	20
ENSG00000143162	2195,582	-0,00296	0,742085	0,9006	-0,32909	0,02314	-0,01593	protein_coding	CREG1	cellular repressor of E1A stimulated genes 1 [Source:HGNC Symbol;Acc:HGNC:2351]	1
ENSG00000150527	1690,155	-0,0033	0,74321	0,90149	-0,32761	0,023809	-0,01805	protein_coding	MIA2	MIA SH3 domain ER export factor 2 [Source:HGNC Symbol;Acc:HGNC:18432]	14
ENSG00000131724	1675,493	0,003882	0,744785	0,902924	0,325524	0,023009	0,015063	protein_coding	IL13RA1	interleukin 13 receptor subunit alpha 1 [Source:HGNC Symbol;Acc:HGNC:5974]	X
ENSG00000176658	5485,139	-0,00452	0,7457	0,903557	-0,32431	0,021926	-0,01248	protein_coding	MYO1D	myosin ID [Source:HGNC Symbol;Acc:HGNC:7598]	17
ENSG00000000971	2614,068	0,003322	0,746245	0,903742	0,323595	0,024588	0,021819	protein_coding	CFH	complement factor H [Source:HGNC Symbol;Acc:HGNC:4883]	1
ENSG00000128487	2787,37	0,003064	0,74702	0,904205	0,322571	0,023155	0,015629	protein_coding	SPECC1	sperm antigen with calponin homology and coiled-coil domains 1 [Source:HGNC Symbol;Acc:HGNC:30615]	17
ENSG00000165169	2204,432	0,003446	0,747724	0,904581	0,321642	0,023412	0,016235	protein_coding	DYNLT3	dynein light chain Tctex-type 3 [Source:HGNC Symbol;Acc:HGNC:11694]	X
ENSG00000182199	1996,484	0,004119	0,748134	0,904602	0,3211	0,024144	0,018552	protein_coding	SHMT2	serine hydroxymethyltransferase 2 [Source:HGNC Symbol;Acc:HGNC:10852]	12
ENSG00000068697	1847,511	0,003422	0,748822	0,904958	0,320193	0,023344	0,015954	protein_coding	LAPTM4A	lysosomal protein transmembrane 4 alpha [Source:HGNC Symbol;Acc:HGNC:6924]	2
ENSG00000087086	33397,71	-0,00895	0,74926	0,905012	-0,31961	0,025981	-0,02031	protein_coding	FTL	ferritin light chain [Source:HGNC Symbol;Acc:HGNC:3999]	19
ENSG00000137409	2050,692	-0,0042	0,75034	0,905366	-0,31819	0,021252	-0,01125	protein_coding	MTCH1	mitochondrial carrier 1 [Source:HGNC Symbol;Acc:HGNC:17586]	6
ENSG00000134278	1767,252	-0,00284	0,750069	0,905366	-0,31855	0,02248	-0,01378	protein_coding	SPIRE1	spire type actin nucleation factor 1 [Source:HGNC Symbol;Acc:HGNC:30622]	18
ENSG00000054793	3088,306	-0,0042	0,751433	0,90621	-0,31675	0,022715	-0,01384	protein_coding	ATP9A	ATPase phospholipid transporting 9A (putative) [Source:HGNC Symbol;Acc:HGNC:13540]	20
ENSG00000171603	5027,669	-0,01768	0,755713	0,90631	-0,31112	0,025884	-0,01142	protein_coding	CLSTN1	calsyntenin 1 [Source:HGNC Symbol;Acc:HGNC:17447]	1
ENSG00000158615	1671,356	0,003965	0,754601	0,90631	0,312578	0,02152	0,011499	protein_coding	PPP1R15B	protein phosphatase 1 regulatory subunit 15B [Source:HGNC Symbol;Acc:HGNC:14951]	1
ENSG00000164091	1878,875	0,003199	0,755459	0,90631	0,31145	0,022603	0,01366	protein_coding	WDR82	WD repeat domain 82 [Source:HGNC Symbol;Acc:HGNC:28826]	3
ENSG00000109685	1678,956	-0,00274	0,753297	0,90631	-0,3143	0,022957	-0,01479	protein_coding	NSD2	nuclear receptor binding SET domain protein 2 [Source:HGNC Symbol;Acc:HGNC:12766]	4
ENSG00000112992	1977,002	-0,0036	0,75553	0,90631	-0,31136	0,022503	-0,01333	protein_coding	NNT	nicotinamide nucleotide transhydrogenase [Source:HGNC Symbol;Acc:HGNC:7863]	5
ENSG00000213694	3177,986	-0,0012	0,753461	0,90631	-0,31408	0,025831	-0,05433	protein_coding	S1PR3	sphingosine-1-phosphate receptor 3 [Source:HGNC Symbol;Acc:HGNC:3167]	9
ENSG00000130559	1938,024	-0,00476	0,755849	0,90631	-0,31094	0,023458	-0,0152	protein_coding	CAMSAP1	calmodulin regulated spectrin associated protein 1 [Source:HGNC Symbol;Acc:HGNC:19946]	9
ENSG00000134900	1744,655	0,003393	0,75562	0,90631	0,311237	0,022575	0,013535	protein_coding	TPP2	tripeptidyl peptidase 2 [Source:HGNC Symbol;Acc:HGNC:12016]	13
ENSG00000100722	1732,242	-0,00399	0,753395	0,90631	-0,31417	0,020985	-0,01077	protein_coding	ZC3H14	zinc finger CCCH-type containing 14 [Source:HGNC Symbol;Acc:HGNC:20509]	14
ENSG00000141385	1741,779	-0,00413	0,752126	0,90631	-0,31584	0,021494	-0,01155	protein_coding	AFG3L2	AFG3 like matrix AAA peptidase subunit 2 [Source:HGNC Symbol;Acc:HGNC:315]	18
ENSG00000167642	2153,242	0,002763	0,755771	0,90631	0,311039	0,024368	0,020172	protein_coding	SPINT2	serine peptidase inhibitor, Kunitz type 2 [Source:HGNC Symbol;Acc:HGNC:11247]	19
ENSG00000087460	15728,11	0,003225	0,756567	0,906699	0,309991	0,020303	0,009857	protein_coding	GNAS	GNAS complex locus [Source:HGNC Symbol;Acc:HGNC:4392]	20
ENSG00000064042	2025,907	-0,00237	0,758643	0,906729	-0,30726	0,02492	-0,02437	protein_coding	LIMCH1	LIM and calponin homology domains 1 [Source:HGNC Symbol;Acc:HGNC:29191]	4
ENSG00000188994	1830,867	0,004491	0,757849	0,906729	0,308307	0,022459	0,012842	protein_coding	ZNF292	zinc finger protein 292 [Source:HGNC Symbol;Acc:HGNC:18410]	6
ENSG00000136868	1688,042	-0,0032	0,758441	0,906729	-0,30753	0,022902	-0,01419	protein_coding	SLC31A1	solute carrier family 31 member 1 [Source:HGNC Symbol;Acc:HGNC:11016]	9

ENSG00000107854	1643,26	0,003821	0,75832	0,906729	0,307687	0,020894	0,01045	protein_coding	TNKS2	tankyrase 2 [Source:HGNC Symbol;Acc:HGNC:15677]	10
ENSG00000183283	1924,585	-0,00364	0,75904	0,906729	-0,30674	0,022297	-0,0127	protein_coding	DAZAP2	DAZ associated protein 2 [Source:HGNC Symbol;Acc:HGNC:2684]	12
ENSG00000053747	3288,745	0,002001	0,759351	0,906729	0,306333	0,025304	0,029971	protein_coding	LAMA3	laminin subunit alpha 3 [Source:HGNC Symbol;Acc:HGNC:6483]	18
ENSG00000132002	1622,741	0,001353	0,757974	0,906729	0,308143	0,022853	0,014503	protein_coding	DNAJB1	DnaJ heat shock protein family (Hsp40) member B1 [Source:HGNC Symbol;Acc:HGNC:5270]	19
ENSG00000143420	1965,302	-0,00444	0,762687	0,907651	-0,30195	0,02261	-0,0129	protein_coding	ENSA	endosulfine alpha [Source:HGNC Symbol;Acc:HGNC:3360]	1
ENSG00000114999	2405,542	-0,0036	0,763633	0,907651	-0,30071	0,021887	-0,01172	protein_coding	TTL	tubulin tyrosine ligase [Source:HGNC Symbol;Acc:HGNC:21586]	2
ENSG00000165280	6588,877	-0,00392	0,764201	0,907651	-0,29997	0,018245	-0,00754	protein_coding	VCP	valosin containing protein [Source:HGNC Symbol;Acc:HGNC:12666]	9
ENSG00000062650	1903,638	0,003588	0,763474	0,907651	0,300922	0,021715	0,011444	protein_coding	WAPL	WAPL cohesin release factor [Source:HGNC Symbol;Acc:HGNC:23293]	10
ENSG00000171206	2565,251	-0,00492	0,763972	0,907651	-0,30027	0,022041	-0,01164	protein_coding	TRIM8	tripartite motif containing 8 [Source:HGNC Symbol;Acc:HGNC:15579]	10
ENSG00000255717	1721,799	-0,00076	0,763719	0,907651	-0,3006	0,025969	-0,0773	lncRNA	SNHG1	small nucleolar RNA host gene 1 [Source:HGNC Symbol;Acc:HGNC:32688]	11
ENSG00000084234	10802,23	0,003958	0,764462	0,907651	0,299626	0,022287	0,012303	protein_coding	APLP2	amyloid beta precursor like protein 2 [Source:HGNC Symbol;Acc:HGNC:598]	11
ENSG00000174197	1943,583	-0,00314	0,764212	0,907651	-0,29995	0,022734	-0,01345	protein_coding	MGA	MAX dimerization protein MGA [Source:HGNC Symbol;Acc:HGNC:14010]	15
ENSG00000125398	1867,756	-0,00342	0,763353	0,907651	-0,30108	0,023758	-0,01632	protein_coding	SOX9	SRY-box transcription factor 9 [Source:HGNC Symbol;Acc:HGNC:11204]	17
ENSG00000167491	3213,629	0,004017	0,761399	0,907651	0,303644	0,02052	0,009819	protein_coding	GATAD2A	GATA zinc finger domain containing 2A [Source:HGNC Symbol;Acc:HGNC:29989]	19
ENSG00000124486	5189,331	-0,0039	0,763954	0,907651	-0,30029	0,020713	-0,00996	protein_coding	USP9X	ubiquitin specific peptidase 9 X-linked [Source:HGNC Symbol;Acc:HGNC:12632]	X
ENSG00000116285	3924,691	0,003594	0,766218	0,909266	0,297326	0,022904	0,013596	protein_coding	ERRFI1	ERBB receptor feedback inhibitor 1 [Source:HGNC Symbol;Acc:HGNC:18185]	1
ENSG00000135535	3679,669	0,003352	0,766858	0,909556	0,296488	0,022869	0,013564	protein_coding	CD164	CD164 molecule [Source:HGNC Symbol;Acc:HGNC:1632]	6
ENSG00000159176	3270,746	0,003998	0,770738	0,909743	0,291409	0,022238	0,011865	protein_coding	CSRP1	cysteine and glycine rich protein 1 [Source:HGNC Symbol;Acc:HGNC:2469]	1
ENSG00000055332	1962,931	-0,0034	0,768313	0,909743	-0,29458	0,022613	-0,01288	protein_coding	EIF2AK2	eukaryotic translation initiation factor 2 alpha kinase 2 [Source:HGNC Symbol;Acc:HGNC:9437]	2
ENSG00000157020	2422,74	0,002967	0,769104	0,909743	0,293547	0,021724	0,011278	protein_coding	SEC13	SEC13 homolog, nuclear pore and COPII coat complex component [Source:HGNC Symbol;Acc:HGNC:10697]	3
ENSG00000173230	6341,312	-0,00391	0,770353	0,909743	-0,29191	0,020531	-0,00947	protein_coding	GOLGB1	golgin B1 [Source:HGNC Symbol;Acc:HGNC:4429]	3
ENSG00000173889	1650,92	-0,00377	0,770968	0,909743	-0,29111	0,02293	-0,01332	protein_coding	PHC3	polyhomeotic homolog 3 [Source:HGNC Symbol;Acc:HGNC:15682]	3
ENSG00000185963	1715,683	-0,00346	0,767751	0,909743	-0,29532	0,02245	-0,01256	protein_coding	BICD2	BICD cargo adaptor 2 [Source:HGNC Symbol;Acc:HGNC:17208]	9
ENSG00000133318	1965,731	-0,0039	0,769391	0,909743	-0,29317	0,021166	-0,0103	protein_coding	RTN3	reticulon 3 [Source:HGNC Symbol;Acc:HGNC:10469]	11
ENSG00000062485	1860,482	-0,00406	0,769767	0,909743	-0,29268	0,021333	-0,01048	protein_coding	CS	citrate synthase [Source:HGNC Symbol;Acc:HGNC:2422]	12
ENSG00000100647	1924,628	-0,00044	0,770386	0,909743	-0,29187	0,023005	-0,01423	protein_coding	SUSD6	sushi domain containing 6 [Source:HGNC Symbol;Acc:HGNC:19956]	14
ENSG00000169375	1944,178	-0,00349	0,769933	0,909743	-0,29246	0,020985	-0,01009	protein_coding	SIN3A	SIN3 transcription regulator family member A [Source:HGNC Symbol;Acc:HGNC:19353]	15
ENSG00000202354	2341,394	-0,00081	0,772426	0,910996	-0,2892	0,025944	-0,06801	misc_RNA	RNY3	RNA, Ro60-associated Y3 [Source:HGNC Symbol;Acc:HGNC:10243]	7
ENSG00000077782	4590,715	0,00425	0,773168	0,911403	0,288234	0,022061	0,011371	protein_coding	FGFR1	fibroblast growth factor receptor 1 [Source:HGNC Symbol;Acc:HGNC:3688]	8
ENSG00000100796	1771,933	-0,00487	0,774316	0,912289	-0,28673	0,022007	-0,01109	protein_coding	PPP4R3A	protein phosphatase 4 regulatory subunit 3A [Source:HGNC Symbol;Acc:HGNC:20219]	14
ENSG00000124831	4196,926	0,003395	0,775739	0,912564	0,284877	0,02208	0,011463	protein_coding	LRRFIP1	LRR binding FLII interacting protein 1 [Source:HGNC Symbol;Acc:HGNC:6702]	2
ENSG00000131620	2014,411	-0,00077	0,77545	0,912564	0,285254	0,02578	0,046704	protein_coding	ANO1	anoctamin 1 [Source:HGNC Symbol;Acc:HGNC:21625]	11
ENSG00000171681	1849,995	0,003184	0,775047	0,912564	0,28578	0,022406	0,012136	protein_coding	ATF7IP	activating transcription factor 7 interacting protein [Source:HGNC Symbol;Acc:HGNC:20092]	12
ENSG00000202538	5937,822	-0,00071	0,777052	0,913175	-0,28316	0,025969	-0,07362	snRNA	RNU4-2	RNA, U4 small nuclear 2 [Source:HGNC Symbol;Acc:HGNC:10193]	12
ENSG00000176915	2487,222	0,003697	0,776847	0,913175	0,28343	0,022913	0,012962	protein_coding	ANKLE2	ankyrin repeat and LEM domain containing 2 [Source:HGNC Symbol;Acc:HGNC:29101]	12
ENSG00000143799	2696,866	-0,00195	0,777541	0,913283	-0,28252	0,021536	-0,01069	protein_coding	PARP1	poly(ADP-ribose) polymerase 1 [Source:HGNC Symbol;Acc:HGNC:270]	1
ENSG00000145623	1689,528	0,002359	0,780196	0,91591	0,279064	0,024593	0,019659	protein_coding	OSMR	oncostatin M receptor [Source:HGNC Symbol;Acc:HGNC:8507]	5
ENSG00000149311	2232,578	-0,00314	0,780574	0,91591	-0,27857	0,023012	-0,01313	protein_coding	ATM	ATM serine/threonine kinase [Source:HGNC Symbol;Acc:HGNC:795]	11
ENSG00000152291	6356,91	0,005432	0,782631	0,917051	0,275893	0,02204	0,010573	protein_coding	TGOLN2	trans-golgi network protein 2 [Source:HGNC Symbol;Acc:HGNC:15450]	2
ENSG00000084090	2427,482	-0,0034	0,782321	0,917051	-0,2763	0,020793	-0,00932	protein_coding	STARD7	StAR related lipid transfer domain containing 7 [Source:HGNC Symbol;Acc:HGNC:18063]	2
ENSG00000197170	1741,506	0,001843	0,782741	0,917051	-0,27575	0,022226	-0,0116	protein_coding	PSMD12	proteasome 26S subunit, non-ATPase 12 [Source:HGNC Symbol;Acc:HGNC:9557]	17
ENSG00000170242	2035,944	0,003282	0,783268	0,917201	0,275062	0,021787	0,010617	protein_coding	USP47	ubiquitin specific peptidase 47 [Source:HGNC Symbol;Acc:HGNC:20076]	11
ENSG00000167470	2411,131	0,003091	0,783924	0,917502	0,274209	0,02338	0,013873	protein_coding	MIDN	midnolin [Source:HGNC Symbol;Acc:HGNC:16298]	19
ENSG00000011304	4871,266	-0,00374	0,78462	0,917849	-0,2733	0,02101	-0,00943	protein_coding	PTBP1	polypyrimidine tract binding protein 1 [Source:HGNC Symbol;Acc:HGNC:9583]	19
ENSG00000177733	1671,302	-0,00534	0,785849	0,918467	-0,27171	0,022068	-0,01048	protein_coding	HNRNPA0	heterogeneous nuclear ribonucleoprotein A0 [Source:HGNC Symbol;Acc:HGNC:5030]	5
ENSG00000203485	2441,291	0,000728	0,785946	0,918467	-0,27158	0,023247	-0,01389	protein_coding	INF2	inverted formin 2 [Source:HGNC Symbol;Acc:HGNC:23791]	14
ENSG00000112739	1778,475	0,0029	0,787602	0,919406	0,269426	0,023398	0,013749	protein_coding	PRPF4B	pre-mRNA processing factor 4B [Source:HGNC Symbol;Acc:HGNC:17346]	6
ENSG00000112379	3646,992	0,002142	0,787948	0,919406	0,268976	0,024823	0,020827	protein_coding	ARFGEF3	ARFGEF family member 3 [Source:HGNC Symbol;Acc:HGNC:21213]	6
ENSG00000141367	15957,16	0,003313	0,787768	0,919406	0,26921	0,021426	0,009877	protein_coding	CLTC	clathrin heavy chain [Source:HGNC Symbol;Acc:HGNC:2092]	17
ENSG00000087245	11507,99	0,002199	0,788465	0,919542	0,268305	0,024726	0,01991	protein_coding	MMP2	matrix metallopeptidase 2 [Source:HGNC Symbol;Acc:HGNC:7166]	16
ENSG00000176853	2196,54	-0,00307	0,789688	0,919715	-0,26672	0,022893	-0,01233	protein_coding	FAM91A1	family with sequence similarity 91 member A1 [Source:HGNC Symbol;Acc:HGNC:26306]	8
ENSG00000166340	1724,987	0,003066	0,790212	0,919715	0,266036	0,022592	0,011676	protein_coding	TPP1	tripeptidyl peptidase 1 [Source:HGNC Symbol;Acc:HGNC:2073]	11

ENSG00000119596	3320,689	-0,0026	0,789831	0,919715	-0,26653	0,021208	-0,00957	protein_coding	YLPM1	YLP motif containing 1 [Source:HGNC Symbol;Acc:HGNC:17798]	14
ENSG00000120063	1891,182	0,006821	0,789544	0,919715	0,266903	0,024229	0,013757	protein_coding	GNA13	G protein subunit alpha 13 [Source:HGNC Symbol;Acc:HGNC:4381]	17
ENSG00000111057	4145,412	0,002089	0,790622	0,919727	0,265503	0,024837	0,020584	protein_coding	KRT18	keratin 18 [Source:HGNC Symbol;Acc:HGNC:6430]	12
ENSG00000115419	2480,231	-0,00287	0,791624	0,919963	-0,2642	0,023167	-0,01289	protein_coding	GLS	glutaminase [Source:HGNC Symbol;Acc:HGNC:4331]	2
ENSG00000167635	1876,448	-0,00298	0,79155	0,919963	-0,2643	0,022076	-0,0107	protein_coding	ZNF146	zinc finger protein 146 [Source:HGNC Symbol;Acc:HGNC:12931]	19
ENSG00000198369	1816,452	0,002972	0,792206	0,92005	0,263448	0,022836	0,01207	protein_coding	SPRED2	sprouty related EVH1 domain containing 2 [Source:HGNC Symbol;Acc:HGNC:17722]	2
ENSG00000109572	2618,673	0,002565	0,792498	0,92005	0,263068	0,02066	0,0088	protein_coding	CLCN3	chloride voltage-gated channel 3 [Source:HGNC Symbol;Acc:HGNC:2021]	4
ENSG00000128245	1958,114	0,002922	0,793342	0,920565	0,261973	0,022857	0,012066	protein_coding	YWHAH	tyrosine 3-monooxygenase/tryptophan 5-monooxygenase activation protein eta [Source:HGNC Symbol;Acc:HGNC:1285	22
ENSG00000117707	2536,724	0,001561	0,79486	0,921396	0,260005	0,025401	0,027595	protein_coding	PROX1	prospero homeobox 1 [Source:HGNC Symbol;Acc:HGNC:9459]	1
ENSG00000163946	1998,7	0,003265	0,794584	0,921396	0,260363	0,021088	0,009136	protein_coding	TASOR	transcription activation suppressor [Source:HGNC Symbol;Acc:HGNC:30314]	3
ENSG00000180776	1966,128	0,003185	0,796056	0,921854	0,258455	0,023329	0,012915	protein_coding	ZDHHC20	zinc finger DHHC-type palmitoyltransferase 20 [Source:HGNC Symbol;Acc:HGNC:20749]	13
ENSG00000134871	45945,05	0,001071	0,796029	0,921854	0,258489	0,02578	0,041587	protein_coding	COL4A2	collagen type IV alpha 2 chain [Source:HGNC Symbol;Acc:HGNC:2203]	13
ENSG00000137776	2546,157	-0,00286	0,79745	0,923004	-0,25665	0,0227	-0,01151	protein_coding	SLTM	SAFB like transcription modulator [Source:HGNC Symbol;Acc:HGNC:20709]	15
ENSG00000159658	2389,792	0,002525	0,801684	0,924242	0,251168	0,021841	0,009875	protein_coding	EFCAB14	EF-hand calcium binding domain 14 [Source:HGNC Symbol;Acc:HGNC:29051]	1
ENSG00000143776	3508,423	0,002759	0,79917	0,924242	0,254421	0,022662	0,011354	protein_coding	CDC42BPA	CDC42 binding protein kinase alpha [Source:HGNC Symbol;Acc:HGNC:1737]	1
ENSG00000118985	1582,026	0,003541	0,801057	0,924242	0,251979	0,023449	0,012772	protein_coding	ELL2	elongation factor for RNA polymerase II 2 [Source:HGNC Symbol;Acc:HGNC:17064]	5
ENSG00000158604	1854,905	-0,0025	0,800343	0,924242	-0,2529	0,023016	-0,01208	protein_coding	TMED4	transmembrane p24 trafficking protein 4 [Source:HGNC Symbol;Acc:HGNC:22301]	7
ENSG00000155561	1598,273	0,003261	0,800251	0,924242	0,253022	0,022625	0,011123	protein_coding	NUP205	nucleoporin 205 [Source:HGNC Symbol;Acc:HGNC:18658]	7
ENSG00000149187	1927,21	-0,00293	0,800464	0,924242	-0,25275	0,021437	-0,00933	protein_coding	CELF1	CUGBP Elav-like family member 1 [Source:HGNC Symbol;Acc:HGNC:2549]	11
ENSG00000175727	2286,145	-0,00275	0,801512	0,924242	-0,25139	0,022665	-0,01123	protein_coding	MLXIP	MLX interacting protein [Source:HGNC Symbol;Acc:HGNC:17055]	12
ENSG00000100604	24427,96	0,00029	0,801733	0,924242	0,251106	0,02606	0,147254	protein_coding	CHGA	chromogranin A [Source:HGNC Symbol;Acc:HGNC:1929]	14
ENSG00000113721	13704,79	-0,00094	0,802927	0,925155	-0,24956	0,025823	-0,04376	protein_coding	PDGFRB	platelet derived growth factor receptor beta [Source:HGNC Symbol;Acc:HGNC:8804]	5
ENSG00000159346	2236,122	-0,00372	0,804065	0,925578	-0,24809	0,021818	-0,00955	protein_coding	ADIPOR1	adiponectin receptor 1 [Source:HGNC Symbol;Acc:HGNC:24040]	1
ENSG00000142186	2160,148	-0,00336	0,804099	0,925578	-0,24805	0,020771	-0,00834	protein_coding	SCYL1	SCY1 like pseudokinase 1 [Source:HGNC Symbol;Acc:HGNC:14372]	11
ENSG00000153207	1629,079	-0,00305	0,806834	0,927534	-0,24451	0,023188	-0,01193	protein_coding	AHCTF1	AT-hook containing transcription factor 1 [Source:HGNC Symbol;Acc:HGNC:24618]	1
ENSG00000003436	2605,319	-0,00222	0,807575	0,927534	-0,24356	0,024254	-0,01553	protein_coding	TFPI	tissue factor pathway inhibitor [Source:HGNC Symbol;Acc:HGNC:11760]	2
ENSG00000124788	1867,577	0,002465	0,807799	0,927534	0,243267	0,023304	0,012305	protein_coding	ATXN1	ataxin 1 [Source:HGNC Symbol;Acc:HGNC:10548]	6
ENSG00000160877	1900,296	0,003127	0,807813	0,927534	0,243248	0,022374	0,010287	protein_coding	NACC1	nucleus accumbens associated 1 [Source:HGNC Symbol;Acc:HGNC:20967]	19
ENSG00000100207	2758,019	-0,00341	0,806825	0,927534	-0,24452	0,021619	-0,00919	protein_coding	TCF20	transcription factor 20 [Source:HGNC Symbol;Acc:HGNC:11631]	22
ENSG00000154380	4864,848	0,003098	0,808362	0,927702	0,242539	0,021617	0,009163	protein_coding	ENAH	ENAH actin regulator [Source:HGNC Symbol;Acc:HGNC:18271]	1
ENSG00000172845	1892,262	-0,00328	0,810769	0,929214	-0,23943	0,020539	-0,00783	protein_coding	SP3	Sp3 transcription factor [Source:HGNC Symbol;Acc:HGNC:11208]	2
ENSG00000253729	9359,668	0,002298	0,810892	0,929214	0,239276	0,021936	0,009571	protein_coding	PRKDC	protein kinase, DNA-activated, catalytic subunit [Source:HGNC Symbol;Acc:HGNC:9413]	8
ENSG00000125970	3012,846	0,001406	0,810526	0,929214	0,239748	0,021575	0,009162	protein_coding	RALY	RALY heterogeneous nuclear ribonucleoprotein [Source:HGNC Symbol;Acc:HGNC:15921]	20
ENSG00000146247	2093,614	-0,00268	0,812142	0,929258	-0,23766	0,023183	-0,01169	protein_coding	PHIP	pleckstrin homology domain interacting protein [Source:HGNC Symbol;Acc:HGNC:15673]	6
ENSG00000107290	3825,714	0,003638	0,811887	0,929258	0,237993	0,020038	0,0073	protein_coding	SETX	senataxin [Source:HGNC Symbol;Acc:HGNC:445]	9
ENSG00000196611	10948,94	-0,00054	0,811368	0,929258	-0,23866	0,025986	-0,06847	protein_coding	MMP1	matrix metallopeptidase 1 [Source:HGNC Symbol;Acc:HGNC:7155]	11
ENSG00000162819	1592,584	-0,00317	0,81597	0,929438	-0,23273	0,023777	-0,01281	protein_coding	BROX	BRO1 domain and CAAX motif containing [Source:HGNC Symbol;Acc:HGNC:26512]	1
ENSG00000180370	2691,916	0,001731	0,815379	0,929438	0,233493	0,020749	0,007955	protein_coding	PAK2	p21 (RAC1) activated kinase 2 [Source:HGNC Symbol;Acc:HGNC:8591]	3
ENSG00000182158	3448,733	0,00285	0,816529	0,929438	0,232012	0,020145	0,007263	protein_coding	CREB3L2	cAMP responsive element binding protein 3 like 2 [Source:HGNC Symbol;Acc:HGNC:23720]	7
ENSG00000137575	2102,568	-0,0025	0,813588	0,929438	-0,2358	0,022935	-0,01111	protein_coding	SDCBP	syndecan binding protein [Source:HGNC Symbol;Acc:HGNC:10662]	8
ENSG00000048828	4070,417	-0,00235	0,813475	0,929438	-0,23595	0,01893	-0,00646	protein_coding	FAM120A	family with sequence similarity 120A [Source:HGNC Symbol;Acc:HGNC:13247]	9
ENSG00000110344	2066,945	0,00257	0,81684	0,929438	0,231611	0,021468	0,008625	protein_coding	UBE4A	ubiquitination factor E4A [Source:HGNC Symbol;Acc:HGNC:12499]	11
ENSG00000120686	1815,713	0,003187	0,816188	0,929438	0,23245	0,022816	0,010571	protein_coding	UFM1	ubiquitin fold modifier 1 [Source:HGNC Symbol;Acc:HGNC:20597]	13
ENSG00000102804	3634,426	0,003962	0,815522	0,929438	0,233308	0,020351	0,007399	protein_coding	TSC22D1	TSC22 domain family member 1 [Source:HGNC Symbol;Acc:HGNC:16826]	13
ENSG00000165801	1643,364	0,002266	0,817146	0,929438	0,231217	0,024304	0,014922	protein_coding	ARHGEF40	Rho guanine nucleotide exchange factor 40 [Source:HGNC Symbol;Acc:HGNC:25516]	14
ENSG00000166923	11791,49	0,001119	0,813647	0,929438	0,235724	0,025673	0,032867	protein_coding	GREM1	gremlin 1, DAN family BMP antagonist [Source:HGNC Symbol;Acc:HGNC:2001]	15
ENSG00000205937	2104,795	0,002868	0,814787	0,929438	0,234255	0,021774	0,009073	protein_coding	RNPS1	RNA binding protein with serine rich domain 1 [Source:HGNC Symbol;Acc:HGNC:10080]	16
ENSG00000203930	3405,8	0,001692	0,813979	0,929438	0,235296	0,025062	0,020516	lncRNA	LINC00632	long intergenic non-protein coding RNA 632 [Source:HGNC Symbol;Acc:HGNC:27865]	X
ENSG00000114302	1794,442	0,002815	0,818674	0,930615	0,229251	0,021594	0,008666	protein_coding	PRKAR2A	protein kinase cAMP-dependent type II regulatory subunit alpha [Source:HGNC Symbol;Acc:HGNC:9391]	3
ENSG00000164816	3207,969	4,24E-05	0,819394	0,930615	0,228324	0,026082	0,835427	protein_coding	DEFA5	defensin alpha 5 [Source:HGNC Symbol;Acc:HGNC:2764]	8
ENSG00000130477	3765,722	0,002802	0,819297	0,930615	0,228449	0,023437	0,011791	protein_coding	UNC13A	unc-13 homolog A [Source:HGNC Symbol;Acc:HGNC:23150]	19
ENSG00000106665	3395,294	0,002264	0,819994	0,930836	0,227553	0,023642	0,012417	protein_coding	CLIP2	CAP-Gly domain containing linker protein 2 [Source:HGNC Symbol;Acc:HGNC:2586]	7

ENSG00000152127	4204,624	0,004152	0,820538	0,930995	0,226853	0,022944	0,010303	protein_coding	MGAT5	alpha-1,6-mannosylglycoprotein 6-beta-N-acetylglucosaminyltransferase [Source:HGNC Symbol;Acc:HGNC:7049]	2
ENSG00000106799	1790,893	0,009825	0,824041	0,934049	0,22235	0,026341	0,014051	protein_coding	TGFBR1	transforming growth factor beta receptor 1 [Source:HGNC Symbol;Acc:HGNC:11772]	9
ENSG00000004897	2016,505	0,002431	0,823719	0,934049	0,222764	0,022846	0,010335	protein_coding	CDC27	cell division cycle 27 [Source:HGNC Symbol;Acc:HGNC:1728]	17
ENSG00000197746	20135,68	-0,00192	0,825117	0,934348	-0,22097	0,022244	-0,0093	protein_coding	PSAP	prosaposin [Source:HGNC Symbol;Acc:HGNC:9498]	10
ENSG00000179954	1792,395	0,002076	0,824728	0,934348	0,221468	0,024129	0,013676	protein_coding	SSC5D	scavenger receptor cysteine rich family member with 5 domains [Source:HGNC Symbol;Acc:HGNC:26641]	19
ENSG00000005022	2234,996	0,002797	0,825734	0,934586	0,220176	0,022999	0,010431	protein_coding	SLC25A5	solute carrier family 25 member 5 [Source:HGNC Symbol;Acc:HGNC:10991]	X
ENSG00000144136	9182,841	-0,00246	0,826278	0,934742	-0,21948	0,022778	-0,01004	protein_coding	SLC20A1	solute carrier family 20 member 1 [Source:HGNC Symbol;Acc:HGNC:10946]	2
ENSG00000108588	2336,324	0,002725	0,828067	0,936306	0,217181	0,021653	0,008287	protein_coding	CCDC47	coiled-coil domain containing 47 [Source:HGNC Symbol;Acc:HGNC:24856]	17
ENSG00000165637	1583,645	0,002607	0,828958	0,936392	0,216039	0,022007	0,008701	protein_coding	VDAC2	voltage dependent anion channel 2 [Source:HGNC Symbol;Acc:HGNC:12672]	10
ENSG00000108883	1653,777	0,002649	0,828749	0,936392	0,216307	0,022053	0,008768	protein_coding	EFTUD2	elongation factor Tu GTP binding domain containing 2 [Source:HGNC Symbol;Acc:HGNC:30858]	17
ENSG00000065882	2578,987	-0,00257	0,831009	0,937935	-0,21341	0,021798	-0,00833	protein_coding	TBC1D1	TBC1 domain family member 1 [Source:HGNC Symbol;Acc:HGNC:11578]	4
ENSG00000118816	4817,83	-0,00232	0,832842	0,937935	-0,21106	0,021689	-0,00814	protein_coding	CCNI	cyclin I [Source:HGNC Symbol;Acc:HGNC:1595]	4
ENSG00000112902	1969,643	0,001618	0,833176	0,937935	0,21063	0,024874	0,016905	protein_coding	SEMA5A	semaphorin 5A [Source:HGNC Symbol;Acc:HGNC:10736]	5
ENSG00000201098	2866,374	-0,00055	0,832322	0,937935	-0,21172	0,025952	-0,05275	misc_RNA	RNY1	RNA, Ro60-associated Y1 [Source:HGNC Symbol;Acc:HGNC:10242]	7
ENSG00000186318	2307,43	0,002563	0,832094	0,937935	0,212016	0,021801	0,008278	protein_coding	BACE1	beta-secretase 1 [Source:HGNC Symbol;Acc:HGNC:933]	11
ENSG00000166734	3912,188	-0,00388	0,833032	0,937935	-0,21082	0,022197	-0,00856	protein_coding	GOLM2	golgi membrane protein 2 [Source:HGNC Symbol;Acc:HGNC:24892]	15
ENSG00000264364	1640,526	0,002679	0,831499	0,937935	0,212779	0,02138	0,007816	protein_coding	DYNLL2	dynein light chain LC8-type 2 [Source:HGNC Symbol;Acc:HGNC:24596]	17
ENSG00000160710	5984,451	-0,00305	0,834117	0,938075	-0,20942	0,020215	-0,0066	protein_coding	ADAR	adenosine deaminase RNA specific [Source:HGNC Symbol;Acc:HGNC:225]	1
ENSG00000132466	4090,717	-0,00277	0,833797	0,938075	-0,20983	0,01839	-0,0054	protein_coding	ANKRD17	ankyrin repeat domain 17 [Source:HGNC Symbol;Acc:HGNC:23575]	4
ENSG00000170373	21729,01	0,000661	0,834552	0,938107	0,208867	0,025903	0,044701	protein_coding	CST1	cystatin SN [Source:HGNC Symbol;Acc:HGNC:2473]	20
ENSG00000064726	1834,206	0,002404	0,835249	0,938432	0,207974	0,022164	0,008607	protein_coding	BTBD1	BTB domain containing 1 [Source:HGNC Symbol;Acc:HGNC:1120]	15
ENSG00000067704	1707,234	0,002303	0,841964	0,939809	0,199381	0,022747	0,009104	protein_coding	IARS2	isoleucyl-tRNA synthetase 2, mitochondrial [Source:HGNC Symbol;Acc:HGNC:29685]	1
ENSG00000135916	2123,411	-0,00208	0,840519	0,939809	-0,20123	0,023472	-0,01061	protein_coding	ITM2C	integral membrane protein 2C [Source:HGNC Symbol;Acc:HGNC:6175]	2
ENSG00000204592	3117,435	0,003643	0,838214	0,939809	0,204179	0,023012	0,009508	protein_coding	HLA-E	major histocompatibility complex, class I, E [Source:HGNC Symbol;Acc:HGNC:4962]	6
ENSG00000110917	4120,635	0,0025	0,837001	0,939809	0,205731	0,020467	0,006718	protein_coding	MLEC	malectin [Source:HGNC Symbol;Acc:HGNC:28973]	12
ENSG00000140553	1668,22	0,002599	0,841922	0,939809	0,199436	0,022245	0,008336	protein_coding	UNC45A	unc-45 myosin chaperone A [Source:HGNC Symbol;Acc:HGNC:30594]	15
ENSG00000198231	2554,775	-0,00232	0,83926	0,939809	-0,20284	0,021457	-0,00756	protein_coding	DDX42	DEAD-box helicase 42 [Source:HGNC Symbol;Acc:HGNC:18676]	17
ENSG00000126934	2091,942	0,002687	0,837672	0,939809	0,204873	0,019841	0,006195	protein_coding	MAP2K2	mitogen-activated protein kinase kinase 2 [Source:HGNC Symbol;Acc:HGNC:6842]	19
ENSG00000105426	2975,875	-0,00156	0,841973	0,939809	-0,19937	0,023501	-0,01065	protein_coding	PTPRS	protein tyrosine phosphatase receptor type S [Source:HGNC Symbol;Acc:HGNC:9681]	19
ENSG00000129351	5159,489	0,003007	0,839198	0,939809	0,20292	0,020624	0,006725	protein_coding	ILF3	interleukin enhancer binding factor 3 [Source:HGNC Symbol;Acc:HGNC:6038]	19
ENSG00000125818	1980,935	-0,00336	0,839632	0,939809	-0,20236	0,020716	-0,00676	protein_coding	PSMF1	proteasome inhibitor subunit 1 [Source:HGNC Symbol;Acc:HGNC:9571]	20
ENSG00000089063	1740,812	0,001063	0,838422	0,939809	0,203912	0,021234	0,00744	protein_coding	TMEM230	transmembrane protein 230 [Source:HGNC Symbol;Acc:HGNC:15876]	20
ENSG00000124214	2960,479	0,002619	0,842085	0,939809	0,199227	0,021402	0,007346	protein_coding	STAU1	staufen double-stranded RNA binding protein 1 [Source:HGNC Symbol;Acc:HGNC:11370]	20
ENSG00000125534	2356,991	0,002044	0,841913	0,939809	0,199448	0,023407	0,010378	protein_coding	PPDPF	pancreatic progenitor cell differentiation and proliferation factor [Source:HGNC Symbol;Acc:HGNC:16142]	20
ENSG00000100364	1800,488	0,002327	0,842193	0,939809	0,199089	0,022443	0,008628	protein_coding	KIAA0930	KIAA0930 [Source:HGNC Symbol;Acc:HGNC:1314]	22
ENSG00000104093	2154,448	-0,00147	0,843049	0,940309	-0,19799	0,024092	-0,01226	protein_coding	DMXL2	Dmx like 2 [Source:HGNC Symbol;Acc:HGNC:2938]	15
ENSG00000132356	1713,436	-0,00177	0,844505	0,941476	-0,19613	0,022565	-0,00874	protein_coding	PRKAA1	protein kinase AMP-activated catalytic subunit alpha 1 [Source:HGNC Symbol;Acc:HGNC:9376]	5
ENSG00000013288	1582,586	-0,00218	0,845334	0,941525	-0,19508	0,022753	-0,00893	protein_coding	MAN2B2	mannosidase alpha class 2B member 2 [Source:HGNC Symbol;Acc:HGNC:29623]	4
ENSG00000160209	2314,181	0,002325	0,845368	0,941525	0,195032	0,022022	0,007897	protein_coding	PDXK	pyridoxal kinase [Source:HGNC Symbol;Acc:HGNC:8819]	21
ENSG00000099204	1794,64	-0,00171	0,848824	0,944917	-0,19062	0,024267	-0,01235	protein_coding	ABLIM1	actin binding LIM protein 1 [Source:HGNC Symbol;Acc:HGNC:78]	10
ENSG00000115310	6613,842	0,002311	0,85283	0,946143	0,185509	0,021182	0,006661	protein_coding	RTN4	reticulon 4 [Source:HGNC Symbol;Acc:HGNC:14085]	2
ENSG00000049656	2414,087	-0,00331	0,854037	0,946143	-0,18397	0,021104	-0,00646	protein_coding	CLPTM1L	CLPTM1 like [Source:HGNC Symbol;Acc:HGNC:24308]	5
ENSG00000198833	1738,646	-0,00255	0,853214	0,946143	-0,18502	0,022662	-0,00829	protein_coding	UBE2J1	ubiquitin conjugating enzyme E2 J1 [Source:HGNC Symbol;Acc:HGNC:17598]	6
ENSG00000136813	2741,81	-0,00196	0,852623	0,946143	-0,18577	0,022076	-0,00762	protein_coding	ECPAS	Ecm29 proteasome adaptor and scaffold [Source:HGNC Symbol;Acc:HGNC:29020]	9
ENSG00000149480	1731,074	0,002362	0,852769	0,946143	0,185587	0,021057	0,006546	protein_coding	MTA2	metastasis associated 1 family member 2 [Source:HGNC Symbol;Acc:HGNC:7411]	11
ENSG00000200795	1702,528	-0,00022	0,853973	0,946143	-0,18405	0,025958	-0,04811	snRNA	RNU4-1	RNA, U4 small nuclear 1 [Source:HGNC Symbol;Acc:HGNC:10192]	12
ENSG00000108582	4379,519	0,003581	0,851479	0,946143	0,187231	0,02127	0,006696	protein_coding	CPD	carboxypeptidase D [Source:HGNC Symbol;Acc:HGNC:2301]	17
ENSG00000198265	1788,753	0,002455	0,8536	0,946143	0,184527	0,021524	0,006935	protein_coding	HELZ	helicase with zinc finger [Source:HGNC Symbol;Acc:HGNC:16878]	17
ENSG00000081923	1671,046	-0,00153	0,851131	0,946143	-0,18768	0,024638	-0,01378	protein_coding	ATP8B1	ATPase phospholipid transporting 8B1 [Source:HGNC Symbol;Acc:HGNC:3706]	18
ENSG00000071553	2205,987	-0,00343	0,851913	0,946143	-0,18668	0,021726	-0,00713	protein_coding	ATP6AP1	ATPase H+ transporting accessory protein 1 [Source:HGNC Symbol;Acc:HGNC:868]	X
ENSG00000134352	19898,48	-0,00219	0,85482	0,946555	-0,18297	0,021874	-0,00726	protein_coding	IL6ST	interleukin 6 signal transducer [Source:HGNC Symbol;Acc:HGNC:6021]	5
ENSG00000125304	4250,929	-0,00248	0,855528	0,946883	-0,18207	0,022118	-0,00747	protein_coding	TM9SF2	transmembrane 9 superfamily member 2 [Source:HGNC Symbol;Acc:HGNC:11865]	13

ENSG00000161013	2814,648	-0,0021	0,857398	0,948041	-0,17969	0,023612	-0,00977	protein_coding	MGAT4B	alpha-1,3-mannosyl-glycoprotein 4-beta-N-acetylglucosaminyltransferase B [Source:HGNC Symbol;Acc:HGNC:7048]	5
ENSG00000140943	2964,691	-0,00117	0,857125	0,948041	-0,18004	0,02117	-0,0065	protein_coding	MBTPS1	membrane bound transcription factor peptidase, site 1 [Source:HGNC Symbol;Acc:HGNC:15456]	16
ENSG00000154864	4981,798	0,00123	0,85857	0,948881	0,178194	0,025121	0,016221	protein_coding	PIEZO2	piezo type mechanosensitive ion channel component 2 [Source:HGNC Symbol;Acc:HGNC:26270]	18
ENSG00000145833	2361,656	0,002764	0,859509	0,949006	0,177	0,022428	0,007605	protein_coding	DDX46	DEAD-box helicase 46 [Source:HGNC Symbol;Acc:HGNC:18681]	5
ENSG00000152767	3127,557	-0,00215	0,859143	0,949006	-0,17747	0,021585	-0,00675	protein_coding	FARP1	FERM, ARH/RhoGEF and pleckstrin domain protein 1 [Source:HGNC Symbol;Acc:HGNC:3591]	13
ENSG00000070214	2878,507	0,001437	0,860822	0,949508	0,175328	0,023365	0,009121	protein_coding	SLC44A1	solute carrier family 44 member 1 [Source:HGNC Symbol;Acc:HGNC:18798]	9
ENSG00000111371	3443,53	-0,0018	0,861201	0,949508	-0,17485	0,023455	-0,00923	protein_coding	SLC38A1	solute carrier family 38 member 1 [Source:HGNC Symbol;Acc:HGNC:13447]	12
ENSG00000171552	1959,262	0,001353	0,861008	0,949508	0,175091	0,021823	0,006947	protein_coding	BCL2L1	BCL2 like 1 [Source:HGNC Symbol;Acc:HGNC:992]	20
ENSG00000116754	2312,139	-0,00196	0,86205	0,949989	-0,17377	0,023854	-0,01003	protein_coding	SRSF11	serine and arginine rich splicing factor 11 [Source:HGNC Symbol;Acc:HGNC:10782]	1
ENSG00000149485	2799,353	-0,00236	0,862589	0,950128	-0,17308	0,02243	-0,00747	protein_coding	FADS1	fatty acid desaturase 1 [Source:HGNC Symbol;Acc:HGNC:3574]	11
ENSG00000157933	3049,027	-0,00173	0,863919	0,950172	-0,17139	0,022234	-0,00723	protein_coding	SKI	SKI proto-oncogene [Source:HGNC Symbol;Acc:HGNC:10896]	1
ENSG00000135932	1718,295	-0,00171	0,864281	0,950172	-0,17093	0,023737	-0,00964	protein_coding	CAB39	calcium binding protein 39 [Source:HGNC Symbol;Acc:HGNC:20292]	2
ENSG00000077147	4617,268	0,001927	0,863586	0,950172	0,171812	0,022586	0,007667	protein_coding	TM9SF3	transmembrane 9 superfamily member 3 [Source:HGNC Symbol;Acc:HGNC:21529]	10
ENSG00000136436	1892,968	-0,00166	0,863593	0,950172	-0,1718	0,022609	-0,00772	protein_coding	CALCOCO2	calcium binding and coiled-coil domain 2 [Source:HGNC Symbol;Acc:HGNC:29912]	17
ENSG00000173166	1810,26	0,001517	0,86583	0,951421	0,168957	0,023375	0,008811	protein_coding	RAPH1	Ras association (RalGDS/AF-6) and pleckstrin homology domains 1 [Source:HGNC Symbol;Acc:HGNC:14436]	2
ENSG00000143157	1968,562	-0,00143	0,866747	0,951519	-0,16779	0,021582	-0,00642	protein_coding	POGK	pogo transposable element derived with KRAB domain [Source:HGNC Symbol;Acc:HGNC:18800]	1
ENSG00000177189	2160,291	-0,00193	0,866418	0,951519	-0,16821	0,0226	-0,00752	protein_coding	RPS6KA3	ribosomal protein S6 kinase A3 [Source:HGNC Symbol;Acc:HGNC:10432]	X
ENSG00000115464	4182,627	0,001932	0,867489	0,951662	0,166848	0,020767	0,005688	protein_coding	USP34	ubiquitin specific peptidase 34 [Source:HGNC Symbol;Acc:HGNC:20066]	2
ENSG00000110090	1824,874	-0,00158	0,867704	0,951662	-0,16658	0,023832	-0,00963	protein_coding	CPT1A	carnitine palmitoyltransferase 1A [Source:HGNC Symbol;Acc:HGNC:2328]	11
ENSG00000072274	3080,374	0,001678	0,868313	0,951876	0,165802	0,022423	0,007214	protein_coding	TFRC	transferrin receptor [Source:HGNC Symbol;Acc:HGNC:11763]	3
ENSG00000168542	52893,6	0,001422	0,869451	0,952362	0,164356	0,02446	0,011389	protein_coding	COL3A1	collagen type III alpha 1 chain [Source:HGNC Symbol;Acc:HGNC:2201]	2
ENSG00000149639	3752,748	-0,00186	0,869998	0,952362	-0,16366	0,022411	-0,00709	protein_coding	SOGA1	suppressor of glucose, autophagy associated 1 [Source:HGNC Symbol;Acc:HGNC:16111]	20
ENSG00000215301	7176,365	-0,00218	0,869965	0,952362	-0,1637	0,018172	-0,00413	protein_coding	DDX3X	DEAD-box helicase 3 X-linked [Source:HGNC Symbol;Acc:HGNC:2745]	X
ENSG00000008294	3702,529	-0,00212	0,870459	0,952414	-0,16308	0,020124	-0,00512	protein_coding	SPAG9	sperm associated antigen 9 [Source:HGNC Symbol;Acc:HGNC:14524]	17
ENSG00000116539	3680,378	-0,00214	0,87127	0,952478	-0,16205	0,019939	-0,00498	protein_coding	ASH1L	ASH1 like histone lysine methyltransferase [Source:HGNC Symbol;Acc:HGNC:19088]	1
ENSG00000263934	4721,513	-0,00058	0,871346	0,952478	-0,16195	0,025826	-0,02918	snoRNA	SNORD3A	small nucleolar RNA, C/D box 3A [Source:HGNC Symbol;Acc:HGNC:33189]	17
ENSG00000089248	2232,419	0,003817	0,872113	0,952864	0,160976	0,024003	0,009102	protein_coding	ERP29	endoplasmic reticulum protein 29 [Source:HGNC Symbol;Acc:HGNC:13799]	12
ENSG00000137145	1718,246	0,002266	0,872871	0,953239	0,160013	0,0217	0,006181	protein_coding	DENND4C	DENN domain containing 4C [Source:HGNC Symbol;Acc:HGNC:26079]	9
ENSG00000168724	1725,362	0,001648	0,874431	0,954141	0,158033	0,0217	0,006141	protein_coding	DNAJC21	DnaJ heat shock protein family (Hsp40) member C21 [Source:HGNC Symbol;Acc:HGNC:27030]	5
ENSG00000064115	2239,994	0,001453	0,874525	0,954141	0,157913	0,021989	0,006415	protein_coding	TM7SF3	transmembrane 7 superfamily member 3 [Source:HGNC Symbol;Acc:HGNC:23049]	12
ENSG00000114554	4420,489	0,001945	0,876361	0,954658	0,155584	0,021957	0,006263	protein_coding	PLXNA1	plexin A1 [Source:HGNC Symbol;Acc:HGNC:9099]	3
ENSG00000133110	10445,89	-0,0006	0,876519	0,954658	-0,15538	0,025783	-0,02597	protein_coding	POSTN	periostin [Source:HGNC Symbol;Acc:HGNC:16953]	13
ENSG00000141279	1658,815	-0,00194	0,876659	0,954658	-0,15521	0,022127	-0,00642	protein_coding	NPEPPS	aminopeptidase puromycin sensitive [Source:HGNC Symbol;Acc:HGNC:7900]	17
ENSG00000228253	34638,74	-0,00112	0,876253	0,954658	-0,15572	0,024979	-0,01329	protein_coding	MT-ATP8	mitochondrially encoded ATP synthase membrane subunit 8 [Source:HGNC Symbol;Acc:HGNC:7415]	MT
ENSG00000184216	3080,963	-0,00288	0,877467	0,955085	-0,15418	0,020253	-0,00489	protein_coding	IRAK1	interleukin 1 receptor associated kinase 1 [Source:HGNC Symbol;Acc:HGNC:6112]	X
ENSG00000108349	2356,886	0,001849	0,878418	0,955668	0,152976	0,02175	0,005975	protein_coding	CASC3	CASC3 exon junction complex subunit [Source:HGNC Symbol;Acc:HGNC:17040]	17
ENSG00000130816	3123,951	-0,00201	0,880264	0,957225	-0,15063	0,021136	-0,00539	protein_coding	DNMT1	DNA methyltransferase 1 [Source:HGNC Symbol;Acc:HGNC:2976]	19
ENSG00000148672	2187,808	0,000894	0,881053	0,95763	0,149634	0,021009	0,005297	protein_coding	GLUD1	glutamate dehydrogenase 1 [Source:HGNC Symbol;Acc:HGNC:4335]	10
ENSG00000197956	11456,9	0,001235	0,881556	0,957724	0,148997	0,022391	0,006472	protein_coding	S100A6	S100 calcium binding protein A6 [Source:HGNC Symbol;Acc:HGNC:10496]	1
ENSG00000177311	4246,702	0,001568	0,884279	0,958871	0,145547	0,020317	0,004698	protein_coding	ZBTB38	zinc finger and BTB domain containing 38 [Source:HGNC Symbol;Acc:HGNC:26636]	3
ENSG00000127914	4340,72	0,001762	0,88417	0,958871	0,145685	0,02173	0,005679	protein_coding	AKAP9	A-kinase anchoring protein 9 [Source:HGNC Symbol;Acc:HGNC:379]	7
ENSG00000222414	2210,155	-0,0005	0,883336	0,958871	-0,14674	0,025847	-0,02769	snRNA	RNU2-59P	RNA, U2 small nuclear 59, pseudogene [Source:HGNC Symbol;Acc:HGNC:48552]	10
ENSG00000103512	2339,861	-0,00156	0,883504	0,958871	-0,14653	0,023375	-0,00764	protein_coding	NOMO1	NODAL modulator 1 [Source:HGNC Symbol;Acc:HGNC:30060]	16
ENSG00000116688	1782,608	-0,00148	0,88624	0,959707	-0,14306	0,02238	-0,00619	protein_coding	MFN2	mitofusin 2 [Source:HGNC Symbol;Acc:HGNC:16877]	1
ENSG00000138246	2475,046	5,92E-05	0,886244	0,959707	0,143059	0,022536	0,006401	protein_coding	DNAJC13	DnaJ heat shock protein family (Hsp40) member C13 [Source:HGNC Symbol;Acc:HGNC:30343]	3
ENSG00000156976	4004,385	0,001826	0,886301	0,959707	0,142986	0,021536	0,005418	protein_coding	EIF4A2	eukaryotic translation initiation factor 4A2 [Source:HGNC Symbol;Acc:HGNC:3284]	3
ENSG00000058085	3598,766	-0,00055	0,88684	0,95984	-0,1423	0,025791	-0,02422	protein_coding	LAMC2	laminin subunit gamma 2 [Source:HGNC Symbol;Acc:HGNC:6493]	1
ENSG00000168159	1779,131	-0,00116	0,887884	0,960518	-0,14098	0,022839	-0,00663	protein_coding	RNF187	ring finger protein 187 [Source:HGNC Symbol;Acc:HGNC:27146]	1
ENSG00000116977	1724,677	0,001704	0,889229	0,960961	0,13928	0,021513	0,005262	protein_coding	LGALS8	galectin 8 [Source:HGNC Symbol;Acc:HGNC:6569]	1
ENSG00000124299	1700,973	0,001183	0,889116	0,960961	0,139423	0,023114	0,00692	protein_coding	PEPD	peptidase D [Source:HGNC Symbol;Acc:HGNC:8840]	19
ENSG00000100219	3976,998	0,001089	0,889547	0,960961	0,138878	0,022832	0,006529	protein_coding	XBP1	X-box binding protein 1 [Source:HGNC Symbol;Acc:HGNC:12801]	22
ENSG00000112419	3704,422	0,001579	0,890647	0,961698	0,137486	0,022485	0,00605	protein_coding	PHACTR2	phosphatase and actin regulator 2 [Source:HGNC Symbol;Acc:HGNC:20956]	6

ENSG00000175215	2976,67	0,002403	0,891105	0,961742	0,136906	0,020269	0,00437	protein_coding	CTDSP2	CTD small phosphatase 2 [Source:HGNC Symbol;Acc:HGNC:17077]	12
ENSG00000029364	2078,021	0,001161	0,891679	0,96191	0,136179	0,020944	0,004773	protein_coding	SLC39A9	solute carrier family 39 member 9 [Source:HGNC Symbol;Acc:HGNC:20182]	14
ENSG00000167986	4353,003	0,001794	0,892326	0,962157	0,135361	0,020798	0,004637	protein_coding	DDB1	damage specific DNA binding protein 1 [Source:HGNC Symbol;Acc:HGNC:2717]	11
ENSG00000164190	3715,042	-0,00358	0,893084	0,962159	-0,1344	0,020252	-0,00423	protein_coding	NIPBL	NIPBL cohesin loading factor [Source:HGNC Symbol;Acc:HGNC:28862]	5
ENSG00000113282	2321,704	0,001451	0,893582	0,962159	0,133773	0,02181	0,005292	protein_coding	CLINT1	clathrin interactor 1 [Source:HGNC Symbol;Acc:HGNC:23186]	5
ENSG00000153317	3019,234	-0,00151	0,893502	0,962159	-0,13387	0,021532	-0,00508	protein_coding	ASAP1	ArfGAP with SH3 domain, ankyrin repeat and PH domain 1 [Source:HGNC Symbol;Acc:HGNC:2720]	8
ENSG00000165029	2603,828	0,001075	0,894165	0,962336	0,133035	0,024684	0,010042	protein_coding	ABCA1	ATP binding cassette subfamily A member 1 [Source:HGNC Symbol;Acc:HGNC:29]	9
ENSG00000096433	4721,797	0,000929	0,897065	0,965005	0,12937	0,025021	0,011336	protein_coding	ITPR3	inositol 1,4,5-trisphosphate receptor type 3 [Source:HGNC Symbol;Acc:HGNC:6182]	6
ENSG00000126261	2866,496	0,002529	0,897875	0,965425	0,128347	0,022323	0,005433	protein_coding	UBA2	ubiquitin like modifier activating enzyme 2 [Source:HGNC Symbol;Acc:HGNC:30661]	19
ENSG00000166747	2016,863	0,001999	0,899993	0,96725	-0,12567	0,021575	-0,00478	protein_coding	AP1G1	adaptor related protein complex 1 subunit gamma 1 [Source:HGNC Symbol;Acc:HGNC:555]	16
ENSG00000014824	1588,974	-0,00148	0,901562	0,96741	-0,12369	0,022771	-0,00573	protein_coding	SLC30A9	solute carrier family 30 member 9 [Source:HGNC Symbol;Acc:HGNC:1329]	4
ENSG00000009954	5062,189	-0,0015	0,903925	0,96741	-0,1207	0,019373	-0,00348	protein_coding	BAZ1B	bromodomain adjacent to zinc finger domain 1B [Source:HGNC Symbol;Acc:HGNC:961]	7
ENSG00000147416	1731,211	0,001461	0,902628	0,96741	0,122342	0,021924	0,004923	protein_coding	ATP6V1B2	ATPase H+ transporting V1 subunit B2 [Source:HGNC Symbol;Acc:HGNC:854]	8
ENSG00000164684	2097,168	0,000764	0,902625	0,96741	0,122346	0,025299	0,012571	protein_coding	ZNF704	zinc finger protein 704 [Source:HGNC Symbol;Acc:HGNC:32291]	8
ENSG00000139289	12141,22	0,003808	0,903309	0,96741	0,121482	0,02276	0,005392	protein_coding	PHLDA1	pleckstrin homology like domain family A member 1 [Source:HGNC Symbol;Acc:HGNC:8933]	12
ENSG00000072062	2480,974	0,00164	0,90268	0,96741	0,122277	0,021739	0,004777	protein_coding	PRKACA	protein kinase cAMP-activated catalytic subunit alpha [Source:HGNC Symbol;Acc:HGNC:9380]	19
ENSG00000006016	3765,109	0,000608	0,90114	0,96741	0,124221	0,025621	0,016715	protein_coding	CRLF1	cytokine receptor like factor 1 [Source:HGNC Symbol;Acc:HGNC:2364]	19
ENSG00000126001	1796,422	0,000964	0,901325	0,96741	0,123988	0,022789	0,005787	protein_coding	CEP250	centrosomal protein 250 [Source:HGNC Symbol;Acc:HGNC:1859]	20
ENSG00000182492	18039,09	0,001098	0,903536	0,96741	0,121196	0,024243	0,007891	protein_coding	BGN	biglycan [Source:HGNC Symbol;Acc:HGNC:1044]	X
ENSG00000132153	1737,101	-0,00133	0,904938	0,968044	-0,11943	0,022416	-0,0052	protein_coding	DHX30	DExH-box helicase 30 [Source:HGNC Symbol;Acc:HGNC:16716]	3
ENSG00000106105	6278,032	-0,00144	0,905576	0,968276	-0,11862	0,021459	-0,00446	protein_coding	GARS1	glycyl-tRNA synthetase 1 [Source:HGNC Symbol;Acc:HGNC:4162]	7
ENSG00000099331	5155,619	0,001499	0,906136	0,968425	0,117914	0,021721	0,004599	protein_coding	MYO9B	myosin IXB [Source:HGNC Symbol;Acc:HGNC:7609]	19
ENSG00000125676	1870,34	0,001499	0,90673	0,96861	0,117164	0,021991	0,004762	protein_coding	THOC2	THO complex 2 [Source:HGNC Symbol;Acc:HGNC:19073]	X
ENSG00000164176	2386,8	0,001592	0,907979	0,969494	0,115588	0,024734	0,008785	protein_coding	EDIL3	EGF like repeats and discoidin domains 3 [Source:HGNC Symbol;Acc:HGNC:3173]	5
ENSG00000115738	1606,732	0,000985	0,909018	0,970153	0,114277	0,023217	0,00579	protein_coding	ID2	inhibitor of DNA binding 2 [Source:HGNC Symbol;Acc:HGNC:5361]	2
ENSG00000117318	1782,777	0,000734	0,912373	0,971031	0,110046	0,023718	0,00625	protein_coding	ID3	inhibitor of DNA binding 3, HLH protein [Source:HGNC Symbol;Acc:HGNC:5362]	1
ENSG00000116350	1721,483	-0,00083	0,911949	0,971031	-0,11058	0,023609	-0,00612	protein_coding	SRSF4	serine and arginine rich splicing factor 4 [Source:HGNC Symbol;Acc:HGNC:10786]	1
ENSG00000092964	2392,421	-0,00024	0,912209	0,971031	-0,11025	0,022929	-0,0053	protein_coding	DPYSL2	dihydropyrimidinase like 2 [Source:HGNC Symbol;Acc:HGNC:3014]	8
ENSG00000133872	4497,227	0,001515	0,911018	0,971031	0,111755	0,021367	0,004143	protein_coding	SARAF	store-operated calcium entry associated regulatory factor [Source:HGNC Symbol;Acc:HGNC:28789]	8
ENSG00000100600	1610,644	0,000679	0,910978	0,971031	0,111806	0,024693	0,008517	protein_coding	LGMN	legumain [Source:HGNC Symbol;Acc:HGNC:9472]	14
ENSG00000198740	1564,228	-0,00187	0,911501	0,971031	-0,11115	0,02101	-0,00391	protein_coding	ZNF652	zinc finger protein 652 [Source:HGNC Symbol;Acc:HGNC:29147]	17
ENSG00000080371	1681,357	0,001191	0,913005	0,971255	0,109249	0,022828	0,00513	protein_coding	RAB21	RAB21, member RAS oncogene family [Source:HGNC Symbol;Acc:HGNC:18263]	12
ENSG00000123983	2802,918	-0,00063	0,915779	0,971479	-0,10575	0,020775	-0,00363	protein_coding	ACSL3	acyl-CoA synthetase long chain family member 3 [Source:HGNC Symbol;Acc:HGNC:3570]	2
ENSG00000104763	2290,904	0,001738	0,915686	0,971479	0,105869	0,023717	0,005936	protein_coding	ASAH1	N-acylsphingosine amidohydrolase 1 [Source:HGNC Symbol;Acc:HGNC:735]	8
ENSG00000089154	3940,272	-0,0016	0,916171	0,971479	-0,10526	0,019536	-0,00309	protein_coding	GCN1	GCN1 activator of EIF2AK4 [Source:HGNC Symbol;Acc:HGNC:4199]	12
ENSG00000266037	17076,83	0,000878	0,914127	0,971479	0,107835	0,024663	0,008111				
ENSG00000166233	1977,487	-0,00123	0,915603	0,971479	-0,10597	0,022485	-0,00468	protein_coding	ARIH1	ariadne RBR E3 ubiquitin protein ligase 1 [Source:HGNC Symbol;Acc:HGNC:689]	15
ENSG00000173517	3787,229	-0,00149	0,915372	0,971479	-0,10627	0,023361	-0,00553	protein_coding	PEAK1	pseudopodium enriched atypical kinase 1 [Source:HGNC Symbol;Acc:HGNC:29431]	15
ENSG00000108861	1908,538	0,001218	0,914744	0,971479	0,107056	0,022585	0,004811	protein_coding	DUSP3	dual specificity phosphatase 3 [Source:HGNC Symbol;Acc:HGNC:3069]	17
ENSG00000011275	1622,92	-0,00136	0,916977	0,971886	-0,10424	0,021774	-0,0041	protein_coding	RNF216	ring finger protein 216 [Source:HGNC Symbol;Acc:HGNC:21698]	7
ENSG00000101752	3293,264	0,001055	0,918236	0,972772	-0,10266	0,020221	-0,00328	protein_coding	MIB1	mindbomb E3 ubiquitin protein ligase 1 [Source:HGNC Symbol;Acc:HGNC:21086]	18
ENSG00000112893	1755,876	-0,00107	0,920077	0,973116	-0,10034	0,022274	-0,00428	protein_coding	MAN2A1	mannosidase alpha class 2A member 1 [Source:HGNC Symbol;Acc:HGNC:6824]	5
ENSG00000127870	1919,691	0,001232	0,919066	0,973116	0,10161	0,022647	0,004615	protein_coding	RNF6	ring finger protein 6 [Source:HGNC Symbol;Acc:HGNC:10069]	13
ENSG00000166963	8701,983	0,001057	0,920253	0,973116	0,100116	0,023442	0,005326	protein_coding	MAP1A	microtubule associated protein 1A [Source:HGNC Symbol;Acc:HGNC:6835]	15
ENSG00000068305	1848,727	-0,00139	0,919711	0,973116	-0,1008	0,022953	-0,00484	protein_coding	MEF2A	myocyte enhancer factor 2A [Source:HGNC Symbol;Acc:HGNC:6993]	15
ENSG00000136381	1561,113	0,000159	0,92187	0,974379	-0,09808	0,021229	-0,00359	protein_coding	IREB2	iron responsive element binding protein 2 [Source:HGNC Symbol;Acc:HGNC:6115]	15
ENSG00000089693	1932,819	0,001282	0,922541	0,97464	0,097234	0,02228	0,004144	protein_coding	MLF2	myeloid leukemia factor 2 [Source:HGNC Symbol;Acc:HGNC:7126]	12
ENSG00000181555	2293,221	-0,00089	0,923569	0,974832	-0,09594	0,021314	-0,00354	protein_coding	SETD2	SET domain containing 2, histone lysine methyltransferase [Source:HGNC Symbol;Acc:HGNC:18420]	3
ENSG00000041982	3570,749	-0,00035	0,923296	0,974832	-0,09628	0,025804	-0,01689	protein_coding	TNC	tenascin C [Source:HGNC Symbol;Acc:HGNC:5318]	9
ENSG00000148175	1574,433	-0,00088	0,924348	0,974952	-0,09496	0,024067	-0,0059	protein_coding	STOM	stomatin [Source:HGNC Symbol;Acc:HGNC:3383]	9
ENSG00000197102	23923,96	-0,00116	0,924531	0,974952	-0,09473	0,021238	-0,00345	protein_coding	DYNC1H1	dynein cytoplasmic 1 heavy chain 1 [Source:HGNC Symbol;Acc:HGNC:2961]	14
ENSG00000198700	2953,005	0,001166	0,926659	0,975855	0,092049	0,020568	0,003072	protein_coding	IPO9	importin 9 [Source:HGNC Symbol;Acc:HGNC:19425]	1

ENSG00000113580	2820,341	-0,00089	0,926218	0,975855	-0,0926	0,022269	-0,00395	protein_coding	NR3C1	nuclear receptor subfamily 3 group C member 1 [Source:HGNC Symbol;Acc:HGNC:7978]	5
ENSG00000097007	2633,162	-0,00105	0,926386	0,975855	-0,09239	0,020345	-0,003	protein_coding	ABL1	ABL proto-oncogene 1, non-receptor tyrosine kinase [Source:HGNC Symbol;Acc:HGNC:76]	9
ENSG00000102265	172728,1	0,00093	0,927305	0,976088	0,091236	0,023586	0,005014	protein_coding	TIMP1	TIMP metallopeptidase inhibitor 1 [Source:HGNC Symbol;Acc:HGNC:11820]	X
ENSG00000123091	1936,103	-9,7E-05	0,929326	0,976659	0,088693	0,021669	0,003455	protein_coding	RNF11	ring finger protein 11 [Source:HGNC Symbol;Acc:HGNC:10056]	1
ENSG00000112159	2295,101	0,001932	0,928912	0,976659	0,089213	0,022427	0,003878	protein_coding	MDN1	midasin AAA ATPase 1 [Source:HGNC Symbol;Acc:HGNC:18302]	6
ENSG00000152818	4227,21	-0,001	0,928948	0,976659	-0,08917	0,022561	-0,004	protein_coding	UTRN	utrophin [Source:HGNC Symbol;Acc:HGNC:12635]	6
ENSG00000074181	6641,788	0,000424	0,929545	0,976659	0,088418	0,025646	0,01236	protein_coding	NOTCH3	notch receptor 3 [Source:HGNC Symbol;Acc:HGNC:7883]	19
ENSG00000100596	1903,435	-0,00095	0,9302	0,976902	-0,08759	0,022837	-0,00413	protein_coding	SPTLC2	serine palmitoyltransferase long chain base subunit 2 [Source:HGNC Symbol;Acc:HGNC:11278]	14
ENSG00000127483	3074,662	-0,00093	0,931952	0,977184	-0,08539	0,022882	-0,00406	protein_coding	HP1BP3	heterochromatin protein 1 binding protein 3 [Source:HGNC Symbol;Acc:HGNC:24973]	1
ENSG00000146463	1573,482	0,000288	0,932168	0,977184	0,085117	0,021626	0,003293	protein_coding	ZMYM4	zinc finger MYM-type containing 4 [Source:HGNC Symbol;Acc:HGNC:13055]	1
ENSG00000162909	7902,899	-0,00129	0,93132	0,977184	0,086185	0,02305	0,004216	protein_coding	CAPN2	calpain 2 [Source:HGNC Symbol;Acc:HGNC:1479]	1
ENSG00000116962	10469,22	-0,00094	0,931738	0,977184	-0,08566	0,022811	-0,00401	protein_coding	NID1	nidogen 1 [Source:HGNC Symbol;Acc:HGNC:7821]	1
ENSG00000112697	4095,208	-0,00114	0,934325	0,977219	-0,0824	0,023202	-0,00416	protein_coding	TMEM30A	transmembrane protein 30A [Source:HGNC Symbol;Acc:HGNC:16667]	6
ENSG00000169826	3303,003	-0,00059	0,932851	0,977219	-0,08426	0,021767	-0,00333	protein_coding	CSGALNAC	chondroitin sulfate N-acetylgalactosaminyltransferase 2 [Source:HGNC Symbol;Acc:HGNC:24292]	10
ENSG00000123094	1729,943	0,001634	0,93404	0,977219	0,082763	0,024138	0,00518	protein_coding	RASSF8	Ras association domain family member 8 [Source:HGNC Symbol;Acc:HGNC:13232]	12
ENSG00000104964	4099,777	0,002154	0,934053	0,977219	0,082746	0,022929	0,00391	protein_coding	TLE5	TLE family member 5, transcriptional modulator [Source:HGNC Symbol;Acc:HGNC:307]	19
ENSG00000198900	3817,45	-0,0006	0,933538	0,977219	-0,08339	0,021323	-0,00308	protein_coding	TOP1	DNA topoisomerase I [Source:HGNC Symbol;Acc:HGNC:11986]	20
ENSG00000017260	2116,951	-0,00091	0,934894	0,97737	-0,08169	0,023017	-0,00399	protein_coding	ATP2C1	ATPase secretory pathway Ca2+ transporting 1 [Source:HGNC Symbol;Acc:HGNC:13211]	3
ENSG00000130340	3663,194	-0,00155	0,936048	0,977688	-0,08024	0,022017	-0,00327	protein_coding	SNX9	sorting nexin 9 [Source:HGNC Symbol;Acc:HGNC:14973]	6
ENSG00000088256	1869,318	-0,001	0,935793	0,977688	-0,08056	0,022447	-0,00354	protein_coding	GNA11	G protein subunit alpha 11 [Source:HGNC Symbol;Acc:HGNC:4379]	19
ENSG00000109790	1999,08	7E-05	0,937235	0,978484	0,078745	0,023743	0,004517	protein_coding	KLHL5	kelch like family member 5 [Source:HGNC Symbol;Acc:HGNC:6356]	4
ENSG00000054523	2569,227	-0,00089	0,941178	0,979962	-0,07379	0,021894	-0,00297	protein_coding	KIF1B	kinesin family member 1B [Source:HGNC Symbol;Acc:HGNC:16636]	1
ENSG00000143614	1701,464	0,002358	0,947145	0,979962	0,066293	0,021313	0,002421	protein_coding	GATAD2B	GATA zinc finger domain containing 2B [Source:HGNC Symbol;Acc:HGNC:30778]	1
ENSG00000163820	1766,674	-0,00065	0,943258	0,979962	-0,07118	0,023558	-0,0039	protein_coding	FYCO1	FYVE and coiled-coil domain autophagy adaptor 1 [Source:HGNC Symbol;Acc:HGNC:14673]	3
ENSG00000083896	1656,52	-0,00073	0,947103	0,979962	-0,06634	0,022154	-0,00278	protein_coding	YTHDC1	YTH domain containing 1 [Source:HGNC Symbol;Acc:HGNC:30626]	4
ENSG00000133835	2165,477	0,000478	0,94488	0,979962	0,069137	0,021399	0,002585	protein_coding	HSD17B4	hydroxysteroid 17-beta dehydrogenase 4 [Source:HGNC Symbol;Acc:HGNC:5213]	5
ENSG00000146701	2003,314	-0,00093	0,945058	0,979962	-0,06891	0,021841	-0,00275	protein_coding	MDH2	malate dehydrogenase 2 [Source:HGNC Symbol;Acc:HGNC:6971]	7
ENSG00000166508	2039,726	0,000787	0,94173	0,979962	0,073095	0,023128	0,003645	protein_coding	MCM7	minichromosome maintenance complex component 7 [Source:HGNC Symbol;Acc:HGNC:6950]	7
ENSG00000005483	2498,8	-0,0008	0,947169	0,979962	-0,06626	0,020994	-0,00234	protein_coding	KMT2E	lysine methyltransferase 2E (inactive) [Source:HGNC Symbol;Acc:HGNC:18541]	7
ENSG00000003989	1606,205	-0,00038	0,946236	0,979962	-0,06743	0,025065	-0,00609	protein_coding	SLC7A2	solute carrier family 7 member 2 [Source:HGNC Symbol;Acc:HGNC:11060]	8
ENSG00000149428	7656,768	0,002478	0,939876	0,979962	0,075425	0,022412	0,003246	protein_coding	HYOU1	hypoxia up-regulated 1 [Source:HGNC Symbol;Acc:HGNC:16931]	11
ENSG00000011465	2349,838	0,000534	0,940786	0,979962	0,074282	0,025008	0,006506	protein_coding	DCN	decorin [Source:HGNC Symbol;Acc:HGNC:2705]	12
ENSG00000011028	11236,03	-0,00067	0,94348	0,979962	-0,0709	0,023924	-0,00424	protein_coding	MRC2	mannose receptor C type 2 [Source:HGNC Symbol;Acc:HGNC:16875]	17
ENSG00000136485	2489,499	-0,00083	0,946012	0,979962	-0,06772	0,020468	-0,00223	protein_coding	DCAF7	DDB1 and CUL4 associated factor 7 [Source:HGNC Symbol;Acc:HGNC:30915]	17
ENSG00000171634	4064,167	-0,00091	0,94231	0,979962	-0,07237	0,019713	-0,00218	protein_coding	BPTF	bromodomain PHD finger transcription factor [Source:HGNC Symbol;Acc:HGNC:3581]	17
ENSG00000101557	1818,83	-0,00083	0,943792	0,979962	-0,0705	0,021584	-0,00271	protein_coding	USP14	ubiquitin specific peptidase 14 [Source:HGNC Symbol;Acc:HGNC:12612]	18
ENSG00000105701	2475,087	-0,00114	0,944486	0,979962	-0,06963	0,022587	-0,00313	protein_coding	FKBP8	FKBP prolyl isomerase 8 [Source:HGNC Symbol;Acc:HGNC:3724]	19
ENSG00000101224	2250,001	0,002899	0,944484	0,979962	0,069635	0,022398	0,002966	protein_coding	CDC25B	cell division cycle 25B [Source:HGNC Symbol;Acc:HGNC:1726]	20
ENSG00000198768	3137,893	-0,00022	0,944897	0,979962	-0,06912	0,025867	-0,01383	protein_coding	APCDD1L	APC down-regulated 1 like [Source:HGNC Symbol;Acc:HGNC:26892]	20
ENSG00000130706	1937,812	-0,00078	0,945015	0,979962	-0,06897	0,021948	-0,0028	protein_coding	ADRM1	adhesion regulating molecule 1 [Source:HGNC Symbol;Acc:HGNC:15759]	20
ENSG00000102038	1770,095	-0,00047	0,942787	0,979962	-0,07177	0,02514	-0,00674	protein_coding	SMARCA1	SWI/SNF related, matrix associated, actin dependent regulator of chromatin, subfamily a, member 1 [Source:HGNC Sym	X
ENSG00000165650	2770,4	0,000619	0,947749	0,980121	0,065534	0,02182	0,002608	protein_coding	PDZD8	PDZ domain containing 8 [Source:HGNC Symbol;Acc:HGNC:26974]	10
ENSG00000177606	3263,474	0,000471	0,950174	0,980426	0,062488	0,0249	0,005208	protein_coding	JUN	Jun proto-oncogene, AP-1 transcription factor subunit [Source:HGNC Symbol;Acc:HGNC:6204]	1
ENSG00000112473	3582,335	0,003232	0,94968	0,980426	-0,06311	0,021013	-0,00219	protein_coding	SLC39A7	solute carrier family 39 member 7 [Source:HGNC Symbol;Acc:HGNC:4927]	6
ENSG00000111011	1670,757	-0,00106	0,94877	0,980426	0,064252	0,021664	0,002493	protein_coding	RSRC2	arginine and serine rich coiled-coil 2 [Source:HGNC Symbol;Acc:HGNC:30559]	12
ENSG00000101577	1845,483	-0,00078	0,949761	0,980426	-0,06301	0,021823	-0,00251	protein_coding	LPIN2	lipin 2 [Source:HGNC Symbol;Acc:HGNC:14450]	18
ENSG00000101266	2289,865	-0,00095	0,94896	0,980426	-0,06401	0,020053	-0,002	protein_coding	CSNK2A1	casein kinase 2 alpha 1 [Source:HGNC Symbol;Acc:HGNC:2457]	20
ENSG00000105825	4046,914	0,000262	0,950802	0,980481	0,0617	0,025735	0,009677	protein_coding	TFPI2	tissue factor pathway inhibitor 2 [Source:HGNC Symbol;Acc:HGNC:11761]	7
ENSG00000141027	5025,156	0,004267	0,951431	0,980481	0,06091	0,022279	0,002482	protein_coding	NCOR1	nuclear receptor corepressor 1 [Source:HGNC Symbol;Acc:HGNC:7672]	17
ENSG00000109111	5219,503	-0,00054	0,951505	0,980481	-0,06082	0,020446	-0,002	protein_coding	SUPT6H	SPT6 homolog, histone chaperone and transcription elongation factor [Source:HGNC Symbol;Acc:HGNC:11470]	17
ENSG00000134644	2163,482	-0,00067	0,95602	0,981252	-0,05515	0,019559	-0,00163	protein_coding	PUM1	pumilio RNA binding family member 1 [Source:HGNC Symbol;Acc:HGNC:14957]	1
ENSG00000084652	3001,717	0,00108	0,956727	0,981252	0,054261	0,019583	0,001606	protein_coding	TXLNA	taxilin alpha [Source:HGNC Symbol;Acc:HGNC:30685]	1

ENSG00000213064	1985,818	-0,00171	0,955198	0,981252	0,05618	0,0229	0,002655	protein_coding	SFT2D2	SFT2 domain containing 2 [Source:HGNC Symbol;Acc:HGNC:25140]	1
ENSG00000123989	2455,762	0,000653	0,956245	0,981252	0,054866	0,021971	0,002233	protein_coding	CHPF	chondroitin polymerizing factor [Source:HGNC Symbol;Acc:HGNC:24291]	2
ENSG00000153827	6918,738	0,000729	0,955357	0,981252	0,055981	0,017395	0,001306	protein_coding	TRIP12	thyroid hormone receptor interactor 12 [Source:HGNC Symbol;Acc:HGNC:12306]	2
ENSG00000169714	4044,013	0,00068	0,956375	0,981252	0,054704	0,020454	0,001802	protein_coding	CNBP	CCHC-type zinc finger nucleic acid binding protein [Source:HGNC Symbol;Acc:HGNC:13164]	3
ENSG00000056097	3314,167	0,000612	0,957136	0,981252	0,053748	0,021413	0,002014	protein_coding	ZFR	zinc finger RNA binding protein [Source:HGNC Symbol;Acc:HGNC:17277]	5
ENSG00000171604	1807,704	0,000476	0,956518	0,981252	0,054524	0,022721	0,00252	protein_coding	CXXC5	CXXC finger protein 5 [Source:HGNC Symbol;Acc:HGNC:26943]	5
ENSG00000204394	1946,919	-0,00065	0,954034	0,981252	-0,05764	0,02265	-0,00263	protein_coding	VARS1	valyl-tRNA synthetase 1 [Source:HGNC Symbol;Acc:HGNC:12651]	6
ENSG00000197694	13155,84	0,001262	0,953576	0,981252	0,058217	0,022724	0,00268	protein_coding	SPTAN1	spectrin alpha, non-erythrocytic 1 [Source:HGNC Symbol;Acc:HGNC:11273]	9
ENSG00000196712	3316,426	0,000468	0,954493	0,981252	0,057066	0,023067	0,002818	protein_coding	NF1	neurofibromin 1 [Source:HGNC Symbol;Acc:HGNC:7765]	17
ENSG00000074695	5297,829	-0,00057	0,957371	0,981252	-0,05345	0,021027	-0,0019	protein_coding	LMAN1	lectin, mannose binding 1 [Source:HGNC Symbol;Acc:HGNC:6631]	18
ENSG00000137207	1820,442	0,00056	0,958208	0,981349	0,052403	0,0227	0,002412	protein_coding	YIPF3	Yip1 domain family member 3 [Source:HGNC Symbol;Acc:HGNC:21023]	6
ENSG00000150636	1582,941	1,26E-05	0,958319	0,981349	0,052263	0,024498	0,003719	protein_coding	CCDC102B	coiled-coil domain containing 102B [Source:HGNC Symbol;Acc:HGNC:26295]	18
ENSG00000105968	3236,411	0,000428	0,959512	0,982134	0,050766	0,021686	0,001981	protein_coding	H2AZ2	H2A.Z variant histone 2 [Source:HGNC Symbol;Acc:HGNC:20664]	7
ENSG00000187605	1616,063	-0,00119	0,963775	0,984355	-0,04542	0,024085	-0,00282	protein_coding	TET3	tet methylcytosine dioxygenase 3 [Source:HGNC Symbol;Acc:HGNC:28313]	2
ENSG00000147459	1667,765	0,00143	0,963821	0,984355	0,045359	0,023392	0,002378	protein_coding	DOCK5	dedicator of cytokinesis 5 [Source:HGNC Symbol;Acc:HGNC:23476]	8
ENSG00000082805	2409,433	0,001287	0,963682	0,984355	-0,04553	0,020887	-0,00158	protein_coding	ERC1	ELKS/RAB6-interacting/CAST family member 1 [Source:HGNC Symbol;Acc:HGNC:17072]	12
ENSG00000089737	3031,691	-0,00059	0,963465	0,984355	-0,04581	0,020431	-0,0015	protein_coding	DDX24	DEAD-box helicase 24 [Source:HGNC Symbol;Acc:HGNC:13266]	14
ENSG00000132670	1998,45	-0,00269	0,963493	0,984355	-0,04577	0,022004	-0,00184	protein_coding	PTPRA	protein tyrosine phosphatase receptor type A [Source:HGNC Symbol;Acc:HGNC:9664]	20
ENSG00000196365	2347,75	0,00064	0,965688	0,985825	0,043017	0,021971	0,001751	protein_coding	LONP1	lon peptidase 1, mitochondrial [Source:HGNC Symbol;Acc:HGNC:9479]	19
ENSG00000183853	2777,204	0,000272	0,967161	0,98689	0,041169	0,025223	0,004072	protein_coding	KIRREL1	kirre like nephrin family adhesion molecule 1 [Source:HGNC Symbol;Acc:HGNC:15734]	1
ENSG00000162576	2126,454	-0,00029	0,968504	0,987413	-0,03949	0,024884	-0,00327	protein_coding	MXRA8	matrix remodeling associated 8 [Source:HGNC Symbol;Acc:HGNC:7542]	1
ENSG00000103495	2149,25	7,17E-05	0,968532	0,987413	0,03945	0,022714	0,001823	protein_coding	MAZ	MYC associated zinc finger protein [Source:HGNC Symbol;Acc:HGNC:6914]	16
ENSG00000075391	1736,501	0,000509	0,969925	0,98788	0,037702	0,023611	0,002094	protein_coding	RASAL2	RAS protein activator like 2 [Source:HGNC Symbol;Acc:HGNC:9874]	1
ENSG00000132424	1900,641	-0,00031	0,970277	0,98788	-0,03726	0,022887	-0,00178	protein_coding	PNISR	PNN interacting serine and arginine rich protein [Source:HGNC Symbol;Acc:HGNC:21222]	6
ENSG00000102316	2892,239	0,000242	0,969972	0,98788	0,037643	0,021622	0,001455	protein_coding	MAGED2	MAGE family member D2 [Source:HGNC Symbol;Acc:HGNC:16353]	X
ENSG00000094975	2495,154	0,0012	0,970751	0,987925	0,036666	0,022207	0,001546	protein_coding	SUCO	SUN domain containing ossification factor [Source:HGNC Symbol;Acc:HGNC:1240]	1
ENSG00000112655	3542,965	0,00037	0,972166	0,988069	0,034891	0,023334	0,00182	protein_coding	PTK7	protein tyrosine kinase 7 (inactive) [Source:HGNC Symbol;Acc:HGNC:9618]	6
ENSG00000156011	2648,769	-0,00083	0,971327	0,988069	-0,03594	0,021699	-0,0014	protein_coding	PSD3	pleckstrin and Sec7 domain containing 3 [Source:HGNC Symbol;Acc:HGNC:19093]	8
ENSG00000088812	2428,498	0,010388	0,972181	0,988069	0,034873	0,023356	0,001344	protein_coding	ATRN	attractin [Source:HGNC Symbol;Acc:HGNC:885]	20
ENSG00000130513	5721,057	0,000153	0,973359	0,988829	0,033396	0,025704	0,005027	protein_coding	GDF15	growth differentiation factor 15 [Source:HGNC Symbol;Acc:HGNC:30142]	19
ENSG00000116147	1751,636	0,000419	0,976168	0,989337	0,029873	0,025958	0,0075	protein_coding	TNR	tenascin R [Source:HGNC Symbol;Acc:HGNC:11953]	1
ENSG00000113712	3299,642	0,000318	0,975608	0,989337	0,030575	0,021493	0,001159	protein_coding	CSNK1A1	casein kinase 1 alpha 1 [Source:HGNC Symbol;Acc:HGNC:2451]	5
ENSG00000066777	2217,336	0,00031	0,97506	0,989337	0,031263	0,021663	0,001216	protein_coding	ARFGEF1	ADP ribosylation factor guanine nucleotide exchange factor 1 [Source:HGNC Symbol;Acc:HGNC:15772]	8
ENSG00000107929	1769,708	0,000858	0,975114	0,989337	-0,03119	0,020737	-0,00106	protein_coding	LARP4B	La ribonucleoprotein 4B [Source:HGNC Symbol;Acc:HGNC:28987]	10
ENSG00000014216	2110,811	-0,00037	0,976823	0,989337	-0,02905	0,022555	-0,0013	protein_coding	CAPN1	calpain 1 [Source:HGNC Symbol;Acc:HGNC:1476]	11
ENSG00000077235	3431,679	-0,00105	0,977541	0,989337	0,028152	0,019075	0,000787	protein_coding	GTF3C1	general transcription factor IIIC subunit 1 [Source:HGNC Symbol;Acc:HGNC:4664]	16
ENSG00000126581	1594,159	-0,00038	0,975368	0,989337	-0,03088	0,020964	-0,00109	protein_coding	BECN1	beclin 1 [Source:HGNC Symbol;Acc:HGNC:1034]	17
ENSG00000100393	4355,384	-0,00117	0,977429	0,989337	0,028292	0,02029	0,000911	protein_coding	EP300	E1A binding protein p300 [Source:HGNC Symbol;Acc:HGNC:3373]	22
ENSG00000185275	1952,004	-7,1E-06	0,977729	0,989337	-0,02792	0,025278	-0,00286	processed_pseudo	CD24P4	CD24 molecule pseudogene 4 [Source:HGNC Symbol;Acc:HGNC:1649]	Y
ENSG00000117114	1592,543	-0,00026	0,979176	0,99	-0,0261	0,023516	-0,00142	protein_coding	ADGRL2	adhesion G protein-coupled receptor L2 [Source:HGNC Symbol;Acc:HGNC:18582]	1
ENSG00000204256	5038,918	-0,00032	0,979244	0,99	-0,02602	0,021981	-0,00106	protein_coding	BRD2	bromodomain containing 2 [Source:HGNC Symbol;Acc:HGNC:1103]	6
ENSG00000124193	1711,682	-0,0003	0,979845	0,990172	-0,02526	0,021597	-0,00097	protein_coding	SRSF6	serine and arginine rich splicing factor 6 [Source:HGNC Symbol;Acc:HGNC:10788]	20
ENSG00000136051	1619,595	-0,00026	0,980755	0,990475	-0,02412	0,023888	-0,00144	protein_coding	WASHC4	WASH complex subunit 4 [Source:HGNC Symbol;Acc:HGNC:29174]	12
ENSG00000136156	6646,503	-0,00023	0,981005	0,990475	-0,02381	0,019877	-0,00073	protein_coding	ITM2B	integral membrane protein 2B [Source:HGNC Symbol;Acc:HGNC:6174]	13
ENSG00000172115	3239,601	-0,00027	0,982725	0,991234	0,021652	0,021711	0,000848	protein_coding	CYCS	cytochrome c, somatic [Source:HGNC Symbol;Acc:HGNC:19986]	7
ENSG00000168172	1917,244	0,000234	0,982408	0,991234	0,02205	0,023406	0,001169	protein_coding	HOOK3	hook microtubule tethering protein 3 [Source:HGNC Symbol;Acc:HGNC:23576]	8
ENSG00000100994	2584,173	0,008647	0,983049	0,991234	0,021247	0,02332	0,000875	protein_coding	PYGB	glycogen phosphorylase B [Source:HGNC Symbol;Acc:HGNC:9723]	20
ENSG00000055917	2462,531	-0,0001	0,983623	0,991378	-0,02053	0,021439	-0,00077	protein_coding	PUM2	pumilio RNA binding family member 2 [Source:HGNC Symbol;Acc:HGNC:14958]	2
ENSG00000084636	2687,861	0,000177	0,984411	0,991738	0,019539	0,022889	0,000932	protein_coding	COL16A1	collagen type XVI alpha 1 chain [Source:HGNC Symbol;Acc:HGNC:2193]	1
ENSG00000198586	1828,466	0,002498	0,98621	0,993115	-0,01728	0,022535	-0,00076	protein_coding	TLK1	tousled like kinase 1 [Source:HGNC Symbol;Acc:HGNC:11841]	2
ENSG00000071967	3121,946	-0,00031	0,989078	0,995568	-0,01369	0,022771	-0,00064	protein_coding	CYBRD1	cytochrome b reductase 1 [Source:HGNC Symbol;Acc:HGNC:20797]	2
ENSG00000145734	3417,373	0,000214	0,990857	0,996224	0,011459	0,021022	0,000407	protein_coding	BDP1	B double prime 1, subunit of RNA polymerase III transcription initiation factor IIIB [Source:HGNC Symbol;Acc:HGNC:136	5

ENSG00000205531	1737,775	-0,00345	0,991753	0,996224	0,010337	0,021489	0,000382	protein_coding	NAP1L4	nucleosome assembly protein 1 like 4 [Source:HGNC Symbol;Acc:HGNC:7640]	11
ENSG00000090382	2438,548	6,09E-05	0,990207	0,996224	0,012275	0,025675	0,001779	protein_coding	LYZ	lysozyme [Source:HGNC Symbol;Acc:HGNC:6740]	12
ENSG00000176105	1791,243	-0,00011	0,99165	0,996224	-0,01047	0,023149	-0,00053	protein_coding	YES1	YES proto-oncogene 1, Src family tyrosine kinase [Source:HGNC Symbol;Acc:HGNC:12841]	18
ENSG00000101126	2719,901	0,00019	0,991894	0,996224	0,010159	0,020801	0,00035	protein_coding	ADNP	activity dependent neuroprotector homeobox [Source:HGNC Symbol;Acc:HGNC:15766]	20
ENSG00000070018	2179,801	0,000879	0,993041	0,99694	0,008722	0,022832	0,000411	protein_coding	LRP6	LDL receptor related protein 6 [Source:HGNC Symbol;Acc:HGNC:6698]	12
ENSG00000004478	1941,706	-0,00014	0,994717	0,997318	-0,00662	0,021255	-0,00024	protein_coding	FKBP4	FKBP prolyl isomerase 4 [Source:HGNC Symbol;Acc:HGNC:3720]	12
ENSG00000102699	3014,098	0,000274	0,994431	0,997318	0,00698	0,022824	0,000329	protein_coding	PARP4	poly(ADP-ribose) polymerase family member 4 [Source:HGNC Symbol;Acc:HGNC:271]	13
ENSG00000185624	19378,93	0,003838	0,994386	0,997318	-0,00704	0,022154	-0,00028	protein_coding	P4HB	prolyl 4-hydroxylase subunit beta [Source:HGNC Symbol;Acc:HGNC:8548]	17
ENSG00000189091	2222,905	0,000113	0,995273	0,99744	-0,00592	0,0218	-0,00024	protein_coding	SF3B3	splicing factor 3b subunit 3 [Source:HGNC Symbol;Acc:HGNC:10770]	16
ENSG00000266412	2758,291	6,67E-05	0,995974	0,997708	0,005046	0,021582	0,000194	protein_coding	NCOA4	nuclear receptor coactivator 4 [Source:HGNC Symbol;Acc:HGNC:7671]	10
ENSG00000133313	1905,125	-0,0005	0,99659	0,997891	0,004274	0,021119	0,000154	protein_coding	CNDP2	carnosine dipeptidase 2 [Source:HGNC Symbol;Acc:HGNC:24437]	18
ENSG00000134109	1698,425	8,22E-05	0,99705	0,997917	0,003697	0,022629	0,000168	protein_coding	EDEM1	ER degradation enhancing alpha-mannosidase like protein 1 [Source:HGNC Symbol;Acc:HGNC:18967]	3
ENSG00000086232	3099,541	-0,00012	0,997645	0,998079	0,002952	0,019951	9,14E-05	protein_coding	EIF2AK1	eukaryotic translation initiation factor 2 alpha kinase 1 [Source:HGNC Symbol;Acc:HGNC:24921]	7
ENSG00000179295	4646,89	9,03E-05	0,998584	0,998584	-0,00177	0,020975	-6,3E-05	protein_coding	PTPN11	protein tyrosine phosphatase non-receptor type 11 [Source:HGNC Symbol;Acc:HGNC:9644]	12
ENSG00000227232	34,58357	-0,00321	0,296493		-1,04398	0,026169	-0,21646	unprocessed_pseu	WASH7P	WASP family homolog 7, pseudogene [Source:HGNC Symbol;Acc:HGNC:38034]	1
ENSG00000237683	10,31838	-0,00178	0,208112		-1,25877	0,026136	-0,6155				
ENSG00000241860	12,34244	-0,00112	0,571295		-0,56615	0,026044	-0,19722	lncRNA	AL627309.	novel transcript	1
ENSG00000228463	14,92875	-0,00332	0,138292		-1,48218	0,026301	-0,44384	transcribed_proces	AP006222.	ribosomal protein L23a (RPL23A) pseudogene	1
ENSG00000230021	25,60046	0,000129	0,942965		0,071544	0,026022	0,0271	transcribed_proces	AL669831.	septin 14 (SEPT14) pseudogene	1
ENSG00000225972	116,6199	-0,00061	0,85416		-0,18381	0,025874	-0,03674	unprocessed_pseu	MTND1P2	MT-ND1 pseudogene 23 [Source:HGNC Symbol;Acc:HGNC:42092]	1
ENSG00000229344	201,0901	-0,00404	0,8298		-0,21496	0,026335	-0,04401	unprocessed_pseu	MTCO2P12	MT-CO2 pseudogene 12 [Source:HGNC Symbol;Acc:HGNC:52028]	1
ENSG00000198744	192,9835	-0,00167	0,596914		-0,52884	0,025961	-0,11053	unprocessed_pseu	MTCO3P12	MT-CO3 pseudogene 12 [Source:HGNC Symbol;Acc:HGNC:52042]	1
ENSG00000228327	48,44943	0,00035	0,740537		-0,33114	0,025859	-0,0649	transcribed_unpro	AL669831.	general transcription factor IIi (GTF2I) pseudogene	1
ENSG00000237491	20,79361	-0,00242	0,311896		-1,01125	0,026142	-0,29251	lncRNA	LINC01409	long intergenic non-protein coding RNA 1409 [Source:HGNC Symbol;Acc:HGNC:50701]	1
ENSG00000230092	14,18704	-0,00013	0,950879		-0,0616	0,026	-0,02008	transcribed_unpro	AL669831.	F-box only protein 25 (FBXO25) pseudogene	1
ENSG00000228794	429,8282	-0,02437	0,01895		-2,34651	0,042864	-0,19633	lncRNA	LINC01128	long intergenic non-protein coding RNA 1128 [Source:HGNC Symbol;Acc:HGNC:49377]	1
ENSG00000225880	13,742	-0,0006	0,768585		-0,29423	0,026022	-0,10357	lncRNA	LINC00115	long intergenic non-protein coding RNA 115 [Source:HGNC Symbol;Acc:HGNC:26211]	1
ENSG00000230699	11,0541	-0,00152	0,400709		-0,84036	0,026085	-0,31267	lncRNA	AL645608.	novel transcript	1
ENSG00000223764	148,6631	-0,00288	0,643227		-0,46319	0,025576	-0,05007	lncRNA	LINC02593	long intergenic non-protein coding RNA 2593 [Source:HGNC Symbol;Acc:HGNC:53933]	1
ENSG00000187634	440,1209	-0,01508	0,091023		-1,69003	0,030864	-0,13783	protein_coding	SAMD11	sterile alpha motif domain containing 11 [Source:HGNC Symbol;Acc:HGNC:28706]	1
ENSG00000188976	1325,167	0,017411	0,191109		1,307307	0,026719	0,051841	protein_coding	NOC2L	NOC2 like nucleolar associated transcriptional repressor [Source:HGNC Symbol;Acc:HGNC:24517]	1
ENSG00000187961	126,3934	-0,00086	0,876626		-0,15525	0,02558	-0,01988	protein_coding	KLHL17	kelch like family member 17 [Source:HGNC Symbol;Acc:HGNC:24023]	1
ENSG00000187583	40,26864	0,000834	0,869954		0,163717	0,025914	0,035186	protein_coding	PLEKHN1	pleckstrin homology domain containing N1 [Source:HGNC Symbol;Acc:HGNC:25284]	1
ENSG00000272512	12,98506	-1,1E-05	0,994359		-0,00707	0,026029	-0,00288	lncRNA	AL645608.	novel transcript	1
ENSG00000188290	136,0123	0,007509	0,225471		1,212108	0,026866	0,130785	protein_coding	HES4	hes family bHLH transcription factor 4 [Source:HGNC Symbol;Acc:HGNC:24149]	1
ENSG00000187608	348,7709	-0,00323	0,197991		-1,2873	0,026213	-0,29165	protein_coding	ISG15	ISG15 ubiquitin like modifier [Source:HGNC Symbol;Acc:HGNC:4053]	1
ENSG00000217801	54,83204	-0,00427	0,099159		-1,64894	0,026473	-0,42777	transcribed_unpro	AL390719.	protein tyrosine phosphatase family pseudogene	1
ENSG00000131591	121,2807	-0,00363	0,545808		-0,60405	0,025642	-0,0626	protein_coding	C1orf159	chromosome 1 open reading frame 159 [Source:HGNC Symbol;Acc:HGNC:26062]	1
ENSG00000207730	8,919285	-0,0009	0,394348		-0,85176	0,026068	-0,38393	miRNA	MIR200B	microRNA 200b [Source:HGNC Symbol;Acc:HGNC:31579]	1
ENSG00000198976	8,785389	0,000476	0,769713		0,29275	0,026061	0,155218	miRNA	MIR429	microRNA 429 [Source:HGNC Symbol;Acc:HGNC:13784]	1
ENSG00000272141	46,0345	9,88E-05	0,968571		0,0394	0,025951	0,010134	lncRNA	AL390719.	novel transcript	1
ENSG00000162571	11,44623	-2,4E-05	0,787479		-0,26959	0,026053	-0,13587	protein_coding	TTLL10	tubulin tyrosine ligase like 10 [Source:HGNC Symbol;Acc:HGNC:26693]	1
ENSG00000176022	418,5536	0,005462	0,544548		0,60595	0,024946	0,039932	protein_coding	B3GALT6	beta-1,3-galactosyltransferase 6 [Source:HGNC Symbol;Acc:HGNC:17978]	1
ENSG00000160087	734,3284	0,00527	0,605038		0,517169	0,024205	0,028722	protein_coding	UBE2J2	ubiquitin conjugating enzyme E2 J2 [Source:HGNC Symbol;Acc:HGNC:19268]	1
ENSG00000162572	22,45937	0,001712	0,559752		0,58321	0,026005	0,136154	protein_coding	SCNN1D	sodium channel epithelial 1 subunit delta [Source:HGNC Symbol;Acc:HGNC:10601]	1
ENSG00000131584	1111,28	-0,0111	0,220907		-1,22412	0,027438	-0,08569	protein_coding	ACAP3	ArfGAP with coiled-coil, ankyrin repeat and PH domains 3 [Source:HGNC Symbol;Acc:HGNC:16754]	1
ENSG00000169972	163,7957	-0,00152	0,80502		-0,24686	0,025292	-0,02455	protein_coding	PUSL1	pseudouridine synthase like 1 [Source:HGNC Symbol;Acc:HGNC:26914]	1
ENSG00000127054	877,1044	-0,00058	0,982908		-0,02142	0,023383	-0,00113	protein_coding	INTS11	integrator complex subunit 11 [Source:HGNC Symbol;Acc:HGNC:26052]	1
ENSG00000224051	268,2545	0,003601	0,658866		0,441479	0,025051	0,034357	protein_coding	CPTP	ceramide-1-phosphate transfer protein [Source:HGNC Symbol;Acc:HGNC:28116]	1
ENSG00000169962	11,37426	0,001828	0,23581		1,185525	0,026131	0,506356	protein_coding	TAS1R3	taste 1 receptor member 3 [Source:HGNC Symbol;Acc:HGNC:15661]	1
ENSG00000107404	921,4774	-0,00824	0,496842		-0,67947	0,023474	-0,02904	protein_coding	DVL1	dishevelled segment polarity protein 1 [Source:HGNC Symbol;Acc:HGNC:3084]	1
ENSG00000175756	1002,666	-0,00074	0,94087		-0,07418	0,023492	-0,004	protein_coding	AURKAIP1	aurora kinase A interacting protein 1 [Source:HGNC Symbol;Acc:HGNC:24114]	1

ENSG00000224870	123,2998	-0,00114	0,833448	-0,21028	0,025405	-0,02296	lncRNA	MRPL20-AS	MRPL20 antisense RNA 1 [Source:HGNC Symbol;Acc:HGNC:27125]	1
ENSG00000242485	515,073	0,044458	0,027152	2,209325	0,056536	0,107388	protein_coding	MRPL20	mitochondrial ribosomal protein L20 [Source:HGNC Symbol;Acc:HGNC:14478]	1
ENSG00000272455	26,05465	-0,00106	0,722952	-0,35452	0,025937	-0,07894	lncRNA	MRPL20-DT	MRPL20 divergent transcript [Source:HGNC Symbol;Acc:HGNC:54814]	1
ENSG00000235098	50,14213	-0,0019	0,362127	-0,91132	0,026099	-0,28684	protein_coding	ANKRD65	ankyrin repeat domain 65 [Source:HGNC Symbol;Acc:HGNC:42950]	1
ENSG00000179403	224,8664	0,007512	0,158224	1,411069	0,027098	0,177876	protein_coding	VWA1	von Willebrand factor A domain containing 1 [Source:HGNC Symbol;Acc:HGNC:30910]	1
ENSG00000215915	66,44642	-0,0048	0,235376	-1,18662	0,026411	-0,19653	protein_coding	ATAD3C	ATPase family AAA domain containing 3C [Source:HGNC Symbol;Acc:HGNC:32151]	1
ENSG00000160072	439,8547	-0,01046	0,3112	-1,01271	0,026446	-0,06149	protein_coding	ATAD3B	ATPase family AAA domain containing 3B [Source:HGNC Symbol;Acc:HGNC:24007]	1
ENSG00000197785	700,7978	0,006949	0,437026	0,777226	0,025534	0,053475	protein_coding	ATAD3A	ATPase family AAA domain containing 3A [Source:HGNC Symbol;Acc:HGNC:25567]	1
ENSG00000205090	10,08883	-0,00077	0,573135	-0,56344	0,026062	-0,27161	protein_coding	TMEM240	transmembrane protein 240 [Source:HGNC Symbol;Acc:HGNC:25186]	1
ENSG00000215014	13,67415	-0,00016	0,937832	-0,078	0,026005	-0,02608	lncRNA	AL645728.	novel transcript	1
ENSG00000228594	113,0218	0,004999	0,230152	1,199966	0,026442	0,191102	protein_coding	FNDC10	fibronectin type III domain containing 10 [Source:HGNC Symbol;Acc:HGNC:42951]	1
ENSG00000272106	70,93867	0,003515	0,419814	0,806744	0,026099	0,130972	lncRNA	AL691432.	artifact	1
ENSG00000197530	580,6132	-0,05644	0,012241	-2,50511	0,143945	-0,16681	protein_coding	MIB2	mindbomb E3 ubiquitin protein ligase 2 [Source:HGNC Symbol;Acc:HGNC:30577]	1
ENSG00000248333	365,8543	-0,00148	0,857466	-0,1796	0,024394	-0,01218	protein_coding	CDK11B	cyclin dependent kinase 11B [Source:HGNC Symbol;Acc:HGNC:1729]	1
ENSG00000189339	733,199	-0,0415	0,02927	-2,17983	0,055619	-0,109	protein_coding	SLC35E2B	solute carrier family 35 member E2B [Source:HGNC Symbol;Acc:HGNC:33941]	1
ENSG00000008128	40,94811	-0,00307	0,380976	-0,8761	0,026117	-0,1705	protein_coding	CDK11A	cyclin dependent kinase 11A [Source:HGNC Symbol;Acc:HGNC:1730]	1
ENSG00000268575	68,52067	-0,0044	0,326777	-0,98063	0,026224	-0,14514	lncRNA	AL031282.	novel transcript, readthrough between SLC35E2 and CDK11A	1
ENSG00000215790	62,07858	0,000812	0,849681	0,189525	0,025739	0,029313	transcribed_unproc	SLC35E2A	solute carrier family 35 member E2A [Source:HGNC Symbol;Acc:HGNC:20863]	1
ENSG00000008130	865,1356	-0,01452	0,196928	-1,29035	0,028314	-0,07263	protein_coding	NADK	NAD kinase [Source:HGNC Symbol;Acc:HGNC:29831]	1
ENSG00000067606	191,1346	-0,00094	0,898031	-0,12815	0,025135	-0,0119	protein_coding	PRKCZ	protein kinase C zeta [Source:HGNC Symbol;Acc:HGNC:9412]	1
ENSG00000182873	16,95471	-0,00172	0,39179	-0,85637	0,026087	-0,28242	lncRNA	PRKCZ-AS1	PRKCZ antisense RNA 1 [Source:HGNC Symbol;Acc:HGNC:40477]	1
ENSG00000162585	384,4809	0,008058	0,326316	0,981561	0,02602	0,069098	protein_coding	FAAP20	FA core complex associated protein 20 [Source:HGNC Symbol;Acc:HGNC:26428]	1
ENSG00000116151	120,5665	-0,01027	0,09788	-1,65522	0,02829	-0,18327	protein_coding	MORN1	MORN repeat containing 1 [Source:HGNC Symbol;Acc:HGNC:25852]	1
ENSG00000269896	21,68617	0,000353	0,889218	0,139293	0,025955	0,035835	transcribed_proces	AL513477.	small nuclear ribonucleoprotein N (SNRPN) pseudogene	1
ENSG00000157916	1314,68	0,030826	0,065586	1,841242	0,033839	0,068653	protein_coding	RER1	retention in endoplasmic reticulum sorting receptor 1 [Source:HGNC Symbol;Acc:HGNC:30309]	1
ENSG00000157911	270,8769	-0,00093	0,938428	-0,07725	0,024765	-0,00604	protein_coding	PEX10	peroxisomal biogenesis factor 10 [Source:HGNC Symbol;Acc:HGNC:8851]	1
ENSG00000149527	21,28617	-8,3E-05	0,967746	-0,04044	0,025996	-0,0128	protein_coding	PLCH2	phospholipase C eta 2 [Source:HGNC Symbol;Acc:HGNC:29037]	1
ENSG00000157881	364,3507	-0,00805	0,421729	-0,80342	0,025558	-0,05031	protein_coding	PANK4	pantothenate kinase 4 (inactive) [Source:HGNC Symbol;Acc:HGNC:19366]	1
ENSG00000238164	75,5152	-0,00399	0,388357	-0,8626	0,026101	-0,12385	lncRNA	TNFRSF14-	TNFRSF14 antisense RNA 1 [Source:HGNC Symbol;Acc:HGNC:26966]	1
ENSG00000157873	412,5068	-0,01004	0,436183	-0,77866	0,02778	-0,07212	protein_coding	TNFRSF14	TNF receptor superfamily member 14 [Source:HGNC Symbol;Acc:HGNC:11912]	1
ENSG00000157870	319,7009	0,001551	0,810761	0,239444	0,02508	0,02116	protein_coding	PRXL2B	peroxiredoxin like 2B [Source:HGNC Symbol;Acc:HGNC:28390]	1
ENSG00000215912	16,84275	0,001381	0,400437	0,840841	0,026065	0,291061	protein_coding	TTC34	tetratricopeptide repeat domain 34 [Source:HGNC Symbol;Acc:HGNC:34297]	1
ENSG00000177133	9,473101	0,000227	0,843681	0,197187	0,026052	0,100302	lncRNA	PRDM16-D	PRDM16 divergent transcript [Source:HGNC Symbol;Acc:HGNC:48664]	1
ENSG00000142611	44,73334	0,003447	0,22351	1,217248	0,026265	0,289567	protein_coding	PRDM16	PR/SET domain 16 [Source:HGNC Symbol;Acc:HGNC:14000]	1
ENSG00000130762	302,8394	-0,00577	0,378285	-0,88106	0,026092	-0,0868	protein_coding	ARHGEF16	Rho guanine nucleotide exchange factor 16 [Source:HGNC Symbol;Acc:HGNC:15515]	1
ENSG00000162591	263,9228	0,002271	0,591824	0,536195	0,025866	0,082657	protein_coding	MEGF6	multiple EGF like domains 6 [Source:HGNC Symbol;Acc:HGNC:3232]	1
ENSG00000158109	766,0767	0,001121	0,923637	0,095854	0,024035	0,005884	protein_coding	TPRG1L	tumor protein p63 regulated 1 like [Source:HGNC Symbol;Acc:HGNC:27007]	1
ENSG00000116213	246,6639	-0,01262	0,139428	-1,47792	0,028589	-0,10916	protein_coding	WRAP73	WD repeat containing, antisense to TP73 [Source:HGNC Symbol;Acc:HGNC:12759]	1
ENSG00000078900	150,8833	0,001979	0,333449	0,96719	0,02611	0,308721	protein_coding	TP73	tumor protein p73 [Source:HGNC Symbol;Acc:HGNC:12003]	1
ENSG00000227372	212,1398	-0,00749	0,269534	-1,10414	0,026676	-0,1075	transcribed_unitar	TP73-AS1	TP73 antisense RNA 1 [Source:HGNC Symbol;Acc:HGNC:29052]	1
ENSG00000130764	706,2292	-0,0036	0,729928	-0,34522	0,023654	-0,01823	protein_coding	LRRC47	leucine rich repeat containing 47 [Source:HGNC Symbol;Acc:HGNC:29207]	1
ENSG00000116198	1093,82	-0,0006	0,957904	-0,05278	0,022432	-0,00232	protein_coding	CEP104	centrosomal protein 104 [Source:HGNC Symbol;Acc:HGNC:24866]	1
ENSG00000169598	84,10244	-0,00059	0,901721	-0,12349	0,025625	-0,01679	protein_coding	DFFB	DNA fragmentation factor subunit beta [Source:HGNC Symbol;Acc:HGNC:2773]	1
ENSG00000198912	273,0688	-0,00053	0,976353	0,029641	0,024674	0,002249	protein_coding	C1orf174	chromosome 1 open reading frame 174 [Source:HGNC Symbol;Acc:HGNC:27915]	1
ENSG00000236423	37,7788	0,002701	0,298353	1,039971	0,026157	0,265183	lncRNA	LINC01134	long intergenic non-protein coding RNA 1134 [Source:HGNC Symbol;Acc:HGNC:49449]	1
ENSG00000233304	14,24184	0,000176	0,943411	0,070983	0,026053	0,03544	lncRNA	LINC01346	long intergenic non-protein coding RNA 1346 [Source:HGNC Symbol;Acc:HGNC:50563]	1
ENSG00000196581	134,1274	-0,00542	0,101654	-1,63689	0,026695	-0,32433	protein_coding	AJAP1	adherens junctions associated protein 1 [Source:HGNC Symbol;Acc:HGNC:30801]	1
ENSG00000131697	288,4347	-0,00512	0,501539	-0,67207	0,025624	-0,05773	protein_coding	NPHP4	nephrocystin 4 [Source:HGNC Symbol;Acc:HGNC:19104]	1
ENSG00000069424	498,9909	-0,0866	0,00976	-2,58423	0,116002	-0,16995	protein_coding	KCNAB2	potassium voltage-gated channel subfamily A regulatory beta subunit 2 [Source:HGNC Symbol;Acc:HGNC:6229]	1
ENSG00000116254	214,3162	-0,01732	0,028005	-2,19721	0,033818	-0,21411	protein_coding	CHD5	chromodomain helicase DNA binding protein 5 [Source:HGNC Symbol;Acc:HGNC:16816]	1
ENSG00000116251	619,1359	-0,00878	0,297979	-1,04078	0,026393	-0,07474	protein_coding	RPL22	ribosomal protein L22 [Source:HGNC Symbol;Acc:HGNC:10315]	1
ENSG00000158286	64,34925	-0,00604	0,071228	-1,80402	0,026906	-0,36037	protein_coding	RNF207	ring finger protein 207 [Source:HGNC Symbol;Acc:HGNC:32947]	1

ENSG00000158292	356,405	-0,01586	0,085169	-1,72145	0,030984	-0,12551	protein_coding	GPR153	G protein-coupled receptor 153 [Source:HGNC Symbol;Acc:HGNC:23618]	1
ENSG00000097021	1142,705	-0,0039	0,654726	-0,44721	0,024735	-0,03063	protein_coding	ACOT7	acyl-CoA thioesterase 7 [Source:HGNC Symbol;Acc:HGNC:24157]	1
ENSG00000069812	30,77225	0,000972	0,616399	0,50096	0,026037	0,17399	protein_coding	HES2	hes family bHLH transcription factor 2 [Source:HGNC Symbol;Acc:HGNC:16005]	1
ENSG00000187017	305,0055	0,000616	0,912717	0,109612	0,025448	0,012558	protein_coding	ESPN	espin [Source:HGNC Symbol;Acc:HGNC:13281]	1
ENSG00000215788	178,8698	-0,00275	0,340318	-0,95354	0,026132	-0,21767	protein_coding	TNFRSF25	TNF receptor superfamily member 25 [Source:HGNC Symbol;Acc:HGNC:11910]	1
ENSG00000171680	510,9953	-0,00871	0,118324	-1,56185	0,027601	-0,19015	protein_coding	PLEKHG5	pleckstrin homology and RhoGEF domain containing G5 [Source:HGNC Symbol;Acc:HGNC:29105]	1
ENSG00000162408	824,1312	0,0004	0,968099	0,039992	0,023444	0,002137	protein_coding	NOL9	nucleolar protein 9 [Source:HGNC Symbol;Acc:HGNC:26265]	1
ENSG00000204859	166,363	-0,00743	0,325845	-0,98252	0,026925	-0,11192	protein_coding	ZBTB48	zinc finger and BTB domain containing 48 [Source:HGNC Symbol;Acc:HGNC:4930]	1
ENSG00000162413	658,2372	-0,00703	0,505567	-0,66576	0,024472	-0,03555	protein_coding	KLHL21	kelch like family member 21 [Source:HGNC Symbol;Acc:HGNC:29041]	1
ENSG00000116273	340,5069	-0,01214	0,20874	-1,25704	0,027737	-0,08236	protein_coding	PHF13	PHD finger protein 13 [Source:HGNC Symbol;Acc:HGNC:22983]	1
ENSG00000041988	123,9603	-0,00643	0,327344	-0,97948	0,026446	-0,10491	protein_coding	THAP3	THAP domain containing 3 [Source:HGNC Symbol;Acc:HGNC:20855]	1
ENSG00000007923	960,752	-0,00121	0,900063	-0,12558	0,022277	-0,00536	protein_coding	DNAJC11	DnaJ heat shock protein family (Hsp40) member C11 [Source:HGNC Symbol;Acc:HGNC:25570]	1
ENSG00000171735	383,8795	0,010136	0,296098	1,044838	0,026527	0,066652	protein_coding	CAMTA1	calmodulin binding transcription activator 1 [Source:HGNC Symbol;Acc:HGNC:18806]	1
ENSG00000049245	1204,069	0,013271	0,306324	1,022966	0,02528	0,043187	protein_coding	VAMP3	vesicle associated membrane protein 3 [Source:HGNC Symbol;Acc:HGNC:12644]	1
ENSG00000049246	541,8459	0,000921	0,974463	0,032012	0,02435	0,002158	protein_coding	PER3	period circadian regulator 3 [Source:HGNC Symbol;Acc:HGNC:8847]	1
ENSG00000049249	31,22697	0,001806	0,535265	0,619989	0,026029	0,151252	protein_coding	TNFRSF9	TNF receptor superfamily member 9 [Source:HGNC Symbol;Acc:HGNC:11924]	1
ENSG00000162426	74,24173	-0,00396	0,398415	-0,84445	0,026073	-0,11829	protein_coding	SLC45A1	solute carrier family 45 member 1 [Source:HGNC Symbol;Acc:HGNC:17939]	1
ENSG00000131686	90,19244	3,26E-05	0,918542	0,10227	0,026082	0,242204	protein_coding	CA6	carbonic anhydrase 6 [Source:HGNC Symbol;Acc:HGNC:1380]	1
ENSG00000142583	21,0854	-0,00187	0,404334	-0,83391	0,026084	-0,24977	protein_coding	SLC2A5	solute carrier family 2 member 5 [Source:HGNC Symbol;Acc:HGNC:11010]	1
ENSG00000180758	327,2544	-0,01257	0,081655	-1,74116	0,029501	-0,16951	protein_coding	GPR157	G protein-coupled receptor 157 [Source:HGNC Symbol;Acc:HGNC:23687]	1
ENSG00000228526	331,9872	0,001658	0,664601	0,433569	0,025731	0,061721	lncRNA	MIR34AHG	MIR34A host gene [Source:HGNC Symbol;Acc:HGNC:51913]	1
ENSG00000234546	18,79027	0,004517	0,051976	1,943333	0,026568	0,581167	lncRNA	LNCTAM34	long non coding transcriptional activator of miR34a [Source:HGNC Symbol;Acc:HGNC:52548]	1
ENSG00000171621	693,8242	0,012451	0,208414	1,257938	0,026822	0,068209	protein_coding	SPSB1	splA/ryanodine receptor domain and SOCS box containing 1 [Source:HGNC Symbol;Acc:HGNC:30628]	1
ENSG00000284693	16,23697	0,001958	0,346029	0,94232	0,02611	0,312268	lncRNA	LINC02606	long intergenic non-protein coding RNA 2606 [Source:HGNC Symbol;Acc:HGNC:54047]	1
ENSG00000171612	196,5306	0,001977	0,914667	0,107154	0,025334	0,010666	protein_coding	SLC25A33	solute carrier family 25 member 33 [Source:HGNC Symbol;Acc:HGNC:29681]	1
ENSG00000188807	290,7294	-0,00012	0,984881	-0,01895	0,025101	-0,00175	protein_coding	TMEM201	transmembrane protein 201 [Source:HGNC Symbol;Acc:HGNC:33719]	1
ENSG00000171608	179,9479	0,003598	0,527108	0,632426	0,025752	0,071018	protein_coding	PIK3CD	phosphatidylinositol-4,5-bisphosphate 3-kinase catalytic subunit delta [Source:HGNC Symbol;Acc:HGNC:8977]	1
ENSG00000231789	22,75531	-0,00149	0,56253	-0,57909	0,026015	-0,15033	lncRNA	PIK3CD-AS:	PIK3CD antisense RNA 2 [Source:HGNC Symbol;Acc:HGNC:51334]	1
ENSG00000178585	282,3522	0,00529	0,444811	0,764094	0,02586	0,071834	protein_coding	CTNNBIP1	catenin beta interacting protein 1 [Source:HGNC Symbol;Acc:HGNC:16913]	1
ENSG00000162441	627,2068	0,014175	0,199118	1,284068	0,028175	0,073153	protein_coding	LZIC	leucine zipper and CTNNBIP1 domain containing [Source:HGNC Symbol;Acc:HGNC:17497]	1
ENSG00000173614	138,5072	0,000843	0,898758	0,12723	0,025195	0,012268	protein_coding	NMNAT1	nicotinamide nucleotide adenylyltransferase 1 [Source:HGNC Symbol;Acc:HGNC:17877]	1
ENSG00000162444	10,06906	8,34E-05	0,949833	0,062917	0,02605	0,033078	protein_coding	RBP7	retinol binding protein 7 [Source:HGNC Symbol;Acc:HGNC:30316]	1
ENSG00000130939	1374,41	0,008682	0,489147	0,691665	0,022687	0,026224	protein_coding	UBE4B	ubiquitination factor E4B [Source:HGNC Symbol;Acc:HGNC:12500]	1
ENSG00000199562	87,94723	-0,00106	0,644268	-0,46174	0,026007	-0,12948	snRNA	RNU6-37P	RNA, U6 small nuclear 37, pseudogene [Source:HGNC Symbol;Acc:HGNC:34281]	1
ENSG00000175279	11,20119	0,000286	0,886761	0,142404	0,026007	0,047706	protein_coding	CENPS	centromere protein S [Source:HGNC Symbol;Acc:HGNC:23163]	1
ENSG00000160049	808,4553	-0,01006	0,346412	-0,94157	0,025574	-0,05032	protein_coding	DFFA	DNA fragmentation factor subunit alpha [Source:HGNC Symbol;Acc:HGNC:2772]	1
ENSG00000142655	311,6393	-0,014	0,093761	-1,67589	0,030078	-0,13966	protein_coding	PEX14	peroxisomal biogenesis factor 14 [Source:HGNC Symbol;Acc:HGNC:8856]	1
ENSG00000130940	496,7234	-0,24162	0,00122	-3,23426	0,119825	-0,31937	protein_coding	CASZ1	castor zinc finger 1 [Source:HGNC Symbol;Acc:HGNC:26002]	1
ENSG00000175262	42,08469	-0,00091	0,334497	-0,96509	0,026076	-0,47109	protein_coding	C1orf127	chromosome 1 open reading frame 127 [Source:HGNC Symbol;Acc:HGNC:26730]	1
ENSG00000116649	1473,049	0,010949	0,374568	0,88795	0,025057	0,0414	protein_coding	SRM	spermidine synthase [Source:HGNC Symbol;Acc:HGNC:11296]	1
ENSG00000171824	1219,934	0,019515	0,214478	1,241346	0,02963	0,055988	protein_coding	EXOSC10	exosome component 10 [Source:HGNC Symbol;Acc:HGNC:9138]	1
ENSG00000120942	237,3156	0,004362	0,569959	0,568112	0,025181	0,043888	protein_coding	UBIAD1	UbiA prenyltransferase domain containing 1 [Source:HGNC Symbol;Acc:HGNC:30791]	1
ENSG00000204624	46,38398	-0,00055	0,862609	-0,17305	0,025894	-0,03629	protein_coding	DISP3	dispatched RND transporter family member 3 [Source:HGNC Symbol;Acc:HGNC:29251]	1
ENSG00000116661	83,83242	-0,00568	0,052177	-1,94167	0,026842	-0,44752	protein_coding	FBXO2	F-box protein 2 [Source:HGNC Symbol;Acc:HGNC:13581]	1
ENSG00000132879	125,3301	-0,00222	0,70779	-0,37483	0,025639	-0,04562	protein_coding	FBXO44	F-box protein 44 [Source:HGNC Symbol;Acc:HGNC:24847]	1
ENSG00000116663	31,97395	-0,00198	0,513124	-0,65398	0,026028	-0,15054	protein_coding	FBXO6	F-box protein 6 [Source:HGNC Symbol;Acc:HGNC:13585]	1
ENSG00000116670	285,5818	0,007466	0,355692	0,923606	0,026133	0,072168	protein_coding	MAD2L2	mitotic arrest deficient 2 like 2 [Source:HGNC Symbol;Acc:HGNC:6764]	1
ENSG00000162490	42,99094	0,002659	0,280247	1,079766	0,026163	0,288756	protein_coding	DRAXIN	dorsal inhibitory axon guidance protein [Source:HGNC Symbol;Acc:HGNC:25054]	1
ENSG00000177674	180,0918	0,014083	0,112204	1,588365	0,030282	0,137601	protein_coding	AGTRAP	angiotensin II receptor associated protein [Source:HGNC Symbol;Acc:HGNC:13539]	1
ENSG00000177000	565,2967	-0,01743	0,100389	-1,64297	0,031824	-0,10975	protein_coding	MTHFR	methylenetetrahydrofolate reductase [Source:HGNC Symbol;Acc:HGNC:7436]	1
ENSG00000011021	307,1326	-0,06388	0,011568	-2,52506	0,135071	-0,16337	protein_coding	CLCN6	chloride voltage-gated channel 6 [Source:HGNC Symbol;Acc:HGNC:2024]	1
ENSG00000175206	161,5676	-0,00036	0,737269	-0,33547	0,026065	-0,20435	protein_coding	NPPA	natriuretic peptide A [Source:HGNC Symbol;Acc:HGNC:7939]	1

ENSG00000285646	38,48128	-0,00172	0,571769	-0,56545	0,025986	-0,12534	lncRNA	AL021155.	novel transcript	1
ENSG00000201801	14,26897	0,000521	0,77526	0,285502	0,026026	0,10672	snRNA	RNU5E-4P	RNA, U5E small nuclear 4, pseudogene [Source:HGNC Symbol;Acc:HGNC:42513]	1
ENSG00000116685	1007,884	0,006673	0,556418	0,58817	0,023596	0,027341	protein_coding	KIAA2013	KIAA2013 [Source:HGNC Symbol;Acc:HGNC:28513]	1
ENSG00000116691	149,9353	0,008074	0,20688	1,262192	0,02709	0,134788	protein_coding	MIIP	migration and invasion inhibitory protein [Source:HGNC Symbol;Acc:HGNC:25715]	1
ENSG00000028137	202,6248	-0,00075	0,969735	-0,03794	0,025635	-0,00518	protein_coding	TNFRSF1B	TNF receptor superfamily member 1B [Source:HGNC Symbol;Acc:HGNC:11917]	1
ENSG00000162496	222,2885	0,006327	0,173125	1,36223	0,026781	0,196166	protein_coding	DHRS3	dehydrogenase/reductase 3 [Source:HGNC Symbol;Acc:HGNC:17693]	1
ENSG00000116726	12,22065	-5,9E-05	0,758343	-0,30766	0,026082	-1,12261	protein_coding	PRAMEF12	PRAME family member 12 [Source:HGNC Symbol;Acc:HGNC:22125]	1
ENSG00000162493	84,16866	0,000729	0,776658	0,283677	0,025968	0,073497	protein_coding	PDPN	podoplanin [Source:HGNC Symbol;Acc:HGNC:29602]	1
ENSG00000189337	265,7768	0,000483	0,979999	0,02507	0,025001	0,00219	protein_coding	KAZN	kazrin, periplakin interacting protein [Source:HGNC Symbol;Acc:HGNC:29173]	1
ENSG00000175147	63,6045	-0,00587	0,102265	-1,63397	0,026816	-0,31247	lncRNA	TMEM51-A	TMEM51 antisense RNA 1 [Source:HGNC Symbol;Acc:HGNC:26301]	1
ENSG00000171729	455,2288	-0,00726	0,44568	-0,76264	0,025286	-0,04784	protein_coding	TMEM51	transmembrane protein 51 [Source:HGNC Symbol;Acc:HGNC:25488]	1
ENSG00000142621	20,34074	0,002008	0,367152	0,901822	0,0261	0,270128	protein_coding	FHAD1	forkhead associated phosphopeptide binding domain 1 [Source:HGNC Symbol;Acc:HGNC:29408]	1
ENSG00000142634	1150,844	0,011303	0,316147	1,002408	0,025543	0,048968	protein_coding	EFHD2	EF-hand domain family member D2 [Source:HGNC Symbol;Acc:HGNC:28670]	1
ENSG00000132906	133,1887	-0,00613	0,315523	-1,0037	0,026428	-0,11348	protein_coding	CASP9	caspase 9 [Source:HGNC Symbol;Acc:HGNC:1511]	1
ENSG00000116138	602,7838	-0,01122	0,280468	-1,07927	0,026508	-0,06178	protein_coding	DNAJC16	DnaJ heat shock protein family (Hsp40) member C16 [Source:HGNC Symbol;Acc:HGNC:29157]	1
ENSG00000116771	123,9782	-0,00339	0,368433	-0,89941	0,026133	-0,16036	protein_coding	AGMAT	agmatinase [Source:HGNC Symbol;Acc:HGNC:18407]	1
ENSG00000197312	949,7611	0,009461	0,368481	0,899322	0,025275	0,047656	protein_coding	DDI2	DNA damage inducible 1 homolog 2 [Source:HGNC Symbol;Acc:HGNC:24578]	1
ENSG00000162461	35,82818	0,000564	0,844777	0,195787	0,025929	0,045369	protein_coding	SLC25A34	solute carrier family 25 member 34 [Source:HGNC Symbol;Acc:HGNC:27653]	1
ENSG00000162458	984,1854	-0,00111	0,876771	-0,15506	0,024164	-0,00987	protein_coding	FBLIM1	filamin binding LIM protein 1 [Source:HGNC Symbol;Acc:HGNC:24686]	1
ENSG00000233954	69,17786	0,002046	0,632561	0,478126	0,025838	0,074321	protein_coding	UQCRHL	ubiquinol-cytochrome c reductase hinge protein like [Source:HGNC Symbol;Acc:HGNC:51714]	1
ENSG00000179743	57,51634	-0,00563	0,182415	-1,33336	0,026636	-0,21502	lncRNA	FLJ37453	uncharacterized LOC729614 [Source:NCBI gene (formerly Entrezgene);Acc:729614]	1
ENSG00000116809	492,5165	2,94E-05	0,984086	-0,01995	0,023889	-0,00119	protein_coding	ZBTB17	zinc finger and BTB domain containing 17 [Source:HGNC Symbol;Acc:HGNC:12936]	1
ENSG00000173641	159,6281	-0,00208	0,674357	-0,42018	0,025704	-0,05525	protein_coding	HSPB7	heat shock protein family B (small) member 7 [Source:HGNC Symbol;Acc:HGNC:5249]	1
ENSG00000185519	47,34041	-0,00266	0,359753	-0,91584	0,026122	-0,21611	protein_coding	FAM131C	family with sequence similarity 131 member C [Source:HGNC Symbol;Acc:HGNC:26717]	1
ENSG00000142627	1347,2	-0,02607	0,05964	-1,88344	0,038996	-0,1036	protein_coding	EPHA2	EPH receptor A2 [Source:HGNC Symbol;Acc:HGNC:3386]	1
ENSG00000142632	63,05065	-0,0031	0,489531	-0,69105	0,026002	-0,10926	protein_coding	ARHGEF19	Rho guanine nucleotide exchange factor 19 [Source:HGNC Symbol;Acc:HGNC:26604]	1
ENSG00000237276	10,70439	-0,00026	0,886391	-0,14287	0,026019	-0,05254	transcribed_unpro	ANO7L1	anoctamin 7 like 1 (pseudogene) [Source:HGNC Symbol;Acc:HGNC:32248]	1
ENSG00000288398	74,00461	0,000868	0,818453	0,229535	0,025823	0,040302	lncRNA	AL109627.	novel transcript	1
ENSG00000132881	38,66033	0,001635	0,664113	0,434242	0,025879	0,076955	protein_coding	CPLANE2	ciliogenesis and planar polarity effector 2 [Source:HGNC Symbol;Acc:HGNC:28127]	1
ENSG00000037637	446,9683	-0,00526	0,548552	-0,59993	0,02503	-0,04108	protein_coding	FBXO42	F-box protein 42 [Source:HGNC Symbol;Acc:HGNC:29249]	1
ENSG00000157191	713,4365	0,001666	0,876403	0,155531	0,023847	0,009015	protein_coding	NECAP2	NECAP endocytosis associated 2 [Source:HGNC Symbol;Acc:HGNC:25528]	1
ENSG00000226029	19,15252	-0,00192	0,442825	-0,76743	0,026069	-0,2094	lncRNA	LINC01772	long intergenic non-protein coding RNA 1772 [Source:HGNC Symbol;Acc:HGNC:52562]	1
ENSG00000080947	63,12925	-0,00264	0,518636	-0,64545	0,025993	-0,11243	transcribed_unpro	CROCCP3	CROCC pseudogene 3 [Source:HGNC Symbol;Acc:HGNC:29405]	1
ENSG00000206652	275,3034	0,000582	0,934852	0,081742	0,025091	0,007484	snRNA	RNU1-1	RNA, U1 small nuclear 1 [Source:HGNC Symbol;Acc:HGNC:10120]	1
ENSG00000219481	1105,33	-0,10261	0,003547	-2,91591	0,057398	-0,13993	protein_coding	NBPF1	NBPF member 1 [Source:HGNC Symbol;Acc:HGNC:26088]	1
ENSG00000215908	242,3876	-0,01758	0,056943	-1,90375	0,034285	-0,19402	transcribed_unpro	CROCCP2	CROCC pseudogene 2 [Source:HGNC Symbol;Acc:HGNC:28170]	1
ENSG00000186301	19,94128	0,000312	0,902765	0,12217	0,02596	0,032309	unprocessed_pseu	MST1P2	macrophage stimulating 1 pseudogene 2 [Source:HGNC Symbol;Acc:HGNC:7383]	1
ENSG00000207513	24,76201	-0,00251	0,250849	-1,14829	0,026167	-0,34382	snRNA	RNU1-3	RNA, U1 small nuclear 3 [Source:HGNC Symbol;Acc:HGNC:10130]	1
ENSG00000236698	13,57088	-9,7E-05	0,923733	-0,09573	0,025977	-0,02761	processed_pseudo	EIF1AXP1	EIF1AX pseudogene 1 [Source:HGNC Symbol;Acc:HGNC:19900]	1
ENSG00000268869	10,80748	0,002518	0,101202	1,639053	0,02622	0,734507	transcribed_unpro	ESPNP	espin pseudogene [Source:HGNC Symbol;Acc:HGNC:23285]	1
ENSG00000058453	498,5325	-0,01945	0,085293	-1,72077	0,033132	-0,10653	protein_coding	CROCC	ciliary rootlet coiled-coil, rootletin [Source:HGNC Symbol;Acc:HGNC:21299]	1
ENSG00000207389	21,76238	-0,00491	0,029921	-2,17113	0,026679	-0,65283	snRNA	RNU1-4	RNA, U1 small nuclear 4 [Source:HGNC Symbol;Acc:HGNC:10128]	1
ENSG00000228549	10,87348	0,001123	0,350934	0,932779	0,026091	0,529263	lncRNA	BX284668.	novel transcript	1
ENSG00000238142	33,96895	0,001767	0,598309	0,526834	0,025951	0,105498	lncRNA	BX284668.	novel transcript	1
ENSG00000207005	49,29569	-0,0047	0,246556	-1,15876	0,026389	-0,19412	snRNA	RNU1-2	RNA, U1 small nuclear 2 [Source:HGNC Symbol;Acc:HGNC:10123]	1
ENSG00000117122	1017,672	0,001551	0,985481	0,018198	0,02562	0,002322	protein_coding	MFAP2	microfibril associated protein 2 [Source:HGNC Symbol;Acc:HGNC:7033]	1
ENSG00000159363	938,9541	0,005983	0,552148	0,594545	0,024341	0,032767	protein_coding	ATP13A2	ATPase cation transporting 13A2 [Source:HGNC Symbol;Acc:HGNC:30213]	1
ENSG00000117118	1020,839	0,004109	0,712867	0,368009	0,022884	0,016563	protein_coding	SDHB	succinate dehydrogenase complex iron sulfur subunit B [Source:HGNC Symbol;Acc:HGNC:10681]	1
ENSG00000117115	343,7686	-0,0002	0,913163	-0,10905	0,026014	-0,03895	protein_coding	PADI2	peptidyl arginine deiminase 2 [Source:HGNC Symbol;Acc:HGNC:18341]	1
ENSG00000159339	10,07099	4,21E-06	0,98668	0,016696	0,026082	0,061354	protein_coding	PADI4	peptidyl arginine deiminase 4 [Source:HGNC Symbol;Acc:HGNC:18368]	1
ENSG00000074964	699,0275	-0,00731	0,312802	-1,00936	0,026416	-0,08999	protein_coding	ARHGEF10	Rho guanine nucleotide exchange factor 10 like [Source:HGNC Symbol;Acc:HGNC:25540]	1
ENSG00000117154	104,7938	0,002581	0,393638	0,853038	0,026092	0,188924	protein_coding	IGSF21	immunoglobin superfamily member 21 [Source:HGNC Symbol;Acc:HGNC:28246]	1

ENSG00000179023	150,7204	0,002054	0,580318	0,55292	0,02593	0,099462	protein_coding	KLHDC7A	kelch domain containing 7A [Source:HGNC Symbol;Acc:HGNC:26791]	1
ENSG00000159423	82,80828	-0,00221	0,52793	-0,63117	0,025919	-0,10714	protein_coding	ALDH4A1	aldehyde dehydrogenase 4 family member A1 [Source:HGNC Symbol;Acc:HGNC:406]	1
ENSG00000169991	204,38	0,002604	0,697854	0,388219	0,02532	0,036718	protein_coding	IFFO2	intermediate filament family orphan 2 [Source:HGNC Symbol;Acc:HGNC:27006]	1
ENSG00000272084	11,87879	-0,00086	0,661346	-0,43806	0,026033	-0,15296	lncRNA	AL137127.	novel transcript	1
ENSG00000230424	17,67651	-0,00082	0,749497	-0,3193	0,025971	-0,0829	lncRNA	EMC1-AS1	EMC1 antisense RNA 1 [Source:HGNC Symbol;Acc:HGNC:54050]	1
ENSG00000053372	532,7774	0,009983	0,31807	0,998433	0,025593	0,053885	protein_coding	MRTO4	MRT4 homolog, ribosome maturation factor [Source:HGNC Symbol;Acc:HGNC:18477]	1
ENSG00000211454	26,25081	-0,00246	0,351249	-0,93217	0,026155	-0,27764	polymorphic_pseu	AKR7L	aldo-keto reductase family 7 like (gene/pseudogene) [Source:HGNC Symbol;Acc:HGNC:24056]	1
ENSG00000162482	84,86379	0,002283	0,321562	0,991252	0,026125	0,280199	protein_coding	AKR7A3	aldo-keto reductase family 7 member A3 [Source:HGNC Symbol;Acc:HGNC:390]	1
ENSG00000053371	404,1251	0,005429	0,539026	0,614286	0,025171	0,043368	protein_coding	AKR7A2	aldo-keto reductase family 7 member A2 [Source:HGNC Symbol;Acc:HGNC:389]	1
ENSG00000040487	233,1515	-0,00717	0,361864	-0,91182	0,026172	-0,07537	protein_coding	SLC66A1	solute carrier family 66 member 1 [Source:HGNC Symbol;Acc:HGNC:26001]	1
ENSG00000173436	285,2582	0,008306	0,31567	1,003395	0,026414	0,077146	protein_coding	MICOS10	mitochondrial contact site and cristae organizing system subunit 10 [Source:HGNC Symbol;Acc:HGNC:32068]	1
ENSG00000158747	708,934	0,003358	0,721559	0,356376	0,02411	0,021057	protein_coding	NBL1	NBL1, DAN family BMP antagonist [Source:HGNC Symbol;Acc:HGNC:7650]	1
ENSG00000162542	116,5015	-0,00229	0,661981	-0,43718	0,025705	-0,05629	protein_coding	TMCO4	transmembrane and coiled-coil domains 4 [Source:HGNC Symbol;Acc:HGNC:27393]	1
ENSG00000178828	12,60263	0,000903	0,529496	0,628775	0,026067	0,296512	protein_coding	RNF186	ring finger protein 186 [Source:HGNC Symbol;Acc:HGNC:25978]	1
ENSG00000169914	267,7393	0,00668	0,332591	0,968908	0,025876	0,076573	protein_coding	OTUD3	OTU deubiquitinase 3 [Source:HGNC Symbol;Acc:HGNC:29038]	1
ENSG00000188257	16,60956	2,32E-05	0,976099	0,02996	0,026082	0,110095	protein_coding	PLA2G2A	phospholipase A2 group IIA [Source:HGNC Symbol;Acc:HGNC:9031]	1
ENSG00000127472	15,11754	-0,00276	0,137946	-1,48348	0,026234	-0,53387	protein_coding	PLA2G5	phospholipase A2 group V [Source:HGNC Symbol;Acc:HGNC:9038]	1
ENSG00000162543	27,34166	-0,00167	0,536493	-0,61813	0,026018	-0,15279	protein_coding	UBXN10	UBX domain protein 10 [Source:HGNC Symbol;Acc:HGNC:26354]	1
ENSG00000162545	1312,401	-0,01383	0,142068	-1,46813	0,029252	-0,10445	protein_coding	CAMK2N1	calcium/calmodulin dependent protein kinase II inhibitor 1 [Source:HGNC Symbol;Acc:HGNC:24190]	1
ENSG00000090432	536,2014	0,004071	0,649475	0,454491	0,023494	0,02289	protein_coding	MUL1	mitochondrial E3 ubiquitin protein ligase 1 [Source:HGNC Symbol;Acc:HGNC:25762]	1
ENSG00000183114	31,56816	-0,00099	0,65428	-0,44782	0,026013	-0,13406	protein_coding	FAM43B	family with sequence similarity 43 member B [Source:HGNC Symbol;Acc:HGNC:31791]	1
ENSG00000158825	29,36697	-0,00022	0,90387	-0,12077	0,026016	-0,04264	protein_coding	CDA	cytidine deaminase [Source:HGNC Symbol;Acc:HGNC:1712]	1
ENSG00000158828	1368,423	-0,00567	0,596042	-0,5301	0,022554	-0,02171	protein_coding	PINK1	PTEN induced kinase 1 [Source:HGNC Symbol;Acc:HGNC:14581]	1
ENSG00000117242	103,5999	-0,00171	0,768845	-0,29389	0,02554	-0,03449	lncRNA	PINK1-AS	PINK1 antisense RNA [Source:HGNC Symbol;Acc:HGNC:38872]	1
ENSG00000117245	33,06659	0,000769	0,748872	0,320127	0,025986	0,088713	protein_coding	KIF17	kinesin family member 17 [Source:HGNC Symbol;Acc:HGNC:19167]	1
ENSG00000189410	26,74771	0,00093	0,74478	0,325529	0,025961	0,079993	protein_coding	SH2D5	SH2 domain containing 5 [Source:HGNC Symbol;Acc:HGNC:28819]	1
ENSG00000142794	194,6556	-0,00569	0,386271	-0,8664	0,026066	-0,08551	protein_coding	NBPF3	NBPF member 3 [Source:HGNC Symbol;Acc:HGNC:25076]	1
ENSG00000162551	34,02053	-0,00068	0,736179	-0,33692	0,026016	-0,1131	protein_coding	ALPL	alkaline phosphatase, biomineralization associated [Source:HGNC Symbol;Acc:HGNC:438]	1
ENSG00000076864	853,631	0,004546	0,489797	0,690632	0,025733	0,066983	protein_coding	RAP1GAP	RAP1 GTPase activating protein [Source:HGNC Symbol;Acc:HGNC:9858]	1
ENSG00000090686	1222,342	0,008509	0,496188	0,680499	0,023332	0,028215	protein_coding	USP48	ubiquitin specific peptidase 48 [Source:HGNC Symbol;Acc:HGNC:18533]	1
ENSG00000218510	39,87093	0,000582	0,869557	0,164222	0,025847	0,030959	lncRNA	LINC00339	long intergenic non-protein coding RNA 339 [Source:HGNC Symbol;Acc:HGNC:25011]	1
ENSG00000162552	245,7026	0,000946	0,838164	0,204242	0,025681	0,029067	protein_coding	WNT4	Wnt family member 4 [Source:HGNC Symbol;Acc:HGNC:12783]	1
ENSG00000184677	967,6403	-0,02178	0,097327	-1,65795	0,033304	-0,0856	protein_coding	ZBTB40	zinc finger and BTB domain containing 40 [Source:HGNC Symbol;Acc:HGNC:29045]	1
ENSG00000070886	10,6933	0,000908	0,433444	0,783312	0,026082	0,459279	protein_coding	EPHA8	EPH receptor A8 [Source:HGNC Symbol;Acc:HGNC:3391]	1
ENSG00000173372	42,13822	-0,0034	0,124539	-1,536	0,026317	-0,45721	protein_coding	C1QA	complement C1q A chain [Source:HGNC Symbol;Acc:HGNC:1241]	1
ENSG00000159189	240,1419	-0,00608	0,199995	-1,28156	0,026709	-0,1873	protein_coding	C1QC	complement C1q C chain [Source:HGNC Symbol;Acc:HGNC:1245]	1
ENSG00000173369	223,5978	-0,00423	0,206025	-1,26457	0,026368	-0,24899	protein_coding	C1QB	complement C1q B chain [Source:HGNC Symbol;Acc:HGNC:1242]	1
ENSG00000133216	348,4286	0,002845	0,641229	0,465982	0,025484	0,047459	protein_coding	EPHB2	EPH receptor B2 [Source:HGNC Symbol;Acc:HGNC:3393]	1
ENSG00000179546	15,7977	-0,00064	0,734589	-0,33903	0,026023	-0,11892	protein_coding	HTR1D	5-hydroxytryptamine receptor 1D [Source:HGNC Symbol;Acc:HGNC:5289]	1
ENSG00000261326	41,04483	-0,0082	0,007752	-2,66268	0,027885	-0,64112	lncRNA	LINC01355	long intergenic non-protein coding RNA 1355 [Source:HGNC Symbol;Acc:HGNC:50584]	1
ENSG00000125945	260,501	-0,0016	0,831787	-0,21241	0,024985	-0,01791	protein_coding	ZNF436	zinc finger protein 436 [Source:HGNC Symbol;Acc:HGNC:20814]	1
ENSG00000249087	19,35936	-0,00264	0,290335	-1,05739	0,026147	-0,27096	lncRNA	ZNF436-AS	ZNF436 antisense RNA 1 [Source:HGNC Symbol;Acc:HGNC:25122]	1
ENSG00000204219	194,5937	0,007034	0,134275	1,497455	0,027053	0,217973	protein_coding	TCEA3	transcription elongation factor A3 [Source:HGNC Symbol;Acc:HGNC:11615]	1
ENSG00000088280	137,0916	0,004266	0,478086	0,709384	0,025838	0,076704	protein_coding	ASAP3	ArfGAP with SH3 domain, ankyrin repeat and PH domain 3 [Source:HGNC Symbol;Acc:HGNC:14987]	1
ENSG00000007968	205,5445	0,003993	0,412661	0,81922	0,026047	0,110243	protein_coding	E2F2	E2F transcription factor 2 [Source:HGNC Symbol;Acc:HGNC:3114]	1
ENSG00000236810	13,16136	0,00154	0,448578	0,757787	0,026075	0,256874	lncRNA	ELOA-AS1	ELOA antisense RNA 1 [Source:HGNC Symbol;Acc:HGNC:50582]	1
ENSG00000057757	673,8208	-0,00644	0,512471	-0,65499	0,0249	-0,03983	protein_coding	PITHD1	PITH domain containing 1 [Source:HGNC Symbol;Acc:HGNC:25022]	1
ENSG00000011009	488,3578	-0,00049	0,941906	-0,07287	0,023849	-0,00429	protein_coding	LYPLA2	lysophospholipase 2 [Source:HGNC Symbol;Acc:HGNC:6738]	1
ENSG00000117308	242,87	0,009137	0,263168	1,118935	0,026908	0,089496	protein_coding	GALE	UDP-galactose-4-epimerase [Source:HGNC Symbol;Acc:HGNC:4116]	1
ENSG00000117305	264,4663	0,006064	0,449664	0,755975	0,025632	0,058665	protein_coding	HMGCL	3-hydroxy-3-methylglutaryl-CoA lyase [Source:HGNC Symbol;Acc:HGNC:5005]	1
ENSG00000179163	1239,96	-0,00633	0,382537	-0,87323	0,026036	-0,07663	protein_coding	FUCA1	alpha-L-fucosidase 1 [Source:HGNC Symbol;Acc:HGNC:4006]	1
ENSG00000189266	349,6714	0,007086	0,361879	0,911791	0,025898	0,069089	protein_coding	PNRC2	proline rich nuclear receptor coactivator 2 [Source:HGNC Symbol;Acc:HGNC:23158]	1

ENSG00000188529	772,7471	0,017345	0,094753	1,670844	0,031888	0,113882	protein_coding	SRSF10	serine and arginine rich splicing factor 10 [Source:HGNC Symbol;Acc:HGNC:16713]	1
ENSG00000142661	36,8923	-0,00043	0,837487	-0,20511	0,026001	-0,0645	protein_coding	MYOM3	myomesin 3 [Source:HGNC Symbol;Acc:HGNC:26679]	1
ENSG00000142677	70,32442	-0,00018	0,958422	-0,05213	0,025835	-0,00979	protein_coding	IL22RA1	interleukin 22 receptor subunit alpha 1 [Source:HGNC Symbol;Acc:HGNC:13700]	1
ENSG00000185436	90,09724	0,000137	0,997435	0,003215	0,025584	0,000424	protein_coding	IFNLR1	interferon lambda receptor 1 [Source:HGNC Symbol;Acc:HGNC:18584]	1
ENSG00000158055	13,09883	0,000486	0,763236	0,301234	0,026039	0,126931	protein_coding	GRHL3	grainyhead like transcription factor 3 [Source:HGNC Symbol;Acc:HGNC:25839]	1
ENSG00000001460	113,6334	-0,00082	0,868781	-0,16521	0,025494	-0,0196	protein_coding	STPG1	sperm tail PG-rich repeat containing 1 [Source:HGNC Symbol;Acc:HGNC:28070]	1
ENSG00000001461	1037,684	-9,5E-05	0,979914	0,025176	0,024762	0,001983	protein_coding	NIPAL3	NIPA like domain containing 3 [Source:HGNC Symbol;Acc:HGNC:25233]	1
ENSG00000117602	703,0579	0,010038	0,349907	0,93477	0,025695	0,051165	protein_coding	RCAN3	RCAN family member 3 [Source:HGNC Symbol;Acc:HGNC:3042]	1
ENSG00000184454	38,02795	0,000814	0,787444	0,269632	0,025919	0,059056	protein_coding	NCMAP	non-compact myelin associated protein [Source:HGNC Symbol;Acc:HGNC:29332]	1
ENSG00000020633	129,8859	0,00183	0,573786	0,562485	0,025968	0,114711	protein_coding	RUNX3	RUNX family transcription factor 3 [Source:HGNC Symbol;Acc:HGNC:10473]	1
ENSG00000117614	858,6727	0,00969	0,462483	0,734765	0,02473	0,035129	protein_coding	SYF2	SYF2 pre-mRNA splicing factor [Source:HGNC Symbol;Acc:HGNC:19824]	1
ENSG00000284602	11,84512	0,000148	0,945544	0,068303	0,025996	0,021648	lncRNA	AL031432.	novel transcript	1
ENSG00000117616	651,3203	-0,01063	0,296903	-1,0431	0,026678	-0,06519	protein_coding	RSRP1	arginine and serine rich protein 1 [Source:HGNC Symbol;Acc:HGNC:25234]	1
ENSG00000183726	897,3357	0,009422	0,424915	0,797924	0,024475	0,03728	protein_coding	TMEM50A	transmembrane protein 50A [Source:HGNC Symbol;Acc:HGNC:30590]	1
ENSG00000204178	1040,069	0,001054	0,911032	0,111737	0,02263	0,005065	protein_coding	MACO1	macoilin 1 [Source:HGNC Symbol;Acc:HGNC:25572]	1
ENSG00000157978	326,4767	0,00088	0,881971	0,148471	0,025081	0,013388	protein_coding	LDLRAP1	low density lipoprotein receptor adaptor protein 1 [Source:HGNC Symbol;Acc:HGNC:18640]	1
ENSG00000117643	123,4406	-0,0073	0,147513	-1,44837	0,027082	-0,19465	protein_coding	MAN1C1	mannosidase alpha class 1C member 1 [Source:HGNC Symbol;Acc:HGNC:19080]	1
ENSG00000228172	21,56877	-0,00132	0,600694	-0,5234	0,026008	-0,13902	lncRNA	AL020996.	novel transcript	1
ENSG00000117640	370,4181	-0,00609	0,487092	-0,69494	0,025344	-0,0489	protein_coding	MTFR1L	mitochondrial fission regulator 1 like [Source:HGNC Symbol;Acc:HGNC:28836]	1
ENSG00000127423	49,94161	-0,00299	0,336632	-0,96084	0,026144	-0,20527	protein_coding	AUNIP	aurora kinase A and ninein interacting protein [Source:HGNC Symbol;Acc:HGNC:28363]	1
ENSG00000182749	166,8677	-0,00077	0,904559	-0,1199	0,025073	-0,01082	protein_coding	PAQR7	progestin and adipoQ receptor family member 7 [Source:HGNC Symbol;Acc:HGNC:23146]	1
ENSG00000158006	243,9667	0,002578	0,711538	0,369791	0,025233	0,033458	protein_coding	PAFAH2	platelet activating factor acetylhydrolase 2 [Source:HGNC Symbol;Acc:HGNC:8579]	1
ENSG00000158008	34,3167	-0,00312	0,338238	-0,95765	0,026186	-0,21605	protein_coding	EXTL1	exostosin like glycosyltransferase 1 [Source:HGNC Symbol;Acc:HGNC:3515]	1
ENSG00000175087	174,5328	-0,00293	0,683571	-0,4076	0,025198	-0,03535	protein_coding	PDIK1L	PDLIM1 interacting kinase 1 like [Source:HGNC Symbol;Acc:HGNC:18981]	1
ENSG00000142684	259,9472	-0,00147	0,822415	-0,22444	0,025233	-0,02156	protein_coding	ZNF593	zinc finger protein 593 [Source:HGNC Symbol;Acc:HGNC:30943]	1
ENSG00000142675	98,05304	7,53E-05	0,985845	0,017741	0,025746	0,00285	protein_coding	CNKSR1	connector enhancer of kinase suppressor of Ras 1 [Source:HGNC Symbol;Acc:HGNC:19700]	1
ENSG00000130695	461,8272	-0,00867	0,339169	-0,95581	0,026301	-0,06786	protein_coding	CEP85	centrosomal protein 85 [Source:HGNC Symbol;Acc:HGNC:25309]	1
ENSG00000158062	248,507	-0,00393	0,554165	-0,59153	0,025568	-0,05661	protein_coding	UBXN11	UBX domain protein 11 [Source:HGNC Symbol;Acc:HGNC:30600]	1
ENSG00000169442	22,12534	0,002472	0,109086	1,602316	0,02621	0,676121	protein_coding	CD52	CD52 molecule [Source:HGNC Symbol;Acc:HGNC:1804]	1
ENSG00000117682	717,5178	-0,00964	0,347565	-0,93932	0,025636	-0,05244	protein_coding	DHDDS	dehydrodolichyl diphosphate synthase subunit [Source:HGNC Symbol;Acc:HGNC:20603]	1
ENSG00000117676	357,1497	-0,01807	0,069552	-1,81482	0,032766	-0,12709	protein_coding	RPS6KA1	ribosomal protein S6 kinase A1 [Source:HGNC Symbol;Acc:HGNC:10430]	1
ENSG00000260063	15,42353	-0,00215	0,397591	-0,84593	0,026093	-0,22765	lncRNA	AL512408.	novel transcript, antisense to ARID1A	1
ENSG00000060642	117,93	-0,01108	0,087372	-1,70943	0,02872	-0,18327	protein_coding	PIGV	phosphatidylinositol glycan anchor biosynthesis class V [Source:HGNC Symbol;Acc:HGNC:26031]	1
ENSG00000204160	420,0653	0,000274	0,988758	0,01409	0,02424	0,000925	protein_coding	ZDHHC18	zinc finger DHHC-type palmitoyltransferase 18 [Source:HGNC Symbol;Acc:HGNC:20712]	1
ENSG00000175793	513,3287	-0,00482	0,284891	-1,0694	0,026327	-0,15692	protein_coding	SFN	stratifin [Source:HGNC Symbol;Acc:HGNC:10773]	1
ENSG00000142751	343,9923	-0,08356	0,00878	-2,62049	0,163347	-0,18573	protein_coding	GPN2	GPN-loop GTPase 2 [Source:HGNC Symbol;Acc:HGNC:25513]	1
ENSG00000198746	243,0106	-0,00112	0,882255	-0,14811	0,024917	-0,0123	protein_coding	GPATCH3	G-patch domain containing 3 [Source:HGNC Symbol;Acc:HGNC:25720]	1
ENSG00000131910	48,32263	-0,00034	0,855133	-0,18257	0,026018	-0,06513	protein_coding	NR0B2	nuclear receptor subfamily 0 group B member 2 [Source:HGNC Symbol;Acc:HGNC:7961]	1
ENSG00000175707	109,4932	-0,00695	0,078674	-1,75843	0,027164	-0,30874	protein_coding	KDF1	keratinocyte differentiation factor 1 [Source:HGNC Symbol;Acc:HGNC:26624]	1
ENSG00000253368	404,6747	0,001799	0,687705	0,401971	0,025609	0,049552	protein_coding	TRNP1	TMF1 regulated nuclear protein 1 [Source:HGNC Symbol;Acc:HGNC:34348]	1
ENSG00000158246	59,58722	-0,00066	0,768513	-0,29432	0,025996	-0,08606	protein_coding	TENT5B	terminal nucleotidyltransferase 5B [Source:HGNC Symbol;Acc:HGNC:28273]	1
ENSG00000090020	1365,198	0,01912	0,115581	1,573594	0,031704	0,087042	protein_coding	SLC9A1	solute carrier family 9 member A1 [Source:HGNC Symbol;Acc:HGNC:11071]	1
ENSG00000142784	816,5517	-0,01651	0,192462	-1,30333	0,028476	-0,06383	protein_coding	WDTC1	WD and tetratricopeptide repeats 1 [Source:HGNC Symbol;Acc:HGNC:29175]	1
ENSG00000186501	306,5594	-0,00039	0,982072	0,022472	0,024656	0,001694	protein_coding	TMEM222	transmembrane protein 222 [Source:HGNC Symbol;Acc:HGNC:25363]	1
ENSG00000241547	12,09659	-0,00062	0,690153	-0,39865	0,026045	-0,1672	processed_pseudo	ACTG1P20	actin gamma 1 pseudogene 20 [Source:HGNC Symbol;Acc:HGNC:51500]	1
ENSG00000142765	121,3303	-0,0066	0,243758	-1,16565	0,026645	-0,13691	protein_coding	SYTL1	synaptotagmin like 1 [Source:HGNC Symbol;Acc:HGNC:15584]	1
ENSG00000142733	389,0134	-0,00205	0,810397	-0,23991	0,024524	-0,01671	protein_coding	MAP3K6	mitogen-activated protein kinase kinase kinase 6 [Source:HGNC Symbol;Acc:HGNC:6858]	1
ENSG00000181773	45,14516	0,001516	0,657022	0,444029	0,025945	0,092004	protein_coding	GPR3	G protein-coupled receptor 3 [Source:HGNC Symbol;Acc:HGNC:4484]	1
ENSG00000126705	744,6919	-0,1009	0,005393	-2,78254	0,075013	-0,15778	protein_coding	AHDC1	AT-hook DNA binding motif containing 1 [Source:HGNC Symbol;Acc:HGNC:25230]	1
ENSG00000000938	40,96265	0,005164	0,086454	1,714406	0,026669	0,38615	protein_coding	FGR	FGR proto-oncogene, Src family tyrosine kinase [Source:HGNC Symbol;Acc:HGNC:3697]	1
ENSG00000126709	448,7473	0,000238	0,857355	0,179742	0,026048	0,084175	protein_coding	IFI6	interferon alpha inducible protein 6 [Source:HGNC Symbol;Acc:HGNC:4054]	1
ENSG00000009780	217,2283	-0,00509	0,499642	-0,67505	0,025585	-0,05723	protein_coding	FAM76A	family with sequence similarity 76 member A [Source:HGNC Symbol;Acc:HGNC:28530]	1

ENSG00000117758	837,5049	-0,00536	0,66069	-0,43896	0,024226	-0,0244	protein_coding	STX12	syntaxin 12 [Source:HGNC Symbol;Acc:HGNC:11430]	1
ENSG00000117751	652,5258	0,014964	0,194737	1,296693	0,028516	0,072045	protein_coding	PPP1R8	protein phosphatase 1 regulatory subunit 8 [Source:HGNC Symbol;Acc:HGNC:9296]	1
ENSG00000252947	220,2468	0,000115	0,811587	-0,23838	0,025744	-0,03807	scaRNA	SCARNA1	small Cajal body-specific RNA 1 [Source:HGNC Symbol;Acc:HGNC:32555]	1
ENSG00000268552	18,22479	-0,00019	0,927612	-0,09085	0,025989	-0,02743				
ENSG00000130775	206,6418	-0,00021	0,970505	-0,03697	0,025246	-0,00371	protein_coding	THEMIS2	thymocyte selection associated family member 2 [Source:HGNC Symbol;Acc:HGNC:16839]	1
ENSG00000117748	450,4868	0,003916	0,722191	0,355532	0,024124	0,020633	protein_coding	RPA2	replication protein A2 [Source:HGNC Symbol;Acc:HGNC:10290]	1
ENSG00000130768	99,38016	-0,00586	0,192197	-1,30411	0,026657	-0,19741	protein_coding	SMPDL3B	sphingomyelin phosphodiesterase acid like 3B [Source:HGNC Symbol;Acc:HGNC:21416]	1
ENSG00000158156	147,3541	-0,00144	0,717831	-0,36136	0,025585	-0,04501	protein_coding	XKR8	XK related 8 [Source:HGNC Symbol;Acc:HGNC:25508]	1
ENSG00000158161	728,3715	-0,0038	0,729684	-0,34555	0,023433	-0,01733	protein_coding	EYA3	EYA transcriptional coactivator and phosphatase 3 [Source:HGNC Symbol;Acc:HGNC:3521]	1
ENSG00000228589	9,761787	-0,00209	0,211242	-1,25016	0,026153	-0,49777	processed_pseudog	SPCS2P4	signal peptidase complex subunit 2 pseudogene 4 [Source:HGNC Symbol;Acc:HGNC:45237]	1
ENSG00000169403	46,21939	-0,00532	0,120164	-1,55409	0,026662	-0,30796	protein_coding	PTAFR	platelet activating factor receptor [Source:HGNC Symbol;Acc:HGNC:9582]	1
ENSG00000126698	1201,661	0,033311	0,047422	1,982513	0,042193	0,090558	protein_coding	DNAJC8	DnaJ heat shock protein family (Hsp40) member C8 [Source:HGNC Symbol;Acc:HGNC:15470]	1
ENSG00000130766	439,3158	-0,00311	0,706954	-0,37595	0,024802	-0,02749	protein_coding	SESN2	sestrin 2 [Source:HGNC Symbol;Acc:HGNC:20746]	1
ENSG00000130772	123,8111	-0,00618	0,298238	-1,04022	0,026414	-0,11516	protein_coding	MED18	mediator complex subunit 18 [Source:HGNC Symbol;Acc:HGNC:25944]	1
ENSG00000204138	1024,205	-0,00192	0,821572	-0,22552	0,023956	-0,01336	protein_coding	PHACTR4	phosphatase and actin regulator 4 [Source:HGNC Symbol;Acc:HGNC:25793]	1
ENSG00000180198	543,3072	0,00095	0,939019	0,076503	0,023642	0,004261	protein_coding	RCC1	regulator of chromosome condensation 1 [Source:HGNC Symbol;Acc:HGNC:1913]	1
ENSG00000279443	17,2634	-0,00427	0,082392	-1,73697	0,026488	-0,47846	TEC	AL513497.	TEC	1
ENSG00000180098	183,561	0,007445	0,259489	1,1276	0,026748	0,115361	protein_coding	TRNAU1AP	tRNA selenocysteine 1 associated protein 1 [Source:HGNC Symbol;Acc:HGNC:30813]	1
ENSG00000197989	1393,063	0,001857	0,964338	-0,04471	0,025999	-0,00979	lncRNA	SNHG12	small nucleolar RNA host gene 12 [Source:HGNC Symbol;Acc:HGNC:30062]	1
ENSG00000221539	135,9801	0,000239	0,901459	0,123819	0,026013	0,042991	snoRNA	SNORD99	small nucleolar RNA, C/D box 99 [Source:HGNC Symbol;Acc:HGNC:32762]	1
ENSG00000120656	319,6924	0,001624	0,846232	0,193929	0,024396	0,013107	protein_coding	TAF12	TATA-box binding protein associated factor 12 [Source:HGNC Symbol;Acc:HGNC:11545]	1
ENSG00000188060	42,24637	0,001237	0,610788	0,508948	0,026012	0,142182	protein_coding	RAB42	RAB42, member RAS oncogene family [Source:HGNC Symbol;Acc:HGNC:28702]	1
ENSG00000162419	407,4679	0,017424	0,081948	1,739493	0,032244	0,123615	protein_coding	GMEB1	glucocorticoid modulatory element binding protein 1 [Source:HGNC Symbol;Acc:HGNC:4370]	1
ENSG00000198492	1467,833	0,003389	0,781195	0,277763	0,021201	0,009874	protein_coding	YTHDF2	YTH N6-methyladenosine RNA binding protein 2 [Source:HGNC Symbol;Acc:HGNC:31675]	1
ENSG00000116329	292,8927	-0,00112	0,491772	-0,68749	0,026069	-0,29537	protein_coding	OPRD1	opioid receptor delta 1 [Source:HGNC Symbol;Acc:HGNC:8153]	1
ENSG00000159023	1339,876	-0,04198	0,025005	-2,24132	0,062263	-0,12155	protein_coding	EPB41	erythrocyte membrane protein band 4.1 [Source:HGNC Symbol;Acc:HGNC:3377]	1
ENSG00000253304	63,60136	0,00037	0,933061	0,083995	0,025796	0,014501	protein_coding	TMEM200I	transmembrane protein 200B [Source:HGNC Symbol;Acc:HGNC:33785]	1
ENSG00000116353	149,1303	0,001743	0,692005	0,396135	0,02559	0,048294	protein_coding	MECR	mitochondrial trans-2-enoyl-CoA reductase [Source:HGNC Symbol;Acc:HGNC:19691]	1
ENSG00000060656	418,9895	-0,00476	0,529631	-0,62857	0,025356	-0,05014	protein_coding	PTPRU	protein tyrosine phosphatase receptor type U [Source:HGNC Symbol;Acc:HGNC:9683]	1
ENSG00000186056	19,30058	-0,00119	0,587313	-0,54273	0,026035	-0,17219	lncRNA	MATN1-AS	MATN1 antisense RNA 1 [Source:HGNC Symbol;Acc:HGNC:40364]	1
ENSG00000162511	631,6858	0,010981	0,057511	1,899409	0,02888	0,228579	protein_coding	LAPTM5	lysosomal protein transmembrane 5 [Source:HGNC Symbol;Acc:HGNC:29612]	1
ENSG00000162512	854,2609	0,002948	0,708181	0,3743	0,024996	0,029658	protein_coding	SDC3	syndecan 3 [Source:HGNC Symbol;Acc:HGNC:10660]	1
ENSG00000200181	42,17817	-0,00016	0,880153	-0,15078	0,026008	-0,0507				
ENSG00000084628	32,29785	-0,00029	0,912838	-0,10946	0,025949	-0,02768	protein_coding	NKAIN1	sodium/potassium transporting ATPase interacting 1 [Source:HGNC Symbol;Acc:HGNC:25743]	1
ENSG00000060688	528,265	0,007558	0,428825	0,791203	0,025173	0,04721	protein_coding	SNRNP40	small nuclear ribonucleoprotein U5 subunit 40 [Source:HGNC Symbol;Acc:HGNC:30857]	1
ENSG00000121766	574,1842	0,014898	0,150057	1,43933	0,029083	0,087172	protein_coding	ZCCHC17	zinc finger CCHC-type containing 17 [Source:HGNC Symbol;Acc:HGNC:30246]	1
ENSG00000121769	71,97171	0,001464	0,655964	0,445493	0,025932	0,090695	protein_coding	FABP3	fatty acid binding protein 3 [Source:HGNC Symbol;Acc:HGNC:3557]	1
ENSG00000168528	1096,575	0,016848	0,142315	1,467225	0,030462	0,087544	protein_coding	SERINC2	serine incorporator 2 [Source:HGNC Symbol;Acc:HGNC:23231]	1
ENSG00000268950	17,1085	-0,00537	0,017244	-2,38146	0,026816	-0,70938				
ENSG00000284543	52,02762	-0,00258	0,257389	-1,13258	0,026168	-0,32805	lncRNA	LINC01226	long intergenic non-protein coding RNA 1226 [Source:HGNC Symbol;Acc:HGNC:49678]	1
ENSG00000228682	10,40032	0,000393	0,91351	0,108612	0,026055	0,053528				
ENSG00000142910	239,9512	-0,00051	0,909647	-0,11348	0,02568	-0,01647	protein_coding	TINAGL1	tubulointerstitial nephritis antigen like 1 [Source:HGNC Symbol;Acc:HGNC:19168]	1
ENSG00000162517	502,2257	-0,00158	0,917452	-0,10364	0,023469	-0,00551	protein_coding	PEF1	penta-EF-hand domain containing 1 [Source:HGNC Symbol;Acc:HGNC:30009]	1
ENSG00000121753	309,9282	-0,00104	0,895768	-0,13101	0,025435	-0,01465	protein_coding	ADGRB2	adhesion G protein-coupled receptor B2 [Source:HGNC Symbol;Acc:HGNC:944]	1
ENSG00000134668	442,7812	0,317291	0,000149	3,793076	0,111471	0,380284	protein_coding	SPOCD1	SPOC domain containing 1 [Source:HGNC Symbol;Acc:HGNC:26338]	1
ENSG00000121775	134,3296	-0,00506	0,360765	-0,91391	0,026162	-0,1081	protein_coding	TMEM39B	transmembrane protein 39B [Source:HGNC Symbol;Acc:HGNC:25510]	1
ENSG00000160050	112,3095	0,000364	0,936634	0,079501	0,025638	0,011009	protein_coding	CCDC28B	coiled-coil domain containing 28B [Source:HGNC Symbol;Acc:HGNC:28163]	1
ENSG00000224066	16,56868	-0,00097	0,675679	-0,41837	0,025995	-0,11606	lncRNA	AL049795.	novel transcript	1
ENSG00000160051	77,39018	-0,01032	0,033845	-2,12191	0,028744	-0,31838	protein_coding	IQCC	IQ motif containing C [Source:HGNC Symbol;Acc:HGNC:25545]	1
ENSG00000160055	140,5354	-0,00462	0,439441	-0,77314	0,025862	-0,08027	protein_coding	TMEM234	transmembrane protein 234 [Source:HGNC Symbol;Acc:HGNC:28837]	1
ENSG00000220785	66,60925	0,000361	0,900531	0,12499	0,025924	0,028746	transcribed_unproc	MTMR9LP	myotubularin related protein 9 like, pseudogene [Source:HGNC Symbol;Acc:HGNC:27920]	1
ENSG00000183615	9,876056	0,000257	0,885162	0,144429	0,026024	0,055079	protein_coding	FAM167B	family with sequence similarity 167 member B [Source:HGNC Symbol;Acc:HGNC:28133]	1

ENSG00000182866	111,3312	-0,00176	0,486187	-0,69639	0,026046	-0,18583	protein_coding	LCK	LCK proto-oncogene, Src family tyrosine kinase [Source:HGNC Symbol;Acc:HGNC:6524]	1
ENSG00000116478	1409,232	-0,00379	0,71626	-0,36346	0,023668	-0,01912	protein_coding	HDAC1	histone deacetylase 1 [Source:HGNC Symbol;Acc:HGNC:4852]	1
ENSG00000225828	37,30963	-0,00302	0,316458	-1,00176	0,026162	-0,22124	protein_coding	FAM229A	family with sequence similarity 229 member A [Source:HGNC Symbol;Acc:HGNC:44652]	1
ENSG00000160058	1127,762	-0,01303	0,281838	-1,0762	0,026221	-0,05143	protein_coding	BSDC1	BSD domain containing 1 [Source:HGNC Symbol;Acc:HGNC:25501]	1
ENSG00000273274	23,29984	-0,00258	0,05742	-1,9001	0,026236	-0,83419	protein_coding	ZBTB8B	zinc finger and BTB domain containing 8B [Source:HGNC Symbol;Acc:HGNC:37057]	1
ENSG00000160062	96,94613	0,002015	0,681177	0,410857	0,025713	0,055099	protein_coding	ZBTB8A	zinc finger and BTB domain containing 8A [Source:HGNC Symbol;Acc:HGNC:24172]	1
ENSG00000176261	385,969	0,007387	0,394071	0,852258	0,02567	0,05839	protein_coding	ZBTB8OS	zinc finger and BTB domain containing 8 opposite strand [Source:HGNC Symbol;Acc:HGNC:24094]	1
ENSG00000162520	128,4012	0,002488	0,496071	0,680685	0,02599	0,122267	protein_coding	SYNC	syncoilin, intermediate filament protein [Source:HGNC Symbol;Acc:HGNC:28897]	1
ENSG00000162522	922,7564	-0,0086	0,376698	-0,884	0,025518	-0,05232	protein_coding	KIAA1522	KIAA1522 [Source:HGNC Symbol;Acc:HGNC:29301]	1
ENSG00000116497	290,5796	-0,00378	0,58547	-0,54541	0,025243	-0,0453	protein_coding	S100PBP	S100P binding protein [Source:HGNC Symbol;Acc:HGNC:25768]	1
ENSG00000160097	10,64332	0,000361	0,86718	0,167242	0,02603	0,065628	protein_coding	FNDC5	fibronectin type III domain containing 5 [Source:HGNC Symbol;Acc:HGNC:20240]	1
ENSG00000121900	416,4397	0,002978	0,646941	0,458016	0,025436	0,044733	protein_coding	TMEM54	transmembrane protein 54 [Source:HGNC Symbol;Acc:HGNC:24143]	1
ENSG00000116514	538,7974	-0,00978	0,352184	-0,93036	0,02588	-0,05382	protein_coding	RNF19B	ring finger protein 19B [Source:HGNC Symbol;Acc:HGNC:26886]	1
ENSG00000004455	1124,321	0,015969	0,270522	1,101861	0,026883	0,047584	protein_coding	AK2	adenylate kinase 2 [Source:HGNC Symbol;Acc:HGNC:362]	1
ENSG00000142920	60,87669	-0,00136	0,703093	-0,38115	0,02588	-0,07034	protein_coding	AZIN2	antizyme inhibitor 2 [Source:HGNC Symbol;Acc:HGNC:29957]	1
ENSG00000116525	317,7566	-0,00591	0,54968	-0,59824	0,025586	-0,0465	protein_coding	TRIM62	tripartite motif containing 62 [Source:HGNC Symbol;Acc:HGNC:25574]	1
ENSG00000160094	456,5054	-0,00735	0,381587	-0,87498	0,025863	-0,06354	protein_coding	ZNF362	zinc finger protein 362 [Source:HGNC Symbol;Acc:HGNC:18079]	1
ENSG00000121903	184,8461	0,002856	0,636536	0,472547	0,025551	0,050307	protein_coding	ZSCAN20	zinc finger and SCAN domain containing 20 [Source:HGNC Symbol;Acc:HGNC:13093]	1
ENSG00000121904	83,94784	-0,00011	0,957854	-0,05285	0,02599	-0,01589	protein_coding	CSMD2	CUB and Sushi multiple domains 2 [Source:HGNC Symbol;Acc:HGNC:19290]	1
ENSG00000163866	491,7486	-0,00095	0,918814	-0,10193	0,02357	-0,00559	protein_coding	SMIM12	small integral membrane protein 12 [Source:HGNC Symbol;Acc:HGNC:25154]	1
ENSG00000188910	27,3552	-0,00031	0,879823	-0,15119	0,025989	-0,04534	protein_coding	GJB3	gap junction protein beta 3 [Source:HGNC Symbol;Acc:HGNC:4285]	1
ENSG00000116544	70,37017	0,003045	0,458072	0,742025	0,026004	0,119203	protein_coding	DLGAP3	DLG associated protein 3 [Source:HGNC Symbol;Acc:HGNC:30368]	1
ENSG00000243749	17,74313	-1,1E-05	0,994252	-0,0072	0,026013	-0,00256	protein_coding	TMEM35B	transmembrane protein 35B [Source:HGNC Symbol;Acc:HGNC:40021]	1
ENSG00000163867	140,4128	-0,00187	0,741077	-0,33043	0,025579	-0,03955	protein_coding	ZMYM6	zinc finger MYM-type containing 6 [Source:HGNC Symbol;Acc:HGNC:13050]	1
ENSG00000197056	218,0276	0,004584	0,529339	0,629015	0,025394	0,051894	protein_coding	ZMYM1	zinc finger MYM-type containing 1 [Source:HGNC Symbol;Acc:HGNC:26253]	1
ENSG00000229994	10,49785	0,000637	0,746582	0,32315	0,026019	0,11029	processed_pseudo	RPL5P4	ribosomal protein L5 pseudogene 4 [Source:HGNC Symbol;Acc:HGNC:36095]	1
ENSG00000142687	1067,864	0,000746	0,94935	0,063523	0,02318	0,003205	protein_coding	KIAA0319L	KIAA0319 like [Source:HGNC Symbol;Acc:HGNC:30071]	1
ENSG00000020129	805,3153	-0,01043	0,354456	-0,92598	0,025799	-0,05002	protein_coding	NCDN	neurochondrin [Source:HGNC Symbol;Acc:HGNC:17597]	1
ENSG00000126067	1087,364	0,04879	0,025269	2,23726	0,049936	0,095644	protein_coding	PSMB2	proteasome 20S subunit beta 2 [Source:HGNC Symbol;Acc:HGNC:9539]	1
ENSG00000142686	429,6319	-0,00118	0,904016	-0,12059	0,024098	-0,00753	protein_coding	C1orf216	chromosome 1 open reading frame 216 [Source:HGNC Symbol;Acc:HGNC:26800]	1
ENSG00000092853	867,6413	0,004506	0,600057	0,524319	0,025435	0,044298	protein_coding	CLSPN	claspin [Source:HGNC Symbol;Acc:HGNC:19715]	1
ENSG00000134698	350,1492	-0,01197	0,189998	-1,31058	0,028099	-0,09518	protein_coding	AGO4	argonaute RISC component 4 [Source:HGNC Symbol;Acc:HGNC:18424]	1
ENSG00000126070	847,2235	-0,01018	0,261103	-1,12379	0,027078	-0,08134	protein_coding	AGO3	argonaute RISC catalytic component 3 [Source:HGNC Symbol;Acc:HGNC:18421]	1
ENSG00000116863	264,957	0,003262	0,712443	0,368577	0,024725	0,026085	protein_coding	ADPRS	ADP-ribosylserine hydrolase [Source:HGNC Symbol;Acc:HGNC:21304]	1
ENSG00000171812	30,28129	-0,00634	0,025491	-2,23387	0,027088	-0,54064	protein_coding	COL8A2	collagen type VIII alpha 2 chain [Source:HGNC Symbol;Acc:HGNC:2216]	1
ENSG00000054116	505,638	0,003939	0,144794	1,458169	0,024516	0,093466	protein_coding	TRAPPC3	trafficking protein particle complex 3 [Source:HGNC Symbol;Acc:HGNC:19942]	1
ENSG00000214193	129,3563	-0,01029	0,128446	-1,52026	0,028201	-0,1603	protein_coding	SH3D21	SH3 domain containing 21 [Source:HGNC Symbol;Acc:HGNC:26236]	1
ENSG00000142694	631,1323	0,001859	0,787595	0,269435	0,024845	0,021192	protein_coding	EVA1B	eva-1 homolog B [Source:HGNC Symbol;Acc:HGNC:25558]	1
ENSG00000196182	1396,904	-0,09406	0,001372	-3,20046	0,04279	-0,12186	protein_coding	STK40	serine/threonine kinase 40 [Source:HGNC Symbol;Acc:HGNC:21373]	1
ENSG00000181817	282,7279	-0,00039	0,960742	-0,04922	0,024671	-0,00374	protein_coding	LSM10	LSM10, U7 small nuclear RNA associated [Source:HGNC Symbol;Acc:HGNC:17562]	1
ENSG00000116885	49,75898	0,003605	0,362265	0,911058	0,02614	0,153875	protein_coding	OSCP1	organic solute carrier partner 1 [Source:HGNC Symbol;Acc:HGNC:29971]	1
ENSG00000201448	25,25728	0,001198	0,687392	0,402397	0,025965	0,094943	snoRNA	SNORA63C	small nucleolar RNA, H/ACA box 63C [Source:HGNC Symbol;Acc:HGNC:52210]	1
ENSG00000116898	787,1015	0,017242	0,129886	1,514551	0,028799	0,072928	protein_coding	MRPS15	mitochondrial ribosomal protein S15 [Source:HGNC Symbol;Acc:HGNC:14504]	1
ENSG00000163873	81,95147	-0,00182	0,367674	-0,90084	0,0261	-0,29674	protein_coding	GRIK3	glutamate ionotropic receptor kainate type subunit 3 [Source:HGNC Symbol;Acc:HGNC:4581]	1
ENSG00000233621	75,53868	-0,00074	0,849708	-0,18949	0,025725	-0,02888	lncRNA	LINC01137	long intergenic non-protein coding RNA 1137 [Source:HGNC Symbol;Acc:HGNC:49453]	1
ENSG00000163874	292,3206	-0,01075	0,155068	-1,42186	0,028071	-0,12666	protein_coding	ZC3H12A	zinc finger CCCH-type containing 12A [Source:HGNC Symbol;Acc:HGNC:26259]	1
ENSG00000163875	751,8343	-0,00073	0,949693	-0,06309	0,022965	-0,00305	protein_coding	MEAF6	MYST/Esa1 associated factor 6 [Source:HGNC Symbol;Acc:HGNC:25674]	1
ENSG00000163877	397,4451	-0,00283	0,751684	-0,31642	0,024458	-0,02097	protein_coding	SNIP1	Smad nuclear interacting protein 1 [Source:HGNC Symbol;Acc:HGNC:30587]	1
ENSG00000163879	250,5459	0,025362	0,01651	2,397443	0,044514	0,197682	protein_coding	DNALI1	dynein axonemal light intermediate chain 1 [Source:HGNC Symbol;Acc:HGNC:14353]	1
ENSG00000134697	1412,669	0,006894	0,574175	0,561914	0,023527	0,025605	protein_coding	GNL2	G protein nucleolar 2 [Source:HGNC Symbol;Acc:HGNC:29925]	1
ENSG00000116922	214,9545	-4,6E-05	0,994146	-0,00734	0,025032	-0,00065	protein_coding	C1orf109	chromosome 1 open reading frame 109 [Source:HGNC Symbol;Acc:HGNC:26039]	1
ENSG00000134690	380,4304	0,004102	0,430631	0,788112	0,026088	0,108112	protein_coding	CDCA8	cell division cycle associated 8 [Source:HGNC Symbol;Acc:HGNC:14629]	1

ENSG00000183317	65,26506	0,001066	0,795095		0,259701	0,025778	0,041824	protein_coding	EPHA10	EPH receptor A10 [Source:HGNC Symbol;Acc:HGNC:19987]	1
ENSG00000185090	476,8585	0,0005	0,935022		0,081528	0,02532	0,008588	protein_coding	MANEAL	mannosidase endo-alpha like [Source:HGNC Symbol;Acc:HGNC:26452]	1
ENSG00000196449	247,5616	-0,01136	0,190033		-1,31048	0,028137	-0,10608	protein_coding	YRDC	yrdC N6-threonylcarbamoyltransferase domain containing [Source:HGNC Symbol;Acc:HGNC:28905]	1
ENSG00000197982	449,0617	0,037831	0,027884		2,198915	0,058992	0,125789	protein_coding	C1orf122	chromosome 1 open reading frame 122 [Source:HGNC Symbol;Acc:HGNC:24789]	1
ENSG00000188786	554,2082	-0,00448	0,641018		-0,46628	0,024412	-0,02827	protein_coding	MTF1	metal regulatory transcription factor 1 [Source:HGNC Symbol;Acc:HGNC:7428]	1
ENSG00000204084	395,159	0,005148	0,597965		0,527329	0,024585	0,032234	protein_coding	INPP5B	inositol polyphosphate-5-phosphatase B [Source:HGNC Symbol;Acc:HGNC:6077]	1
ENSG00000183386	315,7523	0,00188	0,792549		0,263002	0,025327	0,026203	protein_coding	FHL3	four and a half LIM domains 3 [Source:HGNC Symbol;Acc:HGNC:3704]	1
ENSG00000183520	763,9228	0,041343	0,031268		2,153645	0,050073	0,099013	protein_coding	UTP11	UTP11 small subunit processome component [Source:HGNC Symbol;Acc:HGNC:24329]	1
ENSG00000185668	148,4364	-0,00023	0,842732		-0,1984	0,026056	-0,11146	protein_coding	POU3F1	POU class 3 homeobox 1 [Source:HGNC Symbol;Acc:HGNC:9214]	1
ENSG00000224592	10,88359	-0,00131	0,176383		-1,35198	0,026114	-0,91259	lncRNA	MIR3659H	MIR3659 host gene [Source:HGNC Symbol;Acc:HGNC:54306]	1
ENSG00000215895	13,44036	-0,00117	0,514913		-0,65121	0,026057	-0,23913	processed_pseudo	HSPA5P1	HSPA5 pseudogene 1 [Source:HGNC Symbol;Acc:HGNC:54532]	1
ENSG00000116954	284,2248	0,016349	0,073285		1,791049	0,031841	0,141397	protein_coding	RRAGC	Ras related GTP binding C [Source:HGNC Symbol;Acc:HGNC:19902]	1
ENSG00000228436	36,55646	-0,001	0,732232		-0,34216	0,025883	-0,067	lncRNA	AL139260.	novel transcript	1
ENSG00000214114	116,3675	0,005294	0,341099		0,951995	0,026198	0,110183	protein_coding	MYCBP	MYC binding protein [Source:HGNC Symbol;Acc:HGNC:7554]	1
ENSG00000158315	27,9334	-0,00091	0,714189		-0,36624	0,026008	-0,10939	protein_coding	RHBDL2	rhomboid like 2 [Source:HGNC Symbol;Acc:HGNC:16083]	1
ENSG00000174574	1207,62	-0,00173	0,87014		-0,16348	0,023267	-0,00831	protein_coding	AKIRIN1	akirin 1 [Source:HGNC Symbol;Acc:HGNC:25744]	1
ENSG00000183682	513,6841	-0,00165	0,885932		-0,14345	0,025734	-0,02037	protein_coding	BMP8A	bone morphogenetic protein 8a [Source:HGNC Symbol;Acc:HGNC:21650]	1
ENSG00000237624	16,75184	-0,00176	0,457304		-0,74329	0,026068	-0,21745	unprocessed_pseu	OXCT2P1	3-oxoacid CoA-transferase 2 pseudogene 1 [Source:HGNC Symbol;Acc:HGNC:21627]	1
ENSG00000243970	37,0807	-0,00525	0,077428		-1,76581	0,026698	-0,39938	transcribed_unpro	PPIEL	peptidylprolyl isomerase E like pseudogene [Source:HGNC Symbol;Acc:HGNC:33195]	1
ENSG00000163909	555,3686	0,00076	0,771519		0,290388	0,025968	0,075053	protein_coding	HEYL	hes related family bHLH transcription factor with YRPW motif like [Source:HGNC Symbol;Acc:HGNC:4882]	1
ENSG00000116983	28,08397	0,002028	0,424736		0,798232	0,026072	0,208831	protein_coding	HPCAL4	hippocalcin like 4 [Source:HGNC Symbol;Acc:HGNC:18212]	1
ENSG00000084072	458,1287	0,000821	0,940285		0,074912	0,024155	0,004778	protein_coding	PPIE	peptidylprolyl isomerase E [Source:HGNC Symbol;Acc:HGNC:9258]	1
ENSG00000116985	462,2408	-0,00147	0,790712		-0,26539	0,025263	-0,02589	protein_coding	BMP8B	bone morphogenetic protein 8b [Source:HGNC Symbol;Acc:HGNC:1075]	1
ENSG00000198754	18,27586	-0,00453	0,074741		-1,78205	0,026546	-0,48348	protein_coding	OXCT2	3-oxoacid CoA-transferase 2 [Source:HGNC Symbol;Acc:HGNC:18606]	1
ENSG00000043514	358,2542	-0,01868	0,058943		-1,88862	0,033881	-0,14161	protein_coding	TRIT1	tRNA isopentenyltransferase 1 [Source:HGNC Symbol;Acc:HGNC:20286]	1
ENSG00000116990	491,5295	-0,00097	0,794681		-0,26024	0,02583	-0,04577	protein_coding	MYCL	MYCL proto-oncogene, bHLH transcription factor [Source:HGNC Symbol;Acc:HGNC:7555]	1
ENSG00000168389	133,8424	-0,00548	0,371191		-0,89424	0,026155	-0,09647	protein_coding	MFSD2A	major facilitator superfamily domain containing 2A [Source:HGNC Symbol;Acc:HGNC:25897]	1
ENSG00000131238	1332,458	0,000662	0,949818		0,062936	0,021913	0,002541	protein_coding	PPT1	palmitoyl-protein thioesterase 1 [Source:HGNC Symbol;Acc:HGNC:9325]	1
ENSG00000117000	608,6182	-0,00188	0,781243		-0,2777	0,023827	-0,01596	protein_coding	RLF	RLF zinc finger [Source:HGNC Symbol;Acc:HGNC:10025]	1
ENSG00000259943	75,91397	0,000725	0,728945		0,346529	0,025701	0,051373	lncRNA	AL050341.	novel transcript, antisense to ZMPSTE24	1
ENSG00000084073	1498,804	0,004283	0,670476		0,425495	0,022815	0,018854	protein_coding	ZMPSTE24	zinc metallopeptidase STE24 [Source:HGNC Symbol;Acc:HGNC:12877]	1
ENSG00000049089	38,04536	-0,00442	0,242811		-1,16799	0,026361	-0,21008	protein_coding	COL9A2	collagen type IX alpha 2 chain [Source:HGNC Symbol;Acc:HGNC:2218]	1
ENSG00000084070	1088,907	-0,01042	0,327109		-0,97995	0,025049	-0,04705	protein_coding	SMAP2	small ArfGAP2 [Source:HGNC Symbol;Acc:HGNC:25082]	1
ENSG00000187801	189,2424	-0,00809	0,212027		-1,24801	0,02695	-0,12282	protein_coding	ZFP69B	ZFP69 zinc finger protein B [Source:HGNC Symbol;Acc:HGNC:28053]	1
ENSG00000260920	94,76994	0,00294	0,44411		0,765271	0,025706	0,090678	lncRNA	AL031985.	novel transcript, sense overlapping with ZNF643	1
ENSG00000187815	117,9516	0,006005	0,263797		1,117462	0,026525	0,140492	protein_coding	ZFP69	ZFP69 zinc finger protein [Source:HGNC Symbol;Acc:HGNC:24708]	1
ENSG00000164002	164,558	-0,00588	0,407698		-0,82795	0,025931	-0,07416	protein_coding	EXO5	exonuclease 5 [Source:HGNC Symbol;Acc:HGNC:26115]	1
ENSG00000117010	61,63748	0,001937	0,600306		0,52396	0,025792	0,07769	protein_coding	ZNF684	zinc finger protein 684 [Source:HGNC Symbol;Acc:HGNC:28418]	1
ENSG00000117016	89,04517	0,003822	0,344955		0,94442	0,026171	0,156132	protein_coding	RIMS3	regulating synaptic membrane exocytosis 3 [Source:HGNC Symbol;Acc:HGNC:21292]	1
ENSG00000272145	30,27628	0,000336	0,908064		0,115481	0,025924	0,02692	lncRNA	NFYC-AS1	NFYC antisense RNA 1 [Source:HGNC Symbol;Acc:HGNC:49451]	1
ENSG00000066136	775,5937	0,0016	0,897181		0,129224	0,023219	0,006517	protein_coding	NFYC	nuclear transcription factor Y subunit gamma [Source:HGNC Symbol;Acc:HGNC:7806]	1
ENSG00000179862	35,13681	0,00492	0,126495		1,52807	0,02657	0,321716	protein_coding	CITED4	Cbp/p300 interacting transactivator with Glu/Asp rich carboxy-terminal domain 4 [Source:HGNC Symbol;Acc:HGNC:186	1
ENSG00000171793	925,8845	0,000791	0,936893		0,079176	0,023485	0,004263	protein_coding	CTPS1	CTP synthase 1 [Source:HGNC Symbol;Acc:HGNC:2519]	1
ENSG00000281207	27,08282	-0,00489	0,057161		-1,90208	0,026643	-0,50739	lncRNA	SLFNL1-AS	SLFNL1 antisense RNA 1 [Source:HGNC Symbol;Acc:HGNC:44126]	1
ENSG00000010803	523,3269	-0,01579	0,18388		-1,3289	0,028776	-0,07113	protein_coding	SCMH1	Scm polycomb group protein homolog 1 [Source:HGNC Symbol;Acc:HGNC:19003]	1
ENSG00000204060	60,69657	0,000351	0,926662		0,092046	0,025801	0,016158	protein_coding	FOXO6	forkhead box O6 [Source:HGNC Symbol;Acc:HGNC:24814]	1
ENSG00000127124	751,5013	0,007439	0,415422		0,814389	0,025519	0,053309	protein_coding	HIVEP3	HIVEP zinc finger 3 [Source:HGNC Symbol;Acc:HGNC:13561]	1
ENSG00000177181	944,0458	0,000851	0,897805		0,128435	0,025188	0,012338	protein_coding	RIMKLA	ribosomal modification protein rimK like family member A [Source:HGNC Symbol;Acc:HGNC:28725]	1
ENSG00000127125	687,1557	0,000431	0,969248		0,038551	0,022807	0,00181	protein_coding	PPCS	phosphopantothenoylcysteine synthetase [Source:HGNC Symbol;Acc:HGNC:25686]	1
ENSG00000186409	64,47038	-0,00032	0,913326		-0,10884	0,025834	-0,02034	protein_coding	CCDC30	coiled-coil domain containing 30 [Source:HGNC Symbol;Acc:HGNC:26103]	1
ENSG00000171960	168,3641	0,007521	0,290415		1,057212	0,02656	0,09705	protein_coding	PPIH	peptidylprolyl isomerase H [Source:HGNC Symbol;Acc:HGNC:14651]	1
ENSG00000285728	9,944864	-0,00021	0,971073		-0,03626	0,026034	-0,01534	lncRNA	AC098484.	novel transcript, antisense to PPIH	1

ENSG00000164008	66,17979	-0,00077	0,993085	-0,00867	0,025634	-0,00118	protein_coding	C1orf50	chromosome 1 open reading frame 50 [Source:HGNC Symbol;Acc:HGNC:28795]	1
ENSG00000177868	150,4979	0,003906	0,722892	0,354596	0,02559	0,034504	protein_coding	SVBP	small vasohibin binding protein [Source:HGNC Symbol;Acc:HGNC:29204]	1
ENSG00000164010	177,8442	-0,00728	0,265856	-1,11266	0,02655	-0,10618	protein_coding	ERMAP	erythroblast membrane associated protein (Scianna blood group) [Source:HGNC Symbol;Acc:HGNC:15743]	1
ENSG00000228192	15,51225	-0,00056	0,82377	-0,2227	0,02598	-0,06232	lncRNA	AL512353.	novel transcript, antisense to ERMAP	1
ENSG00000164011	70,20418	0,005206	0,302189	1,031751	0,026286	0,130816	protein_coding	ZNF691	zinc finger protein 691 [Source:HGNC Symbol;Acc:HGNC:28028]	1
ENSG00000117394	1121,367	-0,00283	0,730778	-0,34409	0,02452	-0,02321	protein_coding	SLC2A1	solute carrier family 2 member 1 [Source:HGNC Symbol;Acc:HGNC:11005]	1
ENSG00000227533	10,98281	-0,0013	0,428645	-0,79151	0,026077	-0,3154	lncRNA	SLC2A1-AS	SLC2A1 antisense RNA 1 [Source:HGNC Symbol;Acc:HGNC:44187]	1
ENSG00000117395	1162,886	0,117718	0,000287	3,626808	0,042527	0,141084	protein_coding	EBNA1BP2	EBNA1 binding protein 2 [Source:HGNC Symbol;Acc:HGNC:15531]	1
ENSG00000243710	9,680123	-0,0014	0,301541	-1,03313	0,026103	-0,50877	protein_coding	CFAP57	cilia and flagella associated protein 57 [Source:HGNC Symbol;Acc:HGNC:26485]	1
ENSG00000179178	61,17231	-0,00333	0,241027	-1,17242	0,026237	-0,27065	protein_coding	TMEM125	transmembrane protein 125 [Source:HGNC Symbol;Acc:HGNC:28275]	1
ENSG00000253313	77,65443	0,00037	0,918682	0,102094	0,025801	0,017875	protein_coding	C1orf210	chromosome 1 open reading frame 210 [Source:HGNC Symbol;Acc:HGNC:28755]	1
ENSG00000117400	12,4863	-0,00079	0,651091	-0,45225	0,02604	-0,17299	protein_coding	MPL	MPL proto-oncogene, thrombopoietin receptor [Source:HGNC Symbol;Acc:HGNC:7217]	1
ENSG00000234694	9,352332	-0,00041	0,868346	-0,16576	0,026031	-0,06587	lncRNA	AL139289.	novel transcript	1
ENSG00000117399	403,4027	0,000506	0,921508	0,098534	0,02557	0,012668	protein_coding	CDC20	cell division cycle 20 [Source:HGNC Symbol;Acc:HGNC:1723]	1
ENSG00000066322	778,8417	0,034695	0,020094	2,324589	0,059117	0,150508	protein_coding	ELOVL1	ELOVL fatty acid elongase 1 [Source:HGNC Symbol;Acc:HGNC:14418]	1
ENSG00000159479	555,6446	0,00393	0,658492	0,441997	0,023789	0,023747	protein_coding	MED8	mediator complex subunit 8 [Source:HGNC Symbol;Acc:HGNC:19971]	1
ENSG00000198198	1088,985	-0,02839	0,062897	-1,85992	0,0395	-0,09247	protein_coding	SZT2	SZT2 subunit of KICSTOR complex [Source:HGNC Symbol;Acc:HGNC:29040]	1
ENSG00000178922	314,7771	0,009334	0,336637	0,960831	0,026774	0,071763	protein_coding	HYI	hydroxypyruvate isomerase (putative) [Source:HGNC Symbol;Acc:HGNC:26948]	1
ENSG00000142949	1505,8	0,012915	0,237801	1,1805	0,027017	0,063409	protein_coding	PTPRF	protein tyrosine phosphatase receptor type F [Source:HGNC Symbol;Acc:HGNC:9670]	1
ENSG00000066135	431,4673	-0,01765	0,101767	-1,63635	0,031971	-0,10838	protein_coding	KDM4A	lysine demethylase 4A [Source:HGNC Symbol;Acc:HGNC:22978]	1
ENSG00000236200	11,29391	0,001266	0,450393	0,754761	0,026074	0,302238	lncRNA	KDM4A-AS	KDM4A antisense RNA 1 [Source:HGNC Symbol;Acc:HGNC:40528]	1
ENSG00000126091	48,73502	-0,00034	0,915377	-0,10626	0,025885	-0,0222	protein_coding	ST3GAL3	ST3 beta-galactoside alpha-2,3-sialyltransferase 3 [Source:HGNC Symbol;Acc:HGNC:10866]	1
ENSG00000117407	20,06195	-0,00044	0,810201	-0,24017	0,026023	-0,08863	protein_coding	ARTN	artemin [Source:HGNC Symbol;Acc:HGNC:727]	1
ENSG00000117408	662,0147	0,002617	0,757675	0,308535	0,024671	0,022065	protein_coding	IPO13	importin 13 [Source:HGNC Symbol;Acc:HGNC:16853]	1
ENSG00000132768	320,0088	-0,0009	0,914925	-0,10683	0,024503	-0,00758	protein_coding	DPH2	diphthamide biosynthesis 2 [Source:HGNC Symbol;Acc:HGNC:3004]	1
ENSG00000117410	791,4418	0,002056	0,837929	0,204543	0,023663	0,011259	protein_coding	ATP6V0B	ATPase H+ transporting V0 subunit b [Source:HGNC Symbol;Acc:HGNC:861]	1
ENSG00000117411	499,4117	0,003511	0,605217	0,516913	0,024575	0,034448	protein_coding	B4GALT2	beta-1,4-galactosyltransferase 2 [Source:HGNC Symbol;Acc:HGNC:925]	1
ENSG00000159214	90,37494	-0,00357	0,377312	-0,88286	0,026102	-0,14177	protein_coding	CCDC24	coiled-coil domain containing 24 [Source:HGNC Symbol;Acc:HGNC:28688]	1
ENSG00000196517	192,1783	-0,01923	0,008718	-2,62291	0,036622	-0,28652	protein_coding	SLC6A9	solute carrier family 6 member 9 [Source:HGNC Symbol;Acc:HGNC:11056]	1
ENSG00000230615	16,13817	0,003059	0,205581	1,265809	0,026253	0,380569	lncRNA	AL139220.:	novel transcript	1
ENSG00000178028	406,6637	-0,01408	0,193106	-1,30145	0,027977	-0,07267	protein_coding	DMAP1	DNA methyltransferase 1 associated protein 1 [Source:HGNC Symbol;Acc:HGNC:18291]	1
ENSG00000117419	470,4538	0,015985	0,20125	1,277998	0,028853	0,067925	protein_coding	ERI3	ERI1 exoribonuclease family member 3 [Source:HGNC Symbol;Acc:HGNC:17276]	1
ENSG00000187147	703,2565	-0,00562	0,619704	-0,49627	0,024358	-0,02799	protein_coding	RNF220	ring finger protein 220 [Source:HGNC Symbol;Acc:HGNC:25552]	1
ENSG00000126106	118,1254	-0,00275	0,494123	-0,68377	0,025664	-0,08023	protein_coding	TMEM53	transmembrane protein 53 [Source:HGNC Symbol;Acc:HGNC:26186]	1
ENSG00000199377	27,47112	0,000214	0,909054	0,114231	0,026016	0,040761	snRNA	RNU5F-1	RNA, U5F small nuclear 1 [Source:HGNC Symbol;Acc:HGNC:10216]	1
ENSG00000200169	53,07351	0,00151	0,592868	0,534685	0,025991	0,126744	snRNA	RNU5D-1	RNA, U5D small nuclear 1 [Source:HGNC Symbol;Acc:HGNC:10214]	1
ENSG00000142945	505,1968	0,001305	0,777172	0,283006	0,025703	0,040018	protein_coding	KIF2C	kinesin family member 2C [Source:HGNC Symbol;Acc:HGNC:6393]	1
ENSG00000225721	17,91346	-0,00141	0,500719	-0,67336	0,026054	-0,21795	lncRNA	AL592166.	novel transcript	1
ENSG00000200913	256,9544	-0,00129	0,60906	-0,51142	0,026003	-0,13359	snoRNA	SNORD46	small nucleolar RNA, C/D box 46 [Source:HGNC Symbol;Acc:HGNC:10186]	1
ENSG00000173846	747,5535	-0,00908	0,295818	-1,04544	0,0269	-0,08403	protein_coding	PLK3	polo like kinase 3 [Source:HGNC Symbol;Acc:HGNC:2154]	1
ENSG00000222009	270,7841	-0,01841	0,04291	-2,02458	0,034185	-0,16661	protein_coding	BTBD19	BTB domain containing 19 [Source:HGNC Symbol;Acc:HGNC:27145]	1
ENSG00000117425	22,58398	-0,00095	0,650693	-0,4528	0,025944	-0,10474	protein_coding	PTCH2	patched 2 [Source:HGNC Symbol;Acc:HGNC:9586]	1
ENSG00000070785	424,5408	0,01062	0,315068	1,004645	0,026151	0,056838	protein_coding	EIF2B3	eukaryotic translation initiation factor 2B subunit gamma [Source:HGNC Symbol;Acc:HGNC:3259]	1
ENSG00000126107	1049,442	-0,00041	0,989661	-0,01296	0,022897	-0,00062	protein_coding	HECTD3	HECT domain E3 ubiquitin protein ligase 3 [Source:HGNC Symbol;Acc:HGNC:26117]	1
ENSG00000126088	741,7451	0,000525	0,752729	0,315043	0,023238	0,016103	protein_coding	UROD	uroporphyrinogen decarboxylase [Source:HGNC Symbol;Acc:HGNC:12591]	1
ENSG00000162415	243,6214	-0,00635	0,385711	-0,86742	0,0261	-0,07834	protein_coding	ZSWIM5	zinc finger SWIM-type containing 5 [Source:HGNC Symbol;Acc:HGNC:29299]	1
ENSG00000186603	22,24059	0,000702	0,738225	0,334205	0,026013	0,109394	protein_coding	HPDL	4-hydroxyphenylpyruvate dioxygenase like [Source:HGNC Symbol;Acc:HGNC:28242]	1
ENSG00000132781	20,08021	-0,0032	0,201609	-1,27698	0,02628	-0,38274	protein_coding	MUTYH	mutY DNA glycosylase [Source:HGNC Symbol;Acc:HGNC:7527]	1
ENSG00000132773	183,0539	0,006752	0,276208	1,088878	0,026552	0,115738	protein_coding	TOE1	target of EGR1, exonuclease [Source:HGNC Symbol;Acc:HGNC:15954]	1
ENSG00000070759	53,94146	-0,00266	0,542416	-0,60916	0,025873	-0,0892	protein_coding	TESK2	testis associated actin remodelling kinase 2 [Source:HGNC Symbol;Acc:HGNC:11732]	1
ENSG00000280670	26,2085	0,002722	0,276415	1,088408	0,026172	0,292932	protein_coding	CCDC163	coiled-coil domain containing 163 [Source:HGNC Symbol;Acc:HGNC:27003]	1
ENSG00000132763	127,2599	-0,00292	0,625749	-0,48772	0,025546	-0,05142	protein_coding	MMACHC	metabolism of cobalamin associated C [Source:HGNC Symbol;Acc:HGNC:24525]	1

ENSG00000117448	1039,549	-0,0006	0,9497	-0,06308	0,023875	-0,00373	protein_coding	AKR1A1	aldo-keto reductase family 1 member A1 [Source:HGNC Symbol;Acc:HGNC:380]	1
ENSG00000132780	1458,513	0,080249	0,00807	2,649117	0,063518	0,130015	protein_coding	NASP	nuclear autoantigenic sperm protein [Source:HGNC Symbol;Acc:HGNC:7644]	1
ENSG00000159588	15,92196	-0,00173	0,334625	-0,96484	0,026106	-0,36015	protein_coding	CCDC17	coiled-coil domain containing 17 [Source:HGNC Symbol;Acc:HGNC:26574]	1
ENSG00000234329	10,93763	-0,00081	0,56611	-0,57379	0,026061	-0,2634	processed_pseudog	AL604028.1	ribosomal protein L7a (RPL7A) pseudogene	1
ENSG00000159596	213,7899	0,001515	0,838841	0,203376	0,024918	0,016712	protein_coding	TMEM69	transmembrane protein 69 [Source:HGNC Symbol;Acc:HGNC:28035]	1
ENSG00000197429	176,2581	0,008452	0,25036	1,149475	0,026871	0,10182	protein_coding	IPP	intracisternal A particle-promoted polypeptide [Source:HGNC Symbol;Acc:HGNC:6108]	1
ENSG00000117461	253,3674	-0,00683	0,331462	-0,97117	0,026308	-0,08964	protein_coding	PIK3R3	phosphoinositide-3-kinase regulatory subunit 3 [Source:HGNC Symbol;Acc:HGNC:8981]	1
ENSG00000117472	193,8253	-0,00327	0,263735	-1,11761	0,026219	-0,25671	protein_coding	TSPAN1	tetraspanin 1 [Source:HGNC Symbol;Acc:HGNC:20657]	1
ENSG00000085998	1162,889	0,003573	0,750896	0,317458	0,022933	0,014608	protein_coding	POMGNT1	protein O-linked mannose N-acetylglucosaminyltransferase 1 (beta 1,2-) [Source:HGNC Symbol;Acc:HGNC:19139]	1
ENSG00000171357	21,38553	0,001244	0,642912	0,463631	0,025987	0,116473	protein_coding	LURAP1	leucine rich adaptor protein 1 [Source:HGNC Symbol;Acc:HGNC:32327]	1
ENSG00000085999	88,9542	-0,00093	0,798253	-0,25561	0,025841	-0,04623	protein_coding	RAD54L	RAD54 like [Source:HGNC Symbol;Acc:HGNC:9826]	1
ENSG00000132128	997,3506	-0,00189	0,8481	-0,19154	0,023501	-0,01021	protein_coding	LRRC41	leucine rich repeat containing 41 [Source:HGNC Symbol;Acc:HGNC:16917]	1
ENSG00000173660	974,6035	0,03175	0,025142	2,239213	0,051532	0,141328	protein_coding	UQCRH	ubiquinol-cytochrome c reductase hinge protein [Source:HGNC Symbol;Acc:HGNC:12590]	1
ENSG00000117481	310,8473	-0,00999	0,255607	-1,13684	0,02715	-0,08616	protein_coding	NSUN4	NOP2/Sun RNA methyltransferase 4 [Source:HGNC Symbol;Acc:HGNC:31802]	1
ENSG00000117480	181,1234	-0,01218	0,071478	-1,80243	0,029372	-0,18647	protein_coding	FAAH	fatty acid amide hydrolase [Source:HGNC Symbol;Acc:HGNC:3553]	1
ENSG00000079277	322,6488	-0,01643	0,102825	-1,63131	0,031457	-0,11835	protein_coding	MKNK1	MAPK interacting serine/threonine kinase 1 [Source:HGNC Symbol;Acc:HGNC:7110]	1
ENSG00000142961	193,9306	-0,00179	0,669465	-0,42688	0,025064	-0,0371	protein_coding	MOB3C	MOB kinase activator 3C [Source:HGNC Symbol;Acc:HGNC:29800]	1
ENSG00000123472	597,7097	-0,00345	0,76649	-0,29697	0,023085	-0,01408	protein_coding	ATPAF1	ATP synthase mitochondrial F1 complex assembly factor 1 [Source:HGNC Symbol;Acc:HGNC:18803]	1
ENSG00000187048	18,82038	0,000271	0,596614	0,529276	0,026068	0,324999	protein_coding	CYP4A11	cytochrome P450 family 4 subfamily A member 11 [Source:HGNC Symbol;Acc:HGNC:2642]	1
ENSG00000162366	9,622275	0,00085	0,35114	0,93238	0,026087	0,627669	protein_coding	PDZK1IP1	PDZK1 interacting protein 1 [Source:HGNC Symbol;Acc:HGNC:16887]	1
ENSG00000123473	378,2762	-0,00374	0,803781	-0,24846	0,025776	-0,02779	protein_coding	STIL	STIL centriolar assembly protein [Source:HGNC Symbol;Acc:HGNC:10879]	1
ENSG00000237424	31,8971	0,002581	0,301271	1,033712	0,026135	0,262877	lncRNA	FOXD2-AS1	FOXD2 adjacent opposite strand RNA 1 [Source:HGNC Symbol;Acc:HGNC:44256]	1
ENSG00000186564	15,64035	-0,0043	0,052623	-1,938	0,026521	-0,60514	protein_coding	FOXD2	forkhead box D2 [Source:HGNC Symbol;Acc:HGNC:3803]	1
ENSG00000269113	46,45498	-0,00086	0,721643	-0,35626	0,025988	-0,09769	protein_coding	TRABD2B	TraB domain containing 2B [Source:HGNC Symbol;Acc:HGNC:44200]	1
ENSG00000117834	24,31249	0,001419	0,197112	1,289824	0,026119	0,79023	protein_coding	SLC5A9	solute carrier family 5 member 9 [Source:HGNC Symbol;Acc:HGNC:22146]	1
ENSG00000132122	123,8393	0,002676	0,64476	0,461053	0,025581	0,051077	protein_coding	SPATA6	spermatogenesis associated 6 [Source:HGNC Symbol;Acc:HGNC:18309]	1
ENSG00000162373	11,75007	-0,00093	0,56752	-0,57171	0,026054	-0,23103	protein_coding	BEND5	BEN domain containing 5 [Source:HGNC Symbol;Acc:HGNC:25668]	1
ENSG00000162374	35,28278	0,002214	0,647443	0,457318	0,026042	0,095079	protein_coding	ELAVL4	ELAV like RNA binding protein 4 [Source:HGNC Symbol;Acc:HGNC:3315]	1
ENSG00000185104	897,5489	-0,00367	0,977417	-0,02831	0,02347	-0,00144	protein_coding	FAF1	Fas associated factor 1 [Source:HGNC Symbol;Acc:HGNC:3578]	1
ENSG00000123080	520,4888	0,006707	0,345969	0,942437	0,026225	0,085027	protein_coding	CDKN2C	cyclin dependent kinase inhibitor 2C [Source:HGNC Symbol;Acc:HGNC:1789]	1
ENSG00000085831	517,6447	-0,01787	0,038127	-2,07349	0,033977	-0,18333	protein_coding	TTC39A	tetratricopeptide repeat domain 39A [Source:HGNC Symbol;Acc:HGNC:18657]	1
ENSG00000261664	11,77647	0,001702	0,361647	0,912231	0,026102	0,340435	lncRNA	TTC39A-AS	TTC39A antisense RNA 1 [Source:HGNC Symbol;Acc:HGNC:40851]	1
ENSG00000085832	1558,172	-0,02798	0,087549	-1,70847	0,034236	-0,06967	protein_coding	EPS15	epidermal growth factor receptor pathway substrate 15 [Source:HGNC Symbol;Acc:HGNC:3419]	1
ENSG00000266993	13,05549	-0,00014	0,96356	-0,04569	0,025998	-0,01466	lncRNA	AL050343.1	novel transcript, antisense to NRD1	1
ENSG00000117862	269,0958	0,004043	0,584895	0,546249	0,02522	0,044021	protein_coding	TXNDC12	thioredoxin domain containing 12 [Source:HGNC Symbol;Acc:HGNC:24626]	1
ENSG00000198841	62,37658	-0,00369	0,340936	-0,95232	0,026168	-0,16317	protein_coding	KTI12	KTI12 chromatin associated homolog [Source:HGNC Symbol;Acc:HGNC:25160]	1
ENSG00000134717	530,8541	0,027176	0,063669	1,854494	0,041097	0,105399	protein_coding	BTF3L4	basic transcription factor 3 like 4 [Source:HGNC Symbol;Acc:HGNC:30547]	1
ENSG00000157077	776,7581	-0,01463	0,214678	-1,2408	0,027123	-0,05956	protein_coding	ZFYVE9	zinc finger FYVE-type containing 9 [Source:HGNC Symbol;Acc:HGNC:6775]	1
ENSG00000154222	819,5149	-0,0089	0,439685	-0,77272	0,024321	-0,03628	protein_coding	CC2D1B	coiled-coil and C2 domain containing 1B [Source:HGNC Symbol;Acc:HGNC:29386]	1
ENSG00000085840	169,2995	0,003449	0,537508	0,616586	0,025907	0,080978	protein_coding	ORC1	origin recognition complex subunit 1 [Source:HGNC Symbol;Acc:HGNC:8487]	1
ENSG00000134748	654,335	0,016548	0,116317	1,570424	0,030808	0,101375	protein_coding	PRPF38A	pre-mRNA processing factor 38A [Source:HGNC Symbol;Acc:HGNC:25930]	1
ENSG00000134744	984,5366	-0,01147	0,291232	-1,05542	0,026216	-0,0564	protein_coding	TUT4	terminal uridylyl transferase 4 [Source:HGNC Symbol;Acc:HGNC:28981]	1
ENSG00000116157	283,4787	0,004329	0,525345	0,635127	0,025609	0,059372	protein_coding	GPX7	glutathione peroxidase 7 [Source:HGNC Symbol;Acc:HGNC:4559]	1
ENSG00000162377	406,3066	0,015459	0,125908	1,530438	0,030002	0,10017	protein_coding	COA7	cytochrome c oxidase assembly factor 7 (putative) [Source:HGNC Symbol;Acc:HGNC:25716]	1
ENSG00000162378	1130,567	0,002236	0,826101	0,219704	0,024252	0,014003	protein_coding	ZYG11B	zyg-11 family member B, cell cycle regulator [Source:HGNC Symbol;Acc:HGNC:25820]	1
ENSG00000228929	11,49709	0,00273	0,171344	1,367899	0,026219	0,467042	processed_pseudog	RPS13P2	ribosomal protein S13 pseudogene 2 [Source:HGNC Symbol;Acc:HGNC:36364]	1
ENSG00000203995	16,22651	0,000572	0,659936	0,440002	0,026066	0,243853	protein_coding	ZYG11A	zyg-11 family member A, cell cycle regulator [Source:HGNC Symbol;Acc:HGNC:32058]	1
ENSG00000121310	247,2839	-0,00683	0,304854	-1,02608	0,026403	-0,09895	protein_coding	ECHDC2	enoyl-CoA hydratase domain containing 2 [Source:HGNC Symbol;Acc:HGNC:23408]	1
ENSG00000116171	1482,518	0,004174	0,720095	0,358332	0,022622	0,015434	protein_coding	SCP2	sterol carrier protein 2 [Source:HGNC Symbol;Acc:HGNC:10606]	1
ENSG00000174348	152,9657	-0,00108	0,753886	-0,31352	0,025882	-0,06131	protein_coding	PODN	podocan [Source:HGNC Symbol;Acc:HGNC:23174]	1
ENSG00000162383	41,4036	9,12E-05	0,960064	0,050073	0,026022	0,018903	protein_coding	SLC1A7	solute carrier family 1 member 7 [Source:HGNC Symbol;Acc:HGNC:10945]	1
ENSG00000157184	361,8391	-0,00108	0,992306	0,009643	0,025005	0,000833	protein_coding	CPT2	carnitine palmitoyltransferase 2 [Source:HGNC Symbol;Acc:HGNC:2330]	1

ENSG00000236723	9,754757	0,001845	0,340587		0,953007	0,026107	0,332098	lncRNA	AL606760.	novel transcript, antisense to CPT2	1
ENSG00000162384	265,1362	0,005558	0,524852		0,635884	0,025475	0,049193	protein_coding	CZIB	CXXC motif containing zinc binding protein [Source:HGNC Symbol;Acc:HGNC:26059]	1
ENSG00000162385	350,2463	0,009122	0,333838		0,966413	0,026258	0,064481	protein_coding	MAGOH	mago homolog, exon junction complex subunit [Source:HGNC Symbol;Acc:HGNC:6815]	1
ENSG00000226754	14,42362	-0,00318	0,192205		-1,30408	0,026256	-0,36294	lncRNA	AL606760.	novel transcript, antisense to MAGOH	1
ENSG00000157193	833,8111	-0,05597	0,01444		-2,44612	0,088697	-0,14052	protein_coding	LRP8	LDL receptor related protein 8 [Source:HGNC Symbol;Acc:HGNC:6700]	1
ENSG00000058804	444,5275	0,015677	0,108909		1,603118	0,030643	0,113244	protein_coding	NDC1	NDC1 transmembrane nucleoporin [Source:HGNC Symbol;Acc:HGNC:25525]	1
ENSG00000058799	306,1397	0,009039	0,322121		0,990109	0,026318	0,067732	protein_coding	YIPF1	Yip1 domain family member 1 [Source:HGNC Symbol;Acc:HGNC:25231]	1
ENSG00000211452	90,70148	-0,00053	0,483848		-0,70013	0,026079	-0,56954	protein_coding	DIO1	iodothyronine deiodinase 1 [Source:HGNC Symbol;Acc:HGNC:2883]	1
ENSG00000081870	267,7493	0,003278	0,696939		0,389456	0,024778	0,028027	protein_coding	HSPB11	heat shock protein family B (small) member 11 [Source:HGNC Symbol;Acc:HGNC:25019]	1
ENSG00000116212	473,8765	-0,01073	0,253255		-1,14248	0,027076	-0,07701	protein_coding	LRRC42	leucine rich repeat containing 42 [Source:HGNC Symbol;Acc:HGNC:28792]	1
ENSG00000203985	114,3676	0,000649	0,770005		0,292368	0,026003	0,092232	protein_coding	LDLRAD1	low density lipoprotein receptor class A domain containing 1 [Source:HGNC Symbol;Acc:HGNC:32069]	1
ENSG00000116205	200,0487	0,000937	0,914698		0,107114	0,024923	0,008968	protein_coding	TCEANC2	transcription elongation factor A N-terminal and central domain containing 2 [Source:HGNC Symbol;Acc:HGNC:26494]	1
ENSG00000215883	53,36658	-0,00637	0,135268		-1,49365	0,026918	-0,25021	protein_coding	CYB5RL	cytochrome b5 reductase like [Source:HGNC Symbol;Acc:HGNC:32220]	1
ENSG00000116221	883,0035	0,007347	0,599753		0,524756	0,02427	0,026518	protein_coding	MRPL37	mitochondrial ribosomal protein L37 [Source:HGNC Symbol;Acc:HGNC:14034]	1
ENSG00000157216	1021,221	-0,00397	0,877055		-0,1547	0,022707	-0,00679	protein_coding	SSBP3	single stranded DNA binding protein 3 [Source:HGNC Symbol;Acc:HGNC:15674]	1
ENSG00000162390	174,4773	-0,00463	0,426334		-0,79548	0,025953	-0,08752	protein_coding	ACOT11	acyl-CoA thioesterase 11 [Source:HGNC Symbol;Acc:HGNC:18156]	1
ENSG00000243725	69,39564	-0,00084	0,860872		-0,17526	0,025636	-0,02387	protein_coding	TTC4	tetratricopeptide repeat domain 4 [Source:HGNC Symbol;Acc:HGNC:12394]	1
ENSG00000162396	71,50729	-0,00231	0,593899		-0,53319	0,02586	-0,08133	protein_coding	PARS2	prolyl-tRNA synthetase 2, mitochondrial [Source:HGNC Symbol;Acc:HGNC:30563]	1
ENSG00000006555	110,0997	-0,00381	0,366308		-0,90341	0,026147	-0,14422	protein_coding	TTC22	tetratricopeptide repeat domain 22 [Source:HGNC Symbol;Acc:HGNC:26067]	1
ENSG00000287724	11,1596	0,000333	0,845598		0,194738	0,02603	0,077433	lncRNA	AC096536.	novel transcript, antisense to DHCR24	1
ENSG00000242396	36,1227	-0,00167	0,613763		-0,50471	0,025948	-0,10266	lncRNA	AC096536.	novel transcript	1
ENSG00000143001	90,35183	-0,00334	0,324895		-0,98445	0,026146	-0,17998	protein_coding	TMEM61	transmembrane protein 61 [Source:HGNC Symbol;Acc:HGNC:27296]	1
ENSG00000162399	68,89157	-7,6E-05	0,862114		-0,17368	0,026069	-0,12466	protein_coding	BSND	barttin CLCNK type accessory subunit beta [Source:HGNC Symbol;Acc:HGNC:16512]	1
ENSG00000169174	72,63278	-0,00535	0,020729		-2,31289	0,026788	-0,62933	protein_coding	PCSK9	proprotein convertase subtilisin/kexin type 9 [Source:HGNC Symbol;Acc:HGNC:20001]	1
ENSG00000162407	259,0512	-0,00419	0,585217		-0,54578	0,025882	-0,06143	protein_coding	PLPP3	phospholipid phosphatase 3 [Source:HGNC Symbol;Acc:HGNC:9229]	1
ENSG00000162409	206,953	0,004757	0,451424		0,753043	0,025724	0,071132	protein_coding	PRKAA2	protein kinase AMP-activated catalytic subunit alpha 2 [Source:HGNC Symbol;Acc:HGNC:9377]	1
ENSG00000187889	19,40462	0,002156	0,264842		1,115019	0,026148	0,404404	protein_coding	FYB2	FYN binding protein 2 [Source:HGNC Symbol;Acc:HGNC:27295]	1
ENSG00000157131	48,79707	0,000692	0,662043		0,437094	0,026031	0,156592	protein_coding	C8A	complement C8 alpha chain [Source:HGNC Symbol;Acc:HGNC:1352]	1
ENSG00000021852	26,223	0,000353	0,854323		0,183605	0,026028	0,069048	protein_coding	C8B	complement C8 beta chain [Source:HGNC Symbol;Acc:HGNC:1353]	1
ENSG00000173406	16,59907	-0,00134	0,455712		-0,74593	0,026072	-0,28495	protein_coding	DAB1	DAB adaptor protein 1 [Source:HGNC Symbol;Acc:HGNC:2661]	1
ENSG00000162600	132,2193	0,009213	0,095864		1,665246	0,027907	0,210471	protein_coding	OMA1	OMA1 zinc metallopeptidase [Source:HGNC Symbol;Acc:HGNC:29661]	1
ENSG00000184292	287,3631	7,41E-05	0,977935		0,027658	0,025967	0,007517	protein_coding	TACSTD2	tumor associated calcium signal transducer 2 [Source:HGNC Symbol;Acc:HGNC:11530]	1
ENSG00000162601	683,8068	-0,00407	0,674534		-0,41993	0,024134	-0,02424	protein_coding	MYSM1	Myb like, SWIRM and MPN domains 1 [Source:HGNC Symbol;Acc:HGNC:29401]	1
ENSG00000234807	9,859949	-0,00038	0,775018		-0,28582	0,026049	-0,13424	lncRNA	LINC01135	long intergenic non-protein coding RNA 1135 [Source:HGNC Symbol;Acc:HGNC:49450]	1
ENSG00000232453	9,620449	-0,00123	0,435994		-0,77898	0,026075	-0,32387	lncRNA	LINC02777	long intergenic non-protein coding RNA 2777 [Source:HGNC Symbol;Acc:HGNC:54297]	1
ENSG00000224609	10,56606	0,002026	0,232983		1,192709	0,026144	0,47381	lncRNA	HSD52	uncharacterized LOC729467 [Source:NCBI gene (formerly Entrezgene);Acc:729467]	1
ENSG00000172456	90,65398	-0,00714	0,188648		-1,31459	0,026929	-0,16414	protein_coding	FGGY	FGGY carbohydrate kinase domain containing [Source:HGNC Symbol;Acc:HGNC:25610]	1
ENSG00000134709	583,8643	0,001863	0,87155		0,16169	0,024287	0,010525	protein_coding	HOOK1	hook microtubule tethering protein 1 [Source:HGNC Symbol;Acc:HGNC:19884]	1
ENSG00000134716	47,12068	0,003966	0,187757		1,317243	0,026353	0,293551	protein_coding	CYP2J2	cytochrome P450 family 2 subfamily J member 2 [Source:HGNC Symbol;Acc:HGNC:2634]	1
ENSG00000226476	85,33651	3,16E-05	0,795653		-0,25898	0,02594	-0,05988	lncRNA	LINC01748	long intergenic non-protein coding RNA 1748 [Source:HGNC Symbol;Acc:HGNC:52535]	1
ENSG00000162599	604,2024	-0,00215	0,781591		-0,27725	0,024917	-0,0222	protein_coding	NFIA	nuclear factor I A [Source:HGNC Symbol;Acc:HGNC:7784]	1
ENSG00000162604	589,2708	0,009168	0,366397		0,903242	0,025489	0,050857	protein_coding	TM2D1	TM2 domain containing 1 [Source:HGNC Symbol;Acc:HGNC:24142]	1
ENSG00000132849	999,9372	0,000801	0,911703		0,110891	0,024994	0,009597	protein_coding	PATJ	PATJ crumbs cell polarity complex component [Source:HGNC Symbol;Acc:HGNC:28881]	1
ENSG00000240563	149,5929	-0,00105	0,504726		-0,66707	0,026069	-0,28797	protein_coding	L1TD1	LINE1 type transposase domain containing 1 [Source:HGNC Symbol;Acc:HGNC:25595]	1
ENSG00000132854	38,7508	0,003023	0,070988		1,805554	0,026288	0,694457	protein_coding	KANK4	KN motif and ankyrin repeat domains 4 [Source:HGNC Symbol;Acc:HGNC:27263]	1
ENSG00000162607	1056,298	0,007001	0,424322		0,798946	0,025627	0,056119	protein_coding	USP1	ubiquitin specific peptidase 1 [Source:HGNC Symbol;Acc:HGNC:12607]	1
ENSG00000132855	17,14079	0,000574	0,695558		0,391324	0,026045	0,163612	protein_coding	ANGPTL3	angiopoietin like 3 [Source:HGNC Symbol;Acc:HGNC:491]	1
ENSG00000125703	167,1528	0,004236	0,503199		0,669464	0,025709	0,066981	protein_coding	ATG4C	autophagy related 4C cysteine peptidase [Source:HGNC Symbol;Acc:HGNC:16040]	1
ENSG00000230798	9,655725	0,000769	0,494223		0,683608	0,026076	0,388291	lncRNA	FOXD3-AS1	FOXD3 antisense RNA 1 [Source:HGNC Symbol;Acc:HGNC:40241]	1
ENSG00000187140	16,13437	0,000896	0,499378		0,675469	0,026076	0,36834	protein_coding	FOXD3	forkhead box D3 [Source:HGNC Symbol;Acc:HGNC:3804]	1
ENSG00000088035	79,60385	0,005385	0,275513		1,090453	0,026408	0,147609	protein_coding	ALG6	ALG6 alpha-1,3-glucosyltransferase [Source:HGNC Symbol;Acc:HGNC:23157]	1
ENSG00000142856	218,8671	0,008194	0,235524		1,186249	0,026969	0,11552	protein_coding	ITGB3BP	integrin subunit beta 3 binding protein [Source:HGNC Symbol;Acc:HGNC:6157]	1

ENSG00000203965	120,2216	0,002307	0,669898	0,426288	0,025621	0,050681	protein_coding	EFCAB7	EF-hand calcium binding domain 7 [Source:HGNC Symbol;Acc:HGNC:29379]	1
ENSG00000116652	10,50459	-0,00106	0,529388	-0,62894	0,026049	-0,23081	lncRNA	DLEU2L	deleted in lymphocytic leukemia 2 like [Source:HGNC Symbol;Acc:HGNC:13225]	1
ENSG00000079739	670,5728	0,006446	0,438805	0,774212	0,02564	0,057736	protein_coding	PGM1	phosphoglucomutase 1 [Source:HGNC Symbol;Acc:HGNC:8905]	1
ENSG00000185483	268,3202	0,003427	0,534282	0,621483	0,025784	0,073112	protein_coding	ROR1	receptor tyrosine kinase like orphan receptor 1 [Source:HGNC Symbol;Acc:HGNC:10256]	1
ENSG00000158966	361,7479	0,003721	0,509665	0,659359	0,025369	0,058273	protein_coding	CACHD1	cache domain containing 1 [Source:HGNC Symbol;Acc:HGNC:29314]	1
ENSG00000162437	201,6698	-0,00169	0,809064	-0,24163	0,025115	-0,02164	protein_coding	RAVER2	ribonucleoprotein, PTB binding 2 [Source:HGNC Symbol;Acc:HGNC:25577]	1
ENSG00000162433	135,3217	0,005944	0,236702	1,183271	0,026548	0,154179	protein_coding	AK4	adenylate kinase 4 [Source:HGNC Symbol;Acc:HGNC:363]	1
ENSG00000116675	644,0213	-0,15137	0,000341	-3,58165	0,057236	-0,18436	protein_coding	DNAJC6	DnaJ heat shock protein family (Hsp40) member C6 [Source:HGNC Symbol;Acc:HGNC:15469]	1
ENSG00000213625	1168,497	-0,00837	0,507451	-0,66281	0,023697	-0,02907	protein_coding	LEPROT	leptin receptor overlapping transcript [Source:HGNC Symbol;Acc:HGNC:29477]	1
ENSG00000116678	134,0545	0,001331	0,778212	0,28165	0,025668	0,038321	protein_coding	LEPR	leptin receptor [Source:HGNC Symbol;Acc:HGNC:6554]	1
ENSG00000184588	259,0459	-0,00374	0,35241	-0,92992	0,026158	-0,15541	protein_coding	PDE4B	phosphodiesterase 4B [Source:HGNC Symbol;Acc:HGNC:8781]	1
ENSG00000118473	366,8681	-0,00959	0,14665	-1,45147	0,027736	-0,1471	protein_coding	SGIP1	SH3GL interacting endocytic adaptor 1 [Source:HGNC Symbol;Acc:HGNC:25412]	1
ENSG00000152760	10,19829	-0,00248	0,161374	-1,40047	0,026198	-0,53535	protein_coding	TCTEX1D1	Tctex1 domain containing 1 [Source:HGNC Symbol;Acc:HGNC:26882]	1
ENSG00000152763	84,65559	-0,00317	0,52341	-0,6381	0,025861	-0,08394	protein_coding	DNAI4	dynein axonemal intermediate chain 4 [Source:HGNC Symbol;Acc:HGNC:26252]	1
ENSG00000198160	1542,541	0,006352	0,608072	0,512828	0,023441	0,023558	protein_coding	MIER1	MIER1 transcriptional regulator [Source:HGNC Symbol;Acc:HGNC:29657]	1
ENSG00000116704	639,8758	0,004545	0,577704	0,556741	0,024514	0,034538	protein_coding	SLC35D1	solute carrier family 35 member D1 [Source:HGNC Symbol;Acc:HGNC:20800]	1
ENSG00000116717	831,3125	0,010487	0,208382	1,258026	0,027332	0,093177	protein_coding	GADD45A	growth arrest and DNA damage inducible alpha [Source:HGNC Symbol;Acc:HGNC:4095]	1
ENSG00000162595	150,8457	-0,00606	0,133614	-1,5	0,026819	-0,25768	protein_coding	DIRAS3	DIRAS family GTPase 3 [Source:HGNC Symbol;Acc:HGNC:687]	1
ENSG00000234383	23,25809	-0,00585	0,009318	-2,60017	0,026973	-0,77669	processed_pseudog	CTBP2P8	CTBP2 pseudogene 8 [Source:HGNC Symbol;Acc:HGNC:45200]	1
ENSG00000024526	302,1122	0,004138	0,373816	0,889348	0,026132	0,127269	protein_coding	DEPDC1	DEP domain containing 1 [Source:HGNC Symbol;Acc:HGNC:22949]	1
ENSG00000066557	259,6148	0,007604	0,320258	0,993928	0,026373	0,083307	protein_coding	LRRC40	leucine rich repeat containing 40 [Source:HGNC Symbol;Acc:HGNC:26004]	1
ENSG00000118454	684,2705	-0,00599	0,509695	-0,65931	0,02422	-0,03545	protein_coding	ANKRD13C	ankyrin repeat domain 13C [Source:HGNC Symbol;Acc:HGNC:25374]	1
ENSG00000197568	46,45167	-0,003	0,426074	-0,79593	0,026055	-0,14045	lncRNA	HHLA3	HERV-H LTR-associating 3 [Source:HGNC Symbol;Acc:HGNC:4906]	1
ENSG00000116761	44,8631	-0,00128	0,727318	-0,34869	0,025862	-0,06316	protein_coding	CTH	cystathionine gamma-lyase [Source:HGNC Symbol;Acc:HGNC:2501]	1
ENSG00000050628	100,9196	-0,00124	0,711376	-0,37001	0,025811	-0,06151	protein_coding	PTGER3	prostaglandin E receptor 3 [Source:HGNC Symbol;Acc:HGNC:9595]	1
ENSG00000132485	1203,745	0,037081	0,040051	2,053225	0,038429	0,078651	protein_coding	ZRANB2	zinc finger RANBP2-type containing 2 [Source:HGNC Symbol;Acc:HGNC:13058]	1
ENSG00000172260	144,9821	1,31E-05	0,998625	0,001724	0,025793	0,000298	protein_coding	NEGR1	neuronal growth regulator 1 [Source:HGNC Symbol;Acc:HGNC:17302]	1
ENSG00000162620	17,50829	0,00129	0,56517	0,575179	0,026032	0,17167	protein_coding	LRRIQ3	leucine rich repeats and IQ motif containing 3 [Source:HGNC Symbol;Acc:HGNC:28318]	1
ENSG00000254685	92,0285	0,006251	0,234369	1,189179	0,026544	0,142129	protein_coding	FPGT	fucose-1-phosphate guanylyltransferase [Source:HGNC Symbol;Acc:HGNC:3825]	1
ENSG00000178965	34,55902	-0,00342	0,146827	-1,45083	0,026306	-0,40985	protein_coding	ERICH3	glutamate rich 3 [Source:HGNC Symbol;Acc:HGNC:25346]	1
ENSG00000116791	616,7968	0,018484	0,108445	1,605222	0,031954	0,096813	protein_coding	CRYZ	crystallin zeta [Source:HGNC Symbol;Acc:HGNC:2419]	1
ENSG00000162623	601,9565	0,001713	0,856415	0,180939	0,023889	0,010592	protein_coding	TYW3	tRNA-yW synthesizing protein 3 homolog [Source:HGNC Symbol;Acc:HGNC:24757]	1
ENSG00000137968	78,75738	0,004648	0,123204	1,541465	0,026503	0,325172	protein_coding	SLC44A5	solute carrier family 44 member 5 [Source:HGNC Symbol;Acc:HGNC:28524]	1
ENSG00000117054	727,0206	-0,00127	0,882196	-0,14819	0,023268	-0,00758	protein_coding	ACADM	acyl-CoA dehydrogenase medium chain [Source:HGNC Symbol;Acc:HGNC:89]	1
ENSG00000137955	802,9024	0,011255	0,276511	1,08819	0,026566	0,062738	protein_coding	RABGGTB	Rab geranylgeranyltransferase subunit beta [Source:HGNC Symbol;Acc:HGNC:9796]	1
ENSG00000057468	12,10208	0,000645	0,710372	0,371357	0,026034	0,142504	protein_coding	MSH4	mutS homolog 4 [Source:HGNC Symbol;Acc:HGNC:7327]	1
ENSG00000184005	10,46432	-0,00171	0,270042	-1,10297	0,02612	-0,47881	protein_coding	ST6GALNA	ST6 N-acetylgalactosaminide alpha-2,6-sialyltransferase 3 [Source:HGNC Symbol;Acc:HGNC:19343]	1
ENSG00000226415	35,52833	0,00172	0,575414	0,560096	0,025981	0,121652	processed_pseudog	TPI1P1	triosephosphate isomerase 1 pseudogene 1 [Source:HGNC Symbol;Acc:HGNC:35449]	1
ENSG00000117069	24,61236	0,000991	0,72347	0,353825	0,025957	0,084742	protein_coding	ST6GALNA	ST6 N-acetylgalactosaminide alpha-2,6-sialyltransferase 5 [Source:HGNC Symbol;Acc:HGNC:19342]	1
ENSG00000142892	771,8716	0,017748	0,129782	1,514961	0,030788	0,086103	protein_coding	PIGK	phosphatidylinositol glycan anchor biosynthesis class K [Source:HGNC Symbol;Acc:HGNC:8965]	1
ENSG00000226084	48,40227	-0,00135	0,630479	-0,48105	0,025855	-0,08524	processed_pseudog	AC113935.	ribosomal protein L17 (RPL17) pseudogene	1
ENSG00000154027	169,8869	0,005494	0,237538	1,181163	0,0265	0,169391	protein_coding	AK5	adenylate kinase 5 [Source:HGNC Symbol;Acc:HGNC:365]	1
ENSG00000036549	951,5712	0,000406	0,991353	0,010838	0,022748	0,000504	protein_coding	ZZZ3	zinc finger ZZ-type containing 3 [Source:HGNC Symbol;Acc:HGNC:24523]	1
ENSG00000077254	1235,381	0,016572	0,214574	1,241087	0,026311	0,049394	protein_coding	USP33	ubiquitin specific peptidase 33 [Source:HGNC Symbol;Acc:HGNC:20059]	1
ENSG00000180488	641,4273	0,009416	0,400443	0,84083	0,02459	0,039973	protein_coding	MIGA1	mitoguardin 1 [Source:HGNC Symbol;Acc:HGNC:24741]	1
ENSG00000219201	18,07921	-0,0032	0,250818	-1,14837	0,026255	-0,31137	processed_pseudog	AC138392.	novel pseudogene	1
ENSG00000235927	17,33353	0,002132	0,379922	0,878041	0,026113	0,261743	lncRNA	NEXN-AS1	NEXN antisense RNA 1 [Source:HGNC Symbol;Acc:HGNC:31983]	1
ENSG00000162614	1090,637	0,014456	0,074568	1,783115	0,03063	0,155793	protein_coding	NEXN	nexilin F-actin binding protein [Source:HGNC Symbol;Acc:HGNC:29557]	1
ENSG00000162616	785,2778	0,007072	0,489667	0,690838	0,024295	0,035591	protein_coding	DNAJB4	DnaJ heat shock protein family (Hsp40) member B4 [Source:HGNC Symbol;Acc:HGNC:14886]	1
ENSG00000137960	62,95958	0,005974	0,108334	1,605727	0,026831	0,295017	protein_coding	GIPC2	GIPC PDZ domain containing family member 2 [Source:HGNC Symbol;Acc:HGNC:18177]	1
ENSG00000122420	13,20188	-0,00057	0,759233	-0,30649	0,026023	-0,10898	protein_coding	PTGFR	prostaglandin F receptor [Source:HGNC Symbol;Acc:HGNC:9600]	1
ENSG00000137959	33,19079	-0,00218	0,035929	-2,09773	0,026198	-1,229	protein_coding	IFI44L	interferon induced protein 44 like [Source:HGNC Symbol;Acc:HGNC:17817]	1

ENSG00000137965	228,1325	-0,00477	0,458976	-0,74053	0,02584	-0,07412	protein_coding	IFI44	interferon induced protein 44 [Source:HGNC Symbol;Acc:HGNC:16938]	1
ENSG00000162618	139,1472	0,004646	0,383709	0,871083	0,025998	0,098588	protein_coding	ADGRL4	adhesion G protein-coupled receptor L4 [Source:HGNC Symbol;Acc:HGNC:20822]	1
ENSG00000137941	730,6152	-0,01897	0,169099	-1,3751	0,028779	-0,0597	protein_coding	TTLL7	tubulin tyrosine ligase like 7 [Source:HGNC Symbol;Acc:HGNC:26242]	1
ENSG00000271576	10,20237	0,001822	0,303382	1,029208	0,026117	0,392214	lncRNA	AL359504.	novel transcript	1
ENSG00000142875	906,3186	0,00717	0,550855	0,596479	0,023934	0,028688	protein_coding	PRKACB	protein kinase cAMP-activated catalytic subunit beta [Source:HGNC Symbol;Acc:HGNC:9381]	1
ENSG00000117133	527,3809	0,001607	0,954434	0,05714	0,024189	0,003633	protein_coding	RPF1	ribosome production factor 1 homolog [Source:HGNC Symbol;Acc:HGNC:30350]	1
ENSG00000174021	340,8339	-0,00044	0,954698	-0,05681	0,024934	-0,00481	protein_coding	GNG5	G protein subunit gamma 5 [Source:HGNC Symbol;Acc:HGNC:4408]	1
ENSG00000117151	381,3371	-0,00788	0,510483	-0,65809	0,025625	-0,04252	protein_coding	CTBS	chitobiase [Source:HGNC Symbol;Acc:HGNC:2496]	1
ENSG00000117155	678,4789	0,008052	0,349999	0,934592	0,026128	0,06794	protein_coding	SSX2IP	SSX family member 2 interacting protein [Source:HGNC Symbol;Acc:HGNC:16509]	1
ENSG00000162643	28,82897	0,000793	0,768411	0,294454	0,025957	0,072795	protein_coding	DNAI3	dynein axonemal intermediate chain 3 [Source:HGNC Symbol;Acc:HGNC:30711]	1
ENSG00000055732	187,6425	0,001079	0,841952	0,199397	0,025517	0,023888	protein_coding	MCOLN3	mucolipin 3 [Source:HGNC Symbol;Acc:HGNC:13358]	1
ENSG00000097096	116,6228	-0,0037	0,499542	-0,67521	0,025848	-0,08043	protein_coding	SYDE2	synapse defective Rho GTPase homolog 2 [Source:HGNC Symbol;Acc:HGNC:25841]	1
ENSG00000162642	298,4619	0,002244	0,774698	0,286235	0,024872	0,02242	protein_coding	C1orf52	chromosome 1 open reading frame 52 [Source:HGNC Symbol;Acc:HGNC:24871]	1
ENSG00000142867	484,5495	0,011442	0,235959	1,185148	0,02735	0,077965	protein_coding	BCL10	BCL10 immune signaling adaptor [Source:HGNC Symbol;Acc:HGNC:989]	1
ENSG00000153904	1256,351	0,011484	0,311916	1,011209	0,025931	0,051645	protein_coding	DDAH1	dimethylarginine dimethylaminohydrolase 1 [Source:HGNC Symbol;Acc:HGNC:2715]	1
ENSG00000282057	25,91766	0,00348	0,11077	1,594743	0,026331	0,477623	lncRNA	AC092807.	novel transcript	1
ENSG00000117174	494,6842	0,010678	0,238483	1,178788	0,027248	0,083391	protein_coding	ZNHIT6	zinc finger HIT-type containing 6 [Source:HGNC Symbol;Acc:HGNC:26089]	1
ENSG00000171502	60,09445	-0,00138	0,719472	-0,35916	0,025841	-0,06155	protein_coding	COL24A1	collagen type XXIV alpha 1 chain [Source:HGNC Symbol;Acc:HGNC:20821]	1
ENSG00000122417	528,8667	0,002435	0,824604	0,221627	0,024066	0,013343	protein_coding	ODF2L	outer dense fiber of sperm tails 2 like [Source:HGNC Symbol;Acc:HGNC:29225]	1
ENSG00000261737	23,87405	0,000787	0,626927	0,486057	0,025965	0,123776	lncRNA	CLCA4-AS1	CLCA4 antisense RNA 1 [Source:NCBI gene (formerly Entrezgene);Acc:105378828]	1
ENSG00000183291	1552,087	0,008209	0,462435	0,734843	0,024698	0,038167	protein_coding	SELENOF	selenoprotein F [Source:HGNC Symbol;Acc:HGNC:17705]	1
ENSG00000153936	1102,851	0,003785	0,720392	0,357935	0,023584	0,018519	protein_coding	HS2ST1	heparan sulfate 2-O-sulfotransferase 1 [Source:HGNC Symbol;Acc:HGNC:5193]	1
ENSG00000143013	737,9483	0,001952	0,829679	0,215113	0,024183	0,013532	protein_coding	LMO4	LIM domain only 4 [Source:HGNC Symbol;Acc:HGNC:6644]	1
ENSG00000237505	18,73377	0,001108	0,271832	1,098854	0,026096	0,670472	lncRNA	PKN2-AS1	PKN2 antisense RNA 1 [Source:HGNC Symbol;Acc:HGNC:50597]	1
ENSG00000065243	1099,955	-0,01002	0,423644	-0,80012	0,023511	-0,03194	protein_coding	PKN2	protein kinase N2 [Source:HGNC Symbol;Acc:HGNC:9406]	1
ENSG00000137947	460,5283	0,005964	0,454968	0,747159	0,025629	0,058489	protein_coding	GTF2B	general transcription factor IIB [Source:HGNC Symbol;Acc:HGNC:4648]	1
ENSG00000137944	291,6543	-0,00384	0,613125	-0,50562	0,02522	-0,0414	protein_coding	KYAT3	kynurenine aminotransferase 3 [Source:HGNC Symbol;Acc:HGNC:33238]	1
ENSG00000213516	270,8111	0,010799	0,21623	1,236614	0,027457	0,090623	protein_coding	RBMXL1	RBMX like 1 [Source:HGNC Symbol;Acc:HGNC:25073]	1
ENSG00000117226	1379,446	0,020132	0,054166	1,925508	0,035245	0,139008	protein_coding	GBP3	guanylate binding protein 3 [Source:HGNC Symbol;Acc:HGNC:4184]	1
ENSG00000117228	486,88	0,008333	0,095037	1,669405	0,027534	0,222317	protein_coding	GBP1	guanylate binding protein 1 [Source:HGNC Symbol;Acc:HGNC:4182]	1
ENSG00000234518	43,314	0,001394	0,619675	0,49631	0,025979	0,116159	processed_pseudog	PTGES3P1	prostaglandin E synthase 3 pseudogene 1 [Source:HGNC Symbol;Acc:HGNC:43824]	1
ENSG00000162645	370,2706	0,003087	0,526778	0,632932	0,025872	0,08576	protein_coding	GBP2	guanylate binding protein 2 [Source:HGNC Symbol;Acc:HGNC:4183]	1
ENSG00000162654	93,10994	-0,00092	0,545263	-0,60487	0,026055	-0,24927	protein_coding	GBP4	guanylate binding protein 4 [Source:HGNC Symbol;Acc:HGNC:20480]	1
ENSG00000225492	10,22235	-0,00029	0,816479	-0,23208	0,026053	-0,11842	transcribed_unpro	GBP1P1	guanylate binding protein 1 pseudogene 1 [Source:HGNC Symbol;Acc:HGNC:39561]	1
ENSG00000197147	447,3575	-0,01312	0,115554	-1,57371	0,029214	-0,12444	protein_coding	LRRC8B	leucine rich repeat containing 8 VRAC subunit B [Source:HGNC Symbol;Acc:HGNC:30692]	1
ENSG00000231999	23,85577	0,001662	0,533265	0,62303	0,026028	0,159808	lncRNA	LRRC8C-DT	LRRC8C divergent transcript [Source:HGNC Symbol;Acc:HGNC:53731]	1
ENSG00000171488	620,4096	0,013901	0,135094	1,494314	0,029408	0,108823	protein_coding	LRRC8C	leucine rich repeat containing 8 VRAC subunit C [Source:HGNC Symbol;Acc:HGNC:25075]	1
ENSG00000171492	496,8399	-0,17204	0,000759	-3,36731	0,076601	-0,22234	protein_coding	LRRC8D	leucine rich repeat containing 8 VRAC subunit D [Source:HGNC Symbol;Acc:HGNC:16992]	1
ENSG00000228175	15,23652	0,001527	0,441389	0,76985	0,026073	0,261535	processed_pseudog	GEMIN8P4	gem nuclear organelle associated protein 8 pseudogene 4 [Source:HGNC Symbol;Acc:HGNC:37979]	1
ENSG00000162664	840,3052	-0,01876	0,175267	-1,35547	0,028286	-0,05721	protein_coding	ZNF326	zinc finger protein 326 [Source:HGNC Symbol;Acc:HGNC:14104]	1
ENSG00000233593	47,93157	0,000582	0,986659	-0,01672	0,025829	-0,00304	lncRNA	LINC02609	long intergenic non-protein coding RNA 2609 [Source:HGNC Symbol;Acc:HGNC:27140]	1
ENSG00000122482	1388,162	0,001215	0,900759	0,124703	0,023647	0,006936	protein_coding	ZNF644	zinc finger protein 644 [Source:HGNC Symbol;Acc:HGNC:29222]	1
ENSG00000097046	176,5488	0,001399	0,831517	0,212757	0,025232	0,020525	protein_coding	CDC7	cell division cycle 7 [Source:HGNC Symbol;Acc:HGNC:1745]	1
ENSG00000069702	210,6131	-0,01197	0,076543	-1,77111	0,029205	-0,18094	protein_coding	TGFBR3	transforming growth factor beta receptor 3 [Source:HGNC Symbol;Acc:HGNC:11774]	1
ENSG00000172031	15,874	0,000694	0,697288	0,388983	0,026035	0,147104	protein_coding	EPHX4	epoxide hydrolase 4 [Source:HGNC Symbol;Acc:HGNC:23758]	1
ENSG00000189195	129,0069	0,003506	0,502019	0,671317	0,025811	0,07991	protein_coding	BTBD8	BTB domain containing 8 [Source:HGNC Symbol;Acc:HGNC:21019]	1
ENSG00000174842	119,9258	-0,00079	0,897675	-0,1286	0,025301	-0,01323	protein_coding	GLMN	glomulin, FKBP associated protein [Source:HGNC Symbol;Acc:HGNC:14373]	1
ENSG00000122484	548,8883	-0,00582	0,527435	-0,63193	0,025119	-0,04253	protein_coding	RPAP2	RNA polymerase II associated protein 2 [Source:HGNC Symbol;Acc:HGNC:25791]	1
ENSG00000162676	81,86421	-0,0054	0,132415	-1,50465	0,026661	-0,28365	protein_coding	GFI1	growth factor independent 1 transcriptional repressor [Source:HGNC Symbol;Acc:HGNC:4237]	1
ENSG00000067208	1489,159	0,003132	0,732191	0,342213	0,023652	0,018321	protein_coding	EVI5	ecotropic viral integration site 5 [Source:HGNC Symbol;Acc:HGNC:3501]	1
ENSG00000154511	257,2317	0,006642	0,34358	0,947115	0,026239	0,087166	protein_coding	DIPK1A	divergent protein kinase domain 1A [Source:HGNC Symbol;Acc:HGNC:32213]	1
ENSG00000207523	223,5358	-0,00057	0,792477	-0,2631	0,025995	-0,07848	snoRNA	SNORA66	small nucleolar RNA, H/ACA box 66 [Source:HGNC Symbol;Acc:HGNC:10223]	1

ENSG00000143033	331,2073	0,006578	0,456377	0,744826	0,025314	0,049865	protein_coding	MTF2	metal response element binding transcription factor 2 [Source:HGNC Symbol;Acc:HGNC:29535]	1
ENSG00000117500	1419,097	-0,00203	0,850821	-0,18807	0,02311	-0,00922	protein_coding	TMED5	transmembrane p24 trafficking protein 5 [Source:HGNC Symbol;Acc:HGNC:24251]	1
ENSG00000122483	244,511	0,005292	0,372457	0,891881	0,026219	0,104735	protein_coding	CCDC18	coiled-coil domain containing 18 [Source:HGNC Symbol;Acc:HGNC:30370]	1
ENSG00000223745	88,84795	-0,00363	0,510244	-0,65846	0,025825	-0,07781	lncRNA	CCDC18-AS	CCDC18 antisense RNA 1 [Source:HGNC Symbol;Acc:HGNC:52262]	1
ENSG00000117505	1519,43	0,0069	0,556938	0,587396	0,023287	0,025769	protein_coding	DR1	down-regulator of transcription 1 [Source:HGNC Symbol;Acc:HGNC:3017]	1
ENSG00000137942	946,7866	-0,00582	0,607067	-0,51427	0,023335	-0,02368	protein_coding	FNBP1L	formin binding protein 1 like [Source:HGNC Symbol;Acc:HGNC:20851]	1
ENSG00000137936	506,6676	0,005614	0,442589	0,767828	0,025786	0,066668	protein_coding	BCAR3	BCAR3 adaptor protein, NSP family member [Source:HGNC Symbol;Acc:HGNC:973]	1
ENSG00000224093	50,495	-0,00143	0,536584	-0,61799	0,026037	-0,17992	lncRNA	BCAR3-AS1	BCAR3 antisense RNA 1 [Source:HGNC Symbol;Acc:HGNC:40093]	1
ENSG00000067334	1368,109	0,015445	0,254658	1,139108	0,025595	0,044754	protein_coding	DNTTIP2	deoxynucleotidyltransferase terminal interacting protein 2 [Source:HGNC Symbol;Acc:HGNC:24013]	1
ENSG00000023909	1560,388	-0,01367	0,218186	-1,23137	0,027198	-0,06392	protein_coding	GCLM	glutamate-cysteine ligase modifier subunit [Source:HGNC Symbol;Acc:HGNC:4312]	1
ENSG00000198691	23,7582	0,003087	0,087561	1,708408	0,026292	0,640096	protein_coding	ABCA4	ATP binding cassette subfamily A member 4 [Source:HGNC Symbol;Acc:HGNC:34]	1
ENSG00000137962	454,3227	-0,00929	0,224553	-1,21451	0,027148	-0,10294	protein_coding	ARHGAP29	Rho GTPase activating protein 29 [Source:HGNC Symbol;Acc:HGNC:30207]	1
ENSG00000117528	1011,248	0,020241	0,152077	1,432234	0,029654	0,062231	protein_coding	ABCD3	ATP binding cassette subfamily D member 3 [Source:HGNC Symbol;Acc:HGNC:67]	1
ENSG00000117525	777,3884	-0,00547	0,393421	-0,85343	0,025977	-0,08294	protein_coding	F3	coagulation factor III, tissue factor [Source:HGNC Symbol;Acc:HGNC:3541]	1
ENSG00000224081	42,85879	-0,00271	0,390073	-0,85948	0,026071	-0,17152	transcribed_proces	SLC44A3-A	SLC44A3 antisense RNA 1 [Source:HGNC Symbol;Acc:HGNC:49057]	1
ENSG00000143036	72,90728	0,000461	0,913779	0,108273	0,025793	0,018493	protein_coding	SLC44A3	solute carrier family 44 member 3 [Source:HGNC Symbol;Acc:HGNC:28689]	1
ENSG00000235501	62,52113	-0,00142	0,720429	-0,35789	0,025825	-0,05975	lncRNA	CNN3-DT	CNN3 divergent transcript [Source:HGNC Symbol;Acc:HGNC:54176]	1
ENSG00000172339	132,6997	-0,00561	0,344489	-0,94533	0,026187	-0,10159	protein_coding	ALG14	ALG14 UDP-N-acetylglucosaminyltransferase subunit [Source:HGNC Symbol;Acc:HGNC:28287]	1
ENSG00000152078	210,8629	-0,01916	0,028404	-2,19166	0,035381	-0,19057	protein_coding	TLCD4	TLC domain containing 4 [Source:HGNC Symbol;Acc:HGNC:26477]	1
ENSG00000122481	47,31134	0,001162	0,777813	0,282171	0,025786	0,045515	protein_coding	RWDD3	RWD domain containing 3 [Source:HGNC Symbol;Acc:HGNC:21393]	1
ENSG00000228502	14,44444	-0,00063	0,732878	-0,3413	0,026006	-0,10809	processed_pseudo	EEF1A1P11	eukaryotic translation elongation factor 1 alpha 1 pseudogene 11 [Source:HGNC Symbol;Acc:HGNC:3206]	1
ENSG00000137970	32,03168	0,000545	0,850448	0,188547	0,025894	0,039728	processed_pseudo	RPL7P9	ribosomal protein L7 pseudogene 9 [Source:HGNC Symbol;Acc:HGNC:37028]	1
ENSG00000117569	173,9996	-0,00237	0,712474	-0,36854	0,025372	-0,03661	protein_coding	PTBP2	polypyrimidine tract binding protein 2 [Source:HGNC Symbol;Acc:HGNC:17662]	1
ENSG00000188641	404,5331	-0,00108	0,935544	-0,08087	0,024153	-0,00514	protein_coding	DPYD	dihydropyrimidine dehydrogenase [Source:HGNC Symbol;Acc:HGNC:3012]	1
ENSG00000228057	33,58023	0,001557	0,44758	0,759456	0,026039	0,212027	processed_pseudo	SEC63P1	SEC63 homolog, protein translocation regulator pseudogene 1 [Source:HGNC Symbol;Acc:HGNC:41953]	1
ENSG00000225206	16,76892	0,001152	0,52715	0,632362	0,026056	0,232045	lncRNA	MIR137HG	MIR137 host gene [Source:HGNC Symbol;Acc:HGNC:42871]	1
ENSG00000162627	562,359	0,033327	0,028896	2,184901	0,052919	0,131706	protein_coding	SNX7	sorting nexin 7 [Source:HGNC Symbol;Acc:HGNC:14971]	1
ENSG00000117600	506,268	0,001094	0,72536	0,351304	0,025927	0,075416	protein_coding	PLPPR4	phospholipid phosphatase related 4 [Source:HGNC Symbol;Acc:HGNC:23496]	1
ENSG00000099260	53,89551	-0,0004	0,866224	-0,16846	0,025974	-0,04688	protein_coding	PALMD	palmdelphin [Source:HGNC Symbol;Acc:HGNC:15846]	1
ENSG00000156869	209,809	0,003545	0,641031	0,466258	0,025411	0,042779	protein_coding	FRRS1	ferric chelate reductase 1 [Source:HGNC Symbol;Acc:HGNC:27622]	1
ENSG00000162688	680,8213	-0,01994	0,094835	-1,67043	0,032329	-0,0915	protein_coding	AGL	amylo-alpha-1, 6-glucosidase, 4-alpha-glucanotransferase [Source:HGNC Symbol;Acc:HGNC:321]	1
ENSG00000117620	294,8418	0,029772	0,018783	2,349815	0,052353	0,176683	protein_coding	SLC35A3	solute carrier family 35 member A3 [Source:HGNC Symbol;Acc:HGNC:11023]	1
ENSG00000156875	215,8842	0,005907	0,417399	0,810942	0,025963	0,073424	protein_coding	MFSD14A	major facilitator superfamily domain containing 14A [Source:HGNC Symbol;Acc:HGNC:23363]	1
ENSG00000156876	139,704	0,009299	0,13385	1,499091	0,027714	0,162975	protein_coding	SASS6	SAS-6 centriolar assembly protein [Source:HGNC Symbol;Acc:HGNC:25403]	1
ENSG00000122435	190,0987	0,004635	0,479191	0,707604	0,025773	0,069746	protein_coding	TRMT13	tRNA methyltransferase 13 homolog [Source:HGNC Symbol;Acc:HGNC:25502]	1
ENSG00000137992	390,2153	-0,00086	0,915638	-0,10593	0,02445	-0,00739	protein_coding	DBT	dihydrolipoamide branched chain transacylase E2 [Source:HGNC Symbol;Acc:HGNC:2698]	1
ENSG00000224616	60,91859	0,005003	0,239278	1,176794	0,02643	0,185022	lncRNA	RTCA-AS1	RTCA antisense RNA 1 [Source:HGNC Symbol;Acc:HGNC:50573]	1
ENSG00000137996	990,7829	0,032029	0,049142	1,967361	0,042808	0,095126	protein_coding	RTCA	RNA 3'-terminal phosphate cyclase [Source:HGNC Symbol;Acc:HGNC:17981]	1
ENSG00000079335	110,0495	-0,00723	0,178181	-1,34638	0,02698	-0,17001	protein_coding	CDC14A	cell division cycle 14A [Source:HGNC Symbol;Acc:HGNC:1718]	1
ENSG00000162692	807,9777	0,001171	0,811381	0,238645	0,025638	0,031898	protein_coding	VCAM1	vascular cell adhesion molecule 1 [Source:HGNC Symbol;Acc:HGNC:12663]	1
ENSG00000162694	543,6024	-0,00444	0,659216	-0,441	0,024078	-0,02477	protein_coding	EXTL2	exostosin like glycosyltransferase 2 [Source:HGNC Symbol;Acc:HGNC:3516]	1
ENSG00000162695	1387,728	-0,01162	0,371833	-0,89304	0,023483	-0,03334	protein_coding	SLC30A7	solute carrier family 30 member 7 [Source:HGNC Symbol;Acc:HGNC:19306]	1
ENSG00000117543	182,7741	0,008182	0,287107	1,064489	0,026596	0,088754	protein_coding	DPH5	diphthamide biosynthesis 5 [Source:HGNC Symbol;Acc:HGNC:24270]	1
ENSG00000233184	44,69111	-0,00086	0,801291	-0,25168	0,025869	-0,0484	lncRNA	AC093157.	novel transcript	1
ENSG00000170989	17,17852	-0,00044	0,722104	-0,35565	0,026011	-0,11921	protein_coding	S1PR1	sphingosine-1-phosphate receptor 1 [Source:HGNC Symbol;Acc:HGNC:3165]	1
ENSG00000183298	13,83792	-0,00221	0,313028	-1,00889	0,026128	-0,30779	processed_pseudo	RPSAP19	ribosomal protein SA pseudogene 19 [Source:HGNC Symbol;Acc:HGNC:36508]	1
ENSG00000060718	113,5627	-0,00025	0,801044	-0,252	0,026062	-0,15424	protein_coding	COL11A1	collagen type XI alpha 1 chain [Source:HGNC Symbol;Acc:HGNC:2186]	1
ENSG00000185946	198,4516	-0,00976	0,196596	-1,29131	0,027497	-0,11402	protein_coding	RNPC3	RNA binding region (RNP1, RRM) containing 3 [Source:HGNC Symbol;Acc:HGNC:18666]	1
ENSG00000240038	29,3504	-0,00345	0,180082	-1,3405	0,026292	-0,34745	protein_coding	AMY2B	amylase alpha 2B [Source:HGNC Symbol;Acc:HGNC:478]	1
ENSG00000198890	301,9051	-0,00413	0,62241	-0,49244	0,024824	-0,0342	protein_coding	PRMT6	protein arginine methyltransferase 6 [Source:HGNC Symbol;Acc:HGNC:18241]	1
ENSG00000162631	40,53031	0,001592	0,276195	1,088907	0,026113	0,486494	protein_coding	NTNG1	netrin G1 [Source:HGNC Symbol;Acc:HGNC:23319]	1
ENSG00000134215	92,98277	-0,00099	0,824926	-0,22121	0,025705	-0,03244	protein_coding	VAV3	vav guanine nucleotide exchange factor 3 [Source:HGNC Symbol;Acc:HGNC:12659]	1

ENSG00000085491	956,4562	0,017032	0,154632	1,423359	0,029985	0,078875	protein_coding	SLC25A24	solute carrier family 25 member 24 [Source:HGNC Symbol;Acc:HGNC:20662]	1
ENSG00000162636	863,6585	-0,01402	0,187087	-1,31925	0,028369	-0,07851	protein_coding	FAM102B	family with sequence similarity 102 member B [Source:HGNC Symbol;Acc:HGNC:27637]	1
ENSG00000162639	132,3913	0,003452	0,383237	0,871947	0,025714	0,096977	protein_coding	HENMT1	HEN methyltransferase 1 [Source:HGNC Symbol;Acc:HGNC:26400]	1
ENSG00000134186	1185,014	-0,00556	0,631934	-0,47901	0,023038	-0,0212	protein_coding	PRPF38B	pre-mRNA processing factor 38B [Source:HGNC Symbol;Acc:HGNC:25512]	1
ENSG00000116266	769,1039	0,008939	0,404185	0,83417	0,024945	0,043236	protein_coding	STXBP3	syntaxin binding protein 3 [Source:HGNC Symbol;Acc:HGNC:11446]	1
ENSG00000121957	626,4843	0,003935	0,605388	0,516668	0,025153	0,040811	protein_coding	GPSM2	G protein signaling modulator 2 [Source:HGNC Symbol;Acc:HGNC:29501]	1
ENSG00000121940	931,9691	-0,00175	0,873056	-0,15978	0,023519	-0,00857	protein_coding	CLCC1	chloride channel CLIC like 1 [Source:HGNC Symbol;Acc:HGNC:29675]	1
ENSG00000085433	545,2116	-0,00414	0,651415	-0,4518	0,02391	-0,02473	protein_coding	WDR47	WD repeat domain 47 [Source:HGNC Symbol;Acc:HGNC:29141]	1
ENSG00000244716	26,75593	0,000957	0,745019	0,325214	0,025923	0,070577	processed_pseudog	BX679664.	ribosomal protein L17 (RPL17) pseudogene	1
ENSG00000197780	597,3291	0,018897	0,060624	1,876228	0,033985	0,137932	protein_coding	TAF13	TATA-box binding protein associated factor 13 [Source:HGNC Symbol;Acc:HGNC:11546]	1
ENSG00000273382	27,92606	-0,00101	0,695296	-0,39168	0,025978	-0,09957	lncRNA	TMEM167I	TMEM167B divergent transcript [Source:HGNC Symbol;Acc:HGNC:55076]	1
ENSG00000215717	544,8131	0,014061	0,203879	1,270577	0,02809	0,072261	protein_coding	TMEM167I	transmembrane protein 167B [Source:HGNC Symbol;Acc:HGNC:30187]	1
ENSG00000116299	971,2987	-0,01608	0,117711	-1,56445	0,030419	-0,10106	protein_coding	ELAPOR1	endosome-lysosome associated apoptosis and autophagy regulator 1 [Source:HGNC Symbol;Acc:HGNC:29618]	1
ENSG00000143126	730,9224	-0,00551	0,526915	-0,63272	0,025147	-0,04406	protein_coding	CELSR2	cadherin EGF LAG seven-pass G-type receptor 2 [Source:HGNC Symbol;Acc:HGNC:3231]	1
ENSG00000134222	170,8345	0,009397	0,074386	1,784233	0,027942	0,216017	protein_coding	PSRC1	proline and serine rich coiled-coil 1 [Source:HGNC Symbol;Acc:HGNC:24472]	1
ENSG00000143106	1382,887	0,049567	0,018741	2,350637	0,052957	0,104344	protein_coding	PSMA5	proteasome 20S subunit alpha 5 [Source:HGNC Symbol;Acc:HGNC:9534]	1
ENSG00000143028	100,3782	0,000412	0,914338	0,107568	0,02561	0,01443	protein_coding	SYPL2	synaptophysin like 2 [Source:HGNC Symbol;Acc:HGNC:27638]	1
ENSG00000162650	138,0535	-0,00541	0,361885	-0,91178	0,025892	-0,0857	protein_coding	ATXN7L2	ataxin 7 like 2 [Source:HGNC Symbol;Acc:HGNC:28713]	1
ENSG00000174151	259,9663	-0,01163	0,179249	-1,34307	0,028132	-0,10363	protein_coding	CYB561D1	cytochrome b561 family member D1 [Source:HGNC Symbol;Acc:HGNC:26804]	1
ENSG00000181754	94,51212	-0,00236	0,606405	-0,51521	0,025817	-0,07403	protein_coding	AMIGO1	adhesion molecule with Ig like domain 1 [Source:HGNC Symbol;Acc:HGNC:20824]	1
ENSG00000116337	1247,339	-0,03401	0,042575	-2,02785	0,042895	-0,09263	protein_coding	AMPD2	adenosine monophosphate deaminase 2 [Source:HGNC Symbol;Acc:HGNC:469]	1
ENSG00000168765	196,3142	0,002793	0,681735	0,410097	0,025482	0,041986	protein_coding	GSTM4	glutathione S-transferase mu 4 [Source:HGNC Symbol;Acc:HGNC:4636]	1
ENSG00000213366	76,73394	0,004008	0,398804	0,843759	0,026082	0,118214	protein_coding	GSTM2	glutathione S-transferase mu 2 [Source:HGNC Symbol;Acc:HGNC:4634]	1
ENSG00000134184	92,19694	0,009025	0,01267	2,492917	0,028205	0,483582	protein_coding	GSTM1	glutathione S-transferase mu 1 [Source:HGNC Symbol;Acc:HGNC:4632]	1
ENSG00000134201	66,93257	0,002544	0,47043	0,72178	0,02602	0,136165	protein_coding	GSTM5	glutathione S-transferase mu 5 [Source:HGNC Symbol;Acc:HGNC:4637]	1
ENSG00000134202	802,5683	-0,02128	0,10166	-1,63686	0,032656	-0,08333	protein_coding	GSTM3	glutathione S-transferase mu 3 [Source:HGNC Symbol;Acc:HGNC:4635]	1
ENSG00000198758	363,9096	0,001507	0,71887	0,35997	0,025798	0,056887	protein_coding	EPS8L3	EPS8 like 3 [Source:HGNC Symbol;Acc:HGNC:21297]	1
ENSG00000184371	973,2629	-0,00116	0,897078	-0,12935	0,024659	-0,00966	protein_coding	CSF1	colony stimulating factor 1 [Source:HGNC Symbol;Acc:HGNC:2432]	1
ENSG00000143093	773,8881	-0,0068	0,518377	-0,64585	0,024448	-0,03476	protein_coding	STRIP1	striatin interacting protein 1 [Source:HGNC Symbol;Acc:HGNC:25916]	1
ENSG00000258634	127,7385	0,001346	0,870697	0,162773	0,025439	0,018069	lncRNA	AL160006.	novel transcript, antisense to ALX3	1
ENSG00000197106	167,7183	-0,00355	0,451075	-0,75362	0,025988	-0,10532	protein_coding	SLC6A17	solute carrier family 6 member 17 [Source:HGNC Symbol;Acc:HGNC:31399]	1
ENSG00000116396	557,0702	-0,0044	0,509484	-0,65964	0,025459	-0,05681	protein_coding	KCNC4	potassium voltage-gated channel subfamily C member 4 [Source:HGNC Symbol;Acc:HGNC:6236]	1
ENSG00000162775	655,5626	-0,01127	0,308352	-1,01869	0,025991	-0,05337	protein_coding	RBM15	RNA binding motif protein 15 [Source:HGNC Symbol;Acc:HGNC:14959]	1
ENSG00000224699	53,75777	-0,00381	0,324554	-0,98514	0,0262	-0,17201	lncRNA	LAMTOR5-	LAMTOR5 antisense RNA 1 [Source:HGNC Symbol;Acc:HGNC:40823]	1
ENSG00000168679	253,8711	-0,00199	0,718022	-0,3611	0,025565	-0,04218	protein_coding	SLC16A4	solute carrier family 16 member 4 [Source:HGNC Symbol;Acc:HGNC:10925]	1
ENSG00000273373	28,14965	-0,00332	0,233196	-1,19217	0,026249	-0,29349	lncRNA	AL355488.	novel transcript, antisense to SLC16A4	1
ENSG00000134248	366,4686	0,087659	0,007985	2,652715	0,08014	0,15061	protein_coding	LAMTOR5	late endosomal/lysosomal adaptor, MAPK and MTOR activator 5 [Source:HGNC Symbol;Acc:HGNC:17955]	1
ENSG00000177301	258,9067	-0,00411	0,360272	-0,91485	0,026158	-0,13682	protein_coding	KCNA2	potassium voltage-gated channel subfamily A member 2 [Source:HGNC Symbol;Acc:HGNC:6220]	1
ENSG00000259834	13,71498	-0,00264	0,097698	-1,65612	0,026234	-0,70821	lncRNA	AL365361.	novel transcript	1
ENSG00000177272	23,46892	0,000945	0,435688	0,779496	0,026035	0,269165	protein_coding	KCNA3	potassium voltage-gated channel subfamily A member 3 [Source:HGNC Symbol;Acc:HGNC:6221]	1
ENSG00000143119	71,46964	0,001477	0,646554	0,458554	0,025939	0,09457	protein_coding	CD53	CD53 molecule [Source:HGNC Symbol;Acc:HGNC:1686]	1
ENSG00000261654	14,08402	-0,00282	0,18485	-1,32597	0,026229	-0,44175	lncRNA	AL360270.	novel transcript, sense overlapping C1orf103	1
ENSG00000121931	411,9703	-0,00051	0,986233	0,017255	0,02491	0,001443	protein_coding	LRIF1	ligand dependent nuclear receptor interacting factor 1 [Source:HGNC Symbol;Acc:HGNC:30299]	1
ENSG00000156171	275,825	-0,00236	0,775619	-0,28503	0,025156	-0,02517	protein_coding	DRAM2	DNA damage regulated autophagy modulator 2 [Source:HGNC Symbol;Acc:HGNC:28769]	1
ENSG00000134255	346,6575	0,004313	0,614859	0,503149	0,024964	0,036142	protein_coding	CEPT1	choline/ethanolamine phosphotransferase 1 [Source:HGNC Symbol;Acc:HGNC:24289]	1
ENSG00000162777	178,9608	-0,00038	0,961015	-0,04888	0,025442	-0,00561	protein_coding	DENND2D	DENN domain containing 2D [Source:HGNC Symbol;Acc:HGNC:26192]	1
ENSG00000173947	12,85117	0,000479	0,76418	0,299996	0,026039	0,123719	protein_coding	PIFO	primary cilia formation [Source:HGNC Symbol;Acc:HGNC:27009]	1
ENSG00000085465	50,4606	-0,00606	0,067094	-1,83104	0,02691	-0,36993	protein_coding	OVGP1	oviductal glycoprotein 1 [Source:HGNC Symbol;Acc:HGNC:8524]	1
ENSG00000116455	221,703	0,001806	0,776156	0,284332	0,025329	0,028526	protein_coding	WDR77	WD repeat domain 77 [Source:HGNC Symbol;Acc:HGNC:29652]	1
ENSG00000116459	1378,03	0,021954	0,11149	1,591529	0,032616	0,077491	protein_coding	ATP5PB	ATP synthase peripheral stalk-membrane subunit b [Source:HGNC Symbol;Acc:HGNC:840]	1
ENSG00000143110	27,92984	0,002108	0,383838	0,870847	0,026107	0,25218	protein_coding	C1orf162	chromosome 1 open reading frame 162 [Source:HGNC Symbol;Acc:HGNC:28344]	1
ENSG00000116473	973,7	0,011441	0,196178	1,292517	0,026732	0,075483	protein_coding	RAP1A	RAP1A, member of RAS oncogene family [Source:HGNC Symbol;Acc:HGNC:9855]	1

ENSG00000197852	451,883	-0,00454	0,474708	-0,71484	0,025836	-0,07382	protein_coding	INKA2	inka box actin regulator 2 [Source:HGNC Symbol;Acc:HGNC:28045]	1
ENSG00000227811	31,07662	0,000545	0,842091	0,19922	0,025947	0,048934	lncRNA	INKA2-AS1	INKA2 antisense RNA 1 [Source:HGNC Symbol;Acc:HGNC:49446]	1
ENSG00000064703	481,4876	-0,00483	0,520054	-0,64326	0,025025	-0,04545	protein_coding	DDX20	DEAD-box helicase 20 [Source:HGNC Symbol;Acc:HGNC:2743]	1
ENSG00000171385	517,5959	-0,00678	0,256051	-1,13577	0,026656	-0,12797	protein_coding	KCND3	potassium voltage-gated channel subfamily D member 3 [Source:HGNC Symbol;Acc:HGNC:6239]	1
ENSG00000143079	1202,233	0,007277	0,444078	0,765325	0,025127	0,046166	protein_coding	CTTNBP2N	CTTNBP2 N-terminal like [Source:HGNC Symbol;Acc:HGNC:25330]	1
ENSG00000134245	25,32688	-0,00082	0,758266	-0,30776	0,025961	-0,07648	protein_coding	WNT2B	Wnt family member 2B [Source:HGNC Symbol;Acc:HGNC:12781]	1
ENSG00000007341	262,6491	0,000769	0,941701	0,073133	0,024754	0,005708	protein_coding	ST7L	suppression of tumorigenicity 7 like [Source:HGNC Symbol;Acc:HGNC:18441]	1
ENSG00000155363	637,2427	-0,01263	0,300775	-1,03477	0,026372	-0,0519	protein_coding	MOV10	Mov10 RISC complex RNA helicase [Source:HGNC Symbol;Acc:HGNC:7200]	1
ENSG00000155366	1131,25	0,027226	0,049743	1,962167	0,041402	0,112711	protein_coding	RHOC	ras homolog family member C [Source:HGNC Symbol;Acc:HGNC:669]	1
ENSG00000155367	10,15383	-0,00185	0,25913	-1,12845	0,026127	-0,45484	protein_coding	PPM1J	protein phosphatase, Mg2+/Mn2+ dependent 1J [Source:HGNC Symbol;Acc:HGNC:20785]	1
ENSG00000155380	829,09	0,082033	0,007427	2,677059	0,062285	0,130264	protein_coding	SLC16A1	solute carrier family 16 member 1 [Source:HGNC Symbol;Acc:HGNC:10922]	1
ENSG00000226419	71,97868	0,006494	0,157555	1,413341	0,026914	0,222874	lncRNA	SLC16A1-A	SLC16A1 antisense RNA 1 [Source:HGNC Symbol;Acc:HGNC:49445]	1
ENSG00000198799	407,8564	-0,02522	0,046072	-1,99474	0,040333	-0,12591	protein_coding	LRIG2	leucine rich repeats and immunoglobulin like domains 2 [Source:HGNC Symbol;Acc:HGNC:20889]	1
ENSG00000081026	525,8329	-0,00464	0,56101	-0,58134	0,025249	-0,04508	protein_coding	MAGI3	membrane associated guanylate kinase, WW and PDZ domain containing 3 [Source:HGNC Symbol;Acc:HGNC:29647]	1
ENSG00000116793	777,9951	-0,00137	0,88201	-0,14842	0,0234	-0,0078	protein_coding	PHTF1	putative homeodomain transcription factor 1 [Source:HGNC Symbol;Acc:HGNC:8939]	1
ENSG00000081019	373,9695	-0,00126	0,886569	-0,14265	0,024489	-0,01002	protein_coding	RSBN1	round spermatid basic protein 1 [Source:HGNC Symbol;Acc:HGNC:25642]	1
ENSG00000134242	15,2678	0,0017	0,402928	0,836404	0,026087	0,281561	protein_coding	PTPN22	protein tyrosine phosphatase non-receptor type 22 [Source:HGNC Symbol;Acc:HGNC:9652]	1
ENSG00000134262	219,1529	-0,02216	0,022393	-2,28364	0,039258	-0,19209	protein_coding	AP4B1	adaptor related protein complex 4 subunit beta 1 [Source:HGNC Symbol;Acc:HGNC:572]	1
ENSG00000118655	203,5136	-0,00526	0,52801	-0,63105	0,025534	-0,05146	protein_coding	DCLRE1B	DNA cross-link repair 1B [Source:HGNC Symbol;Acc:HGNC:17641]	1
ENSG00000116774	392,7081	0,003921	0,559245	0,583964	0,025532	0,054806	protein_coding	OLFML3	olfactomedin like 3 [Source:HGNC Symbol;Acc:HGNC:24956]	1
ENSG00000197323	1335,395	-0,04921	0,023831	-2,25985	0,052797	-0,10036	protein_coding	TRIM33	tripartite motif containing 33 [Source:HGNC Symbol;Acc:HGNC:16290]	1
ENSG00000236480	15,00617	-0,00032	0,868375	-0,16572	0,026012	-0,05705	processed_pseudog	PKMP1	pyruvate kinase M1/2 pseudogene 1 [Source:HGNC Symbol;Acc:HGNC:44243]	1
ENSG00000116752	444,4264	0,008704	0,37566	0,885921	0,025659	0,053631	protein_coding	BCAS2	BCAS2 pre-mRNA processing factor [Source:HGNC Symbol;Acc:HGNC:975]	1
ENSG00000175984	63,269	0,002007	0,657728	0,443052	0,025814	0,066965	protein_coding	DENND2C	DENN domain containing 2C [Source:HGNC Symbol;Acc:HGNC:24748]	1
ENSG00000213281	1250,443	0,016502	0,137385	1,485599	0,030345	0,090575	protein_coding	NRAS	NRAS proto-oncogene, GTPase [Source:HGNC Symbol;Acc:HGNC:7989]	1
ENSG00000052723	776,4174	-0,02148	0,125127	-1,53361	0,031357	-0,07043	protein_coding	SIKE1	suppressor of IKBKE 1 [Source:HGNC Symbol;Acc:HGNC:26119]	1
ENSG00000134198	42,06322	0,001789	0,464992	0,730651	0,026057	0,200846	protein_coding	TSPAN2	tetraspanin 2 [Source:HGNC Symbol;Acc:HGNC:20659]	1
ENSG00000134259	82,53633	0,000647	0,80542	0,246338	0,025994	0,072299	protein_coding	NGF	nerve growth factor [Source:HGNC Symbol;Acc:HGNC:7808]	1
ENSG00000173218	499,7124	0,006538	0,449103	0,756911	0,025523	0,053967	protein_coding	VANGL1	VANGL planar cell polarity protein 1 [Source:HGNC Symbol;Acc:HGNC:15512]	1
ENSG00000177551	20,10567	0,002441	0,144181	1,460396	0,026201	0,588859	protein_coding	NHLH2	nescient helix-loop-helix 2 [Source:HGNC Symbol;Acc:HGNC:7818]	1
ENSG00000163393	173,7399	-0,00055	0,487296	-0,69462	0,025179	-0,0666	protein_coding	SLC22A15	solute carrier family 22 member 15 [Source:HGNC Symbol;Acc:HGNC:20301]	1
ENSG00000173212	11,56246	0,002451	0,211824	1,248565	0,026179	0,429969	protein_coding	MAB21L3	mab-21 like 3 [Source:HGNC Symbol;Acc:HGNC:26787]	1
ENSG00000203865	189,2117	-0,0017	0,795084	-0,25971	0,025371	-0,02691	lncRNA	ATP1A1-AS	ATP1A1 antisense RNA 1 [Source:HGNC Symbol;Acc:HGNC:28262]	1
ENSG00000116815	149,0832	-0,00446	0,528946	-0,62962	0,025558	-0,05662	protein_coding	CD58	CD58 molecule [Source:HGNC Symbol;Acc:HGNC:1688]	1
ENSG00000177173	12,10855	-0,00026	0,897637	-0,12865	0,02602	-0,0468	processed_pseudog	NAP1L4P1	nucleosome assembly protein 1 like 4 pseudogene 1 [Source:HGNC Symbol;Acc:HGNC:39740]	1
ENSG00000143061	287,5729	0,008339	0,168375	1,377444	0,027281	0,152708	protein_coding	IGSF3	immunoglobulin superfamily member 3 [Source:HGNC Symbol;Acc:HGNC:5950]	1
ENSG00000134247	1403,547	0,011354	0,200882	1,279041	0,027782	0,093876	protein_coding	PTGFRN	prostaglandin F2 receptor inhibitor [Source:HGNC Symbol;Acc:HGNC:9601]	1
ENSG00000134256	17,35984	0,000775	0,706594	0,376434	0,026017	0,122563	protein_coding	CD101	CD101 molecule [Source:HGNC Symbol;Acc:HGNC:5949]	1
ENSG00000116830	343,7502	-0,00182	0,61861	-0,49782	0,025346	-0,05035	protein_coding	TTF2	transcription termination factor 2 [Source:HGNC Symbol;Acc:HGNC:12398]	1
ENSG00000134253	45,41387	-0,00434	0,412093	-0,82022	0,026341	-0,14712	protein_coding	TRIM45	tripartite motif containing 45 [Source:HGNC Symbol;Acc:HGNC:19018]	1
ENSG00000183508	284,5659	0,003223	0,619255	0,496906	0,025558	0,051095	protein_coding	TENT5C	terminal nucleotidyltransferase 5C [Source:HGNC Symbol;Acc:HGNC:24712]	1
ENSG00000196505	752,436	-0,00631	0,507527	-0,66269	0,025154	-0,0434	protein_coding	GDAP2	ganglioside induced differentiation associated protein 2 [Source:HGNC Symbol;Acc:HGNC:18010]	1
ENSG00000065183	722,432	0,020231	0,119632	1,556319	0,031524	0,077985	protein_coding	WDR3	WD repeat domain 3 [Source:HGNC Symbol;Acc:HGNC:12755]	1
ENSG00000155761	23,43314	-0,00057	0,646887	-0,45809	0,026059	-0,22835	protein_coding	SPAG17	sperm associated antigen 17 [Source:HGNC Symbol;Acc:HGNC:26620]	1
ENSG00000092607	91,98094	-0,00037	0,890624	-0,13752	0,02594	-0,03323	protein_coding	TBX15	T-box transcription factor 15 [Source:HGNC Symbol;Acc:HGNC:11594]	1
ENSG00000116874	164,5416	-0,00696	0,325789	-0,98263	0,026306	-0,08858	protein_coding	WARS2	tryptophanyl tRNA synthetase 2, mitochondrial [Source:HGNC Symbol;Acc:HGNC:12730]	1
ENSG00000231365	218,8011	-0,01075	0,120017	-1,5547	0,028341	-0,15381	lncRNA	WARS2-AS	WARS2 antisense RNA 1 [Source:HGNC Symbol;Acc:HGNC:40612]	1
ENSG00000260941	13,03023	-0,00049	0,816902	-0,23153	0,026002	-0,07368	lncRNA	LINC00622	long intergenic non-protein coding RNA 622 [Source:HGNC Symbol;Acc:HGNC:44251]	1
ENSG00000143067	391,4125	0,005377	0,559032	0,58428	0,025048	0,039911	protein_coding	ZNF697	zinc finger protein 697 [Source:HGNC Symbol;Acc:HGNC:32034]	1
ENSG00000092621	1156,309	0,003706	0,740497	0,331195	0,02336	0,01642	protein_coding	PHGDH	phosphoglycerate dehydrogenase [Source:HGNC Symbol;Acc:HGNC:8923]	1
ENSG00000134240	431,3159	-0,00032	0,839583	-0,20243	0,026016	-0,07023	protein_coding	HMGCS2	3-hydroxy-3-methylglutaryl-CoA synthase 2 [Source:HGNC Symbol;Acc:HGNC:5008]	1
ENSG00000134193	343,9785	0,000725	0,348439	0,937622	0,02609	0,839751	protein_coding	REG4	regenerating family member 4 [Source:HGNC Symbol;Acc:HGNC:22977]	1

ENSG00000261662	15,18908	0,002929	0,183539	1,329937	0,026234	0,408426				
ENSG00000273131	67,39786	-0,00359	0,421848	-0,80322	0,026044	-0,11895				
ENSG00000188610	33,29365	1,19E-05	0,997653	0,002942	0,025934	0,000713	protein_coding	FAM72B	family with sequence similarity 72 member B [Source:HGNC Symbol;Acc:HGNC:24805]	1
ENSG00000213244	12,54113	0,00098	0,623983	0,490214	0,026033	0,166856	unprocessed_pseu	H3P4	H3 histone pseudogene 4 [Source:HGNC Symbol;Acc:HGNC:43797]	1
ENSG00000223345	37,09292	0,004362	0,160215	1,404348	0,026477	0,341619	transcribed_unpro	H2BP1	H2B histone pseudogene 1 [Source:HGNC Symbol;Acc:HGNC:20560]	1
ENSG00000171943	156,842	0,000875	0,884112	0,145759	0,025352	0,015524	protein_coding	SRGAP2C	SLIT-ROBO Rho GTPase activating protein 2C [Source:HGNC Symbol;Acc:HGNC:30584]	1
ENSG00000269996	175,532	0,000725	0,911402	0,111271	0,025216	0,010879				
ENSG00000231752	46,13894	0,002268	0,463804	0,732598	0,026041	0,158845	transcribed_unpro	EMBP1	embigin pseudogene 1 [Source:HGNC Symbol;Acc:HGNC:38661]	1
ENSG00000230880	17,41991	-0,00084	0,629844	-0,48195	0,026044	-0,18706				
ENSG00000234978	14,69668	-0,00272	0,23453	-1,18877	0,026192	-0,35151				
ENSG00000230850	18,92827	1,11E-05	0,996993	0,003769	0,025992	0,001187				
ENSG00000185044	24,51394	-0,00322	0,272081	-1,09828	0,026217	-0,25922				
ENSG00000238261	11,33039	-0,00109	0,487848	-0,69374	0,026069	-0,29826				
ENSG00000223804	39,71131	-0,00044	0,885446	-0,14407	0,025905	-0,03142	transcribed_unpro	AC244669.	Poly [ADP-ribose] polymerase 8 (PARP8) pseudogene	1
ENSG00000215784	25,2589	0,003444	0,107275	1,610562	0,026334	0,510735	protein_coding	FAM72D	family with sequence similarity 72 member D [Source:HGNC Symbol;Acc:HGNC:33593]	1
ENSG00000196369	199,4503	-0,01034	0,180874	-1,33807	0,027737	-0,11509	protein_coding	SRGAP2B	SLIT-ROBO Rho GTPase activating protein 2B [Source:HGNC Symbol;Acc:HGNC:35237]	1
ENSG00000236943	102,0881	0,006857	0,154556	1,423622	0,026945	0,19896				
ENSG00000225241	374,24	-0,01063	0,249104	-1,15253	0,027133	-0,07971				
ENSG00000168614	286,5913	0,004514	0,568131	0,570806	0,025205	0,043963				
ENSG00000178104	1452,226	-0,01586	0,240076	-1,1748	0,025247	-0,04393	protein_coding	PDE4DIP	phosphodiesterase 4D interacting protein [Source:HGNC Symbol;Acc:HGNC:15580]	1
ENSG00000272755	12,72902	0,000586	0,712657	0,36829	0,026042	0,151722	lncRNA	AC245297.	novel transcript	1
ENSG00000265808	1075,149	0,007456	0,453089	0,750274	0,025261	0,046016	protein_coding	SEC22B	SEC22 homolog B, vesicle trafficking protein [Source:HGNC Symbol;Acc:HGNC:10700]	1
ENSG00000264343	137,7971	-0,00253	0,779537	-0,27992	0,025742	-0,03612	protein_coding	NOTCH2NL	notch 2 N-terminal like A [Source:HGNC Symbol;Acc:HGNC:31862]	1
ENSG00000286219	90,98994	0,000221	0,958138	0,05249	0,025732	0,008213	protein_coding	NOTCH2NL	notch 2 N-terminal like C [Source:HGNC Symbol;Acc:HGNC:53924]	1
ENSG00000271425	123,0206	-0,00949	0,107389	-1,61004	0,02788	-0,18137	protein_coding	NBPF10	NBPF member 10 [Source:HGNC Symbol;Acc:HGNC:31992]	1
ENSG00000286185	17,96303	-0,00114	0,62012	-0,49568	0,026024	-0,15056	protein_coding	AC242842.	novel protein, identical to neuroblastoma breakpoint family, member 19 NBPF19	1
ENSG00000233396	26,26061	-0,00155	0,533055	-0,62335	0,02603	-0,16877	lncRNA	LINC01719	long intergenic non-protein coding RNA 1719 [Source:HGNC Symbol;Acc:HGNC:52506]	1
ENSG00000287190	9,387843	0,002004	0,131512	1,508165	0,026164	0,758137	lncRNA	AC239799.	novel transcript	1
ENSG00000265972	987,778	0,001619	0,841463	0,200023	0,024703	0,014989	protein_coding	TXNIP	thioredoxin interacting protein [Source:HGNC Symbol;Acc:HGNC:16952]	1
ENSG00000121851	202,2923	0,014337	0,080981	1,745024	0,03048	0,150719	protein_coding	POLR3GL	RNA polymerase III subunit GL [Source:HGNC Symbol;Acc:HGNC:28466]	1
ENSG00000272031	60,26062	-0,00525	0,188805	-1,31412	0,026466	-0,19838	protein_coding	ANKRD34A	ankyrin repeat domain 34A [Source:HGNC Symbol;Acc:HGNC:27639]	1
ENSG00000234222	14,96344	0,001914	0,280416	1,079384	0,026125	0,403325	lncRNA	LIX1L-AS1	LIX1L antisense RNA 1 [Source:HGNC Symbol;Acc:HGNC:41210]	1
ENSG00000271601	722,8463	0,015459	0,161654	1,399532	0,029469	0,084547	protein_coding	LIX1L	limb and CNS expressed 1 like [Source:HGNC Symbol;Acc:HGNC:28715]	1
ENSG00000211451	13,18367	-0,00426	0,040762	-2,04594	0,02652	-0,67152	transcribed_unitar	GNRHR2	gonadotropin releasing hormone receptor 2 (pseudogene) [Source:HGNC Symbol;Acc:HGNC:16341]	1
ENSG00000131779	184,3274	-0,00666	0,347617	-0,93922	0,026214	-0,08523	protein_coding	PEX11B	peroxisomal biogenesis factor 11 beta [Source:HGNC Symbol;Acc:HGNC:8853]	1
ENSG00000143127	192,4661	-0,28583	0,002527	-3,0201	0,187134	-0,41865	protein_coding	ITGA10	integrin subunit alpha 10 [Source:HGNC Symbol;Acc:HGNC:6135]	1
ENSG00000198483	38,43006	0,000831	0,767494	0,295655	0,025947	0,070087	protein_coding	ANKRD35	ankyrin repeat domain 35 [Source:HGNC Symbol;Acc:HGNC:26323]	1
ENSG00000131788	736,196	-0,00766	0,460694	-0,7377	0,024787	-0,04031	protein_coding	PIAS3	protein inhibitor of activated STAT 3 [Source:HGNC Symbol;Acc:HGNC:16861]	1
ENSG00000186364	35,54879	-0,00385	0,321674	-0,99102	0,026296	-0,20528	protein_coding	NUDT17	nudix hydrolase 17 [Source:HGNC Symbol;Acc:HGNC:26618]	1
ENSG00000186141	745,5215	0,016166	0,196417	1,291826	0,028722	0,066668	protein_coding	POLR3C	RNA polymerase III subunit C [Source:HGNC Symbol;Acc:HGNC:30076]	1
ENSG00000265491	1063,975	-0,00271	0,858703	-0,17803	0,022403	-0,00763	protein_coding	RNF115	ring finger protein 115 [Source:HGNC Symbol;Acc:HGNC:18154]	1
ENSG00000174827	195,4036	0,005277	0,182175	1,334089	0,026594	0,238362	protein_coding	PDZK1	PDZ domain containing 1 [Source:HGNC Symbol;Acc:HGNC:8821]	1
ENSG00000117262	103,389	0,000416	0,959549	0,050719	0,025598	0,006742	protein_coding	GPR89A	G protein-coupled receptor 89A [Source:HGNC Symbol;Acc:HGNC:31984]	1
ENSG00000207418	39,42825	-0,00264	0,275262	-1,09103	0,026165	-0,29758				
ENSG00000152042	15,53685	0,001104	0,63125	0,479968	0,026016	0,141158				
ENSG00000232637	61,08634	-0,0058	0,284601	-1,07004	0,026637	-0,16149				
ENSG00000186275	88,59565	-0,00128	0,787163	-0,27	0,025672	-0,03715				
ENSG00000286172	13,49514	-0,0025	0,167259	-1,38107	0,026204	-0,54619	snRNA	RNVU1-8	RNA, variant U1 small nuclear 8 [Source:HGNC Symbol;Acc:HGNC:48315]	1
ENSG00000131791	610,5521	0,005695	0,584438	0,546914	0,024215	0,029819	protein_coding	PRKAB2	protein kinase AMP-activated non-catalytic subunit beta 2 [Source:HGNC Symbol;Acc:HGNC:9379]	1
ENSG00000180867	123,0471	0,002785	0,616923	0,500216	0,025681	0,059263	transcribed_proces	PDIA3P1	protein disulfide isomerase family A member 3 pseudogene 1 [Source:HGNC Symbol;Acc:HGNC:4607]	1
ENSG00000131781	199,4332	-0,00542	0,385366	-0,86805	0,026033	-0,08803	protein_coding	FMO5	flavin containing dimethylaniline monoxygenase 5 [Source:HGNC Symbol;Acc:HGNC:3773]	1
ENSG00000271721	27,82336	-0,00139	0,669094	-0,42739	0,025971	-0,09811				

ENSG00000226015	19,33222	-0,0009	0,707778		-0,37484	0,025991	-0,10308	processed_pseudog	CCT8P1	chaperonin containing TCP1 subunit 8 pseudogene 1 [Source:HGNC Symbol;Acc:HGNC:35144]	1
ENSG00000131778	71,75716	-0,00355	0,402329		-0,83747	0,026079	-0,13133	protein_coding	CHD1L	chromodomain helicase DNA binding protein 1 like [Source:HGNC Symbol;Acc:HGNC:1916]	1
ENSG00000116128	754,3455	-0,01687	0,12829		-1,52088	0,029984	-0,08551	protein_coding	BCL9	BCL9 transcription coactivator [Source:HGNC Symbol;Acc:HGNC:1008]	1
ENSG00000162836	152,864	0,001705	0,733683		0,340231	0,025411	0,036306	protein_coding	ACP6	acid phosphatase 6, lysophosphatidic [Source:HGNC Symbol;Acc:HGNC:29609]	1
ENSG00000265107	13,17904	0,000546	0,630767		0,480648	0,026064	0,256964	protein_coding	GJA5	gap junction protein alpha 5 [Source:HGNC Symbol;Acc:HGNC:4279]	1
ENSG00000188092	27,99357	-0,00158	0,523013		-0,63871	0,026032	-0,1706	protein_coding	GPR89B	G protein-coupled receptor 89B [Source:HGNC Symbol;Acc:HGNC:13840]	1
ENSG00000206585	58,92338	-0,00097	0,708132		-0,37437	0,025973	-0,09397	snRNA	RNVU1-7	RNA, variant U1 small nuclear 7 [Source:HGNC Symbol;Acc:HGNC:37500]	1
ENSG00000280649	12,67397	0,000933	0,519419		0,644242	0,026036	0,225083	TEC	AC245100.	TEC	1
ENSG00000212456	23,71576	0,002455	0,3068		1,02196	0,026148	0,292182				
ENSG00000122497	177,3938	0,003137	0,65833		0,44222	0,025312	0,039961				
ENSG00000223612	29,50755	-0,00198	0,486395		-0,69605	0,026037	-0,16583	processed_pseudog	AC241585.	EGFR-coamplified and overexpressed protein (ECOP) pseudogene	1
ENSG00000276216	14,91826	0,004822	0,020492		2,317217	0,02667	0,77482	lncRNA	AC245014.	novel transcript	1
ENSG00000203832	84,80988	-0,00169	0,737679		-0,33493	0,025655	-0,04376				
ENSG00000266338	271,34	-0,00064	0,936149		-0,08011	0,024513	-0,00573	protein_coding	NBPF15	NBPF member 15 [Source:HGNC Symbol;Acc:HGNC:28791]	1
ENSG00000207205	14,6945	0,00184	0,307787		1,019876	0,026117	0,384182	snRNA	RNVU1-15	RNA, variant U1 small nuclear 15 [Source:HGNC Symbol;Acc:HGNC:48320]	1
ENSG00000203827	21,88018	-0,00077	0,790875		-0,26517	0,025939	-0,06203				
ENSG00000232527	27,27316	-0,00029	0,919402		-0,10119	0,025919	-0,02314	lncRNA	LINC02802	long intergenic non-protein coding RNA 2802 [Source:HGNC Symbol;Acc:HGNC:54326]	1
ENSG00000206737	166,5146	-0,00513	0,31889		-0,99674	0,026309	-0,13144	snRNA	RNVU1-18	RNA, variant U1 small nuclear 18 [Source:HGNC Symbol;Acc:HGNC:37496]	1
ENSG00000235999	58,57572	-0,00079	0,800364		-0,25288	0,02577	-0,04101				
ENSG00000273213	21,91477	0,002815	0,277091		1,086878	0,026179	0,285031	protein_coding	H3-2	H3.2 histone (putative) [Source:HGNC Symbol;Acc:HGNC:32060]	1
ENSG00000223759	11,86932	-0,00048	0,815924		-0,23279	0,026005	-0,07476				
ENSG00000203815	18,3118	-0,0018	0,457928		-0,74226	0,02605	-0,19685				
ENSG00000203814	495,5007	0,009319	0,116502		1,569627	0,02782	0,179245	protein_coding	H2BC18	H2B clustered histone 18 [Source:HGNC Symbol;Acc:HGNC:24700]	1
ENSG00000183598	59,55463	0,004206	0,180665		1,33871	0,026364	0,26763	protein_coding	H3C13	H3 clustered histone 13 [Source:HGNC Symbol;Acc:HGNC:25311]	1
ENSG00000270882	12,29888	0,000253	0,890473		0,137705	0,02602	0,050848	protein_coding	H4C14	H4 clustered histone 14 [Source:HGNC Symbol;Acc:HGNC:4794]	1
ENSG00000220323	142,766	-0,00515	0,333226		-0,96764	0,02637	-0,13519	transcribed_unproc	H2BC19P	H2B clustered histone 19, pseudogene [Source:HGNC Symbol;Acc:HGNC:20517]	1
ENSG00000261716	200,1683	-0,00417	0,501602		-0,67197	0,025757	-0,06994	transcribed_unproc	H2BC20P	H2B clustered histone 20, pseudogene [Source:HGNC Symbol;Acc:HGNC:20516]	1
ENSG00000184678	610,3761	0,003422	0,686849		0,403135	0,024765	0,028621	protein_coding	H2BC21	H2B clustered histone 21 [Source:HGNC Symbol;Acc:HGNC:4760]	1
ENSG00000184260	1076,612	0,13558	0,002476		3,026198	0,077942	0,190386	protein_coding	H2AC20	H2A clustered histone 20 [Source:HGNC Symbol;Acc:HGNC:4738]	1
ENSG00000184270	321,6028	0,021007	0,012894		2,486674	0,038546	0,240517	protein_coding	H2AC21	H2A clustered histone 21 [Source:HGNC Symbol;Acc:HGNC:20508]	1
ENSG00000178096	76,65452	-0,00304	0,576618		-0,55833	0,025872	-0,07614	protein_coding	BOLA1	bolA family member 1 [Source:HGNC Symbol;Acc:HGNC:24263]	1
ENSG00000159164	923,652	0,008824	0,376768		0,883868	0,025582	0,052043	protein_coding	SV2A	synaptic vesicle glycoprotein 2A [Source:HGNC Symbol;Acc:HGNC:20566]	1
ENSG00000143368	1202,255	0,048561	0,026248		2,222521	0,05392	0,100603	protein_coding	SF3B4	splicing factor 3b subunit 4 [Source:HGNC Symbol;Acc:HGNC:10771]	1
ENSG00000014914	273,1514	-0,0032	0,567067		-0,57238	0,024891	-0,04304	protein_coding	MTMR11	myotubularin related protein 11 [Source:HGNC Symbol;Acc:HGNC:24307]	1
ENSG00000264522	1162,695	-0,00385	0,716496		-0,36315	0,023521	-0,0185	protein_coding	OTUD7B	OTU deubiquitinase 7B [Source:HGNC Symbol;Acc:HGNC:16683]	1
ENSG00000285184	55,44646	0,001643	0,653518		0,44888	0,025919	0,085599	lncRNA	AC244033.	novel transcript	1
ENSG00000136631	555,433	0,011033	0,298729		1,039162	0,026325	0,058612	protein_coding	VPS45	vacuolar protein sorting 45 homolog [Source:HGNC Symbol;Acc:HGNC:14579]	1
ENSG00000023902	1170,672	0,046293	0,022927		2,274651	0,061644	0,115065	protein_coding	PLEKHO1	pleckstrin homology domain containing O1 [Source:HGNC Symbol;Acc:HGNC:24310]	1
ENSG00000143401	1198,119	0,01692	0,10395		1,625997	0,031198	0,106055	protein_coding	ANP32E	acidic nuclear phosphoprotein 32 family member E [Source:HGNC Symbol;Acc:HGNC:16673]	1
ENSG00000117362	729,3541	0,011425	0,309783		1,015678	0,026211	0,054363	protein_coding	APH1A	aph-1 homolog A, gamma-secretase subunit [Source:HGNC Symbol;Acc:HGNC:29509]	1
ENSG00000118292	165,8376	-0,00076	0,904511		-0,11997	0,025188	-0,01151	protein_coding	C1orf54	chromosome 1 open reading frame 54 [Source:HGNC Symbol;Acc:HGNC:26258]	1
ENSG00000159208	120,5759	-0,00359	0,531305		-0,62601	0,025804	-0,07312	protein_coding	CIART	circadian associated repressor of transcription [Source:HGNC Symbol;Acc:HGNC:25200]	1
ENSG00000266472	472,1988	0,009596	0,304504		1,026822	0,02647	0,068458	protein_coding	MRPS21	mitochondrial ribosomal protein S21 [Source:HGNC Symbol;Acc:HGNC:14046]	1
ENSG00000117360	884,9961	-0,0054	0,538858		-0,61454	0,024607	-0,03717	protein_coding	PRPF3	pre-mRNA processing factor 3 [Source:HGNC Symbol;Acc:HGNC:17348]	1
ENSG00000143374	327,9154	-0,00238	0,82361		-0,2229	0,024689	-0,01621	protein_coding	TARS2	threonyl-tRNA synthetase 2, mitochondrial [Source:HGNC Symbol;Acc:HGNC:30740]	1
ENSG00000237781	20,75175	-0,00107	0,591832		-0,53618	0,026037	-0,17795	lncRNA	ADAMTSL4	ADAMTSL4 antisense RNA 2 [Source:HGNC Symbol;Acc:HGNC:40895]	1
ENSG00000143382	34,35728	-0,00118	0,820985		-0,22628	0,025947	-0,05045	protein_coding	ADAMTSL4	ADAMTS like 4 [Source:HGNC Symbol;Acc:HGNC:19706]	1
ENSG00000203804	23,1516	-0,00449	0,052184		-1,94162	0,026559	-0,57179	lncRNA	ADAMTSL4	ADAMTSL4 antisense RNA 1 [Source:HGNC Symbol;Acc:HGNC:32041]	1
ENSG00000143457	442,0186	-0,01691	0,128645		-1,51947	0,030526	-0,09076	protein_coding	GOLPH3L	golgi phosphoprotein 3 like [Source:HGNC Symbol;Acc:HGNC:24882]	1
ENSG00000163131	520,6713	0,007639	0,098652		1,651425	0,027318	0,240838	protein_coding	CTSS	cathepsin S [Source:HGNC Symbol;Acc:HGNC:2545]	1
ENSG00000143387	1101,801	-0,00733	0,207721		-1,25986	0,026895	-0,14502	protein_coding	CTSK	cathepsin K [Source:HGNC Symbol;Acc:HGNC:2536]	1
ENSG00000143437	1291,483	0,013891	0,287064		1,064585	0,024746	0,041283	protein_coding	ARNT	aryl hydrocarbon receptor nuclear translocator [Source:HGNC Symbol;Acc:HGNC:700]	1

ENSG00000143379	626,3165	-0,00219	0,896825		-0,12967	0,023856	-0,00746	protein_coding	SETDB1	SET domain bifurcated histone lysine methyltransferase 1 [Source:HGNC Symbol;Acc:HGNC:10761]	1
ENSG00000259357	13,98631	-0,00342	0,089493		-1,69808	0,026326	-0,53072	lncRNA	AL590133.	novel transcript, antisense to CERS2	1
ENSG00000143412	16,62789	-0,00161	0,413152		-0,81836	0,026076	-0,27499	protein_coding	ANXA9	annexin A9 [Source:HGNC Symbol;Acc:HGNC:547]	1
ENSG00000143409	328,3442	-0,00815	0,248841		-1,15317	0,026708	-0,10071	protein_coding	MINDY1	MINDY lysine 48 deubiquitinase 1 [Source:HGNC Symbol;Acc:HGNC:25648]	1
ENSG00000143363	351,2777	-0,00542	0,550123		-0,59758	0,024937	-0,03939	protein_coding	PRUNE1	prune exopolyphosphatase 1 [Source:HGNC Symbol;Acc:HGNC:13420]	1
ENSG00000163141	9,768631	-0,0018	0,255559		-1,13695	0,026125	-0,47229	protein_coding	BNIPL	BCL2 interacting protein like [Source:HGNC Symbol;Acc:HGNC:16976]	1
ENSG00000143443	106,0238	-0,01267	0,020971		-2,3085	0,030225	-0,30557	protein_coding	C1orf56	chromosome 1 open reading frame 56 [Source:HGNC Symbol;Acc:HGNC:26045]	1
ENSG00000213190	1391,265	-0,00291	0,715812		-0,36406	0,024885	-0,02774	protein_coding	MLLT11	MLLT11 transcription factor 7 cofactor [Source:HGNC Symbol;Acc:HGNC:16997]	1
ENSG00000143458	307,9718	0,006879	0,357251		0,920615	0,026023	0,074792	protein_coding	GABPB2	GA binding protein transcription factor subunit beta 2 [Source:HGNC Symbol;Acc:HGNC:28441]	1
ENSG00000143434	100,5153	0,000921	0,823778		0,222689	0,025715	0,033168	protein_coding	SEMA6C	semaphorin 6C [Source:HGNC Symbol;Acc:HGNC:10740]	1
ENSG00000163156	366,5393	0,0057	0,595043		0,531542	0,025521	0,041185	protein_coding	SCNM1	sodium channel modifier 1 [Source:HGNC Symbol;Acc:HGNC:23136]	1
ENSG00000163155	157,7636	0,005309	0,357005		0,921086	0,02598	0,092084	protein_coding	LYSMD1	LysM domain containing 1 [Source:HGNC Symbol;Acc:HGNC:32070]	1
ENSG00000163159	677,2659	0,003855	0,694921		0,392186	0,024248	0,023497	protein_coding	VPS72	vacuolar protein sorting 72 homolog [Source:HGNC Symbol;Acc:HGNC:11644]	1
ENSG00000143398	1159,015	-0,01034	0,371187		-0,89425	0,024303	-0,03891	protein_coding	PIP5K1A	phosphatidylinositol-4-phosphate 5-kinase type 1 alpha [Source:HGNC Symbol;Acc:HGNC:8994]	1
ENSG00000143373	613,0555	-0,02774	0,038882		-2,06543	0,04222	-0,11908	protein_coding	ZNF687	zinc finger protein 687 [Source:HGNC Symbol;Acc:HGNC:29277]	1
ENSG00000143393	1478,204	0,00401	0,742627		0,328376	0,022088	0,013077	protein_coding	PI4KB	phosphatidylinositol 4-kinase beta [Source:HGNC Symbol;Acc:HGNC:8984]	1
ENSG00000143390	631,7831	-0,00508	0,705825		-0,37747	0,024143	-0,02086	protein_coding	RFX5	regulatory factor X5 [Source:HGNC Symbol;Acc:HGNC:9986]	1
ENSG00000143416	288,0665	-0,00259	0,670028		-0,42611	0,025384	-0,04185	protein_coding	SELENBP1	selenium binding protein 1 [Source:HGNC Symbol;Acc:HGNC:10719]	1
ENSG00000159377	1365,943	0,005085	0,597961		0,527335	0,023508	0,025706	protein_coding	PSMB4	proteasome 20S subunit beta 4 [Source:HGNC Symbol;Acc:HGNC:9541]	1
ENSG00000143375	566,0878	-0,00875	0,189574		-1,31184	0,027289	-0,13197	protein_coding	CGN	cingulin [Source:HGNC Symbol;Acc:HGNC:17429]	1
ENSG00000143367	470,7564	0,000299	0,978569		0,026863	0,025009	0,00237	protein_coding	TUFT1	tuftelin 1 [Source:HGNC Symbol;Acc:HGNC:12422]	1
ENSG00000143376	1350,997	-0,00294	0,83727		-0,20539	0,021186	-0,00733	protein_coding	SNX27	sorting nexin 27 [Source:HGNC Symbol;Acc:HGNC:20073]	1
ENSG00000159409	412,9105	-0,00719	0,295036		-1,04714	0,026535	-0,10071	protein_coding	CELF3	CUGBP Elav-like family member 3 [Source:HGNC Symbol;Acc:HGNC:11967]	1
ENSG00000143436	742,5502	0,015497	0,189491		1,312086	0,028409	0,068817	protein_coding	MRPL9	mitochondrial ribosomal protein L9 [Source:HGNC Symbol;Acc:HGNC:14277]	1
ENSG00000143450	19,39993	0,001574	0,490409		0,689659	0,026009	0,170452	protein_coding	OAZ3	ornithine decarboxylase antizyme 3 [Source:HGNC Symbol;Acc:HGNC:8097]	1
ENSG00000182134	240,4505	-0,00856	0,191644		-1,30573	0,027223	-0,13265	protein_coding	TDRKH	tudor and KH domain containing [Source:HGNC Symbol;Acc:HGNC:11713]	1
ENSG00000203288	21,9133	-0,00092	0,717804		-0,3614	0,02598	-0,09479	lncRNA	TDRKH-AS1	TDRKH antisense RNA 1 [Source:HGNC Symbol;Acc:HGNC:40578]	1
ENSG00000143365	54,73911	0,000894	0,731923		0,342569	0,025972	0,08712	protein_coding	RORC	RAR related orphan receptor C [Source:HGNC Symbol;Acc:HGNC:10260]	1
ENSG00000234614	10,20362	0,001562	0,371489		0,893687	0,026091	0,337466	lncRNA	C2CD4D-AS	C2CD4D and THEM5 antisense RNA 1 [Source:HGNC Symbol;Acc:HGNC:54045]	1
ENSG00000159445	244,2415	0,01022	0,145326		1,45624	0,027978	0,140879	protein_coding	THEM4	thioesterase superfamily member 4 [Source:HGNC Symbol;Acc:HGNC:17947]	1
ENSG00000159450	66,41042	0,001101	0,686286		0,4039	0,025969	0,097426	protein_coding	TCHH	trichohyalin [Source:HGNC Symbol;Acc:HGNC:11791]	1
ENSG00000237975	22,24356	-0,00179	0,409289		-0,82515	0,026079	-0,2477	lncRNA	FLG-AS1	FLG antisense RNA 1 [Source:HGNC Symbol;Acc:HGNC:27913]	1
ENSG00000163209	12,40389	0,001555	0,272261		1,097871	0,026117	0,538188	protein_coding	SPRR3	small proline rich protein 3 [Source:HGNC Symbol;Acc:HGNC:11268]	1
ENSG00000203783	34,51997	-0,0001	0,741856		-0,3294	0,026081	-0,53995	protein_coding	PRR9	proline rich 9 [Source:HGNC Symbol;Acc:HGNC:32057]	1
ENSG00000163220	44,55523	0,001341	0,443801		0,765791	0,02607	0,27351	protein_coding	S100A9	S100 calcium binding protein A9 [Source:HGNC Symbol;Acc:HGNC:10499]	1
ENSG00000196154	269,432	-0,30177	0,001229		-3,23214	0,149771	-0,39905	protein_coding	S100A4	S100 calcium binding protein A4 [Source:HGNC Symbol;Acc:HGNC:10494]	1
ENSG00000188015	167,4301	-0,00252	0,496083		-0,68067	0,025991	-0,12279	protein_coding	S100A3	S100 calcium binding protein A3 [Source:HGNC Symbol;Acc:HGNC:10493]	1
ENSG00000196754	35,76203	0,001835	0,449938		0,755518	0,026062	0,206793	protein_coding	S100A2	S100 calcium binding protein A2 [Source:HGNC Symbol;Acc:HGNC:10492]	1
ENSG00000285867	16,70133	-0,00146	0,481481		-0,70392	0,02606	-0,22893	lncRNA	BX470102.	Novel transcript	1
ENSG00000188643	1323,86	0,021477	0,049917		1,960676	0,036604	0,137163	protein_coding	S100A16	S100 calcium binding protein A16 [Source:HGNC Symbol;Acc:HGNC:20441]	1
ENSG00000189334	35,27827	-0,00309	0,11696		-1,56766	0,026282	-0,53343	protein_coding	S100A14	S100 calcium binding protein A14 [Source:HGNC Symbol;Acc:HGNC:18901]	1
ENSG00000189171	864,2665	0,030476	0,041605		2,037453	0,046975	0,119822	protein_coding	S100A13	S100 calcium binding protein A13 [Source:HGNC Symbol;Acc:HGNC:10490]	1
ENSG00000272030	9,901702	0,000864	0,612228		0,506896	0,026043	0,191651	lncRNA	AL162258.	novel transcript, antisense to S100A1 and CHTOP	1
ENSG00000160679	675,6306	-0,00662	0,490559		-0,68942	0,025004	-0,04242	protein_coding	CHTOP	chromatin target of PRMT1 [Source:HGNC Symbol;Acc:HGNC:24511]	1
ENSG00000143553	250,2113	-0,00359	0,668279		-0,42851	0,024844	-0,031	protein_coding	SNAPIN	SNAP associated protein [Source:HGNC Symbol;Acc:HGNC:17145]	1
ENSG00000169418	13,6999	-0,00196	0,329612		-0,9749	0,026115	-0,33093	protein_coding	NPR1	natriuretic peptide receptor 1 [Source:HGNC Symbol;Acc:HGNC:7943]	1
ENSG00000143624	1283,873	-0,00211	0,854109		-0,18388	0,021389	-0,0068	protein_coding	INTS3	integrator complex subunit 3 [Source:HGNC Symbol;Acc:HGNC:26153]	1
ENSG00000143554	289,8652	0,004912	0,521005		0,641797	0,025458	0,052605	protein_coding	SLC27A3	solute carrier family 27 member 3 [Source:HGNC Symbol;Acc:HGNC:10997]	1
ENSG00000198837	900,2123	-0,07873	0,009613		-2,58943	0,104195	-0,15989	protein_coding	DENND4B	DENN domain containing 4B [Source:HGNC Symbol;Acc:HGNC:29044]	1
ENSG00000160741	671,9942	-0,00324	0,718069		-0,36104	0,023622	-0,01912	protein_coding	CRTC2	CREB regulated transcription coactivator 2 [Source:HGNC Symbol;Acc:HGNC:27301]	1
ENSG00000143570	19,02267	-0,00178	0,430972		-0,78753	0,026072	-0,23035	protein_coding	SLC39A1	solute carrier family 39 member 1 [Source:HGNC Symbol;Acc:HGNC:12876]	1
ENSG00000282386	10,72256	0,003118	0,087689		1,707714	0,026299	0,646078	lncRNA	AL358472.	novel transcript, antisense to SLC39A1	1

ENSG00000143578	133,3004	0,002967	0,5759	0,559384	0,025759	0,069313	protein_coding	CREB3L4	cAMP responsive element binding protein 3 like 4 [Source:HGNC Symbol;Acc:HGNC:18854]	1
ENSG00000143543	591,5308	0,006701	0,516934	0,648078	0,024814	0,037933	protein_coding	JTB	jumping translocation breakpoint [Source:HGNC Symbol;Acc:HGNC:6201]	1
ENSG00000285641	10,03024	0,000827	0,654121	0,448045	0,026029	0,155872	protein_coding	AL358472.	novel protein	1
ENSG00000272654	10,04234	-0,00015	0,915179	-0,10651	0,026041	-0,04864	lncRNA	AL358472.	novel transcript	1
ENSG00000143545	573,7136	0,009588	0,205134	1,267061	0,02738	0,110463	protein_coding	RAB13	RAB13, member RAS oncogene family [Source:HGNC Symbol;Acc:HGNC:9762]	1
ENSG00000143575	1021,185	-0,00085	0,934691	-0,08194	0,023349	-0,00428	protein_coding	HAX1	HCLS1 associated protein X-1 [Source:HGNC Symbol;Acc:HGNC:16915]	1
ENSG00000160712	201,9164	-0,01658	0,030645	-2,16165	0,033084	-0,21504	protein_coding	IL6R	interleukin 6 receptor [Source:HGNC Symbol;Acc:HGNC:6019]	1
ENSG00000169291	10,36383	-0,00015	0,917892	-0,10309	0,026038	-0,04501	protein_coding	SHE	Src homology 2 domain containing E [Source:HGNC Symbol;Acc:HGNC:27004]	1
ENSG00000160714	1554,762	3,46E-05	0,813131	0,236389	0,022497	0,01051	protein_coding	UBE2Q1	ubiquitin conjugating enzyme E2 Q1 [Source:HGNC Symbol;Acc:HGNC:15698]	1
ENSG00000286391	21,24767	0,002093	0,377602	0,882324	0,026127	0,284812	lncRNA	AL592078.	novel transcript	1
ENSG00000160716	765,4653	0,003344	0,701239	0,383648	0,024633	0,026281	protein_coding	CHRNB2	cholinergic receptor nicotinic beta 2 subunit [Source:HGNC Symbol;Acc:HGNC:1962]	1
ENSG00000287064	73,01248	0,000639	0,718553	0,360393	0,026036	0,140198	lncRNA	AL606500.	novel transcript	1
ENSG00000143603	129,2117	-0,00247	0,515011	-0,65105	0,025966	-0,11304	protein_coding	KCNN3	potassium calcium-activated channel subfamily N member 3 [Source:HGNC Symbol;Acc:HGNC:6292]	1
ENSG00000163344	261,2333	-0,00441	0,586925	-0,5433	0,025172	-0,04169	protein_coding	PMVK	phosphomevalonate kinase [Source:HGNC Symbol;Acc:HGNC:9141]	1
ENSG00000163346	1442,697	-0,01096	0,341921	-0,95038	0,024151	-0,03937	protein_coding	PBXIP1	PBX homeobox interacting protein 1 [Source:HGNC Symbol;Acc:HGNC:21199]	1
ENSG00000163348	729,9203	-0,01759	0,146789	-1,45097	0,03027	-0,07902	protein_coding	PYGO2	pygopus family PHD finger 2 [Source:HGNC Symbol;Acc:HGNC:30257]	1
ENSG00000271380	28,0285	-0,00069	0,792748	-0,26274	0,025959	-0,06601	lncRNA	AL451085.	uncharacterized LOC101928120 [Source:NCBI gene (formerly Entrezgene);Acc:101928120]	1
ENSG00000173207	300,8307	0,005585	0,415626	0,814032	0,026065	0,081612	protein_coding	CKS1B	CDC28 protein kinase regulatory subunit 1B [Source:HGNC Symbol;Acc:HGNC:19083]	1
ENSG00000160688	427,1621	-0,00584	0,501454	-0,6722	0,025121	-0,04521	protein_coding	FLAD1	flavin adenine dinucleotide synthetase 1 [Source:HGNC Symbol;Acc:HGNC:24671]	1
ENSG00000160685	764,0057	-0,02986	0,050987	-1,95159	0,047531	-0,1214	protein_coding	ZBTB7B	zinc finger and BTB domain containing 7B [Source:HGNC Symbol;Acc:HGNC:18668]	1
ENSG00000232093	9,48173	0,001113	0,41044	0,823119	0,026082	0,410478	lncRNA	DCST1-AS1	DCST1 antisense RNA 1 [Source:HGNC Symbol;Acc:HGNC:41147]	1
ENSG00000143537	785,0459	-0,01484	0,192026	-1,30461	0,028204	-0,07031	protein_coding	ADAM15	ADAM metallopeptidase domain 15 [Source:HGNC Symbol;Acc:HGNC:193]	1
ENSG00000243364	45,68984	-0,00162	0,662376	-0,43664	0,025918	-0,08353	protein_coding	EFNA4	ephrin A4 [Source:HGNC Symbol;Acc:HGNC:3224]	1
ENSG00000143590	40,48174	0,001148	0,690509	0,398164	0,025965	0,094216	protein_coding	EFNA3	ephrin A3 [Source:HGNC Symbol;Acc:HGNC:3223]	1
ENSG00000169242	145,5501	-6,9E-06	0,977333	0,028413	0,025346	0,003051	protein_coding	EFNA1	ephrin A1 [Source:HGNC Symbol;Acc:HGNC:3221]	1
ENSG00000169241	420,6817	-0,00151	0,861681	-0,17423	0,024401	-0,01181	protein_coding	SLC50A1	solute carrier family 50 member 1 [Source:HGNC Symbol;Acc:HGNC:30657]	1
ENSG00000179085	264,9696	0,005536	0,367705	0,90078	0,02605	0,090216	protein_coding	DPM3	dolichyl-phosphate mannosyltransferase subunit 3, regulatory [Source:HGNC Symbol;Acc:HGNC:3007]	1
ENSG00000163463	48,70631	-0,00082	0,818366	-0,22965	0,025847	-0,04238	protein_coding	KRTCAP2	keratinocyte associated protein 2 [Source:HGNC Symbol;Acc:HGNC:28942]	1
ENSG00000163462	53,72689	-0,00561	0,048945	-1,96907	0,026829	-0,46534	protein_coding	TRIM46	tripartite motif containing 46 [Source:HGNC Symbol;Acc:HGNC:19019]	1
ENSG00000185499	414,4307	0,002364	0,578176	0,556051	0,025877	0,085824	protein_coding	MUC1	mucin 1, cell surface associated [Source:HGNC Symbol;Acc:HGNC:7508]	1
ENSG00000231064	19,79067	0,000617	0,816435	0,232133	0,025954	0,058141	lncRNA	THBS3-AS1	THBS3 antisense RNA 1 [Source:HGNC Symbol;Acc:HGNC:40582]	1
ENSG00000169231	289,904	-0,00201	0,708473	-0,37391	0,025448	-0,0399	protein_coding	THBS3	thrombospondin 3 [Source:HGNC Symbol;Acc:HGNC:11787]	1
ENSG00000173171	201,5522	0,011738	0,133106	1,501964	0,028587	0,129725	protein_coding	MTX1	metaxin 1 [Source:HGNC Symbol;Acc:HGNC:7504]	1
ENSG00000160766	71,01333	-0,00423	0,328595	-0,97695	0,026176	-0,14339	transcribed_unpro	GBAP1	glucosylceramidase beta pseudogene 1 [Source:HGNC Symbol;Acc:HGNC:4178]	1
ENSG00000177628	1377,226	-0,00074	0,649784	0,454063	0,023326	0,023614	protein_coding	GBA	glucosylceramidase beta [Source:HGNC Symbol;Acc:HGNC:4177]	1
ENSG00000160767	433,7346	-0,01236	0,256988	-1,13354	0,027719	-0,07233	protein_coding	FAM189B	family with sequence similarity 189 member B [Source:HGNC Symbol;Acc:HGNC:1233]	1
ENSG00000116521	1070,048	0,00075	0,942656	0,071931	0,022836	0,003393	protein_coding	SCAMP3	secretory carrier membrane protein 3 [Source:HGNC Symbol;Acc:HGNC:10565]	1
ENSG00000176444	664,9586	-0,0054	0,527011	-0,63258	0,025192	-0,045	protein_coding	CLK2	CDC like kinase 2 [Source:HGNC Symbol;Acc:HGNC:2069]	1
ENSG00000143630	190,9362	-0,01218	0,058466	-1,89219	0,02948	-0,20453	protein_coding	HCN3	hyperpolarization activated cyclic nucleotide gated potassium channel 3 [Source:HGNC Symbol;Acc:HGNC:19183]	1
ENSG00000143627	91,15813	-0,00129	0,174625	-1,35749	0,026111	-0,90223	protein_coding	PKLR	pyruvate kinase L/R [Source:HGNC Symbol;Acc:HGNC:9020]	1
ENSG00000225855	35,81639	0,004698	0,149035	1,442947	0,026504	0,299029	lncRNA	RUSC1-AS1	RUSC1 antisense RNA 1 [Source:HGNC Symbol;Acc:HGNC:26680]	1
ENSG00000160753	609,6382	-0,005	0,597785	-0,52759	0,024387	-0,03095	protein_coding	RUSC1	RUN and SH3 domain containing 1 [Source:HGNC Symbol;Acc:HGNC:17153]	1
ENSG00000235919	22,95751	-0,0025	0,33754	-0,95904	0,026127	-0,2466	lncRNA	ASH1L-AS1	ASH1L antisense RNA 1 [Source:HGNC Symbol;Acc:HGNC:44146]	1
ENSG00000125459	156,7752	0,004094	0,509665	0,65936	0,025756	0,069163	protein_coding	MSTO1	misato mitochondrial distribution and morphology regulator 1 [Source:HGNC Symbol;Acc:HGNC:29678]	1
ENSG00000163374	1093,388	0,008613	0,490104	0,690143	0,024454	0,033586	protein_coding	YY1AP1	YY1 associated protein 1 [Source:HGNC Symbol;Acc:HGNC:30935]	1
ENSG00000132676	1191,466	0,032101	0,057181	1,901925	0,037009	0,076735	protein_coding	DAP3	death associated protein 3 [Source:HGNC Symbol;Acc:HGNC:2673]	1
ENSG00000203761	44,44149	-0,00649	0,094647	-1,67138	0,026992	-0,29366	unprocessed_pseu	MSTO2P	misato family member 2, pseudogene [Source:HGNC Symbol;Acc:HGNC:26329]	1
ENSG00000143622	773,2727	0,004761	0,662498	0,436467	0,023694	0,022313	protein_coding	RIT1	Ras like without CAAX 1 [Source:HGNC Symbol;Acc:HGNC:10023]	1
ENSG00000132680	700,5347	-0,03341	0,025842	-2,22858	0,054736	-0,13973	protein_coding	KHDC4	KH domain containing 4, pre-mRNA splicing factor [Source:HGNC Symbol;Acc:HGNC:29145]	1
ENSG00000207475	407,8591	-0,0007	0,79591	-0,25864	0,02595	-0,06274	snoRNA	SNORA80E	small nucleolar RNA, H/ACA box 80E [Source:HGNC Symbol;Acc:HGNC:32635]	1
ENSG00000252808	363,5355	0,000382	0,928767	0,089396	0,025957	0,022996				
ENSG00000160803	892,3217	0,001791	0,869838	0,163864	0,023019	0,007919	protein_coding	UBQLN4	ubiquilin 4 [Source:HGNC Symbol;Acc:HGNC:1237]	1

ENSG00000116586	373,5388	0,003765	0,652683		0,450038	0,024982	0,033727	protein_coding	LAMTOR2	late endosomal/lysosomal adaptor, MAPK and MTOR activator 2 [Source:HGNC Symbol;Acc:HGNC:29796]	1
ENSG00000132698	181,1267	-0,00078	0,885036		-0,14459	0,025507	-0,01747	protein_coding	RAB25	RAB25, member RAS oncogene family [Source:HGNC Symbol;Acc:HGNC:18238]	1
ENSG00000254726	317,4107	0,004514	0,481055		0,704607	0,02579	0,071345	protein_coding	MEX3A	mex-3 RNA binding family member A [Source:HGNC Symbol;Acc:HGNC:33482]	1
ENSG00000196189	22,34731	-0,00058	0,78276		-0,27572	0,026004	-0,08667	protein_coding	SEMA4A	semaphorin 4A [Source:HGNC Symbol;Acc:HGNC:10729]	1
ENSG00000160785	841,4095	-0,02316	0,141672		-1,46959	0,030761	-0,0601	protein_coding	SLC25A44	solute carrier family 25 member 44 [Source:HGNC Symbol;Acc:HGNC:29036]	1
ENSG00000160783	75,40778	-0,00058	0,904923		-0,11944	0,025615	-0,01605	protein_coding	PMF1	polyamine modulated factor 1 [Source:HGNC Symbol;Acc:HGNC:9112]	1
ENSG00000160781	50,53467	-0,00116	0,761458		-0,30357	0,025912	-0,06178	protein_coding	PAQR6	progestin and adipoQ receptor family member 6 [Source:HGNC Symbol;Acc:HGNC:30132]	1
ENSG00000163472	48,30537	-0,00353	0,392161		-0,85571	0,026107	-0,14053	protein_coding	TMEM79	transmembrane protein 79 [Source:HGNC Symbol;Acc:HGNC:28196]	1
ENSG00000198715	747,643	0,003852	0,729859		0,345313	0,023367	0,017075	protein_coding	GLMP	glycosylated lysosomal membrane protein [Source:HGNC Symbol;Acc:HGNC:29436]	1
ENSG00000125462	21,55088	0,001037	0,635205		0,474414	0,026021	0,146985	lncRNA	MIR9-1HG	MIR9-1 host gene [Source:HGNC Symbol;Acc:HGNC:30780]	1
ENSG00000183856	532,4642	0,003726	0,546793		0,602573	0,025669	0,062746	protein_coding	IQGAP3	IQ motif containing GTPase activating protein 3 [Source:HGNC Symbol;Acc:HGNC:20669]	1
ENSG00000163382	829,3454	-0,00289	0,775996		-0,28454	0,024074	-0,01694	protein_coding	NAXE	NAD(P)HX epimerase [Source:HGNC Symbol;Acc:HGNC:18453]	1
ENSG00000160818	737,6502	0,007058	0,508634		0,660966	0,024922	0,038669	protein_coding	GPATCH4	G-patch domain containing 4 [Source:HGNC Symbol;Acc:HGNC:25982]	1
ENSG00000272068	42,12998	-0,00082	0,78411		-0,27397	0,025925	-0,06102	lncRNA	AL365181.:	novel transcript	1
ENSG00000272405	176,3348	-0,00279	0,580817		-0,55219	0,025786	-0,07214	lncRNA	AL365181.:	novel transcript, antisense to BCAN	1
ENSG00000229953	97,46871	-0,00336	0,442856		-0,76738	0,02602	-0,11698	lncRNA	AL590666.:	novel transcript	1
ENSG00000143320	273,5915	0,001003	0,625398		0,488214	0,026028	0,156208	protein_coding	CRABP2	cellular retinoic acid binding protein 2 [Source:HGNC Symbol;Acc:HGNC:2339]	1
ENSG00000143319	780,5912	0,009367	0,360498		0,914417	0,024882	0,045622	protein_coding	ISG20L2	interferon stimulated exonuclease gene 20 like 2 [Source:HGNC Symbol;Acc:HGNC:25745]	1
ENSG00000143303	217,2636	-0,02058	0,03993		-2,05447	0,036214	-0,15623	protein_coding	RRNAD1	ribosomal RNA adenine dimethylase domain containing 1 [Source:HGNC Symbol;Acc:HGNC:24273]	1
ENSG00000143314	499,8835	0,026621	0,054044		1,926482	0,042443	0,121788	protein_coding	MRPL24	mitochondrial ribosomal protein L24 [Source:HGNC Symbol;Acc:HGNC:14037]	1
ENSG00000143294	1140,927	0,003316	0,831458		0,212832	0,02231	0,008876	protein_coding	PRCC	proline rich mitotic checkpoint control factor [Source:HGNC Symbol;Acc:HGNC:9343]	1
ENSG00000027869	19,86284	-0,00141	0,373176		-0,89054	0,026082	-0,34102	protein_coding	SH2D2A	SH2 domain containing 2A [Source:HGNC Symbol;Acc:HGNC:10821]	1
ENSG00000027644	18,88694	-0,0035	0,079188		-1,75542	0,026358	-0,60062	protein_coding	INSRR	insulin receptor related receptor [Source:HGNC Symbol;Acc:HGNC:6093]	1
ENSG00000187800	40,89371	-0,00201	0,434938		-0,78077	0,026059	-0,19482	protein_coding	PEAR1	platelet endothelial aggregation receptor 1 [Source:HGNC Symbol;Acc:HGNC:33631]	1
ENSG00000224520	20,27497	0,001809	0,385031		0,868664	0,026091	0,27722	processed_pseudog	KRT8P45	keratin 8 pseudogene 45 [Source:HGNC Symbol;Acc:HGNC:39879]	1
ENSG00000117036	944,0449	-0,01321	0,262638		-1,12018	0,026626	-0,05578	protein_coding	ETV3	ETS variant transcription factor 3 [Source:HGNC Symbol;Acc:HGNC:3492]	1
ENSG00000158473	18,01956	-0,0013	0,409493		-0,82479	0,026084	-0,36276	protein_coding	CD1D	CD1d molecule [Source:HGNC Symbol;Acc:HGNC:1637]	1
ENSG00000231434	16,04713	0,000769	0,680575		0,411678	0,026031	0,147447	processed_pseudog	HSP90AA3I	heat shock protein 90 alpha family class A member 3, pseudogene [Source:HGNC Symbol;Acc:HGNC:5254]	1
ENSG00000163565	1137,868	0,015946	0,064534		1,848475	0,031757	0,154567	protein_coding	IFI16	interferon gamma inducible protein 16 [Source:HGNC Symbol;Acc:HGNC:5395]	1
ENSG00000162706	38,17344	0,00166	0,44217		0,768535	0,02607	0,236802	protein_coding	CADM3	cell adhesion molecule 3 [Source:HGNC Symbol;Acc:HGNC:17601]	1
ENSG00000132703	93,17356	0,000225	0,871098		0,162264	0,02606	0,094421	protein_coding	APCS	amyloid P component, serum [Source:HGNC Symbol;Acc:HGNC:584]	1
ENSG00000132693	784,5124	9,39E-05	0,914702		0,10711	0,026079	0,134346	protein_coding	CRP	C-reactive protein [Source:HGNC Symbol;Acc:HGNC:2367]	1
ENSG00000158716	225,1958	-0,00435	0,479614		-0,70692	0,025813	-0,07413	protein_coding	DUSP23	dual specificity phosphatase 23 [Source:HGNC Symbol;Acc:HGNC:21480]	1
ENSG00000158714	55,55932	0,003408	0,23317		1,192233	0,026251	0,276162	protein_coding	SLAMF8	SLAM family member 8 [Source:HGNC Symbol;Acc:HGNC:21391]	1
ENSG00000272668	49,10868	0,00047	0,971228		0,036068	0,025908	0,007846	lncRNA	AL590560.:	uncharacterized LOC107985216 [Source:NCBI gene (formerly Entrezgene);Acc:107985216]	1
ENSG00000213085	40,80052	-0,00045	0,821018		-0,22624	0,025831	-0,04153	protein_coding	CFAP45	cilia and flagella associated protein 45 [Source:HGNC Symbol;Acc:HGNC:17229]	1
ENSG00000085552	179,3251	0,000524	0,846636		0,193412	0,025945	0,046909	protein_coding	IGSF9	immunoglobulin superfamily member 9 [Source:HGNC Symbol;Acc:HGNC:18132]	1
ENSG00000143315	394,9336	-0,00422	0,667422		-0,42969	0,024182	-0,02494	protein_coding	PIGM	phosphatidylinositol glycan anchor biosynthesis class M [Source:HGNC Symbol;Acc:HGNC:18858]	1
ENSG00000162728	79,93846	-8,7E-05	0,702787		-0,38156	0,026083	-1,39762	protein_coding	KCNJ9	potassium inwardly rectifying channel subfamily J member 9 [Source:HGNC Symbol;Acc:HGNC:6270]	1
ENSG00000162729	773,0944	-0,00717	0,349549		-0,93546	0,026234	-0,07904	protein_coding	IGSF8	immunoglobulin superfamily member 8 [Source:HGNC Symbol;Acc:HGNC:17813]	1
ENSG00000132716	468,2079	-0,00895	0,359515		-0,91629	0,025683	-0,0546	protein_coding	DCAF8	DDB1 and CUL4 associated factor 8 [Source:HGNC Symbol;Acc:HGNC:24891]	1
ENSG00000162735	475,785	0,00494	0,608599		0,512074	0,024744	0,033049	protein_coding	PEX19	peroxisomal biogenesis factor 19 [Source:HGNC Symbol;Acc:HGNC:9713]	1
ENSG00000162738	200,8063	-0,00562	0,392472		-0,85514	0,02619	-0,09215	protein_coding	VANGL2	VANGL planar cell polarity protein 2 [Source:HGNC Symbol;Acc:HGNC:15511]	1
ENSG00000066294	185,8406	0,00533	0,085846		1,717731	0,026701	0,362732	protein_coding	CD84	CD84 molecule [Source:HGNC Symbol;Acc:HGNC:1704]	1
ENSG00000117091	14,57932	0,001896	0,326923		0,980331	0,026114	0,347151	protein_coding	CD48	CD48 molecule [Source:HGNC Symbol;Acc:HGNC:1683]	1
ENSG00000026751	148,2532	0,004142	0,402666		0,836869	0,02611	0,116647	protein_coding	SLAMF7	SLAM family member 7 [Source:HGNC Symbol;Acc:HGNC:21394]	1
ENSG00000158769	580,1395	0,012008	0,064388		1,849487	0,029377	0,202559	protein_coding	F11R	F11 receptor [Source:HGNC Symbol;Acc:HGNC:14685]	1
ENSG00000215845	143,4477	0,000984	0,865581		0,169274	0,025628	0,022791	protein_coding	TSTD1	thiosulfate sulfurtransferase like domain containing 1 [Source:HGNC Symbol;Acc:HGNC:35410]	1
ENSG00000158773	528,0896	0,008496	0,379993		0,877909	0,025899	0,057208	protein_coding	USF1	upstream transcription factor 1 [Source:HGNC Symbol;Acc:HGNC:12593]	1
ENSG00000186517	47,00638	0,003076	0,224881		1,213652	0,026223	0,313633	protein_coding	ARHGAP30	Rho GTPase activating protein 30 [Source:HGNC Symbol;Acc:HGNC:27414]	1
ENSG00000143217	33,56222	0,001578	0,564603		0,576019	0,026063	0,178912	protein_coding	NECTIN4	nectin cell adhesion molecule 4 [Source:HGNC Symbol;Acc:HGNC:19688]	1
ENSG00000162755	24,62735	-0,00186	0,441934		-0,76893	0,026066	-0,21107	protein_coding	KLHDC9	kelch domain containing 9 [Source:HGNC Symbol;Acc:HGNC:28489]	1

ENSG00000143256	834,2456	0,04286	0,025585	2,232447	0,061759	0,118289	protein_coding	PFDN2	prefoldin subunit 2 [Source:HGNC Symbol;Acc:HGNC:8867]	1
ENSG00000158793	250,0758	0,007421	0,290817	1,05633	0,026452	0,093954	protein_coding	NIT1	nitrilase 1 [Source:HGNC Symbol;Acc:HGNC:7828]	1
ENSG00000158796	393,3007	0,004967	0,532174	0,624691	0,025294	0,047817	protein_coding	DEDD	death effector domain containing [Source:HGNC Symbol;Acc:HGNC:2755]	1
ENSG00000143222	763,4057	-0,00131	0,886923	-0,1422	0,023798	-0,00819	protein_coding	UFC1	ubiquitin-fold modifier conjugating enzyme 1 [Source:HGNC Symbol;Acc:HGNC:26941]	1
ENSG00000143258	249,5185	-0,00809	0,327353	-0,97946	0,026297	-0,0746	protein_coding	USP21	ubiquitin specific peptidase 21 [Source:HGNC Symbol;Acc:HGNC:12620]	1
ENSG00000143224	96,6055	-0,00116	0,886252	-0,14305	0,025387	-0,01543	protein_coding	PPOX	protoporphyrinogen oxidase [Source:HGNC Symbol;Acc:HGNC:9280]	1
ENSG00000158850	585,7298	-0,00418	0,677618	-0,41572	0,023944	-0,02289	protein_coding	B4GALT3	beta-1,4-galactosyltransferase 3 [Source:HGNC Symbol;Acc:HGNC:926]	1
ENSG00000158859	526,2429	0,004577	0,299226	1,038096	0,026226	0,146169	protein_coding	ADAMTS4	ADAM metallopeptidase with thrombospondin type 1 motif 4 [Source:HGNC Symbol;Acc:HGNC:220]	1
ENSG00000158864	1306,998	0,013653	0,301776	1,032632	0,025248	0,042603	protein_coding	NDUFS2	NADH:ubiquinone oxidoreductase core subunit S2 [Source:HGNC Symbol;Acc:HGNC:7708]	1
ENSG00000158869	121,872	0,00343	0,383056	0,872279	0,026101	0,14568	protein_coding	FCER1G	Fc fragment of IgE receptor Ig [Source:HGNC Symbol;Acc:HGNC:3611]	1
ENSG00000158874	281,3131	0,000385	0,533151	0,623203	0,026081	0,685233	protein_coding	APOA2	apolipoprotein A2 [Source:HGNC Symbol;Acc:HGNC:601]	1
ENSG00000158882	224,4679	-0,00152	0,795257	-0,25949	0,025377	-0,02724	protein_coding	TOMM40L	translocase of outer mitochondrial membrane 40 like [Source:HGNC Symbol;Acc:HGNC:25756]	1
ENSG00000158887	60,16091	-0,00202	0,620836	-0,49467	0,025868	-0,08029	protein_coding	MPZ	myelin protein zero [Source:HGNC Symbol;Acc:HGNC:7225]	1
ENSG00000143252	404,0886	0,011009	0,294704	1,047859	0,026263	0,058565	protein_coding	SDHC	succinate dehydrogenase complex subunit C [Source:HGNC Symbol;Acc:HGNC:10682]	1
ENSG00000288670	103,738	-0,01327	0,051382	-1,94828	0,03033	-0,21331	lncRNA	AL592295.[illegible]	novel transcript	1
ENSG00000288093	10,46741	-0,00027	0,878036	-0,15346	0,026022	-0,05736	lncRNA	AL592295.[illegible]	novel transcript	1
ENSG00000143226	72,14989	-0,00251	0,384367	-0,86988	0,026099	-0,20011	protein_coding	FCGR2A	Fc fragment of IgG receptor IIa [Source:HGNC Symbol;Acc:HGNC:3616]	1
ENSG00000173110	54,89861	-0,00147	0,436354	-0,77837	0,02608	-0,27897	protein_coding	HSPA6	heat shock protein family A (Hsp70) member 6 [Source:HGNC Symbol;Acc:HGNC:5239]	1
ENSG00000203747	79,8376	-0,00079	0,761395	-0,30365	0,025964	-0,07605	protein_coding	FCGR3A	Fc fragment of IgG receptor IIIa [Source:HGNC Symbol;Acc:HGNC:3619]	1
ENSG00000072694	14,45005	0,001086	0,391749	0,85645	0,026083	0,431963	protein_coding	FCGR2B	Fc fragment of IgG receptor IIb [Source:HGNC Symbol;Acc:HGNC:3618]	1
ENSG00000132185	17,83671	0,00412	0,158269	1,410916	0,026457	0,400167	protein_coding	FCRLA	Fc receptor like A [Source:HGNC Symbol;Acc:HGNC:18504]	1
ENSG00000162746	28,93131	-0,00183	0,535564	-0,61953	0,026005	-0,13968	protein_coding	FCRLB	Fc receptor like B [Source:HGNC Symbol;Acc:HGNC:26431]	1
ENSG00000081721	353,3143	0,005197	0,538655	0,614849	0,025297	0,046136	protein_coding	DUSP12	dual specificity phosphatase 12 [Source:HGNC Symbol;Acc:HGNC:3067]	1
ENSG00000162745	492,8942	0,001586	0,614947	0,503024	0,025957	0,105466	protein_coding	OLFML2B	olfactomedin like 2B [Source:HGNC Symbol;Acc:HGNC:24558]	1
ENSG00000198929	75,41363	-0,01113	0,02202	-2,29002	0,029266	-0,34363	protein_coding	NOS1AP	nitric oxide synthase 1 adaptor protein [Source:HGNC Symbol;Acc:HGNC:16859]	1
ENSG00000239887	57,33512	0,001634	0,644718	0,461112	0,02591	0,086413	protein_coding	C1orf226	chromosome 1 open reading frame 226 [Source:HGNC Symbol;Acc:HGNC:34351]	1
ENSG00000117143	905,1745	0,067128	0,015432	2,422085	0,072861	0,125627	protein_coding	UAP1	UDP-N-acetylglucosamine pyrophosphorylase 1 [Source:HGNC Symbol;Acc:HGNC:12457]	1
ENSG00000132196	111,4038	-0,01974	0,007333	-2,68132	0,037369	-0,29652	protein_coding	HSD17B7	hydroxysteroid 17-beta dehydrogenase 7 [Source:HGNC Symbol;Acc:HGNC:5215]	1
ENSG00000232995	16,18327	0,00152	0,460427	0,738144	0,026068	0,245977	lncRNA	RGS5	regulator of G protein signaling 5 [Source:NCBI gene (formerly Entrezgene);Acc:8490]	1
ENSG00000143228	335,0843	0,004864	0,225302	1,21255	0,026432	0,20138	protein_coding	NUF2	NUF2 component of NDC80 kinetochore complex [Source:HGNC Symbol;Acc:HGNC:14621]	1
ENSG00000185630	1128,738	-0,01274	0,248498	-1,15401	0,027538	-0,0683	protein_coding	PBX1	PBX homeobox 1 [Source:HGNC Symbol;Acc:HGNC:8632]	1
ENSG00000162761	11,25593	-0,00075	0,341809	-0,9506	0,026077	-0,5586	protein_coding	LMX1A	LIM homeobox transcription factor 1 alpha [Source:HGNC Symbol;Acc:HGNC:6653]	1
ENSG00000143198	766,6536	0,017963	0,12653	1,527931	0,030496	0,082625	protein_coding	MGST3	microsomal glutathione S-transferase 3 [Source:HGNC Symbol;Acc:HGNC:7064]	1
ENSG00000143149	990,0809	0,006707	0,524819	0,635935	0,024374	0,033948	protein_coding	ALDH9A1	aldehyde dehydrogenase 9 family member A1 [Source:HGNC Symbol;Acc:HGNC:412]	1
ENSG00000224358	15,9389	-0,00258	0,206852	-1,26227	0,026191	-0,41384	lncRNA	TMCO1-AS	TMCO1 antisense RNA 1 [Source:HGNC Symbol;Acc:HGNC:54046]	1
ENSG00000143179	1110,98	0,024668	0,085455	1,719877	0,034822	0,082275	protein_coding	UCK2	uridine-cytidine kinase 2 [Source:HGNC Symbol;Acc:HGNC:12562]	1
ENSG00000188859	77,05617	0,001582	0,63319	0,477241	0,025942	0,098226	protein_coding	FAM78B	family with sequence similarity 78 member B [Source:HGNC Symbol;Acc:HGNC:13495]	1
ENSG00000152382	160,2012	-0,00431	0,505291	-0,66619	0,025712	-0,06612	protein_coding	TADA1	transcriptional adaptor 1 [Source:HGNC Symbol;Acc:HGNC:30631]	1
ENSG00000143195	210,1591	0,001381	0,71905	0,359729	0,02584	0,061314	protein_coding	ILDR2	immunoglobulin like domain containing receptor 2 [Source:HGNC Symbol;Acc:HGNC:18131]	1
ENSG00000143167	19,6639	0,00071	0,679864	0,412649	0,026051	0,176853	protein_coding	GPA33	glycoprotein A33 [Source:HGNC Symbol;Acc:HGNC:4445]	1
ENSG00000198842	41,24259	4,9E-05	0,976185	-0,02985	0,02608	-0,04405	protein_coding	STYXL2	serine/threonine/tyrosine interacting like 2 [Source:HGNC Symbol;Acc:HGNC:25034]	1
ENSG00000143190	708,38	-0,00778	0,473722	-0,71644	0,025477	-0,04505	protein_coding	POU2F1	POU class 2 homeobox 1 [Source:HGNC Symbol;Acc:HGNC:9212]	1
ENSG00000198771	118,825	-0,00056	0,728283	-0,34741	0,025685	-0,05051	protein_coding	RCSD1	RCSD domain containing 1 [Source:HGNC Symbol;Acc:HGNC:28310]	1
ENSG00000143158	762,871	0,020763	0,118348	1,561746	0,032119	0,079118	protein_coding	MPC2	mitochondrial pyruvate carrier 2 [Source:HGNC Symbol;Acc:HGNC:24515]	1
ENSG00000143147	309,2023	0,003929	0,691684	0,396571	0,025634	0,039413	protein_coding	GPR161	G protein-coupled receptor 161 [Source:HGNC Symbol;Acc:HGNC:23694]	1
ENSG00000143155	1019,207	0,01549	0,173622	1,360658	0,028819	0,075046	protein_coding	TIPRL	TOR signaling pathway regulator [Source:HGNC Symbol;Acc:HGNC:30231]	1
ENSG00000143178	24,34928	-0,0019	0,512575	-0,65483	0,02602	-0,15116	protein_coding	TBX19	T-box transcription factor 19 [Source:HGNC Symbol;Acc:HGNC:11596]	1
ENSG00000227722	10,33844	-0,00041	0,79668	-0,25765	0,026043	-0,11344	transcribed_proces	AL022100.[illegible]	dynactin 4 pseudogene	1
ENSG00000143184	8,820479	0,000673	0,691197	0,397231	0,026065	0,204343	protein_coding	XCL1	X-C motif chemokine ligand 1 [Source:HGNC Symbol;Acc:HGNC:10645]	1
ENSG00000143156	295,5175	0,004421	0,585916	0,544764	0,025134	0,04124	protein_coding	NME7	NME/NM23 family member 7 [Source:HGNC Symbol;Acc:HGNC:20461]	1
ENSG00000117475	624,2677	0,029016	0,060053	1,880402	0,042932	0,103823	protein_coding	BLZF1	basic leucine zipper nuclear factor 1 [Source:HGNC Symbol;Acc:HGNC:1065]	1
ENSG00000117477	116,3116	-0,00316	0,469054	-0,72402	0,025983	-0,11027	protein_coding	CCDC181	coiled-coil domain containing 181 [Source:HGNC Symbol;Acc:HGNC:28051]	1

ENSG00000117479	252,2648	0,004403	0,499263	0,67565	0,025762	0,068142	protein_coding	SLC19A2	solute carrier family 19 member 2 [Source:HGNC Symbol;Acc:HGNC:10938]	1
ENSG00000213062	69,68505	-0,00265	0,395332	-0,84999	0,026119	-0,1961	lncRNA	Z99572.1	novel transcript	1
ENSG00000000460	186,9423	0,004879	0,392931	0,854315	0,026076	0,09843	protein_coding	C1orf112	chromosome 1 open reading frame 112 [Source:HGNC Symbol;Acc:HGNC:25565]	1
ENSG00000188404	12,22266	-0,00438	0,013504	-2,4702	0,026575	-0,94627	protein_coding	SELL	selectin L [Source:HGNC Symbol;Acc:HGNC:10720]	1
ENSG00000171806	98,16461	0,003629	0,312949	1,009054	0,025894	0,12649	protein_coding	METTL18	methyltransferase like 18 [Source:HGNC Symbol;Acc:HGNC:28793]	1
ENSG00000000457	239,7684	-0,00429	0,52275	-0,63911	0,025148	-0,0491	protein_coding	SCYL3	SCY1 like pseudokinase 3 [Source:HGNC Symbol;Acc:HGNC:19285]	1
ENSG00000075945	687,006	0,007299	0,464253	0,731862	0,025822	0,052912	protein_coding	KIFAP3	kinesin associated protein 3 [Source:HGNC Symbol;Acc:HGNC:17060]	1
ENSG00000120370	256,7747	0,001907	0,796098	0,2584	0,025011	0,0218	protein_coding	GORAB	golgin, RAB6 interacting [Source:HGNC Symbol;Acc:HGNC:25676]	1
ENSG00000271811	18,11034	0,001142	0,626484	0,486681	0,026016	0,141429	lncRNA	Z97200.1	novel transcript	1
ENSG00000007933	34,36073	0,001815	0,512703	0,654635	0,026026	0,157788	protein_coding	FMO3	flavin containing dimethylaniline monoxygenase 3 [Source:HGNC Symbol;Acc:HGNC:3771]	1
ENSG00000076258	21,51586	-0,00063	0,780959	-0,27807	0,025991	-0,08043	protein_coding	FMO4	flavin containing dimethylaniline monoxygenase 4 [Source:HGNC Symbol;Acc:HGNC:3772]	1
ENSG00000117533	493,1221	-0,00101	0,916719	-0,10457	0,023962	-0,00631	protein_coding	VAMP4	vesicle associated membrane protein 4 [Source:HGNC Symbol;Acc:HGNC:12645]	1
ENSG00000010165	666,329	-0,00503	0,637405	-0,47133	0,023879	-0,02473	protein_coding	EEF1AKNM	eEF1A lysine and N-terminal methyltransferase [Source:HGNC Symbol;Acc:HGNC:24248]	1
ENSG00000197959	142,2084	-0,00078	0,833222	-0,21057	0,025625	-0,02845	protein_coding	DNM3	dynamin 3 [Source:HGNC Symbol;Acc:HGNC:29125]	1
ENSG00000230630	335,5228	0,005935	0,319499	0,995488	0,026165	0,09928	lncRNA	DNM3OS	DNM3 opposite strand/antisense RNA [Source:HGNC Symbol;Acc:HGNC:41228]	1
ENSG00000135845	444,0484	0,020333	0,055197	1,917325	0,035468	0,138289	protein_coding	PIGC	phosphatidylinositol glycan anchor biosynthesis class C [Source:HGNC Symbol;Acc:HGNC:8960]	1
ENSG00000117560	14,01782	0,000195	0,94251	0,072115	0,02607	0,048695	protein_coding	FASLG	Fas ligand [Source:HGNC Symbol;Acc:HGNC:11936]	1
ENSG00000120337	20,31137	0,000855	0,689961	0,398908	0,026012	0,122924	protein_coding	TNFSF18	TNF superfamily member 18 [Source:HGNC Symbol;Acc:HGNC:11932]	1
ENSG00000117586	214,7415	0,005869	0,214714	1,240706	0,026608	0,176498	protein_coding	TNFSF4	TNF superfamily member 4 [Source:HGNC Symbol;Acc:HGNC:11934]	1
ENSG00000203739	52,86291	-0,00351	0,34135	-0,9515	0,026159	-0,17117	lncRNA	PRDX6-AS1	PRDX6 antisense RNA 1 [Source:HGNC Symbol;Acc:HGNC:54870]	1
ENSG00000076321	545,2701	0,007726	0,387988	0,863273	0,025921	0,061302	protein_coding	KLHL20	kelch like family member 20 [Source:HGNC Symbol;Acc:HGNC:25056]	1
ENSG00000120334	347,4111	0,000849	0,933823	0,083036	0,025183	0,007941	protein_coding	CENPL	centromere protein L [Source:HGNC Symbol;Acc:HGNC:17879]	1
ENSG00000117593	625,1381	0,015529	0,169732	1,373065	0,029206	0,079439	protein_coding	DARS2	aspartyl-tRNA synthetase 2, mitochondrial [Source:HGNC Symbol;Acc:HGNC:25538]	1
ENSG00000270084	9,761891	-0,0017	0,277185	-1,08666	0,026116	-0,45248	lncRNA	GAS5-AS1	GAS5 antisense RNA 1 [Source:HGNC Symbol;Acc:HGNC:44119]	1
ENSG00000185278	510,357	-0,00683	0,390122	-0,8594	0,025855	-0,06605	protein_coding	ZBTB37	zinc finger and BTB domain containing 37 [Source:HGNC Symbol;Acc:HGNC:28365]	1
ENSG00000135870	1447,153	0,003616	0,800845	0,252253	0,022387	0,010597	protein_coding	RC3H1	ring finger and CCCH-type domains 1 [Source:HGNC Symbol;Acc:HGNC:29434]	1
ENSG00000152061	1088,426	-0,00116	0,897104	-0,12932	0,022226	-0,00547	protein_coding	RABGAP1L	RAB GTPase activating protein 1 like [Source:HGNC Symbol;Acc:HGNC:24663]	1
ENSG00000287697	22,6315	0,000908	0,747386	0,322088	0,025953	0,077791	lncRNA	Z99127.3	novel transcript, antisense to CACYBP	1
ENSG00000120333	401,7662	0,015611	0,128783	1,518919	0,030223	0,100976	protein_coding	MRPS14	mitochondrial ribosomal protein S14 [Source:HGNC Symbol;Acc:HGNC:14049]	1
ENSG00000235750	746,9657	-0,09362	0,005582	-2,77137	0,06507	-0,14223	protein_coding	KIAA0040	KIAA0040 [Source:HGNC Symbol;Acc:HGNC:28950]	1
ENSG00000143207	1352,604	0,007883	0,590119	0,538663	0,024074	0,025625	protein_coding	COP1	COP1 E3 ubiquitin ligase [Source:HGNC Symbol;Acc:HGNC:17440]	1
ENSG00000252906	90,97582	-2,8E-06	0,997957	-0,00256	0,025919	-0,00059	scaRNA	SCARNA3	small Cajal body-specific RNA 3 [Source:HGNC Symbol;Acc:HGNC:32577]	1
ENSG00000236021	15,13177	-0,00032	0,862478	-0,17322	0,026017	-0,06229	lncRNA	AL591043.	novel transcript	1
ENSG00000116183	267,1902	-0,0012	0,763129	-0,30137	0,025797	-0,04867	protein_coding	PAPPA2	pappalysin 2 [Source:HGNC Symbol;Acc:HGNC:14615]	1
ENSG00000152092	75,12999	-0,00052	0,884358	-0,14545	0,025833	-0,02691	protein_coding	ASTN1	astrotactin 1 [Source:HGNC Symbol;Acc:HGNC:773]	1
ENSG00000198797	46,65776	-0,00335	0,220591	-1,22496	0,026251	-0,2944	protein_coding	BRINP2	BMP/retinoic acid inducible neural specific 2 [Source:HGNC Symbol;Acc:HGNC:13746]	1
ENSG00000242193	114,1506	-0,00527	0,339093	-0,95596	0,026255	-0,11596	transcribed_unitar	CRYZL2P	crystallin zeta like 2, pseudogene [Source:HGNC Symbol;Acc:HGNC:52164]	1
ENSG00000224687	20,61362	0,001185	0,662064	0,437065	0,02598	0,108419	lncRNA	RASAL2-AS	RASAL2 antisense RNA 1 [Source:HGNC Symbol;Acc:HGNC:44170]	1
ENSG00000213057	15,64626	0,000953	0,536611	0,617946	0,026061	0,26099	lncRNA	C1orf220	chromosome 1 open reading frame 220 [Source:HGNC Symbol;Acc:HGNC:33805]	1
ENSG00000116191	385,5007	0,002191	0,708073	0,374445	0,025298	0,036077	protein_coding	RALGPS2	Ral GEF with PH domain and SH3 binding motif 2 [Source:HGNC Symbol;Acc:HGNC:30279]	1
ENSG00000116194	114,5814	0,002399	0,512128	0,655528	0,025981	0,119258	protein_coding	ANGPTL1	angiopoietin like 1 [Source:HGNC Symbol;Acc:HGNC:489]	1
ENSG00000186283	533,6948	-0,00299	0,759773	-0,30578	0,024292	-0,01921	protein_coding	TOR3A	torsin family 3 member A [Source:HGNC Symbol;Acc:HGNC:11997]	1
ENSG00000057252	1422,857	0,022454	0,081074	1,744489	0,034594	0,094196	protein_coding	SOAT1	sterol O-acyltransferase 1 [Source:HGNC Symbol;Acc:HGNC:11177]	1
ENSG00000162782	11,55101	0,000376	0,661956	0,437214	0,026058	0,242774	protein_coding	TDRD5	tudor domain containing 5 [Source:HGNC Symbol;Acc:HGNC:20614]	1
ENSG00000143340	53,47562	0,000144	0,949964	0,062752	0,025979	0,018406	protein_coding	FAM163A	family with sequence similarity 163 member A [Source:HGNC Symbol;Acc:HGNC:28274]	1
ENSG00000272906	17,09696	-0,00223	0,333854	-0,96638	0,026121	-0,28214	lncRNA	AL353708.	novel transcript, antisense to TOR1AIP1	1
ENSG00000230124	798,3707	0,012822	0,277026	1,087025	0,025549	0,048512	protein_coding	ACBD6	acyl-CoA binding domain containing 6 [Source:HGNC Symbol;Acc:HGNC:23339]	1
ENSG00000143324	1472,714	0,012089	0,304766	1,026267	0,026032	0,051039	protein_coding	XPR1	xenotropic and polytropic retrovirus receptor 1 [Source:HGNC Symbol;Acc:HGNC:12827]	1
ENSG00000135835	102,0934	0,002134	0,678546	0,414448	0,025671	0,05244	protein_coding	KIAA1614	KIAA1614 [Source:HGNC Symbol;Acc:HGNC:29327]	1
ENSG00000243155	10,01397	-0,00149	0,349214	-0,93612	0,026096	-0,39283	lncRNA	AL162431.	novel transcript	1
ENSG00000232586	19,00402	-0,00014	0,935696	-0,08068	0,026025	-0,03142	lncRNA	KIAA1614-	KIAA1614 antisense RNA 1 [Source:HGNC Symbol;Acc:HGNC:50644]	1
ENSG00000135823	723,3813	-0,00531	0,619343	-0,49678	0,023822	-0,02546	protein_coding	STX6	syntaxin 6 [Source:HGNC Symbol;Acc:HGNC:11441]	1

ENSG00000153029	347,4316	-7E-05	0,991785	-0,0103	0,024937	-0,00087	protein_coding	MR1	major histocompatibility complex, class I-related [Source:HGNC Symbol;Acc:HGNC:4975]	1
ENSG00000162783	547,8985	0,007364	0,450306	0,754905	0,025254	0,046596	protein_coding	IER5	immediate early response 5 [Source:HGNC Symbol;Acc:HGNC:5393]	1
ENSG00000198216	35,14928	-0,00035	0,859637	-0,17684	0,026024	-0,06723	protein_coding	CACNA1E	calcium voltage-gated channel subunit alpha1 E [Source:HGNC Symbol;Acc:HGNC:1392]	1
ENSG00000135828	227,6423	0,003082	0,66474	0,433378	0,025253	0,0382	protein_coding	RNASEL	ribonuclease L [Source:HGNC Symbol;Acc:HGNC:10050]	1
ENSG00000143333	199,4413	-0,00067	0,824702	-0,2215	0,025909	-0,04759	protein_coding	RGS16	regulator of G protein signaling 16 [Source:HGNC Symbol;Acc:HGNC:9997]	1
ENSG00000135838	116,2899	0,005019	0,237419	1,181463	0,026435	0,185616	protein_coding	NPL	N-acetylneuraminate pyruvate lyase [Source:HGNC Symbol;Acc:HGNC:16781]	1
ENSG00000287808	9,63809	0,002447	0,147048	1,450038	0,026199	0,582997	lncRNA	AL355999.	novel transcript, antisense to DHX9	1
ENSG00000157064	506,6923	-0,00528	0,37623	-0,88486	0,026123	-0,09748	protein_coding	NMNAT2	nicotinamide nucleotide adenylyltransferase 2 [Source:HGNC Symbol;Acc:HGNC:16789]	1
ENSG00000287929	10,71502	-0,00064	0,672802	-0,42231	0,02605	-0,18836	lncRNA	AL354953.	novel transcript, antisense to NMNAT2	1
ENSG00000116701	156,6349	0,004872	0,183961	1,328656	0,026488	0,239933	protein_coding	NCF2	neutrophil cytosolic factor 2 [Source:HGNC Symbol;Acc:HGNC:7661]	1
ENSG00000143344	1489,651	-0,15041	0,000813	-3,34841	0,067918	-0,19625	protein_coding	RGL1	ral guanine nucleotide dissociation stimulator like 1 [Source:HGNC Symbol;Acc:HGNC:30281]	1
ENSG00000198756	19,63609	-0,00052	0,812721	-0,23692	0,026036	-0,09335	protein_coding	COLGALT2	collagen beta(1-O)galactosyltransferase 2 [Source:HGNC Symbol;Acc:HGNC:16790]	1
ENSG00000198860	372,5532	0,004187	0,555274	0,589876	0,024953	0,042578	protein_coding	TSEN15	tRNA splicing endonuclease subunit 15 [Source:HGNC Symbol;Acc:HGNC:16791]	1
ENSG00000116667	279,4316	0,004712	0,540552	0,611979	0,025351	0,048997	protein_coding	C1orf21	chromosome 1 open reading frame 21 [Source:HGNC Symbol;Acc:HGNC:15494]	1
ENSG00000135842	956,7895	0,092613	0,00814	2,646205	0,257087	0,20802	protein_coding	NIBAN1	niban apoptosis regulator 1 [Source:HGNC Symbol;Acc:HGNC:16784]	1
ENSG00000121481	441,4764	-0,00452	0,643661	-0,46259	0,024215	-0,02672	protein_coding	RNF2	ring finger protein 2 [Source:HGNC Symbol;Acc:HGNC:10061]	1
ENSG00000121486	482,9104	0,004568	0,620177	0,495599	0,02483	0,033615	protein_coding	TRMT1L	tRNA methyltransferase 1 like [Source:HGNC Symbol;Acc:HGNC:16782]	1
ENSG00000116668	142,9637	0,004299	0,52508	0,635535	0,025806	0,066986	protein_coding	SWT1	SWT1 RNA endoribonuclease homolog [Source:HGNC Symbol;Acc:HGNC:16785]	1
ENSG00000279838	50,33809	0,000869	0,717187	0,362221	0,025991	0,100544	TEC	AL356273.	TEC	1
ENSG00000116690	980,635	0,000809	0,832665	0,211285	0,025811	0,036375	protein_coding	PRG4	proteoglycan 4 [Source:HGNC Symbol;Acc:HGNC:9364]	1
ENSG00000157181	585,1574	-0,00197	0,854851	-0,18293	0,023199	-0,00914	protein_coding	ODR4	odr-4 GPCR localization factor homolog [Source:HGNC Symbol;Acc:HGNC:24299]	1
ENSG00000229739	13,32741	2,32E-05	0,991392	0,010788	0,026016	0,003915	lncRNA	PDC-AS1	PDC antisense RNA 1 [Source:HGNC Symbol;Acc:HGNC:40432]	1
ENSG00000273129	17,13171	0,001525	0,462453	0,734813	0,026066	0,240315	lncRNA	PACERR	PTGS2 antisense NFKB1 complex-mediated expression regulator RNA [Source:HGNC Symbol;Acc:HGNC:50552]	1
ENSG00000116711	900,7706	0,004226	0,552924	0,593385	0,025456	0,051975	protein_coding	PLA2G4A	phospholipase A2 group IVA [Source:HGNC Symbol;Acc:HGNC:9035]	1
ENSG00000230426	19,52411	0,000382	0,993735	0,007853	0,026028	0,002882	lncRNA	LINC01036	long intergenic non-protein coding RNA 1036 [Source:HGNC Symbol;Acc:HGNC:49024]	1
ENSG00000090104	158,1615	0,00012	0,982872	0,021468	0,025826	0,003928	protein_coding	RGS1	regulator of G protein signaling 1 [Source:HGNC Symbol;Acc:HGNC:9991]	1
ENSG00000231767	15,68177	0,00193	0,416947	0,811729	0,026079	0,230354	protein_coding	RPS27AP5	ribosomal protein S27a pseudogene 5 [Source:HGNC Symbol;Acc:HGNC:36867]	1
ENSG00000116741	1199,299	-0,00046	0,910527	-0,11237	0,025312	-0,01172	protein_coding	RGS2	regulator of G protein signaling 2 [Source:HGNC Symbol;Acc:HGNC:9998]	1
ENSG00000116750	731,6758	0,004948	0,592833	0,534734	0,024672	0,033814	protein_coding	UCHL5	ubiquitin C-terminal hydrolase L5 [Source:HGNC Symbol;Acc:HGNC:19678]	1
ENSG00000023572	391,3196	0,003838	0,652148	0,450781	0,024777	0,031432	protein_coding	GLRX2	glutaredoxin 2 [Source:HGNC Symbol;Acc:HGNC:16065]	1
ENSG00000134371	1297,436	0,014754	0,209064	1,256142	0,027476	0,062311	protein_coding	CDC73	cell division cycle 73 [Source:HGNC Symbol;Acc:HGNC:16783]	1
ENSG00000162630	128,5443	0,001708	0,684381	0,406492	0,025862	0,068947	protein_coding	B3GALT2	beta-1,3-galactosyltransferase 2 [Source:HGNC Symbol;Acc:HGNC:917]	1
ENSG00000162687	86,15506	-0,00182	0,582046	-0,5504	0,025957	-0,10932	protein_coding	KCNT2	potassium sodium-activated channel subfamily T member 2 [Source:HGNC Symbol;Acc:HGNC:18866]	1
ENSG00000116785	30,84517	0,001018	0,606726	0,514752	0,026036	0,175759	protein_coding	CFHR3	complement factor H related 3 [Source:HGNC Symbol;Acc:HGNC:16980]	1
ENSG00000244414	23,53242	0,000265	0,856645	0,180646	0,026041	0,079606	protein_coding	CFHR1	complement factor H related 1 [Source:HGNC Symbol;Acc:HGNC:4888]	1
ENSG00000143278	10,99073	0,000963	0,393493	0,8533	0,026081	0,45806	protein_coding	F13B	coagulation factor XIII B chain [Source:HGNC Symbol;Acc:HGNC:3534]	1
ENSG00000177888	1036,245	-0,00617	0,591855	-0,53615	0,02276	-0,02231	protein_coding	ZBTB41	zinc finger and BTB domain containing 41 [Source:HGNC Symbol;Acc:HGNC:24819]	1
ENSG00000213047	227,9354	0,012974	0,143015	1,464656	0,029068	0,114833	protein_coding	DENND1B	DENN domain containing 1B [Source:HGNC Symbol;Acc:HGNC:28404]	1
ENSG00000203724	25,41847	0,001417	0,66879	0,427809	0,026003	0,10897	protein_coding	C1orf53	chromosome 1 open reading frame 53 [Source:HGNC Symbol;Acc:HGNC:30003]	1
ENSG00000151414	1367,011	0,003826	0,63026	0,481361	0,024697	0,032713	protein_coding	NEK7	NIMA related kinase 7 [Source:HGNC Symbol;Acc:HGNC:13386]	1
ENSG00000081237	111,694	0,001667	0,599726	0,524795	0,025908	0,097206	protein_coding	PTPRC	protein tyrosine phosphatase receptor type C [Source:HGNC Symbol;Acc:HGNC:9666]	1
ENSG00000229989	75,97554	0,001125	0,726912	0,349236	0,025946	0,0787	lncRNA	MIR181A1I	MIR181A1 host gene [Source:HGNC Symbol;Acc:HGNC:48659]	1
ENSG00000116833	43,38341	0,00415	0,193792	1,299444	0,026394	0,287919	protein_coding	NR5A2	nuclear receptor subfamily 5 group A member 2 [Source:HGNC Symbol;Acc:HGNC:7984]	1
ENSG00000203721	15,8901	-0,00091	0,654813	-0,44709	0,026027	-0,14934	lncRNA	LINC00862	long intergenic non-protein coding RNA 862 [Source:HGNC Symbol;Acc:HGNC:21901]	1
ENSG00000162702	1263,528	0,109374	0,002413	3,03403	0,057869	0,148354	protein_coding	ZNF281	zinc finger protein 281 [Source:HGNC Symbol;Acc:HGNC:13075]	1
ENSG00000118193	631,7954	0,00356	0,49864	0,67663	0,025901	0,086569	protein_coding	KIF14	kinesin family member 14 [Source:HGNC Symbol;Acc:HGNC:19181]	1
ENSG00000118197	359,9071	0,008967	0,33936	0,955431	0,026192	0,063799	protein_coding	DDX59	DEAD-box helicase 59 [Source:HGNC Symbol;Acc:HGNC:25360]	1
ENSG00000163362	110,7474	0,003159	0,347167	0,940099	0,026162	0,193434	protein_coding	INAVA	innate immunity activator [Source:HGNC Symbol;Acc:HGNC:25599]	1
ENSG00000116852	1147,042	0,001436	0,834735	0,208633	0,025144	0,019197	protein_coding	KIF21B	kinesin family member 21B [Source:HGNC Symbol;Acc:HGNC:29442]	1
ENSG00000081248	511,685	-0,00025	0,626132	-0,48718	0,02608	-0,59471	protein_coding	CACNA1S	calcium voltage-gated channel subunit alpha1 S [Source:HGNC Symbol;Acc:HGNC:1397]	1
ENSG00000232237	559,0492	-0,00072	0,70656	-0,37648	0,026026	-0,13276	protein_coding	ASCL5	achaete-scute family bHLH transcription factor 5 [Source:HGNC Symbol;Acc:HGNC:33169]	1
ENSG00000116857	1244,938	0,007729	0,40745	0,82839	0,025593	0,053722	protein_coding	TMEM9	transmembrane protein 9 [Source:HGNC Symbol;Acc:HGNC:18823]	1

ENSG00000163395	474,7185	-0,00345	0,170844	-1,3695	0,026297	-0,36215	protein_coding	IGFN1	immunoglobulin like and fibronectin type III domain containing 1 [Source:HGNC Symbol;Acc:HGNC:24607]	1
ENSG00000081277	473,1839	0,001537	0,258121	1,130842	0,026111	0,515543	protein_coding	PKP1	plakophilin 1 [Source:HGNC Symbol;Acc:HGNC:9023]	1
ENSG00000159166	357,1383	-0,00048	0,870871	-0,16255	0,025632	-0,02224	protein_coding	LAD1	ladinin 1 [Source:HGNC Symbol;Acc:HGNC:6472]	1
ENSG00000282221	40,77378	-0,00344	0,351487	-0,93171	0,026199	-0,18839	lncRNA	AC119427.	novel transcript, antisense to LAD1	1
ENSG00000159173	10,50625	0,000229	0,803363	0,248997	0,02607	0,187744	protein_coding	TNNI1	troponin I1, slow skeletal type [Source:HGNC Symbol;Acc:HGNC:11945]	1
ENSG00000174307	531,3938	0,02258	0,05917	1,886929	0,036945	0,120312	protein_coding	PHLDA3	pleckstrin homology like domain family A member 3 [Source:HGNC Symbol;Acc:HGNC:8934]	1
ENSG00000224536	18,10391	-0,00063	0,771664	-0,2902	0,026001	-0,08923	lncRNA	AC096677.	novel transcript	1
ENSG00000223396	42,36395	-0,0032	0,348804	-0,93691	0,026093	-0,16657	transcribed_proces	RPS10P7	ribosomal protein S10 pseudogene 7 [Source:HGNC Symbol;Acc:HGNC:36423]	1
ENSG00000231871	17,05937	0,001931	0,387305	0,864515	0,026092	0,260438	lncRNA	IPO9-AS1	IPO9 antisense RNA 1 [Source:HGNC Symbol;Acc:HGNC:40892]	1
ENSG00000198892	273,899	0,000752	0,942812	0,071736	0,024887	0,005917	protein_coding	SHISA4	shisa family member 4 [Source:HGNC Symbol;Acc:HGNC:27139]	1
ENSG00000163431	111,3401	0,006558	0,113184	1,584045	0,026941	0,250497	protein_coding	LMOD1	leiomodin 1 [Source:HGNC Symbol;Acc:HGNC:6647]	1
ENSG00000134375	836,1891	0,016469	0,187506	1,317992	0,028372	0,064039	protein_coding	TIMM17A	translocase of inner mitochondrial membrane 17A [Source:HGNC Symbol;Acc:HGNC:17315]	1
ENSG00000176393	888,9771	-0,00415	0,67798	-0,41522	0,023642	-0,02149	protein_coding	RNPEP	arginyl aminopeptidase [Source:HGNC Symbol;Acc:HGNC:10078]	1
ENSG00000234678	91,09685	-0,00384	0,377863	-0,88184	0,02612	-0,13499	lncRNA	ELF3-AS1	ELF3 antisense RNA 1 [Source:HGNC Symbol;Acc:HGNC:40211]	1
ENSG00000163435	948,7327	-0,00622	0,318682	-0,99717	0,026345	-0,10481	protein_coding	ELF3	E74 like ETS transcription factor 3 [Source:HGNC Symbol;Acc:HGNC:3318]	1
ENSG00000170075	8,92504	-0,00048	0,642669	-0,46397	0,026068	-0,28851	protein_coding	GPR37L1	G protein-coupled receptor 37 like 1 [Source:HGNC Symbol;Acc:HGNC:14923]	1
ENSG00000143862	647,7904	-0,00658	0,514268	-0,65221	0,0242	-0,03389	protein_coding	ARL8A	ADP ribosylation factor like GTPase 8A [Source:HGNC Symbol;Acc:HGNC:25192]	1
ENSG00000143851	141,1551	0,002057	0,039076	2,063383	0,026184	1,35784	protein_coding	PTPN7	protein tyrosine phosphatase non-receptor type 7 [Source:HGNC Symbol;Acc:HGNC:9659]	1
ENSG00000133067	13,23949	-0,00044	0,701255	-0,38363	0,026062	-0,21609	protein_coding	LGR6	leucine rich repeat containing G protein-coupled receptor 6 [Source:HGNC Symbol;Acc:HGNC:19719]	1
ENSG00000077152	229,4818	0,009175	0,188823	1,314069	0,027396	0,125517	protein_coding	UBE2T	ubiquitin conjugating enzyme E2 T [Source:HGNC Symbol;Acc:HGNC:25009]	1
ENSG00000077157	439,8551	-0,00711	0,416876	-0,81185	0,025723	-0,05813	protein_coding	PPP1R12B	protein phosphatase 1 regulatory subunit 12B [Source:HGNC Symbol;Acc:HGNC:7619]	1
ENSG00000143858	34,52491	-0,00079	0,630807	-0,48059	0,026049	-0,20345	protein_coding	SYT2	synaptotagmin 2 [Source:HGNC Symbol;Acc:HGNC:11510]	1
ENSG00000228288	22,81961	-0,00133	0,738395	-0,33398	0,025976	-0,07906	lncRNA	PCAT6	prostate cancer associated transcript 6 [Source:HGNC Symbol;Acc:HGNC:43714]	1
ENSG00000214796	24,7905	-0,00455	0,066808	-1,83297	0,026557	-0,49989	transcribed_unpro	TUBA5P	tubulin alpha 5, pseudogene [Source:HGNC Symbol;Acc:HGNC:54799]	1
ENSG00000234996	10,90751	0,00064	0,622631	0,492125	0,026062	0,256721	transcribed_proces	ACTG1P25	actin gamma 1 pseudogene 25 [Source:HGNC Symbol;Acc:HGNC:54872]	1
ENSG00000183155	263,2452	-0,00927	0,241574	-1,17106	0,026929	-0,09251	protein_coding	RABIF	RAB interacting factor [Source:HGNC Symbol;Acc:HGNC:9797]	1
ENSG00000117153	589,5936	-0,00105	0,88845	-0,14027	0,024252	-0,00918	protein_coding	KLHL12	kelch like family member 12 [Source:HGNC Symbol;Acc:HGNC:19360]	1
ENSG00000159348	553,5198	-0,00209	0,823372	-0,22321	0,023552	-0,01198	protein_coding	CYB5R1	cytochrome b5 reductase 1 [Source:HGNC Symbol;Acc:HGNC:13397]	1
ENSG00000163444	657,7664	-0,00053	0,961641	-0,04809	0,023372	-0,00253	protein_coding	TMEM183/	transmembrane protein 183A [Source:HGNC Symbol;Acc:HGNC:20173]	1
ENSG00000143847	30,61688	0,001451	0,65205	0,450916	0,026008	0,115856	protein_coding	PPFIA4	PTPRF interacting protein alpha 4 [Source:HGNC Symbol;Acc:HGNC:9248]	1
ENSG00000163485	44,32794	-0,00126	0,66547	-0,43237	0,025959	-0,09817	protein_coding	ADORA1	adenosine A1 receptor [Source:HGNC Symbol;Acc:HGNC:262]	1
ENSG00000133048	332,2799	0,003859	0,05998	1,880938	0,026425	0,58509	protein_coding	CHI3L1	chitinase 3 like 1 [Source:HGNC Symbol;Acc:HGNC:1932]	1
ENSG00000133063	23,33984	0,001887	0,056212	1,909388	0,026164	1,156033	protein_coding	CHIT1	chitinase 1 [Source:HGNC Symbol;Acc:HGNC:1936]	1
ENSG00000159388	675,7187	-0,00138	0,832461	-0,21155	0,02524	-0,02056	protein_coding	BTG2	BTG anti-proliferation factor 2 [Source:HGNC Symbol;Acc:HGNC:1131]	1
ENSG00000122176	933,3919	-0,00109	0,73137	-0,3433	0,025893	-0,06738	protein_coding	FMOD	fibromodulin [Source:HGNC Symbol;Acc:HGNC:3774]	1
ENSG00000188783	36,03614	-0,00235	0,251686	-1,14626	0,026157	-0,36871	protein_coding	PRELP	proline and arginine rich end leucine rich repeat protein [Source:HGNC Symbol;Acc:HGNC:9357]	1
ENSG00000221643	14,10532	-0,00024	0,864652	-0,17045	0,026044	-0,07854	snoRNA	SNORA77	small nucleolar RNA, H/ACA box 77 [Source:HGNC Symbol;Acc:HGNC:32663]	1
ENSG00000058673	33,3626	-0,00203	0,531827	-0,62522	0,025991	-0,12817	protein_coding	ZC3H11A	zinc finger CCCH-type containing 11A [Source:HGNC Symbol;Acc:HGNC:29093]	1
ENSG00000257315	100,7564	-0,00096	0,802675	-0,24989	0,025637	-0,03376	protein_coding	ZBED6	zinc finger BED-type containing 6 [Source:HGNC Symbol;Acc:HGNC:33273]	1
ENSG00000182004	517,9419	0,071174	0,012128	2,508402	0,080425	0,137195	protein_coding	SNRPE	small nuclear ribonucleoprotein polypeptide E [Source:HGNC Symbol;Acc:HGNC:11161]	1
ENSG00000143842	507,039	-0,00445	0,592084	-0,53582	0,02501	-0,03869	protein_coding	SOX13	SRY-box transcription factor 13 [Source:HGNC Symbol;Acc:HGNC:11192]	1
ENSG00000143845	90,98251	-0,00258	0,589676	-0,53931	0,025809	-0,07427	protein_coding	ETNK2	ethanolamine kinase 2 [Source:HGNC Symbol;Acc:HGNC:25575]	1
ENSG00000174567	35,08149	0,00213	0,340153	0,953863	0,026104	0,268661	protein_coding	GOLT1A	golgi transport 1A [Source:HGNC Symbol;Acc:HGNC:24766]	1
ENSG00000143850	511,6584	-0,01708	0,064954	-1,84558	0,032325	-0,14032	protein_coding	PLEKHA6	pleckstrin homology domain containing A6 [Source:HGNC Symbol;Acc:HGNC:17053]	1
ENSG00000133056	276,718	-0,00086	0,88084	-0,1499	0,025469	-0,01735	protein_coding	PIK3C2B	phosphatidylinositol-4-phosphate 3-kinase catalytic subunit type 2 beta [Source:HGNC Symbol;Acc:HGNC:8972]	1
ENSG00000198625	797,6657	-0,00323	0,769241	-0,29337	0,024319	-0,01838	protein_coding	MDM4	MDM4 regulator of p53 [Source:HGNC Symbol;Acc:HGNC:6974]	1
ENSG00000170382	185,1777	-0,00162	0,641474	-0,46564	0,025803	-0,07303	protein_coding	LRRN2	leucine rich repeat neuronal 2 [Source:HGNC Symbol;Acc:HGNC:16914]	1
ENSG00000163531	1463,28	-0,0088	0,229938	-1,20052	0,025108	-0,06481	protein_coding	NFASC	neurofascin [Source:HGNC Symbol;Acc:HGNC:29866]	1
ENSG00000184144	9,591246	0,000256	0,873792	0,158844	0,026066	0,104896	protein_coding	CNTN2	contactin 2 [Source:HGNC Symbol;Acc:HGNC:2172]	1
ENSG00000174529	20,36159	-0,00271	0,284075	-1,07121	0,026167	-0,28391	protein_coding	TMEM81	transmembrane protein 81 [Source:HGNC Symbol;Acc:HGNC:32349]	1
ENSG00000117222	639,6527	0,001089	0,886682	0,142504	0,023926	0,008503	protein_coding	RBBP5	RB binding protein 5, histone lysine methyltransferase complex subunit [Source:HGNC Symbol;Acc:HGNC:9888]	1
ENSG00000133059	694,6496	-0,01028	0,446636	-0,76104	0,026229	-0,04501	protein_coding	DSTYK	dual serine/threonine and tyrosine protein kinase [Source:HGNC Symbol;Acc:HGNC:29043]	1

ENSG00000133069	157,8143	-0,00338	0,564775	-0,57576	0,025706	-0,06436	protein_coding	TMCC2	transmembrane and coiled-coil domain family 2 [Source:HGNC Symbol;Acc:HGNC:24239]	1
ENSG00000163545	171,4933	-0,0051	0,231419	-1,19671	0,026462	-0,19011	protein_coding	NUAK2	NUAK family kinase 2 [Source:HGNC Symbol;Acc:HGNC:29558]	1
ENSG00000162873	19,04137	-0,00055	0,795465	-0,25922	0,026003	-0,0818	protein_coding	KLHDC8A	kelch domain containing 8A [Source:HGNC Symbol;Acc:HGNC:25573]	1
ENSG00000235363	62,13347	0,001148	0,631283	0,479922	0,025827	0,082673	processed_pseudo	SNRPGP10	small nuclear ribonucleoprotein polypeptide G pseudogene 10 [Source:HGNC Symbol;Acc:HGNC:39329]	1
ENSG00000281406	54,89179	-0,00165	0,445602	-0,76277	0,026065	-0,23379	protein_coding	BLACAT1	bladder cancer associated transcript 1 [Source:HGNC Symbol;Acc:HGNC:48597]	1
ENSG00000117266	105,6788	-0,00806	0,0772	-1,76717	0,027554	-0,27091	protein_coding	CDK18	cyclin dependent kinase 18 [Source:HGNC Symbol;Acc:HGNC:8751]	1
ENSG00000158715	142,6813	-0,00114	0,81806	-0,23004	0,025571	-0,02876	protein_coding	SLC45A3	solute carrier family 45 member 3 [Source:HGNC Symbol;Acc:HGNC:8642]	1
ENSG00000117280	361,5027	-0,01959	0,048411	-1,97374	0,034991	-0,14954	protein_coding	RAB29	RAB29, member RAS oncogene family [Source:HGNC Symbol;Acc:HGNC:9789]	1
ENSG00000133065	702,1598	0,003102	0,774111	0,287002	0,023273	0,014217	protein_coding	SLC41A1	solute carrier family 41 member 1 [Source:HGNC Symbol;Acc:HGNC:19429]	1
ENSG00000286619	66,67577	-0,00227	0,585633	-0,54518	0,025883	-0,08691	lncRNA	AC119673.	novel transcript, antisense to PM20D1	1
ENSG00000229874	18,14731	0,001985	0,369949	0,896568	0,0261	0,274129				
ENSG00000196550	24,23424	0,000883	0,767493	0,295656	0,025932	0,066781	protein_coding	FAM72A	family with sequence similarity 72 member A [Source:HGNC Symbol;Acc:HGNC:24044]	1
ENSG00000163486	858,939	0,001762	0,810967	0,239178	0,024666	0,017643				
ENSG00000263528	260,2224	-0,01099	0,214173	-1,24217	0,027672	-0,09375	protein_coding	IKBKE	inhibitor of nuclear factor kappa B kinase subunit epsilon [Source:HGNC Symbol;Acc:HGNC:14552]	1
ENSG00000266094	172,6584	-0,00879	0,147737	-1,44757	0,027501	-0,16127	protein_coding	RASSF5	Ras association domain family member 5 [Source:HGNC Symbol;Acc:HGNC:17609]	1
ENSG00000143486	414,1596	-0,00256	0,824211	-0,22213	0,023921	-0,01285	protein_coding	EIF2D	eukaryotic translation initiation factor 2D [Source:HGNC Symbol;Acc:HGNC:6583]	1
ENSG00000143479	821,4562	0,001122	0,872061	0,161042	0,023724	0,009132	protein_coding	DYRK3	dual specificity tyrosine phosphorylation regulated kinase 3 [Source:HGNC Symbol;Acc:HGNC:3094]	1
ENSG00000162892	804,183	-0,00243	0,161349	-1,40055	0,026192	-0,52435	protein_coding	IL24	interleukin 24 [Source:HGNC Symbol;Acc:HGNC:11346]	1
ENSG00000162894	70,29441	-0,00207	0,606876	-0,51454	0,025926	-0,09144	protein_coding	FCMR	Fc fragment of IgM receptor [Source:HGNC Symbol;Acc:HGNC:14315]	1
ENSG00000162896	20,61163	0,003261	0,010884	2,546394	0,026358	1,289322	protein_coding	PIGR	polymeric immunoglobulin receptor [Source:HGNC Symbol;Acc:HGNC:8968]	1
ENSG00000182795	368,5358	-0,01171	0,1372	-1,4863	0,02886	-0,1418	protein_coding	C1orf116	chromosome 1 open reading frame 116 [Source:HGNC Symbol;Acc:HGNC:28667]	1
ENSG00000123836	581,6506	-0,00388	0,669161	-0,4273	0,024711	-0,02912	protein_coding	PFKFB2	6-phosphofructo-2-kinase/fructose-2,6-biphosphatase 2 [Source:HGNC Symbol;Acc:HGNC:8873]	1
ENSG00000180667	305,4011	0,003517	0,659585	0,440486	0,025081	0,034965	protein_coding	YOD1	YOD1 deubiquitinase [Source:HGNC Symbol;Acc:HGNC:25035]	1
ENSG00000123843	11,61552	-0,00017	0,909635	-0,1135	0,026043	-0,05182	protein_coding	C4BPB	complement component 4 binding protein beta [Source:HGNC Symbol;Acc:HGNC:1328]	1
ENSG00000123838	32,55384	-0,00131	0,063237	-1,85752	0,026121	-1,47654	protein_coding	C4BPA	complement component 4 binding protein alpha [Source:HGNC Symbol;Acc:HGNC:1325]	1
ENSG00000196352	1035,213	0,01985	0,0385	2,06949	0,035517	0,161211	protein_coding	CD55	CD55 molecule (Cromer blood group) [Source:HGNC Symbol;Acc:HGNC:2665]	1
ENSG00000203709	191,5193	-0,26956	0,000976	-3,29743	0,128562	-0,35347	lncRNA	MIR29B2CI	MIR29B2 and MIR29C host gene [Source:HGNC Symbol;Acc:HGNC:32018]	1
ENSG00000008118	39,15087	-0,00145	0,521234	-0,64144	0,02604	-0,18704	protein_coding	CAMK1G	calcium/calmodulin dependent protein kinase IG [Source:HGNC Symbol;Acc:HGNC:14585]	1
ENSG00000196878	971,4487	-0,00245	0,701431	-0,38339	0,025382	-0,03796	protein_coding	LAMB3	laminin subunit beta 3 [Source:HGNC Symbol;Acc:HGNC:6490]	1
ENSG00000123689	408,1856	-0,00378	0,471656	-0,71979	0,025912	-0,08894	protein_coding	G0S2	G0/G1 switch 2 [Source:HGNC Symbol;Acc:HGNC:30229]	1
ENSG00000117594	467,1586	-0,00572	0,28954	-1,05913	0,026401	-0,12954	protein_coding	HSD11B1	hydroxysteroid 11-beta dehydrogenase 1 [Source:HGNC Symbol;Acc:HGNC:5208]	1
ENSG00000162757	96,13469	0,010433	0,061577	1,869332	0,028555	0,229243	protein_coding	C1orf74	chromosome 1 open reading frame 74 [Source:HGNC Symbol;Acc:HGNC:26319]	1
ENSG00000117595	511,1235	-0,00316	0,50435	-0,66766	0,02582	-0,08512	protein_coding	IRF6	interferon regulatory factor 6 [Source:HGNC Symbol;Acc:HGNC:6121]	1
ENSG00000117597	693,3887	0,013079	0,185123	1,325144	0,028298	0,086602	protein_coding	UTP25	UTP25 small subunit processor component [Source:HGNC Symbol;Acc:HGNC:28440]	1
ENSG00000143469	147,9206	0,001729	0,599404	0,525257	0,025956	0,107581	protein_coding	SYT14	synaptotagmin 14 [Source:HGNC Symbol;Acc:HGNC:23143]	1
ENSG00000203706	22,9286	0,00071	0,71502	0,365122	0,026005	0,111068	lncRNA	SERTAD4-A	SERTAD4 antisense RNA 1 [Source:HGNC Symbol;Acc:HGNC:32019]	1
ENSG00000082497	61,38645	-0,00077	0,673325	-0,42159	0,025967	-0,10717	protein_coding	SERTAD4	SERTA domain containing 4 [Source:HGNC Symbol;Acc:HGNC:25236]	1
ENSG00000054392	63,55566	-0,00062	0,871559	-0,16168	0,025742	-0,02538	protein_coding	HHAT	hedgehog acyltransferase [Source:HGNC Symbol;Acc:HGNC:18270]	1
ENSG00000200972	11,8528	-0,00092	0,506342	-0,66454	0,026068	-0,30826	snRNA	RNU5A-8P	RNA, U5A small nuclear 8, pseudogene [Source:HGNC Symbol;Acc:HGNC:42530]	1
ENSG00000143473	36,04928	4,77E-05	0,91551	0,106091	0,026054	0,058331	protein_coding	KCNH1	potassium voltage-gated channel subfamily H member 1 [Source:HGNC Symbol;Acc:HGNC:6250]	1
ENSG00000117625	475,8857	0,000812	0,952603	0,059438	0,024028	0,003657	protein_coding	RCOR3	REST corepressor 3 [Source:HGNC Symbol;Acc:HGNC:25594]	1
ENSG00000082512	279,5905	0,014608	0,049522	1,964071	0,031138	0,191757	protein_coding	TRAF5	TNF receptor associated factor 5 [Source:HGNC Symbol;Acc:HGNC:12035]	1
ENSG00000153363	77,01859	-0,00101	0,750944	-0,31739	0,025808	-0,05365	lncRNA	LINC00467	long intergenic non-protein coding RNA 467 [Source:HGNC Symbol;Acc:HGNC:28227]	1
ENSG00000170385	1152,163	0,001493	0,901645	0,123584	0,021743	0,004839	protein_coding	SLC30A1	solute carrier family 30 member 1 [Source:HGNC Symbol;Acc:HGNC:11012]	1
ENSG00000117650	192,6189	0,005159	0,205464	1,266138	0,026508	0,208175	protein_coding	NEK2	NIMA related kinase 2 [Source:HGNC Symbol;Acc:HGNC:7745]	1
ENSG00000143493	532,7052	-0,01826	0,110354	-1,59661	0,031777	-0,09644	protein_coding	INTS7	integrator complex subunit 7 [Source:HGNC Symbol;Acc:HGNC:24484]	1
ENSG00000143476	293,9756	0,003013	0,625599	0,48793	0,025545	0,051067	protein_coding	DTL	denticleless E3 ubiquitin protein ligase homolog [Source:HGNC Symbol;Acc:HGNC:30288]	1
ENSG00000220749	17,76159	0,000544	0,811208	0,238868	0,025992	0,071527	processed_pseudo	RPL21P28	ribosomal protein L21 pseudogene 28 [Source:HGNC Symbol;Acc:HGNC:36258]	1
ENSG00000066027	421,5915	-0,00476	0,856557	-0,18076	0,024342	-0,01063	protein_coding	PPP2R5A	protein phosphatase 2 regulatory subunit B'alpha [Source:HGNC Symbol;Acc:HGNC:9309]	1
ENSG00000065600	176,4157	0,002157	0,740178	0,331618	0,025488	0,036118	protein_coding	PACC1	proton activated chloride channel 1 [Source:HGNC Symbol;Acc:HGNC:25593]	1
ENSG00000117691	672,8294	0,023674	0,06	1,880791	0,037438	0,112524	protein_coding	NENF	neudesin neurotrophic factor [Source:HGNC Symbol;Acc:HGNC:30384]	1
ENSG00000162772	483,533	-0,00456	0,283434	-1,07264	0,026264	-0,1589	protein_coding	ATF3	activating transcription factor 3 [Source:HGNC Symbol;Acc:HGNC:785]	1

ENSG00000123685	28,0153	0,002786	0,306482		1,022631	0,026161	0,254421	protein_coding	BATF3	basic leucine zipper ATF-like transcription factor 3 [Source:HGNC Symbol;Acc:HGNC:28915]	1
ENSG00000117697	487,4639	-0,00027	0,973036		-0,0338	0,023688	-0,00191	protein_coding	NSL1	NSL1 component of MIS12 kinetochore complex [Source:HGNC Symbol;Acc:HGNC:24548]	1
ENSG00000203705	180,9477	0,001867	0,759392		0,30628	0,02541	0,032202	protein_coding	TATDN3	TatD DNase domain containing 3 [Source:HGNC Symbol;Acc:HGNC:27010]	1
ENSG00000198468	62,53155	-0,00045	0,900953		-0,12446	0,025822	-0,02258	lncRNA	FLVCR1-DT	FLVCR1 divergent transcript [Source:HGNC Symbol;Acc:HGNC:39077]	1
ENSG00000162769	393,4389	-0,0027	0,758656		-0,30725	0,024505	-0,0208	protein_coding	FLVCR1	FLVCR heme transporter 1 [Source:HGNC Symbol;Acc:HGNC:24682]	1
ENSG00000143494	59,88195	-0,00036	0,904108		-0,12047	0,025885	-0,02512	protein_coding	VASH2	vasohibin 2 [Source:HGNC Symbol;Acc:HGNC:25723]	1
ENSG00000174606	434,352	-0,00198	0,77482		-0,28608	0,024146	-0,01781	protein_coding	ANGEL2	angel homolog 2 [Source:HGNC Symbol;Acc:HGNC:30534]	1
ENSG00000136643	856,2481	-0,00901	0,439684		-0,77273	0,024605	-0,03769	protein_coding	RPS6KC1	ribosomal protein S6 kinase C1 [Source:HGNC Symbol;Acc:HGNC:10439]	1
ENSG00000230461	130,8494	-0,00174	0,655753		-0,44578	0,025862	-0,07488	lncRNA	PROX1-AS1	PROX1 antisense RNA 1 [Source:HGNC Symbol;Acc:HGNC:43656]	1
ENSG00000274895	23,65621	0,00016	0,932312		0,084936	0,026017	0,031316	lncRNA	AC011700.	novel transcript, antisense to PROX1	1
ENSG00000143499	578,9871	0,005411	0,58192		0,550582	0,024785	0,034882	protein_coding	SMYD2	SET and MYND domain containing 2 [Source:HGNC Symbol;Acc:HGNC:20982]	1
ENSG00000082482	27,43692	0,001136	0,088374		1,704036	0,026111	1,516321	protein_coding	KCNK2	potassium two pore domain channel subfamily K member 2 [Source:HGNC Symbol;Acc:HGNC:6277]	1
ENSG00000136636	1111,032	0,009251	0,373328		0,890256	0,024995	0,045605	protein_coding	KCTD3	potassium channel tetramerization domain containing 3 [Source:HGNC Symbol;Acc:HGNC:21305]	1
ENSG00000042781	19,82559	-0,00159	0,265484		-1,11352	0,026115	-0,51498	protein_coding	USH2A	usherin [Source:HGNC Symbol;Acc:HGNC:12601]	1
ENSG00000196482	169,1706	-0,00049	0,912109		-0,11038	0,025807	-0,0194	protein_coding	ESRRG	estrogen related receptor gamma [Source:HGNC Symbol;Acc:HGNC:3474]	1
ENSG00000092978	488,1893	-0,00414	0,620111		-0,49569	0,024346	-0,03009	protein_coding	GPATCH2	G-patch domain containing 2 [Source:HGNC Symbol;Acc:HGNC:25499]	1
ENSG00000162814	10,42672	1,19E-05	0,893475		-0,13391	0,026027	-0,05304	protein_coding	SPATA17	spermatogenesis associated 17 [Source:HGNC Symbol;Acc:HGNC:25184]	1
ENSG00000067533	513,3105	0,058481	0,01697		2,387363	0,096569	0,14075	protein_coding	RRP15	ribosomal RNA processing 15 homolog [Source:HGNC Symbol;Acc:HGNC:24255]	1
ENSG00000092969	294,6658	0,010202	0,20677		1,262497	0,027559	0,104975	protein_coding	TGFB2	transforming growth factor beta 2 [Source:HGNC Symbol;Acc:HGNC:11768]	1
ENSG00000228063	24,04925	0,00251	0,339883		0,954397	0,026127	0,244196	lncRNA	LYPLAL1-D	LYPLAL1 divergent transcript [Source:HGNC Symbol;Acc:HGNC:50560]	1
ENSG00000143353	175,6909	0,011791	0,118332		1,561815	0,028771	0,141957	protein_coding	LYPLAL1	lysophospholipase like 1 [Source:HGNC Symbol;Acc:HGNC:20440]	1
ENSG00000215817	14,68875	0,000177	0,920732		0,099511	0,026024	0,038502	protein_coding	ZC3H11B	zinc finger CCCH-type containing 11B [Source:HGNC Symbol;Acc:HGNC:25659]	1
ENSG00000196660	28,67872	0,000852	0,598994		0,525847	0,026066	0,24901	protein_coding	SLC30A10	solute carrier family 30 member 10 [Source:HGNC Symbol;Acc:HGNC:25355]	1
ENSG00000162813	334,095	-0,00935	0,256142		-1,13556	0,026961	-0,08947	protein_coding	BPNT1	3'(2'), 5'-bisphosphate nucleotidase 1 [Source:HGNC Symbol;Acc:HGNC:1096]	1
ENSG00000118873	1302,258	-0,00223	0,797518		-0,25656	0,02164	-0,00983	protein_coding	RAB3GAP2	RAB3 GTPase activating non-catalytic protein subunit 2 [Source:HGNC Symbol;Acc:HGNC:17168]	1
ENSG00000222370	26,56241	-0,00035	0,84035		-0,20145	0,026017	-0,07039	snoRNA	SNORA36B	small nucleolar RNA, H/ACA box 36B [Source:HGNC Symbol;Acc:HGNC:32629]	1
ENSG00000218283	314,7247	0,012371	0,158529		1,410036	0,029067	0,12404	processed_pseudog	MORF4L1P	mortality factor 4 like 1 pseudogene 1 [Source:HGNC Symbol;Acc:HGNC:20400]	1
ENSG00000116141	533,5434	-0,02645	0,04172		-2,0363	0,042132	-0,12838	protein_coding	MARK1	microtubule affinity regulating kinase 1 [Source:HGNC Symbol;Acc:HGNC:6896]	1
ENSG00000162817	195,0865	0,004816	0,56345		0,577724	0,026026	0,064688	protein_coding	C1orf115	chromosome 1 open reading frame 115 [Source:HGNC Symbol;Acc:HGNC:25873]	1
ENSG00000117791	135,0464	0,003995	0,517774		0,64678	0,025731	0,067762	protein_coding	MTARC2	mitochondrial amidoxime reducing component 2 [Source:HGNC Symbol;Acc:HGNC:26064]	1
ENSG00000186205	48,35578	0,004595	0,236966		1,182603	0,026384	0,205263	protein_coding	MTARC1	mitochondrial amidoxime reducing component 1 [Source:HGNC Symbol;Acc:HGNC:26189]	1
ENSG00000136630	257,3878	0,002884	0,627727		0,484929	0,025134	0,040971	protein_coding	HLX	H2.0 like homeobox [Source:HGNC Symbol;Acc:HGNC:4978]	1
ENSG00000143507	465,2828	-0,00781	0,283403		-1,07271	0,026604	-0,09533	protein_coding	DUSP10	dual specificity phosphatase 10 [Source:HGNC Symbol;Acc:HGNC:3065]	1
ENSG00000238042	21,31252	0,000659	0,695138		0,391892	0,026039	0,152927	lncRNA	LINC02257	long intergenic non-protein coding RNA 2257 [Source:HGNC Symbol;Acc:HGNC:53159]	1
ENSG00000232679	117,2725	0,000813	0,78787		0,269077	0,025923	0,059542	lncRNA	LINC01705	long intergenic non-protein coding RNA 1705 [Source:HGNC Symbol;Acc:HGNC:52493]	1
ENSG00000143512	21,86152	0,001429	0,402909		0,836437	0,026087	0,344217	protein_coding	HHIPL2	HHIP like 2 [Source:HGNC Symbol;Acc:HGNC:25842]	1
ENSG00000143498	121,3964	-0,00779	0,112541		-1,58688	0,02732	-0,21778	protein_coding	TAF1A	TATA-box binding protein associated factor, RNA polymerase I subunit A [Source:HGNC Symbol;Acc:HGNC:11532]	1
ENSG00000272750	26,09779	0,004987	0,090187		1,69441	0,026618	0,384614	lncRNA	AL592148.:	novel transcript, antisense to MIA3	1
ENSG00000186063	1081,1	0,002273	0,744769		0,325544	0,024208	0,020457	protein_coding	AIDA	axin interactor, dorsalization associated [Source:HGNC Symbol;Acc:HGNC:25761]	1
ENSG00000197520	11,71743	0,001858	0,224242		1,215324	0,026136	0,545932	protein_coding	FAM177B	family with sequence similarity 177 member B [Source:HGNC Symbol;Acc:HGNC:34395]	1
ENSG00000154309	269,0558	-0,00225	0,741702		-0,3296	0,025032	-0,02763	protein_coding	DISP1	dispatched RND transporter family member 1 [Source:HGNC Symbol;Acc:HGNC:19711]	1
ENSG00000187554	11,26731	-0,00086	0,509372		-0,65982	0,026058	-0,28308	protein_coding	TLR5	toll like receptor 5 [Source:HGNC Symbol;Acc:HGNC:11851]	1
ENSG00000143502	68,44803	-0,00292	0,423828		-0,7998	0,026061	-0,14658	protein_coding	SUSD4	sushi domain containing 4 [Source:HGNC Symbol;Acc:HGNC:25470]	1
ENSG00000203697	243,6976	-0,00855	0,115383		-1,57445	0,027561	-0,19699	protein_coding	CAPN8	calpain 8 [Source:HGNC Symbol;Acc:HGNC:1485]	1
ENSG00000143514	1393,492	-0,00066	0,951098		-0,06133	0,023061	-0,00302	protein_coding	TP53BP2	tumor protein p53 binding protein 2 [Source:HGNC Symbol;Acc:HGNC:12000]	1
ENSG00000272645	40,56524	-0,00266	0,424307		-0,79897	0,026065	-0,16069	transcribed_unproc	GTF2IP20	general transcription factor IIi pseudogene 20 [Source:HGNC Symbol;Acc:HGNC:51732]	1
ENSG00000185495	60,62808	0,000362	0,919009		0,101683	0,025845	0,019323	transcribed_unproc	SEPTIN7P1	septin 7 pseudogene 13 [Source:HGNC Symbol;Acc:HGNC:55092]	1
ENSG00000278467	10,38758	-0,00366	0,032646		-2,13641	0,026411	-0,84261	lncRNA	AC138393.	novel transcript	1
ENSG00000143756	655,4257	0,003063	0,782615		0,275913	0,024216	0,01693	protein_coding	FBXO28	F-box protein 28 [Source:HGNC Symbol;Acc:HGNC:29046]	1
ENSG00000143753	1399,854	0,010645	0,341226		0,951746	0,025102	0,045466	protein_coding	DEGS1	delta 4-desaturase, sphingolipid 1 [Source:HGNC Symbol;Acc:HGNC:13709]	1
ENSG00000143748	379,8271	0,01448	0,120007		1,554746	0,02994	0,115421	protein_coding	NVL	nuclear VCP like [Source:HGNC Symbol;Acc:HGNC:8070]	1
ENSG00000143771	391,6634	0,005647	0,51934		0,644363	0,024988	0,042418	protein_coding	CNIH4	cornichon family AMPA receptor auxiliary protein 4 [Source:HGNC Symbol;Acc:HGNC:25013]	1

ENSG00000143786	363,0747	0,000941	0,872744		0,160174	0,025579	0,020425	protein_coding	CNIH3	cornichon family AMPA receptor auxiliary protein 3 [Source:HGNC Symbol;Acc:HGNC:26802]	1
ENSG00000233384	27,80965	0,000343	0,902454		0,122561	0,025936	0,029735	lncRNA	CNIH3-AS2	CNIH3 antisense RNA 2 [Source:HGNC Symbol;Acc:HGNC:41162]	1
ENSG00000185842	508,1831	-0,00349	0,683014		-0,40835	0,024747	-0,02877	protein_coding	DNAH14	dynein axonemal heavy chain 14 [Source:HGNC Symbol;Acc:HGNC:2945]	1
ENSG00000143815	580,1077	0,004537	0,613842		0,504597	0,024651	0,032488	protein_coding	LBR	lamin B receptor [Source:HGNC Symbol;Acc:HGNC:6518]	1
ENSG00000227496	10,48726	-0,00126	0,464838		-0,7309	0,026071	-0,28463	lncRNA	AC099066.	novel transcript	1
ENSG00000143819	1420,15	-0,0098	0,175277		-1,35544	0,027631	-0,12462	protein_coding	EPHX1	epoxide hydrolase 1 [Source:HGNC Symbol;Acc:HGNC:3401]	1
ENSG00000196187	500,459	-0,08811	0,007879		-2,65723	0,082333	-0,15301	protein_coding	TMEM63A	transmembrane protein 63A [Source:HGNC Symbol;Acc:HGNC:29118]	1
ENSG00000143811	313,402	0,001467	0,86793		0,166289	0,024343	0,011088	protein_coding	PYCR2	pyrroline-5-carboxylate reductase 2 [Source:HGNC Symbol;Acc:HGNC:30262]	1
ENSG00000143751	715,5523	-0,00068	0,947285		-0,06612	0,023415	-0,0035	protein_coding	SDE2	SDE2 telomere maintenance homolog [Source:HGNC Symbol;Acc:HGNC:26643]	1
ENSG00000163041	1197,225	0,019608	0,143844		1,461626	0,030993	0,073119	protein_coding	H3-3A	H3.3 histone A [Source:HGNC Symbol;Acc:HGNC:4764]	1
ENSG00000183814	146,0522	0,007343	0,191137		1,307223	0,026861	0,146593	protein_coding	LIN9	lin-9 DREAM MuvB core complex component [Source:HGNC Symbol;Acc:HGNC:30830]	1
ENSG00000203685	14,20041	-0,00123	0,537986		-0,61586	0,026048	-0,20829	protein_coding	STUM	stum, mechanosensory transduction mediator homolog [Source:HGNC Symbol;Acc:HGNC:30491]	1
ENSG00000143772	335,3513	-0,00959	0,289024		-1,06026	0,026928	-0,07984	protein_coding	ITPKB	inositol-trisphosphate 3-kinase B [Source:HGNC Symbol;Acc:HGNC:6179]	1
ENSG00000143801	38,23526	-0,00143	0,629386		-0,48259	0,025983	-0,1132	protein_coding	PSEN2	presenilin 2 [Source:HGNC Symbol;Acc:HGNC:9509]	1
ENSG00000163050	18,70893	-0,00015	0,951744		-0,06052	0,025958	-0,01608	protein_coding	COQ8A	coenzyme Q8A [Source:HGNC Symbol;Acc:HGNC:16812]	1
ENSG00000181450	157,5905	0,004014	0,507468		0,662786	0,025811	0,072923	protein_coding	ZNF678	zinc finger protein 678 [Source:HGNC Symbol;Acc:HGNC:28652]	1
ENSG00000143740	492,9884	-0,00788	0,44632		-0,76156	0,024986	-0,04265	protein_coding	SNAP47	synaptosome associated protein 47 [Source:HGNC Symbol;Acc:HGNC:30669]	1
ENSG00000081692	228,4643	-0,00233	0,743189		-0,32763	0,025078	-0,02791	protein_coding	JMJD4	jumonji domain containing 4 [Source:HGNC Symbol;Acc:HGNC:25724]	1
ENSG00000143816	128,2754	0,001522	0,73157		0,343037	0,025854	0,059047	protein_coding	WNT9A	Wnt family member 9A [Source:HGNC Symbol;Acc:HGNC:12778]	1
ENSG00000143793	238,5144	-0,0038	0,527708		-0,63151	0,025631	-0,06363	protein_coding	C1orf35	chromosome 1 open reading frame 35 [Source:HGNC Symbol;Acc:HGNC:19032]	1
ENSG00000162910	169,8447	-0,0036	0,588753		-0,54064	0,025498	-0,05162	protein_coding	MRPL55	mitochondrial ribosomal protein L55 [Source:HGNC Symbol;Acc:HGNC:16686]	1
ENSG00000181873	179,2749	-0,00187	0,764745		-0,29926	0,025243	-0,02841	protein_coding	IBA57	iron-sulfur cluster assembly factor IBA57 [Source:HGNC Symbol;Acc:HGNC:27302]	1
ENSG00000154358	207,1866	-0,00854	0,141113		-1,47166	0,027432	-0,16938	protein_coding	OBSCN	obscurin, cytoskeletal calmodulin and titin-interacting RhoGEF [Source:HGNC Symbol;Acc:HGNC:15719]	1
ENSG00000269934	33,17286	0,00199	0,49527		0,681951	0,02602	0,153032	lncRNA	AL353593.	novel transcript, antisense to OBSCN	1
ENSG00000154370	430,0716	-0,00976	0,327301		-0,97957	0,026274	-0,06139	protein_coding	TRIM11	tripartite motif containing 11 [Source:HGNC Symbol;Acc:HGNC:16281]	1
ENSG00000181218	150,9346	0,000357	0,922875		-0,09681	0,025309	-0,01011	protein_coding	H2AW	H2A.W histone [Source:HGNC Symbol;Acc:HGNC:20507]	1
ENSG00000196890	62,11751	0,001668	0,641686		0,465342	0,025864	0,079376	protein_coding	H2BU1	H2B.U histone 1 [Source:HGNC Symbol;Acc:HGNC:20514]	1
ENSG00000234975	112,5581	0,000546	0,914488		0,10738	0,025761	0,017397	processed_pseudog	FTH1P2	ferritin heavy chain 1 pseudogene 2 [Source:HGNC Symbol;Acc:HGNC:3989]	1
ENSG00000116574	511,576	0,000689	0,922606		0,097152	0,025525	0,011901	protein_coding	RHOU	ras homolog family member U [Source:HGNC Symbol;Acc:HGNC:17794]	1
ENSG00000177788	104,7611	-0,00351	0,49344		-0,68485	0,025915	-0,08975	lncRNA	AL162595.	novel transcript	1
ENSG00000168118	418,3352	0,0147	0,142554		1,466348	0,029495	0,097094	protein_coding	RAB4A	RAB4A, member RAS oncogene family [Source:HGNC Symbol;Acc:HGNC:9781]	1
ENSG00000154429	269,2128	0,008714	0,231757		1,195845	0,027064	0,109131	protein_coding	CCSAP	centriole, cilia and spindle associated protein [Source:HGNC Symbol;Acc:HGNC:29578]	1
ENSG00000069248	739,7529	-0,01265	0,278099		-1,0846	0,026471	-0,05503	protein_coding	NUP133	nucleoporin 133 [Source:HGNC Symbol;Acc:HGNC:18016]	1
ENSG00000135776	469,3153	-0,0005	0,94621		-0,06747	0,024888	-0,0056	protein_coding	ABCB10	ATP binding cassette subfamily B member 10 [Source:HGNC Symbol;Acc:HGNC:41]	1
ENSG00000135801	402,7982	-0,01257	0,214205		-1,24209	0,027721	-0,07731	protein_coding	TAF5L	TATA-box binding protein associated factor 5 like [Source:HGNC Symbol;Acc:HGNC:17304]	1
ENSG00000135763	393,4555	0,016702	0,132564		1,504066	0,030648	0,093407	protein_coding	URB2	URB2 ribosome biogenesis homolog [Source:HGNC Symbol;Acc:HGNC:28967]	1
ENSG00000177614	480,255	-0,01278	0,141211		-1,4713	0,028857	-0,11294	protein_coding	PGBD5	piggyBac transposable element derived 5 [Source:HGNC Symbol;Acc:HGNC:19405]	1
ENSG00000135775	614,3228	-0,00545	0,592999		-0,53449	0,024241	-0,02966	protein_coding	COG2	component of oligomeric golgi complex 2 [Source:HGNC Symbol;Acc:HGNC:6546]	1
ENSG00000135773	23,14066	0,001935	0,385686		0,867467	0,026094	0,264792	protein_coding	CAPN9	calpain 9 [Source:HGNC Symbol;Acc:HGNC:1486]	1
ENSG00000119280	1394,61	0,003403	0,714023		0,366459	0,024474	0,023826	protein_coding	C1orf198	chromosome 1 open reading frame 198 [Source:HGNC Symbol;Acc:HGNC:25900]	1
ENSG00000143643	399,988	-0,00268	0,768478		-0,29437	0,024599	-0,02052	protein_coding	TTC13	tetratricopeptide repeat domain 13 [Source:HGNC Symbol;Acc:HGNC:26204]	1
ENSG00000173409	227,7535	0,010095	0,197018		1,290093	0,027626	0,111806	protein_coding	ARV1	ARV1 homolog, fatty acid homeostasis modulator [Source:HGNC Symbol;Acc:HGNC:29561]	1
ENSG00000182118	77,76415	0,000146	0,963206		0,04613	0,025852	0,008949	protein_coding	FAM89A	family with sequence similarity 89 member A [Source:HGNC Symbol;Acc:HGNC:25057]	1
ENSG00000119283	56,99057	-0,00226	0,639159		-0,46887	0,026016	-0,09496	protein_coding	TRIM67	tripartite motif containing 67 [Source:HGNC Symbol;Acc:HGNC:31859]	1
ENSG00000143633	259,6345	0,004903	0,560983		0,581382	0,025235	0,043794	protein_coding	C1orf131	chromosome 1 open reading frame 131 [Source:HGNC Symbol;Acc:HGNC:25332]	1
ENSG00000116906	697,5883	0,005719	0,621606		0,493575	0,023405	0,023083	protein_coding	GNPAT	glyceronephosphate O-acyltransferase [Source:HGNC Symbol;Acc:HGNC:4416]	1
ENSG00000116903	682,0651	-0,00864	0,444362		-0,76485	0,024364	-0,03663	protein_coding	EXOC8	exocyst complex component 8 [Source:HGNC Symbol;Acc:HGNC:24659]	1
ENSG00000010072	323,6552	0,000481	0,835746		0,207338	0,024338	0,014019	protein_coding	SPRTN	SprT-like N-terminal domain [Source:HGNC Symbol;Acc:HGNC:25356]	1
ENSG00000135766	443,2783	-0,00609	0,397201		-0,84663	0,025991	-0,07567	protein_coding	EGLN1	egl-9 family hypoxia inducible factor 1 [Source:HGNC Symbol;Acc:HGNC:1232]	1
ENSG00000233461	69,97314	-0,00797	0,060722		-1,87552	0,027474	-0,27824	lncRNA	AL445524.	novel transcript	1
ENSG00000116918	457,2301	0,010336	0,305916		1,023829	0,026575	0,064621	protein_coding	TSNAX	translin associated factor X [Source:HGNC Symbol;Acc:HGNC:12380]	1
ENSG00000162946	164,2903	-0,0018	0,696745		-0,38972	0,02574	-0,05503	protein_coding	DISC1	DISC1 scaffold protein [Source:HGNC Symbol;Acc:HGNC:2888]	1

ENSG00000116991	916,6585	0,005032	0,616111		0,50137	0,025144	0,036174	protein_coding	SIPA1L2	signal induced proliferation associated 1 like 2 [Source:HGNC Symbol;Acc:HGNC:23800]	1
ENSG00000212916	83,95075	0,002688	0,620591		0,495013	0,025685	0,059617	protein_coding	MAP10	microtubule associated protein 10 [Source:HGNC Symbol;Acc:HGNC:29265]	1
ENSG00000135778	347,0463	-0,00025	0,979778		0,025347	0,024376	0,001734	protein_coding	NTPCR	nucleoside-triphosphatase, cancer-related [Source:HGNC Symbol;Acc:HGNC:28204]	1
ENSG00000135749	729,5427	-0,00309	0,723791		-0,3534	0,02427	-0,02197	protein_coding	PCNX2	pecanex 2 [Source:HGNC Symbol;Acc:HGNC:8736]	1
ENSG00000143674	198,267	-0,0157	0,025555		-2,2329	0,03242	-0,23779	protein_coding	MAP3K21	mitogen-activated protein kinase kinase kinase 21 [Source:HGNC Symbol;Acc:HGNC:29798]	1
ENSG00000135750	84,75466	-0,00303	0,518431		-0,64577	0,025895	-0,0904	protein_coding	KCNK1	potassium two pore domain channel subfamily K member 1 [Source:HGNC Symbol;Acc:HGNC:6272]	1
ENSG00000183780	47,77135	0,003074	0,33304		0,968009	0,026155	0,206465	protein_coding	SLC35F3	solute carrier family 35 member F3 [Source:HGNC Symbol;Acc:HGNC:23616]	1
ENSG00000168275	233,9374	0,00148	0,819919		0,22765	0,024986	0,01932	protein_coding	COA6	cytochrome c oxidase assembly factor 6 [Source:HGNC Symbol;Acc:HGNC:18025]	1
ENSG00000059588	354,8299	-0,00822	0,337315		-0,95948	0,026513	-0,07722	protein_coding	TARBP1	TAR (HIV-1) RNA binding protein 1 [Source:HGNC Symbol;Acc:HGNC:11568]	1
ENSG00000238005	12,27067	-0,00149	0,470971		-0,7209	0,026063	-0,23458	lncRNA	AL391832.:	novel transcript	1
ENSG00000173726	1535,989	0,005555	0,594836		0,531841	0,023725	0,026497	protein_coding	TOMM20	translocase of outer mitochondrial membrane 20 [Source:HGNC Symbol;Acc:HGNC:20947]	1
ENSG00000207181	305,5029	0,000196	0,890358		-0,13785	0,025981	-0,03992	snoRNA	SNORA14B	small nucleolar RNA, H/ACA box 14B [Source:HGNC Symbol;Acc:HGNC:32603]	1
ENSG00000188739	14,54455	7,98E-06	0,997976		0,002536	0,026001	0,000839	protein_coding	RBM34	RNA binding motif protein 34 [Source:HGNC Symbol;Acc:HGNC:28965]	1
ENSG00000054267	1449,035	0,010974	0,377548		0,882423	0,023967	0,035728	protein_coding	ARID4B	AT-rich interaction domain 4B [Source:HGNC Symbol;Acc:HGNC:15550]	1
ENSG00000152904	180,3976	0,008349	0,212036		1,247986	0,027124	0,126618	protein_coding	GGPS1	geranylgeranyl diphosphate synthase 1 [Source:HGNC Symbol;Acc:HGNC:4249]	1
ENSG00000285053	54,25067	0,000466	0,962853		0,046573	0,025742	0,007373	protein_coding	TBCE	tubulin folding cofactor E [Source:HGNC Symbol;Acc:HGNC:11582]	1
ENSG00000284770	24,80975	0,000284	0,901442		0,12384	0,025984	0,036187	protein_coding	TBCE	tubulin folding cofactor E [Source:HGNC Symbol;Acc:HGNC:11582]	1
ENSG00000162885	684,9506	-0,01115	0,253884		-1,14097	0,027072	-0,07321	protein_coding	B3GALNT2	beta-1,3-N-acetylgalactosaminyltransferase 2 [Source:HGNC Symbol;Acc:HGNC:28596]	1
ENSG00000168243	974,7525	0,002221	0,776342		0,284089	0,024869	0,022238	protein_coding	GNG4	G protein subunit gamma 4 [Source:HGNC Symbol;Acc:HGNC:4407]	1
ENSG00000235371	12,34094	1,03E-05	0,969055		-0,03879	0,026063	-0,02429	processed_pseudog	AL122018.	aconitase 2, mitochondrial (ACO2) pseudogene	1
ENSG00000077585	1012,451	-0,00917	0,324888		-0,98446	0,026326	-0,06636	protein_coding	GPR137B	G protein-coupled receptor 137B [Source:HGNC Symbol;Acc:HGNC:11862]	1
ENSG00000086619	274,7647	-0,00307	0,535699		-0,61933	0,025204	-0,05346	protein_coding	ERO1B	endoplasmic reticulum oxidoreductase 1 beta [Source:HGNC Symbol;Acc:HGNC:14355]	1
ENSG00000186197	154,7133	-0,0013	0,827202		-0,21829	0,025395	-0,02353	protein_coding	EDARADD	EDAR associated death domain [Source:HGNC Symbol;Acc:HGNC:14341]	1
ENSG00000223776	10,09923	-0,00075	0,674412		-0,4201	0,026035	-0,1553	lncRNA	LGALS8-AS	LGALS8 antisense RNA 1 [Source:HGNC Symbol;Acc:HGNC:40340]	1
ENSG00000119285	1176,137	0,02281	0,154647		1,423307	0,032195	0,064428	protein_coding	HEATR1	HEAT repeat containing 1 [Source:HGNC Symbol;Acc:HGNC:25517]	1
ENSG00000077522	57,91193	-0,00542	0,010534		-2,55778	0,026847	-0,84619	protein_coding	ACTN2	actinin alpha 2 [Source:HGNC Symbol;Acc:HGNC:164]	1
ENSG00000116984	1333,831	0,037175	0,042723		2,026408	0,042421	0,085536	protein_coding	MTR	5-methyltetrahydrofolate-homocysteine methyltransferase [Source:HGNC Symbol;Acc:HGNC:7468]	1
ENSG00000198626	86,77587	-0,00061	0,818961		-0,22888	0,025955	-0,05827	protein_coding	RYR2	ryanodine receptor 2 [Source:HGNC Symbol;Acc:HGNC:10484]	1
ENSG00000133019	72,58131	-0,00125	0,665678		-0,43209	0,025958	-0,09757	protein_coding	CHRM3	cholinergic receptor muscarinic 3 [Source:HGNC Symbol;Acc:HGNC:1952]	1
ENSG00000155816	128,0414	0,000417	0,816744		0,231735	0,026022	0,081683	protein_coding	FMN2	formin 2 [Source:HGNC Symbol;Acc:HGNC:14074]	1
ENSG00000180875	41,66443	0,001763	0,11855		1,560889	0,026145	0,848307	protein_coding	GREM2	gremlin 2, DAN family BMP antagonist [Source:HGNC Symbol;Acc:HGNC:17655]	1
ENSG00000182901	130,7368	0,006355	0,260397		1,125452	0,026694	0,14297	protein_coding	RGS7	regulator of G protein signaling 7 [Source:HGNC Symbol;Acc:HGNC:10003]	1
ENSG00000091483	688,7644	0,012443	0,214166		1,242191	0,02772	0,078434	protein_coding	FH	fumarate hydratase [Source:HGNC Symbol;Acc:HGNC:3700]	1
ENSG00000117009	26,15154	0,003479	0,083405		1,731264	0,026352	0,588438	protein_coding	KMO	kynurenine 3-monooxygenase [Source:HGNC Symbol;Acc:HGNC:6381]	1
ENSG00000054277	164,3168	0,001427	0,754813		0,3123	0,025429	0,034228	protein_coding	OPN3	opsin 3 [Source:HGNC Symbol;Acc:HGNC:14007]	1
ENSG00000203668	621,9666	0,013937	0,127075		1,525739	0,029551	0,113843	protein_coding	CHML	CHM like Rab escort protein [Source:HGNC Symbol;Acc:HGNC:1941]	1
ENSG00000174371	215,565	0,001855	0,755828		0,310964	0,025451	0,033855	protein_coding	EXO1	exonuclease 1 [Source:HGNC Symbol;Acc:HGNC:3511]	1
ENSG00000224625	20,67095	1,83E-06	0,992494		0,009407	0,026082	0,03457	unprocessed_pseu	TUBB8P6	tubulin beta 8 class VIII pseudogene 6 [Source:HGNC Symbol;Acc:HGNC:42343]	1
ENSG00000180287	97,25906	-0,0013	0,646882		-0,4581	0,025983	-0,11021	protein_coding	PLD5	phospholipase D family member 5 [Source:HGNC Symbol;Acc:HGNC:26879]	1
ENSG00000272865	27,61248	1,51E-05	0,960627		0,049367	0,026082	0,180875	lncRNA	AL591686.:	novel transcript, antisense to PLD5	1
ENSG00000214837	10,56147	-0,00171	0,22494		-1,2135	0,02612	-0,52125	transcribed_unpro	LINC01347	long intergenic non-protein coding RNA 1347 [Source:HGNC Symbol;Acc:HGNC:50566]	1
ENSG00000054282	1000,325	0,024672	0,106014		1,616372	0,030184	0,060695	protein_coding	SDCCAG8	SHH signaling and ciliogenesis regulator SDCCAG8 [Source:HGNC Symbol;Acc:HGNC:10671]	1
ENSG00000179456	616,3271	-0,00242	0,812041		-0,23779	0,02441	-0,01576	protein_coding	ZBTB18	zinc finger and BTB domain containing 18 [Source:HGNC Symbol;Acc:HGNC:13030]	1
ENSG00000035687	853,1216	0,003057	0,756387		0,310229	0,024188	0,018909	protein_coding	ADSS2	adenylosuccinate synthase 2 [Source:HGNC Symbol;Acc:HGNC:292]	1
ENSG00000179397	13,60256	-0,0021	0,254657		-1,13911	0,026141	-0,40619	protein_coding	CATSPERE	catsper channel auxiliary subunit epsilon [Source:HGNC Symbol;Acc:HGNC:28491]	1
ENSG00000121644	938,1977	-0,00941	0,448954		-0,75716	0,024008	-0,03311	protein_coding	DESI2	desumoylating isopeptidase 2 [Source:HGNC Symbol;Acc:HGNC:24264]	1
ENSG00000203667	723,9408	0,128309	0,002442		3,030455	0,072004	0,178509	protein_coding	COX20	cytochrome c oxidase assembly factor COX20 [Source:HGNC Symbol;Acc:HGNC:26970]	1
ENSG00000272195	13,51955	-0,00032	0,903712		-0,12097	0,025996	-0,03813	lncRNA	AL356512.	novel transcript, antisense to EFCAB2	1
ENSG00000203666	191,2918	-0,00075	0,955101		-0,0563	0,025195	-0,00543	protein_coding	EFCAB2	EF-hand calcium binding domain 2 [Source:HGNC Symbol;Acc:HGNC:28166]	1
ENSG00000162849	979,9581	0,011451	0,153529		1,427176	0,028315	0,119889	protein_coding	KIF26B	kinesin family member 26B [Source:HGNC Symbol;Acc:HGNC:25484]	1
ENSG00000185420	327,6558	-0,00557	0,406705		-0,82971	0,025604	-0,06726	protein_coding	SMYD3	SET and MYND domain containing 3 [Source:HGNC Symbol;Acc:HGNC:15513]	1
ENSG00000162851	250,9843	0,007814	0,33524		0,963613	0,026275	0,07576	protein_coding	TFB2M	transcription factor B2, mitochondrial [Source:HGNC Symbol;Acc:HGNC:18559]	1

ENSG00000162852	627,8499	-0,03063	0,051235	-1,9495	0,044123	-0,10369	protein_coding	CNST	consortin, connexin sorting protein [Source:HGNC Symbol;Acc:HGNC:26486]	1
ENSG00000143653	651,8514	0,022271	0,057217	1,901649	0,037797	0,134992	protein_coding	SCCPDH	saccharopine dehydrogenase (putative) [Source:HGNC Symbol;Acc:HGNC:24275]	1
ENSG00000260855	12,31276	0,000719	0,700523	0,384615	0,02603	0,138458	lncRNA	AL591848.:	novel transcript	1
ENSG00000227953	15,94411	-0,00146	0,461476	-0,73642	0,026065	-0,24496	lncRNA	LINC01341	long intergenic non-protein coding RNA 1341 [Source:HGNC Symbol;Acc:HGNC:49457]	1
ENSG00000197472	14,7349	0,002024	0,364215	0,907362	0,026133	0,327418	protein_coding	ZNF695	zinc finger protein 695 [Source:HGNC Symbol;Acc:HGNC:30954]	1
ENSG00000277462	42,99579	-0,00097	0,776835	-0,28345	0,025878	-0,05511	protein_coding	ZNF670	zinc finger protein 670 [Source:HGNC Symbol;Acc:HGNC:28167]	1
ENSG00000188295	119,3192	-0,00335	0,548582	-0,59989	0,025752	-0,06969	protein_coding	ZNF669	zinc finger protein 669 [Source:HGNC Symbol;Acc:HGNC:25736]	1
ENSG00000196418	361,8416	0,006971	0,590252	0,538471	0,025871	0,041023	protein_coding	ZNF124	zinc finger protein 124 [Source:HGNC Symbol;Acc:HGNC:12907]	1
ENSG00000259865	29,67077	-0,00187	0,529323	-0,62904	0,026007	-0,14117	lncRNA	AL390728.:	novel transcript	1
ENSG00000227671	296,3555	-0,00437	0,488896	-0,69207	0,025633	-0,06512	transcribed_unproc	ZNF731P	zinc finger protein 731, pseudogene [Source:HGNC Symbol;Acc:HGNC:32471]	1
ENSG00000162714	569,9963	0,000931	0,941536	0,07334	0,024396	0,005026	protein_coding	ZNF496	zinc finger protein 496 [Source:HGNC Symbol;Acc:HGNC:23713]	1
ENSG00000162711	27,90891	-0,00427	0,141828	-1,46902	0,026438	-0,34267	protein_coding	NLRP3	NLR family pyrin domain containing 3 [Source:HGNC Symbol;Acc:HGNC:16400]	1
ENSG00000162722	165,2508	-0,00145	0,671684	-0,42384	0,025912	-0,08281	protein_coding	TRIM58	tripartite motif containing 58 [Source:HGNC Symbol;Acc:HGNC:24150]	1
ENSG00000175137	871,6749	0,005791	0,556716	0,587726	0,024453	0,033546	protein_coding	SH3BP5L	SH3 binding domain protein 5 like [Source:HGNC Symbol;Acc:HGNC:29360]	1
ENSG00000171161	423,5435	-0,00147	0,842179	-0,19911	0,024372	-0,01341	protein_coding	ZNF672	zinc finger protein 672 [Source:HGNC Symbol;Acc:HGNC:26179]	1
ENSG00000171163	178,0002	-0,01568	0,025314	-2,23658	0,032371	-0,23539	protein_coding	ZNF692	zinc finger protein 692 [Source:HGNC Symbol;Acc:HGNC:26049]	1
ENSG00000185220	84,47207	0,004402	0,306869	1,021814	0,026234	0,152876	protein_coding	PGBD2	piggyBac transposable element derived 2 [Source:HGNC Symbol;Acc:HGNC:19399]	1
ENSG00000184731	124,0002	-0,00111	0,805084	-0,24677	0,02571	-0,03612	protein_coding	FAM110C	family with sequence similarity 110 member C [Source:HGNC Symbol;Acc:HGNC:33340]	2
ENSG00000035115	362,8194	-0,01305	0,145266	-1,45646	0,029007	-0,11095	protein_coding	SH3YL1	SH3 and SYLF domain containing 1 [Source:HGNC Symbol;Acc:HGNC:29546]	2
ENSG00000143727	1142,949	0,01355	0,301189	1,033887	0,024743	0,040691	protein_coding	ACP1	acid phosphatase 1 [Source:HGNC Symbol;Acc:HGNC:122]	2
ENSG00000151353	290,6017	1,71E-05	0,994964	0,006312	0,024414	0,000438	protein_coding	TMEM18	transmembrane protein 18 [Source:HGNC Symbol;Acc:HGNC:25257]	2
ENSG00000186487	382,6133	-0,00041	0,773592	-0,28768	0,026045	-0,12788	protein_coding	MYT1L	myelin transcription factor 1 like [Source:HGNC Symbol;Acc:HGNC:7623]	2
ENSG00000234929	18,5744	0,000282	0,946836	0,06668	0,026066	0,040608	lncRNA	AC018685.	novel transcript	2
ENSG00000237720	23,06333	0,002779	0,137597	1,4848	0,02625	0,584686	lncRNA	AC011995.	novel transcript	2
ENSG00000234423	10,63189	0,0008	0,551306	0,595804	0,026075	0,322204	lncRNA	LINC01250	long intergenic non-protein coding RNA 1250 [Source:HGNC Symbol;Acc:HGNC:49844]	2
ENSG00000032389	376,6312	0,025137	0,033915	2,121085	0,041736	0,147515	protein_coding	EIPR1	EARP complex and GARP complex interacting protein 1 [Source:HGNC Symbol;Acc:HGNC:12383]	2
ENSG00000171853	1039,004	0,005612	0,621551	0,493653	0,023494	0,023533	protein_coding	TRAPPC12	trafficking protein particle complex 12 [Source:HGNC Symbol;Acc:HGNC:24284]	2
ENSG00000182551	1523,208	0,013286	0,362006	0,91155	0,026129	0,042392	protein_coding	ADI1	acireductone dioxygenase 1 [Source:HGNC Symbol;Acc:HGNC:30576]	2
ENSG00000171865	506,1874	0,011672	0,270153	1,102709	0,026848	0,064356	protein_coding	RNASEH1	ribonuclease H1 [Source:HGNC Symbol;Acc:HGNC:18466]	2
ENSG00000234171	85,78651	0,002554	0,586127	0,544456	0,025913	0,08556	lncRNA	RNASEH1-/	RNASEH1 antisense RNA 1 [Source:HGNC Symbol;Acc:HGNC:49289]	2
ENSG00000287126	53,07224	0,00266	0,508246	0,661571	0,025977	0,112127	lncRNA	AC108488.	novel transcript	2
ENSG00000118004	270,4439	-0,00239	0,441137	-0,77027	0,026007	-0,14683	protein_coding	COLEC11	collectin subfamily member 11 [Source:HGNC Symbol;Acc:HGNC:17213]	2
ENSG00000151360	24,6813	-0,00034	0,837266	-0,20539	0,026035	-0,08394	protein_coding	ALLC	allantoicase [Source:HGNC Symbol;Acc:HGNC:17377]	2
ENSG00000176887	34,45773	0,000483	0,396532	0,847831	0,026082	0,761736	protein_coding	SOX11	SRY-box transcription factor 11 [Source:HGNC Symbol;Acc:HGNC:11191]	2
ENSG00000232044	17,61987	-0,00083	0,672501	-0,42272	0,026027	-0,14457	lncRNA	SILC1	sciatic injury induced lincRNA upregulator of SOX11 [Source:HGNC Symbol;Acc:HGNC:26403]	2
ENSG00000134326	131,4443	-0,00425	0,477199	-0,71081	0,025839	-0,07695	protein_coding	CMPK2	cytidine/uridine monophosphate kinase 2 [Source:HGNC Symbol;Acc:HGNC:27015]	2
ENSG00000134321	205,9666	-0,00855	0,141473	-1,47033	0,027813	-0,23172	protein_coding	RSAD2	radical S-adenosyl methionine domain containing 2 [Source:HGNC Symbol;Acc:HGNC:30908]	2
ENSG00000151692	240,7888	-0,00714	0,380945	-0,87616	0,025983	-0,06794	protein_coding	RNF144A	ring finger protein 144A [Source:HGNC Symbol;Acc:HGNC:20457]	2
ENSG00000236790	68,88673	0,001367	0,484556	0,698994	0,026061	0,240837	lncRNA	LINC00299	long intergenic non-protein coding RNA 299 [Source:HGNC Symbol;Acc:HGNC:27940]	2
ENSG00000236008	75,48566	-0,00077	0,55019	-0,59748	0,02607	-0,33508	lncRNA	LINC01814	long intergenic non-protein coding RNA 1814 [Source:HGNC Symbol;Acc:HGNC:52618]	2
ENSG00000235092	117,003	0,010119	0,043736	2,016612	0,028541	0,280432	lncRNA	ID2-AS1	ID2 antisense RNA 1 [Source:HGNC Symbol;Acc:HGNC:51103]	2
ENSG00000143797	868,4085	-0,00249	0,800082	-0,25324	0,023876	-0,01451	protein_coding	MBOAT2	membrane bound O-acyltransferase domain containing 2 [Source:HGNC Symbol;Acc:HGNC:25193]	2
ENSG00000119185	611,2542	0,018295	0,093015	1,679702	0,032036	0,104243	protein_coding	ITGB1BP1	integrin subunit beta 1 binding protein 1 [Source:HGNC Symbol;Acc:HGNC:23927]	2
ENSG00000119203	772,0816	0,000248	0,98747	0,015704	0,023899	0,000936	protein_coding	CPSF3	cleavage and polyadenylation specific factor 3 [Source:HGNC Symbol;Acc:HGNC:2326]	2
ENSG00000134330	297,1324	-0,00976	0,222646	-1,21952	0,027252	-0,09877	protein_coding	IAH1	isoamyl acetate hydrolyzing esterase 1 (putative) [Source:HGNC Symbol;Acc:HGNC:27696]	2
ENSG00000151694	881,3021	-0,01173	0,34436	-0,94559	0,025382	-0,04399	protein_coding	ADAM17	ADAM metallopeptidase domain 17 [Source:HGNC Symbol;Acc:HGNC:195]	2
ENSG00000115750	302,2783	0,006078	0,471395	0,720211	0,02555	0,054216	protein_coding	TAF1B	TATA-box binding protein associated factor, RNA polymerase I subunit B [Source:HGNC Symbol;Acc:HGNC:11533]	2
ENSG00000269973	49,53371	0,001054	0,790953	0,265073	0,025801	0,044132	lncRNA	AC010969.	novel transcript	2
ENSG00000134317	85,0783	-0,00247	0,588127	-0,54155	0,025839	-0,07889	protein_coding	GRHL1	grainyhead like transcription factor 1 [Source:HGNC Symbol;Acc:HGNC:17923]	2
ENSG00000260077	30,90102	-0,00219	0,415204	-0,81477	0,026077	-0,20232	lncRNA	AC104794.	novel transcript	2
ENSG00000172059	393,1822	0,006031	0,565578	0,574576	0,025229	0,039332	protein_coding	KLF11	Kruppel like factor 11 [Source:HGNC Symbol;Acc:HGNC:11811]	2
ENSG00000205795	10,33295	0,000129	0,925964	0,092924	0,026046	0,044459	protein_coding	CYS1	cystin 1 [Source:HGNC Symbol;Acc:HGNC:18525]	2

ENSG00000171848	1075,334	0,020075	0,041277	2,040732	0,035803	0,159188	protein_coding	RRM2	ribonucleotide reductase regulatory subunit M2 [Source:HGNC Symbol;Acc:HGNC:10452]	2
ENSG00000285872	8,693172	0,002698	0,099843	1,645615	0,02624	0,692029	lncRNA	AC007240.	novel transcript	2
ENSG00000115756	989,2289	-0,00281	0,761548	-0,30345	0,024254	-0,01898	protein_coding	HPCAL1	hippocalcin like 1 [Source:HGNC Symbol;Acc:HGNC:5145]	2
ENSG00000206633	66,27565	-0,00048	0,929867	0,088012	0,025974	0,024272	snoRNA	SNORA80B	small nucleolar RNA, H/ACA box 80B [Source:HGNC Symbol;Acc:HGNC:34355]	2
ENSG00000257135	39,04049	-0,00408	0,170779	-1,3697	0,026381	-0,30615	lncRNA	ODC1-DT	ODC1 divergent transcript [Source:HGNC Symbol;Acc:HGNC:54070]	2
ENSG00000115761	601,5009	0,013309	0,156863	1,415702	0,02874	0,097901	protein_coding	NOL10	nucleolar protein 10 [Source:HGNC Symbol;Acc:HGNC:25862]	2
ENSG00000143882	51,63159	0,004017	0,273845	1,094252	0,026281	0,20238	protein_coding	ATP6V1C2	ATPase H+ transporting V1 subunit C2 [Source:HGNC Symbol;Acc:HGNC:18264]	2
ENSG00000272275	10,71196	0,001373	0,416111	0,813187	0,026081	0,324317	lncRNA	AC092687.	novel transcript	2
ENSG00000162975	49,0077	-0,001	0,699794	-0,3856	0,025979	-0,09971	protein_coding	KCNF1	potassium voltage-gated channel modifier subfamily F member 1 [Source:HGNC Symbol;Acc:HGNC:6246]	2
ENSG00000162976	206,1605	0,015454	0,059138	1,887165	0,03193	0,185533	protein_coding	SLC66A3	solute carrier family 66 member 3 [Source:HGNC Symbol;Acc:HGNC:28503]	2
ENSG00000169016	272,9845	-0,01007	0,224373	-1,21498	0,027299	-0,09494	protein_coding	E2F6	E2F transcription factor 6 [Source:HGNC Symbol;Acc:HGNC:3120]	2
ENSG00000196208	132,7349	0,004257	0,474892	0,714543	0,025877	0,079437	protein_coding	GREB1	growth regulating estrogen receptor binding 1 [Source:HGNC Symbol;Acc:HGNC:24885]	2
ENSG00000239899	193,1693	0,001897	0,578065	0,556214	0,025955	0,108456	misc_RNA	RN7SL674F	RNA, 7SL, cytoplasmic 674, pseudogene [Source:HGNC Symbol;Acc:HGNC:46690]	2
ENSG00000286427	10,63235	0,000622	0,633443	0,476886	0,026043	0,19765	lncRNA	AC009486.	novel transcript	2
ENSG00000071575	1468,083	0,01145	0,31981	0,994848	0,025726	0,049348	protein_coding	TRIB2	tribbles pseudokinase 2 [Source:HGNC Symbol;Acc:HGNC:30809]	2
ENSG00000225649	19,99521	-0,00111	0,571137	-0,56638	0,026042	-0,19347	lncRNA	AC064875.	novel transcript	2
ENSG00000162981	604,1154	-0,00395	0,575276	-0,5603	0,025803	-0,06222	protein_coding	LRATD1	LRAT domain containing 1 [Source:HGNC Symbol;Acc:HGNC:20743]	2
ENSG00000134323	90,15401	0,002959	0,164562	1,389892	0,026247	0,451352	protein_coding	MYCN	MYCN proto-oncogene, bHLH transcription factor [Source:HGNC Symbol;Acc:HGNC:7559]	2
ENSG00000197872	125,8208	0,003817	0,501596	0,671981	0,025833	0,077781	protein_coding	CYRIA	CYFIP related Rac1 interactor A [Source:HGNC Symbol;Acc:HGNC:25373]	2
ENSG00000163032	65,84964	0,003646	0,201862	1,276265	0,026301	0,299726	protein_coding	VSNL1	visinin like 1 [Source:HGNC Symbol;Acc:HGNC:12722]	2
ENSG00000163029	1163,193	0,020829	0,126151	1,529458	0,030177	0,066983	protein_coding	SMC6	structural maintenance of chromosomes 6 [Source:HGNC Symbol;Acc:HGNC:20466]	2
ENSG00000178295	292,5836	0,003817	0,618098	0,498548	0,025192	0,04046	protein_coding	GEN1	GEN1 Holliday junction 5' flap endonuclease [Source:HGNC Symbol;Acc:HGNC:26881]	2
ENSG00000170745	241,2926	-0,0032	0,591593	-0,53653	0,025624	-0,0579	protein_coding	KCNS3	potassium voltage-gated channel modifier subfamily S member 3 [Source:HGNC Symbol;Acc:HGNC:6302]	2
ENSG00000240857	294,7643	0,00075	0,92828	0,09001	0,02475	0,007013	protein_coding	RDH14	retinol dehydrogenase 14 [Source:HGNC Symbol;Acc:HGNC:19979]	2
ENSG00000236204	10,0503	0,000592	0,715937	0,363894	0,026027	0,132805	lncRNA	LINC01376	long intergenic non-protein coding RNA 1376 [Source:HGNC Symbol;Acc:HGNC:50637]	2
ENSG00000143867	57,04506	-0,00114	0,741839	-0,32942	0,025882	-0,06327	protein_coding	OSR1	odd-skipped related transcription factor 1 [Source:HGNC Symbol;Acc:HGNC:8111]	2
ENSG00000228784	26,29115	-0,00136	0,545159	-0,60503	0,026035	-0,17679	lncRNA	LINC00954	long intergenic non-protein coding RNA 954 [Source:HGNC Symbol;Acc:HGNC:48668]	2
ENSG00000183891	87,00818	0,000475	0,931511	0,085944	0,025666	0,012265	protein_coding	TTC32	tetratricopeptide repeat domain 32 [Source:HGNC Symbol;Acc:HGNC:32954]	2
ENSG00000118965	479,0491	0,002592	0,765138	0,29874	0,024554	0,020602	protein_coding	WDR35	WD repeat domain 35 [Source:HGNC Symbol;Acc:HGNC:29250]	2
ENSG00000132031	23,36887	-0,00106	0,703245	-0,38094	0,026015	-0,11335	protein_coding	MATN3	matrilin 3 [Source:HGNC Symbol;Acc:HGNC:6909]	2
ENSG00000115884	916,1902	0,01029	0,070213	1,810531	0,028459	0,218281	protein_coding	SDC1	syndecan 1 [Source:HGNC Symbol;Acc:HGNC:10658]	2
ENSG00000143878	1117,263	0,067771	0,016655	2,394245	0,214287	0,167541	protein_coding	RHOB	ras homolog family member B [Source:HGNC Symbol;Acc:HGNC:668]	2
ENSG00000118960	485,0444	0,00089	0,920217	0,100161	0,024313	0,006685	protein_coding	HS1BP3	HCLS1 binding protein 3 [Source:HGNC Symbol;Acc:HGNC:24979]	2
ENSG00000143869	14,70267	-0,00085	0,601076	-0,52285	0,02605	-0,21141	protein_coding	GDF7	growth differentiation factor 7 [Source:HGNC Symbol;Acc:HGNC:4222]	2
ENSG00000118961	195,4825	0,006966	0,364515	0,906796	0,026117	0,075569	protein_coding	LDAH	lipid droplet associated hydrolase [Source:HGNC Symbol;Acc:HGNC:26145]	2
ENSG00000119771	334,8632	-0,00434	0,596391	-0,5296	0,025076	-0,0394	protein_coding	KLHL29	kelch like family member 29 [Source:HGNC Symbol;Acc:HGNC:29404]	2
ENSG00000119778	453,4757	-0,02547	0,034914	-2,10935	0,040507	-0,13173	protein_coding	ATAD2B	ATPase family AAA domain containing 2B [Source:HGNC Symbol;Acc:HGNC:29230]	2
ENSG00000173960	496,2331	-0,00113	0,907642	-0,11601	0,024147	-0,00735	protein_coding	UBXN2A	UBX domain protein 2A [Source:HGNC Symbol;Acc:HGNC:27265]	2
ENSG00000163026	127,9009	0,00483	0,412073	0,820252	0,025969	0,088349	protein_coding	WDCP	WD repeat and coiled coil containing [Source:HGNC Symbol;Acc:HGNC:26157]	2
ENSG00000119782	54,2567	0,000696	0,927031	0,091581	0,02574	0,014267	protein_coding	FKBP1B	FKBP prolyl isomerase 1B [Source:HGNC Symbol;Acc:HGNC:3712]	2
ENSG00000115128	806,8878	0,010781	0,257877	1,131422	0,027	0,074552	protein_coding	SF3B6	splicing factor 3b subunit 6 [Source:HGNC Symbol;Acc:HGNC:30096]	2
ENSG00000219626	83,58432	-0,00194	0,67888	-0,41399	0,025744	-0,05792	protein_coding	FAM228B	family with sequence similarity 228 member B [Source:HGNC Symbol;Acc:HGNC:24736]	2
ENSG00000115129	537,4656	0,014059	0,212062	1,247916	0,029947	0,099787	protein_coding	TP53I3	tumor protein p53 inducible protein 3 [Source:HGNC Symbol;Acc:HGNC:19373]	2
ENSG00000176732	18,60191	-0,00106	0,592606	-0,53506	0,026045	-0,189	protein_coding	PFN4	profilin family member 4 [Source:HGNC Symbol;Acc:HGNC:31103]	2
ENSG00000186453	11,443	-0,0007	0,639401	-0,46854	0,026052	-0,20462	protein_coding	FAM228A	family with sequence similarity 228 member A [Source:HGNC Symbol;Acc:HGNC:34418]	2
ENSG00000198399	1487,432	-0,00715	0,542595	-0,60889	0,023926	-0,02928	protein_coding	ITSN2	intersectin 2 [Source:HGNC Symbol;Acc:HGNC:6184]	2
ENSG00000084676	1480,705	0,008634	0,4623	0,735066	0,02355	0,031241	protein_coding	NCOA1	nuclear receptor coactivator 1 [Source:HGNC Symbol;Acc:HGNC:7668]	2
ENSG00000184924	203,3799	-0,00202	0,808348	-0,24256	0,025138	-0,0216	protein_coding	PTRHD1	peptidyl-tRNA hydrolase domain containing 1 [Source:HGNC Symbol;Acc:HGNC:33782]	2
ENSG00000138092	460,4173	0,011799	0,227781	1,206095	0,027566	0,079667	protein_coding	CENPO	centromere protein O [Source:HGNC Symbol;Acc:HGNC:28152]	2
ENSG00000138031	1311,237	-0,00697	0,61264	-0,50631	0,023126	-0,02164	protein_coding	ADCY3	adenylate cyclase 3 [Source:HGNC Symbol;Acc:HGNC:234]	2
ENSG00000271936	41,66989	0,000264	0,931083	0,086482	0,025894	0,018483	lncRNA	AC012073.	novel transcript, antisense to ADCY3	2
ENSG00000115137	99,94955	-0,00283	0,570816	-0,56685	0,0258	-0,07428	protein_coding	DNAJC27	DnaJ heat shock protein family (Hsp40) member C27 [Source:HGNC Symbol;Acc:HGNC:30290]	2

ENSG00000224165	9,668817	-0,00122	0,520808		-0,6421	0,026057	-0,23132	lncRNA	DNAJC27-A	DNAJC27 antisense RNA 1 [Source:HGNC Symbol;Acc:HGNC:42943]	2
ENSG00000084710	566,5652	-0,00067	0,931894		-0,08546	0,024737	-0,00663	protein_coding	EFR3B	EFR3 homolog B [Source:HGNC Symbol;Acc:HGNC:29155]	2
ENSG00000119772	824,847	-0,00114	0,921443		-0,09862	0,023743	-0,00561	protein_coding	DNMT3A	DNA methyltransferase 3 alpha [Source:HGNC Symbol;Acc:HGNC:2978]	2
ENSG00000235072	9,305527	0,000992	0,493159		0,685293	0,026071	0,320652	lncRNA	ARNILA	androgen receptor negatively regulated lncRNA [Source:HGNC Symbol;Acc:HGNC:54484]	2
ENSG00000138101	165,8544	-0,00309	0,621454		-0,49379	0,025527	-0,05057	protein_coding	DTNB	dystrobrevin beta [Source:HGNC Symbol;Acc:HGNC:3058]	2
ENSG00000084731	957,8479	0,009665	0,396787		0,847373	0,025609	0,04697	protein_coding	KIF3C	kinesin family member 3C [Source:HGNC Symbol;Acc:HGNC:6321]	2
ENSG00000157833	108,5133	0,006406	0,250533		1,149056	0,026598	0,137196	protein_coding	GAREM2	GRB2 associated regulator of MAPK1 subtype 2 [Source:HGNC Symbol;Acc:HGNC:27172]	2
ENSG00000138029	1324,956	0,003705	0,655962		0,445495	0,023618	0,023258	protein_coding	HADHB	hydroxyacyl-CoA dehydrogenase trifunctional multienzyme complex subunit beta [Source:HGNC Symbol;Acc:HGNC:480	2
ENSG00000173567	30,83237	-0,00367	0,152871		-1,42946	0,02634	-0,37965	protein_coding	ADGRF3	adhesion G protein-coupled receptor F3 [Source:HGNC Symbol;Acc:HGNC:18989]	2
ENSG00000138018	1004,712	0,004645	0,676254		0,41758	0,023153	0,019412	protein_coding	SELENOI	selenoprotein I [Source:HGNC Symbol;Acc:HGNC:29361]	2
ENSG00000115155	10,85868	0,000127	0,939607		0,075764	0,026028	0,030068	protein_coding	OTOF	otoferlin [Source:HGNC Symbol;Acc:HGNC:8515]	2
ENSG00000173557	31,72383	-0,00087	0,491092		-0,68857	0,025953	-0,14647	protein_coding	FAM166C	family with sequence similarity 166 member C [Source:HGNC Symbol;Acc:HGNC:27938]	2
ENSG00000171303	859,8668	-0,0013	0,724288		-0,35273	0,025856	-0,06267	protein_coding	KCNK3	potassium two pore domain channel subfamily K member 3 [Source:HGNC Symbol;Acc:HGNC:6278]	2
ENSG00000213699	1369,45	-0,01784	0,206406		-1,26351	0,026325	-0,04765	protein_coding	SLC35F6	solute carrier family 35 member F6 [Source:HGNC Symbol;Acc:HGNC:26055]	2
ENSG00000115163	113,9115	-0,00248	0,528164		-0,63081	0,025945	-0,10561	protein_coding	CENPA	centromere protein A [Source:HGNC Symbol;Acc:HGNC:1851]	2
ENSG00000157851	129,8334	-0,00135	0,463029		-0,73387	0,025994	-0,17202	protein_coding	DPYSL5	dihydropyrimidinase like 5 [Source:HGNC Symbol;Acc:HGNC:20637]	2
ENSG00000084764	532,3472	-0,03001	0,035198		-2,10607	0,046991	-0,12743	protein_coding	MAPRE3	microtubule associated protein RP/EB family member 3 [Source:HGNC Symbol;Acc:HGNC:6892]	2
ENSG00000084693	365,6285	-0,01381	0,237675		-1,18082	0,028479	-0,07315	protein_coding	AGBL5	ATP/GTP binding protein like 5 [Source:HGNC Symbol;Acc:HGNC:26147]	2
ENSG00000228474	1183,94	0,063483	0,013068		2,481905	0,055679	0,110237	protein_coding	OST4	oligosaccharyltransferase complex subunit 4, non-catalytic [Source:HGNC Symbol;Acc:HGNC:32483]	2
ENSG00000138030	118,7411	0,000423	0,697417		0,388809	0,025568	0,049952	protein_coding	KHK	ketohexokinase [Source:HGNC Symbol;Acc:HGNC:6315]	2
ENSG00000138028	127,9966	-0,00203	0,67431		-0,42024	0,025763	-0,05974	protein_coding	CGREF1	cell growth regulator with EF-hand domain 1 [Source:HGNC Symbol;Acc:HGNC:16962]	2
ENSG00000138073	1064,28	0,013759	0,274216		1,093404	0,027672	0,060152	protein_coding	PREB	prolactin regulatory element binding [Source:HGNC Symbol;Acc:HGNC:9356]	2
ENSG00000138074	405,3936	-0,0017	0,819403		-0,22831	0,024407	-0,01545	protein_coding	SLC5A6	solute carrier family 5 member 6 [Source:HGNC Symbol;Acc:HGNC:11041]	2
ENSG00000138085	765,1354	0,020778	0,12231		1,54515	0,032266	0,079081	protein_coding	ATRAID	all-trans retinoic acid induced differentiation factor [Source:HGNC Symbol;Acc:HGNC:24090]	2
ENSG00000084774	1403,915	0,005518	0,679756		0,412796	0,021978	0,015647	protein_coding	CAD	carbamoyl-phosphate synthetase 2, aspartate transcarbamylase, and dihydroorotase [Source:HGNC Symbol;Acc:HGNC:	2
ENSG00000115194	49,54742	-0,00059	0,736411		-0,33661	0,026031	-0,12703	protein_coding	SLC30A3	solute carrier family 30 member 3 [Source:HGNC Symbol;Acc:HGNC:11014]	2
ENSG00000138100	21,47643	-0,00066	0,776188		-0,28429	0,025988	-0,08148	protein_coding	TRIM54	tripartite motif containing 54 [Source:HGNC Symbol;Acc:HGNC:16008]	2
ENSG00000115204	399,0946	-0,0087	0,364928		-0,90602	0,025936	-0,05842	protein_coding	MPV17	mitochondrial inner membrane protein MPV17 [Source:HGNC Symbol;Acc:HGNC:7224]	2
ENSG00000115207	767,6331	0,00687	0,509585		0,659485	0,024193	0,033695	protein_coding	GTF3C2	general transcription factor IIIC subunit 2 [Source:HGNC Symbol;Acc:HGNC:4665]	2
ENSG00000234072	76,7533	-0,00125	0,550301		-0,59731	0,025774	-0,09355	lncRNA	AC074117.	novel transcript, antisense to GTF3C2 and EIF2B4	2
ENSG00000115211	594,6117	0,002127	0,838945		0,203243	0,023292	0,010316	protein_coding	EIF2B4	eukaryotic translation initiation factor 2B subunit delta [Source:HGNC Symbol;Acc:HGNC:3260]	2
ENSG00000115234	1168,371	0,011456	0,299694		1,037089	0,026455	0,057668	protein_coding	SNX17	sorting nexin 17 [Source:HGNC Symbol;Acc:HGNC:14979]	2
ENSG00000163795	215,368	0,002867	0,617182		0,499849	0,025531	0,052418	protein_coding	ZNF513	zinc finger protein 513 [Source:HGNC Symbol;Acc:HGNC:26498]	2
ENSG00000213453	13,72506	-0,0022	0,32524		-0,98375	0,026125	-0,29778	processed_pseudo	FTH1P3	ferritin heavy chain 1 pseudogene 3 [Source:HGNC Symbol;Acc:HGNC:3990]	2
ENSG00000157992	32,57766	0,002314	0,3746		0,887889	0,026103	0,229591	protein_coding	KRTCAP3	keratinocyte associated protein 3 [Source:HGNC Symbol;Acc:HGNC:28943]	2
ENSG00000138002	363,8877	0,007092	0,433561		0,783112	0,025576	0,053805	protein_coding	IFT172	intraflagellar transport 172 [Source:HGNC Symbol;Acc:HGNC:30391]	2
ENSG00000115226	215,8369	0,004457	0,663294		0,43537	0,02563	0,040489	protein_coding	FNDC4	fibronectin type III domain containing 4 [Source:HGNC Symbol;Acc:HGNC:20239]	2
ENSG00000084734	16,2474	0,001959	0,217914		1,232093	0,026142	0,50739	protein_coding	GCKR	glucokinase regulator [Source:HGNC Symbol;Acc:HGNC:4196]	2
ENSG00000243943	218,0321	0,007195	0,425031		0,797723	0,025949	0,060767	protein_coding	ZNF512	zinc finger protein 512 [Source:HGNC Symbol;Acc:HGNC:29380]	2
ENSG00000176714	33,43872	0,000981	0,758359		0,307636	0,025911	0,064664	protein_coding	CCDC121	coiled-coil domain containing 121 [Source:HGNC Symbol;Acc:HGNC:25833]	2
ENSG00000198522	295,8793	0,006195	0,458429		0,741435	0,025644	0,056989	protein_coding	GPN1	GPN-loop GTPase 1 [Source:HGNC Symbol;Acc:HGNC:17030]	2
ENSG00000119760	897,1221	-0,00536	0,670514		-0,42544	0,022299	-0,01694	protein_coding	SUPT7L	SPT7 like, STAGA complex subunit gamma [Source:HGNC Symbol;Acc:HGNC:30632]	2
ENSG00000163798	853,518	-0,01136	0,487318		-0,69458	0,025715	-0,03465	protein_coding	SLC4A1AP	solute carrier family 4 member 1 adaptor protein [Source:HGNC Symbol;Acc:HGNC:13813]	2
ENSG00000243147	355,1773	0,008288	0,300163		1,036084	0,026547	0,08355	protein_coding	MRPL33	mitochondrial ribosomal protein L33 [Source:HGNC Symbol;Acc:HGNC:14487]	2
ENSG00000171174	18,15612	-0,00437	0,092855		-1,68053	0,026503	-0,44454	protein_coding	RBKS	ribokinase [Source:HGNC Symbol;Acc:HGNC:30325]	2
ENSG00000158019	162,2553	0,000915	0,882475		0,147833	0,025087	0,013403	protein_coding	BABAM2	BRISC and BRCA1 A complex member 2 [Source:HGNC Symbol;Acc:HGNC:1106]	2
ENSG00000075426	1448,259	0,001748	0,829971		0,214739	0,022015	0,008745	protein_coding	FOSL2	FOS like 2, AP-1 transcription factor subunit [Source:HGNC Symbol;Acc:HGNC:3798]	2
ENSG00000163803	45,35899	-0,0018	0,555194		-0,58999	0,025993	-0,13041	protein_coding	PLB1	phospholipase B1 [Source:HGNC Symbol;Acc:HGNC:30041]	2
ENSG00000226833	11,06379	-0,00055	0,741809		-0,32946	0,026036	-0,13199	lncRNA	AC092164.	novel transcript	2
ENSG00000163806	11,08361	0,000551	0,787876		0,26907	0,026011	0,089889	protein_coding	SPDYA	speedy/RINGO cell cycle regulator family member A [Source:HGNC Symbol;Acc:HGNC:30613]	2
ENSG00000171103	226,7752	-0,00072	0,922558		-0,09721	0,024815	-0,00779	protein_coding	TRMT61B	tRNA methyltransferase 61B [Source:HGNC Symbol;Acc:HGNC:26070]	2
ENSG00000163811	1302,738	0,016491	0,253453		1,142002	0,027571	0,051149	protein_coding	WDR43	WD repeat domain 43 [Source:HGNC Symbol;Acc:HGNC:28945]	2

ENSG00000264994	14,69979	-0,00173	0,297753	-1,04126	0,026115	-0,42056	snoRNA	SNORD92	small nucleolar RNA, C/D box 92 [Source:HGNC Symbol;Acc:HGNC:32754]	2
ENSG00000189350	178,9378	-0,00381	0,234217	-1,18956	0,026297	-0,25417	protein_coding	TOGARAM	TOG array regulator of axonemal microtubules 2 [Source:HGNC Symbol;Acc:HGNC:33715]	2
ENSG00000115295	602,6542	-0,00214	0,713914	-0,3666	0,024755	-0,02754	protein_coding	CLIP4	CAP-Gly domain containing linker protein family member 4 [Source:HGNC Symbol;Acc:HGNC:26108]	2
ENSG00000171094	32,63819	-0,00365	0,189335	-1,31255	0,026312	-0,31993	protein_coding	ALK	ALK receptor tyrosine kinase [Source:HGNC Symbol;Acc:HGNC:427]	2
ENSG00000119801	1313,211	0,001953	0,882076	0,148338	0,023169	0,007368	protein_coding	YPEL5	yippee like 5 [Source:HGNC Symbol;Acc:HGNC:18329]	2
ENSG00000213626	805,8011	0,013999	0,073731	1,788277	0,030622	0,174388	protein_coding	LBH	LBH regulator of WNT signaling pathway [Source:HGNC Symbol;Acc:HGNC:29532]	2
ENSG00000172954	570,8522	0,013627	0,25905	1,128639	0,028335	0,069774	protein_coding	LCLAT1	lysocardiolipin acyltransferase 1 [Source:HGNC Symbol;Acc:HGNC:26756]	2
ENSG00000162949	19,66665	0,001501	0,422352	0,802348	0,026085	0,303497	protein_coding	CAPN13	calpain 13 [Source:HGNC Symbol;Acc:HGNC:16663]	2
ENSG00000158089	54,96189	0,004756	0,186681	1,320461	0,02647	0,246115	protein_coding	GALNT14	polypeptide N-acetylgalactosaminyltransferase 14 [Source:HGNC Symbol;Acc:HGNC:22946]	2
ENSG00000214711	11,85371	-0,00262	0,121521	-1,54842	0,026224	-0,61869	protein_coding	CAPN14	calpain 14 [Source:HGNC Symbol;Acc:HGNC:16664]	2
ENSG00000158125	63,67068	0,000481	0,871095	0,162268	0,02592	0,036939	protein_coding	XDH	xanthine dehydrogenase [Source:HGNC Symbol;Acc:HGNC:12805]	2
ENSG00000162959	48,83154	5,99E-05	0,999651	0,000438	0,025733	6,9E-05	protein_coding	MEMO1	mediator of cell motility 1 [Source:HGNC Symbol;Acc:HGNC:14014]	2
ENSG00000162961	382,3285	0,00983	0,307946	1,019542	0,026024	0,06035	protein_coding	DPY30	dpy-30 histone methyltransferase complex regulatory subunit [Source:HGNC Symbol;Acc:HGNC:24590]	2
ENSG00000021574	528,9273	-0,00347	0,728703	-0,34685	0,023535	-0,01794	protein_coding	SPAST	spastin [Source:HGNC Symbol;Acc:HGNC:11233]	2
ENSG00000152683	563,7249	0,008894	0,338802	0,956535	0,02602	0,061744	protein_coding	SLC30A6	solute carrier family 30 member 6 [Source:HGNC Symbol;Acc:HGNC:19305]	2
ENSG00000229816	13,80219	-0,0022	0,286606	-1,0656	0,026139	-0,35005	processed_pseudog	DDX50P1	DEAD-box helicase 50 pseudogene 1 [Source:HGNC Symbol;Acc:HGNC:18974]	2
ENSG00000119820	1195,045	-0,00148	0,815063	-0,2339	0,02282	-0,01094	protein_coding	YIPF4	Yip1 domain family member 4 [Source:HGNC Symbol;Acc:HGNC:28145]	2
ENSG00000276334	18,98108	0,001145	0,677155	0,416349	0,025986	0,106891	lncRNA	AL133243.:	novel transcript, sense intronic to BIRC6	2
ENSG00000276517	19,95006	0,001512	0,481908	0,703237	0,026044	0,205394	lncRNA	AL133243.:	novel transcript, sense intronic to BIRC6	2
ENSG00000018699	249,7278	0,007347	0,292589	1,052458	0,026496	0,096839	protein_coding	TTC27	tetratricopeptide repeat domain 27 [Source:HGNC Symbol;Acc:HGNC:25986]	2
ENSG00000152689	109,729	-0,00756	0,023819	-2,26003	0,027518	-0,45979	protein_coding	RASGRP3	RAS guanyl releasing protein 3 [Source:HGNC Symbol;Acc:HGNC:14545]	2
ENSG00000119812	867,1112	0,114779	0,001621	3,15202	0,056859	0,153566	protein_coding	FAM98A	family with sequence similarity 98 member A [Source:HGNC Symbol;Acc:HGNC:24520]	2
ENSG00000228262	44,06328	0,000861	0,653136	0,44941	0,026073	0,218128	lncRNA	LINC01320	long intergenic non-protein coding RNA 1320 [Source:HGNC Symbol;Acc:HGNC:50526]	2
ENSG00000171055	670,9131	0,004071	0,69092	0,397607	0,023889	0,021722	protein_coding	FEZ2	fasciculation and elongation protein zeta 2 [Source:HGNC Symbol;Acc:HGNC:3660]	2
ENSG00000115808	1154,759	0,049526	0,021003	2,307929	0,053996	0,103995	protein_coding	STRN	striatin [Source:HGNC Symbol;Acc:HGNC:11424]	2
ENSG00000008869	653,4294	0,002707	0,769646	0,292838	0,023897	0,016785	protein_coding	HEATR5B	HEAT repeat containing 5B [Source:HGNC Symbol;Acc:HGNC:29273]	2
ENSG00000152133	544,8678	0,014722	0,096051	1,664306	0,030447	0,132041	protein_coding	GPATCH11	G-patch domain containing 11 [Source:HGNC Symbol;Acc:HGNC:26768]	2
ENSG00000218739	462,8238	-0,00679	0,493035	-0,68549	0,024578	-0,03792	protein_coding	CEBPZOS	CEBPZ opposite strand [Source:HGNC Symbol;Acc:HGNC:49288]	2
ENSG00000115816	1230,763	0,021692	0,107811	1,608111	0,03283	0,080732	protein_coding	CEBPZ	CCAAT enhancer binding protein zeta [Source:HGNC Symbol;Acc:HGNC:24218]	2
ENSG00000272054	11,35497	0,001026	0,570278	0,567643	0,026049	0,211643	lncRNA	AC007390.	novel transcript	2
ENSG00000003509	188,9295	0,005594	0,466632	0,72797	0,025589	0,058504	protein_coding	NDUFAF7	NADH:ubiquinone oxidoreductase complex assembly factor 7 [Source:HGNC Symbol;Acc:HGNC:28816]	2
ENSG00000115825	1098,747	0,01976	0,147784	1,447404	0,029101	0,061844	protein_coding	PRKD3	protein kinase D3 [Source:HGNC Symbol;Acc:HGNC:9408]	2
ENSG00000115828	730,5592	0,004175	0,569172	0,569271	0,025397	0,048863	protein_coding	QPCT	glutaminyl-peptide cyclotransferase [Source:HGNC Symbol;Acc:HGNC:9753]	2
ENSG00000115841	156,1617	-0,00561	0,375391	-0,88642	0,026076	-0,08914	protein_coding	RMDN2	regulator of microtubule dynamics 2 [Source:HGNC Symbol;Acc:HGNC:26567]	2
ENSG00000232973	41,68487	0,004878	0,154859	1,422575	0,026532	0,282812	lncRNA	CYP1B1-AS	CYP1B1 antisense RNA 1 [Source:HGNC Symbol;Acc:HGNC:28543]	2
ENSG00000119787	606,108	-0,00067	0,956702	-0,05429	0,023972	-0,0033	protein_coding	ATL2	atlastin GTPase 2 [Source:HGNC Symbol;Acc:HGNC:24047]	2
ENSG00000213553	135,1787	0,0012	0,832731	0,211201	0,025465	0,02408	processed_pseudog	RPLP0P6	ribosomal protein lateral stalk subunit P0 pseudogene 6 [Source:HGNC Symbol;Acc:HGNC:36404]	2
ENSG00000143889	656,251	-0,01747	0,169618	-1,37343	0,029865	-0,07242	protein_coding	HNRNPLL	heterogeneous nuclear ribonucleoprotein L like [Source:HGNC Symbol;Acc:HGNC:25127]	2
ENSG00000143891	218,2292	0,002772	0,613069	0,505697	0,025424	0,050159	protein_coding	GALM	galactose mutarotase [Source:HGNC Symbol;Acc:HGNC:24063]	2
ENSG00000115875	1440,254	0,020173	0,143223	1,463895	0,029451	0,062911	protein_coding	SRSF7	serine and arginine rich splicing factor 7 [Source:HGNC Symbol;Acc:HGNC:10789]	2
ENSG00000152147	165,7469	0,014443	0,066939	1,832082	0,030738	0,165852	protein_coding	GEMIN6	gem nuclear organelle associated protein 6 [Source:HGNC Symbol;Acc:HGNC:20044]	2
ENSG00000163214	553,5465	0,003173	0,710725	0,370883	0,024694	0,026106	protein_coding	DHX57	DExH-box helicase 57 [Source:HGNC Symbol;Acc:HGNC:20086]	2
ENSG00000188010	140,6976	0,007225	0,230567	1,198899	0,02684	0,136369	protein_coding	MORN2	MORN repeat containing 2 [Source:HGNC Symbol;Acc:HGNC:30166]	2
ENSG00000229692	17,46399	-0,00071	0,733701	-0,34021	0,025993	-0,09956	lncRNA	SOS1-IT1	SOS1 intronic transcript 1 [Source:HGNC Symbol;Acc:HGNC:41385]	2
ENSG00000205111	22,9521	-0,00126	0,604595	-0,5178	0,026012	-0,14309	protein_coding	CDKL4	cyclin dependent kinase like 4 [Source:HGNC Symbol;Acc:HGNC:19287]	2
ENSG00000011566	1155,046	-0,00126	0,906931	-0,11691	0,023642	-0,00649	protein_coding	MAP4K3	mitogen-activated protein kinase kinase kinase kinase 3 [Source:HGNC Symbol;Acc:HGNC:6865]	2
ENSG00000231312	203,8261	-0,00176	0,802599	-0,24999	0,025094	-0,02206	lncRNA	MAP4K3-D	MAP4K3 divergent transcript [Source:HGNC Symbol;Acc:HGNC:54056]	2
ENSG00000152154	14,44758	-0,00139	0,433854	-0,78261	0,026076	-0,29315	protein_coding	TMEM178/	transmembrane protein 178A [Source:HGNC Symbol;Acc:HGNC:28517]	2
ENSG00000138050	153,2258	0,002849	0,645354	0,460226	0,025464	0,04638	protein_coding	THUMPD2	THUMP domain containing 2 [Source:HGNC Symbol;Acc:HGNC:14890]	2
ENSG00000183023	246,437	0,004715	0,25967	1,127172	0,026365	0,180175	protein_coding	SLC8A1	solute carrier family 8 member A1 [Source:HGNC Symbol;Acc:HGNC:11068]	2
ENSG00000162878	123,1621	-0,00297	0,496402	-0,68016	0,025956	-0,10391	protein_coding	PKDCC	protein kinase domain containing, cytoplasmic [Source:HGNC Symbol;Acc:HGNC:25123]	2
ENSG00000115944	816,8997	0,012079	0,252319	1,144736	0,027105	0,06718	protein_coding	COX7A2L	cytochrome c oxidase subunit 7A2 like [Source:HGNC Symbol;Acc:HGNC:2289]	2

ENSG00000171126	74,0233	-0,00326	0,189284	-1,3127	0,026268	-0,36274	protein_coding	KCNG3	potassium voltage-gated channel modifier subfamily G member 3 [Source:HGNC Symbol;Acc:HGNC:18306]	2
ENSG00000057935	447,8019	-0,00384	0,668042	-0,42884	0,024469	-0,02726	protein_coding	MTA3	metastasis associated 1 family member 3 [Source:HGNC Symbol;Acc:HGNC:23784]	2
ENSG00000162882	21,20652	-0,00106	0,650181	-0,45351	0,026006	-0,12822	protein_coding	HAAO	3-hydroxyanthranilate 3,4-dioxygenase [Source:HGNC Symbol;Acc:HGNC:4796]	2
ENSG00000152518	575,7903	-0,01628	0,13333	-1,5011	0,029817	-0,08745	protein_coding	ZFP36L2	ZFP36 ring finger protein like 2 [Source:HGNC Symbol;Acc:HGNC:1108]	2
ENSG00000115970	645,4842	0,000159	0,979209	0,02606	0,024327	0,001758	protein_coding	THADA	THADA armadillo repeat containing [Source:HGNC Symbol;Acc:HGNC:19217]	2
ENSG00000152527	392,8409	-0,01846	0,029204	-2,18072	0,034976	-0,20586	protein_coding	PLEKHH2	pleckstrin homology, MyTH4 and FERM domain containing H2 [Source:HGNC Symbol;Acc:HGNC:30506]	2
ENSG00000223658	18,58551	-0,00224	0,429434	-0,79016	0,026092	-0,20385	protein_coding	C1GALT1C1	C1GALT1 specific chaperone 1 like [Source:HGNC Symbol;Acc:HGNC:51617]	2
ENSG00000138036	167,3756	1,81E-05	0,999439	0,000703	0,025159	6,7E-05	protein_coding	DYNC2LI1	dynein cytoplasmic 2 light intermediate chain 1 [Source:HGNC Symbol;Acc:HGNC:24595]	2
ENSG00000138075	11,18127	0,00024	0,757663	0,308552	0,026072	0,224769	protein_coding	ABCG5	ATP binding cassette subfamily G member 5 [Source:HGNC Symbol;Acc:HGNC:13886]	2
ENSG00000143921	37,06466	0,001027	0,110217	1,597218	0,026106	1,591133	protein_coding	ABCG8	ATP binding cassette subfamily G member 8 [Source:HGNC Symbol;Acc:HGNC:13887]	2
ENSG00000138032	183,0498	0,002922	0,677029	0,416521	0,025377	0,039638	protein_coding	PPM1B	protein phosphatase, Mg2+/Mn2+ dependent 1B [Source:HGNC Symbol;Acc:HGNC:9276]	2
ENSG00000138078	1359,456	-0,01977	0,161852	-1,39887	0,028218	-0,05607	protein_coding	PREPL	prolyl endopeptidase like [Source:HGNC Symbol;Acc:HGNC:30228]	2
ENSG00000143919	92,0173	0,003658	0,472888	0,717788	0,025946	0,094285	protein_coding	CAMKMT	calmodulin-lysine N-methyltransferase [Source:HGNC Symbol;Acc:HGNC:26276]	2
ENSG00000170577	101,3416	-0,00153	0,674879	-0,41946	0,025893	-0,07764	protein_coding	SIX2	SIX homeobox 2 [Source:HGNC Symbol;Acc:HGNC:10888]	2
ENSG00000068784	303,6949	-0,00093	0,906437	-0,11753	0,024338	-0,00791	protein_coding	SRBD1	S1 RNA binding domain 1 [Source:HGNC Symbol;Acc:HGNC:25521]	2
ENSG00000171132	330,6184	-0,00938	0,196585	-1,29134	0,027371	-0,11699	protein_coding	PRKCE	protein kinase C epsilon [Source:HGNC Symbol;Acc:HGNC:9401]	2
ENSG00000250565	67,49925	0,003667	0,39615	0,848517	0,026137	0,139486	protein_coding	ATP6V1E2	ATPase H+ transporting V1 subunit E2 [Source:HGNC Symbol;Acc:HGNC:18125]	2
ENSG00000119729	1260,724	0,005823	0,584931	0,546197	0,024292	0,030064	protein_coding	RHOQ	ras homolog family member Q [Source:HGNC Symbol;Acc:HGNC:17736]	2
ENSG00000151665	171,0496	0,007122	0,26645	1,111276	0,02669	0,118219	protein_coding	PIGF	phosphatidylinositol glycan anchor biosynthesis class F [Source:HGNC Symbol;Acc:HGNC:8962]	2
ENSG00000119878	335,0301	-0,00022	0,975057	-0,03127	0,024765	-0,00246	protein_coding	CRIPT	CXXC repeat containing interactor of PDZ3 domain [Source:HGNC Symbol;Acc:HGNC:14312]	2
ENSG00000239332	17,93589	0,003567	0,138752	1,480454	0,026331	0,414861	lncRNA	LINC01119	long intergenic non-protein coding RNA 1119 [Source:HGNC Symbol;Acc:HGNC:49262]	2
ENSG00000068724	530,1422	0,000988	0,914175	0,107775	0,023969	0,006511	protein_coding	TTC7A	tetratricopeptide repeat domain 7A [Source:HGNC Symbol;Acc:HGNC:19750]	2
ENSG00000234690	13,28965	-0,00055	0,765853	-0,2978	0,026026	-0,11072	lncRNA	EPCAM-DT	EPCAM divergent transcript [Source:HGNC Symbol;Acc:HGNC:52639]	2
ENSG00000236824	628,7638	0,013746	0,007845	2,658674	0,031238	0,378312	scRNA	BCYRN1	brain cytoplasmic RNA 1 [Source:HGNC Symbol;Acc:HGNC:1022]	2
ENSG00000095002	905,7009	0,025923	0,059628	1,883536	0,039079	0,105292	protein_coding	MSH2	mutS homolog 2 [Source:HGNC Symbol;Acc:HGNC:7325]	2
ENSG00000184261	53,62395	0,00082	0,81058	0,239678	0,025849	0,044533	protein_coding	KCNK12	potassium two pore domain channel subfamily K member 12 [Source:HGNC Symbol;Acc:HGNC:6274]	2
ENSG00000116062	1559,812	0,016017	0,296664	1,043613	0,027203	0,046353	protein_coding	MSH6	mutS homolog 6 [Source:HGNC Symbol;Acc:HGNC:7329]	2
ENSG00000233230	11,21374	0,002918	0,160726	1,402633	0,026242	0,458902	lncRNA	AC079807.	novel transcript	2
ENSG00000170802	587,6101	-0,00494	0,630258	-0,48136	0,024488	-0,02906	protein_coding	FOXN2	forkhead box N2 [Source:HGNC Symbol;Acc:HGNC:5281]	2
ENSG00000162869	345,1136	-0,01134	0,218267	-1,23115	0,027757	-0,09013	protein_coding	PPP1R21	protein phosphatase 1 regulatory subunit 21 [Source:HGNC Symbol;Acc:HGNC:30595]	2
ENSG00000243244	159,4679	-0,00531	0,344192	-0,94592	0,026217	-0,11029	protein_coding	STON1	stonin 1 [Source:HGNC Symbol;Acc:HGNC:17003]	2
ENSG00000179915	52,91226	4,14E-05	0,987357	0,015846	0,025904	0,003514	protein_coding	NRXN1	neurexin 1 [Source:HGNC Symbol;Acc:HGNC:8008]	2
ENSG00000115239	253,8792	0,002698	0,702209	0,38234	0,025126	0,032604	protein_coding	ASB3	ankyrin repeat and SOCS box containing 3 [Source:HGNC Symbol;Acc:HGNC:16013]	2
ENSG00000143942	72,0395	0,007615	0,111003	1,593702	0,027184	0,209253	protein_coding	CHAC2	ChaC glutathione specific gamma-glutamylcyclotransferase 2 [Source:HGNC Symbol;Acc:HGNC:32363]	2
ENSG00000119737	28,86021	-0,00434	0,136216	-1,49003	0,026462	-0,35504	protein_coding	GPR75	G protein-coupled receptor 75 [Source:HGNC Symbol;Acc:HGNC:4526]	2
ENSG00000170634	131,4405	0,004397	0,480431	0,70561	0,025809	0,073359	protein_coding	ACYP2	acylphosphatase 2 [Source:HGNC Symbol;Acc:HGNC:180]	2
ENSG00000214595	206,6416	-0,00733	0,328889	-0,97635	0,026679	-0,09835	protein_coding	EML6	EMAP like 6 [Source:HGNC Symbol;Acc:HGNC:35412]	2
ENSG00000162994	77,55847	-0,00171	0,754192	-0,31312	0,025756	-0,04571	protein_coding	CLHC1	clathrin heavy chain linker domain containing 1 [Source:HGNC Symbol;Acc:HGNC:26453]	2
ENSG00000085760	675,1315	0,018941	0,113416	1,583026	0,031819	0,090027	protein_coding	MTIF2	mitochondrial translational initiation factor 2 [Source:HGNC Symbol;Acc:HGNC:7441]	2
ENSG00000162997	16,95459	-0,00065	0,791371	-0,26453	0,025974	-0,07138	transcribed_unitary	PRORSD1P	prolyl-tRNA synthetase associated domain containing 1, pseudogene [Source:HGNC Symbol;Acc:HGNC:34379]	2
ENSG00000163001	811,6051	0,007271	0,470368	0,721881	0,024865	0,041099	protein_coding	CFAP36	cilia and flagella associated protein 36 [Source:HGNC Symbol;Acc:HGNC:30540]	2
ENSG00000272606	11,13473	0,001182	0,486907	0,695237	0,026065	0,270003	lncRNA	PPP4R3B-D	PPP4R3B divergent transcript [Source:HGNC Symbol;Acc:HGNC:55209]	2
ENSG00000138035	599,234	0,010122	0,33855	0,957034	0,025912	0,054057	protein_coding	PNPT1	polyribonucleotide nucleotidyltransferase 1 [Source:HGNC Symbol;Acc:HGNC:23166]	2
ENSG00000115380	431,9696	-0,00348	0,238816	-1,17795	0,026256	-0,26847	protein_coding	EFEMP1	EGF containing fibulin extracellular matrix protein 1 [Source:HGNC Symbol;Acc:HGNC:3218]	2
ENSG00000055813	20,48839	-0,00308	0,068121	-1,82421	0,026297	-0,7016	protein_coding	CCDC85A	coiled-coil domain containing 85A [Source:HGNC Symbol;Acc:HGNC:29400]	2
ENSG00000028116	367,4934	0,018995	0,072047	1,798819	0,033595	0,124157	protein_coding	VRK2	VRK serine/threonine kinase 2 [Source:HGNC Symbol;Acc:HGNC:12719]	2
ENSG00000115392	217,1444	0,007998	0,268665	1,106143	0,026727	0,100316	protein_coding	FANCL	FA complementation group L [Source:HGNC Symbol;Acc:HGNC:20748]	2
ENSG00000233426	11,19847	-0,00149	0,409259	-0,8252	0,026061	-0,26592	processed_pseudog	EIF3FP3	eukaryotic translation initiation factor 3 subunit F pseudogene 3 [Source:HGNC Symbol;Acc:HGNC:37625]	2
ENSG00000231043	12,39481	-0,00015	0,932395	-0,08483	0,025989	-0,02593	processed_pseudog	AC007238.	IK cytokine, down-regulator of HLA II (IK) pseudogene	2
ENSG00000119866	153,7805	-0,00138	0,82899	-0,216	0,025855	-0,03798	protein_coding	BCL11A	BAF chromatin remodeling complex subunit BCL11A [Source:HGNC Symbol;Acc:HGNC:13221]	2
ENSG00000115421	392,0993	0,014353	0,137881	1,483727	0,029597	0,104808	protein_coding	PAPOLG	poly(A) polymerase gamma [Source:HGNC Symbol;Acc:HGNC:14982]	2
ENSG00000162924	221,9844	-0,0012	0,89632	-0,13031	0,02481	-0,01032	protein_coding	REL	REL proto-oncogene, NF-kB subunit [Source:HGNC Symbol;Acc:HGNC:9954]	2

ENSG00000162927	73,69281	0,005748	0,192438	1,303401	0,026614	0,195221	protein_coding	PUS10	pseudouridine synthase 10 [Source:HGNC Symbol;Acc:HGNC:26505]	2
ENSG00000162928	516,908	0,005435	0,583164	0,548768	0,024992	0,036759	protein_coding	PEX13	peroxisomal biogenesis factor 13 [Source:HGNC Symbol;Acc:HGNC:8855]	2
ENSG00000162929	338,5291	0,003578	0,631938	0,479001	0,025291	0,041346	protein_coding	KIAA1841	KIAA1841 [Source:HGNC Symbol;Acc:HGNC:29387]	2
ENSG00000212978	79,68479	0,004657	0,319212	0,996079	0,02628	0,146058	lncRNA	AC016747.	novel transcript	2
ENSG00000237651	18,76054	-0,00076	0,774019	-0,28712	0,025963	-0,07343	protein_coding	C2orf74	chromosome 2 open reading frame 74 [Source:HGNC Symbol;Acc:HGNC:34439]	2
ENSG00000173209	369,6312	-0,00624	0,364732	-0,90639	0,02602	-0,08022	transcribed_unitary	AHSA2P	activator of HSP90 ATPase homolog 2, pseudogene [Source:HGNC Symbol;Acc:HGNC:20437]	2
ENSG00000270820	26,12246	-0,0012	0,742495	-0,32855	0,025959	-0,07602	lncRNA	AC016727.	novel transcript, antisense to XPO1	2
ENSG00000170264	141,4712	-0,01247	0,058841	-1,88938	0,029688	-0,20416	protein_coding	FAM161A	FAM161 centrosomal protein A [Source:HGNC Symbol;Acc:HGNC:25808]	2
ENSG00000173163	244,3831	0,010569	0,201047	1,278574	0,027639	0,102317	protein_coding	COMMD1	copper metabolism domain containing 1 [Source:HGNC Symbol;Acc:HGNC:23024]	2
ENSG00000170340	719,2501	-0,00159	0,87247	-0,16052	0,023934	-0,00953	protein_coding	B3GNT2	UDP-GlcNAc:betaGal beta-1,3-N-acetylglucosaminyltransferase 2 [Source:HGNC Symbol;Acc:HGNC:15629]	2
ENSG00000186889	43,44385	-0,00069	0,839922	-0,20199	0,025847	-0,03783	protein_coding	TMEM17	transmembrane protein 17 [Source:HGNC Symbol;Acc:HGNC:26623]	2
ENSG00000115504	1321,059	0,000609	0,95829	0,052299	0,022447	0,002304	protein_coding	EHBP1	EH domain binding protein 1 [Source:HGNC Symbol;Acc:HGNC:29144]	2
ENSG00000115507	13,23231	0,000181	0,929247	0,088792	0,026013	0,031629	protein_coding	OTX1	orthodenticle homeobox 1 [Source:HGNC Symbol;Acc:HGNC:8521]	2
ENSG00000286480	9,746674	-0,00019	0,953715	-0,05804	0,026048	-0,02803	lncRNA	AC009501.	novel transcript, antisense to WDPCP	2
ENSG00000143951	134,8316	0,007631	0,225761	1,211351	0,026904	0,129565	protein_coding	WDPCP	WD repeat containing planar cell polarity effector [Source:HGNC Symbol;Acc:HGNC:28027]	2
ENSG00000014641	1527,171	0,023531	0,09651	1,662016	0,033576	0,078672	protein_coding	MDH1	malate dehydrogenase 1 [Source:HGNC Symbol;Acc:HGNC:6970]	2
ENSG00000169764	1195,632	0,071902	0,008507	2,631248	0,051074	0,111019	protein_coding	UGP2	UDP-glucose pyrophosphorylase 2 [Source:HGNC Symbol;Acc:HGNC:12527]	2
ENSG00000143952	962,4866	-0,02228	0,194906	-1,2962	0,033622	-0,06627	protein_coding	VPS54	VPS54 subunit of GARP complex [Source:HGNC Symbol;Acc:HGNC:18652]	2
ENSG00000197329	609,5044	-0,0213	0,046772	-1,98836	0,036644	-0,14297	protein_coding	PELI1	pellino E3 ubiquitin protein ligase 1 [Source:HGNC Symbol;Acc:HGNC:8827]	2
ENSG00000225889	9,704686	-0,00049	0,730218	-0,34483	0,026049	-0,15957	lncRNA	AC012368.	novel transcript, antisense to PELI1	2
ENSG00000119862	596,307	-0,00302	0,59982	-0,52466	0,025575	-0,05577	protein_coding	LGALSL	galectin like [Source:HGNC Symbol;Acc:HGNC:25012]	2
ENSG00000223863	79,41327	-1,3E-05	0,955877	-0,05533	0,026082	-0,20231	lncRNA	LINC01805	long intergenic non-protein coding RNA 1805 [Source:HGNC Symbol;Acc:HGNC:52597]	2
ENSG00000260101	10,10005	0,000691	0,606332	0,515316	0,026058	0,238692	lncRNA	AC008074.	novel transcript	2
ENSG00000119844	880,2046	-0,00689	0,752513	-0,31533	0,023985	-0,01552	protein_coding	AFTPH	aftiphilin [Source:HGNC Symbol;Acc:HGNC:25951]	2
ENSG00000179833	639,6392	0,002257	0,814913	0,234093	0,024375	0,015462	protein_coding	SERTAD2	SERTA domain containing 2 [Source:HGNC Symbol;Acc:HGNC:30784]	2
ENSG00000115902	900,8875	-0,00162	0,982855	0,02149	0,023934	0,001274	protein_coding	SLC1A4	solute carrier family 1 member 4 [Source:HGNC Symbol;Acc:HGNC:10942]	2
ENSG00000011523	257,6976	0,00704	0,405784	0,831336	0,025896	0,06324	protein_coding	CEP68	centrosomal protein 68 [Source:HGNC Symbol;Acc:HGNC:29076]	2
ENSG00000172974	32,52094	0,000857	0,802147	0,25057	0,025874	0,048931	processed_pseudog	VDAC2P5	VDAC2 pseudogene 5 [Source:HGNC Symbol;Acc:HGNC:54753]	2
ENSG00000143995	547,5011	0,001623	0,857145	0,180009	0,024498	0,012579	protein_coding	MEIS1	Meis homeobox 1 [Source:HGNC Symbol;Acc:HGNC:7000]	2
ENSG00000143971	478,3627	0,010782	0,222476	1,21997	0,027449	0,089537	protein_coding	ETAA1	ETAA1 activator of ATR kinase [Source:HGNC Symbol;Acc:HGNC:24648]	2
ENSG00000197223	223,711	0,012666	0,076198	1,773184	0,029638	0,176909	protein_coding	C1D	C1D nuclear receptor corepressor [Source:HGNC Symbol;Acc:HGNC:29911]	2
ENSG00000243667	52,30047	0,000219	0,958182	0,052435	0,02575	0,008483	protein_coding	WDR92	WD repeat domain 92 [Source:HGNC Symbol;Acc:HGNC:25176]	2
ENSG00000115946	475,4774	0,003879	0,70173	0,382986	0,024405	0,023908	protein_coding	PNO1	partner of NOB1 homolog [Source:HGNC Symbol;Acc:HGNC:32790]	2
ENSG00000221823	1046,308	0,000309	0,718375	-0,36063	0,023179	-0,01821	protein_coding	PPP3R1	protein phosphatase 3 regulatory subunit B, alpha [Source:HGNC Symbol;Acc:HGNC:9317]	2
ENSG00000119865	164,0786	0,015768	0,0279	2,198683	0,03237	0,225942	protein_coding	CNRIP1	cannabinoid receptor interacting protein 1 [Source:HGNC Symbol;Acc:HGNC:24546]	2
ENSG00000115956	73,42277	0,009692	0,002611	3,010141	0,028674	0,692481	protein_coding	PLEK	pleckstrin [Source:HGNC Symbol;Acc:HGNC:9070]	2
ENSG00000204923	48,32249	-0,00071	0,840886	-0,20076	0,025848	-0,03758	protein_coding	FBXO48	F-box protein 48 [Source:HGNC Symbol;Acc:HGNC:33857]	2
ENSG00000169621	175,0058	0,004915	0,427399	0,79365	0,025768	0,074929	protein_coding	APLF	aprataxin and PNKP like factor [Source:HGNC Symbol;Acc:HGNC:28724]	2
ENSG00000163219	286,9006	0,017279	0,031728	2,147823	0,033626	0,203087	protein_coding	ARHGAP25	Rho GTPase activating protein 25 [Source:HGNC Symbol;Acc:HGNC:28951]	2
ENSG00000169599	463,2503	0,006509	0,473939	0,716085	0,025202	0,046754	protein_coding	NFU1	NFU1 iron-sulfur cluster scaffold [Source:HGNC Symbol;Acc:HGNC:16287]	2
ENSG00000196975	816,5337	0,016761	0,111801	1,590149	0,030731	0,099014	protein_coding	ANXA4	annexin A4 [Source:HGNC Symbol;Acc:HGNC:542]	2
ENSG00000087338	503,9091	0,011417	0,256013	1,135866	0,027085	0,071017	protein_coding	GMCL1	germ cell-less 1, spermatogenesis associated [Source:HGNC Symbol;Acc:HGNC:23843]	2
ENSG00000124380	654,0889	0,011466	0,277149	1,086746	0,026627	0,062164	protein_coding	SNRNP27	small nuclear ribonucleoprotein U4/U6.U5 subunit 27 [Source:HGNC Symbol;Acc:HGNC:30240]	2
ENSG00000059728	858,6088	-0,05549	0,01721	-2,38218	0,078887	-0,13003	protein_coding	MXD1	MAX dimerization protein 1 [Source:HGNC Symbol;Acc:HGNC:6761]	2
ENSG00000179818	294,0797	-0,01132	0,109539	-1,60027	0,028642	-0,15454	lncRNA	PCBP1-AS1	PCBP1 antisense RNA 1 [Source:HGNC Symbol;Acc:HGNC:42948]	2
ENSG00000231327	9,78357	-0,00213	0,227617	-1,20652	0,026152	-0,46732	lncRNA	LINC01816	long intergenic non-protein coding RNA 1816 [Source:HGNC Symbol;Acc:HGNC:52621]	2
ENSG00000115998	164,0011	0,004026	0,572649	0,564154	0,025431	0,04994	protein_coding	C2orf42	chromosome 2 open reading frame 42 [Source:HGNC Symbol;Acc:HGNC:26056]	2
ENSG00000116001	829,8302	0,007328	0,488131	0,693285	0,024563	0,03705	protein_coding	TIA1	TIA1 cytotoxic granule associated RNA binding protein [Source:HGNC Symbol;Acc:HGNC:11802]	2
ENSG00000143977	632,9897	0,012587	0,235663	1,185897	0,027415	0,069868	protein_coding	SNRPG	small nuclear ribonucleoprotein polypeptide G [Source:HGNC Symbol;Acc:HGNC:11163]	2
ENSG00000035141	718,7931	0,025181	0,070293	1,810018	0,038041	0,100299	protein_coding	FAM136A	family with sequence similarity 136 member A [Source:HGNC Symbol;Acc:HGNC:25911]	2
ENSG00000163235	597,5324	0,00365	0,504594	0,667279	0,025852	0,080931	protein_coding	TGFA	transforming growth factor alpha [Source:HGNC Symbol;Acc:HGNC:11765]	2
ENSG00000075340	181,9187	0,003177	0,305031	1,025705	0,026183	0,224913	protein_coding	ADD2	adducin 2 [Source:HGNC Symbol;Acc:HGNC:244]	2

ENSG00000116035	19,98723	-0,0033	0,185391	-1,32434	0,026275	-0,3649	protein_coding	VAX2	ventral anterior homeobox 2 [Source:HGNC Symbol;Acc:HGNC:12661]	2
ENSG00000144043	857,0996	-0,00436	0,689414	-0,39965	0,023371	-0,01948	protein_coding	TEX261	testis expressed 261 [Source:HGNC Symbol;Acc:HGNC:30712]	2
ENSG00000124357	669,9681	0,011597	0,27345	1,095152	0,026818	0,063995	protein_coding	NAGK	N-acetylglucosamine kinase [Source:HGNC Symbol;Acc:HGNC:17174]	2
ENSG00000124370	100,9941	-0,00158	0,761729	-0,30321	0,025435	-0,03312	protein_coding	MCEE	methylmalonyl-CoA epimerase [Source:HGNC Symbol;Acc:HGNC:16732]	2
ENSG00000124383	1094,235	0,022831	0,110448	1,596182	0,030821	0,066385	protein_coding	MPHOSPH:	M-phase phosphoprotein 10 [Source:HGNC Symbol;Acc:HGNC:7213]	2
ENSG00000124374	107,5786	-0,00165	0,675832	-0,41816	0,025769	-0,06229	protein_coding	PAIP2B	poly(A) binding protein interacting protein 2B [Source:HGNC Symbol;Acc:HGNC:29200]	2
ENSG00000281195	27,27442	0,000411	0,864747	0,170334	0,02597	0,046532	lncRNA	AC007878.	novel transcript	2
ENSG00000135636	553,9919	0,00372	0,420034	0,806363	0,026039	0,114222	protein_coding	DYSF	dysferlin [Source:HGNC Symbol;Acc:HGNC:3097]	2
ENSG00000003137	78,7238	-0,00532	0,09777	-1,65576	0,02669	-0,34627	protein_coding	CYP26B1	cytochrome P450 family 26 subfamily B member 1 [Source:HGNC Symbol;Acc:HGNC:20581]	2
ENSG00000144036	937,2424	-0,00045	0,989954	-0,01259	0,022116	-0,00053	protein_coding	EXOC6B	exocyst complex component 6B [Source:HGNC Symbol;Acc:HGNC:17085]	2
ENSG00000116096	256,8077	0,000526	0,924126	0,095238	0,025209	0,009292	protein_coding	SPR	sepiapterin reductase [Source:HGNC Symbol;Acc:HGNC:11257]	2
ENSG00000135638	23,60255	-0,0009	0,546435	-0,60311	0,026011	-0,17317	protein_coding	EMX1	empty spiracles homeobox 1 [Source:HGNC Symbol;Acc:HGNC:3340]	2
ENSG00000144040	329,5067	-0,00278	0,778628	-0,28111	0,024756	-0,02057	protein_coding	SFXN5	sideroflexin 5 [Source:HGNC Symbol;Acc:HGNC:16073]	2
ENSG00000135631	649,3503	0,001661	0,873266	0,159512	0,023427	0,008389	protein_coding	RAB11FIP5	RAB11 family interacting protein 5 [Source:HGNC Symbol;Acc:HGNC:24845]	2
ENSG00000135632	252,6311	0,009948	0,237888	1,180281	0,027225	0,092072	protein_coding	SMYD5	SMYD family member 5 [Source:HGNC Symbol;Acc:HGNC:16258]	2
ENSG00000135617	169,5385	-0,00295	0,670304	-0,42573	0,025293	-0,03868	protein_coding	PRADC1	protease associated domain containing 1 [Source:HGNC Symbol;Acc:HGNC:16047]	2
ENSG00000163013	318,7385	-0,01996	0,030503	-2,16349	0,036226	-0,18296	protein_coding	FBXO41	F-box protein 41 [Source:HGNC Symbol;Acc:HGNC:29409]	2
ENSG00000116127	1145,099	0,008597	0,464623	0,731256	0,023954	0,032976	protein_coding	ALMS1	ALMS1 centrosome and basal body associated protein [Source:HGNC Symbol;Acc:HGNC:428]	2
ENSG00000144034	200,406	0,002428	0,722853	0,354648	0,02527	0,033072	protein_coding	TPRKB	TP53RK binding protein [Source:HGNC Symbol;Acc:HGNC:24259]	2
ENSG00000144048	328,1133	0,013054	0,157369	1,413976	0,028798	0,102681	protein_coding	DUSP11	dual specificity phosphatase 11 [Source:HGNC Symbol;Acc:HGNC:3066]	2
ENSG00000124356	745,456	0,002502	0,828737	0,216322	0,022919	0,010155	protein_coding	STAMBP	STAM binding protein [Source:HGNC Symbol;Acc:HGNC:16950]	2
ENSG00000163017	100,3593	0,005289	0,028374	2,192077	0,026772	0,595447	protein_coding	ACTG2	actin gamma 2, smooth muscle [Source:HGNC Symbol;Acc:HGNC:145]	2
ENSG00000114956	352,5921	-0,00395	0,672122	-0,42324	0,024541	-0,02731	protein_coding	DGUOK	deoxyguanosine kinase [Source:HGNC Symbol;Acc:HGNC:2858]	2
ENSG00000237883	10,20339	0,000801	0,523719	0,637623	0,026043	0,252294	lncRNA	DGUOK-AS	DGUOK antisense RNA 1 [Source:HGNC Symbol;Acc:HGNC:43441]	2
ENSG00000257800	66,94608	0,005202	0,166691	1,382913	0,026574	0,24765	processed_pseudog	FNBP1P1	formin binding protein 1 pseudogene 1 [Source:HGNC Symbol;Acc:HGNC:44528]	2
ENSG00000163170	316,883	0,00953	0,318058	0,998457	0,026403	0,065993	protein_coding	BOLA3	bolA family member 3 [Source:HGNC Symbol;Acc:HGNC:24415]	2
ENSG00000225439	56,13714	0,003832	0,351401	0,931875	0,026189	0,157943	lncRNA	BOLA3-AS1	BOLA3 divergent transcript [Source:HGNC Symbol;Acc:HGNC:42922]	2
ENSG00000284308	14,27973	-0,00097	0,445254	-0,76335	0,026047	-0,2843	protein_coding	C2orf81	chromosome 2 open reading frame 81 [Source:HGNC Symbol;Acc:HGNC:34350]	2
ENSG00000005448	139,3888	0,006145	0,349054	0,936426	0,026249	0,094417	protein_coding	WDR54	WD repeat domain 54 [Source:HGNC Symbol;Acc:HGNC:25770]	2
ENSG00000114993	473,1073	0,004543	0,590401	0,538255	0,025061	0,039302	protein_coding	RTKN	rhotekin [Source:HGNC Symbol;Acc:HGNC:10466]	2
ENSG00000115274	11,6466	-0,00184	0,388394	-0,86253	0,026091	-0,27573	protein_coding	INO80B	INO80 complex subunit B [Source:HGNC Symbol;Acc:HGNC:13324]	2
ENSG00000239779	56,64652	0,005072	0,277225	1,086575	0,026389	0,159324	protein_coding	WBP1	WW domain binding protein 1 [Source:HGNC Symbol;Acc:HGNC:12737]	2
ENSG00000115275	943,7569	-0,00219	0,776881	-0,28339	0,023822	-0,01616	protein_coding	MOGS	mannosyl-oligosaccharide glucosidase [Source:HGNC Symbol;Acc:HGNC:24862]	2
ENSG00000135637	184,0221	0,001145	0,879179	0,15201	0,025374	0,016248	protein_coding	CCDC142	coiled-coil domain containing 142 [Source:HGNC Symbol;Acc:HGNC:25889]	2
ENSG00000115282	304,6942	0,007186	0,43775	0,775999	0,025607	0,053283	protein_coding	TTC31	tetratricopeptide repeat domain 31 [Source:HGNC Symbol;Acc:HGNC:25759]	2
ENSG00000179528	24,30788	-0,00126	0,646653	-0,45842	0,025978	-0,11061	protein_coding	LBX2	ladybird homeobox 2 [Source:HGNC Symbol;Acc:HGNC:15525]	2
ENSG00000272183	8,921283	-0,00049	0,613344	-0,50531	0,026053	-0,23566	lncRNA	AC005041.	novel transcript, antisense to LBX2	2
ENSG00000257702	76,89843	-0,0015	0,698084	-0,38791	0,025809	-0,06236	lncRNA	LBX2-AS1	LBX2 antisense RNA 1 [Source:HGNC Symbol;Acc:HGNC:25136]	2
ENSG00000115289	182,5849	0,007072	0,243977	1,165105	0,026672	0,123811	protein_coding	PCGF1	polycomb group ring finger 1 [Source:HGNC Symbol;Acc:HGNC:17615]	2
ENSG00000115297	20,84292	0,001511	0,421301	0,804166	0,026103	0,340013	protein_coding	TLX2	T cell leukemia homeobox 2 [Source:HGNC Symbol;Acc:HGNC:5057]	2
ENSG00000115317	243,3049	-0,00303	0,697649	-0,3885	0,024981	-0,03053	protein_coding	HTRA2	HtrA serine peptidase 2 [Source:HGNC Symbol;Acc:HGNC:14348]	2
ENSG00000115318	169,2908	-0,00866	0,024544	-2,2485	0,027969	-0,40308	protein_coding	LOXL3	lysyl oxidase like 3 [Source:HGNC Symbol;Acc:HGNC:13869]	2
ENSG00000115325	143,3488	0,003378	0,600066	0,524305	0,025617	0,055122	protein_coding	DOK1	docking protein 1 [Source:HGNC Symbol;Acc:HGNC:2990]	2
ENSG00000159374	14,76885	0,000345	0,774637	0,286314	0,02607	0,19519	protein_coding	M1AP	meiosis 1 associated protein [Source:HGNC Symbol;Acc:HGNC:25183]	2
ENSG00000135622	194,3539	0,021257	0,022375	2,283934	0,038051	0,195262	protein_coding	SEMA4F	ssemaphorin 4F [Source:HGNC Symbol;Acc:HGNC:10734]	2
ENSG00000272711	51,95679	-0,00305	0,459804	-0,73917	0,026005	-0,11905	lncRNA	HK2-DT	HK2 divergent transcript [Source:HGNC Symbol;Acc:HGNC:55204]	2
ENSG00000159399	1459,117	0,026848	0,059392	1,885281	0,039386	0,100824	protein_coding	HK2	hexokinase 2 [Source:HGNC Symbol;Acc:HGNC:4923]	2
ENSG00000115350	214,0386	0,010476	0,198524	1,285768	0,027958	0,1165	protein_coding	POLE4	DNA polymerase epsilon 4, accessory subunit [Source:HGNC Symbol;Acc:HGNC:18755]	2
ENSG00000115353	28,62834	-0,00161	0,494514	-0,68315	0,026048	-0,19457	protein_coding	TACR1	tachykinin receptor 1 [Source:HGNC Symbol;Acc:HGNC:11526]	2
ENSG00000115363	294,0773	0,006622	0,328673	0,976791	0,026285	0,092035	protein_coding	EVA1A	eva-1 homolog A, regulator of programmed cell death [Source:HGNC Symbol;Acc:HGNC:25816]	2
ENSG00000115364	819,1157	0,012391	0,255768	1,136452	0,027355	0,067559	protein_coding	MRPL19	mitochondrial ribosomal protein L19 [Source:HGNC Symbol;Acc:HGNC:14052]	2
ENSG00000005436	253,0008	0,028303	0,015946	2,41014	0,050224	0,192723	protein_coding	GCFC2	GC-rich sequence DNA-binding factor 2 [Source:HGNC Symbol;Acc:HGNC:1317]	2

ENSG00000271452	17,08261	-0,00124	0,532119	-0,62478	0,026044	-0,20266	lncRNA	AC005034.	novel transcript, sense intronic to MRPL19	2
ENSG00000200488	75,2799	0,000661	0,880163	0,150763	0,025716	0,022786	misc_RNA	RN7SKP203	RN7SK pseudogene 203 [Source:HGNC Symbol;Acc:HGNC:45927]	2
ENSG00000143954	24,46437	2,29E-05	0,970995	0,036361	0,026082	0,133505	protein_coding	REG3G	regenerating family member 3 gamma [Source:HGNC Symbol;Acc:HGNC:29595]	2
ENSG00000115386	190,0971	-0,00359	0,126221	-1,52918	0,026352	-0,44603	protein_coding	REG1A	regenerating family member 1 alpha [Source:HGNC Symbol;Acc:HGNC:9951]	2
ENSG00000066032	132,5826	0,00126	0,651644	0,451479	0,025985	0,113929	protein_coding	CTNNA2	catenin alpha 2 [Source:HGNC Symbol;Acc:HGNC:2510]	2
ENSG00000162951	15,62982	6,03E-05	0,987036	-0,01625	0,026041	-0,00722	protein_coding	LRRTM1	leucine rich repeat transmembrane neuronal 1 [Source:HGNC Symbol;Acc:HGNC:19408]	2
ENSG00000163541	660,1842	0,009145	0,332694	0,968702	0,026212	0,063839	protein_coding	SUCLG1	succinate-CoA ligase GDP/ADP-forming subunit alpha [Source:HGNC Symbol;Acc:HGNC:11449]	2
ENSG00000115423	16,65129	0,00042	0,794519	0,260447	0,026039	0,113085	protein_coding	DNAH6	dynein axonemal heavy chain 6 [Source:HGNC Symbol;Acc:HGNC:2951]	2
ENSG00000186854	56,54461	-0,00258	0,385215	-0,86833	0,026103	-0,19851	protein_coding	TRABD2A	TraB domain containing 2A [Source:HGNC Symbol;Acc:HGNC:27013]	2
ENSG00000176407	1398,203	0,004038	0,74381	0,326813	0,021332	0,011708	protein_coding	KCMF1	potassium channel modulatory factor 1 [Source:HGNC Symbol;Acc:HGNC:20589]	2
ENSG00000152284	109,7342	0,007282	0,194845	1,296378	0,026932	0,155227	protein_coding	TCF7L1	transcription factor 7 like 1 [Source:HGNC Symbol;Acc:HGNC:11640]	2
ENSG00000042445	663,8403	-0,00071	0,947784	-0,06549	0,023776	-0,00378	protein_coding	RETSAT	retinol saturase [Source:HGNC Symbol;Acc:HGNC:25991]	2
ENSG00000115459	170,4444	0,003757	0,493302	0,685067	0,025769	0,076057	protein_coding	ELMOD3	ELMO domain containing 3 [Source:HGNC Symbol;Acc:HGNC:26158]	2
ENSG00000042493	323,5607	0,009974	0,091504	1,687519	0,028224	0,198631	protein_coding	CAPG	capping actin protein, gelsolin like [Source:HGNC Symbol;Acc:HGNC:1474]	2
ENSG00000286532	23,45424	-0,00251	0,359127	-0,91703	0,026116	-0,22415	lncRNA	PARTICL	promoter of MAT2A antisense radiation-induced circulating long non-coding RNA [Source:HGNC Symbol;Acc:HGNC:508	2
ENSG00000115486	969,7361	0,01428	0,279698	1,080998	0,025492	0,0444	protein_coding	GGCX	gamma-glutamyl carboxylase [Source:HGNC Symbol;Acc:HGNC:4247]	2
ENSG00000118640	176,0953	-0,00453	0,456833	-0,74407	0,025859	-0,07847	protein_coding	VAMP8	vesicle associated membrane protein 8 [Source:HGNC Symbol;Acc:HGNC:12647]	2
ENSG00000168899	96,52636	0,010174	0,036447	2,091911	0,02863	0,306116	protein_coding	VAMP5	vesicle associated membrane protein 5 [Source:HGNC Symbol;Acc:HGNC:12646]	2
ENSG00000168894	342,0514	0,023279	0,030815	2,159448	0,039649	0,15933	protein_coding	RNF181	ring finger protein 181 [Source:HGNC Symbol;Acc:HGNC:28037]	2
ENSG00000168890	111,9247	-0,00054	0,94206	-0,07268	0,025328	-0,00766	protein_coding	TMEM150A	transmembrane protein 150A [Source:HGNC Symbol;Acc:HGNC:24677]	2
ENSG00000168883	799,2557	0,005919	0,571545	0,565777	0,024061	0,029571	protein_coding	USP39	ubiquitin specific peptidase 39 [Source:HGNC Symbol;Acc:HGNC:20071]	2
ENSG00000168887	535,6488	-0,0255	0,070298	-1,80999	0,037275	-0,09433	protein_coding	C2orf68	chromosome 2 open reading frame 68 [Source:HGNC Symbol;Acc:HGNC:34353]	2
ENSG00000168874	327,818	-0,01384	0,125951	-1,53027	0,029526	-0,11528	protein_coding	ATOH8	atonal bHLH transcription factor 8 [Source:HGNC Symbol;Acc:HGNC:24126]	2
ENSG00000115525	183,2935	0,004354	0,441783	0,769186	0,025908	0,085815	protein_coding	ST3GAL5	ST3 beta-galactoside alpha-2,3-sialyltransferase 5 [Source:HGNC Symbol;Acc:HGNC:10872]	2
ENSG00000132300	1048,953	-0,01085	0,34247	-0,9493	0,024818	-0,0431	protein_coding	PTCD3	pentatricopeptide repeat domain 3 [Source:HGNC Symbol;Acc:HGNC:24717]	2
ENSG00000208772	182,9358	-0,00354	0,285316	-1,06845	0,026165	-0,19172	snoRNA	SNORD94	small nucleolar RNA, C/D box 94 [Source:HGNC Symbol;Acc:HGNC:32756]	2
ENSG00000132305	1470,072	0,013614	0,315936	1,002843	0,02623	0,046272	protein_coding	IMMT	inner membrane mitochondrial protein [Source:HGNC Symbol;Acc:HGNC:6047]	2
ENSG00000132313	524,0849	0,019162	0,101805	1,636166	0,032024	0,093105	protein_coding	MRPL35	mitochondrial ribosomal protein L35 [Source:HGNC Symbol;Acc:HGNC:14489]	2
ENSG00000068615	215,5618	-0,00881	0,17716	-1,34955	0,027294	-0,13401	protein_coding	REEP1	receptor accessory protein 1 [Source:HGNC Symbol;Acc:HGNC:25786]	2
ENSG00000115548	1024,548	0,001596	0,888738	0,139901	0,023487	0,007463	protein_coding	KDM3A	lysine demethylase 3A [Source:HGNC Symbol;Acc:HGNC:20815]	2
ENSG00000115561	881,3613	0,016557	0,165933	1,38539	0,029116	0,072426	protein_coding	CHMP3	charged multivesicular body protein 3 [Source:HGNC Symbol;Acc:HGNC:29865]	2
ENSG00000239305	879,6594	0,008981	0,464237	0,731888	0,023795	0,031692	protein_coding	RNF103	ring finger protein 103 [Source:HGNC Symbol;Acc:HGNC:12859]	2
ENSG00000153561	1263,327	-0,00418	0,688179	-0,40133	0,022798	-0,01777	protein_coding	RMND5A	required for meiotic nuclear division 5 homolog A [Source:HGNC Symbol;Acc:HGNC:25850]	2
ENSG00000153563	9,607603	0,001404	0,205623	1,265694	0,026114	0,740751	protein_coding	CD8A	CD8a molecule [Source:HGNC Symbol;Acc:HGNC:1706]	2
ENSG00000172116	79,07216	0,000746	0,158809	1,409089	0,026094	1,538976	protein_coding	CD8B	CD8b molecule [Source:HGNC Symbol;Acc:HGNC:1707]	2
ENSG00000285793	26,49815	-0,00309	0,222102	-1,22096	0,026216	-0,30986	lncRNA	ANAPC1P2	ANAPC1 pseudogene 2 [Source:NCBI gene (formerly Entrezgene);Acc:285074]	2
ENSG00000204745	20,08117	-0,00089	0,726254	-0,35011	0,025978	-0,09201	unprocessed_pseu	AC083899.	anaphase promoting complex subunit 1 (ANAPC1) pseudogene	2
ENSG00000280721	20,50988	-0,00144	0,498647	-0,67662	0,026052	-0,21171	lncRNA	LINC01943	long intergenic non-protein coding RNA 1943 [Source:HGNC Symbol;Acc:HGNC:52767]	2
ENSG00000222041	315,2812	0,228472	0,000554	3,452996	0,096166	0,288332	lncRNA	CYTOR	cytoskeleton regulator RNA [Source:HGNC Symbol;Acc:HGNC:28717]	2
ENSG00000229604	33,56063	-0,00187	0,665487	-0,43235	0,026054	-0,1137	processed_pseudog	MTATP8P2	MT-ATP8 pseudogene 2 [Source:HGNC Symbol;Acc:HGNC:44572]	2
ENSG00000172086	464,7547	0,032715	0,028069	2,196319	0,053018	0,136863	protein_coding	KRCC1	lysine rich coiled-coil 1 [Source:HGNC Symbol;Acc:HGNC:28039]	2
ENSG00000163586	185,8217	0,000962	0,285768	1,067451	0,026092	0,683025	protein_coding	FABP1	fatty acid binding protein 1 [Source:HGNC Symbol;Acc:HGNC:3555]	2
ENSG00000144115	132,3385	-0,00336	0,441912	-0,76897	0,02607	-0,12435	protein_coding	THNSL2	threonine synthase like 2 [Source:HGNC Symbol;Acc:HGNC:25602]	2
ENSG00000172071	776,7301	-0,02405	0,082541	-1,73613	0,035856	-0,09127	protein_coding	EIF2AK3	eukaryotic translation initiation factor 2 alpha kinase 3 [Source:HGNC Symbol;Acc:HGNC:3255]	2
ENSG00000234028	20,074	-0,00114	0,5854	-0,54551	0,026007	-0,15244	lncRNA	EIF2AK3-DT	EIF2AK3 divergent transcript [Source:HGNC Symbol;Acc:HGNC:54066]	2
ENSG00000153574	130,7195	0,00439	0,624863	0,48897	0,025772	0,049705	protein_coding	RPIA	ribose 5-phosphate isomerase A [Source:HGNC Symbol;Acc:HGNC:10297]	2
ENSG00000230006	11,73509	0,00218	0,225269	1,212636	0,026156	0,45582	transcribed_unproc	ANKRD36B	ankyrin repeat domain 36B pseudogene 2 [Source:HGNC Symbol;Acc:HGNC:33607]	2
ENSG00000211592	21,28521	1,37E-05	0,096415	1,662489	0,026082	6,061277	IG_C_gene	IGKC	immunoglobulin kappa constant [Source:HGNC Symbol;Acc:HGNC:5716]	2
ENSG00000143429	35,07023	-0,00117	0,710956	-0,37057	0,025924	-0,07783	transcribed_unproc	LSP1P4	LSP1 pseudogene 4 [Source:HGNC Symbol;Acc:HGNC:53915]	2
ENSG00000283196	20,44591	0,000363	0,879063	0,152157	0,025978	0,043082	transcribed_unproc	LSP1P5	LSP1 pseudogene 5 [Source:NCBI gene (formerly Entrezgene);Acc:645166]	2
ENSG00000259848	17,89811	-0,00151	0,143707	-1,46212	0,026116	-0,64182	transcribed_unproc	AC097374.	pseudogene similar to part of asparagine synthetase (ASNS)	2
ENSG00000144029	906,7335	-0,01535	0,261648	-1,1225	0,026209	-0,04693	protein_coding	MRPS5	mitochondrial ribosomal protein S5 [Source:HGNC Symbol;Acc:HGNC:14498]	2

ENSG00000144026	239,4076	-0,00474	0,454045	-0,74869	0,025635	-0,0676	protein_coding	ZNF514	zinc finger protein 514 [Source:HGNC Symbol;Acc:HGNC:25894]	2
ENSG00000275111	106,7203	-0,00255	0,619079	-0,49716	0,025777	-0,06634	protein_coding	ZNF2	zinc finger protein 2 [Source:HGNC Symbol;Acc:HGNC:12991]	2
ENSG00000233757	75,7719	0,001443	0,722357	0,355311	0,025813	0,057779	protein_coding	AC092835.	novel C2H2 type zinc finger protein	2
ENSG00000115041	44,91678	-0,00369	0,390023	-0,85957	0,026116	-0,13653	protein_coding	KCNIP3	potassium voltage-gated channel interacting protein 3 [Source:HGNC Symbol;Acc:HGNC:15523]	2
ENSG00000115042	221,1358	-0,00369	0,610794	-0,50894	0,025279	-0,04325	protein_coding	FAHD2A	fumarylacetoacetate hydrolase domain containing 2A [Source:HGNC Symbol;Acc:HGNC:24252]	2
ENSG00000272913	55,66338	-0,00186	0,644388	-0,46157	0,025847	-0,07459	lncRNA	AC009237.	novel transcript	2
ENSG00000229689	54,1	-0,00451	0,238136	-1,17966	0,026373	-0,20877	transcribed_unproc	AC009237.	Ankyrin repeat domain-containing protein pseudogene	2
ENSG00000232931	307,882	-0,00922	0,08211	-1,73857	0,027998	-0,23454	lncRNA	LINC00342	long intergenic non-protein coding RNA 342 [Source:HGNC Symbol;Acc:HGNC:42470]	2
ENSG00000174501	581,8524	-0,00595	0,294435	-1,04844	0,026412	-0,12246	protein_coding	ANKRD36C	ankyrin repeat domain 36C [Source:HGNC Symbol;Acc:HGNC:32946]	2
ENSG00000231584	25,09429	0,000149	0,941092	0,073897	0,025955	0,019269	transcribed_unproc	FAHD2CP	fumarylacetoacetate hydrolase domain containing 2C, pseudogene [Source:HGNC Symbol;Acc:HGNC:44135]	2
ENSG00000186281	41,63586	0,000753	0,651549	0,451611	0,02598	0,121828	protein_coding	GPAT2	glycerol-3-phosphate acyltransferase 2, mitochondrial [Source:HGNC Symbol;Acc:HGNC:27168]	2
ENSG00000158050	32,20369	0,001899	0,462711	0,73439	0,026055	0,19105	protein_coding	DUSP2	dual specificity phosphatase 2 [Source:HGNC Symbol;Acc:HGNC:3068]	2
ENSG00000204685	82,90284	-0,00254	0,620688	-0,49488	0,025683	-0,06046	lncRNA	STARD7-AS	STARD7 antisense RNA 1 [Source:HGNC Symbol;Acc:HGNC:40827]	2
ENSG00000144021	1318,074	-0,01342	0,224387	-1,21494	0,026098	-0,05586	protein_coding	CIAO1	cytosolic iron-sulfur assembly component 1 [Source:HGNC Symbol;Acc:HGNC:14280]	2
ENSG00000198885	38,23681	0,000298	0,920996	0,099179	0,025911	0,022135	protein_coding	ITPRIPL1	ITPRIP like 1 [Source:HGNC Symbol;Acc:HGNC:29371]	2
ENSG00000121152	311,1493	0,007117	0,190385	1,309441	0,026921	0,163108	protein_coding	NCAPH	non-SMC condensin I complex subunit H [Source:HGNC Symbol;Acc:HGNC:1112]	2
ENSG00000163121	14,34697	-0,00083	0,590916	-0,53751	0,026054	-0,22583	protein_coding	NEURL3	neuralized E3 ubiquitin protein ligase 3 [Source:HGNC Symbol;Acc:HGNC:25162]	2
ENSG00000196843	259,7266	0,019609	0,031965	2,144845	0,035736	0,180627	protein_coding	ARID5A	AT-rich interaction domain 5A [Source:HGNC Symbol;Acc:HGNC:17361]	2
ENSG00000114982	1078,371	-0,02478	0,095324	-1,66796	0,032752	-0,07117	protein_coding	KANSL3	KAT8 regulatory NSL complex subunit 3 [Source:HGNC Symbol;Acc:HGNC:25473]	2
ENSG00000249715	11,89987	0,000584	0,756499	0,310081	0,026025	0,11279	protein_coding	FER1L5	fer-1 like family member 5 [Source:HGNC Symbol;Acc:HGNC:19044]	2
ENSG00000114988	273,4568	-0,00439	0,626166	-0,48713	0,024697	-0,03208	protein_coding	LMAN2L	lectin, mannose binding 2 like [Source:HGNC Symbol;Acc:HGNC:19263]	2
ENSG00000158158	393,6334	-0,01645	0,114926	-1,57644	0,030822	-0,10341	protein_coding	CNNM4	cyclin and CBS domain divalent metal cation transport mediator 4 [Source:HGNC Symbol;Acc:HGNC:105]	2
ENSG00000168763	472,2068	-0,013	0,185158	-1,32504	0,028326	-0,08792	protein_coding	CNNM3	cyclin and CBS domain divalent metal cation transport mediator 3 [Source:HGNC Symbol;Acc:HGNC:104]	2
ENSG00000163126	16,87237	-0,00092	0,756447	-0,31015	0,026033	-0,10606	protein_coding	ANKRD23	ankyrin repeat domain 23 [Source:HGNC Symbol;Acc:HGNC:24470]	2
ENSG00000213337	61,4079	-0,00196	0,372561	-0,89169	0,025728	-0,12221	protein_coding	ANKRD39	ankyrin repeat domain 39 [Source:HGNC Symbol;Acc:HGNC:28640]	2
ENSG00000168758	939,22	-0,00174	0,858273	-0,17857	0,024536	-0,01258	protein_coding	SEMA4C	semaphorin 4C [Source:HGNC Symbol;Acc:HGNC:10731]	2
ENSG00000168754	99,68669	0,005458	0,141979	1,468462	0,026663	0,270704	protein_coding	FAM178B	family with sequence similarity 178 member B [Source:HGNC Symbol;Acc:HGNC:28036]	2
ENSG00000144199	77,94434	0,003041	0,477266	0,710707	0,025973	0,109911	protein_coding	FAHD2B	fumarylacetoacetate hydrolase domain containing 2B [Source:HGNC Symbol;Acc:HGNC:25318]	2
ENSG00000135976	506,681	-0,00261	0,666409	-0,43108	0,025508	-0,04574	protein_coding	ANKRD36	ankyrin repeat domain 36 [Source:HGNC Symbol;Acc:HGNC:24079]	2
ENSG00000277701	38,73853	-0,00222	0,407261	-0,82872	0,026081	-0,20512	lncRNA	AC159540.	novel transcript	2
ENSG00000230606	25,49586	0,001504	0,590992	0,537399	0,025994	0,128689	lncRNA	AC092683.	novel transcript	2
ENSG00000196912	234,4007	-0,00484	0,392103	-0,85581	0,025963	-0,0916	protein_coding	ANKRD36B	ankyrin repeat domain 36B [Source:HGNC Symbol;Acc:HGNC:29333]	2
ENSG00000135940	1075,109	0,025395	0,066942	1,832062	0,037518	0,097292	protein_coding	COX5B	cytochrome c oxidase subunit 5B [Source:HGNC Symbol;Acc:HGNC:2269]	2
ENSG00000115073	705,9262	-0,00873	0,395497	-0,84969	0,025592	-0,05056	protein_coding	ACTR1B	actin related protein 1B [Source:HGNC Symbol;Acc:HGNC:168]	2
ENSG00000228486	28,70507	-0,00309	0,165682	-1,38621	0,026257	-0,41518	protein_coding	C2orf92	chromosome 2 open reading frame 92 [Source:HGNC Symbol;Acc:HGNC:49272]	2
ENSG00000115085	127,7216	-0,00049	0,728798	-0,34672	0,026049	-0,15824	protein_coding	ZAP70	zeta chain of T cell receptor associated protein kinase 70 [Source:HGNC Symbol;Acc:HGNC:12858]	2
ENSG00000040933	794,8958	-0,02154	0,134519	-1,49652	0,030381	-0,06414	protein_coding	INPP4A	inositol polyphosphate-4-phosphatase type I A [Source:HGNC Symbol;Acc:HGNC:6074]	2
ENSG00000183513	287,7118	0,006854	0,455497	0,746282	0,025481	0,051056	protein_coding	COA5	cytochrome c oxidase assembly factor 5 [Source:HGNC Symbol;Acc:HGNC:33848]	2
ENSG00000115446	398,2174	0,00237	0,805814	0,24583	0,024156	0,015188	protein_coding	UNC50	unc-50 inner nuclear membrane RNA binding protein [Source:HGNC Symbol;Acc:HGNC:16046]	2
ENSG00000071073	920,0422	0,001569	0,848732	0,190737	0,02417	0,012073	protein_coding	MGAT4A	alpha-1,3-mannosyl-glycoprotein 4-beta-N-acetylglucosaminyltransferase A [Source:HGNC Symbol;Acc:HGNC:7047]	2
ENSG00000226791	19,58912	0,00206	0,354823	0,925276	0,026141	0,335395	lncRNA	LINC02611	long intergenic non-protein coding RNA 2611 [Source:HGNC Symbol;Acc:HGNC:54060]	2
ENSG00000196872	113,7641	0,000513	0,859245	0,177335	0,025922	0,040028	protein_coding	CRACDL	CRACD like [Source:HGNC Symbol;Acc:HGNC:33454]	2
ENSG00000135951	114,7902	0,000247	0,97816	0,027376	0,025462	0,003211	protein_coding	TSGA10	testis specific 10 [Source:HGNC Symbol;Acc:HGNC:14927]	2
ENSG00000273045	50,36389	0,001728	0,572145	0,564896	0,025983	0,122788	protein_coding	C2orf15	chromosome 2 open reading frame 15 [Source:HGNC Symbol;Acc:HGNC:28436]	2
ENSG00000144182	43,96586	0,000416	0,911501	0,111146	0,025815	0,019855	protein_coding	LIPT1	lipoyltransferase 1 [Source:HGNC Symbol;Acc:HGNC:29569]	2
ENSG00000158411	270,5229	0,003134	0,709687	0,372277	0,024752	0,026751	protein_coding	MITD1	microtubule interacting and trafficking domain containing 1 [Source:HGNC Symbol;Acc:HGNC:25207]	2
ENSG00000185414	351,9177	-0,00018	0,749743	0,318978	0,024098	0,020085	protein_coding	MRPL30	mitochondrial ribosomal protein L30 [Source:HGNC Symbol;Acc:HGNC:14036]	2
ENSG00000144214	11,60339	-0,00185	0,312267	-1,01048	0,026121	-0,3857	protein_coding	LYG1	lysozyme g1 [Source:HGNC Symbol;Acc:HGNC:27014]	2
ENSG00000115514	589,18	0,003885	0,643354	0,463015	0,023433	0,023172	protein_coding	TXNDC9	thioredoxin domain containing 9 [Source:HGNC Symbol;Acc:HGNC:24110]	2
ENSG00000135945	520,9752	0,005308	0,61441	0,503789	0,024173	0,027752	protein_coding	REV1	REV1 DNA directed polymerase [Source:HGNC Symbol;Acc:HGNC:14060]	2
ENSG00000144218	142,877	-0,00038	0,905025	-0,11932	0,025881	-0,02447	protein_coding	AFF3	AF4/FMR2 family member 3 [Source:HGNC Symbol;Acc:HGNC:6473]	2
ENSG00000170500	444,4512	-0,0065	0,181981	-1,33468	0,026811	-0,18522	protein_coding	LONRF2	LON peptidase N-terminal domain and ring finger 2 [Source:HGNC Symbol;Acc:HGNC:24788]	2

ENSG00000115526	173,4983	-0,0042	0,529802	-0,62831	0,025619	-0,05998	protein_coding	CHST10	carbohydrate sulfotransferase 10 [Source:HGNC Symbol;Acc:HGNC:19650]	2
ENSG00000115539	341,1813	0,018507	0,05909	1,887525	0,033887	0,145477	protein_coding	PDCL3	phosducin like 3 [Source:HGNC Symbol;Acc:HGNC:28860]	2
ENSG00000170485	999,4383	-0,00342	0,691192	-0,39724	0,024699	-0,02761	protein_coding	NPAS2	neuronal PAS domain protein 2 [Source:HGNC Symbol;Acc:HGNC:7895]	2
ENSG00000204634	560,0988	-0,03315	0,010949	-2,54431	0,061698	-0,19148	protein_coding	TBC1D8	TBC1 domain family member 8 [Source:HGNC Symbol;Acc:HGNC:17791]	2
ENSG00000272902	13,22459	0,001944	0,173487	1,361085	0,02615	0,616857	lncRNA	TBC1D8-AS	TBC1D8 antisense RNA 1 [Source:HGNC Symbol;Acc:HGNC:52782]	2
ENSG00000158435	773,181	0,005458	0,597751	0,527637	0,024099	0,028419	protein_coding	CNOT11	CCR4-NOT transcription complex subunit 11 [Source:HGNC Symbol;Acc:HGNC:25217]	2
ENSG00000163162	562,0347	0,000257	0,967134	0,041203	0,023652	0,002312	protein_coding	RNF149	ring finger protein 149 [Source:HGNC Symbol;Acc:HGNC:23137]	2
ENSG00000212283	227,3551	0,000732	0,972495	-0,03448	0,025864	-0,00663	snoRNA	SNORD89	small nucleolar RNA, C/D box 89 [Source:HGNC Symbol;Acc:HGNC:32750]	2
ENSG00000175874	35,41354	-0,00207	0,301048	-1,03419	0,026123	-0,33206	protein_coding	CREG2	cellular repressor of E1A stimulated genes 2 [Source:HGNC Symbol;Acc:HGNC:14272]	2
ENSG00000196460	114,5901	0,007225	0,124092	1,537821	0,027129	0,22268	protein_coding	RFX8	regulatory factor X8 [Source:HGNC Symbol;Acc:HGNC:37253]	2
ENSG00000115590	21,63447	-0,00248	0,193057	-1,30159	0,026187	-0,45167	protein_coding	IL1R2	interleukin 1 receptor type 2 [Source:HGNC Symbol;Acc:HGNC:5994]	2
ENSG00000115598	20,25052	0,000861	0,694743	0,392426	0,02601	0,11979	protein_coding	IL1RL2	interleukin 1 receptor like 2 [Source:HGNC Symbol;Acc:HGNC:5999]	2
ENSG00000115602	12,89519	-0,00097	0,430099	-0,78902	0,026078	-0,40784	protein_coding	IL1RL1	interleukin 1 receptor like 1 [Source:HGNC Symbol;Acc:HGNC:5998]	2
ENSG00000115604	64,34514	-0,00479	0,12645	-1,52825	0,026546	-0,32896	protein_coding	IL18R1	interleukin 18 receptor 1 [Source:HGNC Symbol;Acc:HGNC:5988]	2
ENSG00000115616	13,82791	-0,00099	0,495055	-0,68229	0,026069	-0,31187	protein_coding	SLC9A2	solute carrier family 9 member A2 [Source:HGNC Symbol;Acc:HGNC:11072]	2
ENSG00000135953	290,9389	-0,00434	0,56673	-0,57287	0,025333	-0,04703	protein_coding	MFSD9	major facilitator superfamily domain containing 9 [Source:HGNC Symbol;Acc:HGNC:28158]	2
ENSG00000170417	51,27606	-0,00428	0,295514	-1,0461	0,026344	-0,1932	protein_coding	TMEM182	transmembrane protein 182 [Source:HGNC Symbol;Acc:HGNC:26391]	2
ENSG00000198914	72,64102	-1,6E-05	0,953031	-0,0589	0,026082	-0,21578	protein_coding	POU3F3	POU class 3 homeobox 3 [Source:HGNC Symbol;Acc:HGNC:9216]	2
ENSG00000135972	501,7965	-3,6E-05	0,996981	-0,00378	0,023856	-0,00022	protein_coding	MRPS9	mitochondrial ribosomal protein S9 [Source:HGNC Symbol;Acc:HGNC:14501]	2
ENSG00000135966	837,8518	-0,00587	0,600803	-0,52325	0,024025	-0,02721	protein_coding	TGFBRAP1	transforming growth factor beta receptor associated protein 1 [Source:HGNC Symbol;Acc:HGNC:16836]	2
ENSG00000272994	28,07082	-0,00307	0,336211	-0,96168	0,026191	-0,22609	lncRNA	C2orf49-DT	C2orf49 divergent transcript [Source:HGNC Symbol;Acc:HGNC:55178]	2
ENSG00000135974	379,6906	-0,00089	0,916634	-0,10468	0,024348	-0,00706	protein_coding	C2orf49	chromosome 2 open reading frame 49 [Source:HGNC Symbol;Acc:HGNC:28772]	2
ENSG00000115641	1199,469	0,00108	0,8921	0,135647	0,023697	0,007645	protein_coding	FHL2	four and a half LIM domains 2 [Source:HGNC Symbol;Acc:HGNC:3703]	2
ENSG00000071051	1502,935	-0,03048	0,060448	-1,87751	0,041969	-0,09349	protein_coding	NCK2	NCK adaptor protein 2 [Source:HGNC Symbol;Acc:HGNC:7665]	2
ENSG00000115652	958,9999	-0,00345	0,659506	-0,4406	0,024561	-0,0293	protein_coding	UXS1	UDP-glucuronate decarboxylase 1 [Source:HGNC Symbol;Acc:HGNC:17729]	2
ENSG00000153165	10,71768	-0,00134	0,414049	-0,81679	0,026081	-0,32924	protein_coding	RGPD3	RANBP2 like and GRIP domain containing 3 [Source:HGNC Symbol;Acc:HGNC:32416]	2
ENSG00000254126	78,38161	2,96E-05	0,885142	0,144454	0,026082	0,528866	protein_coding	CD8B2	CD8b2 molecule [Source:HGNC Symbol;Acc:HGNC:1708]	2
ENSG00000144057	460,8523	0,001822	0,426637	0,79496	0,02605	0,206338	protein_coding	ST6GAL2	ST6 beta-galactoside alpha-2,6-sialyltransferase 2 [Source:HGNC Symbol;Acc:HGNC:10861]	2
ENSG00000237880	22,48506	0,001151	0,20873	1,257065	0,026107	0,889649	lncRNA	LINC01885	long intergenic non-protein coding RNA 1885 [Source:HGNC Symbol;Acc:HGNC:52704]	2
ENSG00000286769	25,86778	1,04E-06	0,844331	-0,19636	0,026048	-0,09216	lncRNA	AC064869.	novel transcript	2
ENSG00000196862	21,78511	-0,00061	0,803498	-0,24882	0,025971	-0,06633	protein_coding	RGPD4	RANBP2 like and GRIP domain containing 4 [Source:HGNC Symbol;Acc:HGNC:32417]	2
ENSG00000196228	57,81706	-0,00072	0,26131	-1,1233	0,026086	-0,79987	protein_coding	SULT1C3	sulfotransferase family 1C member 3 [Source:HGNC Symbol;Acc:HGNC:33543]	2
ENSG00000198203	100,3215	0,003655	0,155185	1,421453	0,026332	0,364386	protein_coding	SULT1C2	sulfotransferase family 1C member 2 [Source:HGNC Symbol;Acc:HGNC:11456]	2
ENSG00000198075	17,3354	0,000303	0,840501	0,201253	0,026029	0,079689	protein_coding	SULT1C4	sulfotransferase family 1C member 4 [Source:HGNC Symbol;Acc:HGNC:11457]	2
ENSG00000163006	149,1976	0,003588	0,387905	0,863422	0,026083	0,135703	protein_coding	CCDC138	coiled-coil domain containing 138 [Source:HGNC Symbol;Acc:HGNC:26531]	2
ENSG00000135960	30,78716	3,22E-05	0,920928	0,099265	0,026082	0,244168	protein_coding	EDAR	ectodysplasin A receptor [Source:HGNC Symbol;Acc:HGNC:2895]	2
ENSG00000259863	17,73821	-0,00235	0,226437	-1,20959	0,026166	-0,41361	lncRNA	SH3RF3-AS	SH3RF3 antisense RNA 1 [Source:HGNC Symbol;Acc:HGNC:44168]	2
ENSG00000172985	386,8662	0,001112	0,829032	0,215943	0,025267	0,021497	protein_coding	SH3RF3	SH3 domain containing ring finger 3 [Source:HGNC Symbol;Acc:HGNC:24699]	2
ENSG00000186522	1241,527	0,009672	0,336973	0,960164	0,025908	0,05628	protein_coding	SEPTIN10	septin 10 [Source:HGNC Symbol;Acc:HGNC:14349]	2
ENSG00000198142	438,5512	0,010054	0,253976	1,140745	0,027006	0,082393	protein_coding	SOWAHC	sosondowah ankyrin repeat domain family member C [Source:HGNC Symbol;Acc:HGNC:26149]	2
ENSG00000204588	19,30581	0,002529	0,440854	0,770752	0,026164	0,228557	lncRNA	LINC01123	long intergenic non-protein coding RNA 1123 [Source:HGNC Symbol;Acc:HGNC:49269]	2
ENSG00000144063	95,61616	0,000713	0,831656	0,212578	0,025876	0,042145	protein_coding	MALL	mal, T cell differentiation protein like [Source:HGNC Symbol;Acc:HGNC:6818]	2
ENSG00000144061	95,89791	-0,00862	0,1373	-1,48592	0,027487	-0,17312	protein_coding	NPHP1	nephrocystin 1 [Source:HGNC Symbol;Acc:HGNC:7905]	2
ENSG00000175701	100,7153	-0,00297	0,619586	-0,49644	0,025566	-0,05288	protein_coding	MTLN	mitoregulin [Source:HGNC Symbol;Acc:HGNC:27339]	2
ENSG00000175772	14,07818	-0,00396	0,043418	-2,01966	0,026458	-0,69083	lncRNA	LINC01106	long intergenic non-protein coding RNA 1106 [Source:HGNC Symbol;Acc:HGNC:26769]	2
ENSG00000169679	646,7895	0,002403	0,624131	0,490004	0,025667	0,059922	protein_coding	BUB1	BUB1 mitotic checkpoint serine/threonine kinase [Source:HGNC Symbol;Acc:HGNC:1148]	2
ENSG00000153093	38,99031	-5,4E-05	0,968882	-0,03901	0,025996	-0,01247	protein_coding	ACOXL	acyl-CoA oxidase like [Source:HGNC Symbol;Acc:HGNC:25621]	2
ENSG00000172965	693,2664	0,032304	0,010999	2,542743	0,061086	0,202121	lncRNA	MIR4435-2	MIR4435-2 host gene [Source:HGNC Symbol;Acc:HGNC:35163]	2
ENSG00000153094	386,4933	-0,01129	0,158264	-1,41093	0,028172	-0,11685	protein_coding	BCL2L11	BCL2 like 11 [Source:HGNC Symbol;Acc:HGNC:994]	2
ENSG00000227992	30,26233	-0,00203	0,551663	-0,59527	0,02597	-0,1169	processed_pseudog	AC108463.	pseudogene similar to part of ubiquitin carboxyl-terminal hydrolase L5 UCHL5	2
ENSG00000271590	10,31616	4,79E-05	0,976853	0,029014	0,026035	0,012496	lncRNA	AC108463.	novel transcript	2
ENSG00000153107	690,782	-0,00104	0,905595	-0,1186	0,023392	-0,00624	protein_coding	ANAPC1	anaphase promoting complex subunit 1 [Source:HGNC Symbol;Acc:HGNC:19988]	2

ENSG00000153208	178,9575	-0,00832	0,102156		-1,63449	0,027533	-0,21784	protein_coding	MERTK	MER proto-oncogene, tyrosine kinase [Source:HGNC Symbol;Acc:HGNC:7027]	2
ENSG00000153214	1427,084	-0,00299	0,794612		-0,26033	0,022569	-0,0114	protein_coding	TMEM87B	transmembrane protein 87B [Source:HGNC Symbol;Acc:HGNC:25913]	2
ENSG00000144152	201,4704	-0,002	0,668766		-0,42784	0,02575	-0,05962	protein_coding	FBLN7	fibulin 7 [Source:HGNC Symbol;Acc:HGNC:26740]	2
ENSG00000278962	69,89242	-0,00075	0,827301		-0,21816	0,025864	-0,0423	TEC	AC092645.	TEC	2
ENSG00000144161	189,777	-0,0022	0,761534		-0,30347	0,025281	-0,02892	protein_coding	ZC3H8	zinc finger CCCH-type containing 8 [Source:HGNC Symbol;Acc:HGNC:30941]	2
ENSG00000286545	9,655045	-0,00024	0,512545		-0,65488	0,026066	-0,3911	lncRNA	AC092645.	novel transcript, antisense to ZC3H8	2
ENSG00000188177	395,5138	0,00325	0,712721		0,368204	0,024632	0,025249	protein_coding	ZC3H6	zinc finger CCCH-type containing 6 [Source:HGNC Symbol;Acc:HGNC:24762]	2
ENSG00000169629	47,93948	-0,00072	0,831808		-0,21238	0,025815	-0,03734	protein_coding	RGPD8	RANBP2 like and GRIP domain containing 8 [Source:HGNC Symbol;Acc:HGNC:9849]	2
ENSG00000125630	476,9769	-0,00267	0,776227		-0,28424	0,024426	-0,01878	protein_coding	POLR1B	RNA polymerase I subunit B [Source:HGNC Symbol;Acc:HGNC:20454]	2
ENSG00000125611	82,63399	0,000604	0,898041		0,128136	0,025701	0,019063	protein_coding	CHCHD5	coiled-coil-helix-coiled-coil-helix domain containing 5 [Source:HGNC Symbol;Acc:HGNC:17840]	2
ENSG00000237753	34,00384	0,000197	0,954549		0,056995	0,025858	0,011198	lncRNA	AC079922.	novel transcript (FLJ42351)	2
ENSG00000169607	476,4579	0,013762	0,04724		1,984138	0,030702	0,213529	protein_coding	CKAP2L	cytoskeleton associated protein 2 like [Source:HGNC Symbol;Acc:HGNC:26877]	2
ENSG00000115008	29,69779	-0,00231	0,291145		-1,05561	0,02614	-0,31854	protein_coding	IL1A	interleukin 1 alpha [Source:HGNC Symbol;Acc:HGNC:5991]	2
ENSG00000125538	47,14777	0,001896	0,358362		0,918491	0,026097	0,283205	protein_coding	IL1B	interleukin 1 beta [Source:HGNC Symbol;Acc:HGNC:5992]	2
ENSG00000136689	62,26407	0,001139	0,540393		0,612219	0,026052	0,217933	protein_coding	IL1RN	interleukin 1 receptor antagonist [Source:HGNC Symbol;Acc:HGNC:6000]	2
ENSG00000125637	465,397	-0,15956	0,003826		-2,89213	0,12488	-0,25198	protein_coding	PSD4	pleckstrin and Sec7 domain containing 4 [Source:HGNC Symbol;Acc:HGNC:19096]	2
ENSG00000189223	312,7561	-0,00658	0,302662		-1,03074	0,026423	-0,10494	lncRNA	PAX8-AS1	PAX8 antisense RNA 1 [Source:HGNC Symbol;Acc:HGNC:49271]	2
ENSG00000125618	36,97617	-0,00118	0,675844		-0,41814	0,025964	-0,09815	protein_coding	PAX8	paired box 8 [Source:HGNC Symbol;Acc:HGNC:8622]	2
ENSG00000136682	329,4078	0,011084	0,25228		1,144828	0,027127	0,074933	protein_coding	CBWD2	COBW domain containing 2 [Source:HGNC Symbol;Acc:HGNC:17907]	2
ENSG00000146556	28,5939	-0,00143	0,639939		-0,46778	0,025957	-0,10242	transcribed_unpro	WASH2P	WASP family homolog 2, pseudogene [Source:HGNC Symbol;Acc:HGNC:33145]	2
ENSG00000236397	16,28218	-0,00223	0,261502		-1,12285	0,026147	-0,37636	unprocessed_pseu	DDX11L2	DEAD/H-box helicase 11 like 2 (pseudogene) [Source:HGNC Symbol;Acc:HGNC:37103]	2
ENSG00000279267	29,41276	0,000694	0,761078		0,304065	0,025995	0,089326	TEC	AL078621.	TEC	2
ENSG00000240356	122,3639	3,7E-05	0,99012		0,012383	0,025473	0,001468	transcribed_proces	RPL23AP7	ribosomal protein L23a pseudogene 7 [Source:HGNC Symbol;Acc:HGNC:17336]	2
ENSG00000144134	89,23168	-0,00536	0,286708		-1,06537	0,026378	-0,14075	protein_coding	RABL2A	RAB, member of RAS oncogene family like 2A [Source:HGNC Symbol;Acc:HGNC:9799]	2
ENSG00000227359	39,01901	-0,00258	0,334643		-0,9648	0,026131	-0,24031	lncRNA	AC017074.	novel transcript	2
ENSG00000270019	16,73626	-0,00161	0,382099		-0,87403	0,026089	-0,30969	lncRNA	AC110769.	novel transcript	2
ENSG00000175497	47,2109	0,000732	0,699098		0,386538	0,025997	0,114162	protein_coding	DPP10	dipeptidyl peptidase like 10 [Source:HGNC Symbol;Acc:HGNC:20823]	2
ENSG00000235026	12,61534	-0,00159	0,21274		-1,24607	0,026121	-0,61451	lncRNA	DPP10-AS1	DPP10 antisense RNA 1 [Source:HGNC Symbol;Acc:HGNC:40941]	2
ENSG00000088205	1159,49	0,015857	0,209663		1,254492	0,027503	0,058027	protein_coding	DDX18	DEAD-box helicase 18 [Source:HGNC Symbol;Acc:HGNC:2741]	2
ENSG00000236255	38,33489	-0,00477	0,159381		-1,40716	0,02651	-0,28416	lncRNA	AC009404.	novel transcript	2
ENSG00000125633	1086,497	-0,00749	0,512206		-0,65541	0,023915	-0,03097	protein_coding	CCDC93	coiled-coil domain containing 93 [Source:HGNC Symbol;Acc:HGNC:25611]	2
ENSG00000279227	15,43183	0,000684	0,667741		0,42925	0,026015	0,141062	TEC	AC009303.	novel transcript	2
ENSG00000125629	463,5861	-0,01568	0,136859		-1,48759	0,029671	-0,09027	protein_coding	INSIG2	insulin induced gene 2 [Source:HGNC Symbol;Acc:HGNC:20452]	2
ENSG00000226856	12,59585	-0,00083	0,581583		-0,55107	0,026057	-0,23904	lncRNA	THORLNC	testis associated oncogenic lncRNA [Source:HGNC Symbol;Acc:HGNC:53788]	2
ENSG00000115107	408,1099	0,017546	0,078834		1,757494	0,032762	0,132971	protein_coding	STEAP3	STEAP3 metalloreductase [Source:HGNC Symbol;Acc:HGNC:24592]	2
ENSG00000186132	57,28907	0,001581	0,740583		0,331081	0,025812	0,053028	protein_coding	C2orf76	chromosome 2 open reading frame 76 [Source:HGNC Symbol;Acc:HGNC:27017]	2
ENSG00000171227	72,8962	-0,00164	0,587958		-0,5418	0,025976	-0,11789	protein_coding	TMEM37	transmembrane protein 37 [Source:HGNC Symbol;Acc:HGNC:18216]	2
ENSG00000144120	84,38507	-0,00726	0,291256		-1,05537	0,027016	-0,13595	protein_coding	TMEM177	transmembrane protein 177 [Source:HGNC Symbol;Acc:HGNC:28143]	2
ENSG00000088179	641,866	-0,0047	0,575683		-0,5597	0,02493	-0,03873	protein_coding	PTPN4	protein tyrosine phosphatase non-receptor type 4 [Source:HGNC Symbol;Acc:HGNC:9656]	2
ENSG00000115109	597,8818	-0,01403	0,189837		-1,31106	0,028393	-0,07822	protein_coding	EPB41L5	erythrocyte membrane protein band 4.1 like 5 [Source:HGNC Symbol;Acc:HGNC:19819]	2
ENSG00000224789	10,27656	-4,2E-05	0,970329		-0,0372	0,026064	-0,02486	lncRNA	AC012363.	novel transcript	2
ENSG00000226479	1045,09	0,005379	0,57722		0,55745	0,024155	0,030491	protein_coding	TMEM185	transmembrane protein 185B [Source:HGNC Symbol;Acc:HGNC:18896]	2
ENSG00000144118	1341,495	0,160122	0,000253		3,659282	0,058709	0,193576	protein_coding	RALB	RAS like proto-oncogene B [Source:HGNC Symbol;Acc:HGNC:9840]	2
ENSG00000163083	633,5138	-0,00031	0,879905		-0,15109	0,025999	-0,0475	protein_coding	INHBB	inhibin subunit beta B [Source:HGNC Symbol;Acc:HGNC:6067]	2
ENSG00000074047	1534,987	0,021017	0,038089		2,0739	0,036903	0,159827	protein_coding	GLI2	GLI family zinc finger 2 [Source:HGNC Symbol;Acc:HGNC:4318]	2
ENSG00000115112	392,2433	0,00114	0,798815		0,254881	0,025719	0,037442	protein_coding	TFCP2L1	transcription factor CP2 like 1 [Source:HGNC Symbol;Acc:HGNC:17925]	2
ENSG00000264229	201,1083	0,000295	0,90655		0,117392	0,025965	0,031587	snRNA	RNU4ATAC	RNA, U4atac small nuclear (U12-dependent splicing) [Source:HGNC Symbol;Acc:HGNC:34016]	2
ENSG00000236859	82,62161	-0,00693	0,175168		-1,35578	0,02699	-0,1925	lncRNA	NIFK-AS1	NIFK antisense RNA 1 [Source:HGNC Symbol;Acc:HGNC:27385]	2
ENSG00000155438	727,3751	0,010881	0,363686		0,908363	0,02556	0,045743	protein_coding	NIFK	nucleolar protein interacting with the FHA domain of MKI67 [Source:HGNC Symbol;Acc:HGNC:17838]	2
ENSG00000211460	1351,446	0,032732	0,058737		1,890155	0,038925	0,07965	protein_coding	TSN	translin [Source:HGNC Symbol;Acc:HGNC:12379]	2
ENSG00000155052	36,91069	-0,00042	0,840431		-0,20134	0,025988	-0,05872	protein_coding	CNTNAP5	contactin associated protein family member 5 [Source:HGNC Symbol;Acc:HGNC:18748]	2
ENSG00000213236	15,88953	0,001742	0,401943		0,838156	0,026084	0,266476	processed_pseudo	YWHAZP2	tyrosine 3-monooxygenase/tryptophan 5-monooxygenase activation protein zeta pseudogene 2 [Source:HGNC Symbol;	2

ENSG00000136732	382,0529	0,01047	0,310097	1,015019	0,027236	0,073447	protein_coding	GYPC	glycophorin C (Gerbich blood group) [Source:HGNC Symbol;Acc:HGNC:4704]	2
ENSG00000136717	505,7429	-0,00094	0,907895	-0,11569	0,02476	-0,00901	protein_coding	BIN1	bridging integrator 1 [Source:HGNC Symbol;Acc:HGNC:1052]	2
ENSG00000163161	897,8399	-0,00434	0,790029	-0,26627	0,022597	-0,01137	protein_coding	ERCC3	ERCC excision repair 3, TFIIH core complex helicase subunit [Source:HGNC Symbol;Acc:HGNC:3435]	2
ENSG00000236682	38,20579	-0,00294	0,315048	-1,00469	0,026159	-0,22824	lncRNA	MAP3K2-D	MAP3K2 divergent transcript [Source:HGNC Symbol;Acc:HGNC:54088]	2
ENSG00000115718	210,255	-0,00225	0,658644	-0,44179	0,025748	-0,05986	protein_coding	PROC	protein C, inactivator of coagulation factors Va and VIIIa [Source:HGNC Symbol;Acc:HGNC:9451]	2
ENSG00000169994	171,3424	0,005777	0,149506	1,441278	0,026719	0,246885	protein_coding	MYO7B	myosin VIIB [Source:HGNC Symbol;Acc:HGNC:7607]	2
ENSG00000072163	132,1702	0,004128	0,187046	1,319369	0,026375	0,280761	protein_coding	LIMS2	LIM zinc finger domain containing 2 [Source:HGNC Symbol;Acc:HGNC:16084]	2
ENSG00000136709	1255,263	0,016371	0,190073	1,310363	0,025934	0,050961	protein_coding	WDR33	WD repeat domain 33 [Source:HGNC Symbol;Acc:HGNC:25651]	2
ENSG00000173349	173,295	0,001836	0,770853	0,291259	0,025349	0,029513	protein_coding	SFT2D3	SFT2 domain containing 3 [Source:HGNC Symbol;Acc:HGNC:28767]	2
ENSG00000144231	800,4344	0,016634	0,163712	1,392697	0,029484	0,075403	protein_coding	POLR2D	RNA polymerase II subunit D [Source:HGNC Symbol;Acc:HGNC:9191]	2
ENSG00000144233	582,1705	-0,00805	0,448739	-0,75752	0,024741	-0,04002	protein_coding	AMMECR1	AMMECR1 like [Source:HGNC Symbol;Acc:HGNC:28658]	2
ENSG00000272667	28,63487	-0,00166	0,59272	-0,5349	0,025971	-0,11512	lncRNA	AC012306.	novel transcript	2
ENSG00000286873	14,8592	-0,00185	0,39094	-0,85791	0,026092	-0,27118	lncRNA	AC012306.	novel transcript	2
ENSG00000136715	683,6132	0,024364	0,081827	1,740181	0,035417	0,087514	protein_coding	SAP130	Sin3A associated protein 130 [Source:HGNC Symbol;Acc:HGNC:29813]	2
ENSG00000136720	427,4707	-0,00932	0,329993	-0,97413	0,025904	-0,05879	protein_coding	HS6ST1	heparan sulfate 6-O-sulfotransferase 1 [Source:HGNC Symbol;Acc:HGNC:5201]	2
ENSG00000196604	17,61145	0,00038	0,828909	0,216101	0,02603	0,085858	protein_coding	POTEF	POTE ankyrin domain family member F [Source:HGNC Symbol;Acc:HGNC:33905]	2
ENSG00000152076	93,25808	-0,00174	0,711929	-0,36927	0,025719	-0,05118	protein_coding	CCDC74B	coiled-coil domain containing 74B [Source:HGNC Symbol;Acc:HGNC:25267]	2
ENSG00000136699	1036,679	-0,00272	0,782221	-0,27643	0,022883	-0,01284	protein_coding	SMPD4	sphingomyelin phosphodiesterase 4 [Source:HGNC Symbol;Acc:HGNC:32949]	2
ENSG00000152082	506,2559	0,023874	0,054662	1,921553	0,038258	0,119502	protein_coding	MZT2B	mitotic spindle organizing protein 2B [Source:HGNC Symbol;Acc:HGNC:25886]	2
ENSG00000213225	31,43215	0,002376	0,244453	1,163929	0,026163	0,384237	transcribed_proces	NOC2LP1	NOC2 like nucleolar associated transcriptional repressor pseudogene 1 [Source:HGNC Symbol;Acc:HGNC:52285]	2
ENSG00000136710	199,4401	0,007542	0,294161	1,049036	0,026589	0,09713	protein_coding	CCDC115	coiled-coil domain containing 115 [Source:HGNC Symbol;Acc:HGNC:28178]	2
ENSG00000136718	565,5071	0,017491	0,110223	1,597193	0,031273	0,098573	protein_coding	IMP4	IMP U3 small nucleolar ribonucleoprotein 4 [Source:HGNC Symbol;Acc:HGNC:30856]	2
ENSG00000072135	1016,775	0,000994	0,891591	0,136292	0,023326	0,007068	protein_coding	PTPN18	protein tyrosine phosphatase non-receptor type 18 [Source:HGNC Symbol;Acc:HGNC:9649]	2
ENSG00000178162	48,90218	0,000119	0,959793	0,050414	0,025979	0,014848	transcribed_unpro	FAR2P2	fatty acyl-CoA reductase 2 pseudogene 2 [Source:HGNC Symbol;Acc:HGNC:49279]	2
ENSG00000178171	293,9038	0,000287	0,948371	0,064753	0,025598	0,008598	protein_coding	AMER3	APC membrane recruitment protein 3 [Source:HGNC Symbol;Acc:HGNC:26771]	2
ENSG00000136002	126,5594	-0,00632	0,167706	-1,37961	0,026837	-0,2149	protein_coding	ARHGEF4	Rho guanine nucleotide exchange factor 4 [Source:HGNC Symbol;Acc:HGNC:684]	2
ENSG00000188219	30,05496	-0,00021	0,910095	-0,11292	0,025966	-0,03088	protein_coding	POTEE	POTE ankyrin domain family member E [Source:HGNC Symbol;Acc:HGNC:33895]	2
ENSG00000232742	10,257	0,001667	0,388571	0,862212	0,02609	0,302444	processed_pseudo	RHOQP2	ras homolog family member Q pseudogene 2 [Source:HGNC Symbol;Acc:HGNC:37836]	2
ENSG00000173272	230,8929	0,019282	0,022451	2,282654	0,035726	0,205246	protein_coding	MZT2A	mitotic spindle organizing protein 2A [Source:HGNC Symbol;Acc:HGNC:33187]	2
ENSG00000152117	255,2104	-0,00425	0,581583	-0,55107	0,025264	-0,04439	transcribed_proces	SMPD4BP	sphingomyelin phosphodiesterase 4B, pseudogene [Source:HGNC Symbol;Acc:HGNC:24761]	2
ENSG00000163040	336,9479	0,018683	0,05928	1,886113	0,033894	0,141579	protein_coding	CCDC74A	coiled-coil domain containing 74A [Source:HGNC Symbol;Acc:HGNC:25197]	2
ENSG00000204434	12,98023	-0,00056	0,680822	-0,41134	0,026056	-0,20541	unprocessed_pseu	POTEKP	POTE ankyrin domain family member K, pseudogene [Source:HGNC Symbol;Acc:HGNC:30182]	2
ENSG00000287151	288,0213	0,011071	0,089722	1,696867	0,028637	0,175122	lncRNA	C2orf27A	chromosome 2 open reading frame 27A [Source:HGNC Symbol;Acc:HGNC:25077]	2
ENSG00000288031	20,73016	-0,00019	0,829802	0,214955	0,025977	0,061304	transcribed_unpro	AC093838.	novel pseudogene	2
ENSG00000197927	26,35336	0,004126	0,1016	1,637145	0,026429	0,412972	transcribed_unpro	NBEAP2	neurobeachin pseudogene 2 [Source:HGNC Symbol;Acc:HGNC:40003]	2
ENSG00000286833	15,08153	-5,7E-05	0,969874	-0,03777	0,026035	-0,01608	lncRNA	AC097532.	novel transcript	2
ENSG00000183840	126,7923	0,006266	0,254713	1,138976	0,026574	0,139522	protein_coding	GPR39	G protein-coupled receptor 39 [Source:HGNC Symbol;Acc:HGNC:4496]	2
ENSG00000150551	445,4815	-0,00137	0,924958	-0,09419	0,025536	-0,01132	protein_coding	LYPD1	LY6/PLAUR domain containing 1 [Source:HGNC Symbol;Acc:HGNC:28431]	2
ENSG00000176771	705,269	-0,0277	0,005071	-2,80251	0,052021	-0,27055	protein_coding	NCKAP5	NCK associated protein 5 [Source:HGNC Symbol;Acc:HGNC:29847]	2
ENSG00000226953	13,9029	-0,00126	0,482868	-0,7017	0,026067	-0,2663	lncRNA	NCKAP5-AS	NCKAP5 antisense RNA 2 [Source:HGNC Symbol;Acc:HGNC:54065]	2
ENSG00000152128	47,67194	-0,00571	0,094066	-1,67433	0,026792	-0,3397	protein_coding	TMEM163	transmembrane protein 163 [Source:HGNC Symbol;Acc:HGNC:25380]	2
ENSG00000224043	50,71276	0,000595	0,797208	0,256962	0,025805	0,04472	lncRNA	CCNT2-AS1	CCNT2 antisense RNA 1 [Source:HGNC Symbol;Acc:HGNC:40130]	2
ENSG00000082258	680,7404	-0,00794	0,42843	-0,79188	0,025788	-0,05303	protein_coding	CCNT2	cyclin T2 [Source:HGNC Symbol;Acc:HGNC:1600]	2
ENSG00000115839	1502,942	-0,02105	0,179569	-1,34208	0,029882	-0,05697	protein_coding	RAB3GAP1	RAB3 GTPase activating protein catalytic subunit 1 [Source:HGNC Symbol;Acc:HGNC:17063]	2
ENSG00000208308	52,16048	-0,00039	0,885501	-0,144	0,025936	-0,03456	snoRNA	SNORA40B	small nucleolar RNA, H/ACA box 40B [Source:HGNC Symbol;Acc:HGNC:52204]	2
ENSG00000121988	97,97226	0,013728	0,032753	2,1351	0,030918	0,258752	protein_coding	ZRANB3	zinc finger RANBP2-type containing 3 [Source:HGNC Symbol;Acc:HGNC:25249]	2
ENSG00000048991	1263,85	0,000144	0,748765	0,320268	0,022609	0,014534	protein_coding	R3HDM1	R3H domain containing 1 [Source:HGNC Symbol;Acc:HGNC:9757]	2
ENSG00000076003	918,2333	0,018108	0,125105	1,533694	0,030364	0,080897	protein_coding	MCM6	minichromosome maintenance complex component 6 [Source:HGNC Symbol;Acc:HGNC:6949]	2
ENSG00000115866	1143,303	0,014179	0,29409	1,049191	0,025844	0,044955	protein_coding	DARS1	aspartyl-tRNA synthetase 1 [Source:HGNC Symbol;Acc:HGNC:2678]	2
ENSG00000121966	55,99898	-0,00484	0,173693	-1,36043	0,026502	-0,25987	protein_coding	CXCR4	C-X-C motif chemokine receptor 4 [Source:HGNC Symbol;Acc:HGNC:2561]	2
ENSG00000227347	12,58763	0,000945	0,619238	0,496931	0,026038	0,175861	processed_pseudo	HNRNPKP2	heterogeneous nuclear ribonucleoprotein K pseudogene 2 [Source:HGNC Symbol;Acc:HGNC:42375]	2
ENSG00000150540	215,386	0,004515	0,471542	0,719972	0,025984	0,082672	protein_coding	HNMT	histamine N-methyltransferase [Source:HGNC Symbol;Acc:HGNC:5028]	2

ENSG00000144228	497,4383	-0,00486	0,560982	-0,58138	0,025168	-0,043	protein_coding	SPOPL	speckle type BTB/POZ protein like [Source:HGNC Symbol;Acc:HGNC:27934]	2
ENSG00000168702	82,90486	-0,00095	0,703897	-0,38006	0,026011	-0,11389	protein_coding	LRP1B	LDL receptor related protein 1B [Source:HGNC Symbol;Acc:HGNC:6693]	2
ENSG00000115919	111,3637	0,000937	0,728428	0,347217	0,025869	0,065982	protein_coding	KYNU	kynureninase [Source:HGNC Symbol;Acc:HGNC:6469]	2
ENSG00000121964	262,8458	0,006647	0,419351	0,807548	0,025757	0,061392	protein_coding	GTDC1	glycosyltransferase like domain containing 1 [Source:HGNC Symbol;Acc:HGNC:20887]	2
ENSG00000279166	14,56341	-0,00175	0,292468	-1,05272	0,026114	-0,41121	TEC	AC009951.	TEC	2
ENSG00000226674	43,1244	-0,00033	0,909379	-0,11382	0,025916	-0,02572	lncRNA	TEX41	testis expressed 41 [Source:HGNC Symbol;Acc:HGNC:48667]	2
ENSG00000121989	311,8481	0,001695	0,672071	0,423307	0,024479	0,029338	protein_coding	ACVR2A	activin A receptor type 2A [Source:HGNC Symbol;Acc:HGNC:173]	2
ENSG00000115947	514,3407	0,010358	0,340878	0,952432	0,026393	0,057809	protein_coding	ORC4	origin recognition complex subunit 4 [Source:HGNC Symbol;Acc:HGNC:8490]	2
ENSG00000204406	573,3827	-0,00994	0,315581	-1,00358	0,026165	-0,06052	protein_coding	MBD5	methyl-CpG binding domain protein 5 [Source:HGNC Symbol;Acc:HGNC:20444]	2
ENSG00000135999	1077,511	0,066895	0,010372	2,563163	0,053304	0,110745	protein_coding	EPC2	enhancer of polycomb homolog 2 [Source:HGNC Symbol;Acc:HGNC:24543]	2
ENSG00000168280	1498,946	-0,0053	0,560144	-0,58263	0,024669	-0,03601	protein_coding	KIF5C	kinesin family member 5C [Source:HGNC Symbol;Acc:HGNC:6325]	2
ENSG00000150556	49,65774	-0,0005	0,857688	-0,17932	0,025936	-0,04321	protein_coding	LYPD6B	LY6/PLAUR domain containing 6B [Source:HGNC Symbol;Acc:HGNC:27018]	2
ENSG00000187123	61,39241	0,004462	0,163925	1,39199	0,026445	0,289829	protein_coding	LYPD6	LY6/PLAUR domain containing 6 [Source:HGNC Symbol;Acc:HGNC:28751]	2
ENSG00000168288	1062,913	0,024403	0,080553	1,747486	0,03632	0,092434	protein_coding	MMADHC	metabolism of cobalamin associated D [Source:HGNC Symbol;Acc:HGNC:25221]	2
ENSG00000231969	15,36859	0,000469	0,835371	0,207818	0,02599	0,062019	lncRNA	MMADHC-	MMADHC divergent transcript [Source:HGNC Symbol;Acc:HGNC:41087]	2
ENSG00000184898	137,5119	0,008385	0,160366	1,403843	0,027275	0,153604	protein_coding	RBM43	RNA binding motif protein 43 [Source:HGNC Symbol;Acc:HGNC:24790]	2
ENSG00000123609	272,5074	0,021098	0,016731	2,392571	0,038295	0,21839	protein_coding	NMI	N-myc and STAT interactor [Source:HGNC Symbol;Acc:HGNC:7854]	2
ENSG00000123610	918,4953	0,001021	0,911384	0,111293	0,025457	0,012682	protein_coding	TNFAIP6	TNF alpha induced protein 6 [Source:HGNC Symbol;Acc:HGNC:11898]	2
ENSG00000183091	413,3869	-0,00086	0,826492	-0,2192	0,025787	-0,03596	protein_coding	NEB	nebulin [Source:HGNC Symbol;Acc:HGNC:7720]	2
ENSG00000162980	550,2844	0,003111	0,774277	0,286785	0,024374	0,018354	protein_coding	ARL5A	ADP ribosylation factor like GTPase 5A [Source:HGNC Symbol;Acc:HGNC:696]	2
ENSG00000182389	32,45826	0,003647	0,144344	1,459803	0,026342	0,398555	protein_coding	CACNB4	calcium voltage-gated channel auxiliary subunit beta 4 [Source:HGNC Symbol;Acc:HGNC:1404]	2
ENSG00000115145	1247,423	0,009341	0,422391	0,80228	0,02496	0,040641	protein_coding	STAM2	signal transducing adaptor molecule 2 [Source:HGNC Symbol;Acc:HGNC:11358]	2
ENSG00000157827	1024,235	-0,01082	0,32941	-0,9753	0,025745	-0,05073	protein_coding	FMNL2	formin like 2 [Source:HGNC Symbol;Acc:HGNC:18267]	2
ENSG00000177917	163,7382	1,09E-06	0,997717	-0,00286	0,025149	-0,00027	protein_coding	ARL6IP6	ADP ribosylation factor like GTPase 6 interacting protein 6 [Source:HGNC Symbol;Acc:HGNC:24048]	2
ENSG00000144278	90,20756	0,003919	0,170182	1,37162	0,026307	0,254366	protein_coding	GALNT13	polypeptide N-acetylgalactosaminyltransferase 13 [Source:HGNC Symbol;Acc:HGNC:23242]	2
ENSG00000287900	12,729	-0,00053	0,712075	-0,36907	0,026045	-0,16048	lncRNA	AC061961.	novel transcript	2
ENSG00000162989	96,9363	0,001103	0,952354	0,059751	0,025916	0,011557	protein_coding	KCNJ3	potassium inwardly rectifying channel subfamily J member 3 [Source:HGNC Symbol;Acc:HGNC:6264]	2
ENSG00000226383	48,42598	-0,0033	0,28641	-1,06603	0,026207	-0,23261	lncRNA	LINC01876	long intergenic non-protein coding RNA 1876 [Source:HGNC Symbol;Acc:HGNC:52695]	2
ENSG00000153234	345,3631	-0,00695	0,079006	-1,75648	0,027162	-0,30397	protein_coding	NR4A2	nuclear receptor subfamily 4 group A member 2 [Source:HGNC Symbol;Acc:HGNC:7981]	2
ENSG00000115159	829,5619	0,006996	0,602927	0,520196	0,0245	0,027993	protein_coding	GPD2	glycerol-3-phosphate dehydrogenase 2 [Source:HGNC Symbol;Acc:HGNC:4456]	2
ENSG00000136542	502,3193	-0,01303	0,093056	-1,67949	0,02959	-0,15035	protein_coding	GALNT5	polypeptide N-acetylgalactosaminyltransferase 5 [Source:HGNC Symbol;Acc:HGNC:4127]	2
ENSG00000136541	9,077276	-0,00116	0,187803	-1,31711	0,026106	-0,93533	protein_coding	ERMN	ermin [Source:HGNC Symbol;Acc:HGNC:29208]	2
ENSG00000115165	28,58936	-0,00269	0,210155	-1,25314	0,026198	-0,38439	protein_coding	CYTIP	cytohesin 1 interacting protein [Source:HGNC Symbol;Acc:HGNC:9506]	2
ENSG00000123612	43,61213	-0,00427	0,156548	-1,41678	0,02643	-0,32231	protein_coding	ACVR1C	activin A receptor type 1C [Source:HGNC Symbol;Acc:HGNC:18123]	2
ENSG00000115170	1477,291	-0,00111	0,926654	-0,09206	0,021979	-0,00375	protein_coding	ACVR1	activin A receptor type 1 [Source:HGNC Symbol;Acc:HGNC:171]	2
ENSG00000153237	55,76604	-0,00239	0,435992	-0,77898	0,026061	-0,17107	protein_coding	CCDC148	coiled-coil domain containing 148 [Source:HGNC Symbol;Acc:HGNC:25191]	2
ENSG00000144283	1549,739	-0,00229	0,77027	-0,29202	0,022926	-0,01376	protein_coding	PKP4	plakophilin 4 [Source:HGNC Symbol;Acc:HGNC:9026]	2
ENSG00000163331	10,72957	9,75E-05	0,975303	0,030958	0,026063	0,018253	protein_coding	DAPL1	death associated protein like 1 [Source:HGNC Symbol;Acc:HGNC:21490]	2
ENSG00000213189	13,94992	0,003366	0,191269	1,306834	0,026297	0,373364	processed_pseudo(	BTF3L4P2	basic transcription factor 3 like 4 pseudogene 2 [Source:HGNC Symbol;Acc:HGNC:39646]	2
ENSG00000196151	113,4487	-0,00988	0,080838	-1,74584	0,028187	-0,20778	protein_coding	WDSUB1	WD repeat, sterile alpha motif and U-box domain containing 1 [Source:HGNC Symbol;Acc:HGNC:26697]	2
ENSG00000224152	40,60076	0,003173	0,33454	0,96501	0,026157	0,196985	lncRNA	AC009506.	novel transcript, antisense to BAZ2B	2
ENSG00000226266	25,05114	-0,00042	0,90395	-0,12067	0,025929	-0,02847	lncRNA	AC009961.	novel transcript	2
ENSG00000241399	145,329	-0,00107	0,83525	-0,20797	0,025564	-0,02591	protein_coding	CD302	CD302 molecule [Source:HGNC Symbol;Acc:HGNC:30843]	2
ENSG00000287091	12,02893	-0,00193	0,303458	-1,02905	0,026121	-0,3727	lncRNA	AC009961.	novel transcript, sense intronic to CD302and LY75-CD302	2
ENSG00000054219	9,509948	0,000453	0,732026	0,342431	0,026053	0,166385	protein_coding	LY75	lymphocyte antigen 75 [Source:HGNC Symbol;Acc:HGNC:6729]	2
ENSG00000153246	418,7681	-0,00209	0,790395	-0,2658	0,025559	-0,03058	protein_coding	PLA2R1	phospholipase A2 receptor 1 [Source:HGNC Symbol;Acc:HGNC:9042]	2
ENSG00000115221	155,0278	0,003752	0,321762	0,990844	0,026195	0,173258	protein_coding	ITGB6	integrin subunit beta 6 [Source:HGNC Symbol;Acc:HGNC:6161]	2
ENSG00000153250	1555,362	0,027919	0,065887	1,839191	0,035885	0,08047	protein_coding	RBMS1	RNA binding motif single stranded interacting protein 1 [Source:HGNC Symbol;Acc:HGNC:9907]	2
ENSG00000136560	910,386	-0,00525	0,59104	-0,53733	0,024403	-0,03126	protein_coding	TANK	TRAF family member associated NFKB activator [Source:HGNC Symbol;Acc:HGNC:11562]	2
ENSG00000227403	12,84946	0,000567	0,747145	0,322407	0,026065	0,174005	lncRNA	LINC01806	long intergenic non-protein coding RNA 1806 [Source:HGNC Symbol;Acc:HGNC:52599]	2
ENSG00000225813	18,04773	-0,00114	0,512482	-0,65498	0,026063	-0,24984	processed_pseudo(	AC009299.	novel pseudogene	2
ENSG00000115233	1499,478	0,02587	0,046422	1,991536	0,031307	0,076226	protein_coding	PSMD14	proteasome 26S subunit, non-ATPase 14 [Source:HGNC Symbol;Acc:HGNC:16889]	2

ENSG00000232320	15,7638	0,003313	0,124946	1,53434	0,026304	0,477776	processed_pseudog	MXRA7P1	MXRA7 pseudogene 1 [Source:HGNC Symbol;Acc:HGNC:54643]	2
ENSG00000144290	47,93512	-0,00041	0,677397	-0,41602	0,026007	-0,12967	protein_coding	SLC4A10	solute carrier family 4 member 10 [Source:HGNC Symbol;Acc:HGNC:13811]	2
ENSG00000226121	23,67933	0,004858	0,084946	1,72268	0,026582	0,390763	processed_pseudog	AHCTF1P1	AT-hook containing transcription factor 1 pseudogene 1 [Source:HGNC Symbol;Acc:HGNC:24620]	2
ENSG00000197635	574,8123	-0,00108	0,807235	-0,244	0,025724	-0,03643	protein_coding	DPP4	dipeptidyl peptidase 4 [Source:HGNC Symbol;Acc:HGNC:3009]	2
ENSG00000115267	279,6128	-0,00817	0,258385	-1,13022	0,026798	-0,10213	protein_coding	IFIH1	interferon induced with helicase C domain 1 [Source:HGNC Symbol;Acc:HGNC:18873]	2
ENSG00000115271	177,2092	-0,00165	0,807214	-0,24402	0,025319	-0,02451	protein_coding	GCA	grancalcin [Source:HGNC Symbol;Acc:HGNC:15990]	2
ENSG00000184611	150,6369	-0,00577	0,196037	-1,29293	0,026627	-0,19427	protein_coding	KCNH7	potassium voltage-gated channel subfamily H member 7 [Source:HGNC Symbol;Acc:HGNC:18863]	2
ENSG00000182263	189,8956	-0,00614	0,402025	-0,83801	0,025937	-0,07266	protein_coding	FIGN	fidgetin, microtubule severing factor [Source:HGNC Symbol;Acc:HGNC:13285]	2
ENSG00000115290	62,57586	-0,0036	0,3796	-0,87863	0,026113	-0,14245	protein_coding	GRB14	growth factor receptor bound protein 14 [Source:HGNC Symbol;Acc:HGNC:4565]	2
ENSG00000082438	832,8131	0,000156	0,999118	0,001106	0,024166	7,1E-05	protein_coding	COBLL1	cordon-bleu WH2 repeat protein like 1 [Source:HGNC Symbol;Acc:HGNC:23571]	2
ENSG00000236283	14,7168	0,000764	0,677589	0,415755	0,026035	0,15567	lncRNA	AC019197.	novel transcript, antisense to SLC38A11 and SCN3A	2
ENSG00000169507	78,87232	0,000288	0,934822	0,081779	0,025849	0,015909	protein_coding	SLC38A11	solute carrier family 38 member 11 [Source:HGNC Symbol;Acc:HGNC:26836]	2
ENSG00000153253	956,4926	-0,00431	0,480985	-0,70472	0,025814	-0,07429	protein_coding	SCN3A	sodium voltage-gated channel alpha subunit 3 [Source:HGNC Symbol;Acc:HGNC:10590]	2
ENSG00000136531	308,8366	-0,00207	0,683543	-0,40763	0,025676	-0,05228	protein_coding	SCN2A	sodium voltage-gated channel alpha subunit 2 [Source:HGNC Symbol;Acc:HGNC:10588]	2
ENSG00000288089	10,7406	7,12E-05	0,972968	0,033886	0,026078	0,031065	lncRNA	AC011303.	novel transcript	2
ENSG00000178662	842,839	-0,00173	0,833754	-0,20989	0,024648	-0,01543	protein_coding	CSRNP3	cysteine and serine rich nuclear protein 3 [Source:HGNC Symbol;Acc:HGNC:30729]	2
ENSG00000115339	454,2728	-0,00325	0,583348	-0,5485	0,025758	-0,06534	protein_coding	GALNT3	polypeptide N-acetylgalactosaminyltransferase 3 [Source:HGNC Symbol;Acc:HGNC:4125]	2
ENSG00000123607	574,659	0,000344	0,9696	0,03811	0,023527	0,002078	protein_coding	TTC21B	tetratricopeptide repeat domain 21B [Source:HGNC Symbol;Acc:HGNC:25660]	2
ENSG00000169432	287,3137	-0,00703	0,217993	-1,23188	0,026806	-0,14465	protein_coding	SCN9A	sodium voltage-gated channel alpha subunit 9 [Source:HGNC Symbol;Acc:HGNC:10597]	2
ENSG00000136546	84,75282	0,001698	0,52011	0,643176	0,026029	0,16415	protein_coding	SCN7A	sodium voltage-gated channel alpha subunit 7 [Source:HGNC Symbol;Acc:HGNC:10594]	2
ENSG00000228222	13,09571	-0,00111	0,529687	-0,62848	0,026059	-0,24555	lncRNA	AC073050.	novel transcript (hCG1660379)	2
ENSG00000172318	52,16933	-0,00289	0,205732	-1,26539	0,026219	-0,36847	protein_coding	B3GALT1	beta-1,3-galactosyltransferase 1 [Source:HGNC Symbol;Acc:HGNC:916]	2
ENSG00000198648	1029,531	0,010074	0,331002	0,972097	0,026015	0,056183	protein_coding	STK39	serine/threonine kinase 39 [Source:HGNC Symbol;Acc:HGNC:17717]	2
[truncated: 546,098 more chars]
